# Biogeographical venom variation in the Indian spectacled cobra (*Naja naja*) underscores the pressing need for pan-India efficacious snakebite therapy

**DOI:** 10.1371/journal.pntd.0009150

**Published:** 2021-02-18

**Authors:** R. R. Senji Laxme, Saurabh Attarde, Suyog Khochare, Vivek Suranse, Gerard Martin, Nicholas R. Casewell, Romulus Whitaker, Kartik Sunagar

**Affiliations:** 1 Evolutionary Venomics Lab. Centre for Ecological Sciences, Indian Institute of Science, Bangalore, Karnataka, India; 2 The Liana Trust, Survey #1418/1419 Rathnapuri, Hunsur, Karnataka, India; 3 Centre for Snakebite Research & Interventions, Liverpool School of Tropical Medicine, Pembroke Place, Liverpool, United Kingdom; 4 Madras Crocodile Bank Trust/Centre for Herpetology, Mamallapuram, Tamil Nadu, India; Goethe University, GERMANY

## Abstract

**Background:**

Snake venom composition is dictated by various ecological and environmental factors, and can exhibit dramatic variation across geographically disparate populations of the same species. This molecular diversity can undermine the efficacy of snakebite treatments, as antivenoms produced against venom from one population may fail to neutralise others. India is the world’s snakebite hotspot, with 58,000 fatalities and 140,000 morbidities occurring annually. Spectacled cobra (*Naja naja*) and Russell’s viper (*Daboia russelii*) are known to cause the majority of these envenomations, in part due to their near country-wide distributions. However, the impact of differing ecologies and environment on their venom compositions has not been comprehensively studied.

**Methods:**

Here, we used a multi-disciplinary approach consisting of venom proteomics, biochemical and pharmacological analyses, and *in vivo* research to comparatively analyse *N*. *naja* venoms across a broad region (>6000 km; seven populations) covering India’s six distinct biogeographical zones.

**Findings:**

By generating the most comprehensive pan-Indian proteomic and toxicity profiles to date, we unveil considerable differences in the composition, pharmacological effects and potencies of geographically-distinct venoms from this species and, through the use of immunological assays and preclinical experiments, demonstrate alarming repercussions on antivenom therapy. We find that commercially-available antivenom fails to effectively neutralise envenomations by the pan-Indian populations of *N*. *naja*, including a complete lack of neutralisation against the desert *Naja* population.

**Conclusion:**

Our findings highlight the significant influence of ecology and environment on snake venom composition and potency, and stress the pressing need to innovate pan-India effective antivenoms to safeguard the lives, limbs and livelihoods of the country’s 200,000 annual snakebite victims.

## Author summary

Annually, India is burdened by the highest number of snake envenomations across the globe, with over 58,000 fatalities and three times the number of morbidities, predominantly affecting the rural agrarian communities. The spectacled cobra (*Naja naja*) and Russell’s viper (*Daboia russelii*) are responsible for the vast majority of envenomations in the country, in part, due to their near country-wide distributions. In this study, we unveil the astounding differences in venom composition of *N*. *naja* from six different biogeographical zones across the country (>6000 km). We provide a comprehensive account of their disparate venom proteomic profiles, biochemical and pharmacological effects, and the associated potencies. Our study uncovers alarming differences in the efficacy of the marketed polyvalent antivenoms in neutralising these venoms, thereby, emphasising the pressing need to develop dose-efficacious and pan-India effective antivenoms for the treatment of snakebites in the country. This study also highlights the significant influence of ecology and diverse environments on the venom variability, insinuating the necessity for innovating cost-effective and pan-India efficacious solutions to safeguard the lives, limbs and livelihoods of India’s two hundred thousand annual snakebite victims.

## Introduction

Venom is an adaptive trait that has evolved multiple times across the animal kingdom to facilitate various ecological functions, including defence, predation, competition, or a combination thereof [1–4]. Given their medical relevance to humans in the form of snakebite, and the tremendous biodiscovery potential of their toxic molecules, snake venoms have received unparalleled research attention. In India, there are over 60 described snake species capable of inflicting clinically significant envenomations in humans, among which 14 species have been documented to cause human fatalities [5]. Nevertheless, existing antivenoms—only available specific treatment for snakebite—are produced exclusively against the so-called ‘big four’ snakes: the spectacled cobra (*Naja naja*), common krait (*Bungarus caeruleus*), Russell’s viper (*Daboia russelii*) and saw-scaled viper (*Echis carinatus*). Despite the availability of polyvalent antivenom, snakebite continues to be a severe burden on the rural agrarian communities in India, resulting in an annual toll greater than that of any other country [6,7].

The composition of venom, which is theorised to be influenced by various ecological and environmental factors, including diet, predator pressure, climatic zones, and ontogenetic shifts, can vary across the geographical distribution of snake species [8–12], even at very short distances [13,14]. This variation not only underpins the ecological adaptations of the animal but also severely impacts the efficacy of snakebite therapy. Commercial Indian antivenoms are produced by hyperimmunising equines with the ‘big four’ snake venoms and purifying the resultant anti-snake venom toxin antibodies. However, venoms are sourced from only a couple of districts in the southern part of the country, which may therefore render them incapable of neutralising the toxic effects of other more distant populations where venom composition may vary [15]. While such variation has been noted in the venoms of selected populations of *N*. *naja* [16–25], the true extent of biogeographic venom variation and its impact on the efficacy of marketed antivenoms is yet to be comprehensively elucidated.

To address these shortcomings, we investigated the venoms of one of the most medically important Indian snakes, *N*. *naja*, which has been reported to be responsible for the majority of snakebite fatalities and disabilities in the Indian subcontinent [7]. We characterised the composition and function of venom from this snake species from six distinct biogeographical zones across the country (>6000 km), thereby, generating the most comprehensive proteomic and toxicity profiles of this species to date. The results of our *in vitro* and *in vivo* experiments revealed dramatic differences in toxin compositions, synergistic pharmacological effects, and *in vivo* potency of the venoms. We also reveal the disturbing impact this variation has on the effectiveness of commercial Indian antivenoms to neutralise venoms sourced from different parts of the country. Our results highlight the significant impact that ecology and environment can have in shaping these complex biochemical cocktails, and emphasise the urgent need to develop pan-India effective snakebite therapies.

## Methods

### Ethics statement

The median lethal dose (LD_50_) of venoms and the median effective dose (ED_50_) of commercially available antivenoms were determined as per World Health Organization (WHO)-recommended protocols at the Central Animal Facility, Indian Institute of Science (IISc), Bangalore (Registration number 48/GO/ReBi/SL/1999 /CPCSEA; 11-03-1999). For these assays, male CD-1 mice (18–22 g) were used with due approval from (i) the Committee for the Purpose of Control and Supervision of Experiments on Animals (CPCSEA), Government of India; and (ii) the Institutional Animal Ethics Committee (IAEC), IISc, Bangalore (CAF/Ethics/642/2018; CAF/Ethics/643/2018). Based on the results of *in vitro* venom recognition experiments (enzyme-linked immunosorbent assay and immunoblotting), a single commercial antivenom was selected for the ED_50_ experiments to limit the numbers of experimental animals subjected to these severe-rated experiments. Animals were handled according to the institutional guidelines during and after the completion of the experiment. To evaluate snake venom-induced coagulopathies on human blood, ethical permission was obtained from the Institute Human Ethical Committee (IHEC No: 5–24072019), IISc, Bangalore, and blood was collected from healthy volunteers after explaining the details of the study.

### Sampling permits, snake venoms and antivenoms

Snake venoms were collected from 80+ individuals across a range of 6000 km from the following regions with appropriate permissions from the respective State Forest Departments: North (Punjab: #3615;11/10/12), South (Tamil Nadu), Southeast (Andhra Pradesh:#13526/2017/WL-3), East (West Bengal: 386/WL/4R-6/2017), West (Rajasthan: P.3(3)Forest/2004), Southwest (Maharashtra: Desk-22(8)/Research/CR-80(16–17) /943/2017-18), and Central (Madhya Pradesh: #/TK-1/48-II/606) India. The venom samples were collected from individuals with or without pooling, flash-frozen, and stored at -80° C following lyophilisation, until use (S1A Table). Details of the investigated Indian antivenoms produced by four major commercial antivenom manufacturers are provided in the S1B Table.

### Protein concentration

Following reconstitution in molecular grade water, protein concentrations of the venoms were estimated using the Bradford method, with bovine serum albumin (BSA) as standard [[26]; S1A Table]. The antivenom vials were reconstituted as per the manufacturer’s guidelines, and the total IgG content was estimated using the bovine gamma globulin (BGG) standard curve (S1B Table).

### Gel electrophoresis

Venom samples were normalised for protein content (12 μg), and the components were separated using sodium dodecyl sulfate-polyacrylamide gel electrophoresis (SDS-PAGE) under reducing conditions [27]. Coomassie Brilliant Blue R-250 (Sisco Research Laboratories Pvt. Ltd, India) stained gels were visualised in an iBright CL1000 (Thermo Fisher Scientific, USA) gel documentation system.

### Reversed-phase high-performance liquid chromatography (RP-HPLC)

The reconstituted venoms were fractionated using a Shimadzu LC-20AD series HPLC system (Kyoto, Japan) using a previously described protocol with modifications [28]. 200 μg of each venom was loaded onto a 4.6 × 250 mm, C18 (5 μm, 300 Å) reversed-phase column (Shimadzu, Japan), and equilibrated with solution A [0.1% trifluoroacetic acid (TFA) in water (v/v)]. The fractions were eluted at a flow rate of 1 ml/min using the following concentration gradients of solution B [0.1% TFA in 100% acetonitrile (v/v)]: 5–15%, 15–45% and 45–70% for 10, 60 and 10 min, respectively, and the absorbance was monitored at 215 nm.

### Liquid chromatography-tandem mass spectrometry (LC-MS/MS)

The proteomic profiles of the collected HPLC fractions (40 μg) were characterised via electrospray ionisation tandem mass spectrometry (ESI-MS/MS). Following reduction with dithiothreitol (DTT; 10 mM), alkylation using iodoacetamide (IAA; 30 mM), and an overnight trypsin (0.2 μg/μl) digestion, each HPLC fraction was desalted. Liquid chromatography of these processed samples was performed using a Thermo EASY nLC 1200 series system (Thermo Fisher Scientific, MA, USA) with a 50 cm × 75 μm, C18 (3 μm, 100 Å) nano-LC column. The sample (injection volume of 2 μl) was run at a flow rate of 300 nL/min in buffer A (0.1% formic acid in HPLC grade water) and buffer B (0.1% formic acid in 80% acetonitrile) solutions. The gradient of buffer B used for the elution was 10–45% over the first 98 min, 45–95% over the next 4 min, followed by 95% over the last 18 min. Mass spectrometric analyses of the samples were performed using the Thermo Orbitrap Fusion Mass Spectrometer (Thermo Fisher Scientific, MA, USA). For the MS scan, the following parameters were used: scan range (m/z) of 375–1700 with a resolution of 120000 and maximum injection time of 50 ms. For the fragment scans, an ion trap detector was used with high collision energy fragmentation (30%), scan range (m/z) of 100–2000, and maximum injection time of 35 ms. The raw MS/MS spectra were searched against the SwissProt database (www.uniprot.org) using PEAKS Studio X (Bioinformatics Solutions Inc., ON, Canada) with the following parameters: parent and fragment mass error tolerance limits of 10 ppm and 0.6 Da, respectively; ‘monoisotopic’ precursor ion search type; and ‘semispecific’ trypsin digestion. Carbamidomethylation and oxidation were specified as fixed and variable modifications, respectively. Error in the identification of peptides was minimised by fixing the False Discovery Rate (FDR) for peptide-spectrum matching at 0.1% and the corresponding -10lgP cutoff value was automatically determined by PEAKS Studio. Only hits with one or more unique peptides were considered for downstream analyses. The mass spectrometry data generated in this study have been deposited to the ProteomeXchange Consortium via the PRIDE partner repository [29], with data identifier: PXD020497. The relative abundance of each toxin hit in a fraction was determined by estimating its area under the spectral intensity curve (i.e., AUC) relative to the total AUC for all toxins in that fraction. The AUC values obtained from PEAKS Studio analyses represented the mean spectral intensities [30] and were normalised across fractions using the percentage of peak areas for the respective RP-HPLC fractions [31]. Thus, the relative abundance of a toxin hit (X) was calculated as follows (here, N indicates the number of fractions obtained from RP-HPLC):

$$Relative\;abundance\;of\;X\;(\%)=\sum_{n=1}^{N}\frac{AUC\;of\;X\;in\;Fraction\;F_{n\;}\times AUC\;of\;the\;chromatographic\;fraction\;F_{n}\;(\%)}{Total\;AUC\;of\;all\;toxin\;hits\;in\;Fraction\;F_{n}\;}$$

### Venom biochemistry

The biochemical activities of the various venom samples were evaluated in the following assays using previously described methods [14].

### Phospholipase A_2_ (PLA_2_) assay

Slightly modified turbidimetric assays were conducted to assess the PLA_2_ activities of venoms as described previously [14,32]. A fresh chicken egg was used to prepare the egg-yolk substrate solution in 0.9% NaCl solution, such that its absorbance at 740 nm corresponded to 1. A fixed concentration of crude venom samples (1 μg) prepared in 20 mM Tris-HCl buffer, time-dependent kinetic assays were performed in triplicate. Following the addition of 250 μl of the egg yolk solution, absorbance was measured for 60 min at 740 nm in an EPOCH 2 microplate spectrophotometer (BioTek Instruments, Inc., USA). Unit activity was calculated as the amount of crude venom required to reduce the absorbance of the substrate by 0.01 OD unit per min at the given wavelength [33].

### Snake venom protease assay

Protease activity was estimated using azocasein as a substrate using the protocol described previously [34]. A known volume of crude venom was incubated with 80 μl of the substrate at 37° C for 90 min in triplicate. The reaction was stopped using 200 μl of trichloroacetic acid, and the supernatant was obtained by centrifuging at 1000 × g for 5 min. To this, an equal volume of 0.5 M NaOH was added, and the absorbance was measured at 440 nm. Purified protease from bovine pancreas (Sigma-Aldrich, USA) was used as a positive control to calculate the relative protease activity of the crude venoms.

### L-amino acid oxidase (LAAO) assay

LAAO activity was assessed using a previously described endpoint assay with slight modifications [14,35]. Briefly, the L-leucine substrate solution, containing Tris-HCl buffer (50 mM), L-leucine (5 mM), horseradish peroxidase (5 IU/ml), and o-phenylenediamine dihydrochloride (2 mM), was mixed with crude venom (10 μg) in a 9:1 ratio and incubated at 37° C for 60 min in triplicates. The reaction was stopped by adding 2 M H_2_SO_4,_ and the absorbance was measured at 492 nm with an EPOCH 2 microplate spectrophotometer.

### DNase assay

To assay the DNase activities of venoms, a modified protocol was employed wherein purified DNA from calf thymus (Sigma-Aldrich, USA) dissolved in phosphate buffer saline (PBS; pH 7.4) was incubated with a known concentration of crude venom at 37° C for 60 min. Post-incubation, samples were subjected to agarose gel electrophoresis on 0.8% agarose gel, followed by visualisation on an iBright CL1000 [14,36].

### Fibrinogenolytic assay

Fibrinogenolytic activities of snake venoms against human fibrinogen were determined using a method previously described by Ouyang and Teng [14,37]. The reaction mixture contained 15 μg of human fibrinogen (Sigma-Aldrich, USA) dissolved in PBS (pH 7.4), and a known concentration of venom, ranging between 1 and 10 μg and was incubated at 37° C for 60 min. After incubation, an equal volume of loading dye (1 M Tris-HCl pH 6.8, 50% glycerol, 0.5% bromophenol blue, 10% SDS, 20% β-mercaptoethanol) was added and the samples heated at 70° C for 10 min. Subsequently, samples were separated by 15% SDS-PAGE, staining the gel with Coomassie Brilliant Blue R-250, prior to visualisation in an iBright CL1000 (Thermo Fisher Scientific, USA) gel documentation system. Results are interpreted with respect to a negative control that only consists of human fibrinogen without venom, where all three bands are seen intact.

### Blood coagulation assays

The effect of snake venom on the two major coagulation cascades, namely, the extrinsic and intrinsic pathways, were evaluated by measuring prothrombin time (PT) and activated partial thromboplastin time (aPTT), respectively. In brief, platelet-poor plasma (PPP), obtained by centrifuging human blood at 3000 × g for 10 min at 4° C, was mixed with different venom concentrations. A Hemostar XF 2.0 coagulometer and commercially available UNIPLASTIN and LIQUICELIN-E diagnostic kits (Tulip Diagnostics, Mumbai) were used for conducting PT and aPTT tests, respectively.

### Haemolytic assay

Haemolytic activities of venoms were assessed as described previously [14,38]. For assaying haemolytic activities of venoms, human red blood cells (RBC), obtained after the separation of PPP, were washed five times with 1× PBS buffer (pH 7.4) and centrifuged at 3000 × g for 10 min at 4° C. Following the resuspension of the RBC pellet in PBS, samples were incubated with different concentrations of venoms (5, 10, 20 and 40 μg) at 37° C for 24 hours in triplicate. Thereafter, reaction mixtures were centrifuged at 3000 × g for 10 min at 4° C, and the absorbance of the supernatant was measured at 540 nm using an Epoch 2 microplate spectrophotometer. Triton X (0.5%) and PBS were used as positive and negative controls, respectively.

### Enzyme-linked immunosorbent assay (ELISA)

Indirect ELISA experiments were used to quantify the *in vitro* binding titres between the venoms and commercial antivenoms. ELISAs were performed using minor modifications of previously described protocols [14,39]. Venom samples (100 ng) were diluted in a carbonate buffer (pH 9.6) and coated onto 96-well plates. After overnight incubation at 4° C, the unbound venom was washed off using Tris-buffered saline (0.01 M Tris pH 8.5, 0.15 M NaCl) containing 1% Tween 20 (TBST), and incubated with blocking buffer (5% skimmed milk in TBST) for 3 hours at room temperature. Following another round of TBST washing, the venom-bound plates were incubated overnight with different dilutions of commercial antivenoms at 4° C. All four antivenoms (Premium Serums, VINS, Bharat, and Haffkine), with sequential fivefold dilutions (starting from 1:4 dilution) in blocking buffer (1 mg/ml), were added to the plates in triplicates. Thereafter, unbound antibodies were removed by TBST washing and the plates were incubated at room temperature for 2 hours following the addition of horseradish peroxidase (HRP)-conjugated, rabbit anti-horse secondary antibody (Sigma-Aldrich, USA), diluted at a ratio of 1:1000 in PBS. Finally, 100 μl of 2,2/-azino-bis (2-ethylbenzthiazoline-6-sulphonic acid) substrate solution (Sigma-Aldrich, USA) was added, the resulting optical density measured at a wavelength of 405 nm for 40 min, and plotted against the respective dilution. The 40^th^ min was chosen as the endpoint based on the results of the standardisation experiments that showed the highest binding at this time interval. The cut off for non-specific binding was determined as described earlier, using IgG from unimmunised (naïve) horses as a negative control [14].

### Immunoblotting

Immunoblotting experiments were performed following the protocol described with modifications [14,39]. Venoms were first electrophoretically separated by SDS-PAGE (12.5% gel) and then transferred to a nitrocellulose membrane at 25 V and 2.5 A for 7 min, following the manufacturer’s protocol (BioRad, USA). Ponceau S reversible stain was used for assessing the transfer efficiency, following which the non-specific regions on the membrane were blocked overnight with 5% skimmed milk in TBST at 4° C. This was followed by six TBST washes over a period of an hour, before an overnight incubation at 4° C following the addition of the respective polyvalent antivenom at a 1:200 dilution in the blocking buffer. HRP-conjugated, rabbit anti-horse secondary antibody was added at a dilution of 1:2000 following six TBST washes to remove unbound antivenom. The binding of antivenom to venom was captured by the addition of enhanced chemiluminescence substrate as per the manufacturer’s instructions (Thermo Fisher Scientific, USA) and imaged in an iBright CL1000 (Thermo Fisher Scientific, USA).

### *In vivo* venom toxicity and antivenom efficacy assays

To evaluate the pan-India toxicity profiles of *N*. *naja* venoms, and the preclinical efficacy of currently marketed Indian antivenoms against the lethal venom effects, we conducted *in vivo* neutralisation assays in murine models.

### The intravenous median lethal dose (LD_50_)

The potency of the venom sample corresponding to a biogeographic zone was determined by calculating the LD_50_ or the amount of venom required to kill 50% of the test population of mice [40]. In brief, five different venom concentrations were prepared in physiological saline (0.9% NaCl), followed by the intravenous injection into the tail vein of the mice (500 μl/mouse). Five CD-1 mice in the weight range of 18–22 g were used per group, with one control group receiving normal saline alone. Following injection, mice were kept under observation for 24 hours, and the number of dead and surviving animals recorded for the calculation of LD_50_ values using Probit statistics [41].

### The median effective dose (ED_50_)

The preclinical efficacy of an antivenom, in effect, its capability to neutralise the lethal systemic effects of snake venom, can be evaluated by calculating the ED_50_ value, which is defined as the minimum amount of antivenom required to protect 50% of mice injected with lethal doses of venom [40]. For these experiments, we used the Premium Serums antivenom, as this product was found to recognise the *Naja* venoms to a greater extent than all of the other Indian marketed antivenoms, as determined by our *in vitro* assays. We chose to test only the most promising of the marketed Indian antivenoms in these experiments to reduce the burden of suffering on experimental animals. Venom doses equivalent to five LD_50_ determined in the experiments above were used as the ‘challenge dose’. Different volumes of antivenom were mixed with the challenge dose of venom, followed by an incubation period of 30 min at 37° C. Immediately after incubation, each venom-antivenom mixture (n = 4 per venom) was intravenously injected into a group of five male CD-1 mice (18–22 g). A group of five male mice injected with 1× LD_50_ of venom, served as the positive control. The ED_50_ values of the antivenom against each venom were calculated using Probit statistics [41]. Antivenom neutralisation potency was calculated as described before [14,42].

$$Antivenom\;neutralisation\;potency\;(mg/ml)=\frac{\left(n-1){\times LD}_{50}\;of\;venom\;(mg/mouse\right)}{{ED}_{50}\;(ml)}$$

Here, n is equal to the number of LD_50_ used as the challenge dose.

### Statistical analysis

One-way ANOVA and Two-way ANOVA with Tukey’s and Dunnett’s multiple comparison tests were used for the statistical comparisons of biochemical assays and ELISA results, respectively, and were performed in GraphPad Prism (GraphPad Software 8.0, San Diego, California USA, www.graphpad.com).

## Results

### Venom proteomics

**Fig 1:**
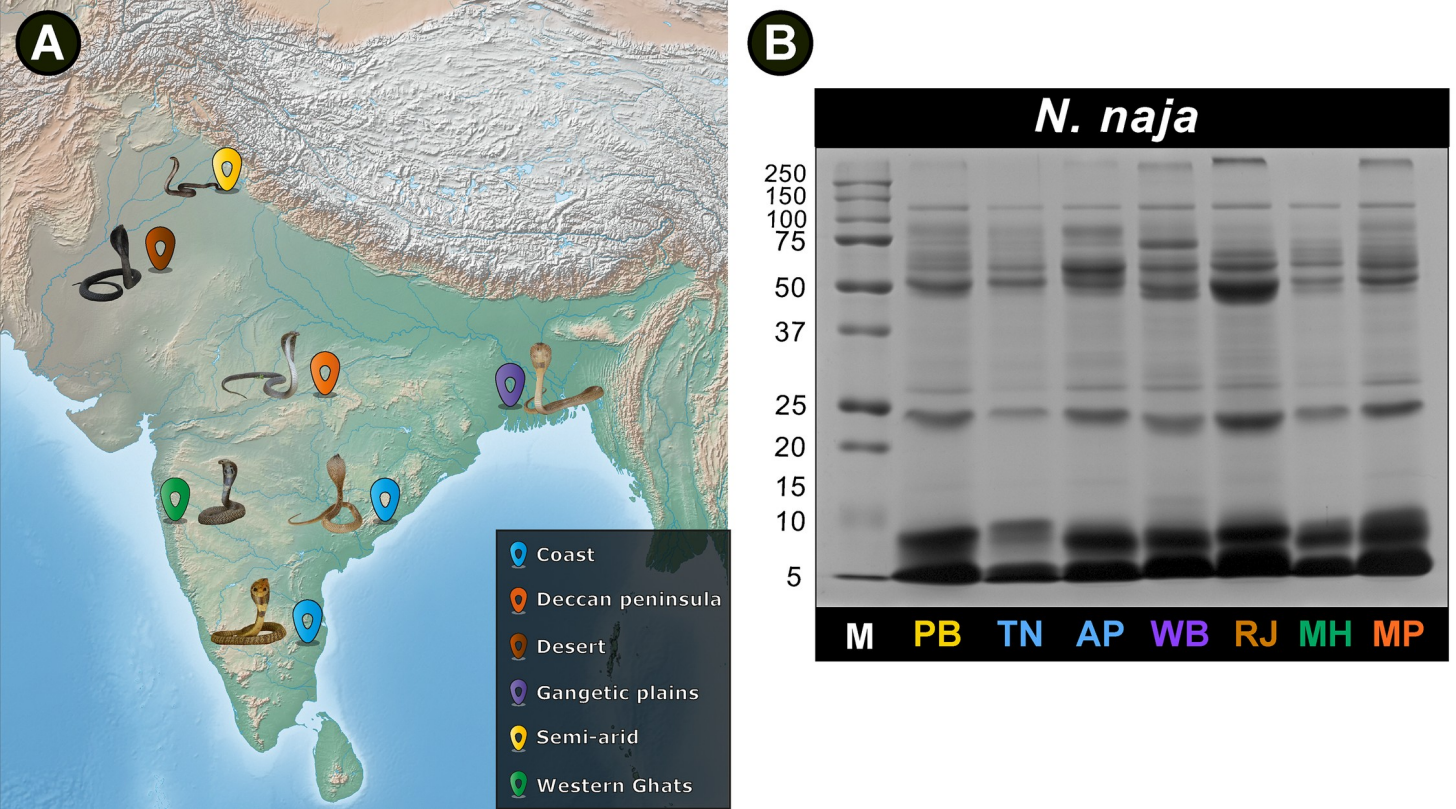
Sampling locations and SDS-PAGE profiles of *N*. *naja* venoms from distinct biogeographic zones of India. This figure depicts (A) the venom sampling locations across distinct biogeographic zones of India and (B) SDS-PAGE profiles of *N*. *naja* venoms under reducing conditions. **M:** Protein marker (units in kDa); **PB:** Punjab; **TN:** Tamil Nadu; **AP:** Andhra Pradesh; **WB:** West Bengal; **RJ:** Rajasthan; **MH:** Maharashtra; and **MP:** Madhya Pradesh. The map of India shown here was prepared with QGIS 3.8 [43].

**Fig 2:**
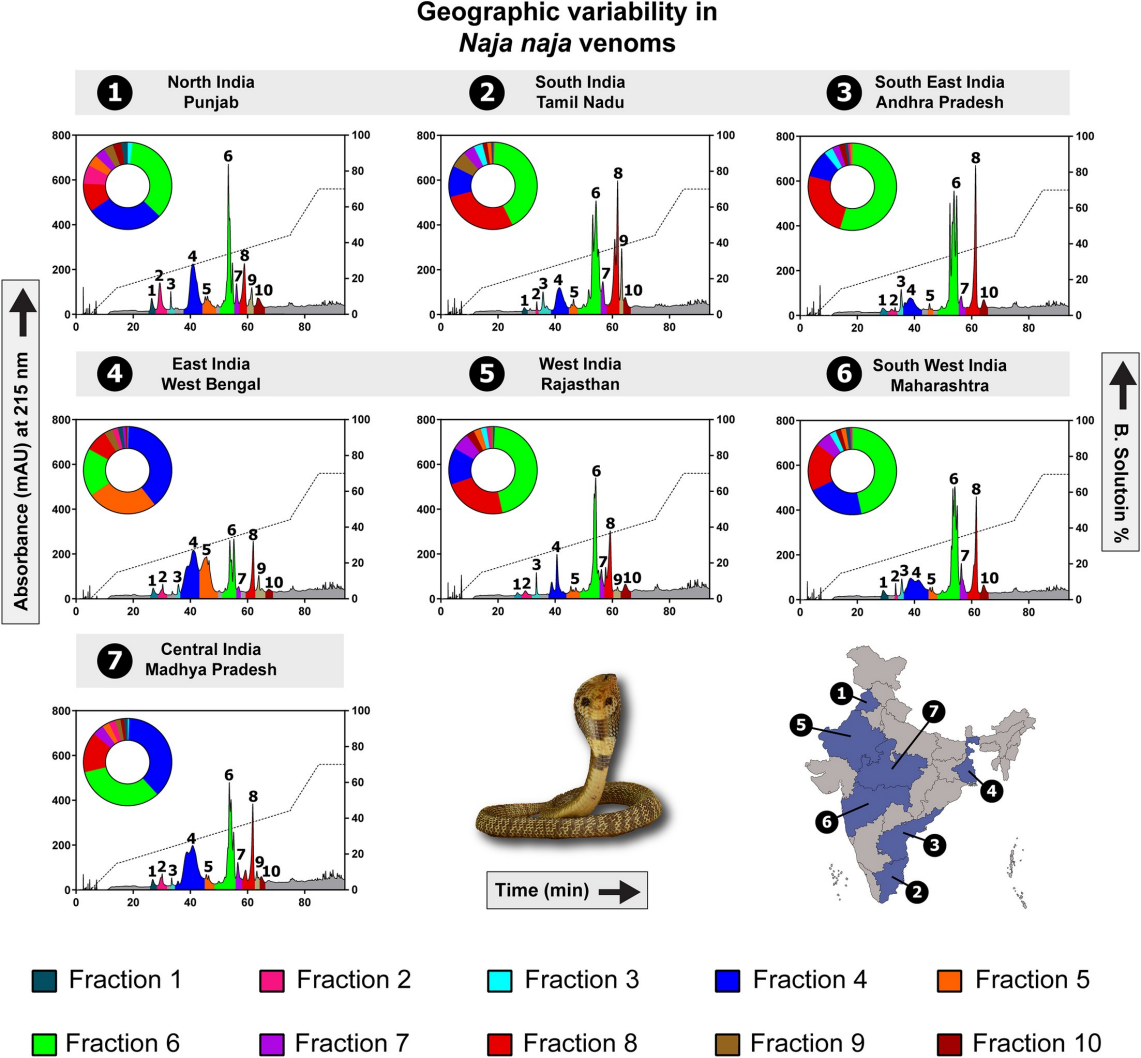
Biogeographic venom variability in *N*. *naja*. HPLC profiles of *N*. *naja* venoms from various biogeographic zones of India are depicted here. A plot of absorbance values (mAU) at 215 nm against retention time (min) highlights the dramatic variation in the pan-Indian populations of this species. The doughnut charts are based on the area under the curve of the respective fractions (uniquely encoded with colours and numbers).

The proteomic profiles of *N*. *naja* venoms collected from seven populations in six distinct biogeographical zones across India (Fig 1A and S1A Table) were elucidated using SDS-PAGE and RP-HPLC. In addition, three populations [i.e., the semi-arid (Punjab: PB), Gangetic Plains (West Bengal: WB) and desert (Rajasthan: RJ) populations] were selected based on their unique HPLC and toxicity profiles and were subjected to tandem mass spectrometry. While SDS-PAGE profiles revealed molecular weights of toxins and the primary differences in the composition of venom proteins between populations (Fig 1B), finer differences in venom composition were unravelled by RP-HPLC (Fig 2). To identify venom components in each fraction, we further subjected individual fractions to LC-MS/MS. Differences were not only noted in the number of fractions shared between populations but also in their intensities, which corresponds to protein abundances.

Mass spectrometry of venom fractions identified between 48 to 59 non-redundant toxin proteins from 11 toxin families in the pan-Indian populations of *N*. *naja* (S2A–S2C Table and S1 Data). Tandem mass spectrometry identified a plethora of toxin protein families including three-finger toxin (3FTx), cobra venom factor (CVF), phospholipase A_2_ (PLA_2_), Kunitz-type serine protease inhibitor (Kunitz), cysteine-rich secretory proteins (CRISP), snake venom metalloproteinase (SVMP), nerve growth factor (NGF), L-amino-acid oxidase (LAAO), 5’-nucleotidase, vespryn and cystatin in the venoms of *N*. *naja* (S2A–S2C Table and S1 Data).

**Fig 3:**
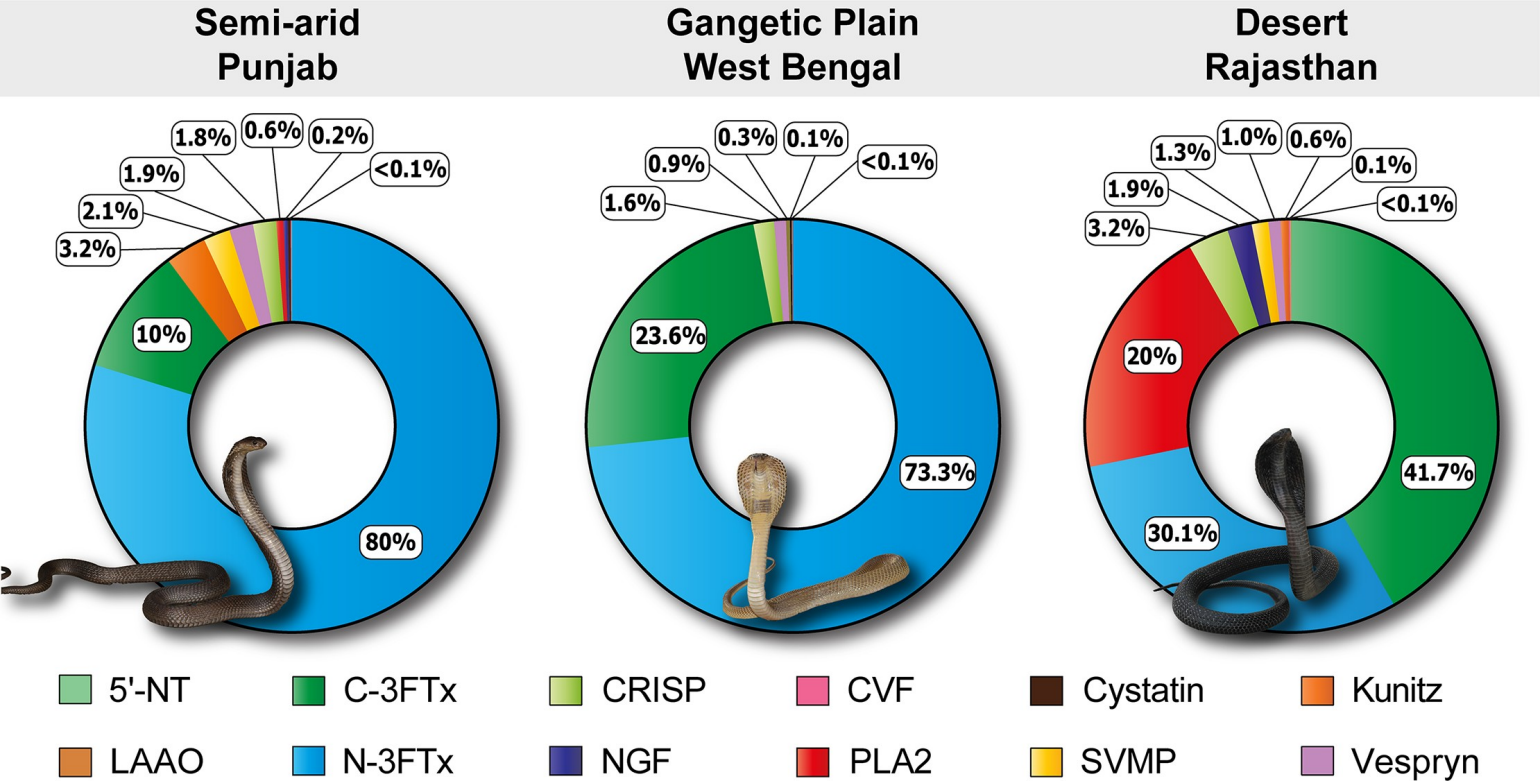
Proteomic compositions of *N*. *naja* venoms from various biogeographic regions. Doughnut charts depicting the relative abundances of various toxins comprising the venoms of *N*. *naja* are presented here. Individual toxins are colour coded, and their relative abundances are indicated in percentages.

3FTx are a major family of functionally diverse low molecular weight toxins (6–9 kDa) that target a wide range of receptors and ion channels [44–46]. 3FTxs were identified as the most abundant venom protein family in all populations of *N*. *naja* across the Indian subcontinent. They are abundantly secreted in the venoms of most Elapidae snakes and are known to inflict a plethora of toxic effects in bite victims, including neurotoxicity, cytotoxicity, anti-platelet activity and cardiotoxicity [44,47–50]. Here, we detected major differences in the amounts of neurotoxic 3FTx (N-3FTx) between the pan-Indian populations of *N*. *naja* (Figs 2 and 3). Mass spectrometric analyses revealed that, while this toxin type constituted 80% and 73.3% of the venom profiles of semi-arid (PB) and Gangetic plain (WB) populations, respectively, only ~30% of the venom was comprised of N-3FTx in the desert (RJ) population of *N*. *naja* (Fig 3). In contrast, the desert population (RJ) secreted 2 to 4 times more cytotoxic/cardiotoxic 3FTxs (C-3FTx; 41.7%) in comparison to the Gangetic Plain (WB) and semi-arid (PB) populations (23.6% and 10%, respectively; Fig 3). Interestingly, we observed that the abundance of PLA_2_ also varied significantly between populations (0.04 to 20%). While the abundance of PLA_2_ in the desert population (RJ) was in line with the literature [18], the relatively lower abundances in the semi-arid (PB) and Gangetic Plain (WB) populations highlight the remarkable biogeographic variations in the venoms of *N*. *naja*. Furthermore, minor differences were observed in the abundance of CRISP (1.6 to 3.2%), vespryn (0.94 to 1.9%), SVMP (1.3 to 2.1%), Kunitz (0.05 to 3.2%) and NGF (0.13 to 1.9%) across populations (Fig 3 and S2A–S2C Table). In contrast to previous reports [14,23,24], we detected limited amounts of CVF (<0.001 to 0.11) in these populations. Another noteworthy discovery was the identification of the PLA_2_ inhibitor (PLI) (Uniprot ID: Q7LZI1) from the Gangetic Plain (WB) population of *N*. *naja*. PLIs have been previously identified in the blood of several snake species, and are implicated in preventing self-envenomation [51].

### Venom biochemistry

Snakebite victims often present with a wide range of symptoms post-envenomation owing to the compositional and functional diversity of toxins. In order to understand the biochemical roles of toxins and the pharmacological implications associated with snakebite, we conducted several enzymatic (PLA_2_, protease, LAAO, DNase, fibrinogenolytic and haemolytic) and pharmacological (PT and aPTT) assays.

### Phospholipase A_2_ (PLA_2_) assay

Venoms of both elapid and viperid snakes are enriched with PLA_2_s, which are amongst the most important snake venom toxin superfamilies [52]. The clinical manifestations resulting from these may vary, depending on the relative abundance and types of PLA_2_ toxins present in the venom [53]. Therefore, to evaluate the catalytic activities of venom PLA_2_s, we conducted enzymatic assays on the venoms of geographically disparate *N*. *naja* populations. These experiments revealed low to negligible differences (p > 0.05) in PLA_2_ activities between these populations (S1 Fig), despite the observed proteomic variation.

### Snake venom protease and L-amino acid oxidase (LAAO) assays

Despite being secreted in limited amounts, elapid venom protease (SVMP and Snake Venom Serine Protease) and LAAO venom proteins may contribute to toxicity by exerting a variety of pharmacological effects. SVMPs in Elapidae snakes have been shown to affect haemostasis by inhibiting the aggregation of platelets [54]. Similarly, SVSPs too could interfere with the clotting cascade by exhibiting thrombin- and plasminogen-like activities [55–57]. In addition to fibrinogenolysis, kallikrein-like SVSPs are also known to affect the blood pressure by inducing the release of hypotensive bradykinin [58]. On the other hand, LAAO is responsible for cytotoxicity, cell death, haemorrhage and inhibition of platelet aggregation [59–61]. When crude venoms of *N*. *naja* were assayed for their ability to cleave azocasein, none of the populations showed significant activity (p > 0.05), consistent with the low abundance of venom proteases (SVSP and SVMP) in these venoms (S1 Fig). In contrast, all populations of *N*. *naja* oxidised the L-leucine substrate and exhibited significant intrapopulation differences (p < 0.05; S1 Fig). However, further investigations are required to understand the precise biological and pharmacological consequence of such difference in activities.

### DNase assay

Post-envenomation, the nuclear material released by the lysis of various cells of the bite victim (e.g., neutrophils) can form an extracellular mesh to restrict toxins from entering circulation [62]. DNases present in venoms of certain snakes have been shown to actively destruct these extracellular traps by enzymatic cleavage of the nucleotides [63,64]. Considering this, we performed DNase assays on the venoms of *N*. *naja* from various biogeographic zones. Not surprisingly, all populations of *N*. *naja* were found to exhibit very high DNase activities (64–100%), higher than even the purified DNase I from bovine pancreas, which served as the positive control (~78%) (S2 Fig).

### Fibrinogenolytic assay

Fibrinogen is a precursor that undergoes catalytic activation into fibrin, which, in turn, initiates clot formation upon injury. Many snake venoms are known to affect haemostasis by cleaving fibrinogen, which can, in turn, help to prolong haemorrhage caused by other toxins [65]. Therefore, we evaluated the ability of *Naja* venoms to induce fibrinogenolysis. Human fibrinogen, which was used as a substrate in this assay, consists of three subunits—Aɑ, Bβ and γ—and all are crucial for fibrin clot formation. Venoms from all populations of *N*. *naja* exhibited complete degradation of the Aα subunit following incubation for an hour (S3 Fig), while the Bβ- and γ-chains of human fibrinogen were unaffected. This contrasts with previous findings showing that the eastern *N*. *naja* population exhibits negligible effects on human fibrinogen [17].

### Coagulation assay

**Fig 4:**
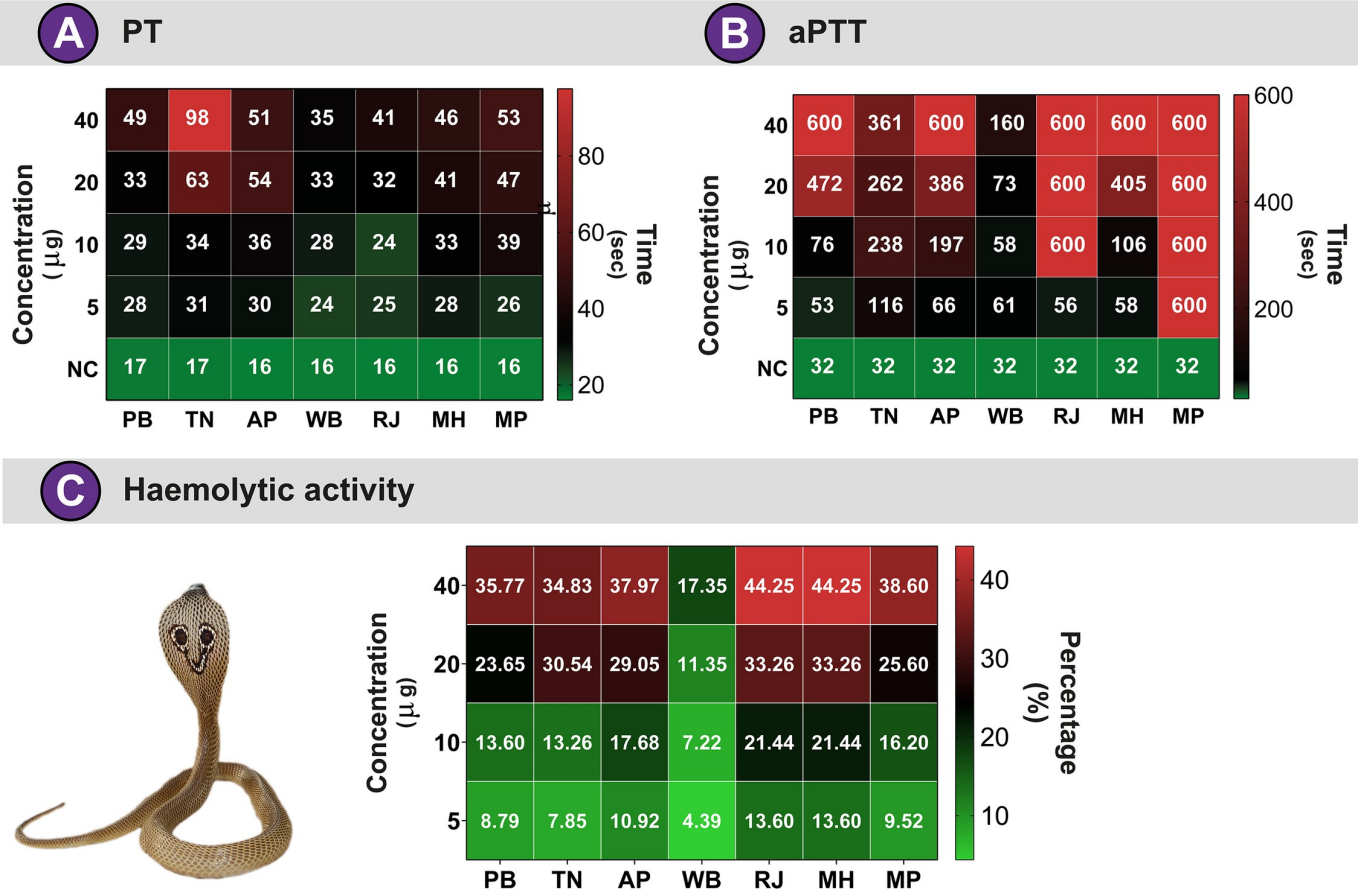
Pan-Indian *N*. *naja* venom-induced coagulopathies. The abilities of venoms of various populations of *N*. *naja* to cause perturbations to the blood coagulation cascade via extrinsic **(A)** and intrinsic **(B)** pathways are depicted here as heatmaps. Numbers inside cells indicate the time (sec) required for the formation of the first fibrin clot. A colour key representing time in sec is also provided for each heatmap. Haemolytic activities of *N*. *naja* **(C)** venoms, defined as the percentage relative activity of the positive control (0.5% Triton X), are also shown.

Snake venom proteins can disrupt homeostasis by affecting various components of the blood coagulation cascade [58,65,66], including proteolytic snake venom toxins that act on factors that activate or inactivate either the intrinsic or extrinsic pathways [67]. As such toxins have the potential to alter the clinical outcome of envenomation significantly, we evaluated the abilities of pan-Indian *Naja* venoms to disrupt the coagulation cascade. We used measures of the PT to test for perturbations in the extrinsic pathway, and the aPTT for the intrinsic pathway of blood coagulation (Fig 4A and 4B). In line with previous findings described for many *Naja* species [17,22,68], venoms of all *N*. *naja* populations were found to exhibit potent anticoagulant properties as they mostly affected the intrinsic coagulation cascade (aPTT; Fig 4B). Interestingly, only one of the coastal populations (Tamil Nadu: TN) was found to affect the extrinsic cascade (PT), as it delayed blood coagulation by 81 sec at a very low venom concentration (40 μg) but had relatively lower effects on the intrinsic coagulation cascade (Fig 4A and 4B). Most other populations of *N*. *naja*, including the semi-arid (PB), desert (RJ), Western Ghats (Maharashtra: MH), Deccan plateau (Madhya Pradesh: MP) and the other coastal population (Andhra Pradesh: AP), significantly affected the intrinsic coagulation pathway and delayed blood coagulation by ~568 sec at the 40 μg venom concentration (Fig 4B). Among these, the Deccan plateau (MP) population was found to be the most potent anticoagulant as it achieved strong anticoagulatory effects at the 5 μg venom concentration, closely followed by the desert population (RJ; 10 μg). Interestingly, the Gangetic Plain (WB) population neither significantly affected the intrinsic, nor the extrinsic blood coagulation cascades (Fig 4A and 4B). Considering the limitation of the coagulometer in recording clotting time beyond 600 sec, and the ability of *N*. *naja* to inject very large amounts of venom in a single bite [on average ~300 mg of venom was obtained from 18 *N*. *naja* individuals across biogeographic zones, with as much as 413 mg from a single individual in the Deccan plateau (MP) region], it is very likely that these outcomes grossly underestimate the true anticoagulatory potential of *Naja* venoms.

### Haemolytic assay

Snake venom toxins inflict various pharmacological effects that disrupt homeostasis. Secretory PLA_2_s, for example, are known to cause cytotoxicity, myotoxicity, neurotoxicity, hypoxia and platelet aggregation [53,69–72]. In addition, PLA_2_s are also known to cause haemolysis by hydrolysing phospholipid molecules of the cellular membrane [73]. The breakdown of RBC by venom PLA_2_s results in oxidative stress and inflammation, further accelerating tissue damage and necrosis [74,75]. While this effect can also be induced by C-3FTx [76], PLA_2_s are known to enhance the haemolytic potential of snake venoms [77–79]. When assayed for the ability to break down erythrocytes, various populations of *Naja* venoms showed differing degrees of haemotoxicity that were concentration-dependent (Fig 4C). At the highest concentration tested (40 μg), *N*. *naja* venom from the Gangetic Plain (WB) showed the least activity (17%), while the highest activity was observed in the Western Ghats (MH) and the desert (RJ) populations (44% of the positive control for both; Fig 4C).

### Immunological cross-reactivity between commercial Indian antivenoms and *Naja* venoms

**Fig 5:**
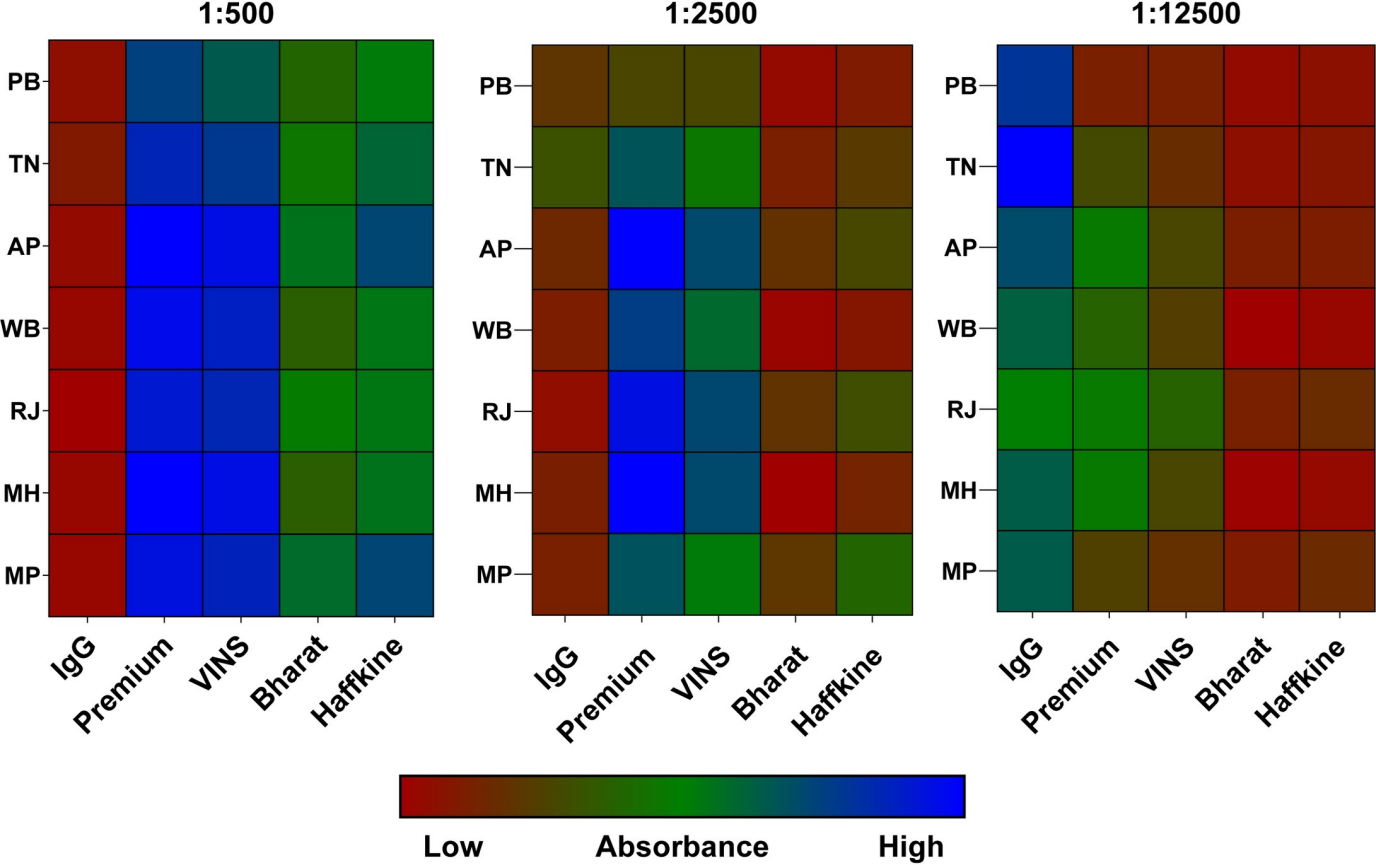
The immunological cross-reactivity of commercial Indian antivenoms against *Naja* venoms. Quantification of antibody binding of various commercial Indian antivenoms and naive horse IgG to the various snake venoms, determined by ELISA. Absorbance was measured at 405 nm for various dilutions (1:500, 1:2500 and 1:12500) of the antivenom, and the extent of binding shown as a colour gradient from red (low binding) to blue (high binding).

As the geographic variability in snake venom composition has been implicated in influencing the efficacy of antivenoms [13,14,16,80], the immunological cross-reactivities of four major commercial Indian antivenoms against *N*. *naja* venoms from various populations were assessed using endpoint ELISA and immunoblotting experiments. In endpoint ELISA, varying dilutions of antivenoms were incubated with a fixed concentration of venom, and the absorbance values at 405 nm, which directly correlate to the amount of antivenom antibody-venom protein binding, were plotted (S4 Fig). In these experiments, the Premium Serums antivenom consistently outperformed its comparators in recognising the venoms of the pan-Indian populations of *N*. *naja* (end-point titres between 1:2500 to 1:12,500), followed by VINS (Figs 5 and S4; p < 0.05). In contrast, antivenoms manufactured by Bharat Serums and Haffkine poorly recognised various *N*. *naja* venoms found across India (p < 0.05). Interestingly, the Haffkine antivenom failed to exhibit high binding titres against the Maharashtra snake population that it is manufactured against (i.e., used as venom immunogen). Similarly, western blotting experiments revealed that several venom components were unrecognised or exhibited low levels of antibody binding when probed with the Bharat Serums and Haffkine antivenoms (S5A and S5B Fig). Overall, both VINS and Premium Serums antivenoms exhibited increased recognition of venom proteins, with the latter being relatively better than all other tested antivenoms in terms of both end-point titres and absorbance values (p < 0.05). Incidentally, the naïve horse IgG exhibited a degree of non-specific cross-reactivity against the largely abundant toxins found in the high (25–50 kDa) and low (<10 kDa) molecular weight ranges (S5A and S5B Fig). These findings suggest that a degree of non-specific binding occurs between the equine antibodies and the venom proteins, in line with previous work [14]. It should be noted that the low molecular weight toxins (e.g., 3FTx) are known to exhibit poor immunogenicity [81–83]. Hence, it is very likely that this further contributes to the lack of low molecular weight toxin specific antibodies.

### Venom potency by median lethal dose (LD_50_)

**Fig 6:**
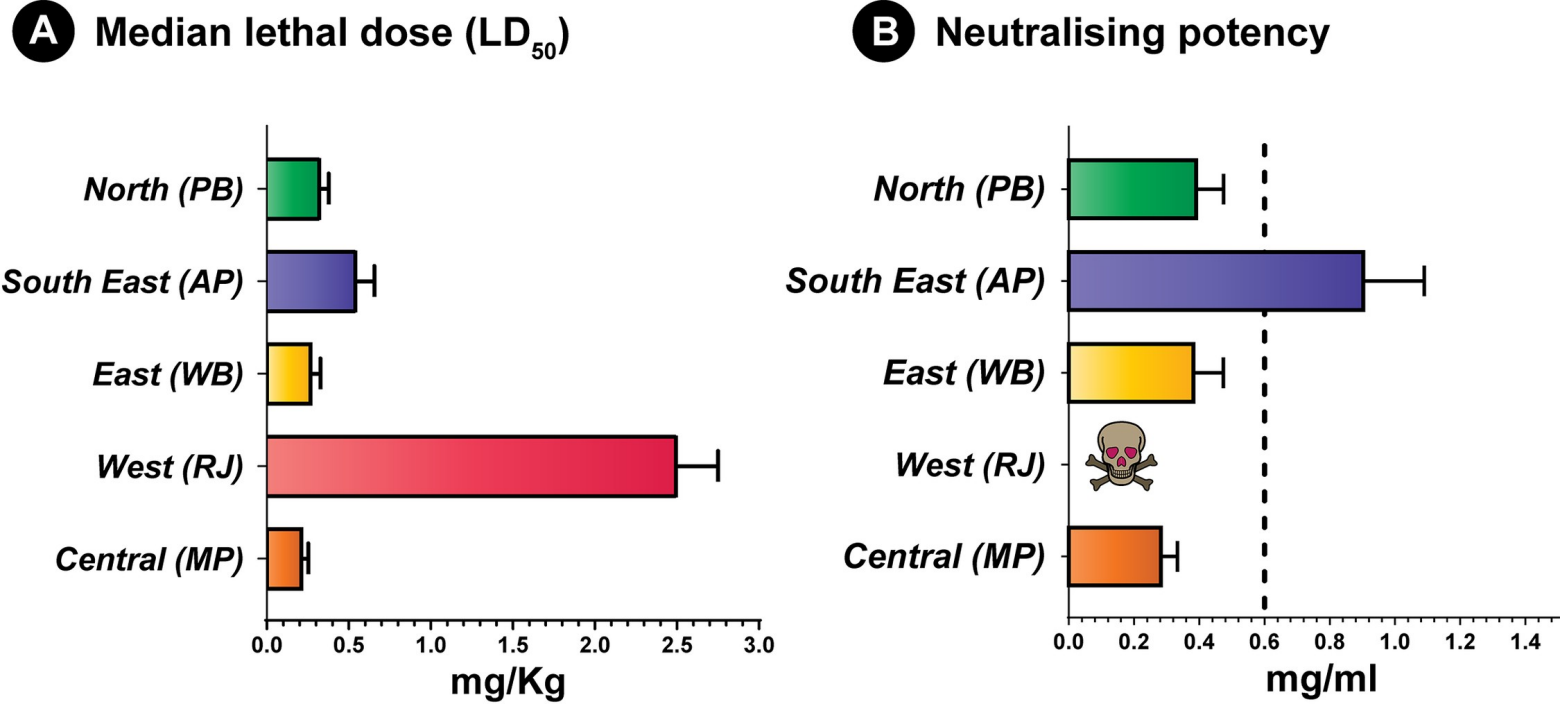
Toxicity profiles of *N*. *naja* from various biogeographic zones across India, and the neutralisation potencies of commercial Indian antivenom against these venoms. Murine intravenous median lethal doses (expressed in mg/kg) of various populations of *N*. *naja* venoms (A) and the neutralising potencies (expressed in mg/ml) of the Premium Serums commercial antivenom against these venoms (B). The vertical dotted lines in panel B indicate the marketed neutralising potency (0.60 mg/ml) of commercial antivenoms against the *N*. *naja* venom.

Snake venom compositions are predominantly shaped by the ecology and environment. Resulting compositional differences in venoms, as a result of local adaptations, can significantly alter the clinical pathogenesis observed in human snakebite victims. While investigating the lethal effects of the *N*. *naja* venoms sourced from various Indian biogeographic populations, fascinating observations were made (S3A Table). While the Deccan plateau (MP: 0.22 mg/kg), Gangetic plain (WB: 0.27 mg/kg) and the semi-arid (PB: 0.33 mg/kg) populations of *N*. *naja* venoms were determined to be extremely toxic to mice, the desert population (RJ: 2.53 mg/kg) proved to be dramatically less toxic (Fig 6A). In addition, the venom of one of the coastal populations (AP: 0.55 mg/kg) was found to exhibit relatively lower venom potencies (Fig 6A).

### Antivenom efficacy via median effective dose (ED_50_)

Considering that the Premium Serums antivenom exhibited the highest *in vitro* venom recognition of the various marketed antivenom products tested in this study (S1B Table), we selected this antivenom for *in vivo* venom neutralisation experiments. Despite this best-case scenario, the results of our preclinical ED_50_ experiments highlighted poor pan-India venom neutralisation efficacies of this product, with the estimated neutralising potencies observed well below that of the marketed claims of neutralisation (0.6 mg/ml for *N*. *naja;* S3B Table). With the exception of *N*. *naja* venom from the coastal population in Andhra Pradesh (0.80 mg/ml), the Premium Serums antivenom exhibited extremely low neutralising potencies against the lethal effects of the venoms of all other biogeographical populations of this species (0.28 to 0.38 mg/ml; Fig 6B). Alarmingly, this antivenom was found to be completely ineffective at protecting mice envenomed with 5× LD_50_ of venom from the desert population (RJ) of *N*. *naja*, as even the highest antivenom doses tested (166.66 μl) failed to protect the experimental animals from the lethal effects of the venom. However, when the venom challenge dose was reduced to 3× LD_50_, a neutralising potency slightly greater than that marketed (0.74 mg/ml), was observed. It should be noted that the amount of venom injected by individual *N*. *naja* snakes can be very large (300 mg on average).

## Discussions

### Geographic variability in venom complexity and potency is dictated by differing ecologies and environments

From a biogeographical perspective, India can be divided into ten zones: 1. Himalayas; 2. Trans-Himalayas; 3. Semi-arid regions; 4. Desert; 5. Western Ghats; 6. Deccan plateau; 7. Gangetic plains; 8. Coasts; 9. Northeast India; and 10. Islands [84]. The remarkable adaptability of *N*. *naja* is illustrated by its broad distribution across complex climatic conditions, including hot and dry semi-arid and arid regions, tropical monsoon forests, hot and humid coastline, and the fertile Gangetic plains. There are scarce reports of *N*. *naja* in northeastern India, albeit from only the northern parts of West Bengal and southern Assam [85]. However, none of the Indian ‘big four’ snake species are found in the Trans-Himalayas and the Andaman and Nicobar Islands. Despite such biogeographical variation, the influence of distinct ecologies and environment on the venom composition of *N*. *naja* has not been previously investigated. To address this shortcoming, venom samples from the pan-Indian populations of *N*. *naja* were collected from six of the seven biogeographic zones of India inhabited by this species.

Proteomic characterisation unveiled dramatic differences in the venom compositions of snakes from distinct biogeographical zones. For example, the venoms of *N*. *naja* showed remarkable differences in relative amounts of 3FTx subtypes. Among the pan-Indian populations of *N*. *naja*, the semiarid (PB) and Gangetic Plain (WB) populations secreted the highest amounts of N-3FTx in their venom. In contrast, the venoms of the desert (RJ) population secreted relatively limited amounts of the N-3FTx, while largely being composed of cytotoxic/cardiotoxic 3FTXs (Fig 3 and S2A–S2C Table). Large amounts of C-3FTxs have also been previously reported from the venoms of captive snakes sourced from Western India (Rajasthan and Gujarat) [18]. Similarly, the venom of the Western Ghats (MH) population was previously reported to be comprised of ~42% N-3FTx [14]. Such considerable differences in the amounts of neurotoxins were found to significantly influence venom potencies towards mice (Fig 6A and S3A Table). Populations with large amounts of neurotoxins, such as the semi-arid (PB) and Gangetic Plain (WB), were characterised by increased venom toxicities (LD_50_: 0.33 and 0.27 mg/kg, respectively), whereas the Western Ghats (MH) and desert (RJ) populations were characterised by relatively lower lethal potencies [LD_50_: 0.73 and 2.53 mg/kg, respectively; [14]]. A correlation between the amounts of N-3FTx and venom potency has also been reported before in the Southeast Asian *Naja* spp. [86]. Although the prey spectrum of various Indian populations of *N*. *naja* is poorly understood, the extremely low potency of the desert population towards mice (2.53 mg/kg) could be perceived as indicative of non-mammalian prey animals chiefly featuring in the diet of this population. However, since the desert population (RJ), despite having the least potent venom, caused murine lethality much more rapidly (15 min) than all other populations (45–60 min), it may suggest the reliance of this population on cytotoxic/cardiotoxic 3FTxs that constituted a large portion of the venom. Unlike this arid population, *N*. *naja* from semi-arid (PB) regions secreted neurotoxic 3FTxs in abundance and, hence, required minuscule amounts of the venom to inflict respiratory failure in mice. Thus, albeit requiring different amounts of venom, both strategies seem to be equally effective in capturing prey in harsh, arid environments. In contrast to the desert population (RJ), the extreme potency of *N*. *naja* in the Deccan plateau region (MP: 0.22 mg/kg), and their ability to inject large amounts of venom (as high as 413 mg), makes them one of the most medically important ‘big four’ snake populations in the country. While neurotoxins affect the nervous system of prey animals, C-3FTxs induce cell necrosis and apoptosis by inflicting pores on the phospholipid membrane [87,88]. Not surprisingly, in cell viability assays, the C-3FTx-rich western Indian populations (desert and the Western Ghats) exhibited the highest haemolytic activity, while the neurotoxin-rich eastern Indian population (Gangetic Plains) was the least haemotoxic (Figs 3 and 4C). Thus, the compositional and biochemical venom variation observed here has the potential to result in pathological variation in cobra snakebite victims found across different regions of India.

### Venom pathology of *N*. *naja* is driven by complex synergistic actions

Snake venom is a concoction of diverse biochemical components that often work synergistically to facilitate effective prey capture [79,89–91]. Various enzymatic toxins, such as hyaluronidase and DNase, are known to function as ‘spreading factors’ [63,92]. Upon envenomation, the cells of the host are lysed by cytolytic toxins (e.g., C-3FTx and PLA_2_), resulting in cell death and the extrusion of nuclear DNA. The released genetic material, in turn, ensnares venom components into extracellular traps that function as barriers, thereby restricting the venom from accessing the blood circulation [63,64]. In order to overcome this barrier, *N*. *naja* seemingly employs DNase enzymes that catalyse the breakdown of the traps and facilitate the rapid spread of venom to the other parts of the body. Elapidae and Viperidae snakes employ distinct strategies for killing their prey, with many elapid snakes secreting venoms enriched with neurotoxins, while most viperid venoms predominantly contain components that cause haemodynamic alteration, local tissue necrosis, and myotoxicity [93,94]. Therefore, an increase in DNase activity could confer an evolutionary advantage to elapid snake venoms, as it may enhance the diffusion of neurotoxic components. In support of this hypothesis, we observed that the populations with increased amounts of N-3FTxs also exhibited the highest DNase activities (S1 and S2 Figs).

### Biogeographic venom variability negatively impacts upon snakebite therapy

The polyvalent antivenoms available for the treatment of snakebites in India have been historically manufactured from the venoms of the south Indian (Tamil Nadu) population of the ‘big four’ snakes. When the Premium Serums commercial antivenom, which exhibited relatively increased *in vitro* venom cross-reactivity in comparison with the other antivenoms under investigation, was tested for its *in vivo* efficacy against venoms from the pan-Indian populations of *N*. *naja*, alarming results were observed (Fig 6B and S3B Table). Among the five investigated populations of *N*. *naja*, only the venom from the coastal region (Andhra Pradesh, the neighbouring state to Tamil Nadu) was neutralised at a dose comparable to the marketed therapeutic potency (0.80 mg/ml). While the antivenom was able to neutralise the toxic effects of the highly neurotoxic venoms from the Gangetic Plain (WB), semi-arid region (PB) and the Deccan plateau (MP), very high doses were required, and thus the neutralising potency was well below the marketed efficacy (0.28 to 0.38 mg/ml). Even more concerningly, the antivenom completely failed to neutralise the lethal effects of the less toxic venom sourced from the desert population (RJ), despite exhibiting a binding efficiency that was comparable to the efficiency exhibited towards the coastal (Andhra Pradesh) population (Figs 5 and 6B and S3B Table). Interestingly, similar observations were recently described for the cytotoxin/cardiotoxin-rich venoms of monocled cobra (*N*. *kaouthia*) in northeast India (Arunachal Pradesh), where the overall venom potency was low, but the tested Premium Serums antivenom completely failed to neutralise the lethal effects in a murine model of envenomation [14]. These results are indicative of the presence of novel toxin isoforms that are currently unrecognised by the commercial Indian antivenom, which is exclusively produced against the southern population of ‘big four’ snakes. Ultimately, the *in vivo* venom neutralisation experiments performed here reveal disturbing deficiencies of the tested Indian antivenoms against most populations of *N*. *naja*. Despite exhibiting better *in vitro* binding compared to other commercial antivenoms, Premium Serums antivenom performed poorly under *in vivo* conditions. Given the relatively decreased venom recognition capabilities of the other commercial antivenoms tested in this study, and the identical strategies of antivenom production that involves sourcing of venom from a single population, it is highly unlikely that the other antivenoms will effectively neutralise the lethal effects of the distant *N*. *naja* populations. Furthermore, this interpretation is supported by preclinical antivenom efficacy testing (VINS and Bharat antivenoms) on *N*. *naja* venoms (population undisclosed) [95]. Thus, it is essential to alter existing antivenom manufacturing strategies to generate efficacious pan-Indian snakebite treatment.

### The road map to pan-India effective antivenoms

In contrast to the rapid acquisition of knowledge relating to the composition and diversification of snake venoms, antivenom manufacturing strategies have remained virtually unchanged over the past century. To improve the plight of India’s million snakebite victims, significant strategic changes are warranted in both the manufacturing and marketing of commercial Indian antivenoms.

### Immediate solutions: The development of region-specific antivenoms and the implementation of vital health policy decisions

**Fig 7:**
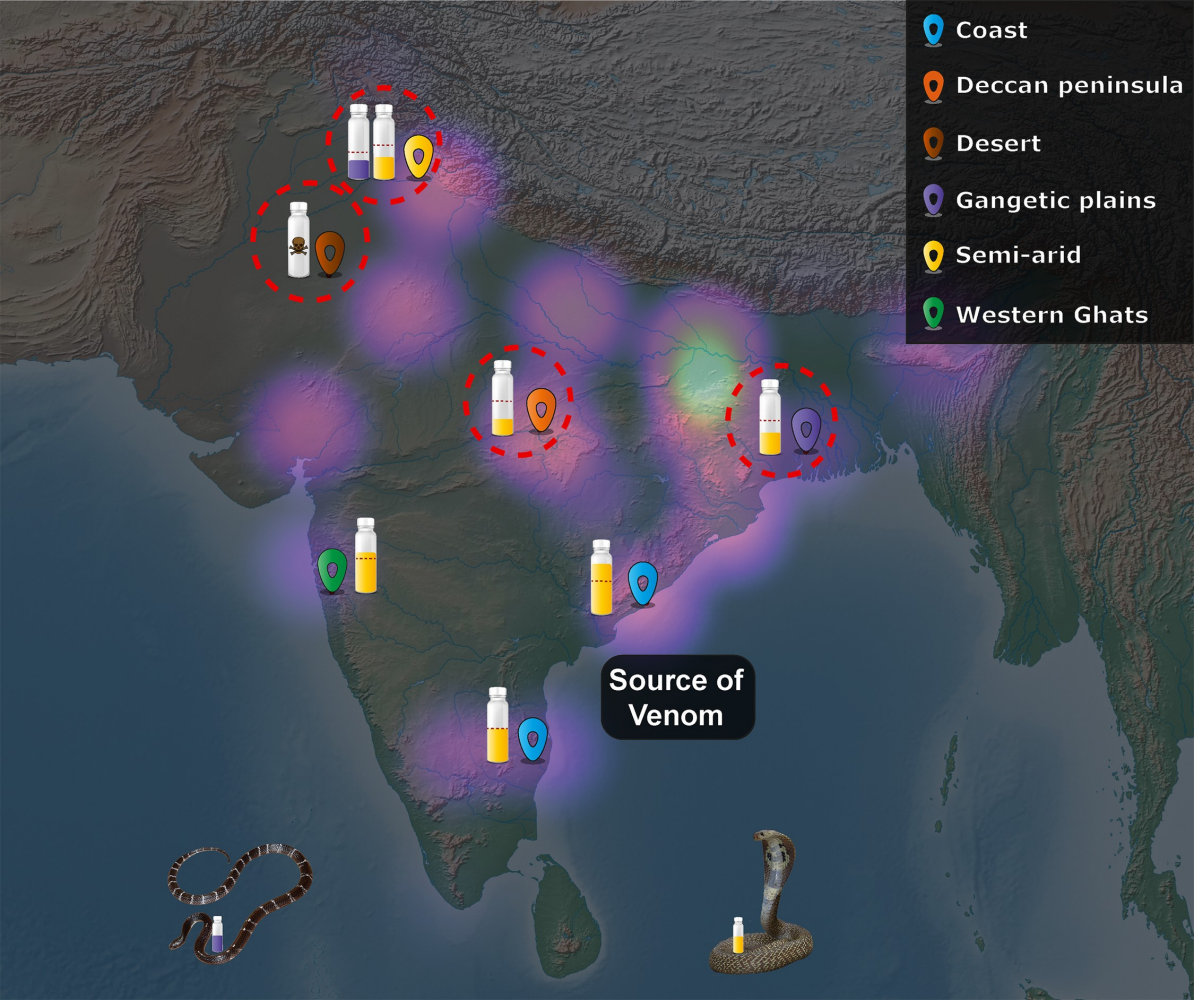
The negative impact of biogeographic venom variability on Indian snakebite therapy. This figure depicts the repercussions of biogeographic venom variability on snakebite treatment in India. Antivenom vials indicate the relative differences in neutralisation potencies against the geographically distinct populations of *N*. *naja* (yellow), and *B*. *caeruleus* (purple) in comparison to the source population in southern India, where the red dotted line on the vials represents the marketed neutralising potency of commercial Indian antivenoms. The intensity of purple clouds on the map is indicative of the estimated standardised snakebite death rates per million reported by Suraweera et al. 2020 [6], where the brighter regions represent the major hotspots. Geographical locales are defined by the box in the top right. The map of India shown here was prepared with QGIS 3.8 [43].

Commercial Indian antivenoms manufactured against the coastal Tamil Nadu population (TN) of snakes, which secrete a very distinct venom cocktail in comparison to conspecifics in other biogeographical regions, lack pan-India efficacy. In addition to providing evidence for the ineffectiveness against various populations of *N*. *naja* in this study, we have previously reported the inefficacy of the marketed antivenoms against the common krait (*B*. *caeruleus*) from Punjab [14]. This highlights the inability of the marketed antivenoms in neutralising venoms of two of the ‘big four’ snake species from the northern Indian region (Fig 7). This unfortunate outcome is a result of discounting the remarkable inter- and intra-specific venom diversity in snakes and producing a single antivenom for use across the large Indian subcontinent. Given the considerable biotic and abiotic diversity in India, and the remarkable geographic venom variability among snakes, the conventional antivenom is doomed to failure in regions with disparate populations of ‘big four’ and/or other distinct venomous snake species. An immediate solution to this problem could be the identification of medically important snakes by regions, i.e., consideration of both the ‘big four’ and the ‘neglected many’ (medically important yet neglected lineages of snakes), and the inclusion of their venoms in the immunisation mixture for formulating regionally-effective antivenoms. Based on the outcomes of this study, research on medically important yet neglected snakes [14,96], and the geographical distribution of ‘big four’ snakes, several Indian regions can be identified that would benefit from regional antivenoms: 1. North(west) India; 2. East India; 3. Northeast India; 4. Andaman and Nicobar islands; 5. Central India; and 6. South India.

Since Indian antivenoms have never undergone clinical validation through formal clinical trials, robust data on their efficacy and safety is currently unavailable. Given the potential for significant batch-to-batch variation and treatment failure due to venom variation, stringent evaluation of the preclinical efficacy of antivenoms, ideally by an independent external laboratory or at the very least the publication of manufacturer-generated data for independent assessment, should be mandatory prior to marketing [14,97,98]. Moreover, the license to sell commercial antivenoms in various Indian states is currently based on a tender system. Instead, licensure should be strictly based on the outcomes of such rigorous preclinical evaluation. In addition, the procurement and qualification guidelines for venoms used for immunisation during the manufacturing process should take into account the influence of various ecological and environmental factors on venom variability. Unfortunately, these factors are currently being ignored during the commercial manufacture of Indian antivenoms. For example, venoms that exhibit either very high or low potencies are generally not used in the immunisation process by many Indian antivenom manufacturers (KS, personal communication with manufacturers). This could explain the complete lack of neutralisation against the desert population of *N*. *naja* that exhibited very low potency in the murine model. Overall, in the absence of broadly neutralising next-generation antivenoms, these measures can help improve the efficacy of snakebite therapies in the country.

### The long-term solution: Innovation of broadly neutralising recombinant antivenoms

Immunisation of animals with crude ‘whole’ venoms that could potentially contain snakebite-irrelevant antigens, e.g., bacteria, viruses and/or other impurities, along with the environmental antigens that the immunised animals get exposed to over their lifetime, increases the proportion of non-toxin-specific redundant antibodies in the finished product. In addition to toxins that result in severe pathophysiology in humans, snake venom cocktails also contain venom components that target non-mammalian prey/predatory animals. Therefore, using crude venoms for immunisation results in the inclusion of antibodies against such medically unimportant toxins, and significantly lowers the proportion of therapeutically important IgGs in the marketed product. This, in turn, significantly increases the number of antivenom vials required to effect cure (typically >20 in India). Therefore, in addition to their inability to counter toxic effects of pan-Indian populations of snakes, conventional serum therapy is marred by other inadequacies, including dose inefficacy, inconsistent batch effectiveness, and the risk of inducing fatal anaphylaxis via the intravenous delivery of animal IgG. Several immunochromatographic techniques, such as immunoaffinity purification, which involves the down-selection of antibodies using antigenic baits [99], could also help in improving the concentrations of therapeutically relevant antibodies in the marketed product.

Although regionally-effective antivenoms could serve as an interim solution to address local variations in snake venom and species diversity, they would still suffer from the aforementioned limitations. Hence, the discovery of broadly neutralising recombinant antivenom offers a long-term solution for treating snakebites in India. Recombinant antibodies could be developed by various approaches and in different formats (e.g., monoclonal, oligoclonal, intact IgG, nanobodies, etc.), and could be human-derived or humanised, and engineered to specifically target clinically important toxins detected across distinct snake populations and species [97,98]. Thus, recombinant therapy has the potential to deliver many advantages over conventional antivenom therapy, including high dose efficacy, pan-Indian efficacy, and improved safety profiles. The cost of production is the only current limitation of recombinant therapy as this entirely depends on the number of neutralising antibodies in the commercial antivenom concoction. However, this could be overcome by discovering and engineering broadly effective/paraspecific antibodies. The recombinant expression of such broadly neutralising antibodies should therefore be strongly pursued as long-term replacements of conventional antivenoms to enable rural Indian communities to access safe and efficacious life-saving snakebite therapies.

### Limitations of the study

While a considerable amount of PLA_2_ (20%) was detected by tandem mass spectrometry of the *N*. *naja* venom from the desert population (RJ), very limited differences were noted in phospholipase activities of populations from distinct biogeographic regions. This could, indeed, result from an overestimation of PLA_2_ in the venom of the desert (RJ) population or an underestimation of this toxin superfamily in other populations. It should be noted, however, that these estimates are in line with the literature, where a similar abundance of PLA_2_ was reported for *Naja* venoms sourced from Rajasthan and Gujarat [18]. Moreover, as the prominent role of neurotoxic and cytotoxic 3FTxs in *Naja* envenomation has been very well-established, the differences in the lowly abundant PLA_2_ toxins are unlikely to affect the major interpretations and conclusions of this study. Consistently, SDS-PAGE analysis clearly shows the abundance of 3FTxs in the molecular weight range of 6–9 kDa in all *Naja* venoms [46]. Further, while our analyses recovered LAAO only from the desert population (RJ), acetylcholinesterase was not detected in any of the populations subjected to mass spectrometry. The inability to detect such minor components in *Naja* venoms is mostly due to the lack of well-characterised toxin sequences from the medically important Indian snakes in the public repositories, highlighting the importance of conducting venom gland transcriptomics studies of Indian snakes.

It should also be noted that given the limited approvals from the authorities and the logistic and financial constraints associated with sampling, venom samples could not be collected from multiple individuals of certain populations. For others, while venoms were pooled from multiple individuals and were subjected to preliminary quality screening, we selected individual venoms for assessing the influence of biogeography on snake venom composition and potency. Nonetheless, the results of our proteomic analyses (SDS-PAGE, HPLC and mass spectrometry) are consistent with the literature and agree with the reported overall venom compositions. Considering the possibility of individual variability, we do not claim that these results necessarily represent an entire population/region. Future investigations incorporating much larger sampling efforts, not just by collecting the venoms of four to five individuals from the same location as reported in the literature, but by sampling many snakes across multiple regions in a given biogeographic zone. Such studies may reveal further intrapopulation differences in venom compositions and activities, and the implications of such variation on the effectiveness of antivenoms.

## Conclusion

In conclusion, an array of *in vitro* and *in vivo* experiments performed in this study reveals significant intraspecific differences in the venom proteomic composition and toxicities of *N*. *naja* venoms across six distinct biogeographical regions in India. Although *in vitro* antivenom screening experiments revealed increased venom binding by the Premium Serums antivenom, in comparison to those manufactured by VINS, Bharat Serums and Haffkine, *in vivo* antivenom neutralising experiments revealed alarming efficacy shortcomings of India’s snakebite therapies. Antivenom was found to be incapable of effectively neutralising the venoms of most *N*. *naja* populations (four out of five populations), including failing completely to prevent against venom-induced lethality caused by the desert (RJ) population. These data highlight the complexity and importance of understanding intra-specific venom variation and the impact that it can have on snakebite treatment. Our findings emphasise the pressing need to develop highly specific and dose-efficacious antivenoms for the treatment of snakebites in the Indian subcontinent. While in the long term this can likely be achieved via the application of innovative recombinant antibody technologies, in the interim, we strongly advocate for the production of regionally effective antivenoms that can circumvent medically important inter- and intra-specific differences in snake venoms found across the different biogeographical regions of India.

## Supporting information

S1 Fig Biochemical variation in the pan-Indian populations of *N*. *naja venoms*. (PDF)

S2 Fig Agarose gel electrophoresis showing DNase activities of *N*. *naja* venoms. (PDF)

S3 Fig Fibrinogenolytic activities of *N*. *naja* venoms from distinct locations across India. (PDF)

S4 Fig Immunological cross-reactivity between commercial Indian antivenoms and *N*. *naja* venoms. (PDF)

S5 Fig **A** Western blotting of commercial Indian antivenoms against the venoms of pan-Indian populations of *N*. *naja*. **B** Heatmap of venom recognition potential of commercial Indian antivenoms against the venoms of pan-Indian populations of *N*. *naja*.
(PDF)

S1 Table **A** Details of the *N*. *naja* venom samples tested. **B** Details of the investigated commercial Indian antivenoms.
(PDF)

S2 Table **A-C** Toxin compositions of *N*. *naja* venoms from various populations across India.
(PDF)

S3 Table **A** The median lethal dose of the pan-Indian populations of *N*. *naja*. **B** Neutralising potencies of Premium Serums antivenom against the pan-Indian populations of *N*. *naja*.
(PDF)

S1 Data Results of mass spectrometry analyses for semi-arid (Punjab), Gangetic plain (West Bengal) and desert population (Rajasthan) of *Naja naja* venoms (S1 Data.zip). (ZIP)
