# Supplementary material for: Biogeographical venom variation in the Indian spectacled cobra (Naja naja) underscores the pressing need for pan-India efficacious snakebite therapy
Source: PLoS Negl Trop Dis. 2021 Feb 18;15(2):e0009150. doi: 10.1371/journal.pntd.0009150 (PMC7924803; doi:10.1371/journal.pntd.0009150)
Supplement: S1 Fig — (PDF) [file pntd.0009150.s001.pdf]

Fig. S1. Biochemical variation in pan-Indian populations of *N. naja*

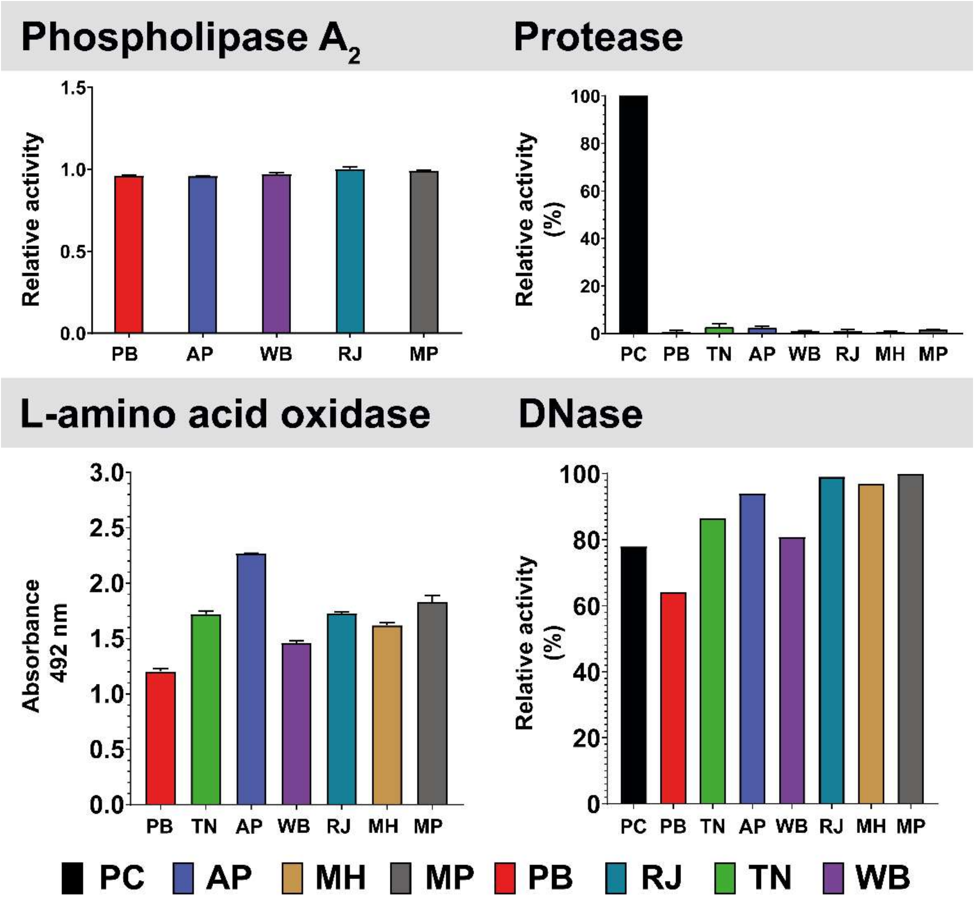

Graphs depicting the relative biochemical activities of toxins from the venoms of pan-Indian populations of *N. naja* are shown here. All assays, barring the DNase assay, were performed in triplicates and the standard deviation is indicated by error bars. Distinct snake populations are uniquely color coded. **PC:** Positive Control; **PB:** Punjab (North India); **TN:** Tamil Nadu (South India); **AP:** Andhra Pradesh (Southeast India); **WB:** West Bengal (East India); **RJ:** Rajasthan; **MH:** Maharashtra (Southwest India); and **MP:** Madhya Pradesh (Central India).
