# Supplementary material for: Biogeographical venom variation in the Indian spectacled cobra (Naja naja) underscores the pressing need for pan-India efficacious snakebite therapy
Source: PLoS Negl Trop Dis. 2021 Feb 18;15(2):e0009150. doi: 10.1371/journal.pntd.0009150 (PMC7924803; doi:10.1371/journal.pntd.0009150)
Supplement: S2 Fig — (PDF) [file pntd.0009150.s002.pdf]

Fig. S2. Agarose gel electrophoresis showing DNase activities of *N. naja* venoms.

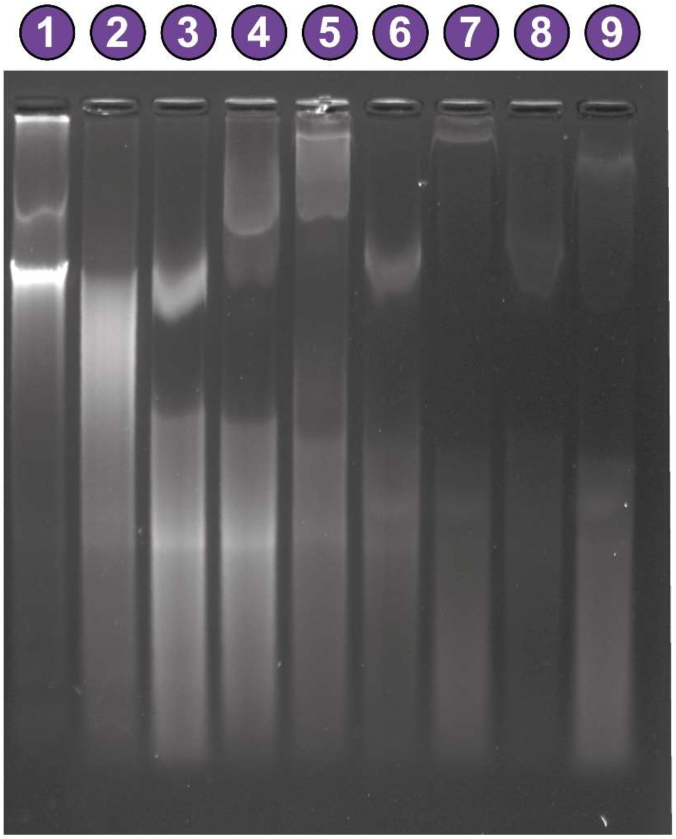

Agarose gels, depicting DNase activities, are shown here for *N. naja* venoms (50 μg/ml). 1: DNA only (negative control); 2: DNA + 15 U DNase (positive control); 3: Punjab (North India); 4: Tamil Nadu (South India); 5: Andhra Pradesh (Southeast India); 6: West Bengal (East India); 7: Rajasthan (West India); 8: Maharashtra (Southwest India); and 9: Madhya Pradesh (Central India).
