# Supplementary material for: Biogeographical venom variation in the Indian spectacled cobra (Naja naja) underscores the pressing need for pan-India efficacious snakebite therapy
Source: PLoS Negl Trop Dis. 2021 Feb 18;15(2):e0009150. doi: 10.1371/journal.pntd.0009150 (PMC7924803; doi:10.1371/journal.pntd.0009150)
Supplement: S3 Fig — (PDF) [file pntd.0009150.s003.pdf]

Fig. S3. Fibrinogenolytic activities of *N. naja* venoms from distinct locations across India.

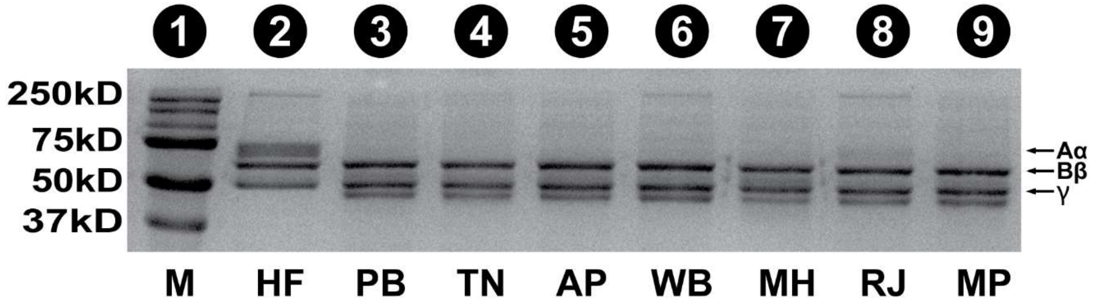

The enzymatic cleavage of fibrinogen by venoms of various populations of *N. naja* is shown here. **M:** Pre-stained protein ladder; **HF:** human fibrinogen; **PB:** Punjab (North India); **TN:** Tamil Nadu (South India); **AP:** Andhra Pradesh (Southeast India); **WB:** West Bengal (East India), **RJ:** Rajasthan (West India), **MH:** Maharashtra (Southwest India); and **MP:** Madhya Pradesh (Central India).
