# Supplementary material for: Biogeographical venom variation in the Indian spectacled cobra (Naja naja) underscores the pressing need for pan-India efficacious snakebite therapy
Source: PLoS Negl Trop Dis. 2021 Feb 18;15(2):e0009150. doi: 10.1371/journal.pntd.0009150 (PMC7924803; doi:10.1371/journal.pntd.0009150)
Supplement: S4 Fig — (PDF) [file pntd.0009150.s004.pdf]

Fig. S4. Immunological cross-reactivity between commercial Indian antivenoms and *N. naja* venoms.

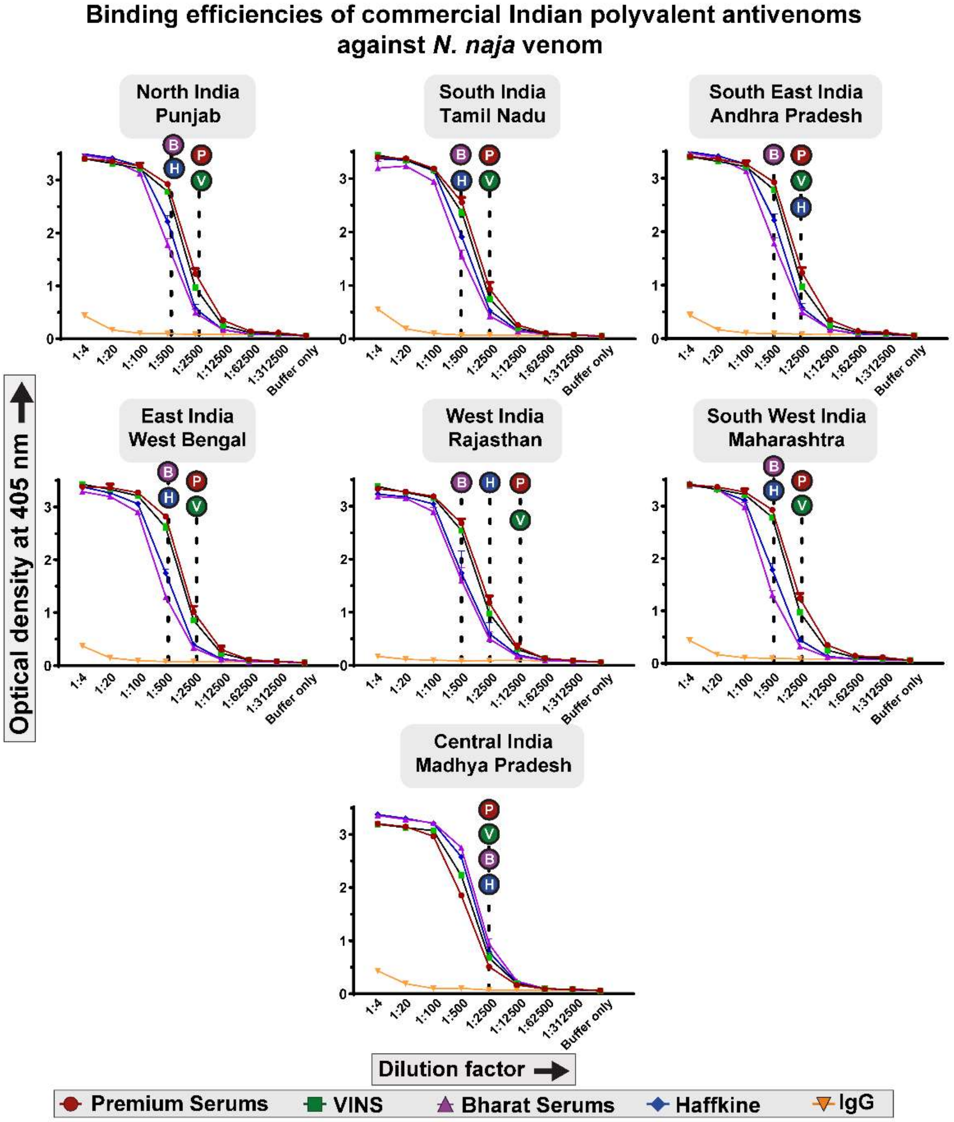

End-point ELISA for estimating the binding efficiencies of commercial Indian antivenoms against the pan-Indian populations of *N. naja* were carried out for various dilutions of antivenoms (1:4 to 1:312,500). The absorbance values, which directly correspond to the venom recognition potential of antivenoms, is measured at 405 nm, and plotted against the respective dilution of antivenom. All assays were carried out in triplicates and the standard deviation is shown as error bars. The alphabets indicated against the dotted lines represent the titres of the respective antivenom, which were determined using the naive horse IgG at 1:4 dilution (**P:** Premium Serums & Vaccines Pvt. Ltd.; **V:** VINS Bioproducts Ltd.; **B:** Bharat Serums and Vaccines Ltd; and **H:** Haffkine BioPharmaceutical Corporation Ltd.)
