# Supplementary material for: Biogeographical venom variation in the Indian spectacled cobra (Naja naja) underscores the pressing need for pan-India efficacious snakebite therapy
Source: PLoS Negl Trop Dis. 2021 Feb 18;15(2):e0009150. doi: 10.1371/journal.pntd.0009150 (PMC7924803; doi:10.1371/journal.pntd.0009150)
Supplement: S5 Fig — A Western blotting of commercial Indian antivenoms against the venoms of pan-Indian populations of N. naja. B Heatmap of venom recognition potential of commercial Indian antivenoms against the venoms of pan-Indian populations of N. naja. (PDF) [file pntd.0009150.s005.pdf]

Fig. S5A. Western blotting of commercial Indian antivenoms against the venoms of pan-Indian populations of *N. naja.*

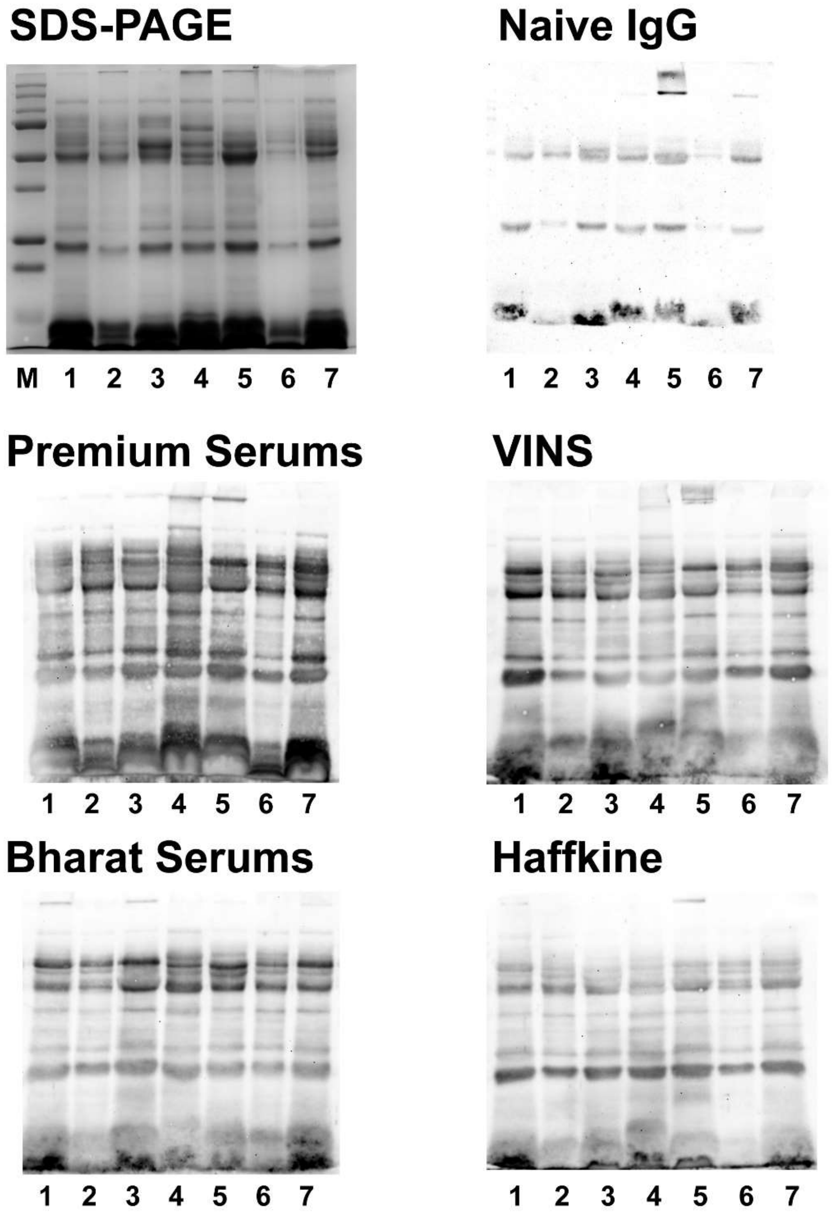

This figure depicts the binding affinities (western blots) of commercial Indian antivenoms against the venoms of biogeographically distinct populations of *N. naja*. **M.** Marker; **1.** PB (north India); **2.** TN (south India); **3.** AP (southeast India); **4.** WB (east India); **5.** RJ (west India); **6.** MH (southwest India); and **7.** MP (central India).

Fig. S5B. Heatmap of venom recognition potential of commercial Indian antivenoms against the venoms of pan-Indian populations of *N. naja.*

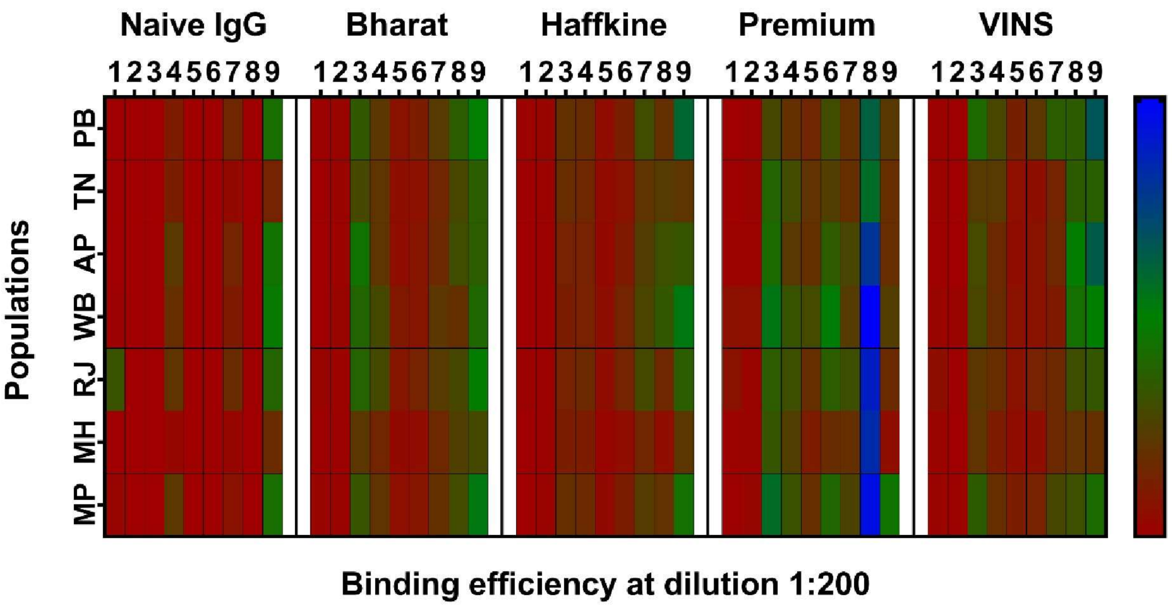

This figure depicts the binding affinities of commercial Indian antivenoms and naive horse IgGs against the *N. naja* venoms as a heatmap, on a gradient scale of red (low binding) to blue (high binding). The values were determined for individual immunoblot bands (1 to 9) using densitometric analysis. Populations: PB (north India); TN (south India); AP (southeast India); WB (east India); RJ (west India); MH (southwest India); and MP (central India). This heatmap was generated based on the densitometric analysis of immunoblot bands using ImageJ.
