# Supplementary material for: Biogeographical venom variation in the Indian spectacled cobra (Naja naja) underscores the pressing need for pan-India efficacious snakebite therapy
Source: PLoS Negl Trop Dis. 2021 Feb 18;15(2):e0009150. doi: 10.1371/journal.pntd.0009150 (PMC7924803; doi:10.1371/journal.pntd.0009150)
Supplement: S1 Table — A Details of the N. naja venom samples tested. B Details of the investigated commercial Indian antivenoms. (PDF) [file pntd.0009150.s006.pdf]

Supplementary Tables

Table S1A. Details of the *N. naja* venom samples tested

Sample ID	Sampling location	Biogeographical Zone	No. of individuals	Protein Content (mg/mL)
NaNaPB01	North India Nawanshahr, Punjab (PB)	Semiarid	5	0.159
NaNaTN01	South India Kancheepuram, Tamil Nadu (TN)	Coastal	*Multiple	0.360
NaNaAP04	Southeast India Visakhapatnam, Andhra Pradesh (AP)	Coastal	1	0.169
NaNaWB05	East India Kolkata, West Bengal (WB)	Gangetic plains	1	0.149
NaNaRJ01	West India Bikaner, Rajasthan (RJ)	Desert	1	0.112
NaNaMH07	West India Mahad, Maharashtra (MH)	Western Ghats	4	0.563
NaNaMP03	Central India Jabalpur, Madhya Pradesh (MP)	Deccan peninsula	1	0.135

This table shows the sampling locations of *N. naja* venoms from various biogeographic zones in the country. The total number of individual snakes from which the venoms were collected, and their respective venom protein concentrations have also been presented.

* This venom was sourced from the Irula Snake Catchers Industrial Cooperative Society and, hence, is likely pooled from multiple individuals.

Table S1B. Details of the investigated commercial Indian antivenoms

Manufacturer	Batch	Manufacture (M) and expiry (E) dates	Protein content (mg/ml)	Marketed neutralizing efficacy (mg/ml)
**Bharat Serums and Vaccines Ltd.**	A05318087	**M:** 10/2018<br>**E:** 09/2022	26.5 ± 0.77	*N. naja*: 0.60<br>*B. caeruleus*: 0.45<br>*D. russelii*: 0.60<br>*E. carinatus*: 0.45
**Haffkine BioPharmaceutical Corporation Ltd.**	AS180611	**M:** 06/2018<br>**E:** 11/2022	24.7± 0.5	
**Premium Serums & Vaccines Pvt. Ltd.**	ASVS(I)-Lyo013	**M:** 11/2018<br>**E:** 11/2022	26.2± 1.2	
**VINS Bioproducts Ltd.**	01AS18067	**M:** 11/2018<br>**E:** 10/2022	31.4 ± 0.54	

This table shows the details of commercial Indian antivenoms used in this study. Batch numbers, manufacturing and expiration dates, protein concentrations of the vials as estimated by Bradford method, and the marketed neutralisation potencies against the 'big four' snakes have also been shown here.
