# Supplementary material for: Biogeographical venom variation in the Indian spectacled cobra (Naja naja) underscores the pressing need for pan-India efficacious snakebite therapy
Source: PLoS Negl Trop Dis. 2021 Feb 18;15(2):e0009150. doi: 10.1371/journal.pntd.0009150 (PMC7924803; doi:10.1371/journal.pntd.0009150)
Supplement: S2 Table — A-C Toxin compositions of N. naja venoms from various populations across India. (PDF) [file pntd.0009150.s007.pdf]

**S2A-C Tables:** Toxin compositions of *N. naja* venoms from various populations across India.

Peaks Studio X was used to search raw MS/MS spectra against Uniprot's SwissProt database (www.uniprot.com) for the identification of toxin classes present in the venom. The key statistics of these searches, including the accession number, species name, -10lgP values, number of high confidence peptides, unique peptides, percent abundance of each toxin hit, average molecular mass (KDa), the toxin family of the matching Uniprot entry and the number of HPLC fraction in which the toxin was identified, have been shown. The percentage indicated adjacent to the toxin family corresponds to its proportion in the venom of *N. naja* populations from **(A)** Punjab; **(B)** West Bengal; and **(C)** Rajasthan, as determined by tandem mass spectrometry.

**S2A Table.** Venom composition of *N. naja* from Punjab

| Sr.no. | Accession | Species | -10lgP | #Peptides | #Unique | Relative abundance of toxin hit (%) | Avg mass (KDa) | Toxin type | Fraction no. |
|---|---|---|---|---|---|---|---|---|---|
| **Neurotoxic three-finger toxins (N-3FTx): 80.0429%** | | | | | | | | | |
| 1 | P25668 | *Naja naja* | 346.76 | 25 | 5 | 28.2587 | 7.847 | Type II (long) α-neurotoxin | 4-7,10 |
| 2 | P01391 | *Naja kaouthia* | 399.57 | 32 | 9 | 22.2327 | 7.831 | Type II (long) α-neurotoxin | 2-10 |
| 3 | P01427 | *Naja oxiana* | 256.98 | 10 | 10 | 9.9462 | 6.885 | Type I (short) α-neurotoxin | 1-6 |
| 4 | P82463 | *Naja kaouthia* | 339.63 | 16 | 16 | 7.2217 | 7.298 | Muscarinic | 5,6 |
| 5 | P82464 | *Naja kaouthia* | 239.27 | 10 | 10 | 2.6802 | 7.624 | Muscarinic | 5,6 |
| 6 | Q9YGI2 | *Naja atra* | 235.1 | 12 | 10 | 2.0447 | 9.845 | Neurotoxin | 4 |
| 7 | O93422 | *Naja atra* | 235.1 | 12 | 10 | 2.0447 | 9.815 | Type II (long) α-neurotoxin | 4 |

| | | | | | | | | | |
|---|---|---|---|---|---|---|---|---|---|
| 8 | P25672 | *Naja naja* | 326.66 | 21 | 3 | 1.7886 | 7.889 | Type II (long) α-neurotoxin | 4,6 |
| 9 | P58370 | *Micrurus corallinus* | 57.74 | 1 | 1 | 1.6974 | 9.591 | Type II (long) α-neurotoxin | 6 |
| 10 | P25673 | *Naja naja* | 321.71 | 19 | 1 | 1.6454 | 7.863 | Type II (long) α-neurotoxin | 4 |
| 11 | P25674 | *Naja haje* | 286.56 | 13 | 2 | 0.3085 | 7.821 | Type II (long) α-neurotoxin | 6 |
| 12 | P25680 | *Naja nivea* | 44.01 | 1 | 1 | 0.1154 | 7.545 | Neurotoxin | 4 |
| 13 | P29181 | *Naja naja* | 96.32 | 3 | 3 | 0.0282 | 7.637 | Neurotoxin | 4 |
| 14 | P82462 | *Naja kaouthia* | 83.08 | 2 | 2 | 0.0213 | 7.366 | Muscarinic | 5 |
| 15 | P82849 | *Naja kaouthia* | 61.32 | 1 | 1 | 0.0022 | 6.862 | Type I (short) α-neurotoxin | 2 |
| 16 | P25669 | *Naja naja* | 331.19 | 21 | 1 | 0.002 | 7.821 | Type II (long) α-neurotoxin | 6 |
| 17 | P14613 | *Naja kaouthia* | 102.68 | 1 | 1 | 0.001 | 6.983 | Type I (short) α-neurotoxin | 3 |
| 18 | Q9PSN6 | *Naja sputatrix* | 102.68 | 1 | 1 | 0.001 | 6.958 | Type I (short) α-neurotoxin | 3 |
| 19 | P60770 | *Naja atra* | 102.68 | 1 | 1 | 0.001 | 9.262 | Type I (short) α-neurotoxin | 3 |
| 20 | P60771 | *Naja kaouthia* | 102.68 | 1 | 1 | 0.001 | 9.262 | Type I (short) α-neurotoxin | 3 |
| 21 | Q9PTT0 | *Naja naja* | 102.68 | 1 | 1 | 0.001 | 9.262 | Type I (short) α-neurotoxin | 3 |
| **Cytotoxic three-finger toxins (C-3FTx): 10.0436%** | | | | | | | | | |
| 22 | P62377 | *Naja naja* | 250.41 | 13 | 13 | 9.668 | 7.014 | Cytotoxin | 5,8,9 |
| 23 | P01441 | *Naja oxiana* | 266.65 | 16 | 1 | 0.2697 | 6.636 | Cytotoxin | 8,9 |
| 24 | P01440 | *Naja naja* | 209.91 | 9 | 1 | 0.0479 | 6.763 | Cytotoxin | 10 |

| | | | | | | | | | |
|---|---|---|---|---|---|---|---|---|---|
| 25 | P01445 | *Naja kaouthia* | 289.07 | 16 | 1 | 0.0158 | 6.745 | Cytotoxin | 5,8 |
| 26 | P01454 | *Naja annulifera* | 141.19 | 6 | 1 | 0.0143 | 6.669 | Cytotoxin | 9 |
| 27 | P01453 | *Naja annulifera* | 141.19 | 6 | 1 | 0.0143 | 6.682 | Cytotoxin | 9 |
| 28 | P80245 | *Naja atra* | 229.69 | 11 | 1 | 0.0137 | 8.98 | Cytotoxin | 8 |
| **Kunitz-type serine protease inhibitor (Kunitz): 3.2163%** | | | | | | | | | |
| 29 | P19859 | *Naja naja* | 275.01 | 13 | 11 | 1.248 | 6.508 | Kunitz | 5,6 |
| 30 | P20229 | *Naja naja* | 268.4 | 16 | 13 | 1.7319 | 6.371 | Kunitz | 3,4 |
| 31 | Q5ZPJ7 | *Naja atra* | 269.64 | 12 | 9 | 0.2357 | 8.815 | Kunitz | 6 |
| 32 | P00986 | *Naja nivea* | 122.63 | 2 | 1 | 0.0006 | 6.466 | Kunitz | 3 |
| **Snake venom metalloproteinase (SVMP): 2.1007%** | | | | | | | | | |
| 33 | D6PXE8 | *Naja atra* | 221.44 | 8 | 5 | 0.7256 | 66.246 | SVMP | 5,6,8-10 |
| 34 | D3TTC1 | *Naja atra* | 221.44 | 8 | 5 | 0.7256 | 66.292 | SVMP | 5,6,8-10 |
| 35 | D5LMJ3 | *Naja atra* | 208.56 | 9 | 9 | 0.4558 | 68.254 | SVMP | 4 |
| 36 | P82942 | *Naja kaouthia* | 299.56 | 10 | 7 | 0.1834 | 44.493 | SVMP | 5,6,8-10 |
| 37 | Q9PVK7 | *Naja kaouthia* | 161.82 | 3 | 3 | 0.0103 | 67.662 | SVMP | 5,6 |
| **Vespryn: 1.8884%** | | | | | | | | | |
| 38 | P82885 | *Naja kaouthia* | 237.31 | 6 | 6 | 1.8884 | 12.038 | Vespyrn | 6,8-10 |
| **Cysteine-rich secretory proteins (CRISP): 1.8262%** | | | | | | | | | |
| 39 | P84808 | *Naja kaouthia* | 328.08 | 21 | 21 | 1.5721 | 26.216 | CRISP | 9,10 |
| 40 | P84805 | *Naja kaouthia* | 225.61 | 9 | 9 | 0.127 | 26.846 | CRISP | 6,8-10 |
| 41 | Q7T1K6 | *Naja atra* | 225.61 | 9 | 9 | 0.127 | 26.882 | CRISP | 6,8-10 |
| **Phospholipase A2 ($PLA_2$): 0.5721%** | | | | | | | | | |

| | | | | | | | | | |
|---|---|---|---|---|---|---|---|---|---|
| 42 | P10117 | *Laticauda colubrina* | 251.27 | 8 | 5 | 0.449 | 13.024 | Basic PLA2 | 5,6,8-10 |
| 43 | P15445 | *Naja naja* | 439.86 | 54 | 3 | 0.1087 | 13.346 | Acidic PLA2 | 5 |
| 44 | P25498 | *Naja oxiana* | 290.35 | 14 | 1 | 0.0144 | 13.229 | Acidic PLA2 | 5 |
| **Nerve growth factor (NGF): 0.2224%** | | | | | | | | | |
| 45 | Q5YF89 | *Naja sputatrix* | 244.88 | 8 | 8 | 0.2224 | 27.03 | NGF | 5,6 |
| **Cystatin: 0.0636%** | | | | | | | | | |
| 46 | E3P6P4 | *Naja kaouthia* | 118.34 | 3 | 3 | 0.0636 | 15.772 | Cystatin | 9 |
| **Cobra venom factor (CVF): 0.0190%** | | | | | | | | | |
| 47 | I2C090 | *Ophiophagus hannah* | 130.56 | 4 | 4 | 0.019 | 183.927 | CVF | 6,10 |
| **5′-nucleotidase: 0.0048%** | | | | | | | | | |
| 48 | B6EWW8 | *Gloydius brevicaudus* | 103.4 | 1 | 1 | 0.0048 | 64.434 | 5'-nucleotidase | 9 |

**S2B Table.** Venom composition of *N. naja* from West Bengal

| Sr.no. | Accession | Species | -10lgP | #Peptides | #Unique | Relative abundance of toxin hit (%) | Avg mass (KDa) | Toxin type | Fraction no. |
|---|---|---|---|---|---|---|---|---|---|
| **Neurotoxic three-finger toxins (N-3FTx): 73.3238%** | | | | | | | | | |
| 1 | P01391 | *Naja kaouthia* | 389.67 | 34 | 7 | 54.3459 | 7.831 | Type II (long) α-neurotoxin | 2-7,9,10 |
| 2 | P25668 | *Naja naja* | 335.05 | 24 | 2 | 10.6537 | 7.847 | Type II (long) α-neurotoxin | 4,5,8,10 |
| 3 | P25672 | *Naja naja* | 316.5 | 24 | 3 | 0.5434 | 7.889 | Type II (long) α-neurotoxin | 5,7,9 |
| 4 | P25674 | *Naja haje haje* | 276.01 | 14 | 2 | 0.1677 | 7.821 | Type II (long) α-neurotoxin | 4,5 |
| 5 | P01427 | *Naja oxiana* | 176.19 | 5 | 5 | 2.734 | 6.885 | Type I (short) α-neurotoxin | 1-5,9 |
| 6 | P82464 | *Naja kaouthia* | 147.47 | 6 | 6 | 0.1114 | 7.624 | Muscarinic | 6,7,9 |
| 7 | P58370 | *Micrurus corallinus* | 117.76 | 2 | 2 | 4.343 | 9.591 | Type II (long) α-neurotoxin | 4,5,7-9 |
| 8 | Q6IZ95 | *Bungarus candidus* | 103.03 | 2 | 1 | 0.007 | 9.723 | Neurotoxin | 9 |
| 9 | A2CKF7 | *Bungarus fasciatus* | 103.03 | 2 | 1 | 0.007 | 9.723 | Neurotoxin | 9 |
| 10 | O93422 | *Naja atra* | 103.03 | 2 | 1 | 0.007 | 9.815 | Type II (long) α-neurotoxin | 9 |
| 11 | Q9YGI2 | *Naja atra* | 103.03 | 2 | 1 | 0.007 | 9.845 | Neurotoxin | 9 |
| 12 | P01400 | *Naja melanoleuca* | 103.03 | 2 | 1 | 0.007 | 7.43 | Neurotoxin | 9 |
| 13 | P85520 | *Naja oxiana* | 103.03 | 2 | 1 | 0.007 | 7.482 | Neurotoxin | 9 |
| 14 | Q9W717 | *Naja atra* | 100.28 | 2 | 2 | 0.0641 | 9.695 | Neurotoxin | 6,7 |
| 15 | Q9PSN6 | *Naja sputatrix* | 71.28 | 1 | 1 | 0.0402 | 6.958 | Neurotoxin | 2,4,5 |

| | | | | | | | | | |
|---|---|---|---|---|---|---|---|---|---|
| 16 | P14613 | *Naja kaouthia* | 71.28 | 1 | 1 | 0.0402 | 6.983 | Type I (short) α-neurotoxin | 2,4,5 |
| 17 | P60770 | *Naja atra* | 71.28 | 1 | 1 | 0.0402 | 9.262 | Type I (short) α-neurotoxin | 2,4,5 |
| 18 | P60771 | *Naja kaouthia* | 71.28 | 1 | 1 | 0.0402 | 9.262 | Type I (short) α-neurotoxin | 2,4,5 |
| 19 | Q9PTT0 | *Naja naja* | 71.28 | 1 | 1 | 0.0402 | 9.262 | Type I (short) α-neurotoxin | 2,4,5 |
| 20 | P82849 | *Naja kaouthia* | 59.75 | 1 | 1 | 0.1158 | 6.862 | Type I (short) α-neurotoxin | 2 |
| 21 | P0CAR1 | *Micrurus pyrrhocryptus* | 54.64 | 1 | 1 | 0.0015 | 6.533 | Type I (short) α-neurotoxin | 2 |
| **Cytotoxic three-finger toxins (C-3FTx): 23.6035%** | | | | | | | | | |
| 22 | Q9PST3 | *Naja sputatrix* | 159.16 | 7 | 2 | 1.385 | 9.042 | Cytotoxin | 6-8 |
| 23 | Q9PST4 | *Naja sputatrix* | 159.16 | 7 | 2 | 1.385 | 9.054 | Cytotoxin | 6-8 |
| 24 | A0A0U5 | *Naja naja* | 159.16 | 7 | 2 | 1.385 | 8.041 | Cytotoxin | 6-8 |
| 25 | P86541 | *Naja naja* | 159.16 | 7 | 2 | 1.385 | 6.764 | Cytotoxin | 6-8 |
| 26 | P86382 | *Naja naja* | 149.73 | 6 | 4 | 8.2447 | 6.792 | Cytotoxin | 6 |
| 27 | P86540 | *Naja naja* | 149.73 | 6 | 4 | 8.2447 | 6.793 | Cytotoxin | 6 |
| 28 | Q91137 | *Naja atra* | 137.93 | 3 | 3 | 0.1748 | 9.309 | Cytotoxin | 4,6,7 |
| 29 | Q9W716 | *Naja atra* | 137.93 | 3 | 3 | 0.1748 | 9.333 | Cytotoxin | 4,6,7 |
| 30 | Q91126 | *Naja atra* | 137.93 | 3 | 3 | 0.1748 | 9.319 | Cytotoxin | 4,6,7 |
| 31 | Q91996 | *Naja atra* | 137.93 | 3 | 3 | 0.1748 | 9.305 | Cytotoxin | 4,6,7 |
| 32 | P62377 | *Naja naja* | 137.93 | 3 | 3 | 0.1748 | 7.014 | Cytotoxin | 4,6,7 |
| 33 | P62375 | *Naja atra* | 137.93 | 3 | 3 | 0.1748 | 9.323 | Cytotoxin | 4,6,7 |
| 34 | P62390 | *Naja annulifera* | 137.93 | 3 | 3 | 0.1748 | 6.842 | Cytotoxin | 4,6,7 |
| 35 | P62394 | *Naja haje haje* | 137.93 | 3 | 3 | 0.1748 | 6.842 | Cytotoxin | 4,6,7 |
| 36 | P14541 | *Naja kaouthia* | 137.93 | 3 | 3 | 0.1748 | 6.994 | Cytotoxin | 4,6,7 |
| 37 | P80245 | *Naja atra* | 125.44 | 4 | 1 | 0.0003 | 8.98 | Cytotoxin | 6 |

| | | | | | | | | | |
|---|---|---|---|---|---|---|---|---|---|
| 38 | Q98965 | *Naja atra* | 125.44 | 4 | 1 | 0.0003 | 9.201 | Cytotoxin | 6 |
| **Cysteine-rich secretory proteins (CRISP): 1.6185%** | | | | | | | | | |
| 39 | P84808 | *Naja kaouthia* | 166.23 | 10 | 10 | 1.6084 | 26.216 | CRISP | 8 |
| 40 | P84805 | *Naja kaouthia* | 43.45 | 1 | 1 | 0.0001 | 26.846 | CRISP | 5 |
| 41 | Q7T1K6 | *Naja atra* | 43.45 | 1 | 1 | 0.0001 | 26.882 | CRISP | 5 |
| **Vespryn: 0.9392%** | | | | | | | | | |
| 42 | P82885 | *Naja kaouthia* | 147.02 | 4 | 4 | 0.9392 | 12.038 | Vespyrn | 8 |
| **Snake venom metalloproteinase (SVMP): 0.3122%** | | | | | | | | | |
| 43 | D5LMJ3 | *Naja atra* | 189.17 | 6 | 6 | 0.1614 | 68.254 | SVMP | 4,5,7 |
| 44 | D6PXE8 | *Naja atra* | 108.75 | 1 | 1 | 0.0711 | 66.246 | SVMP | 4 |
| 45 | D3TTC1 | *Naja atra* | 108.75 | 1 | 1 | 0.0711 | 66.292 | SVMP | 4 |
| 46 | P82942 | *Naja kaouthia* | 79.51 | 2 | 2 | 0.0056 | 44.493 | SVMP | 6,8 |
| 47 | Q9PVK7 | *Naja kaouthia* | 65.09 | 1 | 1 | 0.0019 | 67.662 | SVMP | 6,7 |
| 48 | F8RKW1 | *Drysdalia coronoides* | 62.07 | 1 | 1 | 0.0011 | 68.24 | SVMP | 4 |
| **Nerve growth factor (NGF): 0.1290%** | | | | | | | | | |
| 49 | Q5YF89 | *Naja sputatrix* | 176.32 | 8 | 8 | 0.129 | 27.03 | NGF | 7,8 |
| **Kunitz-type serine protease inhibitor (Kunitz): 0.0460%** | | | | | | | | | |
| 50 | P20229 | *Naja naja* | 137.86 | 5 | 5 | 0.046 | 6.371 | Kunitz | 3-5 |
| **Phospholipase A2 ($PLA_2$): 0.0382%** | | | | | | | | | |
| 51 | P25498 | *Naja oxiana* | 243 | 10 | 1 | 0.0293 | 13.229 | Acidic PLA2 | 6 |
| 52 | P10117 | *Laticauda colubrina* | 149.8 | 3 | 1 | 0.0004 | 13.024 | Basic PLA2 | 7 |

| | | | | | | | | | |
|---|---|---|---|---|---|---|---|---|---|
| 53 | F8J2D0 | *Drysdalia coronoides* | 123.44 | 3 | 1 | 0.0042 | 16.055 | PLA2 | 6 |
| 54 | F8J2D2 | *Drysdalia coronoides* | 123.44 | 3 | 1 | 0.0042 | 16.011 | PLA2 | 6 |
| **Cobra venom factor (CVF): <0.0001%** | | | | | | | | | |
| 55 | Q91132 | *Naja kaouthia* | 94.99 | 2 | 1 | 0 | 184.517 | CVF | 7 |
| **Phospholipase $A_2$ inhibitor (Physiological protein)** | | | | | | | | | |
| 56 | Q7LZI1 | *Naja kaouthia* | 73.89 | 1 | 1 | Not applicable | 20.452 | Physiological | 7 |

**S2C Table.** Venom composition of *N. naja* from Rajasthan

| Sr.no. | Accession | Species | -10lgP | #Peptides | #Unique | Relative abundance of toxin hit (%) | Avg mass (KDa) | Toxin type | Fraction no. |
|---|---|---|---|---|---|---|---|---|---|
| **Cytotoxic three-finger toxins (C-3FTx): 41.7274%** | | | | | | | | | |
| 1 | P01441 | *Naja oxiana* | 308.49 | 21 | 16 | 28.7003 | 6.636 | Cytotoxin | 6-9 |
| 2 | P86538 | *Naja naja* | 231.77 | 8 | 1 | 0.1203 | 6.711 | Cytotoxin | 6 |
| 3 | P62377 | *Naja naja* | 185.15 | 5 | 5 | 2.1395 | 7.014 | Cytotoxin | 6-8 |
| 4 | Q9W716 | *Naja atra* | 185.15 | 5 | 5 | 2.1395 | 9.333 | Cytotoxin | 6-8 |
| 5 | P62375 | *Naja atra* | 185.15 | 5 | 5 | 2.1395 | 9.323 | Cytotoxin | 6-8 |
| 6 | Q91126 | *Naja atra* | 185.15 | 5 | 5 | 2.1395 | 9.319 | Cytotoxin | 6-8 |
| 7 | Q91137 | *Naja atra* | 185.15 | 5 | 5 | 2.1395 | 9.309 | Cytotoxin | 6-8 |
| 8 | Q91996 | *Naja atra* | 185.15 | 5 | 5 | 2.1395 | 9.305 | Cytotoxin | 6-8 |
| 9 | P07525 | *Naja atra* | 165.49 | 8 | 1 | 0.034 | 6.81 | Cytotoxin | 8 |
| 10 | Q9PST3 | *Naja sputatrix* | 160.35 | 7 | 1 | 0.0009 | 9.042 | Cytotoxin | 9 |
| 11 | A0A0U5 | *Naja naja* | 160.35 | 7 | 1 | 0.0009 | 8.041 | Cytotoxin | 9 |
| 12 | Q9PST4 | *Naja sputatrix* | 160.35 | 7 | 1 | 0.0009 | 9.054 | Cytotoxin | 9 |
| 13 | P01452 | *Naja mossambica* | 138.02 | 3 | 1 | 0.0079 | 6.715 | Cytotoxin | 7,8 |
| 14 | P01453 | *Naja annulifera* | 90.14 | 2 | 1 | 0.0063 | 6.682 | Cytotoxin | 8 |
| 15 | P01454 | *Naja annulifera* | 90.14 | 2 | 1 | 0.0063 | 6.669 | Cytotoxin | 8 |
| 16 | P01455 | *Naja annulifera* | 90.14 | 2 | 1 | 0.0063 | 6.696 | Cytotoxin | 8 |
| 17 | P01456 | *Naja nivea* | 90.14 | 2 | 1 | 0.0063 | 6.697 | Cytotoxin | 8 |
| **Neurotoxic three-finger toxins (N-3FTx): 30.0568%** | | | | | | | | | |
| 18 | P01391 | *Naja kaouthia* | 304.29 | 15 | 7 | 4.7024 | 7.831 | Type II (long) α-neurotoxin | 3-10 |

| | | | | | | | | | |
|---|---|---|---|---|---|---|---|---|---|
| 19 | P25668 | *Naja naja* | 242.86 | 9 | 2 | 2.9269 | 7.847 | Type II (long) α-neurotoxin | 4 |
| 20 | P25669 | *Naja naja* | 240.39 | 8 | 1 | 0.024 | 7.821 | Type II (long) α-neurotoxin | 4 |
| 21 | P25672 | *Naja naja* | 233.35 | 9 | 1 | 0.0127 | 7.889 | Type II (long) α-neurotoxin | 4,5 |
| 22 | P01427 | *Naja oxiana* | 225.01 | 7 | 7 | 3.5249 | 6.885 | Type I (short) α-neurotoxin | 1-6,9 |
| 23 | P82464 | *Naja kaouthia* | 205.01 | 5 | 5 | 7.9822 | 7.624 | Muscarinic | 6,7 |
| 24 | P25676 | *Hemachatus haemachatus* | 137.1 | 4 | 4 | 2.0118 | 6.917 | Neurotoxin | 4,5 |
| 25 | P01400 | *Naja melanoleuca* | 169.34 | 7 | 2 | 0.0465 | 7.43 | Neurotoxin | 4 |
| 26 | P29182 | *Naja naja* | 167.07 | 5 | 5 | 3.8753 | 7.581 | Neurotoxin | 4-7 |
| 27 | P29181 | *Naja naja* | 167.07 | 5 | 5 | 3.8753 | 7.637 | Neurotoxin | 4-7 |
| 28 | P82463 | *Naja kaouthia* | 153.3 | 3 | 3 | 0.8317 | 7.298 | Muscarinic | 5-8 |
| 29 | P85520 | *Naja oxiana* | 143.46 | 5 | 1 | 0.0033 | 7.482 | Neurotoxin | 4 |
| 30 | C1IC49 | *Walterinnesia aegyptia* | 97.77 | 2 | 1 | 0.0001 | 9.715 | Neurotoxin | 4 |
| 31 | P82849 | *Naja kaouthia* | 78.89 | 1 | 1 | 0.2217 | 6.862 | Type I (short) α-neurotoxin | 3 |
| 32 | P14613 | *Naja kaouthia* | 68.86 | 1 | 1 | 0.0027 | 6.983 | Type I (short) α-neurotoxin | 2,4 |
| 33 | Q9PSN6 | *Naja sputatrix* | 68.86 | 1 | 1 | 0.0027 | 6.958 | Neurotoxin | 2,4 |
| 34 | P60771 | *Naja kaouthia* | 68.86 | 1 | 1 | 0.0027 | 9.262 | Type I (short) α-neurotoxin | 2,4 |
| 35 | P60770 | *Naja atra* | 68.86 | 1 | 1 | 0.0027 | 9.262 | Type I (short) α-neurotoxin | 2,4 |
| 36 | Q9PTT0 | *Naja naja* | 68.86 | 1 | 1 | 0.0027 | 9.262 | Type I (short) α-neurotoxin | 2,4 |
| 37 | P58370 | *Micrurus corallinus* | 51.22 | 1 | 1 | 0.0042 | 9.591 | Type II (long) α-neurotoxin | 7 |

| | | | | | | | | | |
|---|---|---|---|---|---|---|---|---|---|
| 38 | Q802B2 | *Naja sputatrix* | 49.58 | 1 | 1 | 0.0003 | 9.92 | Neurotoxin | 4 |
| **Phospholipase A2 ($PLA_2$): 19.9941%** | | | | | | | | | |
| 39 | P15445 | *Naja naja* | 420.4 | 36 | 6 | 19.8156 | 13.346 | Acidic PLA2 | 5-8,9 |
| 40 | P25498 | *Naja oxiana* | 267.68 | 11 | 1 | 0.0015 | 13.229 | Acidic PLA2 | 8 |
| 41 | P10117 | *Laticauda colubrina* | 195.82 | 6 | 5 | 0.1274 | 13.024 | Basic PLA2 | 6-9 |
| 42 | P00601 | *Naja melanoleuca* | 102.01 | 2 | 1 | 0.0248 | 13.36 | Acidic PLA2 | 6 |
| 43 | P00600 | *Naja melanoleuca* | 102.01 | 2 | 1 | 0.0248 | 13.427 | Acidic PLA2 | 6 |
| **Cysteine-rich secretory proteins (CRISP): 3.2008%** | | | | | | | | | |
| 44 | P84808 | *Naja kaouthia* | 239.84 | 17 | 9 | 0.0798 | 26.216 | CRISP | 9 |
| 45 | Q7T1K6 | *Naja atra* | 200.5 | 10 | 9 | 1.5605 | 26.882 | CRISP | 5,9,10 |
| 46 | P84805 | *Naja kaouthia* | 200.5 | 10 | 9 | 1.5605 | 26.846 | CRISP | 5,9,10 |
| **Nerve growth factor (NGF): 1.9243%** | | | | | | | | | |
| 47 | Q5YF89 | *Naja sputatrix* | 300.13 | 12 | 12 | 1.9243 | 27.03 | NGF | 3,5-10 |
| **Snake venom metalloproteinase (SVMP): 1.3066%** | | | | | | | | | |
| 48 | P82942 | *Naja kaouthia* | 227.09 | 8 | 5 | 0.067 | 44.493 | SVMP | 6,8,9 |
| 49 | D5LMJ3 | *Naja atra* | 218.17 | 9 | 8 | 0.0666 | 68.254 | SVMP | 8,9 |
| 50 | D6PXE8 | *Naja atra* | 170.43 | 7 | 5 | 0.404 | 66.246 | SVMP | 6-9 |
| 51 | D3TTC1 | *Naja atra* | 170.43 | 7 | 5 | 0.404 | 66.292 | SVMP | 6-9 |
| 52 | Q9PVK7 | *Naja kaouthia* | 154.13 | 5 | 5 | 0.365 | 67.662 | SVMP | 6-8 |
| **Vespryn: 0.9571%** | | | | | | | | | |
| 53 | P82885 | *Naja kaouthia* | 316.44 | 17 | 5 | 0.9571 | 12.038 | Vespyrn | 3,4,6-10 |
| **Kunitz-type serine protease inhibitor (Kunitz): 0.6337%** | | | | | | | | | |

| | | | | | | | | | |
|---|---|---|---|---|---|---|---|---|---|
| 54 | P20229 | *Naja naja* | 210.9 | 6 | 5 | 0.5993 | 6.371 | Kunitz | 2,4,6,7 |
| 55 | Q5ZPJ7 | *Naja atra* | 128.37 | 5 | 4 | 0.0344 | 8.815 | Kunitz | 4,5 |
| **Cobra venom factor (CVF): 0.1053%** | | | | | | | | | |
| 56 | Q91132 | *Naja kaouthia* | 153.32 | 5 | 5 | 0.1053 | 184.517 | CVF | 5,6,8,9 |
| **5′-nucleotidase: 0.0522%** | | | | | | | | | |
| 57 | B6EWW8 | *Gloydius brevicaudus* | 88.05 | 1 | 1 | 0.0522 | 64.434 | 5'-nucleotidase | 6,7 |
| **L-amino-acid oxidase (LAAO): 0.0373%** | | | | | | | | | |
| 58 | A8QL58 | *Naja atra* | 157.4 | 5 | 4 | 0.0373 | 51.439 | LAAO | 6-9 |
| **Cystatin: 0.0047%** | | | | | | | | | |
| 59 | E3P6P4 | *Naja kaouthia* | 63.66 | 1 | 1 | 0.0047 | 15.772 | Cystatin | 8 |
