# Supplementary material for: Biogeographical venom variation in the Indian spectacled cobra (Naja naja) underscores the pressing need for pan-India efficacious snakebite therapy
Source: PLoS Negl Trop Dis. 2021 Feb 18;15(2):e0009150. doi: 10.1371/journal.pntd.0009150 (PMC7924803; doi:10.1371/journal.pntd.0009150)
Supplement: S3 Table — A The median lethal dose of the pan-Indian populations of N. naja. B Neutralising potencies of Premium Serums antivenom against the pan-Indian populations of N. naja. (PDF) [file pntd.0009150.s008.pdf]

S3A Table. Median lethal dose of pan-Indian *N. naja* populations

N. naja	Dose of venom (ug)					Number of survivors					LD_{50} µg/mouse	LD_{50} mg/Kg
**North India Punjab**	3.56	5.33	8	12	18	5	4	1	0	0	6.53<br>5.65-7.54	0.33<br>0.28-0.38
**Southeast India Andhra Pradesh**	3.56	5.33	8	12	18	5	5	4	2	0	10.93<br>9.10-13.10	0.55<br>0.45-0.65
**East India West Bengal**	2.67	4	6	9	13.5	5	4	2	0	0	5.46<br>4.56-6.53	0.27<br>0.22-0.32
**West India Rajasthan**	28.8	36	45	56.25	70.31	5	5	5	0	0	50.63	2.53
**Central India Madhya Pradesh**	3.56	5.33	8	12	18	4	1	0	0	0	4.36<br>3.76-5.04	0.22<br>0.188-0.25

This table represents the median lethal doses and survivorship data associated with venom toxicities of pan-Indian populations of *N. naja*.

S3B Table. Neutralising potencies of Premium Serums antivenom against the pan-Indian populations of *N. naja*

N. naja	**Volume of antivenom injected in venom-antivenom mixture**				**ED_{50}**	**Potency of antivenom**	**Marketed potency of antivenom**
	**µL**				**µL**	**mg/mL**	**mg/mL**
**North India Punjab**	73.96	49.40	32.93	21.96	67.37	0.387	0.60
**South East India Andhra Pradesh**	111.11	73.96	49.40	32.93	54.24	0.806	
**East India West Bengal**	111.11	73.96	49.40	32.93	60.45	0.361	
**West India Rajasthan**	166.66	111.11	73.96	49.40	NA	All mice were dead at 5X LD_{50}	
**Central India Madhya Pradesh**	111.11	73.96	49.40	32.93	60.45	0.288	

This table highlights the neutralising potencies of Premium Serums antivenom against the pan-Indian populations of *N. naja*. For populations, where the estimated neutralising potency meets the marketed potency of the commercial antivenom, the cells are indicated in green. Venoms that are poorly neutralized by the antivenom in the mouse challenge model are indicated in light red. The Rajasthan population, against which the antivenom completely failed, is highlighted in dark red.
