# Supplementary material for: Biogeographical venom variation in the Indian spectacled cobra (Naja naja) underscores the pressing need for pan-India efficacious snakebite therapy
Source: PLoS Negl Trop Dis. 2021 Feb 18;15(2):e0009150. doi: 10.1371/journal.pntd.0009150 (PMC7924803; doi:10.1371/journal.pntd.0009150)
Supplement: S1 Data — (ZIP) [file pntd.0009150.s009.zip › S1 Data/N. naja_Punjab/N. naja_Punjab.html]

proteins


Summary

  

# 1. Notes

# 2. Result Statistics

**Figure 1.**
False discovery rate (FDR) curve. X axis is the number of peptide-spectrum matches (PSM) being kept. Y axis is the corresponding FDR.

  


  

**Figure 2.**
PSM score distribution. **(a)**
Distribution of PEAKS peptide score; **(b)**
Scatterplot of PEAKS peptide score versus precursor mass error.

|  |  |  |  |
| --- | --- | --- | --- |
| **(a)**  |  | | **(b)**  |  | |

**Figure 3.**
Distribution of peptide feature detection. **(a)**
Feature m/z distribution; **(b)**
Feature RT distribution

|  |  |  |  |
| --- | --- | --- | --- |
| **(a)**  |  | | **(b)**  |  | |

**Figure 4.**
Distribution of identified peptide features. **(a)**
Feature abundance distribution **(b)**
*De novo*
sequencing validation.

|  |  |  |  |
| --- | --- | --- | --- |
| **(a)**  |  | | **(b)**  |  | |

|  |  |  |  |  |  |  |  |  |  |  |  |  |  |  |  |  |  |  |  |  |  |  |  |  |  |  |  |  |  |  |  |  |  |  |  |  |  |  |  |  |  |  |  |  |  |  |  |  |  |  |  |  |  |  |  |  |  |  |  |  |
| --- | --- | --- | --- | --- | --- | --- | --- | --- | --- | --- | --- | --- | --- | --- | --- | --- | --- | --- | --- | --- | --- | --- | --- | --- | --- | --- | --- | --- | --- | --- | --- | --- | --- | --- | --- | --- | --- | --- | --- | --- | --- | --- | --- | --- | --- | --- | --- | --- | --- | --- | --- | --- | --- | --- | --- | --- | --- | --- | --- | --- |
| **Table 1.** Statistics of data.  | # of MS scans | 162545 | | # of MS/MS scans | 153177 | | # of Features | 428953 | | # of Chimera scans | 32274 |    **Table 2.** Result filtration parameters.  | Peptide -10lgP | ≥42 | | Peptide Ascore | ≥0 | | Protein -10lgP | ≥20 | | Proteins unique peptides | ≥1 | | De novo ALC Score | ≥50% |    **Table 3.** Statistics of filtered result.  | Peptide-Spectrum Matches | 2627 | | Peptide sequences | 852 | | Protein groups | 111 | | Proteins | 165 | | Proteins (#Unique Peptides) | 59 (>2); 13 (=2); 93 (=1); | | FDR (Peptide-Spectrum Matches) | 0.1% | | FDR (Peptide Sequences) | 0.2% | | FDR (Protein Group) | 0.0% | | De Novo Only Spectra | 20796 | | # of identified Features | 17107 | | **Table 4.** PTM profile.  | Name | ∆Mass | Position | #PSM | -10lgP | Abundance | AScore || Carbamidomethyl | 57.02 | C | 944 | 138.46 | 2.08E6 | 1000.00 | | Oxidation | 15.99 | M | 68 | 101.70 | 8.04E4 | 1000.00 | |

# 3. Experiment Control

**Figure 5.**
Precursor mass error of peptide-spectrum matches (PSM) in filtered result. **(a)**
Distribution of precursor mass error in ppm; **(b)**
Scatterplot of precursor m/z versus precursor mass error in ppm.

|  |  |  |  |
| --- | --- | --- | --- |
| **(a)**  |  | | **(b)**  |  | |

**Table 5.**
Number of identified peptides in each sample by the number of missed cleavages

|  |  |  |  |  |  |  |  |  |  |  |  |  |  |  |  |  |  |  |  |  |  |  |  |  |  |  |  |  |  |  |  |  |  |  |  |  |  |  |  |  |  |  |  |  |  |  |  |  |  |  |  |  |  |  |  |  |  |  |  |  |  |  |  |  |  |  |
| --- | --- | --- | --- | --- | --- | --- | --- | --- | --- | --- | --- | --- | --- | --- | --- | --- | --- | --- | --- | --- | --- | --- | --- | --- | --- | --- | --- | --- | --- | --- | --- | --- | --- | --- | --- | --- | --- | --- | --- | --- | --- | --- | --- | --- | --- | --- | --- | --- | --- | --- | --- | --- | --- | --- | --- | --- | --- | --- | --- | --- | --- | --- | --- | --- | --- | --- |
| |  |  |  |  |  |  | | --- | --- | --- | --- | --- | --- | | Missed Cleavages | 0 | 1 | 2 | 3 | 4+ | | F1 | 90 | 6 | 0 | 0 | 0 | | F10 | 77 | 11 | 2 | 1 | 0 | | F2 | 55 | 4 | 1 | 0 | 0 | | F3 | 20 | 6 | 0 | 0 | 0 | | F4 | 55 | 81 | 53 | 29 | 0 | | F5 | 77 | 10 | 5 | 1 | 0 | | F6 | 103 | 21 | 2 | 0 | 0 | | F7 | 10 | 4 | 0 | 0 | 0 | | F8 | 54 | 16 | 21 | 7 | 0 | | F9 | 21 | 6 | 3 | 0 | 0 | |

# 4. Other Information

|  |  |  |  |
| --- | --- | --- | --- |
| **Table 6.** Search parameters.  | Search Engine Name: PEAKS Parent Mass Error Tolerance: 10.0 ppm Fragment Mass Error Tolerance: 0.6 Da Precursor Mass Search Type: monoisotopic Enzyme: Trypsin Max Missed Cleavages: 3 Digest Mode: Semispecific Fixed Modifications:    Carbamidomethylation: 57.02 Variable Modifications:    Oxidation (M): 15.99 Max Variable PTM Per Peptide: 3 Database: SwissProt Taxon: All Contaminant Database: cRAP\_contaminants Searched Entry: 560234 FDR Estimation: Enabled Merge Options: no merge Precursor Options: corrected Charge Options: no correction Filter Charge: 2 - 8 Process: true Associate chimera: yes | | **Table 7.** Instrument parameters.  | Fractions: 29102019\_RID\_1313\_NaNaPb\_F1.raw, 29102019\_RID\_1313\_N aNaPb\_F10.raw, 29102019\_RID\_1313\_NaNaPb\_F2.raw, 29102019\_RID\_ 1313\_NaNaPb\_F3.raw, 29102019\_RID\_1313\_NaNaPb\_F4.raw, 29102019 \_RID\_1313\_NaNaPb\_F5.raw, 29102019\_RID\_1313\_NaNaPb\_F6.raw, 291 02019\_RID\_1313\_NaNaPb\_F7.raw, 29102019\_RID\_1313\_NaNaPb\_F8.raw , 29102019\_RID\_1313\_NaNaPb\_F9.raw Ion Source: ESI(nano-spray) Fragmentation Mode: CID, CAD(y and b ions) MS Scan Mode: FT-ICR/Orbitrap MS/MS Scan Mode: Linear Ion Trap | |

  

Protein List

  

|  |
| --- |
| Protein Accession Contains: |
| Protein Description Contains: |
| Peptide Sample Area >= |
| Protein Ptm Contains: |

| Protein Group | Protein ID | Accession | -10lgP | Coverage (%) | Coverage (%) F1 | Coverage (%) F10 | Coverage (%) F2 | Coverage (%) F3 | Coverage (%) F4 | Coverage (%) F5 | Coverage (%) F6 | Coverage (%) F7 | Coverage (%) F8 | Coverage (%) F9 | Area F1 | Area F10 | Area F2 | Area F3 | Area F4 | Area F5 | Area F6 | Area F7 | Area F8 | Area F9 | #Peptides | #Unique | #Spec F1 | #Spec F10 | #Spec F2 | #Spec F3 | #Spec F4 | #Spec F5 | #Spec F6 | #Spec F7 | #Spec F8 | #Spec F9 | PTM | Avg. Mass | Description |
| --- | --- | --- | --- | --- | --- | --- | --- | --- | --- | --- | --- | --- | --- | --- | --- | --- | --- | --- | --- | --- | --- | --- | --- | --- | --- | --- | --- | --- | --- | --- | --- | --- | --- | --- | --- | --- | --- | --- | --- |
| 3 | 57 | P15445|PA2A2\_NAJNA | 439.86 | 100 | 0 | 71 | 0 | 0 | 0 | 88 | 64 | 12 | 100 | 60 |  |  |  |  |  | 2.65E6 |  |  | 2.34E5 |  | 54 | 3 | 0 | 11 | 0 | 0 | 0 | 126 | 7 | 1 | 68 | 9 | Y | 13346 | Acidic phospholipase A2 2 OS=Naja naja OX=35670 PE=1 SV=1 |
| 6 | 1 | P35527|K1C9\_HUMAN | 432.78 | 72 | 41 | 51 | 55 | 0 | 11 | 54 | 42 | 3 | 20 | 18 | 7.97E6 | 1.06E7 | 2.05E7 |  | 6.12E5 | 2.32E7 | 5.7E6 | 4.28E3 | 4.07E5 | 5.81E5 | 46 | 45 | 31 | 30 | 42 | 0 | 3 | 28 | 21 | 2 | 7 | 7 | Y | 62064 | Keratin, type I cytoskeletal 9 OS=Homo sapiens OX=9606 GN=KRT9 PE=1 SV=3 |
| 2 | 5 | P04264|K2C1\_HUMAN | 428.54 | 58 | 45 | 47 | 56 | 15 | 14 | 47 | 41 | 3 | 30 | 18 | 1.34E7 | 2.07E7 | 2.43E7 | 3.07E5 | 4.94E6 | 4.5E7 | 6.38E6 | 6.08E4 | 1.42E6 | 1.72E6 | 42 | 32 | 39 | 35 | 49 | 8 | 8 | 40 | 32 | 1 | 15 | 9 | Y | 66039 | Keratin, type II cytoskeletal 1 OS=Homo sapiens OX=9606 GN=KRT1 PE=1 SV=6 |
| 4 | 3 | P13645|K1C10\_HUMAN | 409.61 | 60 | 51 | 50 | 42 | 17 | 12 | 49 | 30 | 0 | 39 | 7 | 4.99E6 | 1.57E7 | 1.9E6 | 5.23E5 | 1.06E5 | 2.57E7 | 5.76E5 |  | 1E6 | 1.17E5 | 46 | 24 | 30 | 45 | 21 | 8 | 3 | 38 | 17 | 0 | 18 | 3 | Y | 58827 | Keratin, type I cytoskeletal 10 OS=Homo sapiens OX=9606 GN=KRT10 PE=1 SV=6 |
| 7 | 186 | P01391|3L21\_NAJKA | 399.57 | 83 | 13 | 59 | 21 | 41 | 83 | 45 | 83 | 59 | 41 | 59 |  | 3.48E6 | 1.11E5 | 7.65E5 | 1.41E8 | 2.84E7 | 1.14E9 | 6.97E4 | 3.25E6 | 4.07E6 | 32 | 9 | 1 | 5 | 3 | 6 | 37 | 4 | 99 | 4 | 2 | 5 | Y | 7831 | Alpha-cobratoxin OS=Naja kaouthia OX=8649 PE=1 SV=1 |
| 11 | 8 | P35908|K22E\_HUMAN | 382.66 | 68 | 31 | 47 | 29 | 10 | 6 | 36 | 30 | 0 | 17 | 4 | 2.68E4 |  |  |  |  | 2.79E5 |  |  |  |  | 34 | 2 | 19 | 23 | 15 | 6 | 4 | 21 | 12 | 0 | 9 | 2 | Y | 65433 | Keratin, type II cytoskeletal 2 epidermal OS=Homo sapiens OX=9606 GN=KRT2 PE=1 SV=2 |
| 12 | 7 | #CONTAM#K22E\_HUMAN| | 382.25 | 68 | 28 | 46 | 29 | 10 | 6 | 31 | 36 | 0 | 17 | 4 |  |  |  |  |  |  | 2.46E4 |  |  |  | 33 | 1 | 18 | 23 | 15 | 6 | 4 | 20 | 13 | 0 | 9 | 2 | Y | 65865 | sp|#CONTAM#K22E\_HUMAN| |
| 10 | 198 | P60043|PA2B1\_NAJSG | 381.32 | 37 | 0 | 28 | 0 | 0 | 0 | 31 | 17 | 0 | 35 | 17 |  |  |  |  |  | 0E0 |  |  |  |  | 34 | 1 | 0 | 5 | 0 | 0 | 0 | 75 | 2 | 0 | 49 | 4 | Y | 14003 | Basic phospholipase A2 1 (Fragment) OS=Naja sagittifera OX=195058 PE=1 SV=2 |
| 52 | 13 | P78386|KRT85\_HUMAN | 355.14 | 43 | 0 | 0 | 0 | 0 | 43 | 0 | 0 | 0 | 0 | 0 |  |  |  |  | 1.83E8 |  |  |  |  |  | 28 | 5 | 0 | 0 | 0 | 0 | 45 | 0 | 0 | 0 | 0 | 0 | Y | 55802 | Keratin, type II cuticular Hb5 OS=Homo sapiens OX=9606 GN=KRT85 PE=1 SV=1 |
| 52 | 14 | #CONTAM#KRHB5\_HUMAN| | 355.14 | 43 | 0 | 0 | 0 | 0 | 43 | 0 | 0 | 0 | 0 | 0 |  |  |  |  | 1.83E8 |  |  |  |  |  | 28 | 5 | 0 | 0 | 0 | 0 | 45 | 0 | 0 | 0 | 0 | 0 | Y | 55802 | sp|#CONTAM#KRHB5\_HUMAN| |
| 8 | 185 | P25668|3L21\_NAJNA | 346.76 | 80 | 0 | 46 | 0 | 14 | 69 | 32 | 80 | 46 | 32 | 32 |  | 1.17E5 |  |  | 9.36E8 | 1.09E7 | 6.16E8 | 4.46E4 |  |  | 25 | 5 | 0 | 3 | 0 | 1 | 49 | 4 | 93 | 3 | 2 | 2 | Y | 7847 | Long neurotoxin 1 OS=Naja naja OX=35670 PE=1 SV=1 |
| 53 | 220 | P82463|3SUC2\_NAJKA | 339.63 | 88 | 0 | 0 | 0 | 0 | 0 | 29 | 88 | 0 | 0 | 0 |  |  |  |  |  | 3.28E4 | 5.36E8 |  |  |  | 16 | 16 | 0 | 0 | 0 | 0 | 0 | 1 | 38 | 0 | 0 | 0 | Y | 7298 | Muscarinic toxin-like protein 2 OS=Naja kaouthia OX=8649 PE=1 SV=1 |
| 21 | 10 | P13647|K2C5\_HUMAN | 335.46 | 49 | 20 | 21 | 30 | 4 | 24 | 17 | 10 | 0 | 6 | 4 | 2.36E5 | 2.92E5 | 5.33E5 |  | 1.72E7 | 5.95E5 |  |  |  | 5.04E4 | 31 | 10 | 12 | 12 | 19 | 2 | 16 | 10 | 5 | 0 | 3 | 2 | Y | 62378 | Keratin, type II cytoskeletal 5 OS=Homo sapiens OX=9606 GN=KRT5 PE=1 SV=3 |
| 49 | 22 | O43790|KRT86\_HUMAN | 331.95 | 45 | 0 | 0 | 0 | 0 | 45 | 0 | 0 | 0 | 0 | 0 |  |  |  |  | 3.13E7 |  |  |  |  |  | 27 | 6 | 0 | 0 | 0 | 0 | 44 | 0 | 0 | 0 | 0 | 0 | Y | 53501 | Keratin, type II cuticular Hb6 OS=Homo sapiens OX=9606 GN=KRT86 PE=1 SV=1 |
| 49 | 23 | #CONTAM#KRHB6\_HUMAN| | 331.95 | 45 | 0 | 0 | 0 | 0 | 45 | 0 | 0 | 0 | 0 | 0 |  |  |  |  | 3.13E7 |  |  |  |  |  | 27 | 6 | 0 | 0 | 0 | 0 | 44 | 0 | 0 | 0 | 0 | 0 | Y | 53501 | sp|#CONTAM#KRHB6\_HUMAN| |
| 13 | 193 | P25669|3L22\_NAJNA | 331.19 | 80 | 0 | 32 | 0 | 14 | 55 | 18 | 80 | 32 | 32 | 32 |  |  |  |  |  |  | 1.45E5 |  |  |  | 21 | 1 | 0 | 2 | 0 | 1 | 37 | 2 | 76 | 2 | 2 | 2 | Y | 7821 | Long neurotoxin 2 OS=Naja naja OX=35670 PE=1 SV=1 |
| 66 | 45 | P84808|CRVP2\_NAJKA | 328.08 | 58 | 0 | 58 | 0 | 0 | 0 | 0 | 0 | 0 | 0 | 16 |  | 3.59E7 |  |  |  |  |  |  |  | 2.46E5 | 21 | 21 | 0 | 28 | 0 | 0 | 0 | 0 | 0 | 0 | 0 | 2 | Y | 26216 | Cysteine-rich venom protein kaouthin-2 OS=Naja kaouthia OX=8649 PE=1 SV=2 |
| 16 | 191 | P25672|3L24\_NAJNA | 326.66 | 80 | 0 | 32 | 0 | 14 | 69 | 18 | 77 | 32 | 14 | 32 |  |  |  |  | 4.37E7 |  | 7.02E7 |  |  |  | 21 | 3 | 0 | 2 | 0 | 1 | 30 | 1 | 59 | 2 | 1 | 2 | Y | 7889 | Long neurotoxin 4 OS=Naja naja OX=35670 PE=1 SV=1 |
| 23 | 11 | P02533|K1C14\_HUMAN | 324.44 | 55 | 25 | 21 | 30 | 0 | 26 | 29 | 27 | 0 | 1 | 6 | 5.29E4 | 4.27E5 | 3.24E5 |  | 1.09E7 | 6.01E5 | 3.27E5 |  |  | 5.3E4 | 25 | 8 | 9 | 7 | 13 | 0 | 16 | 11 | 11 | 0 | 1 | 3 | Y | 51562 | Keratin, type I cytoskeletal 14 OS=Homo sapiens OX=9606 GN=KRT14 PE=1 SV=4 |
| 19 | 208 | P25673|3L25\_NAJNA | 321.71 | 69 | 0 | 32 | 0 | 14 | 58 | 18 | 66 | 32 | 14 | 32 |  |  |  |  | 8.53E7 |  |  |  |  |  | 19 | 1 | 0 | 2 | 0 | 1 | 27 | 1 | 56 | 2 | 1 | 2 | Y | 7863 | Long neurotoxin 5 OS=Naja naja OX=35670 PE=1 SV=1 |
| 48 | 15 | P15924|DESP\_HUMAN | 311.81 | 12 | 1 | 1 | 4 | 0 | 7 | 2 | 1 | 0 | 0 | 0 | 3.4E4 | 3.69E4 | 1.32E5 |  | 1.32E7 | 2.24E5 | 1.46E4 |  |  |  | 23 | 23 | 2 | 2 | 8 | 0 | 15 | 5 | 1 | 0 | 0 | 0 | Y | 331774 | Desmoplakin OS=Homo sapiens OX=9606 GN=DSP PE=1 SV=3 |
| 63 | 34 | #CONTAM#K1HB\_HUMAN| | 307.45 | 44 | 2 | 2 | 2 | 0 | 44 | 2 | 2 | 0 | 2 | 0 |  |  |  |  | 7.96E7 |  |  |  |  |  | 21 | 6 | 1 | 1 | 1 | 0 | 28 | 1 | 1 | 0 | 1 | 0 | Y | 46214 | sp|#CONTAM#K1HB\_HUMAN| |
| 63 | 35 | Q14525|KT33B\_HUMAN | 307.45 | 44 | 2 | 2 | 2 | 0 | 44 | 2 | 2 | 0 | 2 | 0 |  |  |  |  | 7.96E7 |  |  |  |  |  | 21 | 6 | 1 | 1 | 1 | 0 | 28 | 1 | 1 | 0 | 1 | 0 | Y | 46214 | Keratin, type I cuticular Ha3-II OS=Homo sapiens OX=9606 GN=KRT33B PE=1 SV=3 |
| 72 | 56 | O76011|KRT34\_HUMAN | 305.93 | 29 | 0 | 0 | 0 | 0 | 29 | 0 | 0 | 0 | 0 | 0 |  |  |  |  | 4.2E8 |  |  |  |  |  | 19 | 14 | 0 | 0 | 0 | 0 | 35 | 0 | 0 | 0 | 0 | 0 | Y | 49424 | Keratin, type I cuticular Ha4 OS=Homo sapiens OX=9606 GN=KRT34 PE=1 SV=2 |
| 96 | 108 | P82942|VM3K\_NAJKA | 299.56 | 22 | 0 | 16 | 0 | 0 | 0 | 11 | 17 | 0 | 2 | 9 |  | 8.49E5 |  |  |  | 3.55E5 | 3.8E6 |  | 3.42E4 | 8.21E5 | 10 | 7 | 0 | 4 | 0 | 0 | 0 | 2 | 9 | 0 | 1 | 3 | Y | 44493 | Hemorrhagic metalloproteinase-disintegrin-like kaouthiagin OS=Naja kaouthia OX=8649 PE=1 SV=1 |
| 77 | 19 | #CONTAM#KRHB4\_HUMAN| | 295.45 | 36 | 0 | 1 | 0 | 0 | 36 | 1 | 0 | 0 | 1 | 0 |  |  |  |  | 8.13E7 |  |  |  |  |  | 21 | 16 | 0 | 1 | 0 | 0 | 26 | 1 | 0 | 0 | 1 | 0 | N | 64895 | sp|#CONTAM#KRHB4\_HUMAN| |
| 50 | 32 | A5A6M5|K1H1\_PANTR | 294.92 | 39 | 2 | 2 | 2 | 0 | 39 | 2 | 2 | 0 | 2 | 0 |  |  |  |  | 1.96E7 |  |  |  |  |  | 23 | 6 | 1 | 1 | 1 | 0 | 34 | 1 | 1 | 0 | 1 | 0 | Y | 47247 | Keratin, type I cuticular Ha1 OS=Pan troglodytes OX=9598 GN=KRT31 PE=2 SV=1 |
| 50 | 33 | Q15323|K1H1\_HUMAN | 294.92 | 39 | 2 | 2 | 2 | 0 | 39 | 2 | 2 | 0 | 2 | 0 |  |  |  |  | 1.96E7 |  |  |  |  |  | 23 | 6 | 1 | 1 | 1 | 0 | 34 | 1 | 1 | 0 | 1 | 0 | Y | 47237 | Keratin, type I cuticular Ha1 OS=Homo sapiens OX=9606 GN=KRT31 PE=1 SV=3 |
| 31 | 205 | P25498|PA2AE\_NAJOX | 290.35 | 48 | 0 | 29 | 0 | 0 | 0 | 47 | 22 | 12 | 38 | 29 |  |  |  |  |  | 4.13E5 |  |  |  |  | 14 | 1 | 0 | 4 | 0 | 0 | 0 | 42 | 2 | 1 | 11 | 3 | Y | 13229 | Acidic phospholipase A2 E OS=Naja oxiana OX=8657 PE=1 SV=1 |
| 26 | 17 | P48668|K2C6C\_HUMAN | 289.14 | 41 | 14 | 20 | 29 | 4 | 13 | 21 | 16 | 0 | 8 | 9 |  |  |  |  | 2.45E5 | 5.2E4 |  |  |  | 2.76E4 | 21 | 2 | 8 | 11 | 15 | 2 | 5 | 11 | 8 | 0 | 4 | 4 | Y | 60025 | Keratin, type II cytoskeletal 6C OS=Homo sapiens OX=9606 GN=KRT6C PE=1 SV=3 |
| 30 | 197 | P01445|3SA7A\_NAJKA | 289.07 | 88 | 0 | 13 | 0 | 0 | 0 | 80 | 13 | 0 | 88 | 13 |  |  |  |  |  | 1.08E5 |  |  | 1.71E5 |  | 16 | 1 | 0 | 2 | 0 | 0 | 0 | 15 | 1 | 0 | 18 | 1 | Y | 6745 | Cytotoxin 2 OS=Naja kaouthia OX=8649 PE=1 SV=1 |
| 43 | 245 | P25674|3L21\_NAJHH | 286.56 | 52 | 13 | 34 | 17 | 0 | 20 | 34 | 52 | 18 | 0 | 34 |  |  |  |  |  |  | 2.29E7 |  |  |  | 13 | 2 | 1 | 2 | 2 | 0 | 3 | 2 | 33 | 1 | 0 | 2 | Y | 7821 | Long neurotoxin 1 OS=Naja haje haje OX=8642 PE=1 SV=1 |
| 37 | 20 | P08779|K1C16\_HUMAN | 277.30 | 41 | 16 | 13 | 20 | 0 | 13 | 22 | 19 | 0 | 1 | 8 | 2.93E4 | 1.44E5 | 1.75E4 |  | 3.8E5 | 2.78E5 | 5.5E5 |  |  | 7.48E4 | 17 | 6 | 7 | 5 | 9 | 0 | 3 | 10 | 9 | 0 | 1 | 3 | Y | 51268 | Keratin, type I cytoskeletal 16 OS=Homo sapiens OX=9606 GN=KRT16 PE=1 SV=4 |
| 55 | 372 | P19859|VKTCI\_NAJNA | 275.01 | 61 | 0 | 0 | 0 | 0 | 0 | 39 | 61 | 0 | 0 | 0 |  |  |  |  |  | 2.11E5 | 9.21E7 |  |  |  | 13 | 11 | 0 | 0 | 0 | 0 | 0 | 2 | 32 | 0 | 0 | 0 | Y | 6508 | Kunitz-type serine protease inhibitor OS=Naja naja OX=35670 PE=1 SV=1 |
| 101 | 260 | Q5ZPJ7|VKT\_NAJAT | 269.64 | 62 | 0 | 0 | 0 | 14 | 0 | 0 | 62 | 0 | 0 | 0 |  |  |  |  |  |  | 1.75E7 |  |  |  | 12 | 9 | 0 | 0 | 0 | 1 | 0 | 0 | 15 | 0 | 0 | 0 | Y | 8815 | Kunitz-type serine protease inhibitor NACI OS=Naja atra OX=8656 PE=1 SV=1 |
| 27 | 212 | P20229|VKTTI\_NAJNA | 268.40 | 100 | 0 | 0 | 0 | 100 | 19 | 0 | 40 | 21 | 0 | 0 |  |  |  | 3.45E8 | 2.37E6 |  |  |  |  |  | 16 | 13 | 0 | 0 | 0 | 65 | 2 | 0 | 2 | 1 | 0 | 0 | Y | 6371 | Kunitz-type serine protease inhibitor OS=Naja naja OX=35670 PE=1 SV=1 |
| 29 | 82 | P01441|3SA2\_NAJOX | 266.65 | 93 | 0 | 62 | 0 | 0 | 13 | 67 | 33 | 0 | 92 | 85 |  |  |  |  |  |  |  |  | 1.8E5 | 2.61E6 | 16 | 1 | 0 | 7 | 0 | 0 | 2 | 10 | 2 | 0 | 10 | 27 | Y | 6636 | Cytotoxin 2 OS=Naja oxiana OX=8657 PE=1 SV=1 |
| 87 | 41 | O76009|KT33A\_HUMAN | 264.80 | 38 | 0 | 0 | 0 | 0 | 38 | 0 | 0 | 0 | 0 | 0 |  |  |  |  | 2.8E5 |  |  |  |  |  | 16 | 1 | 0 | 0 | 0 | 0 | 21 | 0 | 0 | 0 | 0 | 0 | Y | 45940 | Keratin, type I cuticular Ha3-I OS=Homo sapiens OX=9606 GN=KRT33A PE=2 SV=2 |
| 1 | 106 | P01946|HBA\_RAT | 263.64 | 54 | 52 | 15 | 46 | 35 | 0 | 35 | 8 | 54 | 35 | 35 | 2.24E7 | 2.18E4 | 4.64E6 | 3.7E6 |  | 8.69E5 | 2.96E5 | 2.34E6 | 1.44E5 | 2.21E5 | 6 | 6 | 253 | 1 | 26 | 18 | 0 | 8 | 2 | 13 | 3 | 4 | N | 15329 | Hemoglobin subunit alpha-1/2 OS=Rattus norvegicus OX=10116 GN=Hba1 PE=1 SV=3 |
| 40 | 298 | P01427|3S11\_NAJOX | 256.98 | 74 | 64 | 0 | 74 | 18 | 64 | 18 | 18 | 0 | 0 | 0 | 6.35E7 |  | 1.2E8 | 2.43E7 | 2.86E7 | 7.51E4 | 2.16E5 |  |  |  | 10 | 10 | 16 | 0 | 13 | 5 | 6 | 1 | 2 | 0 | 0 | 0 | Y | 6885 | Short neurotoxin 1 OS=Naja oxiana OX=8657 PE=1 SV=1 |
| 98 | 958 | P10117|PA2H1\_LATCO | 251.27 | 17 | 0 | 13 | 0 | 0 | 0 | 17 | 17 | 0 | 16 | 13 |  | 8.67E4 |  |  |  | 3.41E5 | 4.54E4 |  | 6.05E6 | 5.96E4 | 8 | 5 | 0 | 2 | 0 | 0 | 0 | 10 | 2 | 0 | 5 | 2 | Y | 13024 | Basic phospholipase A2 homolog 1 OS=Laticauda colubrina OX=8628 PE=1 SV=1 |
| 89 | 107 | P62377|3SOFL\_NAJNA | 250.41 | 81 | 0 | 0 | 0 | 0 | 0 | 18 | 0 | 0 | 81 | 18 |  |  |  |  |  | 1.62E6 |  |  | 1.36E8 | 1.42E5 | 13 | 13 | 0 | 0 | 0 | 0 | 0 | 2 | 0 | 0 | 16 | 1 | Y | 7014 | Cytotoxin-like basic protein OS=Naja naja OX=35670 PE=1 SV=1 |
| 110 | 84 | Q5YF89|NGFV2\_NAJSP | 244.88 | 36 | 0 | 7 | 0 | 0 | 0 | 7 | 18 | 0 | 17 | 12 |  | 1.66E5 |  |  |  | 5.84E5 | 1.76E6 |  | 1.59E6 | 5.99E5 | 8 | 8 | 0 | 3 | 0 | 0 | 0 | 2 | 5 | 0 | 5 | 3 | Y | 27030 | Venom nerve growth factor 2 OS=Naja sputatrix OX=33626 PE=2 SV=1 |
| 119 | 190 | P82464|3SO8\_NAJKA | 239.27 | 86 | 0 | 0 | 0 | 0 | 0 | 86 | 15 | 0 | 0 | 0 |  |  |  |  |  | 7.7E7 | 1.52E5 |  |  |  | 10 | 10 | 0 | 0 | 0 | 0 | 0 | 12 | 1 | 0 | 0 | 0 | Y | 7624 | Muscarinic toxin-like protein 3 OS=Naja kaouthia OX=8649 PE=1 SV=1 |
| 95 | 244 | P82885|VESP\_NAJKA | 237.31 | 56 | 0 | 56 | 0 | 0 | 0 | 0 | 19 | 0 | 19 | 56 |  | 1.47E7 |  |  |  |  | 2.07E5 |  | 1.88E4 | 1.27E7 | 6 | 6 | 0 | 11 | 0 | 0 | 0 | 0 | 1 | 0 | 1 | 7 | Y | 12038 | Thaicobrin OS=Naja kaouthia OX=8649 PE=1 SV=1 |
| 104 | 140 | Q9YGI2|3NO21\_NAJAT | 235.10 | 60 | 0 | 0 | 0 | 0 | 60 | 10 | 0 | 0 | 0 | 0 |  |  |  |  | 1.06E8 |  |  |  |  |  | 12 | 10 | 0 | 0 | 0 | 0 | 20 | 1 | 0 | 0 | 0 | 0 | Y | 9845 | Probable weak neurotoxin NNAM1 OS=Naja atra OX=8656 PE=3 SV=1 |
| 104 | 141 | O93422|3NO2H\_NAJAT | 235.10 | 60 | 0 | 0 | 0 | 0 | 60 | 10 | 0 | 0 | 0 | 0 |  |  |  |  | 1.06E8 |  |  |  |  |  | 12 | 10 | 0 | 0 | 0 | 0 | 20 | 1 | 0 | 0 | 0 | 0 | Y | 9815 | Long neurotoxin homolog OS=Naja atra OX=8656 PE=3 SV=1 |
| 73 | 251 | P80245|3SAN\_NAJAT | 229.69 | 43 | 0 | 30 | 0 | 0 | 0 | 11 | 10 | 0 | 42 | 21 |  |  |  |  |  |  |  |  | 1.94E5 |  | 11 | 1 | 0 | 8 | 0 | 0 | 0 | 6 | 1 | 0 | 9 | 3 | Y | 8980 | Cytotoxin 6 OS=Naja atra OX=8656 PE=1 SV=2 |
| 15 | 62 | P02091|HBB1\_RAT | 226.62 | 71 | 48 | 0 | 25 | 32 | 0 | 7 | 27 | 59 | 0 | 14 | 7.08E4 |  | 3.68E4 | 7.36E3 |  |  | 9.27E3 | 8.46E4 |  | 0E0 | 14 | 3 | 40 | 0 | 11 | 10 | 0 | 2 | 3 | 14 | 0 | 2 | Y | 15979 | Hemoglobin subunit beta-1 OS=Rattus norvegicus OX=10116 GN=Hbb PE=1 SV=3 |
| 120 | 64 | P84805|CRVP1\_NAJKA | 225.61 | 47 | 0 | 47 | 0 | 0 | 0 | 0 | 4 | 0 | 4 | 11 |  | 2.52E6 |  |  |  |  | 9.28E4 |  | 5.18E4 | 1.37E5 | 9 | 9 | 0 | 11 | 0 | 0 | 0 | 0 | 1 | 0 | 1 | 1 | Y | 26846 | Cysteine-rich venom protein kaouthin-1 OS=Naja kaouthia OX=8649 PE=1 SV=2 |
| 120 | 65 | Q7T1K6|CRVP1\_NAJAT | 225.61 | 47 | 0 | 47 | 0 | 0 | 0 | 0 | 4 | 0 | 4 | 11 |  | 2.52E6 |  |  |  |  | 9.28E4 |  | 5.18E4 | 1.37E5 | 9 | 9 | 0 | 11 | 0 | 0 | 0 | 0 | 1 | 0 | 1 | 1 | Y | 26882 | Cysteine-rich venom protein natrin-1 OS=Naja atra OX=8656 PE=1 SV=1 |
| 85 | 115 | D6PXE8|VM3B\_NAJAT | 221.44 | 16 | 0 | 11 | 0 | 0 | 0 | 2 | 8 | 0 | 6 | 6 |  | 4.86E6 |  |  |  | 1.78E4 | 9.75E4 |  | 3.37E5 | 4.98E6 | 8 | 5 | 0 | 8 | 0 | 0 | 0 | 1 | 4 | 0 | 3 | 7 | Y | 66246 | Zinc metalloproteinase-disintegrin-like atrase-B OS=Naja atra OX=8656 PE=1 SV=1 |
| 85 | 122 | D3TTC1|VM3KL\_NAJAT | 221.44 | 16 | 0 | 11 | 0 | 0 | 0 | 2 | 8 | 0 | 6 | 6 |  | 4.86E6 |  |  |  | 1.78E4 | 9.75E4 |  | 3.37E5 | 4.98E6 | 8 | 5 | 0 | 8 | 0 | 0 | 0 | 1 | 4 | 0 | 3 | 7 | Y | 66292 | Zinc metalloproteinase-disintegrin-like kaouthiagin-like OS=Naja atra OX=8656 PE=1 SV=1 |
| 93 | 78 | O76013|KRT36\_HUMAN | 217.76 | 26 | 1 | 1 | 1 | 0 | 24 | 1 | 1 | 0 | 1 | 0 |  |  |  |  | 4.14E7 |  |  |  |  |  | 10 | 6 | 1 | 1 | 1 | 0 | 13 | 1 | 1 | 0 | 1 | 0 | N | 52247 | Keratin, type I cuticular Ha6 OS=Homo sapiens OX=9606 GN=KRT36 PE=2 SV=1 |
| 93 | 79 | #CONTAM#K1H6\_HUMAN| | 217.76 | 26 | 1 | 1 | 1 | 0 | 24 | 1 | 1 | 0 | 1 | 0 |  |  |  |  | 4.14E7 |  |  |  |  |  | 10 | 6 | 1 | 1 | 1 | 0 | 13 | 1 | 1 | 0 | 1 | 0 | N | 52247 | sp|#CONTAM#K1H6\_HUMAN| |
| 60 | 60 | Q04695|K1C17\_HUMAN | 215.48 | 22 | 11 | 9 | 11 | 0 | 2 | 12 | 8 | 0 | 4 | 4 |  | 1.97E4 |  |  |  |  |  |  |  |  | 11 | 1 | 5 | 4 | 5 | 0 | 1 | 6 | 4 | 0 | 2 | 2 | N | 48106 | Keratin, type I cytoskeletal 17 OS=Homo sapiens OX=9606 GN=KRT17 PE=1 SV=2 |
| 17 | 81 | P11517|HBB2\_RAT | 211.50 | 50 | 35 | 0 | 19 | 19 | 0 | 7 | 14 | 50 | 0 | 5 | 1.25E4 |  |  |  |  |  |  | 3.53E3 |  |  | 12 | 1 | 39 | 0 | 10 | 8 | 0 | 2 | 2 | 13 | 0 | 1 | Y | 15982 | Hemoglobin subunit beta-2 OS=Rattus norvegicus OX=10116 PE=1 SV=2 |
| 107 | 37 | P14923|PLAK\_HUMAN | 210.93 | 22 | 7 | 0 | 10 | 0 | 10 | 5 | 0 | 0 | 0 | 0 | 3.94E4 |  | 1.53E5 |  | 9.76E5 | 1.63E5 |  |  |  |  | 12 | 12 | 3 | 0 | 5 | 0 | 5 | 3 | 0 | 0 | 0 | 0 | Y | 81745 | Junction plakoglobin OS=Homo sapiens OX=9606 GN=JUP PE=1 SV=3 |
| 107 | 38 | Q8SPJ1|PLAK\_BOVIN | 210.93 | 22 | 7 | 0 | 10 | 0 | 10 | 5 | 0 | 0 | 0 | 0 | 3.94E4 |  | 1.53E5 |  | 9.76E5 | 1.63E5 |  |  |  |  | 12 | 12 | 3 | 0 | 5 | 0 | 5 | 3 | 0 | 0 | 0 | 0 | Y | 81821 | Junction plakoglobin OS=Bos taurus OX=9913 GN=JUP PE=2 SV=1 |
| 107 | 39 | Q6P0K8|PLAK\_RAT | 210.93 | 22 | 7 | 0 | 10 | 0 | 10 | 5 | 0 | 0 | 0 | 0 | 3.94E4 |  | 1.53E5 |  | 9.76E5 | 1.63E5 |  |  |  |  | 12 | 12 | 3 | 0 | 5 | 0 | 5 | 3 | 0 | 0 | 0 | 0 | Y | 81801 | Junction plakoglobin OS=Rattus norvegicus OX=10116 GN=Jup PE=1 SV=1 |
| 107 | 40 | Q02257|PLAK\_MOUSE | 210.93 | 22 | 7 | 0 | 10 | 0 | 10 | 5 | 0 | 0 | 0 | 0 | 3.94E4 |  | 1.53E5 |  | 9.76E5 | 1.63E5 |  |  |  |  | 12 | 12 | 3 | 0 | 5 | 0 | 5 | 3 | 0 | 0 | 0 | 0 | Y | 81801 | Junction plakoglobin OS=Mus musculus OX=10090 GN=Jup PE=1 SV=3 |
| 58 | 121 | P01440|3SA2\_NAJNA | 209.91 | 93 | 0 | 92 | 0 | 0 | 0 | 33 | 13 | 0 | 47 | 13 |  | 1.11E6 |  |  |  |  |  |  |  |  | 9 | 1 | 0 | 12 | 0 | 0 | 0 | 7 | 1 | 0 | 4 | 1 | Y | 6763 | Cytotoxin 2 OS=Naja naja OX=35670 PE=1 SV=1 |
| 76 | 73 | P19012|K1C15\_HUMAN | 209.27 | 16 | 12 | 4 | 8 | 0 | 2 | 12 | 8 | 0 | 2 | 5 | 5.76E3 |  |  |  |  |  |  |  |  |  | 7 | 1 | 5 | 2 | 4 | 0 | 1 | 6 | 4 | 0 | 1 | 2 | N | 49212 | Keratin, type I cytoskeletal 15 OS=Homo sapiens OX=9606 GN=KRT15 PE=1 SV=3 |
| 112 | 86 | D5LMJ3|VM3A\_NAJAT | 208.56 | 13 | 0 | 10 | 0 | 0 | 3 | 0 | 0 | 0 | 2 | 11 |  | 3.46E6 |  |  | 2.17E6 |  |  |  | 4.05E4 | 2.66E6 | 9 | 9 | 0 | 8 | 0 | 0 | 2 | 0 | 0 | 0 | 1 | 7 | Y | 68254 | Zinc metalloproteinase-disintegrin-like atrase-A OS=Naja atra OX=8656 PE=2 SV=1 |
| 86 | 76 | P13646|K1C13\_HUMAN | 198.66 | 16 | 7 | 7 | 9 | 2 | 0 | 11 | 7 | 0 | 4 | 8 |  |  |  |  |  |  |  |  |  | 3.33E4 | 7 | 1 | 3 | 3 | 4 | 1 | 0 | 5 | 3 | 0 | 2 | 2 | Y | 49588 | Keratin, type I cytoskeletal 13 OS=Homo sapiens OX=9606 GN=KRT13 PE=1 SV=4 |
| 57 | 97 | Q8BGZ7|K2C75\_MOUSE | 191.22 | 12 | 4 | 7 | 7 | 4 | 5 | 5 | 4 | 0 | 3 | 2 |  | 3.05E4 |  |  |  |  |  |  |  |  | 7 | 1 | 4 | 7 | 6 | 2 | 3 | 7 | 4 | 0 | 2 | 1 | Y | 59741 | Keratin, type II cytoskeletal 75 OS=Mus musculus OX=10090 GN=Krt75 PE=1 SV=1 |
| 46 | 124 | P00761|TRYP\_PIG | 173.39 | 35 | 11 | 13 | 21 | 20 | 25 | 3 | 7 | 0 | 0 | 0 | 2.51E6 | 1.57E6 | 4.98E6 | 6.46E6 | 1.5E9 | 2.74E6 | 2.91E6 |  |  |  | 10 | 10 | 2 | 2 | 7 | 4 | 6 | 1 | 2 | 0 | 0 | 0 | N | 24409 | Trypsin OS=Sus scrofa OX=9823 PE=1 SV=1 |
| 46 | 125 | #CONTAM#TRYP\_PIG| | 173.39 | 35 | 11 | 13 | 21 | 20 | 25 | 3 | 7 | 0 | 0 | 0 | 2.51E6 | 1.57E6 | 4.98E6 | 6.46E6 | 1.5E9 | 2.74E6 | 2.91E6 |  |  |  | 10 | 10 | 2 | 2 | 7 | 4 | 6 | 1 | 2 | 0 | 0 | 0 | N | 24409 | sp|#CONTAM#TRYP\_PIG| |
| 67 | 109 | P19013|K2C4\_HUMAN | 170.36 | 12 | 4 | 5 | 4 | 0 | 0 | 10 | 4 | 0 | 3 | 4 |  |  |  |  |  | 5.02E4 |  |  |  | 5.33E4 | 6 | 1 | 3 | 8 | 3 | 0 | 0 | 9 | 4 | 0 | 2 | 2 | N | 57285 | Keratin, type II cytoskeletal 4 OS=Homo sapiens OX=9606 GN=KRT4 PE=1 SV=4 |
| 129 | 127 | Q9PVK7|VM3\_NAJKA | 161.82 | 8 | 0 | 0 | 0 | 0 | 0 | 2 | 6 | 0 | 0 | 0 |  |  |  |  |  | 1.23E5 | 4.47E5 |  |  |  | 3 | 3 | 0 | 0 | 0 | 0 | 0 | 1 | 2 | 0 | 0 | 0 | Y | 67662 | Zinc metalloproteinase-disintegrin-like cobrin OS=Naja kaouthia OX=8649 PE=2 SV=1 |
| 134 | 234 | Q8TF66|LRC15\_HUMAN | 153.29 | 7 | 0 | 0 | 0 | 0 | 7 | 0 | 0 | 0 | 0 | 0 |  |  |  |  | 5.03E7 |  |  |  |  |  | 3 | 3 | 0 | 0 | 0 | 0 | 4 | 0 | 0 | 0 | 0 | 0 | N | 64366 | Leucine-rich repeat-containing protein 15 OS=Homo sapiens OX=9606 GN=LRRC15 PE=2 SV=2 |
| 131 | 142 | Q02413|DSG1\_HUMAN | 142.63 | 6 | 2 | 0 | 1 | 0 | 3 | 0 | 0 | 0 | 0 | 0 | 2.87E4 |  | 1.67E4 |  | 1.09E6 |  |  |  |  |  | 4 | 4 | 2 | 0 | 1 | 0 | 2 | 0 | 0 | 0 | 0 | 0 | N | 113748 | Desmoglein-1 OS=Homo sapiens OX=9606 GN=DSG1 PE=1 SV=2 |
| 103 | 258 | P01454|3SA9\_NAJHA | 141.19 | 57 | 0 | 13 | 0 | 0 | 0 | 15 | 13 | 0 | 45 | 35 |  |  |  |  |  |  |  |  |  | 1.45E5 | 6 | 1 | 0 | 2 | 0 | 0 | 0 | 6 | 1 | 0 | 5 | 2 | Y | 6669 | Cytotoxin 9 OS=Naja annulifera OX=96794 PE=1 SV=1 |
| 103 | 325 | P01453|3SAA\_NAJHA | 141.19 | 57 | 0 | 13 | 0 | 0 | 0 | 15 | 13 | 0 | 45 | 35 |  |  |  |  |  |  |  |  |  | 1.45E5 | 6 | 1 | 0 | 2 | 0 | 0 | 0 | 6 | 1 | 0 | 5 | 2 | Y | 6682 | Cytotoxin 10 OS=Naja annulifera OX=96794 PE=1 SV=1 |
| 115 | 264 | P81605|DCD\_HUMAN | 140.93 | 41 | 41 | 31 | 41 | 31 | 10 | 21 | 41 | 0 | 20 | 0 | 1.28E5 | 1.67E5 | 3.74E4 | 8.83E4 | 2.67E5 | 1.8E5 | 4.86E4 |  | 3.22E4 |  | 3 | 3 | 3 | 2 | 3 | 2 | 1 | 1 | 3 | 0 | 2 | 0 | Y | 11284 | Dermcidin OS=Homo sapiens OX=9606 GN=DCD PE=1 SV=2 |
| 128 | 342 | P01400|3NO2B\_NAJME | 134.39 | 43 | 0 | 0 | 0 | 0 | 43 | 14 | 0 | 0 | 0 | 0 |  |  |  |  | 0E0 |  |  |  |  |  | 4 | 1 | 0 | 0 | 0 | 0 | 7 | 1 | 0 | 0 | 0 | 0 | Y | 7430 | Weak toxin S4C11 OS=Naja melanoleuca OX=8643 PE=1 SV=1 |
| 148 | 423 | Q01469|FABP5\_HUMAN | 130.68 | 16 | 0 | 0 | 0 | 0 | 16 | 0 | 0 | 0 | 0 | 0 |  |  |  |  | 3.95E6 |  |  |  |  |  | 2 | 2 | 0 | 0 | 0 | 0 | 3 | 0 | 0 | 0 | 0 | 0 | N | 15164 | Fatty acid-binding protein 5 OS=Homo sapiens OX=9606 GN=FABP5 PE=1 SV=3 |
| 139 | 218 | I2C090|VCO3\_OPHHA | 130.56 | 2 | 0 | 2 | 0 | 0 | 0 | 0 | 0 | 0 | 0 | 0 |  | 3.3E5 |  |  |  |  | 3.52E5 |  |  |  | 4 | 4 | 0 | 3 | 0 | 0 | 0 | 0 | 1 | 0 | 0 | 0 | Y | 183927 | Ophiophagus venom factor OS=Ophiophagus hannah OX=8665 PE=1 SV=1 |
| 133 | 399 | Q63041|A1M\_RAT | 126.43 | 2 | 2 | 0 | 2 | 2 | 0 | 0 | 0 | 2 | 0 | 0 | 4.93E4 |  | 4.26E4 | 2.1E4 |  |  |  | 8.58E4 |  |  | 2 | 2 | 1 | 0 | 1 | 1 | 0 | 0 | 0 | 2 | 0 | 0 | N | 167124 | Alpha-1-macroglobulin OS=Rattus norvegicus OX=10116 GN=A1m PE=1 SV=1 |
| 151 | 329 | P01085|IAA1\_WHEAT | 124.90 | 26 | 0 | 0 | 0 | 0 | 0 | 0 | 0 | 0 | 26 | 0 |  |  |  |  |  |  |  |  | 1.49E5 |  | 2 | 2 | 0 | 0 | 0 | 0 | 0 | 0 | 0 | 0 | 2 | 0 | Y | 13337 | Alpha-amylase inhibitor 0.19 OS=Triticum aestivum OX=4565 PE=1 SV=1 |
| 78 | 1184 | P00986|VKT2\_NAJNI | 122.63 | 28 | 0 | 0 | 0 | 28 | 0 | 0 | 21 | 21 | 0 | 0 |  |  |  | 1.25E5 |  |  |  |  |  |  | 2 | 1 | 0 | 0 | 0 | 24 | 0 | 0 | 1 | 1 | 0 | 0 | Y | 6466 | Kunitz-type serine protease inhibitor 2 OS=Naja nivea OX=8655 PE=1 SV=1 |
| 141 | 240 | P02755|LACB\_BUBBU | 122.10 | 25 | 25 | 0 | 0 | 0 | 0 | 0 | 0 | 0 | 0 | 0 | 3.97E4 |  |  |  |  |  |  |  |  |  | 3 | 3 | 3 | 0 | 0 | 0 | 0 | 0 | 0 | 0 | 0 | 0 | N | 20023 | Beta-lactoglobulin OS=Bubalus bubalis OX=89462 GN=LGB PE=1 SV=2 |
| 142 | 308 | E3P6P4|CYT\_NAJKA | 118.34 | 25 | 0 | 0 | 0 | 0 | 0 | 0 | 0 | 0 | 0 | 25 |  |  |  |  |  |  |  |  |  | 6.46E5 | 3 | 3 | 0 | 0 | 0 | 0 | 0 | 0 | 0 | 0 | 0 | 3 | Y | 15772 | Cystatin OS=Naja kaouthia OX=8649 PE=2 SV=1 |
| 152 | 336 | P09919|CSF3\_HUMAN | 116.90 | 14 | 0 | 0 | 0 | 0 | 14 | 0 | 0 | 0 | 0 | 0 |  |  |  |  | 5.67E5 |  |  |  |  |  | 2 | 2 | 0 | 0 | 0 | 0 | 2 | 0 | 0 | 0 | 0 | 0 | N | 22293 | Granulocyte colony-stimulating factor OS=Homo sapiens OX=9606 GN=CSF3 PE=1 SV=1 |
| 147 | 410 | P02768|ALBU\_HUMAN | 111.52 | 4 | 0 | 0 | 0 | 0 | 4 | 0 | 0 | 0 | 0 | 0 |  |  |  |  | 0E0 |  |  |  |  |  | 2 | 1 | 0 | 0 | 0 | 0 | 3 | 0 | 0 | 0 | 0 | 0 | N | 69367 | Serum albumin OS=Homo sapiens OX=9606 GN=ALB PE=1 SV=2 |
| 147 | 411 | #CONTAM#ALBU\_HUMAN| | 111.52 | 4 | 0 | 0 | 0 | 0 | 4 | 0 | 0 | 0 | 0 | 0 |  |  |  |  | 0E0 |  |  |  |  |  | 2 | 1 | 0 | 0 | 0 | 0 | 3 | 0 | 0 | 0 | 0 | 0 | N | 69367 | sp|#CONTAM#ALBU\_HUMAN| |
| 147 | 412 | Q5NVH5|ALBU\_PONAB | 111.52 | 4 | 0 | 0 | 0 | 0 | 4 | 0 | 0 | 0 | 0 | 0 |  |  |  |  | 0E0 |  |  |  |  |  | 2 | 1 | 0 | 0 | 0 | 0 | 3 | 0 | 0 | 0 | 0 | 0 | N | 69480 | Serum albumin OS=Pongo abelii OX=9601 GN=ALB PE=2 SV=2 |
| 137 | 301 | P04764|ENOA\_RAT | 105.72 | 3 | 0 | 0 | 0 | 0 | 3 | 0 | 0 | 0 | 0 | 0 |  |  |  |  | 4.86E4 |  |  |  |  |  | 1 | 1 | 0 | 0 | 0 | 0 | 2 | 0 | 0 | 0 | 0 | 0 | N | 47128 | Alpha-enolase OS=Rattus norvegicus OX=10116 GN=Eno1 PE=1 SV=4 |
| 144 | 263 | B6EWW8|V5NTD\_GLOBR | 103.40 | 3 | 0 | 0 | 0 | 0 | 0 | 0 | 0 | 0 | 0 | 3 |  |  |  |  |  |  |  |  |  | 4.84E4 | 1 | 1 | 0 | 0 | 0 | 0 | 0 | 0 | 0 | 0 | 0 | 1 | N | 64434 | Snake venom 5'-nucleotidase OS=Gloydius brevicaudus OX=259325 PE=2 SV=1 |
| 163 | 934 | P14613|3S1B1\_NAJKA | 102.68 | 19 | 0 | 0 | 0 | 19 | 0 | 0 | 0 | 0 | 0 | 0 |  |  |  | 1.97E5 |  |  |  |  |  |  | 1 | 1 | 0 | 0 | 0 | 1 | 0 | 0 | 0 | 0 | 0 | 0 | Y | 6983 | Short neurotoxin 1 OS=Naja kaouthia OX=8649 PE=1 SV=1 |
| 163 | 1156 | Q9PSN6|3S13\_NAJSP | 102.68 | 19 | 0 | 0 | 0 | 19 | 0 | 0 | 0 | 0 | 0 | 0 |  |  |  | 1.97E5 |  |  |  |  |  |  | 1 | 1 | 0 | 0 | 0 | 1 | 0 | 0 | 0 | 0 | 0 | 0 | Y | 6958 | Neurotoxin 3 OS=Naja sputatrix OX=33626 PE=1 SV=1 |
| 163 | 1179 | P60770|3S1CB\_NAJAT | 102.68 | 14 | 0 | 0 | 0 | 14 | 0 | 0 | 0 | 0 | 0 | 0 |  |  |  | 1.97E5 |  |  |  |  |  |  | 1 | 1 | 0 | 0 | 0 | 1 | 0 | 0 | 0 | 0 | 0 | 0 | Y | 9262 | Cobrotoxin OS=Naja atra OX=8656 PE=1 SV=1 |
| 163 | 1180 | P60771|3S1CB\_NAJKA | 102.68 | 14 | 0 | 0 | 0 | 14 | 0 | 0 | 0 | 0 | 0 | 0 |  |  |  | 1.97E5 |  |  |  |  |  |  | 1 | 1 | 0 | 0 | 0 | 1 | 0 | 0 | 0 | 0 | 0 | 0 | Y | 9262 | Cobrotoxin OS=Naja kaouthia OX=8649 PE=1 SV=2 |
| 163 | 1181 | Q9PTT0|3S1CB\_NAJNA | 102.68 | 14 | 0 | 0 | 0 | 14 | 0 | 0 | 0 | 0 | 0 | 0 |  |  |  | 1.97E5 |  |  |  |  |  |  | 1 | 1 | 0 | 0 | 0 | 1 | 0 | 0 | 0 | 0 | 0 | 0 | Y | 9262 | Cobrotoxin homolog OS=Naja naja OX=35670 PE=1 SV=1 |
| 162 | 493 | P31947|1433S\_HUMAN | 100.45 | 14 | 0 | 0 | 0 | 0 | 14 | 0 | 0 | 0 | 0 | 0 |  |  |  |  | 4.88E5 |  |  |  |  |  | 2 | 2 | 0 | 0 | 0 | 0 | 2 | 0 | 0 | 0 | 0 | 0 | N | 27774 | 14-3-3 protein sigma OS=Homo sapiens OX=9606 GN=SFN PE=1 SV=1 |
| 162 | 501 | Q0VC36|1433S\_BOVIN | 100.45 | 14 | 0 | 0 | 0 | 0 | 14 | 0 | 0 | 0 | 0 | 0 |  |  |  |  | 4.88E5 |  |  |  |  |  | 2 | 2 | 0 | 0 | 0 | 0 | 2 | 0 | 0 | 0 | 0 | 0 | N | 27849 | 14-3-3 protein sigma OS=Bos taurus OX=9913 GN=SFN PE=2 SV=1 |
| 162 | 502 | O77642|1433S\_SHEEP | 100.45 | 14 | 0 | 0 | 0 | 0 | 14 | 0 | 0 | 0 | 0 | 0 |  |  |  |  | 4.88E5 |  |  |  |  |  | 2 | 2 | 0 | 0 | 0 | 0 | 2 | 0 | 0 | 0 | 0 | 0 | N | 27849 | 14-3-3 protein sigma OS=Ovis aries OX=9940 GN=SFN PE=2 SV=1 |
| 162 | 545 | O70456|1433S\_MOUSE | 100.45 | 14 | 0 | 0 | 0 | 0 | 14 | 0 | 0 | 0 | 0 | 0 |  |  |  |  | 4.88E5 |  |  |  |  |  | 2 | 2 | 0 | 0 | 0 | 0 | 2 | 0 | 0 | 0 | 0 | 0 | N | 27706 | 14-3-3 protein sigma OS=Mus musculus OX=10090 GN=Sfn PE=1 SV=2 |
| 140 | 965 | P29181|3NO27\_NAJNA | 96.32 | 20 | 0 | 0 | 0 | 0 | 20 | 0 | 0 | 0 | 0 | 0 |  |  |  |  | 1.46E6 |  |  |  |  |  | 3 | 3 | 0 | 0 | 0 | 0 | 3 | 0 | 0 | 0 | 0 | 0 | Y | 7637 | Weak neurotoxin 7 OS=Naja naja OX=35670 PE=1 SV=1 |
| 132 | 236 | Q5T749|KPRP\_HUMAN | 94.89 | 3 | 3 | 0 | 0 | 0 | 0 | 0 | 0 | 0 | 0 | 0 | 2.74E4 |  |  |  |  |  |  |  |  |  | 2 | 2 | 2 | 0 | 0 | 0 | 0 | 0 | 0 | 0 | 0 | 0 | Y | 64136 | Keratinocyte proline-rich protein OS=Homo sapiens OX=9606 GN=KPRP PE=1 SV=1 |
| 146 | 271 | P04406|G3P\_HUMAN | 93.08 | 12 | 0 | 4 | 0 | 0 | 4 | 4 | 0 | 0 | 0 | 0 |  | 1.56E4 |  |  | 9.65E4 | 1.92E4 |  |  |  |  | 3 | 3 | 0 | 1 | 0 | 0 | 1 | 1 | 0 | 0 | 0 | 0 | Y | 36053 | Glyceraldehyde-3-phosphate dehydrogenase OS=Homo sapiens OX=9606 GN=GAPDH PE=1 SV=3 |
| 138 | 294 | P02845|VIT2\_CHICK | 89.40 | 1 | 0 | 1 | 0 | 0 | 0 | 1 | 0 | 0 | 1 | 0 |  | 4.15E4 |  |  |  | 2.18E4 |  |  | 9.55E3 |  | 1 | 1 | 0 | 1 | 0 | 0 | 0 | 1 | 0 | 0 | 1 | 0 | N | 204807 | Vitellogenin-2 OS=Gallus gallus OX=9031 GN=VTG2 PE=1 SV=1 |
| 149 | 400 | P02769|ALBU\_BOVIN | 89.19 | 4 | 0 | 0 | 0 | 0 | 4 | 0 | 0 | 0 | 0 | 0 |  |  |  |  | 2.76E4 |  |  |  |  |  | 2 | 1 | 0 | 0 | 0 | 0 | 3 | 0 | 0 | 0 | 0 | 0 | N | 69294 | Serum albumin OS=Bos taurus OX=9913 GN=ALB PE=1 SV=4 |
| 149 | 401 | #CONTAM#ALBU\_BOVIN| | 89.19 | 4 | 0 | 0 | 0 | 0 | 4 | 0 | 0 | 0 | 0 | 0 |  |  |  |  | 2.76E4 |  |  |  |  |  | 2 | 1 | 0 | 0 | 0 | 0 | 3 | 0 | 0 | 0 | 0 | 0 | N | 69294 | sp|#CONTAM#ALBU\_BOVIN| |
| 161 | 657 | P82462|3SUC1\_NAJKA | 83.08 | 35 | 0 | 0 | 0 | 0 | 0 | 35 | 0 | 0 | 0 | 0 |  |  |  |  |  | 6.13E5 |  |  |  |  | 2 | 2 | 0 | 0 | 0 | 0 | 0 | 2 | 0 | 0 | 0 | 0 | Y | 7366 | Muscarinic toxin-like protein 1 OS=Naja kaouthia OX=8649 PE=1 SV=1 |
| 165 | 348 | P02863|GDA0\_WHEAT | 80.56 | 9 | 0 | 0 | 0 | 0 | 0 | 0 | 0 | 0 | 9 | 0 |  |  |  |  |  |  |  |  | 7.99E4 |  | 1 | 1 | 0 | 0 | 0 | 0 | 0 | 0 | 0 | 0 | 1 | 0 | N | 32963 | Alpha/beta-gliadin OS=Triticum aestivum OX=4565 PE=2 SV=2 |
| 160 | 343 | P30171|ACT11\_SOLTU | 78.19 | 3 | 0 | 0 | 0 | 0 | 3 | 0 | 0 | 0 | 0 | 0 |  |  |  |  | 1.62E5 |  |  |  |  |  | 1 | 1 | 0 | 0 | 0 | 0 | 1 | 0 | 0 | 0 | 0 | 0 | N | 41643 | Actin-97 OS=Solanum tuberosum OX=4113 GN=AC97 PE=3 SV=1 |
| 160 | 506 | A3C6D7|ACT2\_ORYSJ | 78.19 | 3 | 0 | 0 | 0 | 0 | 3 | 0 | 0 | 0 | 0 | 0 |  |  |  |  | 1.62E5 |  |  |  |  |  | 1 | 1 | 0 | 0 | 0 | 0 | 1 | 0 | 0 | 0 | 0 | 0 | N | 41680 | Actin-2 OS=Oryza sativa subsp. japonica OX=39947 GN=ACT2 PE=2 SV=1 |
| 160 | 543 | P30173|ACT13\_SOLTU | 78.19 | 3 | 0 | 0 | 0 | 0 | 3 | 0 | 0 | 0 | 0 | 0 |  |  |  |  | 1.62E5 |  |  |  |  |  | 1 | 1 | 0 | 0 | 0 | 0 | 1 | 0 | 0 | 0 | 0 | 0 | N | 41644 | Actin-101 OS=Solanum tuberosum OX=4113 GN=AC101 PE=3 SV=1 |
| 159 | 443 | #CONTAM#CAS1\_BOVIN| | 76.12 | 5 | 5 | 0 | 0 | 0 | 0 | 0 | 0 | 0 | 0 | 0 | 1.7E4 |  |  |  |  |  |  |  |  |  | 1 | 1 | 1 | 0 | 0 | 0 | 0 | 0 | 0 | 0 | 0 | 0 | N | 24529 | sp|#CONTAM#CAS1\_BOVIN| |
| 159 | 444 | P02662|CASA1\_BOVIN | 76.12 | 5 | 5 | 0 | 0 | 0 | 0 | 0 | 0 | 0 | 0 | 0 | 1.7E4 |  |  |  |  |  |  |  |  |  | 1 | 1 | 1 | 0 | 0 | 0 | 0 | 0 | 0 | 0 | 0 | 0 | N | 24529 | Alpha-S1-casein OS=Bos taurus OX=9913 GN=CSN1S1 PE=1 SV=2 |
| 159 | 710 | O62823|CASA1\_BUBBU | 76.12 | 5 | 5 | 0 | 0 | 0 | 0 | 0 | 0 | 0 | 0 | 0 | 1.7E4 |  |  |  |  |  |  |  |  |  | 1 | 1 | 1 | 0 | 0 | 0 | 0 | 0 | 0 | 0 | 0 | 0 | N | 24327 | Alpha-S1-casein OS=Bubalus bubalis OX=89462 GN=CSN1S1 PE=2 SV=2 |
| 193 | 591 | Q6IE52|MUG2\_RAT | 68.20 | 1 | 1 | 0 | 0 | 0 | 0 | 0 | 0 | 0 | 0 | 0 | 4.82E4 |  |  |  |  |  |  |  |  |  | 1 | 1 | 1 | 0 | 0 | 0 | 0 | 0 | 0 | 0 | 0 | 0 | N | 161588 | Murinoglobulin-2 OS=Rattus norvegicus OX=10116 GN=Mug2 PE=1 SV=1 |
| 193 | 720 | Q03626|MUG1\_RAT | 68.20 | 1 | 1 | 0 | 0 | 0 | 0 | 0 | 0 | 0 | 0 | 0 | 4.82E4 |  |  |  |  |  |  |  |  |  | 1 | 1 | 1 | 0 | 0 | 0 | 0 | 0 | 0 | 0 | 0 | 0 | N | 165325 | Murinoglobulin-1 OS=Rattus norvegicus OX=10116 GN=Mug1 PE=2 SV=1 |
| 229 | 978 | Q767L7|TBB5\_PIG | 63.42 | 3 | 0 | 0 | 0 | 0 | 3 | 0 | 0 | 0 | 0 | 0 |  |  |  |  | 3.51E4 |  |  |  |  |  | 1 | 1 | 0 | 0 | 0 | 0 | 1 | 0 | 0 | 0 | 0 | 0 | N | 49671 | Tubulin beta chain OS=Sus scrofa OX=9823 GN=TUBB PE=2 SV=1 |
| 232 | 1196 | P82849|3S1B2\_NAJKA | 61.32 | 27 | 0 | 0 | 27 | 0 | 0 | 0 | 0 | 0 | 0 | 0 |  |  | 3.78E4 |  |  |  |  |  |  |  | 1 | 1 | 0 | 0 | 1 | 0 | 0 | 0 | 0 | 0 | 0 | 0 | Y | 6862 | Cobrotoxin II OS=Naja kaouthia OX=8649 PE=1 SV=1 |
| 164 | 1063 | P27139|CAH2\_RAT | 58.12 | 9 | 9 | 0 | 0 | 0 | 0 | 0 | 0 | 0 | 0 | 0 | 3.06E5 |  |  |  |  |  |  |  |  |  | 1 | 1 | 1 | 0 | 0 | 0 | 0 | 0 | 0 | 0 | 0 | 0 | N | 29114 | Carbonic anhydrase 2 OS=Rattus norvegicus OX=10116 GN=Ca2 PE=1 SV=2 |
| 158 | 1207 | P58370|3NO48\_MICCO | 57.74 | 15 | 0 | 0 | 0 | 0 | 0 | 0 | 15 | 0 | 0 | 0 |  |  |  |  |  |  | 1.26E8 |  |  |  | 1 | 1 | 0 | 0 | 0 | 0 | 0 | 0 | 2 | 0 | 0 | 0 | Y | 9591 | Alpha-neurotoxin homolog 8 OS=Micrurus corallinus OX=54390 GN=NXH8 PE=1 SV=1 |
| 166 | 819 | Q9TSI0|CASB\_BUBBU | 57.09 | 5 | 5 | 0 | 0 | 0 | 0 | 5 | 0 | 0 | 0 | 0 | 2.53E5 |  |  |  |  | 3.09E4 |  |  |  |  | 1 | 1 | 1 | 0 | 0 | 0 | 0 | 1 | 0 | 0 | 0 | 0 | N | 25106 | Beta-casein OS=Bubalus bubalis OX=89462 GN=CSN2 PE=2 SV=1 |
| 166 | 1213 | P11839|CASB\_SHEEP | 57.09 | 5 | 5 | 0 | 0 | 0 | 0 | 5 | 0 | 0 | 0 | 0 | 2.53E5 |  |  |  |  | 3.09E4 |  |  |  |  | 1 | 1 | 1 | 0 | 0 | 0 | 0 | 1 | 0 | 0 | 0 | 0 | N | 24875 | Beta-casein OS=Ovis aries OX=9940 GN=CSN2 PE=1 SV=3 |
| 166 | 1214 | P33048|CASB\_CAPHI | 57.09 | 5 | 5 | 0 | 0 | 0 | 0 | 5 | 0 | 0 | 0 | 0 | 2.53E5 |  |  |  |  | 3.09E4 |  |  |  |  | 1 | 1 | 1 | 0 | 0 | 0 | 0 | 1 | 0 | 0 | 0 | 0 | N | 24865 | Beta-casein OS=Capra hircus OX=9925 GN=CSN2 PE=2 SV=1 |
| 166 | 1215 | #CONTAM#CASB\_BOVIN| | 57.09 | 5 | 5 | 0 | 0 | 0 | 0 | 5 | 0 | 0 | 0 | 0 | 2.53E5 |  |  |  |  | 3.09E4 |  |  |  |  | 1 | 1 | 1 | 0 | 0 | 0 | 0 | 1 | 0 | 0 | 0 | 0 | N | 25107 | sp|#CONTAM#CASB\_BOVIN| |
| 166 | 1216 | P02666|CASB\_BOVIN | 57.09 | 5 | 5 | 0 | 0 | 0 | 0 | 5 | 0 | 0 | 0 | 0 | 2.53E5 |  |  |  |  | 3.09E4 |  |  |  |  | 1 | 1 | 1 | 0 | 0 | 0 | 0 | 1 | 0 | 0 | 0 | 0 | N | 25107 | Beta-casein OS=Bos taurus OX=9913 GN=CSN2 PE=1 SV=2 |
| 202 | 377 | Q13835|PKP1\_HUMAN | 56.19 | 2 | 0 | 0 | 0 | 0 | 2 | 0 | 0 | 0 | 0 | 0 |  |  |  |  | 0E0 |  |  |  |  |  | 1 | 1 | 0 | 0 | 0 | 0 | 1 | 0 | 0 | 0 | 0 | 0 | N | 82861 | Plakophilin-1 OS=Homo sapiens OX=9606 GN=PKP1 PE=1 SV=2 |
| 143 | 464 | Q5D862|FILA2\_HUMAN | 53.39 | 0 | 0 | 0 | 0 | 0 | 0 | 0 | 0 | 0 | 0 | 0 |  | 6.98E4 | 1.36E4 |  |  | 1.36E5 |  |  |  |  | 1 | 1 | 0 | 1 | 1 | 0 | 0 | 1 | 0 | 0 | 0 | 0 | N | 248072 | Filaggrin-2 OS=Homo sapiens OX=9606 GN=FLG2 PE=1 SV=1 |
| 200 | 737 | Q28864|TFPI1\_MACMU | 52.11 | 4 | 0 | 0 | 0 | 4 | 0 | 0 | 0 | 0 | 0 | 0 |  |  |  | 3.46E6 |  |  |  |  |  |  | 1 | 1 | 0 | 0 | 0 | 1 | 0 | 0 | 0 | 0 | 0 | 0 | Y | 35085 | Tissue factor pathway inhibitor OS=Macaca mulatta OX=9544 GN=TFPI PE=2 SV=1 |
| 228 | 923 | P12001|RL18\_RAT | 50.50 | 5 | 0 | 0 | 0 | 0 | 5 | 0 | 0 | 0 | 0 | 0 |  |  |  |  | 1.17E4 |  |  |  |  |  | 1 | 1 | 0 | 0 | 0 | 0 | 1 | 0 | 0 | 0 | 0 | 0 | N | 21659 | 60S ribosomal protein L18 OS=Rattus norvegicus OX=10116 GN=Rpl18 PE=1 SV=2 |
| 228 | 924 | P35980|RL18\_MOUSE | 50.50 | 5 | 0 | 0 | 0 | 0 | 5 | 0 | 0 | 0 | 0 | 0 |  |  |  |  | 1.17E4 |  |  |  |  |  | 1 | 1 | 0 | 0 | 0 | 0 | 1 | 0 | 0 | 0 | 0 | 0 | N | 21645 | 60S ribosomal protein L18 OS=Mus musculus OX=10090 GN=Rpl18 PE=1 SV=3 |
| 228 | 925 | Q5E973|RL18\_BOVIN | 50.50 | 5 | 0 | 0 | 0 | 0 | 5 | 0 | 0 | 0 | 0 | 0 |  |  |  |  | 1.17E4 |  |  |  |  |  | 1 | 1 | 0 | 0 | 0 | 0 | 1 | 0 | 0 | 0 | 0 | 0 | N | 21535 | 60S ribosomal protein L18 OS=Bos taurus OX=9913 GN=RPL18 PE=2 SV=3 |
| 228 | 926 | D0VWQ3|RL18\_CANLF | 50.50 | 5 | 0 | 0 | 0 | 0 | 5 | 0 | 0 | 0 | 0 | 0 |  |  |  |  | 1.17E4 |  |  |  |  |  | 1 | 1 | 0 | 0 | 0 | 0 | 1 | 0 | 0 | 0 | 0 | 0 | N | 21592 | Ribosomal protein L18 OS=Canis lupus familiaris OX=9615 GN=RPL18 PE=1 SV=2 |
| 228 | 927 | Q4R5H8|RL18\_MACFA | 50.50 | 5 | 0 | 0 | 0 | 0 | 5 | 0 | 0 | 0 | 0 | 0 |  |  |  |  | 1.17E4 |  |  |  |  |  | 1 | 1 | 0 | 0 | 0 | 0 | 1 | 0 | 0 | 0 | 0 | 0 | N | 21634 | 60S ribosomal protein L18 OS=Macaca fascicularis OX=9541 GN=RPL18 PE=2 SV=1 |
| 228 | 928 | Q07020|RL18\_HUMAN | 50.50 | 5 | 0 | 0 | 0 | 0 | 5 | 0 | 0 | 0 | 0 | 0 |  |  |  |  | 1.17E4 |  |  |  |  |  | 1 | 1 | 0 | 0 | 0 | 0 | 1 | 0 | 0 | 0 | 0 | 0 | N | 21634 | 60S ribosomal protein L18 OS=Homo sapiens OX=9606 GN=RPL18 PE=1 SV=2 |
| 228 | 929 | Q95342|RL18\_PIG | 50.50 | 5 | 0 | 0 | 0 | 0 | 5 | 0 | 0 | 0 | 0 | 0 |  |  |  |  | 1.17E4 |  |  |  |  |  | 1 | 1 | 0 | 0 | 0 | 0 | 1 | 0 | 0 | 0 | 0 | 0 | N | 22304 | 60S ribosomal protein L18 OS=Sus scrofa OX=9823 GN=RPL18 PE=1 SV=4 |
| 237 | 1222 | #CONTAM#THIO\_HUMAN| | 50.08 | 12 | 0 | 0 | 12 | 0 | 0 | 0 | 0 | 0 | 0 | 0 |  |  | 1.05E4 |  |  |  |  |  |  |  | 1 | 1 | 0 | 0 | 1 | 0 | 0 | 0 | 0 | 0 | 0 | 0 | N | 11606 | sp|#CONTAM#THIO\_HUMAN| |
| 237 | 1223 | P10599|THIO\_HUMAN | 50.08 | 12 | 0 | 0 | 12 | 0 | 0 | 0 | 0 | 0 | 0 | 0 |  |  | 1.05E4 |  |  |  |  |  |  |  | 1 | 1 | 0 | 0 | 1 | 0 | 0 | 0 | 0 | 0 | 0 | 0 | N | 11737 | Thioredoxin OS=Homo sapiens OX=9606 GN=TXN PE=1 SV=3 |
| 237 | 1224 | Q5R9M3|THIO\_PONAB | 50.08 | 12 | 0 | 0 | 12 | 0 | 0 | 0 | 0 | 0 | 0 | 0 |  |  | 1.05E4 |  |  |  |  |  |  |  | 1 | 1 | 0 | 0 | 1 | 0 | 0 | 0 | 0 | 0 | 0 | 0 | N | 11885 | Thioredoxin OS=Pongo abelii OX=9601 GN=TXN PE=3 SV=3 |
| 205 | 588 | Q08554|DSC1\_HUMAN | 49.57 | 1 | 0 | 0 | 1 | 0 | 0 | 0 | 0 | 0 | 0 | 0 |  |  | 5.65E3 |  |  |  |  |  |  |  | 1 | 1 | 0 | 0 | 1 | 0 | 0 | 0 | 0 | 0 | 0 | 0 | Y | 99987 | Desmocollin-1 OS=Homo sapiens OX=9606 GN=DSC1 PE=1 SV=2 |
| 205 | 730 | Q01107|DSC1\_BOVIN | 49.57 | 1 | 0 | 0 | 1 | 0 | 0 | 0 | 0 | 0 | 0 | 0 |  |  | 5.65E3 |  |  |  |  |  |  |  | 1 | 1 | 0 | 0 | 1 | 0 | 0 | 0 | 0 | 0 | 0 | 0 | Y | 99648 | Desmocollin-1 OS=Bos taurus OX=9913 GN=DSC1 PE=1 SV=1 |
| 218 | 832 | P17314|IAAC3\_WHEAT | 46.84 | 9 | 0 | 0 | 0 | 0 | 0 | 0 | 0 | 0 | 9 | 0 |  |  |  |  |  |  |  |  | 2.2E5 |  | 1 | 1 | 0 | 0 | 0 | 0 | 0 | 0 | 0 | 0 | 1 | 0 | N | 18221 | Alpha-amylase/trypsin inhibitor CM3 OS=Triticum aestivum OX=4565 PE=1 SV=1 |
| 224 | 1272 | P21292|GDBX\_WHEAT | 45.57 | 4 | 0 | 0 | 0 | 0 | 0 | 0 | 0 | 0 | 4 | 0 |  |  |  |  |  |  |  |  | 1.49E4 |  | 1 | 1 | 0 | 0 | 0 | 0 | 0 | 0 | 0 | 0 | 1 | 0 | N | 34300 | Gamma-gliadin OS=Triticum aestivum OX=4565 PE=3 SV=1 |
| 190 | 509 | Q23977|HEP\_DROME | 45.24 | 2 | 0 | 0 | 0 | 0 | 2 | 0 | 0 | 0 | 0 | 0 |  |  |  |  | 0E0 |  |  |  |  |  | 1 | 1 | 0 | 0 | 0 | 0 | 1 | 0 | 0 | 0 | 0 | 0 | Y | 125108 | Dual specificity mitogen-activated protein kinase kinase hemipterous OS=Drosophila melanogaster OX=7227 GN=hep PE=1 SV=2 |
| 212 | 746 | Q62673|PLK1\_RAT | 45.10 | 3 | 0 | 0 | 0 | 0 | 0 | 0 | 0 | 0 | 3 | 0 |  |  |  |  |  |  |  |  | 1.3E4 |  | 1 | 1 | 0 | 0 | 0 | 0 | 0 | 0 | 0 | 0 | 1 | 0 | N | 68291 | Serine/threonine-protein kinase PLK1 OS=Rattus norvegicus OX=10116 GN=Plk1 PE=2 SV=2 |
| 167 | 1259 | Q5T750|XP32\_HUMAN | 45.07 | 3 | 3 | 0 | 3 | 0 | 0 | 0 | 0 | 0 | 0 | 0 | 2.99E4 |  | 3.18E4 |  |  |  |  |  |  |  | 1 | 1 | 1 | 0 | 1 | 0 | 0 | 0 | 0 | 0 | 0 | 0 | N | 26238 | Skin-specific protein 32 OS=Homo sapiens OX=9606 GN=XP32 PE=1 SV=1 |
| 174 | 600 | Q6P158|DHX57\_HUMAN | 44.63 | 2 | 0 | 0 | 0 | 0 | 2 | 0 | 0 | 0 | 0 | 0 |  |  |  |  | 7.46E4 |  |  |  |  |  | 1 | 1 | 0 | 0 | 0 | 0 | 1 | 0 | 0 | 0 | 0 | 0 | N | 155604 | Putative ATP-dependent RNA helicase DHX57 OS=Homo sapiens OX=9606 GN=DHX57 PE=1 SV=2 |
| 238 | 1378 | Q2G9A3|RSMH\_NOVAD | 44.31 | 5 | 0 | 0 | 0 | 0 | 5 | 0 | 0 | 0 | 0 | 0 |  |  |  |  | 4.4E6 |  |  |  |  |  | 1 | 1 | 0 | 0 | 0 | 0 | 1 | 0 | 0 | 0 | 0 | 0 | N | 34987 | Ribosomal RNA small subunit methyltransferase H OS=Novosphingobium aromaticivorans (strain ATCC 700278 / DSM 12444 / CIP 105152 / NBRC 16084 / F199) OX=279238 GN=rsmH PE=3 SV=1 |
| 222 | 1020 | P25680|3NO2A\_NAJNI | 44.01 | 15 | 0 | 0 | 0 | 0 | 15 | 0 | 0 | 0 | 0 | 0 |  |  |  |  | 5.98E6 |  |  |  |  |  | 1 | 1 | 0 | 0 | 0 | 0 | 1 | 0 | 0 | 0 | 0 | 0 | N | 7545 | Weak toxin CM-10 OS=Naja nivea OX=8655 PE=1 SV=1 |
| 204 | 495 | Q96P63|SPB12\_HUMAN | 43.94 | 2 | 2 | 0 | 0 | 0 | 0 | 0 | 0 | 0 | 0 | 0 | 1.18E4 |  |  |  |  |  |  |  |  |  | 1 | 1 | 1 | 0 | 0 | 0 | 0 | 0 | 0 | 0 | 0 | 0 | N | 46276 | Serpin B12 OS=Homo sapiens OX=9606 GN=SERPINB12 PE=1 SV=1 |
| 207 | 701 | A0L061|LPTD\_SHESA | 43.40 | 2 | 2 | 0 | 0 | 0 | 0 | 0 | 0 | 0 | 0 | 0 | 3.21E4 |  |  |  |  |  |  |  |  |  | 1 | 1 | 1 | 0 | 0 | 0 | 0 | 0 | 0 | 0 | 0 | 0 | Y | 87406 | LPS-assembly protein LptD OS=Shewanella sp. (strain ANA-3) OX=94122 GN=lptD PE=3 SV=1 |
| 207 | 708 | Q0HS09|LPTD\_SHESR | 43.40 | 2 | 2 | 0 | 0 | 0 | 0 | 0 | 0 | 0 | 0 | 0 | 3.21E4 |  |  |  |  |  |  |  |  |  | 1 | 1 | 1 | 0 | 0 | 0 | 0 | 0 | 0 | 0 | 0 | 0 | Y | 87460 | LPS-assembly protein LptD OS=Shewanella sp. (strain MR-7) OX=60481 GN=lptD PE=3 SV=1 |
| 207 | 709 | Q0HLS9|LPTD\_SHESM | 43.40 | 2 | 2 | 0 | 0 | 0 | 0 | 0 | 0 | 0 | 0 | 0 | 3.21E4 |  |  |  |  |  |  |  |  |  | 1 | 1 | 1 | 0 | 0 | 0 | 0 | 0 | 0 | 0 | 0 | 0 | Y | 87460 | LPS-assembly protein LptD OS=Shewanella sp. (strain MR-4) OX=60480 GN=lptD PE=3 SV=1 |
| 208 | 729 | A2RT91|ANKAR\_MOUSE | 43.39 | 1 | 0 | 0 | 0 | 0 | 1 | 0 | 0 | 0 | 0 | 0 |  |  |  |  | 1.68E6 |  |  |  |  |  | 1 | 1 | 0 | 0 | 0 | 0 | 1 | 0 | 0 | 0 | 0 | 0 | N | 165289 | Ankyrin and armadillo repeat-containing protein OS=Mus musculus OX=10090 GN=Ankar PE=2 SV=1 |
| 177 | 478 | S3DQP3|GLOA\_GLAL2 | 42.98 | 0 | 0 | 0 | 0 | 0 | 0 | 0 | 0 | 0 | 0 | 0 | 3.12E4 |  |  |  |  |  |  |  |  |  | 1 | 1 | 1 | 0 | 0 | 0 | 0 | 0 | 0 | 0 | 0 | 0 | N | 796121 | Nonribosomal peptide synthetase gloA OS=Glarea lozoyensis (strain ATCC 20868 / MF5171) OX=1116229 GN=gloA PE=1 SV=1 |
| 203 | 425 | Q5RC63|PRDX2\_PONAB | 42.75 | 6 | 0 | 0 | 6 | 0 | 0 | 0 | 0 | 0 | 0 | 0 |  |  | 8.71E3 |  |  |  |  |  |  |  | 1 | 1 | 0 | 0 | 1 | 0 | 0 | 0 | 0 | 0 | 0 | 0 | N | 19430 | Peroxiredoxin-2 OS=Pongo abelii OX=9601 GN=PRDX2 PE=2 SV=3 |
| 203 | 426 | Q8K3U7|PRDX2\_CRIGR | 42.75 | 6 | 0 | 0 | 6 | 0 | 0 | 0 | 0 | 0 | 0 | 0 |  |  | 8.71E3 |  |  |  |  |  |  |  | 1 | 1 | 0 | 0 | 1 | 0 | 0 | 0 | 0 | 0 | 0 | 0 | N | 21813 | Peroxiredoxin-2 OS=Cricetulus griseus OX=10029 GN=PRDX2 PE=2 SV=3 |
| 203 | 427 | Q2PFZ3|PRDX2\_MACFA | 42.75 | 6 | 0 | 0 | 6 | 0 | 0 | 0 | 0 | 0 | 0 | 0 |  |  | 8.71E3 |  |  |  |  |  |  |  | 1 | 1 | 0 | 0 | 1 | 0 | 0 | 0 | 0 | 0 | 0 | 0 | N | 21892 | Peroxiredoxin-2 OS=Macaca fascicularis OX=9541 GN=PRDX2 PE=2 SV=3 |
| 203 | 428 | P32119|PRDX2\_HUMAN | 42.75 | 6 | 0 | 0 | 6 | 0 | 0 | 0 | 0 | 0 | 0 | 0 |  |  | 8.71E3 |  |  |  |  |  |  |  | 1 | 1 | 0 | 0 | 1 | 0 | 0 | 0 | 0 | 0 | 0 | 0 | N | 21892 | Peroxiredoxin-2 OS=Homo sapiens OX=9606 GN=PRDX2 PE=1 SV=5 |
| 203 | 552 | Q06830|PRDX1\_HUMAN | 42.75 | 6 | 0 | 0 | 6 | 0 | 0 | 0 | 0 | 0 | 0 | 0 |  |  | 8.71E3 |  |  |  |  |  |  |  | 1 | 1 | 0 | 0 | 1 | 0 | 0 | 0 | 0 | 0 | 0 | 0 | N | 22110 | Peroxiredoxin-1 OS=Homo sapiens OX=9606 GN=PRDX1 PE=1 SV=1 |
| 203 | 553 | Q6B4U9|PRDX1\_MYOLU | 42.75 | 6 | 0 | 0 | 6 | 0 | 0 | 0 | 0 | 0 | 0 | 0 |  |  | 8.71E3 |  |  |  |  |  |  |  | 1 | 1 | 0 | 0 | 1 | 0 | 0 | 0 | 0 | 0 | 0 | 0 | N | 22124 | Peroxiredoxin-1 OS=Myotis lucifugus OX=59463 GN=PRDX1 PE=1 SV=1 |
| 203 | 554 | #CONTAM#PRDX1\_HUMAN| | 42.75 | 6 | 0 | 0 | 6 | 0 | 0 | 0 | 0 | 0 | 0 | 0 |  |  | 8.71E3 |  |  |  |  |  |  |  | 1 | 1 | 0 | 0 | 1 | 0 | 0 | 0 | 0 | 0 | 0 | 0 | N | 22110 | sp|#CONTAM#PRDX1\_HUMAN| |
| 203 | 779 | Q61171|PRDX2\_MOUSE | 42.75 | 6 | 0 | 0 | 6 | 0 | 0 | 0 | 0 | 0 | 0 | 0 |  |  | 8.71E3 |  |  |  |  |  |  |  | 1 | 1 | 0 | 0 | 1 | 0 | 0 | 0 | 0 | 0 | 0 | 0 | N | 21779 | Peroxiredoxin-2 OS=Mus musculus OX=10090 GN=Prdx2 PE=1 SV=3 |
| 203 | 780 | P35704|PRDX2\_RAT | 42.75 | 6 | 0 | 0 | 6 | 0 | 0 | 0 | 0 | 0 | 0 | 0 |  |  | 8.71E3 |  |  |  |  |  |  |  | 1 | 1 | 0 | 0 | 1 | 0 | 0 | 0 | 0 | 0 | 0 | 0 | N | 21784 | Peroxiredoxin-2 OS=Rattus norvegicus OX=10116 GN=Prdx2 PE=1 SV=3 |
| 203 | 997 | Q9NL98|PRDX\_ASCSU | 42.75 | 6 | 0 | 0 | 6 | 0 | 0 | 0 | 0 | 0 | 0 | 0 |  |  | 8.71E3 |  |  |  |  |  |  |  | 1 | 1 | 0 | 0 | 1 | 0 | 0 | 0 | 0 | 0 | 0 | 0 | N | 21590 | Peroxiredoxin OS=Ascaris suum OX=6253 PE=2 SV=1 |
| 203 | 1255 | P52552|PRDX2\_PIG | 42.75 | 9 | 0 | 0 | 9 | 0 | 0 | 0 | 0 | 0 | 0 | 0 |  |  | 8.71E3 |  |  |  |  |  |  |  | 1 | 1 | 0 | 0 | 1 | 0 | 0 | 0 | 0 | 0 | 0 | 0 | N | 14198 | Peroxiredoxin-2 (Fragment) OS=Sus scrofa OX=9823 GN=PRDX2 PE=2 SV=1 |
| 203 | 1256 | Q9V3P0|PRDX1\_DROME | 42.75 | 6 | 0 | 0 | 6 | 0 | 0 | 0 | 0 | 0 | 0 | 0 |  |  | 8.71E3 |  |  |  |  |  |  |  | 1 | 1 | 0 | 0 | 1 | 0 | 0 | 0 | 0 | 0 | 0 | 0 | N | 21738 | Peroxiredoxin 1 OS=Drosophila melanogaster OX=7227 GN=Jafrac1 PE=1 SV=1 |
| 217 | 733 | P08735|G3PC1\_MAIZE | 42.42 | 4 | 0 | 0 | 4 | 0 | 0 | 0 | 0 | 0 | 0 | 0 |  |  | 7.56E3 |  |  |  |  |  |  |  | 1 | 1 | 0 | 0 | 1 | 0 | 0 | 0 | 0 | 0 | 0 | 0 | N | 36523 | Glyceraldehyde-3-phosphate dehydrogenase 1, cytosolic OS=Zea mays OX=4577 GN=GAPC1 PE=2 SV=2 |
| total 165 proteins |
| --- |

  

P15445|PA2A2\_NAJNA

back to list

  

| Protein Coverage
| Supporting Peptides
|

Protein Coverage:

Supporting Peptides:

| Peptide | Uniq | -10lgP | Mass | Length | ppm | m/z | z | RT | Fraction | Scan | Source File | Area F1 | Area F10 | Area F2 | Area F3 | Area F4 | Area F5 | Area F6 | Area F7 | Area F8 | Area F9 | #Feature | #Feature F1 | #Feature F10 | #Feature F2 | #Feature F3 | #Feature F4 | #Feature F5 | #Feature F6 | #Feature F7 | #Feature F8 | #Feature F9 | Start | End | PTM |
| --- | --- | --- | --- | --- | --- | --- | --- | --- | --- | --- | --- | --- | --- | --- | --- | --- | --- | --- | --- | --- | --- | --- | --- | --- | --- | --- | --- | --- | --- | --- | --- | --- | --- | --- | --- |
| R.LAAIC(+57.02)FAGAPYNDNNYNIDLK.A | N | 122.34 | 2356.1157 | 21 | 0.2 | 1179.0654 | 2 | 24.76 | 9 | F9:9809 | 29102019\_RID\_1313\_NaNaPb\_F8.raw |  | 4.51E6 |  |  |  | 1.14E9 | 8.96E5 |  | 3.88E8 | 2.3E5 | 38 | 0 | 2 | 0 | 0 | 0 | 22 | 2 | 0 | 10 | 2 | 95 | 115 | Carbamidomethylation |
| K.TYSYEC(+57.02)SQGTLTC(+57.02)K.G | N | 117.54 | 1696.7073 | 14 | 0.1 | 849.3610 | 2 | 11.33 | 9 | F9:2180 | 29102019\_RID\_1313\_NaNaPb\_F8.raw |  | 6.41E5 |  |  |  | 3.72E8 | 4.1E5 | 2.61E4 | 2.12E7 | 1.61E6 | 22 | 0 | 1 | 0 | 0 | 0 | 17 | 1 | 1 | 1 | 1 | 66 | 79 | Carbamidomethylation |
| R.LAAIC(+57.02)FAGAPYNDNNYNIDLKAR.C | N | 106.85 | 2583.2539 | 23 | 0.7 | 862.0925 | 3 | 17.00 | 9 | F9:5977 | 29102019\_RID\_1313\_NaNaPb\_F8.raw |  |  |  |  |  |  |  |  | 7.29E5 |  | 1 | 0 | 0 | 0 | 0 | 0 | 0 | 0 | 0 | 1 | 0 | 95 | 117 | Carbamidomethylation |
| C.FAGAPYNDNNYNIDLK.A | N | 102.86 | 1827.8428 | 16 | 0.5 | 914.9291 | 2 | 12.58 | 9 | F9:3195 | 29102019\_RID\_1313\_NaNaPb\_F8.raw |  | 9.47E4 |  |  |  | 3.82E7 |  |  | 1.3E7 | 1.77E5 | 8 | 0 | 1 | 0 | 0 | 0 | 4 | 0 | 0 | 2 | 1 | 100 | 115 |  |
| F.AGAPYNDNNYNIDLK.A | N | 99.89 | 1680.7743 | 15 | -1.6 | 841.3931 | 2 | 11.85 | 9 | F9:2622 | 29102019\_RID\_1313\_NaNaPb\_F8.raw |  | 7.76E5 |  |  |  | 2.78E8 |  |  | 9.54E7 | 1.75E6 | 14 | 0 | 1 | 0 | 0 | 0 | 7 | 0 | 0 | 5 | 1 | 101 | 115 |  |
| T.YSYEC(+57.02)SQGTLTC(+57.02)K.G | N | 95.19 | 1595.6595 | 13 | -0.2 | 798.8369 | 2 | 11.29 | 9 | F9:2138 | 29102019\_RID\_1313\_NaNaPb\_F8.raw |  |  |  |  |  |  |  |  | 2.43E6 |  | 1 | 0 | 0 | 0 | 0 | 0 | 0 | 0 | 0 | 1 | 0 | 67 | 79 | Carbamidomethylation |
| I.C(+57.02)FAGAPYNDNNYNIDLKARC(+57.02)Q | N | 94.16 | 2503.1008 | 21 | 0.2 | 835.3744 | 3 | 11.81 | 9 | F9:2615 | 29102019\_RID\_1313\_NaNaPb\_F8.raw |  |  |  |  |  |  |  |  | 1.08E6 |  | 2 | 0 | 0 | 0 | 0 | 0 | 0 | 0 | 0 | 2 | 0 | 99 | 119 | Carbamidomethylation |
| K.GDNNAC(+57.02)AASVC(+57.02)DC(+57.02)DR.L | N | 94.00 | 1683.6035 | 15 | -0.2 | 842.8089 | 2 | 10.83 | 9 | F9:1687 | 29102019\_RID\_1313\_NaNaPb\_F8.raw |  | 4.8E5 |  |  |  | 3.39E7 | 5.2E5 |  | 6.78E7 | 1.55E5 | 9 | 0 | 1 | 0 | 0 | 0 | 3 | 1 | 0 | 3 | 1 | 80 | 94 | Carbamidomethylation |
| Y.SYEC(+57.02)SQGTLTC(+57.02)K.G | N | 93.04 | 1432.5963 | 12 | 0.7 | 717.3059 | 2 | 11.00 | 9 | F9:1851 | 29102019\_RID\_1313\_NaNaPb\_F8.raw |  | 2.83E4 |  |  |  | 3.69E5 |  |  | 1.21E7 |  | 3 | 0 | 1 | 0 | 0 | 0 | 1 | 0 | 0 | 1 | 0 | 68 | 79 | Carbamidomethylation |
| R.SWWDFADYGC(+57.02)YC(+57.02)GR.G | N | 92.75 | 1841.6926 | 14 | 0.3 | 921.8539 | 2 | 35.05 | 9 | F9:13640 | 29102019\_RID\_1313\_NaNaPb\_F8.raw |  | 7.26E5 |  |  |  | 4.63E8 |  |  | 2.05E7 |  | 31 | 0 | 1 | 0 | 0 | 0 | 23 | 0 | 0 | 7 | 0 | 17 | 30 | Carbamidomethylation |
| R.C(+57.02)C(+57.02)QVHDNC(+57.02)YNEAEK.I | N | 91.84 | 1825.6818 | 14 | 0.0 | 609.5679 | 3 | 10.67 | 9 | F9:1581 | 29102019\_RID\_1313\_NaNaPb\_F8.raw |  |  |  |  |  | 5.99E7 | 0 |  | 7.85E6 |  | 6 | 0 | 0 | 0 | 0 | 0 | 3 | 1 | 0 | 2 | 0 | 43 | 56 | Carbamidomethylation |
| A.AIC(+57.02)FAGAPYNDNNYNIDLKARC(+57.02)Q | N | 85.99 | 2687.2219 | 23 | 0.8 | 896.7487 | 3 | 12.51 | 9 | F9:3154 | 29102019\_RID\_1313\_NaNaPb\_F8.raw |  |  |  |  |  |  |  |  | 5.59E5 |  | 1 | 0 | 0 | 0 | 0 | 0 | 0 | 0 | 0 | 1 | 0 | 97 | 119 | Carbamidomethylation |
| K.TYSYEC(+57.02)SQGTLTC(+57.02)KGDNNAC(+57.02)AASVC(+57.02)DC(+57.02)DR.L | Y | 83.26 | 3362.3003 | 29 | -0.8 | 1121.7732 | 3 | 11.44 | 6 | F6:2259 | 29102019\_RID\_1313\_NaNaPb\_F5.raw |  |  |  |  |  | 2.35E6 |  |  | 6.22E4 |  | 2 | 0 | 0 | 0 | 0 | 0 | 1 | 0 | 0 | 1 | 0 | 66 | 94 | Carbamidomethylation |
| A.IC(+57.02)FAGAPYNDNNYNIDLKARC(+57.02)Q | N | 82.66 | 2616.1848 | 22 | 1.1 | 873.0698 | 3 | 12.27 | 9 | F9:2982 | 29102019\_RID\_1313\_NaNaPb\_F8.raw |  |  |  |  |  |  |  |  | 2.77E5 |  | 1 | 0 | 0 | 0 | 0 | 0 | 0 | 0 | 0 | 1 | 0 | 98 | 119 | Carbamidomethylation |
| C.FAGAPYNDNNYNIDLKARC(+57.02)Q | N | 81.68 | 2343.0701 | 20 | 0.5 | 782.0310 | 3 | 11.75 | 9 | F9:2555 | 29102019\_RID\_1313\_NaNaPb\_F8.raw |  |  |  |  |  |  |  |  | 1.05E6 |  | 2 | 0 | 0 | 0 | 0 | 0 | 0 | 0 | 0 | 2 | 0 | 100 | 119 | Carbamidomethylation |
| G.DNNAC(+57.02)AASVC(+57.02)DC(+57.02)DR.L | N | 81.00 | 1626.5820 | 14 | 0.4 | 814.2986 | 2 | 10.83 | 9 | F9:1737 | 29102019\_RID\_1313\_NaNaPb\_F8.raw |  | 8.45E4 |  |  |  | 4.22E6 | 2.83E4 |  | 2.85E6 | 3.58E4 | 5 | 0 | 1 | 0 | 0 | 0 | 1 | 1 | 0 | 1 | 1 | 81 | 94 | Carbamidomethylation |
| R.GGSGTPVDDLDR.C | N | 79.46 | 1187.5417 | 12 | -1.4 | 594.7773 | 2 | 11.42 | 7 | F7:2302 | 29102019\_RID\_1313\_NaNaPb\_F6.raw |  | 3.83E5 |  |  |  | 9.47E5 | 2.08E5 |  |  | 7.26E5 | 10 | 0 | 1 | 0 | 0 | 0 | 7 | 1 | 0 | 0 | 1 | 31 | 42 |  |
| D.NNAC(+57.02)AASVC(+57.02)DC(+57.02)DR.L | N | 77.88 | 1511.5552 | 13 | 0.4 | 756.7852 | 2 | 10.83 | 6 | F6:1688 | 29102019\_RID\_1313\_NaNaPb\_F5.raw |  |  |  |  |  | 8.09E4 |  |  | 3.39E4 |  | 2 | 0 | 0 | 0 | 0 | 0 | 1 | 0 | 0 | 1 | 0 | 82 | 94 | Carbamidomethylation |
| K.TYSYEC(+57.02)SQGTLTC(+57.02)KG.D | N | 76.57 | 1753.7288 | 15 | 0.9 | 877.8724 | 2 | 11.37 | 9 | F9:2181 | 29102019\_RID\_1313\_NaNaPb\_F8.raw |  |  |  |  |  |  |  |  | 6.41E6 |  | 1 | 0 | 0 | 0 | 0 | 0 | 0 | 0 | 0 | 1 | 0 | 66 | 80 | Carbamidomethylation |
| D.RLAAIC(+57.02)FAGAPYNDNNYNIDLKARC(+57.02)Q | N | 75.67 | 3027.4443 | 26 | 0.9 | 757.8690 | 4 | 12.31 | 9 | F9:3010 | 29102019\_RID\_1313\_NaNaPb\_F8.raw |  |  |  |  |  |  |  |  | 2.97E5 |  | 1 | 0 | 0 | 0 | 0 | 0 | 0 | 0 | 0 | 1 | 0 | 94 | 119 | Carbamidomethylation |
| K.ISGC(+57.02)WPYFK.T | N | 73.47 | 1156.5375 | 9 | 0.7 | 579.2764 | 2 | 16.15 | 6 | F6:5636 | 29102019\_RID\_1313\_NaNaPb\_F5.raw |  | 4.91E5 |  |  |  | 7.32E8 |  |  | 9.7E6 | 1.42E6 | 16 | 0 | 1 | 0 | 0 | 0 | 11 | 0 | 0 | 3 | 1 | 57 | 65 | Carbamidomethylation |
| D.RLAAIC(+57.02)FAGAPYNDNNYNIDLK.A | N | 72.38 | 2512.2168 | 22 | 0.2 | 838.4130 | 3 | 17.30 | 6 | F6:6122 | 29102019\_RID\_1313\_NaNaPb\_F5.raw |  |  |  |  |  | 7.25E5 |  |  |  |  | 1 | 0 | 0 | 0 | 0 | 0 | 1 | 0 | 0 | 0 | 0 | 94 | 115 | Carbamidomethylation |
| A.GAPYNDNNYNIDLK.A | N | 71.35 | 1609.7372 | 14 | 0.1 | 805.8760 | 2 | 11.93 | 6 | F6:2692 | 29102019\_RID\_1313\_NaNaPb\_F5.raw |  |  |  |  |  | 1.61E6 |  |  |  |  | 1 | 0 | 0 | 0 | 0 | 0 | 1 | 0 | 0 | 0 | 0 | 102 | 115 |  |
| F.AGAPYNDNNYNIDLKARC(+57.02)Q | N | 70.05 | 2196.0017 | 19 | 0.5 | 733.0082 | 3 | 11.51 | 9 | F9:2340 | 29102019\_RID\_1313\_NaNaPb\_F8.raw |  |  |  |  |  |  |  |  | 7.33E5 |  | 1 | 0 | 0 | 0 | 0 | 0 | 0 | 0 | 0 | 1 | 0 | 101 | 119 | Carbamidomethylation |
| A.IC(+57.02)FAGAPYNDNNYNIDLKAR.C | N | 69.77 | 2328.0957 | 20 | 1.0 | 777.0399 | 3 | 12.51 | 9 | F9:3176 | 29102019\_RID\_1313\_NaNaPb\_F8.raw |  |  |  |  |  |  |  |  | 8.95E4 |  | 1 | 0 | 0 | 0 | 0 | 0 | 0 | 0 | 0 | 1 | 0 | 98 | 117 | Carbamidomethylation |
| S.YEC(+57.02)SQGTLTC(+57.02)K.G | N | 69.49 | 1345.5642 | 11 | 0.0 | 673.7894 | 2 | 10.91 | 9 | F9:1786 | 29102019\_RID\_1313\_NaNaPb\_F8.raw |  |  |  |  |  |  |  |  | 1.8E5 |  | 1 | 0 | 0 | 0 | 0 | 0 | 0 | 0 | 0 | 1 | 0 | 69 | 79 | Carbamidomethylation |
| G.APYNDNNYNIDLK.A | N | 68.55 | 1552.7157 | 13 | 0.7 | 777.3657 | 2 | 11.91 | 6 | F6:2696 | 29102019\_RID\_1313\_NaNaPb\_F5.raw |  |  |  |  |  | 3.08E5 |  |  |  |  | 1 | 0 | 0 | 0 | 0 | 0 | 1 | 0 | 0 | 0 | 0 | 103 | 115 |  |
| Y.NDNNYNIDLK.A | N | 67.89 | 1221.5625 | 10 | 1.0 | 611.7891 | 2 | 11.68 | 6 | F6:2444 | 29102019\_RID\_1313\_NaNaPb\_F5.raw |  |  |  |  |  | 3.26E6 |  |  | 1.93E6 |  | 2 | 0 | 0 | 0 | 0 | 0 | 1 | 0 | 0 | 1 | 0 | 106 | 115 |  |
| A.IC(+57.02)FAGAPYNDNNYNIDLK.A | N | 66.89 | 2100.9575 | 18 | 0.0 | 701.3264 | 3 | 17.93 | 6 | F6:6564 | 29102019\_RID\_1313\_NaNaPb\_F5.raw |  |  |  |  |  | 1.53E5 |  |  | 7.23E6 |  | 4 | 0 | 0 | 0 | 0 | 0 | 2 | 0 | 0 | 2 | 0 | 98 | 115 | Carbamidomethylation |
| K.ISGC(+57.02)WPYFKTYSYEC(+57.02)SQGTLTC(+57.02)KGDNNAC(+57.02)AASVC(+57.02)DC(+57.02)DR.L | Y | 66.65 | 4500.8271 | 38 | 1.1 | 1126.2153 | 4 | 12.54 | 6 | F6:3192 | 29102019\_RID\_1313\_NaNaPb\_F5.raw |  |  |  |  |  | 3.01E5 |  |  |  |  | 1 | 0 | 0 | 0 | 0 | 0 | 1 | 0 | 0 | 0 | 0 | 57 | 94 | Carbamidomethylation |
| K.C(+57.02)TVPSRSWWDFADYGC(+57.02)YC(+57.02)GR.G | N | 64.99 | 2542.0251 | 20 | 2.2 | 848.3508 | 3 | 23.52 | 6 | F6:9372 | 29102019\_RID\_1313\_NaNaPb\_F5.raw |  |  |  |  |  | 3.36E5 |  |  |  |  | 1 | 0 | 0 | 0 | 0 | 0 | 1 | 0 | 0 | 0 | 0 | 11 | 30 | Carbamidomethylation |
| Y.NDNNYNIDLKARC(+57.02)Q | N | 62.36 | 1736.7900 | 14 | -0.7 | 579.9369 | 3 | 11.26 | 9 | F9:2124 | 29102019\_RID\_1313\_NaNaPb\_F8.raw |  |  |  |  |  |  |  |  | 2.67E5 |  | 1 | 0 | 0 | 0 | 0 | 0 | 0 | 0 | 0 | 1 | 0 | 106 | 119 | Carbamidomethylation |
| R.SWWDFADYGC(+57.02)YC(+57.02)GRGGSGTPVDDLDR.C | N | 62.27 | 3011.2239 | 26 | 0.1 | 1004.7487 | 3 | 30.80 | 9 | F9:12192 | 29102019\_RID\_1313\_NaNaPb\_F8.raw |  |  |  |  |  | 1.56E5 |  |  | 2.61E4 |  | 2 | 0 | 0 | 0 | 0 | 0 | 1 | 0 | 0 | 1 | 0 | 17 | 42 | Carbamidomethylation |
| W.DFADYGC(+57.02)YC(+57.02)GR.G | N | 59.28 | 1382.5020 | 11 | -0.8 | 692.2577 | 2 | 11.98 | 6 | F6:2741 | 29102019\_RID\_1313\_NaNaPb\_F5.raw |  |  |  |  |  | 6.86E5 |  |  |  |  | 1 | 0 | 0 | 0 | 0 | 0 | 1 | 0 | 0 | 0 | 0 | 20 | 30 | Carbamidomethylation |
| R.LAAIC(+57.02)FAGAPYNDNNYNIDLKARC(+57.02)Q | N | 58.75 | 2871.3433 | 25 | 0.6 | 958.1223 | 3 | 16.04 | 9 | F9:5714 | 29102019\_RID\_1313\_NaNaPb\_F8.raw |  |  |  |  |  |  |  |  | 1.54E6 |  | 1 | 0 | 0 | 0 | 0 | 0 | 0 | 0 | 0 | 1 | 0 | 95 | 119 | Carbamidomethylation |
| R.LAAIC(+57.02)FAGAPYN.D | N | 58.35 | 1266.6067 | 12 | 0.1 | 634.3107 | 2 | 24.51 | 6 | F6:9784 | 29102019\_RID\_1313\_NaNaPb\_F5.raw |  |  |  |  |  | 2.29E6 |  |  | 1.49E5 |  | 3 | 0 | 0 | 0 | 0 | 0 | 2 | 0 | 0 | 1 | 0 | 95 | 106 | Carbamidomethylation |
| R.SWWDFADYGC(+57.02)YC(+57.02)GRG.G | N | 56.62 | 1898.7141 | 15 | 0.4 | 950.3647 | 2 | 33.02 | 9 | F9:12959 | 29102019\_RID\_1313\_NaNaPb\_F8.raw |  |  |  |  |  | 2.73E7 |  |  | 3.48E5 |  | 6 | 0 | 0 | 0 | 0 | 0 | 4 | 0 | 0 | 2 | 0 | 17 | 31 | Carbamidomethylation |
| K.TYSYEC(+57.02)SQGTL.T | N | 56.52 | 1307.5339 | 11 | 0.9 | 654.7748 | 2 | 12.20 | 6 | F6:2929 | 29102019\_RID\_1313\_NaNaPb\_F5.raw |  |  |  |  |  | 8.99E5 |  |  |  |  | 1 | 0 | 0 | 0 | 0 | 0 | 1 | 0 | 0 | 0 | 0 | 66 | 76 | Carbamidomethylation |
| D.FADYGC(+57.02)YC(+57.02)GR.G | N | 54.59 | 1267.4750 | 10 | 0.4 | 634.7450 | 2 | 11.57 | 6 | F6:2343 | 29102019\_RID\_1313\_NaNaPb\_F5.raw |  |  |  |  |  | 4.49E5 |  |  |  |  | 1 | 0 | 0 | 0 | 0 | 0 | 1 | 0 | 0 | 0 | 0 | 21 | 30 | Carbamidomethylation |
| I.C(+57.02)FAGAPYNDNNYNIDLK.A | N | 54.20 | 1987.8734 | 17 | 0.6 | 994.9446 | 2 | 13.27 | 9 | F9:3677 | 29102019\_RID\_1313\_NaNaPb\_F8.raw |  |  |  |  |  |  |  |  | 2.25E5 |  | 1 | 0 | 0 | 0 | 0 | 0 | 0 | 0 | 0 | 1 | 0 | 99 | 115 | Carbamidomethylation |
| R.SWWDFADYGC(+57.02)Y.C | N | 53.68 | 1468.5394 | 11 | 0.0 | 735.2770 | 2 | 55.23 | 6 | F6:21957 | 29102019\_RID\_1313\_NaNaPb\_F5.raw |  |  |  |  |  | 2.22E7 |  |  |  |  | 1 | 0 | 0 | 0 | 0 | 0 | 1 | 0 | 0 | 0 | 0 | 17 | 27 | Carbamidomethylation |
| N.DNNYNIDLK.A | N | 51.97 | 1107.5197 | 9 | -1.6 | 554.7662 | 2 | 11.86 | 6 | F6:2632 | 29102019\_RID\_1313\_NaNaPb\_F5.raw |  |  |  |  |  | 2.04E5 |  |  | 3.21E4 |  | 2 | 0 | 0 | 0 | 0 | 0 | 1 | 0 | 0 | 1 | 0 | 107 | 115 |  |
| C.DC(+57.02)DRLAAIC(+57.02)FAGAPYNDNNYNIDLKAR.C | N | 51.86 | 3129.4397 | 27 | 0.8 | 783.3678 | 4 | 16.75 | 9 | F9:5867 | 29102019\_RID\_1313\_NaNaPb\_F8.raw |  |  |  |  |  |  |  |  | 8.64E4 |  | 1 | 0 | 0 | 0 | 0 | 0 | 0 | 0 | 0 | 1 | 0 | 91 | 117 | Carbamidomethylation |
| R.C(+57.02)C(+57.02)QVHDNC(+57.02)YNEAEKISGC(+57.02)WPYFK.T | N | 48.97 | 2964.2087 | 23 | 0.0 | 742.0594 | 4 | 12.43 | 6 | F6:3102 | 29102019\_RID\_1313\_NaNaPb\_F5.raw |  |  |  |  |  | 3.95E5 |  |  |  |  | 1 | 0 | 0 | 0 | 0 | 0 | 1 | 0 | 0 | 0 | 0 | 43 | 65 | Carbamidomethylation |
| NLYQFKNMIKC(+57.02)TVPSRSWWDFADYGC(+57.02)YC(+57.02)GR.G | Y | 48.93 | 3821.7000 | 30 | -8.4 | 956.4242 | 4 | 35.64 | 9 | F9:13738 | 29102019\_RID\_1313\_NaNaPb\_F8.raw |  |  |  |  |  |  |  |  | 1.71E5 |  | 1 | 0 | 0 | 0 | 0 | 0 | 0 | 0 | 0 | 1 | 0 | 1 | 30 | Carbamidomethylation |
| R.LAAIC(+57.02)FAGAPY.N | N | 48.00 | 1152.5637 | 11 | -0.1 | 577.2891 | 2 | 29.05 | 9 | F9:11682 | 29102019\_RID\_1313\_NaNaPb\_F8.raw |  |  |  |  |  |  |  |  | 1.7E6 |  | 1 | 0 | 0 | 0 | 0 | 0 | 0 | 0 | 0 | 1 | 0 | 95 | 105 | Carbamidomethylation |
| G.SGTPVDDLDR.C | N | 47.92 | 1073.4989 | 10 | -2.3 | 537.7555 | 2 | 11.45 | 6 | F6:2250 | 29102019\_RID\_1313\_NaNaPb\_F5.raw |  |  |  |  |  | 2.06E5 |  |  |  |  | 1 | 0 | 0 | 0 | 0 | 0 | 1 | 0 | 0 | 0 | 0 | 33 | 42 |  |
| P.YNDNNYNIDLK.A | N | 47.70 | 1384.6259 | 11 | -1.6 | 693.3191 | 2 | 11.80 | 6 | F6:2577 | 29102019\_RID\_1313\_NaNaPb\_F5.raw |  |  |  |  |  | 1.37E5 |  |  |  |  | 1 | 0 | 0 | 0 | 0 | 0 | 1 | 0 | 0 | 0 | 0 | 105 | 115 |  |
| S.GTPVDDLDR.C | N | 47.24 | 986.4669 | 9 | 0.0 | 494.2407 | 2 | 11.37 | 9 | F9:2225 | 29102019\_RID\_1313\_NaNaPb\_F8.raw |  |  |  |  |  |  |  |  | 5.17E4 |  | 1 | 0 | 0 | 0 | 0 | 0 | 0 | 0 | 0 | 1 | 0 | 34 | 42 |  |
| W.WDFADYGC(+57.02)YC(+57.02)GR.G | N | 46.98 | 1568.5813 | 12 | 0.7 | 785.2985 | 2 | 17.36 | 6 | F6:6148 | 29102019\_RID\_1313\_NaNaPb\_F5.raw |  |  |  |  |  | 3.91E5 |  |  |  |  | 1 | 0 | 0 | 0 | 0 | 0 | 1 | 0 | 0 | 0 | 0 | 19 | 30 | Carbamidomethylation |
| A.AIC(+57.02)FAGAPYNDNNYNIDLKAR.C | N | 46.86 | 2399.1328 | 21 | 1.0 | 800.7190 | 3 | 13.04 | 9 | F9:3508 | 29102019\_RID\_1313\_NaNaPb\_F8.raw |  |  |  |  |  |  |  |  | 2.26E5 |  | 1 | 0 | 0 | 0 | 0 | 0 | 0 | 0 | 0 | 1 | 0 | 97 | 117 | Carbamidomethylation |
| D.C(+57.02)DRLAAIC(+57.02)FAGAPYNDNNYNIDLKARC(+57.02)Q | N | 46.30 | 3302.5020 | 28 | 0.5 | 826.6332 | 4 | 13.71 | 9 | F9:3965 | 29102019\_RID\_1313\_NaNaPb\_F8.raw |  |  |  |  |  |  |  |  | 4.11E5 |  | 1 | 0 | 0 | 0 | 0 | 0 | 0 | 0 | 0 | 1 | 0 | 92 | 119 | Carbamidomethylation |
| K.TYSYEC(+57.02)SQGTLT.C | N | 46.12 | 1408.5817 | 12 | -1.9 | 705.2968 | 2 | 11.97 | 6 | F6:2736 | 29102019\_RID\_1313\_NaNaPb\_F5.raw |  |  |  |  |  | 2.41E5 |  |  |  |  | 1 | 0 | 0 | 0 | 0 | 0 | 1 | 0 | 0 | 0 | 0 | 66 | 77 | Carbamidomethylation |
| L.AAIC(+57.02)FAGAPYNDNNYNIDLKAR.C | N | 42.40 | 2470.1699 | 22 | 1.3 | 824.3983 | 3 | 13.65 | 9 | F9:3964 | 29102019\_RID\_1313\_NaNaPb\_F8.raw |  |  |  |  |  |  |  |  | 1.29E5 |  | 1 | 0 | 0 | 0 | 0 | 0 | 0 | 0 | 0 | 1 | 0 | 96 | 117 | Carbamidomethylation |
| total 54 peptides |
| --- |

P35527|K1C9\_HUMAN

back to list

  

| Protein Coverage
| Supporting Peptides
|

Protein Coverage:

Supporting Peptides:

| Peptide | Uniq | -10lgP | Mass | Length | ppm | m/z | z | RT | Fraction | Scan | Source File | Area F1 | Area F10 | Area F2 | Area F3 | Area F4 | Area F5 | Area F6 | Area F7 | Area F8 | Area F9 | #Feature | #Feature F1 | #Feature F10 | #Feature F2 | #Feature F3 | #Feature F4 | #Feature F5 | #Feature F6 | #Feature F7 | #Feature F8 | #Feature F9 | Start | End | PTM |
| --- | --- | --- | --- | --- | --- | --- | --- | --- | --- | --- | --- | --- | --- | --- | --- | --- | --- | --- | --- | --- | --- | --- | --- | --- | --- | --- | --- | --- | --- | --- | --- | --- | --- | --- | --- |
| Y.SYGGGSGGGFSASSLGGGFGGGSR.G | Y | 125.95 | 2021.8827 | 24 | 0.5 | 1011.9491 | 2 | 12.45 | 6 | F6:3108 | 29102019\_RID\_1313\_NaNaPb\_F5.raw | 6.19E5 | 3.89E5 | 8.39E5 |  |  | 1.11E6 | 7.73E4 |  |  | 6.2E4 | 7 | 1 | 1 | 2 | 0 | 0 | 1 | 1 | 0 | 0 | 1 | 72 | 95 |  |
| K.EIETYHNLLEGGQEDFESSGAGK.I | Y | 118.18 | 2509.1245 | 23 | 0.3 | 837.3823 | 3 | 12.54 | 6 | F6:3188 | 29102019\_RID\_1313\_NaNaPb\_F5.raw | 1.6E6 | 1.01E6 | 2.5E6 |  |  | 3.09E6 | 0 |  |  | 1.6E5 | 9 | 1 | 2 | 2 | 0 | 0 | 2 | 1 | 0 | 0 | 1 | 450 | 472 |  |
| K.VQALEEANNDLENK.I | Y | 113.40 | 1585.7583 | 14 | 1.1 | 793.8873 | 2 | 11.73 | 1 | F1:3242 | 29102019\_RID\_1313\_NaNaPb\_F1.raw | 7.52E5 | 7.67E5 | 2.51E6 |  |  | 1.61E6 | 7.85E4 |  |  | 1.9E5 | 8 | 2 | 1 | 2 | 0 | 0 | 1 | 1 | 0 | 0 | 1 | 171 | 184 |  |
| R.GGSGGSHGGGSGFGGESGGSYGGGEEASGSGGGYGGGSGK.S | Y | 106.12 | 3222.2742 | 40 | 1.1 | 1075.0999 | 3 | 11.11 | 6 | F6:1935 | 29102019\_RID\_1313\_NaNaPb\_F5.raw |  | 1.15E5 | 1.08E5 |  | 6.79E4 | 2.17E5 |  |  | 0 |  | 5 | 0 | 1 | 1 | 0 | 1 | 1 | 0 | 0 | 1 | 0 | 580 | 619 |  |
| R.SGGGGGGGLGSGGSIR.S | Y | 104.19 | 1231.5905 | 16 | 1.0 | 616.8031 | 2 | 11.01 | 7 | F7:1879 | 29102019\_RID\_1313\_NaNaPb\_F6.raw | 4.66E4 |  | 3.66E5 |  | 5.44E5 |  | 8.31E4 | 2.23E3 | 2.41E4 |  | 6 | 1 | 0 | 1 | 0 | 1 | 0 | 1 | 1 | 1 | 0 | 14 | 29 |  |
| K.DIENQYETQITQIEHEVSSSGQEVQSSAK.E | Y | 93.73 | 3263.5066 | 29 | 0.5 | 1088.8434 | 3 | 27.80 | 7 | F7:9913 | 29102019\_RID\_1313\_NaNaPb\_F6.raw |  | 1.54E6 |  |  |  | 2.76E6 | 1.19E6 |  |  |  | 5 | 0 | 2 | 0 | 0 | 0 | 2 | 1 | 0 | 0 | 0 | 340 | 368 |  |
| R.GGGGSFGYSYGGGSGGGFSASSLGGGFGGGSR.G | Y | 92.09 | 2704.1538 | 32 | 1.0 | 902.3928 | 3 | 17.71 | 7 | F7:6108 | 29102019\_RID\_1313\_NaNaPb\_F6.raw |  | 2E5 |  |  |  |  | 8.77E5 |  |  |  | 4 | 0 | 2 | 0 | 0 | 0 | 0 | 2 | 0 | 0 | 0 | 64 | 95 |  |
| R.GGSGGSYGGGGSGGGYGGGSGSR.G | Y | 90.91 | 1790.7203 | 23 | 0.5 | 896.3679 | 2 | 10.84 | 3 | F3:1717 | 29102019\_RID\_1313\_NaNaPb\_F2.raw |  |  | 3.9E5 |  |  |  | 3.12E5 |  |  |  | 3 | 0 | 0 | 2 | 0 | 0 | 0 | 1 | 0 | 0 | 0 | 491 | 513 |  |
| R.QGVDADINGLR.Q | Y | 84.77 | 1156.5836 | 11 | 0.5 | 579.2994 | 2 | 11.72 | 7 | F7:2553 | 29102019\_RID\_1313\_NaNaPb\_F6.raw | 4.81E5 | 1.97E5 | 1.74E6 |  |  | 8.74E5 | 1.39E5 |  |  |  | 5 | 1 | 1 | 1 | 0 | 0 | 1 | 1 | 0 | 0 | 0 | 251 | 261 |  |
| R.FSSSSGYGGGSSR.V | Y | 83.64 | 1234.5214 | 13 | -0.2 | 618.2678 | 2 | 10.74 | 7 | F7:1620 | 29102019\_RID\_1313\_NaNaPb\_F6.raw | 8.29E4 |  | 4.24E5 |  |  |  | 2.33E5 |  |  |  | 3 | 1 | 0 | 1 | 0 | 0 | 0 | 1 | 0 | 0 | 0 | 47 | 59 |  |
| F.GGFGGFGGGAGGGDGGILTANEK.S | Y | 81.22 | 1951.9023 | 23 | 1.4 | 976.9598 | 2 | 16.09 | 6 | F6:5501 | 29102019\_RID\_1313\_NaNaPb\_F5.raw |  |  | 3.24E4 |  |  | 0 |  |  |  |  | 2 | 0 | 0 | 1 | 0 | 0 | 1 | 0 | 0 | 0 | 0 | 132 | 154 |  |
| K.NYSPYYNTIDDLK.D | Y | 79.68 | 1604.7358 | 13 | 0.8 | 803.3759 | 2 | 13.13 | 6 | F6:3676 | 29102019\_RID\_1313\_NaNaPb\_F5.raw |  | 3.57E5 |  |  |  | 1.18E6 |  |  |  | 6.66E4 | 3 | 0 | 1 | 0 | 0 | 0 | 1 | 0 | 0 | 0 | 1 | 200 | 212 |  |
| K.STMQELNSR.L | Y | 78.66 | 1064.4921 | 9 | -0.3 | 533.2532 | 2 | 11.03 | 7 | F7:1906 | 29102019\_RID\_1313\_NaNaPb\_F6.raw |  |  |  |  |  |  | 3.91E4 |  |  |  | 1 | 0 | 0 | 0 | 0 | 0 | 0 | 1 | 0 | 0 | 0 | 155 | 163 |  |
| Y.YNTIDDLKDQIVDLTVGNNK.T | Y | 77.78 | 2277.1489 | 20 | 0.8 | 760.0575 | 3 | 29.15 | 2 | F2:10013 | 29102019\_RID\_1313\_NaNaPb\_F10.raw |  | 3.67E5 |  |  |  |  | 1.57E5 |  |  |  | 2 | 0 | 1 | 0 | 0 | 0 | 0 | 1 | 0 | 0 | 0 | 205 | 224 |  |
| F.GGFGGGAGGGDGGILTANEK.S | Y | 77.11 | 1690.7910 | 20 | 0.3 | 846.4030 | 2 | 12.26 | 1 | F1:3764 | 29102019\_RID\_1313\_NaNaPb\_F1.raw | 1.98E5 | 1.84E5 | 3.46E5 |  |  | 0 |  |  |  |  | 4 | 1 | 1 | 1 | 0 | 0 | 1 | 0 | 0 | 0 | 0 | 135 | 154 |  |
| N.TIDDLKDQIVDLTVGNNK.T | Y | 76.90 | 2000.0426 | 18 | 0.5 | 667.6885 | 3 | 25.50 | 1 | F1:7573 | 29102019\_RID\_1313\_NaNaPb\_F1.raw | 8.72E4 |  | 1.48E5 |  |  |  |  |  |  |  | 2 | 1 | 0 | 1 | 0 | 0 | 0 | 0 | 0 | 0 | 0 | 207 | 224 |  |
| R.QEYEQLIAK.N | Y | 76.46 | 1120.5764 | 9 | 0.4 | 561.2957 | 2 | 11.95 | 1 | F1:3452 | 29102019\_RID\_1313\_NaNaPb\_F1.raw | 5.06E5 | 4.66E5 | 1.66E6 |  |  | 8.76E5 | 1.56E5 |  | 0 | 6.37E4 | 7 | 1 | 1 | 1 | 0 | 0 | 1 | 1 | 0 | 1 | 1 | 328 | 336 |  |
| R.HGVQELEIELQSQLSK.K | Y | 76.03 | 1836.9581 | 16 | -0.3 | 613.3265 | 3 | 21.21 | 1 | F1:6455 | 29102019\_RID\_1313\_NaNaPb\_F1.raw | 9.49E5 | 1.1E6 | 1.46E6 |  |  | 9.39E5 | 4.5E5 |  | 8.53E4 | 3.54E3 | 14 | 3 | 3 | 3 | 0 | 0 | 1 | 2 | 0 | 1 | 1 | 375 | 390 |  |
| K.DQIVDLTVGNNK.T | Y | 75.34 | 1314.6780 | 12 | 0.7 | 658.3467 | 2 | 13.41 | 1 | F1:4326 | 29102019\_RID\_1313\_NaNaPb\_F1.raw | 3.07E5 | 2.37E5 | 7.29E5 |  |  | 7.06E5 |  |  |  | 3.62E4 | 5 | 1 | 1 | 1 | 0 | 0 | 1 | 0 | 0 | 0 | 1 | 213 | 224 |  |
| K.NYSPYYNTIDDLKDQIVDLTVGNNK.T | Y | 73.12 | 2901.4031 | 25 | 0.2 | 968.1418 | 3 | 40.01 | 7 | F7:13630 | 29102019\_RID\_1313\_NaNaPb\_F6.raw |  | 1.13E6 |  |  |  | 2.7E6 | 1.26E6 |  | 1.67E5 |  | 7 | 0 | 2 | 0 | 0 | 0 | 2 | 2 | 0 | 1 | 0 | 200 | 224 |  |
| R.QEIEC(+57.02)QNQEYSLLLSIK.M | Y | 72.95 | 2094.0303 | 17 | 0.5 | 1048.0229 | 2 | 29.01 | 1 | F1:8484 | 29102019\_RID\_1313\_NaNaPb\_F1.raw | 2.44E5 | 1.27E6 | 1.26E5 |  |  | 3.63E6 | 6.91E4 |  |  |  | 8 | 2 | 2 | 1 | 0 | 0 | 2 | 1 | 0 | 0 | 0 | 428 | 444 | Carbamidomethylation |
| N.LLEGGQEDFESSGAGK.I | Y | 67.69 | 1622.7423 | 16 | 1.3 | 812.3795 | 2 | 11.79 | 3 | F3:2595 | 29102019\_RID\_1313\_NaNaPb\_F2.raw |  |  | 5.97E4 |  |  |  |  |  |  |  | 1 | 0 | 0 | 1 | 0 | 0 | 0 | 0 | 0 | 0 | 0 | 457 | 472 |  |
| R.QFSSSYLSR.S | Y | 65.94 | 1073.5142 | 9 | 0.4 | 537.7646 | 2 | 11.87 | 1 | F1:3368 | 29102019\_RID\_1313\_NaNaPb\_F1.raw | 0 |  | 2.94E5 |  |  | 1.09E5 |  |  |  |  | 3 | 1 | 0 | 1 | 0 | 0 | 1 | 0 | 0 | 0 | 0 | 5 | 13 |  |
| K.TLLDIDNTR.M | Y | 64.63 | 1059.5560 | 9 | -0.1 | 530.7852 | 2 | 12.27 | 7 | F7:3028 | 29102019\_RID\_1313\_NaNaPb\_F6.raw | 1.37E6 | 8.35E5 | 3.8E6 |  |  | 2E6 | 5.76E5 |  | 1.17E5 |  | 8 | 2 | 1 | 2 | 0 | 0 | 1 | 1 | 0 | 1 | 0 | 225 | 233 |  |
| R.QVLDNLTM(+15.99)EK.S | Y | 62.58 | 1205.5962 | 10 | 0.8 | 603.8058 | 2 | 11.95 | 1 | F1:3453 | 29102019\_RID\_1313\_NaNaPb\_F1.raw | 8.54E4 | 0 | 2.33E5 |  |  | 1.58E5 |  |  |  |  | 4 | 1 | 1 | 1 | 0 | 0 | 1 | 0 | 0 | 0 | 0 | 262 | 271 | Oxidation (M) |
| K.EEM(+15.99)SQLTGQNSGDVNVEINVAPGK.D | Y | 61.17 | 2531.1809 | 24 | 0.3 | 1266.5981 | 2 | 12.12 | 6 | F6:2875 | 29102019\_RID\_1313\_NaNaPb\_F5.raw |  |  |  |  |  | 4.57E4 |  |  |  |  | 1 | 0 | 0 | 0 | 0 | 0 | 1 | 0 | 0 | 0 | 0 | 294 | 317 | Oxidation (M) |
| S.SLGGGFGGGSR.G | Y | 60.25 | 950.4570 | 11 | 1.1 | 476.2363 | 2 | 11.52 | 3 | F3:2332 | 29102019\_RID\_1313\_NaNaPb\_F2.raw |  |  | 1.59E5 |  |  |  |  |  |  |  | 1 | 0 | 0 | 1 | 0 | 0 | 0 | 0 | 0 | 0 | 0 | 85 | 95 |  |
| H.NLLEGGQEDFESSGAGK.I | Y | 59.87 | 1736.7853 | 17 | 1.1 | 869.4009 | 2 | 12.33 | 3 | F3:3078 | 29102019\_RID\_1313\_NaNaPb\_F2.raw |  |  | 9.5E4 |  |  |  |  |  |  |  | 1 | 0 | 0 | 1 | 0 | 0 | 0 | 0 | 0 | 0 | 0 | 456 | 472 |  |
| Q.ISNLEAQITDVR.Q | Y | 59.82 | 1357.7201 | 12 | 0.2 | 679.8674 | 2 | 12.18 | 2 | F2:3080 | 29102019\_RID\_1313\_NaNaPb\_F10.raw | 1.43E5 | 7.68E4 | 2.85E5 |  |  |  |  |  |  |  | 3 | 1 | 1 | 1 | 0 | 0 | 0 | 0 | 0 | 0 | 0 | 416 | 427 |  |
| R.YC(+57.02)GQLQM(+15.99)IQEQISNLEAQITDVR.Q | Y | 59.60 | 2752.3159 | 23 | 0.8 | 918.4467 | 3 | 52.03 | 6 | F6:20887 | 29102019\_RID\_1313\_NaNaPb\_F5.raw |  | 7.54E4 |  |  |  | 3.62E5 |  |  |  |  | 3 | 0 | 1 | 0 | 0 | 0 | 2 | 0 | 0 | 0 | 0 | 405 | 427 | Carbamidomethylation; Oxidation (M) |
| F.SASSLGGGFGGGSR.G | Y | 59.47 | 1195.5581 | 14 | 0.5 | 598.7866 | 2 | 11.56 | 3 | F3:2376 | 29102019\_RID\_1313\_NaNaPb\_F2.raw |  |  | 1.75E5 |  |  |  |  |  |  |  | 1 | 0 | 0 | 1 | 0 | 0 | 0 | 0 | 0 | 0 | 0 | 82 | 95 |  |
| M.IQEQISNLEAQITDVR.Q | Y | 59.06 | 1855.9639 | 16 | 0.7 | 619.6624 | 3 | 21.91 | 1 | F1:6667 | 29102019\_RID\_1313\_NaNaPb\_F1.raw | 8.87E4 | 9.08E4 |  |  |  | 1.91E5 |  |  |  |  | 3 | 1 | 1 | 0 | 0 | 0 | 1 | 0 | 0 | 0 | 0 | 412 | 427 |  |
| Y.HNLLEGGQEDFESSGAGK.I | Y | 58.63 | 1873.8442 | 18 | -0.1 | 625.6219 | 3 | 11.83 | 3 | F3:2639 | 29102019\_RID\_1313\_NaNaPb\_F2.raw |  |  | 5.41E4 |  |  |  |  |  |  |  | 1 | 0 | 0 | 1 | 0 | 0 | 0 | 0 | 0 | 0 | 0 | 455 | 472 |  |
| K.IQDWYDK.K | Y | 57.18 | 966.4447 | 7 | 0.8 | 484.2300 | 2 | 11.66 | 6 | F6:2447 | 29102019\_RID\_1313\_NaNaPb\_F5.raw | 1.17E5 | 1.38E5 | 5.24E5 |  |  | 4.35E5 |  |  |  |  | 4 | 1 | 1 | 1 | 0 | 0 | 1 | 0 | 0 | 0 | 0 | 185 | 191 |  |
| R.QVLDNLTMEK.S | Y | 56.73 | 1189.6013 | 10 | 0.7 | 595.8083 | 2 | 12.36 | 3 | F3:3148 | 29102019\_RID\_1313\_NaNaPb\_F2.raw |  |  | 2.88E5 |  |  |  |  |  |  |  | 1 | 0 | 0 | 1 | 0 | 0 | 0 | 0 | 0 | 0 | 0 | 262 | 271 |  |
| K.FEMEQNLR.Q | Y | 56.23 | 1065.4913 | 8 | 0.1 | 533.7530 | 2 | 11.98 | 1 | F1:3474 | 29102019\_RID\_1313\_NaNaPb\_F1.raw | 4.88E4 |  | 1.63E5 |  |  |  |  |  |  |  | 2 | 1 | 0 | 1 | 0 | 0 | 0 | 0 | 0 | 0 | 0 | 243 | 250 |  |
| R.MTLDDFR.I | Y | 56.00 | 896.4062 | 7 | -0.8 | 449.2100 | 2 | 12.42 | 7 | F7:3151 | 29102019\_RID\_1313\_NaNaPb\_F6.raw | 1.08E5 | 5.17E4 | 2.38E5 |  |  |  | 0 |  | 1.39E4 |  | 5 | 1 | 1 | 1 | 0 | 0 | 0 | 1 | 0 | 1 | 0 | 234 | 240 |  |
| R.LASYLDK.V | N | 55.97 | 808.4330 | 7 | -0.9 | 405.2234 | 2 | 11.66 | 1 | F1:3180 | 29102019\_RID\_1313\_NaNaPb\_F1.raw | 9.27E4 |  | 3.47E5 |  |  | 0 | 5.32E4 |  |  |  | 4 | 1 | 0 | 1 | 0 | 0 | 1 | 1 | 0 | 0 | 0 | 164 | 170 |  |
| Y.GGGSGGGFSASSLGGGFGGGSR.G | Y | 55.45 | 1771.7874 | 22 | 0.6 | 886.9015 | 2 | 12.92 | 1 | F1:4155 | 29102019\_RID\_1313\_NaNaPb\_F1.raw | 1.63E4 |  | 3.4E4 |  |  |  |  |  |  |  | 2 | 1 | 0 | 1 | 0 | 0 | 0 | 0 | 0 | 0 | 0 | 74 | 95 |  |
| K.IQDWYDKK.G | Y | 53.18 | 1094.5397 | 8 | 0.2 | 548.2772 | 2 | 11.42 | 3 | F3:2226 | 29102019\_RID\_1313\_NaNaPb\_F2.raw |  |  | 5.82E4 |  |  |  |  |  |  |  | 1 | 0 | 0 | 1 | 0 | 0 | 0 | 0 | 0 | 0 | 0 | 185 | 192 |  |
| K.SDLEM(+15.99)QYETLQEELM(+15.99)ALK.K | Y | 52.11 | 2202.0071 | 18 | 0.6 | 1102.0115 | 2 | 41.33 | 6 | F6:17507 | 29102019\_RID\_1313\_NaNaPb\_F5.raw |  |  |  |  |  | 2.25E5 |  |  |  |  | 1 | 0 | 0 | 0 | 0 | 0 | 1 | 0 | 0 | 0 | 0 | 272 | 289 | Oxidation (M) |
| Y.YNTIDDLK.D | Y | 52.06 | 980.4814 | 8 | 0.7 | 491.2483 | 2 | 11.76 | 3 | F3:2561 | 29102019\_RID\_1313\_NaNaPb\_F2.raw | 1.94E4 |  | 1.18E5 |  |  |  |  |  |  |  | 2 | 1 | 0 | 1 | 0 | 0 | 0 | 0 | 0 | 0 | 0 | 205 | 212 |  |
| K.EEMSQLTGQNSGDVNVEINVAPGK.D | Y | 48.54 | 2515.1860 | 24 | 1.4 | 1258.6021 | 2 | 13.17 | 2 | F2:3833 | 29102019\_RID\_1313\_NaNaPb\_F10.raw |  | 2.38E4 |  |  |  |  |  |  |  |  | 1 | 0 | 1 | 0 | 0 | 0 | 0 | 0 | 0 | 0 | 0 | 294 | 317 |  |
| R.LASYLDKVQALEEANNDLENK.I | Y | 48.37 | 2376.1809 | 21 | 0.4 | 793.0679 | 3 | 19.21 | 1 | F1:6040 | 29102019\_RID\_1313\_NaNaPb\_F1.raw | 2.87E4 |  |  |  |  |  |  |  |  |  | 1 | 1 | 0 | 0 | 0 | 0 | 0 | 0 | 0 | 0 | 0 | 164 | 184 |  |
| S.GGGGGGGLGSGGSIR.S | Y | 47.49 | 1144.5585 | 15 | 0.7 | 573.2869 | 2 | 10.97 | 8 | F8:1648 | 29102019\_RID\_1313\_NaNaPb\_F7.raw |  |  |  |  |  |  |  | 2.05E3 |  |  | 1 | 0 | 0 | 0 | 0 | 0 | 0 | 0 | 1 | 0 | 0 | 15 | 29 |  |
| G.NYGGGSGSGGGSGGGYGGGSGSR.G | Y | 47.06 | 1847.7418 | 23 | 0.5 | 924.8787 | 2 | 10.84 | 3 | F3:1711 | 29102019\_RID\_1313\_NaNaPb\_F2.raw |  |  | 3.89E5 |  |  |  |  |  |  |  | 1 | 0 | 0 | 1 | 0 | 0 | 0 | 0 | 0 | 0 | 0 | 557 | 579 |  |
| R.HGVQELEIELQSQLSKK.A | Y | 47.02 | 1965.0531 | 17 | -0.1 | 656.0249 | 3 | 16.60 | 1 | F1:5381 | 29102019\_RID\_1313\_NaNaPb\_F1.raw | 2.84E4 |  |  |  |  |  |  |  |  |  | 1 | 1 | 0 | 0 | 0 | 0 | 0 | 0 | 0 | 0 | 0 | 375 | 391 |  |
| R.M(+15.99)TLDDFR.I | Y | 43.79 | 912.4011 | 7 | -0.1 | 457.2078 | 2 | 11.93 | 1 | F1:3392 | 29102019\_RID\_1313\_NaNaPb\_F1.raw | 4.62E4 |  | 1.49E5 |  |  |  |  |  |  |  | 2 | 1 | 0 | 1 | 0 | 0 | 0 | 0 | 0 | 0 | 0 | 234 | 240 | Oxidation (M) |
| K.FEM(+15.99)EQNLR.Q | Y | 42.31 | 1081.4862 | 8 | 0.7 | 541.7507 | 2 | 11.27 | 3 | F3:2098 | 29102019\_RID\_1313\_NaNaPb\_F2.raw |  |  | 2.61E4 |  |  |  |  |  |  |  | 1 | 0 | 0 | 1 | 0 | 0 | 0 | 0 | 0 | 0 | 0 | 243 | 250 | Oxidation (M) |
| R.IKFEMEQNLR.Q | Y | 42.24 | 1306.6703 | 10 | 0.7 | 436.5643 | 3 | 52.79 | 5 | F5:17973 | 29102019\_RID\_1313\_NaNaPb\_F4.raw |  |  |  |  | 0 |  |  |  |  |  | 1 | 0 | 0 | 0 | 0 | 1 | 0 | 0 | 0 | 0 | 0 | 241 | 250 |  |
| total 50 peptides |
| --- |

P04264|K2C1\_HUMAN

back to list

  

| Protein Coverage
| Supporting Peptides
|

Protein Coverage:

Supporting Peptides:

| Peptide | Uniq | -10lgP | Mass | Length | ppm | m/z | z | RT | Fraction | Scan | Source File | Area F1 | Area F10 | Area F2 | Area F3 | Area F4 | Area F5 | Area F6 | Area F7 | Area F8 | Area F9 | #Feature | #Feature F1 | #Feature F10 | #Feature F2 | #Feature F3 | #Feature F4 | #Feature F5 | #Feature F6 | #Feature F7 | #Feature F8 | #Feature F9 | Start | End | PTM |
| --- | --- | --- | --- | --- | --- | --- | --- | --- | --- | --- | --- | --- | --- | --- | --- | --- | --- | --- | --- | --- | --- | --- | --- | --- | --- | --- | --- | --- | --- | --- | --- | --- | --- | --- | --- |
| R.FSSC(+57.02)GGGGGSFGAGGGFGSR.S | Y | 127.70 | 1764.7274 | 20 | -1.1 | 883.3700 | 2 | 11.73 | 7 | F7:2577 | 29102019\_RID\_1313\_NaNaPb\_F6.raw | 9.25E5 | 1.79E6 | 7.59E5 | 2.59E4 |  | 3.32E6 | 4.7E5 | 6.08E4 |  | 0 | 8 | 1 | 1 | 1 | 1 | 0 | 1 | 1 | 1 | 0 | 1 | 46 | 65 | Carbamidomethylation |
| R.GGGGGGYGSGGSSYGSGGGSYGSGGGGGGGR.G | Y | 117.70 | 2382.9446 | 31 | -0.1 | 1192.4795 | 2 | 11.04 | 6 | F6:1872 | 29102019\_RID\_1313\_NaNaPb\_F5.raw |  | 1.64E5 | 1.51E5 |  |  | 4.32E5 | 1.54E5 |  | 5.66E4 |  | 7 | 0 | 1 | 2 | 0 | 0 | 1 | 2 | 0 | 1 | 0 | 519 | 549 |  |
| K.QISNLQQSISDAEQR.G | Y | 113.04 | 1715.8438 | 15 | 0.3 | 858.9294 | 2 | 12.22 | 1 | F1:3680 | 29102019\_RID\_1313\_NaNaPb\_F1.raw | 1.41E6 | 1.64E6 | 4.44E5 |  |  | 3.25E6 | 3.41E5 |  |  | 1.86E5 | 14 | 3 | 2 | 3 | 0 | 0 | 3 | 2 | 0 | 0 | 1 | 418 | 432 |  |
| R.SGGGFSSGSAGIINYQR.R | Y | 108.04 | 1656.7855 | 17 | 0.9 | 829.4008 | 2 | 11.97 | 6 | F6:2725 | 29102019\_RID\_1313\_NaNaPb\_F5.raw | 1.65E5 | 1.06E5 | 2.53E5 |  |  | 4.41E5 | 1.14E5 |  |  |  | 5 | 1 | 1 | 1 | 0 | 0 | 1 | 1 | 0 | 0 | 0 | 13 | 29 |  |
| R.THNLEPYFESFINNLR.R | Y | 101.84 | 1992.9694 | 16 | 1.2 | 665.3312 | 3 | 32.16 | 6 | F6:13857 | 29102019\_RID\_1313\_NaNaPb\_F5.raw | 1.39E5 | 4.17E6 | 1.57E5 |  | 0 | 4.56E6 | 9.45E5 |  | 2.69E5 |  | 12 | 1 | 3 | 1 | 0 | 1 | 3 | 2 | 0 | 1 | 0 | 224 | 239 |  |
| R.FLEQQNQVLQTK.W | N | 97.49 | 1474.7780 | 12 | 0.1 | 738.3963 | 2 | 12.00 | 1 | F1:3480 | 29102019\_RID\_1313\_NaNaPb\_F1.raw | 3.66E6 | 4.06E6 | 5.53E6 | 1.56E5 |  | 6.8E6 |  |  | 2.74E5 |  | 6 | 1 | 1 | 1 | 1 | 0 | 1 | 0 | 0 | 1 | 0 | 200 | 211 |  |
| K.LNDLEDALQQAK.E | Y | 96.95 | 1356.6885 | 12 | 1.5 | 679.3525 | 2 | 12.53 | 9 | F9:3194 | 29102019\_RID\_1313\_NaNaPb\_F8.raw | 3.67E6 | 3.76E6 | 6.66E6 | 1.19E5 |  | 1.06E7 | 9.42E5 |  | 2.89E5 | 5.16E5 | 12 | 3 | 1 | 3 | 1 | 0 | 1 | 1 | 0 | 1 | 1 | 444 | 455 |  |
| K.SKAEAESLYQSK.Y | Y | 94.70 | 1339.6619 | 12 | 0.6 | 670.8386 | 2 | 10.84 | 7 | F7:1725 | 29102019\_RID\_1313\_NaNaPb\_F6.raw |  |  | 8.68E4 |  |  |  | 2.75E5 |  |  |  | 5 | 0 | 0 | 2 | 0 | 0 | 0 | 3 | 0 | 0 | 0 | 365 | 376 |  |
| R.TNAENEFVTIK.K | Y | 90.52 | 1264.6299 | 11 | 1.2 | 633.3230 | 2 | 12.39 | 1 | F1:3828 | 29102019\_RID\_1313\_NaNaPb\_F1.raw | 1.48E6 | 1.9E6 | 4.21E6 | 5.92E4 |  | 1.64E6 |  |  |  | 1.85E5 | 7 | 1 | 1 | 2 | 1 | 0 | 1 | 0 | 0 | 0 | 1 | 278 | 288 |  |
| K.WELLQQVDTSTR.T | Y | 89.01 | 1474.7416 | 12 | 1.0 | 738.3788 | 2 | 16.97 | 6 | F6:6044 | 29102019\_RID\_1313\_NaNaPb\_F5.raw | 1.78E6 | 1.76E6 | 2.62E6 |  |  | 6E6 | 1.74E6 |  | 3.42E4 | 1.43E5 | 16 | 3 | 2 | 3 | 0 | 0 | 3 | 3 | 0 | 1 | 1 | 212 | 223 |  |
| R.MSGEC(+57.02)APNVSVSVSTSHTTISGGGSR.G | Y | 87.84 | 2564.1594 | 26 | 0.1 | 855.7272 | 3 | 11.66 | 1 | F1:3202 | 29102019\_RID\_1313\_NaNaPb\_F1.raw | 5.17E4 |  | 9.46E4 |  |  |  |  |  |  |  | 2 | 1 | 0 | 1 | 0 | 0 | 0 | 0 | 0 | 0 | 0 | 493 | 518 | Carbamidomethylation |
| K.SLNNQFASFIDK.V | Y | 81.07 | 1382.6830 | 12 | 1.2 | 692.3496 | 2 | 17.16 | 6 | F6:6314 | 29102019\_RID\_1313\_NaNaPb\_F5.raw | 2.62E6 | 2.93E6 | 3.46E6 | 1.03E5 |  | 1.14E7 |  |  | 5.54E5 | 5.33E5 | 13 | 2 | 2 | 3 | 1 | 0 | 3 | 0 | 0 | 1 | 1 | 186 | 197 |  |
| K.YEELQITAGR.H | N | 80.75 | 1178.5931 | 10 | 0.0 | 590.3038 | 2 | 11.73 | 9 | F9:2532 | 29102019\_RID\_1313\_NaNaPb\_F8.raw | 1.43E6 |  | 0 | 3.88E4 |  | 3.39E6 | 5.56E5 |  | 2.91E5 | 2.75E5 | 7 | 1 | 0 | 1 | 1 | 0 | 1 | 1 | 0 | 1 | 1 | 377 | 386 |  |
| K.AEAESLYQSK.Y | Y | 80.29 | 1124.5349 | 10 | 0.8 | 563.2752 | 2 | 11.33 | 1 | F1:2872 | 29102019\_RID\_1313\_NaNaPb\_F1.raw | 8.01E4 | 2.09E5 | 4.9E5 |  |  | 1.34E5 |  |  | 5.7E4 |  | 5 | 1 | 1 | 1 | 0 | 0 | 1 | 0 | 0 | 1 | 0 | 367 | 376 |  |
| K.LALDLEIATYR.T | N | 79.81 | 1276.7026 | 11 | 1.0 | 639.3593 | 2 | 28.28 | 1 | F1:8310 | 29102019\_RID\_1313\_NaNaPb\_F1.raw | 4.75E5 | 2.26E6 | 3.53E5 |  |  | 3.39E6 | 8.11E5 |  | 1.78E5 |  | 19 | 2 | 6 | 2 | 0 | 0 | 5 | 3 | 0 | 1 | 0 | 473 | 483 |  |
| R.M(+15.99)SGEC(+57.02)APNVSVSVSTSHTTISGGGSR.G | Y | 79.66 | 2580.1545 | 26 | 0.6 | 861.0593 | 3 | 11.60 | 3 | F3:2429 | 29102019\_RID\_1313\_NaNaPb\_F2.raw |  | 4.75E4 | 8.01E4 |  |  |  |  |  |  |  | 2 | 0 | 1 | 1 | 0 | 0 | 0 | 0 | 0 | 0 | 0 | 493 | 518 | Oxidation (M); Carbamidomethylation |
| R.SLDLDSIIAEVK.A | N | 78.89 | 1301.7078 | 12 | 0.4 | 651.8614 | 2 | 34.51 | 9 | F9:13199 | 29102019\_RID\_1313\_NaNaPb\_F8.raw | 1.93E6 | 5.03E6 | 3.54E6 | 9.45E4 | 0 | 5.83E6 | 2.88E6 |  | 7.49E5 | 2.61E5 | 20 | 3 | 3 | 3 | 1 | 1 | 4 | 3 | 0 | 1 | 1 | 344 | 355 |  |
| K.AQYEDIAQK.S | N | 73.27 | 1064.5138 | 9 | 0.4 | 533.2644 | 2 | 11.09 | 7 | F7:1952 | 29102019\_RID\_1313\_NaNaPb\_F6.raw | 1.11E5 | 2.26E5 | 5.4E5 |  |  |  | 1.24E5 |  |  |  | 4 | 1 | 1 | 1 | 0 | 0 | 0 | 1 | 0 | 0 | 0 | 356 | 364 |  |
| R.TLLEGEESR.M | Y | 70.82 | 1032.5087 | 9 | 0.7 | 517.2620 | 2 | 11.60 | 1 | F1:3118 | 29102019\_RID\_1313\_NaNaPb\_F1.raw | 2.57E5 | 4.87E5 | 9.07E5 |  |  | 2.35E5 |  |  |  |  | 4 | 1 | 1 | 1 | 0 | 0 | 1 | 0 | 0 | 0 | 0 | 484 | 492 |  |
| K.SLNNQFASFIDKVR.F | Y | 69.00 | 1637.8525 | 14 | 0.2 | 546.9583 | 3 | 71.57 | 5 | F5:33759 | 29102019\_RID\_1313\_NaNaPb\_F4.raw | 2.97E5 |  | 1.71E4 |  | 1.14E6 |  | 9.03E5 |  |  |  | 4 | 1 | 0 | 1 | 0 | 1 | 0 | 1 | 0 | 0 | 0 | 186 | 199 |  |
| R.TNAENEFVTIKK.D | Y | 67.48 | 1392.7249 | 12 | 0.5 | 465.2491 | 3 | 44.34 | 5 | F5:11648 | 29102019\_RID\_1313\_NaNaPb\_F4.raw |  |  |  |  | 4.32E5 |  | 7.22E4 |  |  |  | 2 | 0 | 0 | 0 | 0 | 1 | 0 | 1 | 0 | 0 | 0 | 278 | 289 |  |
| R.DYQELMNTK.L | N | 66.30 | 1140.5121 | 9 | 1.4 | 571.2641 | 2 | 12.11 | 1 | F1:3593 | 29102019\_RID\_1313\_NaNaPb\_F1.raw | 1.41E5 |  | 3.07E5 |  |  |  |  |  |  |  | 2 | 1 | 0 | 1 | 0 | 0 | 0 | 0 | 0 | 0 | 0 | 464 | 472 |  |
| R.SLDLDSIIAEVKAQYEDIAQK.S | Y | 65.59 | 2348.2109 | 21 | 0.8 | 783.7449 | 3 | 54.40 | 9 | F9:18866 | 29102019\_RID\_1313\_NaNaPb\_F8.raw |  |  |  |  |  |  |  |  | 3.29E4 |  | 1 | 0 | 0 | 0 | 0 | 0 | 0 | 0 | 0 | 1 | 0 | 344 | 364 |  |
| R.THNLEPYFESFINNLRR.R | Y | 64.21 | 2149.0703 | 17 | 0.0 | 538.2748 | 4 | 24.53 | 7 | F7:9009 | 29102019\_RID\_1313\_NaNaPb\_F6.raw |  |  |  |  | 8.05E5 |  | 1.38E5 |  |  |  | 3 | 0 | 0 | 0 | 0 | 2 | 0 | 1 | 0 | 0 | 0 | 224 | 240 |  |
| R.SLVNLGGSK.S | Y | 64.21 | 873.4919 | 9 | 0.0 | 437.7532 | 2 | 12.04 | 1 | F1:3537 | 29102019\_RID\_1313\_NaNaPb\_F1.raw | 1.87E5 | 2.33E5 | 8E5 |  | 1.21E6 | 2.03E5 | 0 |  |  |  | 6 | 1 | 1 | 1 | 0 | 1 | 1 | 1 | 0 | 0 | 0 | 66 | 74 |  |
| C.GGGGGSFGAGGGFGSR.S | Y | 63.65 | 1283.5642 | 16 | 0.4 | 642.7896 | 2 | 11.66 | 3 | F3:2496 | 29102019\_RID\_1313\_NaNaPb\_F2.raw | 4.89E3 |  | 6E4 |  |  |  |  |  |  |  | 2 | 1 | 0 | 1 | 0 | 0 | 0 | 0 | 0 | 0 | 0 | 50 | 65 |  |
| K.NMQDMVEDYR.N | Y | 61.41 | 1299.5223 | 10 | 0.7 | 650.7689 | 2 | 12.43 | 1 | F1:3877 | 29102019\_RID\_1313\_NaNaPb\_F1.raw | 3.3E4 |  | 3.1E4 |  |  |  |  |  |  |  | 2 | 1 | 0 | 1 | 0 | 0 | 0 | 0 | 0 | 0 | 0 | 258 | 267 |  |
| N.VSVSVSTSHTTISGGGSR.G | Y | 61.26 | 1717.8595 | 18 | 1.0 | 573.6277 | 3 | 11.34 | 3 | F3:2158 | 29102019\_RID\_1313\_NaNaPb\_F2.raw | 2.4E4 | 2.16E4 | 1.01E5 |  |  | 3.54E4 |  |  |  |  | 4 | 1 | 1 | 1 | 0 | 0 | 1 | 0 | 0 | 0 | 0 | 501 | 518 |  |
| Q.SLLQPLNVEIDPEIQK.V | Y | 59.87 | 1835.0040 | 16 | 0.2 | 918.5095 | 2 | 33.05 | 1 | F1:9617 | 29102019\_RID\_1313\_NaNaPb\_F1.raw | 2.73E4 |  |  |  |  |  |  |  |  |  | 1 | 1 | 0 | 0 | 0 | 0 | 0 | 0 | 0 | 0 | 0 | 160 | 175 |  |
| R.NKYEDEINKR.T | N | 59.85 | 1307.6470 | 10 | -0.3 | 436.8895 | 3 | 10.75 | 6 | F6:1631 | 29102019\_RID\_1313\_NaNaPb\_F5.raw |  | 2.17E5 | 1.52E5 |  |  | 3.47E4 | 2.69E5 |  |  |  | 4 | 0 | 1 | 1 | 0 | 0 | 1 | 1 | 0 | 0 | 0 | 268 | 277 |  |
| L.NVEIDPEIQK.V | N | 57.64 | 1183.6084 | 10 | -0.1 | 592.8114 | 2 | 12.00 | 1 | F1:3473 | 29102019\_RID\_1313\_NaNaPb\_F1.raw | 5.02E5 | 4.97E5 | 1.77E6 |  |  |  | 1.29E5 |  |  |  | 4 | 1 | 1 | 1 | 0 | 0 | 0 | 1 | 0 | 0 | 0 | 166 | 175 |  |
| K.IEISELNR.V | N | 55.32 | 972.5240 | 8 | 0.4 | 487.2695 | 2 | 11.76 | 7 | F7:2595 | 29102019\_RID\_1313\_NaNaPb\_F6.raw | 9.39E5 |  | 2.11E6 | 7.37E4 |  | 2.16E6 | 2.78E5 |  | 1.23E5 |  | 6 | 1 | 0 | 1 | 1 | 0 | 1 | 1 | 0 | 1 | 0 | 396 | 403 |  |
| R.THNLEPYFESF.I | Y | 55.10 | 1382.6143 | 11 | 0.4 | 692.3147 | 2 | 19.52 | 6 | F6:7323 | 29102019\_RID\_1313\_NaNaPb\_F5.raw |  | 1.39E6 | 1.63E6 |  |  | 1.83E6 |  |  | 5.55E4 | 1.62E5 | 6 | 0 | 1 | 1 | 0 | 0 | 2 | 0 | 0 | 1 | 1 | 224 | 234 |  |
| R.GSYGSGGSSYGSGGGSYGSGGGGGGHGSYGSGSSSGGYR.G | Y | 53.14 | 3311.3008 | 39 | 0.2 | 1104.7744 | 3 | 11.04 | 9 | F9:1922 | 29102019\_RID\_1313\_NaNaPb\_F8.raw |  | 6.72E4 | 9.68E4 |  |  | 1.23E5 |  |  | 7.65E4 |  | 4 | 0 | 1 | 1 | 0 | 0 | 1 | 0 | 0 | 1 | 0 | 550 | 588 |  |
| R.FLEQQNQVLQTKWELLQQVDTSTR.T | Y | 52.44 | 2931.5090 | 24 | 0.0 | 978.1770 | 3 | 23.58 | 7 | F7:8703 | 29102019\_RID\_1313\_NaNaPb\_F6.raw |  |  |  |  | 1.35E6 |  | 2.92E5 |  |  |  | 2 | 0 | 0 | 0 | 0 | 1 | 0 | 1 | 0 | 0 | 0 | 200 | 223 |  |
| K.NM(+15.99)QDM(+15.99)VEDYR.N | Y | 51.09 | 1331.5122 | 10 | 1.1 | 666.7641 | 2 | 11.20 | 3 | F3:2028 | 29102019\_RID\_1313\_NaNaPb\_F2.raw |  |  | 5.09E4 |  |  |  |  |  |  |  | 1 | 0 | 0 | 1 | 0 | 0 | 0 | 0 | 0 | 0 | 0 | 258 | 267 | Oxidation (M) |
| R.GGGGGGYGSGGSSYGSGGGSYGSGGGGGGGRG.S | Y | 50.32 | 2439.9661 | 32 | 0.9 | 1220.9915 | 2 | 11.14 | 3 | F3:1971 | 29102019\_RID\_1313\_NaNaPb\_F2.raw |  |  | 1.28E5 |  |  | 1.41E5 |  |  |  |  | 2 | 0 | 0 | 1 | 0 | 0 | 1 | 0 | 0 | 0 | 0 | 519 | 550 |  |
| W.ELLQQVDTSTR.T | Y | 48.85 | 1288.6622 | 11 | 0.2 | 645.3385 | 2 | 11.95 | 1 | F1:3467 | 29102019\_RID\_1313\_NaNaPb\_F1.raw | 1.77E4 |  |  |  |  |  |  |  |  |  | 1 | 1 | 0 | 0 | 0 | 0 | 0 | 0 | 0 | 0 | 0 | 213 | 223 |  |
| Q.FASFIDK.V | N | 46.57 | 826.4225 | 7 | -0.3 | 414.2184 | 2 | 11.89 | 2 | F2:2810 | 29102019\_RID\_1313\_NaNaPb\_F10.raw |  | 1.96E5 |  |  |  | 3.22E5 |  |  | 0 |  | 3 | 0 | 1 | 0 | 0 | 0 | 1 | 0 | 0 | 1 | 0 | 191 | 197 |  |
| R.THNLEPYFES.F | Y | 45.78 | 1235.5459 | 10 | 0.7 | 618.7806 | 2 | 12.52 | 3 | F3:3222 | 29102019\_RID\_1313\_NaNaPb\_F2.raw |  |  | 8.54E4 |  |  |  |  |  |  |  | 1 | 0 | 0 | 1 | 0 | 0 | 0 | 0 | 0 | 0 | 0 | 224 | 233 |  |
| Q.ISETNVILSMDNNR.S | Y | 45.76 | 1604.7828 | 14 | 0.7 | 803.3993 | 2 | 14.36 | 3 | F3:3983 | 29102019\_RID\_1313\_NaNaPb\_F2.raw | 4.08E4 |  | 5.06E4 |  |  |  |  |  |  |  | 2 | 1 | 0 | 1 | 0 | 0 | 0 | 0 | 0 | 0 | 0 | 330 | 343 |  |
| K.SISISVAR.G | Y | 45.43 | 831.4814 | 8 | 0.4 | 416.7481 | 2 | 11.97 | 1 | F1:3458 | 29102019\_RID\_1313\_NaNaPb\_F1.raw | 1.79E5 |  |  |  |  | 6.94E5 | 0 |  |  |  | 3 | 1 | 0 | 0 | 0 | 0 | 1 | 1 | 0 | 0 | 0 | 75 | 82 |  |
| K.AEAESLYQSKYEELQITAGR.H | Y | 45.30 | 2285.1174 | 20 | -0.1 | 762.7130 | 3 | 13.09 | 1 | F1:4239 | 29102019\_RID\_1313\_NaNaPb\_F1.raw | 1.2E4 |  |  |  |  |  |  |  |  |  | 1 | 1 | 0 | 0 | 0 | 0 | 0 | 0 | 0 | 0 | 0 | 367 | 386 |  |
| R.THNLEPYFE.S | Y | 44.93 | 1148.5138 | 9 | -0.3 | 575.2640 | 2 | 12.76 | 3 | F3:3341 | 29102019\_RID\_1313\_NaNaPb\_F2.raw |  |  | 8.52E5 |  |  |  |  |  |  |  | 1 | 0 | 0 | 1 | 0 | 0 | 0 | 0 | 0 | 0 | 0 | 224 | 232 |  |
| total 44 peptides |
| --- |

P13645|K1C10\_HUMAN

back to list

  

| Protein Coverage
| Supporting Peptides
|

Protein Coverage:

Supporting Peptides:

| Peptide | Uniq | -10lgP | Mass | Length | ppm | m/z | z | RT | Fraction | Scan | Source File | Area F1 | Area F10 | Area F2 | Area F3 | Area F4 | Area F5 | Area F6 | Area F7 | Area F8 | Area F9 | #Feature | #Feature F1 | #Feature F10 | #Feature F2 | #Feature F3 | #Feature F4 | #Feature F5 | #Feature F6 | #Feature F7 | #Feature F8 | #Feature F9 | Start | End | PTM |
| --- | --- | --- | --- | --- | --- | --- | --- | --- | --- | --- | --- | --- | --- | --- | --- | --- | --- | --- | --- | --- | --- | --- | --- | --- | --- | --- | --- | --- | --- | --- | --- | --- | --- | --- | --- |
| R.GSSGGGC(+57.02)FGGSSGGYGGLGGFGGGSFR.G | Y | 121.49 | 2341.9771 | 27 | 0.1 | 1171.9960 | 2 | 16.10 | 6 | F6:5559 | 29102019\_RID\_1313\_NaNaPb\_F5.raw |  | 1.45E6 | 1.83E4 |  |  | 5.27E6 | 2.77E5 |  |  |  | 10 | 0 | 3 | 1 | 0 | 0 | 4 | 2 | 0 | 0 | 0 | 60 | 86 | Carbamidomethylation |
| K.ELTTEIDNNIEQISSYK.S | Y | 117.47 | 1995.9636 | 17 | 0.7 | 998.9897 | 2 | 22.16 | 1 | F1:6720 | 29102019\_RID\_1313\_NaNaPb\_F1.raw | 1.76E6 | 2.79E6 | 5.41E5 | 2.04E5 |  | 4.26E6 | 2.18E4 |  | 3.58E5 |  | 13 | 2 | 4 | 1 | 1 | 0 | 3 | 1 | 0 | 1 | 0 | 346 | 362 |  |
| R.ALEESNYELEGK.I | N | 104.50 | 1380.6409 | 12 | 0.6 | 691.3281 | 2 | 11.73 | 1 | F1:3243 | 29102019\_RID\_1313\_NaNaPb\_F1.raw | 6.15E5 | 1.46E6 | 4.47E5 | 6.12E4 |  | 1.11E6 | 1.38E5 |  |  |  | 6 | 1 | 1 | 1 | 1 | 0 | 1 | 1 | 0 | 0 | 0 | 166 | 177 |  |
| K.QSLEASLAETEGR.Y | N | 102.17 | 1389.6736 | 13 | 0.2 | 695.8442 | 2 | 12.26 | 1 | F1:3723 | 29102019\_RID\_1313\_NaNaPb\_F1.raw | 9.8E5 | 1.24E6 |  | 3.98E4 |  | 1.68E6 |  |  |  | 0 | 6 | 1 | 1 | 0 | 1 | 0 | 2 | 0 | 0 | 0 | 1 | 387 | 399 |  |
| K.NQILNLTTDNANILLQIDNAR.L | Y | 100.19 | 2366.2554 | 21 | 0.9 | 789.7598 | 3 | 37.31 | 2 | F2:12450 | 29102019\_RID\_1313\_NaNaPb\_F10.raw |  | 3.78E6 |  |  |  | 2.07E6 |  |  | 1.36E5 |  | 7 | 0 | 4 | 0 | 0 | 0 | 2 | 0 | 0 | 1 | 0 | 208 | 228 |  |
| K.GSLGGGFSSGGFSGGSFSR.G | Y | 93.04 | 1706.7648 | 19 | 1.4 | 854.3909 | 2 | 12.82 | 6 | F6:3407 | 29102019\_RID\_1313\_NaNaPb\_F5.raw | 4.17E5 | 4.21E5 | 8.93E4 |  |  | 1.73E6 |  |  | 1.54E5 |  | 5 | 1 | 1 | 1 | 0 | 0 | 1 | 0 | 0 | 1 | 0 | 41 | 59 |  |
| R.SQYEQLAEQNR.K | Y | 90.83 | 1364.6321 | 11 | 0.2 | 683.3234 | 2 | 11.17 | 9 | F9:2033 | 29102019\_RID\_1313\_NaNaPb\_F8.raw | 0 | 8.03E5 | 3.11E5 | 0 |  | 5.23E5 | 6.85E4 |  | 1.95E5 |  | 7 | 1 | 1 | 1 | 1 | 0 | 1 | 1 | 0 | 1 | 0 | 323 | 333 |  |
| R.SLLEGEGSSGGGGR.G | Y | 86.71 | 1261.5898 | 14 | 0.7 | 631.8026 | 2 | 11.53 | 1 | F1:3061 | 29102019\_RID\_1313\_NaNaPb\_F1.raw | 7.39E4 | 3.79E5 | 1.92E5 |  |  | 5.29E4 | 5.89E4 |  | 5.73E4 |  | 6 | 1 | 1 | 1 | 0 | 0 | 1 | 1 | 0 | 1 | 0 | 451 | 464 |  |
| R.LENEIQTYR.S | N | 77.64 | 1164.5775 | 9 | -0.3 | 583.2958 | 2 | 11.60 | 1 | F1:3119 | 29102019\_RID\_1313\_NaNaPb\_F1.raw | 4.55E5 | 1.04E6 | 4E5 |  |  | 8.28E5 | 1.21E5 |  |  |  | 5 | 1 | 1 | 1 | 0 | 0 | 1 | 1 | 0 | 0 | 0 | 442 | 450 |  |
| R.AETEC(+57.02)QNTEYQQLLDIK.I | Y | 75.86 | 2081.9575 | 17 | 0.3 | 1041.9863 | 2 | 16.15 | 6 | F6:5459 | 29102019\_RID\_1313\_NaNaPb\_F5.raw | 2.16E6 | 3.74E6 | 6.56E5 | 2.94E5 |  | 1.01E7 |  |  | 2.23E4 | 1.17E5 | 12 | 2 | 3 | 1 | 1 | 0 | 3 | 0 | 0 | 1 | 1 | 423 | 439 | Carbamidomethylation |
| R.SQYEQLAEQNRK.D | Y | 75.63 | 1492.7269 | 12 | -0.4 | 498.5827 | 3 | 10.91 | 7 | F7:1794 | 29102019\_RID\_1313\_NaNaPb\_F6.raw |  |  |  |  |  |  | 3.71E4 |  |  |  | 1 | 0 | 0 | 0 | 0 | 0 | 0 | 1 | 0 | 0 | 0 | 323 | 334 |  |
| R.QSVEADINGLR.R | N | 73.92 | 1200.6099 | 11 | -0.1 | 601.3121 | 2 | 11.73 | 9 | F9:2547 | 29102019\_RID\_1313\_NaNaPb\_F8.raw | 6.63E5 | 1.4E6 | 2.92E5 | 4.55E4 |  |  | 4.34E4 |  | 1.09E5 |  | 6 | 1 | 1 | 1 | 1 | 0 | 0 | 1 | 0 | 1 | 0 | 246 | 256 |  |
| R.NVQALEIELQSQLALK.Q | Y | 73.80 | 1796.0043 | 16 | 0.9 | 899.0102 | 2 | 31.83 | 2 | F2:11083 | 29102019\_RID\_1313\_NaNaPb\_F10.raw | 3.89E4 | 1.22E6 |  |  |  | 1.31E6 | 5.85E4 |  | 2.91E4 |  | 11 | 1 | 5 | 0 | 0 | 0 | 3 | 1 | 0 | 1 | 0 | 371 | 386 |  |
| R.YC(+57.02)VQLSQIQAQISALEEQLQQIR.A | Y | 73.16 | 2745.4119 | 23 | 0.6 | 916.1451 | 3 | 60.77 | 9 | F9:20217 | 29102019\_RID\_1313\_NaNaPb\_F8.raw |  |  |  |  |  | 5.86E4 |  |  | 3.09E4 |  | 2 | 0 | 0 | 0 | 0 | 0 | 1 | 0 | 0 | 1 | 0 | 400 | 422 | Carbamidomethylation |
| K.YENEVALR.Q | N | 67.71 | 992.4927 | 8 | -0.4 | 497.2534 | 2 | 11.60 | 1 | F1:3120 | 29102019\_RID\_1313\_NaNaPb\_F1.raw | 1.54E5 | 3.31E5 | 1.98E5 |  |  | 6.09E4 | 3.89E4 |  |  |  | 5 | 1 | 1 | 1 | 0 | 0 | 1 | 1 | 0 | 0 | 0 | 238 | 245 |  |
| R.VLDELTLTK.A | N | 66.60 | 1030.5911 | 9 | 0.4 | 516.3030 | 2 | 12.39 | 2 | F2:3275 | 29102019\_RID\_1313\_NaNaPb\_F10.raw | 1.23E6 | 1.37E6 | 6.44E5 |  |  | 1.74E6 | 1.16E5 |  | 2.5E5 | 6.2E4 | 7 | 1 | 1 | 1 | 0 | 0 | 1 | 1 | 0 | 1 | 1 | 258 | 266 |  |
| K.ADLEM(+15.99)QIESLTEELAYLK.K | N | 65.59 | 2111.0344 | 18 | 0.4 | 704.6857 | 3 | 61.82 | 2 | F2:18580 | 29102019\_RID\_1313\_NaNaPb\_F10.raw |  | 3.12E5 |  |  |  |  |  |  | 1.94E4 |  | 4 | 0 | 3 | 0 | 0 | 0 | 0 | 0 | 0 | 1 | 0 | 267 | 284 | Oxidation (M) |
| L.TTDNANILLQIDNAR.L | Y | 64.61 | 1670.8588 | 15 | 1.3 | 836.4377 | 2 | 16.81 | 2 | F2:5710 | 29102019\_RID\_1313\_NaNaPb\_F10.raw |  | 3.84E4 |  |  |  |  |  |  |  |  | 1 | 0 | 1 | 0 | 0 | 0 | 0 | 0 | 0 | 0 | 0 | 214 | 228 |  |
| K.DAEAWFNEK.S | N | 62.34 | 1108.4825 | 9 | -0.2 | 555.2484 | 2 | 12.90 | 2 | F2:3648 | 29102019\_RID\_1313\_NaNaPb\_F10.raw | 8.2E5 | 1.25E6 | 3.5E5 | 8.55E4 |  | 1.58E6 | 2.55E5 |  | 2.78E5 |  | 7 | 1 | 1 | 1 | 1 | 0 | 1 | 1 | 0 | 1 | 0 | 335 | 343 |  |
| K.NQILNLTTDNAN.I | Y | 61.73 | 1329.6525 | 12 | 0.5 | 665.8339 | 2 | 17.05 | 1 | F1:5478 | 29102019\_RID\_1313\_NaNaPb\_F1.raw | 1.68E5 |  | 8.74E4 | 2.53E4 |  | 2.23E5 |  |  |  |  | 4 | 1 | 0 | 1 | 1 | 0 | 1 | 0 | 0 | 0 | 0 | 208 | 219 |  |
| Q.ISALEEQLQQIR.A | N | 60.76 | 1426.7780 | 12 | 1.2 | 714.3971 | 2 | 14.36 | 6 | F6:4471 | 29102019\_RID\_1313\_NaNaPb\_F5.raw | 1.15E6 | 1.93E6 | 4.69E5 |  |  | 2.28E6 |  |  | 1.12E5 |  | 8 | 3 | 1 | 1 | 0 | 0 | 2 | 0 | 0 | 1 | 0 | 411 | 422 |  |
| R.NVSTGDVNVEM(+15.99)NAAPGVDLTQLLNNM(+15.99)R.S | N | 59.10 | 2903.3752 | 27 | 0.7 | 968.7997 | 3 | 29.27 | 3 | F3:6955 | 29102019\_RID\_1313\_NaNaPb\_F2.raw | 5.13E4 | 1.28E5 | 1.92E4 |  |  |  |  |  |  |  | 3 | 1 | 1 | 1 | 0 | 0 | 0 | 0 | 0 | 0 | 0 | 296 | 322 | Oxidation (M) |
| K.VTMQNLNDRLASYLDKVR.A | N | 58.29 | 2135.1157 | 18 | 0.2 | 534.7863 | 4 | 70.04 | 5 | F5:32678 | 29102019\_RID\_1313\_NaNaPb\_F4.raw |  |  |  |  | 2.61E5 |  |  |  |  |  | 1 | 0 | 0 | 0 | 0 | 1 | 0 | 0 | 0 | 0 | 0 | 148 | 165 |  |
| R.LAADDFR.L | N | 56.98 | 806.3922 | 7 | 0.4 | 404.2036 | 2 | 11.73 | 1 | F1:3258 | 29102019\_RID\_1313\_NaNaPb\_F1.raw | 1E5 | 2.78E5 | 1.15E5 |  |  | 1.11E5 | 5.12E4 |  | 2.99E4 |  | 6 | 1 | 1 | 1 | 0 | 0 | 1 | 1 | 0 | 1 | 0 | 229 | 235 |  |
| K.TIDDLKNQILNLTTDNANILLQIDNAR.L | Y | 56.67 | 3051.6201 | 27 | 0.3 | 1018.2143 | 3 | 51.34 | 6 | F6:20555 | 29102019\_RID\_1313\_NaNaPb\_F5.raw |  |  |  |  |  | 5.51E4 |  |  | 1.62E4 |  | 2 | 0 | 0 | 0 | 0 | 0 | 1 | 0 | 0 | 1 | 0 | 202 | 228 |  |
| Q.IQAQISALEEQLQQIR.A | Y | 56.43 | 1867.0162 | 16 | 0.8 | 934.5161 | 2 | 26.87 | 2 | F2:9288 | 29102019\_RID\_1313\_NaNaPb\_F10.raw |  | 9.31E3 |  |  |  |  |  |  |  |  | 1 | 0 | 1 | 0 | 0 | 0 | 0 | 0 | 0 | 0 | 0 | 407 | 422 |  |
| S.SGGYGGLGGFGGGSFR.G | Y | 56.26 | 1431.6531 | 16 | 0.9 | 716.8345 | 2 | 16.18 | 1 | F1:5250 | 29102019\_RID\_1313\_NaNaPb\_F1.raw | 1.62E4 |  |  |  |  | 0 |  |  |  |  | 2 | 1 | 0 | 0 | 0 | 0 | 1 | 0 | 0 | 0 | 0 | 71 | 86 |  |
| R.LKYENEVALR.Q | N | 56.16 | 1233.6716 | 10 | 0.4 | 412.2313 | 3 | 11.60 | 3 | F3:2419 | 29102019\_RID\_1313\_NaNaPb\_F2.raw | 4.18E4 | 5.96E4 | 2.34E4 |  |  |  |  |  |  |  | 3 | 1 | 1 | 1 | 0 | 0 | 0 | 0 | 0 | 0 | 0 | 236 | 245 |  |
| R.LASYLDK.V | N | 55.97 | 808.4330 | 7 | -0.9 | 405.2234 | 2 | 11.66 | 1 | F1:3180 | 29102019\_RID\_1313\_NaNaPb\_F1.raw | 9.27E4 |  | 3.47E5 |  |  | 0 | 5.32E4 |  |  |  | 4 | 1 | 0 | 1 | 0 | 0 | 1 | 1 | 0 | 0 | 0 | 157 | 163 |  |
| K.VTMQNLNDR.L | N | 54.91 | 1089.5237 | 9 | 0.9 | 545.7696 | 2 | 11.21 | 7 | F7:2094 | 29102019\_RID\_1313\_NaNaPb\_F6.raw |  |  | 4.29E4 |  |  |  | 6.38E4 |  |  |  | 2 | 0 | 0 | 1 | 0 | 0 | 0 | 1 | 0 | 0 | 0 | 148 | 156 |  |
| S.ALEEQLQQIR.A | N | 54.06 | 1226.6619 | 10 | 0.7 | 614.3386 | 2 | 12.24 | 1 | F1:3729 | 29102019\_RID\_1313\_NaNaPb\_F1.raw | 5.66E3 |  |  |  |  |  |  |  |  |  | 1 | 1 | 0 | 0 | 0 | 0 | 0 | 0 | 0 | 0 | 0 | 413 | 422 |  |
| R.NVSTGDVNVEM(+15.99)NAAPGVDLTQLLNNMR.S | N | 52.88 | 2887.3804 | 27 | -0.6 | 963.4668 | 3 | 39.95 | 9 | F9:15023 | 29102019\_RID\_1313\_NaNaPb\_F8.raw |  |  |  |  |  |  |  |  | 2.24E4 |  | 1 | 0 | 0 | 0 | 0 | 0 | 0 | 0 | 0 | 1 | 0 | 296 | 322 | Oxidation (M) |
| K.ADLEMQIESLTEELAYLK.K | N | 52.03 | 2095.0396 | 18 | 0.1 | 699.3539 | 3 | 60.65 | 9 | F9:20199 | 29102019\_RID\_1313\_NaNaPb\_F8.raw |  | 2.74E5 |  |  |  |  |  |  | 1.91E4 |  | 3 | 0 | 2 | 0 | 0 | 0 | 0 | 0 | 0 | 1 | 0 | 267 | 284 |  |
| N.ILLQIDNAR.L | N | 52.01 | 1054.6135 | 9 | 0.3 | 528.3142 | 2 | 12.45 | 6 | F6:3131 | 29102019\_RID\_1313\_NaNaPb\_F5.raw |  | 3.86E5 |  |  |  | 3.61E5 |  |  | 2.29E4 |  | 3 | 0 | 1 | 0 | 0 | 0 | 1 | 0 | 0 | 1 | 0 | 220 | 228 |  |
| R.NVQALEIELQSQLALKQSLEASLAETEGR.Y | Y | 51.45 | 3167.6672 | 29 | 3.8 | 1056.9004 | 3 | 85.56 | 5 | F5:45703 | 29102019\_RID\_1313\_NaNaPb\_F4.raw |  |  |  |  | 1.06E5 |  |  |  |  |  | 1 | 0 | 0 | 0 | 0 | 1 | 0 | 0 | 0 | 0 | 0 | 371 | 399 |  |
| G.SLGGGFSSGGFSGGSFSR.G | Y | 51.06 | 1649.7433 | 18 | -0.3 | 825.8787 | 2 | 12.90 | 6 | F6:3540 | 29102019\_RID\_1313\_NaNaPb\_F5.raw |  |  |  |  |  | 1.07E4 |  |  |  |  | 1 | 0 | 0 | 0 | 0 | 0 | 1 | 0 | 0 | 0 | 0 | 42 | 59 |  |
| K.SEITELR.R | N | 47.79 | 846.4447 | 7 | -0.6 | 424.2294 | 2 | 11.66 | 1 | F1:3181 | 29102019\_RID\_1313\_NaNaPb\_F1.raw | 8.46E4 |  | 8.23E4 |  |  | 0 |  |  |  |  | 3 | 1 | 0 | 1 | 0 | 0 | 1 | 0 | 0 | 0 | 0 | 363 | 369 |  |
| F.GGGGFGGGFGGGFGGDGGLLSGNEK.V | Y | 46.07 | 2113.9453 | 25 | 2.4 | 1057.9824 | 2 | 26.13 | 1 | F1:7738 | 29102019\_RID\_1313\_NaNaPb\_F1.raw | 1.14E4 |  |  |  |  |  |  |  |  |  | 1 | 1 | 0 | 0 | 0 | 0 | 0 | 0 | 0 | 0 | 0 | 123 | 147 |  |
| L.SQIQAQISALEEQLQQIR.A | Y | 45.92 | 2082.1069 | 18 | 0.8 | 695.0435 | 3 | 40.06 | 2 | F2:13200 | 29102019\_RID\_1313\_NaNaPb\_F10.raw |  | 6.75E4 |  |  |  |  |  |  |  |  | 1 | 0 | 1 | 0 | 0 | 0 | 0 | 0 | 0 | 0 | 0 | 405 | 422 |  |
| R.YC(+57.02)VQLSQIQAQ.I | Y | 45.89 | 1336.6445 | 11 | 0.9 | 669.3301 | 2 | 15.73 | 1 | F1:5118 | 29102019\_RID\_1313\_NaNaPb\_F1.raw | 3.45E5 | 5.37E5 |  |  |  |  | 5.36E4 |  |  |  | 3 | 1 | 1 | 0 | 0 | 0 | 0 | 1 | 0 | 0 | 0 | 400 | 410 | Carbamidomethylation |
| A.QISALEEQLQQIR.A | N | 45.27 | 1554.8365 | 13 | 0.4 | 778.4258 | 2 | 19.30 | 1 | F1:6055 | 29102019\_RID\_1313\_NaNaPb\_F1.raw | 9.79E3 |  |  |  |  |  |  |  |  |  | 1 | 1 | 0 | 0 | 0 | 0 | 0 | 0 | 0 | 0 | 0 | 410 | 422 |  |
| R.QSVEADINGLRR.V | N | 43.74 | 1356.7109 | 12 | 0.2 | 453.2444 | 3 | 11.54 | 6 | F6:2345 | 29102019\_RID\_1313\_NaNaPb\_F5.raw |  |  |  |  |  | 1.5E5 |  |  |  |  | 1 | 0 | 0 | 0 | 0 | 0 | 1 | 0 | 0 | 0 | 0 | 246 | 257 |  |
| R.SQYEQLAEQNRKDAEAWFNEK.S | Y | 43.66 | 2583.1990 | 21 | 3.0 | 862.0762 | 3 | 63.59 | 5 | F5:27130 | 29102019\_RID\_1313\_NaNaPb\_F4.raw |  |  |  |  | 0 |  |  |  |  |  | 1 | 0 | 0 | 0 | 0 | 1 | 0 | 0 | 0 | 0 | 0 | 323 | 343 |  |
| D.NANILLQIDNAR.L | N | 43.60 | 1353.7365 | 12 | 0.5 | 677.8759 | 2 | 18.80 | 1 | F1:5928 | 29102019\_RID\_1313\_NaNaPb\_F1.raw | 1.63E4 |  |  |  |  |  |  |  |  |  | 1 | 1 | 0 | 0 | 0 | 0 | 0 | 0 | 0 | 0 | 0 | 217 | 228 |  |
| F.SSGGFSGGSFSR.G | N | 42.87 | 1131.4945 | 12 | 0.3 | 566.7547 | 2 | 11.56 | 3 | F3:2383 | 29102019\_RID\_1313\_NaNaPb\_F2.raw |  |  | 1.99E4 |  |  |  |  |  |  |  | 1 | 0 | 0 | 1 | 0 | 0 | 0 | 0 | 0 | 0 | 0 | 48 | 59 |  |
| R.GSSGGGC(+57.02)FGGSSGGYGGLGGFGGGSFRG.S | Y | 42.72 | 2398.9985 | 28 | 0.5 | 800.6738 | 3 | 15.75 | 2 | F2:5100 | 29102019\_RID\_1313\_NaNaPb\_F10.raw |  | 3.64E5 |  |  |  |  |  |  |  |  | 1 | 0 | 1 | 0 | 0 | 0 | 0 | 0 | 0 | 0 | 0 | 60 | 87 | Carbamidomethylation |
| F.GGGFGGGFGGDGGLLSGNEK.V | Y | 42.39 | 1738.7910 | 20 | 2.2 | 870.4047 | 2 | 16.31 | 6 | F6:5586 | 29102019\_RID\_1313\_NaNaPb\_F5.raw |  |  |  |  |  | 3.49E4 |  |  |  |  | 1 | 0 | 0 | 0 | 0 | 0 | 1 | 0 | 0 | 0 | 0 | 128 | 147 |  |
| K.GSLGGGFSSGGFSGGSF.S | Y | 42.05 | 1463.6317 | 17 | 1.0 | 732.8239 | 2 | 25.00 | 2 | F2:8646 | 29102019\_RID\_1313\_NaNaPb\_F10.raw |  | 7.38E4 |  |  |  |  |  |  |  |  | 1 | 0 | 1 | 0 | 0 | 0 | 0 | 0 | 0 | 0 | 0 | 41 | 57 |  |
| total 48 peptides |
| --- |

P01391|3L21\_NAJKA

back to list

  

| Protein Coverage
| Supporting Peptides
|

Protein Coverage:

Supporting Peptides:

| Peptide | Uniq | -10lgP | Mass | Length | ppm | m/z | z | RT | Fraction | Scan | Source File | Area F1 | Area F10 | Area F2 | Area F3 | Area F4 | Area F5 | Area F6 | Area F7 | Area F8 | Area F9 | #Feature | #Feature F1 | #Feature F10 | #Feature F2 | #Feature F3 | #Feature F4 | #Feature F5 | #Feature F6 | #Feature F7 | #Feature F8 | #Feature F9 | Start | End | PTM |
| --- | --- | --- | --- | --- | --- | --- | --- | --- | --- | --- | --- | --- | --- | --- | --- | --- | --- | --- | --- | --- | --- | --- | --- | --- | --- | --- | --- | --- | --- | --- | --- | --- | --- | --- | --- |
| K.TGVDIQC(+57.02)C(+57.02)STDNC(+57.02)NPFPTR.K | Y | 119.46 | 2240.9248 | 19 | 0.3 | 1121.4700 | 2 | 12.04 | 6 | F6:2769 | 29102019\_RID\_1313\_NaNaPb\_F5.raw |  | 3.48E6 |  | 1.12E5 | 1.1E8 | 2.84E7 | 1.13E9 | 6.97E4 | 3.25E6 | 4.07E6 | 39 | 0 | 2 | 0 | 1 | 4 | 2 | 25 | 2 | 1 | 2 | 50 | 68 | Carbamidomethylation |
| R.C(+57.02)FITPDITSKDC(+57.02)PNGHVC(+57.02)YTK.T | N | 108.82 | 2512.1184 | 21 | -1.1 | 838.3792 | 3 | 11.64 | 7 | F7:2478 | 29102019\_RID\_1313\_NaNaPb\_F6.raw |  |  |  |  | 3.29E8 |  | 5.17E6 |  |  |  | 8 | 0 | 0 | 0 | 0 | 6 | 0 | 2 | 0 | 0 | 0 | 3 | 23 | Carbamidomethylation |
| R.VDLGC(+57.02)AATC(+57.02)PTVK.T | N | 106.40 | 1390.6584 | 13 | -2.6 | 696.3347 | 2 | 11.56 | 7 | F7:2411 | 29102019\_RID\_1313\_NaNaPb\_F6.raw |  | 4.11E5 |  |  | 8.35E7 | 5.37E6 | 2.51E8 | 1.46E4 |  | 5.65E5 | 23 | 0 | 1 | 0 | 0 | 2 | 1 | 17 | 1 | 0 | 1 | 37 | 49 | Carbamidomethylation |
| IRC(+57.02)FITPDITSKDC(+57.02)PNGHVC(+57.02)YTK.T | N | 99.90 | 2781.3037 | 23 | 1.5 | 696.3342 | 4 | 49.09 | 5 | F5:14964 | 29102019\_RID\_1313\_NaNaPb\_F4.raw |  |  |  |  | 1.1E9 |  |  |  |  |  | 8 | 0 | 0 | 0 | 0 | 8 | 0 | 0 | 0 | 0 | 0 | 1 | 23 | Carbamidomethylation |
| D.IQC(+57.02)C(+57.02)STDNC(+57.02)NPFPTR.K | Y | 98.84 | 1868.7604 | 15 | -2.4 | 935.3852 | 2 | 11.56 | 7 | F7:2394 | 29102019\_RID\_1313\_NaNaPb\_F6.raw |  |  | 1.11E5 | 2.41E4 |  |  | 2.17E6 |  |  |  | 3 | 0 | 0 | 1 | 1 | 0 | 0 | 1 | 0 | 0 | 0 | 54 | 68 | Carbamidomethylation |
| K.TGVDIQC(+57.02)C(+57.02)STDNC(+57.02)NPFPTRK.R | Y | 97.73 | 2369.0198 | 20 | -1.0 | 790.6797 | 3 | 11.58 | 7 | F7:2439 | 29102019\_RID\_1313\_NaNaPb\_F6.raw |  |  |  |  | 4.05E6 |  | 7.13E5 |  |  |  | 5 | 0 | 0 | 0 | 0 | 3 | 0 | 2 | 0 | 0 | 0 | 50 | 69 | Carbamidomethylation |
| V.DIQC(+57.02)C(+57.02)STDNC(+57.02)NPFPTR.K | Y | 97.69 | 1983.7874 | 16 | 0.5 | 992.9015 | 2 | 11.80 | 7 | F7:2637 | 29102019\_RID\_1313\_NaNaPb\_F6.raw |  |  |  | 4.91E5 |  |  | 6.6E5 |  |  |  | 2 | 0 | 0 | 0 | 1 | 0 | 0 | 1 | 0 | 0 | 0 | 53 | 68 | Carbamidomethylation |
| Q.C(+57.02)C(+57.02)STDNC(+57.02)NPFPTR.K | N | 95.57 | 1627.6178 | 13 | 0.5 | 814.8166 | 2 | 11.44 | 7 | F7:2301 | 29102019\_RID\_1313\_NaNaPb\_F6.raw |  |  |  |  |  |  | 8.42E6 |  |  |  | 3 | 0 | 0 | 0 | 0 | 0 | 0 | 3 | 0 | 0 | 0 | 56 | 68 | Carbamidomethylation |
| K.RVDLGC(+57.02)AATC(+57.02)PTVK.T | N | 88.57 | 1546.7595 | 14 | -0.4 | 516.5936 | 3 | 11.29 | 7 | F7:2135 | 29102019\_RID\_1313\_NaNaPb\_F6.raw |  |  |  |  | 1.37E7 |  | 1.8E6 |  |  |  | 3 | 0 | 0 | 0 | 0 | 1 | 0 | 2 | 0 | 0 | 0 | 36 | 49 | Carbamidomethylation |
| C.STDNC(+57.02)NPFPTR.K | N | 87.32 | 1307.5564 | 11 | -1.0 | 654.7848 | 2 | 11.42 | 7 | F7:2239 | 29102019\_RID\_1313\_NaNaPb\_F6.raw |  | 1.27E5 | 4.52E4 |  |  | 4.03E5 | 6.19E7 |  |  | 2.64E4 | 7 | 0 | 1 | 1 | 0 | 0 | 1 | 3 | 0 | 0 | 1 | 58 | 68 | Carbamidomethylation |
| T.GVDIQC(+57.02)C(+57.02)STDNC(+57.02)NPFPTR.K | Y | 86.75 | 2139.8772 | 18 | 0.6 | 1070.9465 | 2 | 11.89 | 7 | F7:2748 | 29102019\_RID\_1313\_NaNaPb\_F6.raw |  |  |  | 6.25E4 |  |  | 1.31E5 |  |  |  | 2 | 0 | 0 | 0 | 1 | 0 | 0 | 1 | 0 | 0 | 0 | 51 | 68 | Carbamidomethylation |
| C.C(+57.02)STDNC(+57.02)NPFPTR.K | N | 86.29 | 1467.5872 | 12 | -1.6 | 734.7997 | 2 | 11.42 | 7 | F7:2280 | 29102019\_RID\_1313\_NaNaPb\_F6.raw |  |  | 6.23E4 |  |  |  | 2.57E6 |  |  |  | 2 | 0 | 0 | 1 | 0 | 0 | 0 | 1 | 0 | 0 | 0 | 57 | 68 | Carbamidomethylation |
| K.TGVDIQC(+57.02)C(+57.02)STDNC(+57.02)NPFPTRKRP | Y | 86.07 | 2622.1738 | 22 | -0.9 | 875.0644 | 3 | 11.42 | 7 | F7:2283 | 29102019\_RID\_1313\_NaNaPb\_F6.raw |  |  |  |  | 2.71E7 |  | 1.73E6 |  |  |  | 7 | 0 | 0 | 0 | 0 | 5 | 0 | 2 | 0 | 0 | 0 | 50 | 71 | Carbamidomethylation |
| K.DC(+57.02)PNGHVC(+57.02)YTK.T | N | 84.60 | 1349.5493 | 11 | -0.6 | 675.7816 | 2 | 10.69 | 7 | F7:1588 | 29102019\_RID\_1313\_NaNaPb\_F6.raw |  |  |  |  |  |  | 0 |  |  |  | 2 | 0 | 0 | 0 | 0 | 0 | 0 | 2 | 0 | 0 | 0 | 13 | 23 | Carbamidomethylation |
| R.C(+57.02)FITPDITSK.D | N | 83.01 | 1180.5798 | 10 | 0.0 | 591.2972 | 2 | 13.12 | 7 | F7:3684 | 29102019\_RID\_1313\_NaNaPb\_F6.raw |  | 1.15E6 |  | 4.39E4 | 7.16E7 |  | 4.95E8 | 7.19E4 | 1.97E6 | 1.33E6 | 28 | 0 | 1 | 0 | 1 | 1 | 0 | 22 | 1 | 1 | 1 | 3 | 12 | Carbamidomethylation |
| G.VDIQC(+57.02)C(+57.02)STDNC(+57.02)NPFPTR.K | Y | 76.50 | 2082.8557 | 17 | 1.3 | 1042.4365 | 2 | 11.87 | 7 | F7:2705 | 29102019\_RID\_1313\_NaNaPb\_F6.raw |  |  |  | 7.54E4 |  |  | 1.19E5 |  |  |  | 2 | 0 | 0 | 0 | 1 | 0 | 0 | 1 | 0 | 0 | 0 | 52 | 68 | Carbamidomethylation |
| C.PNGHVC(+57.02)YTK.T | N | 76.22 | 1074.4917 | 9 | -2.6 | 538.2517 | 2 | 10.69 | 7 | F7:1590 | 29102019\_RID\_1313\_NaNaPb\_F6.raw |  |  |  |  |  |  | 2.55E6 |  |  |  | 1 | 0 | 0 | 0 | 0 | 0 | 0 | 1 | 0 | 0 | 0 | 15 | 23 | Carbamidomethylation |
| K.TGVDIQC(+57.02)C(+57.02)STDNC(+57.02)N.P | Y | 73.84 | 1642.6022 | 14 | 0.6 | 822.3088 | 2 | 11.51 | 7 | F7:2373 | 29102019\_RID\_1313\_NaNaPb\_F6.raw |  |  |  |  |  |  | 2.74E5 |  |  |  | 1 | 0 | 0 | 0 | 0 | 0 | 0 | 1 | 0 | 0 | 0 | 50 | 63 | Carbamidomethylation |
| IRC(+57.02)FITPDITSK.D | N | 72.29 | 1449.7650 | 12 | 0.4 | 725.8901 | 2 | 54.58 | 5 | F5:19303 | 29102019\_RID\_1313\_NaNaPb\_F4.raw |  |  |  |  | 2.93E8 |  | 3.64E5 |  |  |  | 6 | 0 | 0 | 0 | 0 | 5 | 0 | 1 | 0 | 0 | 0 | 1 | 12 | Carbamidomethylation |
| S.TDNC(+57.02)NPFPTR.K | N | 67.39 | 1220.5244 | 10 | -1.8 | 611.2684 | 2 | 11.42 | 7 | F7:2303 | 29102019\_RID\_1313\_NaNaPb\_F6.raw |  |  |  |  |  |  | 1.46E5 |  |  |  | 1 | 0 | 0 | 0 | 0 | 0 | 0 | 1 | 0 | 0 | 0 | 59 | 68 | Carbamidomethylation |
| T.DNC(+57.02)NPFPTR.K | N | 61.51 | 1119.4767 | 9 | -0.6 | 560.7453 | 2 | 11.77 | 1 | F1:3298 | 29102019\_RID\_1313\_NaNaPb\_F1.raw | 1.81E4 |  |  |  |  |  |  |  |  |  | 1 | 1 | 0 | 0 | 0 | 0 | 0 | 0 | 0 | 0 | 0 | 60 | 68 | Carbamidomethylation |
| K.RVDLGC(+57.02)AATC(+57.02)PT.V | N | 61.14 | 1319.5962 | 12 | -1.8 | 660.8042 | 2 | 11.42 | 7 | F7:2282 | 29102019\_RID\_1313\_NaNaPb\_F6.raw |  |  |  |  |  |  | 5.44E5 |  |  |  | 1 | 0 | 0 | 0 | 0 | 0 | 0 | 1 | 0 | 0 | 0 | 36 | 47 | Carbamidomethylation |
| R.VDLGC(+57.02)AATC(+57.02)PT.V | N | 59.60 | 1163.4951 | 11 | 0.0 | 582.7548 | 2 | 11.91 | 7 | F7:2756 | 29102019\_RID\_1313\_NaNaPb\_F6.raw |  |  |  |  |  |  | 8.06E5 |  |  |  | 1 | 0 | 0 | 0 | 0 | 0 | 0 | 1 | 0 | 0 | 0 | 37 | 47 | Carbamidomethylation |
| D.LGC(+57.02)AATC(+57.02)PTVK.T | N | 59.20 | 1176.5631 | 11 | -2.3 | 589.2875 | 2 | 11.07 | 7 | F7:1951 | 29102019\_RID\_1313\_NaNaPb\_F6.raw |  |  |  |  |  |  | 1.66E5 |  |  |  | 1 | 0 | 0 | 0 | 0 | 0 | 0 | 1 | 0 | 0 | 0 | 39 | 49 | Carbamidomethylation |
| R.VDLGC(+57.02)AATC(+57.02)PTV.K | N | 57.22 | 1262.5635 | 12 | -0.8 | 632.2885 | 2 | 13.91 | 7 | F7:4209 | 29102019\_RID\_1313\_NaNaPb\_F6.raw |  |  |  |  |  |  | 0 |  |  |  | 1 | 0 | 0 | 0 | 0 | 0 | 0 | 1 | 0 | 0 | 0 | 37 | 48 | Carbamidomethylation |
| K.DC(+57.02)PNGHVC(+57.02)Y.T | N | 54.83 | 1120.4066 | 9 | 0.8 | 561.2111 | 2 | 11.17 | 7 | F7:2028 | 29102019\_RID\_1313\_NaNaPb\_F6.raw |  |  |  |  |  |  | 5.79E6 |  |  |  | 1 | 0 | 0 | 0 | 0 | 0 | 0 | 1 | 0 | 0 | 0 | 13 | 21 | Carbamidomethylation |
| R.VDLGC(+57.02)AATC(+57.02)P.T | N | 54.00 | 1062.4474 | 10 | 1.1 | 532.2316 | 2 | 12.13 | 7 | F7:2931 | 29102019\_RID\_1313\_NaNaPb\_F6.raw |  |  |  |  |  |  | 1.03E5 |  |  |  | 1 | 0 | 0 | 0 | 0 | 0 | 0 | 1 | 0 | 0 | 0 | 37 | 46 | Carbamidomethylation |
| K.TGVDIQC(+57.02)C(+57.02).S | N | 53.10 | 951.3790 | 8 | -0.3 | 476.6967 | 2 | 11.71 | 7 | F7:2547 | 29102019\_RID\_1313\_NaNaPb\_F6.raw |  |  |  |  |  |  | 1.32E6 |  |  |  | 1 | 0 | 0 | 0 | 0 | 0 | 0 | 1 | 0 | 0 | 0 | 50 | 57 | Carbamidomethylation |
| K.TGVDIQC(+57.02)C(+57.02)STDNC(+57.02)NPFPT.R | Y | 50.92 | 2084.8237 | 18 | 1.0 | 1043.4202 | 2 | 17.05 | 7 | F7:6007 | 29102019\_RID\_1313\_NaNaPb\_F6.raw |  |  |  |  |  |  | 7.94E6 |  |  |  | 1 | 0 | 0 | 0 | 0 | 0 | 0 | 1 | 0 | 0 | 0 | 50 | 67 | Carbamidomethylation |
| IRC(+57.02)FITPDITSKD.C | N | 46.76 | 1564.7919 | 13 | 0.1 | 783.4033 | 2 | 55.56 | 5 | F5:20289 | 29102019\_RID\_1313\_NaNaPb\_F4.raw |  |  |  |  | 7.27E6 |  |  |  |  |  | 2 | 0 | 0 | 0 | 0 | 2 | 0 | 0 | 0 | 0 | 0 | 1 | 13 | Carbamidomethylation |
| R.C(+57.02)FITPDITS.K | N | 46.63 | 1052.4849 | 9 | 0.1 | 527.2498 | 2 | 20.20 | 7 | F7:7324 | 29102019\_RID\_1313\_NaNaPb\_F6.raw |  |  |  |  |  |  | 2.43E4 |  |  |  | 1 | 0 | 0 | 0 | 0 | 0 | 0 | 1 | 0 | 0 | 0 | 3 | 11 | Carbamidomethylation |
| C.FITPDITSK.D | N | 42.13 | 1020.5491 | 9 | -2.4 | 511.2806 | 2 | 12.04 | 7 | F7:2836 | 29102019\_RID\_1313\_NaNaPb\_F6.raw |  |  |  |  |  |  | 2.76E5 |  |  |  | 1 | 0 | 0 | 0 | 0 | 0 | 0 | 1 | 0 | 0 | 0 | 4 | 12 |  |
| total 32 peptides |
| --- |

P35908|K22E\_HUMAN

back to list

  

| Protein Coverage
| Supporting Peptides
|

Protein Coverage:

Supporting Peptides:

| Peptide | Uniq | -10lgP | Mass | Length | ppm | m/z | z | RT | Fraction | Scan | Source File | Area F1 | Area F10 | Area F2 | Area F3 | Area F4 | Area F5 | Area F6 | Area F7 | Area F8 | Area F9 | #Feature | #Feature F1 | #Feature F10 | #Feature F2 | #Feature F3 | #Feature F4 | #Feature F5 | #Feature F6 | #Feature F7 | #Feature F8 | #Feature F9 | Start | End | PTM |
| --- | --- | --- | --- | --- | --- | --- | --- | --- | --- | --- | --- | --- | --- | --- | --- | --- | --- | --- | --- | --- | --- | --- | --- | --- | --- | --- | --- | --- | --- | --- | --- | --- | --- | --- | --- |
| R.GGSGGGGSISGGGYGSGGGSGGR.Y | N | 120.05 | 1740.7411 | 23 | -1.0 | 871.3770 | 2 | 10.79 | 7 | F7:1681 | 29102019\_RID\_1313\_NaNaPb\_F6.raw |  | 4.05E4 | 2.41E4 |  |  | 5.8E4 | 0 |  | 8.75E3 |  | 5 | 0 | 1 | 1 | 0 | 0 | 1 | 1 | 0 | 1 | 0 | 550 | 572 |  |
| R.GFSSGSAVVSGGSR.R | N | 104.38 | 1253.6000 | 14 | 0.4 | 627.8075 | 2 | 11.44 | 1 | F1:2975 | 29102019\_RID\_1313\_NaNaPb\_F1.raw | 5.27E4 | 1.09E5 | 4.88E4 |  |  | 1.06E5 | 2.54E4 |  | 1.75E4 |  | 6 | 1 | 1 | 1 | 0 | 0 | 1 | 1 | 0 | 1 | 0 | 21 | 34 |  |
| R.FLEQQNQVLQTK.W | N | 97.49 | 1474.7780 | 12 | 0.1 | 738.3963 | 2 | 12.00 | 1 | F1:3480 | 29102019\_RID\_1313\_NaNaPb\_F1.raw | 3.66E6 | 4.06E6 | 5.53E6 | 1.56E5 |  | 6.8E6 |  |  | 2.74E5 |  | 6 | 1 | 1 | 1 | 1 | 0 | 1 | 0 | 0 | 1 | 0 | 198 | 209 |  |
| K.NVQDAIADAEQR.G | N | 93.38 | 1328.6321 | 12 | 0.7 | 665.3238 | 2 | 11.87 | 1 | F1:3390 | 29102019\_RID\_1313\_NaNaPb\_F1.raw | 2.92E5 |  | 1.03E5 |  |  | 7.26E5 |  |  |  |  | 3 | 1 | 0 | 1 | 0 | 0 | 1 | 0 | 0 | 0 | 0 | 419 | 430 |  |
| R.FGGFGGPGGVGGLGGPGGFGPGGYPGGIHEVSVNQSLLQPLNVK.V | N | 88.68 | 4091.0652 | 44 | 0.0 | 1023.7736 | 4 | 37.76 | 7 | F7:12901 | 29102019\_RID\_1313\_NaNaPb\_F6.raw |  |  |  |  |  |  | 1.14E5 |  |  |  | 2 | 0 | 0 | 0 | 0 | 0 | 0 | 2 | 0 | 0 | 0 | 123 | 166 |  |
| K.LNDLEEALQQAK.E | N | 86.08 | 1370.7041 | 12 | -0.2 | 686.3592 | 2 | 13.13 | 2 | F2:3872 | 29102019\_RID\_1313\_NaNaPb\_F10.raw | 1.12E6 | 1.19E6 | 3.43E5 |  |  | 3.47E6 | 1.54E5 |  | 1.37E5 |  | 8 | 1 | 2 | 1 | 0 | 0 | 2 | 1 | 0 | 1 | 0 | 442 | 453 |  |
| R.HGGGGGGFGGGGFGSR.S | N | 85.14 | 1319.5754 | 16 | -0.3 | 440.8656 | 3 | 39.50 | 5 | F5:8015 | 29102019\_RID\_1313\_NaNaPb\_F4.raw |  | 1.03E4 |  |  | 5.51E4 |  |  |  |  |  | 2 | 0 | 1 | 0 | 0 | 1 | 0 | 0 | 0 | 0 | 0 | 46 | 61 |  |
| R.TAAENDFVTLK.K | N | 84.15 | 1207.6084 | 11 | 0.9 | 604.8120 | 2 | 11.99 | 6 | F6:2757 | 29102019\_RID\_1313\_NaNaPb\_F5.raw | 4.9E5 | 4.77E5 | 2E5 | 5.17E4 |  | 6.82E5 |  |  |  | 1.76E4 | 6 | 1 | 1 | 1 | 1 | 0 | 1 | 0 | 0 | 0 | 1 | 276 | 286 |  |
| R.TSQNSELNNMQDLVEDYKK.K | N | 81.35 | 2255.0376 | 19 | 0.6 | 752.6870 | 3 | 12.39 | 2 | F2:3282 | 29102019\_RID\_1313\_NaNaPb\_F10.raw |  | 5.14E4 |  |  |  |  |  |  |  |  | 1 | 0 | 1 | 0 | 0 | 0 | 0 | 0 | 0 | 0 | 0 | 248 | 266 |  |
| K.VDLLNQEIEFLK.V | N | 80.14 | 1459.7922 | 12 | 0.7 | 730.9039 | 2 | 32.14 | 2 | F2:10898 | 29102019\_RID\_1313\_NaNaPb\_F10.raw | 7.79E5 | 1.69E6 | 2.89E5 | 9.62E4 |  | 2.42E6 | 2.3E5 |  | 1.98E5 |  | 9 | 2 | 1 | 1 | 1 | 0 | 2 | 1 | 0 | 1 | 0 | 303 | 314 |  |
| R.NLDLDSIIAEVK.A | N | 79.85 | 1328.7188 | 12 | 0.3 | 665.3669 | 2 | 34.39 | 9 | F9:13181 | 29102019\_RID\_1313\_NaNaPb\_F8.raw | 6.3E5 | 2.03E6 | 4.86E5 | 7.73E4 |  | 1.95E6 | 1.33E6 |  | 3.92E5 | 1.4E5 | 9 | 2 | 1 | 1 | 1 | 0 | 1 | 1 | 0 | 1 | 1 | 342 | 353 |  |
| K.TLNNKFASFIDKVR.F | N | 77.96 | 1651.9045 | 14 | 0.2 | 413.9835 | 4 | 59.92 | 5 | F5:23986 | 29102019\_RID\_1313\_NaNaPb\_F4.raw |  |  |  |  | 1.32E6 |  |  |  |  |  | 2 | 0 | 0 | 0 | 0 | 2 | 0 | 0 | 0 | 0 | 0 | 184 | 197 |  |
| K.AQYEEIAQR.S | N | 75.95 | 1106.5356 | 9 | -0.6 | 554.2748 | 2 | 11.33 | 1 | F1:2875 | 29102019\_RID\_1313\_NaNaPb\_F1.raw | 7.34E4 | 1.94E5 | 7.33E4 |  |  | 8.43E4 | 1.12E5 |  |  |  | 5 | 1 | 1 | 1 | 0 | 0 | 1 | 1 | 0 | 0 | 0 | 354 | 362 |  |
| K.DVDNAYMIK.V | N | 74.54 | 1067.4957 | 9 | -0.5 | 534.7549 | 2 | 12.36 | 1 | F1:3829 | 29102019\_RID\_1313\_NaNaPb\_F1.raw | 2.65E4 |  |  |  |  |  |  |  |  |  | 1 | 1 | 0 | 0 | 0 | 0 | 0 | 0 | 0 | 0 | 0 | 288 | 296 |  |
| R.STSSFSC(+57.02)LSR.H | N | 72.09 | 1130.5026 | 10 | 0.4 | 566.2588 | 2 | 11.68 | 1 | F1:3204 | 29102019\_RID\_1313\_NaNaPb\_F1.raw | 4.31E4 |  |  |  |  |  |  |  |  |  | 1 | 1 | 0 | 0 | 0 | 0 | 0 | 0 | 0 | 0 | 0 | 36 | 45 | Carbamidomethylation |
| K.LALDVEIATYR.K | N | 72.02 | 1262.6870 | 11 | 1.0 | 632.3514 | 2 | 18.69 | 2 | F2:6620 | 29102019\_RID\_1313\_NaNaPb\_F10.raw | 1.06E5 | 5.46E5 | 3.1E4 | 1.25E4 |  | 1.24E6 | 1.83E5 |  |  |  | 8 | 1 | 2 | 1 | 1 | 0 | 2 | 1 | 0 | 0 | 0 | 471 | 481 |  |
| S.VAGGGGGFGAAGGFGGR.G | N | 71.42 | 1350.6428 | 17 | 0.4 | 676.3290 | 2 | 12.08 | 1 | F1:3584 | 29102019\_RID\_1313\_NaNaPb\_F1.raw | 1.32E4 |  |  |  |  |  |  |  |  |  | 1 | 1 | 0 | 0 | 0 | 0 | 0 | 0 | 0 | 0 | 0 | 76 | 92 |  |
| R.YLDGLTAER.T | N | 67.54 | 1036.5189 | 9 | -0.7 | 519.2664 | 2 | 11.87 | 6 | F6:2624 | 29102019\_RID\_1313\_NaNaPb\_F5.raw |  | 6.11E5 |  |  |  | 6.51E5 |  |  | 3.19E4 |  | 3 | 0 | 1 | 0 | 0 | 0 | 1 | 0 | 0 | 1 | 0 | 239 | 247 |  |
| R.GSSSGGGYSSGSSSYGSGGR.Q | N | 66.04 | 1739.6982 | 20 | 1.2 | 870.8574 | 2 | 10.89 | 3 | F3:1761 | 29102019\_RID\_1313\_NaNaPb\_F2.raw |  |  | 1.94E4 |  |  |  |  |  |  |  | 1 | 0 | 0 | 1 | 0 | 0 | 0 | 0 | 0 | 0 | 0 | 525 | 544 |  |
| K.VLYDAEISQIHQSVTDTNVILSMDNSR.N | N | 60.56 | 3047.4871 | 27 | 0.2 | 1016.8365 | 3 | 26.70 | 2 | F2:9278 | 29102019\_RID\_1313\_NaNaPb\_F10.raw |  | 1.39E5 |  |  |  |  | 3.63E4 |  |  |  | 2 | 0 | 1 | 0 | 0 | 0 | 0 | 1 | 0 | 0 | 0 | 315 | 341 |  |
| R.GGGFGGGSSFGGGSGFSGGGFGGGGFGGGR.F | Y | 59.00 | 2398.0110 | 30 | -1.3 | 800.3433 | 3 | 18.02 | 6 | F6:6560 | 29102019\_RID\_1313\_NaNaPb\_F5.raw |  |  |  |  |  | 2.79E5 |  |  |  |  | 1 | 0 | 0 | 0 | 0 | 0 | 1 | 0 | 0 | 0 | 0 | 93 | 122 |  |
| K.SISISVAGGGGGFGAAGGFGGR.G | N | 58.52 | 1837.9070 | 22 | -0.3 | 919.9604 | 2 | 17.88 | 6 | F6:7072 | 29102019\_RID\_1313\_NaNaPb\_F5.raw |  | 1.73E4 |  |  |  | 3.51E5 | 9.75E3 |  |  |  | 4 | 0 | 1 | 0 | 0 | 0 | 2 | 1 | 0 | 0 | 0 | 71 | 92 |  |
| K.IEISELNR.V | N | 55.32 | 972.5240 | 8 | 0.4 | 487.2695 | 2 | 11.76 | 7 | F7:2595 | 29102019\_RID\_1313\_NaNaPb\_F6.raw | 9.39E5 |  | 2.11E6 | 7.37E4 |  | 2.16E6 | 2.78E5 |  | 1.23E5 |  | 6 | 1 | 0 | 1 | 1 | 0 | 1 | 1 | 0 | 1 | 0 | 394 | 401 |  |
| K.YEELQVTVGR.H | N | 54.26 | 1192.6088 | 10 | 0.8 | 597.3121 | 2 | 11.77 | 2 | F2:2740 | 29102019\_RID\_1313\_NaNaPb\_F10.raw |  | 4.09E5 |  |  |  |  |  |  |  |  | 1 | 0 | 1 | 0 | 0 | 0 | 0 | 0 | 0 | 0 | 0 | 375 | 384 |  |
| K.LLEGEEC(+57.02)R.M | N | 53.11 | 1004.4597 | 8 | 0.4 | 503.2373 | 2 | 11.25 | 3 | F3:2079 | 29102019\_RID\_1313\_NaNaPb\_F2.raw |  |  | 3.26E4 |  |  |  |  |  |  |  | 1 | 0 | 0 | 1 | 0 | 0 | 0 | 0 | 0 | 0 | 0 | 483 | 490 | Carbamidomethylation |
| R.DYQELMNVK.L | N | 52.90 | 1138.5328 | 9 | 1.2 | 570.2744 | 2 | 13.43 | 1 | F1:4343 | 29102019\_RID\_1313\_NaNaPb\_F1.raw | 6.15E4 | 5.37E4 |  |  |  |  |  |  |  |  | 2 | 1 | 1 | 0 | 0 | 0 | 0 | 0 | 0 | 0 | 0 | 462 | 470 |  |
| R.SLVGLGGTK.S | N | 52.29 | 830.4861 | 9 | 0.0 | 416.2504 | 2 | 48.25 | 5 | F5:14296 | 29102019\_RID\_1313\_NaNaPb\_F4.raw |  |  |  |  | 1.17E5 |  |  |  |  |  | 1 | 0 | 0 | 0 | 0 | 1 | 0 | 0 | 0 | 0 | 0 | 62 | 70 |  |
| R.SKEEAEALYHSK.Y | N | 51.94 | 1390.6729 | 12 | 0.0 | 464.5649 | 3 | 10.96 | 3 | F3:1822 | 29102019\_RID\_1313\_NaNaPb\_F2.raw | 1.34E4 |  | 9.16E3 |  |  |  |  |  |  |  | 2 | 1 | 0 | 1 | 0 | 0 | 0 | 0 | 0 | 0 | 0 | 363 | 374 |  |
| K.VDPEIQNVK.A | N | 50.57 | 1040.5502 | 9 | -1.0 | 521.2819 | 2 | 11.44 | 1 | F1:2994 | 29102019\_RID\_1313\_NaNaPb\_F1.raw | 7.93E4 | 1.34E5 | 5.51E4 |  |  | 3.82E4 |  |  |  |  | 4 | 1 | 1 | 1 | 0 | 0 | 1 | 0 | 0 | 0 | 0 | 167 | 175 |  |
| R.PINLEPIFQGYIDSLK.R | N | 50.42 | 1845.9875 | 16 | 1.0 | 924.0020 | 2 | 49.12 | 6 | F6:19854 | 29102019\_RID\_1313\_NaNaPb\_F5.raw |  | 8.17E4 |  |  |  | 4.75E5 |  |  |  |  | 2 | 0 | 1 | 0 | 0 | 0 | 1 | 0 | 0 | 0 | 0 | 222 | 237 |  |
| R.FGGFGGPGGVGGLGGPGGFGPGGYPGGIHEVSVN.Q | N | 48.49 | 2970.4048 | 34 | 0.2 | 991.1424 | 3 | 31.73 | 2 | F2:10809 | 29102019\_RID\_1313\_NaNaPb\_F10.raw |  | 4.57E4 |  |  |  |  |  |  |  |  | 1 | 0 | 1 | 0 | 0 | 0 | 0 | 0 | 0 | 0 | 0 | 123 | 156 |  |
| K.FASFIDK.V | N | 46.57 | 826.4225 | 7 | -0.3 | 414.2184 | 2 | 11.89 | 2 | F2:2810 | 29102019\_RID\_1313\_NaNaPb\_F10.raw |  | 1.96E5 |  |  |  | 3.22E5 |  |  | 0 |  | 3 | 0 | 1 | 0 | 0 | 0 | 1 | 0 | 0 | 1 | 0 | 189 | 195 |  |
| R.GGGFGGGSSFGGGSGF.S | Y | 44.39 | 1290.5265 | 16 | 0.5 | 646.2709 | 2 | 20.80 | 1 | F1:6387 | 29102019\_RID\_1313\_NaNaPb\_F1.raw | 2.68E4 |  |  |  |  |  |  |  |  |  | 1 | 1 | 0 | 0 | 0 | 0 | 0 | 0 | 0 | 0 | 0 | 93 | 108 |  |
| M.NVGTRPINLEPIFQGYIDSLK.R | N | 43.20 | 2373.2693 | 21 | 1.3 | 792.0981 | 3 | 36.11 | 2 | F2:12041 | 29102019\_RID\_1313\_NaNaPb\_F10.raw |  | 7.29E4 |  |  |  |  |  |  |  |  | 1 | 0 | 1 | 0 | 0 | 0 | 0 | 0 | 0 | 0 | 0 | 217 | 237 |  |
| total 34 peptides |
| --- |

#CONTAM#K22E\_HUMAN|

back to list

  

| Protein Coverage
| Supporting Peptides
|

Protein Coverage:

Supporting Peptides:

| Peptide | Uniq | -10lgP | Mass | Length | ppm | m/z | z | RT | Fraction | Scan | Source File | Area F1 | Area F10 | Area F2 | Area F3 | Area F4 | Area F5 | Area F6 | Area F7 | Area F8 | Area F9 | #Feature | #Feature F1 | #Feature F10 | #Feature F2 | #Feature F3 | #Feature F4 | #Feature F5 | #Feature F6 | #Feature F7 | #Feature F8 | #Feature F9 | Start | End | PTM |
| --- | --- | --- | --- | --- | --- | --- | --- | --- | --- | --- | --- | --- | --- | --- | --- | --- | --- | --- | --- | --- | --- | --- | --- | --- | --- | --- | --- | --- | --- | --- | --- | --- | --- | --- | --- |
| R.GGSGGGGSISGGGYGSGGGSGGR.Y | N | 120.05 | 1740.7411 | 23 | -1.0 | 871.3770 | 2 | 10.79 | 7 | F7:1681 | 29102019\_RID\_1313\_NaNaPb\_F6.raw |  | 4.05E4 | 2.41E4 |  |  | 5.8E4 | 0 |  | 8.75E3 |  | 5 | 0 | 1 | 1 | 0 | 0 | 1 | 1 | 0 | 1 | 0 | 556 | 578 |  |
| R.GFSSGSAVVSGGSR.R | N | 104.38 | 1253.6000 | 14 | 0.4 | 627.8075 | 2 | 11.44 | 1 | F1:2975 | 29102019\_RID\_1313\_NaNaPb\_F1.raw | 5.27E4 | 1.09E5 | 4.88E4 |  |  | 1.06E5 | 2.54E4 |  | 1.75E4 |  | 6 | 1 | 1 | 1 | 0 | 0 | 1 | 1 | 0 | 1 | 0 | 21 | 34 |  |
| R.FLEQQNQVLQTK.W | N | 97.49 | 1474.7780 | 12 | 0.1 | 738.3963 | 2 | 12.00 | 1 | F1:3480 | 29102019\_RID\_1313\_NaNaPb\_F1.raw | 3.66E6 | 4.06E6 | 5.53E6 | 1.56E5 |  | 6.8E6 |  |  | 2.74E5 |  | 6 | 1 | 1 | 1 | 1 | 0 | 1 | 0 | 0 | 1 | 0 | 204 | 215 |  |
| K.NVQDAIADAEQR.G | N | 93.38 | 1328.6321 | 12 | 0.7 | 665.3238 | 2 | 11.87 | 1 | F1:3390 | 29102019\_RID\_1313\_NaNaPb\_F1.raw | 2.92E5 |  | 1.03E5 |  |  | 7.26E5 |  |  |  |  | 3 | 1 | 0 | 1 | 0 | 0 | 1 | 0 | 0 | 0 | 0 | 425 | 436 |  |
| R.FGGFGGPGGVGGLGGPGGFGPGGYPGGIHEVSVNQSLLQPLNVK.V | N | 88.68 | 4091.0652 | 44 | 0.0 | 1023.7736 | 4 | 37.76 | 7 | F7:12901 | 29102019\_RID\_1313\_NaNaPb\_F6.raw |  |  |  |  |  |  | 1.14E5 |  |  |  | 2 | 0 | 0 | 0 | 0 | 0 | 0 | 2 | 0 | 0 | 0 | 129 | 172 |  |
| K.LNDLEEALQQAK.E | N | 86.08 | 1370.7041 | 12 | -0.2 | 686.3592 | 2 | 13.13 | 2 | F2:3872 | 29102019\_RID\_1313\_NaNaPb\_F10.raw | 1.12E6 | 1.19E6 | 3.43E5 |  |  | 3.47E6 | 1.54E5 |  | 1.37E5 |  | 8 | 1 | 2 | 1 | 0 | 0 | 2 | 1 | 0 | 1 | 0 | 448 | 459 |  |
| R.HGGGGGGFGGGGFGSR.S | N | 85.14 | 1319.5754 | 16 | -0.3 | 440.8656 | 3 | 39.50 | 5 | F5:8015 | 29102019\_RID\_1313\_NaNaPb\_F4.raw |  | 1.03E4 |  |  | 5.51E4 |  |  |  |  |  | 2 | 0 | 1 | 0 | 0 | 1 | 0 | 0 | 0 | 0 | 0 | 46 | 61 |  |
| R.TAAENDFVTLK.K | N | 84.15 | 1207.6084 | 11 | 0.9 | 604.8120 | 2 | 11.99 | 6 | F6:2757 | 29102019\_RID\_1313\_NaNaPb\_F5.raw | 4.9E5 | 4.77E5 | 2E5 | 5.17E4 |  | 6.82E5 |  |  |  | 1.76E4 | 6 | 1 | 1 | 1 | 1 | 0 | 1 | 0 | 0 | 0 | 1 | 282 | 292 |  |
| R.TSQNSELNNMQDLVEDYKK.K | N | 81.35 | 2255.0376 | 19 | 0.6 | 752.6870 | 3 | 12.39 | 2 | F2:3282 | 29102019\_RID\_1313\_NaNaPb\_F10.raw |  | 5.14E4 |  |  |  |  |  |  |  |  | 1 | 0 | 1 | 0 | 0 | 0 | 0 | 0 | 0 | 0 | 0 | 254 | 272 |  |
| K.VDLLNQEIEFLK.V | N | 80.14 | 1459.7922 | 12 | 0.7 | 730.9039 | 2 | 32.14 | 2 | F2:10898 | 29102019\_RID\_1313\_NaNaPb\_F10.raw | 7.79E5 | 1.69E6 | 2.89E5 | 9.62E4 |  | 2.42E6 | 2.3E5 |  | 1.98E5 |  | 9 | 2 | 1 | 1 | 1 | 0 | 2 | 1 | 0 | 1 | 0 | 309 | 320 |  |
| R.NLDLDSIIAEVK.A | N | 79.85 | 1328.7188 | 12 | 0.3 | 665.3669 | 2 | 34.39 | 9 | F9:13181 | 29102019\_RID\_1313\_NaNaPb\_F8.raw | 6.3E5 | 2.03E6 | 4.86E5 | 7.73E4 |  | 1.95E6 | 1.33E6 |  | 3.92E5 | 1.4E5 | 9 | 2 | 1 | 1 | 1 | 0 | 1 | 1 | 0 | 1 | 1 | 348 | 359 |  |
| K.TLNNKFASFIDKVR.F | N | 77.96 | 1651.9045 | 14 | 0.2 | 413.9835 | 4 | 59.92 | 5 | F5:23986 | 29102019\_RID\_1313\_NaNaPb\_F4.raw |  |  |  |  | 1.32E6 |  |  |  |  |  | 2 | 0 | 0 | 0 | 0 | 2 | 0 | 0 | 0 | 0 | 0 | 190 | 203 |  |
| K.AQYEEIAQR.S | N | 75.95 | 1106.5356 | 9 | -0.6 | 554.2748 | 2 | 11.33 | 1 | F1:2875 | 29102019\_RID\_1313\_NaNaPb\_F1.raw | 7.34E4 | 1.94E5 | 7.33E4 |  |  | 8.43E4 | 1.12E5 |  |  |  | 5 | 1 | 1 | 1 | 0 | 0 | 1 | 1 | 0 | 0 | 0 | 360 | 368 |  |
| K.DVDNAYMIK.V | N | 74.54 | 1067.4957 | 9 | -0.5 | 534.7549 | 2 | 12.36 | 1 | F1:3829 | 29102019\_RID\_1313\_NaNaPb\_F1.raw | 2.65E4 |  |  |  |  |  |  |  |  |  | 1 | 1 | 0 | 0 | 0 | 0 | 0 | 0 | 0 | 0 | 0 | 294 | 302 |  |
| R.STSSFSC(+57.02)LSR.H | N | 72.09 | 1130.5026 | 10 | 0.4 | 566.2588 | 2 | 11.68 | 1 | F1:3204 | 29102019\_RID\_1313\_NaNaPb\_F1.raw | 4.31E4 |  |  |  |  |  |  |  |  |  | 1 | 1 | 0 | 0 | 0 | 0 | 0 | 0 | 0 | 0 | 0 | 36 | 45 | Carbamidomethylation |
| K.LALDVEIATYR.K | N | 72.02 | 1262.6870 | 11 | 1.0 | 632.3514 | 2 | 18.69 | 2 | F2:6620 | 29102019\_RID\_1313\_NaNaPb\_F10.raw | 1.06E5 | 5.46E5 | 3.1E4 | 1.25E4 |  | 1.24E6 | 1.83E5 |  |  |  | 8 | 1 | 2 | 1 | 1 | 0 | 2 | 1 | 0 | 0 | 0 | 477 | 487 |  |
| S.VAGGGGGFGAAGGFGGR.G | N | 71.42 | 1350.6428 | 17 | 0.4 | 676.3290 | 2 | 12.08 | 1 | F1:3584 | 29102019\_RID\_1313\_NaNaPb\_F1.raw | 1.32E4 |  |  |  |  |  |  |  |  |  | 1 | 1 | 0 | 0 | 0 | 0 | 0 | 0 | 0 | 0 | 0 | 76 | 92 |  |
| R.YLDGLTAER.T | N | 67.54 | 1036.5189 | 9 | -0.7 | 519.2664 | 2 | 11.87 | 6 | F6:2624 | 29102019\_RID\_1313\_NaNaPb\_F5.raw |  | 6.11E5 |  |  |  | 6.51E5 |  |  | 3.19E4 |  | 3 | 0 | 1 | 0 | 0 | 0 | 1 | 0 | 0 | 1 | 0 | 245 | 253 |  |
| R.GSSSGGGYSSGSSSYGSGGR.Q | N | 66.04 | 1739.6982 | 20 | 1.2 | 870.8574 | 2 | 10.89 | 3 | F3:1761 | 29102019\_RID\_1313\_NaNaPb\_F2.raw |  |  | 1.94E4 |  |  |  |  |  |  |  | 1 | 0 | 0 | 1 | 0 | 0 | 0 | 0 | 0 | 0 | 0 | 531 | 550 |  |
| K.VLYDAEISQIHQSVTDTNVILSMDNSR.N | N | 60.56 | 3047.4871 | 27 | 0.2 | 1016.8365 | 3 | 26.70 | 2 | F2:9278 | 29102019\_RID\_1313\_NaNaPb\_F10.raw |  | 1.39E5 |  |  |  |  | 3.63E4 |  |  |  | 2 | 0 | 1 | 0 | 0 | 0 | 0 | 1 | 0 | 0 | 0 | 321 | 347 |  |
| K.SISISVAGGGGGFGAAGGFGGR.G | N | 58.52 | 1837.9070 | 22 | -0.3 | 919.9604 | 2 | 17.88 | 6 | F6:7072 | 29102019\_RID\_1313\_NaNaPb\_F5.raw |  | 1.73E4 |  |  |  | 3.51E5 | 9.75E3 |  |  |  | 4 | 0 | 1 | 0 | 0 | 0 | 2 | 1 | 0 | 0 | 0 | 71 | 92 |  |
| K.IEISELNR.V | N | 55.32 | 972.5240 | 8 | 0.4 | 487.2695 | 2 | 11.76 | 7 | F7:2595 | 29102019\_RID\_1313\_NaNaPb\_F6.raw | 9.39E5 |  | 2.11E6 | 7.37E4 |  | 2.16E6 | 2.78E5 |  | 1.23E5 |  | 6 | 1 | 0 | 1 | 1 | 0 | 1 | 1 | 0 | 1 | 0 | 400 | 407 |  |
| K.YEELQVTVGR.H | N | 54.26 | 1192.6088 | 10 | 0.8 | 597.3121 | 2 | 11.77 | 2 | F2:2740 | 29102019\_RID\_1313\_NaNaPb\_F10.raw |  | 4.09E5 |  |  |  |  |  |  |  |  | 1 | 0 | 1 | 0 | 0 | 0 | 0 | 0 | 0 | 0 | 0 | 381 | 390 |  |
| K.LLEGEEC(+57.02)R.M | N | 53.11 | 1004.4597 | 8 | 0.4 | 503.2373 | 2 | 11.25 | 3 | F3:2079 | 29102019\_RID\_1313\_NaNaPb\_F2.raw |  |  | 3.26E4 |  |  |  |  |  |  |  | 1 | 0 | 0 | 1 | 0 | 0 | 0 | 0 | 0 | 0 | 0 | 489 | 496 | Carbamidomethylation |
| R.DYQELMNVK.L | N | 52.90 | 1138.5328 | 9 | 1.2 | 570.2744 | 2 | 13.43 | 1 | F1:4343 | 29102019\_RID\_1313\_NaNaPb\_F1.raw | 6.15E4 | 5.37E4 |  |  |  |  |  |  |  |  | 2 | 1 | 1 | 0 | 0 | 0 | 0 | 0 | 0 | 0 | 0 | 468 | 476 |  |
| R.SLVGLGGTK.S | N | 52.29 | 830.4861 | 9 | 0.0 | 416.2504 | 2 | 48.25 | 5 | F5:14296 | 29102019\_RID\_1313\_NaNaPb\_F4.raw |  |  |  |  | 1.17E5 |  |  |  |  |  | 1 | 0 | 0 | 0 | 0 | 1 | 0 | 0 | 0 | 0 | 0 | 62 | 70 |  |
| R.GGGFGGGSGFGGGSGFGGGSGFSGGGFGGGGFGGGR.F | Y | 52.04 | 2830.1868 | 36 | 0.0 | 944.4029 | 3 | 23.07 | 7 | F7:8555 | 29102019\_RID\_1313\_NaNaPb\_F6.raw |  |  |  |  |  |  | 2.46E4 |  |  |  | 1 | 0 | 0 | 0 | 0 | 0 | 0 | 1 | 0 | 0 | 0 | 93 | 128 |  |
| R.SKEEAEALYHSK.Y | N | 51.94 | 1390.6729 | 12 | 0.0 | 464.5649 | 3 | 10.96 | 3 | F3:1822 | 29102019\_RID\_1313\_NaNaPb\_F2.raw | 1.34E4 |  | 9.16E3 |  |  |  |  |  |  |  | 2 | 1 | 0 | 1 | 0 | 0 | 0 | 0 | 0 | 0 | 0 | 369 | 380 |  |
| K.VDPEIQNVK.A | N | 50.57 | 1040.5502 | 9 | -1.0 | 521.2819 | 2 | 11.44 | 1 | F1:2994 | 29102019\_RID\_1313\_NaNaPb\_F1.raw | 7.93E4 | 1.34E5 | 5.51E4 |  |  | 3.82E4 |  |  |  |  | 4 | 1 | 1 | 1 | 0 | 0 | 1 | 0 | 0 | 0 | 0 | 173 | 181 |  |
| R.PINLEPIFQGYIDSLK.R | N | 50.42 | 1845.9875 | 16 | 1.0 | 924.0020 | 2 | 49.12 | 6 | F6:19854 | 29102019\_RID\_1313\_NaNaPb\_F5.raw |  | 8.17E4 |  |  |  | 4.75E5 |  |  |  |  | 2 | 0 | 1 | 0 | 0 | 0 | 1 | 0 | 0 | 0 | 0 | 228 | 243 |  |
| R.FGGFGGPGGVGGLGGPGGFGPGGYPGGIHEVSVN.Q | N | 48.49 | 2970.4048 | 34 | 0.2 | 991.1424 | 3 | 31.73 | 2 | F2:10809 | 29102019\_RID\_1313\_NaNaPb\_F10.raw |  | 4.57E4 |  |  |  |  |  |  |  |  | 1 | 0 | 1 | 0 | 0 | 0 | 0 | 0 | 0 | 0 | 0 | 129 | 162 |  |
| K.FASFIDK.V | N | 46.57 | 826.4225 | 7 | -0.3 | 414.2184 | 2 | 11.89 | 2 | F2:2810 | 29102019\_RID\_1313\_NaNaPb\_F10.raw |  | 1.96E5 |  |  |  | 3.22E5 |  |  | 0 |  | 3 | 0 | 1 | 0 | 0 | 0 | 1 | 0 | 0 | 1 | 0 | 195 | 201 |  |
| M.NVGTRPINLEPIFQGYIDSLK.R | N | 43.20 | 2373.2693 | 21 | 1.3 | 792.0981 | 3 | 36.11 | 2 | F2:12041 | 29102019\_RID\_1313\_NaNaPb\_F10.raw |  | 7.29E4 |  |  |  |  |  |  |  |  | 1 | 0 | 1 | 0 | 0 | 0 | 0 | 0 | 0 | 0 | 0 | 223 | 243 |  |
| total 33 peptides |
| --- |

P60043|PA2B1\_NAJSG

back to list

  

| Protein Coverage
| Supporting Peptides
|

Protein Coverage:

Supporting Peptides:

| Peptide | Uniq | -10lgP | Mass | Length | ppm | m/z | z | RT | Fraction | Scan | Source File | Area F1 | Area F10 | Area F2 | Area F3 | Area F4 | Area F5 | Area F6 | Area F7 | Area F8 | Area F9 | #Feature | #Feature F1 | #Feature F10 | #Feature F2 | #Feature F3 | #Feature F4 | #Feature F5 | #Feature F6 | #Feature F7 | #Feature F8 | #Feature F9 | Start | End | PTM |
| --- | --- | --- | --- | --- | --- | --- | --- | --- | --- | --- | --- | --- | --- | --- | --- | --- | --- | --- | --- | --- | --- | --- | --- | --- | --- | --- | --- | --- | --- | --- | --- | --- | --- | --- | --- |
| R.LAAIC(+57.02)FAGAPYNDNNYNIDLK.A | N | 122.34 | 2356.1157 | 21 | 0.2 | 1179.0654 | 2 | 24.76 | 9 | F9:9809 | 29102019\_RID\_1313\_NaNaPb\_F8.raw |  | 4.51E6 |  |  |  | 1.14E9 | 8.96E5 |  | 3.88E8 | 2.3E5 | 38 | 0 | 2 | 0 | 0 | 0 | 22 | 2 | 0 | 10 | 2 | 102 | 122 | Carbamidomethylation |
| R.LAAIC(+57.02)FAGAPYNDNNYNIDLKAR.C | N | 106.85 | 2583.2539 | 23 | 0.7 | 862.0925 | 3 | 17.00 | 9 | F9:5977 | 29102019\_RID\_1313\_NaNaPb\_F8.raw |  |  |  |  |  |  |  |  | 7.29E5 |  | 1 | 0 | 0 | 0 | 0 | 0 | 0 | 0 | 0 | 1 | 0 | 102 | 124 | Carbamidomethylation |
| C.FAGAPYNDNNYNIDLK.A | N | 102.86 | 1827.8428 | 16 | 0.5 | 914.9291 | 2 | 12.58 | 9 | F9:3195 | 29102019\_RID\_1313\_NaNaPb\_F8.raw |  | 9.47E4 |  |  |  | 3.82E7 |  |  | 1.3E7 | 1.77E5 | 8 | 0 | 1 | 0 | 0 | 0 | 4 | 0 | 0 | 2 | 1 | 107 | 122 |  |
| F.AGAPYNDNNYNIDLK.A | N | 99.89 | 1680.7743 | 15 | -1.6 | 841.3931 | 2 | 11.85 | 9 | F9:2622 | 29102019\_RID\_1313\_NaNaPb\_F8.raw |  | 7.76E5 |  |  |  | 2.78E8 |  |  | 9.54E7 | 1.75E6 | 14 | 0 | 1 | 0 | 0 | 0 | 7 | 0 | 0 | 5 | 1 | 108 | 122 |  |
| I.C(+57.02)FAGAPYNDNNYNIDLKARC(+57.02)Q | N | 94.16 | 2503.1008 | 21 | 0.2 | 835.3744 | 3 | 11.81 | 9 | F9:2615 | 29102019\_RID\_1313\_NaNaPb\_F8.raw |  |  |  |  |  |  |  |  | 1.08E6 |  | 2 | 0 | 0 | 0 | 0 | 0 | 0 | 0 | 0 | 2 | 0 | 106 | 126 | Carbamidomethylation |
| R.SWWDFADYGC(+57.02)YC(+57.02)GR.G | N | 92.75 | 1841.6926 | 14 | 0.3 | 921.8539 | 2 | 35.05 | 9 | F9:13640 | 29102019\_RID\_1313\_NaNaPb\_F8.raw |  | 7.26E5 |  |  |  | 4.63E8 |  |  | 2.05E7 |  | 31 | 0 | 1 | 0 | 0 | 0 | 23 | 0 | 0 | 7 | 0 | 24 | 37 | Carbamidomethylation |
| A.AIC(+57.02)FAGAPYNDNNYNIDLKARC(+57.02)Q | N | 85.99 | 2687.2219 | 23 | 0.8 | 896.7487 | 3 | 12.51 | 9 | F9:3154 | 29102019\_RID\_1313\_NaNaPb\_F8.raw |  |  |  |  |  |  |  |  | 5.59E5 |  | 1 | 0 | 0 | 0 | 0 | 0 | 0 | 0 | 0 | 1 | 0 | 104 | 126 | Carbamidomethylation |
| A.IC(+57.02)FAGAPYNDNNYNIDLKARC(+57.02)Q | N | 82.66 | 2616.1848 | 22 | 1.1 | 873.0698 | 3 | 12.27 | 9 | F9:2982 | 29102019\_RID\_1313\_NaNaPb\_F8.raw |  |  |  |  |  |  |  |  | 2.77E5 |  | 1 | 0 | 0 | 0 | 0 | 0 | 0 | 0 | 0 | 1 | 0 | 105 | 126 | Carbamidomethylation |
| C.FAGAPYNDNNYNIDLKARC(+57.02)Q | N | 81.68 | 2343.0701 | 20 | 0.5 | 782.0310 | 3 | 11.75 | 9 | F9:2555 | 29102019\_RID\_1313\_NaNaPb\_F8.raw |  |  |  |  |  |  |  |  | 1.05E6 |  | 2 | 0 | 0 | 0 | 0 | 0 | 0 | 0 | 0 | 2 | 0 | 107 | 126 | Carbamidomethylation |
| D.RLAAIC(+57.02)FAGAPYNDNNYNIDLKARC(+57.02)Q | N | 75.67 | 3027.4443 | 26 | 0.9 | 757.8690 | 4 | 12.31 | 9 | F9:3010 | 29102019\_RID\_1313\_NaNaPb\_F8.raw |  |  |  |  |  |  |  |  | 2.97E5 |  | 1 | 0 | 0 | 0 | 0 | 0 | 0 | 0 | 0 | 1 | 0 | 101 | 126 | Carbamidomethylation |
| D.RLAAIC(+57.02)FAGAPYNDNNYNIDLK.A | N | 72.38 | 2512.2168 | 22 | 0.2 | 838.4130 | 3 | 17.30 | 6 | F6:6122 | 29102019\_RID\_1313\_NaNaPb\_F5.raw |  |  |  |  |  | 7.25E5 |  |  |  |  | 1 | 0 | 0 | 0 | 0 | 0 | 1 | 0 | 0 | 0 | 0 | 101 | 122 | Carbamidomethylation |
| A.GAPYNDNNYNIDLK.A | N | 71.35 | 1609.7372 | 14 | 0.1 | 805.8760 | 2 | 11.93 | 6 | F6:2692 | 29102019\_RID\_1313\_NaNaPb\_F5.raw |  |  |  |  |  | 1.61E6 |  |  |  |  | 1 | 0 | 0 | 0 | 0 | 0 | 1 | 0 | 0 | 0 | 0 | 109 | 122 |  |
| F.AGAPYNDNNYNIDLKARC(+57.02)Q | N | 70.05 | 2196.0017 | 19 | 0.5 | 733.0082 | 3 | 11.51 | 9 | F9:2340 | 29102019\_RID\_1313\_NaNaPb\_F8.raw |  |  |  |  |  |  |  |  | 7.33E5 |  | 1 | 0 | 0 | 0 | 0 | 0 | 0 | 0 | 0 | 1 | 0 | 108 | 126 | Carbamidomethylation |
| A.IC(+57.02)FAGAPYNDNNYNIDLKAR.C | N | 69.77 | 2328.0957 | 20 | 1.0 | 777.0399 | 3 | 12.51 | 9 | F9:3176 | 29102019\_RID\_1313\_NaNaPb\_F8.raw |  |  |  |  |  |  |  |  | 8.95E4 |  | 1 | 0 | 0 | 0 | 0 | 0 | 0 | 0 | 0 | 1 | 0 | 105 | 124 | Carbamidomethylation |
| G.APYNDNNYNIDLK.A | N | 68.55 | 1552.7157 | 13 | 0.7 | 777.3657 | 2 | 11.91 | 6 | F6:2696 | 29102019\_RID\_1313\_NaNaPb\_F5.raw |  |  |  |  |  | 3.08E5 |  |  |  |  | 1 | 0 | 0 | 0 | 0 | 0 | 1 | 0 | 0 | 0 | 0 | 110 | 122 |  |
| Y.NDNNYNIDLK.A | N | 67.89 | 1221.5625 | 10 | 1.0 | 611.7891 | 2 | 11.68 | 6 | F6:2444 | 29102019\_RID\_1313\_NaNaPb\_F5.raw |  |  |  |  |  | 3.26E6 |  |  | 1.93E6 |  | 2 | 0 | 0 | 0 | 0 | 0 | 1 | 0 | 0 | 1 | 0 | 113 | 122 |  |
| A.IC(+57.02)FAGAPYNDNNYNIDLK.A | N | 66.89 | 2100.9575 | 18 | 0.0 | 701.3264 | 3 | 17.93 | 6 | F6:6564 | 29102019\_RID\_1313\_NaNaPb\_F5.raw |  |  |  |  |  | 1.53E5 |  |  | 7.23E6 |  | 4 | 0 | 0 | 0 | 0 | 0 | 2 | 0 | 0 | 2 | 0 | 105 | 122 | Carbamidomethylation |
| Y.NDNNYNIDLKARC(+57.02)Q | N | 62.36 | 1736.7900 | 14 | -0.7 | 579.9369 | 3 | 11.26 | 9 | F9:2124 | 29102019\_RID\_1313\_NaNaPb\_F8.raw |  |  |  |  |  |  |  |  | 2.67E5 |  | 1 | 0 | 0 | 0 | 0 | 0 | 0 | 0 | 0 | 1 | 0 | 113 | 126 | Carbamidomethylation |
| W.DFADYGC(+57.02)YC(+57.02)GR.G | N | 59.28 | 1382.5020 | 11 | -0.8 | 692.2577 | 2 | 11.98 | 6 | F6:2741 | 29102019\_RID\_1313\_NaNaPb\_F5.raw |  |  |  |  |  | 6.86E5 |  |  |  |  | 1 | 0 | 0 | 0 | 0 | 0 | 1 | 0 | 0 | 0 | 0 | 27 | 37 | Carbamidomethylation |
| R.LAAIC(+57.02)FAGAPYNDNNYNIDLKARC(+57.02)Q | N | 58.75 | 2871.3433 | 25 | 0.6 | 958.1223 | 3 | 16.04 | 9 | F9:5714 | 29102019\_RID\_1313\_NaNaPb\_F8.raw |  |  |  |  |  |  |  |  | 1.54E6 |  | 1 | 0 | 0 | 0 | 0 | 0 | 0 | 0 | 0 | 1 | 0 | 102 | 126 | Carbamidomethylation |
| R.LAAIC(+57.02)FAGAPYN.D | N | 58.35 | 1266.6067 | 12 | 0.1 | 634.3107 | 2 | 24.51 | 6 | F6:9784 | 29102019\_RID\_1313\_NaNaPb\_F5.raw |  |  |  |  |  | 2.29E6 |  |  | 1.49E5 |  | 3 | 0 | 0 | 0 | 0 | 0 | 2 | 0 | 0 | 1 | 0 | 102 | 113 | Carbamidomethylation |
| R.SWWDFADYGC(+57.02)YC(+57.02)GRG.G | N | 56.62 | 1898.7141 | 15 | 0.4 | 950.3647 | 2 | 33.02 | 9 | F9:12959 | 29102019\_RID\_1313\_NaNaPb\_F8.raw |  |  |  |  |  | 2.73E7 |  |  | 3.48E5 |  | 6 | 0 | 0 | 0 | 0 | 0 | 4 | 0 | 0 | 2 | 0 | 24 | 38 | Carbamidomethylation |
| D.FADYGC(+57.02)YC(+57.02)GR.G | N | 54.59 | 1267.4750 | 10 | 0.4 | 634.7450 | 2 | 11.57 | 6 | F6:2343 | 29102019\_RID\_1313\_NaNaPb\_F5.raw |  |  |  |  |  | 4.49E5 |  |  |  |  | 1 | 0 | 0 | 0 | 0 | 0 | 1 | 0 | 0 | 0 | 0 | 28 | 37 | Carbamidomethylation |
| I.C(+57.02)FAGAPYNDNNYNIDLK.A | N | 54.20 | 1987.8734 | 17 | 0.6 | 994.9446 | 2 | 13.27 | 9 | F9:3677 | 29102019\_RID\_1313\_NaNaPb\_F8.raw |  |  |  |  |  |  |  |  | 2.25E5 |  | 1 | 0 | 0 | 0 | 0 | 0 | 0 | 0 | 0 | 1 | 0 | 106 | 122 | Carbamidomethylation |
| R.SWWDFADYGC(+57.02)Y.C | N | 53.68 | 1468.5394 | 11 | 0.0 | 735.2770 | 2 | 55.23 | 6 | F6:21957 | 29102019\_RID\_1313\_NaNaPb\_F5.raw |  |  |  |  |  | 2.22E7 |  |  |  |  | 1 | 0 | 0 | 0 | 0 | 0 | 1 | 0 | 0 | 0 | 0 | 24 | 34 | Carbamidomethylation |
| N.DNNYNIDLK.A | N | 51.97 | 1107.5197 | 9 | -1.6 | 554.7662 | 2 | 11.86 | 6 | F6:2632 | 29102019\_RID\_1313\_NaNaPb\_F5.raw |  |  |  |  |  | 2.04E5 |  |  | 3.21E4 |  | 2 | 0 | 0 | 0 | 0 | 0 | 1 | 0 | 0 | 1 | 0 | 114 | 122 |  |
| C.DC(+57.02)DRLAAIC(+57.02)FAGAPYNDNNYNIDLKAR.C | N | 51.86 | 3129.4397 | 27 | 0.8 | 783.3678 | 4 | 16.75 | 9 | F9:5867 | 29102019\_RID\_1313\_NaNaPb\_F8.raw |  |  |  |  |  |  |  |  | 8.64E4 |  | 1 | 0 | 0 | 0 | 0 | 0 | 0 | 0 | 0 | 1 | 0 | 98 | 124 | Carbamidomethylation |
| R.LAAIC(+57.02)FAGAPY.N | N | 48.00 | 1152.5637 | 11 | -0.1 | 577.2891 | 2 | 29.05 | 9 | F9:11682 | 29102019\_RID\_1313\_NaNaPb\_F8.raw |  |  |  |  |  |  |  |  | 1.7E6 |  | 1 | 0 | 0 | 0 | 0 | 0 | 0 | 0 | 0 | 1 | 0 | 102 | 112 | Carbamidomethylation |
| P.YNDNNYNIDLK.A | N | 47.70 | 1384.6259 | 11 | -1.6 | 693.3191 | 2 | 11.80 | 6 | F6:2577 | 29102019\_RID\_1313\_NaNaPb\_F5.raw |  |  |  |  |  | 1.37E5 |  |  |  |  | 1 | 0 | 0 | 0 | 0 | 0 | 1 | 0 | 0 | 0 | 0 | 112 | 122 |  |
| W.WDFADYGC(+57.02)YC(+57.02)GR.G | N | 46.98 | 1568.5813 | 12 | 0.7 | 785.2985 | 2 | 17.36 | 6 | F6:6148 | 29102019\_RID\_1313\_NaNaPb\_F5.raw |  |  |  |  |  | 3.91E5 |  |  |  |  | 1 | 0 | 0 | 0 | 0 | 0 | 1 | 0 | 0 | 0 | 0 | 26 | 37 | Carbamidomethylation |
| A.AIC(+57.02)FAGAPYNDNNYNIDLKAR.C | N | 46.86 | 2399.1328 | 21 | 1.0 | 800.7190 | 3 | 13.04 | 9 | F9:3508 | 29102019\_RID\_1313\_NaNaPb\_F8.raw |  |  |  |  |  |  |  |  | 2.26E5 |  | 1 | 0 | 0 | 0 | 0 | 0 | 0 | 0 | 0 | 1 | 0 | 104 | 124 | Carbamidomethylation |
| D.C(+57.02)DRLAAIC(+57.02)FAGAPYNDNNYNIDLKARC(+57.02)Q | N | 46.30 | 3302.5020 | 28 | 0.5 | 826.6332 | 4 | 13.71 | 9 | F9:3965 | 29102019\_RID\_1313\_NaNaPb\_F8.raw |  |  |  |  |  |  |  |  | 4.11E5 |  | 1 | 0 | 0 | 0 | 0 | 0 | 0 | 0 | 0 | 1 | 0 | 99 | 126 | Carbamidomethylation |
| P.KRSWWDFADYGC(+57.02)YC(+57.02)GR.G | Y | 42.86 | 2125.8887 | 16 | -3.7 | 1063.9476 | 2 | 35.42 | 6 | F6:15228 | 29102019\_RID\_1313\_NaNaPb\_F5.raw |  |  |  |  |  | 0 |  |  |  |  | 1 | 0 | 0 | 0 | 0 | 0 | 1 | 0 | 0 | 0 | 0 | 22 | 37 | Carbamidomethylation |
| L.AAIC(+57.02)FAGAPYNDNNYNIDLKAR.C | N | 42.40 | 2470.1699 | 22 | 1.3 | 824.3983 | 3 | 13.65 | 9 | F9:3964 | 29102019\_RID\_1313\_NaNaPb\_F8.raw |  |  |  |  |  |  |  |  | 1.29E5 |  | 1 | 0 | 0 | 0 | 0 | 0 | 0 | 0 | 0 | 1 | 0 | 103 | 124 | Carbamidomethylation |
| total 34 peptides |
| --- |

P78386|KRT85\_HUMAN

back to list

  

| Protein Coverage
| Supporting Peptides
|

Protein Coverage:

Supporting Peptides:

| Peptide | Uniq | -10lgP | Mass | Length | ppm | m/z | z | RT | Fraction | Scan | Source File | Area F1 | Area F10 | Area F2 | Area F3 | Area F4 | Area F5 | Area F6 | Area F7 | Area F8 | Area F9 | #Feature | #Feature F1 | #Feature F10 | #Feature F2 | #Feature F3 | #Feature F4 | #Feature F5 | #Feature F6 | #Feature F7 | #Feature F8 | #Feature F9 | Start | End | PTM |
| --- | --- | --- | --- | --- | --- | --- | --- | --- | --- | --- | --- | --- | --- | --- | --- | --- | --- | --- | --- | --- | --- | --- | --- | --- | --- | --- | --- | --- | --- | --- | --- | --- | --- | --- | --- |
| R.AKLEAAVAEAEQQGEAALSDAR.C | Y | 111.22 | 2227.1079 | 22 | 0.3 | 743.3768 | 3 | 72.53 | 5 | F5:34607 | 29102019\_RID\_1313\_NaNaPb\_F4.raw |  |  |  |  | 8.78E7 |  |  |  |  |  | 2 | 0 | 0 | 0 | 0 | 2 | 0 | 0 | 0 | 0 | 0 | 361 | 382 |  |
| R.LYEEEIRVLQAHISDTSVIVK.M | N | 104.15 | 2441.3164 | 21 | 1.2 | 814.7804 | 3 | 73.47 | 5 | F5:35475 | 29102019\_RID\_1313\_NaNaPb\_F4.raw |  |  |  |  | 9.22E6 |  |  |  |  |  | 2 | 0 | 0 | 0 | 0 | 2 | 0 | 0 | 0 | 0 | 0 | 259 | 279 |  |
| K.LEAAVAEAEQQGEAALSDAR.C | Y | 102.30 | 2027.9760 | 20 | 1.8 | 1014.9971 | 2 | 71.27 | 5 | F5:33689 | 29102019\_RID\_1313\_NaNaPb\_F4.raw |  |  |  |  | 1.17E6 |  |  |  |  |  | 1 | 0 | 0 | 0 | 0 | 1 | 0 | 0 | 0 | 0 | 0 | 363 | 382 |  |
| R.FLEQQNKLLETKWQFYQNQR.C | Y | 101.92 | 2640.3447 | 20 | 2.5 | 881.1244 | 3 | 64.28 | 5 | F5:27697 | 29102019\_RID\_1313\_NaNaPb\_F4.raw |  |  |  |  | 6.72E7 |  |  |  |  |  | 3 | 0 | 0 | 0 | 0 | 3 | 0 | 0 | 0 | 0 | 0 | 143 | 162 |  |
| R.FAAFIDKVRFLEQQNKLLETK.W | N | 95.67 | 2537.4004 | 21 | -0.3 | 846.8072 | 3 | 69.08 | 5 | F5:31940 | 29102019\_RID\_1313\_NaNaPb\_F4.raw |  |  |  |  | 2.99E7 |  |  |  |  |  | 3 | 0 | 0 | 0 | 0 | 3 | 0 | 0 | 0 | 0 | 0 | 134 | 154 |  |
| K.SLNSRFAAFIDKVR.F | N | 84.83 | 1622.8892 | 14 | 0.9 | 541.9708 | 3 | 64.24 | 5 | F5:27586 | 29102019\_RID\_1313\_NaNaPb\_F4.raw |  |  |  |  | 8.58E6 |  |  |  |  |  | 2 | 0 | 0 | 0 | 0 | 2 | 0 | 0 | 0 | 0 | 0 | 129 | 142 |  |
| R.M(+15.99)IQRLTAEIENAK.C | N | 81.18 | 1531.8029 | 13 | -0.3 | 511.6081 | 3 | 51.69 | 5 | F5:17111 | 29102019\_RID\_1313\_NaNaPb\_F4.raw |  |  |  |  | 5.19E5 |  |  |  |  |  | 1 | 0 | 0 | 0 | 0 | 1 | 0 | 0 | 0 | 0 | 0 | 345 | 357 | Oxidation (M) |
| R.LASELNHVQEVLEGYKK.K | N | 77.99 | 1956.0316 | 17 | 0.5 | 490.0154 | 4 | 62.56 | 5 | F5:26250 | 29102019\_RID\_1313\_NaNaPb\_F4.raw |  |  |  |  | 0 |  |  |  |  |  | 1 | 0 | 0 | 0 | 0 | 1 | 0 | 0 | 0 | 0 | 0 | 193 | 209 |  |
| R.TKEEINELNR.M | N | 76.25 | 1244.6360 | 10 | -0.1 | 623.3252 | 2 | 36.79 | 5 | F5:5864 | 29102019\_RID\_1313\_NaNaPb\_F4.raw |  |  |  |  | 1.11E7 |  |  |  |  |  | 2 | 0 | 0 | 0 | 0 | 2 | 0 | 0 | 0 | 0 | 0 | 335 | 344 |  |
| R.KSDLEANVEALVEESSFLRR.L | Y | 73.44 | 2291.1758 | 20 | 0.1 | 573.8013 | 4 | 81.48 | 5 | F5:42166 | 29102019\_RID\_1313\_NaNaPb\_F4.raw |  |  |  |  | 2.58E7 |  |  |  |  |  | 2 | 0 | 0 | 0 | 0 | 2 | 0 | 0 | 0 | 0 | 0 | 239 | 258 |  |
| R.FLEQQNKLLETK.W | N | 69.75 | 1489.8140 | 12 | 0.7 | 745.9148 | 2 | 49.91 | 5 | F5:15503 | 29102019\_RID\_1313\_NaNaPb\_F4.raw |  |  |  |  | 8.24E7 |  |  |  |  |  | 3 | 0 | 0 | 0 | 0 | 3 | 0 | 0 | 0 | 0 | 0 | 143 | 154 |  |
| K.LGLDIEIATYR.R | N | 68.96 | 1262.6870 | 11 | -0.1 | 632.3507 | 2 | 74.93 | 5 | F5:36761 | 29102019\_RID\_1313\_NaNaPb\_F4.raw |  |  |  |  | 1.73E5 |  |  |  |  |  | 1 | 0 | 0 | 0 | 0 | 1 | 0 | 0 | 0 | 0 | 0 | 414 | 424 |  |
| R.FAAFIDKVRFLEQQNK.L | N | 68.77 | 1953.0471 | 16 | -0.2 | 489.2690 | 4 | 65.30 | 5 | F5:28595 | 29102019\_RID\_1313\_NaNaPb\_F4.raw |  |  |  |  | 1.83E7 |  |  |  |  |  | 3 | 0 | 0 | 0 | 0 | 3 | 0 | 0 | 0 | 0 | 0 | 134 | 149 |  |
| R.SRAEAESWYR.S | N | 68.47 | 1253.5789 | 10 | -0.3 | 418.8668 | 3 | 41.96 | 5 | F5:9725 | 29102019\_RID\_1313\_NaNaPb\_F4.raw |  |  |  |  | 6.42E6 |  |  |  |  |  | 2 | 0 | 0 | 0 | 0 | 2 | 0 | 0 | 0 | 0 | 0 | 306 | 315 |  |
| K.LAELEGALQKAK.Q | N | 65.48 | 1269.7292 | 12 | -0.6 | 424.2501 | 3 | 48.27 | 5 | F5:14319 | 29102019\_RID\_1313\_NaNaPb\_F4.raw |  |  |  |  | 6E6 |  |  |  |  |  | 2 | 0 | 0 | 0 | 0 | 2 | 0 | 0 | 0 | 0 | 0 | 385 | 396 |  |
| R.MIQRLTAEIENAK.C | N | 63.86 | 1515.8079 | 13 | 0.1 | 506.2766 | 3 | 51.64 | 5 | F5:16969 | 29102019\_RID\_1313\_NaNaPb\_F4.raw |  |  |  |  | 8.3E6 |  |  |  |  |  | 1 | 0 | 0 | 0 | 0 | 1 | 0 | 0 | 0 | 0 | 0 | 345 | 357 |  |
| R.TKEEINELNRMIQRLTAEIENAK.C | N | 62.17 | 2742.4333 | 23 | 1.5 | 686.6166 | 4 | 84.88 | 5 | F5:45152 | 29102019\_RID\_1313\_NaNaPb\_F4.raw |  |  |  |  | 0 |  |  |  |  |  | 1 | 0 | 0 | 0 | 0 | 1 | 0 | 0 | 0 | 0 | 0 | 335 | 357 |  |
| R.ATAENEFVVLKK.D | N | 61.77 | 1347.7397 | 12 | 1.9 | 450.2547 | 3 | 49.26 | 5 | F5:15143 | 29102019\_RID\_1313\_NaNaPb\_F4.raw |  |  |  |  | 3.3E5 |  |  |  |  |  | 1 | 0 | 0 | 0 | 0 | 1 | 0 | 0 | 0 | 0 | 0 | 219 | 230 |  |
| R.FAAFIDKVR.F | N | 61.47 | 1065.5970 | 9 | 0.0 | 533.8058 | 2 | 53.38 | 5 | F5:18300 | 29102019\_RID\_1313\_NaNaPb\_F4.raw |  |  |  |  | 1.48E8 |  |  |  |  |  | 1 | 0 | 0 | 0 | 0 | 1 | 0 | 0 | 0 | 0 | 0 | 134 | 142 |  |
| K.LAELEGALQK.A | N | 60.04 | 1070.5972 | 10 | -1.3 | 536.3052 | 2 | 53.69 | 5 | F5:18607 | 29102019\_RID\_1313\_NaNaPb\_F4.raw |  |  |  |  | 4.12E7 |  |  |  |  |  | 1 | 0 | 0 | 0 | 0 | 1 | 0 | 0 | 0 | 0 | 0 | 385 | 394 |  |
| R.HGETLRRTKEEINELNR.M | N | 57.51 | 2094.0930 | 17 | 0.0 | 524.5305 | 4 | 36.95 | 5 | F5:5998 | 29102019\_RID\_1313\_NaNaPb\_F4.raw |  |  |  |  | 7.51E6 |  |  |  |  |  | 1 | 0 | 0 | 0 | 0 | 1 | 0 | 0 | 0 | 0 | 0 | 328 | 344 |  |
| R.VLQAHISDTSVIVK.M | N | 56.07 | 1508.8562 | 14 | 1.2 | 755.4363 | 2 | 50.60 | 5 | F5:16233 | 29102019\_RID\_1313\_NaNaPb\_F4.raw |  |  |  |  | 3.07E6 |  |  |  |  |  | 2 | 0 | 0 | 0 | 0 | 2 | 0 | 0 | 0 | 0 | 0 | 266 | 279 |  |
| K.LGLDIEIATYRR.L | N | 54.37 | 1418.7881 | 12 | 0.1 | 473.9367 | 3 | 65.02 | 5 | F5:28353 | 29102019\_RID\_1313\_NaNaPb\_F4.raw |  |  |  |  | 0 |  |  |  |  |  | 1 | 0 | 0 | 0 | 0 | 1 | 0 | 0 | 0 | 0 | 0 | 414 | 425 |  |
| R.FAAFIDK.V | N | 52.16 | 810.4276 | 7 | -0.3 | 406.2209 | 2 | 57.20 | 5 | F5:21640 | 29102019\_RID\_1313\_NaNaPb\_F4.raw |  |  |  |  | 7.64E5 |  |  |  |  |  | 1 | 0 | 0 | 0 | 0 | 1 | 0 | 0 | 0 | 0 | 0 | 134 | 140 |  |
| R.TKEEINELNRMIQR.L | N | 51.55 | 1772.9203 | 14 | 1.0 | 591.9813 | 3 | 51.34 | 5 | F5:16726 | 29102019\_RID\_1313\_NaNaPb\_F4.raw |  |  |  |  | 6.13E7 |  |  |  |  |  | 1 | 0 | 0 | 0 | 0 | 1 | 0 | 0 | 0 | 0 | 0 | 335 | 348 |  |
| K.AQYDDVASR.S | N | 49.56 | 1023.4621 | 9 | 0.6 | 512.7386 | 2 | 37.74 | 5 | F5:6639 | 29102019\_RID\_1313\_NaNaPb\_F4.raw |  |  |  |  | 2.54E5 |  |  |  |  |  | 1 | 0 | 0 | 0 | 0 | 1 | 0 | 0 | 0 | 0 | 0 | 297 | 305 |  |
| R.AEAESWYR.S | N | 49.42 | 1010.4457 | 8 | 0.0 | 506.2302 | 2 | 50.22 | 5 | F5:15799 | 29102019\_RID\_1313\_NaNaPb\_F4.raw |  |  |  |  | 1.33E6 |  |  |  |  |  | 1 | 0 | 0 | 0 | 0 | 1 | 0 | 0 | 0 | 0 | 0 | 308 | 315 |  |
| R.GLTGFGSR.S | Y | 46.33 | 793.4082 | 8 | 1.1 | 397.7118 | 2 | 47.29 | 5 | F5:13639 | 29102019\_RID\_1313\_NaNaPb\_F4.raw |  |  |  |  | 8.05E5 |  |  |  |  |  | 1 | 0 | 0 | 0 | 0 | 1 | 0 | 0 | 0 | 0 | 0 | 47 | 54 |  |
| R.NFSSC(+57.02)SAVAPK.T | N | 46.18 | 1166.5389 | 11 | 0.0 | 584.2767 | 2 | 39.84 | 5 | F5:8274 | 29102019\_RID\_1313\_NaNaPb\_F4.raw |  |  |  |  | 1.68E5 |  |  |  |  |  | 1 | 0 | 0 | 0 | 0 | 1 | 0 | 0 | 0 | 0 | 0 | 17 | 27 | Carbamidomethylation |
| total 29 peptides |
| --- |

#CONTAM#KRHB5\_HUMAN|

back to list

  

| Protein Coverage
| Supporting Peptides
|

Protein Coverage:

Supporting Peptides:

| Peptide | Uniq | -10lgP | Mass | Length | ppm | m/z | z | RT | Fraction | Scan | Source File | Area F1 | Area F10 | Area F2 | Area F3 | Area F4 | Area F5 | Area F6 | Area F7 | Area F8 | Area F9 | #Feature | #Feature F1 | #Feature F10 | #Feature F2 | #Feature F3 | #Feature F4 | #Feature F5 | #Feature F6 | #Feature F7 | #Feature F8 | #Feature F9 | Start | End | PTM |
| --- | --- | --- | --- | --- | --- | --- | --- | --- | --- | --- | --- | --- | --- | --- | --- | --- | --- | --- | --- | --- | --- | --- | --- | --- | --- | --- | --- | --- | --- | --- | --- | --- | --- | --- | --- |
| R.AKLEAAVAEAEQQGEAALSDAR.C | Y | 111.22 | 2227.1079 | 22 | 0.3 | 743.3768 | 3 | 72.53 | 5 | F5:34607 | 29102019\_RID\_1313\_NaNaPb\_F4.raw |  |  |  |  | 8.78E7 |  |  |  |  |  | 2 | 0 | 0 | 0 | 0 | 2 | 0 | 0 | 0 | 0 | 0 | 361 | 382 |  |
| R.LYEEEIRVLQAHISDTSVIVK.M | N | 104.15 | 2441.3164 | 21 | 1.2 | 814.7804 | 3 | 73.47 | 5 | F5:35475 | 29102019\_RID\_1313\_NaNaPb\_F4.raw |  |  |  |  | 9.22E6 |  |  |  |  |  | 2 | 0 | 0 | 0 | 0 | 2 | 0 | 0 | 0 | 0 | 0 | 259 | 279 |  |
| K.LEAAVAEAEQQGEAALSDAR.C | Y | 102.30 | 2027.9760 | 20 | 1.8 | 1014.9971 | 2 | 71.27 | 5 | F5:33689 | 29102019\_RID\_1313\_NaNaPb\_F4.raw |  |  |  |  | 1.17E6 |  |  |  |  |  | 1 | 0 | 0 | 0 | 0 | 1 | 0 | 0 | 0 | 0 | 0 | 363 | 382 |  |
| R.FLEQQNKLLETKWQFYQNQR.C | Y | 101.92 | 2640.3447 | 20 | 2.5 | 881.1244 | 3 | 64.28 | 5 | F5:27697 | 29102019\_RID\_1313\_NaNaPb\_F4.raw |  |  |  |  | 6.72E7 |  |  |  |  |  | 3 | 0 | 0 | 0 | 0 | 3 | 0 | 0 | 0 | 0 | 0 | 143 | 162 |  |
| R.FAAFIDKVRFLEQQNKLLETK.W | N | 95.67 | 2537.4004 | 21 | -0.3 | 846.8072 | 3 | 69.08 | 5 | F5:31940 | 29102019\_RID\_1313\_NaNaPb\_F4.raw |  |  |  |  | 2.99E7 |  |  |  |  |  | 3 | 0 | 0 | 0 | 0 | 3 | 0 | 0 | 0 | 0 | 0 | 134 | 154 |  |
| K.SLNSRFAAFIDKVR.F | N | 84.83 | 1622.8892 | 14 | 0.9 | 541.9708 | 3 | 64.24 | 5 | F5:27586 | 29102019\_RID\_1313\_NaNaPb\_F4.raw |  |  |  |  | 8.58E6 |  |  |  |  |  | 2 | 0 | 0 | 0 | 0 | 2 | 0 | 0 | 0 | 0 | 0 | 129 | 142 |  |
| R.M(+15.99)IQRLTAEIENAK.C | N | 81.18 | 1531.8029 | 13 | -0.3 | 511.6081 | 3 | 51.69 | 5 | F5:17111 | 29102019\_RID\_1313\_NaNaPb\_F4.raw |  |  |  |  | 5.19E5 |  |  |  |  |  | 1 | 0 | 0 | 0 | 0 | 1 | 0 | 0 | 0 | 0 | 0 | 345 | 357 | Oxidation (M) |
| R.LASELNHVQEVLEGYKK.K | N | 77.99 | 1956.0316 | 17 | 0.5 | 490.0154 | 4 | 62.56 | 5 | F5:26250 | 29102019\_RID\_1313\_NaNaPb\_F4.raw |  |  |  |  | 0 |  |  |  |  |  | 1 | 0 | 0 | 0 | 0 | 1 | 0 | 0 | 0 | 0 | 0 | 193 | 209 |  |
| R.TKEEINELNR.M | N | 76.25 | 1244.6360 | 10 | -0.1 | 623.3252 | 2 | 36.79 | 5 | F5:5864 | 29102019\_RID\_1313\_NaNaPb\_F4.raw |  |  |  |  | 1.11E7 |  |  |  |  |  | 2 | 0 | 0 | 0 | 0 | 2 | 0 | 0 | 0 | 0 | 0 | 335 | 344 |  |
| R.KSDLEANVEALVEESSFLRR.L | Y | 73.44 | 2291.1758 | 20 | 0.1 | 573.8013 | 4 | 81.48 | 5 | F5:42166 | 29102019\_RID\_1313\_NaNaPb\_F4.raw |  |  |  |  | 2.58E7 |  |  |  |  |  | 2 | 0 | 0 | 0 | 0 | 2 | 0 | 0 | 0 | 0 | 0 | 239 | 258 |  |
| R.FLEQQNKLLETK.W | N | 69.75 | 1489.8140 | 12 | 0.7 | 745.9148 | 2 | 49.91 | 5 | F5:15503 | 29102019\_RID\_1313\_NaNaPb\_F4.raw |  |  |  |  | 8.24E7 |  |  |  |  |  | 3 | 0 | 0 | 0 | 0 | 3 | 0 | 0 | 0 | 0 | 0 | 143 | 154 |  |
| K.LGLDIEIATYR.R | N | 68.96 | 1262.6870 | 11 | -0.1 | 632.3507 | 2 | 74.93 | 5 | F5:36761 | 29102019\_RID\_1313\_NaNaPb\_F4.raw |  |  |  |  | 1.73E5 |  |  |  |  |  | 1 | 0 | 0 | 0 | 0 | 1 | 0 | 0 | 0 | 0 | 0 | 414 | 424 |  |
| R.FAAFIDKVRFLEQQNK.L | N | 68.77 | 1953.0471 | 16 | -0.2 | 489.2690 | 4 | 65.30 | 5 | F5:28595 | 29102019\_RID\_1313\_NaNaPb\_F4.raw |  |  |  |  | 1.83E7 |  |  |  |  |  | 3 | 0 | 0 | 0 | 0 | 3 | 0 | 0 | 0 | 0 | 0 | 134 | 149 |  |
| R.SRAEAESWYR.S | N | 68.47 | 1253.5789 | 10 | -0.3 | 418.8668 | 3 | 41.96 | 5 | F5:9725 | 29102019\_RID\_1313\_NaNaPb\_F4.raw |  |  |  |  | 6.42E6 |  |  |  |  |  | 2 | 0 | 0 | 0 | 0 | 2 | 0 | 0 | 0 | 0 | 0 | 306 | 315 |  |
| K.LAELEGALQKAK.Q | N | 65.48 | 1269.7292 | 12 | -0.6 | 424.2501 | 3 | 48.27 | 5 | F5:14319 | 29102019\_RID\_1313\_NaNaPb\_F4.raw |  |  |  |  | 6E6 |  |  |  |  |  | 2 | 0 | 0 | 0 | 0 | 2 | 0 | 0 | 0 | 0 | 0 | 385 | 396 |  |
| R.MIQRLTAEIENAK.C | N | 63.86 | 1515.8079 | 13 | 0.1 | 506.2766 | 3 | 51.64 | 5 | F5:16969 | 29102019\_RID\_1313\_NaNaPb\_F4.raw |  |  |  |  | 8.3E6 |  |  |  |  |  | 1 | 0 | 0 | 0 | 0 | 1 | 0 | 0 | 0 | 0 | 0 | 345 | 357 |  |
| R.TKEEINELNRMIQRLTAEIENAK.C | N | 62.17 | 2742.4333 | 23 | 1.5 | 686.6166 | 4 | 84.88 | 5 | F5:45152 | 29102019\_RID\_1313\_NaNaPb\_F4.raw |  |  |  |  | 0 |  |  |  |  |  | 1 | 0 | 0 | 0 | 0 | 1 | 0 | 0 | 0 | 0 | 0 | 335 | 357 |  |
| R.ATAENEFVVLKK.D | N | 61.77 | 1347.7397 | 12 | 1.9 | 450.2547 | 3 | 49.26 | 5 | F5:15143 | 29102019\_RID\_1313\_NaNaPb\_F4.raw |  |  |  |  | 3.3E5 |  |  |  |  |  | 1 | 0 | 0 | 0 | 0 | 1 | 0 | 0 | 0 | 0 | 0 | 219 | 230 |  |
| R.FAAFIDKVR.F | N | 61.47 | 1065.5970 | 9 | 0.0 | 533.8058 | 2 | 53.38 | 5 | F5:18300 | 29102019\_RID\_1313\_NaNaPb\_F4.raw |  |  |  |  | 1.48E8 |  |  |  |  |  | 1 | 0 | 0 | 0 | 0 | 1 | 0 | 0 | 0 | 0 | 0 | 134 | 142 |  |
| K.LAELEGALQK.A | N | 60.04 | 1070.5972 | 10 | -1.3 | 536.3052 | 2 | 53.69 | 5 | F5:18607 | 29102019\_RID\_1313\_NaNaPb\_F4.raw |  |  |  |  | 4.12E7 |  |  |  |  |  | 1 | 0 | 0 | 0 | 0 | 1 | 0 | 0 | 0 | 0 | 0 | 385 | 394 |  |
| R.HGETLRRTKEEINELNR.M | N | 57.51 | 2094.0930 | 17 | 0.0 | 524.5305 | 4 | 36.95 | 5 | F5:5998 | 29102019\_RID\_1313\_NaNaPb\_F4.raw |  |  |  |  | 7.51E6 |  |  |  |  |  | 1 | 0 | 0 | 0 | 0 | 1 | 0 | 0 | 0 | 0 | 0 | 328 | 344 |  |
| R.VLQAHISDTSVIVK.M | N | 56.07 | 1508.8562 | 14 | 1.2 | 755.4363 | 2 | 50.60 | 5 | F5:16233 | 29102019\_RID\_1313\_NaNaPb\_F4.raw |  |  |  |  | 3.07E6 |  |  |  |  |  | 2 | 0 | 0 | 0 | 0 | 2 | 0 | 0 | 0 | 0 | 0 | 266 | 279 |  |
| K.LGLDIEIATYRR.L | N | 54.37 | 1418.7881 | 12 | 0.1 | 473.9367 | 3 | 65.02 | 5 | F5:28353 | 29102019\_RID\_1313\_NaNaPb\_F4.raw |  |  |  |  | 0 |  |  |  |  |  | 1 | 0 | 0 | 0 | 0 | 1 | 0 | 0 | 0 | 0 | 0 | 414 | 425 |  |
| R.FAAFIDK.V | N | 52.16 | 810.4276 | 7 | -0.3 | 406.2209 | 2 | 57.20 | 5 | F5:21640 | 29102019\_RID\_1313\_NaNaPb\_F4.raw |  |  |  |  | 7.64E5 |  |  |  |  |  | 1 | 0 | 0 | 0 | 0 | 1 | 0 | 0 | 0 | 0 | 0 | 134 | 140 |  |
| R.TKEEINELNRMIQR.L | N | 51.55 | 1772.9203 | 14 | 1.0 | 591.9813 | 3 | 51.34 | 5 | F5:16726 | 29102019\_RID\_1313\_NaNaPb\_F4.raw |  |  |  |  | 6.13E7 |  |  |  |  |  | 1 | 0 | 0 | 0 | 0 | 1 | 0 | 0 | 0 | 0 | 0 | 335 | 348 |  |
| K.AQYDDVASR.S | N | 49.56 | 1023.4621 | 9 | 0.6 | 512.7386 | 2 | 37.74 | 5 | F5:6639 | 29102019\_RID\_1313\_NaNaPb\_F4.raw |  |  |  |  | 2.54E5 |  |  |  |  |  | 1 | 0 | 0 | 0 | 0 | 1 | 0 | 0 | 0 | 0 | 0 | 297 | 305 |  |
| R.AEAESWYR.S | N | 49.42 | 1010.4457 | 8 | 0.0 | 506.2302 | 2 | 50.22 | 5 | F5:15799 | 29102019\_RID\_1313\_NaNaPb\_F4.raw |  |  |  |  | 1.33E6 |  |  |  |  |  | 1 | 0 | 0 | 0 | 0 | 1 | 0 | 0 | 0 | 0 | 0 | 308 | 315 |  |
| R.GLTGFGSR.S | Y | 46.33 | 793.4082 | 8 | 1.1 | 397.7118 | 2 | 47.29 | 5 | F5:13639 | 29102019\_RID\_1313\_NaNaPb\_F4.raw |  |  |  |  | 8.05E5 |  |  |  |  |  | 1 | 0 | 0 | 0 | 0 | 1 | 0 | 0 | 0 | 0 | 0 | 47 | 54 |  |
| R.NFSSC(+57.02)SAVAPK.T | N | 46.18 | 1166.5389 | 11 | 0.0 | 584.2767 | 2 | 39.84 | 5 | F5:8274 | 29102019\_RID\_1313\_NaNaPb\_F4.raw |  |  |  |  | 1.68E5 |  |  |  |  |  | 1 | 0 | 0 | 0 | 0 | 1 | 0 | 0 | 0 | 0 | 0 | 17 | 27 | Carbamidomethylation |
| total 29 peptides |
| --- |

P25668|3L21\_NAJNA

back to list

  

| Protein Coverage
| Supporting Peptides
|

Protein Coverage:

Supporting Peptides:

| Peptide | Uniq | -10lgP | Mass | Length | ppm | m/z | z | RT | Fraction | Scan | Source File | Area F1 | Area F10 | Area F2 | Area F3 | Area F4 | Area F5 | Area F6 | Area F7 | Area F8 | Area F9 | #Feature | #Feature F1 | #Feature F10 | #Feature F2 | #Feature F3 | #Feature F4 | #Feature F5 | #Feature F6 | #Feature F7 | #Feature F8 | #Feature F9 | Start | End | PTM |
| --- | --- | --- | --- | --- | --- | --- | --- | --- | --- | --- | --- | --- | --- | --- | --- | --- | --- | --- | --- | --- | --- | --- | --- | --- | --- | --- | --- | --- | --- | --- | --- | --- | --- | --- | --- |
| R.C(+57.02)FITPDITSKDC(+57.02)PNGHVC(+57.02)YTK.T | N | 108.82 | 2512.1184 | 21 | -1.1 | 838.3792 | 3 | 11.64 | 7 | F7:2478 | 29102019\_RID\_1313\_NaNaPb\_F6.raw |  |  |  |  | 3.29E8 |  | 5.17E6 |  |  |  | 8 | 0 | 0 | 0 | 0 | 6 | 0 | 2 | 0 | 0 | 0 | 3 | 23 | Carbamidomethylation |
| R.VDLGC(+57.02)AATC(+57.02)PTVR.T | N | 108.71 | 1418.6646 | 13 | 1.0 | 710.3403 | 2 | 12.11 | 7 | F7:2909 | 29102019\_RID\_1313\_NaNaPb\_F6.raw |  | 1.21E6 |  |  | 2.34E8 | 1.19E7 | 4.59E8 | 3.09E4 | 1.2E6 | 1.6E6 | 42 | 0 | 1 | 0 | 0 | 3 | 2 | 33 | 1 | 1 | 1 | 37 | 49 | Carbamidomethylation |
| K.RVDLGC(+57.02)AATC(+57.02)PTVR.T | N | 102.24 | 1574.7657 | 14 | -0.6 | 788.3896 | 2 | 11.34 | 7 | F7:2170 | 29102019\_RID\_1313\_NaNaPb\_F6.raw |  |  |  |  | 1E9 |  | 2.54E7 |  |  |  | 11 | 0 | 0 | 0 | 0 | 8 | 0 | 3 | 0 | 0 | 0 | 36 | 49 | Carbamidomethylation |
| IRC(+57.02)FITPDITSKDC(+57.02)PNGHVC(+57.02)YTK.T | N | 99.90 | 2781.3037 | 23 | 1.5 | 696.3342 | 4 | 49.09 | 5 | F5:14964 | 29102019\_RID\_1313\_NaNaPb\_F4.raw |  |  |  |  | 1.1E9 |  |  |  |  |  | 8 | 0 | 0 | 0 | 0 | 8 | 0 | 0 | 0 | 0 | 0 | 1 | 23 | Carbamidomethylation |
| IRC(+57.02)FITPDITSKDC(+57.02)PNGHVC(+57.02)YTKTWC(+57.02)DGFC(+57.02)SIR.G | Y | 89.63 | 4063.8259 | 33 | -2.9 | 1016.9608 | 4 | 61.25 | 5 | F5:25098 | 29102019\_RID\_1313\_NaNaPb\_F4.raw |  |  |  |  | 6.64E7 |  |  |  |  |  | 3 | 0 | 0 | 0 | 0 | 3 | 0 | 0 | 0 | 0 | 0 | 1 | 33 | Carbamidomethylation |
| K.DC(+57.02)PNGHVC(+57.02)YTK.T | N | 84.60 | 1349.5493 | 11 | -0.6 | 675.7816 | 2 | 10.69 | 7 | F7:1588 | 29102019\_RID\_1313\_NaNaPb\_F6.raw |  |  |  |  |  |  | 0 |  |  |  | 2 | 0 | 0 | 0 | 0 | 0 | 0 | 2 | 0 | 0 | 0 | 13 | 23 | Carbamidomethylation |
| R.C(+57.02)FITPDITSK.D | N | 83.01 | 1180.5798 | 10 | 0.0 | 591.2972 | 2 | 13.12 | 7 | F7:3684 | 29102019\_RID\_1313\_NaNaPb\_F6.raw |  | 1.15E6 |  | 4.39E4 | 7.16E7 |  | 4.95E8 | 7.19E4 | 1.97E6 | 1.33E6 | 28 | 0 | 1 | 0 | 1 | 1 | 0 | 22 | 1 | 1 | 1 | 3 | 12 | Carbamidomethylation |
| R.GKRVDLGC(+57.02)AATC(+57.02)PTVR.T | N | 79.56 | 1759.8821 | 16 | 0.9 | 587.6352 | 3 | 37.79 | 5 | F5:6637 | 29102019\_RID\_1313\_NaNaPb\_F4.raw |  |  |  |  | 9.04E7 |  | 1.39E5 |  |  |  | 3 | 0 | 0 | 0 | 0 | 2 | 0 | 1 | 0 | 0 | 0 | 34 | 49 | Carbamidomethylation |
| C.PNGHVC(+57.02)YTK.T | N | 76.22 | 1074.4917 | 9 | -2.6 | 538.2517 | 2 | 10.69 | 7 | F7:1590 | 29102019\_RID\_1313\_NaNaPb\_F6.raw |  |  |  |  |  |  | 2.55E6 |  |  |  | 1 | 0 | 0 | 0 | 0 | 0 | 0 | 1 | 0 | 0 | 0 | 15 | 23 | Carbamidomethylation |
| K.TWC(+57.02)DGFC(+57.02)SIRGK.R | Y | 74.29 | 1485.6493 | 12 | -0.8 | 496.2233 | 3 | 54.65 | 5 | F5:19406 | 29102019\_RID\_1313\_NaNaPb\_F4.raw |  |  |  |  | 1.91E8 |  |  |  |  |  | 4 | 0 | 0 | 0 | 0 | 4 | 0 | 0 | 0 | 0 | 0 | 24 | 35 | Carbamidomethylation |
| IRC(+57.02)FITPDITSK.D | N | 72.29 | 1449.7650 | 12 | 0.4 | 725.8901 | 2 | 54.58 | 5 | F5:19303 | 29102019\_RID\_1313\_NaNaPb\_F4.raw |  |  |  |  | 2.93E8 |  | 3.64E5 |  |  |  | 6 | 0 | 0 | 0 | 0 | 5 | 0 | 1 | 0 | 0 | 0 | 1 | 12 | Carbamidomethylation |
| K.TWC(+57.02)DGFC(+57.02)SIR.G | Y | 65.10 | 1300.5328 | 10 | 0.6 | 651.2741 | 2 | 14.99 | 7 | F7:4861 | 29102019\_RID\_1313\_NaNaPb\_F6.raw |  | 1.17E5 |  |  | 6.74E8 | 1.09E7 | 6.16E8 | 4.46E4 |  |  | 25 | 0 | 1 | 0 | 0 | 4 | 2 | 17 | 1 | 0 | 0 | 24 | 33 | Carbamidomethylation |
| R.C(+57.02)FITPDITSKDC(+57.02)PNGHVC(+57.02)YTKTWC(+57.02)DGFC(+57.02)SIR.G | Y | 63.13 | 3794.6409 | 31 | 2.4 | 759.9373 | 5 | 63.77 | 5 | F5:27275 | 29102019\_RID\_1313\_NaNaPb\_F4.raw |  |  |  |  | 4.8E6 |  |  |  |  |  | 1 | 0 | 0 | 0 | 0 | 1 | 0 | 0 | 0 | 0 | 0 | 3 | 33 | Carbamidomethylation |
| L.GC(+57.02)AATC(+57.02)PTVR.T | N | 62.53 | 1091.4852 | 10 | 0.0 | 546.7499 | 2 | 10.76 | 7 | F7:1638 | 29102019\_RID\_1313\_NaNaPb\_F6.raw |  |  |  |  |  |  | 4.59E5 |  |  |  | 1 | 0 | 0 | 0 | 0 | 0 | 0 | 1 | 0 | 0 | 0 | 40 | 49 | Carbamidomethylation |
| D.LGC(+57.02)AATC(+57.02)PTVR.T | N | 61.18 | 1204.5692 | 11 | 1.0 | 603.2925 | 2 | 11.12 | 7 | F7:2008 | 29102019\_RID\_1313\_NaNaPb\_F6.raw |  |  |  |  | 2.52E5 |  | 3.46E5 |  |  |  | 3 | 0 | 0 | 0 | 0 | 2 | 0 | 1 | 0 | 0 | 0 | 39 | 49 | Carbamidomethylation |
| K.RVDLGC(+57.02)AATC(+57.02)PT.V | N | 61.14 | 1319.5962 | 12 | -1.8 | 660.8042 | 2 | 11.42 | 7 | F7:2282 | 29102019\_RID\_1313\_NaNaPb\_F6.raw |  |  |  |  |  |  | 5.44E5 |  |  |  | 1 | 0 | 0 | 0 | 0 | 0 | 0 | 1 | 0 | 0 | 0 | 36 | 47 | Carbamidomethylation |
| R.VDLGC(+57.02)AATC(+57.02)PT.V | N | 59.60 | 1163.4951 | 11 | 0.0 | 582.7548 | 2 | 11.91 | 7 | F7:2756 | 29102019\_RID\_1313\_NaNaPb\_F6.raw |  |  |  |  |  |  | 8.06E5 |  |  |  | 1 | 0 | 0 | 0 | 0 | 0 | 0 | 1 | 0 | 0 | 0 | 37 | 47 | Carbamidomethylation |
| R.VDLGC(+57.02)AATC(+57.02)PTV.R | N | 57.22 | 1262.5635 | 12 | -0.8 | 632.2885 | 2 | 13.91 | 7 | F7:4209 | 29102019\_RID\_1313\_NaNaPb\_F6.raw |  |  |  |  |  |  | 0 |  |  |  | 1 | 0 | 0 | 0 | 0 | 0 | 0 | 1 | 0 | 0 | 0 | 37 | 48 | Carbamidomethylation |
| K.DC(+57.02)PNGHVC(+57.02)Y.T | N | 54.83 | 1120.4066 | 9 | 0.8 | 561.2111 | 2 | 11.17 | 7 | F7:2028 | 29102019\_RID\_1313\_NaNaPb\_F6.raw |  |  |  |  |  |  | 5.79E6 |  |  |  | 1 | 0 | 0 | 0 | 0 | 0 | 0 | 1 | 0 | 0 | 0 | 13 | 21 | Carbamidomethylation |
| R.VDLGC(+57.02)AATC(+57.02)P.T | N | 54.00 | 1062.4474 | 10 | 1.1 | 532.2316 | 2 | 12.13 | 7 | F7:2931 | 29102019\_RID\_1313\_NaNaPb\_F6.raw |  |  |  |  |  |  | 1.03E5 |  |  |  | 1 | 0 | 0 | 0 | 0 | 0 | 0 | 1 | 0 | 0 | 0 | 37 | 46 | Carbamidomethylation |
| R.TGVDIQC(+57.02)C(+57.02).S | N | 53.10 | 951.3790 | 8 | -0.3 | 476.6967 | 2 | 11.71 | 7 | F7:2547 | 29102019\_RID\_1313\_NaNaPb\_F6.raw |  |  |  |  |  |  | 1.32E6 |  |  |  | 1 | 0 | 0 | 0 | 0 | 0 | 0 | 1 | 0 | 0 | 0 | 50 | 57 | Carbamidomethylation |
| IRC(+57.02)FITPDITSKD.C | N | 46.76 | 1564.7919 | 13 | 0.1 | 783.4033 | 2 | 55.56 | 5 | F5:20289 | 29102019\_RID\_1313\_NaNaPb\_F4.raw |  |  |  |  | 7.27E6 |  |  |  |  |  | 2 | 0 | 0 | 0 | 0 | 2 | 0 | 0 | 0 | 0 | 0 | 1 | 13 | Carbamidomethylation |
| R.C(+57.02)FITPDITS.K | N | 46.63 | 1052.4849 | 9 | 0.1 | 527.2498 | 2 | 20.20 | 7 | F7:7324 | 29102019\_RID\_1313\_NaNaPb\_F6.raw |  |  |  |  |  |  | 2.43E4 |  |  |  | 1 | 0 | 0 | 0 | 0 | 0 | 0 | 1 | 0 | 0 | 0 | 3 | 11 | Carbamidomethylation |
| T.WC(+57.02)DGFC(+57.02)SIR.G | Y | 43.13 | 1199.4852 | 9 | 0.2 | 600.7500 | 2 | 12.27 | 7 | F7:3036 | 29102019\_RID\_1313\_NaNaPb\_F6.raw |  |  |  |  |  |  | 1.05E5 |  |  |  | 1 | 0 | 0 | 0 | 0 | 0 | 0 | 1 | 0 | 0 | 0 | 25 | 33 | Carbamidomethylation |
| C.FITPDITSK.D | N | 42.13 | 1020.5491 | 9 | -2.4 | 511.2806 | 2 | 12.04 | 7 | F7:2836 | 29102019\_RID\_1313\_NaNaPb\_F6.raw |  |  |  |  |  |  | 2.76E5 |  |  |  | 1 | 0 | 0 | 0 | 0 | 0 | 0 | 1 | 0 | 0 | 0 | 4 | 12 |  |
| total 25 peptides |
| --- |

P82463|3SUC2\_NAJKA

back to list

  

| Protein Coverage
| Supporting Peptides
|

Protein Coverage:

Supporting Peptides:

| Peptide | Uniq | -10lgP | Mass | Length | ppm | m/z | z | RT | Fraction | Scan | Source File | Area F1 | Area F10 | Area F2 | Area F3 | Area F4 | Area F5 | Area F6 | Area F7 | Area F8 | Area F9 | #Feature | #Feature F1 | #Feature F10 | #Feature F2 | #Feature F3 | #Feature F4 | #Feature F5 | #Feature F6 | #Feature F7 | #Feature F8 | #Feature F9 | Start | End | PTM |
| --- | --- | --- | --- | --- | --- | --- | --- | --- | --- | --- | --- | --- | --- | --- | --- | --- | --- | --- | --- | --- | --- | --- | --- | --- | --- | --- | --- | --- | --- | --- | --- | --- | --- | --- | --- |
| K.SIFGVTTEDC(+57.02)PDGQNLC(+57.02)FK.R | Y | 129.34 | 2186.9612 | 19 | 0.7 | 1094.4886 | 2 | 20.29 | 7 | F7:7371 | 29102019\_RID\_1313\_NaNaPb\_F6.raw |  |  |  |  |  | 3.28E4 | 4.78E8 |  |  |  | 19 | 0 | 0 | 0 | 0 | 0 | 1 | 18 | 0 | 0 | 0 | 8 | 26 | Carbamidomethylation |
| F.GVTTEDC(+57.02)PDGQNLC(+57.02)FK.R | Y | 110.95 | 1839.7767 | 16 | 0.6 | 920.8962 | 2 | 11.76 | 7 | F7:2597 | 29102019\_RID\_1313\_NaNaPb\_F6.raw |  |  |  |  |  |  | 1.55E6 |  |  |  | 1 | 0 | 0 | 0 | 0 | 0 | 0 | 1 | 0 | 0 | 0 | 11 | 26 | Carbamidomethylation |
| K.SIFGVTTEDC(+57.02)PDGQNLC(+57.02)FKR.W | Y | 105.78 | 2343.0623 | 20 | 0.5 | 782.0284 | 3 | 12.89 | 7 | F7:3436 | 29102019\_RID\_1313\_NaNaPb\_F6.raw |  |  |  |  |  |  | 7.18E6 |  |  |  | 3 | 0 | 0 | 0 | 0 | 0 | 0 | 3 | 0 | 0 | 0 | 8 | 27 | Carbamidomethylation |
| R.GC(+57.02)AATC(+57.02)PIAENR.D | Y | 84.20 | 1318.5758 | 12 | -2.1 | 660.2938 | 2 | 11.05 | 7 | F7:1898 | 29102019\_RID\_1313\_NaNaPb\_F6.raw |  |  |  |  |  |  | 2.93E7 |  |  |  | 2 | 0 | 0 | 0 | 0 | 0 | 0 | 2 | 0 | 0 | 0 | 41 | 52 | Carbamidomethylation |
| R.GC(+57.02)AATC(+57.02)PIAENRDVIEC(+57.02)C(+57.02)STDKC(+57.02)NL | Y | 83.79 | 2913.2183 | 25 | -2.1 | 972.0780 | 3 | 11.73 | 7 | F7:2594 | 29102019\_RID\_1313\_NaNaPb\_F6.raw |  |  |  |  |  |  | 9.73E5 |  |  |  | 1 | 0 | 0 | 0 | 0 | 0 | 0 | 1 | 0 | 0 | 0 | 41 | 65 | Carbamidomethylation |
| R.DVIEC(+57.02)C(+57.02)STDK.C | Y | 83.53 | 1225.4955 | 10 | -1.9 | 613.7538 | 2 | 11.05 | 7 | F7:1920 | 29102019\_RID\_1313\_NaNaPb\_F6.raw |  |  |  |  |  |  | 1.35E7 |  |  |  | 1 | 0 | 0 | 0 | 0 | 0 | 0 | 1 | 0 | 0 | 0 | 53 | 62 | Carbamidomethylation |
| K.RWHMIVPGR.Y | Y | 78.50 | 1150.6182 | 9 | -0.9 | 384.5463 | 3 | 11.42 | 7 | F7:2287 | 29102019\_RID\_1313\_NaNaPb\_F6.raw |  |  |  |  |  |  | 2.35E5 |  |  |  | 1 | 0 | 0 | 0 | 0 | 0 | 0 | 1 | 0 | 0 | 0 | 27 | 35 |  |
| R.WHMIVPGR.Y | Y | 76.17 | 994.5171 | 8 | -0.6 | 498.2655 | 2 | 11.78 | 7 | F7:2614 | 29102019\_RID\_1313\_NaNaPb\_F6.raw |  |  |  |  |  |  | 3.24E6 |  |  |  | 1 | 0 | 0 | 0 | 0 | 0 | 0 | 1 | 0 | 0 | 0 | 28 | 35 |  |
| R.GC(+57.02)AATC(+57.02)PIAENRDVIEC(+57.02)C(+57.02)STDK.C | Y | 75.31 | 2526.0608 | 22 | 0.2 | 843.0277 | 3 | 11.47 | 7 | F7:2337 | 29102019\_RID\_1313\_NaNaPb\_F6.raw |  |  |  |  |  |  | 6.82E4 |  |  |  | 1 | 0 | 0 | 0 | 0 | 0 | 0 | 1 | 0 | 0 | 0 | 41 | 62 | Carbamidomethylation |
| D.C(+57.02)PDGQNLC(+57.02)FK.R | Y | 72.70 | 1237.5220 | 10 | 0.3 | 619.7684 | 2 | 11.49 | 7 | F7:2370 | 29102019\_RID\_1313\_NaNaPb\_F6.raw |  |  |  |  |  |  | 3.08E5 |  |  |  | 1 | 0 | 0 | 0 | 0 | 0 | 0 | 1 | 0 | 0 | 0 | 17 | 26 | Carbamidomethylation |
| R.WHM(+15.99)IVPGR.Y | Y | 70.50 | 1010.5120 | 8 | 0.6 | 506.2636 | 2 | 11.25 | 7 | F7:2112 | 29102019\_RID\_1313\_NaNaPb\_F6.raw |  |  |  |  |  |  | 5.48E5 |  |  |  | 1 | 0 | 0 | 0 | 0 | 0 | 0 | 1 | 0 | 0 | 0 | 28 | 35 | Oxidation (M) |
| K.EKSIFGVTTEDC(+57.02)PDGQNLC(+57.02)FK.R | Y | 66.52 | 2444.0989 | 21 | 0.6 | 815.7074 | 3 | 13.65 | 7 | F7:4086 | 29102019\_RID\_1313\_NaNaPb\_F6.raw |  |  |  |  |  |  | 1.36E5 |  |  |  | 1 | 0 | 0 | 0 | 0 | 0 | 0 | 1 | 0 | 0 | 0 | 6 | 26 | Carbamidomethylation |
| K.TRGC(+57.02)AATC(+57.02)PIAENR.D | Y | 65.42 | 1575.7246 | 14 | -1.3 | 526.2481 | 3 | 10.74 | 7 | F7:1654 | 29102019\_RID\_1313\_NaNaPb\_F6.raw |  |  |  |  |  |  | 8.35E4 |  |  |  | 1 | 0 | 0 | 0 | 0 | 0 | 0 | 1 | 0 | 0 | 0 | 39 | 52 | Carbamidomethylation |
| C.AATC(+57.02)PIAENR.D | Y | 64.71 | 1101.5237 | 10 | -0.2 | 551.7690 | 2 | 10.94 | 7 | F7:1793 | 29102019\_RID\_1313\_NaNaPb\_F6.raw |  |  |  |  |  |  | 1.34E5 |  |  |  | 1 | 0 | 0 | 0 | 0 | 0 | 0 | 1 | 0 | 0 | 0 | 43 | 52 | Carbamidomethylation |
| R.DVIEC(+57.02)C(+57.02)STDKC(+57.02)NL | Y | 61.27 | 1612.6531 | 13 | 0.3 | 807.3340 | 2 | 11.64 | 7 | F7:2486 | 29102019\_RID\_1313\_NaNaPb\_F6.raw |  |  |  |  |  |  | 1.52E5 |  |  |  | 1 | 0 | 0 | 0 | 0 | 0 | 0 | 1 | 0 | 0 | 0 | 53 | 65 | Carbamidomethylation |
| K.RWHM(+15.99)IVPGR.Y | Y | 55.58 | 1166.6132 | 9 | 0.7 | 389.8786 | 3 | 11.01 | 7 | F7:1880 | 29102019\_RID\_1313\_NaNaPb\_F6.raw |  |  |  |  |  |  | 3.96E4 |  |  |  | 1 | 0 | 0 | 0 | 0 | 0 | 0 | 1 | 0 | 0 | 0 | 27 | 35 | Oxidation (M) |
| G.C(+57.02)AATC(+57.02)PIAENR.D | Y | 52.63 | 1261.5543 | 11 | 1.0 | 631.7851 | 2 | 11.03 | 7 | F7:1903 | 29102019\_RID\_1313\_NaNaPb\_F6.raw |  |  |  |  |  |  | 3.41E4 |  |  |  | 1 | 0 | 0 | 0 | 0 | 0 | 0 | 1 | 0 | 0 | 0 | 42 | 52 | Carbamidomethylation |
| K.SIFGVTTEDC(+57.02)PD.G | Y | 46.74 | 1339.5602 | 12 | 0.5 | 670.7877 | 2 | 19.83 | 7 | F7:7189 | 29102019\_RID\_1313\_NaNaPb\_F6.raw |  |  |  |  |  |  | 3.3E5 |  |  |  | 1 | 0 | 0 | 0 | 0 | 0 | 0 | 1 | 0 | 0 | 0 | 8 | 19 | Carbamidomethylation |
| total 18 peptides |
| --- |

P13647|K2C5\_HUMAN

back to list

  

| Protein Coverage
| Supporting Peptides
|

Protein Coverage:

Supporting Peptides:

| Peptide | Uniq | -10lgP | Mass | Length | ppm | m/z | z | RT | Fraction | Scan | Source File | Area F1 | Area F10 | Area F2 | Area F3 | Area F4 | Area F5 | Area F6 | Area F7 | Area F8 | Area F9 | #Feature | #Feature F1 | #Feature F10 | #Feature F2 | #Feature F3 | #Feature F4 | #Feature F5 | #Feature F6 | #Feature F7 | #Feature F8 | #Feature F9 | Start | End | PTM |
| --- | --- | --- | --- | --- | --- | --- | --- | --- | --- | --- | --- | --- | --- | --- | --- | --- | --- | --- | --- | --- | --- | --- | --- | --- | --- | --- | --- | --- | --- | --- | --- | --- | --- | --- | --- |
| R.GLGVGFGSGGGSSSSVKFVSTTSSSRK.S | Y | 95.23 | 2519.2615 | 27 | -0.1 | 840.7610 | 3 | 50.82 | 5 | F5:16333 | 29102019\_RID\_1313\_NaNaPb\_F4.raw |  |  |  |  | 9.36E5 |  |  |  |  |  | 1 | 0 | 0 | 0 | 0 | 1 | 0 | 0 | 0 | 0 | 0 | 560 | 586 |  |
| R.SGGSRSFSTASAITPSVSR.T | Y | 91.88 | 1853.9231 | 19 | -0.6 | 618.9813 | 3 | 45.72 | 5 | F5:12541 | 29102019\_RID\_1313\_NaNaPb\_F4.raw |  |  |  |  | 6.34E6 |  |  |  |  |  | 2 | 0 | 0 | 0 | 0 | 2 | 0 | 0 | 0 | 0 | 0 | 11 | 29 |  |
| R.GLGVGFGSGGGSSSSVKFVSTTSSSR.K | Y | 88.78 | 2391.1665 | 26 | 2.0 | 1196.5929 | 2 | 57.05 | 5 | F5:21563 | 29102019\_RID\_1313\_NaNaPb\_F4.raw |  |  |  |  | 3.19E5 |  |  |  |  |  | 1 | 0 | 0 | 0 | 0 | 1 | 0 | 0 | 0 | 0 | 0 | 560 | 585 |  |
| R.VSLAGAC(+57.02)GVGGYGSR.S | Y | 88.04 | 1409.6721 | 15 | 0.1 | 705.8434 | 2 | 11.88 | 3 | F3:2704 | 29102019\_RID\_1313\_NaNaPb\_F2.raw | 4.53E4 | 5.44E4 | 1.01E5 |  |  | 1.38E5 |  |  |  |  | 4 | 1 | 1 | 1 | 0 | 0 | 1 | 0 | 0 | 0 | 0 | 49 | 63 | Carbamidomethylation |
| R.SLYNLGGSKRISISTSGGSFR.N | Y | 83.78 | 2186.1443 | 21 | 0.2 | 547.5435 | 4 | 54.85 | 5 | F5:19686 | 29102019\_RID\_1313\_NaNaPb\_F4.raw |  |  |  |  | 4.88E5 |  |  |  |  |  | 1 | 0 | 0 | 0 | 0 | 1 | 0 | 0 | 0 | 0 | 0 | 64 | 84 |  |
| R.NKLAELEEALQKAKQDMAR.L | Y | 81.78 | 2185.1523 | 19 | 0.8 | 547.2958 | 4 | 68.14 | 5 | F5:31067 | 29102019\_RID\_1313\_NaNaPb\_F4.raw |  |  |  |  | 7.35E4 |  |  |  |  |  | 1 | 0 | 0 | 0 | 0 | 1 | 0 | 0 | 0 | 0 | 0 | 430 | 448 |  |
| K.WTLLQEQGTK.T | N | 80.50 | 1202.6295 | 10 | -0.5 | 602.3217 | 2 | 12.43 | 7 | F7:3159 | 29102019\_RID\_1313\_NaNaPb\_F6.raw |  |  | 2.43E5 |  |  |  | 2.98E5 |  |  |  | 2 | 0 | 0 | 1 | 0 | 0 | 0 | 1 | 0 | 0 | 0 | 200 | 209 |  |
| R.NLDLDSIIAEVK.A | N | 79.85 | 1328.7188 | 12 | 0.3 | 665.3669 | 2 | 34.39 | 9 | F9:13181 | 29102019\_RID\_1313\_NaNaPb\_F8.raw | 6.3E5 | 2.03E6 | 4.86E5 | 7.73E4 |  | 1.95E6 | 1.33E6 |  | 3.92E5 | 1.4E5 | 9 | 2 | 1 | 1 | 1 | 0 | 1 | 1 | 0 | 1 | 1 | 332 | 343 |  |
| K.TLNNKFASFIDKVR.F | N | 77.96 | 1651.9045 | 14 | 0.2 | 413.9835 | 4 | 59.92 | 5 | F5:23986 | 29102019\_RID\_1313\_NaNaPb\_F4.raw |  |  |  |  | 1.32E6 |  |  |  |  |  | 2 | 0 | 0 | 0 | 0 | 2 | 0 | 0 | 0 | 0 | 0 | 174 | 187 |  |
| R.FLEQQNKVLDTKWTLLQEQGTK.T | N | 76.77 | 2646.4016 | 22 | 0.9 | 883.1420 | 3 | 63.86 | 5 | F5:27315 | 29102019\_RID\_1313\_NaNaPb\_F4.raw |  |  |  |  | 2.59E6 |  |  |  |  |  | 1 | 0 | 0 | 0 | 0 | 1 | 0 | 0 | 0 | 0 | 0 | 188 | 209 |  |
| K.LAELEEALQK.A | Y | 75.11 | 1142.6183 | 10 | 0.8 | 572.3169 | 2 | 13.18 | 1 | F1:4244 | 29102019\_RID\_1313\_NaNaPb\_F1.raw | 1.22E5 | 1.5E5 | 2.6E5 |  |  | 3.1E5 |  |  |  | 5.04E4 | 5 | 1 | 1 | 1 | 0 | 0 | 1 | 0 | 0 | 0 | 1 | 432 | 441 |  |
| K.LALDVEIATYR.K | N | 72.02 | 1262.6870 | 11 | 1.0 | 632.3514 | 2 | 18.69 | 2 | F2:6620 | 29102019\_RID\_1313\_NaNaPb\_F10.raw | 1.06E5 | 5.46E5 | 3.1E4 | 1.25E4 |  | 1.24E6 | 1.83E5 |  |  |  | 8 | 1 | 2 | 1 | 1 | 0 | 2 | 1 | 0 | 0 | 0 | 461 | 471 |  |
| K.QC(+57.02)ANLQNAIADAEQR.G | N | 71.30 | 1700.7900 | 15 | 0.8 | 567.9377 | 3 | 12.55 | 1 | F1:3961 | 29102019\_RID\_1313\_NaNaPb\_F1.raw | 6.94E3 |  | 5.18E4 |  |  |  |  |  |  |  | 4 | 1 | 0 | 3 | 0 | 0 | 0 | 0 | 0 | 0 | 0 | 406 | 420 | Carbamidomethylation |
| R.SFSTASAITPSVSR.T | N | 70.67 | 1409.7151 | 14 | 0.1 | 705.8649 | 2 | 53.28 | 5 | F5:18398 | 29102019\_RID\_1313\_NaNaPb\_F4.raw | 2.12E4 |  | 5.26E4 |  | 1.43E7 | 3.74E4 |  |  |  |  | 4 | 1 | 0 | 1 | 0 | 1 | 1 | 0 | 0 | 0 | 0 | 16 | 29 |  |
| R.ISISTSGGSFR.N | Y | 66.11 | 1110.5669 | 11 | 0.2 | 556.2908 | 2 | 52.40 | 5 | F5:17582 | 29102019\_RID\_1313\_NaNaPb\_F4.raw | 6.82E4 | 8.82E4 | 1.72E5 |  | 8.33E6 | 1.47E5 |  |  |  |  | 5 | 1 | 1 | 1 | 0 | 1 | 1 | 0 | 0 | 0 | 0 | 74 | 84 |  |
| R.SLYNLGGSKR.I | N | 64.24 | 1093.5880 | 10 | 0.5 | 547.8016 | 2 | 40.93 | 5 | F5:9053 | 29102019\_RID\_1313\_NaNaPb\_F4.raw |  |  |  |  | 9.06E5 |  |  |  |  |  | 1 | 0 | 0 | 0 | 0 | 1 | 0 | 0 | 0 | 0 | 0 | 64 | 73 |  |
| K.RISISTSGGSFR.N | Y | 64.12 | 1266.6680 | 12 | -0.5 | 423.2297 | 3 | 44.58 | 5 | F5:11845 | 29102019\_RID\_1313\_NaNaPb\_F4.raw |  |  |  |  | 9.09E4 |  |  |  |  |  | 1 | 0 | 0 | 0 | 0 | 1 | 0 | 0 | 0 | 0 | 0 | 73 | 84 |  |
| R.TEAESWYQTK.Y | N | 63.80 | 1241.5564 | 10 | 0.5 | 621.7858 | 2 | 11.42 | 2 | F2:2369 | 29102019\_RID\_1313\_NaNaPb\_F10.raw |  | 2.9E4 | 8.16E4 |  |  |  |  |  |  |  | 2 | 0 | 1 | 1 | 0 | 0 | 0 | 0 | 0 | 0 | 0 | 355 | 364 |  |
| R.QLDSIVGER.G | N | 62.07 | 1015.5298 | 9 | 0.3 | 508.7723 | 2 | 11.95 | 1 | F1:3454 | 29102019\_RID\_1313\_NaNaPb\_F1.raw | 3.34E4 | 7.21E4 | 9.99E4 |  |  |  |  |  |  |  | 3 | 1 | 1 | 1 | 0 | 0 | 0 | 0 | 0 | 0 | 0 | 229 | 237 |  |
| K.YEELQQTAGR.H | N | 60.66 | 1193.5676 | 10 | 0.2 | 597.7912 | 2 | 11.23 | 3 | F3:2060 | 29102019\_RID\_1313\_NaNaPb\_F2.raw | 6.18E3 |  | 4.87E4 |  |  |  |  |  |  |  | 2 | 1 | 0 | 1 | 0 | 0 | 0 | 0 | 0 | 0 | 0 | 365 | 374 |  |
| R.QNLEPLFEQYINNLR.R | N | 59.91 | 1889.9635 | 15 | 0.4 | 630.9954 | 3 | 39.85 | 9 | F9:15005 | 29102019\_RID\_1313\_NaNaPb\_F8.raw |  | 1.19E5 |  |  |  |  | 1.03E5 |  | 1.04E4 |  | 3 | 0 | 1 | 0 | 0 | 0 | 0 | 1 | 0 | 1 | 0 | 213 | 227 |  |
| K.NKYEDEINKR.T | N | 59.85 | 1307.6470 | 10 | -0.3 | 436.8895 | 3 | 10.75 | 6 | F6:1631 | 29102019\_RID\_1313\_NaNaPb\_F5.raw |  | 2.17E5 | 1.52E5 |  |  | 3.47E4 | 2.69E5 |  |  |  | 4 | 0 | 1 | 1 | 0 | 0 | 1 | 1 | 0 | 0 | 0 | 256 | 265 |  |
| R.TTAENEFVMLK.K | N | 59.71 | 1281.6274 | 11 | 0.8 | 641.8215 | 2 | 15.79 | 1 | F1:5150 | 29102019\_RID\_1313\_NaNaPb\_F1.raw | 1.96E4 | 1.75E4 |  |  |  |  |  |  |  |  | 2 | 1 | 1 | 0 | 0 | 0 | 0 | 0 | 0 | 0 | 0 | 266 | 276 |  |
| K.AQYEEIANR.S | N | 58.70 | 1092.5199 | 9 | 0.0 | 547.2672 | 2 | 11.31 | 3 | F3:2130 | 29102019\_RID\_1313\_NaNaPb\_F2.raw |  |  | 5.96E4 |  | 3.71E5 |  |  |  |  |  | 2 | 0 | 0 | 1 | 0 | 1 | 0 | 0 | 0 | 0 | 0 | 344 | 352 |  |
| R.ISISTSGGSFRNR.F | Y | 54.94 | 1380.7109 | 13 | 0.1 | 461.2443 | 3 | 42.83 | 5 | F5:10548 | 29102019\_RID\_1313\_NaNaPb\_F4.raw |  |  |  |  | 6.73E5 |  |  |  |  |  | 1 | 0 | 0 | 0 | 0 | 1 | 0 | 0 | 0 | 0 | 0 | 74 | 86 |  |
| K.LLEGEEC(+57.02)R.L | N | 53.11 | 1004.4597 | 8 | 0.4 | 503.2373 | 2 | 11.25 | 3 | F3:2079 | 29102019\_RID\_1313\_NaNaPb\_F2.raw |  |  | 3.26E4 |  |  |  |  |  |  |  | 1 | 0 | 0 | 1 | 0 | 0 | 0 | 0 | 0 | 0 | 0 | 473 | 480 | Carbamidomethylation |
| R.TSFTSVSR.S | N | 52.73 | 883.4399 | 8 | -0.4 | 442.7271 | 2 | 39.84 | 5 | F5:8184 | 29102019\_RID\_1313\_NaNaPb\_F4.raw |  |  |  |  | 1.03E6 |  |  |  |  |  | 1 | 0 | 0 | 0 | 0 | 1 | 0 | 0 | 0 | 0 | 0 | 30 | 37 |  |
| R.NMQDLVEDFK.N | N | 50.91 | 1237.5648 | 10 | 0.8 | 619.7902 | 2 | 18.95 | 3 | F3:5017 | 29102019\_RID\_1313\_NaNaPb\_F2.raw |  |  | 3.8E4 |  |  |  |  |  |  |  | 1 | 0 | 0 | 1 | 0 | 0 | 0 | 0 | 0 | 0 | 0 | 246 | 255 |  |
| R.TTAENEFVM(+15.99)LK.K | N | 47.93 | 1297.6224 | 11 | 0.5 | 649.8188 | 2 | 12.37 | 3 | F3:3109 | 29102019\_RID\_1313\_NaNaPb\_F2.raw | 2.75E4 |  | 4.97E4 |  |  |  |  |  |  |  | 2 | 1 | 0 | 1 | 0 | 0 | 0 | 0 | 0 | 0 | 0 | 266 | 276 | Oxidation (M) |
| K.VDALM(+15.99)DEINFM(+15.99)K.M | N | 47.34 | 1456.6578 | 12 | 0.8 | 729.3368 | 2 | 13.04 | 6 | F6:3594 | 29102019\_RID\_1313\_NaNaPb\_F5.raw |  |  |  |  |  | 2.41E4 |  |  |  |  | 1 | 0 | 0 | 0 | 0 | 0 | 1 | 0 | 0 | 0 | 0 | 293 | 304 | Oxidation (M) |
| K.FASFIDK.V | N | 46.57 | 826.4225 | 7 | -0.3 | 414.2184 | 2 | 11.89 | 2 | F2:2810 | 29102019\_RID\_1313\_NaNaPb\_F10.raw |  | 1.96E5 |  |  |  | 3.22E5 |  |  | 0 |  | 3 | 0 | 1 | 0 | 0 | 0 | 1 | 0 | 0 | 1 | 0 | 179 | 185 |  |
| N.LQNAIADAEQR.G | N | 46.45 | 1227.6207 | 11 | 1.0 | 614.8182 | 2 | 11.52 | 3 | F3:2333 | 29102019\_RID\_1313\_NaNaPb\_F2.raw |  |  | 0 |  |  |  |  |  |  |  | 1 | 0 | 0 | 1 | 0 | 0 | 0 | 0 | 0 | 0 | 0 | 410 | 420 |  |
| total 32 peptides |
| --- |

O43790|KRT86\_HUMAN

back to list

  

| Protein Coverage
| Supporting Peptides
|

Protein Coverage:

Supporting Peptides:

| Peptide | Uniq | -10lgP | Mass | Length | ppm | m/z | z | RT | Fraction | Scan | Source File | Area F1 | Area F10 | Area F2 | Area F3 | Area F4 | Area F5 | Area F6 | Area F7 | Area F8 | Area F9 | #Feature | #Feature F1 | #Feature F10 | #Feature F2 | #Feature F3 | #Feature F4 | #Feature F5 | #Feature F6 | #Feature F7 | #Feature F8 | #Feature F9 | Start | End | PTM |
| --- | --- | --- | --- | --- | --- | --- | --- | --- | --- | --- | --- | --- | --- | --- | --- | --- | --- | --- | --- | --- | --- | --- | --- | --- | --- | --- | --- | --- | --- | --- | --- | --- | --- | --- | --- |
| K.LEAAVAQSEQQGEAALSDAR.C | Y | 111.87 | 2042.9868 | 20 | 1.2 | 1022.5020 | 2 | 58.62 | 5 | F5:22876 | 29102019\_RID\_1313\_NaNaPb\_F4.raw |  |  |  |  | 1.83E6 |  |  |  |  |  | 1 | 0 | 0 | 0 | 0 | 1 | 0 | 0 | 0 | 0 | 0 | 346 | 365 |  |
| R.FAAFIDKVRFLEQQNKLLETK.L | N | 95.67 | 2537.4004 | 21 | -0.3 | 846.8072 | 3 | 69.08 | 5 | F5:31940 | 29102019\_RID\_1313\_NaNaPb\_F4.raw |  |  |  |  | 2.99E7 |  |  |  |  |  | 3 | 0 | 0 | 0 | 0 | 3 | 0 | 0 | 0 | 0 | 0 | 117 | 137 |  |
| K.SLNSRFAAFIDKVR.F | N | 84.83 | 1622.8892 | 14 | 0.9 | 541.9708 | 3 | 64.24 | 5 | F5:27586 | 29102019\_RID\_1313\_NaNaPb\_F4.raw |  |  |  |  | 8.58E6 |  |  |  |  |  | 2 | 0 | 0 | 0 | 0 | 2 | 0 | 0 | 0 | 0 | 0 | 112 | 125 |  |
| R.LASELNHVQEVLEGYKK.K | N | 77.99 | 1956.0316 | 17 | 0.5 | 490.0154 | 4 | 62.56 | 5 | F5:26250 | 29102019\_RID\_1313\_NaNaPb\_F4.raw |  |  |  |  | 0 |  |  |  |  |  | 1 | 0 | 0 | 0 | 0 | 1 | 0 | 0 | 0 | 0 | 0 | 176 | 192 |  |
| R.MIQRLTAEVENAK.C | Y | 76.29 | 1501.7922 | 13 | 0.2 | 501.6048 | 3 | 46.71 | 5 | F5:13137 | 29102019\_RID\_1313\_NaNaPb\_F4.raw |  |  |  |  | 2.02E7 |  |  |  |  |  | 2 | 0 | 0 | 0 | 0 | 2 | 0 | 0 | 0 | 0 | 0 | 328 | 340 |  |
| R.TKEEINELNR.M | N | 76.25 | 1244.6360 | 10 | -0.1 | 623.3252 | 2 | 36.79 | 5 | F5:5864 | 29102019\_RID\_1313\_NaNaPb\_F4.raw |  |  |  |  | 1.11E7 |  |  |  |  |  | 2 | 0 | 0 | 0 | 0 | 2 | 0 | 0 | 0 | 0 | 0 | 318 | 327 |  |
| R.FLEQQNKLLETKLQFYQNR.E | N | 73.95 | 2439.2910 | 19 | -0.5 | 610.8297 | 4 | 63.53 | 5 | F5:27032 | 29102019\_RID\_1313\_NaNaPb\_F4.raw |  |  |  |  | 6.87E6 |  |  |  |  |  | 1 | 0 | 0 | 0 | 0 | 1 | 0 | 0 | 0 | 0 | 0 | 126 | 144 |  |
| R.VSSVPSNSNVVVGTTNAC(+57.02)APSAR.V | Y | 70.21 | 2273.1069 | 23 | 0.2 | 1137.5609 | 2 | 48.03 | 5 | F5:14168 | 29102019\_RID\_1313\_NaNaPb\_F4.raw |  |  |  |  | 9.92E5 |  |  |  |  |  | 2 | 0 | 0 | 0 | 0 | 2 | 0 | 0 | 0 | 0 | 0 | 453 | 475 | Carbamidomethylation |
| R.FLEQQNKLLETK.L | N | 69.75 | 1489.8140 | 12 | 0.7 | 745.9148 | 2 | 49.91 | 5 | F5:15503 | 29102019\_RID\_1313\_NaNaPb\_F4.raw |  |  |  |  | 8.24E7 |  |  |  |  |  | 3 | 0 | 0 | 0 | 0 | 3 | 0 | 0 | 0 | 0 | 0 | 126 | 137 |  |
| R.ATAENEFVALKK.D | N | 69.36 | 1319.7085 | 12 | -0.7 | 440.9098 | 3 | 46.39 | 5 | F5:12964 | 29102019\_RID\_1313\_NaNaPb\_F4.raw |  |  |  |  | 1.27E6 |  |  |  |  |  | 2 | 0 | 0 | 0 | 0 | 2 | 0 | 0 | 0 | 0 | 0 | 202 | 213 |  |
| K.LLETKLQFYQNR.E | N | 68.97 | 1551.8409 | 12 | -0.1 | 518.2875 | 3 | 56.33 | 5 | F5:20948 | 29102019\_RID\_1313\_NaNaPb\_F4.raw |  |  |  |  | 1.03E6 |  |  |  |  |  | 1 | 0 | 0 | 0 | 0 | 1 | 0 | 0 | 0 | 0 | 0 | 133 | 144 |  |
| K.LGLDIEIATYR.R | N | 68.96 | 1262.6870 | 11 | -0.1 | 632.3507 | 2 | 74.93 | 5 | F5:36761 | 29102019\_RID\_1313\_NaNaPb\_F4.raw |  |  |  |  | 1.73E5 |  |  |  |  |  | 1 | 0 | 0 | 0 | 0 | 1 | 0 | 0 | 0 | 0 | 0 | 397 | 407 |  |
| R.FAAFIDKVRFLEQQNK.L | N | 68.77 | 1953.0471 | 16 | -0.2 | 489.2690 | 4 | 65.30 | 5 | F5:28595 | 29102019\_RID\_1313\_NaNaPb\_F4.raw |  |  |  |  | 1.83E7 |  |  |  |  |  | 3 | 0 | 0 | 0 | 0 | 3 | 0 | 0 | 0 | 0 | 0 | 117 | 132 |  |
| R.SRAEAESWYR.S | N | 68.47 | 1253.5789 | 10 | -0.3 | 418.8668 | 3 | 41.96 | 5 | F5:9725 | 29102019\_RID\_1313\_NaNaPb\_F4.raw |  |  |  |  | 6.42E6 |  |  |  |  |  | 2 | 0 | 0 | 0 | 0 | 2 | 0 | 0 | 0 | 0 | 0 | 289 | 298 |  |
| K.LAELEGALQKAK.Q | N | 65.48 | 1269.7292 | 12 | -0.6 | 424.2501 | 3 | 48.27 | 5 | F5:14319 | 29102019\_RID\_1313\_NaNaPb\_F4.raw |  |  |  |  | 6E6 |  |  |  |  |  | 2 | 0 | 0 | 0 | 0 | 2 | 0 | 0 | 0 | 0 | 0 | 368 | 379 |  |
| R.LYEEEIRVLQSHISDTSVVVK.L | Y | 64.64 | 2443.2959 | 21 | -0.3 | 815.4390 | 3 | 70.44 | 5 | F5:32953 | 29102019\_RID\_1313\_NaNaPb\_F4.raw |  |  |  |  | 4.9E6 |  |  |  |  |  | 2 | 0 | 0 | 0 | 0 | 2 | 0 | 0 | 0 | 0 | 0 | 242 | 262 |  |
| R.KSDLEANVEALIQEIDFLR.R | N | 63.57 | 2202.1531 | 19 | 1.1 | 735.0591 | 3 | 100.24 | 5 | F5:57358 | 29102019\_RID\_1313\_NaNaPb\_F4.raw |  |  |  |  | 1.05E5 |  |  |  |  |  | 2 | 0 | 0 | 0 | 0 | 2 | 0 | 0 | 0 | 0 | 0 | 222 | 240 |  |
| R.M(+15.99)IQRLTAEVENAK.C | Y | 61.94 | 1517.7871 | 13 | 0.2 | 506.9364 | 3 | 46.71 | 5 | F5:13172 | 29102019\_RID\_1313\_NaNaPb\_F4.raw |  |  |  |  | 1.89E6 |  |  |  |  |  | 2 | 0 | 0 | 0 | 0 | 2 | 0 | 0 | 0 | 0 | 0 | 328 | 340 | Oxidation (M) |
| R.FAAFIDKVR.F | N | 61.47 | 1065.5970 | 9 | 0.0 | 533.8058 | 2 | 53.38 | 5 | F5:18300 | 29102019\_RID\_1313\_NaNaPb\_F4.raw |  |  |  |  | 1.48E8 |  |  |  |  |  | 1 | 0 | 0 | 0 | 0 | 1 | 0 | 0 | 0 | 0 | 0 | 117 | 125 |  |
| K.LAELEGALQK.A | N | 60.04 | 1070.5972 | 10 | -1.3 | 536.3052 | 2 | 53.69 | 5 | F5:18607 | 29102019\_RID\_1313\_NaNaPb\_F4.raw |  |  |  |  | 4.12E7 |  |  |  |  |  | 1 | 0 | 0 | 0 | 0 | 1 | 0 | 0 | 0 | 0 | 0 | 368 | 377 |  |
| R.HGETLRRTKEEINELNR.M | N | 57.51 | 2094.0930 | 17 | 0.0 | 524.5305 | 4 | 36.95 | 5 | F5:5998 | 29102019\_RID\_1313\_NaNaPb\_F4.raw |  |  |  |  | 7.51E6 |  |  |  |  |  | 1 | 0 | 0 | 0 | 0 | 1 | 0 | 0 | 0 | 0 | 0 | 311 | 327 |  |
| R.TKEEINELNRMIQRLTAEVENAK.C | Y | 56.28 | 2728.4177 | 23 | 0.7 | 546.6912 | 5 | 82.43 | 5 | F5:43110 | 29102019\_RID\_1313\_NaNaPb\_F4.raw |  |  |  |  | 1.23E6 |  |  |  |  |  | 1 | 0 | 0 | 0 | 0 | 1 | 0 | 0 | 0 | 0 | 0 | 318 | 340 |  |
| R.KSDLEANVEALIQEIDFLRR.L | N | 56.06 | 2358.2542 | 20 | 0.6 | 590.5712 | 4 | 92.42 | 5 | F5:51426 | 29102019\_RID\_1313\_NaNaPb\_F4.raw |  |  |  |  | 5.14E5 |  |  |  |  |  | 1 | 0 | 0 | 0 | 0 | 1 | 0 | 0 | 0 | 0 | 0 | 222 | 241 |  |
| K.KYEEEVSLRATAENEFVALKK.D | Y | 54.46 | 2453.2800 | 21 | -0.3 | 614.3271 | 4 | 58.18 | 5 | F5:22496 | 29102019\_RID\_1313\_NaNaPb\_F4.raw |  |  |  |  | 2.08E5 |  |  |  |  |  | 1 | 0 | 0 | 0 | 0 | 1 | 0 | 0 | 0 | 0 | 0 | 193 | 213 |  |
| K.LGLDIEIATYRR.L | N | 54.37 | 1418.7881 | 12 | 0.1 | 473.9367 | 3 | 65.02 | 5 | F5:28353 | 29102019\_RID\_1313\_NaNaPb\_F4.raw |  |  |  |  | 0 |  |  |  |  |  | 1 | 0 | 0 | 0 | 0 | 1 | 0 | 0 | 0 | 0 | 0 | 397 | 408 |  |
| R.FAAFIDK.V | N | 52.16 | 810.4276 | 7 | -0.3 | 406.2209 | 2 | 57.20 | 5 | F5:21640 | 29102019\_RID\_1313\_NaNaPb\_F4.raw |  |  |  |  | 7.64E5 |  |  |  |  |  | 1 | 0 | 0 | 0 | 0 | 1 | 0 | 0 | 0 | 0 | 0 | 117 | 123 |  |
| R.TKEEINELNRMIQR.L | N | 51.55 | 1772.9203 | 14 | 1.0 | 591.9813 | 3 | 51.34 | 5 | F5:16726 | 29102019\_RID\_1313\_NaNaPb\_F4.raw |  |  |  |  | 6.13E7 |  |  |  |  |  | 1 | 0 | 0 | 0 | 0 | 1 | 0 | 0 | 0 | 0 | 0 | 318 | 331 |  |
| R.AEAESWYR.S | N | 49.42 | 1010.4457 | 8 | 0.0 | 506.2302 | 2 | 50.22 | 5 | F5:15799 | 29102019\_RID\_1313\_NaNaPb\_F4.raw |  |  |  |  | 1.33E6 |  |  |  |  |  | 1 | 0 | 0 | 0 | 0 | 1 | 0 | 0 | 0 | 0 | 0 | 291 | 298 |  |
| total 28 peptides |
| --- |

#CONTAM#KRHB6\_HUMAN|

back to list

  

| Protein Coverage
| Supporting Peptides
|

Protein Coverage:

Supporting Peptides:

| Peptide | Uniq | -10lgP | Mass | Length | ppm | m/z | z | RT | Fraction | Scan | Source File | Area F1 | Area F10 | Area F2 | Area F3 | Area F4 | Area F5 | Area F6 | Area F7 | Area F8 | Area F9 | #Feature | #Feature F1 | #Feature F10 | #Feature F2 | #Feature F3 | #Feature F4 | #Feature F5 | #Feature F6 | #Feature F7 | #Feature F8 | #Feature F9 | Start | End | PTM |
| --- | --- | --- | --- | --- | --- | --- | --- | --- | --- | --- | --- | --- | --- | --- | --- | --- | --- | --- | --- | --- | --- | --- | --- | --- | --- | --- | --- | --- | --- | --- | --- | --- | --- | --- | --- |
| K.LEAAVAQSEQQGEAALSDAR.C | Y | 111.87 | 2042.9868 | 20 | 1.2 | 1022.5020 | 2 | 58.62 | 5 | F5:22876 | 29102019\_RID\_1313\_NaNaPb\_F4.raw |  |  |  |  | 1.83E6 |  |  |  |  |  | 1 | 0 | 0 | 0 | 0 | 1 | 0 | 0 | 0 | 0 | 0 | 346 | 365 |  |
| R.FAAFIDKVRFLEQQNKLLETK.L | N | 95.67 | 2537.4004 | 21 | -0.3 | 846.8072 | 3 | 69.08 | 5 | F5:31940 | 29102019\_RID\_1313\_NaNaPb\_F4.raw |  |  |  |  | 2.99E7 |  |  |  |  |  | 3 | 0 | 0 | 0 | 0 | 3 | 0 | 0 | 0 | 0 | 0 | 117 | 137 |  |
| K.SLNSRFAAFIDKVR.F | N | 84.83 | 1622.8892 | 14 | 0.9 | 541.9708 | 3 | 64.24 | 5 | F5:27586 | 29102019\_RID\_1313\_NaNaPb\_F4.raw |  |  |  |  | 8.58E6 |  |  |  |  |  | 2 | 0 | 0 | 0 | 0 | 2 | 0 | 0 | 0 | 0 | 0 | 112 | 125 |  |
| R.LASELNHVQEVLEGYKK.K | N | 77.99 | 1956.0316 | 17 | 0.5 | 490.0154 | 4 | 62.56 | 5 | F5:26250 | 29102019\_RID\_1313\_NaNaPb\_F4.raw |  |  |  |  | 0 |  |  |  |  |  | 1 | 0 | 0 | 0 | 0 | 1 | 0 | 0 | 0 | 0 | 0 | 176 | 192 |  |
| R.MIQRLTAEVENAK.C | Y | 76.29 | 1501.7922 | 13 | 0.2 | 501.6048 | 3 | 46.71 | 5 | F5:13137 | 29102019\_RID\_1313\_NaNaPb\_F4.raw |  |  |  |  | 2.02E7 |  |  |  |  |  | 2 | 0 | 0 | 0 | 0 | 2 | 0 | 0 | 0 | 0 | 0 | 328 | 340 |  |
| R.TKEEINELNR.M | N | 76.25 | 1244.6360 | 10 | -0.1 | 623.3252 | 2 | 36.79 | 5 | F5:5864 | 29102019\_RID\_1313\_NaNaPb\_F4.raw |  |  |  |  | 1.11E7 |  |  |  |  |  | 2 | 0 | 0 | 0 | 0 | 2 | 0 | 0 | 0 | 0 | 0 | 318 | 327 |  |
| R.FLEQQNKLLETKLQFYQNR.E | N | 73.95 | 2439.2910 | 19 | -0.5 | 610.8297 | 4 | 63.53 | 5 | F5:27032 | 29102019\_RID\_1313\_NaNaPb\_F4.raw |  |  |  |  | 6.87E6 |  |  |  |  |  | 1 | 0 | 0 | 0 | 0 | 1 | 0 | 0 | 0 | 0 | 0 | 126 | 144 |  |
| R.VSSVPSNSNVVVGTTNAC(+57.02)APSAR.V | Y | 70.21 | 2273.1069 | 23 | 0.2 | 1137.5609 | 2 | 48.03 | 5 | F5:14168 | 29102019\_RID\_1313\_NaNaPb\_F4.raw |  |  |  |  | 9.92E5 |  |  |  |  |  | 2 | 0 | 0 | 0 | 0 | 2 | 0 | 0 | 0 | 0 | 0 | 453 | 475 | Carbamidomethylation |
| R.FLEQQNKLLETK.L | N | 69.75 | 1489.8140 | 12 | 0.7 | 745.9148 | 2 | 49.91 | 5 | F5:15503 | 29102019\_RID\_1313\_NaNaPb\_F4.raw |  |  |  |  | 8.24E7 |  |  |  |  |  | 3 | 0 | 0 | 0 | 0 | 3 | 0 | 0 | 0 | 0 | 0 | 126 | 137 |  |
| R.ATAENEFVALKK.D | N | 69.36 | 1319.7085 | 12 | -0.7 | 440.9098 | 3 | 46.39 | 5 | F5:12964 | 29102019\_RID\_1313\_NaNaPb\_F4.raw |  |  |  |  | 1.27E6 |  |  |  |  |  | 2 | 0 | 0 | 0 | 0 | 2 | 0 | 0 | 0 | 0 | 0 | 202 | 213 |  |
| K.LLETKLQFYQNR.E | N | 68.97 | 1551.8409 | 12 | -0.1 | 518.2875 | 3 | 56.33 | 5 | F5:20948 | 29102019\_RID\_1313\_NaNaPb\_F4.raw |  |  |  |  | 1.03E6 |  |  |  |  |  | 1 | 0 | 0 | 0 | 0 | 1 | 0 | 0 | 0 | 0 | 0 | 133 | 144 |  |
| K.LGLDIEIATYR.R | N | 68.96 | 1262.6870 | 11 | -0.1 | 632.3507 | 2 | 74.93 | 5 | F5:36761 | 29102019\_RID\_1313\_NaNaPb\_F4.raw |  |  |  |  | 1.73E5 |  |  |  |  |  | 1 | 0 | 0 | 0 | 0 | 1 | 0 | 0 | 0 | 0 | 0 | 397 | 407 |  |
| R.FAAFIDKVRFLEQQNK.L | N | 68.77 | 1953.0471 | 16 | -0.2 | 489.2690 | 4 | 65.30 | 5 | F5:28595 | 29102019\_RID\_1313\_NaNaPb\_F4.raw |  |  |  |  | 1.83E7 |  |  |  |  |  | 3 | 0 | 0 | 0 | 0 | 3 | 0 | 0 | 0 | 0 | 0 | 117 | 132 |  |
| R.SRAEAESWYR.S | N | 68.47 | 1253.5789 | 10 | -0.3 | 418.8668 | 3 | 41.96 | 5 | F5:9725 | 29102019\_RID\_1313\_NaNaPb\_F4.raw |  |  |  |  | 6.42E6 |  |  |  |  |  | 2 | 0 | 0 | 0 | 0 | 2 | 0 | 0 | 0 | 0 | 0 | 289 | 298 |  |
| K.LAELEGALQKAK.Q | N | 65.48 | 1269.7292 | 12 | -0.6 | 424.2501 | 3 | 48.27 | 5 | F5:14319 | 29102019\_RID\_1313\_NaNaPb\_F4.raw |  |  |  |  | 6E6 |  |  |  |  |  | 2 | 0 | 0 | 0 | 0 | 2 | 0 | 0 | 0 | 0 | 0 | 368 | 379 |  |
| R.LYEEEIRVLQSHISDTSVVVK.L | Y | 64.64 | 2443.2959 | 21 | -0.3 | 815.4390 | 3 | 70.44 | 5 | F5:32953 | 29102019\_RID\_1313\_NaNaPb\_F4.raw |  |  |  |  | 4.9E6 |  |  |  |  |  | 2 | 0 | 0 | 0 | 0 | 2 | 0 | 0 | 0 | 0 | 0 | 242 | 262 |  |
| R.KSDLEANVEALIQEIDFLR.R | N | 63.57 | 2202.1531 | 19 | 1.1 | 735.0591 | 3 | 100.24 | 5 | F5:57358 | 29102019\_RID\_1313\_NaNaPb\_F4.raw |  |  |  |  | 1.05E5 |  |  |  |  |  | 2 | 0 | 0 | 0 | 0 | 2 | 0 | 0 | 0 | 0 | 0 | 222 | 240 |  |
| R.M(+15.99)IQRLTAEVENAK.C | Y | 61.94 | 1517.7871 | 13 | 0.2 | 506.9364 | 3 | 46.71 | 5 | F5:13172 | 29102019\_RID\_1313\_NaNaPb\_F4.raw |  |  |  |  | 1.89E6 |  |  |  |  |  | 2 | 0 | 0 | 0 | 0 | 2 | 0 | 0 | 0 | 0 | 0 | 328 | 340 | Oxidation (M) |
| R.FAAFIDKVR.F | N | 61.47 | 1065.5970 | 9 | 0.0 | 533.8058 | 2 | 53.38 | 5 | F5:18300 | 29102019\_RID\_1313\_NaNaPb\_F4.raw |  |  |  |  | 1.48E8 |  |  |  |  |  | 1 | 0 | 0 | 0 | 0 | 1 | 0 | 0 | 0 | 0 | 0 | 117 | 125 |  |
| K.LAELEGALQK.A | N | 60.04 | 1070.5972 | 10 | -1.3 | 536.3052 | 2 | 53.69 | 5 | F5:18607 | 29102019\_RID\_1313\_NaNaPb\_F4.raw |  |  |  |  | 4.12E7 |  |  |  |  |  | 1 | 0 | 0 | 0 | 0 | 1 | 0 | 0 | 0 | 0 | 0 | 368 | 377 |  |
| R.HGETLRRTKEEINELNR.M | N | 57.51 | 2094.0930 | 17 | 0.0 | 524.5305 | 4 | 36.95 | 5 | F5:5998 | 29102019\_RID\_1313\_NaNaPb\_F4.raw |  |  |  |  | 7.51E6 |  |  |  |  |  | 1 | 0 | 0 | 0 | 0 | 1 | 0 | 0 | 0 | 0 | 0 | 311 | 327 |  |
| R.TKEEINELNRMIQRLTAEVENAK.C | Y | 56.28 | 2728.4177 | 23 | 0.7 | 546.6912 | 5 | 82.43 | 5 | F5:43110 | 29102019\_RID\_1313\_NaNaPb\_F4.raw |  |  |  |  | 1.23E6 |  |  |  |  |  | 1 | 0 | 0 | 0 | 0 | 1 | 0 | 0 | 0 | 0 | 0 | 318 | 340 |  |
| R.KSDLEANVEALIQEIDFLRR.L | N | 56.06 | 2358.2542 | 20 | 0.6 | 590.5712 | 4 | 92.42 | 5 | F5:51426 | 29102019\_RID\_1313\_NaNaPb\_F4.raw |  |  |  |  | 5.14E5 |  |  |  |  |  | 1 | 0 | 0 | 0 | 0 | 1 | 0 | 0 | 0 | 0 | 0 | 222 | 241 |  |
| K.KYEEEVSLRATAENEFVALKK.D | Y | 54.46 | 2453.2800 | 21 | -0.3 | 614.3271 | 4 | 58.18 | 5 | F5:22496 | 29102019\_RID\_1313\_NaNaPb\_F4.raw |  |  |  |  | 2.08E5 |  |  |  |  |  | 1 | 0 | 0 | 0 | 0 | 1 | 0 | 0 | 0 | 0 | 0 | 193 | 213 |  |
| K.LGLDIEIATYRR.L | N | 54.37 | 1418.7881 | 12 | 0.1 | 473.9367 | 3 | 65.02 | 5 | F5:28353 | 29102019\_RID\_1313\_NaNaPb\_F4.raw |  |  |  |  | 0 |  |  |  |  |  | 1 | 0 | 0 | 0 | 0 | 1 | 0 | 0 | 0 | 0 | 0 | 397 | 408 |  |
| R.FAAFIDK.V | N | 52.16 | 810.4276 | 7 | -0.3 | 406.2209 | 2 | 57.20 | 5 | F5:21640 | 29102019\_RID\_1313\_NaNaPb\_F4.raw |  |  |  |  | 7.64E5 |  |  |  |  |  | 1 | 0 | 0 | 0 | 0 | 1 | 0 | 0 | 0 | 0 | 0 | 117 | 123 |  |
| R.TKEEINELNRMIQR.L | N | 51.55 | 1772.9203 | 14 | 1.0 | 591.9813 | 3 | 51.34 | 5 | F5:16726 | 29102019\_RID\_1313\_NaNaPb\_F4.raw |  |  |  |  | 6.13E7 |  |  |  |  |  | 1 | 0 | 0 | 0 | 0 | 1 | 0 | 0 | 0 | 0 | 0 | 318 | 331 |  |
| R.AEAESWYR.S | N | 49.42 | 1010.4457 | 8 | 0.0 | 506.2302 | 2 | 50.22 | 5 | F5:15799 | 29102019\_RID\_1313\_NaNaPb\_F4.raw |  |  |  |  | 1.33E6 |  |  |  |  |  | 1 | 0 | 0 | 0 | 0 | 1 | 0 | 0 | 0 | 0 | 0 | 291 | 298 |  |
| total 28 peptides |
| --- |

P25669|3L22\_NAJNA

back to list

  

| Protein Coverage
| Supporting Peptides
|

Protein Coverage:

Supporting Peptides:

| Peptide | Uniq | -10lgP | Mass | Length | ppm | m/z | z | RT | Fraction | Scan | Source File | Area F1 | Area F10 | Area F2 | Area F3 | Area F4 | Area F5 | Area F6 | Area F7 | Area F8 | Area F9 | #Feature | #Feature F1 | #Feature F10 | #Feature F2 | #Feature F3 | #Feature F4 | #Feature F5 | #Feature F6 | #Feature F7 | #Feature F8 | #Feature F9 | Start | End | PTM |
| --- | --- | --- | --- | --- | --- | --- | --- | --- | --- | --- | --- | --- | --- | --- | --- | --- | --- | --- | --- | --- | --- | --- | --- | --- | --- | --- | --- | --- | --- | --- | --- | --- | --- | --- | --- |
| R.C(+57.02)FITPDITSKDC(+57.02)PNGHVC(+57.02)YTK.T | N | 108.82 | 2512.1184 | 21 | -1.1 | 838.3792 | 3 | 11.64 | 7 | F7:2478 | 29102019\_RID\_1313\_NaNaPb\_F6.raw |  |  |  |  | 3.29E8 |  | 5.17E6 |  |  |  | 8 | 0 | 0 | 0 | 0 | 6 | 0 | 2 | 0 | 0 | 0 | 3 | 23 | Carbamidomethylation |
| R.VDLGC(+57.02)AATC(+57.02)PTVR.T | N | 108.71 | 1418.6646 | 13 | 1.0 | 710.3403 | 2 | 12.11 | 7 | F7:2909 | 29102019\_RID\_1313\_NaNaPb\_F6.raw |  | 1.21E6 |  |  | 2.34E8 | 1.19E7 | 4.59E8 | 3.09E4 | 1.2E6 | 1.6E6 | 42 | 0 | 1 | 0 | 0 | 3 | 2 | 33 | 1 | 1 | 1 | 37 | 49 | Carbamidomethylation |
| K.RVDLGC(+57.02)AATC(+57.02)PTVR.T | N | 102.24 | 1574.7657 | 14 | -0.6 | 788.3896 | 2 | 11.34 | 7 | F7:2170 | 29102019\_RID\_1313\_NaNaPb\_F6.raw |  |  |  |  | 1E9 |  | 2.54E7 |  |  |  | 11 | 0 | 0 | 0 | 0 | 8 | 0 | 3 | 0 | 0 | 0 | 36 | 49 | Carbamidomethylation |
| IRC(+57.02)FITPDITSKDC(+57.02)PNGHVC(+57.02)YTK.T | N | 99.90 | 2781.3037 | 23 | 1.5 | 696.3342 | 4 | 49.09 | 5 | F5:14964 | 29102019\_RID\_1313\_NaNaPb\_F4.raw |  |  |  |  | 1.1E9 |  |  |  |  |  | 8 | 0 | 0 | 0 | 0 | 8 | 0 | 0 | 0 | 0 | 0 | 1 | 23 | Carbamidomethylation |
| K.DC(+57.02)PNGHVC(+57.02)YTK.T | N | 84.60 | 1349.5493 | 11 | -0.6 | 675.7816 | 2 | 10.69 | 7 | F7:1588 | 29102019\_RID\_1313\_NaNaPb\_F6.raw |  |  |  |  |  |  | 0 |  |  |  | 2 | 0 | 0 | 0 | 0 | 0 | 0 | 2 | 0 | 0 | 0 | 13 | 23 | Carbamidomethylation |
| R.C(+57.02)FITPDITSK.D | N | 83.01 | 1180.5798 | 10 | 0.0 | 591.2972 | 2 | 13.12 | 7 | F7:3684 | 29102019\_RID\_1313\_NaNaPb\_F6.raw |  | 1.15E6 |  | 4.39E4 | 7.16E7 |  | 4.95E8 | 7.19E4 | 1.97E6 | 1.33E6 | 28 | 0 | 1 | 0 | 1 | 1 | 0 | 22 | 1 | 1 | 1 | 3 | 12 | Carbamidomethylation |
| R.GKRVDLGC(+57.02)AATC(+57.02)PTVR.T | N | 79.56 | 1759.8821 | 16 | 0.9 | 587.6352 | 3 | 37.79 | 5 | F5:6637 | 29102019\_RID\_1313\_NaNaPb\_F4.raw |  |  |  |  | 9.04E7 |  | 1.39E5 |  |  |  | 3 | 0 | 0 | 0 | 0 | 2 | 0 | 1 | 0 | 0 | 0 | 34 | 49 | Carbamidomethylation |
| C.PNGHVC(+57.02)YTK.T | N | 76.22 | 1074.4917 | 9 | -2.6 | 538.2517 | 2 | 10.69 | 7 | F7:1590 | 29102019\_RID\_1313\_NaNaPb\_F6.raw |  |  |  |  |  |  | 2.55E6 |  |  |  | 1 | 0 | 0 | 0 | 0 | 0 | 0 | 1 | 0 | 0 | 0 | 15 | 23 | Carbamidomethylation |
| IRC(+57.02)FITPDITSK.D | N | 72.29 | 1449.7650 | 12 | 0.4 | 725.8901 | 2 | 54.58 | 5 | F5:19303 | 29102019\_RID\_1313\_NaNaPb\_F4.raw |  |  |  |  | 2.93E8 |  | 3.64E5 |  |  |  | 6 | 0 | 0 | 0 | 0 | 5 | 0 | 1 | 0 | 0 | 0 | 1 | 12 | Carbamidomethylation |
| L.GC(+57.02)AATC(+57.02)PTVR.T | N | 62.53 | 1091.4852 | 10 | 0.0 | 546.7499 | 2 | 10.76 | 7 | F7:1638 | 29102019\_RID\_1313\_NaNaPb\_F6.raw |  |  |  |  |  |  | 4.59E5 |  |  |  | 1 | 0 | 0 | 0 | 0 | 0 | 0 | 1 | 0 | 0 | 0 | 40 | 49 | Carbamidomethylation |
| D.LGC(+57.02)AATC(+57.02)PTVR.T | N | 61.18 | 1204.5692 | 11 | 1.0 | 603.2925 | 2 | 11.12 | 7 | F7:2008 | 29102019\_RID\_1313\_NaNaPb\_F6.raw |  |  |  |  | 2.52E5 |  | 3.46E5 |  |  |  | 3 | 0 | 0 | 0 | 0 | 2 | 0 | 1 | 0 | 0 | 0 | 39 | 49 | Carbamidomethylation |
| K.RVDLGC(+57.02)AATC(+57.02)PT.V | N | 61.14 | 1319.5962 | 12 | -1.8 | 660.8042 | 2 | 11.42 | 7 | F7:2282 | 29102019\_RID\_1313\_NaNaPb\_F6.raw |  |  |  |  |  |  | 5.44E5 |  |  |  | 1 | 0 | 0 | 0 | 0 | 0 | 0 | 1 | 0 | 0 | 0 | 36 | 47 | Carbamidomethylation |
| R.VDLGC(+57.02)AATC(+57.02)PT.V | N | 59.60 | 1163.4951 | 11 | 0.0 | 582.7548 | 2 | 11.91 | 7 | F7:2756 | 29102019\_RID\_1313\_NaNaPb\_F6.raw |  |  |  |  |  |  | 8.06E5 |  |  |  | 1 | 0 | 0 | 0 | 0 | 0 | 0 | 1 | 0 | 0 | 0 | 37 | 47 | Carbamidomethylation |
| R.VDLGC(+57.02)AATC(+57.02)PTV.R | N | 57.22 | 1262.5635 | 12 | -0.8 | 632.2885 | 2 | 13.91 | 7 | F7:4209 | 29102019\_RID\_1313\_NaNaPb\_F6.raw |  |  |  |  |  |  | 0 |  |  |  | 1 | 0 | 0 | 0 | 0 | 0 | 0 | 1 | 0 | 0 | 0 | 37 | 48 | Carbamidomethylation |
| K.DC(+57.02)PNGHVC(+57.02)Y.T | N | 54.83 | 1120.4066 | 9 | 0.8 | 561.2111 | 2 | 11.17 | 7 | F7:2028 | 29102019\_RID\_1313\_NaNaPb\_F6.raw |  |  |  |  |  |  | 5.79E6 |  |  |  | 1 | 0 | 0 | 0 | 0 | 0 | 0 | 1 | 0 | 0 | 0 | 13 | 21 | Carbamidomethylation |
| K.TWC(+57.02)DGFC(+57.02)SSR.G | Y | 54.63 | 1274.4808 | 10 | 0.9 | 638.2483 | 2 | 11.78 | 7 | F7:2565 | 29102019\_RID\_1313\_NaNaPb\_F6.raw |  |  |  |  |  |  | 1.45E5 |  |  |  | 1 | 0 | 0 | 0 | 0 | 0 | 0 | 1 | 0 | 0 | 0 | 24 | 33 | Carbamidomethylation |
| R.VDLGC(+57.02)AATC(+57.02)P.T | N | 54.00 | 1062.4474 | 10 | 1.1 | 532.2316 | 2 | 12.13 | 7 | F7:2931 | 29102019\_RID\_1313\_NaNaPb\_F6.raw |  |  |  |  |  |  | 1.03E5 |  |  |  | 1 | 0 | 0 | 0 | 0 | 0 | 0 | 1 | 0 | 0 | 0 | 37 | 46 | Carbamidomethylation |
| R.TGVDIQC(+57.02)C(+57.02).S | N | 53.10 | 951.3790 | 8 | -0.3 | 476.6967 | 2 | 11.71 | 7 | F7:2547 | 29102019\_RID\_1313\_NaNaPb\_F6.raw |  |  |  |  |  |  | 1.32E6 |  |  |  | 1 | 0 | 0 | 0 | 0 | 0 | 0 | 1 | 0 | 0 | 0 | 50 | 57 | Carbamidomethylation |
| IRC(+57.02)FITPDITSKD.C | N | 46.76 | 1564.7919 | 13 | 0.1 | 783.4033 | 2 | 55.56 | 5 | F5:20289 | 29102019\_RID\_1313\_NaNaPb\_F4.raw |  |  |  |  | 7.27E6 |  |  |  |  |  | 2 | 0 | 0 | 0 | 0 | 2 | 0 | 0 | 0 | 0 | 0 | 1 | 13 | Carbamidomethylation |
| R.C(+57.02)FITPDITS.K | N | 46.63 | 1052.4849 | 9 | 0.1 | 527.2498 | 2 | 20.20 | 7 | F7:7324 | 29102019\_RID\_1313\_NaNaPb\_F6.raw |  |  |  |  |  |  | 2.43E4 |  |  |  | 1 | 0 | 0 | 0 | 0 | 0 | 0 | 1 | 0 | 0 | 0 | 3 | 11 | Carbamidomethylation |
| C.FITPDITSK.D | N | 42.13 | 1020.5491 | 9 | -2.4 | 511.2806 | 2 | 12.04 | 7 | F7:2836 | 29102019\_RID\_1313\_NaNaPb\_F6.raw |  |  |  |  |  |  | 2.76E5 |  |  |  | 1 | 0 | 0 | 0 | 0 | 0 | 0 | 1 | 0 | 0 | 0 | 4 | 12 |  |
| total 21 peptides |
| --- |

P84808|CRVP2\_NAJKA

back to list

  

| Protein Coverage
| Supporting Peptides
|

Protein Coverage:

Supporting Peptides:

| Peptide | Uniq | -10lgP | Mass | Length | ppm | m/z | z | RT | Fraction | Scan | Source File | Area F1 | Area F10 | Area F2 | Area F3 | Area F4 | Area F5 | Area F6 | Area F7 | Area F8 | Area F9 | #Feature | #Feature F1 | #Feature F10 | #Feature F2 | #Feature F3 | #Feature F4 | #Feature F5 | #Feature F6 | #Feature F7 | #Feature F8 | #Feature F9 | Start | End | PTM |
| --- | --- | --- | --- | --- | --- | --- | --- | --- | --- | --- | --- | --- | --- | --- | --- | --- | --- | --- | --- | --- | --- | --- | --- | --- | --- | --- | --- | --- | --- | --- | --- | --- | --- | --- | --- |
| K.SGPPC(+57.02)GDC(+57.02)PSAC(+57.02)VNGLC(+57.02)TNPC(+57.02)K.H | Y | 110.90 | 2406.9482 | 22 | -0.1 | 1204.4813 | 2 | 11.53 | 2 | F2:2466 | 29102019\_RID\_1313\_NaNaPb\_F10.raw |  | 9.7E6 |  |  |  |  |  |  |  | 2.27E5 | 3 | 0 | 2 | 0 | 0 | 0 | 0 | 0 | 0 | 0 | 1 | 180 | 201 | Carbamidomethylation |
| Q.YC(+57.02)PAGNIIGSIATPYK.S | Y | 92.58 | 1723.8604 | 16 | 2.6 | 862.9397 | 2 | 18.64 | 2 | F2:6605 | 29102019\_RID\_1313\_NaNaPb\_F10.raw |  | 3.03E5 |  |  |  |  |  |  |  |  | 1 | 0 | 1 | 0 | 0 | 0 | 0 | 0 | 0 | 0 | 0 | 164 | 179 | Carbamidomethylation |
| K.YLYVC(+57.02)QYC(+57.02)PAGNIIGSIATPYK.S | Y | 90.26 | 2550.2288 | 22 | -0.1 | 1276.1216 | 2 | 33.91 | 2 | F2:11538 | 29102019\_RID\_1313\_NaNaPb\_F10.raw |  | 5.69E6 |  |  |  |  |  |  |  |  | 2 | 0 | 2 | 0 | 0 | 0 | 0 | 0 | 0 | 0 | 0 | 158 | 179 | Carbamidomethylation |
| R.C(+57.02)SFAHSPPHLR.T | Y | 89.56 | 1307.6193 | 11 | 0.0 | 436.8804 | 3 | 10.87 | 2 | F2:1808 | 29102019\_RID\_1313\_NaNaPb\_F10.raw |  | 5.43E5 |  |  |  |  |  |  |  |  | 1 | 0 | 1 | 0 | 0 | 0 | 0 | 0 | 0 | 0 | 0 | 75 | 85 | Carbamidomethylation |
| R.NMLQM(+15.99)EWNSNAAQNAK.R | Y | 88.86 | 1864.8196 | 16 | 0.3 | 933.4174 | 2 | 11.77 | 2 | F2:2694 | 29102019\_RID\_1313\_NaNaPb\_F10.raw |  | 1.5E5 |  |  |  |  |  |  |  |  | 1 | 0 | 1 | 0 | 0 | 0 | 0 | 0 | 0 | 0 | 0 | 54 | 69 | Oxidation (M) |
| R.VIQSWYDENKK.F | Y | 87.72 | 1408.6986 | 11 | 1.0 | 705.3573 | 2 | 11.24 | 2 | F2:2174 | 29102019\_RID\_1313\_NaNaPb\_F10.raw |  | 3.36E6 |  |  |  |  |  |  |  |  | 2 | 0 | 2 | 0 | 0 | 0 | 0 | 0 | 0 | 0 | 0 | 108 | 118 |  |
| K.FVYGVGANPPGSVIGH.Y | Y | 77.96 | 1569.7939 | 16 | 0.8 | 785.9048 | 2 | 14.05 | 2 | F2:4464 | 29102019\_RID\_1313\_NaNaPb\_F10.raw |  | 5.79E6 |  |  |  |  |  |  |  |  | 3 | 0 | 3 | 0 | 0 | 0 | 0 | 0 | 0 | 0 | 0 | 119 | 134 |  |
| Y.VC(+57.02)QYC(+57.02)PAGNIIGSIATPYK.S | Y | 77.91 | 2111.0181 | 19 | 0.5 | 1056.5168 | 2 | 20.73 | 2 | F2:7381 | 29102019\_RID\_1313\_NaNaPb\_F10.raw |  | 7.7E5 |  |  |  |  |  |  |  |  | 1 | 0 | 1 | 0 | 0 | 0 | 0 | 0 | 0 | 0 | 0 | 161 | 179 | Carbamidomethylation |
| R.NMLQMEWNSNAAQNAK.R | Y | 77.54 | 1848.8247 | 16 | 0.8 | 925.4203 | 2 | 12.70 | 2 | F2:3497 | 29102019\_RID\_1313\_NaNaPb\_F10.raw |  | 4.77E5 |  |  |  |  |  |  |  |  | 1 | 0 | 1 | 0 | 0 | 0 | 0 | 0 | 0 | 0 | 0 | 54 | 69 |  |
| K.FVYGVGANPPGSVIGHYTQIVWYNSHLLGC(+57.02)GAAK.C | Y | 75.53 | 3631.8035 | 34 | 0.3 | 908.9584 | 4 | 33.51 | 2 | F2:11352 | 29102019\_RID\_1313\_NaNaPb\_F10.raw |  | 3.67E6 |  |  |  |  |  |  |  |  | 2 | 0 | 2 | 0 | 0 | 0 | 0 | 0 | 0 | 0 | 0 | 119 | 152 | Carbamidomethylation |
| R.VIQSWYDENK.K | Y | 75.20 | 1280.6036 | 10 | -0.2 | 641.3090 | 2 | 11.68 | 2 | F2:2595 | 29102019\_RID\_1313\_NaNaPb\_F10.raw |  | 7.37E5 |  |  |  |  |  |  |  |  | 1 | 0 | 1 | 0 | 0 | 0 | 0 | 0 | 0 | 0 | 0 | 108 | 117 |  |
| Y.C(+57.02)PAGNIIGSIATPYK.S | Y | 72.81 | 1560.7970 | 15 | 0.6 | 781.4062 | 2 | 15.02 | 2 | F2:4733 | 29102019\_RID\_1313\_NaNaPb\_F10.raw |  | 7.92E5 |  |  |  |  |  |  |  | 1.88E4 | 2 | 0 | 1 | 0 | 0 | 0 | 0 | 0 | 0 | 0 | 1 | 165 | 179 | Carbamidomethylation |
| K.FVYGVGANPPGSVIGHYTQIVWYNSH.L | Y | 69.12 | 2861.3926 | 26 | 0.9 | 954.8057 | 3 | 30.98 | 2 | F2:10660 | 29102019\_RID\_1313\_NaNaPb\_F10.raw |  | 8.66E5 |  |  |  |  |  |  |  |  | 1 | 0 | 1 | 0 | 0 | 0 | 0 | 0 | 0 | 0 | 0 | 119 | 144 |  |
| C.QYC(+57.02)PAGNIIGSIATPYK.S | Y | 67.60 | 1851.9188 | 17 | 1.0 | 926.9676 | 2 | 19.04 | 2 | F2:6745 | 29102019\_RID\_1313\_NaNaPb\_F10.raw |  | 6.87E5 |  |  |  |  |  |  |  |  | 1 | 0 | 1 | 0 | 0 | 0 | 0 | 0 | 0 | 0 | 0 | 163 | 179 | Carbamidomethylation |
| K.QNAC(+57.02)QTEWMK.S | Y | 66.70 | 1294.5435 | 10 | -0.1 | 648.2789 | 2 | 11.38 | 2 | F2:2301 | 29102019\_RID\_1313\_NaNaPb\_F10.raw |  | 2.75E5 |  |  |  |  |  |  |  |  | 1 | 0 | 1 | 0 | 0 | 0 | 0 | 0 | 0 | 0 | 0 | 215 | 224 | Carbamidomethylation |
| H.YTQIVWYNSHLLGC(+57.02)GAAK.C | Y | 65.47 | 2080.0200 | 18 | 0.8 | 694.3478 | 3 | 14.05 | 2 | F2:4216 | 29102019\_RID\_1313\_NaNaPb\_F10.raw |  | 6.71E5 |  |  |  |  |  |  |  |  | 1 | 0 | 1 | 0 | 0 | 0 | 0 | 0 | 0 | 0 | 0 | 135 | 152 | Carbamidomethylation |
| S.GTVDFASESSNK.R | Y | 61.92 | 1240.5571 | 12 | 0.7 | 621.2863 | 2 | 11.19 | 2 | F2:2132 | 29102019\_RID\_1313\_NaNaPb\_F10.raw |  | 1.4E5 |  |  |  |  |  |  |  |  | 1 | 0 | 1 | 0 | 0 | 0 | 0 | 0 | 0 | 0 | 0 | 19 | 30 |  |
| K.FVYGVGANPPGSVIGHYTQ.I | Y | 61.77 | 1961.9635 | 19 | 0.7 | 981.9897 | 2 | 16.11 | 2 | F2:5266 | 29102019\_RID\_1313\_NaNaPb\_F10.raw |  | 3.97E5 |  |  |  |  |  |  |  |  | 1 | 0 | 1 | 0 | 0 | 0 | 0 | 0 | 0 | 0 | 0 | 119 | 137 |  |
| G.TVDFASESSNK.R | Y | 56.24 | 1183.5356 | 11 | 0.3 | 592.7753 | 2 | 11.13 | 2 | F2:2059 | 29102019\_RID\_1313\_NaNaPb\_F10.raw |  | 4.42E5 |  |  |  |  |  |  |  |  | 1 | 0 | 1 | 0 | 0 | 0 | 0 | 0 | 0 | 0 | 0 | 20 | 30 |  |
| K.SGPPC(+57.02)GDC(+57.02)PSAC(+57.02)VN.G | Y | 54.33 | 1476.5432 | 14 | 0.6 | 739.2794 | 2 | 11.26 | 2 | F2:2182 | 29102019\_RID\_1313\_NaNaPb\_F10.raw |  | 2.81E5 |  |  |  |  |  |  |  |  | 1 | 0 | 1 | 0 | 0 | 0 | 0 | 0 | 0 | 0 | 0 | 180 | 193 | Carbamidomethylation |
| Y.TQIVWYNSHLLGC(+57.02)GAAK.C | Y | 44.23 | 1916.9567 | 17 | 0.5 | 639.9932 | 3 | 12.55 | 2 | F2:3416 | 29102019\_RID\_1313\_NaNaPb\_F10.raw |  | 5.5E4 |  |  |  |  |  |  |  |  | 1 | 0 | 1 | 0 | 0 | 0 | 0 | 0 | 0 | 0 | 0 | 136 | 152 | Carbamidomethylation |
| S.GTVDFASESSNKR.E | Y | 42.92 | 1396.6582 | 13 | 0.5 | 699.3367 | 2 | 10.87 | 2 | F2:1817 | 29102019\_RID\_1313\_NaNaPb\_F10.raw |  | 7.72E4 |  |  |  |  |  |  |  |  | 1 | 0 | 1 | 0 | 0 | 0 | 0 | 0 | 0 | 0 | 0 | 19 | 31 |  |
| total 22 peptides |
| --- |

P25672|3L24\_NAJNA

back to list

  

| Protein Coverage
| Supporting Peptides
|

Protein Coverage:

Supporting Peptides:

| Peptide | Uniq | -10lgP | Mass | Length | ppm | m/z | z | RT | Fraction | Scan | Source File | Area F1 | Area F10 | Area F2 | Area F3 | Area F4 | Area F5 | Area F6 | Area F7 | Area F8 | Area F9 | #Feature | #Feature F1 | #Feature F10 | #Feature F2 | #Feature F3 | #Feature F4 | #Feature F5 | #Feature F6 | #Feature F7 | #Feature F8 | #Feature F9 | Start | End | PTM |
| --- | --- | --- | --- | --- | --- | --- | --- | --- | --- | --- | --- | --- | --- | --- | --- | --- | --- | --- | --- | --- | --- | --- | --- | --- | --- | --- | --- | --- | --- | --- | --- | --- | --- | --- | --- |
| R.C(+57.02)FITPDITSKDC(+57.02)PNGHVC(+57.02)YTK.T | N | 108.82 | 2512.1184 | 21 | -1.1 | 838.3792 | 3 | 11.64 | 7 | F7:2478 | 29102019\_RID\_1313\_NaNaPb\_F6.raw |  |  |  |  | 3.29E8 |  | 5.17E6 |  |  |  | 8 | 0 | 0 | 0 | 0 | 6 | 0 | 2 | 0 | 0 | 0 | 3 | 23 | Carbamidomethylation |
| R.VDLGC(+57.02)AATC(+57.02)PTVK.T | N | 106.40 | 1390.6584 | 13 | -2.6 | 696.3347 | 2 | 11.56 | 7 | F7:2411 | 29102019\_RID\_1313\_NaNaPb\_F6.raw |  | 4.11E5 |  |  | 8.35E7 | 5.37E6 | 2.51E8 | 1.46E4 |  | 5.65E5 | 23 | 0 | 1 | 0 | 0 | 2 | 1 | 17 | 1 | 0 | 1 | 37 | 49 | Carbamidomethylation |
| IRC(+57.02)FITPDITSKDC(+57.02)PNGHVC(+57.02)YTK.T | N | 99.90 | 2781.3037 | 23 | 1.5 | 696.3342 | 4 | 49.09 | 5 | F5:14964 | 29102019\_RID\_1313\_NaNaPb\_F4.raw |  |  |  |  | 1.1E9 |  |  |  |  |  | 8 | 0 | 0 | 0 | 0 | 8 | 0 | 0 | 0 | 0 | 0 | 1 | 23 | Carbamidomethylation |
| E.RVDLGC(+57.02)AATC(+57.02)PTVK.T | N | 88.57 | 1546.7595 | 14 | -0.4 | 516.5936 | 3 | 11.29 | 7 | F7:2135 | 29102019\_RID\_1313\_NaNaPb\_F6.raw |  |  |  |  | 1.37E7 |  | 1.8E6 |  |  |  | 3 | 0 | 0 | 0 | 0 | 1 | 0 | 2 | 0 | 0 | 0 | 36 | 49 | Carbamidomethylation |
| K.DC(+57.02)PNGHVC(+57.02)YTK.T | N | 84.60 | 1349.5493 | 11 | -0.6 | 675.7816 | 2 | 10.69 | 7 | F7:1588 | 29102019\_RID\_1313\_NaNaPb\_F6.raw |  |  |  |  |  |  | 0 |  |  |  | 2 | 0 | 0 | 0 | 0 | 0 | 0 | 2 | 0 | 0 | 0 | 13 | 23 | Carbamidomethylation |
| R.C(+57.02)FITPDITSK.D | N | 83.01 | 1180.5798 | 10 | 0.0 | 591.2972 | 2 | 13.12 | 7 | F7:3684 | 29102019\_RID\_1313\_NaNaPb\_F6.raw |  | 1.15E6 |  | 4.39E4 | 7.16E7 |  | 4.95E8 | 7.19E4 | 1.97E6 | 1.33E6 | 28 | 0 | 1 | 0 | 1 | 1 | 0 | 22 | 1 | 1 | 1 | 3 | 12 | Carbamidomethylation |
| R.GERVDLGC(+57.02)AATC(+57.02)PTVK.T | N | 82.00 | 1732.8236 | 16 | -1.1 | 578.6145 | 3 | 11.34 | 7 | F7:2220 | 29102019\_RID\_1313\_NaNaPb\_F6.raw |  |  |  |  | 1.24E6 |  | 8.24E4 |  |  |  | 2 | 0 | 0 | 0 | 0 | 1 | 0 | 1 | 0 | 0 | 0 | 34 | 49 | Carbamidomethylation |
| C.PNGHVC(+57.02)YTK.T | N | 76.22 | 1074.4917 | 9 | -2.6 | 538.2517 | 2 | 10.69 | 7 | F7:1590 | 29102019\_RID\_1313\_NaNaPb\_F6.raw |  |  |  |  |  |  | 2.55E6 |  |  |  | 1 | 0 | 0 | 0 | 0 | 0 | 0 | 1 | 0 | 0 | 0 | 15 | 23 | Carbamidomethylation |
| IRC(+57.02)FITPDITSK.D | N | 72.29 | 1449.7650 | 12 | 0.4 | 725.8901 | 2 | 54.58 | 5 | F5:19303 | 29102019\_RID\_1313\_NaNaPb\_F4.raw |  |  |  |  | 2.93E8 |  | 3.64E5 |  |  |  | 6 | 0 | 0 | 0 | 0 | 5 | 0 | 1 | 0 | 0 | 0 | 1 | 12 | Carbamidomethylation |
| K.TWC(+57.02)DGFC(+57.02)R.I | Y | 63.65 | 1100.4167 | 8 | 0.1 | 551.2157 | 2 | 11.76 | 7 | F7:2551 | 29102019\_RID\_1313\_NaNaPb\_F6.raw |  |  |  |  |  |  | 7.02E7 |  |  |  | 3 | 0 | 0 | 0 | 0 | 0 | 0 | 3 | 0 | 0 | 0 | 24 | 31 | Carbamidomethylation |
| IRC(+57.02)FITPDITSKDC(+57.02)PNGHVC(+57.02)YTKTWC(+57.02)DGFC(+57.02)R.I | Y | 60.10 | 3863.7100 | 31 | 0.9 | 773.7500 | 5 | 58.26 | 5 | F5:22490 | 29102019\_RID\_1313\_NaNaPb\_F4.raw |  |  |  |  | 5.91E6 |  |  |  |  |  | 2 | 0 | 0 | 0 | 0 | 2 | 0 | 0 | 0 | 0 | 0 | 1 | 31 | Carbamidomethylation |
| R.VDLGC(+57.02)AATC(+57.02)PT.V | N | 59.60 | 1163.4951 | 11 | 0.0 | 582.7548 | 2 | 11.91 | 7 | F7:2756 | 29102019\_RID\_1313\_NaNaPb\_F6.raw |  |  |  |  |  |  | 8.06E5 |  |  |  | 1 | 0 | 0 | 0 | 0 | 0 | 0 | 1 | 0 | 0 | 0 | 37 | 47 | Carbamidomethylation |
| D.LGC(+57.02)AATC(+57.02)PTVK.T | N | 59.20 | 1176.5631 | 11 | -2.3 | 589.2875 | 2 | 11.07 | 7 | F7:1951 | 29102019\_RID\_1313\_NaNaPb\_F6.raw |  |  |  |  |  |  | 1.66E5 |  |  |  | 1 | 0 | 0 | 0 | 0 | 0 | 0 | 1 | 0 | 0 | 0 | 39 | 49 | Carbamidomethylation |
| R.VDLGC(+57.02)AATC(+57.02)PTV.K | N | 57.22 | 1262.5635 | 12 | -0.8 | 632.2885 | 2 | 13.91 | 7 | F7:4209 | 29102019\_RID\_1313\_NaNaPb\_F6.raw |  |  |  |  |  |  | 0 |  |  |  | 1 | 0 | 0 | 0 | 0 | 0 | 0 | 1 | 0 | 0 | 0 | 37 | 48 | Carbamidomethylation |
| R.IRGERVDLGC(+57.02)AATC(+57.02)PTVK.T | Y | 55.04 | 2002.0088 | 18 | 1.4 | 501.5102 | 4 | 41.61 | 5 | F5:9467 | 29102019\_RID\_1313\_NaNaPb\_F4.raw |  |  |  |  | 3.78E7 |  |  |  |  |  | 2 | 0 | 0 | 0 | 0 | 2 | 0 | 0 | 0 | 0 | 0 | 32 | 49 | Carbamidomethylation |
| K.DC(+57.02)PNGHVC(+57.02)Y.T | N | 54.83 | 1120.4066 | 9 | 0.8 | 561.2111 | 2 | 11.17 | 7 | F7:2028 | 29102019\_RID\_1313\_NaNaPb\_F6.raw |  |  |  |  |  |  | 5.79E6 |  |  |  | 1 | 0 | 0 | 0 | 0 | 0 | 0 | 1 | 0 | 0 | 0 | 13 | 21 | Carbamidomethylation |
| R.VDLGC(+57.02)AATC(+57.02)P.T | N | 54.00 | 1062.4474 | 10 | 1.1 | 532.2316 | 2 | 12.13 | 7 | F7:2931 | 29102019\_RID\_1313\_NaNaPb\_F6.raw |  |  |  |  |  |  | 1.03E5 |  |  |  | 1 | 0 | 0 | 0 | 0 | 0 | 0 | 1 | 0 | 0 | 0 | 37 | 46 | Carbamidomethylation |
| K.TGVDIQC(+57.02)C(+57.02).S | N | 53.10 | 951.3790 | 8 | -0.3 | 476.6967 | 2 | 11.71 | 7 | F7:2547 | 29102019\_RID\_1313\_NaNaPb\_F6.raw |  |  |  |  |  |  | 1.32E6 |  |  |  | 1 | 0 | 0 | 0 | 0 | 0 | 0 | 1 | 0 | 0 | 0 | 50 | 57 | Carbamidomethylation |
| IRC(+57.02)FITPDITSKD.C | N | 46.76 | 1564.7919 | 13 | 0.1 | 783.4033 | 2 | 55.56 | 5 | F5:20289 | 29102019\_RID\_1313\_NaNaPb\_F4.raw |  |  |  |  | 7.27E6 |  |  |  |  |  | 2 | 0 | 0 | 0 | 0 | 2 | 0 | 0 | 0 | 0 | 0 | 1 | 13 | Carbamidomethylation |
| R.C(+57.02)FITPDITS.K | N | 46.63 | 1052.4849 | 9 | 0.1 | 527.2498 | 2 | 20.20 | 7 | F7:7324 | 29102019\_RID\_1313\_NaNaPb\_F6.raw |  |  |  |  |  |  | 2.43E4 |  |  |  | 1 | 0 | 0 | 0 | 0 | 0 | 0 | 1 | 0 | 0 | 0 | 3 | 11 | Carbamidomethylation |
| C.FITPDITSK.D | N | 42.13 | 1020.5491 | 9 | -2.4 | 511.2806 | 2 | 12.04 | 7 | F7:2836 | 29102019\_RID\_1313\_NaNaPb\_F6.raw |  |  |  |  |  |  | 2.76E5 |  |  |  | 1 | 0 | 0 | 0 | 0 | 0 | 0 | 1 | 0 | 0 | 0 | 4 | 12 |  |
| total 21 peptides |
| --- |

P02533|K1C14\_HUMAN

back to list

  

| Protein Coverage
| Supporting Peptides
|

Protein Coverage:

Supporting Peptides:

| Peptide | Uniq | -10lgP | Mass | Length | ppm | m/z | z | RT | Fraction | Scan | Source File | Area F1 | Area F10 | Area F2 | Area F3 | Area F4 | Area F5 | Area F6 | Area F7 | Area F8 | Area F9 | #Feature | #Feature F1 | #Feature F10 | #Feature F2 | #Feature F3 | #Feature F4 | #Feature F5 | #Feature F6 | #Feature F7 | #Feature F8 | #Feature F9 | Start | End | PTM |
| --- | --- | --- | --- | --- | --- | --- | --- | --- | --- | --- | --- | --- | --- | --- | --- | --- | --- | --- | --- | --- | --- | --- | --- | --- | --- | --- | --- | --- | --- | --- | --- | --- | --- | --- | --- |
| R.APSTYGGGLSVSSSR.F | Y | 100.80 | 1424.6896 | 15 | -1.6 | 713.3510 | 2 | 11.42 | 7 | F7:2304 | 29102019\_RID\_1313\_NaNaPb\_F6.raw | 3.16E4 | 8.09E4 | 7.99E4 |  | 7.64E5 | 1.29E5 | 1.48E5 |  |  |  | 6 | 1 | 1 | 1 | 0 | 1 | 1 | 1 | 0 | 0 | 0 | 42 | 56 |  |
| R.ALEEANADLEVK.I | N | 92.34 | 1300.6510 | 12 | 0.2 | 651.3329 | 2 | 11.91 | 1 | F1:3410 | 29102019\_RID\_1313\_NaNaPb\_F1.raw | 9.19E4 | 1.48E5 | 1.95E5 |  |  | 3.99E5 | 0 |  |  | 1.22E5 | 6 | 1 | 1 | 1 | 0 | 0 | 1 | 1 | 0 | 0 | 1 | 135 | 146 |  |
| R.TKVMDVHDGKVVSTHEQVLR.T | N | 92.32 | 2277.1899 | 20 | 0.6 | 456.4456 | 5 | 42.40 | 5 | F5:10135 | 29102019\_RID\_1313\_NaNaPb\_F4.raw |  |  |  |  | 2.6E6 |  |  |  |  |  | 3 | 0 | 0 | 0 | 0 | 3 | 0 | 0 | 0 | 0 | 0 | 450 | 469 |  |
| R.EVATNSELVQSGK.S | N | 81.60 | 1360.6833 | 13 | -0.1 | 681.3489 | 2 | 11.36 | 3 | F3:2164 | 29102019\_RID\_1313\_NaNaPb\_F2.raw |  | 3.73E4 | 1.29E5 |  |  |  | 3.28E4 |  |  |  | 3 | 0 | 1 | 1 | 0 | 0 | 0 | 1 | 0 | 0 | 0 | 316 | 328 |  |
| R.RLLEGEDAHLSSSQFSSGSQSSRDVTSSSR.Q | Y | 79.05 | 3196.4980 | 30 | -0.4 | 800.1315 | 4 | 44.38 | 5 | F5:11730 | 29102019\_RID\_1313\_NaNaPb\_F4.raw |  |  |  |  | 1.12E6 |  |  |  |  |  | 2 | 0 | 0 | 0 | 0 | 2 | 0 | 0 | 0 | 0 | 0 | 417 | 446 |  |
| R.LLEGEDAHLSSSQFSSGSQSSR.D | Y | 78.85 | 2308.0566 | 22 | -0.5 | 770.3591 | 3 | 11.73 | 1 | F1:3249 | 29102019\_RID\_1313\_NaNaPb\_F1.raw | 2.13E4 | 4.96E4 | 6.93E4 |  |  | 6.09E4 | 0 |  |  |  | 5 | 1 | 1 | 1 | 0 | 0 | 1 | 1 | 0 | 0 | 0 | 418 | 439 |  |
| K.VMDVHDGKVVSTHEQVLR.T | N | 78.35 | 2048.0474 | 18 | -0.3 | 513.0190 | 4 | 44.94 | 5 | F5:12063 | 29102019\_RID\_1313\_NaNaPb\_F4.raw |  |  |  |  | 2.62E6 |  |  |  |  |  | 4 | 0 | 0 | 0 | 0 | 4 | 0 | 0 | 0 | 0 | 0 | 452 | 469 |  |
| K.ILTATVDNANVLLQIDNAR.L | Y | 75.12 | 2053.1167 | 19 | 1.1 | 685.3802 | 3 | 26.11 | 2 | F2:9026 | 29102019\_RID\_1313\_NaNaPb\_F10.raw |  | 1.18E5 |  |  |  | 1.13E5 | 5.14E4 |  |  |  | 3 | 0 | 1 | 0 | 0 | 0 | 1 | 1 | 0 | 0 | 0 | 176 | 194 |  |
| K.ASLENSLEETK.G | N | 73.23 | 1219.5931 | 11 | 0.7 | 610.8043 | 2 | 11.70 | 3 | F3:2516 | 29102019\_RID\_1313\_NaNaPb\_F2.raw | 1.86E4 |  | 1.34E5 |  |  |  |  |  |  |  | 2 | 1 | 0 | 1 | 0 | 0 | 0 | 0 | 0 | 0 | 0 | 353 | 363 |  |
| R.LLEGEDAHLSSSQFSSGSQSSRDVTSSSR.Q | Y | 68.04 | 3040.3970 | 29 | 0.4 | 761.1068 | 4 | 47.46 | 5 | F5:13936 | 29102019\_RID\_1313\_NaNaPb\_F4.raw |  |  |  |  | 7.58E6 |  |  |  |  |  | 2 | 0 | 0 | 0 | 0 | 2 | 0 | 0 | 0 | 0 | 0 | 418 | 446 |  |
| R.VLDELTLAR.A | N | 67.89 | 1028.5865 | 9 | 0.8 | 515.3010 | 2 | 62.60 | 5 | F5:26241 | 29102019\_RID\_1313\_NaNaPb\_F4.raw | 1.32E5 |  |  |  | 8.65E5 | 5.32E5 | 1.7E5 |  |  | 1.39E5 | 5 | 1 | 0 | 0 | 0 | 1 | 1 | 1 | 0 | 0 | 1 | 224 | 232 |  |
| R.LEQEIATYR.R | N | 65.29 | 1121.5717 | 9 | 0.7 | 561.7935 | 2 | 11.51 | 6 | F6:2300 | 29102019\_RID\_1313\_NaNaPb\_F5.raw |  |  | 1.24E5 |  |  | 1.68E5 |  |  |  |  | 2 | 0 | 0 | 1 | 0 | 0 | 1 | 0 | 0 | 0 | 0 | 408 | 416 |  |
| R.LAADDFRTKYETELNLR.M | Y | 64.49 | 2054.0432 | 17 | -0.3 | 514.5179 | 4 | 60.52 | 5 | F5:24436 | 29102019\_RID\_1313\_NaNaPb\_F4.raw |  |  |  |  | 1.47E6 |  |  |  |  |  | 1 | 0 | 0 | 0 | 0 | 1 | 0 | 0 | 0 | 0 | 0 | 195 | 211 |  |
| K.GSC(+57.02)GIGGGIGGGSSR.I | N | 62.48 | 1277.5782 | 15 | 0.1 | 639.7964 | 2 | 11.34 | 3 | F3:2162 | 29102019\_RID\_1313\_NaNaPb\_F2.raw |  |  | 2.84E4 |  |  | 7.21E4 |  |  |  |  | 2 | 0 | 0 | 1 | 0 | 0 | 1 | 0 | 0 | 0 | 0 | 16 | 30 | Carbamidomethylation |
| K.DAEEWFFTK.T | Y | 61.06 | 1171.5186 | 9 | 0.0 | 586.7665 | 2 | 23.26 | 7 | F7:8591 | 29102019\_RID\_1313\_NaNaPb\_F6.raw |  | 1.79E5 | 1.75E5 |  |  | 2.98E5 | 9.15E4 |  |  | 5.3E4 | 5 | 0 | 1 | 1 | 0 | 0 | 1 | 1 | 0 | 0 | 1 | 301 | 309 |  |
| K.VTMQNLNDRLASYLDKVR.A | N | 58.29 | 2135.1157 | 18 | 0.2 | 534.7863 | 4 | 70.04 | 5 | F5:32678 | 29102019\_RID\_1313\_NaNaPb\_F4.raw |  |  |  |  | 2.61E5 |  |  |  |  |  | 1 | 0 | 0 | 0 | 0 | 1 | 0 | 0 | 0 | 0 | 0 | 117 | 134 |  |
| R.ISSVLAGGSC(+57.02)R.A | N | 57.91 | 1105.5549 | 11 | 0.1 | 553.7848 | 2 | 11.58 | 3 | F3:2413 | 29102019\_RID\_1313\_NaNaPb\_F2.raw |  |  | 5.81E4 |  |  |  |  |  |  |  | 1 | 0 | 0 | 1 | 0 | 0 | 0 | 0 | 0 | 0 | 0 | 31 | 41 | Carbamidomethylation |
| R.LAADDFR.T | N | 56.98 | 806.3922 | 7 | 0.4 | 404.2036 | 2 | 11.73 | 1 | F1:3258 | 29102019\_RID\_1313\_NaNaPb\_F1.raw | 1E5 | 2.78E5 | 1.15E5 |  |  | 1.11E5 | 5.12E4 |  | 2.99E4 |  | 6 | 1 | 1 | 1 | 0 | 0 | 1 | 1 | 0 | 1 | 0 | 195 | 201 |  |
| K.DAEEWFFTKTEELNR.E | Y | 56.07 | 1913.8795 | 15 | 0.0 | 638.9671 | 3 | 20.58 | 7 | F7:7494 | 29102019\_RID\_1313\_NaNaPb\_F6.raw |  |  |  |  |  |  | 3.62E4 |  |  |  | 1 | 0 | 0 | 0 | 0 | 0 | 0 | 1 | 0 | 0 | 0 | 301 | 315 |  |
| R.LASYLDK.V | N | 55.97 | 808.4330 | 7 | -0.9 | 405.2234 | 2 | 11.66 | 1 | F1:3180 | 29102019\_RID\_1313\_NaNaPb\_F1.raw | 9.27E4 |  | 3.47E5 |  |  | 0 | 5.32E4 |  |  |  | 4 | 1 | 0 | 1 | 0 | 0 | 1 | 1 | 0 | 0 | 0 | 126 | 132 |  |
| R.GQVGGDVNVEM(+15.99)DAAPGVDLSR.I | N | 55.22 | 2100.9746 | 21 | 1.1 | 1051.4957 | 2 | 12.55 | 1 | F1:3945 | 29102019\_RID\_1313\_NaNaPb\_F1.raw | 1.01E4 |  |  |  |  |  |  |  |  |  | 1 | 1 | 0 | 0 | 0 | 0 | 0 | 0 | 0 | 0 | 0 | 262 | 282 | Oxidation (M) |
| K.VTMQNLNDR.L | N | 54.91 | 1089.5237 | 9 | 0.9 | 545.7696 | 2 | 11.21 | 7 | F7:2094 | 29102019\_RID\_1313\_NaNaPb\_F6.raw |  |  | 4.29E4 |  |  |  | 6.38E4 |  |  |  | 2 | 0 | 0 | 1 | 0 | 0 | 0 | 1 | 0 | 0 | 0 | 117 | 125 |  |
| R.MSVEADINGLRR.V | N | 51.59 | 1359.6929 | 12 | 0.2 | 454.2383 | 3 | 53.27 | 5 | F5:18362 | 29102019\_RID\_1313\_NaNaPb\_F4.raw |  |  |  |  | 1.69E6 |  |  |  |  |  | 1 | 0 | 0 | 0 | 0 | 1 | 0 | 0 | 0 | 0 | 0 | 212 | 223 |  |
| M.IGSVEEQLAQLR.C | N | 49.26 | 1341.7252 | 12 | 0.3 | 671.8701 | 2 | 16.80 | 1 | F1:5418 | 29102019\_RID\_1313\_NaNaPb\_F1.raw | 2.93E4 |  |  |  |  | 8.79E4 |  |  |  |  | 2 | 1 | 0 | 0 | 0 | 0 | 1 | 0 | 0 | 0 | 0 | 377 | 388 |  |
| R.LLEGEDAHLSSSQF.S | N | 47.37 | 1531.7155 | 14 | 1.1 | 766.8658 | 2 | 13.08 | 3 | F3:3508 | 29102019\_RID\_1313\_NaNaPb\_F2.raw |  |  | 3.73E4 |  |  |  |  |  |  |  | 1 | 0 | 0 | 1 | 0 | 0 | 0 | 0 | 0 | 0 | 0 | 418 | 431 |  |
| total 25 peptides |
| --- |

P25673|3L25\_NAJNA

back to list

  

| Protein Coverage
| Supporting Peptides
|

Protein Coverage:

Supporting Peptides:

| Peptide | Uniq | -10lgP | Mass | Length | ppm | m/z | z | RT | Fraction | Scan | Source File | Area F1 | Area F10 | Area F2 | Area F3 | Area F4 | Area F5 | Area F6 | Area F7 | Area F8 | Area F9 | #Feature | #Feature F1 | #Feature F10 | #Feature F2 | #Feature F3 | #Feature F4 | #Feature F5 | #Feature F6 | #Feature F7 | #Feature F8 | #Feature F9 | Start | End | PTM |
| --- | --- | --- | --- | --- | --- | --- | --- | --- | --- | --- | --- | --- | --- | --- | --- | --- | --- | --- | --- | --- | --- | --- | --- | --- | --- | --- | --- | --- | --- | --- | --- | --- | --- | --- | --- |
| R.C(+57.02)FITPDITSKDC(+57.02)PNGHVC(+57.02)YTK.T | N | 108.82 | 2512.1184 | 21 | -1.1 | 838.3792 | 3 | 11.64 | 7 | F7:2478 | 29102019\_RID\_1313\_NaNaPb\_F6.raw |  |  |  |  | 3.29E8 |  | 5.17E6 |  |  |  | 8 | 0 | 0 | 0 | 0 | 6 | 0 | 2 | 0 | 0 | 0 | 3 | 23 | Carbamidomethylation |
| R.VDLGC(+57.02)AATC(+57.02)PTVK.T | N | 106.40 | 1390.6584 | 13 | -2.6 | 696.3347 | 2 | 11.56 | 7 | F7:2411 | 29102019\_RID\_1313\_NaNaPb\_F6.raw |  | 4.11E5 |  |  | 8.35E7 | 5.37E6 | 2.51E8 | 1.46E4 |  | 5.65E5 | 23 | 0 | 1 | 0 | 0 | 2 | 1 | 17 | 1 | 0 | 1 | 37 | 49 | Carbamidomethylation |
| IRC(+57.02)FITPDITSKDC(+57.02)PNGHVC(+57.02)YTK.T | N | 99.90 | 2781.3037 | 23 | 1.5 | 696.3342 | 4 | 49.09 | 5 | F5:14964 | 29102019\_RID\_1313\_NaNaPb\_F4.raw |  |  |  |  | 1.1E9 |  |  |  |  |  | 8 | 0 | 0 | 0 | 0 | 8 | 0 | 0 | 0 | 0 | 0 | 1 | 23 | Carbamidomethylation |
| E.RVDLGC(+57.02)AATC(+57.02)PTVK.T | N | 88.57 | 1546.7595 | 14 | -0.4 | 516.5936 | 3 | 11.29 | 7 | F7:2135 | 29102019\_RID\_1313\_NaNaPb\_F6.raw |  |  |  |  | 1.37E7 |  | 1.8E6 |  |  |  | 3 | 0 | 0 | 0 | 0 | 1 | 0 | 2 | 0 | 0 | 0 | 36 | 49 | Carbamidomethylation |
| K.DC(+57.02)PNGHVC(+57.02)YTK.T | N | 84.60 | 1349.5493 | 11 | -0.6 | 675.7816 | 2 | 10.69 | 7 | F7:1588 | 29102019\_RID\_1313\_NaNaPb\_F6.raw |  |  |  |  |  |  | 0 |  |  |  | 2 | 0 | 0 | 0 | 0 | 0 | 0 | 2 | 0 | 0 | 0 | 13 | 23 | Carbamidomethylation |
| R.C(+57.02)FITPDITSK.D | N | 83.01 | 1180.5798 | 10 | 0.0 | 591.2972 | 2 | 13.12 | 7 | F7:3684 | 29102019\_RID\_1313\_NaNaPb\_F6.raw |  | 1.15E6 |  | 4.39E4 | 7.16E7 |  | 4.95E8 | 7.19E4 | 1.97E6 | 1.33E6 | 28 | 0 | 1 | 0 | 1 | 1 | 0 | 22 | 1 | 1 | 1 | 3 | 12 | Carbamidomethylation |
| R.GERVDLGC(+57.02)AATC(+57.02)PTVK.T | N | 82.00 | 1732.8236 | 16 | -1.1 | 578.6145 | 3 | 11.34 | 7 | F7:2220 | 29102019\_RID\_1313\_NaNaPb\_F6.raw |  |  |  |  | 1.24E6 |  | 8.24E4 |  |  |  | 2 | 0 | 0 | 0 | 0 | 1 | 0 | 1 | 0 | 0 | 0 | 34 | 49 | Carbamidomethylation |
| C.PNGHVC(+57.02)YTK.T | N | 76.22 | 1074.4917 | 9 | -2.6 | 538.2517 | 2 | 10.69 | 7 | F7:1590 | 29102019\_RID\_1313\_NaNaPb\_F6.raw |  |  |  |  |  |  | 2.55E6 |  |  |  | 1 | 0 | 0 | 0 | 0 | 0 | 0 | 1 | 0 | 0 | 0 | 15 | 23 | Carbamidomethylation |
| IRC(+57.02)FITPDITSK.D | N | 72.29 | 1449.7650 | 12 | 0.4 | 725.8901 | 2 | 54.58 | 5 | F5:19303 | 29102019\_RID\_1313\_NaNaPb\_F4.raw |  |  |  |  | 2.93E8 |  | 3.64E5 |  |  |  | 6 | 0 | 0 | 0 | 0 | 5 | 0 | 1 | 0 | 0 | 0 | 1 | 12 | Carbamidomethylation |
| S.RRGERVDLGC(+57.02)AATC(+57.02)PTVK.T | Y | 70.03 | 2045.0259 | 18 | -4.9 | 682.6792 | 3 | 52.00 | 5 | F5:17249 | 29102019\_RID\_1313\_NaNaPb\_F4.raw |  |  |  |  | 8.53E7 |  |  |  |  |  | 1 | 0 | 0 | 0 | 0 | 1 | 0 | 0 | 0 | 0 | 0 | 32 | 49 | Carbamidomethylation |
| R.VDLGC(+57.02)AATC(+57.02)PT.V | N | 59.60 | 1163.4951 | 11 | 0.0 | 582.7548 | 2 | 11.91 | 7 | F7:2756 | 29102019\_RID\_1313\_NaNaPb\_F6.raw |  |  |  |  |  |  | 8.06E5 |  |  |  | 1 | 0 | 0 | 0 | 0 | 0 | 0 | 1 | 0 | 0 | 0 | 37 | 47 | Carbamidomethylation |
| D.LGC(+57.02)AATC(+57.02)PTVK.T | N | 59.20 | 1176.5631 | 11 | -2.3 | 589.2875 | 2 | 11.07 | 7 | F7:1951 | 29102019\_RID\_1313\_NaNaPb\_F6.raw |  |  |  |  |  |  | 1.66E5 |  |  |  | 1 | 0 | 0 | 0 | 0 | 0 | 0 | 1 | 0 | 0 | 0 | 39 | 49 | Carbamidomethylation |
| R.VDLGC(+57.02)AATC(+57.02)PTV.K | N | 57.22 | 1262.5635 | 12 | -0.8 | 632.2885 | 2 | 13.91 | 7 | F7:4209 | 29102019\_RID\_1313\_NaNaPb\_F6.raw |  |  |  |  |  |  | 0 |  |  |  | 1 | 0 | 0 | 0 | 0 | 0 | 0 | 1 | 0 | 0 | 0 | 37 | 48 | Carbamidomethylation |
| K.DC(+57.02)PNGHVC(+57.02)Y.T | N | 54.83 | 1120.4066 | 9 | 0.8 | 561.2111 | 2 | 11.17 | 7 | F7:2028 | 29102019\_RID\_1313\_NaNaPb\_F6.raw |  |  |  |  |  |  | 5.79E6 |  |  |  | 1 | 0 | 0 | 0 | 0 | 0 | 0 | 1 | 0 | 0 | 0 | 13 | 21 | Carbamidomethylation |
| R.VDLGC(+57.02)AATC(+57.02)P.T | N | 54.00 | 1062.4474 | 10 | 1.1 | 532.2316 | 2 | 12.13 | 7 | F7:2931 | 29102019\_RID\_1313\_NaNaPb\_F6.raw |  |  |  |  |  |  | 1.03E5 |  |  |  | 1 | 0 | 0 | 0 | 0 | 0 | 0 | 1 | 0 | 0 | 0 | 37 | 46 | Carbamidomethylation |
| K.TGVDIQC(+57.02)C(+57.02).S | N | 53.10 | 951.3790 | 8 | -0.3 | 476.6967 | 2 | 11.71 | 7 | F7:2547 | 29102019\_RID\_1313\_NaNaPb\_F6.raw |  |  |  |  |  |  | 1.32E6 |  |  |  | 1 | 0 | 0 | 0 | 0 | 0 | 0 | 1 | 0 | 0 | 0 | 50 | 57 | Carbamidomethylation |
| IRC(+57.02)FITPDITSKD.C | N | 46.76 | 1564.7919 | 13 | 0.1 | 783.4033 | 2 | 55.56 | 5 | F5:20289 | 29102019\_RID\_1313\_NaNaPb\_F4.raw |  |  |  |  | 7.27E6 |  |  |  |  |  | 2 | 0 | 0 | 0 | 0 | 2 | 0 | 0 | 0 | 0 | 0 | 1 | 13 | Carbamidomethylation |
| R.C(+57.02)FITPDITS.K | N | 46.63 | 1052.4849 | 9 | 0.1 | 527.2498 | 2 | 20.20 | 7 | F7:7324 | 29102019\_RID\_1313\_NaNaPb\_F6.raw |  |  |  |  |  |  | 2.43E4 |  |  |  | 1 | 0 | 0 | 0 | 0 | 0 | 0 | 1 | 0 | 0 | 0 | 3 | 11 | Carbamidomethylation |
| C.FITPDITSK.D | N | 42.13 | 1020.5491 | 9 | -2.4 | 511.2806 | 2 | 12.04 | 7 | F7:2836 | 29102019\_RID\_1313\_NaNaPb\_F6.raw |  |  |  |  |  |  | 2.76E5 |  |  |  | 1 | 0 | 0 | 0 | 0 | 0 | 0 | 1 | 0 | 0 | 0 | 4 | 12 |  |
| total 19 peptides |
| --- |

P15924|DESP\_HUMAN

back to list

  

| Protein Coverage
| Supporting Peptides
|

Protein Coverage:

Supporting Peptides:

| Peptide | Uniq | -10lgP | Mass | Length | ppm | m/z | z | RT | Fraction | Scan | Source File | Area F1 | Area F10 | Area F2 | Area F3 | Area F4 | Area F5 | Area F6 | Area F7 | Area F8 | Area F9 | #Feature | #Feature F1 | #Feature F10 | #Feature F2 | #Feature F3 | #Feature F4 | #Feature F5 | #Feature F6 | #Feature F7 | #Feature F8 | #Feature F9 | Start | End | PTM |
| --- | --- | --- | --- | --- | --- | --- | --- | --- | --- | --- | --- | --- | --- | --- | --- | --- | --- | --- | --- | --- | --- | --- | --- | --- | --- | --- | --- | --- | --- | --- | --- | --- | --- | --- | --- |
| R.AVTGYNDPETGNIISLFQAMNKELIEKGHGIR.L | Y | 96.96 | 3514.7878 | 32 | 2.5 | 703.9666 | 5 | 83.83 | 5 | F5:44253 | 29102019\_RID\_1313\_NaNaPb\_F4.raw |  |  |  |  | 2.76E5 |  |  |  |  |  | 2 | 0 | 0 | 0 | 0 | 2 | 0 | 0 | 0 | 0 | 0 | 2335 | 2366 |  |
| R.TMIQSPSGVILQEAADVHAR.Y | Y | 96.76 | 2122.0840 | 20 | -1.0 | 708.3679 | 3 | 66.68 | 5 | F5:29757 | 29102019\_RID\_1313\_NaNaPb\_F4.raw |  |  |  |  | 4.51E6 |  |  |  |  |  | 1 | 0 | 0 | 0 | 0 | 1 | 0 | 0 | 0 | 0 | 0 | 983 | 1002 |  |
| R.LLEAQIASGGVVDPVNSVFLPKDVALAR.G | Y | 91.58 | 2877.5962 | 28 | 1.3 | 960.2073 | 3 | 78.98 | 5 | F5:40229 | 29102019\_RID\_1313\_NaNaPb\_F4.raw |  |  |  |  | 1.44E6 |  |  |  |  |  | 1 | 0 | 0 | 0 | 0 | 1 | 0 | 0 | 0 | 0 | 0 | 2124 | 2151 |  |
| R.MIRAESGPDLRYEVTSGGGGTSR.M | Y | 90.89 | 2395.1550 | 23 | 0.0 | 599.7960 | 4 | 47.16 | 5 | F5:13450 | 29102019\_RID\_1313\_NaNaPb\_F4.raw |  |  |  |  | 3.22E6 |  |  |  |  |  | 2 | 0 | 0 | 0 | 0 | 2 | 0 | 0 | 0 | 0 | 0 | 17 | 39 |  |
| R.LLEAQIATGGIIDPK.E | Y | 85.51 | 1537.8715 | 15 | -0.5 | 769.9426 | 2 | 17.26 | 6 | F6:6123 | 29102019\_RID\_1313\_NaNaPb\_F5.raw | 1.88E4 | 1.62E4 | 9.1E3 |  |  | 4.89E4 | 1.46E4 |  |  |  | 5 | 1 | 1 | 1 | 0 | 0 | 1 | 1 | 0 | 0 | 0 | 2367 | 2381 |  |
| K.LENINGVTDGYLNSLC(+57.02)TVR.A | Y | 73.24 | 2137.0474 | 19 | -0.9 | 1069.5300 | 2 | 22.20 | 2 | F2:7833 | 29102019\_RID\_1313\_NaNaPb\_F10.raw |  | 2.07E4 |  |  |  | 5.04E4 |  |  |  |  | 2 | 0 | 1 | 0 | 0 | 0 | 1 | 0 | 0 | 0 | 0 | 753 | 771 | Carbamidomethylation |
| R.SLNESKIEIER.L | Y | 66.47 | 1316.6936 | 11 | -0.7 | 439.9048 | 3 | 42.68 | 5 | F5:10430 | 29102019\_RID\_1313\_NaNaPb\_F4.raw |  |  |  |  | 2.95E5 |  |  |  |  |  | 1 | 0 | 0 | 0 | 0 | 1 | 0 | 0 | 0 | 0 | 0 | 1695 | 1705 |  |
| R.GIVDSITGQR.L | Y | 65.29 | 1044.5564 | 10 | -0.8 | 523.2850 | 2 | 56.81 | 5 | F5:21347 | 29102019\_RID\_1313\_NaNaPb\_F4.raw |  |  |  |  | 3.15E5 |  |  |  |  |  | 1 | 0 | 0 | 0 | 0 | 1 | 0 | 0 | 0 | 0 | 0 | 2640 | 2649 |  |
| R.ISTEEAIRKGFIDGR.A | Y | 60.96 | 1690.9001 | 15 | 0.6 | 423.7326 | 4 | 52.84 | 5 | F5:17946 | 29102019\_RID\_1313\_NaNaPb\_F4.raw |  |  |  |  | 4.93E5 |  |  |  |  |  | 1 | 0 | 0 | 0 | 0 | 1 | 0 | 0 | 0 | 0 | 0 | 2745 | 2759 |  |
| R.SMVEDITGLR.L | Y | 60.09 | 1119.5594 | 10 | 0.0 | 560.7870 | 2 | 66.60 | 5 | F5:29716 | 29102019\_RID\_1313\_NaNaPb\_F4.raw |  |  |  |  | 3.71E4 |  |  |  |  |  | 1 | 0 | 0 | 0 | 0 | 1 | 0 | 0 | 0 | 0 | 0 | 2792 | 2801 |  |
| K.SLLATMKTELQK.A | Y | 57.17 | 1361.7588 | 12 | -0.3 | 454.9267 | 3 | 60.26 | 5 | F5:24280 | 29102019\_RID\_1313\_NaNaPb\_F4.raw |  |  |  |  | 0 |  |  |  |  |  | 1 | 0 | 0 | 0 | 0 | 1 | 0 | 0 | 0 | 0 | 0 | 823 | 834 |  |
| R.TM(+15.99)IQSPSGVILQEAADVHAR.Y | Y | 56.54 | 2138.0789 | 20 | -0.4 | 713.7000 | 3 | 14.29 | 3 | F3:3973 | 29102019\_RID\_1313\_NaNaPb\_F2.raw |  |  | 5.48E3 |  |  |  |  |  |  |  | 1 | 0 | 0 | 1 | 0 | 0 | 0 | 0 | 0 | 0 | 0 | 983 | 1002 | Oxidation (M) |
| K.NATILELR.S | Y | 56.26 | 928.5342 | 8 | -0.2 | 465.2743 | 2 | 13.09 | 3 | F3:3534 | 29102019\_RID\_1313\_NaNaPb\_F2.raw |  |  | 1.59E4 |  |  |  |  |  |  |  | 1 | 0 | 0 | 1 | 0 | 0 | 0 | 0 | 0 | 0 | 0 | 1746 | 1753 |  |
| K.ITNLTQQLEQASIVK.K | Y | 55.58 | 1684.9359 | 15 | 0.8 | 843.4759 | 2 | 18.57 | 3 | F3:4942 | 29102019\_RID\_1313\_NaNaPb\_F2.raw |  |  | 1.75E4 |  |  |  |  |  |  |  | 1 | 0 | 0 | 1 | 0 | 0 | 0 | 0 | 0 | 0 | 0 | 1612 | 1626 |  |
| K.AQQIHSQTSQQYPLYDLDLGK.F | Y | 53.02 | 2432.1973 | 21 | 0.8 | 811.7404 | 3 | 13.95 | 3 | F3:3866 | 29102019\_RID\_1313\_NaNaPb\_F2.raw |  |  | 1.25E4 |  |  |  |  |  |  |  | 1 | 0 | 0 | 1 | 0 | 0 | 0 | 0 | 0 | 0 | 0 | 835 | 855 |  |
| R.AELIVQPELK.Y | Y | 51.27 | 1138.6597 | 10 | 0.5 | 570.3374 | 2 | 12.52 | 6 | F6:3212 | 29102019\_RID\_1313\_NaNaPb\_F5.raw | 1.52E4 |  | 4.03E4 |  |  | 2.63E4 |  |  |  |  | 3 | 1 | 0 | 1 | 0 | 0 | 1 | 0 | 0 | 0 | 0 | 85 | 94 |  |
| R.LQRLEDELNRAK.S | Y | 48.81 | 1483.8107 | 12 | 0.4 | 495.6110 | 3 | 42.34 | 5 | F5:10091 | 29102019\_RID\_1313\_NaNaPb\_F4.raw |  |  |  |  | 1.08E6 |  |  |  |  |  | 1 | 0 | 0 | 0 | 0 | 1 | 0 | 0 | 0 | 0 | 0 | 1829 | 1840 |  |
| R.FLNEQKNLHSEISGKR.D | Y | 45.98 | 1898.9962 | 16 | -0.5 | 475.7561 | 4 | 37.44 | 5 | F5:6387 | 29102019\_RID\_1313\_NaNaPb\_F4.raw |  |  |  |  | 7.08E5 |  |  |  |  |  | 1 | 0 | 0 | 0 | 0 | 1 | 0 | 0 | 0 | 0 | 0 | 926 | 941 |  |
| K.IEVLEEELR.L | Y | 44.34 | 1128.6027 | 9 | 0.4 | 565.3088 | 2 | 12.85 | 6 | F6:3487 | 29102019\_RID\_1313\_NaNaPb\_F5.raw |  |  |  |  |  | 4.83E4 |  |  |  |  | 1 | 0 | 0 | 0 | 0 | 0 | 1 | 0 | 0 | 0 | 0 | 1034 | 1042 |  |
| R.TLELQGLINDLQR.E | Y | 43.50 | 1511.8307 | 13 | 1.3 | 756.9236 | 2 | 33.54 | 3 | F3:7730 | 29102019\_RID\_1313\_NaNaPb\_F2.raw |  |  | 1.11E4 |  |  |  |  |  |  |  | 1 | 0 | 0 | 1 | 0 | 0 | 0 | 0 | 0 | 0 | 0 | 1763 | 1775 |  |
| R.YIELLTR.S | Y | 43.14 | 906.5175 | 7 | -0.4 | 454.2658 | 2 | 62.39 | 5 | F5:26060 | 29102019\_RID\_1313\_NaNaPb\_F4.raw |  |  |  |  | 8.67E5 |  |  |  |  |  | 1 | 0 | 0 | 0 | 0 | 1 | 0 | 0 | 0 | 0 | 0 | 1003 | 1009 |  |
| K.GFFDPNTEENLTYLQLK.E | Y | 42.88 | 2027.9840 | 17 | 1.2 | 1015.0005 | 2 | 33.19 | 6 | F6:14142 | 29102019\_RID\_1313\_NaNaPb\_F5.raw |  |  |  |  |  | 5.03E4 |  |  |  |  | 1 | 0 | 0 | 0 | 0 | 0 | 1 | 0 | 0 | 0 | 0 | 2414 | 2430 |  |
| K.NLIDRETGMRLLEAQIASGGVVDPVNSVFLPKDVALAR.G | Y | 42.74 | 4063.1887 | 38 | 1.0 | 1016.8055 | 4 | 83.00 | 5 | F5:43606 | 29102019\_RID\_1313\_NaNaPb\_F4.raw |  |  |  |  | 0 |  |  |  |  |  | 1 | 0 | 0 | 0 | 0 | 1 | 0 | 0 | 0 | 0 | 0 | 2114 | 2151 |  |
| R.QLQNIIQATSR.E | Y | 42.11 | 1270.6993 | 11 | 0.0 | 636.3569 | 2 | 12.25 | 3 | F3:3027 | 29102019\_RID\_1313\_NaNaPb\_F2.raw |  |  | 1.99E4 |  |  |  |  |  |  |  | 1 | 0 | 0 | 1 | 0 | 0 | 0 | 0 | 0 | 0 | 0 | 271 | 281 |  |
| total 24 peptides |
| --- |

#CONTAM#K1HB\_HUMAN|

back to list

  

| Protein Coverage
| Supporting Peptides
|

Protein Coverage:

Supporting Peptides:

| Peptide | Uniq | -10lgP | Mass | Length | ppm | m/z | z | RT | Fraction | Scan | Source File | Area F1 | Area F10 | Area F2 | Area F3 | Area F4 | Area F5 | Area F6 | Area F7 | Area F8 | Area F9 | #Feature | #Feature F1 | #Feature F10 | #Feature F2 | #Feature F3 | #Feature F4 | #Feature F5 | #Feature F6 | #Feature F7 | #Feature F8 | #Feature F9 | Start | End | PTM |
| --- | --- | --- | --- | --- | --- | --- | --- | --- | --- | --- | --- | --- | --- | --- | --- | --- | --- | --- | --- | --- | --- | --- | --- | --- | --- | --- | --- | --- | --- | --- | --- | --- | --- | --- | --- |
| R.TVNALEIELQAQHNLRYSLENTLTESEAR.Y | Y | 96.58 | 3341.6851 | 29 | 1.3 | 836.4296 | 4 | 78.42 | 5 | F5:39657 | 29102019\_RID\_1313\_NaNaPb\_F4.raw |  |  |  |  | 2.34E7 |  |  |  |  |  | 1 | 0 | 0 | 0 | 0 | 1 | 0 | 0 | 0 | 0 | 0 | 278 | 306 |  |
| R.SDLEAQMESLKEELLSLKQNHEQEVNTLR.C | Y | 94.69 | 3410.6987 | 29 | 0.9 | 853.6827 | 4 | 77.16 | 5 | F5:38600 | 29102019\_RID\_1313\_NaNaPb\_F4.raw |  |  |  |  | 1.02E7 |  |  |  |  |  | 2 | 0 | 0 | 0 | 0 | 2 | 0 | 0 | 0 | 0 | 0 | 174 | 202 |  |
| R.TVNALEIELQAQHNLR.Y | N | 85.94 | 1847.9854 | 16 | 1.5 | 617.0033 | 3 | 64.36 | 5 | F5:27680 | 29102019\_RID\_1313\_NaNaPb\_F4.raw |  |  |  |  | 7.74E7 |  |  |  |  |  | 2 | 0 | 0 | 0 | 0 | 2 | 0 | 0 | 0 | 0 | 0 | 278 | 293 |  |
| R.RTVNALEIELQAQHNLR.Y | N | 85.38 | 2004.0864 | 17 | 1.7 | 669.0372 | 3 | 56.81 | 5 | F5:21335 | 29102019\_RID\_1313\_NaNaPb\_F4.raw |  |  |  |  | 8.49E6 |  |  |  |  |  | 1 | 0 | 0 | 0 | 0 | 1 | 0 | 0 | 0 | 0 | 0 | 277 | 293 |  |
| R.LVVQIDNAKLAADDFRTKYQTEQSLR.Q | Y | 84.13 | 3021.5884 | 26 | 0.0 | 756.4044 | 4 | 61.36 | 5 | F5:25221 | 29102019\_RID\_1313\_NaNaPb\_F4.raw |  |  |  |  | 4.81E6 |  |  |  |  |  | 1 | 0 | 0 | 0 | 0 | 1 | 0 | 0 | 0 | 0 | 0 | 127 | 152 |  |
| R.LVVQIDNAKLAADDFR.T | N | 82.77 | 1786.9576 | 16 | 0.8 | 596.6603 | 3 | 63.77 | 5 | F5:27296 | 29102019\_RID\_1313\_NaNaPb\_F4.raw |  |  |  |  | 1.65E7 |  |  |  |  |  | 2 | 0 | 0 | 0 | 0 | 2 | 0 | 0 | 0 | 0 | 0 | 127 | 142 |  |
| R.LVVQIDNAKLAADDFRTK.Y | N | 81.18 | 2016.1003 | 18 | -1.9 | 1009.0555 | 2 | 57.93 | 5 | F5:22304 | 29102019\_RID\_1313\_NaNaPb\_F4.raw |  |  |  |  | 3.78E7 |  |  |  |  |  | 4 | 0 | 0 | 0 | 0 | 4 | 0 | 0 | 0 | 0 | 0 | 127 | 144 |  |
| R.SDLERQNQEYQVLLDVR.A | N | 79.55 | 2104.0549 | 17 | 1.2 | 702.3597 | 3 | 65.80 | 5 | F5:28997 | 29102019\_RID\_1313\_NaNaPb\_F4.raw |  |  |  |  | 2.33E6 |  |  |  |  |  | 1 | 0 | 0 | 0 | 0 | 1 | 0 | 0 | 0 | 0 | 0 | 330 | 346 |  |
| R.QNQEYQVLLDVR.A | N | 78.64 | 1503.7681 | 12 | 0.6 | 752.8917 | 2 | 68.55 | 5 | F5:31362 | 29102019\_RID\_1313\_NaNaPb\_F4.raw |  |  |  |  | 4.7E6 |  |  |  |  |  | 1 | 0 | 0 | 0 | 0 | 1 | 0 | 0 | 0 | 0 | 0 | 335 | 346 |  |
| R.YSLENTLTESEAR.Y | Y | 76.73 | 1511.7103 | 13 | 1.0 | 756.8632 | 2 | 60.53 | 5 | F5:24491 | 29102019\_RID\_1313\_NaNaPb\_F4.raw |  |  |  |  | 1.98E7 |  |  |  |  |  | 1 | 0 | 0 | 0 | 0 | 1 | 0 | 0 | 0 | 0 | 0 | 294 | 306 |  |
| R.EVEQWFATQTEELNKQVVSSSEQLQSYQAEIIELRR.T | N | 64.74 | 4295.1348 | 36 | 0.6 | 1074.7916 | 4 | 88.00 | 5 | F5:47718 | 29102019\_RID\_1313\_NaNaPb\_F4.raw |  |  |  |  | 7.49E5 |  |  |  |  |  | 1 | 0 | 0 | 0 | 0 | 1 | 0 | 0 | 0 | 0 | 0 | 242 | 277 |  |
| K.QVVSSSEQLQSYQAEIIELRR.T | N | 63.37 | 2462.2764 | 21 | 1.2 | 821.7671 | 3 | 67.06 | 5 | F5:30085 | 29102019\_RID\_1313\_NaNaPb\_F4.raw |  |  |  |  | 4.56E6 |  |  |  |  |  | 1 | 0 | 0 | 0 | 0 | 1 | 0 | 0 | 0 | 0 | 0 | 257 | 277 |  |
| K.LAADDFRTKYQTEQSLR.Q | Y | 62.80 | 2041.0228 | 17 | 1.0 | 681.3489 | 3 | 48.43 | 5 | F5:14452 | 29102019\_RID\_1313\_NaNaPb\_F4.raw |  |  |  |  | 6.82E5 |  |  |  |  |  | 1 | 0 | 0 | 0 | 0 | 1 | 0 | 0 | 0 | 0 | 0 | 136 | 152 |  |
| R.QLERDNAELENLIRER.S | N | 61.65 | 1997.0289 | 16 | 0.7 | 666.6841 | 3 | 57.99 | 5 | F5:22273 | 29102019\_RID\_1313\_NaNaPb\_F4.raw |  |  |  |  | 1.02E8 |  |  |  |  |  | 1 | 0 | 0 | 0 | 0 | 1 | 0 | 0 | 0 | 0 | 0 | 76 | 91 |  |
| R.QNQEYQVLLDVRAR.L | N | 58.17 | 1730.9064 | 14 | 0.9 | 577.9766 | 3 | 60.80 | 5 | F5:24777 | 29102019\_RID\_1313\_NaNaPb\_F4.raw |  |  |  |  | 1.2E6 |  |  |  |  |  | 1 | 0 | 0 | 0 | 0 | 1 | 0 | 0 | 0 | 0 | 0 | 335 | 348 |  |
| K.LAADDFR.T | N | 56.98 | 806.3922 | 7 | 0.4 | 404.2036 | 2 | 11.73 | 1 | F1:3258 | 29102019\_RID\_1313\_NaNaPb\_F1.raw | 1E5 | 2.78E5 | 1.15E5 |  |  | 1.11E5 | 5.12E4 |  | 2.99E4 |  | 6 | 1 | 1 | 1 | 0 | 0 | 1 | 1 | 0 | 1 | 0 | 136 | 142 |  |
| R.QLVESDINSLRR.I | Y | 56.06 | 1428.7684 | 12 | 0.3 | 477.2635 | 3 | 49.33 | 5 | F5:15092 | 29102019\_RID\_1313\_NaNaPb\_F4.raw |  |  |  |  | 2.07E7 |  |  |  |  |  | 2 | 0 | 0 | 0 | 0 | 2 | 0 | 0 | 0 | 0 | 0 | 153 | 164 |  |
| R.LVVQIDNAK.L | N | 52.63 | 998.5760 | 9 | -0.2 | 500.2952 | 2 | 48.54 | 5 | F5:14488 | 29102019\_RID\_1313\_NaNaPb\_F4.raw |  |  |  |  | 2.26E7 |  |  |  |  |  | 1 | 0 | 0 | 0 | 0 | 1 | 0 | 0 | 0 | 0 | 0 | 127 | 135 |  |
| R.ARLEC(+57.02)EINTYR.S | N | 49.72 | 1423.6877 | 11 | 0.3 | 475.5700 | 3 | 44.89 | 5 | F5:12054 | 29102019\_RID\_1313\_NaNaPb\_F4.raw |  |  |  |  | 7.57E5 |  |  |  |  |  | 1 | 0 | 0 | 0 | 0 | 1 | 0 | 0 | 0 | 0 | 0 | 347 | 357 | Carbamidomethylation |
| K.VRQLERDNAELENLIR.E | N | 48.97 | 1967.0548 | 16 | 0.3 | 492.7711 | 4 | 55.75 | 5 | F5:20433 | 29102019\_RID\_1313\_NaNaPb\_F4.raw |  |  |  |  | 3.44E6 |  |  |  |  |  | 1 | 0 | 0 | 0 | 0 | 1 | 0 | 0 | 0 | 0 | 0 | 74 | 89 |  |
| R.QLERDNAELENLIR.E | N | 48.55 | 1711.8853 | 14 | 0.2 | 571.6358 | 3 | 60.69 | 5 | F5:24620 | 29102019\_RID\_1313\_NaNaPb\_F4.raw |  |  |  |  | 9.29E6 |  |  |  |  |  | 2 | 0 | 0 | 0 | 0 | 2 | 0 | 0 | 0 | 0 | 0 | 76 | 89 |  |
| total 21 peptides |
| --- |

Q14525|KT33B\_HUMAN

back to list

  

| Protein Coverage
| Supporting Peptides
|

Protein Coverage:

Supporting Peptides:

| Peptide | Uniq | -10lgP | Mass | Length | ppm | m/z | z | RT | Fraction | Scan | Source File | Area F1 | Area F10 | Area F2 | Area F3 | Area F4 | Area F5 | Area F6 | Area F7 | Area F8 | Area F9 | #Feature | #Feature F1 | #Feature F10 | #Feature F2 | #Feature F3 | #Feature F4 | #Feature F5 | #Feature F6 | #Feature F7 | #Feature F8 | #Feature F9 | Start | End | PTM |
| --- | --- | --- | --- | --- | --- | --- | --- | --- | --- | --- | --- | --- | --- | --- | --- | --- | --- | --- | --- | --- | --- | --- | --- | --- | --- | --- | --- | --- | --- | --- | --- | --- | --- | --- | --- |
| R.TVNALEIELQAQHNLRYSLENTLTESEAR.Y | Y | 96.58 | 3341.6851 | 29 | 1.3 | 836.4296 | 4 | 78.42 | 5 | F5:39657 | 29102019\_RID\_1313\_NaNaPb\_F4.raw |  |  |  |  | 2.34E7 |  |  |  |  |  | 1 | 0 | 0 | 0 | 0 | 1 | 0 | 0 | 0 | 0 | 0 | 278 | 306 |  |
| R.SDLEAQMESLKEELLSLKQNHEQEVNTLR.C | Y | 94.69 | 3410.6987 | 29 | 0.9 | 853.6827 | 4 | 77.16 | 5 | F5:38600 | 29102019\_RID\_1313\_NaNaPb\_F4.raw |  |  |  |  | 1.02E7 |  |  |  |  |  | 2 | 0 | 0 | 0 | 0 | 2 | 0 | 0 | 0 | 0 | 0 | 174 | 202 |  |
| R.TVNALEIELQAQHNLR.Y | N | 85.94 | 1847.9854 | 16 | 1.5 | 617.0033 | 3 | 64.36 | 5 | F5:27680 | 29102019\_RID\_1313\_NaNaPb\_F4.raw |  |  |  |  | 7.74E7 |  |  |  |  |  | 2 | 0 | 0 | 0 | 0 | 2 | 0 | 0 | 0 | 0 | 0 | 278 | 293 |  |
| R.RTVNALEIELQAQHNLR.Y | N | 85.38 | 2004.0864 | 17 | 1.7 | 669.0372 | 3 | 56.81 | 5 | F5:21335 | 29102019\_RID\_1313\_NaNaPb\_F4.raw |  |  |  |  | 8.49E6 |  |  |  |  |  | 1 | 0 | 0 | 0 | 0 | 1 | 0 | 0 | 0 | 0 | 0 | 277 | 293 |  |
| R.LVVQIDNAKLAADDFRTKYQTEQSLR.Q | Y | 84.13 | 3021.5884 | 26 | 0.0 | 756.4044 | 4 | 61.36 | 5 | F5:25221 | 29102019\_RID\_1313\_NaNaPb\_F4.raw |  |  |  |  | 4.81E6 |  |  |  |  |  | 1 | 0 | 0 | 0 | 0 | 1 | 0 | 0 | 0 | 0 | 0 | 127 | 152 |  |
| R.LVVQIDNAKLAADDFR.T | N | 82.77 | 1786.9576 | 16 | 0.8 | 596.6603 | 3 | 63.77 | 5 | F5:27296 | 29102019\_RID\_1313\_NaNaPb\_F4.raw |  |  |  |  | 1.65E7 |  |  |  |  |  | 2 | 0 | 0 | 0 | 0 | 2 | 0 | 0 | 0 | 0 | 0 | 127 | 142 |  |
| R.LVVQIDNAKLAADDFRTK.Y | N | 81.18 | 2016.1003 | 18 | -1.9 | 1009.0555 | 2 | 57.93 | 5 | F5:22304 | 29102019\_RID\_1313\_NaNaPb\_F4.raw |  |  |  |  | 3.78E7 |  |  |  |  |  | 4 | 0 | 0 | 0 | 0 | 4 | 0 | 0 | 0 | 0 | 0 | 127 | 144 |  |
| R.SDLERQNQEYQVLLDVR.A | N | 79.55 | 2104.0549 | 17 | 1.2 | 702.3597 | 3 | 65.80 | 5 | F5:28997 | 29102019\_RID\_1313\_NaNaPb\_F4.raw |  |  |  |  | 2.33E6 |  |  |  |  |  | 1 | 0 | 0 | 0 | 0 | 1 | 0 | 0 | 0 | 0 | 0 | 330 | 346 |  |
| R.QNQEYQVLLDVR.A | N | 78.64 | 1503.7681 | 12 | 0.6 | 752.8917 | 2 | 68.55 | 5 | F5:31362 | 29102019\_RID\_1313\_NaNaPb\_F4.raw |  |  |  |  | 4.7E6 |  |  |  |  |  | 1 | 0 | 0 | 0 | 0 | 1 | 0 | 0 | 0 | 0 | 0 | 335 | 346 |  |
| R.YSLENTLTESEAR.Y | Y | 76.73 | 1511.7103 | 13 | 1.0 | 756.8632 | 2 | 60.53 | 5 | F5:24491 | 29102019\_RID\_1313\_NaNaPb\_F4.raw |  |  |  |  | 1.98E7 |  |  |  |  |  | 1 | 0 | 0 | 0 | 0 | 1 | 0 | 0 | 0 | 0 | 0 | 294 | 306 |  |
| R.EVEQWFATQTEELNKQVVSSSEQLQSYQAEIIELRR.T | N | 64.74 | 4295.1348 | 36 | 0.6 | 1074.7916 | 4 | 88.00 | 5 | F5:47718 | 29102019\_RID\_1313\_NaNaPb\_F4.raw |  |  |  |  | 7.49E5 |  |  |  |  |  | 1 | 0 | 0 | 0 | 0 | 1 | 0 | 0 | 0 | 0 | 0 | 242 | 277 |  |
| K.QVVSSSEQLQSYQAEIIELRR.T | N | 63.37 | 2462.2764 | 21 | 1.2 | 821.7671 | 3 | 67.06 | 5 | F5:30085 | 29102019\_RID\_1313\_NaNaPb\_F4.raw |  |  |  |  | 4.56E6 |  |  |  |  |  | 1 | 0 | 0 | 0 | 0 | 1 | 0 | 0 | 0 | 0 | 0 | 257 | 277 |  |
| K.LAADDFRTKYQTEQSLR.Q | Y | 62.80 | 2041.0228 | 17 | 1.0 | 681.3489 | 3 | 48.43 | 5 | F5:14452 | 29102019\_RID\_1313\_NaNaPb\_F4.raw |  |  |  |  | 6.82E5 |  |  |  |  |  | 1 | 0 | 0 | 0 | 0 | 1 | 0 | 0 | 0 | 0 | 0 | 136 | 152 |  |
| R.QLERDNAELENLIRER.S | N | 61.65 | 1997.0289 | 16 | 0.7 | 666.6841 | 3 | 57.99 | 5 | F5:22273 | 29102019\_RID\_1313\_NaNaPb\_F4.raw |  |  |  |  | 1.02E8 |  |  |  |  |  | 1 | 0 | 0 | 0 | 0 | 1 | 0 | 0 | 0 | 0 | 0 | 76 | 91 |  |
| R.QNQEYQVLLDVRAR.L | N | 58.17 | 1730.9064 | 14 | 0.9 | 577.9766 | 3 | 60.80 | 5 | F5:24777 | 29102019\_RID\_1313\_NaNaPb\_F4.raw |  |  |  |  | 1.2E6 |  |  |  |  |  | 1 | 0 | 0 | 0 | 0 | 1 | 0 | 0 | 0 | 0 | 0 | 335 | 348 |  |
| K.LAADDFR.T | N | 56.98 | 806.3922 | 7 | 0.4 | 404.2036 | 2 | 11.73 | 1 | F1:3258 | 29102019\_RID\_1313\_NaNaPb\_F1.raw | 1E5 | 2.78E5 | 1.15E5 |  |  | 1.11E5 | 5.12E4 |  | 2.99E4 |  | 6 | 1 | 1 | 1 | 0 | 0 | 1 | 1 | 0 | 1 | 0 | 136 | 142 |  |
| R.QLVESDINSLRR.I | Y | 56.06 | 1428.7684 | 12 | 0.3 | 477.2635 | 3 | 49.33 | 5 | F5:15092 | 29102019\_RID\_1313\_NaNaPb\_F4.raw |  |  |  |  | 2.07E7 |  |  |  |  |  | 2 | 0 | 0 | 0 | 0 | 2 | 0 | 0 | 0 | 0 | 0 | 153 | 164 |  |
| R.LVVQIDNAK.L | N | 52.63 | 998.5760 | 9 | -0.2 | 500.2952 | 2 | 48.54 | 5 | F5:14488 | 29102019\_RID\_1313\_NaNaPb\_F4.raw |  |  |  |  | 2.26E7 |  |  |  |  |  | 1 | 0 | 0 | 0 | 0 | 1 | 0 | 0 | 0 | 0 | 0 | 127 | 135 |  |
| R.ARLEC(+57.02)EINTYR.S | N | 49.72 | 1423.6877 | 11 | 0.3 | 475.5700 | 3 | 44.89 | 5 | F5:12054 | 29102019\_RID\_1313\_NaNaPb\_F4.raw |  |  |  |  | 7.57E5 |  |  |  |  |  | 1 | 0 | 0 | 0 | 0 | 1 | 0 | 0 | 0 | 0 | 0 | 347 | 357 | Carbamidomethylation |
| K.VRQLERDNAELENLIR.E | N | 48.97 | 1967.0548 | 16 | 0.3 | 492.7711 | 4 | 55.75 | 5 | F5:20433 | 29102019\_RID\_1313\_NaNaPb\_F4.raw |  |  |  |  | 3.44E6 |  |  |  |  |  | 1 | 0 | 0 | 0 | 0 | 1 | 0 | 0 | 0 | 0 | 0 | 74 | 89 |  |
| R.QLERDNAELENLIR.E | N | 48.55 | 1711.8853 | 14 | 0.2 | 571.6358 | 3 | 60.69 | 5 | F5:24620 | 29102019\_RID\_1313\_NaNaPb\_F4.raw |  |  |  |  | 9.29E6 |  |  |  |  |  | 2 | 0 | 0 | 0 | 0 | 2 | 0 | 0 | 0 | 0 | 0 | 76 | 89 |  |
| total 21 peptides |
| --- |

O76011|KRT34\_HUMAN

back to list

  

| Protein Coverage
| Supporting Peptides
|

Protein Coverage:

Supporting Peptides:

| Peptide | Uniq | -10lgP | Mass | Length | ppm | m/z | z | RT | Fraction | Scan | Source File | Area F1 | Area F10 | Area F2 | Area F3 | Area F4 | Area F5 | Area F6 | Area F7 | Area F8 | Area F9 | #Feature | #Feature F1 | #Feature F10 | #Feature F2 | #Feature F3 | #Feature F4 | #Feature F5 | #Feature F6 | #Feature F7 | #Feature F8 | #Feature F9 | Start | End | PTM |
| --- | --- | --- | --- | --- | --- | --- | --- | --- | --- | --- | --- | --- | --- | --- | --- | --- | --- | --- | --- | --- | --- | --- | --- | --- | --- | --- | --- | --- | --- | --- | --- | --- | --- | --- | --- |
| R.SQLGDRLNVEVDTAPTVDLNQVLNETR.S | Y | 101.69 | 2995.5210 | 27 | 1.0 | 999.5153 | 3 | 76.06 | 5 | F5:37727 | 29102019\_RID\_1313\_NaNaPb\_F4.raw |  |  |  |  | 7.63E6 |  |  |  |  |  | 1 | 0 | 0 | 0 | 0 | 1 | 0 | 0 | 0 | 0 | 0 | 245 | 271 |  |
| R.LVVNIDNAKLASDDFR.S | Y | 98.85 | 1788.9370 | 16 | 0.6 | 895.4763 | 2 | 63.07 | 5 | F5:26703 | 29102019\_RID\_1313\_NaNaPb\_F4.raw |  |  |  |  | 1.33E7 |  |  |  |  |  | 2 | 0 | 0 | 0 | 0 | 2 | 0 | 0 | 0 | 0 | 0 | 169 | 184 |  |
| R.LVVNIDNAKLASDDFRSK.Y | Y | 89.72 | 2004.0640 | 18 | 1.2 | 669.0294 | 3 | 56.65 | 5 | F5:21181 | 29102019\_RID\_1313\_NaNaPb\_F4.raw |  |  |  |  | 1.93E7 |  |  |  |  |  | 3 | 0 | 0 | 0 | 0 | 3 | 0 | 0 | 0 | 0 | 0 | 169 | 186 |  |
| R.TVNALEIELQAQHNLR.D | N | 85.94 | 1847.9854 | 16 | 1.5 | 617.0033 | 3 | 64.36 | 5 | F5:27680 | 29102019\_RID\_1313\_NaNaPb\_F4.raw |  |  |  |  | 7.74E7 |  |  |  |  |  | 2 | 0 | 0 | 0 | 0 | 2 | 0 | 0 | 0 | 0 | 0 | 320 | 335 |  |
| R.RTVNALEIELQAQHNLR.D | N | 85.38 | 2004.0864 | 17 | 1.7 | 669.0372 | 3 | 56.81 | 5 | F5:21335 | 29102019\_RID\_1313\_NaNaPb\_F4.raw |  |  |  |  | 8.49E6 |  |  |  |  |  | 1 | 0 | 0 | 0 | 0 | 1 | 0 | 0 | 0 | 0 | 0 | 319 | 335 |  |
| R.QNQEYQVLLDVR.A | N | 78.64 | 1503.7681 | 12 | 0.6 | 752.8917 | 2 | 68.55 | 5 | F5:31362 | 29102019\_RID\_1313\_NaNaPb\_F4.raw |  |  |  |  | 4.7E6 |  |  |  |  |  | 1 | 0 | 0 | 0 | 0 | 1 | 0 | 0 | 0 | 0 | 0 | 377 | 388 |  |
| R.LVVNIDNAKLASDDFRSKYQTEQSLR.L | Y | 76.91 | 3009.5520 | 26 | -4.0 | 753.3923 | 4 | 59.93 | 5 | F5:23945 | 29102019\_RID\_1313\_NaNaPb\_F4.raw |  |  |  |  | 1.21E8 |  |  |  |  |  | 7 | 0 | 0 | 0 | 0 | 7 | 0 | 0 | 0 | 0 | 0 | 169 | 194 |  |
| R.QLERDNAELEKLIQER.S | Y | 76.60 | 1983.0385 | 16 | 0.4 | 662.0204 | 3 | 58.62 | 5 | F5:22774 | 29102019\_RID\_1313\_NaNaPb\_F4.raw |  |  |  |  | 8.05E7 |  |  |  |  |  | 3 | 0 | 0 | 0 | 0 | 3 | 0 | 0 | 0 | 0 | 0 | 118 | 133 |  |
| R.LLVESDINSIRR.I | Y | 73.06 | 1413.7939 | 12 | -1.8 | 707.9030 | 2 | 53.61 | 5 | F5:18582 | 29102019\_RID\_1313\_NaNaPb\_F4.raw |  |  |  |  | 9.73E7 |  |  |  |  |  | 2 | 0 | 0 | 0 | 0 | 2 | 0 | 0 | 0 | 0 | 0 | 195 | 206 |  |
| K.LASDDFRSKYQTEQSLR.L | Y | 70.88 | 2043.0021 | 17 | 0.2 | 511.7579 | 4 | 45.36 | 5 | F5:12422 | 29102019\_RID\_1313\_NaNaPb\_F4.raw |  |  |  |  | 6.51E5 |  |  |  |  |  | 2 | 0 | 0 | 0 | 0 | 2 | 0 | 0 | 0 | 0 | 0 | 178 | 194 |  |
| R.SKYQTEQSLRLLVESDINSIRR.I | Y | 70.47 | 2634.4089 | 22 | -1.2 | 527.8884 | 5 | 67.49 | 5 | F5:30476 | 29102019\_RID\_1313\_NaNaPb\_F4.raw |  |  |  |  | 0 |  |  |  |  |  | 1 | 0 | 0 | 0 | 0 | 1 | 0 | 0 | 0 | 0 | 0 | 185 | 206 |  |
| K.AENARLVVNIDNAK.L | Y | 68.99 | 1525.8212 | 14 | 0.0 | 509.6143 | 3 | 46.93 | 5 | F5:13341 | 29102019\_RID\_1313\_NaNaPb\_F4.raw |  |  |  |  | 6.18E5 |  |  |  |  |  | 1 | 0 | 0 | 0 | 0 | 1 | 0 | 0 | 0 | 0 | 0 | 164 | 177 |  |
| R.DNAELEKLIQER.S | Y | 68.96 | 1456.7521 | 12 | -0.2 | 486.5912 | 3 | 64.66 | 5 | F5:27974 | 29102019\_RID\_1313\_NaNaPb\_F4.raw |  |  |  |  | 3.53E6 |  |  |  |  |  | 2 | 0 | 0 | 0 | 0 | 2 | 0 | 0 | 0 | 0 | 0 | 122 | 133 |  |
| K.VRQLERDNAELEKLIQER.S | Y | 64.08 | 2238.2080 | 18 | 0.5 | 448.6491 | 5 | 55.75 | 5 | F5:20380 | 29102019\_RID\_1313\_NaNaPb\_F4.raw |  |  |  |  | 5.51E7 |  |  |  |  |  | 2 | 0 | 0 | 0 | 0 | 2 | 0 | 0 | 0 | 0 | 0 | 116 | 133 |  |
| R.QNQEYQVLLDVRAR.L | N | 58.17 | 1730.9064 | 14 | 0.9 | 577.9766 | 3 | 60.80 | 5 | F5:24777 | 29102019\_RID\_1313\_NaNaPb\_F4.raw |  |  |  |  | 1.2E6 |  |  |  |  |  | 1 | 0 | 0 | 0 | 0 | 1 | 0 | 0 | 0 | 0 | 0 | 377 | 390 |  |
| R.LVVNIDNAK.L | Y | 55.17 | 984.5604 | 9 | 0.8 | 493.2878 | 2 | 50.08 | 5 | F5:15651 | 29102019\_RID\_1313\_NaNaPb\_F4.raw |  |  |  |  | 1.15E7 |  |  |  |  |  | 1 | 0 | 0 | 0 | 0 | 1 | 0 | 0 | 0 | 0 | 0 | 169 | 177 |  |
| R.ARLEC(+57.02)EINTYR.S | N | 49.72 | 1423.6877 | 11 | 0.3 | 475.5700 | 3 | 44.89 | 5 | F5:12054 | 29102019\_RID\_1313\_NaNaPb\_F4.raw |  |  |  |  | 7.57E5 |  |  |  |  |  | 1 | 0 | 0 | 0 | 0 | 1 | 0 | 0 | 0 | 0 | 0 | 389 | 399 | Carbamidomethylation |
| K.YQTEQSLRLLVESDINSIRR.I | Y | 49.53 | 2419.2820 | 20 | 0.9 | 807.4353 | 3 | 72.28 | 5 | F5:34604 | 29102019\_RID\_1313\_NaNaPb\_F4.raw |  |  |  |  | 5.15E6 |  |  |  |  |  | 1 | 0 | 0 | 0 | 0 | 1 | 0 | 0 | 0 | 0 | 0 | 187 | 206 |  |
| R.LLVESDINSIR.R | Y | 46.11 | 1257.6929 | 11 | 0.4 | 629.8539 | 2 | 62.05 | 5 | F5:25740 | 29102019\_RID\_1313\_NaNaPb\_F4.raw |  |  |  |  | 4.03E6 |  |  |  |  |  | 1 | 0 | 0 | 0 | 0 | 1 | 0 | 0 | 0 | 0 | 0 | 195 | 205 |  |
| total 19 peptides |
| --- |

P82942|VM3K\_NAJKA

back to list

  

| Protein Coverage
| Supporting Peptides
|

Protein Coverage:

Supporting Peptides:

| Peptide | Uniq | -10lgP | Mass | Length | ppm | m/z | z | RT | Fraction | Scan | Source File | Area F1 | Area F10 | Area F2 | Area F3 | Area F4 | Area F5 | Area F6 | Area F7 | Area F8 | Area F9 | #Feature | #Feature F1 | #Feature F10 | #Feature F2 | #Feature F3 | #Feature F4 | #Feature F5 | #Feature F6 | #Feature F7 | #Feature F8 | #Feature F9 | Start | End | PTM |
| --- | --- | --- | --- | --- | --- | --- | --- | --- | --- | --- | --- | --- | --- | --- | --- | --- | --- | --- | --- | --- | --- | --- | --- | --- | --- | --- | --- | --- | --- | --- | --- | --- | --- | --- | --- |
| K.C(+57.02)PTLTNQC(+57.02)IALLGPHFTVSPK.G | Y | 138.46 | 2353.1921 | 21 | 0.3 | 785.4049 | 3 | 18.42 | 7 | F7:6449 | 29102019\_RID\_1313\_NaNaPb\_F6.raw |  | 6.26E4 |  |  |  | 2.84E5 | 2.08E6 |  |  |  | 3 | 0 | 1 | 0 | 0 | 0 | 1 | 1 | 0 | 0 | 0 | 301 | 321 | Carbamidomethylation |
| K.HDC(+57.02)DLPELC(+57.02)TGQSAEC(+57.02)PTDSLQR.N | Y | 124.39 | 2688.1213 | 23 | 1.2 | 897.0488 | 3 | 11.83 | 2 | F2:2727 | 29102019\_RID\_1313\_NaNaPb\_F10.raw |  | 6.32E5 |  |  |  | 7.03E4 | 1.14E6 |  |  | 8.21E5 | 5 | 0 | 1 | 0 | 0 | 0 | 1 | 1 | 0 | 0 | 2 | 262 | 284 | Carbamidomethylation |
| L.IPPNPDGIMAEPGTK.C | N | 80.93 | 1535.7654 | 15 | 1.0 | 768.8907 | 2 | 11.87 | 7 | F7:2694 | 29102019\_RID\_1313\_NaNaPb\_F6.raw |  |  |  |  |  |  | 1.82E5 |  |  |  | 1 | 0 | 0 | 0 | 0 | 0 | 0 | 1 | 0 | 0 | 0 | 368 | 382 |  |
| Q.C(+57.02)IALLGPHFTVSPK.G | Y | 76.43 | 1538.8279 | 14 | 0.0 | 513.9499 | 3 | 13.45 | 7 | F7:3904 | 29102019\_RID\_1313\_NaNaPb\_F6.raw |  |  |  |  |  |  | 2.86E5 |  |  |  | 1 | 0 | 0 | 0 | 0 | 0 | 0 | 1 | 0 | 0 | 0 | 308 | 321 | Carbamidomethylation |
| K.GQC(+57.02)VDVQTAY | N | 68.80 | 1139.4917 | 10 | 0.9 | 570.7537 | 2 | 11.87 | 7 | F7:2699 | 29102019\_RID\_1313\_NaNaPb\_F6.raw |  |  |  |  |  |  | 1.18E5 |  |  |  | 1 | 0 | 0 | 0 | 0 | 0 | 0 | 1 | 0 | 0 | 0 | 392 | 401 | Carbamidomethylation |
| R.TAPAFQFSSC(+57.02)SIR.D | N | 57.10 | 1470.6925 | 13 | 0.3 | 736.3538 | 2 | 12.49 | 10 | F10:3268 | 29102019\_RID\_1313\_NaNaPb\_F9.raw |  | 8.57E3 |  |  |  |  |  |  |  | 3.87E4 | 2 | 0 | 1 | 0 | 0 | 0 | 0 | 0 | 0 | 0 | 1 | 178 | 190 | Carbamidomethylation |
| K.C(+57.02)PTLTNQC(+57.02)IALLGPH.F | Y | 57.01 | 1693.8280 | 15 | 0.9 | 847.9220 | 2 | 17.56 | 7 | F7:6043 | 29102019\_RID\_1313\_NaNaPb\_F6.raw |  |  |  |  |  |  | 1.81E5 |  |  |  | 1 | 0 | 0 | 0 | 0 | 0 | 0 | 1 | 0 | 0 | 0 | 301 | 315 | Carbamidomethylation |
| L.IPPNPDGIM(+15.99)AEPGTK.C | N | 55.45 | 1551.7603 | 15 | 0.5 | 776.8878 | 2 | 11.45 | 7 | F7:2313 | 29102019\_RID\_1313\_NaNaPb\_F6.raw |  |  |  |  |  |  | 9.78E4 |  |  |  | 1 | 0 | 0 | 0 | 0 | 0 | 0 | 1 | 0 | 0 | 0 | 368 | 382 | Oxidation (M) |
| C.IALLGPHFTVSPK.G | Y | 55.13 | 1378.7972 | 13 | 0.3 | 460.6065 | 3 | 12.64 | 7 | F7:3306 | 29102019\_RID\_1313\_NaNaPb\_F6.raw |  |  |  |  |  |  | 8.04E4 |  |  |  | 1 | 0 | 0 | 0 | 0 | 0 | 0 | 1 | 0 | 0 | 0 | 309 | 321 |  |
| K.C(+57.02)PTLTNQC(+57.02)IALLGPHF.T | Y | 52.79 | 1840.8964 | 16 | 1.4 | 921.4568 | 2 | 27.94 | 7 | F7:9878 | 29102019\_RID\_1313\_NaNaPb\_F6.raw |  |  |  |  |  |  | 3.07E4 |  |  |  | 1 | 0 | 0 | 0 | 0 | 0 | 0 | 1 | 0 | 0 | 0 | 301 | 316 | Carbamidomethylation |
| R.DYQEYLLR.D | Y | 49.49 | 1098.5345 | 8 | 0.8 | 550.2750 | 2 | 14.27 | 9 | F9:4402 | 29102019\_RID\_1313\_NaNaPb\_F8.raw |  | 1.55E5 |  |  |  |  |  |  | 3.42E4 |  | 2 | 0 | 1 | 0 | 0 | 0 | 0 | 0 | 0 | 1 | 0 | 191 | 198 |  |
| total 11 peptides |
| --- |

#CONTAM#KRHB4\_HUMAN|

back to list

  

| Protein Coverage
| Supporting Peptides
|

Protein Coverage:

Supporting Peptides:

| Peptide | Uniq | -10lgP | Mass | Length | ppm | m/z | z | RT | Fraction | Scan | Source File | Area F1 | Area F10 | Area F2 | Area F3 | Area F4 | Area F5 | Area F6 | Area F7 | Area F8 | Area F9 | #Feature | #Feature F1 | #Feature F10 | #Feature F2 | #Feature F3 | #Feature F4 | #Feature F5 | #Feature F6 | #Feature F7 | #Feature F8 | #Feature F9 | Start | End | PTM |
| --- | --- | --- | --- | --- | --- | --- | --- | --- | --- | --- | --- | --- | --- | --- | --- | --- | --- | --- | --- | --- | --- | --- | --- | --- | --- | --- | --- | --- | --- | --- | --- | --- | --- | --- | --- |
| R.AKLEAAVAEAEQQGEATLSDAK.C | Y | 89.70 | 2229.1123 | 22 | 0.6 | 1115.5641 | 2 | 67.70 | 5 | F5:30639 | 29102019\_RID\_1313\_NaNaPb\_F4.raw |  |  |  |  | 2.32E6 |  |  |  |  |  | 1 | 0 | 0 | 0 | 0 | 1 | 0 | 0 | 0 | 0 | 0 | 403 | 424 |  |
| K.SLLTPLNLEIDPNAQR.V | Y | 89.38 | 1792.9683 | 16 | 2.2 | 897.4934 | 2 | 79.92 | 5 | F5:41005 | 29102019\_RID\_1313\_NaNaPb\_F4.raw |  |  |  |  | 7.45E6 |  |  |  |  |  | 2 | 0 | 0 | 0 | 0 | 2 | 0 | 0 | 0 | 0 | 0 | 145 | 160 |  |
| K.MDNSRDLNLDGIIAEVK.A | Y | 88.06 | 1901.9517 | 17 | 1.5 | 634.9921 | 3 | 73.29 | 5 | F5:35354 | 29102019\_RID\_1313\_NaNaPb\_F4.raw |  |  |  |  | 1.29E6 |  |  |  |  |  | 1 | 0 | 0 | 0 | 0 | 1 | 0 | 0 | 0 | 0 | 0 | 322 | 338 |  |
| R.FLEQQNKLLETKWSFLQEQK.C | Y | 81.10 | 2536.3325 | 20 | -1.2 | 635.0897 | 4 | 68.42 | 5 | F5:31254 | 29102019\_RID\_1313\_NaNaPb\_F4.raw |  |  |  |  | 2.39E6 |  |  |  |  |  | 1 | 0 | 0 | 0 | 0 | 1 | 0 | 0 | 0 | 0 | 0 | 185 | 204 |  |
| R.VAPATGDLLSTGTR.S | Y | 80.88 | 1357.7201 | 14 | 0.0 | 679.8673 | 2 | 54.39 | 5 | F5:19217 | 29102019\_RID\_1313\_NaNaPb\_F4.raw |  |  |  |  | 1.94E7 |  |  |  |  |  | 1 | 0 | 0 | 0 | 0 | 1 | 0 | 0 | 0 | 0 | 0 | 539 | 552 |  |
| R.VGGVGVPAAPSITAVTVNK.S | Y | 80.38 | 1735.9832 | 19 | 2.3 | 869.0009 | 2 | 62.89 | 5 | F5:26451 | 29102019\_RID\_1313\_NaNaPb\_F4.raw |  |  |  |  | 7E6 |  |  |  |  |  | 1 | 0 | 0 | 0 | 0 | 1 | 0 | 0 | 0 | 0 | 0 | 126 | 144 |  |
| K.TLNNKFASFIDKVR.F | N | 77.96 | 1651.9045 | 14 | 0.2 | 413.9835 | 4 | 59.92 | 5 | F5:23986 | 29102019\_RID\_1313\_NaNaPb\_F4.raw |  |  |  |  | 1.32E6 |  |  |  |  |  | 2 | 0 | 0 | 0 | 0 | 2 | 0 | 0 | 0 | 0 | 0 | 171 | 184 |  |
| R.VGGVGVPAAPSITAVTVNKSLLTPLNLEIDPNAQR.V | Y | 74.57 | 3510.9409 | 35 | 0.6 | 878.7430 | 4 | 82.17 | 5 | F5:42840 | 29102019\_RID\_1313\_NaNaPb\_F4.raw |  |  |  |  | 6.9E6 |  |  |  |  |  | 2 | 0 | 0 | 0 | 0 | 2 | 0 | 0 | 0 | 0 | 0 | 126 | 160 |  |
| R.NIRNEINELTR.L | Y | 73.21 | 1370.7266 | 11 | 0.2 | 457.9162 | 3 | 54.65 | 5 | F5:19471 | 29102019\_RID\_1313\_NaNaPb\_F4.raw |  |  |  |  | 1.93E6 |  |  |  |  |  | 2 | 0 | 0 | 0 | 0 | 2 | 0 | 0 | 0 | 0 | 0 | 376 | 386 |  |
| R.QLREYQELMNAK.L | Y | 71.83 | 1521.7610 | 12 | 1.1 | 761.8886 | 2 | 51.81 | 5 | F5:17186 | 29102019\_RID\_1313\_NaNaPb\_F4.raw |  |  |  |  | 7.97E5 |  |  |  |  |  | 1 | 0 | 0 | 0 | 0 | 1 | 0 | 0 | 0 | 0 | 0 | 444 | 455 |  |
| R.SVITFGSYSPR.I | Y | 70.71 | 1212.6139 | 11 | -0.4 | 607.3140 | 2 | 62.57 | 5 | F5:26256 | 29102019\_RID\_1313\_NaNaPb\_F4.raw |  |  |  |  | 1.59E7 |  |  |  |  |  | 1 | 0 | 0 | 0 | 0 | 1 | 0 | 0 | 0 | 0 | 0 | 54 | 64 |  |
| R.FLEQQNKLLETK.W | N | 69.75 | 1489.8140 | 12 | 0.7 | 745.9148 | 2 | 49.91 | 5 | F5:15503 | 29102019\_RID\_1313\_NaNaPb\_F4.raw |  |  |  |  | 8.24E7 |  |  |  |  |  | 3 | 0 | 0 | 0 | 0 | 3 | 0 | 0 | 0 | 0 | 0 | 185 | 196 |  |
| K.LGLDIEIATYR.R | N | 68.96 | 1262.6870 | 11 | -0.1 | 632.3507 | 2 | 74.93 | 5 | F5:36761 | 29102019\_RID\_1313\_NaNaPb\_F4.raw |  |  |  |  | 1.73E5 |  |  |  |  |  | 1 | 0 | 0 | 0 | 0 | 1 | 0 | 0 | 0 | 0 | 0 | 456 | 466 |  |
| R.SNLEPLFESYITNLRR.Q | Y | 64.23 | 1951.0162 | 16 | 1.0 | 651.3467 | 3 | 79.92 | 5 | F5:40973 | 29102019\_RID\_1313\_NaNaPb\_F4.raw |  |  |  |  | 9.18E6 |  |  |  |  |  | 1 | 0 | 0 | 0 | 0 | 1 | 0 | 0 | 0 | 0 | 0 | 208 | 223 |  |
| R.DLNLDGIIAEVK.A | Y | 64.09 | 1298.7081 | 12 | 1.3 | 650.3622 | 2 | 85.32 | 5 | F5:45490 | 29102019\_RID\_1313\_NaNaPb\_F4.raw |  |  |  |  | 7.46E4 |  |  |  |  |  | 1 | 0 | 0 | 0 | 0 | 1 | 0 | 0 | 0 | 0 | 0 | 327 | 338 |  |
| R.QLEVLVSDQAR.L | Y | 57.45 | 1256.6725 | 11 | -1.7 | 629.3425 | 2 | 55.94 | 5 | F5:20589 | 29102019\_RID\_1313\_NaNaPb\_F4.raw |  |  |  |  | 6.39E6 |  |  |  |  |  | 1 | 0 | 0 | 0 | 0 | 1 | 0 | 0 | 0 | 0 | 0 | 224 | 234 |  |
| K.LGLDIEIATYRR.L | N | 54.37 | 1418.7881 | 12 | 0.1 | 473.9367 | 3 | 65.02 | 5 | F5:28353 | 29102019\_RID\_1313\_NaNaPb\_F4.raw |  |  |  |  | 0 |  |  |  |  |  | 1 | 0 | 0 | 0 | 0 | 1 | 0 | 0 | 0 | 0 | 0 | 456 | 467 |  |
| R.ANAENEFVALKK.D | Y | 46.74 | 1332.7037 | 12 | 1.6 | 667.3602 | 2 | 46.41 | 5 | F5:12995 | 29102019\_RID\_1313\_NaNaPb\_F4.raw |  |  |  |  | 1.32E5 |  |  |  |  |  | 1 | 0 | 0 | 0 | 0 | 1 | 0 | 0 | 0 | 0 | 0 | 261 | 272 |  |
| K.FASFIDK.V | N | 46.57 | 826.4225 | 7 | -0.3 | 414.2184 | 2 | 11.89 | 2 | F2:2810 | 29102019\_RID\_1313\_NaNaPb\_F10.raw |  | 1.96E5 |  |  |  | 3.22E5 |  |  | 0 |  | 3 | 0 | 1 | 0 | 0 | 0 | 1 | 0 | 0 | 1 | 0 | 176 | 182 |  |
| R.GLGSFGSR.S | Y | 46.43 | 779.3926 | 8 | -0.5 | 390.7034 | 2 | 46.56 | 5 | F5:13112 | 29102019\_RID\_1313\_NaNaPb\_F4.raw |  |  |  |  | 4.57E4 |  |  |  |  |  | 1 | 0 | 0 | 0 | 0 | 1 | 0 | 0 | 0 | 0 | 0 | 46 | 53 |  |
| R.NIRNEINELTRLIQR.L | Y | 43.65 | 1881.0544 | 15 | 0.4 | 471.2711 | 4 | 74.40 | 5 | F5:36339 | 29102019\_RID\_1313\_NaNaPb\_F4.raw |  |  |  |  | 0 |  |  |  |  |  | 1 | 0 | 0 | 0 | 0 | 1 | 0 | 0 | 0 | 0 | 0 | 376 | 390 |  |
| total 21 peptides |
| --- |

A5A6M5|K1H1\_PANTR

back to list

  

| Protein Coverage
| Supporting Peptides
|

Protein Coverage:

Supporting Peptides:

| Peptide | Uniq | -10lgP | Mass | Length | ppm | m/z | z | RT | Fraction | Scan | Source File | Area F1 | Area F10 | Area F2 | Area F3 | Area F4 | Area F5 | Area F6 | Area F7 | Area F8 | Area F9 | #Feature | #Feature F1 | #Feature F10 | #Feature F2 | #Feature F3 | #Feature F4 | #Feature F5 | #Feature F6 | #Feature F7 | #Feature F8 | #Feature F9 | Start | End | PTM |
| --- | --- | --- | --- | --- | --- | --- | --- | --- | --- | --- | --- | --- | --- | --- | --- | --- | --- | --- | --- | --- | --- | --- | --- | --- | --- | --- | --- | --- | --- | --- | --- | --- | --- | --- | --- |
| R.TVNALEIELQAQHNLRDSLENTLTESEAR.Y | N | 88.00 | 3293.6487 | 29 | 3.1 | 824.4220 | 4 | 79.92 | 5 | F5:40829 | 29102019\_RID\_1313\_NaNaPb\_F4.raw |  |  |  |  | 1.15E8 |  |  |  |  |  | 2 | 0 | 0 | 0 | 0 | 2 | 0 | 0 | 0 | 0 | 0 | 278 | 306 |  |
| R.TVNALEIELQAQHNLR.D | N | 85.94 | 1847.9854 | 16 | 1.5 | 617.0033 | 3 | 64.36 | 5 | F5:27680 | 29102019\_RID\_1313\_NaNaPb\_F4.raw |  |  |  |  | 7.74E7 |  |  |  |  |  | 2 | 0 | 0 | 0 | 0 | 2 | 0 | 0 | 0 | 0 | 0 | 278 | 293 |  |
| R.RTVNALEIELQAQHNLR.D | N | 85.38 | 2004.0864 | 17 | 1.7 | 669.0372 | 3 | 56.81 | 5 | F5:21335 | 29102019\_RID\_1313\_NaNaPb\_F4.raw |  |  |  |  | 8.49E6 |  |  |  |  |  | 1 | 0 | 0 | 0 | 0 | 1 | 0 | 0 | 0 | 0 | 0 | 277 | 293 |  |
| R.LVVQIDNAKLAADDFR.T | N | 82.77 | 1786.9576 | 16 | 0.8 | 596.6603 | 3 | 63.77 | 5 | F5:27296 | 29102019\_RID\_1313\_NaNaPb\_F4.raw |  |  |  |  | 1.65E7 |  |  |  |  |  | 2 | 0 | 0 | 0 | 0 | 2 | 0 | 0 | 0 | 0 | 0 | 127 | 142 |  |
| R.LVVQIDNAKLAADDFRTKYQTELSLR.Q | Y | 81.20 | 3006.6138 | 26 | 2.0 | 1003.2139 | 3 | 67.74 | 5 | F5:30696 | 29102019\_RID\_1313\_NaNaPb\_F4.raw |  |  |  |  | 2.43E6 |  |  |  |  |  | 2 | 0 | 0 | 0 | 0 | 2 | 0 | 0 | 0 | 0 | 0 | 127 | 152 |  |
| R.LVVQIDNAKLAADDFRTK.Y | N | 81.18 | 2016.1003 | 18 | -1.9 | 1009.0555 | 2 | 57.93 | 5 | F5:22304 | 29102019\_RID\_1313\_NaNaPb\_F4.raw |  |  |  |  | 3.78E7 |  |  |  |  |  | 4 | 0 | 0 | 0 | 0 | 4 | 0 | 0 | 0 | 0 | 0 | 127 | 144 |  |
| R.SDLERQNQEYQVLLDVR.A | N | 79.55 | 2104.0549 | 17 | 1.2 | 702.3597 | 3 | 65.80 | 5 | F5:28997 | 29102019\_RID\_1313\_NaNaPb\_F4.raw |  |  |  |  | 2.33E6 |  |  |  |  |  | 1 | 0 | 0 | 0 | 0 | 1 | 0 | 0 | 0 | 0 | 0 | 330 | 346 |  |
| R.QNQEYQVLLDVR.A | N | 78.64 | 1503.7681 | 12 | 0.6 | 752.8917 | 2 | 68.55 | 5 | F5:31362 | 29102019\_RID\_1313\_NaNaPb\_F4.raw |  |  |  |  | 4.7E6 |  |  |  |  |  | 1 | 0 | 0 | 0 | 0 | 1 | 0 | 0 | 0 | 0 | 0 | 335 | 346 |  |
| K.LAADDFRTKYQTELSLR.Q | Y | 75.07 | 2026.0483 | 17 | -0.3 | 507.5192 | 4 | 58.60 | 5 | F5:22799 | 29102019\_RID\_1313\_NaNaPb\_F4.raw |  |  |  |  | 2.43E6 |  |  |  |  |  | 1 | 0 | 0 | 0 | 0 | 1 | 0 | 0 | 0 | 0 | 0 | 136 | 152 |  |
| K.QVVSSSEQLQSYQAEIIELRR.T | N | 63.37 | 2462.2764 | 21 | 1.2 | 821.7671 | 3 | 67.06 | 5 | F5:30085 | 29102019\_RID\_1313\_NaNaPb\_F4.raw |  |  |  |  | 4.56E6 |  |  |  |  |  | 1 | 0 | 0 | 0 | 0 | 1 | 0 | 0 | 0 | 0 | 0 | 257 | 277 |  |
| R.TKYQTELSLR.Q | Y | 63.27 | 1237.6666 | 10 | 0.0 | 413.5628 | 3 | 44.13 | 5 | F5:11527 | 29102019\_RID\_1313\_NaNaPb\_F4.raw |  |  |  |  | 8.71E5 |  |  |  |  |  | 2 | 0 | 0 | 0 | 0 | 2 | 0 | 0 | 0 | 0 | 0 | 143 | 152 |  |
| K.YQTELSLRQLVESDINGLRR.I | Y | 61.98 | 2389.2712 | 20 | 0.3 | 797.4313 | 3 | 73.32 | 5 | F5:35347 | 29102019\_RID\_1313\_NaNaPb\_F4.raw |  |  |  |  | 1.05E7 |  |  |  |  |  | 1 | 0 | 0 | 0 | 0 | 1 | 0 | 0 | 0 | 0 | 0 | 145 | 164 |  |
| R.QLERDNAELENLIRER.S | N | 61.65 | 1997.0289 | 16 | 0.7 | 666.6841 | 3 | 57.99 | 5 | F5:22273 | 29102019\_RID\_1313\_NaNaPb\_F4.raw |  |  |  |  | 1.02E8 |  |  |  |  |  | 1 | 0 | 0 | 0 | 0 | 1 | 0 | 0 | 0 | 0 | 0 | 76 | 91 |  |
| R.EVEQWFTTQTEELNKQVVSSSEQLQSYQAEIIELRR.T | Y | 61.02 | 4325.1450 | 36 | 0.8 | 1082.2944 | 4 | 86.94 | 5 | F5:46766 | 29102019\_RID\_1313\_NaNaPb\_F4.raw |  |  |  |  | 1.82E6 |  |  |  |  |  | 2 | 0 | 0 | 0 | 0 | 2 | 0 | 0 | 0 | 0 | 0 | 242 | 277 |  |
| R.QLVESDINGLRR.I | N | 59.73 | 1398.7579 | 12 | 0.2 | 700.3864 | 2 | 48.02 | 5 | F5:14116 | 29102019\_RID\_1313\_NaNaPb\_F4.raw |  |  |  |  | 3.26E7 |  |  |  |  |  | 2 | 0 | 0 | 0 | 0 | 2 | 0 | 0 | 0 | 0 | 0 | 153 | 164 |  |
| K.YQTELSLR.Q | Y | 59.62 | 1008.5240 | 8 | 0.3 | 505.2694 | 2 | 52.23 | 5 | F5:17464 | 29102019\_RID\_1313\_NaNaPb\_F4.raw |  |  |  |  | 1.58E6 |  |  |  |  |  | 1 | 0 | 0 | 0 | 0 | 1 | 0 | 0 | 0 | 0 | 0 | 145 | 152 |  |
| R.QNQEYQVLLDVRAR.L | N | 58.17 | 1730.9064 | 14 | 0.9 | 577.9766 | 3 | 60.80 | 5 | F5:24777 | 29102019\_RID\_1313\_NaNaPb\_F4.raw |  |  |  |  | 1.2E6 |  |  |  |  |  | 1 | 0 | 0 | 0 | 0 | 1 | 0 | 0 | 0 | 0 | 0 | 335 | 348 |  |
| K.LAADDFR.T | N | 56.98 | 806.3922 | 7 | 0.4 | 404.2036 | 2 | 11.73 | 1 | F1:3258 | 29102019\_RID\_1313\_NaNaPb\_F1.raw | 1E5 | 2.78E5 | 1.15E5 |  |  | 1.11E5 | 5.12E4 |  | 2.99E4 |  | 6 | 1 | 1 | 1 | 0 | 0 | 1 | 1 | 0 | 1 | 0 | 136 | 142 |  |
| R.LVVQIDNAK.L | N | 52.63 | 998.5760 | 9 | -0.2 | 500.2952 | 2 | 48.54 | 5 | F5:14488 | 29102019\_RID\_1313\_NaNaPb\_F4.raw |  |  |  |  | 2.26E7 |  |  |  |  |  | 1 | 0 | 0 | 0 | 0 | 1 | 0 | 0 | 0 | 0 | 0 | 127 | 135 |  |
| R.ARLEC(+57.02)EINTYR.S | N | 49.72 | 1423.6877 | 11 | 0.3 | 475.5700 | 3 | 44.89 | 5 | F5:12054 | 29102019\_RID\_1313\_NaNaPb\_F4.raw |  |  |  |  | 7.57E5 |  |  |  |  |  | 1 | 0 | 0 | 0 | 0 | 1 | 0 | 0 | 0 | 0 | 0 | 347 | 357 | Carbamidomethylation |
| K.VRQLERDNAELENLIR.E | N | 48.97 | 1967.0548 | 16 | 0.3 | 492.7711 | 4 | 55.75 | 5 | F5:20433 | 29102019\_RID\_1313\_NaNaPb\_F4.raw |  |  |  |  | 3.44E6 |  |  |  |  |  | 1 | 0 | 0 | 0 | 0 | 1 | 0 | 0 | 0 | 0 | 0 | 74 | 89 |  |
| R.QLERDNAELENLIR.E | N | 48.55 | 1711.8853 | 14 | 0.2 | 571.6358 | 3 | 60.69 | 5 | F5:24620 | 29102019\_RID\_1313\_NaNaPb\_F4.raw |  |  |  |  | 9.29E6 |  |  |  |  |  | 2 | 0 | 0 | 0 | 0 | 2 | 0 | 0 | 0 | 0 | 0 | 76 | 89 |  |
| R.SQYEALVETNRR.E | N | 46.20 | 1464.7321 | 12 | 0.0 | 489.2513 | 3 | 43.37 | 5 | F5:10875 | 29102019\_RID\_1313\_NaNaPb\_F4.raw |  |  |  |  | 1.24E7 |  |  |  |  |  | 2 | 0 | 0 | 0 | 0 | 2 | 0 | 0 | 0 | 0 | 0 | 230 | 241 |  |
| total 23 peptides |
| --- |

Q15323|K1H1\_HUMAN

back to list

  

| Protein Coverage
| Supporting Peptides
|

Protein Coverage:

Supporting Peptides:

| Peptide | Uniq | -10lgP | Mass | Length | ppm | m/z | z | RT | Fraction | Scan | Source File | Area F1 | Area F10 | Area F2 | Area F3 | Area F4 | Area F5 | Area F6 | Area F7 | Area F8 | Area F9 | #Feature | #Feature F1 | #Feature F10 | #Feature F2 | #Feature F3 | #Feature F4 | #Feature F5 | #Feature F6 | #Feature F7 | #Feature F8 | #Feature F9 | Start | End | PTM |
| --- | --- | --- | --- | --- | --- | --- | --- | --- | --- | --- | --- | --- | --- | --- | --- | --- | --- | --- | --- | --- | --- | --- | --- | --- | --- | --- | --- | --- | --- | --- | --- | --- | --- | --- | --- |
| R.TVNALEIELQAQHNLRDSLENTLTESEAR.Y | N | 88.00 | 3293.6487 | 29 | 3.1 | 824.4220 | 4 | 79.92 | 5 | F5:40829 | 29102019\_RID\_1313\_NaNaPb\_F4.raw |  |  |  |  | 1.15E8 |  |  |  |  |  | 2 | 0 | 0 | 0 | 0 | 2 | 0 | 0 | 0 | 0 | 0 | 278 | 306 |  |
| R.TVNALEIELQAQHNLR.D | N | 85.94 | 1847.9854 | 16 | 1.5 | 617.0033 | 3 | 64.36 | 5 | F5:27680 | 29102019\_RID\_1313\_NaNaPb\_F4.raw |  |  |  |  | 7.74E7 |  |  |  |  |  | 2 | 0 | 0 | 0 | 0 | 2 | 0 | 0 | 0 | 0 | 0 | 278 | 293 |  |
| R.RTVNALEIELQAQHNLR.D | N | 85.38 | 2004.0864 | 17 | 1.7 | 669.0372 | 3 | 56.81 | 5 | F5:21335 | 29102019\_RID\_1313\_NaNaPb\_F4.raw |  |  |  |  | 8.49E6 |  |  |  |  |  | 1 | 0 | 0 | 0 | 0 | 1 | 0 | 0 | 0 | 0 | 0 | 277 | 293 |  |
| R.LVVQIDNAKLAADDFR.T | N | 82.77 | 1786.9576 | 16 | 0.8 | 596.6603 | 3 | 63.77 | 5 | F5:27296 | 29102019\_RID\_1313\_NaNaPb\_F4.raw |  |  |  |  | 1.65E7 |  |  |  |  |  | 2 | 0 | 0 | 0 | 0 | 2 | 0 | 0 | 0 | 0 | 0 | 127 | 142 |  |
| R.LVVQIDNAKLAADDFRTKYQTELSLR.Q | Y | 81.20 | 3006.6138 | 26 | 2.0 | 1003.2139 | 3 | 67.74 | 5 | F5:30696 | 29102019\_RID\_1313\_NaNaPb\_F4.raw |  |  |  |  | 2.43E6 |  |  |  |  |  | 2 | 0 | 0 | 0 | 0 | 2 | 0 | 0 | 0 | 0 | 0 | 127 | 152 |  |
| R.LVVQIDNAKLAADDFRTK.Y | N | 81.18 | 2016.1003 | 18 | -1.9 | 1009.0555 | 2 | 57.93 | 5 | F5:22304 | 29102019\_RID\_1313\_NaNaPb\_F4.raw |  |  |  |  | 3.78E7 |  |  |  |  |  | 4 | 0 | 0 | 0 | 0 | 4 | 0 | 0 | 0 | 0 | 0 | 127 | 144 |  |
| R.SDLERQNQEYQVLLDVR.A | N | 79.55 | 2104.0549 | 17 | 1.2 | 702.3597 | 3 | 65.80 | 5 | F5:28997 | 29102019\_RID\_1313\_NaNaPb\_F4.raw |  |  |  |  | 2.33E6 |  |  |  |  |  | 1 | 0 | 0 | 0 | 0 | 1 | 0 | 0 | 0 | 0 | 0 | 330 | 346 |  |
| R.QNQEYQVLLDVR.A | N | 78.64 | 1503.7681 | 12 | 0.6 | 752.8917 | 2 | 68.55 | 5 | F5:31362 | 29102019\_RID\_1313\_NaNaPb\_F4.raw |  |  |  |  | 4.7E6 |  |  |  |  |  | 1 | 0 | 0 | 0 | 0 | 1 | 0 | 0 | 0 | 0 | 0 | 335 | 346 |  |
| K.LAADDFRTKYQTELSLR.Q | Y | 75.07 | 2026.0483 | 17 | -0.3 | 507.5192 | 4 | 58.60 | 5 | F5:22799 | 29102019\_RID\_1313\_NaNaPb\_F4.raw |  |  |  |  | 2.43E6 |  |  |  |  |  | 1 | 0 | 0 | 0 | 0 | 1 | 0 | 0 | 0 | 0 | 0 | 136 | 152 |  |
| K.QVVSSSEQLQSYQAEIIELRR.T | N | 63.37 | 2462.2764 | 21 | 1.2 | 821.7671 | 3 | 67.06 | 5 | F5:30085 | 29102019\_RID\_1313\_NaNaPb\_F4.raw |  |  |  |  | 4.56E6 |  |  |  |  |  | 1 | 0 | 0 | 0 | 0 | 1 | 0 | 0 | 0 | 0 | 0 | 257 | 277 |  |
| R.TKYQTELSLR.Q | Y | 63.27 | 1237.6666 | 10 | 0.0 | 413.5628 | 3 | 44.13 | 5 | F5:11527 | 29102019\_RID\_1313\_NaNaPb\_F4.raw |  |  |  |  | 8.71E5 |  |  |  |  |  | 2 | 0 | 0 | 0 | 0 | 2 | 0 | 0 | 0 | 0 | 0 | 143 | 152 |  |
| K.YQTELSLRQLVESDINGLRR.I | Y | 61.98 | 2389.2712 | 20 | 0.3 | 797.4313 | 3 | 73.32 | 5 | F5:35347 | 29102019\_RID\_1313\_NaNaPb\_F4.raw |  |  |  |  | 1.05E7 |  |  |  |  |  | 1 | 0 | 0 | 0 | 0 | 1 | 0 | 0 | 0 | 0 | 0 | 145 | 164 |  |
| R.QLERDNAELENLIRER.S | N | 61.65 | 1997.0289 | 16 | 0.7 | 666.6841 | 3 | 57.99 | 5 | F5:22273 | 29102019\_RID\_1313\_NaNaPb\_F4.raw |  |  |  |  | 1.02E8 |  |  |  |  |  | 1 | 0 | 0 | 0 | 0 | 1 | 0 | 0 | 0 | 0 | 0 | 76 | 91 |  |
| R.EVEQWFTTQTEELNKQVVSSSEQLQSYQAEIIELRR.T | Y | 61.02 | 4325.1450 | 36 | 0.8 | 1082.2944 | 4 | 86.94 | 5 | F5:46766 | 29102019\_RID\_1313\_NaNaPb\_F4.raw |  |  |  |  | 1.82E6 |  |  |  |  |  | 2 | 0 | 0 | 0 | 0 | 2 | 0 | 0 | 0 | 0 | 0 | 242 | 277 |  |
| R.QLVESDINGLRR.I | N | 59.73 | 1398.7579 | 12 | 0.2 | 700.3864 | 2 | 48.02 | 5 | F5:14116 | 29102019\_RID\_1313\_NaNaPb\_F4.raw |  |  |  |  | 3.26E7 |  |  |  |  |  | 2 | 0 | 0 | 0 | 0 | 2 | 0 | 0 | 0 | 0 | 0 | 153 | 164 |  |
| K.YQTELSLR.Q | Y | 59.62 | 1008.5240 | 8 | 0.3 | 505.2694 | 2 | 52.23 | 5 | F5:17464 | 29102019\_RID\_1313\_NaNaPb\_F4.raw |  |  |  |  | 1.58E6 |  |  |  |  |  | 1 | 0 | 0 | 0 | 0 | 1 | 0 | 0 | 0 | 0 | 0 | 145 | 152 |  |
| R.QNQEYQVLLDVRAR.L | N | 58.17 | 1730.9064 | 14 | 0.9 | 577.9766 | 3 | 60.80 | 5 | F5:24777 | 29102019\_RID\_1313\_NaNaPb\_F4.raw |  |  |  |  | 1.2E6 |  |  |  |  |  | 1 | 0 | 0 | 0 | 0 | 1 | 0 | 0 | 0 | 0 | 0 | 335 | 348 |  |
| K.LAADDFR.T | N | 56.98 | 806.3922 | 7 | 0.4 | 404.2036 | 2 | 11.73 | 1 | F1:3258 | 29102019\_RID\_1313\_NaNaPb\_F1.raw | 1E5 | 2.78E5 | 1.15E5 |  |  | 1.11E5 | 5.12E4 |  | 2.99E4 |  | 6 | 1 | 1 | 1 | 0 | 0 | 1 | 1 | 0 | 1 | 0 | 136 | 142 |  |
| R.LVVQIDNAK.L | N | 52.63 | 998.5760 | 9 | -0.2 | 500.2952 | 2 | 48.54 | 5 | F5:14488 | 29102019\_RID\_1313\_NaNaPb\_F4.raw |  |  |  |  | 2.26E7 |  |  |  |  |  | 1 | 0 | 0 | 0 | 0 | 1 | 0 | 0 | 0 | 0 | 0 | 127 | 135 |  |
| R.ARLEC(+57.02)EINTYR.S | N | 49.72 | 1423.6877 | 11 | 0.3 | 475.5700 | 3 | 44.89 | 5 | F5:12054 | 29102019\_RID\_1313\_NaNaPb\_F4.raw |  |  |  |  | 7.57E5 |  |  |  |  |  | 1 | 0 | 0 | 0 | 0 | 1 | 0 | 0 | 0 | 0 | 0 | 347 | 357 | Carbamidomethylation |
| K.VRQLERDNAELENLIR.E | N | 48.97 | 1967.0548 | 16 | 0.3 | 492.7711 | 4 | 55.75 | 5 | F5:20433 | 29102019\_RID\_1313\_NaNaPb\_F4.raw |  |  |  |  | 3.44E6 |  |  |  |  |  | 1 | 0 | 0 | 0 | 0 | 1 | 0 | 0 | 0 | 0 | 0 | 74 | 89 |  |
| R.QLERDNAELENLIR.E | N | 48.55 | 1711.8853 | 14 | 0.2 | 571.6358 | 3 | 60.69 | 5 | F5:24620 | 29102019\_RID\_1313\_NaNaPb\_F4.raw |  |  |  |  | 9.29E6 |  |  |  |  |  | 2 | 0 | 0 | 0 | 0 | 2 | 0 | 0 | 0 | 0 | 0 | 76 | 89 |  |
| R.SQYEALVETNRR.E | N | 46.20 | 1464.7321 | 12 | 0.0 | 489.2513 | 3 | 43.37 | 5 | F5:10875 | 29102019\_RID\_1313\_NaNaPb\_F4.raw |  |  |  |  | 1.24E7 |  |  |  |  |  | 2 | 0 | 0 | 0 | 0 | 2 | 0 | 0 | 0 | 0 | 0 | 230 | 241 |  |
| total 23 peptides |
| --- |

P25498|PA2AE\_NAJOX

back to list

  

| Protein Coverage
| Supporting Peptides
|

Protein Coverage:

Supporting Peptides:

| Peptide | Uniq | -10lgP | Mass | Length | ppm | m/z | z | RT | Fraction | Scan | Source File | Area F1 | Area F10 | Area F2 | Area F3 | Area F4 | Area F5 | Area F6 | Area F7 | Area F8 | Area F9 | #Feature | #Feature F1 | #Feature F10 | #Feature F2 | #Feature F3 | #Feature F4 | #Feature F5 | #Feature F6 | #Feature F7 | #Feature F8 | #Feature F9 | Start | End | PTM |
| --- | --- | --- | --- | --- | --- | --- | --- | --- | --- | --- | --- | --- | --- | --- | --- | --- | --- | --- | --- | --- | --- | --- | --- | --- | --- | --- | --- | --- | --- | --- | --- | --- | --- | --- | --- |
| K.TYSYEC(+57.02)SQGTLTC(+57.02)K.G | N | 117.54 | 1696.7073 | 14 | 0.1 | 849.3610 | 2 | 11.33 | 9 | F9:2180 | 29102019\_RID\_1313\_NaNaPb\_F8.raw |  | 6.41E5 |  |  |  | 3.72E8 | 4.1E5 | 2.61E4 | 2.12E7 | 1.61E6 | 22 | 0 | 1 | 0 | 0 | 0 | 17 | 1 | 1 | 1 | 1 | 66 | 79 | Carbamidomethylation |
| T.YSYEC(+57.02)SQGTLTC(+57.02)K.G | N | 95.19 | 1595.6595 | 13 | -0.2 | 798.8369 | 2 | 11.29 | 9 | F9:2138 | 29102019\_RID\_1313\_NaNaPb\_F8.raw |  |  |  |  |  |  |  |  | 2.43E6 |  | 1 | 0 | 0 | 0 | 0 | 0 | 0 | 0 | 0 | 1 | 0 | 67 | 79 | Carbamidomethylation |
| Y.SYEC(+57.02)SQGTLTC(+57.02)K.G | N | 93.04 | 1432.5963 | 12 | 0.7 | 717.3059 | 2 | 11.00 | 9 | F9:1851 | 29102019\_RID\_1313\_NaNaPb\_F8.raw |  | 2.83E4 |  |  |  | 3.69E5 |  |  | 1.21E7 |  | 3 | 0 | 1 | 0 | 0 | 0 | 1 | 0 | 0 | 1 | 0 | 68 | 79 | Carbamidomethylation |
| R.GGSGTPVDDLDR.C | N | 79.46 | 1187.5417 | 12 | -1.4 | 594.7773 | 2 | 11.42 | 7 | F7:2302 | 29102019\_RID\_1313\_NaNaPb\_F6.raw |  | 3.83E5 |  |  |  | 9.47E5 | 2.08E5 |  |  | 7.26E5 | 10 | 0 | 1 | 0 | 0 | 0 | 7 | 1 | 0 | 0 | 1 | 31 | 42 |  |
| K.TYSYEC(+57.02)SQGTLTC(+57.02)KG.D | N | 76.57 | 1753.7288 | 15 | 0.9 | 877.8724 | 2 | 11.37 | 9 | F9:2181 | 29102019\_RID\_1313\_NaNaPb\_F8.raw |  |  |  |  |  |  |  |  | 6.41E6 |  | 1 | 0 | 0 | 0 | 0 | 0 | 0 | 0 | 0 | 1 | 0 | 66 | 80 | Carbamidomethylation |
| K.ISGC(+57.02)WPYFK.T | N | 73.47 | 1156.5375 | 9 | 0.7 | 579.2764 | 2 | 16.15 | 6 | F6:5636 | 29102019\_RID\_1313\_NaNaPb\_F5.raw |  | 4.91E5 |  |  |  | 7.32E8 |  |  | 9.7E6 | 1.42E6 | 16 | 0 | 1 | 0 | 0 | 0 | 11 | 0 | 0 | 3 | 1 | 57 | 65 | Carbamidomethylation |
| S.YEC(+57.02)SQGTLTC(+57.02)K.G | N | 69.49 | 1345.5642 | 11 | 0.0 | 673.7894 | 2 | 10.91 | 9 | F9:1786 | 29102019\_RID\_1313\_NaNaPb\_F8.raw |  |  |  |  |  |  |  |  | 1.8E5 |  | 1 | 0 | 0 | 0 | 0 | 0 | 0 | 0 | 0 | 1 | 0 | 69 | 79 | Carbamidomethylation |
| R.LAAIC(+57.02)FAGAPYN.N | N | 58.35 | 1266.6067 | 12 | 0.1 | 634.3107 | 2 | 24.51 | 6 | F6:9784 | 29102019\_RID\_1313\_NaNaPb\_F5.raw |  |  |  |  |  | 2.29E6 |  |  | 1.49E5 |  | 3 | 0 | 0 | 0 | 0 | 0 | 2 | 0 | 0 | 1 | 0 | 95 | 106 | Carbamidomethylation |
| K.TYSYEC(+57.02)SQGTL.T | N | 56.52 | 1307.5339 | 11 | 0.9 | 654.7748 | 2 | 12.20 | 6 | F6:2929 | 29102019\_RID\_1313\_NaNaPb\_F5.raw |  |  |  |  |  | 8.99E5 |  |  |  |  | 1 | 0 | 0 | 0 | 0 | 0 | 1 | 0 | 0 | 0 | 0 | 66 | 76 | Carbamidomethylation |
| R.LAAIC(+57.02)FAGAPYNNDNYNINLK.A | Y | 51.83 | 2355.1318 | 21 | 1.2 | 1178.5746 | 2 | 23.41 | 6 | F6:9162 | 29102019\_RID\_1313\_NaNaPb\_F5.raw |  |  |  |  |  | 4.13E5 |  |  |  |  | 1 | 0 | 0 | 0 | 0 | 0 | 1 | 0 | 0 | 0 | 0 | 95 | 115 | Carbamidomethylation |
| R.LAAIC(+57.02)FAGAPY.N | N | 48.00 | 1152.5637 | 11 | -0.1 | 577.2891 | 2 | 29.05 | 9 | F9:11682 | 29102019\_RID\_1313\_NaNaPb\_F8.raw |  |  |  |  |  |  |  |  | 1.7E6 |  | 1 | 0 | 0 | 0 | 0 | 0 | 0 | 0 | 0 | 1 | 0 | 95 | 105 | Carbamidomethylation |
| G.SGTPVDDLDR.C | N | 47.92 | 1073.4989 | 10 | -2.3 | 537.7555 | 2 | 11.45 | 6 | F6:2250 | 29102019\_RID\_1313\_NaNaPb\_F5.raw |  |  |  |  |  | 2.06E5 |  |  |  |  | 1 | 0 | 0 | 0 | 0 | 0 | 1 | 0 | 0 | 0 | 0 | 33 | 42 |  |
| S.GTPVDDLDR.C | N | 47.24 | 986.4669 | 9 | 0.0 | 494.2407 | 2 | 11.37 | 9 | F9:2225 | 29102019\_RID\_1313\_NaNaPb\_F8.raw |  |  |  |  |  |  |  |  | 5.17E4 |  | 1 | 0 | 0 | 0 | 0 | 0 | 0 | 0 | 0 | 1 | 0 | 34 | 42 |  |
| K.TYSYEC(+57.02)SQGTLT.C | N | 46.12 | 1408.5817 | 12 | -1.9 | 705.2968 | 2 | 11.97 | 6 | F6:2736 | 29102019\_RID\_1313\_NaNaPb\_F5.raw |  |  |  |  |  | 2.41E5 |  |  |  |  | 1 | 0 | 0 | 0 | 0 | 0 | 1 | 0 | 0 | 0 | 0 | 66 | 77 | Carbamidomethylation |
| total 14 peptides |
| --- |

P48668|K2C6C\_HUMAN

back to list

  

| Protein Coverage
| Supporting Peptides
|

Protein Coverage:

Supporting Peptides:

| Peptide | Uniq | -10lgP | Mass | Length | ppm | m/z | z | RT | Fraction | Scan | Source File | Area F1 | Area F10 | Area F2 | Area F3 | Area F4 | Area F5 | Area F6 | Area F7 | Area F8 | Area F9 | #Feature | #Feature F1 | #Feature F10 | #Feature F2 | #Feature F3 | #Feature F4 | #Feature F5 | #Feature F6 | #Feature F7 | #Feature F8 | #Feature F9 | Start | End | PTM |
| --- | --- | --- | --- | --- | --- | --- | --- | --- | --- | --- | --- | --- | --- | --- | --- | --- | --- | --- | --- | --- | --- | --- | --- | --- | --- | --- | --- | --- | --- | --- | --- | --- | --- | --- | --- |
| R.ISIGGGSC(+57.02)AISGGYGSR.A | N | 91.11 | 1597.7518 | 17 | -0.9 | 799.8824 | 2 | 11.83 | 6 | F6:2612 | 29102019\_RID\_1313\_NaNaPb\_F5.raw | 2.32E4 | 0 | 6.66E4 |  |  | 1.59E5 |  |  |  |  | 4 | 1 | 1 | 1 | 0 | 0 | 1 | 0 | 0 | 0 | 0 | 70 | 86 | Carbamidomethylation |
| K.WTLLQEQGTK.T | N | 80.50 | 1202.6295 | 10 | -0.5 | 602.3217 | 2 | 12.43 | 7 | F7:3159 | 29102019\_RID\_1313\_NaNaPb\_F6.raw |  |  | 2.43E5 |  |  |  | 2.98E5 |  |  |  | 2 | 0 | 0 | 1 | 0 | 0 | 0 | 1 | 0 | 0 | 0 | 195 | 204 |  |
| R.NLDLDSIIAEVK.A | N | 79.85 | 1328.7188 | 12 | 0.3 | 665.3669 | 2 | 34.39 | 9 | F9:13181 | 29102019\_RID\_1313\_NaNaPb\_F8.raw | 6.3E5 | 2.03E6 | 4.86E5 | 7.73E4 |  | 1.95E6 | 1.33E6 |  | 3.92E5 | 1.4E5 | 9 | 2 | 1 | 1 | 1 | 0 | 1 | 1 | 0 | 1 | 1 | 327 | 338 |  |
| K.ADTLTDEINFLR.A | N | 77.98 | 1406.7041 | 12 | 0.9 | 704.3600 | 2 | 25.09 | 7 | F7:9129 | 29102019\_RID\_1313\_NaNaPb\_F6.raw | 5.86E4 |  | 1.22E5 |  |  | 3.43E5 | 2.52E5 |  | 2.12E4 |  | 5 | 1 | 0 | 1 | 0 | 0 | 1 | 1 | 0 | 1 | 0 | 288 | 299 |  |
| K.TLNNKFASFIDKVR.F | N | 77.96 | 1651.9045 | 14 | 0.2 | 413.9835 | 4 | 59.92 | 5 | F5:23986 | 29102019\_RID\_1313\_NaNaPb\_F4.raw |  |  |  |  | 1.32E6 |  |  |  |  |  | 2 | 0 | 0 | 0 | 0 | 2 | 0 | 0 | 0 | 0 | 0 | 169 | 182 |  |
| R.FLEQQNKVLDTKWTLLQEQGTK.T | N | 76.77 | 2646.4016 | 22 | 0.9 | 883.1420 | 3 | 63.86 | 5 | F5:27315 | 29102019\_RID\_1313\_NaNaPb\_F4.raw |  |  |  |  | 2.59E6 |  |  |  |  |  | 1 | 0 | 0 | 0 | 0 | 1 | 0 | 0 | 0 | 0 | 0 | 183 | 204 |  |
| K.AQYEEIAQR.S | N | 75.95 | 1106.5356 | 9 | -0.6 | 554.2748 | 2 | 11.33 | 1 | F1:2875 | 29102019\_RID\_1313\_NaNaPb\_F1.raw | 7.34E4 | 1.94E5 | 7.33E4 |  |  | 8.43E4 | 1.12E5 |  |  |  | 5 | 1 | 1 | 1 | 0 | 0 | 1 | 1 | 0 | 0 | 0 | 339 | 347 |  |
| K.LALDVEIATYR.K | N | 72.02 | 1262.6870 | 11 | 1.0 | 632.3514 | 2 | 18.69 | 2 | F2:6620 | 29102019\_RID\_1313\_NaNaPb\_F10.raw | 1.06E5 | 5.46E5 | 3.1E4 | 1.25E4 |  | 1.24E6 | 1.83E5 |  |  |  | 8 | 1 | 2 | 1 | 1 | 0 | 2 | 1 | 0 | 0 | 0 | 456 | 466 |  |
| R.TAAENEFVTLK.K | N | 67.67 | 1221.6240 | 11 | 0.1 | 611.8193 | 2 | 12.01 | 6 | F6:2795 | 29102019\_RID\_1313\_NaNaPb\_F5.raw | 3.05E4 | 7.11E4 | 8.52E4 |  |  | 3.39E4 |  |  |  | 5.49E4 | 5 | 1 | 1 | 1 | 0 | 0 | 1 | 0 | 0 | 0 | 1 | 261 | 271 |  |
| R.SGFSSISVSR.S | N | 65.41 | 1025.5142 | 10 | -1.0 | 513.7639 | 2 | 51.05 | 5 | F5:16549 | 29102019\_RID\_1313\_NaNaPb\_F4.raw |  |  | 1.16E4 |  | 3.88E5 |  | 0 |  |  |  | 3 | 0 | 0 | 1 | 0 | 1 | 0 | 1 | 0 | 0 | 0 | 31 | 40 |  |
| R.AIGGGLSSVGGGSSTIK.Y | Y | 63.04 | 1446.7678 | 17 | 0.6 | 724.3916 | 2 | 11.72 | 6 | F6:2517 | 29102019\_RID\_1313\_NaNaPb\_F5.raw |  |  |  |  |  | 5.2E4 |  |  |  | 2.76E4 | 2 | 0 | 0 | 0 | 0 | 0 | 1 | 0 | 0 | 0 | 1 | 534 | 550 |  |
| R.QLDSIVGER.G | N | 62.07 | 1015.5298 | 9 | 0.3 | 508.7723 | 2 | 11.95 | 1 | F1:3454 | 29102019\_RID\_1313\_NaNaPb\_F1.raw | 3.34E4 | 7.21E4 | 9.99E4 |  |  |  |  |  |  |  | 3 | 1 | 1 | 1 | 0 | 0 | 0 | 0 | 0 | 0 | 0 | 224 | 232 |  |
| K.LEGLEDALQK.A | N | 61.53 | 1114.5869 | 10 | 0.0 | 558.3007 | 2 | 12.16 | 2 | F2:3060 | 29102019\_RID\_1313\_NaNaPb\_F10.raw |  | 5.06E4 | 6.7E4 |  |  | 0 |  |  |  | 4.81E4 | 4 | 0 | 1 | 1 | 0 | 0 | 1 | 0 | 0 | 0 | 1 | 427 | 436 |  |
| R.AEAESWYQTK.Y | N | 61.48 | 1211.5459 | 10 | 0.9 | 606.7808 | 2 | 11.68 | 3 | F3:2510 | 29102019\_RID\_1313\_NaNaPb\_F2.raw |  |  | 2.42E4 |  |  |  |  |  |  |  | 1 | 0 | 0 | 1 | 0 | 0 | 0 | 0 | 0 | 0 | 0 | 350 | 359 |  |
| R.QNLEPLFEQYINNLR.R | N | 59.91 | 1889.9635 | 15 | 0.4 | 630.9954 | 3 | 39.85 | 9 | F9:15005 | 29102019\_RID\_1313\_NaNaPb\_F8.raw |  | 1.19E5 |  |  |  |  | 1.03E5 |  | 1.04E4 |  | 3 | 0 | 1 | 0 | 0 | 0 | 0 | 1 | 0 | 1 | 0 | 208 | 222 |  |
| K.NKYEDEINKR.T | N | 59.85 | 1307.6470 | 10 | -0.3 | 436.8895 | 3 | 10.75 | 6 | F6:1631 | 29102019\_RID\_1313\_NaNaPb\_F5.raw |  | 2.17E5 | 1.52E5 |  |  | 3.47E4 | 2.69E5 |  |  |  | 4 | 0 | 1 | 1 | 0 | 0 | 1 | 1 | 0 | 0 | 0 | 251 | 260 |  |
| R.AIGGGLSSVGGGSSTIKYTTTSSSSR.K | Y | 57.31 | 2417.2034 | 26 | 3.0 | 806.7441 | 3 | 50.20 | 5 | F5:15896 | 29102019\_RID\_1313\_NaNaPb\_F4.raw |  |  |  |  | 2.45E5 |  |  |  |  |  | 1 | 0 | 0 | 0 | 0 | 1 | 0 | 0 | 0 | 0 | 0 | 534 | 559 |  |
| K.LLEGEEC(+57.02)R.L | N | 53.11 | 1004.4597 | 8 | 0.4 | 503.2373 | 2 | 11.25 | 3 | F3:2079 | 29102019\_RID\_1313\_NaNaPb\_F2.raw |  |  | 3.26E4 |  |  |  |  |  |  |  | 1 | 0 | 0 | 1 | 0 | 0 | 0 | 0 | 0 | 0 | 0 | 468 | 475 | Carbamidomethylation |
| R.GSGGLGGAC(+57.02)GGAGFGSR.S | N | 50.53 | 1423.6262 | 17 | 0.9 | 712.8210 | 2 | 11.60 | 3 | F3:2418 | 29102019\_RID\_1313\_NaNaPb\_F2.raw |  |  | 2.15E4 |  |  |  |  |  |  |  | 1 | 0 | 0 | 1 | 0 | 0 | 0 | 0 | 0 | 0 | 0 | 43 | 59 | Carbamidomethylation |
| K.FASFIDK.V | N | 46.57 | 826.4225 | 7 | -0.3 | 414.2184 | 2 | 11.89 | 2 | F2:2810 | 29102019\_RID\_1313\_NaNaPb\_F10.raw |  | 1.96E5 |  |  |  | 3.22E5 |  |  | 0 |  | 3 | 0 | 1 | 0 | 0 | 0 | 1 | 0 | 0 | 1 | 0 | 174 | 180 |  |
| K.QEIAEINR.M | N | 42.98 | 971.5036 | 8 | 0.3 | 486.7592 | 2 | 11.39 | 3 | F3:2208 | 29102019\_RID\_1313\_NaNaPb\_F2.raw |  |  | 0 |  |  |  |  |  |  |  | 1 | 0 | 0 | 1 | 0 | 0 | 0 | 0 | 0 | 0 | 0 | 379 | 386 |  |
| total 21 peptides |
| --- |

P01445|3SA7A\_NAJKA

back to list

  

| Protein Coverage
| Supporting Peptides
|

Protein Coverage:

Supporting Peptides:

| Peptide | Uniq | -10lgP | Mass | Length | ppm | m/z | z | RT | Fraction | Scan | Source File | Area F1 | Area F10 | Area F2 | Area F3 | Area F4 | Area F5 | Area F6 | Area F7 | Area F8 | Area F9 | #Feature | #Feature F1 | #Feature F10 | #Feature F2 | #Feature F3 | #Feature F4 | #Feature F5 | #Feature F6 | #Feature F7 | #Feature F8 | #Feature F9 | Start | End | PTM |
| --- | --- | --- | --- | --- | --- | --- | --- | --- | --- | --- | --- | --- | --- | --- | --- | --- | --- | --- | --- | --- | --- | --- | --- | --- | --- | --- | --- | --- | --- | --- | --- | --- | --- | --- | --- |
| K.NSLLVKYVC(+57.02)C(+57.02)NTDRC(+57.02)N | N | 99.64 | 2014.9023 | 16 | 1.0 | 672.6421 | 3 | 11.67 | 9 | F9:2475 | 29102019\_RID\_1313\_NaNaPb\_F8.raw |  |  |  |  |  |  |  |  | 4.5E6 |  | 2 | 0 | 0 | 0 | 0 | 0 | 0 | 0 | 0 | 2 | 0 | 45 | 60 | Carbamidomethylation |
| R.GC(+57.02)IDVC(+57.02)PKNSLLVKYVC(+57.02)C(+57.02)NTDRC(+57.02)N | N | 98.28 | 2944.3123 | 24 | -0.6 | 737.0849 | 4 | 12.04 | 9 | F9:2774 | 29102019\_RID\_1313\_NaNaPb\_F8.raw |  |  |  |  |  | 1.42E5 |  |  | 6.93E6 |  | 3 | 0 | 0 | 0 | 0 | 0 | 1 | 0 | 0 | 2 | 0 | 37 | 60 | Carbamidomethylation |
| LKC(+57.02)NKLIPLAYKTC(+57.02)PAGK.N | N | 81.39 | 2074.1431 | 18 | 0.2 | 519.5432 | 4 | 11.24 | 9 | F9:2117 | 29102019\_RID\_1313\_NaNaPb\_F8.raw |  |  |  |  |  |  |  |  | 1.34E6 |  | 3 | 0 | 0 | 0 | 0 | 0 | 0 | 0 | 0 | 3 | 0 | 1 | 18 | Carbamidomethylation |
| LKC(+57.02)NKLIPLAYK.T | N | 78.87 | 1459.8584 | 12 | 0.7 | 487.6271 | 3 | 11.44 | 9 | F9:2278 | 29102019\_RID\_1313\_NaNaPb\_F8.raw |  |  |  |  |  | 2.11E5 |  |  | 7.53E5 |  | 2 | 0 | 0 | 0 | 0 | 0 | 1 | 0 | 0 | 1 | 0 | 1 | 12 | Carbamidomethylation |
| K.LIPLAYKTC(+57.02)PAGK.N | N | 69.00 | 1430.7955 | 13 | 0.4 | 477.9393 | 3 | 11.66 | 6 | F6:2424 | 29102019\_RID\_1313\_NaNaPb\_F5.raw |  |  |  |  |  | 1.34E6 |  |  |  |  | 2 | 0 | 0 | 0 | 0 | 0 | 2 | 0 | 0 | 0 | 0 | 6 | 18 | Carbamidomethylation |
| R.GC(+57.02)IDVC(+57.02)PKNSLLVK.Y | N | 68.29 | 1601.8269 | 14 | 0.8 | 534.9500 | 3 | 11.67 | 9 | F9:2478 | 29102019\_RID\_1313\_NaNaPb\_F8.raw |  |  |  |  |  | 6.01E5 |  |  | 3.42E5 |  | 3 | 0 | 0 | 0 | 0 | 0 | 2 | 0 | 0 | 1 | 0 | 37 | 50 | Carbamidomethylation |
| I.PLAYKTC(+57.02)PAGK.N | N | 64.86 | 1204.6274 | 11 | 1.1 | 603.3217 | 2 | 11.66 | 6 | F6:2431 | 29102019\_RID\_1313\_NaNaPb\_F5.raw |  |  |  |  |  | 1.89E5 |  |  |  |  | 1 | 0 | 0 | 0 | 0 | 0 | 1 | 0 | 0 | 0 | 0 | 8 | 18 | Carbamidomethylation |
| LKC(+57.02)NKLIPLAY.K | N | 63.90 | 1331.7635 | 11 | 0.5 | 666.8894 | 2 | 12.12 | 9 | F9:2846 | 29102019\_RID\_1313\_NaNaPb\_F8.raw |  |  |  |  |  |  |  |  | 9.2E5 |  | 1 | 0 | 0 | 0 | 0 | 0 | 0 | 0 | 0 | 1 | 0 | 1 | 11 | Carbamidomethylation |
| R.GC(+57.02)IDVC(+57.02)PK.N | N | 63.51 | 947.4205 | 8 | -0.6 | 474.7172 | 2 | 11.17 | 7 | F7:2031 | 29102019\_RID\_1313\_NaNaPb\_F6.raw |  | 2.85E7 |  |  |  | 7.32E7 | 1.17E6 |  | 5.42E6 | 9.79E6 | 10 | 0 | 2 | 0 | 0 | 0 | 5 | 1 | 0 | 1 | 1 | 37 | 44 | Carbamidomethylation |
| L.VKYVC(+57.02)C(+57.02)NTDRC(+57.02)N | N | 63.21 | 1587.6592 | 12 | 0.8 | 530.2274 | 3 | 10.67 | 9 | F9:1583 | 29102019\_RID\_1313\_NaNaPb\_F8.raw |  |  |  |  |  |  |  |  | 2.28E6 |  | 2 | 0 | 0 | 0 | 0 | 0 | 0 | 0 | 0 | 2 | 0 | 49 | 60 | Carbamidomethylation |
| K.LIPLAYKTC(+57.02)PAGKNLC(+57.02)YK.M | Y | 60.17 | 2109.1113 | 18 | 0.0 | 528.2851 | 4 | 11.68 | 6 | F6:2469 | 29102019\_RID\_1313\_NaNaPb\_F5.raw |  |  |  |  |  | 1.08E5 |  |  | 1.71E5 |  | 2 | 0 | 0 | 0 | 0 | 0 | 1 | 0 | 0 | 1 | 0 | 6 | 23 | Carbamidomethylation |
| N.SLLVKYVC(+57.02)C(+57.02)NTDRC(+57.02)N | N | 56.59 | 1900.8594 | 15 | 0.6 | 951.4375 | 2 | 11.57 | 9 | F9:2415 | 29102019\_RID\_1313\_NaNaPb\_F8.raw |  |  |  |  |  |  |  |  | 7.39E5 |  | 2 | 0 | 0 | 0 | 0 | 0 | 0 | 0 | 0 | 2 | 0 | 46 | 60 | Carbamidomethylation |
| K.TC(+57.02)PAGKNLC(+57.02)YK.M | N | 54.37 | 1310.6111 | 11 | -0.4 | 437.8775 | 3 | 10.78 | 6 | F6:1669 | 29102019\_RID\_1313\_NaNaPb\_F5.raw |  |  |  |  |  | 3.23E4 |  |  |  |  | 1 | 0 | 0 | 0 | 0 | 0 | 1 | 0 | 0 | 0 | 0 | 13 | 23 | Carbamidomethylation |
| L.LVKYVC(+57.02)C(+57.02)NTDRC(+57.02)N | N | 54.08 | 1700.7433 | 13 | 0.0 | 567.9217 | 3 | 10.88 | 9 | F9:1785 | 29102019\_RID\_1313\_NaNaPb\_F8.raw |  |  |  |  |  |  |  |  | 2.77E5 |  | 1 | 0 | 0 | 0 | 0 | 0 | 0 | 0 | 0 | 1 | 0 | 48 | 60 | Carbamidomethylation |
| K.RGC(+57.02)IDVC(+57.02)PK.N | N | 48.01 | 1103.5216 | 9 | 0.2 | 552.7682 | 2 | 10.91 | 6 | F6:1732 | 29102019\_RID\_1313\_NaNaPb\_F5.raw |  |  |  |  |  | 6.74E5 |  |  |  |  | 1 | 0 | 0 | 0 | 0 | 0 | 1 | 0 | 0 | 0 | 0 | 36 | 44 | Carbamidomethylation |
| K.TVPVKRGC(+57.02)IDVC(+57.02)PK.N | N | 43.40 | 1627.8538 | 14 | -0.5 | 543.6249 | 3 | 10.93 | 9 | F9:1828 | 29102019\_RID\_1313\_NaNaPb\_F8.raw |  |  |  |  |  |  |  |  | 1.02E5 |  | 1 | 0 | 0 | 0 | 0 | 0 | 0 | 0 | 0 | 1 | 0 | 31 | 44 | Carbamidomethylation |
| total 16 peptides |
| --- |

P25674|3L21\_NAJHH

back to list

  

| Protein Coverage
| Supporting Peptides
|

Protein Coverage:

Supporting Peptides:

| Peptide | Uniq | -10lgP | Mass | Length | ppm | m/z | z | RT | Fraction | Scan | Source File | Area F1 | Area F10 | Area F2 | Area F3 | Area F4 | Area F5 | Area F6 | Area F7 | Area F8 | Area F9 | #Feature | #Feature F1 | #Feature F10 | #Feature F2 | #Feature F3 | #Feature F4 | #Feature F5 | #Feature F6 | #Feature F7 | #Feature F8 | #Feature F9 | Start | End | PTM |
| --- | --- | --- | --- | --- | --- | --- | --- | --- | --- | --- | --- | --- | --- | --- | --- | --- | --- | --- | --- | --- | --- | --- | --- | --- | --- | --- | --- | --- | --- | --- | --- | --- | --- | --- | --- |
| R.VDLGC(+57.02)AATC(+57.02)PTVK.P | N | 106.40 | 1390.6584 | 13 | -2.6 | 696.3347 | 2 | 11.56 | 7 | F7:2411 | 29102019\_RID\_1313\_NaNaPb\_F6.raw |  | 4.11E5 |  |  | 8.35E7 | 5.37E6 | 2.51E8 | 1.46E4 |  | 5.65E5 | 23 | 0 | 1 | 0 | 0 | 2 | 1 | 17 | 1 | 0 | 1 | 37 | 49 | Carbamidomethylation |
| K.C(+57.02)C(+57.02)STDNC(+57.02)NPFPTR.K | N | 95.57 | 1627.6178 | 13 | 0.5 | 814.8166 | 2 | 11.44 | 7 | F7:2301 | 29102019\_RID\_1313\_NaNaPb\_F6.raw |  |  |  |  |  |  | 8.42E6 |  |  |  | 3 | 0 | 0 | 0 | 0 | 0 | 0 | 3 | 0 | 0 | 0 | 56 | 68 | Carbamidomethylation |
| K.RVDLGC(+57.02)AATC(+57.02)PTVK.P | N | 88.57 | 1546.7595 | 14 | -0.4 | 516.5936 | 3 | 11.29 | 7 | F7:2135 | 29102019\_RID\_1313\_NaNaPb\_F6.raw |  |  |  |  | 1.37E7 |  | 1.8E6 |  |  |  | 3 | 0 | 0 | 0 | 0 | 1 | 0 | 2 | 0 | 0 | 0 | 36 | 49 | Carbamidomethylation |
| C.STDNC(+57.02)NPFPTR.K | N | 87.32 | 1307.5564 | 11 | -1.0 | 654.7848 | 2 | 11.42 | 7 | F7:2239 | 29102019\_RID\_1313\_NaNaPb\_F6.raw |  | 1.27E5 | 4.52E4 |  |  | 4.03E5 | 6.19E7 |  |  | 2.64E4 | 7 | 0 | 1 | 1 | 0 | 0 | 1 | 3 | 0 | 0 | 1 | 58 | 68 | Carbamidomethylation |
| C.C(+57.02)STDNC(+57.02)NPFPTR.K | N | 86.29 | 1467.5872 | 12 | -1.6 | 734.7997 | 2 | 11.42 | 7 | F7:2280 | 29102019\_RID\_1313\_NaNaPb\_F6.raw |  |  | 6.23E4 |  |  |  | 2.57E6 |  |  |  | 2 | 0 | 0 | 1 | 0 | 0 | 0 | 1 | 0 | 0 | 0 | 57 | 68 | Carbamidomethylation |
| C.PDGHVC(+57.02)YTK.M | Y | 85.75 | 1075.4757 | 9 | -1.2 | 538.7445 | 2 | 10.78 | 7 | F7:1611 | 29102019\_RID\_1313\_NaNaPb\_F6.raw |  |  |  |  |  |  | 2.2E7 |  |  |  | 1 | 0 | 0 | 0 | 0 | 0 | 0 | 1 | 0 | 0 | 0 | 15 | 23 | Carbamidomethylation |
| S.TDNC(+57.02)NPFPTR.K | N | 67.39 | 1220.5244 | 10 | -1.8 | 611.2684 | 2 | 11.42 | 7 | F7:2303 | 29102019\_RID\_1313\_NaNaPb\_F6.raw |  |  |  |  |  |  | 1.46E5 |  |  |  | 1 | 0 | 0 | 0 | 0 | 0 | 0 | 1 | 0 | 0 | 0 | 59 | 68 | Carbamidomethylation |
| T.DNC(+57.02)NPFPTR.K | N | 61.51 | 1119.4767 | 9 | -0.6 | 560.7453 | 2 | 11.77 | 1 | F1:3298 | 29102019\_RID\_1313\_NaNaPb\_F1.raw | 1.81E4 |  |  |  |  |  |  |  |  |  | 1 | 1 | 0 | 0 | 0 | 0 | 0 | 0 | 0 | 0 | 0 | 60 | 68 | Carbamidomethylation |
| K.RVDLGC(+57.02)AATC(+57.02)PT.V | N | 61.14 | 1319.5962 | 12 | -1.8 | 660.8042 | 2 | 11.42 | 7 | F7:2282 | 29102019\_RID\_1313\_NaNaPb\_F6.raw |  |  |  |  |  |  | 5.44E5 |  |  |  | 1 | 0 | 0 | 0 | 0 | 0 | 0 | 1 | 0 | 0 | 0 | 36 | 47 | Carbamidomethylation |
| R.VDLGC(+57.02)AATC(+57.02)PT.V | N | 59.60 | 1163.4951 | 11 | 0.0 | 582.7548 | 2 | 11.91 | 7 | F7:2756 | 29102019\_RID\_1313\_NaNaPb\_F6.raw |  |  |  |  |  |  | 8.06E5 |  |  |  | 1 | 0 | 0 | 0 | 0 | 0 | 0 | 1 | 0 | 0 | 0 | 37 | 47 | Carbamidomethylation |
| R.VDLGC(+57.02)AATC(+57.02)PTV.K | N | 57.22 | 1262.5635 | 12 | -0.8 | 632.2885 | 2 | 13.91 | 7 | F7:4209 | 29102019\_RID\_1313\_NaNaPb\_F6.raw |  |  |  |  |  |  | 0 |  |  |  | 1 | 0 | 0 | 0 | 0 | 0 | 0 | 1 | 0 | 0 | 0 | 37 | 48 | Carbamidomethylation |
| A.C(+57.02)PDGHVC(+57.02)YTK.M | Y | 55.10 | 1235.5063 | 10 | -4.6 | 412.8409 | 3 | 10.69 | 7 | F7:1599 | 29102019\_RID\_1313\_NaNaPb\_F6.raw |  |  |  |  |  |  | 9.45E5 |  |  |  | 1 | 0 | 0 | 0 | 0 | 0 | 0 | 1 | 0 | 0 | 0 | 14 | 23 | Carbamidomethylation |
| R.VDLGC(+57.02)AATC(+57.02)P.T | N | 54.00 | 1062.4474 | 10 | 1.1 | 532.2316 | 2 | 12.13 | 7 | F7:2931 | 29102019\_RID\_1313\_NaNaPb\_F6.raw |  |  |  |  |  |  | 1.03E5 |  |  |  | 1 | 0 | 0 | 0 | 0 | 0 | 0 | 1 | 0 | 0 | 0 | 37 | 46 | Carbamidomethylation |
| total 13 peptides |
| --- |

P08779|K1C16\_HUMAN

back to list

  

| Protein Coverage
| Supporting Peptides
|

Protein Coverage:

Supporting Peptides:

| Peptide | Uniq | -10lgP | Mass | Length | ppm | m/z | z | RT | Fraction | Scan | Source File | Area F1 | Area F10 | Area F2 | Area F3 | Area F4 | Area F5 | Area F6 | Area F7 | Area F8 | Area F9 | #Feature | #Feature F1 | #Feature F10 | #Feature F2 | #Feature F3 | #Feature F4 | #Feature F5 | #Feature F6 | #Feature F7 | #Feature F8 | #Feature F9 | Start | End | PTM |
| --- | --- | --- | --- | --- | --- | --- | --- | --- | --- | --- | --- | --- | --- | --- | --- | --- | --- | --- | --- | --- | --- | --- | --- | --- | --- | --- | --- | --- | --- | --- | --- | --- | --- | --- | --- |
| R.ALEEANADLEVK.I | N | 92.34 | 1300.6510 | 12 | 0.2 | 651.3329 | 2 | 11.91 | 1 | F1:3410 | 29102019\_RID\_1313\_NaNaPb\_F1.raw | 9.19E4 | 1.48E5 | 1.95E5 |  |  | 3.99E5 | 0 |  |  | 1.22E5 | 6 | 1 | 1 | 1 | 0 | 0 | 1 | 1 | 0 | 0 | 1 | 137 | 148 |  |
| R.APSTYGGGLSVSSR.F | Y | 86.76 | 1337.6575 | 14 | 0.1 | 669.8361 | 2 | 11.44 | 7 | F7:2310 | 29102019\_RID\_1313\_NaNaPb\_F6.raw |  | 1.82E4 |  |  |  | 0 | 1.26E5 |  |  |  | 3 | 0 | 1 | 0 | 0 | 0 | 1 | 1 | 0 | 0 | 0 | 42 | 55 |  |
| R.LLEGEDAHLSSQQASGQSYSSREVFTSSSSSSSR.Q | Y | 81.67 | 3590.6357 | 34 | 1.2 | 898.6673 | 4 | 49.93 | 5 | F5:15718 | 29102019\_RID\_1313\_NaNaPb\_F4.raw |  |  |  |  | 3.8E5 |  |  |  |  |  | 1 | 0 | 0 | 0 | 0 | 1 | 0 | 0 | 0 | 0 | 0 | 420 | 453 |  |
| K.IIAATIENAQPILQIDNAR.L | Y | 78.70 | 2063.1375 | 19 | 0.8 | 688.7203 | 3 | 22.57 | 6 | F6:8728 | 29102019\_RID\_1313\_NaNaPb\_F5.raw | 2.93E4 | 8.41E4 |  |  |  | 2.78E5 | 1.56E5 |  |  | 7.48E4 | 6 | 1 | 1 | 0 | 0 | 0 | 2 | 1 | 0 | 0 | 1 | 178 | 196 |  |
| K.ASLENSLEETK.G | N | 73.23 | 1219.5931 | 11 | 0.7 | 610.8043 | 2 | 11.70 | 3 | F3:2516 | 29102019\_RID\_1313\_NaNaPb\_F2.raw | 1.86E4 |  | 1.34E5 |  |  |  |  |  |  |  | 2 | 1 | 0 | 1 | 0 | 0 | 0 | 0 | 0 | 0 | 0 | 355 | 365 |  |
| R.VLDELTLAR.T | N | 67.89 | 1028.5865 | 9 | 0.8 | 515.3010 | 2 | 62.60 | 5 | F5:26241 | 29102019\_RID\_1313\_NaNaPb\_F4.raw | 1.32E5 |  |  |  | 8.65E5 | 5.32E5 | 1.7E5 |  |  | 1.39E5 | 5 | 1 | 0 | 0 | 0 | 1 | 1 | 1 | 0 | 0 | 1 | 226 | 234 |  |
| R.LEQEIATYR.R | N | 65.29 | 1121.5717 | 9 | 0.7 | 561.7935 | 2 | 11.51 | 6 | F6:2300 | 29102019\_RID\_1313\_NaNaPb\_F5.raw |  |  | 1.24E5 |  |  | 1.68E5 |  |  |  |  | 2 | 0 | 0 | 1 | 0 | 0 | 1 | 0 | 0 | 0 | 0 | 410 | 418 |  |
| R.DAETWFLSK.T | Y | 63.08 | 1095.5237 | 9 | 0.6 | 548.7695 | 2 | 19.72 | 7 | F7:7076 | 29102019\_RID\_1313\_NaNaPb\_F6.raw |  | 4.21E4 |  |  |  |  | 2.16E5 |  |  |  | 2 | 0 | 1 | 0 | 0 | 0 | 0 | 1 | 0 | 0 | 0 | 303 | 311 |  |
| K.GSC(+57.02)GIGGGIGGGSSR.I | N | 62.48 | 1277.5782 | 15 | 0.1 | 639.7964 | 2 | 11.34 | 3 | F3:2162 | 29102019\_RID\_1313\_NaNaPb\_F2.raw |  |  | 2.84E4 |  |  | 7.21E4 |  |  |  |  | 2 | 0 | 0 | 1 | 0 | 0 | 1 | 0 | 0 | 0 | 0 | 16 | 30 | Carbamidomethylation |
| R.NKIIAATIENAQPILQIDNAR.L | Y | 60.97 | 2305.2754 | 21 | 0.9 | 769.4331 | 3 | 17.14 | 7 | F7:5865 | 29102019\_RID\_1313\_NaNaPb\_F6.raw |  |  |  |  |  |  | 5.14E4 |  |  |  | 1 | 0 | 0 | 0 | 0 | 0 | 0 | 1 | 0 | 0 | 0 | 176 | 196 |  |
| K.VTMQNLNDRLASYLDKVR.A | N | 58.29 | 2135.1157 | 18 | 0.2 | 534.7863 | 4 | 70.04 | 5 | F5:32678 | 29102019\_RID\_1313\_NaNaPb\_F4.raw |  |  |  |  | 2.61E5 |  |  |  |  |  | 1 | 0 | 0 | 0 | 0 | 1 | 0 | 0 | 0 | 0 | 0 | 119 | 136 |  |
| R.ISSVLAGGSC(+57.02)R.A | N | 57.91 | 1105.5549 | 11 | 0.1 | 553.7848 | 2 | 11.58 | 3 | F3:2413 | 29102019\_RID\_1313\_NaNaPb\_F2.raw |  |  | 5.81E4 |  |  |  |  |  |  |  | 1 | 0 | 0 | 1 | 0 | 0 | 0 | 0 | 0 | 0 | 0 | 31 | 41 | Carbamidomethylation |
| K.EVASNSELVQSSR.S | Y | 57.28 | 1404.6844 | 13 | 1.0 | 703.3502 | 2 | 11.32 | 3 | F3:2146 | 29102019\_RID\_1313\_NaNaPb\_F2.raw |  |  | 1.75E4 |  |  |  |  |  |  |  | 1 | 0 | 0 | 1 | 0 | 0 | 0 | 0 | 0 | 0 | 0 | 318 | 330 |  |
| R.LAADDFR.T | N | 56.98 | 806.3922 | 7 | 0.4 | 404.2036 | 2 | 11.73 | 1 | F1:3258 | 29102019\_RID\_1313\_NaNaPb\_F1.raw | 1E5 | 2.78E5 | 1.15E5 |  |  | 1.11E5 | 5.12E4 |  | 2.99E4 |  | 6 | 1 | 1 | 1 | 0 | 0 | 1 | 1 | 0 | 1 | 0 | 197 | 203 |  |
| R.LASYLDK.V | N | 55.97 | 808.4330 | 7 | -0.9 | 405.2234 | 2 | 11.66 | 1 | F1:3180 | 29102019\_RID\_1313\_NaNaPb\_F1.raw | 9.27E4 |  | 3.47E5 |  |  | 0 | 5.32E4 |  |  |  | 4 | 1 | 0 | 1 | 0 | 0 | 1 | 1 | 0 | 0 | 0 | 128 | 134 |  |
| K.VTMQNLNDR.L | N | 54.91 | 1089.5237 | 9 | 0.9 | 545.7696 | 2 | 11.21 | 7 | F7:2094 | 29102019\_RID\_1313\_NaNaPb\_F6.raw |  |  | 4.29E4 |  |  |  | 6.38E4 |  |  |  | 2 | 0 | 0 | 1 | 0 | 0 | 0 | 1 | 0 | 0 | 0 | 119 | 127 |  |
| L.IGSVEEQLAQLR.C | N | 49.26 | 1341.7252 | 12 | 0.3 | 671.8701 | 2 | 16.80 | 1 | F1:5418 | 29102019\_RID\_1313\_NaNaPb\_F1.raw | 2.93E4 |  |  |  |  | 8.79E4 |  |  |  |  | 2 | 1 | 0 | 0 | 0 | 0 | 1 | 0 | 0 | 0 | 0 | 379 | 390 |  |
| total 17 peptides |
| --- |

P19859|VKTCI\_NAJNA

back to list

  

| Protein Coverage
| Supporting Peptides
|

Protein Coverage:

Supporting Peptides:

| Peptide | Uniq | -10lgP | Mass | Length | ppm | m/z | z | RT | Fraction | Scan | Source File | Area F1 | Area F10 | Area F2 | Area F3 | Area F4 | Area F5 | Area F6 | Area F7 | Area F8 | Area F9 | #Feature | #Feature F1 | #Feature F10 | #Feature F2 | #Feature F3 | #Feature F4 | #Feature F5 | #Feature F6 | #Feature F7 | #Feature F8 | #Feature F9 | Start | End | PTM |
| --- | --- | --- | --- | --- | --- | --- | --- | --- | --- | --- | --- | --- | --- | --- | --- | --- | --- | --- | --- | --- | --- | --- | --- | --- | --- | --- | --- | --- | --- | --- | --- | --- | --- | --- | --- |
| R.FC(+57.02)ELAPSAGSC(+57.02)FGFVSSYYYNR.Y | Y | 122.91 | 2581.1042 | 22 | 0.3 | 1291.5598 | 2 | 30.26 | 7 | F7:10553 | 29102019\_RID\_1313\_NaNaPb\_F6.raw |  |  |  |  |  | 1.93E5 | 1.49E7 |  |  |  | 9 | 0 | 0 | 0 | 0 | 0 | 1 | 8 | 0 | 0 | 0 | 4 | 25 | Carbamidomethylation |
| R.FC(+57.02)ELAPSAGSC(+57.02)FGF.V | Y | 80.78 | 1548.6377 | 14 | 0.5 | 775.3265 | 2 | 35.63 | 7 | F7:12452 | 29102019\_RID\_1313\_NaNaPb\_F6.raw |  |  |  |  |  |  | 4.08E7 |  |  |  | 10 | 0 | 0 | 0 | 0 | 0 | 0 | 10 | 0 | 0 | 0 | 4 | 17 | Carbamidomethylation |
| R.FC(+57.02)ELAPSAGSC(+57.02)FGFVSSY.Y | Y | 75.36 | 1984.8335 | 18 | 1.2 | 993.4252 | 2 | 39.10 | 7 | F7:13370 | 29102019\_RID\_1313\_NaNaPb\_F6.raw |  |  |  |  |  |  | 2.15E7 |  |  |  | 3 | 0 | 0 | 0 | 0 | 0 | 0 | 3 | 0 | 0 | 0 | 4 | 21 | Carbamidomethylation |
| R.FC(+57.02)ELAPSAGSC(+57.02)FGFVSSYY.Y | Y | 69.05 | 2147.8967 | 19 | 0.8 | 1074.9565 | 2 | 44.42 | 7 | F7:14828 | 29102019\_RID\_1313\_NaNaPb\_F6.raw |  |  |  |  |  |  | 8.5E6 |  |  |  | 1 | 0 | 0 | 0 | 0 | 0 | 0 | 1 | 0 | 0 | 0 | 4 | 22 | Carbamidomethylation |
| R.FC(+57.02)ELAPSAGSC(+57.02).F | N | 65.85 | 1197.4794 | 11 | -1.4 | 599.7461 | 2 | 12.40 | 7 | F7:3139 | 29102019\_RID\_1313\_NaNaPb\_F6.raw |  |  |  |  |  |  | 7.18E5 |  |  |  | 1 | 0 | 0 | 0 | 0 | 0 | 0 | 1 | 0 | 0 | 0 | 4 | 14 | Carbamidomethylation |
| R.FC(+57.02)ELAPSAGSC(+57.02)F.G | N | 62.04 | 1344.5479 | 12 | 0.9 | 673.2818 | 2 | 19.38 | 7 | F7:7085 | 29102019\_RID\_1313\_NaNaPb\_F6.raw |  |  |  |  |  |  | 7.67E6 |  |  |  | 1 | 0 | 0 | 0 | 0 | 0 | 0 | 1 | 0 | 0 | 0 | 4 | 15 | Carbamidomethylation |
| F.GFVSSYYYNR.Y | Y | 61.41 | 1254.5669 | 10 | 0.9 | 628.2913 | 2 | 11.87 | 7 | F7:2719 | 29102019\_RID\_1313\_NaNaPb\_F6.raw |  |  |  |  |  |  | 7.98E5 |  |  |  | 1 | 0 | 0 | 0 | 0 | 0 | 0 | 1 | 0 | 0 | 0 | 16 | 25 |  |
| R.YSNTC(+57.02)HSFTYSGC(+57.02).G | Y | 58.78 | 1582.5817 | 13 | 0.8 | 792.2988 | 2 | 11.45 | 7 | F7:2308 | 29102019\_RID\_1313\_NaNaPb\_F6.raw |  |  |  |  |  |  | 1.9E5 |  |  |  | 1 | 0 | 0 | 0 | 0 | 0 | 0 | 1 | 0 | 0 | 0 | 26 | 38 | Carbamidomethylation |
| R.FC(+57.02)ELAPSAGSC(+57.02)FGFVS.S | Y | 51.14 | 1734.7382 | 16 | 1.3 | 868.3774 | 2 | 34.08 | 7 | F7:11898 | 29102019\_RID\_1313\_NaNaPb\_F6.raw |  |  |  |  |  |  | 1.77E6 |  |  |  | 2 | 0 | 0 | 0 | 0 | 0 | 0 | 2 | 0 | 0 | 0 | 4 | 19 | Carbamidomethylation |
| C.FGFVSSYYYNR.Y | Y | 47.00 | 1401.6353 | 11 | 0.6 | 701.8253 | 2 | 15.78 | 6 | F6:5307 | 29102019\_RID\_1313\_NaNaPb\_F5.raw |  |  |  |  |  | 1.72E4 | 9.6E5 |  |  |  | 2 | 0 | 0 | 0 | 0 | 0 | 1 | 1 | 0 | 0 | 0 | 15 | 25 |  |
| R.FC(+57.02)ELAPSAGSC(+57.02)FGFV.S | Y | 45.76 | 1647.7061 | 15 | 0.6 | 824.8608 | 2 | 39.41 | 7 | F7:13399 | 29102019\_RID\_1313\_NaNaPb\_F6.raw |  |  |  |  |  |  | 1.93E5 |  |  |  | 1 | 0 | 0 | 0 | 0 | 0 | 0 | 1 | 0 | 0 | 0 | 4 | 18 | Carbamidomethylation |
| R.YSNTC(+57.02)HSF.T | Y | 45.10 | 1014.3865 | 8 | -2.5 | 508.1993 | 2 | 11.05 | 7 | F7:1940 | 29102019\_RID\_1313\_NaNaPb\_F6.raw |  |  |  |  |  |  | 1.99E6 |  |  |  | 1 | 0 | 0 | 0 | 0 | 0 | 0 | 1 | 0 | 0 | 0 | 26 | 33 | Carbamidomethylation |
| S.AGSC(+57.02)FGFVSSYYYNR.Y | Y | 44.20 | 1776.7566 | 15 | 0.8 | 889.3863 | 2 | 17.55 | 7 | F7:6022 | 29102019\_RID\_1313\_NaNaPb\_F6.raw |  |  |  |  |  |  | 4.34E5 |  |  |  | 1 | 0 | 0 | 0 | 0 | 0 | 0 | 1 | 0 | 0 | 0 | 11 | 25 | Carbamidomethylation |
| total 13 peptides |
| --- |

Q5ZPJ7|VKT\_NAJAT

back to list

  

| Protein Coverage
| Supporting Peptides
|

Protein Coverage:

Supporting Peptides:

| Peptide | Uniq | -10lgP | Mass | Length | ppm | m/z | z | RT | Fraction | Scan | Source File | Area F1 | Area F10 | Area F2 | Area F3 | Area F4 | Area F5 | Area F6 | Area F7 | Area F8 | Area F9 | #Feature | #Feature F1 | #Feature F10 | #Feature F2 | #Feature F3 | #Feature F4 | #Feature F5 | #Feature F6 | #Feature F7 | #Feature F8 | #Feature F9 | Start | End | PTM |
| --- | --- | --- | --- | --- | --- | --- | --- | --- | --- | --- | --- | --- | --- | --- | --- | --- | --- | --- | --- | --- | --- | --- | --- | --- | --- | --- | --- | --- | --- | --- | --- | --- | --- | --- | --- |
| Q.YSNTC(+57.02)HSFTYSGC(+57.02)GGNANR.F | Y | 100.89 | 2151.8486 | 19 | -2.2 | 1076.9292 | 2 | 11.07 | 7 | F7:1942 | 29102019\_RID\_1313\_NaNaPb\_F6.raw |  |  |  |  |  |  | 1.2E7 |  |  |  | 2 | 0 | 0 | 0 | 0 | 0 | 0 | 2 | 0 | 0 | 0 | 50 | 68 | Carbamidomethylation |
| H.SFTYSGC(+57.02)GGNANR.F | Y | 88.33 | 1389.5731 | 13 | 0.5 | 695.7942 | 2 | 11.12 | 7 | F7:2007 | 29102019\_RID\_1313\_NaNaPb\_F6.raw |  |  |  |  |  |  | 2.16E6 |  |  |  | 1 | 0 | 0 | 0 | 0 | 0 | 0 | 1 | 0 | 0 | 0 | 56 | 68 | Carbamidomethylation |
| Y.SNTC(+57.02)HSFTYSGC(+57.02)GGNANR.F | Y | 85.71 | 1988.7854 | 18 | 0.3 | 663.9360 | 3 | 10.94 | 7 | F7:1800 | 29102019\_RID\_1313\_NaNaPb\_F6.raw |  |  |  |  |  |  | 2.52E5 |  |  |  | 1 | 0 | 0 | 0 | 0 | 0 | 0 | 1 | 0 | 0 | 0 | 51 | 68 | Carbamidomethylation |
| R.TIDEC(+57.02)NRTC(+57.02)VG | N | 79.87 | 1323.5547 | 11 | 0.8 | 662.7852 | 2 | 11.06 | 4 | F4:1956 | 29102019\_RID\_1313\_NaNaPb\_F3.raw |  |  |  | 1.14E6 |  |  | 4.26E5 |  |  |  | 2 | 0 | 0 | 0 | 1 | 0 | 0 | 1 | 0 | 0 | 0 | 71 | 81 | Carbamidomethylation |
| C.HSFTYSGC(+57.02)GGNANR.F | Y | 79.68 | 1526.6321 | 14 | 0.1 | 764.3234 | 2 | 10.79 | 7 | F7:1675 | 29102019\_RID\_1313\_NaNaPb\_F6.raw |  |  |  |  |  |  | 6.94E5 |  |  |  | 2 | 0 | 0 | 0 | 0 | 0 | 0 | 2 | 0 | 0 | 0 | 55 | 68 | Carbamidomethylation |
| N.TC(+57.02)HSFTYSGC(+57.02)GGNANR.F | Y | 79.61 | 1787.7104 | 16 | 0.4 | 596.9110 | 3 | 10.89 | 7 | F7:1773 | 29102019\_RID\_1313\_NaNaPb\_F6.raw |  |  |  |  |  |  | 1.19E6 |  |  |  | 2 | 0 | 0 | 0 | 0 | 0 | 0 | 2 | 0 | 0 | 0 | 53 | 68 | Carbamidomethylation |
| Y.YNQYSNTC(+57.02)HSFTYSGC(+57.02)GGNANR.F | Y | 70.23 | 2557.0134 | 22 | 0.3 | 853.3453 | 3 | 11.25 | 7 | F7:2117 | 29102019\_RID\_1313\_NaNaPb\_F6.raw |  |  |  |  |  |  | 5.72E4 |  |  |  | 1 | 0 | 0 | 0 | 0 | 0 | 0 | 1 | 0 | 0 | 0 | 47 | 68 | Carbamidomethylation |
| R.FC(+57.02)ELAPSAGSC(+57.02).F | N | 65.85 | 1197.4794 | 11 | -1.4 | 599.7461 | 2 | 12.40 | 7 | F7:3139 | 29102019\_RID\_1313\_NaNaPb\_F6.raw |  |  |  |  |  |  | 7.18E5 |  |  |  | 1 | 0 | 0 | 0 | 0 | 0 | 0 | 1 | 0 | 0 | 0 | 28 | 38 | Carbamidomethylation |
| R.FC(+57.02)ELAPSAGSC(+57.02)F.A | N | 62.04 | 1344.5479 | 12 | 0.9 | 673.2818 | 2 | 19.38 | 7 | F7:7085 | 29102019\_RID\_1313\_NaNaPb\_F6.raw |  |  |  |  |  |  | 7.67E6 |  |  |  | 1 | 0 | 0 | 0 | 0 | 0 | 0 | 1 | 0 | 0 | 0 | 28 | 39 | Carbamidomethylation |
| R.FC(+57.02)ELAPSAGSC(+57.02)FA.F | Y | 55.19 | 1415.5850 | 13 | 0.4 | 708.8000 | 2 | 18.63 | 7 | F7:6512 | 29102019\_RID\_1313\_NaNaPb\_F6.raw |  |  |  |  |  |  | 7.86E4 |  |  |  | 1 | 0 | 0 | 0 | 0 | 0 | 0 | 1 | 0 | 0 | 0 | 28 | 40 | Carbamidomethylation |
| R.FC(+57.02)ELAPSAGSC(+57.02)FAFVPS.Y | Y | 48.52 | 1845.8065 | 17 | 1.0 | 923.9115 | 2 | 37.85 | 7 | F7:12883 | 29102019\_RID\_1313\_NaNaPb\_F6.raw |  |  |  |  |  |  | 1.07E6 |  |  |  | 1 | 0 | 0 | 0 | 0 | 0 | 0 | 1 | 0 | 0 | 0 | 28 | 44 | Carbamidomethylation |
| N.QYSNTC(+57.02)HSFTYSGC(+57.02)GGNANR.F | Y | 42.69 | 2279.9072 | 20 | 0.8 | 760.9769 | 3 | 11.10 | 7 | F7:1986 | 29102019\_RID\_1313\_NaNaPb\_F6.raw |  |  |  |  |  |  | 6.33E4 |  |  |  | 1 | 0 | 0 | 0 | 0 | 0 | 0 | 1 | 0 | 0 | 0 | 49 | 68 | Carbamidomethylation |
| total 12 peptides |
| --- |

P20229|VKTTI\_NAJNA

back to list

  

| Protein Coverage
| Supporting Peptides
|

Protein Coverage:

Supporting Peptides:

| Peptide | Uniq | -10lgP | Mass | Length | ppm | m/z | z | RT | Fraction | Scan | Source File | Area F1 | Area F10 | Area F2 | Area F3 | Area F4 | Area F5 | Area F6 | Area F7 | Area F8 | Area F9 | #Feature | #Feature F1 | #Feature F10 | #Feature F2 | #Feature F3 | #Feature F4 | #Feature F5 | #Feature F6 | #Feature F7 | #Feature F8 | #Feature F9 | Start | End | PTM |
| --- | --- | --- | --- | --- | --- | --- | --- | --- | --- | --- | --- | --- | --- | --- | --- | --- | --- | --- | --- | --- | --- | --- | --- | --- | --- | --- | --- | --- | --- | --- | --- | --- | --- | --- | --- |
| K.AHKPAFYYNK.D | Y | 98.80 | 1237.6244 | 10 | 0.1 | 413.5488 | 3 | 10.92 | 4 | F4:1831 | 29102019\_RID\_1313\_NaNaPb\_F3.raw |  |  |  | 5.53E6 |  |  |  |  |  |  | 2 | 0 | 0 | 0 | 2 | 0 | 0 | 0 | 0 | 0 | 0 | 16 | 25 |  |
| K.FIYGGC(+57.02)GGNANR.F | N | 88.02 | 1284.5669 | 12 | -1.3 | 643.2899 | 2 | 11.48 | 4 | F4:2328 | 29102019\_RID\_1313\_NaNaPb\_F3.raw |  |  |  | 1.63E8 |  |  | 7.26E4 | 2.28E4 |  |  | 25 | 0 | 0 | 0 | 23 | 0 | 0 | 1 | 1 | 0 | 0 | 33 | 44 | Carbamidomethylation |
| R.TIDEC(+57.02)NRTC(+57.02)VG | N | 79.87 | 1323.5547 | 11 | 0.8 | 662.7852 | 2 | 11.06 | 4 | F4:1956 | 29102019\_RID\_1313\_NaNaPb\_F3.raw |  |  |  | 1.14E6 |  |  | 4.26E5 |  |  |  | 2 | 0 | 0 | 0 | 1 | 0 | 0 | 1 | 0 | 0 | 0 | 47 | 57 | Carbamidomethylation |
| RPGFC(+57.02)ELPAAK.G | Y | 78.63 | 1244.6335 | 11 | -0.2 | 415.8850 | 3 | 13.49 | 4 | F4:3701 | 29102019\_RID\_1313\_NaNaPb\_F3.raw |  |  |  | 2.52E8 | 2.37E6 |  |  |  |  |  | 28 | 0 | 0 | 0 | 26 | 2 | 0 | 0 | 0 | 0 | 0 | 1 | 11 | Carbamidomethylation |
| P.GFC(+57.02)ELPAAK.G | Y | 72.20 | 991.4797 | 9 | -0.6 | 496.7468 | 2 | 11.46 | 4 | F4:2313 | 29102019\_RID\_1313\_NaNaPb\_F3.raw |  |  |  | 3.67E6 |  |  |  |  |  |  | 1 | 0 | 0 | 0 | 1 | 0 | 0 | 0 | 0 | 0 | 0 | 3 | 11 | Carbamidomethylation |
| RPGFC(+57.02)ELPAAKGLC(+57.02)K.A | Y | 61.44 | 1702.8646 | 15 | -0.5 | 568.6285 | 3 | 11.59 | 4 | F4:2436 | 29102019\_RID\_1313\_NaNaPb\_F3.raw |  |  |  | 1.54E5 |  |  |  |  |  |  | 1 | 0 | 0 | 0 | 1 | 0 | 0 | 0 | 0 | 0 | 0 | 1 | 15 | Carbamidomethylation |
| K.FIYGGC(+57.02)GGNAN.R | Y | 58.61 | 1128.4658 | 11 | -0.6 | 565.2399 | 2 | 12.12 | 4 | F4:2916 | 29102019\_RID\_1313\_NaNaPb\_F3.raw |  |  |  | 5.54E5 |  |  |  |  |  |  | 1 | 0 | 0 | 0 | 1 | 0 | 0 | 0 | 0 | 0 | 0 | 33 | 43 | Carbamidomethylation |
| K.AHKPAFYYNKDSHR.C | Y | 58.22 | 1732.8434 | 14 | -0.3 | 434.2180 | 4 | 10.74 | 4 | F4:1689 | 29102019\_RID\_1313\_NaNaPb\_F3.raw |  |  |  | 1.29E5 |  |  |  |  |  |  | 1 | 0 | 0 | 0 | 1 | 0 | 0 | 0 | 0 | 0 | 0 | 16 | 29 |  |
| K.AHKPAFYY.N | Y | 57.99 | 995.4865 | 8 | -0.6 | 498.7502 | 2 | 11.50 | 4 | F4:2372 | 29102019\_RID\_1313\_NaNaPb\_F3.raw |  |  |  | 5.22E6 |  |  |  |  |  |  | 1 | 0 | 0 | 0 | 1 | 0 | 0 | 0 | 0 | 0 | 0 | 16 | 23 |  |
| R.C(+57.02)QKFIYGGC(+57.02)GGNANR.F | Y | 56.22 | 1700.7511 | 15 | 1.3 | 567.9250 | 3 | 11.21 | 4 | F4:2109 | 29102019\_RID\_1313\_NaNaPb\_F3.raw |  |  |  | 4.71E4 |  |  |  |  |  |  | 1 | 0 | 0 | 0 | 1 | 0 | 0 | 0 | 0 | 0 | 0 | 30 | 44 | Carbamidomethylation |
| K.FIYGGC(+57.02)GGNANRFR.T | Y | 55.82 | 1587.7365 | 14 | 0.4 | 530.2530 | 3 | 11.57 | 4 | F4:2423 | 29102019\_RID\_1313\_NaNaPb\_F3.raw |  |  |  | 4.65E5 |  |  |  |  |  |  | 1 | 0 | 0 | 0 | 1 | 0 | 0 | 0 | 0 | 0 | 0 | 33 | 46 | Carbamidomethylation |
| K.PAFYYNK.D | Y | 52.99 | 901.4333 | 7 | -0.2 | 451.7239 | 2 | 11.59 | 4 | F4:2421 | 29102019\_RID\_1313\_NaNaPb\_F3.raw |  |  |  | 1.37E7 |  |  |  |  |  |  | 2 | 0 | 0 | 0 | 2 | 0 | 0 | 0 | 0 | 0 | 0 | 19 | 25 |  |
| F.C(+57.02)ELPAAK.G | Y | 49.24 | 787.3898 | 7 | -0.5 | 394.7020 | 2 | 11.46 | 4 | F4:2314 | 29102019\_RID\_1313\_NaNaPb\_F3.raw |  |  |  | 5.27E6 |  |  |  |  |  |  | 1 | 0 | 0 | 0 | 1 | 0 | 0 | 0 | 0 | 0 | 0 | 5 | 11 | Carbamidomethylation |
| RPGFC(+57.02)ELPAAKG.L | Y | 47.91 | 1301.6550 | 12 | -0.9 | 434.8919 | 3 | 11.49 | 4 | F4:2300 | 29102019\_RID\_1313\_NaNaPb\_F3.raw |  |  |  | 5.86E7 |  |  |  |  |  |  | 1 | 0 | 0 | 0 | 1 | 0 | 0 | 0 | 0 | 0 | 0 | 1 | 12 | Carbamidomethylation |
| R.PGFC(+57.02)ELPAAK.G | Y | 46.70 | 1088.5325 | 10 | 0.0 | 545.2735 | 2 | 11.50 | 4 | F4:2379 | 29102019\_RID\_1313\_NaNaPb\_F3.raw |  |  |  | 1.78E4 |  |  |  |  |  |  | 1 | 0 | 0 | 0 | 1 | 0 | 0 | 0 | 0 | 0 | 0 | 2 | 11 | Carbamidomethylation |
| K.FIYGGC(+57.02)GGN.A | N | 42.14 | 943.3858 | 9 | -0.1 | 472.7001 | 2 | 12.31 | 4 | F4:3073 | 29102019\_RID\_1313\_NaNaPb\_F3.raw |  |  |  | 5.1E5 |  |  |  |  |  |  | 1 | 0 | 0 | 0 | 1 | 0 | 0 | 0 | 0 | 0 | 0 | 33 | 41 | Carbamidomethylation |
| total 16 peptides |
| --- |

P01441|3SA2\_NAJOX

back to list

  

| Protein Coverage
| Supporting Peptides
|

Protein Coverage:

Supporting Peptides:

| Peptide | Uniq | -10lgP | Mass | Length | ppm | m/z | z | RT | Fraction | Scan | Source File | Area F1 | Area F10 | Area F2 | Area F3 | Area F4 | Area F5 | Area F6 | Area F7 | Area F8 | Area F9 | #Feature | #Feature F1 | #Feature F10 | #Feature F2 | #Feature F3 | #Feature F4 | #Feature F5 | #Feature F6 | #Feature F7 | #Feature F8 | #Feature F9 | Start | End | PTM |
| --- | --- | --- | --- | --- | --- | --- | --- | --- | --- | --- | --- | --- | --- | --- | --- | --- | --- | --- | --- | --- | --- | --- | --- | --- | --- | --- | --- | --- | --- | --- | --- | --- | --- | --- | --- |
| K.SSLLVKYVC(+57.02)C(+57.02)NTDKC(+57.02)N | Y | 100.90 | 1959.8853 | 16 | 0.5 | 980.9504 | 2 | 11.68 | 10 | F10:2502 | 29102019\_RID\_1313\_NaNaPb\_F9.raw |  |  |  |  |  |  |  |  | 1.8E5 | 2.61E6 | 3 | 0 | 0 | 0 | 0 | 0 | 0 | 0 | 0 | 1 | 2 | 45 | 60 | Carbamidomethylation |
| K.MFMVAAPHVPVK.R | N | 94.98 | 1325.6989 | 12 | 0.3 | 663.8569 | 2 | 12.10 | 10 | F10:2858 | 29102019\_RID\_1313\_NaNaPb\_F9.raw |  | 5.94E4 |  |  |  |  |  |  | 6.96E5 | 2.02E7 | 5 | 0 | 1 | 0 | 0 | 0 | 0 | 0 | 0 | 2 | 2 | 24 | 35 |  |
| K.M(+15.99)FMVAAPHVPVK.R | N | 93.79 | 1341.6937 | 12 | -0.5 | 671.8538 | 2 | 11.77 | 10 | F10:2565 | 29102019\_RID\_1313\_NaNaPb\_F9.raw |  |  |  |  |  |  |  |  | 8.19E5 | 9.87E6 | 3 | 0 | 0 | 0 | 0 | 0 | 0 | 0 | 0 | 1 | 2 | 24 | 35 | Oxidation (M) |
| K.M(+15.99)FM(+15.99)VAAPHVPVK.R | N | 82.05 | 1357.6886 | 12 | 0.0 | 453.5701 | 3 | 11.59 | 9 | F9:2418 | 29102019\_RID\_1313\_NaNaPb\_F8.raw |  | 5.42E4 |  |  |  | 2.74E5 | 2.23E5 |  | 5.73E5 | 7.87E6 | 6 | 0 | 1 | 0 | 0 | 0 | 1 | 1 | 0 | 1 | 2 | 24 | 35 | Oxidation (M) |
| M.FM(+15.99)VAAPHVPVK.R | N | 67.72 | 1210.6532 | 11 | 0.4 | 404.5585 | 3 | 11.55 | 10 | F10:2357 | 29102019\_RID\_1313\_NaNaPb\_F9.raw |  |  |  |  |  |  |  |  |  | 4.18E5 | 1 | 0 | 0 | 0 | 0 | 0 | 0 | 0 | 0 | 0 | 1 | 25 | 35 | Oxidation (M) |
| M.FMVAAPHVPVK.R | N | 67.02 | 1194.6583 | 11 | 0.0 | 598.3364 | 2 | 11.70 | 10 | F10:2537 | 29102019\_RID\_1313\_NaNaPb\_F9.raw |  |  |  |  |  |  |  |  |  | 1.88E6 | 2 | 0 | 0 | 0 | 0 | 0 | 0 | 0 | 0 | 0 | 2 | 25 | 35 |  |
| R.GC(+57.02)IDVC(+57.02)PK.S | N | 63.51 | 947.4205 | 8 | -0.6 | 474.7172 | 2 | 11.17 | 7 | F7:2031 | 29102019\_RID\_1313\_NaNaPb\_F6.raw |  | 2.85E7 |  |  |  | 7.32E7 | 1.17E6 |  | 5.42E6 | 9.79E6 | 10 | 0 | 2 | 0 | 0 | 0 | 5 | 1 | 0 | 1 | 1 | 37 | 44 | Carbamidomethylation |
| K.MFMVAAPHVPVKR.G | N | 63.11 | 1481.7999 | 13 | -0.5 | 741.9069 | 2 | 11.60 | 10 | F10:2431 | 29102019\_RID\_1313\_NaNaPb\_F9.raw |  |  |  |  |  |  |  |  |  | 2.66E6 | 2 | 0 | 0 | 0 | 0 | 0 | 0 | 0 | 0 | 0 | 2 | 24 | 36 |  |
| K.M(+15.99)FMVAAPHVPVKR.G | N | 63.11 | 1497.7948 | 13 | 0.7 | 500.2726 | 3 | 11.42 | 10 | F10:2253 | 29102019\_RID\_1313\_NaNaPb\_F9.raw |  |  |  |  |  |  |  |  |  | 6.36E5 | 1 | 0 | 0 | 0 | 0 | 0 | 0 | 0 | 0 | 0 | 1 | 24 | 36 | Oxidation (M) |
| K.MFM(+15.99)VAAPHVPVK.R | N | 62.53 | 1341.6937 | 12 | -0.2 | 448.2384 | 3 | 11.93 | 6 | F6:2704 | 29102019\_RID\_1313\_NaNaPb\_F5.raw |  |  |  |  |  | 8.27E4 |  |  |  |  | 1 | 0 | 0 | 0 | 0 | 0 | 1 | 0 | 0 | 0 | 0 | 24 | 35 | Oxidation (M) |
| R.GC(+57.02)IDVC(+57.02)PKSSLLVK.Y | N | 59.21 | 1574.8160 | 14 | -0.2 | 525.9459 | 3 | 11.68 | 10 | F10:2508 | 29102019\_RID\_1313\_NaNaPb\_F9.raw |  | 5.2E5 |  |  |  |  |  |  | 0 | 9.79E5 | 3 | 0 | 1 | 0 | 0 | 0 | 0 | 0 | 0 | 1 | 1 | 37 | 50 | Carbamidomethylation |
| F.MVAAPHVPVK.R | N | 58.68 | 1047.5898 | 10 | -0.1 | 524.8021 | 2 | 11.17 | 10 | F10:2028 | 29102019\_RID\_1313\_NaNaPb\_F9.raw |  |  |  |  |  |  |  |  |  | 9.71E5 | 2 | 0 | 0 | 0 | 0 | 0 | 0 | 0 | 0 | 0 | 2 | 26 | 35 |  |
| K.M(+15.99)FM(+15.99)VAAPHVPVKR.G | N | 57.13 | 1513.7898 | 13 | 1.0 | 505.6044 | 3 | 11.33 | 10 | F10:2162 | 29102019\_RID\_1313\_NaNaPb\_F9.raw |  |  |  |  |  |  |  |  |  | 2.04E5 | 1 | 0 | 0 | 0 | 0 | 0 | 0 | 0 | 0 | 0 | 1 | 24 | 36 | Oxidation (M) |
| V.AAPHVPVK.R | N | 55.51 | 817.4810 | 8 | 0.2 | 409.7478 | 2 | 11.02 | 9 | F9:1849 | 29102019\_RID\_1313\_NaNaPb\_F8.raw |  |  |  |  |  |  |  |  | 4.92E5 | 8.64E6 | 3 | 0 | 0 | 0 | 0 | 0 | 0 | 0 | 0 | 1 | 2 | 28 | 35 |  |
| K.LVPLFSKTC(+57.02)PAGK.N | N | 54.45 | 1416.7799 | 13 | -1.1 | 473.2667 | 3 | 11.66 | 10 | F10:2505 | 29102019\_RID\_1313\_NaNaPb\_F9.raw |  |  |  |  |  |  |  |  |  | 8.63E5 | 1 | 0 | 0 | 0 | 0 | 0 | 0 | 0 | 0 | 0 | 1 | 6 | 18 | Carbamidomethylation |
| K.TC(+57.02)PAGKNLC(+57.02)YK.M | N | 54.37 | 1310.6111 | 11 | -0.4 | 437.8775 | 3 | 10.78 | 6 | F6:1669 | 29102019\_RID\_1313\_NaNaPb\_F5.raw |  |  |  |  |  | 3.23E4 |  |  |  |  | 1 | 0 | 0 | 0 | 0 | 0 | 1 | 0 | 0 | 0 | 0 | 13 | 23 | Carbamidomethylation |
| M.VAAPHVPVK.R | N | 53.58 | 916.5494 | 9 | -0.2 | 459.2819 | 2 | 11.00 | 10 | F10:1802 | 29102019\_RID\_1313\_NaNaPb\_F9.raw |  |  |  |  |  |  |  |  |  | 1.23E6 | 1 | 0 | 0 | 0 | 0 | 0 | 0 | 0 | 0 | 0 | 1 | 27 | 35 |  |
| K.SSLLVKYVC(+57.02).C | N | 49.20 | 1067.5685 | 9 | 0.8 | 534.7919 | 2 | 12.39 | 2 | F2:3276 | 29102019\_RID\_1313\_NaNaPb\_F10.raw |  | 2.19E5 |  |  |  |  |  |  |  | 2.98E5 | 2 | 0 | 1 | 0 | 0 | 0 | 0 | 0 | 0 | 0 | 1 | 45 | 53 | Carbamidomethylation |
| K.KLVPLFSK.T | N | 49.10 | 930.5902 | 8 | -0.6 | 466.3021 | 2 | 52.91 | 5 | F5:18153 | 29102019\_RID\_1313\_NaNaPb\_F4.raw |  | 1.72E6 |  |  | 6.86E6 | 2.56E6 |  |  | 4.28E6 | 0 | 6 | 0 | 1 | 0 | 0 | 2 | 1 | 0 | 0 | 1 | 1 | 5 | 12 |  |
| K.RGC(+57.02)IDVC(+57.02)PK.S | N | 48.01 | 1103.5216 | 9 | 0.2 | 552.7682 | 2 | 10.91 | 6 | F6:1732 | 29102019\_RID\_1313\_NaNaPb\_F5.raw |  |  |  |  |  | 6.74E5 |  |  |  |  | 1 | 0 | 0 | 0 | 0 | 0 | 1 | 0 | 0 | 0 | 0 | 36 | 44 | Carbamidomethylation |
| K.M(+15.99)FMVAAPHVPVKRG.C | N | 46.57 | 1554.8163 | 14 | 0.3 | 519.2795 | 3 | 11.42 | 10 | F10:2260 | 29102019\_RID\_1313\_NaNaPb\_F9.raw |  |  |  |  |  |  |  |  |  | 3.39E5 | 1 | 0 | 0 | 0 | 0 | 0 | 0 | 0 | 0 | 0 | 1 | 24 | 37 | Oxidation (M) |
| L.FSKTC(+57.02)PAGKNLC(+57.02)YK.M | N | 45.03 | 1672.8065 | 14 | -1.1 | 419.2084 | 4 | 10.67 | 9 | F9:1602 | 29102019\_RID\_1313\_NaNaPb\_F8.raw |  |  |  |  |  |  |  |  | 6.06E5 |  | 1 | 0 | 0 | 0 | 0 | 0 | 0 | 0 | 0 | 1 | 0 | 10 | 23 | Carbamidomethylation |
| K.MFMVAAPHVPVKRG.C | N | 44.82 | 1538.8214 | 14 | -1.2 | 385.7122 | 4 | 11.58 | 10 | F10:2405 | 29102019\_RID\_1313\_NaNaPb\_F9.raw |  |  |  |  |  |  |  |  |  | 1.44E5 | 1 | 0 | 0 | 0 | 0 | 0 | 0 | 0 | 0 | 0 | 1 | 24 | 37 |  |
| total 23 peptides |
| --- |

O76009|KT33A\_HUMAN

back to list

  

| Protein Coverage
| Supporting Peptides
|

Protein Coverage:

Supporting Peptides:

| Peptide | Uniq | -10lgP | Mass | Length | ppm | m/z | z | RT | Fraction | Scan | Source File | Area F1 | Area F10 | Area F2 | Area F3 | Area F4 | Area F5 | Area F6 | Area F7 | Area F8 | Area F9 | #Feature | #Feature F1 | #Feature F10 | #Feature F2 | #Feature F3 | #Feature F4 | #Feature F5 | #Feature F6 | #Feature F7 | #Feature F8 | #Feature F9 | Start | End | PTM |
| --- | --- | --- | --- | --- | --- | --- | --- | --- | --- | --- | --- | --- | --- | --- | --- | --- | --- | --- | --- | --- | --- | --- | --- | --- | --- | --- | --- | --- | --- | --- | --- | --- | --- | --- | --- |
| R.TVNALEIELQAQHNLRDSLENTLTESEAR.Y | N | 88.00 | 3293.6487 | 29 | 3.1 | 824.4220 | 4 | 79.92 | 5 | F5:40829 | 29102019\_RID\_1313\_NaNaPb\_F4.raw |  |  |  |  | 1.15E8 |  |  |  |  |  | 2 | 0 | 0 | 0 | 0 | 2 | 0 | 0 | 0 | 0 | 0 | 278 | 306 |  |
| R.TVNALEIELQAQHNLR.D | N | 85.94 | 1847.9854 | 16 | 1.5 | 617.0033 | 3 | 64.36 | 5 | F5:27680 | 29102019\_RID\_1313\_NaNaPb\_F4.raw |  |  |  |  | 7.74E7 |  |  |  |  |  | 2 | 0 | 0 | 0 | 0 | 2 | 0 | 0 | 0 | 0 | 0 | 278 | 293 |  |
| R.RTVNALEIELQAQHNLR.D | N | 85.38 | 2004.0864 | 17 | 1.7 | 669.0372 | 3 | 56.81 | 5 | F5:21335 | 29102019\_RID\_1313\_NaNaPb\_F4.raw |  |  |  |  | 8.49E6 |  |  |  |  |  | 1 | 0 | 0 | 0 | 0 | 1 | 0 | 0 | 0 | 0 | 0 | 277 | 293 |  |
| R.SDLERQNQEYQVLLDVR.A | N | 79.55 | 2104.0549 | 17 | 1.2 | 702.3597 | 3 | 65.80 | 5 | F5:28997 | 29102019\_RID\_1313\_NaNaPb\_F4.raw |  |  |  |  | 2.33E6 |  |  |  |  |  | 1 | 0 | 0 | 0 | 0 | 1 | 0 | 0 | 0 | 0 | 0 | 330 | 346 |  |
| R.QNQEYQVLLDVR.A | N | 78.64 | 1503.7681 | 12 | 0.6 | 752.8917 | 2 | 68.55 | 5 | F5:31362 | 29102019\_RID\_1313\_NaNaPb\_F4.raw |  |  |  |  | 4.7E6 |  |  |  |  |  | 1 | 0 | 0 | 0 | 0 | 1 | 0 | 0 | 0 | 0 | 0 | 335 | 346 |  |
| R.EVEQWFATQTEELNKQVVSSSEQLQSYQAEIIELRR.T | N | 64.74 | 4295.1348 | 36 | 0.6 | 1074.7916 | 4 | 88.00 | 5 | F5:47718 | 29102019\_RID\_1313\_NaNaPb\_F4.raw |  |  |  |  | 7.49E5 |  |  |  |  |  | 1 | 0 | 0 | 0 | 0 | 1 | 0 | 0 | 0 | 0 | 0 | 242 | 277 |  |
| K.QVVSSSEQLQSYQAEIIELRR.T | N | 63.37 | 2462.2764 | 21 | 1.2 | 821.7671 | 3 | 67.06 | 5 | F5:30085 | 29102019\_RID\_1313\_NaNaPb\_F4.raw |  |  |  |  | 4.56E6 |  |  |  |  |  | 1 | 0 | 0 | 0 | 0 | 1 | 0 | 0 | 0 | 0 | 0 | 257 | 277 |  |
| R.QLERDNAELENLIRER.S | N | 61.65 | 1997.0289 | 16 | 0.7 | 666.6841 | 3 | 57.99 | 5 | F5:22273 | 29102019\_RID\_1313\_NaNaPb\_F4.raw |  |  |  |  | 1.02E8 |  |  |  |  |  | 1 | 0 | 0 | 0 | 0 | 1 | 0 | 0 | 0 | 0 | 0 | 76 | 91 |  |
| R.TKYETELSLRQLVESDINGLRR.I | Y | 60.76 | 2619.3979 | 22 | 7.3 | 524.8907 | 5 | 68.08 | 5 | F5:31021 | 29102019\_RID\_1313\_NaNaPb\_F4.raw |  |  |  |  | 2.8E5 |  |  |  |  |  | 1 | 0 | 0 | 0 | 0 | 1 | 0 | 0 | 0 | 0 | 0 | 143 | 164 |  |
| R.QLVESDINGLRR.I | N | 59.73 | 1398.7579 | 12 | 0.2 | 700.3864 | 2 | 48.02 | 5 | F5:14116 | 29102019\_RID\_1313\_NaNaPb\_F4.raw |  |  |  |  | 3.26E7 |  |  |  |  |  | 2 | 0 | 0 | 0 | 0 | 2 | 0 | 0 | 0 | 0 | 0 | 153 | 164 |  |
| R.QNQEYQVLLDVRAR.L | N | 58.17 | 1730.9064 | 14 | 0.9 | 577.9766 | 3 | 60.80 | 5 | F5:24777 | 29102019\_RID\_1313\_NaNaPb\_F4.raw |  |  |  |  | 1.2E6 |  |  |  |  |  | 1 | 0 | 0 | 0 | 0 | 1 | 0 | 0 | 0 | 0 | 0 | 335 | 348 |  |
| R.LVVQIDNAK.L | N | 52.63 | 998.5760 | 9 | -0.2 | 500.2952 | 2 | 48.54 | 5 | F5:14488 | 29102019\_RID\_1313\_NaNaPb\_F4.raw |  |  |  |  | 2.26E7 |  |  |  |  |  | 1 | 0 | 0 | 0 | 0 | 1 | 0 | 0 | 0 | 0 | 0 | 127 | 135 |  |
| R.ARLEC(+57.02)EINTYR.S | N | 49.72 | 1423.6877 | 11 | 0.3 | 475.5700 | 3 | 44.89 | 5 | F5:12054 | 29102019\_RID\_1313\_NaNaPb\_F4.raw |  |  |  |  | 7.57E5 |  |  |  |  |  | 1 | 0 | 0 | 0 | 0 | 1 | 0 | 0 | 0 | 0 | 0 | 347 | 357 | Carbamidomethylation |
| K.VRQLERDNAELENLIR.E | N | 48.97 | 1967.0548 | 16 | 0.3 | 492.7711 | 4 | 55.75 | 5 | F5:20433 | 29102019\_RID\_1313\_NaNaPb\_F4.raw |  |  |  |  | 3.44E6 |  |  |  |  |  | 1 | 0 | 0 | 0 | 0 | 1 | 0 | 0 | 0 | 0 | 0 | 74 | 89 |  |
| R.QLERDNAELENLIR.E | N | 48.55 | 1711.8853 | 14 | 0.2 | 571.6358 | 3 | 60.69 | 5 | F5:24620 | 29102019\_RID\_1313\_NaNaPb\_F4.raw |  |  |  |  | 9.29E6 |  |  |  |  |  | 2 | 0 | 0 | 0 | 0 | 2 | 0 | 0 | 0 | 0 | 0 | 76 | 89 |  |
| R.SQYEALVETNRR.E | N | 46.20 | 1464.7321 | 12 | 0.0 | 489.2513 | 3 | 43.37 | 5 | F5:10875 | 29102019\_RID\_1313\_NaNaPb\_F4.raw |  |  |  |  | 1.24E7 |  |  |  |  |  | 2 | 0 | 0 | 0 | 0 | 2 | 0 | 0 | 0 | 0 | 0 | 230 | 241 |  |
| total 16 peptides |
| --- |

P01946|HBA\_RAT

back to list

  

| Protein Coverage
| Supporting Peptides
|

Protein Coverage:

Supporting Peptides:

| Peptide | Uniq | -10lgP | Mass | Length | ppm | m/z | z | RT | Fraction | Scan | Source File | Area F1 | Area F10 | Area F2 | Area F3 | Area F4 | Area F5 | Area F6 | Area F7 | Area F8 | Area F9 | #Feature | #Feature F1 | #Feature F10 | #Feature F2 | #Feature F3 | #Feature F4 | #Feature F5 | #Feature F6 | #Feature F7 | #Feature F8 | #Feature F9 | Start | End | PTM |
| --- | --- | --- | --- | --- | --- | --- | --- | --- | --- | --- | --- | --- | --- | --- | --- | --- | --- | --- | --- | --- | --- | --- | --- | --- | --- | --- | --- | --- | --- | --- | --- | --- | --- | --- | --- |
| K.TYFSHIDVSPGSAQVK.A | Y | 126.69 | 1734.8577 | 16 | 0.8 | 579.2936 | 3 | 11.87 | 4 | F4:2681 | 29102019\_RID\_1313\_NaNaPb\_F3.raw | 7.73E6 |  | 2.09E5 | 1.49E6 |  | 1.05E5 |  | 1.17E6 | 1.25E5 | 0 | 137 | 118 | 0 | 10 | 4 | 0 | 1 | 0 | 2 | 1 | 1 | 42 | 57 |  |
| K.IGGHGGEYGEEALQR.M | Y | 93.37 | 1571.7328 | 15 | 0.4 | 524.9184 | 3 | 11.15 | 8 | F8:1684 | 29102019\_RID\_1313\_NaNaPb\_F7.raw | 5.57E4 |  | 3.93E4 |  |  |  |  | 1.14E5 |  |  | 4 | 1 | 0 | 1 | 0 | 0 | 0 | 0 | 2 | 0 | 0 | 18 | 32 |  |
| K.AADHVEDLPGALSTLSDLHAHK.L | Y | 89.45 | 2296.1448 | 22 | 0.7 | 766.3894 | 3 | 4.46 | 1 | F1:1115 | 29102019\_RID\_1313\_NaNaPb\_F1.raw | 5.88E6 | 2.18E4 | 5.36E5 | 6.02E5 |  | 1.94E5 |  | 3.84E5 | 1.93E4 | 9.69E4 | 57 | 39 | 1 | 2 | 6 | 0 | 3 | 0 | 3 | 1 | 2 | 70 | 91 |  |
| K.FLASVSTVLTSK.Y | Y | 87.51 | 1251.7074 | 12 | 0.9 | 626.8615 | 2 | 19.51 | 1 | F1:6123 | 29102019\_RID\_1313\_NaNaPb\_F1.raw | 8.71E6 |  | 3.86E6 | 1.61E6 |  | 5.7E5 | 2.96E5 | 6.68E5 | 0 | 1.25E5 | 127 | 94 | 0 | 13 | 8 | 0 | 4 | 2 | 4 | 1 | 1 | 129 | 140 |  |
| K.AADHVEDLPGALSTLSDLHAHKLR.V | Y | 55.39 | 2565.3298 | 24 | 0.4 | 514.0734 | 5 | 12.06 | 8 | F8:2028 | 29102019\_RID\_1313\_NaNaPb\_F7.raw |  |  |  |  |  |  |  | 7.35E3 |  |  | 1 | 0 | 0 | 0 | 0 | 0 | 0 | 0 | 1 | 0 | 0 | 70 | 93 |  |
| R.MFAAFPTTK.T | Y | 55.14 | 1012.5052 | 9 | 0.0 | 507.2599 | 2 | 15.09 | 1 | F1:4954 | 29102019\_RID\_1313\_NaNaPb\_F1.raw | 1.06E4 |  |  |  |  |  |  | 3.79E3 |  |  | 2 | 1 | 0 | 0 | 0 | 0 | 0 | 0 | 1 | 0 | 0 | 33 | 41 |  |
| total 6 peptides |
| --- |

P01427|3S11\_NAJOX

back to list

  

| Protein Coverage
| Supporting Peptides
|

Protein Coverage:

Supporting Peptides:

| Peptide | Uniq | -10lgP | Mass | Length | ppm | m/z | z | RT | Fraction | Scan | Source File | Area F1 | Area F10 | Area F2 | Area F3 | Area F4 | Area F5 | Area F6 | Area F7 | Area F8 | Area F9 | #Feature | #Feature F1 | #Feature F10 | #Feature F2 | #Feature F3 | #Feature F4 | #Feature F5 | #Feature F6 | #Feature F7 | #Feature F8 | #Feature F9 | Start | End | PTM |
| --- | --- | --- | --- | --- | --- | --- | --- | --- | --- | --- | --- | --- | --- | --- | --- | --- | --- | --- | --- | --- | --- | --- | --- | --- | --- | --- | --- | --- | --- | --- | --- | --- | --- | --- | --- |
| LEC(+57.02)HNQQSSQPPTTK.T | Y | 106.69 | 1753.8053 | 15 | -1.5 | 585.6082 | 3 | 10.80 | 1 | F1:2426 | 29102019\_RID\_1313\_NaNaPb\_F1.raw | 5.94E5 |  | 9.41E7 |  | 5.15E5 |  |  |  |  |  | 4 | 1 | 0 | 2 | 0 | 1 | 0 | 0 | 0 | 0 | 0 | 1 | 15 | Carbamidomethylation |
| K.VKPGVNLNC(+57.02)C(+57.02)R.T | Y | 82.27 | 1315.6489 | 11 | 0.5 | 439.5571 | 3 | 11.64 | 1 | F1:3160 | 29102019\_RID\_1313\_NaNaPb\_F1.raw | 4.84E7 |  | 2.25E7 | 2.14E7 | 1.32E6 | 7.51E4 | 1.91E5 |  |  |  | 16 | 6 | 0 | 5 | 2 | 1 | 1 | 1 | 0 | 0 | 0 | 45 | 55 | Carbamidomethylation |
| K.KWWSDHRGTIIER.G | Y | 81.71 | 1682.8641 | 13 | -0.3 | 421.7232 | 4 | 43.87 | 5 | F5:11297 | 29102019\_RID\_1313\_NaNaPb\_F4.raw | 1.23E5 |  |  |  | 6.15E6 |  |  |  |  |  | 3 | 1 | 0 | 0 | 0 | 2 | 0 | 0 | 0 | 0 | 0 | 26 | 38 |  |
| K.PGVNLNC(+57.02)C(+57.02)R.T | Y | 78.92 | 1088.4856 | 9 | -2.9 | 545.2485 | 2 | 11.14 | 1 | F1:2676 | 29102019\_RID\_1313\_NaNaPb\_F1.raw | 3.83E6 |  | 1.57E6 | 1.91E6 |  |  | 2.41E4 |  |  |  | 5 | 2 | 0 | 1 | 1 | 0 | 0 | 1 | 0 | 0 | 0 | 47 | 55 | Carbamidomethylation |
| K.VKPGVNLNC(+57.02)C(+57.02).R | Y | 72.84 | 1159.5479 | 10 | 0.1 | 580.7812 | 2 | 11.48 | 1 | F1:3013 | 29102019\_RID\_1313\_NaNaPb\_F1.raw | 1.11E6 |  | 9.65E5 | 4.92E5 |  |  |  |  |  |  | 3 | 1 | 0 | 1 | 1 | 0 | 0 | 0 | 0 | 0 | 0 | 45 | 54 | Carbamidomethylation |
| K.WWSDHRGTIIER.G | Y | 62.35 | 1554.7692 | 12 | 0.0 | 389.6996 | 4 | 11.89 | 1 | F1:3369 | 29102019\_RID\_1313\_NaNaPb\_F1.raw | 9.55E5 |  | 1.78E5 |  | 2.06E7 |  |  |  |  |  | 5 | 2 | 0 | 1 | 0 | 2 | 0 | 0 | 0 | 0 | 0 | 27 | 38 |  |
| K.VKPGVNLNC(+57.02).C | Y | 61.60 | 999.5172 | 9 | -0.5 | 500.7656 | 2 | 11.46 | 1 | F1:2992 | 29102019\_RID\_1313\_NaNaPb\_F1.raw | 1.41E6 |  | 6.96E5 | 4.9E5 |  |  |  |  |  |  | 3 | 1 | 0 | 1 | 1 | 0 | 0 | 0 | 0 | 0 | 0 | 45 | 53 | Carbamidomethylation |
| K.KWWSDHR.G | Y | 61.38 | 1013.4832 | 7 | -0.7 | 507.7485 | 2 | 11.24 | 1 | F1:2787 | 29102019\_RID\_1313\_NaNaPb\_F1.raw | 2.64E5 |  | 8.58E4 |  |  |  |  |  |  |  | 2 | 1 | 0 | 1 | 0 | 0 | 0 | 0 | 0 | 0 | 0 | 26 | 32 |  |
| K.VKPGVNLNC(+57.02)C(+57.02)RTDRC(+57.02)NN | Y | 57.00 | 2075.9412 | 17 | -0.6 | 519.9922 | 4 | 11.04 | 3 | F3:1889 | 29102019\_RID\_1313\_NaNaPb\_F2.raw |  |  | 1.73E5 |  |  |  |  |  |  |  | 1 | 0 | 0 | 1 | 0 | 0 | 0 | 0 | 0 | 0 | 0 | 45 | 61 | Carbamidomethylation |
| K.VKPGVNLN.C | Y | 44.32 | 839.4865 | 8 | 0.5 | 420.7507 | 2 | 11.40 | 1 | F1:2924 | 29102019\_RID\_1313\_NaNaPb\_F1.raw | 6.77E6 |  |  |  |  |  |  |  |  |  | 1 | 1 | 0 | 0 | 0 | 0 | 0 | 0 | 0 | 0 | 0 | 45 | 52 |  |
| total 10 peptides |
| --- |

P10117|PA2H1\_LATCO

back to list

  

| Protein Coverage
| Supporting Peptides
|

Protein Coverage:

Supporting Peptides:

| Peptide | Uniq | -10lgP | Mass | Length | ppm | m/z | z | RT | Fraction | Scan | Source File | Area F1 | Area F10 | Area F2 | Area F3 | Area F4 | Area F5 | Area F6 | Area F7 | Area F8 | Area F9 | #Feature | #Feature F1 | #Feature F10 | #Feature F2 | #Feature F3 | #Feature F4 | #Feature F5 | #Feature F6 | #Feature F7 | #Feature F8 | #Feature F9 | Start | End | PTM |
| --- | --- | --- | --- | --- | --- | --- | --- | --- | --- | --- | --- | --- | --- | --- | --- | --- | --- | --- | --- | --- | --- | --- | --- | --- | --- | --- | --- | --- | --- | --- | --- | --- | --- | --- | --- |
| Y.GC(+57.02)YC(+57.02)GPGGSGTPVDDLDR.C | Y | 113.16 | 1881.7621 | 18 | 0.2 | 941.8885 | 2 | 11.70 | 9 | F9:2506 | 29102019\_RID\_1313\_NaNaPb\_F8.raw |  |  |  |  |  |  |  |  | 5.35E6 |  | 1 | 0 | 0 | 0 | 0 | 0 | 0 | 0 | 0 | 1 | 0 | 26 | 43 | Carbamidomethylation |
| D.YGC(+57.02)YC(+57.02)GPGGSGTPVDDLDR.C | Y | 99.54 | 2044.8254 | 19 | 1.1 | 1023.4211 | 2 | 11.87 | 9 | F9:2661 | 29102019\_RID\_1313\_NaNaPb\_F8.raw |  |  |  |  |  |  |  |  | 5.31E5 |  | 1 | 0 | 0 | 0 | 0 | 0 | 0 | 0 | 0 | 1 | 0 | 25 | 43 | Carbamidomethylation |
| Y.C(+57.02)GPGGSGTPVDDLDR.C | Y | 93.71 | 1501.6467 | 15 | 0.1 | 751.8307 | 2 | 11.26 | 9 | F9:2140 | 29102019\_RID\_1313\_NaNaPb\_F8.raw |  | 8.67E4 |  |  |  | 7.57E4 |  |  | 6.78E4 | 5.96E4 | 4 | 0 | 1 | 0 | 0 | 0 | 1 | 0 | 0 | 1 | 1 | 29 | 43 | Carbamidomethylation |
| P.GGSGTPVDDLDR.C | N | 79.46 | 1187.5417 | 12 | -1.4 | 594.7773 | 2 | 11.42 | 7 | F7:2302 | 29102019\_RID\_1313\_NaNaPb\_F6.raw |  | 3.83E5 |  |  |  | 9.47E5 | 2.08E5 |  |  | 7.26E5 | 10 | 0 | 1 | 0 | 0 | 0 | 7 | 1 | 0 | 0 | 1 | 32 | 43 |  |
| M.DYGC(+57.02)YC(+57.02)GPGGSGTPVDDLDR.C | Y | 67.58 | 2159.8523 | 20 | 1.6 | 1080.9352 | 2 | 12.44 | 7 | F7:3172 | 29102019\_RID\_1313\_NaNaPb\_F6.raw |  |  |  |  |  | 2.65E5 | 4.54E4 |  |  |  | 2 | 0 | 0 | 0 | 0 | 0 | 1 | 1 | 0 | 0 | 0 | 24 | 43 | Carbamidomethylation |
| G.C(+57.02)YC(+57.02)GPGGSGTPVDDLDR.C | Y | 65.77 | 1824.7407 | 17 | -0.2 | 913.3774 | 2 | 11.72 | 9 | F9:2526 | 29102019\_RID\_1313\_NaNaPb\_F8.raw |  |  |  |  |  |  |  |  | 1.06E5 |  | 1 | 0 | 0 | 0 | 0 | 0 | 0 | 0 | 0 | 1 | 0 | 27 | 43 | Carbamidomethylation |
| G.SGTPVDDLDR.C | N | 47.92 | 1073.4989 | 10 | -2.3 | 537.7555 | 2 | 11.45 | 6 | F6:2250 | 29102019\_RID\_1313\_NaNaPb\_F5.raw |  |  |  |  |  | 2.06E5 |  |  |  |  | 1 | 0 | 0 | 0 | 0 | 0 | 1 | 0 | 0 | 0 | 0 | 34 | 43 |  |
| S.GTPVDDLDR.C | N | 47.24 | 986.4669 | 9 | 0.0 | 494.2407 | 2 | 11.37 | 9 | F9:2225 | 29102019\_RID\_1313\_NaNaPb\_F8.raw |  |  |  |  |  |  |  |  | 5.17E4 |  | 1 | 0 | 0 | 0 | 0 | 0 | 0 | 0 | 0 | 1 | 0 | 35 | 43 |  |
| total 8 peptides |
| --- |

P62377|3SOFL\_NAJNA

back to list

  

| Protein Coverage
| Supporting Peptides
|

Protein Coverage:

Supporting Peptides:

| Peptide | Uniq | -10lgP | Mass | Length | ppm | m/z | z | RT | Fraction | Scan | Source File | Area F1 | Area F10 | Area F2 | Area F3 | Area F4 | Area F5 | Area F6 | Area F7 | Area F8 | Area F9 | #Feature | #Feature F1 | #Feature F10 | #Feature F2 | #Feature F3 | #Feature F4 | #Feature F5 | #Feature F6 | #Feature F7 | #Feature F8 | #Feature F9 | Start | End | PTM |
| --- | --- | --- | --- | --- | --- | --- | --- | --- | --- | --- | --- | --- | --- | --- | --- | --- | --- | --- | --- | --- | --- | --- | --- | --- | --- | --- | --- | --- | --- | --- | --- | --- | --- | --- | --- |
| K.C(+57.02)HNTQLPFIYK.T | Y | 104.98 | 1419.6969 | 11 | 0.4 | 710.8560 | 2 | 11.82 | 9 | F9:2605 | 29102019\_RID\_1313\_NaNaPb\_F8.raw |  |  |  |  |  | 1.62E6 |  |  | 1.66E7 | 1.42E5 | 6 | 0 | 0 | 0 | 0 | 0 | 2 | 0 | 0 | 3 | 1 | 3 | 13 | Carbamidomethylation |
| K.NSALLKYVC(+57.02)C(+57.02).S | Y | 72.02 | 1226.5787 | 10 | 1.0 | 614.2972 | 2 | 12.37 | 9 | F9:3070 | 29102019\_RID\_1313\_NaNaPb\_F8.raw |  |  |  |  |  |  |  |  | 5.04E4 |  | 1 | 0 | 0 | 0 | 0 | 0 | 0 | 0 | 0 | 1 | 0 | 47 | 56 | Carbamidomethylation |
| LKC(+57.02)HNTQLPFIYK.T | Y | 67.91 | 1660.8759 | 13 | -0.8 | 416.2259 | 4 | 11.51 | 9 | F9:2366 | 29102019\_RID\_1313\_NaNaPb\_F8.raw |  |  |  |  |  |  |  |  | 6.62E5 |  | 2 | 0 | 0 | 0 | 0 | 0 | 0 | 0 | 0 | 2 | 0 | 1 | 13 | Carbamidomethylation |
| K.NSALLKYVC(+57.02).C | Y | 66.88 | 1066.5481 | 9 | 1.2 | 534.2820 | 2 | 12.20 | 9 | F9:2920 | 29102019\_RID\_1313\_NaNaPb\_F8.raw |  |  |  |  |  |  |  |  | 5.21E4 |  | 1 | 0 | 0 | 0 | 0 | 0 | 0 | 0 | 0 | 1 | 0 | 47 | 55 | Carbamidomethylation |
| K.FPLKFPVK.R | Y | 61.45 | 974.5953 | 8 | -0.4 | 488.3047 | 2 | 12.07 | 9 | F9:2762 | 29102019\_RID\_1313\_NaNaPb\_F8.raw |  |  |  |  |  |  |  |  | 6.71E5 |  | 1 | 0 | 0 | 0 | 0 | 0 | 0 | 0 | 0 | 1 | 0 | 30 | 37 |  |
| K.FPLKFPVKR.G | Y | 60.51 | 1130.6964 | 9 | -6.8 | 377.9035 | 3 | 11.53 | 9 | F9:2353 | 29102019\_RID\_1313\_NaNaPb\_F8.raw |  |  |  |  |  |  |  |  | 2.46E5 |  | 1 | 0 | 0 | 0 | 0 | 0 | 0 | 0 | 0 | 1 | 0 | 30 | 38 |  |
| K.KFPLKFPVKR.G | Y | 57.09 | 1258.7914 | 10 | 0.3 | 420.6045 | 3 | 11.24 | 9 | F9:2083 | 29102019\_RID\_1313\_NaNaPb\_F8.raw |  |  |  |  |  |  |  |  | 1.78E5 |  | 1 | 0 | 0 | 0 | 0 | 0 | 0 | 0 | 0 | 1 | 0 | 29 | 38 |  |
| K.NSALLKYV.C | Y | 55.77 | 906.5175 | 8 | 0.5 | 454.2662 | 2 | 12.55 | 9 | F9:3226 | 29102019\_RID\_1313\_NaNaPb\_F8.raw |  |  |  |  |  |  |  |  | 9.86E3 |  | 1 | 0 | 0 | 0 | 0 | 0 | 0 | 0 | 0 | 1 | 0 | 47 | 54 |  |
| L.LKYVC(+57.02)C(+57.02)STDKC(+57.02)N | Y | 53.11 | 1546.6578 | 12 | -0.4 | 516.5597 | 3 | 10.73 | 9 | F9:1637 | 29102019\_RID\_1313\_NaNaPb\_F8.raw |  |  |  |  |  |  |  |  | 6.03E4 |  | 1 | 0 | 0 | 0 | 0 | 0 | 0 | 0 | 0 | 1 | 0 | 51 | 62 | Carbamidomethylation |
| LKC(+57.02)HNTQLPFIYKTC(+57.02)PEGKNLC(+57.02)FK.A | Y | 50.80 | 2995.4871 | 24 | -0.5 | 600.1044 | 5 | 11.57 | 9 | F9:2404 | 29102019\_RID\_1313\_NaNaPb\_F8.raw |  |  |  |  |  |  |  |  | 2.49E5 |  | 1 | 0 | 0 | 0 | 0 | 0 | 0 | 0 | 0 | 1 | 0 | 1 | 24 | Carbamidomethylation |
| K.TC(+57.02)PEGKNLC(+57.02)FK.A | Y | 48.53 | 1352.6217 | 11 | 0.2 | 451.8813 | 3 | 11.04 | 9 | F9:1919 | 29102019\_RID\_1313\_NaNaPb\_F8.raw |  |  |  |  |  |  |  |  | 3.18E4 |  | 1 | 0 | 0 | 0 | 0 | 0 | 0 | 0 | 0 | 1 | 0 | 14 | 24 | Carbamidomethylation |
| K.C(+57.02)HNTQLPFIY.K | Y | 47.91 | 1291.6019 | 10 | 0.3 | 646.8084 | 2 | 18.10 | 9 | F9:6531 | 29102019\_RID\_1313\_NaNaPb\_F8.raw |  |  |  |  |  |  |  |  | 4.88E5 |  | 1 | 0 | 0 | 0 | 0 | 0 | 0 | 0 | 0 | 1 | 0 | 3 | 12 | Carbamidomethylation |
| K.C(+57.02)HNTQLPF.I | Y | 44.02 | 1015.4545 | 8 | -0.6 | 508.7342 | 2 | 12.07 | 9 | F9:2759 | 29102019\_RID\_1313\_NaNaPb\_F8.raw |  |  |  |  |  |  |  |  | 1.16E8 |  | 1 | 0 | 0 | 0 | 0 | 0 | 0 | 0 | 0 | 1 | 0 | 3 | 10 | Carbamidomethylation |
| total 13 peptides |
| --- |

Q5YF89|NGFV2\_NAJSP

back to list

  

| Protein Coverage
| Supporting Peptides
|

Protein Coverage:

Supporting Peptides:

| Peptide | Uniq | -10lgP | Mass | Length | ppm | m/z | z | RT | Fraction | Scan | Source File | Area F1 | Area F10 | Area F2 | Area F3 | Area F4 | Area F5 | Area F6 | Area F7 | Area F8 | Area F9 | #Feature | #Feature F1 | #Feature F10 | #Feature F2 | #Feature F3 | #Feature F4 | #Feature F5 | #Feature F6 | #Feature F7 | #Feature F8 | #Feature F9 | Start | End | PTM |
| --- | --- | --- | --- | --- | --- | --- | --- | --- | --- | --- | --- | --- | --- | --- | --- | --- | --- | --- | --- | --- | --- | --- | --- | --- | --- | --- | --- | --- | --- | --- | --- | --- | --- | --- | --- |
| K.GNTVTVM(+15.99)ENVNLDNK.V | Y | 101.70 | 1662.7883 | 15 | 0.6 | 832.4020 | 2 | 11.51 | 7 | F7:2378 | 29102019\_RID\_1313\_NaNaPb\_F6.raw |  |  |  |  |  |  | 8.04E4 |  |  |  | 1 | 0 | 0 | 0 | 0 | 0 | 0 | 1 | 0 | 0 | 0 | 156 | 170 | Oxidation (M) |
| R.EDHPVHNLGEHSVC(+57.02)DSVSAWVTK.T | Y | 98.45 | 2602.1870 | 23 | 0.9 | 651.5546 | 4 | 11.67 | 9 | F9:2477 | 29102019\_RID\_1313\_NaNaPb\_F8.raw |  |  |  |  |  |  |  |  | 5.3E5 |  | 2 | 0 | 0 | 0 | 0 | 0 | 0 | 0 | 0 | 2 | 0 | 126 | 148 | Carbamidomethylation |
| K.ALTM(+15.99)EGNQASWR.F | Y | 89.43 | 1378.6299 | 12 | -0.3 | 690.3220 | 2 | 11.33 | 9 | F9:2184 | 29102019\_RID\_1313\_NaNaPb\_F8.raw |  |  |  |  |  |  |  |  | 1.02E5 |  | 1 | 0 | 0 | 0 | 0 | 0 | 0 | 0 | 0 | 1 | 0 | 212 | 223 | Oxidation (M) |
| R.IDTAC(+57.02)VC(+57.02)VITK.K | Y | 79.43 | 1278.6312 | 11 | 0.2 | 640.3230 | 2 | 11.80 | 7 | F7:2640 | 29102019\_RID\_1313\_NaNaPb\_F6.raw |  | 9.43E4 |  |  |  | 4.26E5 | 2.81E5 |  |  | 3.18E5 | 5 | 0 | 2 | 0 | 0 | 0 | 1 | 1 | 0 | 0 | 1 | 227 | 237 | Carbamidomethylation |
| K.GNTVTVMENVNLDNK.V | Y | 79.18 | 1646.7933 | 15 | -1.9 | 824.4023 | 2 | 12.05 | 7 | F7:2876 | 29102019\_RID\_1313\_NaNaPb\_F6.raw |  |  |  |  |  |  | 9.93E4 |  |  |  | 1 | 0 | 0 | 0 | 0 | 0 | 0 | 1 | 0 | 0 | 0 | 156 | 170 |  |
| N.SYC(+57.02)TETDTFIK.A | Y | 70.57 | 1363.5966 | 11 | -0.4 | 682.8053 | 2 | 11.77 | 10 | F10:2598 | 29102019\_RID\_1313\_NaNaPb\_F9.raw |  |  |  |  |  |  | 4.59E5 |  |  | 2.62E5 | 2 | 0 | 0 | 0 | 0 | 0 | 0 | 1 | 0 | 0 | 1 | 201 | 211 | Carbamidomethylation |
| R.GIDSSHWNSYC(+57.02)TETDTFIK.A | Y | 66.02 | 2259.9744 | 19 | 0.4 | 754.3324 | 3 | 12.40 | 10 | F10:3176 | 29102019\_RID\_1313\_NaNaPb\_F9.raw |  |  |  |  |  |  |  |  |  | 1.91E4 | 1 | 0 | 0 | 0 | 0 | 0 | 0 | 0 | 0 | 0 | 1 | 193 | 211 | Carbamidomethylation |
| K.QYFFETK.C | Y | 62.82 | 961.4545 | 7 | -0.1 | 481.7345 | 2 | 12.00 | 9 | F9:2752 | 29102019\_RID\_1313\_NaNaPb\_F8.raw |  | 7.12E4 |  |  |  | 1.58E5 | 8.37E5 |  | 8.27E5 |  | 4 | 0 | 1 | 0 | 0 | 0 | 1 | 1 | 0 | 1 | 0 | 174 | 180 |  |
| H.SVC(+57.02)DSVSAWVTK.T | Y | 50.89 | 1337.6285 | 12 | 1.3 | 669.8224 | 2 | 12.86 | 9 | F9:3410 | 29102019\_RID\_1313\_NaNaPb\_F8.raw |  |  |  |  |  |  |  |  | 1.32E5 |  | 1 | 0 | 0 | 0 | 0 | 0 | 0 | 0 | 0 | 1 | 0 | 137 | 148 | Carbamidomethylation |
| total 9 peptides |
| --- |

P82464|3SO8\_NAJKA

back to list

  

| Protein Coverage
| Supporting Peptides
|

Protein Coverage:

Supporting Peptides:

| Peptide | Uniq | -10lgP | Mass | Length | ppm | m/z | z | RT | Fraction | Scan | Source File | Area F1 | Area F10 | Area F2 | Area F3 | Area F4 | Area F5 | Area F6 | Area F7 | Area F8 | Area F9 | #Feature | #Feature F1 | #Feature F10 | #Feature F2 | #Feature F3 | #Feature F4 | #Feature F5 | #Feature F6 | #Feature F7 | #Feature F8 | #Feature F9 | Start | End | PTM |
| --- | --- | --- | --- | --- | --- | --- | --- | --- | --- | --- | --- | --- | --- | --- | --- | --- | --- | --- | --- | --- | --- | --- | --- | --- | --- | --- | --- | --- | --- | --- | --- | --- | --- | --- | --- |
| R.TSETTEIC(+57.02)PDSWYFC(+57.02)YK.I | Y | 100.53 | 2185.8972 | 17 | 0.3 | 1093.9562 | 2 | 22.50 | 6 | F6:8662 | 29102019\_RID\_1313\_NaNaPb\_F5.raw |  |  |  |  |  | 3.45E7 |  |  |  |  | 2 | 0 | 0 | 0 | 0 | 0 | 2 | 0 | 0 | 0 | 0 | 10 | 26 | Carbamidomethylation |
| R.GC(+57.02)TFTC(+57.02)PELRPTGIYVY.C | Y | 91.58 | 2032.9386 | 17 | 0.5 | 1017.4771 | 2 | 22.97 | 6 | F6:8989 | 29102019\_RID\_1313\_NaNaPb\_F5.raw |  |  |  |  |  | 2.14E7 |  |  |  |  | 1 | 0 | 0 | 0 | 0 | 0 | 1 | 0 | 0 | 0 | 0 | 40 | 56 | Carbamidomethylation |
| R.GC(+57.02)TFTC(+57.02)PELRPTGIYVYC(+57.02)C(+57.02)R.R | Y | 71.24 | 2509.1011 | 20 | 1.6 | 837.3757 | 3 | 13.90 | 6 | F6:4481 | 29102019\_RID\_1313\_NaNaPb\_F5.raw |  |  |  |  |  | 5.85E6 |  |  |  |  | 1 | 0 | 0 | 0 | 0 | 0 | 1 | 0 | 0 | 0 | 0 | 40 | 59 | Carbamidomethylation |
| K.ISLADGNDVR.I | Y | 67.95 | 1058.5356 | 10 | 0.0 | 530.2751 | 2 | 11.49 | 7 | F7:2350 | 29102019\_RID\_1313\_NaNaPb\_F6.raw |  |  |  |  |  | 7.27E6 | 1.52E5 |  |  |  | 2 | 0 | 0 | 0 | 0 | 0 | 1 | 1 | 0 | 0 | 0 | 27 | 36 |  |
| R.PTGIYVYC(+57.02)C(+57.02)R.R | Y | 67.60 | 1287.5740 | 10 | -0.4 | 644.7940 | 2 | 11.86 | 6 | F6:2604 | 29102019\_RID\_1313\_NaNaPb\_F5.raw |  |  |  |  |  | 5.75E5 |  |  |  |  | 1 | 0 | 0 | 0 | 0 | 0 | 1 | 0 | 0 | 0 | 0 | 50 | 59 | Carbamidomethylation |
| R.GC(+57.02)TFTC(+57.02)PELRPT.G | Y | 66.45 | 1437.6381 | 12 | 0.3 | 719.8265 | 2 | 11.91 | 6 | F6:2678 | 29102019\_RID\_1313\_NaNaPb\_F5.raw |  |  |  |  |  | 1.81E5 |  |  |  |  | 1 | 0 | 0 | 0 | 0 | 0 | 1 | 0 | 0 | 0 | 0 | 40 | 51 | Carbamidomethylation |
| TIC(+57.02)YNHLTR.T | Y | 66.00 | 1176.5709 | 9 | -2.5 | 393.1966 | 3 | 11.16 | 6 | F6:1976 | 29102019\_RID\_1313\_NaNaPb\_F5.raw |  |  |  |  |  | 5.2E5 |  |  |  |  | 2 | 0 | 0 | 0 | 0 | 0 | 2 | 0 | 0 | 0 | 0 | 1 | 9 | Carbamidomethylation |
| R.GC(+57.02)TFTC(+57.02)PELR.P | Y | 63.30 | 1239.5376 | 10 | 0.7 | 620.7765 | 2 | 11.78 | 6 | F6:2554 | 29102019\_RID\_1313\_NaNaPb\_F5.raw |  |  |  |  |  | 2.85E6 |  |  |  |  | 1 | 0 | 0 | 0 | 0 | 0 | 1 | 0 | 0 | 0 | 0 | 40 | 49 | Carbamidomethylation |
| R.TSETTEIC(+57.02)PDSWY.F | Y | 52.52 | 1587.6399 | 13 | 1.1 | 794.8281 | 2 | 22.91 | 6 | F6:8900 | 29102019\_RID\_1313\_NaNaPb\_F5.raw |  |  |  |  |  | 1.97E6 |  |  |  |  | 1 | 0 | 0 | 0 | 0 | 0 | 1 | 0 | 0 | 0 | 0 | 10 | 22 | Carbamidomethylation |
| R.GC(+57.02)TFTC(+57.02)PELRPTGIY.V | Y | 44.30 | 1770.8069 | 15 | 0.5 | 886.4112 | 2 | 16.02 | 6 | F6:5435 | 29102019\_RID\_1313\_NaNaPb\_F5.raw |  |  |  |  |  | 1.76E6 |  |  |  |  | 1 | 0 | 0 | 0 | 0 | 0 | 1 | 0 | 0 | 0 | 0 | 40 | 54 | Carbamidomethylation |
| total 10 peptides |
| --- |

P82885|VESP\_NAJKA

back to list

  

| Protein Coverage
| Supporting Peptides
|

Protein Coverage:

Supporting Peptides:

| Peptide | Uniq | -10lgP | Mass | Length | ppm | m/z | z | RT | Fraction | Scan | Source File | Area F1 | Area F10 | Area F2 | Area F3 | Area F4 | Area F5 | Area F6 | Area F7 | Area F8 | Area F9 | #Feature | #Feature F1 | #Feature F10 | #Feature F2 | #Feature F3 | #Feature F4 | #Feature F5 | #Feature F6 | #Feature F7 | #Feature F8 | #Feature F9 | Start | End | PTM |
| --- | --- | --- | --- | --- | --- | --- | --- | --- | --- | --- | --- | --- | --- | --- | --- | --- | --- | --- | --- | --- | --- | --- | --- | --- | --- | --- | --- | --- | --- | --- | --- | --- | --- | --- | --- |
| K.TVENVGVSQVAPDNPER.F | Y | 114.06 | 1809.8856 | 17 | 0.9 | 905.9509 | 2 | 11.49 | 2 | F2:2387 | 29102019\_RID\_1313\_NaNaPb\_F10.raw |  | 3.91E6 |  |  |  |  |  |  |  | 7.6E6 | 3 | 0 | 1 | 0 | 0 | 0 | 0 | 0 | 0 | 0 | 2 | 30 | 46 |  |
| K.ADVTFDSNTAFESLVVSPDK.K | Y | 91.02 | 2141.0164 | 20 | 0.8 | 1071.5164 | 2 | 28.83 | 2 | F2:10035 | 29102019\_RID\_1313\_NaNaPb\_F10.raw |  | 2.52E6 |  |  |  |  |  |  |  |  | 3 | 0 | 3 | 0 | 0 | 0 | 0 | 0 | 0 | 0 | 0 | 9 | 28 |  |
| R.FDGSPC(+57.02)VLGSPGFR.S | Y | 85.03 | 1494.6925 | 14 | 1.1 | 748.3544 | 2 | 13.50 | 2 | F2:3976 | 29102019\_RID\_1313\_NaNaPb\_F10.raw |  | 2.01E6 |  |  |  |  |  |  |  | 3.56E6 | 4 | 0 | 1 | 0 | 0 | 0 | 0 | 0 | 0 | 0 | 3 | 47 | 60 | Carbamidomethylation |
| K.ADVTFDSNTAFESLVVSPDKK.T | Y | 78.93 | 2269.1113 | 21 | 0.9 | 757.3784 | 3 | 19.28 | 2 | F2:7075 | 29102019\_RID\_1313\_NaNaPb\_F10.raw |  | 5.03E6 |  |  |  |  | 2.07E5 |  | 1.88E4 | 4.79E5 | 7 | 0 | 4 | 0 | 0 | 0 | 0 | 1 | 0 | 1 | 1 | 9 | 29 |  |
| R.EWAVGLAGK.S | Y | 61.34 | 929.4970 | 9 | -0.4 | 465.7556 | 2 | 12.16 | 2 | F2:3051 | 29102019\_RID\_1313\_NaNaPb\_F10.raw |  | 1.1E6 |  |  |  |  |  |  |  | 1.06E6 | 2 | 0 | 1 | 0 | 0 | 0 | 0 | 0 | 0 | 0 | 1 | 75 | 83 |  |
| C.VLGSPGFR.S | Y | 44.12 | 831.4603 | 8 | 0.2 | 416.7375 | 2 | 11.49 | 2 | F2:2406 | 29102019\_RID\_1313\_NaNaPb\_F10.raw |  | 1.26E5 |  |  |  |  |  |  |  |  | 1 | 0 | 1 | 0 | 0 | 0 | 0 | 0 | 0 | 0 | 0 | 53 | 60 |  |
| total 6 peptides |
| --- |

Q9YGI2|3NO21\_NAJAT

back to list

  

| Protein Coverage
| Supporting Peptides
|

Protein Coverage:

Supporting Peptides:

| Peptide | Uniq | -10lgP | Mass | Length | ppm | m/z | z | RT | Fraction | Scan | Source File | Area F1 | Area F10 | Area F2 | Area F3 | Area F4 | Area F5 | Area F6 | Area F7 | Area F8 | Area F9 | #Feature | #Feature F1 | #Feature F10 | #Feature F2 | #Feature F3 | #Feature F4 | #Feature F5 | #Feature F6 | #Feature F7 | #Feature F8 | #Feature F9 | Start | End | PTM |
| --- | --- | --- | --- | --- | --- | --- | --- | --- | --- | --- | --- | --- | --- | --- | --- | --- | --- | --- | --- | --- | --- | --- | --- | --- | --- | --- | --- | --- | --- | --- | --- | --- | --- | --- | --- |
| T.LTC(+57.02)LIC(+57.02)PEKYC(+57.02)NKVHTC(+57.02)LNGEK.I | Y | 87.17 | 2736.2856 | 22 | 1.1 | 913.1035 | 3 | 50.88 | 5 | F5:16443 | 29102019\_RID\_1313\_NaNaPb\_F4.raw |  |  |  |  | 4E7 |  |  |  |  |  | 4 | 0 | 0 | 0 | 0 | 4 | 0 | 0 | 0 | 0 | 0 | 22 | 43 | Carbamidomethylation |
| K.VHTC(+57.02)LNGEKIC(+57.02)FK.K | Y | 81.18 | 1604.7803 | 13 | -0.6 | 535.9337 | 3 | 43.25 | 5 | F5:10834 | 29102019\_RID\_1313\_NaNaPb\_F4.raw |  |  |  |  | 1.1E6 |  |  |  |  |  | 1 | 0 | 0 | 0 | 0 | 1 | 0 | 0 | 0 | 0 | 0 | 35 | 47 | Carbamidomethylation |
| K.VHTC(+57.02)LNGEKIC(+57.02)FKKYDQR.K | Y | 80.84 | 2295.1252 | 18 | 0.8 | 460.0327 | 5 | 39.25 | 5 | F5:7771 | 29102019\_RID\_1313\_NaNaPb\_F4.raw |  |  |  |  | 3.36E6 |  |  |  |  |  | 3 | 0 | 0 | 0 | 0 | 3 | 0 | 0 | 0 | 0 | 0 | 35 | 52 | Carbamidomethylation |
| K.VHTC(+57.02)LNGEKIC(+57.02)FKK.Y | Y | 79.01 | 1732.8752 | 14 | -0.1 | 578.6323 | 3 | 37.89 | 5 | F5:6743 | 29102019\_RID\_1313\_NaNaPb\_F4.raw |  |  |  |  | 3.36E6 |  |  |  |  |  | 2 | 0 | 0 | 0 | 0 | 2 | 0 | 0 | 0 | 0 | 0 | 35 | 48 | Carbamidomethylation |
| R.YIRGC(+57.02)ADTC(+57.02)PVRKPR.E | Y | 75.04 | 1847.9247 | 15 | 0.4 | 462.9886 | 4 | 36.62 | 5 | F5:5779 | 29102019\_RID\_1313\_NaNaPb\_F4.raw |  |  |  |  | 3.69E7 |  |  |  |  |  | 1 | 0 | 0 | 0 | 0 | 1 | 0 | 0 | 0 | 0 | 0 | 59 | 73 | Carbamidomethylation |
| T.LTC(+57.02)LIC(+57.02)PEKYC(+57.02)NK.V | N | 68.19 | 1697.7939 | 13 | 1.5 | 849.9055 | 2 | 51.23 | 5 | F5:16637 | 29102019\_RID\_1313\_NaNaPb\_F4.raw |  |  |  |  | 9.28E6 |  |  |  |  |  | 3 | 0 | 0 | 0 | 0 | 3 | 0 | 0 | 0 | 0 | 0 | 22 | 34 | Carbamidomethylation |
| K.IC(+57.02)FKKYDQR.K | Y | 63.46 | 1256.6335 | 9 | -0.4 | 419.8849 | 3 | 36.74 | 5 | F5:5862 | 29102019\_RID\_1313\_NaNaPb\_F4.raw |  |  |  |  | 4.3E6 |  |  |  |  |  | 1 | 0 | 0 | 0 | 0 | 1 | 0 | 0 | 0 | 0 | 0 | 44 | 52 | Carbamidomethylation |
| K.YDQRKLLGKR.Y | Y | 55.53 | 1275.7411 | 10 | 0.4 | 426.2545 | 3 | 36.62 | 5 | F5:5763 | 29102019\_RID\_1313\_NaNaPb\_F4.raw |  |  |  |  | 3.61E6 |  |  |  |  |  | 1 | 0 | 0 | 0 | 0 | 1 | 0 | 0 | 0 | 0 | 0 | 49 | 58 |  |
| T.LTC(+57.02)LIC(+57.02)PEK.Y | N | 54.39 | 1132.5620 | 9 | 0.7 | 567.2887 | 2 | 53.22 | 5 | F5:18191 | 29102019\_RID\_1313\_NaNaPb\_F4.raw |  |  |  |  | 1.87E7 | 7.32E4 |  |  |  |  | 2 | 0 | 0 | 0 | 0 | 1 | 1 | 0 | 0 | 0 | 0 | 22 | 30 | Carbamidomethylation |
| T.LTC(+57.02)LIC(+57.02)PEKYC(+57.02)NKVHTC(+57.02)LNGEKIC(+57.02)FK.K | Y | 46.72 | 3284.5637 | 26 | 0.9 | 657.9206 | 5 | 56.76 | 5 | F5:21233 | 29102019\_RID\_1313\_NaNaPb\_F4.raw |  |  |  |  | 8.58E6 |  |  |  |  |  | 1 | 0 | 0 | 0 | 0 | 1 | 0 | 0 | 0 | 0 | 0 | 22 | 47 | Carbamidomethylation |
| K.YC(+57.02)NKVHTC(+57.02)LNGEKIC(+57.02)FKK.Y | Y | 43.62 | 2298.1072 | 18 | 0.6 | 575.5344 | 4 | 37.89 | 5 | F5:6730 | 29102019\_RID\_1313\_NaNaPb\_F4.raw |  |  |  |  | 2.04E6 |  |  |  |  |  | 1 | 0 | 0 | 0 | 0 | 1 | 0 | 0 | 0 | 0 | 0 | 31 | 48 | Carbamidomethylation |
| K.RYIRGC(+57.02)ADTC(+57.02)PVRKPR.E | Y | 42.80 | 2004.0258 | 16 | 0.1 | 401.8125 | 5 | 36.62 | 5 | F5:5784 | 29102019\_RID\_1313\_NaNaPb\_F4.raw |  |  |  |  | 2.37E6 |  |  |  |  |  | 1 | 0 | 0 | 0 | 0 | 1 | 0 | 0 | 0 | 0 | 0 | 58 | 73 | Carbamidomethylation |
| total 12 peptides |
| --- |

O93422|3NO2H\_NAJAT

back to list

  

| Protein Coverage
| Supporting Peptides
|

Protein Coverage:

Supporting Peptides:

| Peptide | Uniq | -10lgP | Mass | Length | ppm | m/z | z | RT | Fraction | Scan | Source File | Area F1 | Area F10 | Area F2 | Area F3 | Area F4 | Area F5 | Area F6 | Area F7 | Area F8 | Area F9 | #Feature | #Feature F1 | #Feature F10 | #Feature F2 | #Feature F3 | #Feature F4 | #Feature F5 | #Feature F6 | #Feature F7 | #Feature F8 | #Feature F9 | Start | End | PTM |
| --- | --- | --- | --- | --- | --- | --- | --- | --- | --- | --- | --- | --- | --- | --- | --- | --- | --- | --- | --- | --- | --- | --- | --- | --- | --- | --- | --- | --- | --- | --- | --- | --- | --- | --- | --- |
| T.LTC(+57.02)LIC(+57.02)PEKYC(+57.02)NKVHTC(+57.02)LNGEK.I | Y | 87.17 | 2736.2856 | 22 | 1.1 | 913.1035 | 3 | 50.88 | 5 | F5:16443 | 29102019\_RID\_1313\_NaNaPb\_F4.raw |  |  |  |  | 4E7 |  |  |  |  |  | 4 | 0 | 0 | 0 | 0 | 4 | 0 | 0 | 0 | 0 | 0 | 22 | 43 | Carbamidomethylation |
| K.VHTC(+57.02)LNGEKIC(+57.02)FK.K | Y | 81.18 | 1604.7803 | 13 | -0.6 | 535.9337 | 3 | 43.25 | 5 | F5:10834 | 29102019\_RID\_1313\_NaNaPb\_F4.raw |  |  |  |  | 1.1E6 |  |  |  |  |  | 1 | 0 | 0 | 0 | 0 | 1 | 0 | 0 | 0 | 0 | 0 | 35 | 47 | Carbamidomethylation |
| K.VHTC(+57.02)LNGEKIC(+57.02)FKKYDQR.K | Y | 80.84 | 2295.1252 | 18 | 0.8 | 460.0327 | 5 | 39.25 | 5 | F5:7771 | 29102019\_RID\_1313\_NaNaPb\_F4.raw |  |  |  |  | 3.36E6 |  |  |  |  |  | 3 | 0 | 0 | 0 | 0 | 3 | 0 | 0 | 0 | 0 | 0 | 35 | 52 | Carbamidomethylation |
| K.VHTC(+57.02)LNGEKIC(+57.02)FKK.Y | Y | 79.01 | 1732.8752 | 14 | -0.1 | 578.6323 | 3 | 37.89 | 5 | F5:6743 | 29102019\_RID\_1313\_NaNaPb\_F4.raw |  |  |  |  | 3.36E6 |  |  |  |  |  | 2 | 0 | 0 | 0 | 0 | 2 | 0 | 0 | 0 | 0 | 0 | 35 | 48 | Carbamidomethylation |
| R.YIRGC(+57.02)ADTC(+57.02)PVRKPR.E | Y | 75.04 | 1847.9247 | 15 | 0.4 | 462.9886 | 4 | 36.62 | 5 | F5:5779 | 29102019\_RID\_1313\_NaNaPb\_F4.raw |  |  |  |  | 3.69E7 |  |  |  |  |  | 1 | 0 | 0 | 0 | 0 | 1 | 0 | 0 | 0 | 0 | 0 | 59 | 73 | Carbamidomethylation |
| T.LTC(+57.02)LIC(+57.02)PEKYC(+57.02)NK.V | N | 68.19 | 1697.7939 | 13 | 1.5 | 849.9055 | 2 | 51.23 | 5 | F5:16637 | 29102019\_RID\_1313\_NaNaPb\_F4.raw |  |  |  |  | 9.28E6 |  |  |  |  |  | 3 | 0 | 0 | 0 | 0 | 3 | 0 | 0 | 0 | 0 | 0 | 22 | 34 | Carbamidomethylation |
| K.IC(+57.02)FKKYDQR.K | Y | 63.46 | 1256.6335 | 9 | -0.4 | 419.8849 | 3 | 36.74 | 5 | F5:5862 | 29102019\_RID\_1313\_NaNaPb\_F4.raw |  |  |  |  | 4.3E6 |  |  |  |  |  | 1 | 0 | 0 | 0 | 0 | 1 | 0 | 0 | 0 | 0 | 0 | 44 | 52 | Carbamidomethylation |
| K.YDQRKLLGKR.Y | Y | 55.53 | 1275.7411 | 10 | 0.4 | 426.2545 | 3 | 36.62 | 5 | F5:5763 | 29102019\_RID\_1313\_NaNaPb\_F4.raw |  |  |  |  | 3.61E6 |  |  |  |  |  | 1 | 0 | 0 | 0 | 0 | 1 | 0 | 0 | 0 | 0 | 0 | 49 | 58 |  |
| T.LTC(+57.02)LIC(+57.02)PEK.Y | N | 54.39 | 1132.5620 | 9 | 0.7 | 567.2887 | 2 | 53.22 | 5 | F5:18191 | 29102019\_RID\_1313\_NaNaPb\_F4.raw |  |  |  |  | 1.87E7 | 7.32E4 |  |  |  |  | 2 | 0 | 0 | 0 | 0 | 1 | 1 | 0 | 0 | 0 | 0 | 22 | 30 | Carbamidomethylation |
| T.LTC(+57.02)LIC(+57.02)PEKYC(+57.02)NKVHTC(+57.02)LNGEKIC(+57.02)FK.K | Y | 46.72 | 3284.5637 | 26 | 0.9 | 657.9206 | 5 | 56.76 | 5 | F5:21233 | 29102019\_RID\_1313\_NaNaPb\_F4.raw |  |  |  |  | 8.58E6 |  |  |  |  |  | 1 | 0 | 0 | 0 | 0 | 1 | 0 | 0 | 0 | 0 | 0 | 22 | 47 | Carbamidomethylation |
| K.YC(+57.02)NKVHTC(+57.02)LNGEKIC(+57.02)FKK.Y | Y | 43.62 | 2298.1072 | 18 | 0.6 | 575.5344 | 4 | 37.89 | 5 | F5:6730 | 29102019\_RID\_1313\_NaNaPb\_F4.raw |  |  |  |  | 2.04E6 |  |  |  |  |  | 1 | 0 | 0 | 0 | 0 | 1 | 0 | 0 | 0 | 0 | 0 | 31 | 48 | Carbamidomethylation |
| K.RYIRGC(+57.02)ADTC(+57.02)PVRKPR.E | Y | 42.80 | 2004.0258 | 16 | 0.1 | 401.8125 | 5 | 36.62 | 5 | F5:5784 | 29102019\_RID\_1313\_NaNaPb\_F4.raw |  |  |  |  | 2.37E6 |  |  |  |  |  | 1 | 0 | 0 | 0 | 0 | 1 | 0 | 0 | 0 | 0 | 0 | 58 | 73 | Carbamidomethylation |
| total 12 peptides |
| --- |

P80245|3SAN\_NAJAT

back to list

  

| Protein Coverage
| Supporting Peptides
|

Protein Coverage:

Supporting Peptides:

| Peptide | Uniq | -10lgP | Mass | Length | ppm | m/z | z | RT | Fraction | Scan | Source File | Area F1 | Area F10 | Area F2 | Area F3 | Area F4 | Area F5 | Area F6 | Area F7 | Area F8 | Area F9 | #Feature | #Feature F1 | #Feature F10 | #Feature F2 | #Feature F3 | #Feature F4 | #Feature F5 | #Feature F6 | #Feature F7 | #Feature F8 | #Feature F9 | Start | End | PTM |
| --- | --- | --- | --- | --- | --- | --- | --- | --- | --- | --- | --- | --- | --- | --- | --- | --- | --- | --- | --- | --- | --- | --- | --- | --- | --- | --- | --- | --- | --- | --- | --- | --- | --- | --- | --- |
| K.SSLLVKYVC(+57.02)C(+57.02)NTDRC(+57.02)N | N | 100.66 | 1987.8914 | 16 | 0.9 | 663.6383 | 3 | 11.70 | 2 | F2:2614 | 29102019\_RID\_1313\_NaNaPb\_F10.raw |  | 1.43E6 |  |  |  |  |  |  | 3.81E4 |  | 3 | 0 | 2 | 0 | 0 | 0 | 0 | 0 | 0 | 1 | 0 | 66 | 81 | Carbamidomethylation |
| K.SSLLVKYVC(+57.02)C(+57.02)NTDR.C | N | 64.23 | 1713.8179 | 14 | 0.5 | 857.9166 | 2 | 11.72 | 2 | F2:2635 | 29102019\_RID\_1313\_NaNaPb\_F10.raw |  | 6.38E5 |  |  |  |  |  |  |  |  | 1 | 0 | 1 | 0 | 0 | 0 | 0 | 0 | 0 | 0 | 0 | 66 | 79 | Carbamidomethylation |
| R.GC(+57.02)IDVC(+57.02)PK.S | N | 63.51 | 947.4205 | 8 | -0.6 | 474.7172 | 2 | 11.17 | 7 | F7:2031 | 29102019\_RID\_1313\_NaNaPb\_F6.raw |  | 2.85E7 |  |  |  | 7.32E7 | 1.17E6 |  | 5.42E6 | 9.79E6 | 10 | 0 | 2 | 0 | 0 | 0 | 5 | 1 | 0 | 1 | 1 | 58 | 65 | Carbamidomethylation |
| L.VKYVC(+57.02)C(+57.02)NTDRC(+57.02)N | N | 63.21 | 1587.6592 | 12 | 0.8 | 530.2274 | 3 | 10.67 | 9 | F9:1583 | 29102019\_RID\_1313\_NaNaPb\_F8.raw |  |  |  |  |  |  |  |  | 2.28E6 |  | 2 | 0 | 0 | 0 | 0 | 0 | 0 | 0 | 0 | 2 | 0 | 70 | 81 | Carbamidomethylation |
| R.GC(+57.02)IDVC(+57.02)PKSSLLVK.Y | N | 59.21 | 1574.8160 | 14 | -0.2 | 525.9459 | 3 | 11.68 | 10 | F10:2508 | 29102019\_RID\_1313\_NaNaPb\_F9.raw |  | 5.2E5 |  |  |  |  |  |  | 0 | 9.79E5 | 3 | 0 | 1 | 0 | 0 | 0 | 0 | 0 | 0 | 1 | 1 | 58 | 71 | Carbamidomethylation |
| R.GC(+57.02)IDVC(+57.02)PKSSLLVKYVC(+57.02)C(+57.02)NTDR.C | N | 58.37 | 2643.2278 | 22 | 0.3 | 661.8144 | 4 | 12.16 | 2 | F2:3053 | 29102019\_RID\_1313\_NaNaPb\_F10.raw |  | 2.73E5 |  |  |  |  |  |  |  |  | 1 | 0 | 1 | 0 | 0 | 0 | 0 | 0 | 0 | 0 | 0 | 58 | 79 | Carbamidomethylation |
| S.SLLVKYVC(+57.02)C(+57.02)NTDRC(+57.02)N | N | 56.59 | 1900.8594 | 15 | 0.6 | 951.4375 | 2 | 11.57 | 9 | F9:2415 | 29102019\_RID\_1313\_NaNaPb\_F8.raw |  |  |  |  |  |  |  |  | 7.39E5 |  | 2 | 0 | 0 | 0 | 0 | 0 | 0 | 0 | 0 | 2 | 0 | 67 | 81 | Carbamidomethylation |
| L.LVKYVC(+57.02)C(+57.02)NTDRC(+57.02)N | N | 54.08 | 1700.7433 | 13 | 0.0 | 567.9217 | 3 | 10.88 | 9 | F9:1785 | 29102019\_RID\_1313\_NaNaPb\_F8.raw |  |  |  |  |  |  |  |  | 2.77E5 |  | 1 | 0 | 0 | 0 | 0 | 0 | 0 | 0 | 0 | 1 | 0 | 69 | 81 | Carbamidomethylation |
| K.SSLLVKYVC(+57.02).C | N | 49.20 | 1067.5685 | 9 | 0.8 | 534.7919 | 2 | 12.39 | 2 | F2:3276 | 29102019\_RID\_1313\_NaNaPb\_F10.raw |  | 2.19E5 |  |  |  |  |  |  |  | 2.98E5 | 2 | 0 | 1 | 0 | 0 | 0 | 0 | 0 | 0 | 0 | 1 | 66 | 74 | Carbamidomethylation |
| K.RGC(+57.02)IDVC(+57.02)PK.S | N | 48.01 | 1103.5216 | 9 | 0.2 | 552.7682 | 2 | 10.91 | 6 | F6:1732 | 29102019\_RID\_1313\_NaNaPb\_F5.raw |  |  |  |  |  | 6.74E5 |  |  |  |  | 1 | 0 | 0 | 0 | 0 | 0 | 1 | 0 | 0 | 0 | 0 | 57 | 65 | Carbamidomethylation |
| K.C(+57.02)NQLIPPFYK.T | Y | 47.86 | 1278.6431 | 10 | 0.3 | 640.3290 | 2 | 15.43 | 9 | F9:5127 | 29102019\_RID\_1313\_NaNaPb\_F8.raw |  |  |  |  |  |  |  |  | 1.94E5 |  | 1 | 0 | 0 | 0 | 0 | 0 | 0 | 0 | 0 | 1 | 0 | 24 | 33 | Carbamidomethylation |
| total 11 peptides |
| --- |

P02091|HBB1\_RAT

back to list

  

| Protein Coverage
| Supporting Peptides
|

Protein Coverage:

Supporting Peptides:

| Peptide | Uniq | -10lgP | Mass | Length | ppm | m/z | z | RT | Fraction | Scan | Source File | Area F1 | Area F10 | Area F2 | Area F3 | Area F4 | Area F5 | Area F6 | Area F7 | Area F8 | Area F9 | #Feature | #Feature F1 | #Feature F10 | #Feature F2 | #Feature F3 | #Feature F4 | #Feature F5 | #Feature F6 | #Feature F7 | #Feature F8 | #Feature F9 | Start | End | PTM |
| --- | --- | --- | --- | --- | --- | --- | --- | --- | --- | --- | --- | --- | --- | --- | --- | --- | --- | --- | --- | --- | --- | --- | --- | --- | --- | --- | --- | --- | --- | --- | --- | --- | --- | --- | --- |
| K.VNPDDVGGEALGR.L | Y | 77.22 | 1297.6262 | 13 | 0.6 | 649.8208 | 2 | 11.57 | 8 | F8:1838 | 29102019\_RID\_1313\_NaNaPb\_F7.raw |  |  |  |  |  |  |  | 5.91E4 |  | 0 | 2 | 0 | 0 | 0 | 0 | 0 | 0 | 0 | 1 | 0 | 1 | 19 | 31 |  |
| K.VVAGVASALAHK.Y | N | 74.76 | 1121.6556 | 12 | 0.8 | 561.8356 | 2 | 11.37 | 8 | F8:1754 | 29102019\_RID\_1313\_NaNaPb\_F7.raw |  |  |  |  |  |  |  | 3.88E4 |  |  | 1 | 0 | 0 | 0 | 0 | 0 | 0 | 0 | 1 | 0 | 0 | 134 | 145 |  |
| K.KVINAFNDGLK.H | N | 74.72 | 1217.6768 | 11 | -0.4 | 406.8994 | 3 | 11.46 | 8 | F8:1803 | 29102019\_RID\_1313\_NaNaPb\_F7.raw | 1.74E4 |  |  |  |  |  |  | 1.15E4 |  |  | 2 | 1 | 0 | 0 | 0 | 0 | 0 | 0 | 1 | 0 | 0 | 67 | 77 |  |
| K.VINAFNDGLK.H | N | 69.92 | 1089.5818 | 10 | 0.6 | 545.7985 | 2 | 12.21 | 4 | F4:2985 | 29102019\_RID\_1313\_NaNaPb\_F3.raw | 3.14E3 |  | 1.48E5 | 7.73E4 |  |  | 0 |  |  |  | 4 | 1 | 0 | 1 | 1 | 0 | 0 | 1 | 0 | 0 | 0 | 68 | 77 |  |
| R.LLVVYPWTQR.Y | N | 66.77 | 1273.7183 | 10 | 1.0 | 637.8671 | 2 | 27.07 | 1 | F1:8363 | 29102019\_RID\_1313\_NaNaPb\_F1.raw | 5.83E6 |  | 1.67E6 | 7.01E5 |  | 2.18E5 | 1.48E5 | 1.91E5 |  |  | 52 | 33 | 0 | 8 | 6 | 0 | 2 | 1 | 2 | 0 | 0 | 32 | 41 |  |
| K.LHVDPENFR.L | N | 64.94 | 1125.5566 | 9 | -0.7 | 376.1926 | 3 | 11.48 | 8 | F8:1809 | 29102019\_RID\_1313\_NaNaPb\_F7.raw | 9.63E4 |  |  |  |  |  |  | 3.19E4 |  |  | 4 | 2 | 0 | 0 | 0 | 0 | 0 | 0 | 2 | 0 | 0 | 97 | 105 |  |
| K.EFTPC(+57.02)AQAAFQK.V | N | 64.83 | 1396.6445 | 12 | 0.2 | 699.3297 | 2 | 12.13 | 1 | F1:3625 | 29102019\_RID\_1313\_NaNaPb\_F1.raw | 5.6E4 |  |  |  |  |  |  | 1.96E4 |  |  | 2 | 1 | 0 | 0 | 0 | 0 | 0 | 0 | 1 | 0 | 0 | 122 | 133 | Carbamidomethylation |
| K.AAVNGLWGK.V | Y | 60.23 | 914.4974 | 9 | -0.3 | 458.2558 | 2 | 11.84 | 8 | F8:1968 | 29102019\_RID\_1313\_NaNaPb\_F7.raw | 7.08E4 |  | 3.68E4 |  |  |  |  | 2.55E4 |  |  | 3 | 1 | 0 | 1 | 0 | 0 | 0 | 0 | 1 | 0 | 0 | 10 | 18 |  |
| V.VAGVASALAHK.Y | N | 59.76 | 1022.5872 | 11 | 0.3 | 512.3010 | 2 | 11.37 | 8 | F8:1759 | 29102019\_RID\_1313\_NaNaPb\_F7.raw |  |  |  |  |  |  |  | 1.84E4 |  |  | 1 | 0 | 0 | 0 | 0 | 0 | 0 | 0 | 1 | 0 | 0 | 135 | 145 |  |
| M.VHLTDAEK.A | N | 59.37 | 911.4712 | 8 | 0.1 | 456.7429 | 2 | 10.69 | 8 | F8:1577 | 29102019\_RID\_1313\_NaNaPb\_F7.raw |  |  | 4.27E4 | 8.25E3 |  |  |  | 4.64E4 |  | 2.36E4 | 4 | 0 | 0 | 1 | 1 | 0 | 0 | 0 | 1 | 0 | 1 | 2 | 9 |  |
| R.YFDSFGDLSSASAIMGNPK.V | Y | 59.00 | 2005.9091 | 19 | 1.3 | 1003.9631 | 2 | 10.43 | 4 | F4:1590 | 29102019\_RID\_1313\_NaNaPb\_F3.raw | 0 |  |  | 0 |  |  |  |  |  |  | 2 | 1 | 0 | 0 | 1 | 0 | 0 | 0 | 0 | 0 | 0 | 42 | 60 |  |
| K.VVAGVASALAHKYH | N | 51.55 | 1421.7778 | 14 | -0.3 | 474.9331 | 3 | 11.41 | 8 | F8:1780 | 29102019\_RID\_1313\_NaNaPb\_F7.raw |  |  |  |  |  |  |  | 1.14E4 |  |  | 1 | 0 | 0 | 0 | 0 | 0 | 0 | 0 | 1 | 0 | 0 | 134 | 147 |  |
| R.YFDSFGDLSSASAIM(+15.99)GNPK.V | Y | 48.37 | 2021.9041 | 19 | -0.1 | 1011.9592 | 2 | 18.96 | 7 | F7:6670 | 29102019\_RID\_1313\_NaNaPb\_F6.raw |  |  |  | 7.36E3 |  |  | 9.27E3 |  |  |  | 2 | 0 | 0 | 0 | 1 | 0 | 0 | 1 | 0 | 0 | 0 | 42 | 60 | Oxidation (M) |
| K.VVAGVASALAH.K | N | 43.43 | 993.5607 | 11 | 0.2 | 497.7877 | 2 | 11.78 | 8 | F8:1933 | 29102019\_RID\_1313\_NaNaPb\_F7.raw |  |  |  |  |  |  |  | 0 |  |  | 1 | 0 | 0 | 0 | 0 | 0 | 0 | 0 | 1 | 0 | 0 | 134 | 144 |  |
| V.AGVASALAHK.Y | N | 43.15 | 923.5188 | 10 | -0.2 | 462.7666 | 2 | 11.37 | 8 | F8:1761 | 29102019\_RID\_1313\_NaNaPb\_F7.raw |  |  |  |  |  |  |  | 4.55E4 |  |  | 1 | 0 | 0 | 0 | 0 | 0 | 0 | 0 | 1 | 0 | 0 | 136 | 145 |  |
| total 15 peptides |
| --- |

P84805|CRVP1\_NAJKA

back to list

  

| Protein Coverage
| Supporting Peptides
|

Protein Coverage:

Supporting Peptides:

| Peptide | Uniq | -10lgP | Mass | Length | ppm | m/z | z | RT | Fraction | Scan | Source File | Area F1 | Area F10 | Area F2 | Area F3 | Area F4 | Area F5 | Area F6 | Area F7 | Area F8 | Area F9 | #Feature | #Feature F1 | #Feature F10 | #Feature F2 | #Feature F3 | #Feature F4 | #Feature F5 | #Feature F6 | #Feature F7 | #Feature F8 | #Feature F9 | Start | End | PTM |
| --- | --- | --- | --- | --- | --- | --- | --- | --- | --- | --- | --- | --- | --- | --- | --- | --- | --- | --- | --- | --- | --- | --- | --- | --- | --- | --- | --- | --- | --- | --- | --- | --- | --- | --- | --- |
| R.VLEGIQC(+57.02)GESIYM(+15.99)SSNAR.T | Y | 98.48 | 2028.9244 | 18 | 0.4 | 1015.4699 | 2 | 12.08 | 2 | F2:2983 | 29102019\_RID\_1313\_NaNaPb\_F10.raw |  | 2.64E5 |  |  |  |  |  |  |  |  | 2 | 0 | 2 | 0 | 0 | 0 | 0 | 0 | 0 | 0 | 0 | 85 | 102 | Carbamidomethylation; Oxidation (M) |
| F.YVC(+57.02)QYC(+57.02)PSGNFQGK.T | Y | 83.85 | 1706.7181 | 14 | 1.1 | 854.3672 | 2 | 11.45 | 2 | F2:2359 | 29102019\_RID\_1313\_NaNaPb\_F10.raw |  | 1.98E5 |  |  |  |  |  |  |  |  | 1 | 0 | 1 | 0 | 0 | 0 | 0 | 0 | 0 | 0 | 0 | 161 | 174 | Carbamidomethylation |
| K.SNC(+57.02)PASC(+57.02)FC(+57.02)R.N | Y | 71.40 | 1257.4689 | 10 | 1.0 | 629.7423 | 2 | 11.11 | 2 | F2:2028 | 29102019\_RID\_1313\_NaNaPb\_F10.raw |  | 4.7E5 |  |  |  |  | 9.28E4 |  | 5.18E4 |  | 3 | 0 | 1 | 0 | 0 | 0 | 0 | 1 | 0 | 1 | 0 | 226 | 235 | Carbamidomethylation |
| K.M(+15.99)EWYPEAASNAER.W | Y | 71.18 | 1568.6565 | 13 | 0.8 | 785.3361 | 2 | 11.68 | 2 | F2:2621 | 29102019\_RID\_1313\_NaNaPb\_F10.raw |  | 1.11E5 |  |  |  |  |  |  |  |  | 1 | 0 | 1 | 0 | 0 | 0 | 0 | 0 | 0 | 0 | 0 | 57 | 69 | Oxidation (M) |
| K.LGPPC(+57.02)GDC(+57.02)PSAC(+57.02)DNGLC(+57.02)TNPC(+57.02)TIYNK.L | Y | 68.60 | 2940.1970 | 26 | 0.4 | 981.0734 | 3 | 12.12 | 2 | F2:2985 | 29102019\_RID\_1313\_NaNaPb\_F10.raw |  | 6.8E5 |  |  |  |  |  |  |  | 1.37E5 | 2 | 0 | 1 | 0 | 0 | 0 | 0 | 0 | 0 | 0 | 1 | 181 | 206 | Carbamidomethylation |
| G.NVDFNSESTR.R | Y | 67.41 | 1167.5156 | 10 | -0.4 | 584.7648 | 2 | 11.09 | 2 | F2:2011 | 29102019\_RID\_1313\_NaNaPb\_F10.raw |  | 2.88E5 |  |  |  |  |  |  |  |  | 1 | 0 | 1 | 0 | 0 | 0 | 0 | 0 | 0 | 0 | 0 | 19 | 28 |  |
| K.MEWYPEAASNAER.W | Y | 66.19 | 1552.6616 | 13 | 0.3 | 777.3383 | 2 | 12.14 | 2 | F2:3029 | 29102019\_RID\_1313\_NaNaPb\_F10.raw |  | 1.28E5 |  |  |  |  |  |  |  |  | 1 | 0 | 1 | 0 | 0 | 0 | 0 | 0 | 0 | 0 | 0 | 57 | 69 |  |
| K.EIVDLHNSLR.R | Y | 55.11 | 1194.6356 | 10 | -0.3 | 399.2190 | 3 | 11.53 | 2 | F2:2449 | 29102019\_RID\_1313\_NaNaPb\_F10.raw |  | 2.8E5 |  |  |  |  |  |  |  |  | 1 | 0 | 1 | 0 | 0 | 0 | 0 | 0 | 0 | 0 | 0 | 35 | 44 |  |
| F.GNVDFNSESTR.R | Y | 45.76 | 1224.5371 | 11 | 0.1 | 613.2759 | 2 | 11.20 | 2 | F2:2118 | 29102019\_RID\_1313\_NaNaPb\_F10.raw |  | 6.11E4 |  |  |  |  |  |  |  |  | 1 | 0 | 1 | 0 | 0 | 0 | 0 | 0 | 0 | 0 | 0 | 18 | 28 |  |
| R.VSPTASNMLK.M | Y | 45.48 | 1046.5430 | 10 | 0.1 | 524.2788 | 2 | 11.34 | 2 | F2:2264 | 29102019\_RID\_1313\_NaNaPb\_F10.raw |  | 3.76E4 |  |  |  |  |  |  |  |  | 1 | 0 | 1 | 0 | 0 | 0 | 0 | 0 | 0 | 0 | 0 | 47 | 56 |  |
| total 10 peptides |
| --- |

Q7T1K6|CRVP1\_NAJAT

back to list

  

| Protein Coverage
| Supporting Peptides
|

Protein Coverage:

Supporting Peptides:

| Peptide | Uniq | -10lgP | Mass | Length | ppm | m/z | z | RT | Fraction | Scan | Source File | Area F1 | Area F10 | Area F2 | Area F3 | Area F4 | Area F5 | Area F6 | Area F7 | Area F8 | Area F9 | #Feature | #Feature F1 | #Feature F10 | #Feature F2 | #Feature F3 | #Feature F4 | #Feature F5 | #Feature F6 | #Feature F7 | #Feature F8 | #Feature F9 | Start | End | PTM |
| --- | --- | --- | --- | --- | --- | --- | --- | --- | --- | --- | --- | --- | --- | --- | --- | --- | --- | --- | --- | --- | --- | --- | --- | --- | --- | --- | --- | --- | --- | --- | --- | --- | --- | --- | --- |
| R.VLEGIQC(+57.02)GESIYM(+15.99)SSNAR.T | Y | 98.48 | 2028.9244 | 18 | 0.4 | 1015.4699 | 2 | 12.08 | 2 | F2:2983 | 29102019\_RID\_1313\_NaNaPb\_F10.raw |  | 2.64E5 |  |  |  |  |  |  |  |  | 2 | 0 | 2 | 0 | 0 | 0 | 0 | 0 | 0 | 0 | 0 | 85 | 102 | Carbamidomethylation; Oxidation (M) |
| F.YVC(+57.02)QYC(+57.02)PSGNFQGK.T | Y | 83.85 | 1706.7181 | 14 | 1.1 | 854.3672 | 2 | 11.45 | 2 | F2:2359 | 29102019\_RID\_1313\_NaNaPb\_F10.raw |  | 1.98E5 |  |  |  |  |  |  |  |  | 1 | 0 | 1 | 0 | 0 | 0 | 0 | 0 | 0 | 0 | 0 | 161 | 174 | Carbamidomethylation |
| K.SNC(+57.02)PASC(+57.02)FC(+57.02)R.N | Y | 71.40 | 1257.4689 | 10 | 1.0 | 629.7423 | 2 | 11.11 | 2 | F2:2028 | 29102019\_RID\_1313\_NaNaPb\_F10.raw |  | 4.7E5 |  |  |  |  | 9.28E4 |  | 5.18E4 |  | 3 | 0 | 1 | 0 | 0 | 0 | 0 | 1 | 0 | 1 | 0 | 226 | 235 | Carbamidomethylation |
| K.M(+15.99)EWYPEAASNAER.W | Y | 71.18 | 1568.6565 | 13 | 0.8 | 785.3361 | 2 | 11.68 | 2 | F2:2621 | 29102019\_RID\_1313\_NaNaPb\_F10.raw |  | 1.11E5 |  |  |  |  |  |  |  |  | 1 | 0 | 1 | 0 | 0 | 0 | 0 | 0 | 0 | 0 | 0 | 57 | 69 | Oxidation (M) |
| K.LGPPC(+57.02)GDC(+57.02)PSAC(+57.02)DNGLC(+57.02)TNPC(+57.02)TIYNK.L | Y | 68.60 | 2940.1970 | 26 | 0.4 | 981.0734 | 3 | 12.12 | 2 | F2:2985 | 29102019\_RID\_1313\_NaNaPb\_F10.raw |  | 6.8E5 |  |  |  |  |  |  |  | 1.37E5 | 2 | 0 | 1 | 0 | 0 | 0 | 0 | 0 | 0 | 0 | 1 | 181 | 206 | Carbamidomethylation |
| G.NVDFNSESTR.R | Y | 67.41 | 1167.5156 | 10 | -0.4 | 584.7648 | 2 | 11.09 | 2 | F2:2011 | 29102019\_RID\_1313\_NaNaPb\_F10.raw |  | 2.88E5 |  |  |  |  |  |  |  |  | 1 | 0 | 1 | 0 | 0 | 0 | 0 | 0 | 0 | 0 | 0 | 19 | 28 |  |
| K.MEWYPEAASNAER.W | Y | 66.19 | 1552.6616 | 13 | 0.3 | 777.3383 | 2 | 12.14 | 2 | F2:3029 | 29102019\_RID\_1313\_NaNaPb\_F10.raw |  | 1.28E5 |  |  |  |  |  |  |  |  | 1 | 0 | 1 | 0 | 0 | 0 | 0 | 0 | 0 | 0 | 0 | 57 | 69 |  |
| K.EIVDLHNSLR.R | Y | 55.11 | 1194.6356 | 10 | -0.3 | 399.2190 | 3 | 11.53 | 2 | F2:2449 | 29102019\_RID\_1313\_NaNaPb\_F10.raw |  | 2.8E5 |  |  |  |  |  |  |  |  | 1 | 0 | 1 | 0 | 0 | 0 | 0 | 0 | 0 | 0 | 0 | 35 | 44 |  |
| F.GNVDFNSESTR.R | Y | 45.76 | 1224.5371 | 11 | 0.1 | 613.2759 | 2 | 11.20 | 2 | F2:2118 | 29102019\_RID\_1313\_NaNaPb\_F10.raw |  | 6.11E4 |  |  |  |  |  |  |  |  | 1 | 0 | 1 | 0 | 0 | 0 | 0 | 0 | 0 | 0 | 0 | 18 | 28 |  |
| R.VSPTASNMLK.M | Y | 45.48 | 1046.5430 | 10 | 0.1 | 524.2788 | 2 | 11.34 | 2 | F2:2264 | 29102019\_RID\_1313\_NaNaPb\_F10.raw |  | 3.76E4 |  |  |  |  |  |  |  |  | 1 | 0 | 1 | 0 | 0 | 0 | 0 | 0 | 0 | 0 | 0 | 47 | 56 |  |
| total 10 peptides |
| --- |

D6PXE8|VM3B\_NAJAT

back to list

  

| Protein Coverage
| Supporting Peptides
|

Protein Coverage:

Supporting Peptides:

| Peptide | Uniq | -10lgP | Mass | Length | ppm | m/z | z | RT | Fraction | Scan | Source File | Area F1 | Area F10 | Area F2 | Area F3 | Area F4 | Area F5 | Area F6 | Area F7 | Area F8 | Area F9 | #Feature | #Feature F1 | #Feature F10 | #Feature F2 | #Feature F3 | #Feature F4 | #Feature F5 | #Feature F6 | #Feature F7 | #Feature F8 | #Feature F9 | Start | End | PTM |
| --- | --- | --- | --- | --- | --- | --- | --- | --- | --- | --- | --- | --- | --- | --- | --- | --- | --- | --- | --- | --- | --- | --- | --- | --- | --- | --- | --- | --- | --- | --- | --- | --- | --- | --- | --- |
| K.DDC(+57.02)DLPELC(+57.02)TGQSAEC(+57.02)PTDSLQR.N | Y | 109.04 | 2666.0894 | 23 | 0.0 | 1334.0520 | 2 | 14.38 | 10 | F10:4158 | 29102019\_RID\_1313\_NaNaPb\_F9.raw |  | 3.03E6 |  |  |  |  | 9.75E4 |  | 1.33E5 | 4.98E6 | 11 | 0 | 3 | 0 | 0 | 0 | 0 | 1 | 0 | 1 | 6 | 454 | 476 | Carbamidomethylation |
| L.IPPNPDGIMAEPGTK.C | N | 80.93 | 1535.7654 | 15 | 1.0 | 768.8907 | 2 | 11.87 | 7 | F7:2694 | 29102019\_RID\_1313\_NaNaPb\_F6.raw |  |  |  |  |  |  | 1.82E5 |  |  |  | 1 | 0 | 0 | 0 | 0 | 0 | 0 | 1 | 0 | 0 | 0 | 560 | 574 |  |
| K.GQC(+57.02)VDVQTAY | N | 68.80 | 1139.4917 | 10 | 0.9 | 570.7537 | 2 | 11.87 | 7 | F7:2699 | 29102019\_RID\_1313\_NaNaPb\_F6.raw |  |  |  |  |  |  | 1.18E5 |  |  |  | 1 | 0 | 0 | 0 | 0 | 0 | 0 | 1 | 0 | 0 | 0 | 584 | 593 | Carbamidomethylation |
| R.TAPAFQFSSC(+57.02)SIR.E | N | 57.10 | 1470.6925 | 13 | 0.3 | 736.3538 | 2 | 12.49 | 10 | F10:3268 | 29102019\_RID\_1313\_NaNaPb\_F9.raw |  | 8.57E3 |  |  |  |  |  |  |  | 3.87E4 | 2 | 0 | 1 | 0 | 0 | 0 | 0 | 0 | 0 | 0 | 1 | 370 | 382 | Carbamidomethylation |
| L.IPPNPDGIM(+15.99)AEPGTK.C | N | 55.45 | 1551.7603 | 15 | 0.5 | 776.8878 | 2 | 11.45 | 7 | F7:2313 | 29102019\_RID\_1313\_NaNaPb\_F6.raw |  |  |  |  |  |  | 9.78E4 |  |  |  | 1 | 0 | 0 | 0 | 0 | 0 | 0 | 1 | 0 | 0 | 0 | 560 | 574 | Oxidation (M) |
| H.IALIGLEIWSNK.D | Y | 51.19 | 1355.7812 | 12 | 0.3 | 678.8981 | 2 | 35.91 | 2 | F2:11989 | 29102019\_RID\_1313\_NaNaPb\_F10.raw |  | 1.04E6 |  |  |  | 1.78E4 |  |  | 1.7E5 |  | 3 | 0 | 1 | 0 | 0 | 0 | 1 | 0 | 0 | 1 | 0 | 250 | 261 |  |
| A.LIGLEIWSNK.D | Y | 49.12 | 1171.6600 | 10 | 0.4 | 586.8375 | 2 | 23.41 | 9 | F9:9081 | 29102019\_RID\_1313\_NaNaPb\_F8.raw |  | 7.51E5 |  |  |  |  |  |  | 3.4E4 |  | 2 | 0 | 1 | 0 | 0 | 0 | 0 | 0 | 0 | 1 | 0 | 252 | 261 |  |
| I.HIALIGLEIWSNK.D | Y | 47.14 | 1492.8402 | 13 | -0.3 | 498.6205 | 3 | 23.25 | 2 | F2:8137 | 29102019\_RID\_1313\_NaNaPb\_F10.raw |  | 3.57E4 |  |  |  |  |  |  |  |  | 1 | 0 | 1 | 0 | 0 | 0 | 0 | 0 | 0 | 0 | 0 | 249 | 261 |  |
| R.NDNAQLLTGIDFNGNTVGR.A | Y | 43.08 | 2017.9817 | 19 | 2.3 | 1010.0005 | 2 | 19.42 | 2 | F2:6962 | 29102019\_RID\_1313\_NaNaPb\_F10.raw |  | 8.79E3 |  |  |  |  |  |  |  |  | 1 | 0 | 1 | 0 | 0 | 0 | 0 | 0 | 0 | 0 | 0 | 291 | 309 |  |
| total 9 peptides |
| --- |

D3TTC1|VM3KL\_NAJAT

back to list

  

| Protein Coverage
| Supporting Peptides
|

Protein Coverage:

Supporting Peptides:

| Peptide | Uniq | -10lgP | Mass | Length | ppm | m/z | z | RT | Fraction | Scan | Source File | Area F1 | Area F10 | Area F2 | Area F3 | Area F4 | Area F5 | Area F6 | Area F7 | Area F8 | Area F9 | #Feature | #Feature F1 | #Feature F10 | #Feature F2 | #Feature F3 | #Feature F4 | #Feature F5 | #Feature F6 | #Feature F7 | #Feature F8 | #Feature F9 | Start | End | PTM |
| --- | --- | --- | --- | --- | --- | --- | --- | --- | --- | --- | --- | --- | --- | --- | --- | --- | --- | --- | --- | --- | --- | --- | --- | --- | --- | --- | --- | --- | --- | --- | --- | --- | --- | --- | --- |
| K.DDC(+57.02)DLPELC(+57.02)TGQSAEC(+57.02)PTDSLQR.N | Y | 109.04 | 2666.0894 | 23 | 0.0 | 1334.0520 | 2 | 14.38 | 10 | F10:4158 | 29102019\_RID\_1313\_NaNaPb\_F9.raw |  | 3.03E6 |  |  |  |  | 9.75E4 |  | 1.33E5 | 4.98E6 | 11 | 0 | 3 | 0 | 0 | 0 | 0 | 1 | 0 | 1 | 6 | 454 | 476 | Carbamidomethylation |
| L.IPPNPDGIMAEPGTK.C | N | 80.93 | 1535.7654 | 15 | 1.0 | 768.8907 | 2 | 11.87 | 7 | F7:2694 | 29102019\_RID\_1313\_NaNaPb\_F6.raw |  |  |  |  |  |  | 1.82E5 |  |  |  | 1 | 0 | 0 | 0 | 0 | 0 | 0 | 1 | 0 | 0 | 0 | 560 | 574 |  |
| K.GQC(+57.02)VDVQTAY | N | 68.80 | 1139.4917 | 10 | 0.9 | 570.7537 | 2 | 11.87 | 7 | F7:2699 | 29102019\_RID\_1313\_NaNaPb\_F6.raw |  |  |  |  |  |  | 1.18E5 |  |  |  | 1 | 0 | 0 | 0 | 0 | 0 | 0 | 1 | 0 | 0 | 0 | 584 | 593 | Carbamidomethylation |
| R.TAPAFQFSSC(+57.02)SIR.E | N | 57.10 | 1470.6925 | 13 | 0.3 | 736.3538 | 2 | 12.49 | 10 | F10:3268 | 29102019\_RID\_1313\_NaNaPb\_F9.raw |  | 8.57E3 |  |  |  |  |  |  |  | 3.87E4 | 2 | 0 | 1 | 0 | 0 | 0 | 0 | 0 | 0 | 0 | 1 | 370 | 382 | Carbamidomethylation |
| L.IPPNPDGIM(+15.99)AEPGTK.C | N | 55.45 | 1551.7603 | 15 | 0.5 | 776.8878 | 2 | 11.45 | 7 | F7:2313 | 29102019\_RID\_1313\_NaNaPb\_F6.raw |  |  |  |  |  |  | 9.78E4 |  |  |  | 1 | 0 | 0 | 0 | 0 | 0 | 0 | 1 | 0 | 0 | 0 | 560 | 574 | Oxidation (M) |
| H.IALIGLEIWSNK.D | Y | 51.19 | 1355.7812 | 12 | 0.3 | 678.8981 | 2 | 35.91 | 2 | F2:11989 | 29102019\_RID\_1313\_NaNaPb\_F10.raw |  | 1.04E6 |  |  |  | 1.78E4 |  |  | 1.7E5 |  | 3 | 0 | 1 | 0 | 0 | 0 | 1 | 0 | 0 | 1 | 0 | 250 | 261 |  |
| A.LIGLEIWSNK.D | Y | 49.12 | 1171.6600 | 10 | 0.4 | 586.8375 | 2 | 23.41 | 9 | F9:9081 | 29102019\_RID\_1313\_NaNaPb\_F8.raw |  | 7.51E5 |  |  |  |  |  |  | 3.4E4 |  | 2 | 0 | 1 | 0 | 0 | 0 | 0 | 0 | 0 | 1 | 0 | 252 | 261 |  |
| I.HIALIGLEIWSNK.D | Y | 47.14 | 1492.8402 | 13 | -0.3 | 498.6205 | 3 | 23.25 | 2 | F2:8137 | 29102019\_RID\_1313\_NaNaPb\_F10.raw |  | 3.57E4 |  |  |  |  |  |  |  |  | 1 | 0 | 1 | 0 | 0 | 0 | 0 | 0 | 0 | 0 | 0 | 249 | 261 |  |
| R.NDNAQLLTGIDFNGNTVGR.A | Y | 43.08 | 2017.9817 | 19 | 2.3 | 1010.0005 | 2 | 19.42 | 2 | F2:6962 | 29102019\_RID\_1313\_NaNaPb\_F10.raw |  | 8.79E3 |  |  |  |  |  |  |  |  | 1 | 0 | 1 | 0 | 0 | 0 | 0 | 0 | 0 | 0 | 0 | 291 | 309 |  |
| total 9 peptides |
| --- |

O76013|KRT36\_HUMAN

back to list

  

| Protein Coverage
| Supporting Peptides
|

Protein Coverage:

Supporting Peptides:

| Peptide | Uniq | -10lgP | Mass | Length | ppm | m/z | z | RT | Fraction | Scan | Source File | Area F1 | Area F10 | Area F2 | Area F3 | Area F4 | Area F5 | Area F6 | Area F7 | Area F8 | Area F9 | #Feature | #Feature F1 | #Feature F10 | #Feature F2 | #Feature F3 | #Feature F4 | #Feature F5 | #Feature F6 | #Feature F7 | #Feature F8 | #Feature F9 | Start | End | PTM |
| --- | --- | --- | --- | --- | --- | --- | --- | --- | --- | --- | --- | --- | --- | --- | --- | --- | --- | --- | --- | --- | --- | --- | --- | --- | --- | --- | --- | --- | --- | --- | --- | --- | --- | --- | --- |
| R.TVNALEIELQAQHSMR.N | N | 89.45 | 1838.9309 | 16 | 0.2 | 613.9844 | 3 | 63.38 | 5 | F5:26952 | 29102019\_RID\_1313\_NaNaPb\_F4.raw |  |  |  |  | 0 |  |  |  |  |  | 1 | 0 | 0 | 0 | 0 | 1 | 0 | 0 | 0 | 0 | 0 | 315 | 330 |  |
| R.TVNALEIELQAQHSMRNSLESTLAETEAR.Y | Y | 76.38 | 3240.6045 | 29 | 4.5 | 1081.2136 | 3 | 77.07 | 5 | F5:38602 | 29102019\_RID\_1313\_NaNaPb\_F4.raw |  |  |  |  | 0 |  |  |  |  |  | 1 | 0 | 0 | 0 | 0 | 1 | 0 | 0 | 0 | 0 | 0 | 315 | 343 |  |
| R.TITEEIRDGKVISSREHVQSRPL | Y | 66.49 | 2649.4197 | 23 | 0.3 | 663.3624 | 4 | 45.76 | 5 | F5:12569 | 29102019\_RID\_1313\_NaNaPb\_F4.raw |  |  |  |  | 3.49E7 |  |  |  |  |  | 4 | 0 | 0 | 0 | 0 | 4 | 0 | 0 | 0 | 0 | 0 | 445 | 467 |  |
| K.NHEEEVSVLR.C | Y | 66.03 | 1210.5942 | 10 | -0.9 | 606.3038 | 2 | 39.48 | 5 | F5:7949 | 29102019\_RID\_1313\_NaNaPb\_F4.raw |  |  |  |  | 1.06E6 |  |  |  |  |  | 1 | 0 | 0 | 0 | 0 | 1 | 0 | 0 | 0 | 0 | 0 | 230 | 239 |  |
| K.TIEDFQQKILLTKSENAR.L | Y | 62.93 | 2133.1428 | 18 | 1.2 | 534.2936 | 4 | 55.58 | 5 | F5:20338 | 29102019\_RID\_1313\_NaNaPb\_F4.raw |  |  |  |  | 0 |  |  |  |  |  | 1 | 0 | 0 | 0 | 0 | 1 | 0 | 0 | 0 | 0 | 0 | 146 | 163 |  |
| R.TITEEIRDGKVISSR.E | Y | 59.97 | 1702.9213 | 15 | 0.6 | 568.6480 | 3 | 43.12 | 5 | F5:10697 | 29102019\_RID\_1313\_NaNaPb\_F4.raw |  |  |  |  | 2.42E6 |  |  |  |  |  | 1 | 0 | 0 | 0 | 0 | 1 | 0 | 0 | 0 | 0 | 0 | 445 | 459 |  |
| K.LAADDFR.T | N | 56.98 | 806.3922 | 7 | 0.4 | 404.2036 | 2 | 11.73 | 1 | F1:3258 | 29102019\_RID\_1313\_NaNaPb\_F1.raw | 1E5 | 2.78E5 | 1.15E5 |  |  | 1.11E5 | 5.12E4 |  | 2.99E4 |  | 6 | 1 | 1 | 1 | 0 | 0 | 1 | 1 | 0 | 1 | 0 | 173 | 179 |  |
| R.QLERENAELESR.I | N | 55.71 | 1472.7219 | 12 | 0.4 | 737.3685 | 2 | 36.84 | 5 | F5:5940 | 29102019\_RID\_1313\_NaNaPb\_F4.raw |  |  |  |  | 6.67E5 |  |  |  |  |  | 2 | 0 | 0 | 0 | 0 | 2 | 0 | 0 | 0 | 0 | 0 | 113 | 124 |  |
| R.LVLQIDNAK.L | Y | 51.52 | 1012.5917 | 9 | 0.7 | 507.3035 | 2 | 55.42 | 5 | F5:20123 | 29102019\_RID\_1313\_NaNaPb\_F4.raw |  |  |  |  | 3.02E6 |  |  |  |  |  | 1 | 0 | 0 | 0 | 0 | 1 | 0 | 0 | 0 | 0 | 0 | 164 | 172 |  |
| R.QLVEADINGLRR.I | N | 43.82 | 1382.7629 | 12 | 1.4 | 692.3897 | 2 | 51.19 | 5 | F5:16715 | 29102019\_RID\_1313\_NaNaPb\_F4.raw |  |  |  |  | 3.44E6 |  |  |  |  |  | 1 | 0 | 0 | 0 | 0 | 1 | 0 | 0 | 0 | 0 | 0 | 190 | 201 |  |
| total 10 peptides |
| --- |

#CONTAM#K1H6\_HUMAN|

back to list

  

| Protein Coverage
| Supporting Peptides
|

Protein Coverage:

Supporting Peptides:

| Peptide | Uniq | -10lgP | Mass | Length | ppm | m/z | z | RT | Fraction | Scan | Source File | Area F1 | Area F10 | Area F2 | Area F3 | Area F4 | Area F5 | Area F6 | Area F7 | Area F8 | Area F9 | #Feature | #Feature F1 | #Feature F10 | #Feature F2 | #Feature F3 | #Feature F4 | #Feature F5 | #Feature F6 | #Feature F7 | #Feature F8 | #Feature F9 | Start | End | PTM |
| --- | --- | --- | --- | --- | --- | --- | --- | --- | --- | --- | --- | --- | --- | --- | --- | --- | --- | --- | --- | --- | --- | --- | --- | --- | --- | --- | --- | --- | --- | --- | --- | --- | --- | --- | --- |
| R.TVNALEIELQAQHSMR.N | N | 89.45 | 1838.9309 | 16 | 0.2 | 613.9844 | 3 | 63.38 | 5 | F5:26952 | 29102019\_RID\_1313\_NaNaPb\_F4.raw |  |  |  |  | 0 |  |  |  |  |  | 1 | 0 | 0 | 0 | 0 | 1 | 0 | 0 | 0 | 0 | 0 | 315 | 330 |  |
| R.TVNALEIELQAQHSMRNSLESTLAETEAR.Y | Y | 76.38 | 3240.6045 | 29 | 4.5 | 1081.2136 | 3 | 77.07 | 5 | F5:38602 | 29102019\_RID\_1313\_NaNaPb\_F4.raw |  |  |  |  | 0 |  |  |  |  |  | 1 | 0 | 0 | 0 | 0 | 1 | 0 | 0 | 0 | 0 | 0 | 315 | 343 |  |
| R.TITEEIRDGKVISSREHVQSRPL | Y | 66.49 | 2649.4197 | 23 | 0.3 | 663.3624 | 4 | 45.76 | 5 | F5:12569 | 29102019\_RID\_1313\_NaNaPb\_F4.raw |  |  |  |  | 3.49E7 |  |  |  |  |  | 4 | 0 | 0 | 0 | 0 | 4 | 0 | 0 | 0 | 0 | 0 | 445 | 467 |  |
| K.NHEEEVSVLR.C | Y | 66.03 | 1210.5942 | 10 | -0.9 | 606.3038 | 2 | 39.48 | 5 | F5:7949 | 29102019\_RID\_1313\_NaNaPb\_F4.raw |  |  |  |  | 1.06E6 |  |  |  |  |  | 1 | 0 | 0 | 0 | 0 | 1 | 0 | 0 | 0 | 0 | 0 | 230 | 239 |  |
| K.TIEDFQQKILLTKSENAR.L | Y | 62.93 | 2133.1428 | 18 | 1.2 | 534.2936 | 4 | 55.58 | 5 | F5:20338 | 29102019\_RID\_1313\_NaNaPb\_F4.raw |  |  |  |  | 0 |  |  |  |  |  | 1 | 0 | 0 | 0 | 0 | 1 | 0 | 0 | 0 | 0 | 0 | 146 | 163 |  |
| R.TITEEIRDGKVISSR.E | Y | 59.97 | 1702.9213 | 15 | 0.6 | 568.6480 | 3 | 43.12 | 5 | F5:10697 | 29102019\_RID\_1313\_NaNaPb\_F4.raw |  |  |  |  | 2.42E6 |  |  |  |  |  | 1 | 0 | 0 | 0 | 0 | 1 | 0 | 0 | 0 | 0 | 0 | 445 | 459 |  |
| K.LAADDFR.T | N | 56.98 | 806.3922 | 7 | 0.4 | 404.2036 | 2 | 11.73 | 1 | F1:3258 | 29102019\_RID\_1313\_NaNaPb\_F1.raw | 1E5 | 2.78E5 | 1.15E5 |  |  | 1.11E5 | 5.12E4 |  | 2.99E4 |  | 6 | 1 | 1 | 1 | 0 | 0 | 1 | 1 | 0 | 1 | 0 | 173 | 179 |  |
| R.QLERENAELESR.I | N | 55.71 | 1472.7219 | 12 | 0.4 | 737.3685 | 2 | 36.84 | 5 | F5:5940 | 29102019\_RID\_1313\_NaNaPb\_F4.raw |  |  |  |  | 6.67E5 |  |  |  |  |  | 2 | 0 | 0 | 0 | 0 | 2 | 0 | 0 | 0 | 0 | 0 | 113 | 124 |  |
| R.LVLQIDNAK.L | Y | 51.52 | 1012.5917 | 9 | 0.7 | 507.3035 | 2 | 55.42 | 5 | F5:20123 | 29102019\_RID\_1313\_NaNaPb\_F4.raw |  |  |  |  | 3.02E6 |  |  |  |  |  | 1 | 0 | 0 | 0 | 0 | 1 | 0 | 0 | 0 | 0 | 0 | 164 | 172 |  |
| R.QLVEADINGLRR.I | N | 43.82 | 1382.7629 | 12 | 1.4 | 692.3897 | 2 | 51.19 | 5 | F5:16715 | 29102019\_RID\_1313\_NaNaPb\_F4.raw |  |  |  |  | 3.44E6 |  |  |  |  |  | 1 | 0 | 0 | 0 | 0 | 1 | 0 | 0 | 0 | 0 | 0 | 190 | 201 |  |
| total 10 peptides |
| --- |

Q04695|K1C17\_HUMAN

back to list

  

| Protein Coverage
| Supporting Peptides
|

Protein Coverage:

Supporting Peptides:

| Peptide | Uniq | -10lgP | Mass | Length | ppm | m/z | z | RT | Fraction | Scan | Source File | Area F1 | Area F10 | Area F2 | Area F3 | Area F4 | Area F5 | Area F6 | Area F7 | Area F8 | Area F9 | #Feature | #Feature F1 | #Feature F10 | #Feature F2 | #Feature F3 | #Feature F4 | #Feature F5 | #Feature F6 | #Feature F7 | #Feature F8 | #Feature F9 | Start | End | PTM |
| --- | --- | --- | --- | --- | --- | --- | --- | --- | --- | --- | --- | --- | --- | --- | --- | --- | --- | --- | --- | --- | --- | --- | --- | --- | --- | --- | --- | --- | --- | --- | --- | --- | --- | --- | --- |
| R.EVATNSELVQSGK.S | N | 81.60 | 1360.6833 | 13 | -0.1 | 681.3489 | 2 | 11.36 | 3 | F3:2164 | 29102019\_RID\_1313\_NaNaPb\_F2.raw |  | 3.73E4 | 1.29E5 |  |  |  | 3.28E4 |  |  |  | 3 | 0 | 1 | 1 | 0 | 0 | 0 | 1 | 0 | 0 | 0 | 285 | 297 |  |
| R.VLDELTLAR.A | N | 67.89 | 1028.5865 | 9 | 0.8 | 515.3010 | 2 | 62.60 | 5 | F5:26241 | 29102019\_RID\_1313\_NaNaPb\_F4.raw | 1.32E5 |  |  |  | 8.65E5 | 5.32E5 | 1.7E5 |  |  | 1.39E5 | 5 | 1 | 0 | 0 | 0 | 1 | 1 | 1 | 0 | 0 | 1 | 193 | 201 |  |
| R.LEQEIATYR.R | N | 65.29 | 1121.5717 | 9 | 0.7 | 561.7935 | 2 | 11.51 | 6 | F6:2300 | 29102019\_RID\_1313\_NaNaPb\_F5.raw |  |  | 1.24E5 |  |  | 1.68E5 |  |  |  |  | 2 | 0 | 0 | 1 | 0 | 0 | 1 | 0 | 0 | 0 | 0 | 377 | 385 |  |
| R.LAADDFR.T | N | 56.98 | 806.3922 | 7 | 0.4 | 404.2036 | 2 | 11.73 | 1 | F1:3258 | 29102019\_RID\_1313\_NaNaPb\_F1.raw | 1E5 | 2.78E5 | 1.15E5 |  |  | 1.11E5 | 5.12E4 |  | 2.99E4 |  | 6 | 1 | 1 | 1 | 0 | 0 | 1 | 1 | 0 | 1 | 0 | 164 | 170 |  |
| R.LASYLDK.V | N | 55.97 | 808.4330 | 7 | -0.9 | 405.2234 | 2 | 11.66 | 1 | F1:3180 | 29102019\_RID\_1313\_NaNaPb\_F1.raw | 9.27E4 |  | 3.47E5 |  |  | 0 | 5.32E4 |  |  |  | 4 | 1 | 0 | 1 | 0 | 0 | 1 | 1 | 0 | 0 | 0 | 95 | 101 |  |
| N.ILLQIDNAR.L | N | 52.01 | 1054.6135 | 9 | 0.3 | 528.3142 | 2 | 12.45 | 6 | F6:3131 | 29102019\_RID\_1313\_NaNaPb\_F5.raw |  | 3.86E5 |  |  |  | 3.61E5 |  |  | 2.29E4 |  | 3 | 0 | 1 | 0 | 0 | 0 | 1 | 0 | 0 | 1 | 0 | 155 | 163 |  |
| L.IGSVEEQLAQLR.C | N | 49.26 | 1341.7252 | 12 | 0.3 | 671.8701 | 2 | 16.80 | 1 | F1:5418 | 29102019\_RID\_1313\_NaNaPb\_F1.raw | 2.93E4 |  |  |  |  | 8.79E4 |  |  |  |  | 2 | 1 | 0 | 0 | 0 | 0 | 1 | 0 | 0 | 0 | 0 | 346 | 357 |  |
| K.DAEDWFFSK.T | N | 46.28 | 1143.4873 | 9 | 0.3 | 572.7511 | 2 | 24.60 | 10 | F10:6820 | 29102019\_RID\_1313\_NaNaPb\_F9.raw |  |  |  |  |  |  |  |  |  | 1.47E4 | 1 | 0 | 0 | 0 | 0 | 0 | 0 | 0 | 0 | 0 | 1 | 270 | 278 |  |
| D.NANILLQIDNAR.L | N | 43.60 | 1353.7365 | 12 | 0.5 | 677.8759 | 2 | 18.80 | 1 | F1:5928 | 29102019\_RID\_1313\_NaNaPb\_F1.raw | 1.63E4 |  |  |  |  |  |  |  |  |  | 1 | 1 | 0 | 0 | 0 | 0 | 0 | 0 | 0 | 0 | 0 | 152 | 163 |  |
| R.LLEGEDAHLTQYK.K | N | 43.27 | 1515.7568 | 13 | 0.4 | 506.2598 | 3 | 11.70 | 3 | F3:2526 | 29102019\_RID\_1313\_NaNaPb\_F2.raw |  |  | 1.05E4 |  |  |  |  |  |  |  | 1 | 0 | 0 | 1 | 0 | 0 | 0 | 0 | 0 | 0 | 0 | 387 | 399 |  |
| L.TATVDNANILLQIDNAR.L | Y | 42.97 | 1840.9642 | 17 | 0.7 | 921.4901 | 2 | 21.24 | 2 | F2:7560 | 29102019\_RID\_1313\_NaNaPb\_F10.raw |  | 1.97E4 |  |  |  |  |  |  |  |  | 1 | 0 | 1 | 0 | 0 | 0 | 0 | 0 | 0 | 0 | 0 | 147 | 163 |  |
| total 11 peptides |
| --- |

P11517|HBB2\_RAT

back to list

  

| Protein Coverage
| Supporting Peptides
|

Protein Coverage:

Supporting Peptides:

| Peptide | Uniq | -10lgP | Mass | Length | ppm | m/z | z | RT | Fraction | Scan | Source File | Area F1 | Area F10 | Area F2 | Area F3 | Area F4 | Area F5 | Area F6 | Area F7 | Area F8 | Area F9 | #Feature | #Feature F1 | #Feature F10 | #Feature F2 | #Feature F3 | #Feature F4 | #Feature F5 | #Feature F6 | #Feature F7 | #Feature F8 | #Feature F9 | Start | End | PTM |
| --- | --- | --- | --- | --- | --- | --- | --- | --- | --- | --- | --- | --- | --- | --- | --- | --- | --- | --- | --- | --- | --- | --- | --- | --- | --- | --- | --- | --- | --- | --- | --- | --- | --- | --- | --- |
| K.VVAGVASALAHK.Y | N | 74.76 | 1121.6556 | 12 | 0.8 | 561.8356 | 2 | 11.37 | 8 | F8:1754 | 29102019\_RID\_1313\_NaNaPb\_F7.raw |  |  |  |  |  |  |  | 3.88E4 |  |  | 1 | 0 | 0 | 0 | 0 | 0 | 0 | 0 | 1 | 0 | 0 | 134 | 145 |  |
| K.KVINAFNDGLK.H | N | 74.72 | 1217.6768 | 11 | -0.4 | 406.8994 | 3 | 11.46 | 8 | F8:1803 | 29102019\_RID\_1313\_NaNaPb\_F7.raw | 1.74E4 |  |  |  |  |  |  | 1.15E4 |  |  | 2 | 1 | 0 | 0 | 0 | 0 | 0 | 0 | 1 | 0 | 0 | 67 | 77 |  |
| K.VINAFNDGLK.H | N | 69.92 | 1089.5818 | 10 | 0.6 | 545.7985 | 2 | 12.21 | 4 | F4:2985 | 29102019\_RID\_1313\_NaNaPb\_F3.raw | 3.14E3 |  | 1.48E5 | 7.73E4 |  |  | 0 |  |  |  | 4 | 1 | 0 | 1 | 1 | 0 | 0 | 1 | 0 | 0 | 0 | 68 | 77 |  |
| R.LLVVYPWTQR.Y | N | 66.77 | 1273.7183 | 10 | 1.0 | 637.8671 | 2 | 27.07 | 1 | F1:8363 | 29102019\_RID\_1313\_NaNaPb\_F1.raw | 5.83E6 |  | 1.67E6 | 7.01E5 |  | 2.18E5 | 1.48E5 | 1.91E5 |  |  | 52 | 33 | 0 | 8 | 6 | 0 | 2 | 1 | 2 | 0 | 0 | 32 | 41 |  |
| K.LHVDPENFR.L | N | 64.94 | 1125.5566 | 9 | -0.7 | 376.1926 | 3 | 11.48 | 8 | F8:1809 | 29102019\_RID\_1313\_NaNaPb\_F7.raw | 9.63E4 |  |  |  |  |  |  | 3.19E4 |  |  | 4 | 2 | 0 | 0 | 0 | 0 | 0 | 0 | 2 | 0 | 0 | 97 | 105 |  |
| K.EFTPC(+57.02)AQAAFQK.V | N | 64.83 | 1396.6445 | 12 | 0.2 | 699.3297 | 2 | 12.13 | 1 | F1:3625 | 29102019\_RID\_1313\_NaNaPb\_F1.raw | 5.6E4 |  |  |  |  |  |  | 1.96E4 |  |  | 2 | 1 | 0 | 0 | 0 | 0 | 0 | 0 | 1 | 0 | 0 | 122 | 133 | Carbamidomethylation |
| K.ATVSGLWGK.V | Y | 61.70 | 917.4971 | 9 | 0.6 | 459.7561 | 2 | 12.90 | 1 | F1:4154 | 29102019\_RID\_1313\_NaNaPb\_F1.raw | 1.25E4 |  |  |  |  |  |  | 3.53E3 |  |  | 2 | 1 | 0 | 0 | 0 | 0 | 0 | 0 | 1 | 0 | 0 | 10 | 18 |  |
| V.VAGVASALAHK.Y | N | 59.76 | 1022.5872 | 11 | 0.3 | 512.3010 | 2 | 11.37 | 8 | F8:1759 | 29102019\_RID\_1313\_NaNaPb\_F7.raw |  |  |  |  |  |  |  | 1.84E4 |  |  | 1 | 0 | 0 | 0 | 0 | 0 | 0 | 0 | 1 | 0 | 0 | 135 | 145 |  |
| M.VHLTDAEK.A | N | 59.37 | 911.4712 | 8 | 0.1 | 456.7429 | 2 | 10.69 | 8 | F8:1577 | 29102019\_RID\_1313\_NaNaPb\_F7.raw |  |  | 4.27E4 | 8.25E3 |  |  |  | 4.64E4 |  | 2.36E4 | 4 | 0 | 0 | 1 | 1 | 0 | 0 | 0 | 1 | 0 | 1 | 2 | 9 |  |
| K.VVAGVASALAHKYH | N | 51.55 | 1421.7778 | 14 | -0.3 | 474.9331 | 3 | 11.41 | 8 | F8:1780 | 29102019\_RID\_1313\_NaNaPb\_F7.raw |  |  |  |  |  |  |  | 1.14E4 |  |  | 1 | 0 | 0 | 0 | 0 | 0 | 0 | 0 | 1 | 0 | 0 | 134 | 147 |  |
| K.VVAGVASALAH.K | N | 43.43 | 993.5607 | 11 | 0.2 | 497.7877 | 2 | 11.78 | 8 | F8:1933 | 29102019\_RID\_1313\_NaNaPb\_F7.raw |  |  |  |  |  |  |  | 0 |  |  | 1 | 0 | 0 | 0 | 0 | 0 | 0 | 0 | 1 | 0 | 0 | 134 | 144 |  |
| V.AGVASALAHK.Y | N | 43.15 | 923.5188 | 10 | -0.2 | 462.7666 | 2 | 11.37 | 8 | F8:1761 | 29102019\_RID\_1313\_NaNaPb\_F7.raw |  |  |  |  |  |  |  | 4.55E4 |  |  | 1 | 0 | 0 | 0 | 0 | 0 | 0 | 0 | 1 | 0 | 0 | 136 | 145 |  |
| total 12 peptides |
| --- |

P14923|PLAK\_HUMAN

back to list

  

| Protein Coverage
| Supporting Peptides
|

Protein Coverage:

Supporting Peptides:

| Peptide | Uniq | -10lgP | Mass | Length | ppm | m/z | z | RT | Fraction | Scan | Source File | Area F1 | Area F10 | Area F2 | Area F3 | Area F4 | Area F5 | Area F6 | Area F7 | Area F8 | Area F9 | #Feature | #Feature F1 | #Feature F10 | #Feature F2 | #Feature F3 | #Feature F4 | #Feature F5 | #Feature F6 | #Feature F7 | #Feature F8 | #Feature F9 | Start | End | PTM |
| --- | --- | --- | --- | --- | --- | --- | --- | --- | --- | --- | --- | --- | --- | --- | --- | --- | --- | --- | --- | --- | --- | --- | --- | --- | --- | --- | --- | --- | --- | --- | --- | --- | --- | --- | --- |
| K.LLNDEDPVVVTK.A | Y | 77.58 | 1340.7188 | 12 | 0.7 | 671.3671 | 2 | 12.63 | 1 | F1:4001 | 29102019\_RID\_1313\_NaNaPb\_F1.raw | 1.18E4 |  | 3.09E4 |  |  |  |  |  |  |  | 2 | 1 | 0 | 1 | 0 | 0 | 0 | 0 | 0 | 0 | 0 | 150 | 161 |  |
| K.SAIVHLINYQDDAELATR.A | Y | 70.76 | 2028.0276 | 18 | 0.9 | 677.0171 | 3 | 16.43 | 1 | F1:5333 | 29102019\_RID\_1313\_NaNaPb\_F1.raw | 8.77E3 |  | 1.92E4 |  |  |  |  |  |  |  | 2 | 1 | 0 | 1 | 0 | 0 | 0 | 0 | 0 | 0 | 0 | 125 | 142 |  |
| R.VAAGVLC(+57.02)ELAQDK.E | Y | 68.24 | 1372.7020 | 13 | 1.0 | 687.3590 | 2 | 14.67 | 3 | F3:4064 | 29102019\_RID\_1313\_NaNaPb\_F2.raw |  |  | 4.41E4 |  |  |  |  |  |  |  | 1 | 0 | 0 | 1 | 0 | 0 | 0 | 0 | 0 | 0 | 0 | 603 | 615 | Carbamidomethylation |
| K.NKTLVTQNSGVEALIHAILR.A | Y | 63.35 | 2176.2327 | 20 | 1.7 | 545.0663 | 4 | 80.87 | 5 | F5:41821 | 29102019\_RID\_1313\_NaNaPb\_F4.raw |  |  |  |  | 0 |  |  |  |  |  | 1 | 0 | 0 | 0 | 0 | 1 | 0 | 0 | 0 | 0 | 0 | 425 | 444 |  |
| R.NLALC(+57.02)PANHAPLQEAAVIPR.L | Y | 56.04 | 2154.1367 | 20 | 0.4 | 719.0531 | 3 | 14.05 | 3 | F3:3886 | 29102019\_RID\_1313\_NaNaPb\_F2.raw | 1.87E4 |  | 3.99E4 |  |  |  |  |  |  |  | 2 | 1 | 0 | 1 | 0 | 0 | 0 | 0 | 0 | 0 | 0 | 507 | 526 | Carbamidomethylation |
| R.VSVELTNSLFK.H | Y | 54.81 | 1235.6761 | 11 | 0.1 | 618.8454 | 2 | 19.11 | 3 | F3:5062 | 29102019\_RID\_1313\_NaNaPb\_F2.raw |  |  | 1.86E4 |  |  | 4.11E4 |  |  |  |  | 2 | 0 | 0 | 1 | 0 | 0 | 1 | 0 | 0 | 0 | 0 | 664 | 674 |  |
| R.LAEPSQLLK.S | Y | 50.58 | 997.5807 | 9 | 1.0 | 499.7982 | 2 | 55.51 | 5 | F5:20271 | 29102019\_RID\_1313\_NaNaPb\_F4.raw |  |  |  |  | 0 |  |  |  |  |  | 1 | 0 | 0 | 0 | 0 | 1 | 0 | 0 | 0 | 0 | 0 | 116 | 124 |  |
| R.ALPELTKLLNDEDPVVVTK.A | Y | 49.93 | 2093.1619 | 19 | 1.8 | 698.7291 | 3 | 75.98 | 5 | F5:37858 | 29102019\_RID\_1313\_NaNaPb\_F4.raw |  |  |  |  | 4.31E5 |  |  |  |  |  | 1 | 0 | 0 | 0 | 0 | 1 | 0 | 0 | 0 | 0 | 0 | 143 | 161 |  |
| R.NEGTATYAAAVLFR.I | Y | 46.85 | 1482.7467 | 14 | -3.6 | 742.3779 | 2 | 19.01 | 6 | F6:7169 | 29102019\_RID\_1313\_NaNaPb\_F5.raw |  |  |  |  |  | 5.05E4 |  |  |  |  | 1 | 0 | 0 | 0 | 0 | 0 | 1 | 0 | 0 | 0 | 0 | 638 | 651 |  |
| K.LLNQPNQWPLVK.A | Y | 46.31 | 1448.8140 | 12 | 1.2 | 725.4152 | 2 | 16.40 | 6 | F6:5632 | 29102019\_RID\_1313\_NaNaPb\_F5.raw |  |  |  |  |  | 7.18E4 |  |  |  |  | 1 | 0 | 0 | 0 | 0 | 0 | 1 | 0 | 0 | 0 | 0 | 488 | 499 |  |
| R.TMQNTSDLDTAR.C | Y | 44.33 | 1351.6038 | 12 | 0.3 | 676.8093 | 2 | 39.32 | 5 | F5:7850 | 29102019\_RID\_1313\_NaNaPb\_F4.raw |  |  |  |  | 6.99E4 |  |  |  |  |  | 1 | 0 | 0 | 0 | 0 | 1 | 0 | 0 | 0 | 0 | 0 | 192 | 203 |  |
| K.AAMIVNQLSKKEASRR.A | Y | 43.20 | 1800.9991 | 16 | 0.2 | 451.2571 | 4 | 36.87 | 5 | F5:5954 | 29102019\_RID\_1313\_NaNaPb\_F4.raw |  |  |  |  | 4.76E5 |  |  |  |  |  | 1 | 0 | 0 | 0 | 0 | 1 | 0 | 0 | 0 | 0 | 0 | 162 | 177 |  |
| total 12 peptides |
| --- |

Q8SPJ1|PLAK\_BOVIN

back to list

  

| Protein Coverage
| Supporting Peptides
|

Protein Coverage:

Supporting Peptides:

| Peptide | Uniq | -10lgP | Mass | Length | ppm | m/z | z | RT | Fraction | Scan | Source File | Area F1 | Area F10 | Area F2 | Area F3 | Area F4 | Area F5 | Area F6 | Area F7 | Area F8 | Area F9 | #Feature | #Feature F1 | #Feature F10 | #Feature F2 | #Feature F3 | #Feature F4 | #Feature F5 | #Feature F6 | #Feature F7 | #Feature F8 | #Feature F9 | Start | End | PTM |
| --- | --- | --- | --- | --- | --- | --- | --- | --- | --- | --- | --- | --- | --- | --- | --- | --- | --- | --- | --- | --- | --- | --- | --- | --- | --- | --- | --- | --- | --- | --- | --- | --- | --- | --- | --- |
| K.LLNDEDPVVVTK.A | Y | 77.58 | 1340.7188 | 12 | 0.7 | 671.3671 | 2 | 12.63 | 1 | F1:4001 | 29102019\_RID\_1313\_NaNaPb\_F1.raw | 1.18E4 |  | 3.09E4 |  |  |  |  |  |  |  | 2 | 1 | 0 | 1 | 0 | 0 | 0 | 0 | 0 | 0 | 0 | 150 | 161 |  |
| K.SAIVHLINYQDDAELATR.A | Y | 70.76 | 2028.0276 | 18 | 0.9 | 677.0171 | 3 | 16.43 | 1 | F1:5333 | 29102019\_RID\_1313\_NaNaPb\_F1.raw | 8.77E3 |  | 1.92E4 |  |  |  |  |  |  |  | 2 | 1 | 0 | 1 | 0 | 0 | 0 | 0 | 0 | 0 | 0 | 125 | 142 |  |
| R.VAAGVLC(+57.02)ELAQDK.E | Y | 68.24 | 1372.7020 | 13 | 1.0 | 687.3590 | 2 | 14.67 | 3 | F3:4064 | 29102019\_RID\_1313\_NaNaPb\_F2.raw |  |  | 4.41E4 |  |  |  |  |  |  |  | 1 | 0 | 0 | 1 | 0 | 0 | 0 | 0 | 0 | 0 | 0 | 603 | 615 | Carbamidomethylation |
| K.NKTLVTQNSGVEALIHAILR.A | Y | 63.35 | 2176.2327 | 20 | 1.7 | 545.0663 | 4 | 80.87 | 5 | F5:41821 | 29102019\_RID\_1313\_NaNaPb\_F4.raw |  |  |  |  | 0 |  |  |  |  |  | 1 | 0 | 0 | 0 | 0 | 1 | 0 | 0 | 0 | 0 | 0 | 425 | 444 |  |
| R.NLALC(+57.02)PANHAPLQEAAVIPR.L | Y | 56.04 | 2154.1367 | 20 | 0.4 | 719.0531 | 3 | 14.05 | 3 | F3:3886 | 29102019\_RID\_1313\_NaNaPb\_F2.raw | 1.87E4 |  | 3.99E4 |  |  |  |  |  |  |  | 2 | 1 | 0 | 1 | 0 | 0 | 0 | 0 | 0 | 0 | 0 | 507 | 526 | Carbamidomethylation |
| R.VSVELTNSLFK.H | Y | 54.81 | 1235.6761 | 11 | 0.1 | 618.8454 | 2 | 19.11 | 3 | F3:5062 | 29102019\_RID\_1313\_NaNaPb\_F2.raw |  |  | 1.86E4 |  |  | 4.11E4 |  |  |  |  | 2 | 0 | 0 | 1 | 0 | 0 | 1 | 0 | 0 | 0 | 0 | 664 | 674 |  |
| R.LAEPSQLLK.S | Y | 50.58 | 997.5807 | 9 | 1.0 | 499.7982 | 2 | 55.51 | 5 | F5:20271 | 29102019\_RID\_1313\_NaNaPb\_F4.raw |  |  |  |  | 0 |  |  |  |  |  | 1 | 0 | 0 | 0 | 0 | 1 | 0 | 0 | 0 | 0 | 0 | 116 | 124 |  |
| R.ALPELTKLLNDEDPVVVTK.A | Y | 49.93 | 2093.1619 | 19 | 1.8 | 698.7291 | 3 | 75.98 | 5 | F5:37858 | 29102019\_RID\_1313\_NaNaPb\_F4.raw |  |  |  |  | 4.31E5 |  |  |  |  |  | 1 | 0 | 0 | 0 | 0 | 1 | 0 | 0 | 0 | 0 | 0 | 143 | 161 |  |
| R.NEGTATYAAAVLFR.I | Y | 46.85 | 1482.7467 | 14 | -3.6 | 742.3779 | 2 | 19.01 | 6 | F6:7169 | 29102019\_RID\_1313\_NaNaPb\_F5.raw |  |  |  |  |  | 5.05E4 |  |  |  |  | 1 | 0 | 0 | 0 | 0 | 0 | 1 | 0 | 0 | 0 | 0 | 638 | 651 |  |
| K.LLNQPNQWPLVK.A | Y | 46.31 | 1448.8140 | 12 | 1.2 | 725.4152 | 2 | 16.40 | 6 | F6:5632 | 29102019\_RID\_1313\_NaNaPb\_F5.raw |  |  |  |  |  | 7.18E4 |  |  |  |  | 1 | 0 | 0 | 0 | 0 | 0 | 1 | 0 | 0 | 0 | 0 | 488 | 499 |  |
| R.TMQNTSDLDTAR.C | Y | 44.33 | 1351.6038 | 12 | 0.3 | 676.8093 | 2 | 39.32 | 5 | F5:7850 | 29102019\_RID\_1313\_NaNaPb\_F4.raw |  |  |  |  | 6.99E4 |  |  |  |  |  | 1 | 0 | 0 | 0 | 0 | 1 | 0 | 0 | 0 | 0 | 0 | 192 | 203 |  |
| K.AAMIVNQLSKKEASRR.A | Y | 43.20 | 1800.9991 | 16 | 0.2 | 451.2571 | 4 | 36.87 | 5 | F5:5954 | 29102019\_RID\_1313\_NaNaPb\_F4.raw |  |  |  |  | 4.76E5 |  |  |  |  |  | 1 | 0 | 0 | 0 | 0 | 1 | 0 | 0 | 0 | 0 | 0 | 162 | 177 |  |
| total 12 peptides |
| --- |

Q6P0K8|PLAK\_RAT

back to list

  

| Protein Coverage
| Supporting Peptides
|

Protein Coverage:

Supporting Peptides:

| Peptide | Uniq | -10lgP | Mass | Length | ppm | m/z | z | RT | Fraction | Scan | Source File | Area F1 | Area F10 | Area F2 | Area F3 | Area F4 | Area F5 | Area F6 | Area F7 | Area F8 | Area F9 | #Feature | #Feature F1 | #Feature F10 | #Feature F2 | #Feature F3 | #Feature F4 | #Feature F5 | #Feature F6 | #Feature F7 | #Feature F8 | #Feature F9 | Start | End | PTM |
| --- | --- | --- | --- | --- | --- | --- | --- | --- | --- | --- | --- | --- | --- | --- | --- | --- | --- | --- | --- | --- | --- | --- | --- | --- | --- | --- | --- | --- | --- | --- | --- | --- | --- | --- | --- |
| K.LLNDEDPVVVTK.A | Y | 77.58 | 1340.7188 | 12 | 0.7 | 671.3671 | 2 | 12.63 | 1 | F1:4001 | 29102019\_RID\_1313\_NaNaPb\_F1.raw | 1.18E4 |  | 3.09E4 |  |  |  |  |  |  |  | 2 | 1 | 0 | 1 | 0 | 0 | 0 | 0 | 0 | 0 | 0 | 150 | 161 |  |
| K.SAIVHLINYQDDAELATR.A | Y | 70.76 | 2028.0276 | 18 | 0.9 | 677.0171 | 3 | 16.43 | 1 | F1:5333 | 29102019\_RID\_1313\_NaNaPb\_F1.raw | 8.77E3 |  | 1.92E4 |  |  |  |  |  |  |  | 2 | 1 | 0 | 1 | 0 | 0 | 0 | 0 | 0 | 0 | 0 | 125 | 142 |  |
| R.VAAGVLC(+57.02)ELAQDK.E | Y | 68.24 | 1372.7020 | 13 | 1.0 | 687.3590 | 2 | 14.67 | 3 | F3:4064 | 29102019\_RID\_1313\_NaNaPb\_F2.raw |  |  | 4.41E4 |  |  |  |  |  |  |  | 1 | 0 | 0 | 1 | 0 | 0 | 0 | 0 | 0 | 0 | 0 | 603 | 615 | Carbamidomethylation |
| K.NKTLVTQNSGVEALIHAILR.A | Y | 63.35 | 2176.2327 | 20 | 1.7 | 545.0663 | 4 | 80.87 | 5 | F5:41821 | 29102019\_RID\_1313\_NaNaPb\_F4.raw |  |  |  |  | 0 |  |  |  |  |  | 1 | 0 | 0 | 0 | 0 | 1 | 0 | 0 | 0 | 0 | 0 | 425 | 444 |  |
| R.NLALC(+57.02)PANHAPLQEAAVIPR.L | Y | 56.04 | 2154.1367 | 20 | 0.4 | 719.0531 | 3 | 14.05 | 3 | F3:3886 | 29102019\_RID\_1313\_NaNaPb\_F2.raw | 1.87E4 |  | 3.99E4 |  |  |  |  |  |  |  | 2 | 1 | 0 | 1 | 0 | 0 | 0 | 0 | 0 | 0 | 0 | 507 | 526 | Carbamidomethylation |
| R.VSVELTNSLFK.H | Y | 54.81 | 1235.6761 | 11 | 0.1 | 618.8454 | 2 | 19.11 | 3 | F3:5062 | 29102019\_RID\_1313\_NaNaPb\_F2.raw |  |  | 1.86E4 |  |  | 4.11E4 |  |  |  |  | 2 | 0 | 0 | 1 | 0 | 0 | 1 | 0 | 0 | 0 | 0 | 664 | 674 |  |
| R.LAEPSQLLK.S | Y | 50.58 | 997.5807 | 9 | 1.0 | 499.7982 | 2 | 55.51 | 5 | F5:20271 | 29102019\_RID\_1313\_NaNaPb\_F4.raw |  |  |  |  | 0 |  |  |  |  |  | 1 | 0 | 0 | 0 | 0 | 1 | 0 | 0 | 0 | 0 | 0 | 116 | 124 |  |
| R.ALPELTKLLNDEDPVVVTK.A | Y | 49.93 | 2093.1619 | 19 | 1.8 | 698.7291 | 3 | 75.98 | 5 | F5:37858 | 29102019\_RID\_1313\_NaNaPb\_F4.raw |  |  |  |  | 4.31E5 |  |  |  |  |  | 1 | 0 | 0 | 0 | 0 | 1 | 0 | 0 | 0 | 0 | 0 | 143 | 161 |  |
| R.NEGTATYAAAVLFR.I | Y | 46.85 | 1482.7467 | 14 | -3.6 | 742.3779 | 2 | 19.01 | 6 | F6:7169 | 29102019\_RID\_1313\_NaNaPb\_F5.raw |  |  |  |  |  | 5.05E4 |  |  |  |  | 1 | 0 | 0 | 0 | 0 | 0 | 1 | 0 | 0 | 0 | 0 | 638 | 651 |  |
| K.LLNQPNQWPLVK.A | Y | 46.31 | 1448.8140 | 12 | 1.2 | 725.4152 | 2 | 16.40 | 6 | F6:5632 | 29102019\_RID\_1313\_NaNaPb\_F5.raw |  |  |  |  |  | 7.18E4 |  |  |  |  | 1 | 0 | 0 | 0 | 0 | 0 | 1 | 0 | 0 | 0 | 0 | 488 | 499 |  |
| R.TMQNTSDLDTAR.C | Y | 44.33 | 1351.6038 | 12 | 0.3 | 676.8093 | 2 | 39.32 | 5 | F5:7850 | 29102019\_RID\_1313\_NaNaPb\_F4.raw |  |  |  |  | 6.99E4 |  |  |  |  |  | 1 | 0 | 0 | 0 | 0 | 1 | 0 | 0 | 0 | 0 | 0 | 192 | 203 |  |
| K.AAMIVNQLSKKEASRR.A | Y | 43.20 | 1800.9991 | 16 | 0.2 | 451.2571 | 4 | 36.87 | 5 | F5:5954 | 29102019\_RID\_1313\_NaNaPb\_F4.raw |  |  |  |  | 4.76E5 |  |  |  |  |  | 1 | 0 | 0 | 0 | 0 | 1 | 0 | 0 | 0 | 0 | 0 | 162 | 177 |  |
| total 12 peptides |
| --- |

Q02257|PLAK\_MOUSE

back to list

  

| Protein Coverage
| Supporting Peptides
|

Protein Coverage:

Supporting Peptides:

| Peptide | Uniq | -10lgP | Mass | Length | ppm | m/z | z | RT | Fraction | Scan | Source File | Area F1 | Area F10 | Area F2 | Area F3 | Area F4 | Area F5 | Area F6 | Area F7 | Area F8 | Area F9 | #Feature | #Feature F1 | #Feature F10 | #Feature F2 | #Feature F3 | #Feature F4 | #Feature F5 | #Feature F6 | #Feature F7 | #Feature F8 | #Feature F9 | Start | End | PTM |
| --- | --- | --- | --- | --- | --- | --- | --- | --- | --- | --- | --- | --- | --- | --- | --- | --- | --- | --- | --- | --- | --- | --- | --- | --- | --- | --- | --- | --- | --- | --- | --- | --- | --- | --- | --- |
| K.LLNDEDPVVVTK.A | Y | 77.58 | 1340.7188 | 12 | 0.7 | 671.3671 | 2 | 12.63 | 1 | F1:4001 | 29102019\_RID\_1313\_NaNaPb\_F1.raw | 1.18E4 |  | 3.09E4 |  |  |  |  |  |  |  | 2 | 1 | 0 | 1 | 0 | 0 | 0 | 0 | 0 | 0 | 0 | 150 | 161 |  |
| K.SAIVHLINYQDDAELATR.A | Y | 70.76 | 2028.0276 | 18 | 0.9 | 677.0171 | 3 | 16.43 | 1 | F1:5333 | 29102019\_RID\_1313\_NaNaPb\_F1.raw | 8.77E3 |  | 1.92E4 |  |  |  |  |  |  |  | 2 | 1 | 0 | 1 | 0 | 0 | 0 | 0 | 0 | 0 | 0 | 125 | 142 |  |
| R.VAAGVLC(+57.02)ELAQDK.E | Y | 68.24 | 1372.7020 | 13 | 1.0 | 687.3590 | 2 | 14.67 | 3 | F3:4064 | 29102019\_RID\_1313\_NaNaPb\_F2.raw |  |  | 4.41E4 |  |  |  |  |  |  |  | 1 | 0 | 0 | 1 | 0 | 0 | 0 | 0 | 0 | 0 | 0 | 603 | 615 | Carbamidomethylation |
| K.NKTLVTQNSGVEALIHAILR.A | Y | 63.35 | 2176.2327 | 20 | 1.7 | 545.0663 | 4 | 80.87 | 5 | F5:41821 | 29102019\_RID\_1313\_NaNaPb\_F4.raw |  |  |  |  | 0 |  |  |  |  |  | 1 | 0 | 0 | 0 | 0 | 1 | 0 | 0 | 0 | 0 | 0 | 425 | 444 |  |
| R.NLALC(+57.02)PANHAPLQEAAVIPR.L | Y | 56.04 | 2154.1367 | 20 | 0.4 | 719.0531 | 3 | 14.05 | 3 | F3:3886 | 29102019\_RID\_1313\_NaNaPb\_F2.raw | 1.87E4 |  | 3.99E4 |  |  |  |  |  |  |  | 2 | 1 | 0 | 1 | 0 | 0 | 0 | 0 | 0 | 0 | 0 | 507 | 526 | Carbamidomethylation |
| R.VSVELTNSLFK.H | Y | 54.81 | 1235.6761 | 11 | 0.1 | 618.8454 | 2 | 19.11 | 3 | F3:5062 | 29102019\_RID\_1313\_NaNaPb\_F2.raw |  |  | 1.86E4 |  |  | 4.11E4 |  |  |  |  | 2 | 0 | 0 | 1 | 0 | 0 | 1 | 0 | 0 | 0 | 0 | 664 | 674 |  |
| R.LAEPSQLLK.S | Y | 50.58 | 997.5807 | 9 | 1.0 | 499.7982 | 2 | 55.51 | 5 | F5:20271 | 29102019\_RID\_1313\_NaNaPb\_F4.raw |  |  |  |  | 0 |  |  |  |  |  | 1 | 0 | 0 | 0 | 0 | 1 | 0 | 0 | 0 | 0 | 0 | 116 | 124 |  |
| R.ALPELTKLLNDEDPVVVTK.A | Y | 49.93 | 2093.1619 | 19 | 1.8 | 698.7291 | 3 | 75.98 | 5 | F5:37858 | 29102019\_RID\_1313\_NaNaPb\_F4.raw |  |  |  |  | 4.31E5 |  |  |  |  |  | 1 | 0 | 0 | 0 | 0 | 1 | 0 | 0 | 0 | 0 | 0 | 143 | 161 |  |
| R.NEGTATYAAAVLFR.I | Y | 46.85 | 1482.7467 | 14 | -3.6 | 742.3779 | 2 | 19.01 | 6 | F6:7169 | 29102019\_RID\_1313\_NaNaPb\_F5.raw |  |  |  |  |  | 5.05E4 |  |  |  |  | 1 | 0 | 0 | 0 | 0 | 0 | 1 | 0 | 0 | 0 | 0 | 638 | 651 |  |
| K.LLNQPNQWPLVK.A | Y | 46.31 | 1448.8140 | 12 | 1.2 | 725.4152 | 2 | 16.40 | 6 | F6:5632 | 29102019\_RID\_1313\_NaNaPb\_F5.raw |  |  |  |  |  | 7.18E4 |  |  |  |  | 1 | 0 | 0 | 0 | 0 | 0 | 1 | 0 | 0 | 0 | 0 | 488 | 499 |  |
| R.TMQNTSDLDTAR.C | Y | 44.33 | 1351.6038 | 12 | 0.3 | 676.8093 | 2 | 39.32 | 5 | F5:7850 | 29102019\_RID\_1313\_NaNaPb\_F4.raw |  |  |  |  | 6.99E4 |  |  |  |  |  | 1 | 0 | 0 | 0 | 0 | 1 | 0 | 0 | 0 | 0 | 0 | 192 | 203 |  |
| K.AAMIVNQLSKKEASRR.A | Y | 43.20 | 1800.9991 | 16 | 0.2 | 451.2571 | 4 | 36.87 | 5 | F5:5954 | 29102019\_RID\_1313\_NaNaPb\_F4.raw |  |  |  |  | 4.76E5 |  |  |  |  |  | 1 | 0 | 0 | 0 | 0 | 1 | 0 | 0 | 0 | 0 | 0 | 162 | 177 |  |
| total 12 peptides |
| --- |

P01440|3SA2\_NAJNA

back to list

  

| Protein Coverage
| Supporting Peptides
|

Protein Coverage:

Supporting Peptides:

| Peptide | Uniq | -10lgP | Mass | Length | ppm | m/z | z | RT | Fraction | Scan | Source File | Area F1 | Area F10 | Area F2 | Area F3 | Area F4 | Area F5 | Area F6 | Area F7 | Area F8 | Area F9 | #Feature | #Feature F1 | #Feature F10 | #Feature F2 | #Feature F3 | #Feature F4 | #Feature F5 | #Feature F6 | #Feature F7 | #Feature F8 | #Feature F9 | Start | End | PTM |
| --- | --- | --- | --- | --- | --- | --- | --- | --- | --- | --- | --- | --- | --- | --- | --- | --- | --- | --- | --- | --- | --- | --- | --- | --- | --- | --- | --- | --- | --- | --- | --- | --- | --- | --- | --- |
| R.GC(+57.02)IDVC(+57.02)PKSSLVLKYVC(+57.02)C(+57.02)NTDRC(+57.02)N | Y | 82.87 | 2917.3013 | 24 | 0.7 | 730.3331 | 4 | 12.14 | 2 | F2:3022 | 29102019\_RID\_1313\_NaNaPb\_F10.raw |  | 1.11E6 |  |  |  |  |  |  |  |  | 1 | 0 | 1 | 0 | 0 | 0 | 0 | 0 | 0 | 0 | 0 | 37 | 60 | Carbamidomethylation |
| K.LVPLFYKTC(+57.02)PAGK.N | N | 74.44 | 1492.8112 | 13 | 0.4 | 498.6112 | 3 | 11.89 | 2 | F2:2800 | 29102019\_RID\_1313\_NaNaPb\_F10.raw |  | 7.41E5 |  |  |  |  |  |  |  |  | 2 | 0 | 2 | 0 | 0 | 0 | 0 | 0 | 0 | 0 | 0 | 6 | 18 | Carbamidomethylation |
| K.C(+57.02)NKLVPLFYK.T | N | 70.11 | 1280.6951 | 10 | 0.3 | 641.3550 | 2 | 12.05 | 2 | F2:2978 | 29102019\_RID\_1313\_NaNaPb\_F10.raw |  | 2.53E5 |  |  |  |  |  |  |  |  | 2 | 0 | 2 | 0 | 0 | 0 | 0 | 0 | 0 | 0 | 0 | 3 | 12 | Carbamidomethylation |
| LKC(+57.02)NKLVPLFYK.T | N | 64.71 | 1521.8741 | 12 | -0.7 | 508.2983 | 3 | 11.69 | 9 | F9:2501 | 29102019\_RID\_1313\_NaNaPb\_F8.raw |  | 2.08E5 |  |  |  |  |  |  | 0 |  | 2 | 0 | 1 | 0 | 0 | 0 | 0 | 0 | 0 | 1 | 0 | 1 | 12 | Carbamidomethylation |
| K.M(+15.99)YM(+15.99)VATPK.V | N | 64.46 | 971.4456 | 8 | 0.4 | 486.7303 | 2 | 11.02 | 2 | F2:1943 | 29102019\_RID\_1313\_NaNaPb\_F10.raw |  | 1.45E6 |  |  |  |  |  |  | 3.17E4 |  | 3 | 0 | 1 | 0 | 0 | 0 | 0 | 0 | 0 | 2 | 0 | 24 | 31 | Oxidation (M) |
| R.GC(+57.02)IDVC(+57.02)PK.S | N | 63.51 | 947.4205 | 8 | -0.6 | 474.7172 | 2 | 11.17 | 7 | F7:2031 | 29102019\_RID\_1313\_NaNaPb\_F6.raw |  | 2.85E7 |  |  |  | 7.32E7 | 1.17E6 |  | 5.42E6 | 9.79E6 | 10 | 0 | 2 | 0 | 0 | 0 | 5 | 1 | 0 | 1 | 1 | 37 | 44 | Carbamidomethylation |
| K.M(+15.99)YMVATPK.V | N | 57.69 | 955.4507 | 8 | -0.5 | 478.7324 | 2 | 11.26 | 2 | F2:2173 | 29102019\_RID\_1313\_NaNaPb\_F10.raw |  | 4.17E6 |  |  |  |  |  |  |  |  | 1 | 0 | 1 | 0 | 0 | 0 | 0 | 0 | 0 | 0 | 0 | 24 | 31 | Oxidation (M) |
| K.TC(+57.02)PAGKNLC(+57.02)YK.M | N | 54.37 | 1310.6111 | 11 | -0.4 | 437.8775 | 3 | 10.78 | 6 | F6:1669 | 29102019\_RID\_1313\_NaNaPb\_F5.raw |  |  |  |  |  | 3.23E4 |  |  |  |  | 1 | 0 | 0 | 0 | 0 | 0 | 1 | 0 | 0 | 0 | 0 | 13 | 23 | Carbamidomethylation |
| K.MYMVATPK.V | N | 54.33 | 939.4558 | 8 | 0.8 | 470.7356 | 2 | 11.55 | 2 | F2:2468 | 29102019\_RID\_1313\_NaNaPb\_F10.raw |  | 1.04E7 |  |  |  |  |  |  |  |  | 1 | 0 | 1 | 0 | 0 | 0 | 0 | 0 | 0 | 0 | 0 | 24 | 31 |  |
| K.NLC(+57.02)YKMYMVATPK.V | N | 53.54 | 1617.7717 | 13 | -0.3 | 540.2643 | 3 | 11.87 | 2 | F2:2798 | 29102019\_RID\_1313\_NaNaPb\_F10.raw |  | 1.87E5 |  |  |  |  |  |  |  |  | 1 | 0 | 1 | 0 | 0 | 0 | 0 | 0 | 0 | 0 | 0 | 19 | 31 | Carbamidomethylation |
| K.RGC(+57.02)IDVC(+57.02)PK.S | N | 48.01 | 1103.5216 | 9 | 0.2 | 552.7682 | 2 | 10.91 | 6 | F6:1732 | 29102019\_RID\_1313\_NaNaPb\_F5.raw |  |  |  |  |  | 6.74E5 |  |  |  |  | 1 | 0 | 0 | 0 | 0 | 0 | 1 | 0 | 0 | 0 | 0 | 36 | 44 | Carbamidomethylation |
| total 11 peptides |
| --- |

P19012|K1C15\_HUMAN

back to list

  

| Protein Coverage
| Supporting Peptides
|

Protein Coverage:

Supporting Peptides:

| Peptide | Uniq | -10lgP | Mass | Length | ppm | m/z | z | RT | Fraction | Scan | Source File | Area F1 | Area F10 | Area F2 | Area F3 | Area F4 | Area F5 | Area F6 | Area F7 | Area F8 | Area F9 | #Feature | #Feature F1 | #Feature F10 | #Feature F2 | #Feature F3 | #Feature F4 | #Feature F5 | #Feature F6 | #Feature F7 | #Feature F8 | #Feature F9 | Start | End | PTM |
| --- | --- | --- | --- | --- | --- | --- | --- | --- | --- | --- | --- | --- | --- | --- | --- | --- | --- | --- | --- | --- | --- | --- | --- | --- | --- | --- | --- | --- | --- | --- | --- | --- | --- | --- | --- |
| R.ALEEANADLEVK.I | N | 92.34 | 1300.6510 | 12 | 0.2 | 651.3329 | 2 | 11.91 | 1 | F1:3410 | 29102019\_RID\_1313\_NaNaPb\_F1.raw | 9.19E4 | 1.48E5 | 1.95E5 |  |  | 3.99E5 | 0 |  |  | 1.22E5 | 6 | 1 | 1 | 1 | 0 | 0 | 1 | 1 | 0 | 0 | 1 | 125 | 136 |  |
| R.VLDELTLAR.T | N | 67.89 | 1028.5865 | 9 | 0.8 | 515.3010 | 2 | 62.60 | 5 | F5:26241 | 29102019\_RID\_1313\_NaNaPb\_F4.raw | 1.32E5 |  |  |  | 8.65E5 | 5.32E5 | 1.7E5 |  |  | 1.39E5 | 5 | 1 | 0 | 0 | 0 | 1 | 1 | 1 | 0 | 0 | 1 | 215 | 223 |  |
| R.LEQEIATYR.S | N | 65.29 | 1121.5717 | 9 | 0.7 | 561.7935 | 2 | 11.51 | 6 | F6:2300 | 29102019\_RID\_1313\_NaNaPb\_F5.raw |  |  | 1.24E5 |  |  | 1.68E5 |  |  |  |  | 2 | 0 | 0 | 1 | 0 | 0 | 1 | 0 | 0 | 0 | 0 | 399 | 407 |  |
| R.LAADDFR.L | N | 56.98 | 806.3922 | 7 | 0.4 | 404.2036 | 2 | 11.73 | 1 | F1:3258 | 29102019\_RID\_1313\_NaNaPb\_F1.raw | 1E5 | 2.78E5 | 1.15E5 |  |  | 1.11E5 | 5.12E4 |  | 2.99E4 |  | 6 | 1 | 1 | 1 | 0 | 0 | 1 | 1 | 0 | 1 | 0 | 186 | 192 |  |
| R.LASYLDK.V | N | 55.97 | 808.4330 | 7 | -0.9 | 405.2234 | 2 | 11.66 | 1 | F1:3180 | 29102019\_RID\_1313\_NaNaPb\_F1.raw | 9.27E4 |  | 3.47E5 |  |  | 0 | 5.32E4 |  |  |  | 4 | 1 | 0 | 1 | 0 | 0 | 1 | 1 | 0 | 0 | 0 | 116 | 122 |  |
| V.GGGFGGGFGGGDGGLLSGNEK.I | Y | 54.95 | 1795.8125 | 21 | -0.2 | 898.9133 | 2 | 18.39 | 1 | F1:5833 | 29102019\_RID\_1313\_NaNaPb\_F1.raw | 5.76E3 |  |  |  |  |  |  |  |  |  | 1 | 1 | 0 | 0 | 0 | 0 | 0 | 0 | 0 | 0 | 0 | 86 | 106 |  |
| R.VILEIDNAR.L | N | 50.50 | 1041.5818 | 9 | -0.4 | 521.7980 | 2 | 12.09 | 6 | F6:2869 | 29102019\_RID\_1313\_NaNaPb\_F5.raw |  |  |  |  |  | 4.56E4 |  |  |  |  | 1 | 0 | 0 | 0 | 0 | 0 | 1 | 0 | 0 | 0 | 0 | 177 | 185 |  |
| total 7 peptides |
| --- |

D5LMJ3|VM3A\_NAJAT

back to list

  

| Protein Coverage
| Supporting Peptides
|

Protein Coverage:

Supporting Peptides:

| Peptide | Uniq | -10lgP | Mass | Length | ppm | m/z | z | RT | Fraction | Scan | Source File | Area F1 | Area F10 | Area F2 | Area F3 | Area F4 | Area F5 | Area F6 | Area F7 | Area F8 | Area F9 | #Feature | #Feature F1 | #Feature F10 | #Feature F2 | #Feature F3 | #Feature F4 | #Feature F5 | #Feature F6 | #Feature F7 | #Feature F8 | #Feature F9 | Start | End | PTM |
| --- | --- | --- | --- | --- | --- | --- | --- | --- | --- | --- | --- | --- | --- | --- | --- | --- | --- | --- | --- | --- | --- | --- | --- | --- | --- | --- | --- | --- | --- | --- | --- | --- | --- | --- | --- |
| K.C(+57.02)GTLYC(+57.02)TEIK.K | Y | 80.94 | 1243.5577 | 10 | -0.3 | 622.7859 | 2 | 11.53 | 2 | F2:2445 | 29102019\_RID\_1313\_NaNaPb\_F10.raw |  | 5.34E5 |  |  |  |  |  |  |  | 5.73E5 | 2 | 0 | 1 | 0 | 0 | 0 | 0 | 0 | 0 | 0 | 1 | 560 | 569 | Carbamidomethylation |
| K.TGC(+57.02)IVPVSPR.D | Y | 76.49 | 1084.5699 | 10 | -0.2 | 543.2921 | 2 | 11.57 | 10 | F10:2379 | 29102019\_RID\_1313\_NaNaPb\_F9.raw |  | 7.88E5 |  |  |  |  |  |  |  | 6.61E5 | 2 | 0 | 1 | 0 | 0 | 0 | 0 | 0 | 0 | 0 | 1 | 571 | 580 | Carbamidomethylation |
| W.GSVAVVQDYSR.R | Y | 75.89 | 1179.5884 | 11 | -0.3 | 590.8013 | 2 | 11.38 | 2 | F2:2298 | 29102019\_RID\_1313\_NaNaPb\_F10.raw |  | 9.7E5 |  |  |  |  |  |  |  | 8.97E5 | 2 | 0 | 1 | 0 | 0 | 0 | 0 | 0 | 0 | 0 | 1 | 317 | 327 |  |
| R.ERPQC(+57.02)ILNKPSR.K | Y | 74.44 | 1496.7881 | 12 | 0.6 | 499.9370 | 3 | 10.61 | 10 | F10:1593 | 29102019\_RID\_1313\_NaNaPb\_F9.raw |  | 7.16E5 |  |  |  |  |  |  |  | 1.85E5 | 3 | 0 | 2 | 0 | 0 | 0 | 0 | 0 | 0 | 0 | 1 | 389 | 400 | Carbamidomethylation |
| K.KTGC(+57.02)IVPVSPRDPDSR.M | Y | 71.12 | 1782.9047 | 16 | -0.2 | 595.3087 | 3 | 38.65 | 5 | F5:7335 | 29102019\_RID\_1313\_NaNaPb\_F4.raw |  |  |  |  | 6.93E5 |  |  |  |  |  | 1 | 0 | 0 | 0 | 0 | 1 | 0 | 0 | 0 | 0 | 0 | 570 | 585 | Carbamidomethylation |
| R.VYEM(+15.99)VNYLNTK.Y | Y | 69.17 | 1388.6646 | 11 | 0.4 | 695.3398 | 2 | 11.69 | 2 | F2:2598 | 29102019\_RID\_1313\_NaNaPb\_F10.raw |  | 0 |  |  |  |  |  |  |  | 1.51E4 | 2 | 0 | 1 | 0 | 0 | 0 | 0 | 0 | 0 | 0 | 1 | 231 | 241 | Oxidation (M) |
| K.TGC(+57.02)IVPVSPRDPDSR.M | Y | 56.45 | 1654.8097 | 15 | 1.3 | 552.6112 | 3 | 44.73 | 5 | F5:12022 | 29102019\_RID\_1313\_NaNaPb\_F4.raw |  |  |  |  | 1.48E6 |  |  |  |  |  | 1 | 0 | 0 | 0 | 0 | 1 | 0 | 0 | 0 | 0 | 0 | 571 | 585 | Carbamidomethylation |
| R.VYEMVNYLNTK.Y | Y | 56.13 | 1372.6697 | 11 | 1.1 | 687.3429 | 2 | 13.09 | 9 | F9:3615 | 29102019\_RID\_1313\_NaNaPb\_F8.raw |  | 4.52E5 |  |  |  |  |  |  | 4.05E4 | 3.05E5 | 3 | 0 | 1 | 0 | 0 | 0 | 0 | 0 | 0 | 1 | 1 | 231 | 241 |  |
| Y.TGTLC(+57.02)TWGSVAVVQDYSR.R | Y | 53.09 | 1998.9469 | 18 | 0.8 | 1000.4816 | 2 | 18.91 | 2 | F2:6718 | 29102019\_RID\_1313\_NaNaPb\_F10.raw |  | 0 |  |  |  |  |  |  |  |  | 1 | 0 | 1 | 0 | 0 | 0 | 0 | 0 | 0 | 0 | 0 | 310 | 327 | Carbamidomethylation |
| E.PFYEFSSC(+57.02)SVR.E | Y | 45.02 | 1377.6023 | 11 | -0.1 | 689.8083 | 2 | 12.30 | 10 | F10:3090 | 29102019\_RID\_1313\_NaNaPb\_F9.raw |  |  |  |  |  |  |  |  |  | 2.25E4 | 1 | 0 | 0 | 0 | 0 | 0 | 0 | 0 | 0 | 0 | 1 | 370 | 380 | Carbamidomethylation |
| total 10 peptides |
| --- |

P13646|K1C13\_HUMAN

back to list

  

| Protein Coverage
| Supporting Peptides
|

Protein Coverage:

Supporting Peptides:

| Peptide | Uniq | -10lgP | Mass | Length | ppm | m/z | z | RT | Fraction | Scan | Source File | Area F1 | Area F10 | Area F2 | Area F3 | Area F4 | Area F5 | Area F6 | Area F7 | Area F8 | Area F9 | #Feature | #Feature F1 | #Feature F10 | #Feature F2 | #Feature F3 | #Feature F4 | #Feature F5 | #Feature F6 | #Feature F7 | #Feature F8 | #Feature F9 | Start | End | PTM |
| --- | --- | --- | --- | --- | --- | --- | --- | --- | --- | --- | --- | --- | --- | --- | --- | --- | --- | --- | --- | --- | --- | --- | --- | --- | --- | --- | --- | --- | --- | --- | --- | --- | --- | --- | --- |
| R.ALEEANADLEVK.I | N | 92.34 | 1300.6510 | 12 | 0.2 | 651.3329 | 2 | 11.91 | 1 | F1:3410 | 29102019\_RID\_1313\_NaNaPb\_F1.raw | 9.19E4 | 1.48E5 | 1.95E5 |  |  | 3.99E5 | 0 |  |  | 1.22E5 | 6 | 1 | 1 | 1 | 0 | 0 | 1 | 1 | 0 | 0 | 1 | 124 | 135 |  |
| R.QSVEADINGLR.R | N | 73.92 | 1200.6099 | 11 | -0.1 | 601.3121 | 2 | 11.73 | 9 | F9:2547 | 29102019\_RID\_1313\_NaNaPb\_F8.raw | 6.63E5 | 1.4E6 | 2.92E5 | 4.55E4 |  |  | 4.34E4 |  | 1.09E5 |  | 6 | 1 | 1 | 1 | 1 | 0 | 0 | 1 | 0 | 1 | 0 | 202 | 212 |  |
| R.LEQEIATYR.S | N | 65.29 | 1121.5717 | 9 | 0.7 | 561.7935 | 2 | 11.51 | 6 | F6:2300 | 29102019\_RID\_1313\_NaNaPb\_F5.raw |  |  | 1.24E5 |  |  | 1.68E5 |  |  |  |  | 2 | 0 | 0 | 1 | 0 | 0 | 1 | 0 | 0 | 0 | 0 | 398 | 406 |  |
| R.LAADDFR.L | N | 56.98 | 806.3922 | 7 | 0.4 | 404.2036 | 2 | 11.73 | 1 | F1:3258 | 29102019\_RID\_1313\_NaNaPb\_F1.raw | 1E5 | 2.78E5 | 1.15E5 |  |  | 1.11E5 | 5.12E4 |  | 2.99E4 |  | 6 | 1 | 1 | 1 | 0 | 0 | 1 | 1 | 0 | 1 | 0 | 185 | 191 |  |
| R.VILEIDNAR.L | N | 50.50 | 1041.5818 | 9 | -0.4 | 521.7980 | 2 | 12.09 | 6 | F6:2869 | 29102019\_RID\_1313\_NaNaPb\_F5.raw |  |  |  |  |  | 4.56E4 |  |  |  |  | 1 | 0 | 0 | 0 | 0 | 0 | 1 | 0 | 0 | 0 | 0 | 176 | 184 |  |
| R.LQSSSASYGGGFGGGSC(+57.02)QLGGGR.G | Y | 45.34 | 2145.9497 | 23 | -0.4 | 1073.9817 | 2 | 11.62 | 10 | F10:2459 | 29102019\_RID\_1313\_NaNaPb\_F9.raw |  |  |  |  |  |  |  |  |  | 3.33E4 | 1 | 0 | 0 | 0 | 0 | 0 | 0 | 0 | 0 | 0 | 1 | 5 | 27 | Carbamidomethylation |
| R.QSVEADINGLRR.V | N | 43.74 | 1356.7109 | 12 | 0.2 | 453.2444 | 3 | 11.54 | 6 | F6:2345 | 29102019\_RID\_1313\_NaNaPb\_F5.raw |  |  |  |  |  | 1.5E5 |  |  |  |  | 1 | 0 | 0 | 0 | 0 | 0 | 1 | 0 | 0 | 0 | 0 | 202 | 213 |  |
| total 7 peptides |
| --- |

Q8BGZ7|K2C75\_MOUSE

back to list

  

| Protein Coverage
| Supporting Peptides
|

Protein Coverage:

Supporting Peptides:

| Peptide | Uniq | -10lgP | Mass | Length | ppm | m/z | z | RT | Fraction | Scan | Source File | Area F1 | Area F10 | Area F2 | Area F3 | Area F4 | Area F5 | Area F6 | Area F7 | Area F8 | Area F9 | #Feature | #Feature F1 | #Feature F10 | #Feature F2 | #Feature F3 | #Feature F4 | #Feature F5 | #Feature F6 | #Feature F7 | #Feature F8 | #Feature F9 | Start | End | PTM |
| --- | --- | --- | --- | --- | --- | --- | --- | --- | --- | --- | --- | --- | --- | --- | --- | --- | --- | --- | --- | --- | --- | --- | --- | --- | --- | --- | --- | --- | --- | --- | --- | --- | --- | --- | --- |
| R.SLDLDSIIAEVK.A | N | 78.89 | 1301.7078 | 12 | 0.4 | 651.8614 | 2 | 34.51 | 9 | F9:13199 | 29102019\_RID\_1313\_NaNaPb\_F8.raw | 1.93E6 | 5.03E6 | 3.54E6 | 9.45E4 | 0 | 5.83E6 | 2.88E6 |  | 7.49E5 | 2.61E5 | 20 | 3 | 3 | 3 | 1 | 1 | 4 | 3 | 0 | 1 | 1 | 314 | 325 |  |
| K.TLNNKFASFIDKVR.F | N | 77.96 | 1651.9045 | 14 | 0.2 | 413.9835 | 4 | 59.92 | 5 | F5:23986 | 29102019\_RID\_1313\_NaNaPb\_F4.raw |  |  |  |  | 1.32E6 |  |  |  |  |  | 2 | 0 | 0 | 0 | 0 | 2 | 0 | 0 | 0 | 0 | 0 | 156 | 169 |  |
[truncated: 115,267 more chars]
