# Supplementary figures and images for: Biogeographical venom variation in the Indian spectacled cobra (Naja naja) underscores the pressing need for pan-India efficacious snakebite therapy (part 2 of 2)
Source: PLoS Negl Trop Dis. 2021 Feb 18;15(2):e0009150. doi: 10.1371/journal.pntd.0009150 (PMC7924803; doi:10.1371/journal.pntd.0009150)

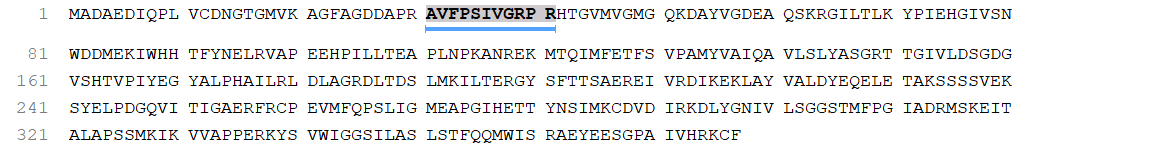

Supplement: S1 Data — (ZIP) [file pntd.0009150.s009.zip › S1 Data/N. naja_Punjab/img/cov_506.png]

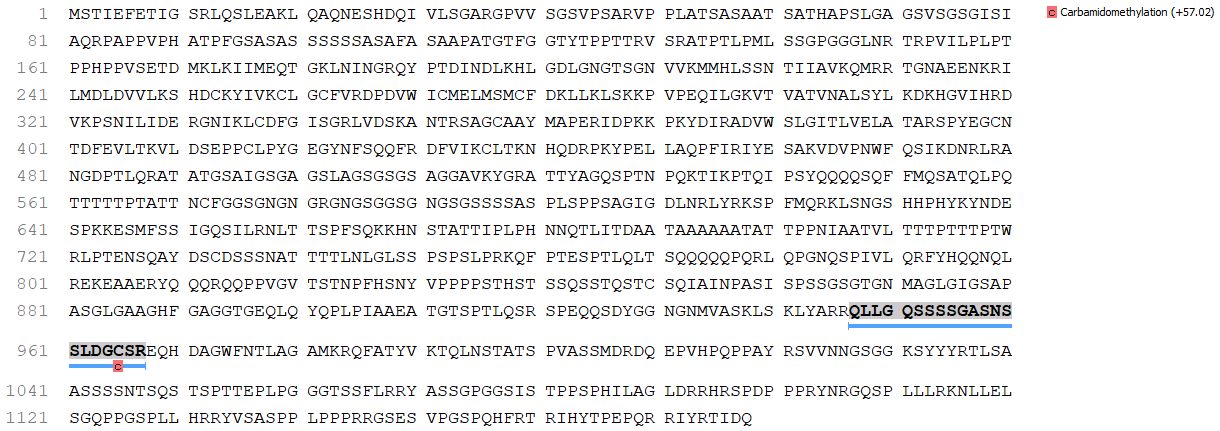

Supplement: S1 Data — (ZIP) [file pntd.0009150.s009.zip › S1 Data/N. naja_Punjab/img/cov_509.png]

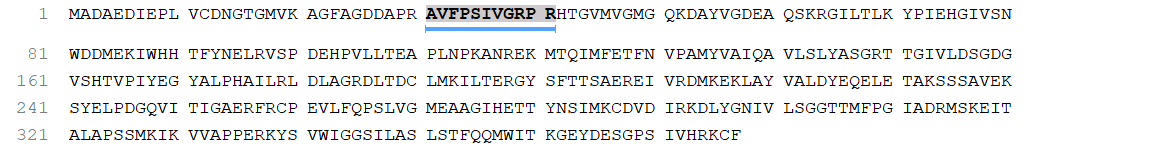

Supplement: S1 Data — (ZIP) [file pntd.0009150.s009.zip › S1 Data/N. naja_Punjab/img/cov_543.png]

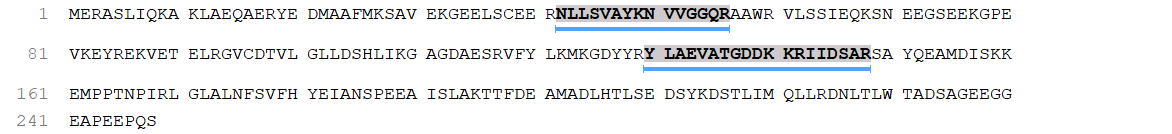

Supplement: S1 Data — (ZIP) [file pntd.0009150.s009.zip › S1 Data/N. naja_Punjab/img/cov_545.png]

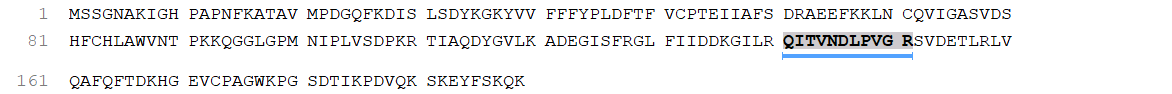

Supplement: S1 Data — (ZIP) [file pntd.0009150.s009.zip › S1 Data/N. naja_Punjab/img/cov_552.png]

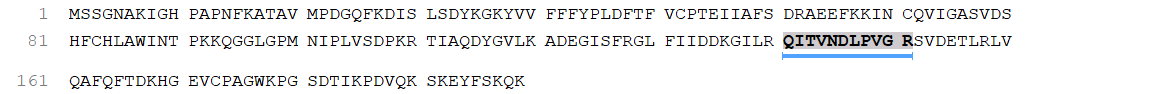

Supplement: S1 Data — (ZIP) [file pntd.0009150.s009.zip › S1 Data/N. naja_Punjab/img/cov_553.png]

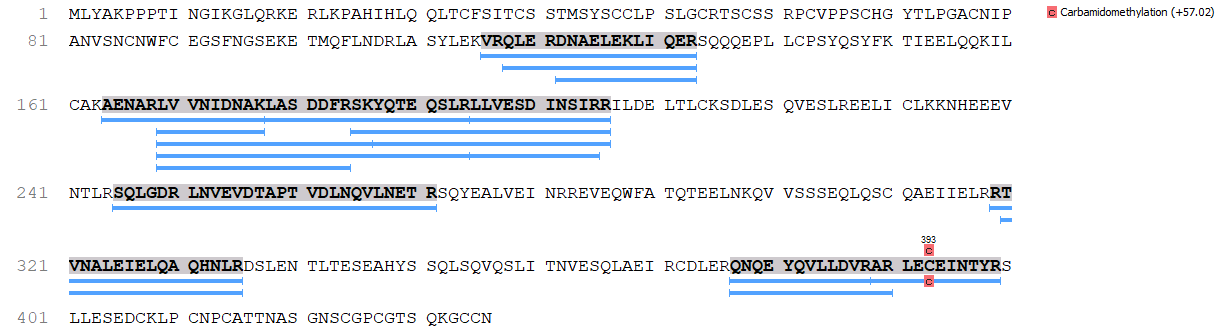

Supplement: S1 Data — (ZIP) [file pntd.0009150.s009.zip › S1 Data/N. naja_Punjab/img/cov_56.png]

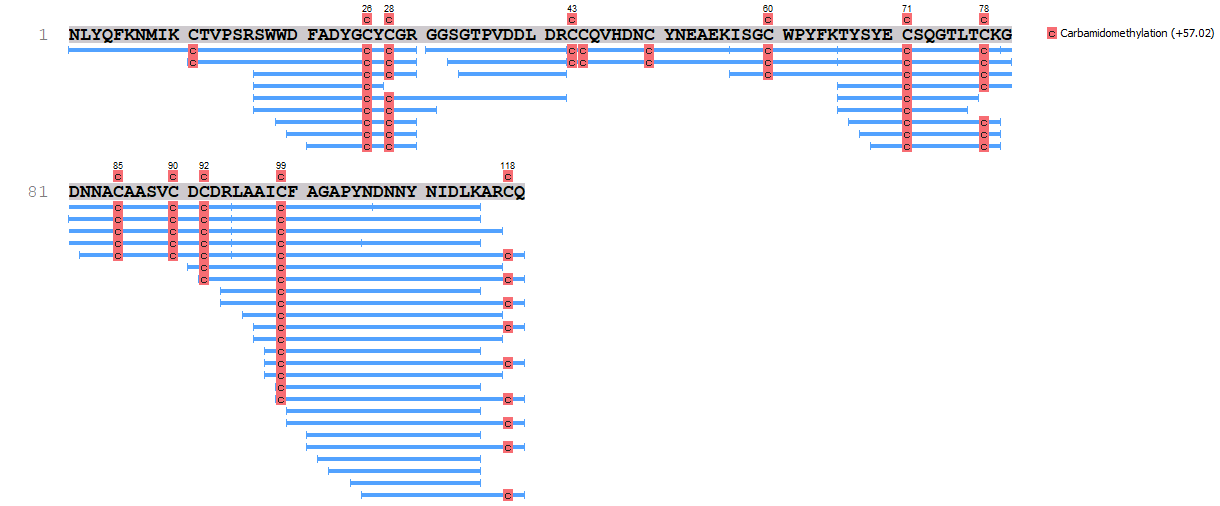

Supplement: S1 Data — (ZIP) [file pntd.0009150.s009.zip › S1 Data/N. naja_Punjab/img/cov_57.png]

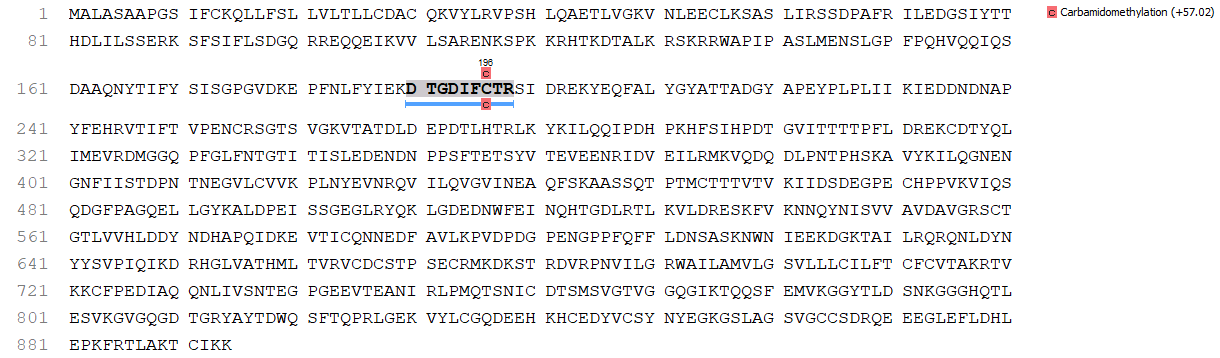

Supplement: S1 Data — (ZIP) [file pntd.0009150.s009.zip › S1 Data/N. naja_Punjab/img/cov_588.png]

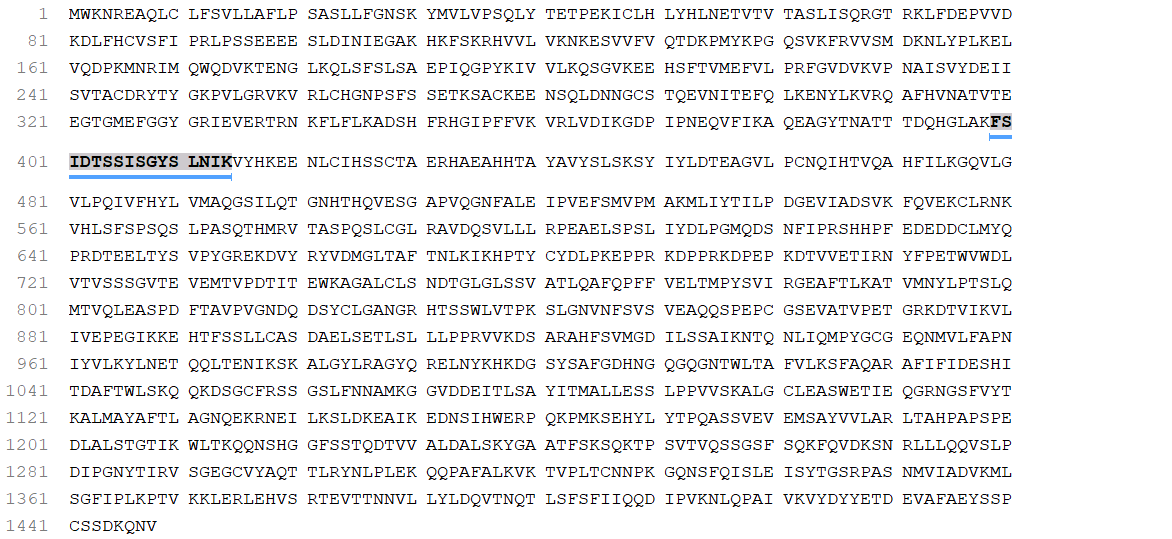

Supplement: S1 Data — (ZIP) [file pntd.0009150.s009.zip › S1 Data/N. naja_Punjab/img/cov_591.png]

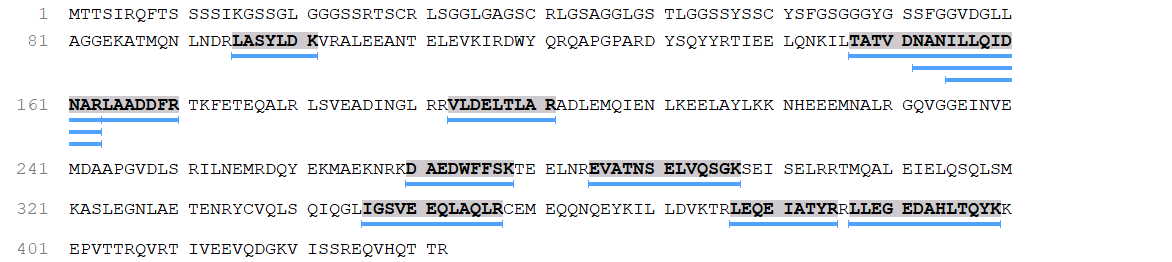

Supplement: S1 Data — (ZIP) [file pntd.0009150.s009.zip › S1 Data/N. naja_Punjab/img/cov_60.png]

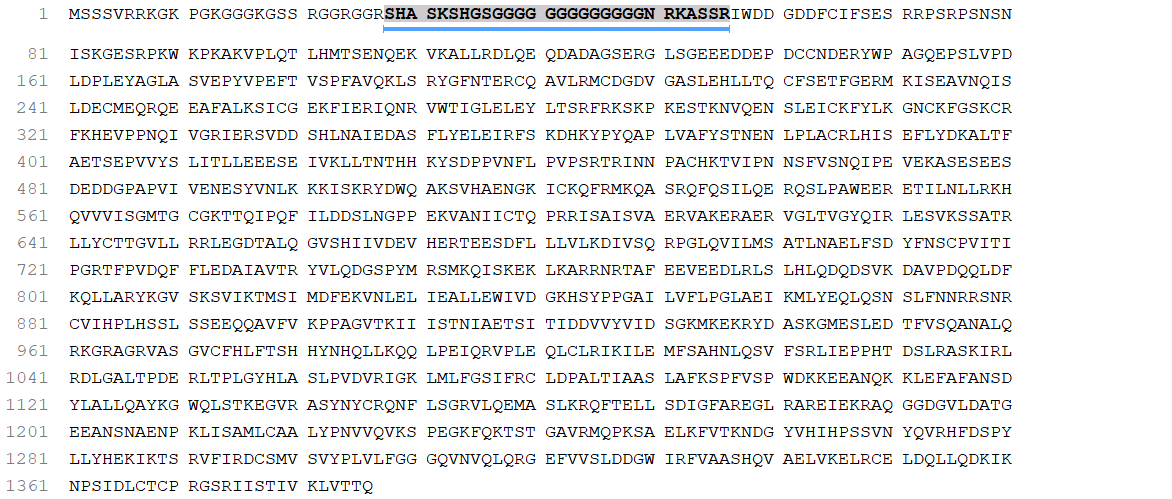

Supplement: S1 Data — (ZIP) [file pntd.0009150.s009.zip › S1 Data/N. naja_Punjab/img/cov_600.png]

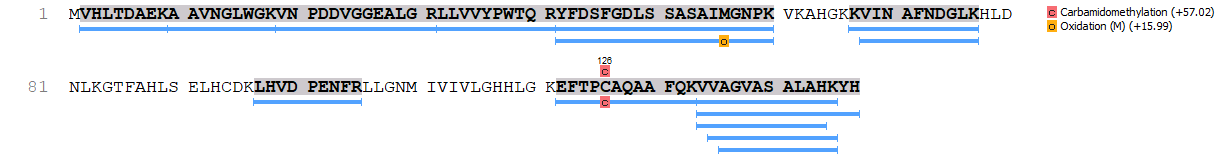

Supplement: S1 Data — (ZIP) [file pntd.0009150.s009.zip › S1 Data/N. naja_Punjab/img/cov_62.png]

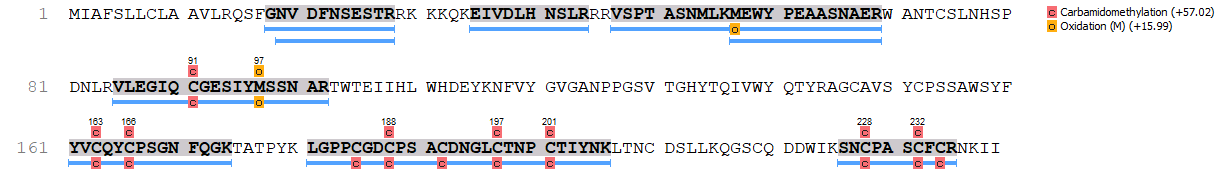

Supplement: S1 Data — (ZIP) [file pntd.0009150.s009.zip › S1 Data/N. naja_Punjab/img/cov_64.png]

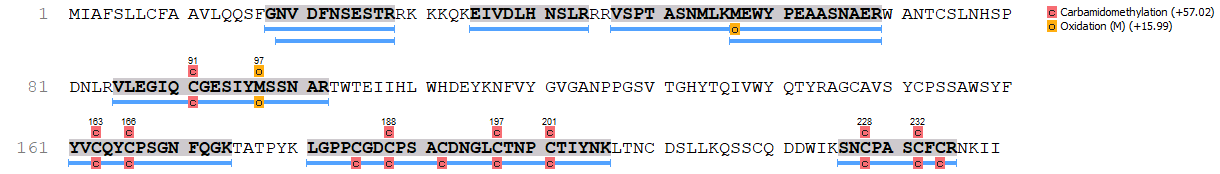

Supplement: S1 Data — (ZIP) [file pntd.0009150.s009.zip › S1 Data/N. naja_Punjab/img/cov_65.png]

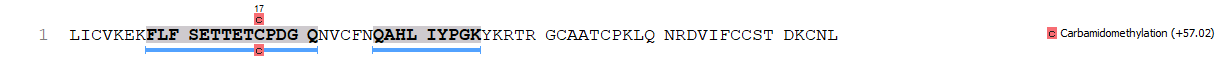

Supplement: S1 Data — (ZIP) [file pntd.0009150.s009.zip › S1 Data/N. naja_Punjab/img/cov_657.png]

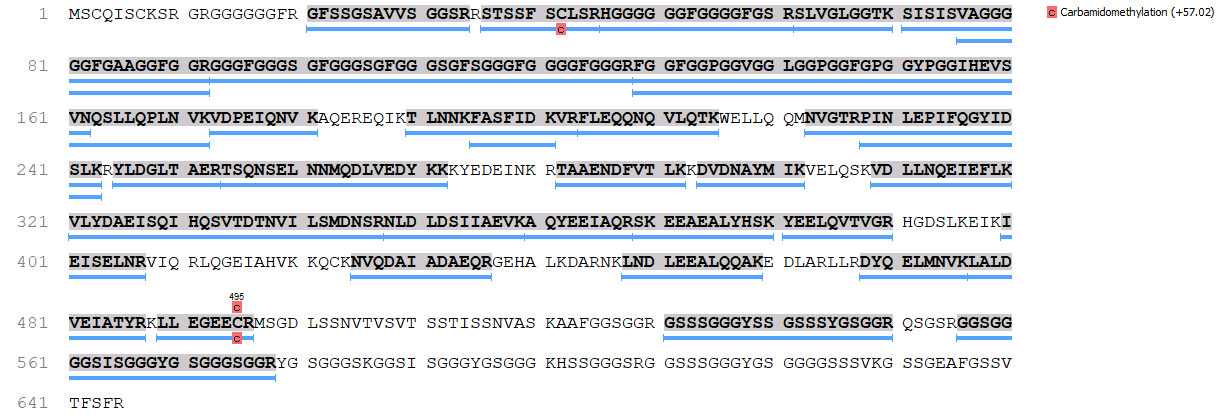

Supplement: S1 Data — (ZIP) [file pntd.0009150.s009.zip › S1 Data/N. naja_Punjab/img/cov_7.png]

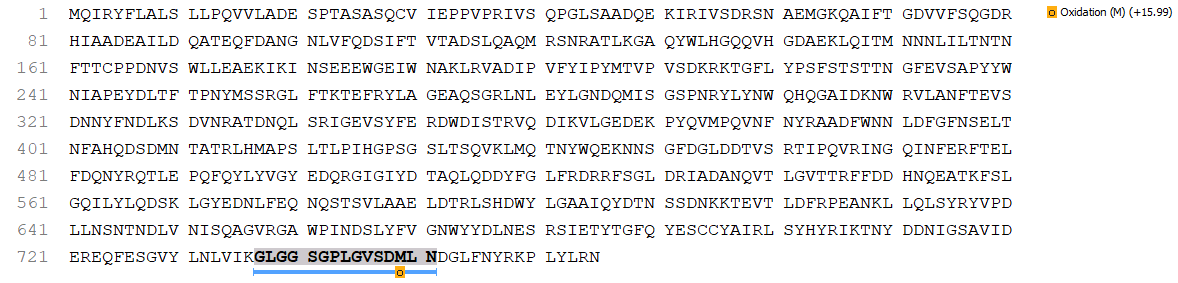

Supplement: S1 Data — (ZIP) [file pntd.0009150.s009.zip › S1 Data/N. naja_Punjab/img/cov_701.png]

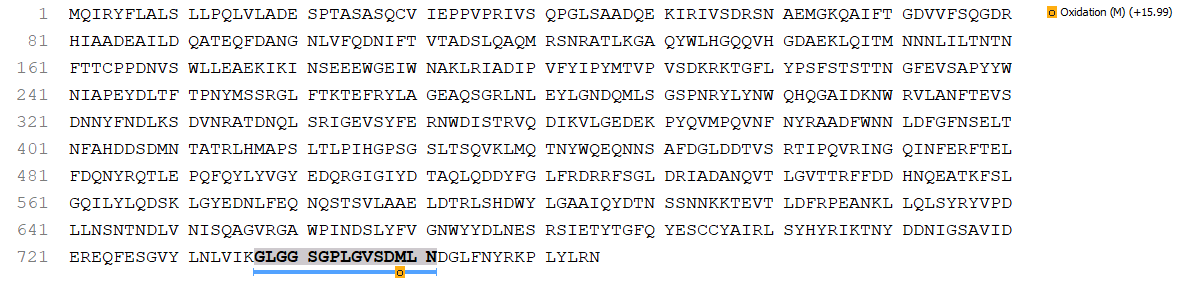

Supplement: S1 Data — (ZIP) [file pntd.0009150.s009.zip › S1 Data/N. naja_Punjab/img/cov_708.png]

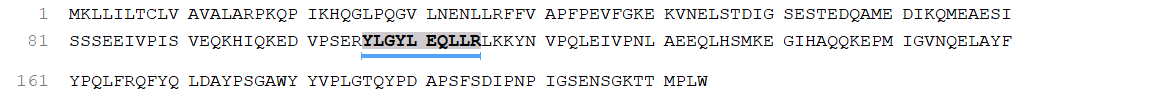

Supplement: S1 Data — (ZIP) [file pntd.0009150.s009.zip › S1 Data/N. naja_Punjab/img/cov_710.png]

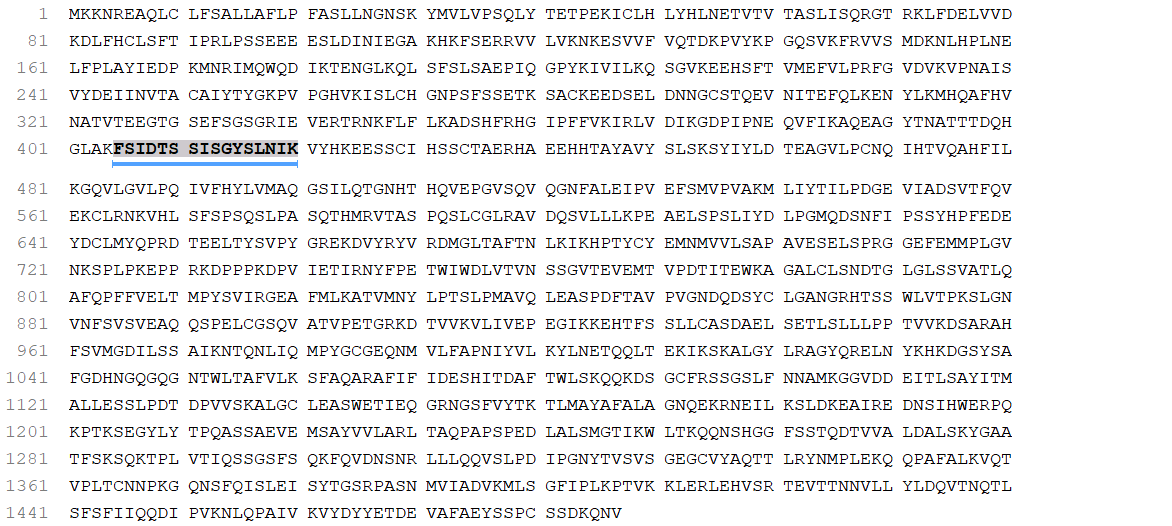

Supplement: S1 Data — (ZIP) [file pntd.0009150.s009.zip › S1 Data/N. naja_Punjab/img/cov_720.png]

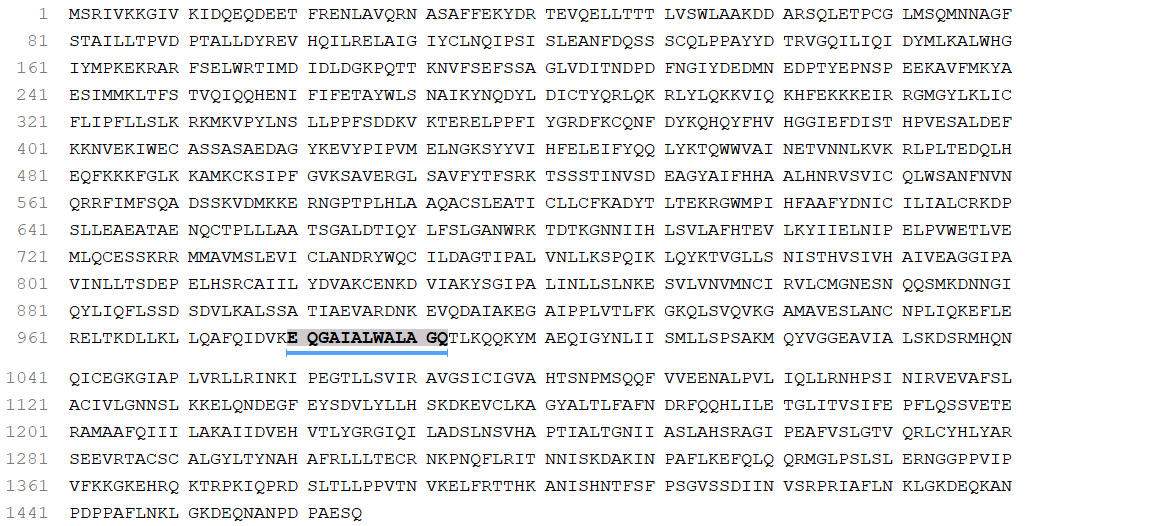

Supplement: S1 Data — (ZIP) [file pntd.0009150.s009.zip › S1 Data/N. naja_Punjab/img/cov_729.png]

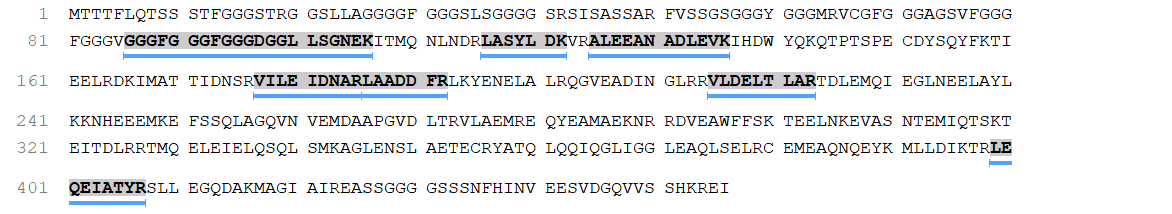

Supplement: S1 Data — (ZIP) [file pntd.0009150.s009.zip › S1 Data/N. naja_Punjab/img/cov_73.png]

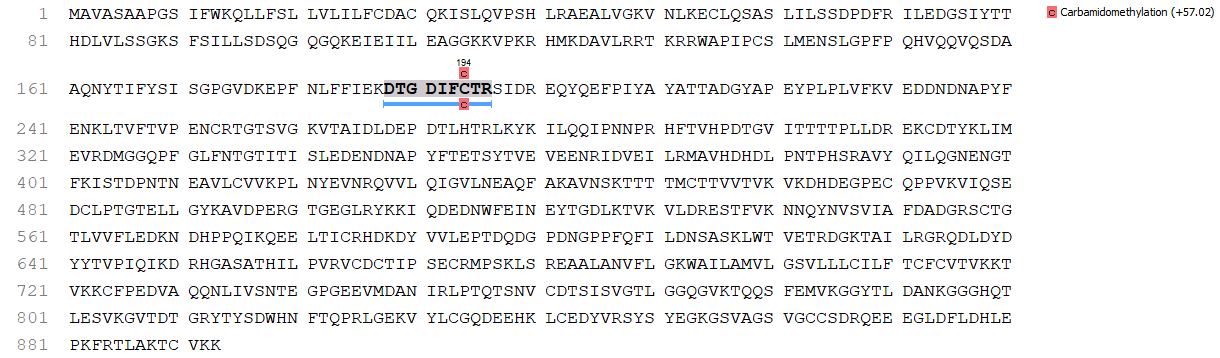

Supplement: S1 Data — (ZIP) [file pntd.0009150.s009.zip › S1 Data/N. naja_Punjab/img/cov_730.png]

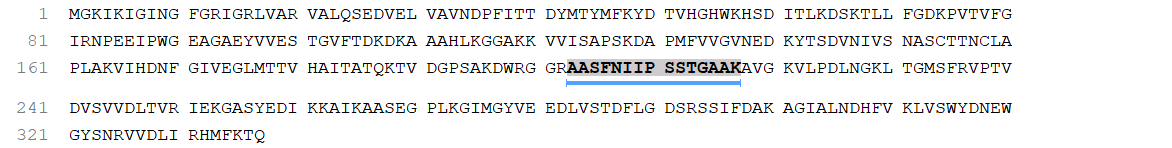

Supplement: S1 Data — (ZIP) [file pntd.0009150.s009.zip › S1 Data/N. naja_Punjab/img/cov_733.png]

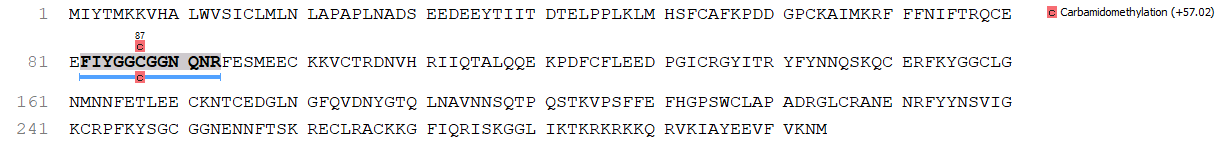

Supplement: S1 Data — (ZIP) [file pntd.0009150.s009.zip › S1 Data/N. naja_Punjab/img/cov_737.png]

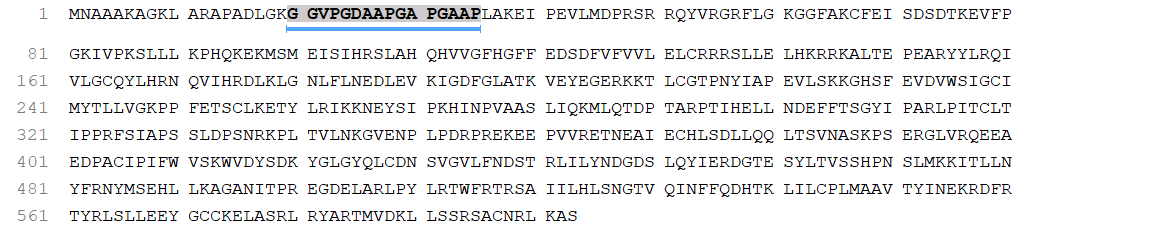

Supplement: S1 Data — (ZIP) [file pntd.0009150.s009.zip › S1 Data/N. naja_Punjab/img/cov_746.png]

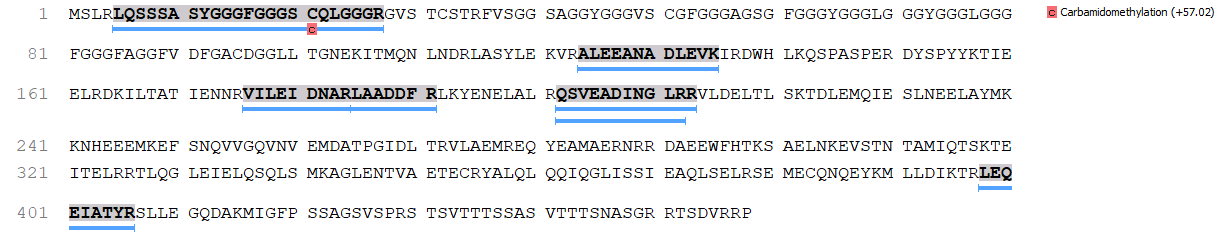

Supplement: S1 Data — (ZIP) [file pntd.0009150.s009.zip › S1 Data/N. naja_Punjab/img/cov_76.png]

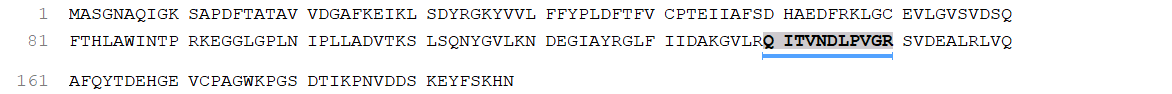

Supplement: S1 Data — (ZIP) [file pntd.0009150.s009.zip › S1 Data/N. naja_Punjab/img/cov_779.png]

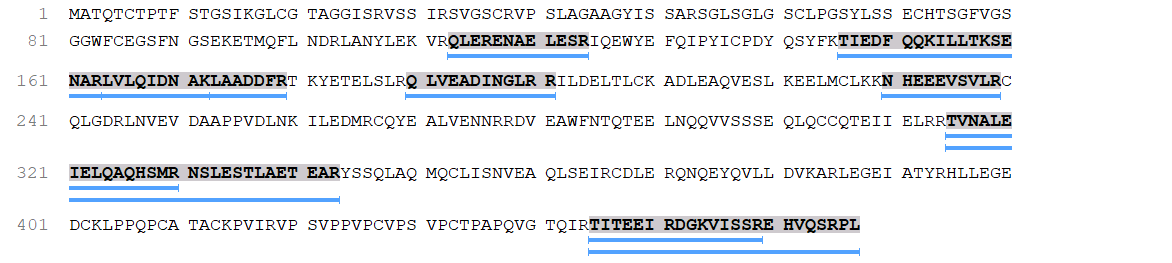

Supplement: S1 Data — (ZIP) [file pntd.0009150.s009.zip › S1 Data/N. naja_Punjab/img/cov_78.png]

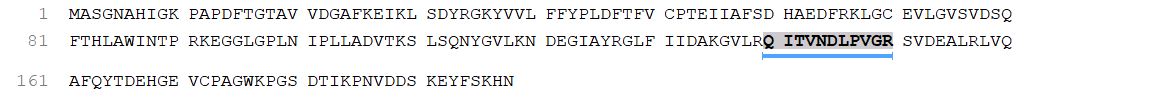

Supplement: S1 Data — (ZIP) [file pntd.0009150.s009.zip › S1 Data/N. naja_Punjab/img/cov_780.png]

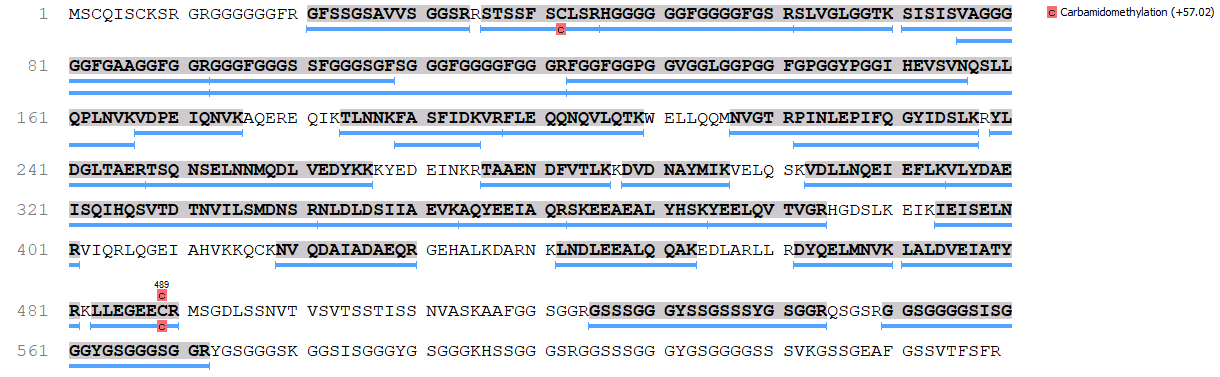

Supplement: S1 Data — (ZIP) [file pntd.0009150.s009.zip › S1 Data/N. naja_Punjab/img/cov_8.png]

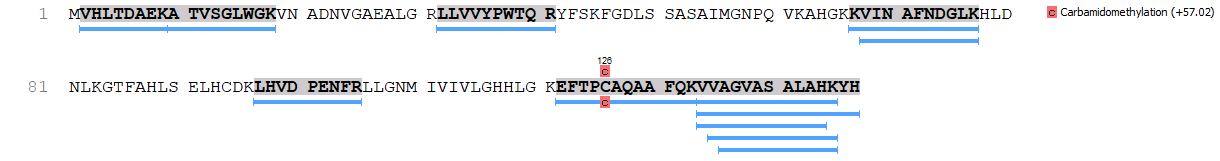

Supplement: S1 Data — (ZIP) [file pntd.0009150.s009.zip › S1 Data/N. naja_Punjab/img/cov_81.png]

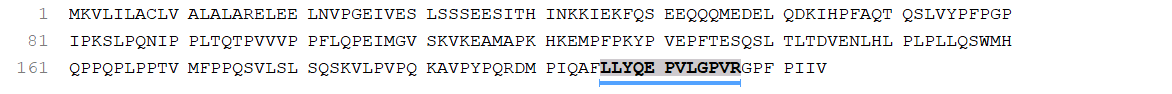

Supplement: S1 Data — (ZIP) [file pntd.0009150.s009.zip › S1 Data/N. naja_Punjab/img/cov_819.png]

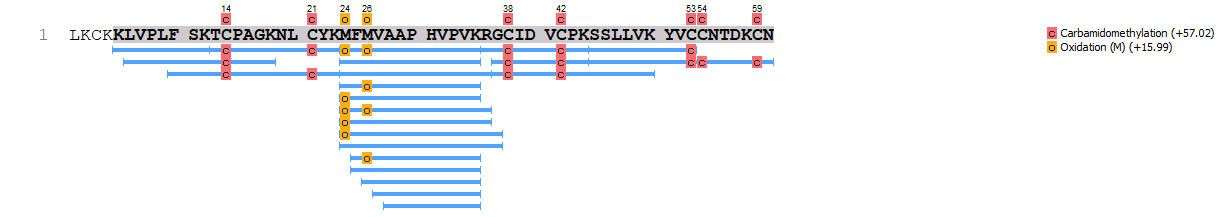

Supplement: S1 Data — (ZIP) [file pntd.0009150.s009.zip › S1 Data/N. naja_Punjab/img/cov_82.png]

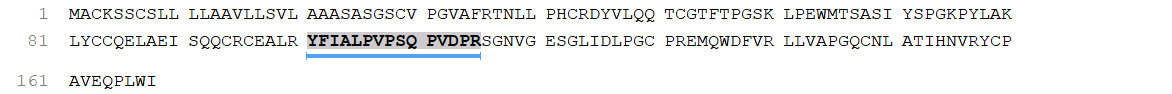

Supplement: S1 Data — (ZIP) [file pntd.0009150.s009.zip › S1 Data/N. naja_Punjab/img/cov_832.png]

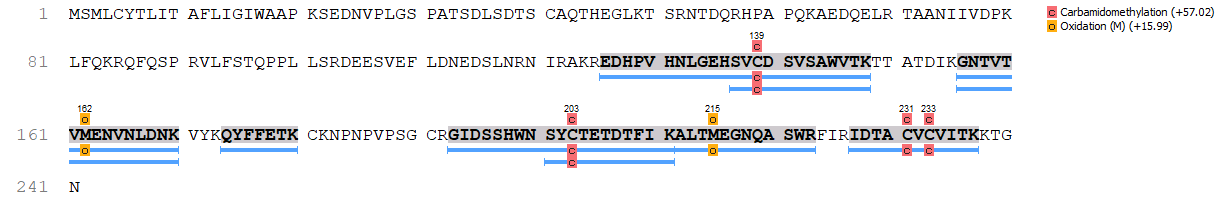

Supplement: S1 Data — (ZIP) [file pntd.0009150.s009.zip › S1 Data/N. naja_Punjab/img/cov_84.png]

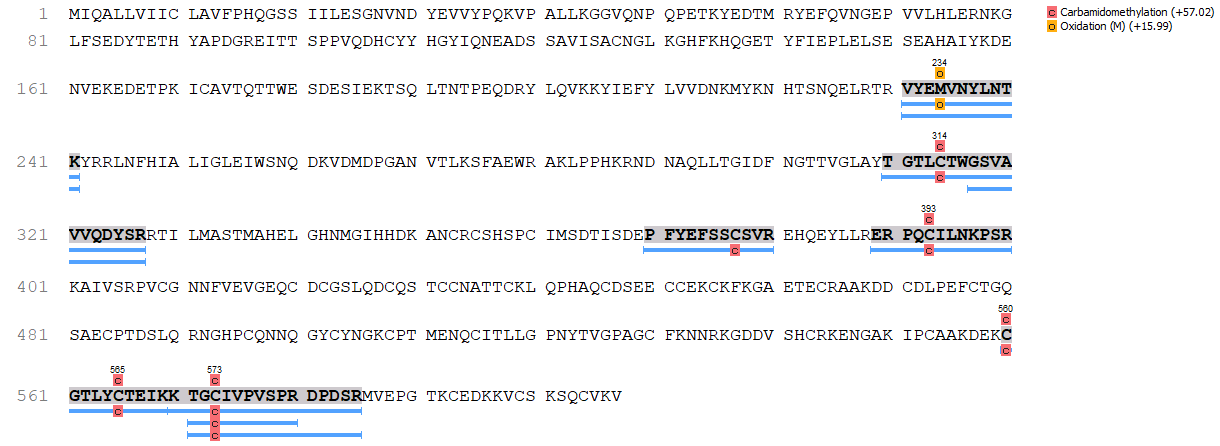

Supplement: S1 Data — (ZIP) [file pntd.0009150.s009.zip › S1 Data/N. naja_Punjab/img/cov_86.png]

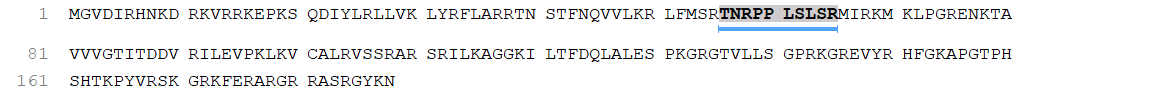

Supplement: S1 Data — (ZIP) [file pntd.0009150.s009.zip › S1 Data/N. naja_Punjab/img/cov_923.png]

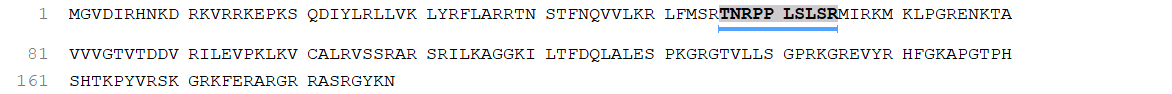

Supplement: S1 Data — (ZIP) [file pntd.0009150.s009.zip › S1 Data/N. naja_Punjab/img/cov_924.png]

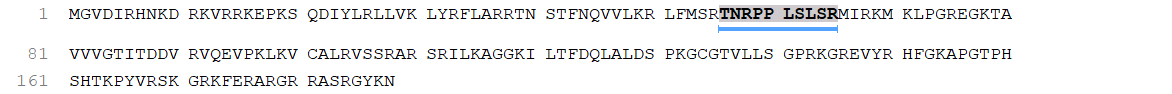

Supplement: S1 Data — (ZIP) [file pntd.0009150.s009.zip › S1 Data/N. naja_Punjab/img/cov_925.png]

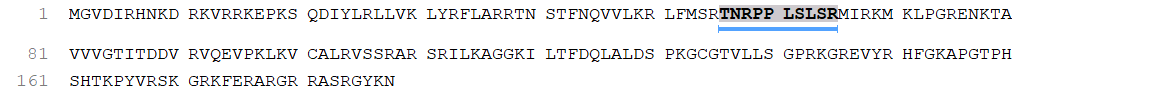

Supplement: S1 Data — (ZIP) [file pntd.0009150.s009.zip › S1 Data/N. naja_Punjab/img/cov_926.png]

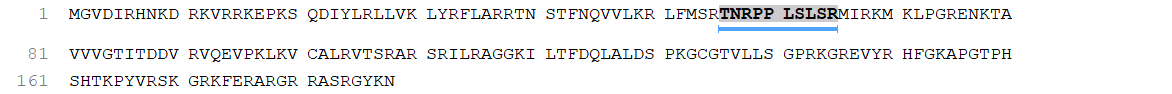

Supplement: S1 Data — (ZIP) [file pntd.0009150.s009.zip › S1 Data/N. naja_Punjab/img/cov_927.png]

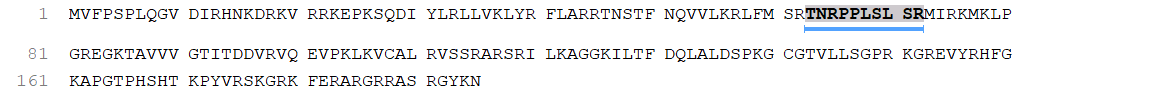

Supplement: S1 Data — (ZIP) [file pntd.0009150.s009.zip › S1 Data/N. naja_Punjab/img/cov_929.png]

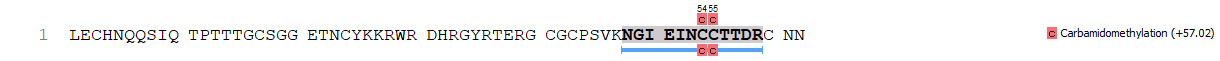

Supplement: S1 Data — (ZIP) [file pntd.0009150.s009.zip › S1 Data/N. naja_Punjab/img/cov_934.png]

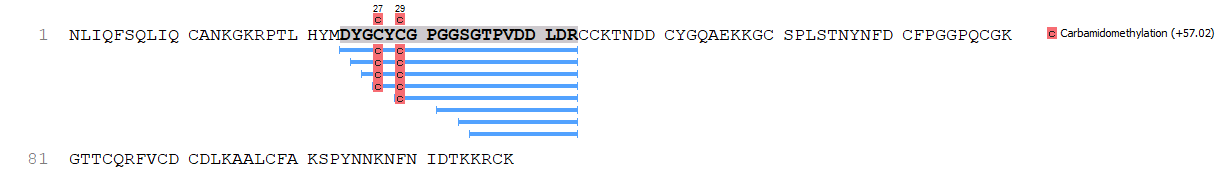

Supplement: S1 Data — (ZIP) [file pntd.0009150.s009.zip › S1 Data/N. naja_Punjab/img/cov_958.png]

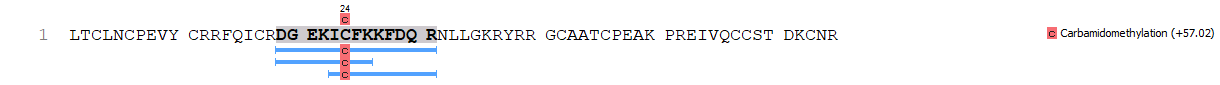

Supplement: S1 Data — (ZIP) [file pntd.0009150.s009.zip › S1 Data/N. naja_Punjab/img/cov_965.png]

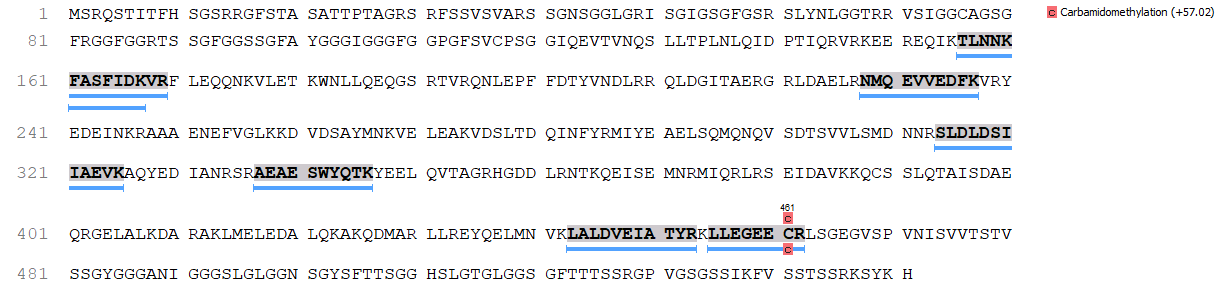

Supplement: S1 Data — (ZIP) [file pntd.0009150.s009.zip › S1 Data/N. naja_Punjab/img/cov_97.png]

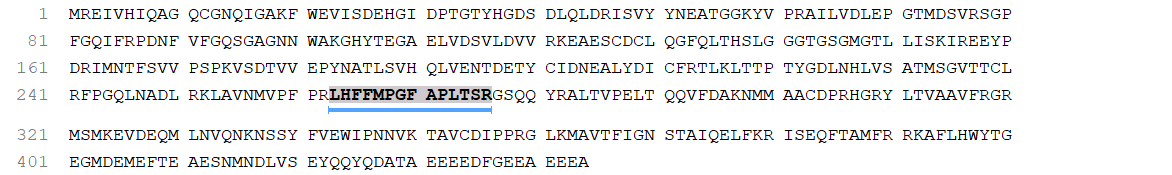

Supplement: S1 Data — (ZIP) [file pntd.0009150.s009.zip › S1 Data/N. naja_Punjab/img/cov_978.png]

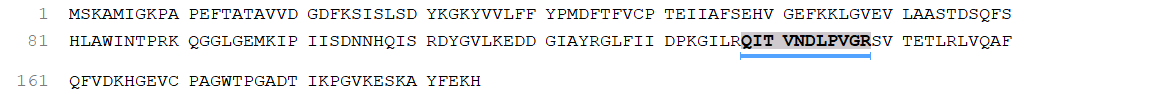

Supplement: S1 Data — (ZIP) [file pntd.0009150.s009.zip › S1 Data/N. naja_Punjab/img/cov_997.png]

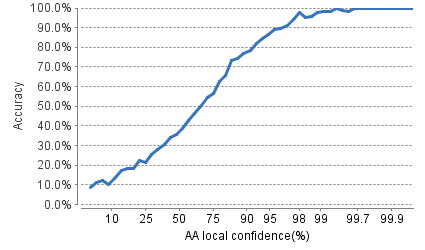

Supplement: S1 Data — (ZIP) [file pntd.0009150.s009.zip › S1 Data/N. naja_Punjab/img/DenovoFDRCurveFigure7472940864028181462.png]

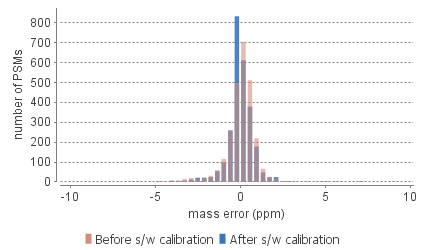

Supplement: S1 Data — (ZIP) [file pntd.0009150.s009.zip › S1 Data/N. naja_Punjab/img/ErrorCalibratedHistogram2910691029406146157.png]

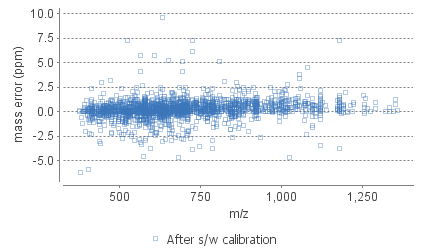

Supplement: S1 Data — (ZIP) [file pntd.0009150.s009.zip › S1 Data/N. naja_Punjab/img/ErrorPlotFigure917962253081684231.png]

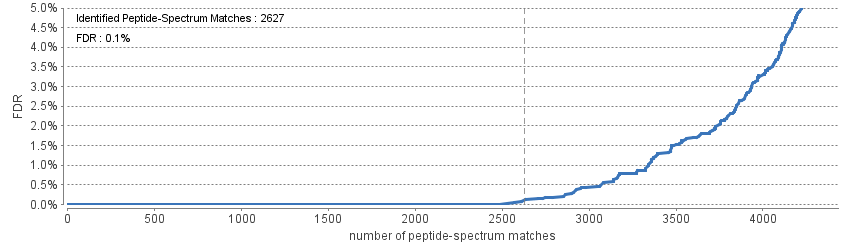

Supplement: S1 Data — (ZIP) [file pntd.0009150.s009.zip › S1 Data/N. naja_Punjab/img/FDRFigure8092046651779801831.png]

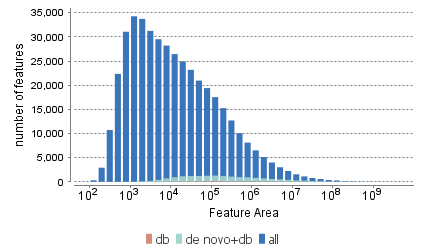

Supplement: S1 Data — (ZIP) [file pntd.0009150.s009.zip › S1 Data/N. naja_Punjab/img/FeatureIntensityDistributionHistogram2933080955431334583.png]

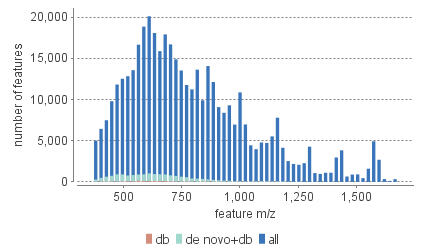

Supplement: S1 Data — (ZIP) [file pntd.0009150.s009.zip › S1 Data/N. naja_Punjab/img/FeatureMzHistogram7451986651341580781.png]

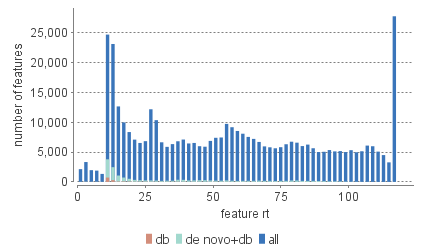

Supplement: S1 Data — (ZIP) [file pntd.0009150.s009.zip › S1 Data/N. naja_Punjab/img/FeatureRtHistogram1898665837529469814.png]

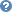

Supplement: S1 Data — (ZIP) [file pntd.0009150.s009.zip › S1 Data/N. naja_Punjab/img/q.png]

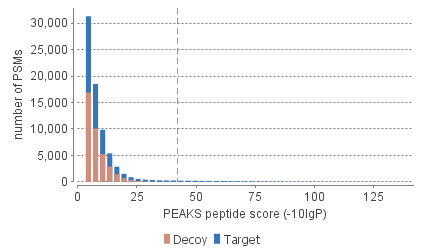

Supplement: S1 Data — (ZIP) [file pntd.0009150.s009.zip › S1 Data/N. naja_Punjab/img/ScoreHistogram4470794427893145060.png]

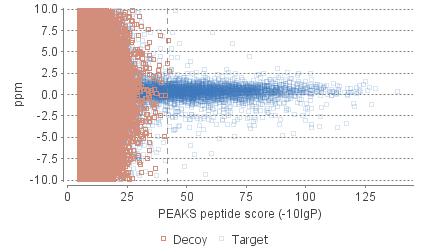

Supplement: S1 Data — (ZIP) [file pntd.0009150.s009.zip › S1 Data/N. naja_Punjab/img/ScorePlotFigure8026565393651079794.png]

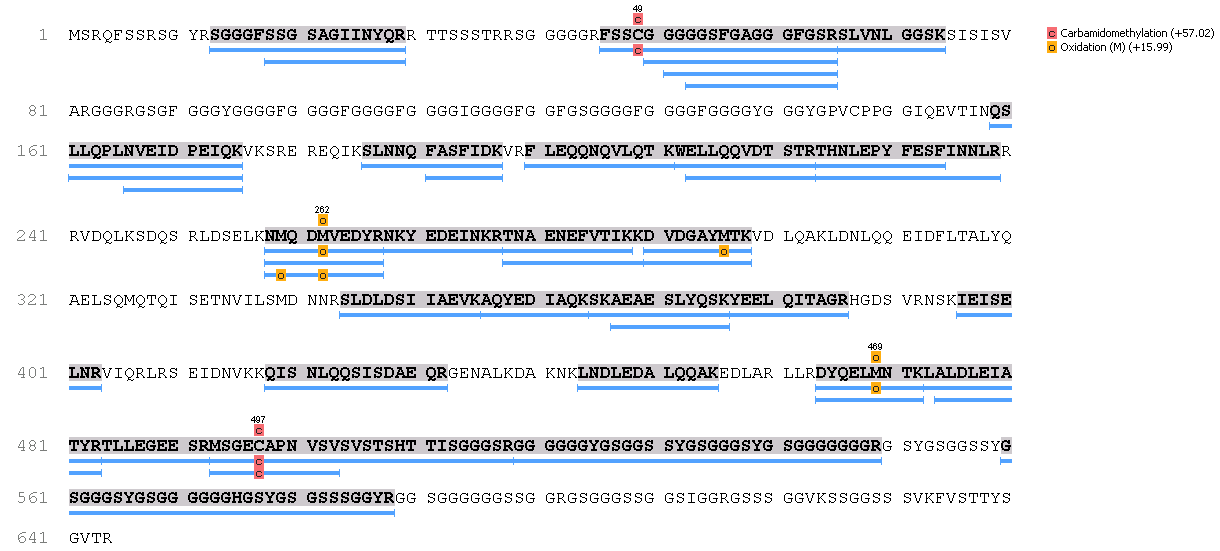

Supplement: S1 Data — (ZIP) [file pntd.0009150.s009.zip › S1 Data/N. naja_Rajasthan/img/cov_1.png]

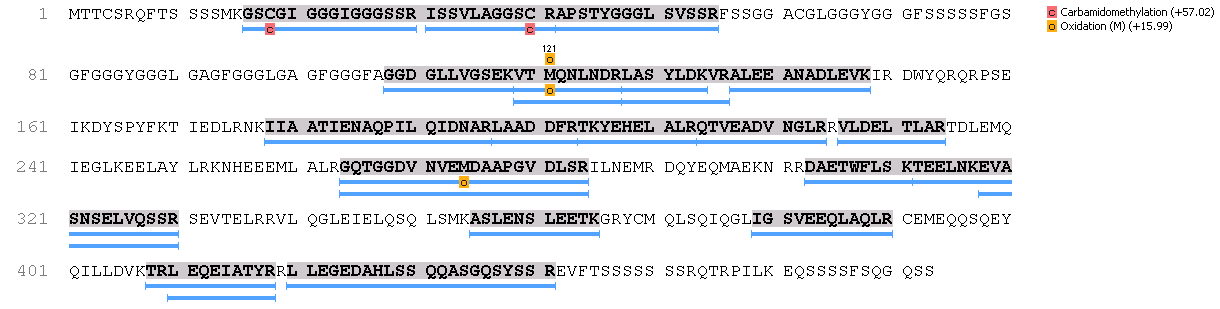

Supplement: S1 Data — (ZIP) [file pntd.0009150.s009.zip › S1 Data/N. naja_Rajasthan/img/cov_10.png]

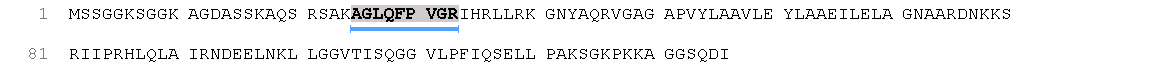

Supplement: S1 Data — (ZIP) [file pntd.0009150.s009.zip › S1 Data/N. naja_Rajasthan/img/cov_1000.png]

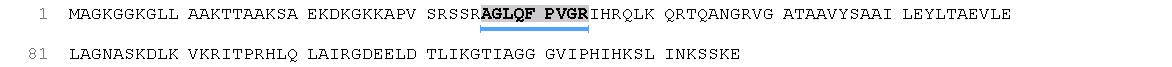

Supplement: S1 Data — (ZIP) [file pntd.0009150.s009.zip › S1 Data/N. naja_Rajasthan/img/cov_1001.png]

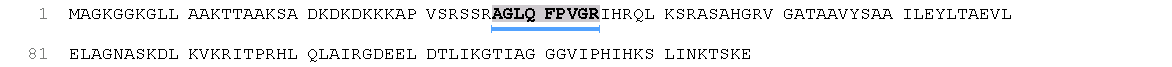

Supplement: S1 Data — (ZIP) [file pntd.0009150.s009.zip › S1 Data/N. naja_Rajasthan/img/cov_1002.png]

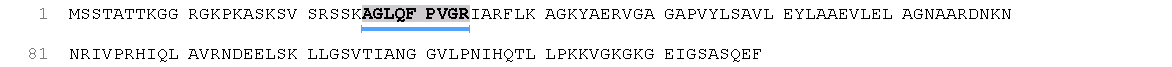

Supplement: S1 Data — (ZIP) [file pntd.0009150.s009.zip › S1 Data/N. naja_Rajasthan/img/cov_1003.png]

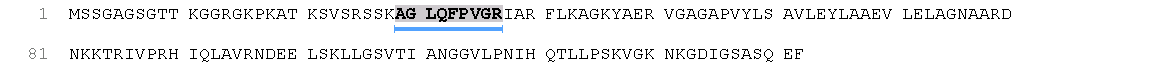

Supplement: S1 Data — (ZIP) [file pntd.0009150.s009.zip › S1 Data/N. naja_Rajasthan/img/cov_1004.png]

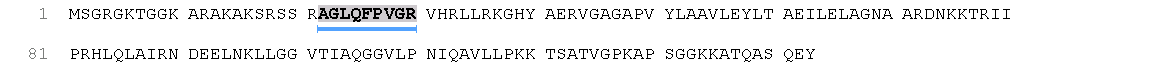

Supplement: S1 Data — (ZIP) [file pntd.0009150.s009.zip › S1 Data/N. naja_Rajasthan/img/cov_1005.png]

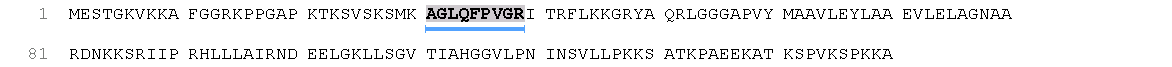

Supplement: S1 Data — (ZIP) [file pntd.0009150.s009.zip › S1 Data/N. naja_Rajasthan/img/cov_1006.png]

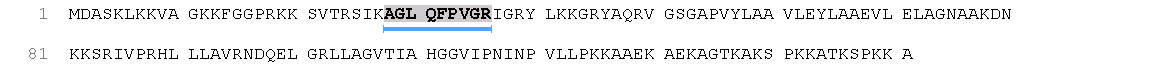

Supplement: S1 Data — (ZIP) [file pntd.0009150.s009.zip › S1 Data/N. naja_Rajasthan/img/cov_1007.png]

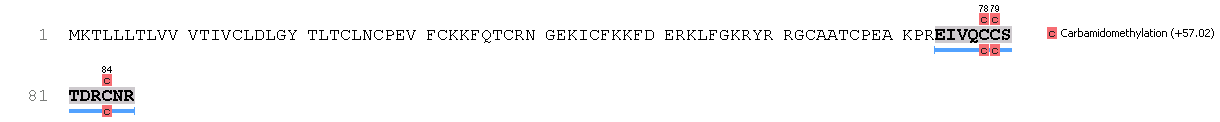

Supplement: S1 Data — (ZIP) [file pntd.0009150.s009.zip › S1 Data/N. naja_Rajasthan/img/cov_1009.png]

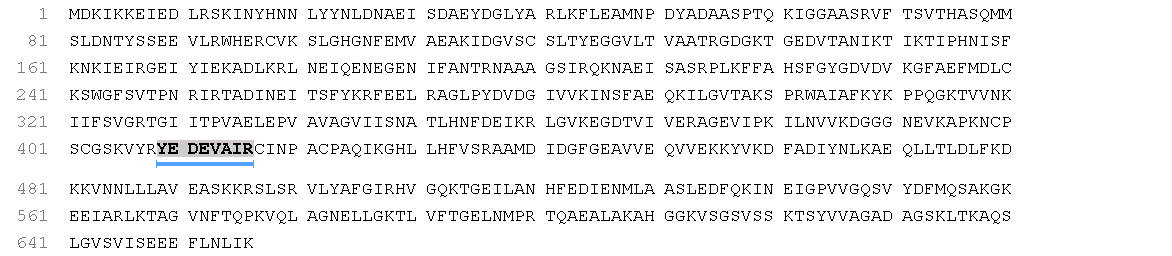

Supplement: S1 Data — (ZIP) [file pntd.0009150.s009.zip › S1 Data/N. naja_Rajasthan/img/cov_1013.png]

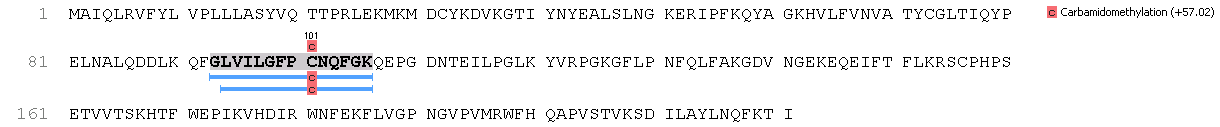

Supplement: S1 Data — (ZIP) [file pntd.0009150.s009.zip › S1 Data/N. naja_Rajasthan/img/cov_1014.png]

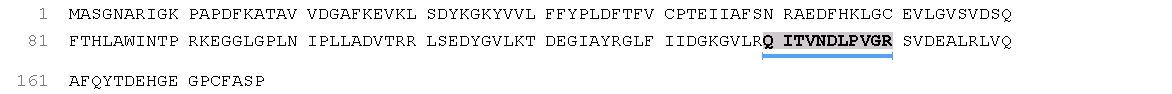

Supplement: S1 Data — (ZIP) [file pntd.0009150.s009.zip › S1 Data/N. naja_Rajasthan/img/cov_1015.png]

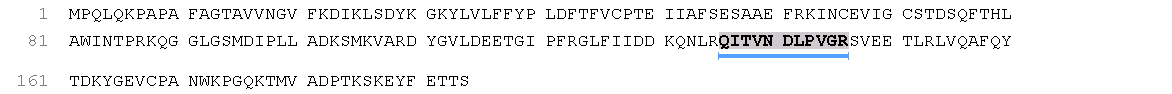

Supplement: S1 Data — (ZIP) [file pntd.0009150.s009.zip › S1 Data/N. naja_Rajasthan/img/cov_1016.png]

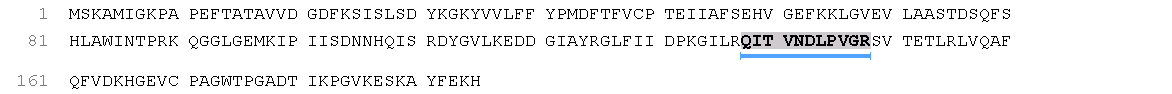

Supplement: S1 Data — (ZIP) [file pntd.0009150.s009.zip › S1 Data/N. naja_Rajasthan/img/cov_1017.png]

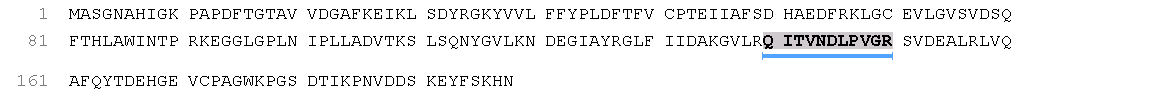

Supplement: S1 Data — (ZIP) [file pntd.0009150.s009.zip › S1 Data/N. naja_Rajasthan/img/cov_1018.png]

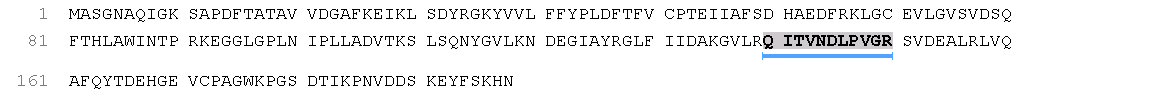

Supplement: S1 Data — (ZIP) [file pntd.0009150.s009.zip › S1 Data/N. naja_Rajasthan/img/cov_1019.png]

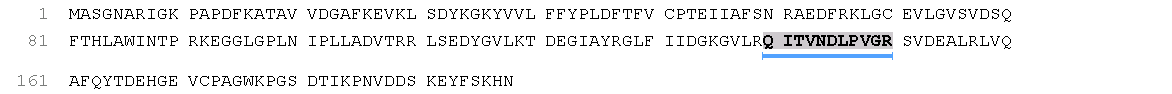

Supplement: S1 Data — (ZIP) [file pntd.0009150.s009.zip › S1 Data/N. naja_Rajasthan/img/cov_1020.png]

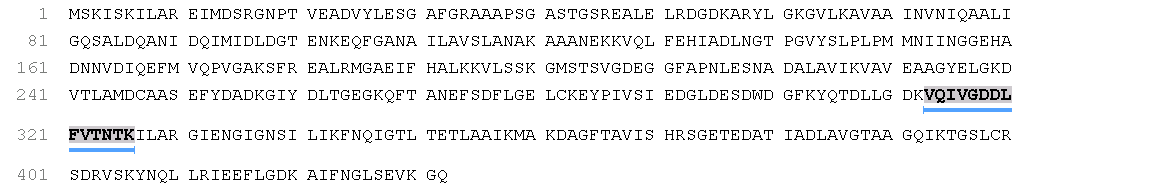

Supplement: S1 Data — (ZIP) [file pntd.0009150.s009.zip › S1 Data/N. naja_Rajasthan/img/cov_1025.png]

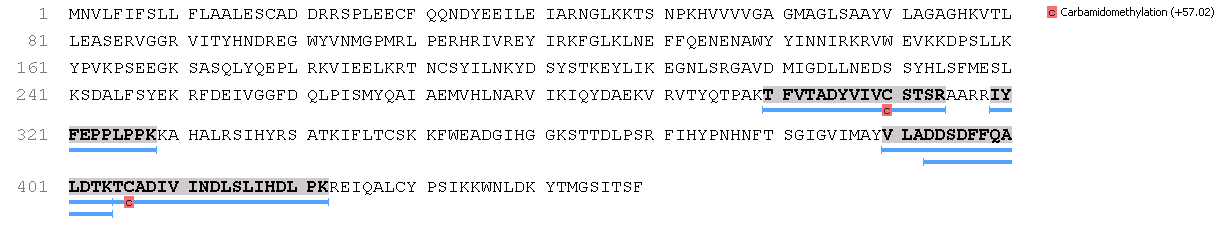

Supplement: S1 Data — (ZIP) [file pntd.0009150.s009.zip › S1 Data/N. naja_Rajasthan/img/cov_107.png]

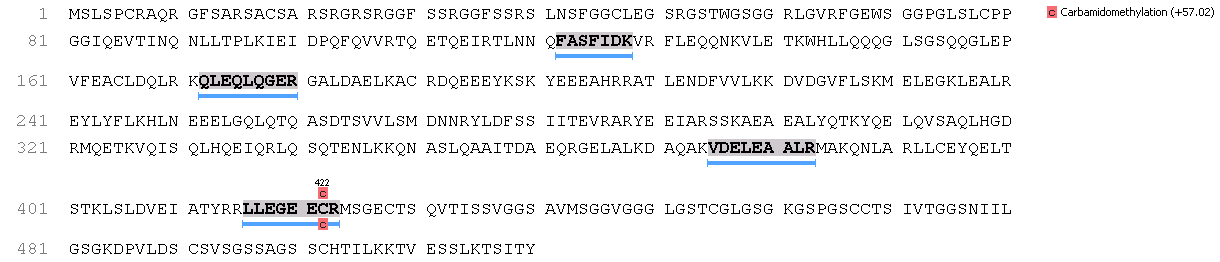

Supplement: S1 Data — (ZIP) [file pntd.0009150.s009.zip › S1 Data/N. naja_Rajasthan/img/cov_108.png]

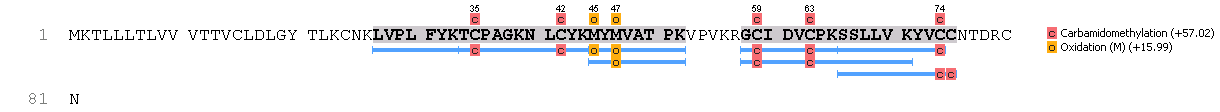

Supplement: S1 Data — (ZIP) [file pntd.0009150.s009.zip › S1 Data/N. naja_Rajasthan/img/cov_113.png]
