# Supplementary figures and images for: Biogeographical venom variation in the Indian spectacled cobra (Naja naja) underscores the pressing need for pan-India efficacious snakebite therapy (part 1 of 2)
Source: PLoS Negl Trop Dis. 2021 Feb 18;15(2):e0009150. doi: 10.1371/journal.pntd.0009150 (PMC7924803; doi:10.1371/journal.pntd.0009150)

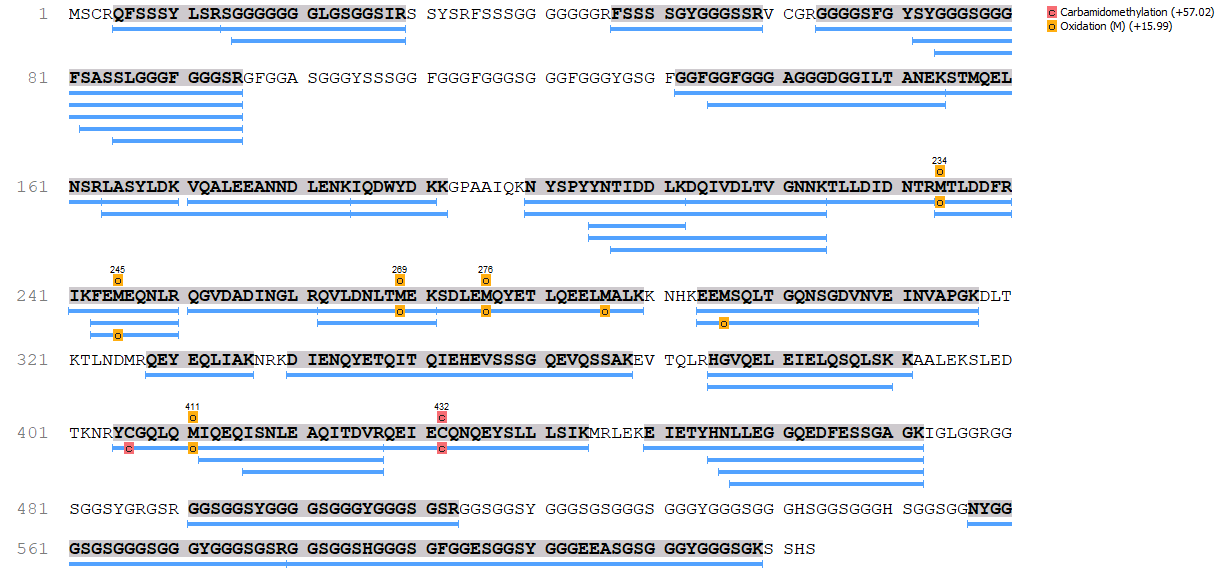

Supplement: S1 Data — (ZIP) [file pntd.0009150.s009.zip › S1 Data/N. naja_Punjab/img/cov_1.png]

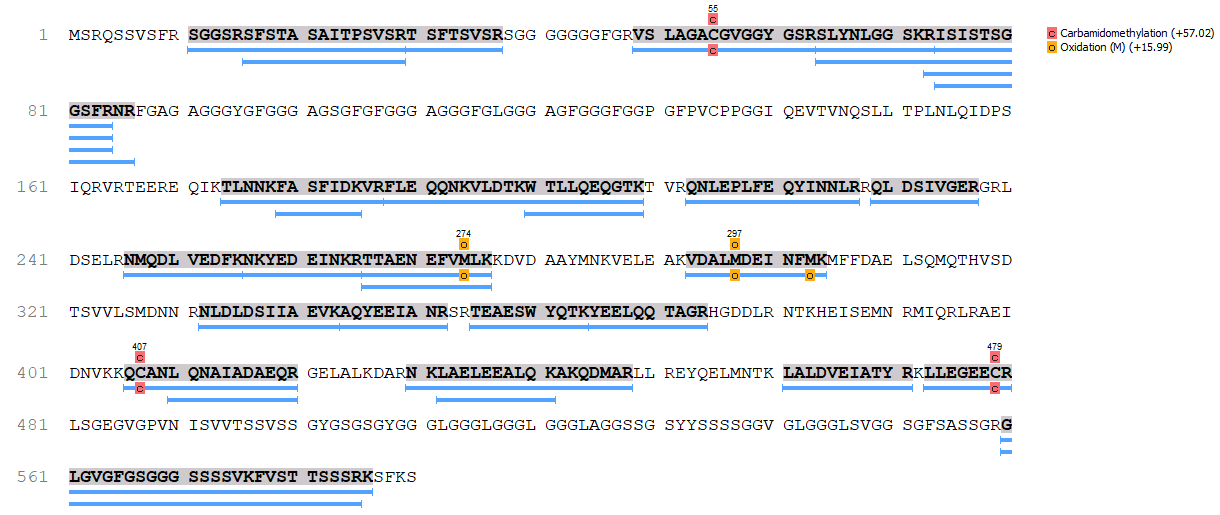

Supplement: S1 Data — (ZIP) [file pntd.0009150.s009.zip › S1 Data/N. naja_Punjab/img/cov_10.png]

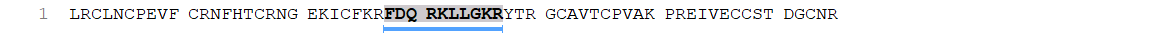

Supplement: S1 Data — (ZIP) [file pntd.0009150.s009.zip › S1 Data/N. naja_Punjab/img/cov_1020.png]

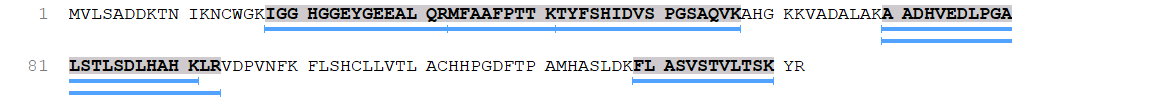

Supplement: S1 Data — (ZIP) [file pntd.0009150.s009.zip › S1 Data/N. naja_Punjab/img/cov_106.png]

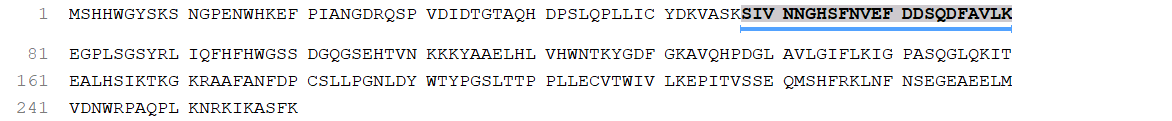

Supplement: S1 Data — (ZIP) [file pntd.0009150.s009.zip › S1 Data/N. naja_Punjab/img/cov_1063.png]

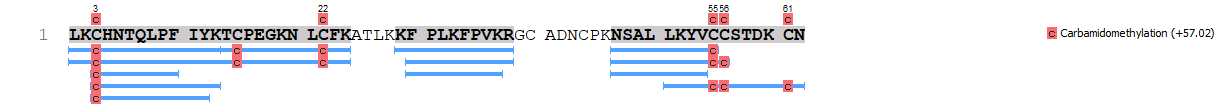

Supplement: S1 Data — (ZIP) [file pntd.0009150.s009.zip › S1 Data/N. naja_Punjab/img/cov_107.png]

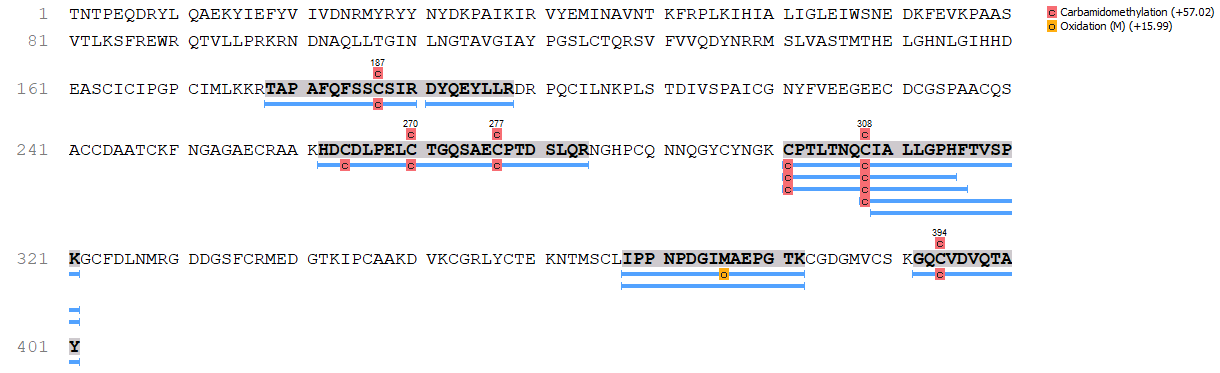

Supplement: S1 Data — (ZIP) [file pntd.0009150.s009.zip › S1 Data/N. naja_Punjab/img/cov_108.png]

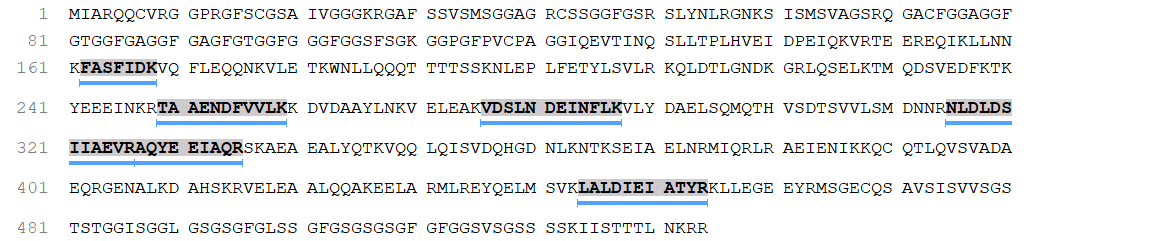

Supplement: S1 Data — (ZIP) [file pntd.0009150.s009.zip › S1 Data/N. naja_Punjab/img/cov_109.png]

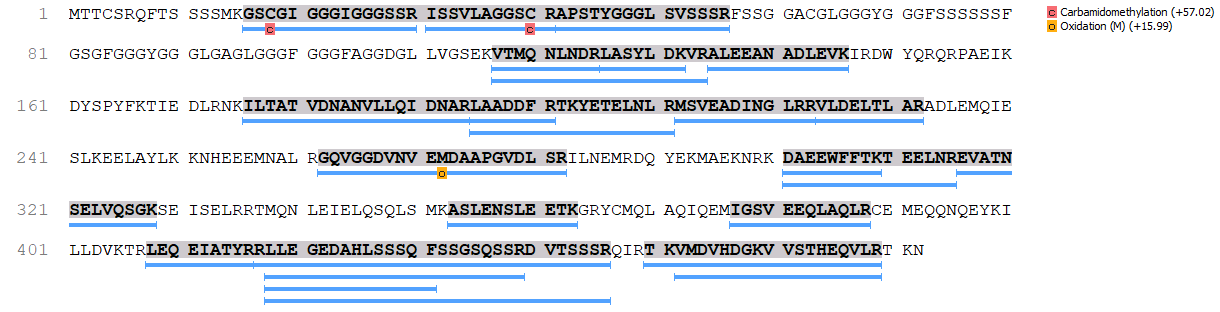

Supplement: S1 Data — (ZIP) [file pntd.0009150.s009.zip › S1 Data/N. naja_Punjab/img/cov_11.png]

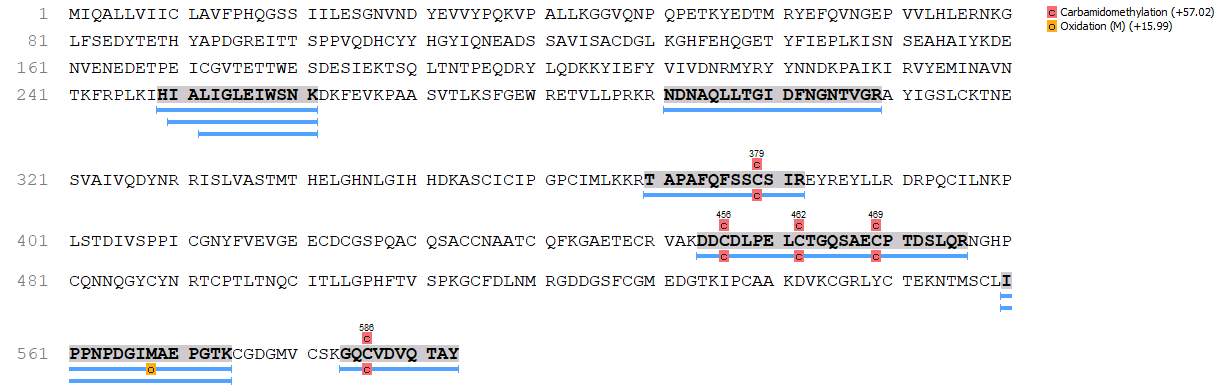

Supplement: S1 Data — (ZIP) [file pntd.0009150.s009.zip › S1 Data/N. naja_Punjab/img/cov_115.png]

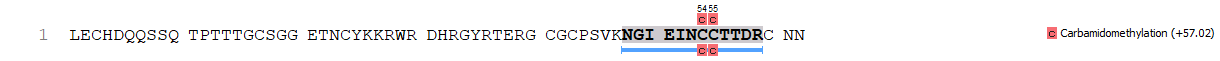

Supplement: S1 Data — (ZIP) [file pntd.0009150.s009.zip › S1 Data/N. naja_Punjab/img/cov_1156.png]

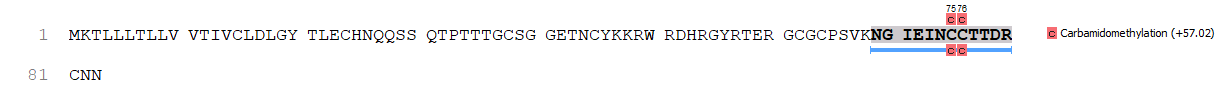

Supplement: S1 Data — (ZIP) [file pntd.0009150.s009.zip › S1 Data/N. naja_Punjab/img/cov_1179.png]

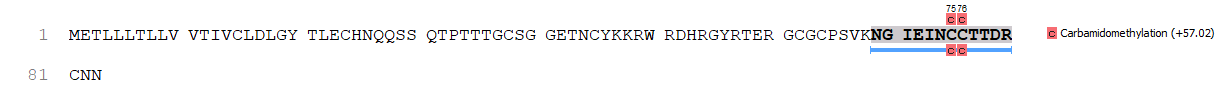

Supplement: S1 Data — (ZIP) [file pntd.0009150.s009.zip › S1 Data/N. naja_Punjab/img/cov_1181.png]

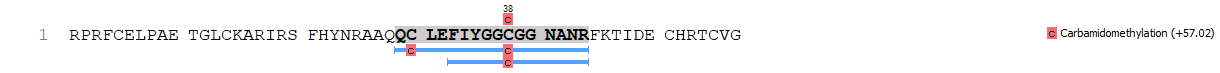

Supplement: S1 Data — (ZIP) [file pntd.0009150.s009.zip › S1 Data/N. naja_Punjab/img/cov_1184.png]

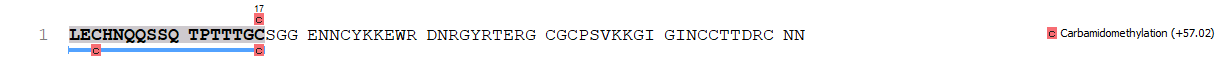

Supplement: S1 Data — (ZIP) [file pntd.0009150.s009.zip › S1 Data/N. naja_Punjab/img/cov_1196.png]

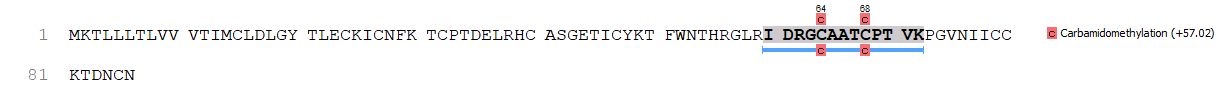

Supplement: S1 Data — (ZIP) [file pntd.0009150.s009.zip › S1 Data/N. naja_Punjab/img/cov_1207.png]

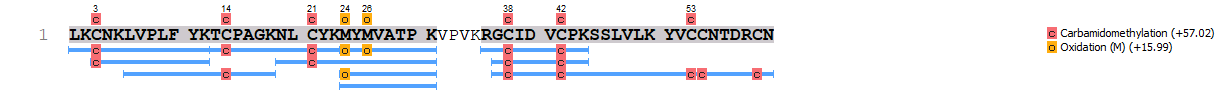

Supplement: S1 Data — (ZIP) [file pntd.0009150.s009.zip › S1 Data/N. naja_Punjab/img/cov_121.png]

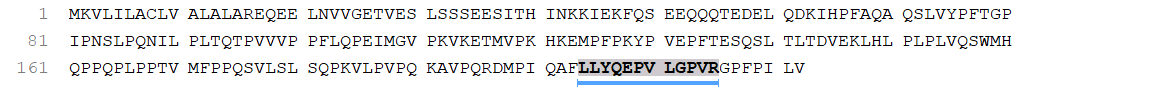

Supplement: S1 Data — (ZIP) [file pntd.0009150.s009.zip › S1 Data/N. naja_Punjab/img/cov_1213.png]

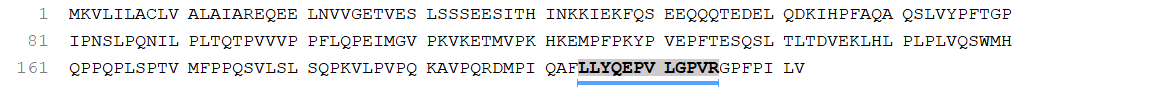

Supplement: S1 Data — (ZIP) [file pntd.0009150.s009.zip › S1 Data/N. naja_Punjab/img/cov_1214.png]

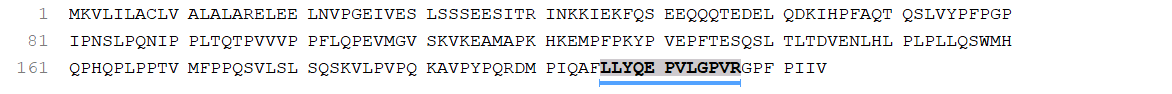

Supplement: S1 Data — (ZIP) [file pntd.0009150.s009.zip › S1 Data/N. naja_Punjab/img/cov_1215.png]

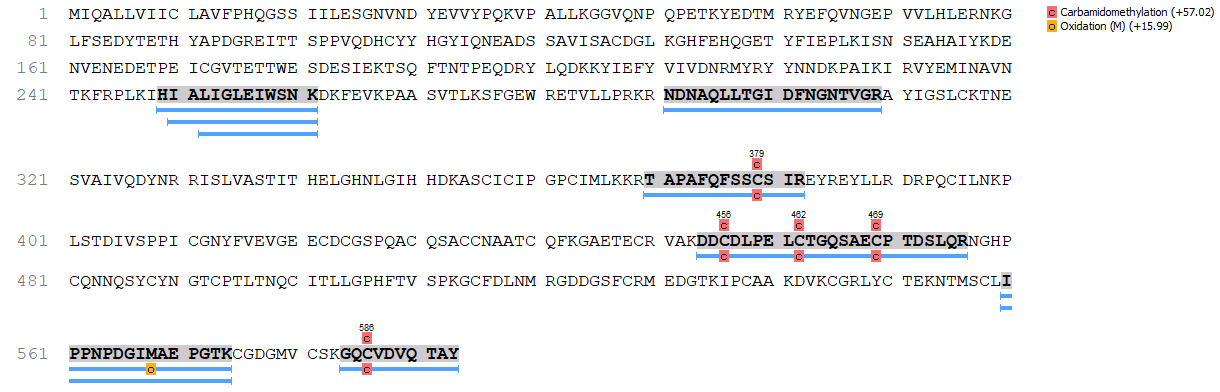

Supplement: S1 Data — (ZIP) [file pntd.0009150.s009.zip › S1 Data/N. naja_Punjab/img/cov_122.png]

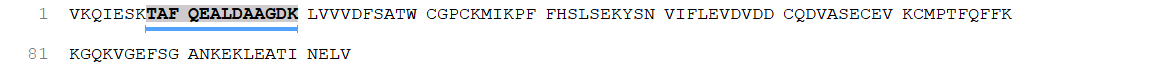

Supplement: S1 Data — (ZIP) [file pntd.0009150.s009.zip › S1 Data/N. naja_Punjab/img/cov_1222.png]

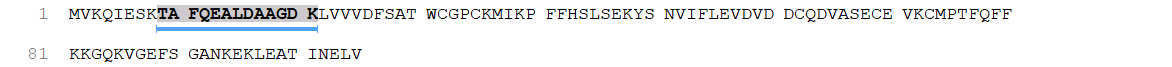

Supplement: S1 Data — (ZIP) [file pntd.0009150.s009.zip › S1 Data/N. naja_Punjab/img/cov_1223.png]

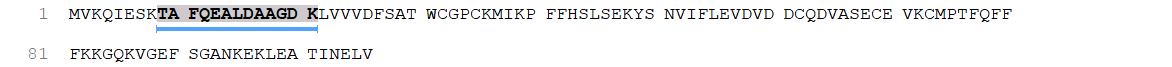

Supplement: S1 Data — (ZIP) [file pntd.0009150.s009.zip › S1 Data/N. naja_Punjab/img/cov_1224.png]

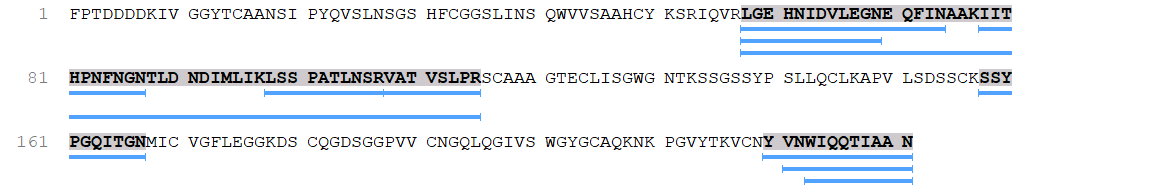

Supplement: S1 Data — (ZIP) [file pntd.0009150.s009.zip › S1 Data/N. naja_Punjab/img/cov_124.png]

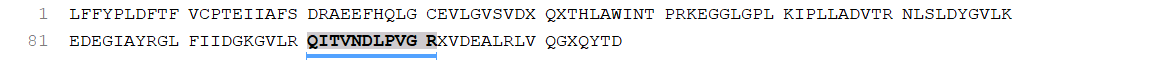

Supplement: S1 Data — (ZIP) [file pntd.0009150.s009.zip › S1 Data/N. naja_Punjab/img/cov_1255.png]

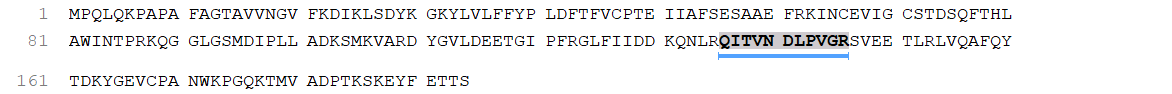

Supplement: S1 Data — (ZIP) [file pntd.0009150.s009.zip › S1 Data/N. naja_Punjab/img/cov_1256.png]

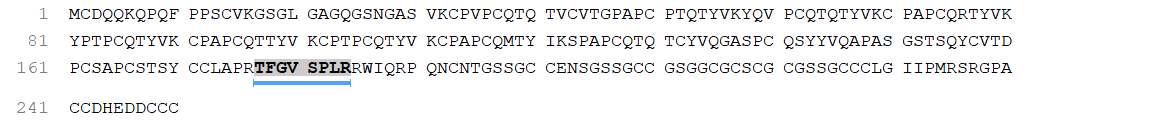

Supplement: S1 Data — (ZIP) [file pntd.0009150.s009.zip › S1 Data/N. naja_Punjab/img/cov_1259.png]

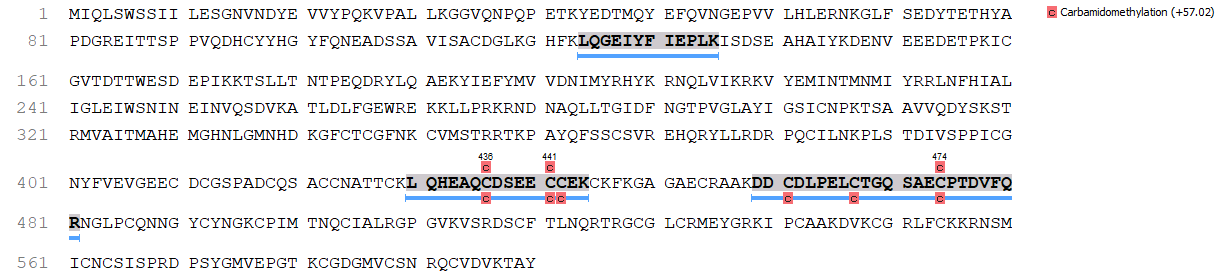

Supplement: S1 Data — (ZIP) [file pntd.0009150.s009.zip › S1 Data/N. naja_Punjab/img/cov_127.png]

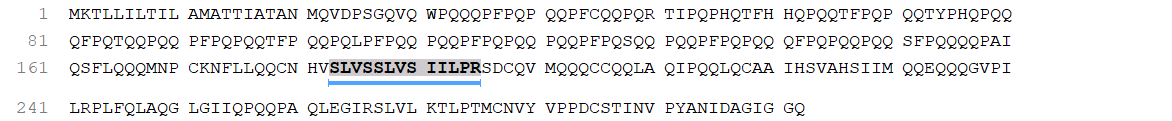

Supplement: S1 Data — (ZIP) [file pntd.0009150.s009.zip › S1 Data/N. naja_Punjab/img/cov_1272.png]

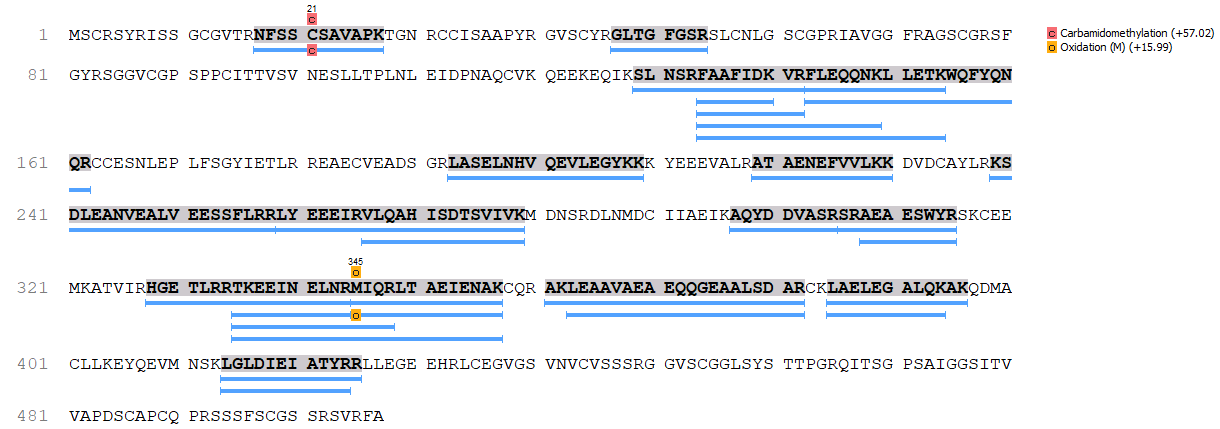

Supplement: S1 Data — (ZIP) [file pntd.0009150.s009.zip › S1 Data/N. naja_Punjab/img/cov_13.png]

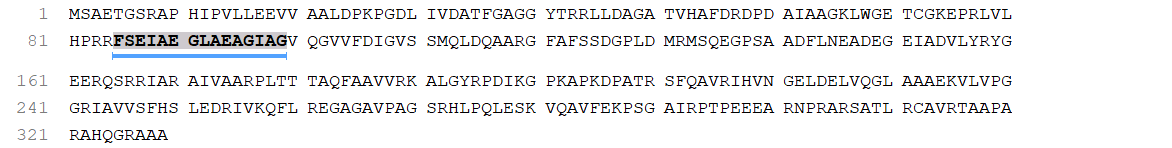

Supplement: S1 Data — (ZIP) [file pntd.0009150.s009.zip › S1 Data/N. naja_Punjab/img/cov_1378.png]

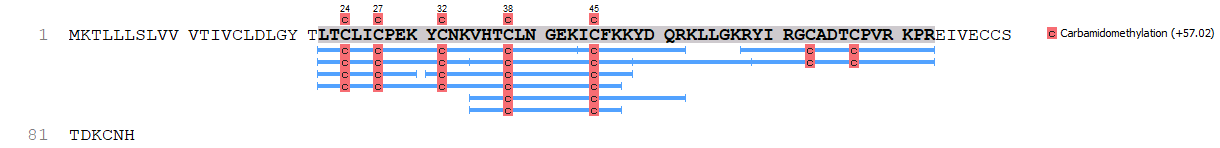

Supplement: S1 Data — (ZIP) [file pntd.0009150.s009.zip › S1 Data/N. naja_Punjab/img/cov_140.png]

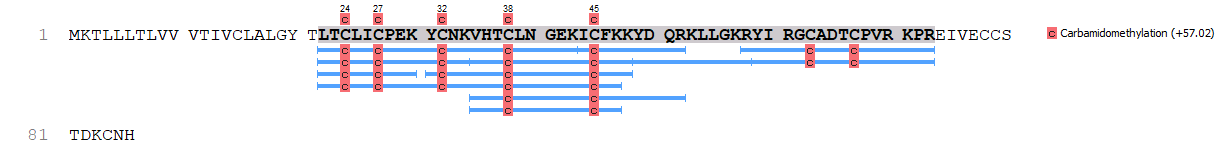

Supplement: S1 Data — (ZIP) [file pntd.0009150.s009.zip › S1 Data/N. naja_Punjab/img/cov_141.png]

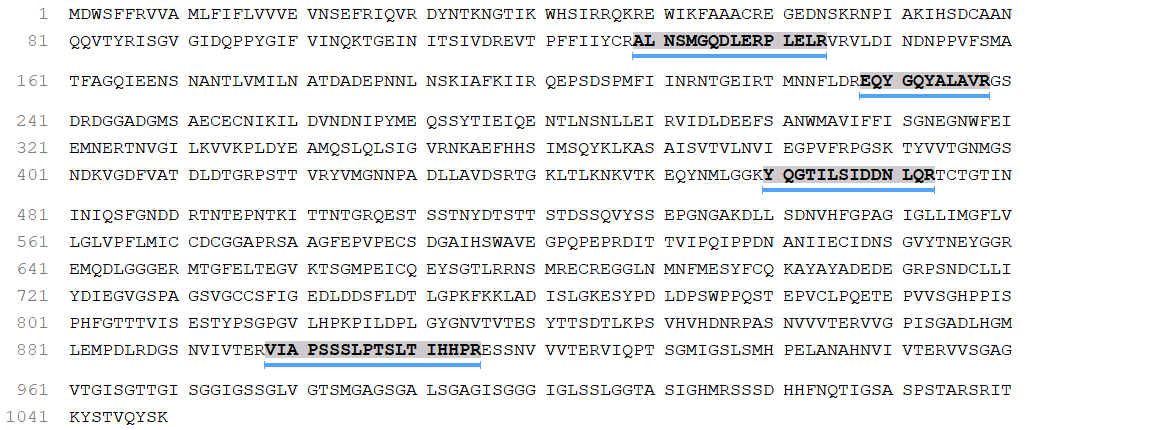

Supplement: S1 Data — (ZIP) [file pntd.0009150.s009.zip › S1 Data/N. naja_Punjab/img/cov_142.png]

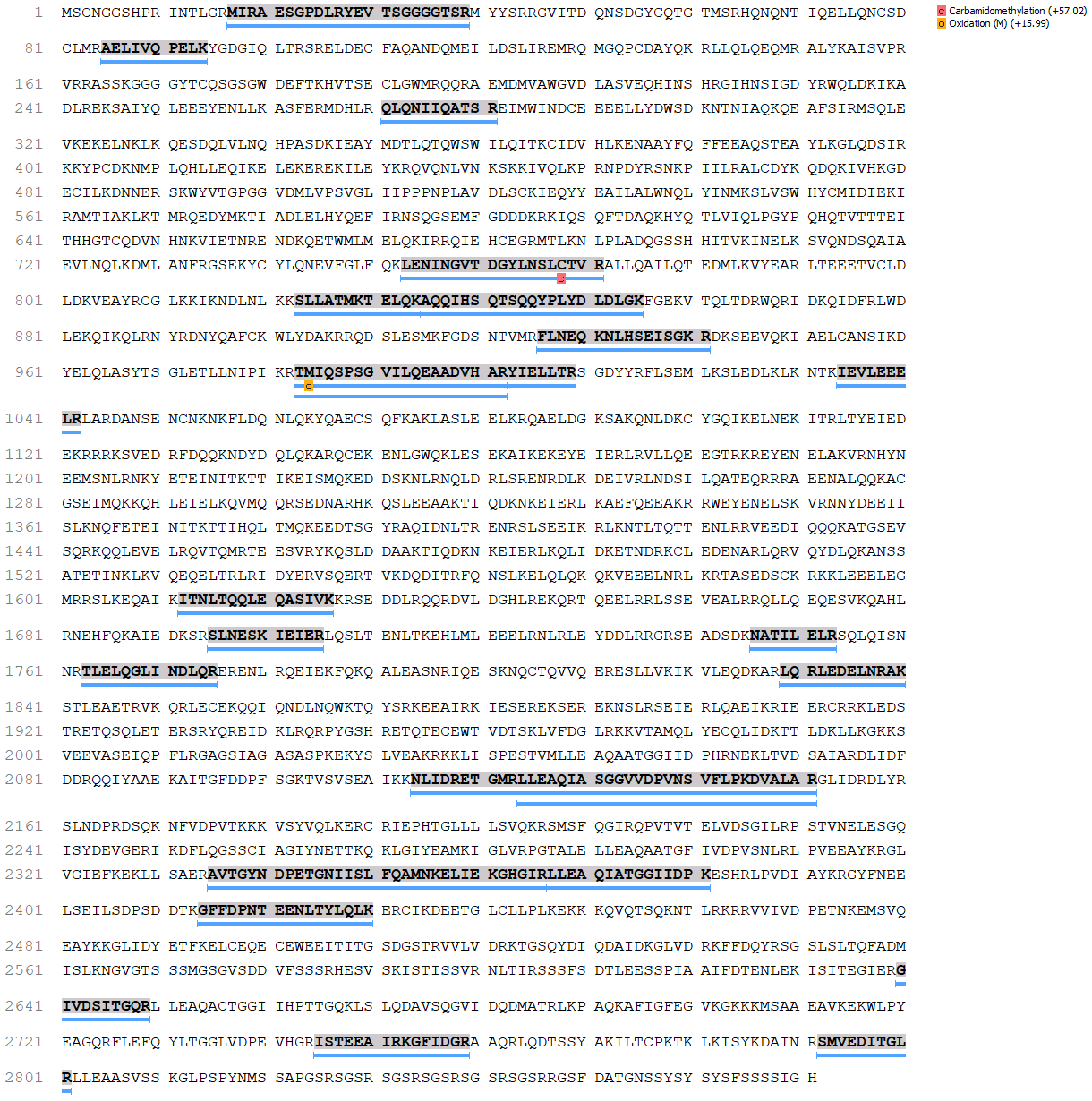

Supplement: S1 Data — (ZIP) [file pntd.0009150.s009.zip › S1 Data/N. naja_Punjab/img/cov_15.png]

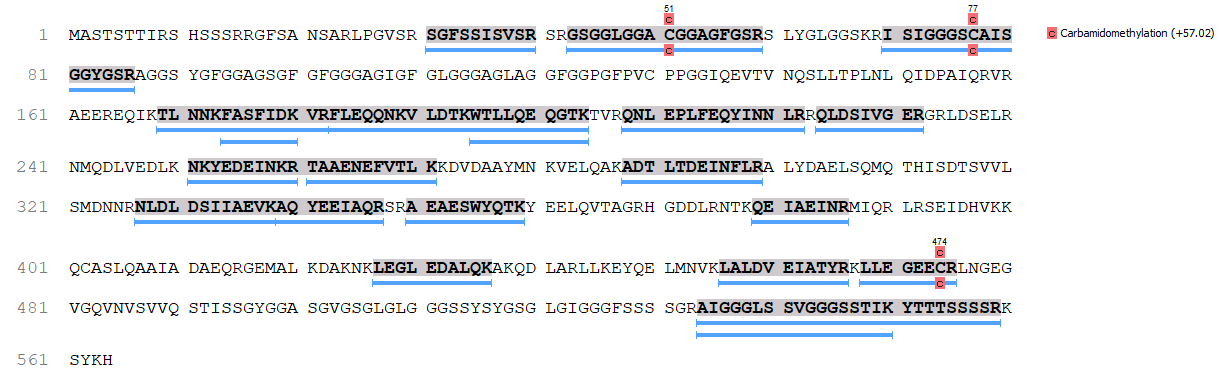

Supplement: S1 Data — (ZIP) [file pntd.0009150.s009.zip › S1 Data/N. naja_Punjab/img/cov_17.png]

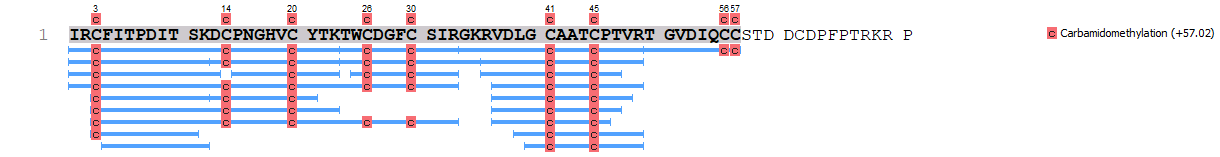

Supplement: S1 Data — (ZIP) [file pntd.0009150.s009.zip › S1 Data/N. naja_Punjab/img/cov_185.png]

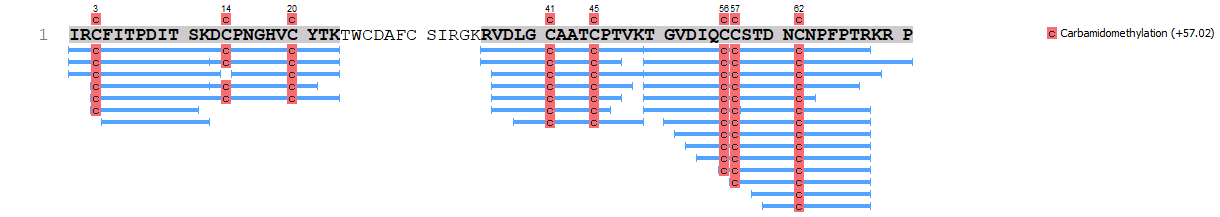

Supplement: S1 Data — (ZIP) [file pntd.0009150.s009.zip › S1 Data/N. naja_Punjab/img/cov_186.png]

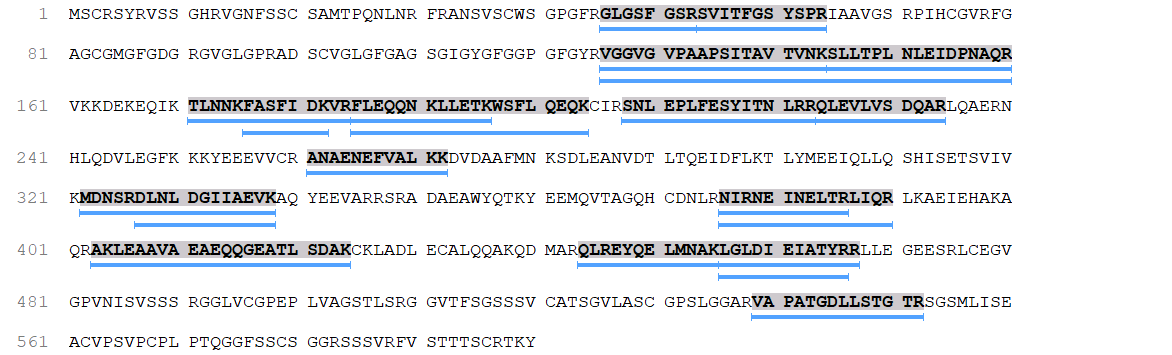

Supplement: S1 Data — (ZIP) [file pntd.0009150.s009.zip › S1 Data/N. naja_Punjab/img/cov_19.png]

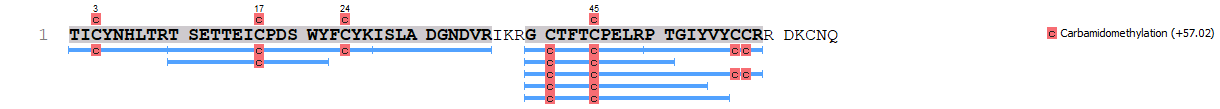

Supplement: S1 Data — (ZIP) [file pntd.0009150.s009.zip › S1 Data/N. naja_Punjab/img/cov_190.png]

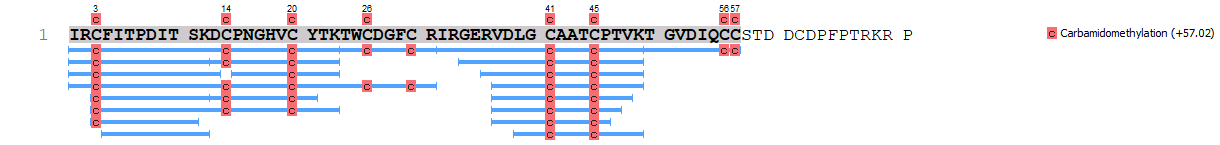

Supplement: S1 Data — (ZIP) [file pntd.0009150.s009.zip › S1 Data/N. naja_Punjab/img/cov_191.png]

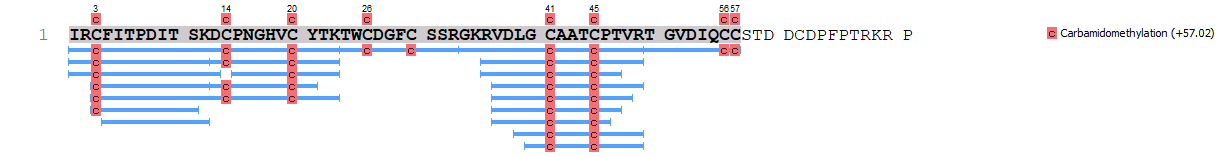

Supplement: S1 Data — (ZIP) [file pntd.0009150.s009.zip › S1 Data/N. naja_Punjab/img/cov_193.png]

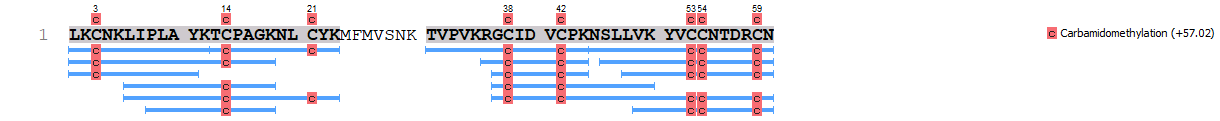

Supplement: S1 Data — (ZIP) [file pntd.0009150.s009.zip › S1 Data/N. naja_Punjab/img/cov_197.png]

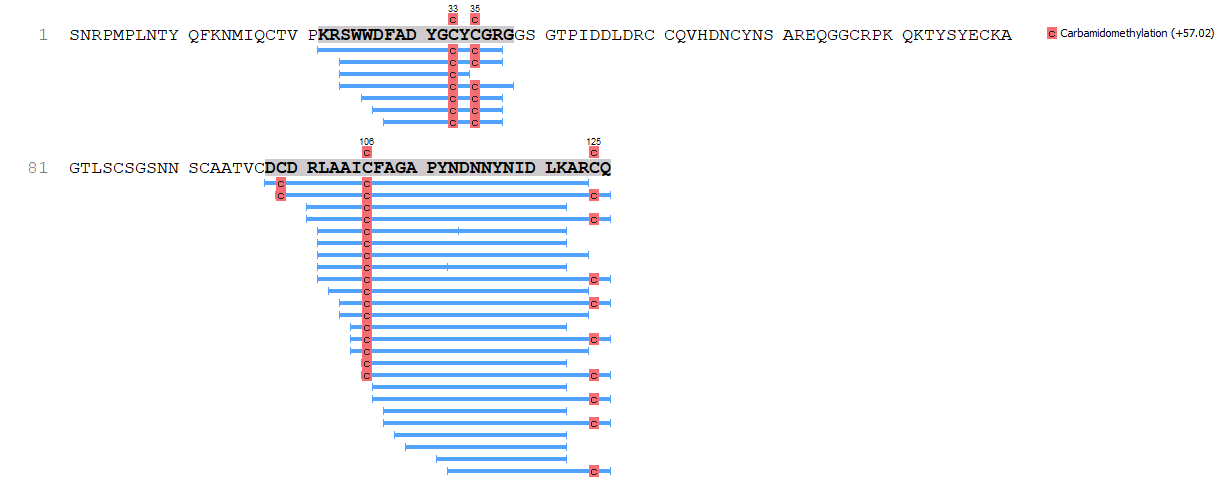

Supplement: S1 Data — (ZIP) [file pntd.0009150.s009.zip › S1 Data/N. naja_Punjab/img/cov_198.png]

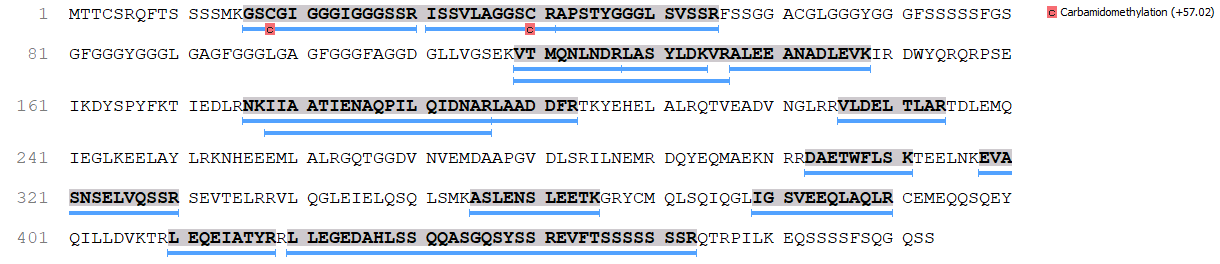

Supplement: S1 Data — (ZIP) [file pntd.0009150.s009.zip › S1 Data/N. naja_Punjab/img/cov_20.png]

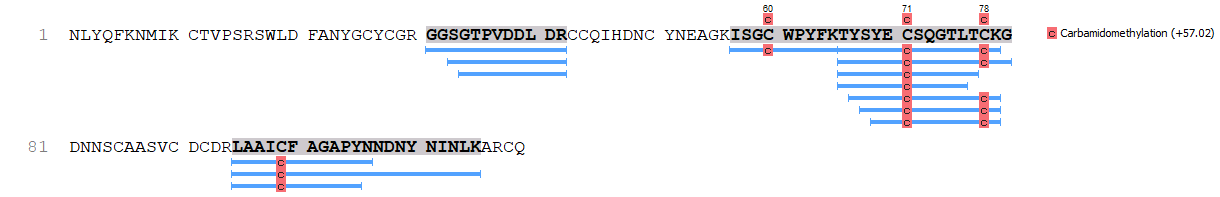

Supplement: S1 Data — (ZIP) [file pntd.0009150.s009.zip › S1 Data/N. naja_Punjab/img/cov_205.png]

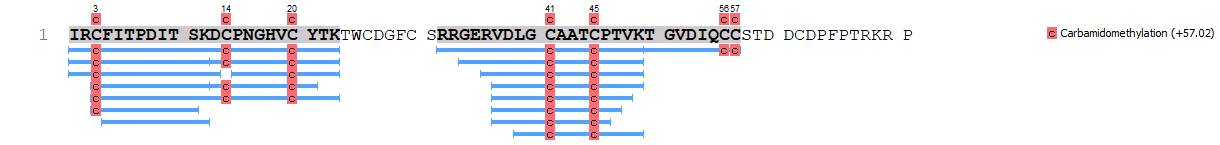

Supplement: S1 Data — (ZIP) [file pntd.0009150.s009.zip › S1 Data/N. naja_Punjab/img/cov_208.png]

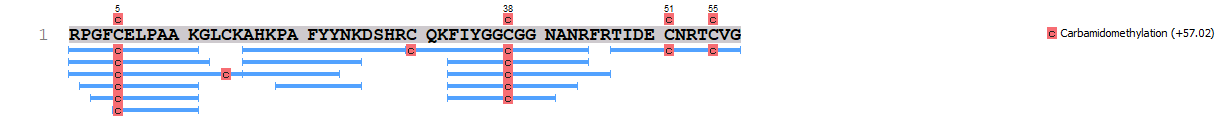

Supplement: S1 Data — (ZIP) [file pntd.0009150.s009.zip › S1 Data/N. naja_Punjab/img/cov_212.png]

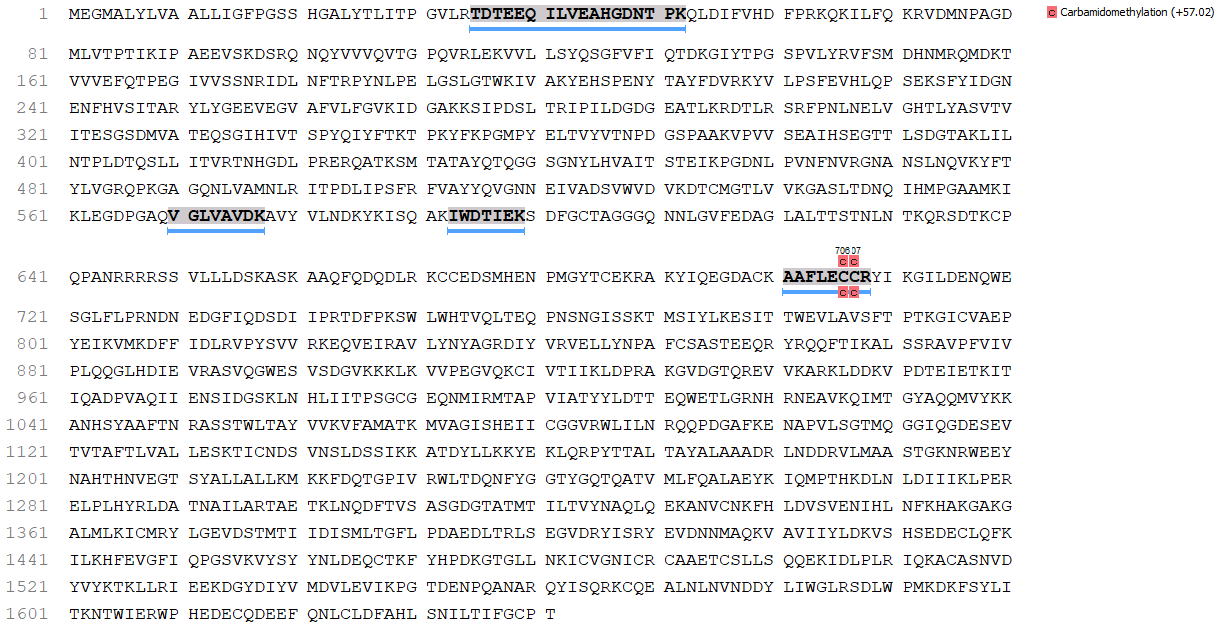

Supplement: S1 Data — (ZIP) [file pntd.0009150.s009.zip › S1 Data/N. naja_Punjab/img/cov_218.png]

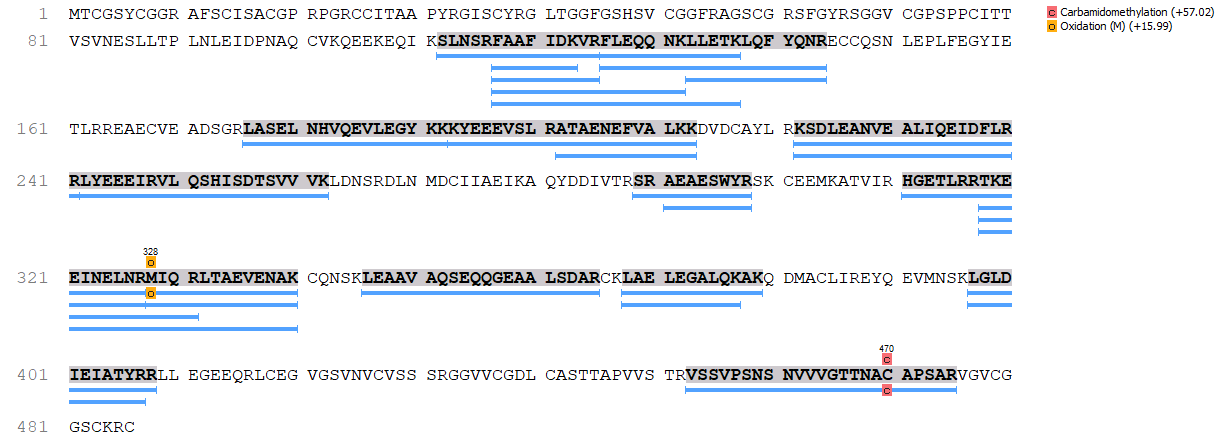

Supplement: S1 Data — (ZIP) [file pntd.0009150.s009.zip › S1 Data/N. naja_Punjab/img/cov_22.png]

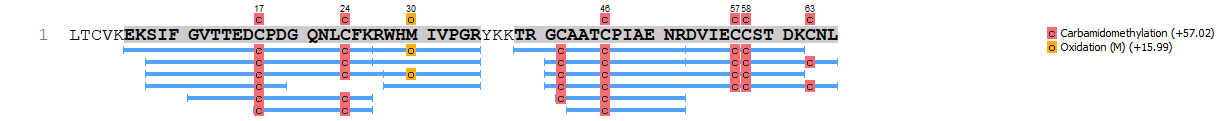

Supplement: S1 Data — (ZIP) [file pntd.0009150.s009.zip › S1 Data/N. naja_Punjab/img/cov_220.png]

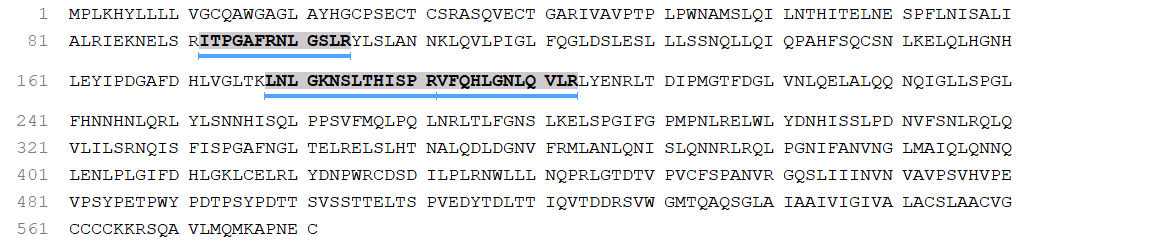

Supplement: S1 Data — (ZIP) [file pntd.0009150.s009.zip › S1 Data/N. naja_Punjab/img/cov_234.png]

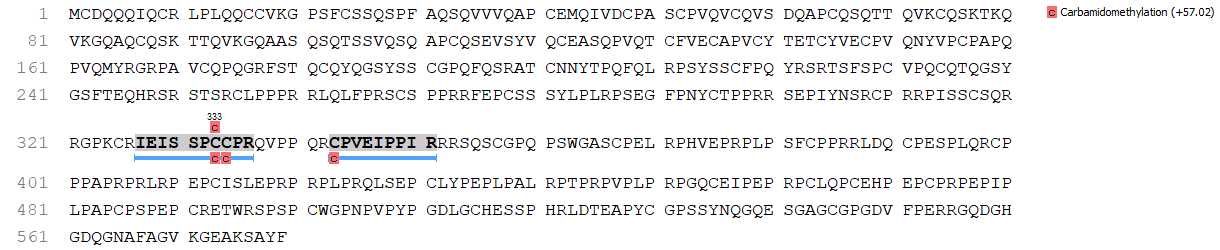

Supplement: S1 Data — (ZIP) [file pntd.0009150.s009.zip › S1 Data/N. naja_Punjab/img/cov_236.png]

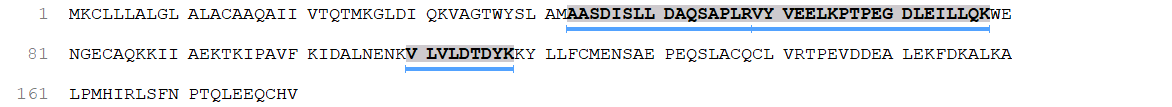

Supplement: S1 Data — (ZIP) [file pntd.0009150.s009.zip › S1 Data/N. naja_Punjab/img/cov_240.png]

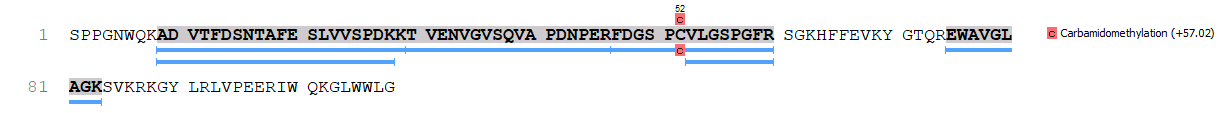

Supplement: S1 Data — (ZIP) [file pntd.0009150.s009.zip › S1 Data/N. naja_Punjab/img/cov_244.png]

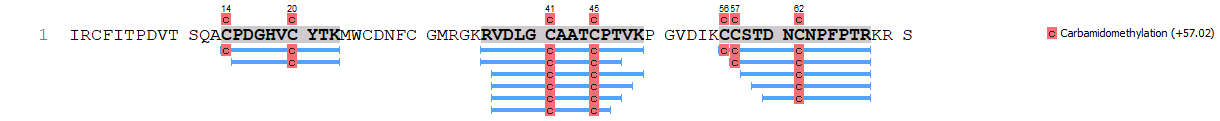

Supplement: S1 Data — (ZIP) [file pntd.0009150.s009.zip › S1 Data/N. naja_Punjab/img/cov_245.png]

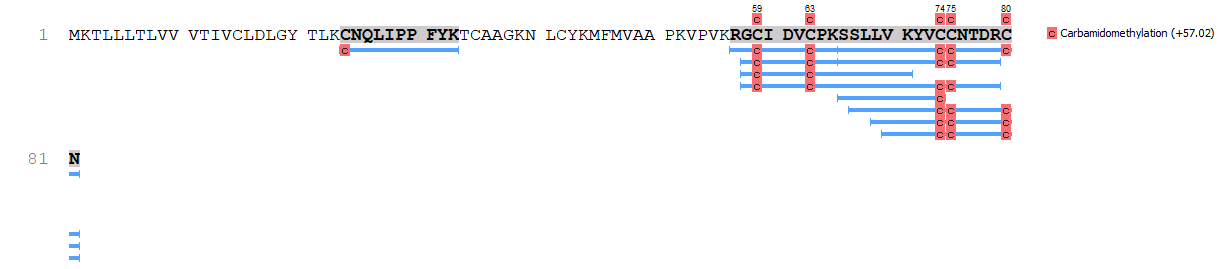

Supplement: S1 Data — (ZIP) [file pntd.0009150.s009.zip › S1 Data/N. naja_Punjab/img/cov_251.png]

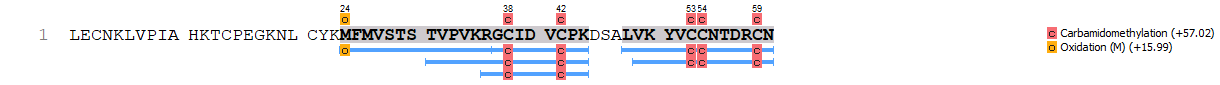

Supplement: S1 Data — (ZIP) [file pntd.0009150.s009.zip › S1 Data/N. naja_Punjab/img/cov_258.png]

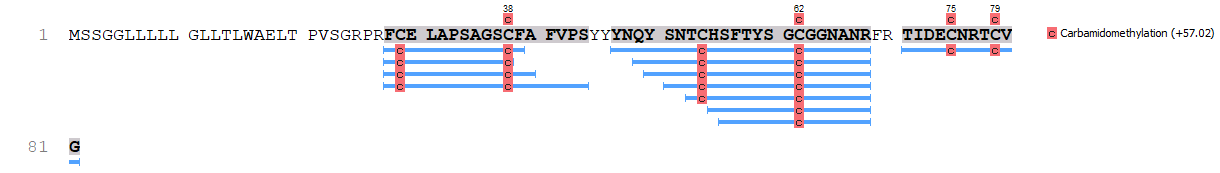

Supplement: S1 Data — (ZIP) [file pntd.0009150.s009.zip › S1 Data/N. naja_Punjab/img/cov_260.png]

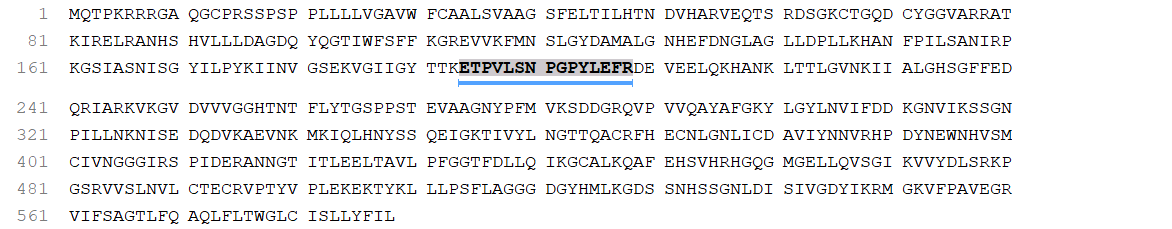

Supplement: S1 Data — (ZIP) [file pntd.0009150.s009.zip › S1 Data/N. naja_Punjab/img/cov_263.png]

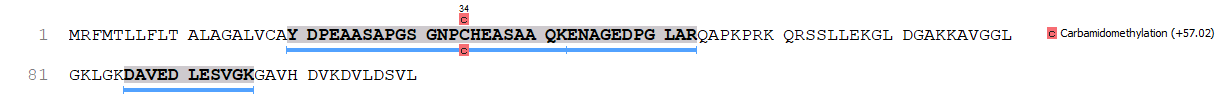

Supplement: S1 Data — (ZIP) [file pntd.0009150.s009.zip › S1 Data/N. naja_Punjab/img/cov_264.png]

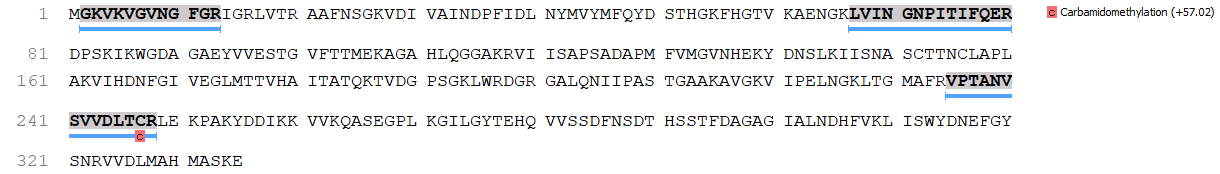

Supplement: S1 Data — (ZIP) [file pntd.0009150.s009.zip › S1 Data/N. naja_Punjab/img/cov_271.png]

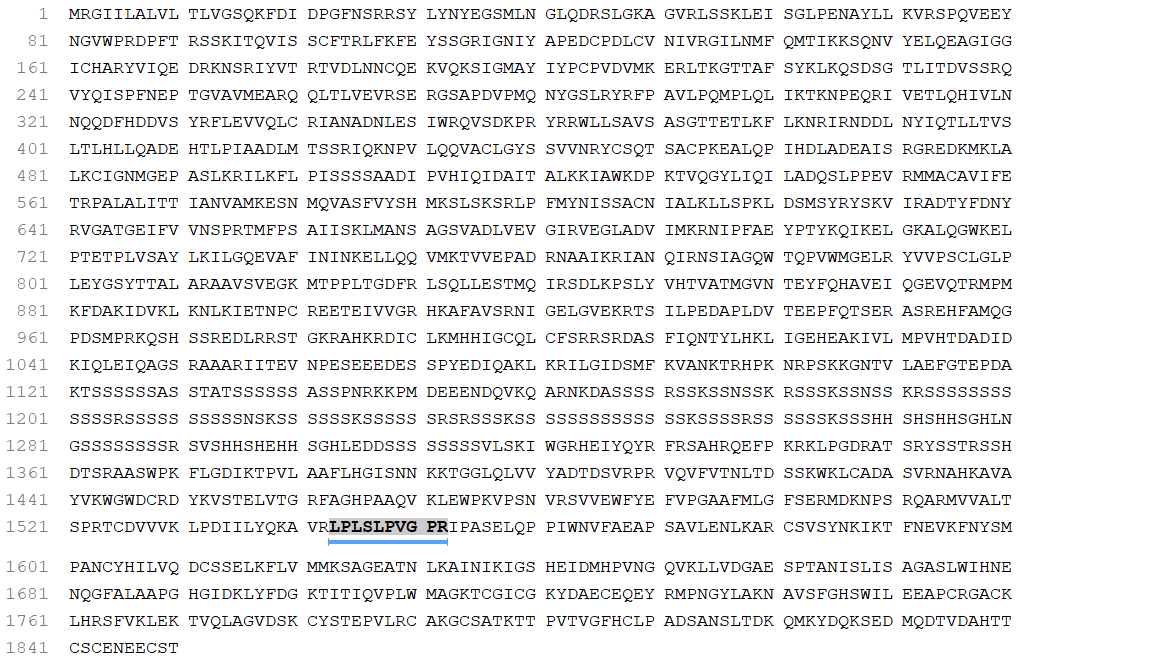

Supplement: S1 Data — (ZIP) [file pntd.0009150.s009.zip › S1 Data/N. naja_Punjab/img/cov_294.png]

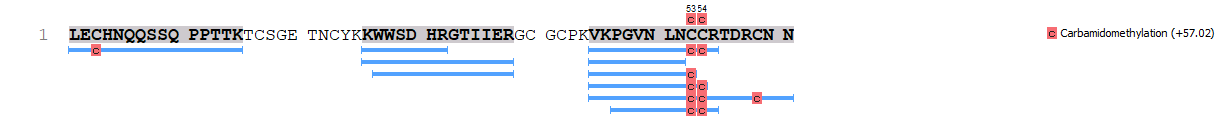

Supplement: S1 Data — (ZIP) [file pntd.0009150.s009.zip › S1 Data/N. naja_Punjab/img/cov_298.png]

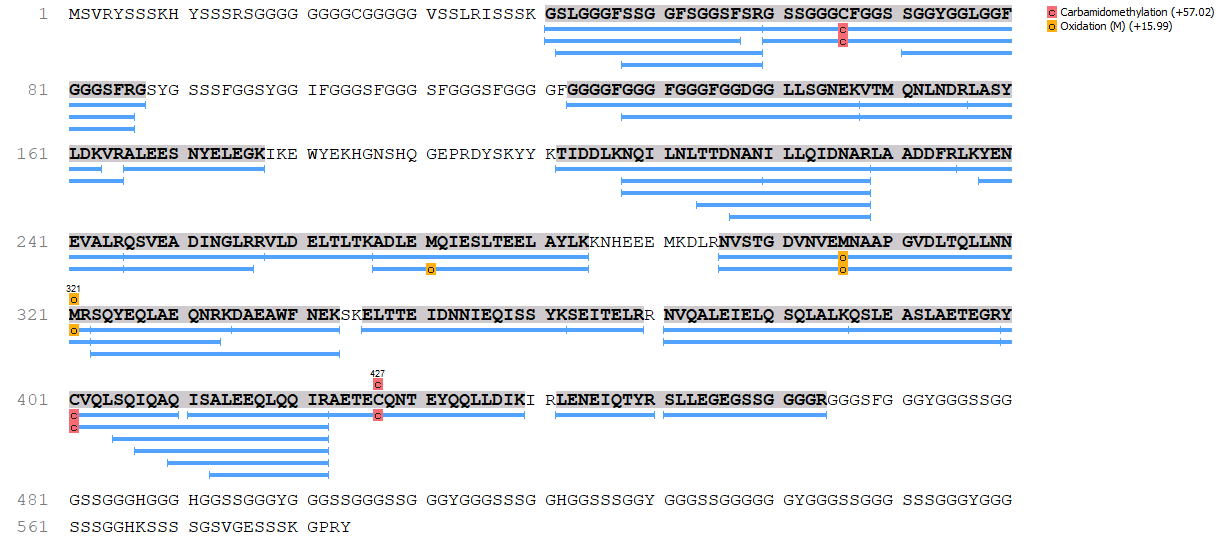

Supplement: S1 Data — (ZIP) [file pntd.0009150.s009.zip › S1 Data/N. naja_Punjab/img/cov_3.png]

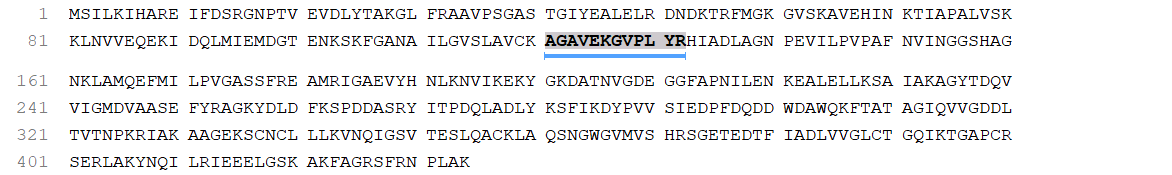

Supplement: S1 Data — (ZIP) [file pntd.0009150.s009.zip › S1 Data/N. naja_Punjab/img/cov_301.png]

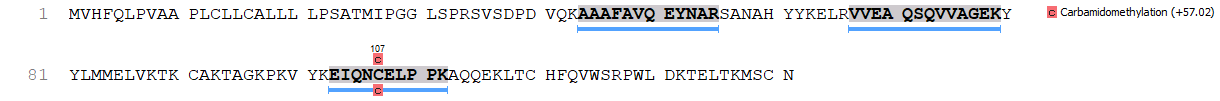

Supplement: S1 Data — (ZIP) [file pntd.0009150.s009.zip › S1 Data/N. naja_Punjab/img/cov_308.png]

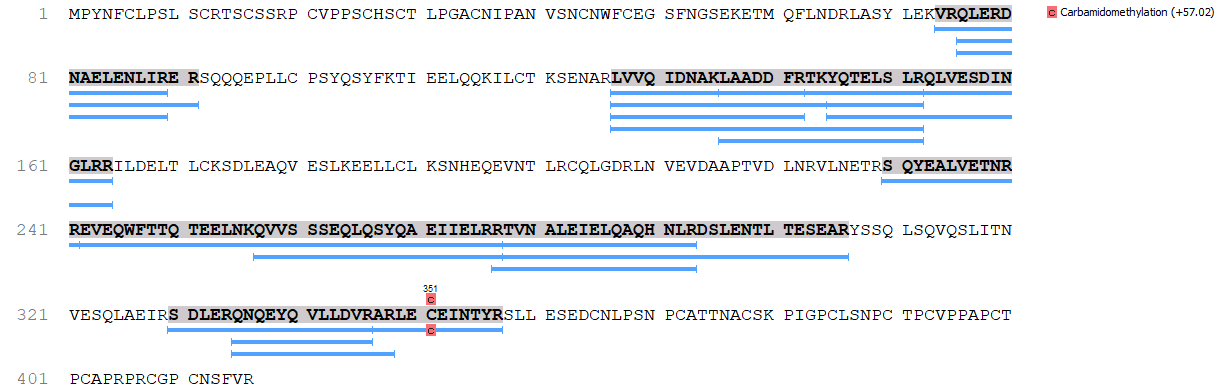

Supplement: S1 Data — (ZIP) [file pntd.0009150.s009.zip › S1 Data/N. naja_Punjab/img/cov_32.png]

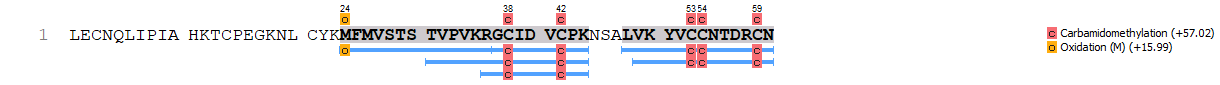

Supplement: S1 Data — (ZIP) [file pntd.0009150.s009.zip › S1 Data/N. naja_Punjab/img/cov_325.png]

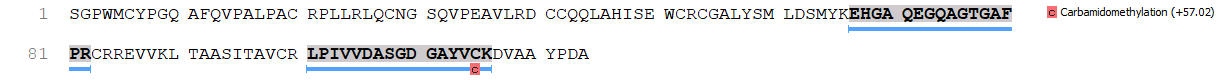

Supplement: S1 Data — (ZIP) [file pntd.0009150.s009.zip › S1 Data/N. naja_Punjab/img/cov_329.png]

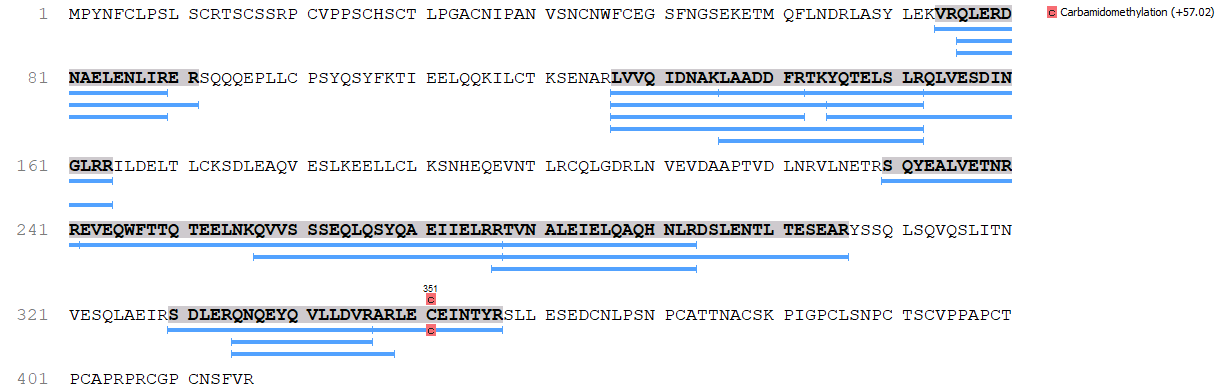

Supplement: S1 Data — (ZIP) [file pntd.0009150.s009.zip › S1 Data/N. naja_Punjab/img/cov_33.png]

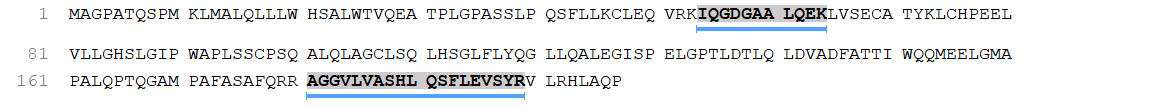

Supplement: S1 Data — (ZIP) [file pntd.0009150.s009.zip › S1 Data/N. naja_Punjab/img/cov_336.png]

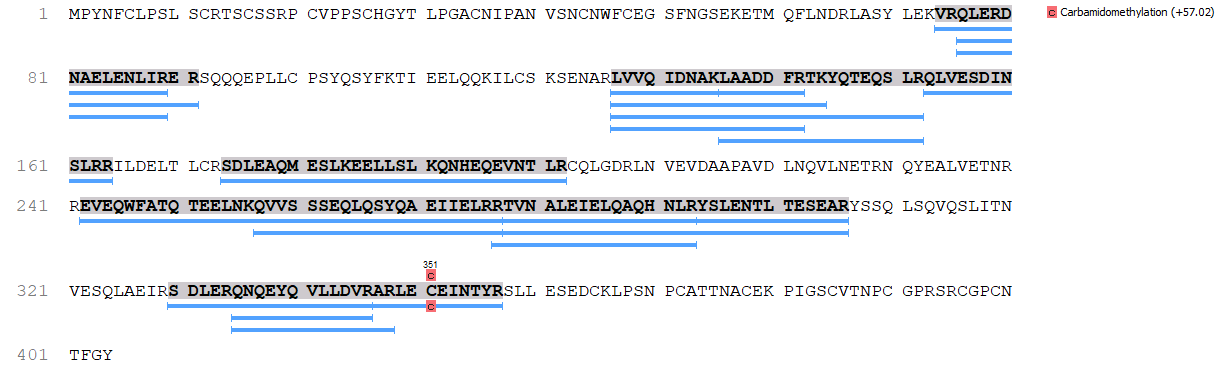

Supplement: S1 Data — (ZIP) [file pntd.0009150.s009.zip › S1 Data/N. naja_Punjab/img/cov_34.png]

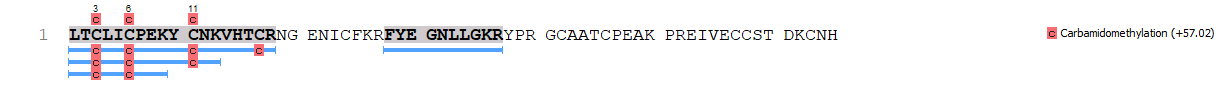

Supplement: S1 Data — (ZIP) [file pntd.0009150.s009.zip › S1 Data/N. naja_Punjab/img/cov_342.png]

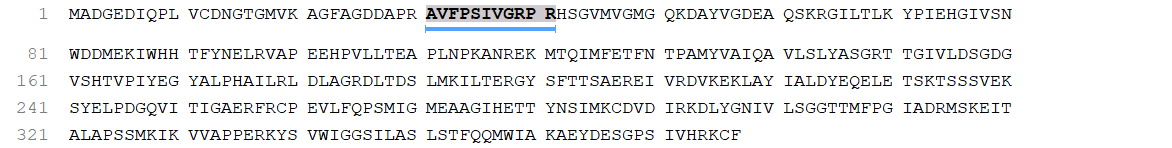

Supplement: S1 Data — (ZIP) [file pntd.0009150.s009.zip › S1 Data/N. naja_Punjab/img/cov_343.png]

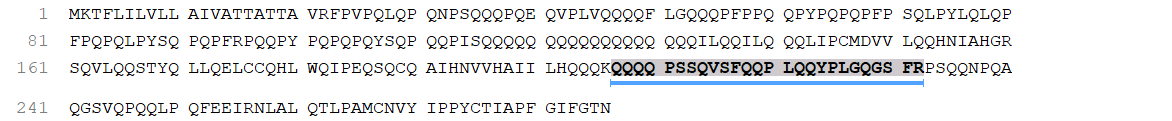

Supplement: S1 Data — (ZIP) [file pntd.0009150.s009.zip › S1 Data/N. naja_Punjab/img/cov_348.png]

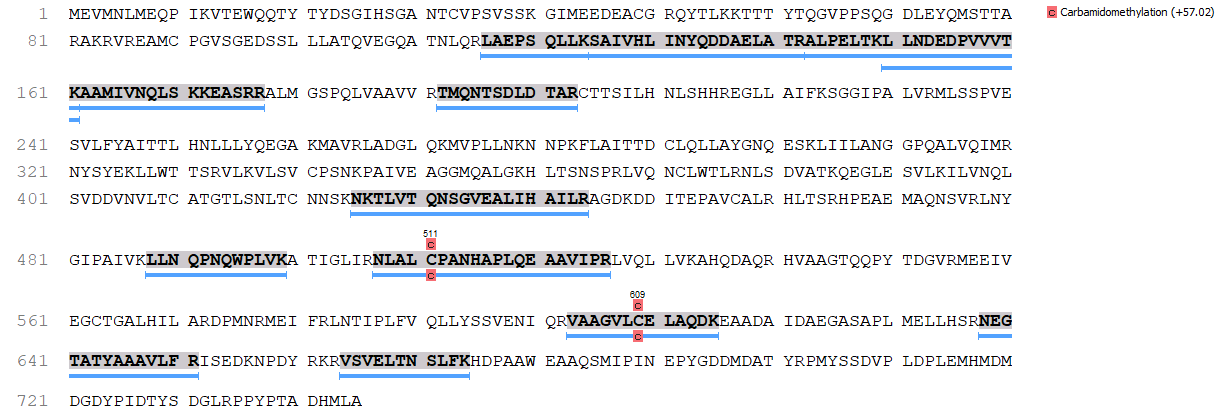

Supplement: S1 Data — (ZIP) [file pntd.0009150.s009.zip › S1 Data/N. naja_Punjab/img/cov_37.png]

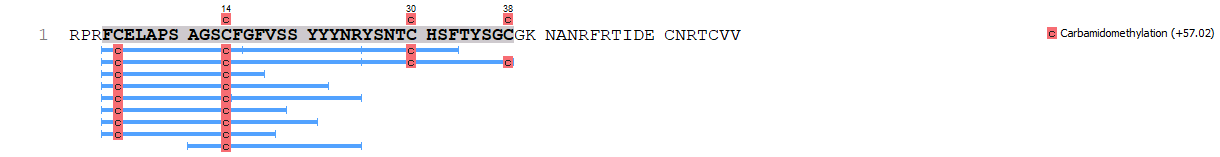

Supplement: S1 Data — (ZIP) [file pntd.0009150.s009.zip › S1 Data/N. naja_Punjab/img/cov_372.png]

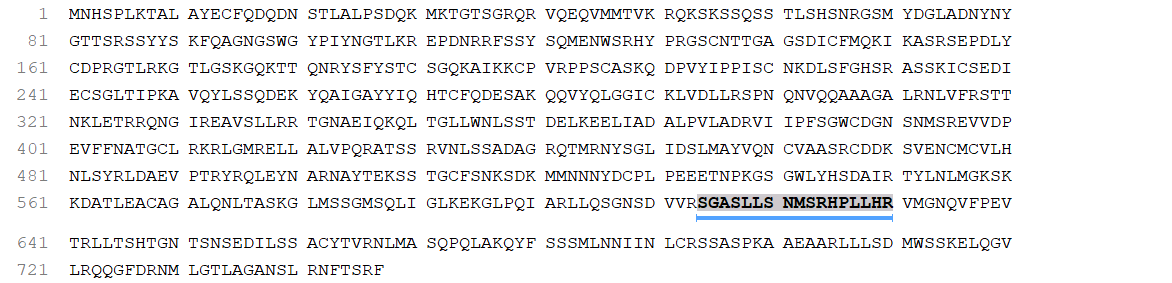

Supplement: S1 Data — (ZIP) [file pntd.0009150.s009.zip › S1 Data/N. naja_Punjab/img/cov_377.png]

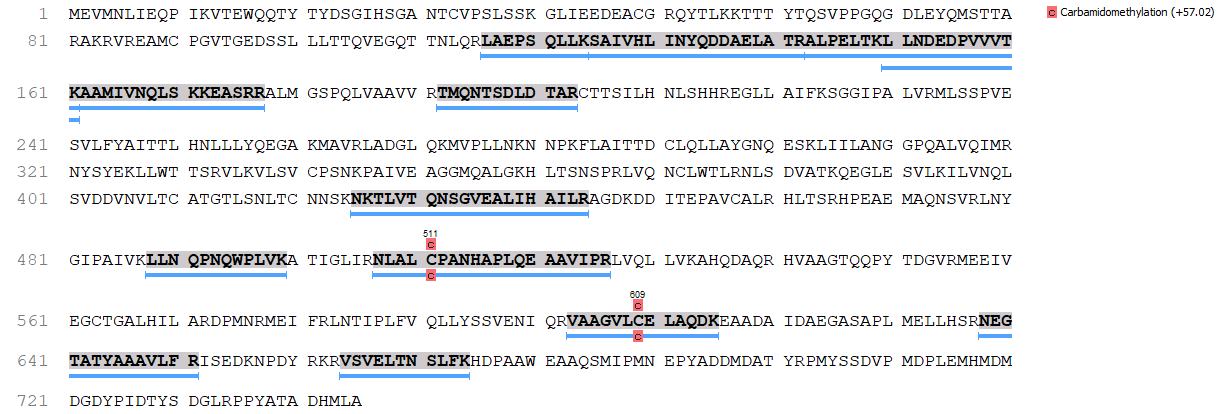

Supplement: S1 Data — (ZIP) [file pntd.0009150.s009.zip › S1 Data/N. naja_Punjab/img/cov_38.png]

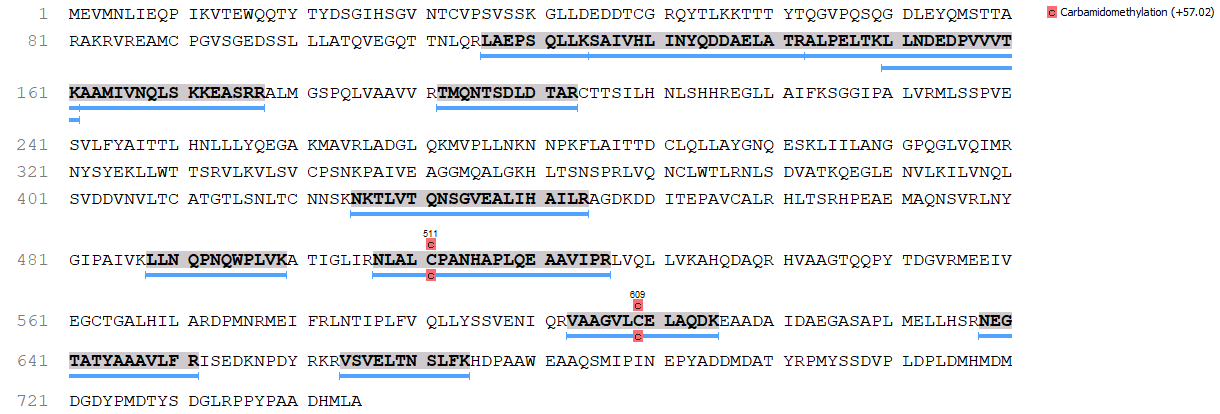

Supplement: S1 Data — (ZIP) [file pntd.0009150.s009.zip › S1 Data/N. naja_Punjab/img/cov_39.png]

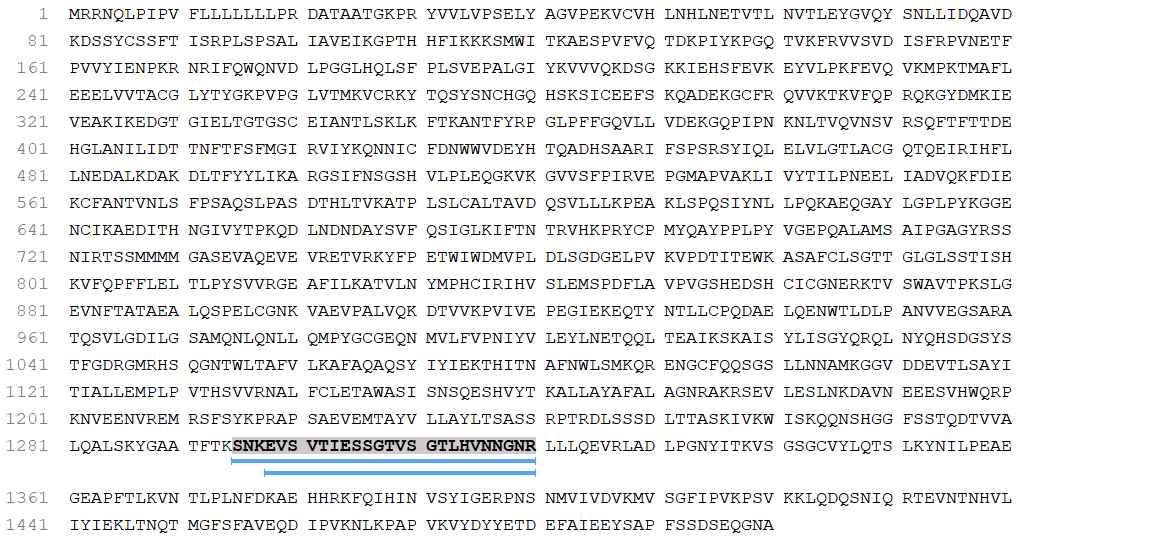

Supplement: S1 Data — (ZIP) [file pntd.0009150.s009.zip › S1 Data/N. naja_Punjab/img/cov_399.png]

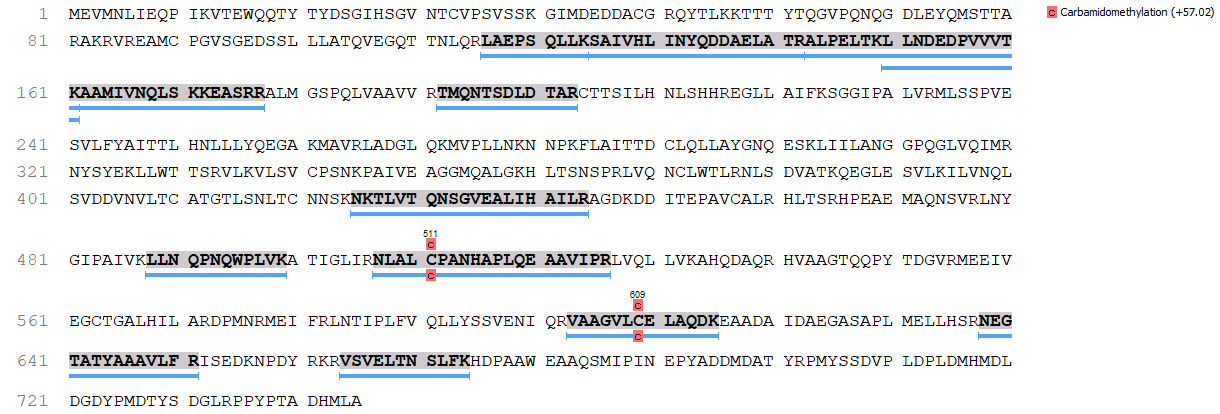

Supplement: S1 Data — (ZIP) [file pntd.0009150.s009.zip › S1 Data/N. naja_Punjab/img/cov_40.png]

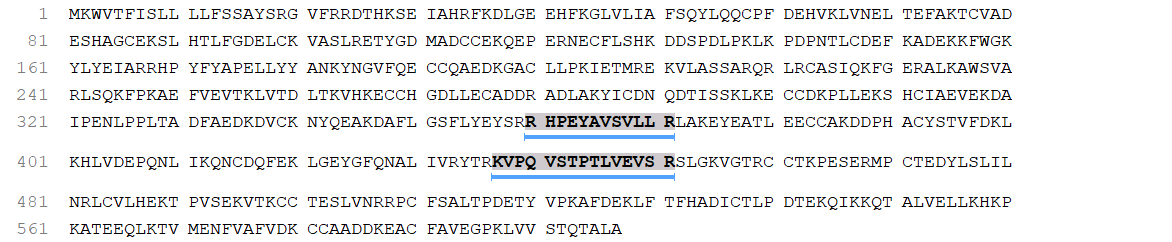

Supplement: S1 Data — (ZIP) [file pntd.0009150.s009.zip › S1 Data/N. naja_Punjab/img/cov_400.png]

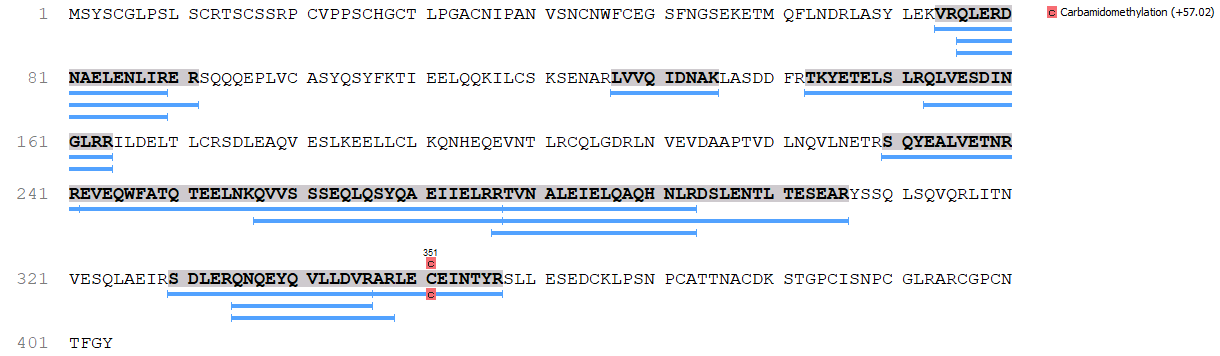

Supplement: S1 Data — (ZIP) [file pntd.0009150.s009.zip › S1 Data/N. naja_Punjab/img/cov_41.png]

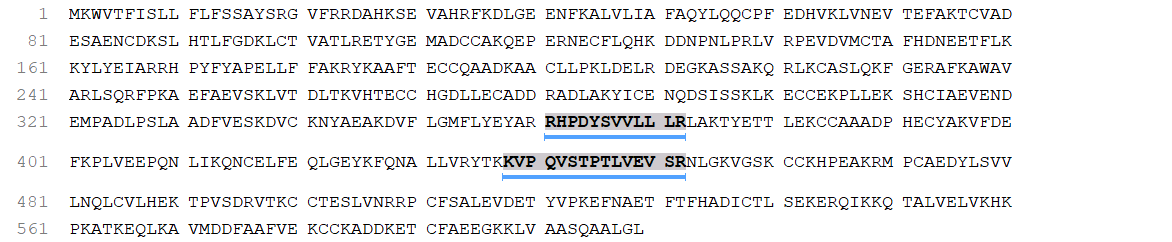

Supplement: S1 Data — (ZIP) [file pntd.0009150.s009.zip › S1 Data/N. naja_Punjab/img/cov_410.png]

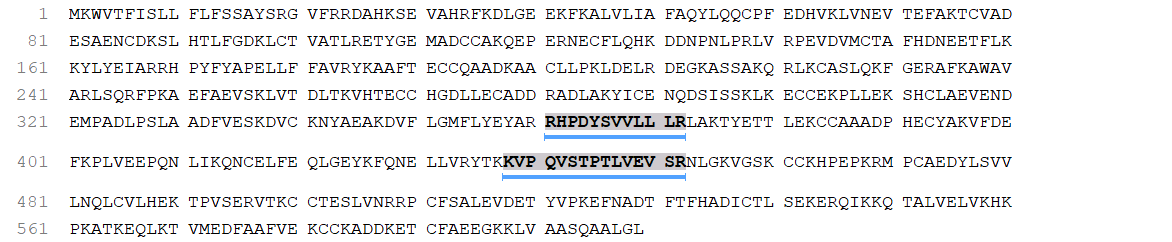

Supplement: S1 Data — (ZIP) [file pntd.0009150.s009.zip › S1 Data/N. naja_Punjab/img/cov_412.png]

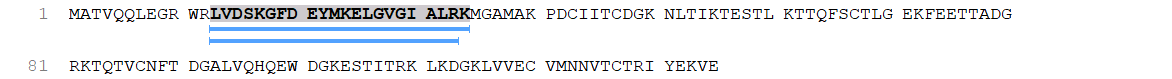

Supplement: S1 Data — (ZIP) [file pntd.0009150.s009.zip › S1 Data/N. naja_Punjab/img/cov_423.png]

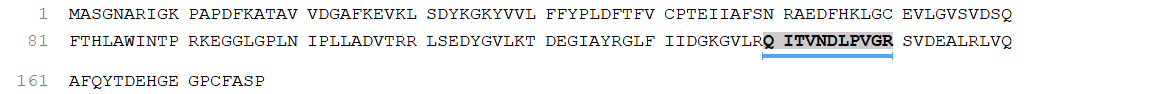

Supplement: S1 Data — (ZIP) [file pntd.0009150.s009.zip › S1 Data/N. naja_Punjab/img/cov_425.png]

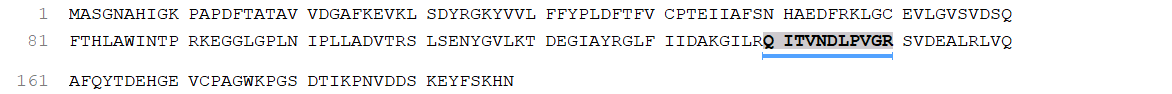

Supplement: S1 Data — (ZIP) [file pntd.0009150.s009.zip › S1 Data/N. naja_Punjab/img/cov_426.png]

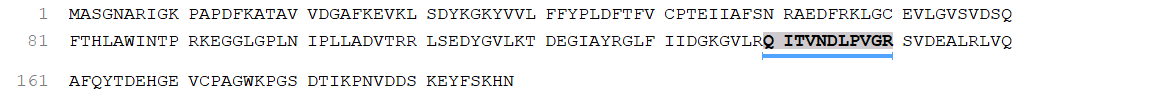

Supplement: S1 Data — (ZIP) [file pntd.0009150.s009.zip › S1 Data/N. naja_Punjab/img/cov_427.png]

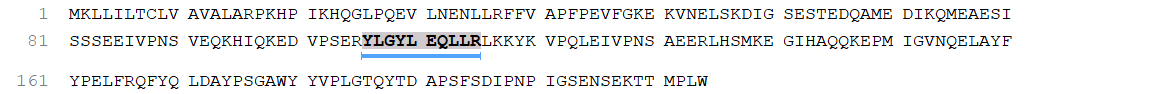

Supplement: S1 Data — (ZIP) [file pntd.0009150.s009.zip › S1 Data/N. naja_Punjab/img/cov_443.png]

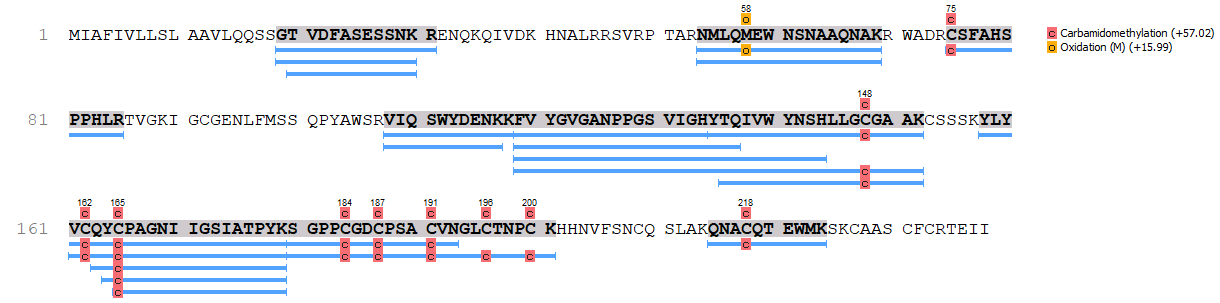

Supplement: S1 Data — (ZIP) [file pntd.0009150.s009.zip › S1 Data/N. naja_Punjab/img/cov_45.png]

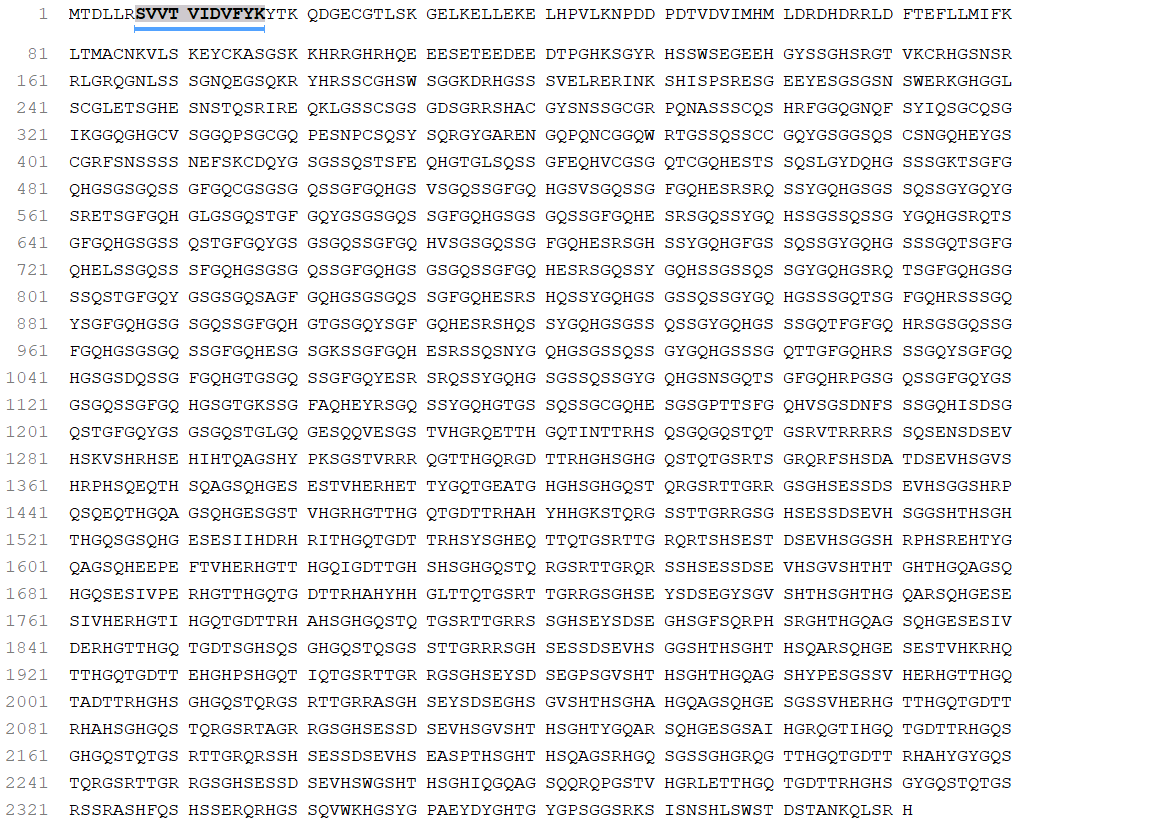

Supplement: S1 Data — (ZIP) [file pntd.0009150.s009.zip › S1 Data/N. naja_Punjab/img/cov_464.png]

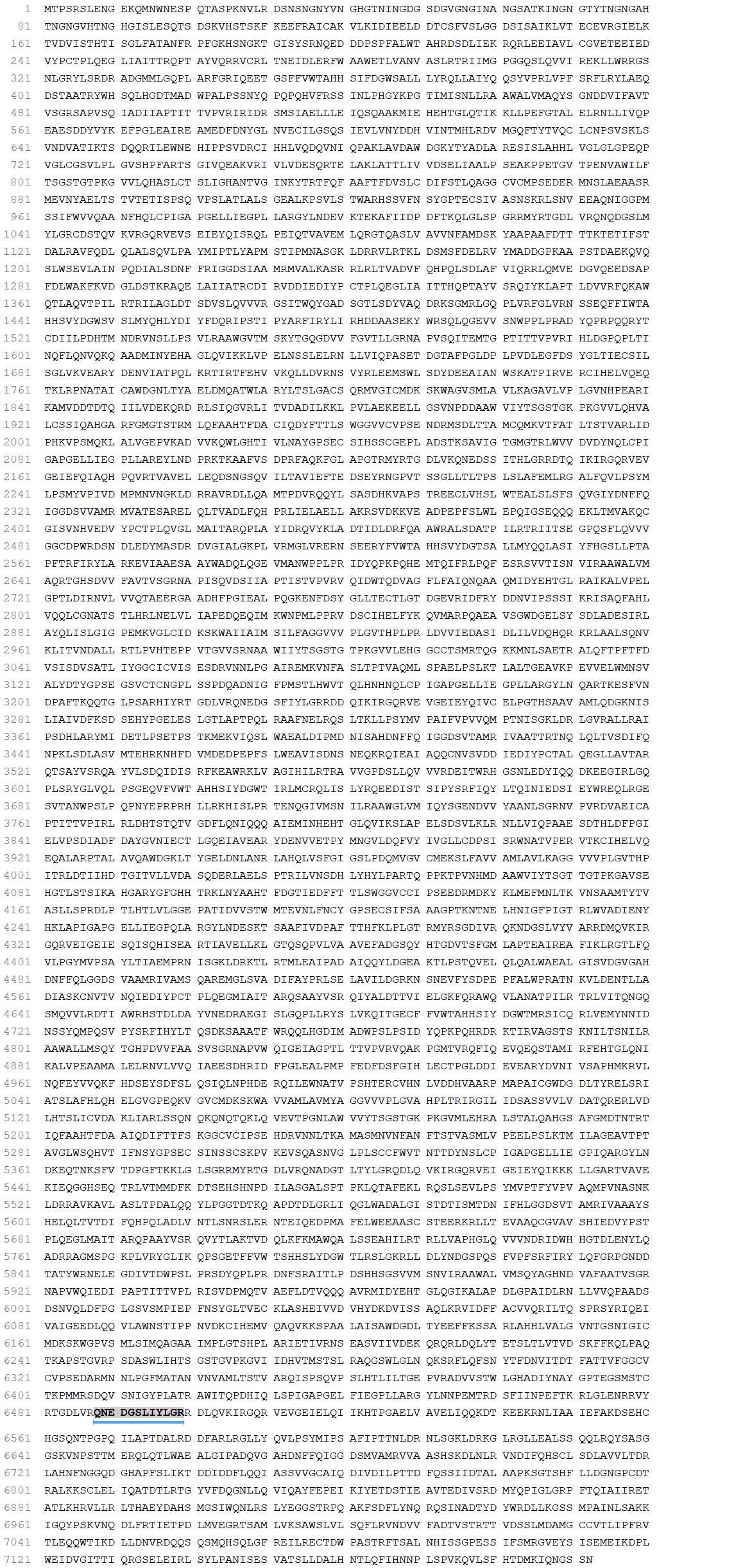

Supplement: S1 Data — (ZIP) [file pntd.0009150.s009.zip › S1 Data/N. naja_Punjab/img/cov_478.png]

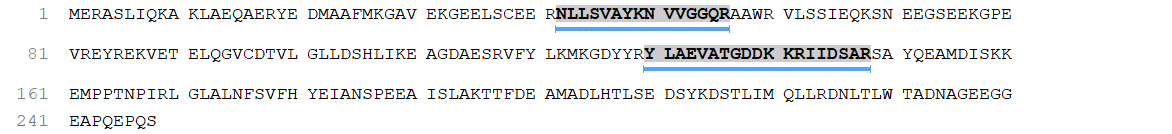

Supplement: S1 Data — (ZIP) [file pntd.0009150.s009.zip › S1 Data/N. naja_Punjab/img/cov_493.png]

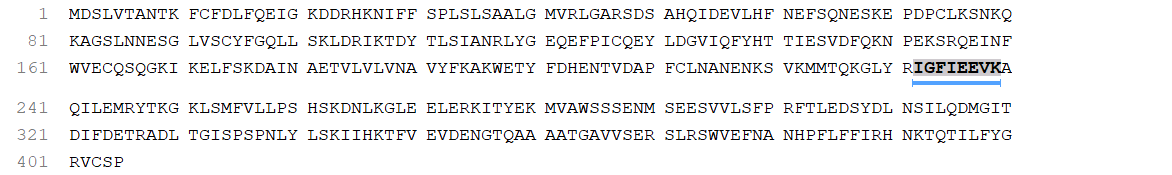

Supplement: S1 Data — (ZIP) [file pntd.0009150.s009.zip › S1 Data/N. naja_Punjab/img/cov_495.png]

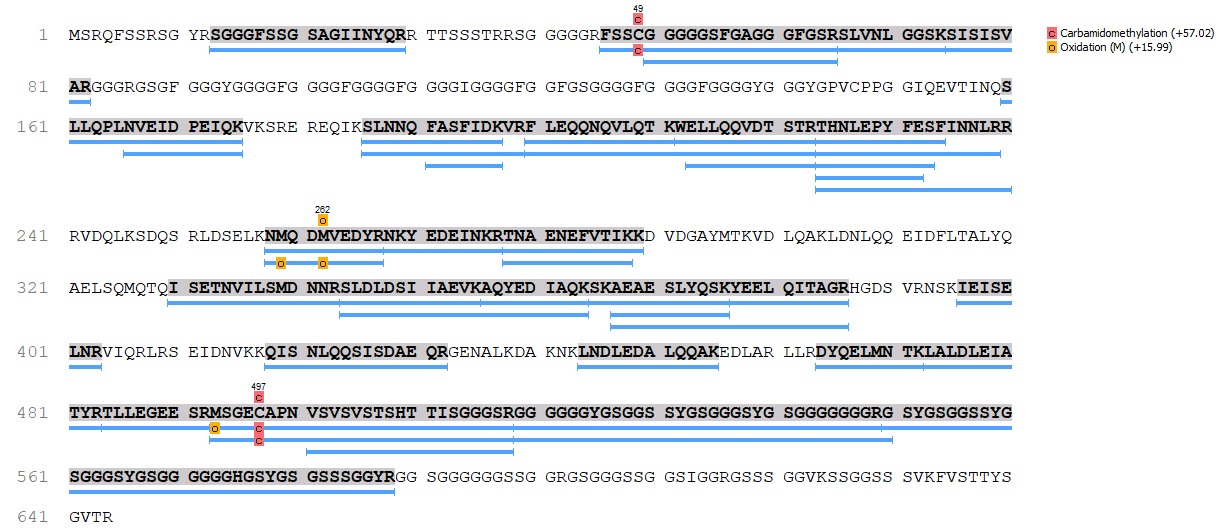

Supplement: S1 Data — (ZIP) [file pntd.0009150.s009.zip › S1 Data/N. naja_Punjab/img/cov_5.png]

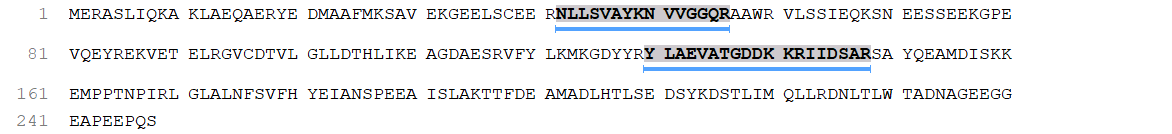

Supplement: S1 Data — (ZIP) [file pntd.0009150.s009.zip › S1 Data/N. naja_Punjab/img/cov_501.png]
