# Supplementary material for: Biogeographical venom variation in the Indian spectacled cobra (Naja naja) underscores the pressing need for pan-India efficacious snakebite therapy
Source: PLoS Negl Trop Dis. 2021 Feb 18;15(2):e0009150. doi: 10.1371/journal.pntd.0009150 (PMC7924803; doi:10.1371/journal.pntd.0009150)
Supplement: S1 Data — (ZIP) [file pntd.0009150.s009.zip › S1 Data/N. naja_Rajasthan/N. naja_Rajasthan.html]

proteins


Summary

  

# 1. Notes

# 2. Result Statistics

**Figure 1.**
False discovery rate (FDR) curve. X axis is the number of peptide-spectrum matches (PSM) being kept. Y axis is the corresponding FDR.

  


  

**Figure 2.**
PSM score distribution.  **(a)**
Distribution of PEAKS peptide score;  **(b)**
Scatterplot of PEAKS peptide score versus precursor mass error.

**(a)**

**(b)**

**Figure 3.**
Distribution of peptide feature detection.  **(a)**
Feature m/z distribution;  **(b)**
Feature RT distribution

**(a)**

**(b)**

**Figure 4.**
Distribution of identified peptide features.  **(a)**
Feature abundance distribution  **(b)**
 *De novo*
sequencing validation.

**(a)**

**(b)**

**Table 1.**
Statistics of data.

|  |  |
| --- | --- |
| # of MS scans | 168352 |
| # of MS/MS scans | 100864 |
| # of Features | 260792 |
| # of Chimera scans | 18415 |


**Table 2.**
Result filtration parameters.

|  |  |
| --- | --- |
| Peptide -10lgP | ≥40 |
| Peptide Ascore | ≥0 |
| Protein -10lgP | ≥20 |
| Proteins unique peptides | ≥1 |
| De novo ALC Score | ≥50% |


**Table 3.**
Statistics of filtered result.

|  |  |
| --- | --- |
| Peptide-Spectrum Matches | 1862 |
| Peptide sequences | 650 |
| Protein groups | 98 |
| Proteins | 224 |
| Proteins (#Unique Peptides) | 59 (>2); 22 (=2); 143 (=1); |
| FDR (Peptide-Spectrum Matches) | 0.1% |
| FDR (Peptide Sequences) | 0.2% |
| FDR (Protein Group) | 0.0% |
| De Novo Only Spectra | 13776 |
| # of identified Features | 11704 |

**Table 4.**
PTM profile.

| Name | ∆Mass | Position | #PSM | -10lgP | Abundance | AScore || Carbamidomethyl | 57.02 | C | 697 | 131.02 | 4.53E6 | 1000.00 |
| Oxidation | 15.99 | M | 90 | 104.27 | 1.78E7 | 39.76 |

# 3. Experiment Control

**Figure 5.**
Precursor mass error of peptide-spectrum matches (PSM) in filtered result.  **(a)**
Distribution of precursor mass error in ppm;  **(b)**
Scatterplot of precursor m/z versus precursor mass error in ppm.

**(a)**

**(b)**

**Table 5.**
Number of identified peptides in each sample by the number of missed cleavages

|  |  |  |  |  |  |
| --- | --- | --- | --- | --- | --- |
| Missed Cleavages | 0 | 1 | 2 | 3 | 4+ |
| F1 | 22 | 0 | 0 | 0 | 0 |
| F2 | 140 | 16 | 1 | 0 | 0 |
| F3 | 57 | 5 | 0 | 0 | 0 |
| F4 | 37 | 10 | 3 | 0 | 0 |
| F5 | 84 | 4 | 0 | 0 | 0 |
| F6 | 58 | 17 | 9 | 2 | 0 |
| F7 | 20 | 5 | 1 | 0 | 0 |
| F8 | 58 | 12 | 6 | 3 | 0 |
| F9 | 60 | 7 | 0 | 0 | 0 |
| F10 | 13 | 0 | 0 | 0 | 0 |

# 4. Other Information

**Table 6.**
Search parameters.

Search Engine Name: PEAKS  
Parent Mass Error Tolerance: 10.0 ppm  
Fragment Mass Error Tolerance: 0.6 Da  
Precursor Mass Search Type: monoisotopic  
Enzyme: Trypsin  
Max Missed Cleavages: 3  
Digest Mode: Semispecific  
Fixed Modifications:   
  Carbamidomethylation: 57.02  
Variable Modifications:   
  Oxidation (M): 15.99  
Max Variable PTM Per Peptide: 3  
Database: SwissProt  
Taxon: All  
Contaminant Database: cRAP\_contaminants  
Searched Entry: 560234  
FDR Estimation: Enabled  
Merge Options: no merge  
Precursor Options: corrected  
Charge Options: no correction  
Filter Charge: 2 - 8  
Process: true  
Associate chimera: yes

**Table 7.**
Instrument parameters.

Fractions: 05112019\_RID\_1313\_NaNaRj\_F1.raw, 09012020\_NaNaRj\_F2.  
raw, 05112019\_RID\_1313\_NaNaRj\_F3.raw, 05112019\_RID\_1313\_NaNaR  
j\_F4.raw, 05112019\_RID\_1313\_NaNaRj\_F5.raw, 05112019\_RID\_1313\_  
NaNaRj\_F6.raw, 05112019\_RID\_1313\_NaNaRj\_F7.raw, 05112019\_RID\_  
1313\_NaNaRj\_F8.raw, 05112019\_RID\_1313\_NaNaRj\_F9.raw, 05112019  
\_RID\_1313\_NaNaRj\_F10.raw  
Ion Source: ESI(nano-spray)  
Fragmentation Mode: high energy CID (y and b ions)  
MS Scan Mode: FT-ICR/Orbitrap  
MS/MS Scan Mode: Linear Ion Trap

  

Protein List

  

Protein Accession Contains:

Protein Description Contains:

Peptide Sample Area >=

Protein Ptm Contains:

| Protein Group | Protein ID | Accession | -10lgP | Coverage (%) | Coverage (%) F1 | Coverage (%) F2 | Coverage (%) F3 | Coverage (%) F4 | Coverage (%) F5 | Coverage (%) F6 | Coverage (%) F7 | Coverage (%) F8 | Coverage (%) F9 | Coverage (%) F10 | Area F1 | Area F2 | Area F3 | Area F4 | Area F5 | Area F6 | Area F7 | Area F8 | Area F9 | Area F10 | #Peptides | #Unique | #Spec F1 | #Spec F2 | #Spec F3 | #Spec F4 | #Spec F5 | #Spec F6 | #Spec F7 | #Spec F8 | #Spec F9 | #Spec F10 | PTM | Avg. Mass | Description |
| --- | --- | --- | --- | --- | --- | --- | --- | --- | --- | --- | --- | --- | --- | --- | --- | --- | --- | --- | --- | --- | --- | --- | --- | --- | --- | --- | --- | --- | --- | --- | --- | --- | --- | --- | --- | --- | --- | --- | --- |
| 2 | 57 | P15445\|PA2A2\_NAJNA | 420.40 | 92 | 0 | 0 | 0 | 0 | 24 | 92 | 73 | 61 | 39 | 24 |  |  |  |  | 5.31E3 | 5.43E7 | 8.01E5 | 6.21E4 |  | 4.98E3 | 36 | 6 | 0 | 0 | 0 | 0 | 2 | 119 | 28 | 32 | 5 | 2 | Y | 13346 | Acidic phospholipase A2 2 OS=Naja naja OX=35670 PE=1 SV=1 |
| 4 | 6 | P35527\|K1C9\_HUMAN | 395.61 | 64 | 14 | 44 | 27 | 31 | 35 | 23 | 22 | 19 | 26 | 29 | 3.15E5 | 1.17E8 | 3.47E6 | 2.18E6 | 4.11E6 | 1.4E6 | 1.19E6 | 1.9E6 | 6.72E5 | 7.14E5 | 47 | 46 | 8 | 50 | 15 | 14 | 20 | 9 | 10 | 14 | 12 | 15 | Y | 62064 | Keratin, type I cytoskeletal 9 OS=Homo sapiens OX=9606 GN=KRT9 PE=1 SV=3 |
| 4 | 7 | #CONTAM#K1C9\_HUMAN\| | 395.61 | 64 | 14 | 44 | 27 | 31 | 35 | 23 | 22 | 19 | 26 | 29 | 3.15E5 | 1.17E8 | 3.47E6 | 2.18E6 | 4.11E6 | 1.4E6 | 1.19E6 | 1.9E6 | 6.72E5 | 7.14E5 | 47 | 46 | 8 | 50 | 15 | 14 | 20 | 9 | 10 | 14 | 12 | 15 | Y | 62129 | sp\|#CONTAM#K1C9\_HUMAN\| |
| 3 | 4 | P13645\|K1C10\_HUMAN | 388.18 | 61 | 23 | 39 | 32 | 23 | 44 | 27 | 25 | 21 | 11 | 22 | 2.1E5 | 1.72E7 | 2.93E6 | 1.34E6 | 1.12E7 | 4.48E6 | 1.09E6 | 1.73E6 | 5.06E4 | 3.43E5 | 42 | 26 | 12 | 39 | 17 | 9 | 29 | 18 | 15 | 9 | 5 | 13 | Y | 58827 | Keratin, type I cytoskeletal 10 OS=Homo sapiens OX=9606 GN=KRT10 PE=1 SV=6 |
| 1 | 1 | P04264\|K2C1\_HUMAN | 385.25 | 54 | 15 | 48 | 37 | 33 | 39 | 17 | 26 | 23 | 20 | 29 | 4.13E5 | 3.18E7 | 1E6 | 1.84E6 | 4.49E6 | 2.98E6 | 1.95E6 | 2.04E6 | 3.23E5 | 3.17E5 | 38 | 17 | 9 | 54 | 24 | 19 | 34 | 11 | 17 | 12 | 12 | 16 | Y | 66039 | Keratin, type II cytoskeletal 1 OS=Homo sapiens OX=9606 GN=KRT1 PE=1 SV=6 |
| 7 | 9 | P35908\|K22E\_HUMAN | 367.12 | 65 | 11 | 40 | 34 | 14 | 45 | 24 | 12 | 10 | 4 | 17 | 6.61E4 | 7.68E6 | 3.85E5 | 7.56E5 | 5.45E6 | 1.06E6 | 5.97E5 | 2.46E5 |  | 2.73E4 | 42 | 27 | 7 | 31 | 19 | 8 | 28 | 9 | 7 | 4 | 3 | 9 | Y | 65433 | Keratin, type II cytoskeletal 2 epidermal OS=Homo sapiens OX=9606 GN=KRT2 PE=1 SV=2 |
| 23 | 94 | P82885\|VESP\_NAJKA | 316.44 | 64 | 24 | 0 | 11 | 13 | 0 | 32 | 13 | 49 | 64 | 16 | 0E0 |  | 3.22E5 | 1.58E5 |  | 2.18E5 | 5.67E4 | 2.73E6 | 3.26E7 | 3.29E4 | 17 | 5 | 2 | 0 | 1 | 1 | 0 | 3 | 1 | 9 | 33 | 1 | Y | 12038 | Thaicobrin OS=Naja kaouthia OX=8649 PE=1 SV=1 |
| 6 | 69 | P01441\|3SA2\_NAJOX | 308.49 | 93 | 13 | 13 | 13 | 13 | 13 | 47 | 57 | 93 | 47 | 13 |  |  |  |  |  | 2.25E7 | 1.11E7 | 5.94E8 | 4.69E5 |  | 21 | 16 | 1 | 1 | 1 | 1 | 1 | 20 | 8 | 73 | 6 | 1 | Y | 6636 | Cytotoxin 2 OS=Naja oxiana OX=8657 PE=1 SV=1 |
| 15 | 197 | P01391\|3L21\_NAJKA | 304.29 | 82 | 0 | 0 | 21 | 82 | 59 | 27 | 45 | 59 | 59 | 59 |  |  | 1.47E4 | 3.44E8 | 9.68E5 | 3.23E6 | 8.02E5 | 4.39E5 | 1.53E5 | 6.89E4 | 15 | 7 | 0 | 0 | 1 | 54 | 5 | 1 | 3 | 3 | 4 | 3 | Y | 7831 | Alpha-cobratoxin OS=Naja kaouthia OX=8649 PE=1 SV=1 |
| 20 | 12 | P02538\|K2C6A\_HUMAN | 302.78 | 43 | 3 | 28 | 25 | 6 | 31 | 7 | 6 | 4 | 1 | 7 |  | 7.54E4 | 2.08E4 |  | 9.06E4 |  |  |  |  | 5.86E3 | 22 | 2 | 2 | 16 | 12 | 3 | 16 | 4 | 3 | 2 | 1 | 4 | Y | 60045 | Keratin, type II cytoskeletal 6A OS=Homo sapiens OX=9606 GN=KRT6A PE=1 SV=3 |
| 16 | 13 | P04259\|K2C6B\_HUMAN | 300.90 | 43 | 5 | 28 | 22 | 8 | 33 | 7 | 8 | 4 | 3 | 5 |  | 3.69E4 | 1.18E4 |  | 5.76E4 |  |  |  |  |  | 22 | 2 | 3 | 21 | 10 | 4 | 17 | 4 | 4 | 2 | 2 | 3 | Y | 60067 | Keratin, type II cytoskeletal 6B OS=Homo sapiens OX=9606 GN=KRT6B PE=1 SV=5 |
| 37 | 95 | Q5YF89\|NGFV2\_NAJSP | 300.13 | 38 | 0 | 0 | 5 | 0 | 7 | 7 | 34 | 38 | 11 | 3 |  |  | 4.39E3 |  | 1.33E5 | 4.91E5 | 6.27E6 | 4.56E7 | 6.48E4 | 3.02E3 | 12 | 12 | 0 | 0 | 1 | 0 | 2 | 2 | 10 | 21 | 2 | 1 | Y | 27030 | Venom nerve growth factor 2 OS=Naja sputatrix OX=33626 PE=2 SV=1 |
| 17 | 10 | P08779\|K1C16\_HUMAN | 289.24 | 51 | 1 | 30 | 35 | 8 | 32 | 4 | 9 | 6 | 0 | 19 |  | 3.14E5 | 1.92E5 | 3.88E4 | 3.33E5 | 8.96E3 | 1.24E5 | 2.3E4 |  | 5.04E4 | 22 | 9 | 1 | 16 | 16 | 3 | 13 | 2 | 4 | 3 | 0 | 8 | Y | 51268 | Keratin, type I cytoskeletal 16 OS=Homo sapiens OX=9606 GN=KRT16 PE=1 SV=4 |
| 14 | 14 | P02533\|K1C14\_HUMAN | 288.91 | 52 | 1 | 40 | 29 | 8 | 36 | 6 | 5 | 6 | 0 | 19 |  | 2E6 | 9.35E4 | 7.75E4 | 1.33E5 | 6.45E4 | 1.85E4 | 2.45E4 |  | 2.99E4 | 22 | 5 | 1 | 20 | 12 | 4 | 14 | 3 | 3 | 2 | 0 | 8 | Y | 51562 | Keratin, type I cytoskeletal 14 OS=Homo sapiens OX=9606 GN=KRT14 PE=1 SV=4 |
| 13 | 15 | P13647\|K2C5\_HUMAN | 269.84 | 42 | 3 | 29 | 21 | 4 | 26 | 6 | 4 | 2 | 1 | 13 | 5.18E3 | 4.57E6 | 1.73E5 |  | 2.96E5 |  |  |  |  | 2.73E4 | 24 | 4 | 2 | 23 | 11 | 2 | 16 | 4 | 2 | 1 | 1 | 7 | Y | 62378 | Keratin, type II cytoskeletal 5 OS=Homo sapiens OX=9606 GN=KRT5 PE=1 SV=3 |
| 24 | 154 | P25498\|PA2AE\_NAJOX | 267.68 | 52 | 0 | 0 | 0 | 0 | 12 | 40 | 30 | 50 | 22 | 12 |  |  |  |  |  |  |  | 4.39E4 |  |  | 11 | 1 | 0 | 0 | 0 | 0 | 1 | 23 | 8 | 7 | 2 | 1 | Y | 13229 | Acidic phospholipase A2 E OS=Naja oxiana OX=8657 PE=1 SV=1 |
| 40 | 200 | P25668\|3L21\_NAJNA | 242.86 | 66 | 0 | 0 | 0 | 66 | 32 | 18 | 18 | 32 | 14 | 32 |  |  |  | 3.1E8 |  |  |  |  |  |  | 9 | 2 | 0 | 0 | 0 | 25 | 2 | 1 | 1 | 2 | 1 | 2 | Y | 7847 | Long neurotoxin 1 OS=Naja naja OX=35670 PE=1 SV=1 |
| 64 | 198 | P25669\|3L22\_NAJNA | 240.39 | 66 | 0 | 0 | 0 | 66 | 32 | 18 | 18 | 32 | 14 | 32 |  |  |  | 2.54E6 |  |  |  |  |  |  | 8 | 1 | 0 | 0 | 0 | 18 | 2 | 1 | 1 | 2 | 1 | 2 | Y | 7821 | Long neurotoxin 2 OS=Naja naja OX=35670 PE=1 SV=1 |
| 61 | 29 | P84808\|CRVP2\_NAJKA | 239.84 | 60 | 0 | 0 | 0 | 0 | 0 | 0 | 0 | 0 | 60 | 0 |  |  |  |  |  |  |  |  | 3.51E6 |  | 17 | 9 | 0 | 0 | 0 | 0 | 0 | 0 | 0 | 0 | 23 | 0 | Y | 26216 | Cysteine-rich venom protein kaouthin-2 OS=Naja kaouthia OX=8649 PE=1 SV=2 |
| 29 | 203 | P25672\|3L24\_NAJNA | 233.35 | 63 | 0 | 0 | 0 | 63 | 44 | 0 | 18 | 32 | 32 | 32 |  |  |  | 6.81E4 | 5.73E4 |  |  |  |  |  | 9 | 1 | 0 | 0 | 0 | 34 | 3 | 0 | 1 | 2 | 2 | 2 | Y | 7889 | Long neurotoxin 4 OS=Naja naja OX=35670 PE=1 SV=1 |
| 49 | 19 | P02768\|ALBU\_HUMAN | 231.82 | 28 | 2 | 26 | 9 | 0 | 0 | 0 | 0 | 0 | 0 | 0 |  | 8.14E6 | 1.01E5 |  |  |  |  |  |  |  | 17 | 12 | 1 | 18 | 5 | 0 | 0 | 0 | 0 | 0 | 0 | 0 | Y | 69367 | Serum albumin OS=Homo sapiens OX=9606 GN=ALB PE=1 SV=2 |
| 49 | 20 | #CONTAM#ALBU\_HUMAN\| | 231.82 | 28 | 2 | 26 | 9 | 0 | 0 | 0 | 0 | 0 | 0 | 0 |  | 8.14E6 | 1.01E5 |  |  |  |  |  |  |  | 17 | 12 | 1 | 18 | 5 | 0 | 0 | 0 | 0 | 0 | 0 | 0 | Y | 69367 | sp\|#CONTAM#ALBU\_HUMAN\| |
| 22 | 235 | P86538\|3SA2A\_NAJNA | 231.77 | 60 | 13 | 13 | 13 | 13 | 13 | 60 | 27 | 40 | 13 | 13 |  |  |  |  |  | 3.3E5 |  |  |  |  | 8 | 1 | 1 | 1 | 1 | 1 | 1 | 32 | 2 | 4 | 1 | 1 | Y | 6711 | Cytotoxin 2a OS=Naja naja OX=35670 PE=1 SV=2 |
| 85 | 52 | P82942\|VM3K\_NAJKA | 227.09 | 30 | 0 | 0 | 0 | 0 | 0 | 2 | 3 | 13 | 25 | 0 |  |  |  |  |  | 5.84E4 |  | 1.18E6 | 2.48E5 |  | 8 | 5 | 0 | 0 | 0 | 0 | 0 | 1 | 1 | 4 | 7 | 0 | Y | 44493 | Hemorrhagic metalloproteinase-disintegrin-like kaouthiagin OS=Naja kaouthia OX=8649 PE=1 SV=1 |
| 27 | 21 | Q04695\|K1C17\_HUMAN | 225.94 | 30 | 2 | 17 | 18 | 2 | 16 | 2 | 6 | 2 | 0 | 7 |  |  |  |  | 6.06E4 |  |  |  |  |  | 14 | 1 | 1 | 10 | 7 | 1 | 7 | 1 | 3 | 1 | 0 | 4 | Y | 48106 | Keratin, type I cytoskeletal 17 OS=Homo sapiens OX=9606 GN=KRT17 PE=1 SV=2 |
| 36 | 260 | P01427\|3S11\_NAJOX | 225.01 | 54 | 43 | 30 | 18 | 18 | 18 | 18 | 0 | 0 | 18 | 0 | 2.33E7 | 1.13E8 | 2.44E7 | 4.48E5 | 8.02E3 | 1.59E4 |  |  | 4.27E3 |  | 7 | 7 | 9 | 16 | 9 | 2 | 1 | 1 | 0 | 0 | 1 | 0 | Y | 6885 | Short neurotoxin 1 OS=Naja oxiana OX=8657 PE=1 SV=1 |
| 18 | 39 | P06394\|K1C10\_BOVIN | 224.19 | 21 | 11 | 15 | 10 | 5 | 18 | 3 | 7 | 2 | 2 | 10 |  |  |  |  | 1.32E4 |  |  |  |  |  | 14 | 1 | 6 | 20 | 6 | 3 | 11 | 2 | 4 | 1 | 1 | 6 | N | 54848 | Keratin, type I cytoskeletal 10 OS=Bos taurus OX=9913 GN=KRT10 PE=3 SV=1 |
| 67 | 41 | D5LMJ3\|VM3A\_NAJAT | 218.17 | 20 | 0 | 0 | 0 | 0 | 0 | 0 | 0 | 16 | 14 | 3 |  |  |  |  |  |  |  | 1.35E6 | 6.8E5 | 1.06E4 | 9 | 8 | 0 | 0 | 0 | 0 | 0 | 0 | 0 | 10 | 8 | 2 | Y | 68254 | Zinc metalloproteinase-disintegrin-like atrase-A OS=Naja atra OX=8656 PE=2 SV=1 |
| 19 | 42 | P00761\|TRYP\_PIG | 215.39 | 42 | 29 | 27 | 20 | 12 | 8 | 8 | 0 | 6 | 26 | 4 | 3.71E6 | 8.42E6 | 4.25E5 | 2.97E6 |  | 2.05E6 |  | 1.16E5 | 7.9E6 | 7.24E4 | 16 | 13 | 6 | 7 | 4 | 3 | 2 | 2 | 0 | 1 | 9 | 1 | Y | 24409 | Trypsin OS=Sus scrofa OX=9823 PE=1 SV=1 |
| 19 | 43 | #CONTAM#TRYP\_PIG\| | 215.39 | 42 | 29 | 27 | 20 | 12 | 8 | 8 | 0 | 6 | 26 | 4 | 3.71E6 | 8.42E6 | 4.25E5 | 2.97E6 |  | 2.05E6 |  | 1.16E5 | 7.9E6 | 7.24E4 | 16 | 13 | 6 | 7 | 4 | 3 | 2 | 2 | 0 | 1 | 9 | 1 | Y | 24409 | sp\|#CONTAM#TRYP\_PIG\| |
| 65 | 202 | P20229\|VKTTI\_NAJNA | 210.90 | 79 | 0 | 60 | 0 | 58 | 19 | 40 | 21 | 0 | 0 | 0 |  | 6.21E7 |  | 5.57E6 |  | 6.39E4 | 6.12E4 |  |  |  | 6 | 5 | 0 | 14 | 0 | 4 | 1 | 2 | 1 | 0 | 0 | 0 | Y | 6371 | Kunitz-type serine protease inhibitor OS=Naja naja OX=35670 PE=1 SV=1 |
| 101 | 214 | P82464\|3SO8\_NAJKA | 205.01 | 72 | 0 | 0 | 0 | 0 | 0 | 72 | 15 | 0 | 0 | 0 |  |  |  |  |  | 2.19E7 | 3.63E4 |  |  |  | 5 | 5 | 0 | 0 | 0 | 0 | 0 | 6 | 1 | 0 | 0 | 0 | Y | 7624 | Muscarinic toxin-like protein 3 OS=Naja kaouthia OX=8649 PE=1 SV=1 |
| 46 | 44 | Q7T1K6\|CRVP1\_NAJAT | 200.50 | 36 | 0 | 0 | 0 | 0 | 4 | 0 | 0 | 0 | 36 | 31 |  |  |  |  | 1.34E5 |  |  |  | 9.73E5 | 3.05E6 | 10 | 9 | 0 | 0 | 0 | 0 | 1 | 0 | 0 | 0 | 13 | 10 | Y | 26882 | Cysteine-rich venom protein natrin-1 OS=Naja atra OX=8656 PE=1 SV=1 |
| 46 | 45 | P84805\|CRVP1\_NAJKA | 200.50 | 36 | 0 | 0 | 0 | 0 | 4 | 0 | 0 | 0 | 36 | 31 |  |  |  |  | 1.34E5 |  |  |  | 9.73E5 | 3.05E6 | 10 | 9 | 0 | 0 | 0 | 0 | 1 | 0 | 0 | 0 | 13 | 10 | Y | 26846 | Cysteine-rich venom protein kaouthin-1 OS=Naja kaouthia OX=8649 PE=1 SV=2 |
| 92 | 626 | P10117\|PA2H1\_LATCO | 195.82 | 17 | 0 | 0 | 0 | 0 | 0 | 17 | 15 | 17 | 13 | 0 |  |  |  |  |  | 2.92E5 | 2.86E4 | 5.92E5 | 2.69E3 |  | 6 | 5 | 0 | 0 | 0 | 0 | 0 | 4 | 2 | 4 | 2 | 0 | Y | 13024 | Basic phospholipase A2 homolog 1 OS=Laticauda colubrina OX=8628 PE=1 SV=1 |
| 57 | 75 | P01946\|HBA\_RAT | 192.86 | 56 | 56 | 0 | 32 | 0 | 0 | 0 | 0 | 0 | 46 | 11 | 1.74E6 |  | 6.12E4 |  |  |  |  |  | 1.44E5 | 8.58E4 | 6 | 6 | 12 | 0 | 4 | 0 | 0 | 0 | 0 | 0 | 4 | 2 | N | 15329 | Hemoglobin subunit alpha-1/2 OS=Rattus norvegicus OX=10116 GN=Hba1 PE=1 SV=3 |
| 81 | 317 | P62377\|3SOFL\_NAJNA | 185.15 | 35 | 0 | 0 | 0 | 0 | 0 | 18 | 35 | 18 | 0 | 0 |  |  |  |  |  | 2.9E6 | 3.7E7 | 3.47E6 |  |  | 5 | 5 | 0 | 0 | 0 | 0 | 0 | 2 | 6 | 2 | 0 | 0 | Y | 7014 | Cytotoxin-like basic protein OS=Naja naja OX=35670 PE=1 SV=1 |
| 81 | 318 | Q9W716\|3SOFV\_NAJAT | 185.15 | 27 | 0 | 0 | 0 | 0 | 0 | 13 | 27 | 13 | 0 | 0 |  |  |  |  |  | 2.9E6 | 3.7E7 | 3.47E6 |  |  | 5 | 5 | 0 | 0 | 0 | 0 | 0 | 2 | 6 | 2 | 0 | 0 | Y | 9333 | Cytotoxin homolog 5V OS=Naja atra OX=8656 PE=3 SV=1 |
| 81 | 319 | P62375\|3SOF5\_NAJAT | 185.15 | 27 | 0 | 0 | 0 | 0 | 0 | 13 | 27 | 13 | 0 | 0 |  |  |  |  |  | 2.9E6 | 3.7E7 | 3.47E6 |  |  | 5 | 5 | 0 | 0 | 0 | 0 | 0 | 2 | 6 | 2 | 0 | 0 | Y | 9323 | Cytotoxin A5 OS=Naja atra OX=8656 PE=1 SV=1 |
| 81 | 320 | Q91126\|3SOFA\_NAJAT | 185.15 | 27 | 0 | 0 | 0 | 0 | 0 | 13 | 27 | 13 | 0 | 0 |  |  |  |  |  | 2.9E6 | 3.7E7 | 3.47E6 |  |  | 5 | 5 | 0 | 0 | 0 | 0 | 0 | 2 | 6 | 2 | 0 | 0 | Y | 9319 | Cardiotoxin 7a OS=Naja atra OX=8656 PE=3 SV=1 |
| 81 | 321 | Q91137\|3SOFL\_NAJAT | 185.15 | 27 | 0 | 0 | 0 | 0 | 0 | 13 | 27 | 13 | 0 | 0 |  |  |  |  |  | 2.9E6 | 3.7E7 | 3.47E6 |  |  | 5 | 5 | 0 | 0 | 0 | 0 | 0 | 2 | 6 | 2 | 0 | 0 | Y | 9309 | Cytotoxin homolog 5 OS=Naja atra OX=8656 PE=3 SV=1 |
| 81 | 322 | Q91996\|3SOFP\_NAJAT | 185.15 | 27 | 0 | 0 | 0 | 0 | 0 | 13 | 27 | 13 | 0 | 0 |  |  |  |  |  | 2.9E6 | 3.7E7 | 3.47E6 |  |  | 5 | 5 | 0 | 0 | 0 | 0 | 0 | 2 | 6 | 2 | 0 | 0 | Y | 9305 | Cardiotoxin 7'' OS=Naja atra OX=8656 PE=3 SV=1 |
| 112 | 58 | O43790\|KRT86\_HUMAN | 184.28 | 17 | 0 | 0 | 17 | 0 | 0 | 0 | 0 | 0 | 0 | 0 |  |  | 8.8E4 |  |  |  |  |  |  |  | 5 | 4 | 0 | 0 | 6 | 0 | 0 | 0 | 0 | 0 | 0 | 0 | Y | 53501 | Keratin, type II cuticular Hb6 OS=Homo sapiens OX=9606 GN=KRT86 PE=1 SV=1 |
| 112 | 59 | #CONTAM#KRHB6\_HUMAN\| | 184.28 | 17 | 0 | 0 | 17 | 0 | 0 | 0 | 0 | 0 | 0 | 0 |  |  | 8.8E4 |  |  |  |  |  |  |  | 5 | 4 | 0 | 0 | 6 | 0 | 0 | 0 | 0 | 0 | 0 | 0 | Y | 53501 | sp\|#CONTAM#KRHB6\_HUMAN\| |
| 51 | 66 | P19012\|K1C15\_HUMAN | 173.59 | 15 | 2 | 9 | 10 | 2 | 14 | 2 | 4 | 2 | 0 | 10 |  |  |  |  | 3.69E4 |  |  |  |  |  | 9 | 2 | 1 | 7 | 5 | 1 | 7 | 1 | 2 | 1 | 0 | 5 | N | 49212 | Keratin, type I cytoskeletal 15 OS=Homo sapiens OX=9606 GN=KRT15 PE=1 SV=3 |
| 83 | 53 | P15924\|DESP\_HUMAN | 171.53 | 4 | 0 | 2 | 1 | 0 | 0 | 0 | 0 | 0 | 0 | 0 |  | 6.27E5 | 4.19E4 |  |  |  |  |  |  |  | 10 | 10 | 0 | 7 | 3 | 0 | 0 | 0 | 0 | 0 | 0 | 0 | N | 331774 | Desmoplakin OS=Homo sapiens OX=9606 GN=DSP PE=1 SV=3 |
| 75 | 67 | D6PXE8\|VM3B\_NAJAT | 170.43 | 13 | 0 | 0 | 0 | 0 | 0 | 4 | 4 | 9 | 10 | 0 |  |  |  |  |  | 5.16E5 | 1.88E5 | 6.09E6 | 1.72E5 |  | 7 | 5 | 0 | 0 | 0 | 0 | 0 | 2 | 2 | 9 | 4 | 0 | Y | 66246 | Zinc metalloproteinase-disintegrin-like atrase-B OS=Naja atra OX=8656 PE=1 SV=1 |
| 75 | 68 | D3TTC1\|VM3KL\_NAJAT | 170.43 | 13 | 0 | 0 | 0 | 0 | 0 | 4 | 4 | 9 | 10 | 0 |  |  |  |  |  | 5.16E5 | 1.88E5 | 6.09E6 | 1.72E5 |  | 7 | 5 | 0 | 0 | 0 | 0 | 0 | 2 | 2 | 9 | 4 | 0 | Y | 66292 | Zinc metalloproteinase-disintegrin-like kaouthiagin-like OS=Naja atra OX=8656 PE=1 SV=1 |
| 84 | 241 | P81605\|DCD\_HUMAN | 170.22 | 44 | 41 | 34 | 31 | 21 | 20 | 0 | 0 | 0 | 20 | 20 | 2.33E5 | 2.11E6 | 1.01E5 | 5.56E4 | 3.39E5 |  |  |  | 9.43E4 | 4.32E4 | 4 | 4 | 3 | 3 | 2 | 1 | 2 | 0 | 0 | 0 | 2 | 2 | Y | 11284 | Dermcidin OS=Homo sapiens OX=9606 GN=DCD PE=1 SV=2 |
| 26 | 262 | P01400\|3NO2B\_NAJME | 169.34 | 60 | 0 | 0 | 0 | 60 | 14 | 28 | 28 | 14 | 0 | 0 |  |  |  | 4.92E6 |  |  |  |  |  |  | 7 | 2 | 0 | 0 | 0 | 40 | 1 | 2 | 2 | 1 | 0 | 0 | Y | 7430 | Weak toxin S4C11 OS=Naja melanoleuca OX=8643 PE=1 SV=1 |
| 50 | 819 | P29182\|3NO28\_NAJNA | 167.07 | 18 | 0 | 0 | 0 | 18 | 18 | 18 | 18 | 0 | 0 | 0 |  |  |  | 3.93E8 | 1.69E4 | 4.12E5 | 4.26E5 |  |  |  | 5 | 5 | 0 | 0 | 0 | 28 | 1 | 1 | 1 | 0 | 0 | 0 | Y | 7581 | Weak neurotoxin 8 OS=Naja naja OX=35670 PE=1 SV=1 |
| 50 | 820 | P29181\|3NO27\_NAJNA | 167.07 | 18 | 0 | 0 | 0 | 18 | 18 | 18 | 18 | 0 | 0 | 0 |  |  |  | 3.93E8 | 1.69E4 | 4.12E5 | 4.26E5 |  |  |  | 5 | 5 | 0 | 0 | 0 | 28 | 1 | 1 | 1 | 0 | 0 | 0 | Y | 7637 | Weak neurotoxin 7 OS=Naja naja OX=35670 PE=1 SV=1 |
| 32 | 118 | P07525\|3SAT\_NAJAT | 165.49 | 82 | 13 | 13 | 13 | 13 | 13 | 25 | 35 | 70 | 25 | 13 |  |  |  |  |  |  |  | 9.99E5 |  |  | 8 | 1 | 1 | 1 | 1 | 1 | 1 | 15 | 2 | 8 | 2 | 1 | Y | 6810 | Cytotoxin 5 OS=Naja atra OX=8656 PE=1 SV=1 |
| 35 | 113 | Q9PST3\|3SA2B\_NAJSP | 160.35 | 54 | 10 | 10 | 10 | 10 | 10 | 10 | 17 | 36 | 28 | 10 |  |  |  |  |  |  |  |  | 3.9E4 |  | 7 | 1 | 1 | 1 | 1 | 1 | 1 | 14 | 1 | 7 | 4 | 1 | Y | 9042 | Cytotoxin 2b OS=Naja sputatrix OX=33626 PE=3 SV=1 |
| 35 | 114 | A0A0U5AUY6\|3SA9\_NAJNA | 160.35 | 61 | 11 | 11 | 11 | 11 | 11 | 11 | 19 | 40 | 32 | 11 |  |  |  |  |  |  |  |  | 3.9E4 |  | 7 | 1 | 1 | 1 | 1 | 1 | 1 | 14 | 1 | 7 | 4 | 1 | Y | 8041 | Cytotoxin 9 (Fragment) OS=Naja naja OX=35670 PE=1 SV=1 |
| 35 | 115 | Q9PST4\|3SA2A\_NAJSP | 160.35 | 54 | 10 | 10 | 10 | 10 | 10 | 10 | 17 | 36 | 28 | 10 |  |  |  |  |  |  |  |  | 3.9E4 |  | 7 | 1 | 1 | 1 | 1 | 1 | 1 | 14 | 1 | 7 | 4 | 1 | Y | 9054 | Cytotoxin 2a OS=Naja sputatrix OX=33626 PE=2 SV=1 |
| 87 | 55 | P02091\|HBB1\_RAT | 158.22 | 56 | 35 | 0 | 7 | 0 | 7 | 0 | 0 | 0 | 22 | 21 | 1.48E5 |  | 2.1E4 |  | 1.21E4 |  |  |  | 4.98E4 | 2.33E4 | 8 | 8 | 5 | 0 | 1 | 0 | 1 | 0 | 0 | 0 | 3 | 3 | Y | 15979 | Hemoglobin subunit beta-1 OS=Rattus norvegicus OX=10116 GN=Hbb PE=1 SV=3 |
| 102 | 107 | A8QL58\|OXLA\_NAJAT | 157.40 | 13 | 0 | 0 | 0 | 2 | 0 | 3 | 7 | 4 | 3 | 0 |  |  |  |  |  | 8.95E4 | 8.58E4 | 5.97E4 | 2.03E4 |  | 5 | 4 | 0 | 0 | 0 | 1 | 0 | 1 | 2 | 1 | 1 | 0 | Y | 51439 | L-amino-acid oxidase (Fragment) OS=Naja atra OX=8656 PE=1 SV=1 |
| 95 | 99 | Q9PVK7\|VM3\_NAJKA | 154.13 | 10 | 0 | 0 | 0 | 0 | 0 | 10 | 6 | 2 | 0 | 0 |  |  |  |  |  | 9.81E5 | 2.34E5 | 4.18E4 |  |  | 5 | 5 | 0 | 0 | 0 | 0 | 0 | 6 | 2 | 1 | 0 | 0 | Y | 67662 | Zinc metalloproteinase-disintegrin-like cobrin OS=Naja kaouthia OX=8649 PE=2 SV=1 |
| 94 | 56 | Q91132\|VCO3\_NAJKA | 153.32 | 5 | 0 | 0 | 0 | 0 | 1 | 2 | 0 | 2 | 2 | 0 |  |  |  |  | 1.25E4 | 2.69E5 |  | 1.17E5 | 3.09E4 |  | 5 | 5 | 0 | 0 | 0 | 0 | 1 | 1 | 0 | 2 | 2 | 0 | Y | 184517 | Cobra venom factor OS=Naja kaouthia OX=8649 PE=1 SV=1 |
| 96 | 288 | P82463\|3SUC2\_NAJKA | 153.30 | 63 | 0 | 0 | 0 | 0 | 63 | 29 | 29 | 29 | 0 | 0 |  |  |  |  | 1.33E6 | 1.51E6 | 6.99E4 | 2.56E4 |  |  | 3 | 3 | 0 | 0 | 0 | 0 | 4 | 3 | 1 | 1 | 0 | 0 | Y | 7298 | Muscarinic toxin-like protein 2 OS=Naja kaouthia OX=8649 PE=1 SV=1 |
| 79 | 137 | Q7Z794\|K2C1B\_HUMAN | 144.16 | 7 | 3 | 7 | 2 | 2 | 3 | 0 | 2 | 2 | 3 | 2 |  | 4.06E4 |  |  |  |  |  |  |  |  | 4 | 2 | 2 | 5 | 1 | 1 | 3 | 0 | 1 | 1 | 2 | 1 | N | 61901 | Keratin, type II cytoskeletal 1b OS=Homo sapiens OX=9606 GN=KRT77 PE=2 SV=3 |
| 39 | 295 | P85520\|3NO2\_NAJOX | 143.46 | 66 | 0 | 0 | 0 | 66 | 14 | 14 | 14 | 0 | 0 | 0 |  |  |  | 3.49E5 |  |  |  |  |  |  | 5 | 1 | 0 | 0 | 0 | 32 | 1 | 1 | 1 | 0 | 0 | 0 | Y | 7482 | Oxiana weak toxin OS=Naja oxiana OX=8657 PE=1 SV=1 |
| 43 | 439 | P01452\|3SA4\_NAJMO | 138.02 | 30 | 0 | 0 | 0 | 0 | 0 | 20 | 30 | 22 | 0 | 0 |  |  |  |  |  |  | 2.3E4 | 2.15E5 |  |  | 3 | 1 | 0 | 0 | 0 | 0 | 0 | 3 | 2 | 1 | 0 | 0 | Y | 6715 | Cytotoxin 4 OS=Naja mossambica OX=8644 PE=1 SV=1 |
| 117 | 97 | P01876\|IGHA1\_HUMAN | 137.23 | 18 | 0 | 18 | 0 | 0 | 0 | 0 | 0 | 0 | 0 | 0 |  | 8.89E5 |  |  |  |  |  |  |  |  | 5 | 5 | 0 | 5 | 0 | 0 | 0 | 0 | 0 | 0 | 0 | 0 | N | 37655 | Immunoglobulin heavy constant alpha 1 OS=Homo sapiens OX=9606 GN=IGHA1 PE=1 SV=2 |
| 117 | 98 | P20758\|IGHA1\_GORGO | 137.23 | 18 | 0 | 18 | 0 | 0 | 0 | 0 | 0 | 0 | 0 | 0 |  | 8.89E5 |  |  |  |  |  |  |  |  | 5 | 5 | 0 | 5 | 0 | 0 | 0 | 0 | 0 | 0 | 0 | 0 | N | 37756 | Ig alpha-1 chain C region OS=Gorilla gorilla gorilla OX=9595 GN=IGHA1 PE=1 SV=1 |
| 108 | 942 | P25676\|3SO61\_HEMHA | 137.10 | 25 | 0 | 0 | 0 | 23 | 25 | 23 | 0 | 0 | 0 | 0 |  |  |  | 4.57E5 | 9.58E6 | 0E0 |  |  |  |  | 4 | 4 | 0 | 0 | 0 | 1 | 6 | 1 | 0 | 0 | 0 | 0 | Y | 6917 | Weak toxin CM-1c OS=Hemachatus haemachatus OX=8626 PE=1 SV=1 |
| 114 | 73 | Q8WNW3\|PLAK\_PIG | 131.53 | 8 | 0 | 8 | 2 | 0 | 0 | 0 | 0 | 0 | 0 | 0 |  | 6.33E5 | 1.73E4 |  |  |  |  |  |  |  | 5 | 5 | 0 | 5 | 1 | 0 | 0 | 0 | 0 | 0 | 0 | 0 | N | 81850 | Junction plakoglobin OS=Sus scrofa OX=9823 GN=Jup PE=2 SV=1 |
| 114 | 74 | P14923\|PLAK\_HUMAN | 131.53 | 8 | 0 | 8 | 2 | 0 | 0 | 0 | 0 | 0 | 0 | 0 |  | 6.33E5 | 1.73E4 |  |  |  |  |  |  |  | 5 | 5 | 0 | 5 | 1 | 0 | 0 | 0 | 0 | 0 | 0 | 0 | N | 81745 | Junction plakoglobin OS=Homo sapiens OX=9606 GN=JUP PE=1 SV=3 |
| 114 | 76 | Q8SPJ1\|PLAK\_BOVIN | 131.53 | 8 | 0 | 8 | 2 | 0 | 0 | 0 | 0 | 0 | 0 | 0 |  | 6.33E5 | 1.73E4 |  |  |  |  |  |  |  | 5 | 5 | 0 | 5 | 1 | 0 | 0 | 0 | 0 | 0 | 0 | 0 | N | 81821 | Junction plakoglobin OS=Bos taurus OX=9913 GN=JUP PE=2 SV=1 |
| 114 | 77 | Q02257\|PLAK\_MOUSE | 131.53 | 8 | 0 | 8 | 2 | 0 | 0 | 0 | 0 | 0 | 0 | 0 |  | 6.33E5 | 1.73E4 |  |  |  |  |  |  |  | 5 | 5 | 0 | 5 | 1 | 0 | 0 | 0 | 0 | 0 | 0 | 0 | N | 81801 | Junction plakoglobin OS=Mus musculus OX=10090 GN=Jup PE=1 SV=3 |
| 114 | 78 | Q6P0K8\|PLAK\_RAT | 131.53 | 8 | 0 | 8 | 2 | 0 | 0 | 0 | 0 | 0 | 0 | 0 |  | 6.33E5 | 1.73E4 |  |  |  |  |  |  |  | 5 | 5 | 0 | 5 | 1 | 0 | 0 | 0 | 0 | 0 | 0 | 0 | N | 81801 | Junction plakoglobin OS=Rattus norvegicus OX=10116 GN=Jup PE=1 SV=1 |
| 107 | 312 | Q5ZPJ7\|VKT\_NAJAT | 128.37 | 37 | 0 | 0 | 0 | 23 | 36 | 0 | 0 | 0 | 0 | 0 |  |  |  | 6.99E4 | 1.61E5 |  |  |  |  |  | 5 | 4 | 0 | 0 | 0 | 1 | 4 | 0 | 0 | 0 | 0 | 0 | Y | 8815 | Kunitz-type serine protease inhibitor NACI OS=Naja atra OX=8656 PE=1 SV=1 |
| 110 | 79 | A1TKQ5\|CH60\_ACIAC | 126.73 | 7 | 0 | 7 | 0 | 0 | 0 | 0 | 0 | 0 | 0 | 0 |  | 1.53E5 |  |  |  |  |  |  |  |  | 4 | 1 | 0 | 4 | 0 | 0 | 0 | 0 | 0 | 0 | 0 | 0 | N | 57008 | 60 kDa chaperonin OS=Acidovorax citrulli (strain AAC00-1) OX=397945 GN=groL PE=3 SV=1 |
| 93 | 89 | #CONTAM#K1H1\_HUMAN\| | 121.32 | 10 | 0 | 2 | 10 | 0 | 2 | 0 | 2 | 0 | 0 | 2 |  |  | 3.31E4 |  |  |  |  |  |  |  | 4 | 3 | 0 | 2 | 4 | 0 | 1 | 0 | 1 | 0 | 0 | 1 | Y | 47232 | sp\|#CONTAM#K1H1\_HUMAN\| |
| 93 | 90 | A5A6M5\|K1H1\_PANTR | 121.32 | 10 | 0 | 2 | 10 | 0 | 2 | 0 | 2 | 0 | 0 | 2 |  |  | 3.31E4 |  |  |  |  |  |  |  | 4 | 3 | 0 | 2 | 4 | 0 | 1 | 0 | 1 | 0 | 0 | 1 | Y | 47247 | Keratin, type I cuticular Ha1 OS=Pan troglodytes OX=9598 GN=KRT31 PE=2 SV=1 |
| 93 | 91 | Q15323\|K1H1\_HUMAN | 121.32 | 10 | 0 | 2 | 10 | 0 | 2 | 0 | 2 | 0 | 0 | 2 |  |  | 3.31E4 |  |  |  |  |  |  |  | 4 | 3 | 0 | 2 | 4 | 0 | 1 | 0 | 1 | 0 | 0 | 1 | Y | 47237 | Keratin, type I cuticular Ha1 OS=Homo sapiens OX=9606 GN=KRT31 PE=1 SV=3 |
| 86 | 108 | Q8N1N4\|K2C78\_HUMAN | 120.50 | 6 | 1 | 5 | 0 | 0 | 5 | 0 | 0 | 0 | 1 | 0 |  | 1.28E5 |  |  | 9.88E3 |  |  |  |  |  | 4 | 2 | 1 | 4 | 0 | 0 | 3 | 0 | 0 | 0 | 1 | 0 | Y | 56866 | Keratin, type II cytoskeletal 78 OS=Homo sapiens OX=9606 GN=KRT78 PE=1 SV=2 |
| 115 | 120 | Q02413\|DSG1\_HUMAN | 114.35 | 4 | 0 | 2 | 3 | 0 | 0 | 0 | 0 | 0 | 0 | 0 |  | 6.35E4 | 1.89E4 |  |  |  |  |  |  |  | 3 | 3 | 0 | 2 | 2 | 0 | 0 | 0 | 0 | 0 | 0 | 0 | Y | 113748 | Desmoglein-1 OS=Homo sapiens OX=9606 GN=DSG1 PE=1 SV=2 |
| 116 | 361 | P00601\|PA2A3\_NAJME | 102.01 | 18 | 0 | 0 | 0 | 0 | 0 | 18 | 0 | 0 | 10 | 0 |  |  |  |  |  | 6.81E4 |  |  |  |  | 2 | 1 | 0 | 0 | 0 | 0 | 0 | 4 | 0 | 0 | 1 | 0 | Y | 13360 | Acidic phospholipase A2 DE-III OS=Naja melanoleuca OX=8643 PE=1 SV=1 |
| 116 | 362 | P00600\|PA2A2\_NAJME | 102.01 | 18 | 0 | 0 | 0 | 0 | 0 | 18 | 0 | 0 | 10 | 0 |  |  |  |  |  | 6.81E4 |  |  |  |  | 2 | 1 | 0 | 0 | 0 | 0 | 0 | 4 | 0 | 0 | 1 | 0 | Y | 13427 | Acidic phospholipase A2 DE-II OS=Naja melanoleuca OX=8643 PE=1 SV=1 |
| 126 | 245 | Q86YZ3\|HORN\_HUMAN | 101.92 | 2 | 0 | 2 | 0 | 0 | 0 | 0 | 0 | 0 | 0 | 0 |  | 8.8E4 |  |  |  |  |  |  |  |  | 2 | 2 | 0 | 2 | 0 | 0 | 0 | 0 | 0 | 0 | 0 | 0 | N | 282389 | Hornerin OS=Homo sapiens OX=9606 GN=HRNR PE=1 SV=2 |
| 100 | 567 | C1IC49\|3NO25\_WALAE | 97.77 | 24 | 0 | 0 | 0 | 24 | 0 | 10 | 10 | 10 | 0 | 0 |  |  |  | 1.3E4 |  |  |  |  |  |  | 2 | 1 | 0 | 0 | 0 | 8 | 0 | 1 | 1 | 1 | 0 | 0 | Y | 9715 | Three finger toxin W-V OS=Walterinnesia aegyptia OX=64182 PE=1 SV=1 |
| 133 | 225 | P78386\|KRT85\_HUMAN | 97.15 | 5 | 0 | 0 | 5 | 0 | 0 | 0 | 0 | 0 | 0 | 0 |  |  | 8.51E3 |  |  |  |  |  |  |  | 2 | 1 | 0 | 0 | 2 | 0 | 0 | 0 | 0 | 0 | 0 | 0 | Y | 55802 | Keratin, type II cuticular Hb5 OS=Homo sapiens OX=9606 GN=KRT85 PE=1 SV=1 |
| 133 | 226 | #CONTAM#KRHB5\_HUMAN\| | 97.15 | 5 | 0 | 0 | 5 | 0 | 0 | 0 | 0 | 0 | 0 | 0 |  |  | 8.51E3 |  |  |  |  |  |  |  | 2 | 1 | 0 | 0 | 2 | 0 | 0 | 0 | 0 | 0 | 0 | 0 | Y | 55802 | sp\|#CONTAM#KRHB5\_HUMAN\| |
| 106 | 246 | P06871\|TRY1\_CANLF | 96.54 | 12 | 4 | 8 | 4 | 4 | 8 | 0 | 0 | 0 | 4 | 0 |  | 2.05E5 |  |  |  |  |  |  |  |  | 3 | 1 | 1 | 2 | 1 | 1 | 2 | 0 | 0 | 0 | 1 | 0 | N | 26170 | Cationic trypsin OS=Canis lupus familiaris OX=9615 PE=2 SV=1 |
| 104 | 210 | P02845\|VIT2\_CHICK | 94.57 | 1 | 0 | 0 | 0 | 0 | 0 | 1 | 1 | 0 | 1 | 0 |  |  |  |  |  | 3.72E4 | 3.25E4 |  | 2.07E4 |  | 1 | 1 | 0 | 0 | 0 | 0 | 0 | 1 | 1 | 0 | 1 | 0 | N | 204807 | Vitellogenin-2 OS=Gallus gallus OX=9031 GN=VTG2 PE=1 SV=1 |
| 52 | 433 | P01453\|3SAA\_NAJHA | 90.14 | 33 | 13 | 13 | 13 | 13 | 13 | 13 | 0 | 33 | 13 | 13 |  |  |  |  |  |  |  | 1.84E5 |  |  | 2 | 1 | 1 | 1 | 1 | 1 | 1 | 14 | 0 | 3 | 1 | 1 | Y | 6682 | Cytotoxin 10 OS=Naja annulifera OX=96794 PE=1 SV=1 |
| 52 | 434 | P01454\|3SA9\_NAJHA | 90.14 | 33 | 13 | 13 | 13 | 13 | 13 | 13 | 0 | 33 | 13 | 13 |  |  |  |  |  |  |  | 1.84E5 |  |  | 2 | 1 | 1 | 1 | 1 | 1 | 1 | 14 | 0 | 3 | 1 | 1 | Y | 6669 | Cytotoxin 9 OS=Naja annulifera OX=96794 PE=1 SV=1 |
| 52 | 444 | P01455\|3SA1\_NAJHA | 90.14 | 33 | 13 | 13 | 13 | 13 | 13 | 13 | 0 | 33 | 13 | 13 |  |  |  |  |  |  |  | 1.84E5 |  |  | 2 | 1 | 1 | 1 | 1 | 1 | 1 | 14 | 0 | 3 | 1 | 1 | Y | 6696 | Cytotoxin 1 OS=Naja annulifera OX=96794 PE=1 SV=1 |
| 52 | 445 | P01456\|3SA1\_NAJNI | 90.14 | 33 | 13 | 13 | 13 | 13 | 13 | 13 | 0 | 33 | 13 | 13 |  |  |  |  |  |  |  | 1.84E5 |  |  | 2 | 1 | 1 | 1 | 1 | 1 | 1 | 14 | 0 | 3 | 1 | 1 | Y | 6697 | Cytotoxin 1 OS=Naja nivea OX=8655 PE=1 SV=1 |
| 127 | 368 | B6EWW8\|V5NTD\_GLOBR | 88.05 | 3 | 0 | 0 | 0 | 0 | 0 | 3 | 3 | 0 | 0 | 0 |  |  |  |  |  | 1.15E5 | 3.95E5 |  |  |  | 1 | 1 | 0 | 0 | 0 | 0 | 0 | 1 | 1 | 0 | 0 | 0 | Y | 64434 | Snake venom 5'-nucleotidase OS=Gloydius brevicaudus OX=259325 PE=2 SV=1 |
| 113 | 496 | P35030\|TRY3\_HUMAN | 86.61 | 4 | 0 | 4 | 0 | 0 | 0 | 0 | 0 | 0 | 0 | 0 |  | 5.33E7 |  |  |  |  |  |  |  |  | 3 | 2 | 0 | 3 | 0 | 0 | 0 | 0 | 0 | 0 | 0 | 0 | N | 32529 | Trypsin-3 OS=Homo sapiens OX=9606 GN=PRSS3 PE=1 SV=2 |
| 145 | 473 | O62823\|CASA1\_BUBBU | 85.24 | 10 | 0 | 0 | 0 | 0 | 0 | 0 | 0 | 10 | 0 | 0 |  |  |  |  |  |  |  | 7.81E4 |  |  | 2 | 2 | 0 | 0 | 0 | 0 | 0 | 0 | 0 | 2 | 0 | 0 | N | 24327 | Alpha-S1-casein OS=Bubalus bubalis OX=89462 GN=CSN1S1 PE=2 SV=2 |
| 145 | 474 | #CONTAM#CAS1\_BOVIN\| | 85.24 | 10 | 0 | 0 | 0 | 0 | 0 | 0 | 0 | 10 | 0 | 0 |  |  |  |  |  |  |  | 7.81E4 |  |  | 2 | 2 | 0 | 0 | 0 | 0 | 0 | 0 | 0 | 2 | 0 | 0 | N | 24529 | sp\|#CONTAM#CAS1\_BOVIN\| |
| 145 | 475 | P02662\|CASA1\_BOVIN | 85.24 | 10 | 0 | 0 | 0 | 0 | 0 | 0 | 0 | 10 | 0 | 0 |  |  |  |  |  |  |  | 7.81E4 |  |  | 2 | 2 | 0 | 0 | 0 | 0 | 0 | 0 | 0 | 2 | 0 | 0 | N | 24529 | Alpha-S1-casein OS=Bos taurus OX=9913 GN=CSN1S1 PE=1 SV=2 |
| 105 | 229 | #CONTAM#KRHB4\_HUMAN\| | 84.36 | 6 | 1 | 3 | 3 | 0 | 1 | 0 | 0 | 0 | 1 | 0 |  |  | 5.28E3 |  |  |  |  |  |  |  | 3 | 1 | 1 | 2 | 1 | 0 | 1 | 0 | 0 | 0 | 1 | 0 | Y | 64895 | sp\|#CONTAM#KRHB4\_HUMAN\| |
| 105 | 230 | Q9NSB2\|KRT84\_HUMAN | 84.36 | 6 | 1 | 3 | 3 | 0 | 1 | 0 | 0 | 0 | 1 | 0 |  |  | 5.28E3 |  |  |  |  |  |  |  | 3 | 1 | 1 | 2 | 1 | 0 | 1 | 0 | 0 | 0 | 1 | 0 | Y | 64842 | Keratin, type II cuticular Hb4 OS=Homo sapiens OX=9606 GN=KRT84 PE=2 SV=2 |
| 119 | 645 | Q9TSI0\|CASB\_BUBBU | 83.33 | 4 | 0 | 0 | 0 | 0 | 0 | 0 | 0 | 0 | 4 | 0 |  |  |  |  |  |  |  |  | 1.17E4 |  | 1 | 1 | 0 | 0 | 0 | 0 | 0 | 0 | 0 | 0 | 1 | 0 | N | 25106 | Beta-casein OS=Bubalus bubalis OX=89462 GN=CSN2 PE=2 SV=1 |
| 138 | 609 | Q9NZT1\|CALL5\_HUMAN | 78.97 | 16 | 0 | 0 | 0 | 0 | 0 | 0 | 0 | 16 | 0 | 0 |  |  |  |  |  |  |  | 1.39E4 |  |  | 1 | 1 | 0 | 0 | 0 | 0 | 0 | 0 | 0 | 1 | 0 | 0 | N | 15893 | Calmodulin-like protein 5 OS=Homo sapiens OX=9606 GN=CALML5 PE=1 SV=2 |
| 200 | 941 | P82849\|3S1B2\_NAJKA | 78.89 | 27 | 0 | 0 | 27 | 0 | 0 | 0 | 0 | 0 | 0 | 0 |  |  | 2.9E6 |  |  |  |  |  |  |  | 1 | 1 | 0 | 0 | 1 | 0 | 0 | 0 | 0 | 0 | 0 | 0 | Y | 6862 | Cobrotoxin II OS=Naja kaouthia OX=8649 PE=1 SV=1 |
| 129 | 329 | P10981\|ACT5\_DROME | 77.54 | 3 | 0 | 3 | 0 | 0 | 0 | 0 | 0 | 0 | 0 | 0 |  | 2.81E4 |  |  |  |  |  |  |  |  | 1 | 1 | 0 | 1 | 0 | 0 | 0 | 0 | 0 | 0 | 0 | 0 | N | 41802 | Actin-87E OS=Drosophila melanogaster OX=7227 GN=Act87E PE=1 SV=1 |
| 129 | 330 | P04752\|ACT3\_XENLA | 77.54 | 3 | 0 | 3 | 0 | 0 | 0 | 0 | 0 | 0 | 0 | 0 |  | 2.81E4 |  |  |  |  |  |  |  |  | 1 | 1 | 0 | 1 | 0 | 0 | 0 | 0 | 0 | 0 | 0 | 0 | N | 41984 | Actin, alpha skeletal muscle 3 OS=Xenopus laevis OX=8355 GN=act3 PE=2 SV=2 |
| 129 | 331 | P62737\|ACTA\_MOUSE | 77.54 | 3 | 0 | 3 | 0 | 0 | 0 | 0 | 0 | 0 | 0 | 0 |  | 2.81E4 |  |  |  |  |  |  |  |  | 1 | 1 | 0 | 1 | 0 | 0 | 0 | 0 | 0 | 0 | 0 | 0 | N | 42009 | Actin, aortic smooth muscle OS=Mus musculus OX=10090 GN=Acta2 PE=1 SV=1 |
| 129 | 332 | P60710\|ACTB\_MOUSE | 77.54 | 3 | 0 | 3 | 0 | 0 | 0 | 0 | 0 | 0 | 0 | 0 |  | 2.81E4 |  |  |  |  |  |  |  |  | 1 | 1 | 0 | 1 | 0 | 0 | 0 | 0 | 0 | 0 | 0 | 0 | N | 41737 | Actin, cytoplasmic 1 OS=Mus musculus OX=10090 GN=Actb PE=1 SV=1 |
| 129 | 333 | O18840\|ACTB\_CANLF | 77.54 | 3 | 0 | 3 | 0 | 0 | 0 | 0 | 0 | 0 | 0 | 0 |  | 2.81E4 |  |  |  |  |  |  |  |  | 1 | 1 | 0 | 1 | 0 | 0 | 0 | 0 | 0 | 0 | 0 | 0 | N | 41737 | Actin, cytoplasmic 1 OS=Canis lupus familiaris OX=9615 GN=ACTB PE=2 SV=3 |
| 129 | 334 | Q5R6G0\|ACTB\_PONAB | 77.54 | 3 | 0 | 3 | 0 | 0 | 0 | 0 | 0 | 0 | 0 | 0 |  | 2.81E4 |  |  |  |  |  |  |  |  | 1 | 1 | 0 | 1 | 0 | 0 | 0 | 0 | 0 | 0 | 0 | 0 | N | 41737 | Actin, cytoplasmic 1 OS=Pongo abelii OX=9601 GN=ACTB PE=2 SV=1 |
| 129 | 335 | Q711N9\|ACTB\_MESAU | 77.54 | 3 | 0 | 3 | 0 | 0 | 0 | 0 | 0 | 0 | 0 | 0 |  | 2.81E4 |  |  |  |  |  |  |  |  | 1 | 1 | 0 | 1 | 0 | 0 | 0 | 0 | 0 | 0 | 0 | 0 | N | 41737 | Actin, cytoplasmic 1 OS=Mesocricetus auratus OX=10036 GN=ACTB PE=1 SV=1 |
| 129 | 336 | P63260\|ACTG\_MOUSE | 77.54 | 3 | 0 | 3 | 0 | 0 | 0 | 0 | 0 | 0 | 0 | 0 |  | 2.81E4 |  |  |  |  |  |  |  |  | 1 | 1 | 0 | 1 | 0 | 0 | 0 | 0 | 0 | 0 | 0 | 0 | N | 41793 | Actin, cytoplasmic 2 OS=Mus musculus OX=10090 GN=Actg1 PE=1 SV=1 |
| 129 | 337 | Q5JAK2\|ACTG\_PELLE | 77.54 | 3 | 0 | 3 | 0 | 0 | 0 | 0 | 0 | 0 | 0 | 0 |  | 2.81E4 |  |  |  |  |  |  |  |  | 1 | 1 | 0 | 1 | 0 | 0 | 0 | 0 | 0 | 0 | 0 | 0 | N | 41779 | Actin, cytoplasmic 2 OS=Pelophylax lessonae OX=45623 GN=actg1 PE=2 SV=1 |
| 129 | 338 | P63261\|ACTG\_HUMAN | 77.54 | 3 | 0 | 3 | 0 | 0 | 0 | 0 | 0 | 0 | 0 | 0 |  | 2.81E4 |  |  |  |  |  |  |  |  | 1 | 1 | 0 | 1 | 0 | 0 | 0 | 0 | 0 | 0 | 0 | 0 | N | 41793 | Actin, cytoplasmic 2 OS=Homo sapiens OX=9606 GN=ACTG1 PE=1 SV=1 |
| 129 | 339 | P53505\|ACT5\_XENLA | 77.54 | 3 | 0 | 3 | 0 | 0 | 0 | 0 | 0 | 0 | 0 | 0 |  | 2.81E4 |  |  |  |  |  |  |  |  | 1 | 1 | 0 | 1 | 0 | 0 | 0 | 0 | 0 | 0 | 0 | 0 | N | 41850 | Actin, cytoplasmic type 5 OS=Xenopus laevis OX=8355 PE=3 SV=1 |
| 129 | 340 | P84185\|ACT5C\_ANOGA | 77.54 | 3 | 0 | 3 | 0 | 0 | 0 | 0 | 0 | 0 | 0 | 0 |  | 2.81E4 |  |  |  |  |  |  |  |  | 1 | 1 | 0 | 1 | 0 | 0 | 0 | 0 | 0 | 0 | 0 | 0 | N | 41822 | Actin-5C OS=Anopheles gambiae OX=7165 GN=Act5C PE=2 SV=1 |
| 129 | 341 | P68135\|ACTS\_RABIT | 77.54 | 3 | 0 | 3 | 0 | 0 | 0 | 0 | 0 | 0 | 0 | 0 |  | 2.81E4 |  |  |  |  |  |  |  |  | 1 | 1 | 0 | 1 | 0 | 0 | 0 | 0 | 0 | 0 | 0 | 0 | N | 42051 | Actin, alpha skeletal muscle OS=Oryctolagus cuniculus OX=9986 GN=ACTA1 PE=1 SV=1 |
| 129 | 342 | P68137\|ACTS\_PIG | 77.54 | 3 | 0 | 3 | 0 | 0 | 0 | 0 | 0 | 0 | 0 | 0 |  | 2.81E4 |  |  |  |  |  |  |  |  | 1 | 1 | 0 | 1 | 0 | 0 | 0 | 0 | 0 | 0 | 0 | 0 | N | 42051 | Actin, alpha skeletal muscle OS=Sus scrofa OX=9823 GN=ACTA1 PE=1 SV=1 |
| 129 | 344 | P53455\|ACT\_AJECG | 77.54 | 3 | 0 | 3 | 0 | 0 | 0 | 0 | 0 | 0 | 0 | 0 |  | 2.81E4 |  |  |  |  |  |  |  |  | 1 | 1 | 0 | 1 | 0 | 0 | 0 | 0 | 0 | 0 | 0 | 0 | N | 41609 | Actin OS=Ajellomyces capsulatus (strain G186AR / H82 / ATCC MYA-2454 / RMSCC 2432) OX=447093 GN=HCBG\_08711 PE=2 SV=2 |
| 129 | 345 | P83967\|ACT6\_DROME | 77.54 | 3 | 0 | 3 | 0 | 0 | 0 | 0 | 0 | 0 | 0 | 0 |  | 2.81E4 |  |  |  |  |  |  |  |  | 1 | 1 | 0 | 1 | 0 | 0 | 0 | 0 | 0 | 0 | 0 | 0 | N | 41700 | Actin, indirect flight muscle OS=Drosophila melanogaster OX=7227 GN=Act88F PE=1 SV=1 |
| 129 | 347 | P91754\|ACT\_LUMRU | 77.54 | 3 | 0 | 3 | 0 | 0 | 0 | 0 | 0 | 0 | 0 | 0 |  | 2.81E4 |  |  |  |  |  |  |  |  | 1 | 1 | 0 | 1 | 0 | 0 | 0 | 0 | 0 | 0 | 0 | 0 | N | 41323 | Actin (Fragment) OS=Lumbricus rubellus OX=35632 PE=2 SV=1 |
| 129 | 348 | P18499\|ACTF\_STRPU | 77.54 | 3 | 0 | 3 | 0 | 0 | 0 | 0 | 0 | 0 | 0 | 0 |  | 2.81E4 |  |  |  |  |  |  |  |  | 1 | 1 | 0 | 1 | 0 | 0 | 0 | 0 | 0 | 0 | 0 | 0 | N | 41832 | Actin, cytoskeletal 3B OS=Strongylocentrotus purpuratus OX=7668 GN=CYIIIB PE=2 SV=1 |
| 129 | 349 | P53474\|ACTE\_STRPU | 77.54 | 3 | 0 | 3 | 0 | 0 | 0 | 0 | 0 | 0 | 0 | 0 |  | 2.81E4 |  |  |  |  |  |  |  |  | 1 | 1 | 0 | 1 | 0 | 0 | 0 | 0 | 0 | 0 | 0 | 0 | N | 41904 | Actin, cytoskeletal 3A OS=Strongylocentrotus purpuratus OX=7668 GN=CYIIIA PE=3 SV=1 |
| 122 | 354 | P01040\|CYTA\_HUMAN | 75.35 | 24 | 0 | 12 | 12 | 0 | 0 | 0 | 0 | 0 | 0 | 0 |  | 8.21E4 | 9.8E3 |  |  |  |  |  |  |  | 2 | 2 | 0 | 1 | 1 | 0 | 0 | 0 | 0 | 0 | 0 | 0 | N | 11006 | Cystatin-A OS=Homo sapiens OX=9606 GN=CSTA PE=1 SV=1 |
| 118 | 351 | Q5T749\|KPRP\_HUMAN | 71.10 | 2 | 0 | 0 | 0 | 0 | 2 | 0 | 0 | 0 | 0 | 0 |  |  |  |  | 5.15E4 |  |  |  |  |  | 1 | 1 | 0 | 0 | 0 | 0 | 1 | 0 | 0 | 0 | 0 | 0 | Y | 64136 | Keratinocyte proline-rich protein OS=Homo sapiens OX=9606 GN=KPRP PE=1 SV=1 |
| 147 | 949 | P14613\|3S1B1\_NAJKA | 68.86 | 19 | 0 | 19 | 0 | 19 | 0 | 0 | 0 | 0 | 0 | 0 |  | 2.97E5 |  | 2.32E4 |  |  |  |  |  |  | 1 | 1 | 0 | 1 | 0 | 1 | 0 | 0 | 0 | 0 | 0 | 0 | Y | 6983 | Short neurotoxin 1 OS=Naja kaouthia OX=8649 PE=1 SV=1 |
| 147 | 950 | Q9PSN6\|3S13\_NAJSP | 68.86 | 19 | 0 | 19 | 0 | 19 | 0 | 0 | 0 | 0 | 0 | 0 |  | 2.97E5 |  | 2.32E4 |  |  |  |  |  |  | 1 | 1 | 0 | 1 | 0 | 1 | 0 | 0 | 0 | 0 | 0 | 0 | Y | 6958 | Neurotoxin 3 OS=Naja sputatrix OX=33626 PE=1 SV=1 |
| 147 | 951 | P60771\|3S1CB\_NAJKA | 68.86 | 14 | 0 | 14 | 0 | 14 | 0 | 0 | 0 | 0 | 0 | 0 |  | 2.97E5 |  | 2.32E4 |  |  |  |  |  |  | 1 | 1 | 0 | 1 | 0 | 1 | 0 | 0 | 0 | 0 | 0 | 0 | Y | 9262 | Cobrotoxin OS=Naja kaouthia OX=8649 PE=1 SV=2 |
| 147 | 952 | P60770\|3S1CB\_NAJAT | 68.86 | 14 | 0 | 14 | 0 | 14 | 0 | 0 | 0 | 0 | 0 | 0 |  | 2.97E5 |  | 2.32E4 |  |  |  |  |  |  | 1 | 1 | 0 | 1 | 0 | 1 | 0 | 0 | 0 | 0 | 0 | 0 | Y | 9262 | Cobrotoxin OS=Naja atra OX=8656 PE=1 SV=1 |
| 147 | 953 | Q9PTT0\|3S1CB\_NAJNA | 68.86 | 14 | 0 | 14 | 0 | 14 | 0 | 0 | 0 | 0 | 0 | 0 |  | 2.97E5 |  | 2.32E4 |  |  |  |  |  |  | 1 | 1 | 0 | 1 | 0 | 1 | 0 | 0 | 0 | 0 | 0 | 0 | Y | 9262 | Cobrotoxin homolog OS=Naja naja OX=35670 PE=1 SV=1 |
| 124 | 435 | Q5D862\|FILA2\_HUMAN | 67.65 | 0 | 0 | 0 | 0 | 0 | 0 | 0 | 0 | 0 | 0 | 0 |  |  |  | 1.63E4 |  | 2.51E4 |  |  |  |  | 1 | 1 | 0 | 0 | 0 | 1 | 0 | 1 | 0 | 0 | 0 | 0 | N | 248072 | Filaggrin-2 OS=Homo sapiens OX=9606 GN=FLG2 PE=1 SV=1 |
| 120 | 520 | P23764\|GPX3\_RAT | 65.81 | 6 | 0 | 0 | 0 | 0 | 0 | 6 | 0 | 6 | 6 | 0 |  |  |  |  |  | 7.57E4 |  | 2.1E5 | 2.48E4 |  | 2 | 2 | 0 | 0 | 0 | 0 | 0 | 2 | 0 | 1 | 1 | 0 | Y | 25424 | Glutathione peroxidase 3 OS=Rattus norvegicus OX=10116 GN=Gpx3 PE=2 SV=2 |
| 120 | 538 | Q4AEH4\|GPX3\_MACFU | 65.81 | 6 | 0 | 0 | 0 | 0 | 0 | 6 | 0 | 6 | 6 | 0 |  |  |  |  |  | 7.57E4 |  | 2.1E5 | 2.48E4 |  | 2 | 2 | 0 | 0 | 0 | 0 | 0 | 2 | 0 | 1 | 1 | 0 | Y | 25576 | Glutathione peroxidase 3 OS=Macaca fuscata fuscata OX=9543 GN=GPX3 PE=2 SV=2 |
| 120 | 627 | P22352\|GPX3\_HUMAN | 65.81 | 6 | 0 | 0 | 0 | 0 | 0 | 6 | 0 | 6 | 6 | 0 |  |  |  |  |  | 7.57E4 |  | 2.1E5 | 2.48E4 |  | 2 | 2 | 0 | 0 | 0 | 0 | 0 | 2 | 0 | 1 | 1 | 0 | Y | 25552 | Glutathione peroxidase 3 OS=Homo sapiens OX=9606 GN=GPX3 PE=1 SV=2 |
| 120 | 628 | Q5RFG3\|GPX3\_PONPY | 65.81 | 6 | 0 | 0 | 0 | 0 | 0 | 6 | 0 | 6 | 6 | 0 |  |  |  |  |  | 7.57E4 |  | 2.1E5 | 2.48E4 |  | 2 | 2 | 0 | 0 | 0 | 0 | 0 | 2 | 0 | 1 | 1 | 0 | Y | 25562 | Glutathione peroxidase 3 OS=Pongo pygmaeus OX=9600 GN=GPX3 PE=2 SV=3 |
| 120 | 685 | P37141\|GPX3\_BOVIN | 65.81 | 6 | 0 | 0 | 0 | 0 | 0 | 6 | 0 | 6 | 6 | 0 |  |  |  |  |  | 7.57E4 |  | 2.1E5 | 2.48E4 |  | 2 | 2 | 0 | 0 | 0 | 0 | 0 | 2 | 0 | 1 | 1 | 0 | Y | 25663 | Glutathione peroxidase 3 OS=Bos taurus OX=9913 GN=GPX3 PE=2 SV=2 |
| 120 | 759 | Q4AEH5\|GPX3\_HYLLA | 65.81 | 6 | 0 | 0 | 0 | 0 | 0 | 6 | 0 | 6 | 6 | 0 |  |  |  |  |  | 7.57E4 |  | 2.1E5 | 2.48E4 |  | 2 | 2 | 0 | 0 | 0 | 0 | 0 | 2 | 0 | 1 | 1 | 0 | Y | 25598 | Glutathione peroxidase 3 OS=Hylobates lar OX=9580 GN=GPX3 PE=2 SV=2 |
| 120 | 772 | P21765\|GPX5\_MOUSE | 65.81 | 6 | 0 | 0 | 0 | 0 | 0 | 6 | 0 | 6 | 6 | 0 |  |  |  |  |  | 7.57E4 |  | 2.1E5 | 2.48E4 |  | 2 | 2 | 0 | 0 | 0 | 0 | 0 | 2 | 0 | 1 | 1 | 0 | Y | 25393 | Epididymal secretory glutathione peroxidase OS=Mus musculus OX=10090 GN=Gpx5 PE=2 SV=3 |
| 120 | 1014 | P30710\|GPX5\_RAT | 65.81 | 6 | 0 | 0 | 0 | 0 | 0 | 6 | 0 | 6 | 6 | 0 |  |  |  |  |  | 7.57E4 |  | 2.1E5 | 2.48E4 |  | 2 | 2 | 0 | 0 | 0 | 0 | 0 | 2 | 0 | 1 | 1 | 0 | Y | 25385 | Epididymal secretory glutathione peroxidase OS=Rattus norvegicus OX=10116 GN=Gpx5 PE=2 SV=1 |
| 137 | 400 | P12273\|PIP\_HUMAN | 65.45 | 10 | 0 | 10 | 0 | 0 | 10 | 0 | 0 | 0 | 0 | 0 |  | 3.63E4 |  |  | 1.28E4 |  |  |  |  |  | 1 | 1 | 0 | 1 | 0 | 0 | 1 | 0 | 0 | 0 | 0 | 0 | Y | 16572 | Prolactin-inducible protein OS=Homo sapiens OX=9606 GN=PIP PE=1 SV=1 |
| 137 | 477 | A0A885\|PIP\_GORGO | 65.45 | 10 | 0 | 10 | 0 | 0 | 10 | 0 | 0 | 0 | 0 | 0 |  | 3.63E4 |  |  | 1.28E4 |  |  |  |  |  | 1 | 1 | 0 | 1 | 0 | 0 | 1 | 0 | 0 | 0 | 0 | 0 | Y | 16503 | Prolactin-inducible protein homolog OS=Gorilla gorilla gorilla OX=9595 GN=PIP PE=3 SV=2 |
| 137 | 478 | P60989\|PIP\_PANTR | 65.45 | 10 | 0 | 10 | 0 | 0 | 10 | 0 | 0 | 0 | 0 | 0 |  | 3.63E4 |  |  | 1.28E4 |  |  |  |  |  | 1 | 1 | 0 | 1 | 0 | 0 | 1 | 0 | 0 | 0 | 0 | 0 | Y | 16575 | Prolactin-inducible protein homolog OS=Pan troglodytes OX=9598 GN=PIP PE=3 SV=1 |
| 137 | 738 | A0A888\|PIP\_PONPY | 65.45 | 10 | 0 | 10 | 0 | 0 | 10 | 0 | 0 | 0 | 0 | 0 |  | 3.63E4 |  |  | 1.28E4 |  |  |  |  |  | 1 | 1 | 0 | 1 | 0 | 0 | 1 | 0 | 0 | 0 | 0 | 0 | Y | 16546 | Prolactin-inducible protein homolog OS=Pongo pygmaeus OX=9600 GN=PIP PE=3 SV=1 |
| 137 | 962 | A0A889\|PIP\_SYMSY | 65.45 | 10 | 0 | 10 | 0 | 0 | 10 | 0 | 0 | 0 | 0 | 0 |  | 3.63E4 |  |  | 1.28E4 |  |  |  |  |  | 1 | 1 | 0 | 1 | 0 | 0 | 1 | 0 | 0 | 0 | 0 | 0 | Y | 16592 | Prolactin-inducible protein homolog OS=Symphalangus syndactylus OX=9590 GN=PIP PE=3 SV=1 |
| 137 | 963 | A0A890\|PIP\_HYLAG | 65.45 | 10 | 0 | 10 | 0 | 0 | 10 | 0 | 0 | 0 | 0 | 0 |  | 3.63E4 |  |  | 1.28E4 |  |  |  |  |  | 1 | 1 | 0 | 1 | 0 | 0 | 1 | 0 | 0 | 0 | 0 | 0 | Y | 16537 | Prolactin-inducible protein homolog OS=Hylobates agilis OX=9579 GN=PIP PE=3 SV=1 |
| 134 | 374 | Q08554\|DSC1\_HUMAN | 65.24 | 2 | 0 | 1 | 0 | 0 | 1 | 0 | 0 | 0 | 0 | 0 |  | 3.77E4 |  |  | 1.23E4 |  |  |  |  |  | 2 | 2 | 0 | 1 | 0 | 0 | 1 | 0 | 0 | 0 | 0 | 0 | Y | 99987 | Desmocollin-1 OS=Homo sapiens OX=9606 GN=DSC1 PE=1 SV=2 |
| 207 | 967 | E3P6P4\|CYT\_NAJKA | 63.66 | 9 | 0 | 0 | 0 | 0 | 0 | 0 | 0 | 9 | 0 | 0 |  |  |  |  |  |  |  | 1.37E5 |  |  | 1 | 1 | 0 | 0 | 0 | 0 | 0 | 0 | 0 | 1 | 0 | 0 | N | 15772 | Cystatin OS=Naja kaouthia OX=8649 PE=2 SV=1 |
| 146 | 664 | Q5T750\|XP32\_HUMAN | 61.09 | 3 | 0 | 3 | 0 | 0 | 0 | 0 | 0 | 0 | 0 | 0 |  | 5.88E5 |  |  |  |  |  |  |  |  | 1 | 1 | 0 | 1 | 0 | 0 | 0 | 0 | 0 | 0 | 0 | 0 | N | 26238 | Skin-specific protein 32 OS=Homo sapiens OX=9606 GN=XP32 PE=1 SV=1 |
| 130 | 355 | Q96P63\|SPB12\_HUMAN | 60.62 | 2 | 0 | 2 | 0 | 0 | 0 | 0 | 0 | 0 | 0 | 0 |  | 0E0 |  |  |  |  |  |  |  |  | 1 | 1 | 0 | 1 | 0 | 0 | 0 | 0 | 0 | 0 | 0 | 0 | N | 46276 | Serpin B12 OS=Homo sapiens OX=9606 GN=SERPINB12 PE=1 SV=1 |
| 163 | 663 | A2SLF9\|EFTU\_METPP | 57.02 | 3 | 0 | 3 | 0 | 0 | 0 | 0 | 0 | 0 | 0 | 0 |  | 8.54E4 |  |  |  |  |  |  |  |  | 1 | 1 | 0 | 1 | 0 | 0 | 0 | 0 | 0 | 0 | 0 | 0 | N | 43088 | Elongation factor Tu OS=Methylibium petroleiphilum (strain ATCC BAA-1232 / LMG 22953 / PM1) OX=420662 GN=tuf1 PE=3 SV=1 |
| 159 | 513 | B8HVR7\|EFTU\_CYAP4 | 56.51 | 3 | 0 | 3 | 0 | 0 | 0 | 0 | 0 | 0 | 0 | 0 |  | 5.04E4 |  |  |  |  |  |  |  |  | 1 | 1 | 0 | 1 | 0 | 0 | 0 | 0 | 0 | 0 | 0 | 0 | N | 44753 | Elongation factor Tu OS=Cyanothece sp. (strain PCC 7425 / ATCC 29141) OX=395961 GN=tuf PE=3 SV=1 |
| 192 | 841 | A4VVK7\|DNLJ\_STRSY | 56.47 | 1 | 0 | 0 | 0 | 0 | 1 | 0 | 0 | 0 | 0 | 0 |  |  |  |  | 2.43E4 |  |  |  |  |  | 1 | 1 | 0 | 0 | 0 | 0 | 1 | 0 | 0 | 0 | 0 | 0 | N | 71559 | DNA ligase OS=Streptococcus suis (strain 05ZYH33) OX=391295 GN=ligA PE=3 SV=1 |
| 192 | 842 | A4W1W6\|DNLJ\_STRS2 | 56.47 | 1 | 0 | 0 | 0 | 0 | 1 | 0 | 0 | 0 | 0 | 0 |  |  |  |  | 2.43E4 |  |  |  |  |  | 1 | 1 | 0 | 0 | 0 | 0 | 1 | 0 | 0 | 0 | 0 | 0 | N | 71586 | DNA ligase OS=Streptococcus suis (strain 98HAH33) OX=391296 GN=ligA PE=3 SV=1 |
| 192 | 1013 | B2KE31\|DNLJ\_ELUMP | 56.47 | 1 | 0 | 0 | 0 | 0 | 1 | 0 | 0 | 0 | 0 | 0 |  |  |  |  | 2.43E4 |  |  |  |  |  | 1 | 1 | 0 | 0 | 0 | 0 | 1 | 0 | 0 | 0 | 0 | 0 | N | 72029 | DNA ligase OS=Elusimicrobium minutum (strain Pei191) OX=445932 GN=ligA PE=3 SV=1 |
| 189 | 635 | A2YVE5\|H2A3\_ORYSI | 52.83 | 7 | 0 | 7 | 0 | 0 | 0 | 0 | 0 | 0 | 0 | 0 |  | 6.76E4 |  |  |  |  |  |  |  |  | 1 | 1 | 0 | 1 | 0 | 0 | 0 | 0 | 0 | 0 | 0 | 0 | N | 13917 | Probable histone H2A.3 OS=Oryza sativa subsp. indica OX=39946 GN=OsI\_028288 PE=3 SV=1 |
| 189 | 789 | Q8LLP5\|H2AXA\_ORYSJ | 52.83 | 7 | 0 | 7 | 0 | 0 | 0 | 0 | 0 | 0 | 0 | 0 |  | 6.76E4 |  |  |  |  |  |  |  |  | 1 | 1 | 0 | 1 | 0 | 0 | 0 | 0 | 0 | 0 | 0 | 0 | N | 14271 | Probable histone H2AXa OS=Oryza sativa subsp. japonica OX=39947 GN=Os03g0721900 PE=3 SV=1 |
| 189 | 791 | P69142\|H2AE\_PSAMI | 52.83 | 7 | 0 | 7 | 0 | 0 | 0 | 0 | 0 | 0 | 0 | 0 |  | 6.76E4 |  |  |  |  |  |  |  |  | 1 | 1 | 0 | 1 | 0 | 0 | 0 | 0 | 0 | 0 | 0 | 0 | N | 13332 | Histone H2A, embryonic OS=Psammechinus miliaris OX=7660 PE=3 SV=2 |
| 189 | 826 | Q3ZBX9\|H2AJ\_BOVIN | 52.83 | 7 | 0 | 7 | 0 | 0 | 0 | 0 | 0 | 0 | 0 | 0 |  | 6.76E4 |  |  |  |  |  |  |  |  | 1 | 1 | 0 | 1 | 0 | 0 | 0 | 0 | 0 | 0 | 0 | 0 | N | 14019 | Histone H2A.J OS=Bos taurus OX=9913 GN=H2AFJ PE=2 SV=1 |
| 189 | 976 | Q6WV67\|H2A\_MYTTR | 52.83 | 7 | 0 | 7 | 0 | 0 | 0 | 0 | 0 | 0 | 0 | 0 |  | 6.76E4 |  |  |  |  |  |  |  |  | 1 | 1 | 0 | 1 | 0 | 0 | 0 | 0 | 0 | 0 | 0 | 0 | N | 13288 | Histone H2A OS=Mytilus trossulus OX=6551 PE=3 SV=3 |
| 189 | 977 | Q8I0T3\|H2A\_MYTED | 52.83 | 7 | 0 | 7 | 0 | 0 | 0 | 0 | 0 | 0 | 0 | 0 |  | 6.76E4 |  |  |  |  |  |  |  |  | 1 | 1 | 0 | 1 | 0 | 0 | 0 | 0 | 0 | 0 | 0 | 0 | N | 13360 | Histone H2A OS=Mytilus edulis OX=6550 PE=3 SV=3 |
| 189 | 978 | Q6WV88\|H2A\_MYTGA | 52.83 | 7 | 0 | 7 | 0 | 0 | 0 | 0 | 0 | 0 | 0 | 0 |  | 6.76E4 |  |  |  |  |  |  |  |  | 1 | 1 | 0 | 1 | 0 | 0 | 0 | 0 | 0 | 0 | 0 | 0 | N | 13360 | Histone H2A OS=Mytilus galloprovincialis OX=29158 PE=3 SV=3 |
| 189 | 979 | Q07135\|H2AO\_CHITH | 52.83 | 7 | 0 | 7 | 0 | 0 | 0 | 0 | 0 | 0 | 0 | 0 |  | 6.76E4 |  |  |  |  |  |  |  |  | 1 | 1 | 0 | 1 | 0 | 0 | 0 | 0 | 0 | 0 | 0 | 0 | N | 13474 | Histone H2A, orphon OS=Chironomus thummi thummi OX=7155 PE=3 SV=2 |
| 189 | 980 | Q5ZMD6\|H2AZ\_CHICK | 52.83 | 7 | 0 | 7 | 0 | 0 | 0 | 0 | 0 | 0 | 0 | 0 |  | 6.76E4 |  |  |  |  |  |  |  |  | 1 | 1 | 0 | 1 | 0 | 0 | 0 | 0 | 0 | 0 | 0 | 0 | N | 13583 | Histone H2A.Z OS=Gallus gallus OX=9031 GN=H2AFZ PE=1 SV=3 |
| 189 | 981 | Q32LA7\|H2AV\_BOVIN | 52.83 | 7 | 0 | 7 | 0 | 0 | 0 | 0 | 0 | 0 | 0 | 0 |  | 6.76E4 |  |  |  |  |  |  |  |  | 1 | 1 | 0 | 1 | 0 | 0 | 0 | 0 | 0 | 0 | 0 | 0 | N | 13509 | Histone H2A.V OS=Bos taurus OX=9913 GN=H2AFV PE=2 SV=3 |
| 189 | 982 | P22647\|H2AZ\_ONCMY | 52.83 | 7 | 0 | 7 | 0 | 0 | 0 | 0 | 0 | 0 | 0 | 0 |  | 6.76E4 |  |  |  |  |  |  |  |  | 1 | 1 | 0 | 1 | 0 | 0 | 0 | 0 | 0 | 0 | 0 | 0 | N | 13553 | Histone H2A.Z OS=Oncorhynchus mykiss OX=8022 GN=h2afz PE=1 SV=4 |
| 189 | 983 | Q5RC42\|H2AZ\_PONAB | 52.83 | 7 | 0 | 7 | 0 | 0 | 0 | 0 | 0 | 0 | 0 | 0 |  | 6.76E4 |  |  |  |  |  |  |  |  | 1 | 1 | 0 | 1 | 0 | 0 | 0 | 0 | 0 | 0 | 0 | 0 | N | 13553 | Histone H2A.Z OS=Pongo abelii OX=9601 GN=H2AFZ PE=2 SV=3 |
| 189 | 984 | P70094\|H2AZL\_XENLA | 52.83 | 7 | 0 | 7 | 0 | 0 | 0 | 0 | 0 | 0 | 0 | 0 |  | 6.76E4 |  |  |  |  |  |  |  |  | 1 | 1 | 0 | 1 | 0 | 0 | 0 | 0 | 0 | 0 | 0 | 0 | N | 13486 | Histone H2A.Z-like OS=Xenopus laevis OX=8355 GN=h2a.zl1 PE=2 SV=3 |
| 189 | 985 | P70082\|H2AJ\_CHICK | 52.83 | 7 | 0 | 7 | 0 | 0 | 0 | 0 | 0 | 0 | 0 | 0 |  | 6.76E4 |  |  |  |  |  |  |  |  | 1 | 1 | 0 | 1 | 0 | 0 | 0 | 0 | 0 | 0 | 0 | 0 | N | 14016 | Histone H2A.J OS=Gallus gallus OX=9031 GN=H2A-IX PE=3 SV=1 |
| 189 | 986 | P02263\|H2A4\_CHICK | 52.83 | 7 | 0 | 7 | 0 | 0 | 0 | 0 | 0 | 0 | 0 | 0 |  | 6.76E4 |  |  |  |  |  |  |  |  | 1 | 1 | 0 | 1 | 0 | 0 | 0 | 0 | 0 | 0 | 0 | 0 | N | 13940 | Histone H2A-IV OS=Gallus gallus OX=9031 PE=1 SV=2 |
| 189 | 987 | A1A4R1\|H2A2C\_BOVIN | 52.83 | 7 | 0 | 7 | 0 | 0 | 0 | 0 | 0 | 0 | 0 | 0 |  | 6.76E4 |  |  |  |  |  |  |  |  | 1 | 1 | 0 | 1 | 0 | 0 | 0 | 0 | 0 | 0 | 0 | 0 | N | 13988 | Histone H2A type 2-C OS=Bos taurus OX=9913 GN=HIST2H2AC PE=2 SV=1 |
| 189 | 988 | Q64523\|H2A2C\_MOUSE | 52.83 | 7 | 0 | 7 | 0 | 0 | 0 | 0 | 0 | 0 | 0 | 0 |  | 6.76E4 |  |  |  |  |  |  |  |  | 1 | 1 | 0 | 1 | 0 | 0 | 0 | 0 | 0 | 0 | 0 | 0 | N | 13988 | Histone H2A type 2-C OS=Mus musculus OX=10090 GN=Hist2h2ac PE=1 SV=3 |
| 189 | 989 | Q16777\|H2A2C\_HUMAN | 52.83 | 7 | 0 | 7 | 0 | 0 | 0 | 0 | 0 | 0 | 0 | 0 |  | 6.76E4 |  |  |  |  |  |  |  |  | 1 | 1 | 0 | 1 | 0 | 0 | 0 | 0 | 0 | 0 | 0 | 0 | N | 13988 | Histone H2A type 2-C OS=Homo sapiens OX=9606 GN=HIST2H2AC PE=1 SV=4 |
| 189 | 990 | Q8BFU2\|H2A3\_MOUSE | 52.83 | 7 | 0 | 7 | 0 | 0 | 0 | 0 | 0 | 0 | 0 | 0 |  | 6.76E4 |  |  |  |  |  |  |  |  | 1 | 1 | 0 | 1 | 0 | 0 | 0 | 0 | 0 | 0 | 0 | 0 | N | 14121 | Histone H2A type 3 OS=Mus musculus OX=10090 GN=Hist3h2a PE=1 SV=3 |
| 189 | 991 | P0CC09\|H2A2A\_RAT | 52.83 | 7 | 0 | 7 | 0 | 0 | 0 | 0 | 0 | 0 | 0 | 0 |  | 6.76E4 |  |  |  |  |  |  |  |  | 1 | 1 | 0 | 1 | 0 | 0 | 0 | 0 | 0 | 0 | 0 | 0 | N | 14095 | Histone H2A type 2-A OS=Rattus norvegicus OX=10116 GN=Hist2h2aa3 PE=1 SV=1 |
| 189 | 992 | P20671\|H2A1D\_HUMAN | 52.83 | 7 | 0 | 7 | 0 | 0 | 0 | 0 | 0 | 0 | 0 | 0 |  | 6.76E4 |  |  |  |  |  |  |  |  | 1 | 1 | 0 | 1 | 0 | 0 | 0 | 0 | 0 | 0 | 0 | 0 | N | 14107 | Histone H2A type 1-D OS=Homo sapiens OX=9606 GN=HIST1H2AD PE=1 SV=2 |
| 189 | 993 | Q64522\|H2A2B\_MOUSE | 52.83 | 7 | 0 | 7 | 0 | 0 | 0 | 0 | 0 | 0 | 0 | 0 |  | 6.76E4 |  |  |  |  |  |  |  |  | 1 | 1 | 0 | 1 | 0 | 0 | 0 | 0 | 0 | 0 | 0 | 0 | N | 14013 | Histone H2A type 2-B OS=Mus musculus OX=10090 GN=Hist2h2ab PE=1 SV=3 |
| 189 | 994 | Q9LHQ5\|H2A2\_ARATH | 52.83 | 7 | 0 | 7 | 0 | 0 | 0 | 0 | 0 | 0 | 0 | 0 |  | 6.76E4 |  |  |  |  |  |  |  |  | 1 | 1 | 0 | 1 | 0 | 0 | 0 | 0 | 0 | 0 | 0 | 0 | N | 13849 | Probable histone H2A.2 OS=Arabidopsis thaliana OX=3702 GN=At3g20670 PE=1 SV=1 |
| 189 | 995 | Q6CUC8\|H2AZ\_KLULA | 52.83 | 7 | 0 | 7 | 0 | 0 | 0 | 0 | 0 | 0 | 0 | 0 |  | 6.76E4 |  |  |  |  |  |  |  |  | 1 | 1 | 0 | 1 | 0 | 0 | 0 | 0 | 0 | 0 | 0 | 0 | N | 14130 | Histone H2A.Z OS=Kluyveromyces lactis (strain ATCC 8585 / CBS 2359 / DSM 70799 / NBRC 1267 / NRRL Y-1140 / WM37) OX=284590 GN=HTZ1 PE=3 SV=3 |
| 189 | 996 | A3GHC1\|H2AZ\_PICST | 52.83 | 7 | 0 | 7 | 0 | 0 | 0 | 0 | 0 | 0 | 0 | 0 |  | 6.76E4 |  |  |  |  |  |  |  |  | 1 | 1 | 0 | 1 | 0 | 0 | 0 | 0 | 0 | 0 | 0 | 0 | N | 14342 | Histone H2A.Z OS=Scheffersomyces stipitis (strain ATCC 58785 / CBS 6054 / NBRC 10063 / NRRL Y-11545) OX=322104 GN=HTZ1 PE=3 SV=2 |
| 189 | 997 | Q9C944\|H2AV3\_ARATH | 52.83 | 7 | 0 | 7 | 0 | 0 | 0 | 0 | 0 | 0 | 0 | 0 |  | 6.76E4 |  |  |  |  |  |  |  |  | 1 | 1 | 0 | 1 | 0 | 0 | 0 | 0 | 0 | 0 | 0 | 0 | N | 14272 | Probable histone H2A variant 3 OS=Arabidopsis thaliana OX=3702 GN=At1g52740 PE=1 SV=1 |
| 189 | 998 | Q43208\|H2A4\_WHEAT | 52.83 | 7 | 0 | 7 | 0 | 0 | 0 | 0 | 0 | 0 | 0 | 0 |  | 6.76E4 |  |  |  |  |  |  |  |  | 1 | 1 | 0 | 1 | 0 | 0 | 0 | 0 | 0 | 0 | 0 | 0 | N | 14102 | Histone H2A.4 OS=Triticum aestivum OX=4565 GN=TH254 PE=2 SV=3 |
| 189 | 999 | A2YMC6\|H2A2\_ORYSI | 52.83 | 7 | 0 | 7 | 0 | 0 | 0 | 0 | 0 | 0 | 0 | 0 |  | 6.76E4 |  |  |  |  |  |  |  |  | 1 | 1 | 0 | 1 | 0 | 0 | 0 | 0 | 0 | 0 | 0 | 0 | N | 13976 | Probable histone H2A.2 OS=Oryza sativa subsp. indica OX=39946 GN=OsI\_025469 PE=3 SV=1 |
| 189 | 1000 | Q4PEF9\|H2A\_USTMA | 52.83 | 7 | 0 | 7 | 0 | 0 | 0 | 0 | 0 | 0 | 0 | 0 |  | 6.76E4 |  |  |  |  |  |  |  |  | 1 | 1 | 0 | 1 | 0 | 0 | 0 | 0 | 0 | 0 | 0 | 0 | N | 14199 | Histone H2A OS=Ustilago maydis (strain 521 / FGSC 9021) OX=237631 GN=HTA1 PE=3 SV=3 |
| 189 | 1001 | Q84MP7\|H2AV3\_ORYSJ | 52.83 | 7 | 0 | 7 | 0 | 0 | 0 | 0 | 0 | 0 | 0 | 0 |  | 6.76E4 |  |  |  |  |  |  |  |  | 1 | 1 | 0 | 1 | 0 | 0 | 0 | 0 | 0 | 0 | 0 | 0 | N | 14459 | Probable histone H2A variant 3 OS=Oryza sativa subsp. japonica OX=39947 GN=Os03g0743400 PE=2 SV=1 |
| 189 | 1002 | Q8H7Y8\|H2AV1\_ORYSJ | 52.83 | 7 | 0 | 7 | 0 | 0 | 0 | 0 | 0 | 0 | 0 | 0 |  | 6.76E4 |  |  |  |  |  |  |  |  | 1 | 1 | 0 | 1 | 0 | 0 | 0 | 0 | 0 | 0 | 0 | 0 | N | 14556 | Probable histone H2A variant 1 OS=Oryza sativa subsp. japonica OX=39947 GN=Os03g0162200 PE=2 SV=1 |
| 189 | 1003 | O65759\|H2AX\_CICAR | 52.83 | 6 | 0 | 6 | 0 | 0 | 0 | 0 | 0 | 0 | 0 | 0 |  | 6.76E4 |  |  |  |  |  |  |  |  | 1 | 1 | 0 | 1 | 0 | 0 | 0 | 0 | 0 | 0 | 0 | 0 | N | 14609 | Histone H2AX OS=Cicer arietinum OX=3827 GN=HIS2A PE=2 SV=1 |
| 189 | 1004 | Q9S9K7\|H2AXB\_ARATH | 52.83 | 6 | 0 | 6 | 0 | 0 | 0 | 0 | 0 | 0 | 0 | 0 |  | 6.76E4 |  |  |  |  |  |  |  |  | 1 | 1 | 0 | 1 | 0 | 0 | 0 | 0 | 0 | 0 | 0 | 0 | N | 14783 | Probable histone H2AXb OS=Arabidopsis thaliana OX=3702 GN=At1g54690 PE=1 SV=1 |
| 189 | 1005 | P16104\|H2AX\_HUMAN | 52.83 | 6 | 0 | 6 | 0 | 0 | 0 | 0 | 0 | 0 | 0 | 0 |  | 6.76E4 |  |  |  |  |  |  |  |  | 1 | 1 | 0 | 1 | 0 | 0 | 0 | 0 | 0 | 0 | 0 | 0 | N | 15145 | Histone H2AX OS=Homo sapiens OX=9606 GN=H2AFX PE=1 SV=2 |
| 189 | 1006 | Q9FJE8\|H2A7\_ARATH | 52.83 | 6 | 0 | 6 | 0 | 0 | 0 | 0 | 0 | 0 | 0 | 0 |  | 6.76E4 |  |  |  |  |  |  |  |  | 1 | 1 | 0 | 1 | 0 | 0 | 0 | 0 | 0 | 0 | 0 | 0 | N | 15966 | Probable histone H2A.7 OS=Arabidopsis thaliana OX=3702 GN=At5g59870 PE=1 SV=1 |
| 189 | 1007 | P02277\|H2A3\_WHEAT | 52.83 | 6 | 0 | 6 | 0 | 0 | 0 | 0 | 0 | 0 | 0 | 0 |  | 6.76E4 |  |  |  |  |  |  |  |  | 1 | 1 | 0 | 1 | 0 | 0 | 0 | 0 | 0 | 0 | 0 | 0 | N | 16070 | Histone H2A.2.2 OS=Triticum aestivum OX=4565 PE=1 SV=1 |
| 125 | 975 | P58370\|3NO48\_MICCO | 51.22 | 15 | 0 | 0 | 0 | 15 | 0 | 0 | 15 | 0 | 0 | 0 |  |  |  | 0E0 |  |  | 1.6E5 |  |  |  | 1 | 1 | 0 | 0 | 0 | 1 | 0 | 0 | 1 | 0 | 0 | 0 | Y | 9591 | Alpha-neurotoxin homolog 8 OS=Micrurus corallinus OX=54390 GN=NXH8 PE=1 SV=1 |
| 194 | 969 | P05109\|S10A8\_HUMAN | 50.11 | 12 | 0 | 0 | 12 | 0 | 0 | 0 | 0 | 0 | 0 | 0 |  |  | 1.47E4 |  |  |  |  |  |  |  | 1 | 1 | 0 | 0 | 1 | 0 | 0 | 0 | 0 | 0 | 0 | 0 | Y | 10835 | Protein S100-A8 OS=Homo sapiens OX=9606 GN=S100A8 PE=1 SV=1 |
| 208 | 1009 | Q802B2\|3NO2A\_NAJSP | 49.58 | 15 | 0 | 0 | 0 | 15 | 0 | 0 | 0 | 0 | 0 | 0 |  |  |  | 3.65E4 |  |  |  |  |  |  | 1 | 1 | 0 | 0 | 0 | 1 | 0 | 0 | 0 | 0 | 0 | 0 | Y | 9920 | Weak neurotoxin 10 OS=Naja sputatrix OX=33626 GN=WNTX10 PE=3 SV=1 |
| 175 | 660 | O97370\|EURM3\_EURMA | 48.02 | 5 | 0 | 0 | 0 | 0 | 0 | 0 | 0 | 0 | 5 | 0 |  |  |  |  |  |  |  |  | 3.97E4 |  | 1 | 1 | 0 | 0 | 0 | 0 | 0 | 0 | 0 | 0 | 1 | 0 | Y | 28038 | Mite allergen Eur m 3 OS=Euroglyphus maynei OX=6958 GN=EURM3 PE=1 SV=1 |
| 175 | 814 | P39675\|DERP3\_DERPT | 48.02 | 5 | 0 | 0 | 0 | 0 | 0 | 0 | 0 | 0 | 5 | 0 |  |  |  |  |  |  |  |  | 3.97E4 |  | 1 | 1 | 0 | 0 | 0 | 0 | 0 | 0 | 0 | 0 | 1 | 0 | Y | 28060 | Mite allergen Der p 3 OS=Dermatophagoides pteronyssinus OX=6956 GN=DERP3 PE=1 SV=1 |
| 175 | 848 | P49275\|DERF3\_DERFA | 48.02 | 5 | 0 | 0 | 0 | 0 | 0 | 0 | 0 | 0 | 5 | 0 |  |  |  |  |  |  |  |  | 3.97E4 |  | 1 | 1 | 0 | 0 | 0 | 0 | 0 | 0 | 0 | 0 | 1 | 0 | Y | 27674 | Mite allergen Der f 3 OS=Dermatophagoides farinae OX=6954 GN=DERF3 PE=1 SV=2 |
| 184 | 760 | Q1LPI5\|ENO1\_CUPMC | 47.18 | 3 | 0 | 3 | 0 | 0 | 0 | 0 | 0 | 0 | 0 | 0 |  | 2E4 |  |  |  |  |  |  |  |  | 1 | 1 | 0 | 1 | 0 | 0 | 0 | 0 | 0 | 0 | 0 | 0 | N | 45938 | Enolase 1 OS=Cupriavidus metallidurans (strain ATCC 43123 / DSM 2839 / NBRC 102507 / CH34) OX=266264 GN=eno1 PE=3 SV=1 |
| 184 | 1025 | Q47WR1\|ENO\_COLP3 | 47.18 | 3 | 0 | 3 | 0 | 0 | 0 | 0 | 0 | 0 | 0 | 0 |  | 2E4 |  |  |  |  |  |  |  |  | 1 | 1 | 0 | 1 | 0 | 0 | 0 | 0 | 0 | 0 | 0 | 0 | N | 45935 | Enolase OS=Colwellia psychrerythraea (strain 34H / ATCC BAA-681) OX=167879 GN=eno PE=3 SV=1 |
| 140 | 697 | P52552\|PRDX2\_PIG | 43.71 | 9 | 0 | 0 | 9 | 0 | 9 | 0 | 0 | 0 | 0 | 0 |  |  | 6.21E3 |  | 7.88E3 |  |  |  |  |  | 1 | 1 | 0 | 0 | 1 | 0 | 1 | 0 | 0 | 0 | 0 | 0 | N | 14198 | Peroxiredoxin-2 (Fragment) OS=Sus scrofa OX=9823 GN=PRDX2 PE=2 SV=1 |
| 140 | 794 | Q8K3U7\|PRDX2\_CRIGR | 43.71 | 6 | 0 | 0 | 6 | 0 | 6 | 0 | 0 | 0 | 0 | 0 |  |  | 6.21E3 |  | 7.88E3 |  |  |  |  |  | 1 | 1 | 0 | 0 | 1 | 0 | 1 | 0 | 0 | 0 | 0 | 0 | N | 21813 | Peroxiredoxin-2 OS=Cricetulus griseus OX=10029 GN=PRDX2 PE=2 SV=3 |
| 140 | 844 | Q06830\|PRDX1\_HUMAN | 43.71 | 6 | 0 | 0 | 6 | 0 | 6 | 0 | 0 | 0 | 0 | 0 |  |  | 6.21E3 |  | 7.88E3 |  |  |  |  |  | 1 | 1 | 0 | 0 | 1 | 0 | 1 | 0 | 0 | 0 | 0 | 0 | N | 22110 | Peroxiredoxin-1 OS=Homo sapiens OX=9606 GN=PRDX1 PE=1 SV=1 |
| 140 | 845 | #CONTAM#PRDX1\_HUMAN\| | 43.71 | 6 | 0 | 0 | 6 | 0 | 6 | 0 | 0 | 0 | 0 | 0 |  |  | 6.21E3 |  | 7.88E3 |  |  |  |  |  | 1 | 1 | 0 | 0 | 1 | 0 | 1 | 0 | 0 | 0 | 0 | 0 | N | 22110 | sp\|#CONTAM#PRDX1\_HUMAN\| |
| 140 | 846 | Q6B4U9\|PRDX1\_MYOLU | 43.71 | 6 | 0 | 0 | 6 | 0 | 6 | 0 | 0 | 0 | 0 | 0 |  |  | 6.21E3 |  | 7.88E3 |  |  |  |  |  | 1 | 1 | 0 | 0 | 1 | 0 | 1 | 0 | 0 | 0 | 0 | 0 | N | 22124 | Peroxiredoxin-1 OS=Myotis lucifugus OX=59463 GN=PRDX1 PE=1 SV=1 |
| 140 | 1015 | Q5RC63\|PRDX2\_PONAB | 43.71 | 6 | 0 | 0 | 6 | 0 | 6 | 0 | 0 | 0 | 0 | 0 |  |  | 6.21E3 |  | 7.88E3 |  |  |  |  |  | 1 | 1 | 0 | 0 | 1 | 0 | 1 | 0 | 0 | 0 | 0 | 0 | N | 19430 | Peroxiredoxin-2 OS=Pongo abelii OX=9601 GN=PRDX2 PE=2 SV=3 |
| 140 | 1016 | Q9V3P0\|PRDX1\_DROME | 43.71 | 6 | 0 | 0 | 6 | 0 | 6 | 0 | 0 | 0 | 0 | 0 |  |  | 6.21E3 |  | 7.88E3 |  |  |  |  |  | 1 | 1 | 0 | 0 | 1 | 0 | 1 | 0 | 0 | 0 | 0 | 0 | N | 21738 | Peroxiredoxin 1 OS=Drosophila melanogaster OX=7227 GN=Jafrac1 PE=1 SV=1 |
| 140 | 1017 | Q9NL98\|PRDX\_ASCSU | 43.71 | 6 | 0 | 0 | 6 | 0 | 6 | 0 | 0 | 0 | 0 | 0 |  |  | 6.21E3 |  | 7.88E3 |  |  |  |  |  | 1 | 1 | 0 | 0 | 1 | 0 | 1 | 0 | 0 | 0 | 0 | 0 | N | 21590 | Peroxiredoxin OS=Ascaris suum OX=6253 PE=2 SV=1 |
| 140 | 1018 | P35704\|PRDX2\_RAT | 43.71 | 6 | 0 | 0 | 6 | 0 | 6 | 0 | 0 | 0 | 0 | 0 |  |  | 6.21E3 |  | 7.88E3 |  |  |  |  |  | 1 | 1 | 0 | 0 | 1 | 0 | 1 | 0 | 0 | 0 | 0 | 0 | N | 21784 | Peroxiredoxin-2 OS=Rattus norvegicus OX=10116 GN=Prdx2 PE=1 SV=3 |
| 140 | 1019 | Q61171\|PRDX2\_MOUSE | 43.71 | 6 | 0 | 0 | 6 | 0 | 6 | 0 | 0 | 0 | 0 | 0 |  |  | 6.21E3 |  | 7.88E3 |  |  |  |  |  | 1 | 1 | 0 | 0 | 1 | 0 | 1 | 0 | 0 | 0 | 0 | 0 | N | 21779 | Peroxiredoxin-2 OS=Mus musculus OX=10090 GN=Prdx2 PE=1 SV=3 |
| 140 | 1020 | P32119\|PRDX2\_HUMAN | 43.71 | 6 | 0 | 0 | 6 | 0 | 6 | 0 | 0 | 0 | 0 | 0 |  |  | 6.21E3 |  | 7.88E3 |  |  |  |  |  | 1 | 1 | 0 | 0 | 1 | 0 | 1 | 0 | 0 | 0 | 0 | 0 | N | 21892 | Peroxiredoxin-2 OS=Homo sapiens OX=9606 GN=PRDX2 PE=1 SV=5 |
| 140 | 1021 | Q2PFZ3\|PRDX2\_MACFA | 43.71 | 6 | 0 | 0 | 6 | 0 | 6 | 0 | 0 | 0 | 0 | 0 |  |  | 6.21E3 |  | 7.88E3 |  |  |  |  |  | 1 | 1 | 0 | 0 | 1 | 0 | 1 | 0 | 0 | 0 | 0 | 0 | N | 21892 | Peroxiredoxin-2 OS=Macaca fascicularis OX=9541 GN=PRDX2 PE=2 SV=3 |
| 139 | 558 | P31025\|LCN1\_HUMAN | 42.15 | 6 | 0 | 0 | 0 | 0 | 0 | 0 | 0 | 0 | 6 | 0 |  |  |  |  |  |  |  |  | 1.97E4 |  | 1 | 1 | 0 | 0 | 0 | 0 | 0 | 0 | 0 | 0 | 1 | 0 | N | 19250 | Lipocalin-1 OS=Homo sapiens OX=9606 GN=LCN1 PE=1 SV=1 |
| 164 | 557 | B4RYQ8\|EFTU\_ALTMD | 41.87 | 3 | 0 | 3 | 0 | 0 | 0 | 0 | 0 | 0 | 0 | 0 |  | 3.1E4 |  |  |  |  |  |  |  |  | 1 | 1 | 0 | 1 | 0 | 0 | 0 | 0 | 0 | 0 | 0 | 0 | N | 43224 | Elongation factor Tu OS=Alteromonas mediterranea (strain DSM 17117 / CIP 110805 / LMG 28347 / Deep ecotype) OX=1774373 GN=tuf PE=3 SV=1 |
| 164 | 648 | Q3J8Q0\|EFTU\_NITOC | 41.87 | 3 | 0 | 3 | 0 | 0 | 0 | 0 | 0 | 0 | 0 | 0 |  | 3.1E4 |  |  |  |  |  |  |  |  | 1 | 1 | 0 | 1 | 0 | 0 | 0 | 0 | 0 | 0 | 0 | 0 | N | 43674 | Elongation factor Tu OS=Nitrosococcus oceani (strain ATCC 19707 / BCRC 17464 / NCIMB 11848 / C-107) OX=323261 GN=tuf1 PE=3 SV=1 |
| 164 | 675 | P33167\|EFTU\_BURCE | 41.87 | 3 | 0 | 3 | 0 | 0 | 0 | 0 | 0 | 0 | 0 | 0 |  | 3.1E4 |  |  |  |  |  |  |  |  | 1 | 1 | 0 | 1 | 0 | 0 | 0 | 0 | 0 | 0 | 0 | 0 | N | 42876 | Elongation factor Tu OS=Burkholderia cepacia OX=292 GN=tuf PE=3 SV=1 |
| 164 | 733 | Q1H4N9\|EFTU2\_METFK | 41.87 | 3 | 0 | 3 | 0 | 0 | 0 | 0 | 0 | 0 | 0 | 0 |  | 3.1E4 |  |  |  |  |  |  |  |  | 1 | 1 | 0 | 1 | 0 | 0 | 0 | 0 | 0 | 0 | 0 | 0 | N | 42950 | Elongation factor Tu 2 OS=Methylobacillus flagellatus (strain KT / ATCC 51484 / DSM 6875) OX=265072 GN=tuf2 PE=3 SV=1 |
| 164 | 913 | A1KRF9\|EFTU\_NEIMF | 41.87 | 3 | 0 | 3 | 0 | 0 | 0 | 0 | 0 | 0 | 0 | 0 |  | 3.1E4 |  |  |  |  |  |  |  |  | 1 | 1 | 0 | 1 | 0 | 0 | 0 | 0 | 0 | 0 | 0 | 0 | N | 42909 | Elongation factor Tu OS=Neisseria meningitidis serogroup C / serotype 2a (strain ATCC 700532 / DSM 15464 / FAM18) OX=272831 GN=tuf1 PE=3 SV=1 |
| 164 | 930 | Q1H4Q1\|EFTU1\_METFK | 41.87 | 3 | 0 | 3 | 0 | 0 | 0 | 0 | 0 | 0 | 0 | 0 |  | 3.1E4 |  |  |  |  |  |  |  |  | 1 | 1 | 0 | 1 | 0 | 0 | 0 | 0 | 0 | 0 | 0 | 0 | N | 42922 | Elongation factor Tu 1 OS=Methylobacillus flagellatus (strain KT / ATCC 51484 / DSM 6875) OX=265072 GN=tuf1 PE=3 SV=1 |
| 190 | 654 | Q11HA6\|EFTU\_CHESB | 41.45 | 3 | 0 | 3 | 0 | 0 | 0 | 0 | 0 | 0 | 0 | 0 |  | 1.1E5 |  |  |  |  |  |  |  |  | 1 | 1 | 0 | 1 | 0 | 0 | 0 | 0 | 0 | 0 | 0 | 0 | N | 42794 | Elongation factor Tu OS=Chelativorans sp. (strain BNC1) OX=266779 GN=tuf1 PE=3 SV=1 |
| 190 | 671 | Q5QWA3\|EFTU\_IDILO | 41.45 | 3 | 0 | 3 | 0 | 0 | 0 | 0 | 0 | 0 | 0 | 0 |  | 1.1E5 |  |  |  |  |  |  |  |  | 1 | 1 | 0 | 1 | 0 | 0 | 0 | 0 | 0 | 0 | 0 | 0 | N | 43254 | Elongation factor Tu OS=Idiomarina loihiensis (strain ATCC BAA-735 / DSM 15497 / L2-TR) OX=283942 GN=tuf1 PE=3 SV=1 |
| 190 | 739 | Q2YM08\|EFTU\_BRUA2 | 41.45 | 3 | 0 | 3 | 0 | 0 | 0 | 0 | 0 | 0 | 0 | 0 |  | 1.1E5 |  |  |  |  |  |  |  |  | 1 | 1 | 0 | 1 | 0 | 0 | 0 | 0 | 0 | 0 | 0 | 0 | N | 42605 | Elongation factor Tu OS=Brucella abortus (strain 2308) OX=359391 GN=tuf1 PE=3 SV=1 |
| 190 | 740 | P64025\|EFTU\_BRUSU | 41.45 | 3 | 0 | 3 | 0 | 0 | 0 | 0 | 0 | 0 | 0 | 0 |  | 1.1E5 |  |  |  |  |  |  |  |  | 1 | 1 | 0 | 1 | 0 | 0 | 0 | 0 | 0 | 0 | 0 | 0 | N | 42605 | Elongation factor Tu OS=Brucella suis biovar 1 (strain 1330) OX=204722 GN=tufA PE=3 SV=1 |
| 190 | 741 | A9M5Q2\|EFTU\_BRUC2 | 41.45 | 3 | 0 | 3 | 0 | 0 | 0 | 0 | 0 | 0 | 0 | 0 |  | 1.1E5 |  |  |  |  |  |  |  |  | 1 | 1 | 0 | 1 | 0 | 0 | 0 | 0 | 0 | 0 | 0 | 0 | N | 42605 | Elongation factor Tu OS=Brucella canis (strain ATCC 23365 / NCTC 10854) OX=483179 GN=tuf1 PE=3 SV=1 |
| 190 | 742 | P64024\|EFTU\_BRUME | 41.45 | 3 | 0 | 3 | 0 | 0 | 0 | 0 | 0 | 0 | 0 | 0 |  | 1.1E5 |  |  |  |  |  |  |  |  | 1 | 1 | 0 | 1 | 0 | 0 | 0 | 0 | 0 | 0 | 0 | 0 | N | 42605 | Elongation factor Tu OS=Brucella melitensis biotype 1 (strain 16M / ATCC 23456 / NCTC 10094) OX=224914 GN=tufA PE=3 SV=1 |
| 190 | 743 | B0CH34\|EFTU\_BRUSI | 41.45 | 3 | 0 | 3 | 0 | 0 | 0 | 0 | 0 | 0 | 0 | 0 |  | 1.1E5 |  |  |  |  |  |  |  |  | 1 | 1 | 0 | 1 | 0 | 0 | 0 | 0 | 0 | 0 | 0 | 0 | N | 42605 | Elongation factor Tu OS=Brucella suis (strain ATCC 23445 / NCTC 10510) OX=470137 GN=tuf1 PE=3 SV=1 |
| 190 | 744 | A5VR08\|EFTU\_BRUO2 | 41.45 | 3 | 0 | 3 | 0 | 0 | 0 | 0 | 0 | 0 | 0 | 0 |  | 1.1E5 |  |  |  |  |  |  |  |  | 1 | 1 | 0 | 1 | 0 | 0 | 0 | 0 | 0 | 0 | 0 | 0 | N | 42605 | Elongation factor Tu OS=Brucella ovis (strain ATCC 25840 / 63/290 / NCTC 10512) OX=444178 GN=tuf1 PE=3 SV=1 |
| 181 | 634 | A5DPU0\|MAK5\_PICGU | 41.05 | 2 | 0 | 0 | 0 | 0 | 0 | 2 | 0 | 0 | 0 | 0 |  |  |  |  |  | 4.89E5 |  |  |  |  | 1 | 1 | 0 | 0 | 0 | 0 | 0 | 1 | 0 | 0 | 0 | 0 | Y | 84501 | ATP-dependent RNA helicase MAK5 OS=Meyerozyma guilliermondii (strain ATCC 6260 / CBS 566 / DSM 6381 / JCM 1539 / NBRC 10279 / NRRL Y-324) OX=294746 GN=MAK5 PE=3 SV=1 |
| 157 | 535 | B8V7Q1\|PCDL\_ACRMI | 40.25 | 0 | 0 | 0 | 0 | 0 | 0 | 0 | 0 | 0 | 0 | 0 |  | 3.36E5 |  |  |  |  |  |  |  |  | 1 | 1 | 0 | 1 | 0 | 0 | 0 | 0 | 0 | 0 | 0 | 0 | N | 488593 | Protocadherin-like protein OS=Acropora millepora OX=45264 PE=1 SV=1 |
| total 224 proteins |  |  |  |  |  |  |  |  |  |  |  |  |  |  |  |  |  |  |  |  |  |  |  |  |  |  |  |  |  |  |  |  |  |  |  |  |  |  |  |
| --- | --- | --- | --- | --- | --- | --- | --- | --- | --- | --- | --- | --- | --- | --- | --- | --- | --- | --- | --- | --- | --- | --- | --- | --- | --- | --- | --- | --- | --- | --- | --- | --- | --- | --- | --- | --- | --- | --- | --- |

  

P15445|PA2A2\_NAJNA

back to list

  

| Protein Coverage
| Supporting Peptides
|

Protein Coverage:

Supporting Peptides:

| Peptide | Uniq | -10lgP | Mass | Length | ppm | m/z | z | RT | Fraction | Scan | Source File | Area F1 | Area F2 | Area F3 | Area F4 | Area F5 | Area F6 | Area F7 | Area F8 | Area F9 | Area F10 | #Feature | #Feature F1 | #Feature F2 | #Feature F3 | #Feature F4 | #Feature F5 | #Feature F6 | #Feature F7 | #Feature F8 | #Feature F9 | #Feature F10 | Start | End | PTM |
| --- | --- | --- | --- | --- | --- | --- | --- | --- | --- | --- | --- | --- | --- | --- | --- | --- | --- | --- | --- | --- | --- | --- | --- | --- | --- | --- | --- | --- | --- | --- | --- | --- | --- | --- | --- |
| K.TYSYEC(+57.02)SQGTLTC(+57.02)KGDNNAC(+57.02)AASVC(+57.02)DC(+57.02)DR.L | Y | 131.02 | 3362.3003 | 29 | -1.4 | 1121.7725 | 3 | 11.39 | 6 | F6:2204 | 05112019\_RID\_1313\_NaNaRj\_F6.raw |  |  |  |  |  | 4.53E6 | 0 |  |  |  | 2 | 0 | 0 | 0 | 0 | 0 | 1 | 1 | 0 | 0 | 0 | 66 | 94 | Carbamidomethylation |
| R.LAAIC(+57.02)FAGAPYNDNNYNIDLK.A | N | 118.73 | 2356.1157 | 21 | -4.1 | 1179.0603 | 2 | 26.06 | 6 | F6:11508 | 05112019\_RID\_1313\_NaNaRj\_F6.raw |  |  |  |  |  | 1.08E9 | 4.03E7 | 4.24E8 | 2.97E5 |  | 40 | 0 | 0 | 0 | 0 | 0 | 21 | 6 | 11 | 2 | 0 | 95 | 115 | Carbamidomethylation |
| K.TYSYEC(+57.02)SQGTLTC(+57.02)K.G | N | 113.63 | 1696.7073 | 14 | 0.8 | 849.3616 | 2 | 11.36 | 7 | F7:2195 | 05112019\_RID\_1313\_NaNaRj\_F7.raw |  |  |  |  | 5.87E4 | 1.81E8 | 9.34E6 | 6.34E5 | 5.1E4 | 1.71E4 | 13 | 0 | 0 | 0 | 0 | 1 | 8 | 1 | 1 | 1 | 1 | 66 | 79 | Carbamidomethylation |
| R.LAAIC(+57.02)FAGAPYNDNNYNIDLKAR.C | N | 111.16 | 2583.2539 | 23 | 1.3 | 862.0930 | 3 | 18.04 | 6 | F6:6804 | 05112019\_RID\_1313\_NaNaRj\_F6.raw |  |  |  |  |  | 4.1E6 |  |  |  |  | 2 | 0 | 0 | 0 | 0 | 0 | 2 | 0 | 0 | 0 | 0 | 95 | 117 | Carbamidomethylation |
| K.ISGC(+57.02)WPYFKTYSYEC(+57.02)SQGTLTC(+57.02)KGDNNAC(+57.02)AASVC(+57.02)DC(+57.02)DR.L | Y | 102.58 | 4500.8271 | 38 | 0.2 | 1126.2142 | 4 | 12.52 | 6 | F6:3143 | 05112019\_RID\_1313\_NaNaRj\_F6.raw |  |  |  |  |  | 1.5E6 |  |  |  |  | 2 | 0 | 0 | 0 | 0 | 0 | 2 | 0 | 0 | 0 | 0 | 57 | 94 | Carbamidomethylation |
| F.AGAPYNDNNYNIDLK.A | N | 98.83 | 1680.7743 | 15 | 0.2 | 841.3946 | 2 | 11.89 | 6 | F6:2650 | 05112019\_RID\_1313\_NaNaRj\_F6.raw |  |  |  |  |  | 1.53E8 | 4.99E6 | 2.52E7 | 8.1E4 |  | 8 | 0 | 0 | 0 | 0 | 0 | 4 | 1 | 2 | 1 | 0 | 101 | 115 |  |
| R.SWWDFADYGC(+57.02)YC(+57.02)GR.G | N | 94.25 | 1841.6926 | 14 | 0.3 | 921.8539 | 2 | 36.59 | 6 | F6:15878 | 05112019\_RID\_1313\_NaNaRj\_F6.raw |  |  |  |  |  | 6.43E8 | 9.48E6 | 4.05E6 |  |  | 50 | 0 | 0 | 0 | 0 | 0 | 41 | 6 | 3 | 0 | 0 | 17 | 30 | Carbamidomethylation |
| R.C(+57.02)C(+57.02)QVHDNC(+57.02)YNEAEK.I | N | 91.62 | 1825.6818 | 14 | -1.8 | 913.8466 | 2 | 10.68 | 6 | F6:1591 | 05112019\_RID\_1313\_NaNaRj\_F6.raw |  |  |  |  |  | 6.91E7 | 2.23E6 |  |  |  | 4 | 0 | 0 | 0 | 0 | 0 | 2 | 2 | 0 | 0 | 0 | 43 | 56 | Carbamidomethylation |
| K.GDNNAC(+57.02)AASVC(+57.02)DC(+57.02)DR.L | Y | 89.87 | 1683.6035 | 15 | -0.2 | 842.8088 | 2 | 10.87 | 6 | F6:1743 | 05112019\_RID\_1313\_NaNaRj\_F6.raw |  |  |  |  | 5.31E3 | 1.24E7 | 7.65E5 | 6.21E4 |  | 4.98E3 | 5 | 0 | 0 | 0 | 0 | 1 | 1 | 1 | 1 | 0 | 1 | 80 | 94 | Carbamidomethylation |
| T.YSYEC(+57.02)SQGTLTC(+57.02)K.G | N | 83.50 | 1595.6595 | 13 | -1.9 | 798.8356 | 2 | 11.31 | 8 | F8:2155 | 05112019\_RID\_1313\_NaNaRj\_F8.raw |  |  |  |  |  |  | 4.19E4 | 2.33E5 |  |  | 2 | 0 | 0 | 0 | 0 | 0 | 0 | 1 | 1 | 0 | 0 | 67 | 79 | Carbamidomethylation |
| K.C(+57.02)TVPSRSWWDFADYGC(+57.02)YC(+57.02)GR.G | N | 83.15 | 2542.0251 | 20 | 0.6 | 848.3495 | 3 | 24.10 | 6 | F6:10504 | 05112019\_RID\_1313\_NaNaRj\_F6.raw |  |  |  |  |  | 2.29E6 |  |  |  |  | 2 | 0 | 0 | 0 | 0 | 0 | 2 | 0 | 0 | 0 | 0 | 11 | 30 | Carbamidomethylation |
| Y.SYEC(+57.02)SQGTLTC(+57.02)K.G | N | 80.85 | 1432.5963 | 12 | -0.8 | 717.3049 | 2 | 10.99 | 8 | F8:1884 | 05112019\_RID\_1313\_NaNaRj\_F8.raw |  |  |  |  |  | 2.31E5 | 2.11E5 | 8.52E5 |  |  | 3 | 0 | 0 | 0 | 0 | 0 | 1 | 1 | 1 | 0 | 0 | 68 | 79 | Carbamidomethylation |
| R.GGSGTPVDDLDR.C | N | 79.15 | 1187.5417 | 12 | -1.8 | 594.7771 | 2 | 11.41 | 6 | F6:2201 | 05112019\_RID\_1313\_NaNaRj\_F6.raw |  |  |  |  |  | 5.21E7 |  |  | 4.03E4 |  | 4 | 0 | 0 | 0 | 0 | 0 | 3 | 0 | 0 | 1 | 0 | 31 | 42 |  |
| R.C(+57.02)C(+57.02)QVHDNC(+57.02)YNEAEKISGC(+57.02)WPYFK.T | N | 76.95 | 2964.2087 | 23 | -2.0 | 989.0749 | 3 | 12.41 | 6 | F6:3039 | 05112019\_RID\_1313\_NaNaRj\_F6.raw |  |  |  |  |  | 6.26E6 |  |  |  |  | 3 | 0 | 0 | 0 | 0 | 0 | 3 | 0 | 0 | 0 | 0 | 43 | 65 | Carbamidomethylation |
| K.ISGC(+57.02)WPYFKTYSYEC(+57.02)SQGTLTC(+57.02)K.G | N | 75.72 | 2835.2341 | 23 | 0.4 | 946.0857 | 3 | 15.40 | 6 | F6:5076 | 05112019\_RID\_1313\_NaNaRj\_F6.raw |  |  |  |  |  | 8.77E5 |  |  |  |  | 1 | 0 | 0 | 0 | 0 | 0 | 1 | 0 | 0 | 0 | 0 | 57 | 79 | Carbamidomethylation |
| K.ISGC(+57.02)WPYFK.T | N | 74.23 | 1156.5375 | 9 | 1.7 | 579.2770 | 2 | 16.27 | 6 | F6:5756 | 05112019\_RID\_1313\_NaNaRj\_F6.raw |  |  |  |  |  | 3.02E8 | 8.06E6 | 1.78E6 |  |  | 12 | 0 | 0 | 0 | 0 | 0 | 8 | 3 | 1 | 0 | 0 | 57 | 65 | Carbamidomethylation |
| C.FAGAPYNDNNYNIDLK.A | N | 71.41 | 1827.8428 | 16 | -0.1 | 914.9286 | 2 | 13.07 | 7 | F7:3413 | 05112019\_RID\_1313\_NaNaRj\_F7.raw |  |  |  |  |  | 2.97E7 | 1.36E6 | 8.91E6 |  |  | 7 | 0 | 0 | 0 | 0 | 0 | 3 | 1 | 3 | 0 | 0 | 100 | 115 |  |
| K.TYSYEC(+57.02)SQGTLTC(+57.02)KG.D | N | 70.12 | 1753.7288 | 15 | -0.1 | 877.8716 | 2 | 11.39 | 6 | F6:2169 | 05112019\_RID\_1313\_NaNaRj\_F6.raw |  |  |  |  |  | 5.33E7 | 4.5E6 |  |  |  | 2 | 0 | 0 | 0 | 0 | 0 | 1 | 1 | 0 | 0 | 0 | 66 | 80 | Carbamidomethylation |
| Y.NDNNYNIDLK.A | N | 66.87 | 1221.5625 | 10 | 0.4 | 611.7888 | 2 | 11.62 | 8 | F8:2428 | 05112019\_RID\_1313\_NaNaRj\_F8.raw |  |  |  |  |  |  | 1.28E5 | 3.26E5 |  |  | 2 | 0 | 0 | 0 | 0 | 0 | 0 | 1 | 1 | 0 | 0 | 106 | 115 |  |
| G.DNNAC(+57.02)AASVC(+57.02)DC(+57.02)DR.L | Y | 63.02 | 1626.5820 | 14 | 0.7 | 814.2988 | 2 | 10.86 | 7 | F7:1762 | 05112019\_RID\_1313\_NaNaRj\_F7.raw |  |  |  |  |  |  | 3.6E4 |  |  |  | 1 | 0 | 0 | 0 | 0 | 0 | 0 | 1 | 0 | 0 | 0 | 81 | 94 | Carbamidomethylation |
| K.GDNNAC(+57.02)AASVC(+57.02)DC(+57.02)DRLAAIC(+57.02)FAGAPYNDNNYNIDLK.A | Y | 62.40 | 4021.7087 | 36 | 0.7 | 1341.5778 | 3 | 24.10 | 6 | F6:10251 | 05112019\_RID\_1313\_NaNaRj\_F6.raw |  |  |  |  |  | 1.17E7 |  |  |  |  | 1 | 0 | 0 | 0 | 0 | 0 | 1 | 0 | 0 | 0 | 0 | 80 | 115 | Carbamidomethylation |
| S.YEC(+57.02)SQGTLTC(+57.02)K.G | N | 61.39 | 1345.5642 | 11 | 0.4 | 673.7897 | 2 | 10.92 | 8 | F8:1818 | 05112019\_RID\_1313\_NaNaRj\_F8.raw |  |  |  |  |  |  |  | 2.81E4 |  |  | 1 | 0 | 0 | 0 | 0 | 0 | 0 | 0 | 1 | 0 | 0 | 69 | 79 | Carbamidomethylation |
| D.RLAAIC(+57.02)FAGAPYNDNNYNIDLK.A | N | 59.64 | 2512.2168 | 22 | 1.3 | 838.4139 | 3 | 17.67 | 6 | F6:6659 | 05112019\_RID\_1313\_NaNaRj\_F6.raw |  |  |  |  |  | 3.33E5 |  | 1.05E5 |  |  | 2 | 0 | 0 | 0 | 0 | 0 | 1 | 0 | 1 | 0 | 0 | 94 | 115 | Carbamidomethylation |
| I.C(+57.02)FAGAPYNDNNYNIDLK.A | N | 59.42 | 1987.8734 | 17 | 2.0 | 994.9459 | 2 | 13.68 | 6 | F6:3937 | 05112019\_RID\_1313\_NaNaRj\_F6.raw |  |  |  |  |  | 3.52E4 |  | 0 |  |  | 2 | 0 | 0 | 0 | 0 | 0 | 1 | 0 | 1 | 0 | 0 | 99 | 115 | Carbamidomethylation |
| R.GGSGTPVDDLDRC(+57.02)C(+57.02)QVHDNC(+57.02)YNEAEKISGC(+57.02)WPYFK.T | N | 54.42 | 4133.7402 | 35 | 3.1 | 1034.4456 | 4 | 13.87 | 6 | F6:3950 | 05112019\_RID\_1313\_NaNaRj\_F6.raw |  |  |  |  |  | 1.69E7 |  |  |  |  | 2 | 0 | 0 | 0 | 0 | 0 | 2 | 0 | 0 | 0 | 0 | 31 | 65 | Carbamidomethylation |
| R.LAAIC(+57.02)FAGAPYN.D | N | 53.65 | 1266.6067 | 12 | 0.3 | 634.3108 | 2 | 25.26 | 7 | F7:7409 | 05112019\_RID\_1313\_NaNaRj\_F7.raw |  |  |  |  |  | 8.75E5 | 2.92E6 |  |  |  | 2 | 0 | 0 | 0 | 0 | 0 | 1 | 1 | 0 | 0 | 0 | 95 | 106 | Carbamidomethylation |
| R.SWWDFADYGC(+57.02)YC(+57.02)GRG.G | N | 52.12 | 1898.7141 | 15 | 0.2 | 950.3645 | 2 | 34.96 | 6 | F6:15352 | 05112019\_RID\_1313\_NaNaRj\_F6.raw |  |  |  |  |  | 1.17E7 |  |  |  |  | 2 | 0 | 0 | 0 | 0 | 0 | 2 | 0 | 0 | 0 | 0 | 17 | 31 | Carbamidomethylation |
| R.LAAIC(+57.02)FAGAPY.N | N | 51.44 | 1152.5637 | 11 | 0.2 | 577.2892 | 2 | 30.44 | 8 | F8:11368 | 05112019\_RID\_1313\_NaNaRj\_F8.raw |  |  |  |  |  |  |  | 1.27E6 |  |  | 1 | 0 | 0 | 0 | 0 | 0 | 0 | 0 | 1 | 0 | 0 | 95 | 105 | Carbamidomethylation |
| L.AAIC(+57.02)FAGAPYNDNNYNIDLK.A | N | 50.37 | 2243.0317 | 20 | 0.0 | 748.6845 | 3 | 20.19 | 6 | F6:8229 | 05112019\_RID\_1313\_NaNaRj\_F6.raw |  |  |  |  |  | 9E4 |  | 4.38E5 |  |  | 2 | 0 | 0 | 0 | 0 | 0 | 1 | 0 | 1 | 0 | 0 | 96 | 115 | Carbamidomethylation |
| W.DFADYGC(+57.02)YC(+57.02)GR.G | N | 48.89 | 1382.5020 | 11 | 0.2 | 692.2584 | 2 | 11.94 | 6 | F6:2719 | 05112019\_RID\_1313\_NaNaRj\_F6.raw |  |  |  |  |  | 8.65E5 |  |  |  |  | 1 | 0 | 0 | 0 | 0 | 0 | 1 | 0 | 0 | 0 | 0 | 20 | 30 | Carbamidomethylation |
| K.C(+57.02)TVPSRSWWDFADYGC(+57.02)YC(+57.02)GRGGSGTPVDDLDR.C | N | 46.56 | 3711.5564 | 32 | 1.0 | 928.8973 | 4 | 22.98 | 6 | F6:9677 | 05112019\_RID\_1313\_NaNaRj\_F6.raw |  |  |  |  |  | 2.09E5 |  |  |  |  | 1 | 0 | 0 | 0 | 0 | 0 | 1 | 0 | 0 | 0 | 0 | 11 | 42 | Carbamidomethylation |
| R.LAAIC(+57.02)FAGAPYNDNNYNIDLKARC(+57.02)Q | N | 45.32 | 2871.3433 | 25 | 1.2 | 958.1228 | 3 | 17.18 | 6 | F6:6176 | 05112019\_RID\_1313\_NaNaRj\_F6.raw |  |  |  |  |  | 9.01E5 |  |  |  |  | 1 | 0 | 0 | 0 | 0 | 0 | 1 | 0 | 0 | 0 | 0 | 95 | 119 | Carbamidomethylation |
| R.SWWDFADYGC(+57.02)Y.C | N | 44.98 | 1468.5394 | 11 | 1.1 | 735.2778 | 2 | 57.54 | 6 | F6:22138 | 05112019\_RID\_1313\_NaNaRj\_F6.raw |  |  |  |  |  | 1.17E7 |  |  |  |  | 1 | 0 | 0 | 0 | 0 | 0 | 1 | 0 | 0 | 0 | 0 | 17 | 27 | Carbamidomethylation |
| R.LAAIC(+57.02)FAGAPYND.N | N | 43.96 | 1381.6335 | 13 | 0.2 | 691.8242 | 2 | 26.17 | 8 | F8:9252 | 05112019\_RID\_1313\_NaNaRj\_F8.raw |  |  |  |  |  | 0 |  | 4.07E4 |  |  | 2 | 0 | 0 | 0 | 0 | 0 | 1 | 0 | 1 | 0 | 0 | 95 | 107 | Carbamidomethylation |
| K.TYSYEC(+57.02)SQGTLTC(+57.02)KGDNNAC(+57.02)AASVC(+57.02)DC(+57.02)DRLAAIC(+57.02)FAGAPYNDNNYNIDLK.A | Y | 43.35 | 5700.4053 | 50 | 1.1 | 1426.1101 | 4 | 20.47 | 6 | F6:8505 | 05112019\_RID\_1313\_NaNaRj\_F6.raw |  |  |  |  |  | 2.41E7 |  |  |  |  | 1 | 0 | 0 | 0 | 0 | 0 | 1 | 0 | 0 | 0 | 0 | 66 | 115 | Carbamidomethylation |
| A.IC(+57.02)FAGAPYNDNNYNIDLK.A | N | 40.45 | 2100.9575 | 18 | 0.3 | 1051.4863 | 2 | 18.53 | 8 | F8:6170 | 05112019\_RID\_1313\_NaNaRj\_F8.raw |  |  |  |  |  |  |  | 2.26E6 |  |  | 1 | 0 | 0 | 0 | 0 | 0 | 0 | 0 | 1 | 0 | 0 | 98 | 115 | Carbamidomethylation |
| total 36 peptides |  |  |  |  |  |  |  |  |  |  |  |  |  |  |  |  |  |  |  |  |  |  |  |  |  |  |  |  |  |  |  |  |  |  |  |
| --- | --- | --- | --- | --- | --- | --- | --- | --- | --- | --- | --- | --- | --- | --- | --- | --- | --- | --- | --- | --- | --- | --- | --- | --- | --- | --- | --- | --- | --- | --- | --- | --- | --- | --- | --- |

P35527|K1C9\_HUMAN

back to list

  

| Protein Coverage
| Supporting Peptides
|

Protein Coverage:

Supporting Peptides:

| Peptide | Uniq | -10lgP | Mass | Length | ppm | m/z | z | RT | Fraction | Scan | Source File | Area F1 | Area F2 | Area F3 | Area F4 | Area F5 | Area F6 | Area F7 | Area F8 | Area F9 | Area F10 | #Feature | #Feature F1 | #Feature F2 | #Feature F3 | #Feature F4 | #Feature F5 | #Feature F6 | #Feature F7 | #Feature F8 | #Feature F9 | #Feature F10 | Start | End | PTM |
| --- | --- | --- | --- | --- | --- | --- | --- | --- | --- | --- | --- | --- | --- | --- | --- | --- | --- | --- | --- | --- | --- | --- | --- | --- | --- | --- | --- | --- | --- | --- | --- | --- | --- | --- | --- |
| K.VQALEEANNDLENK.I | Y | 113.29 | 1585.7583 | 14 | 1.5 | 793.8876 | 2 | 11.54 | 4 | F4:2364 | 05112019\_RID\_1313\_NaNaRj\_F4.raw | 1.42E5 | 1.12E7 | 1.8E5 | 0 | 7.37E5 |  | 1.21E5 | 9.23E4 | 1.11E5 | 1.21E5 | 13 | 1 | 4 | 1 | 1 | 2 | 0 | 1 | 1 | 1 | 1 | 171 | 184 |  |
| R.GGSGGSYGGGGSGGGYGGGSGSR.G | Y | 112.60 | 1790.7203 | 23 | 0.5 | 896.3679 | 2 | 12.46 | 2 | F2:2033 | 09012020\_NaNaRj\_F2.raw |  | 1.19E6 | 1.1E6 |  | 2.24E5 |  |  |  |  | 7.09E4 | 4 | 0 | 1 | 1 | 0 | 1 | 0 | 0 | 0 | 0 | 1 | 491 | 513 |  |
| K.EIETYHNLLEGGQEDFESSGAGK.I | Y | 107.87 | 2509.1245 | 23 | 1.5 | 837.3834 | 3 | 12.55 | 6 | F6:3190 | 05112019\_RID\_1313\_NaNaRj\_F6.raw |  | 3.71E5 |  | 5.5E5 |  | 3.71E5 | 2.78E5 | 2.89E5 | 1.22E5 |  | 9 | 0 | 2 | 0 | 2 | 0 | 1 | 1 | 2 | 1 | 0 | 450 | 472 |  |
| R.SGGGGGGGLGSGGSIR.S | Y | 99.07 | 1231.5905 | 16 | 0.7 | 616.8029 | 2 | 10.96 | 5 | F5:1815 | 05112019\_RID\_1313\_NaNaRj\_F5.raw |  | 0 |  |  | 3.12E5 |  |  |  | 6.84E4 | 6.06E4 | 4 | 0 | 1 | 0 | 0 | 1 | 0 | 0 | 0 | 1 | 1 | 14 | 29 |  |
| R.GGSGGSHGGGSGFGGESGGSYGGGEEASGSGGGYGGGSGK.S | Y | 96.68 | 3222.2742 | 40 | -0.3 | 1075.0984 | 3 | 11.03 | 3 | F3:1882 | 05112019\_RID\_1313\_NaNaRj\_F3.raw |  |  | 1.73E5 | 8.07E4 |  |  |  |  |  |  | 2 | 0 | 0 | 1 | 1 | 0 | 0 | 0 | 0 | 0 | 0 | 580 | 619 |  |
| Y.SYGGGSGGGFSASSLGGGFGGGSR.G | Y | 82.29 | 2021.8827 | 24 | 1.6 | 1011.9502 | 2 | 12.48 | 6 | F6:3134 | 05112019\_RID\_1313\_NaNaRj\_F6.raw |  |  |  | 1.94E5 |  | 7.44E4 | 9.41E4 |  | 3.63E4 |  | 4 | 0 | 0 | 0 | 1 | 0 | 1 | 1 | 0 | 1 | 0 | 72 | 95 |  |
| R.QGVDADINGLR.Q | Y | 78.90 | 1156.5836 | 11 | 0.5 | 579.2994 | 2 | 11.72 | 4 | F4:2538 | 05112019\_RID\_1313\_NaNaRj\_F4.raw | 0 | 9.09E6 | 7.17E5 | 5.04E4 | 2.45E5 |  | 6.06E4 |  | 2.98E4 | 8.23E4 | 9 | 1 | 2 | 1 | 1 | 1 | 0 | 1 | 0 | 1 | 1 | 251 | 261 |  |
| R.HGVQELEIELQSQLSK.K | Y | 77.69 | 1836.9581 | 16 | 0.2 | 613.3268 | 3 | 17.38 | 8 | F8:5648 | 05112019\_RID\_1313\_NaNaRj\_F8.raw |  |  |  | 3.61E5 |  | 1.2E5 | 2.13E5 | 3.71E5 | 5.69E4 |  | 6 | 0 | 0 | 0 | 1 | 0 | 1 | 1 | 2 | 1 | 0 | 375 | 390 |  |
| K.DQIVDLTVGNNK.T | Y | 72.89 | 1314.6780 | 12 | 0.7 | 658.3467 | 2 | 46.67 | 2 | F2:15219 | 09012020\_NaNaRj\_F2.raw | 3.47E4 | 5.1E5 | 7.42E3 | 6.58E4 | 1.8E5 |  | 4.19E4 |  | 3.69E4 | 2.11E4 | 8 | 1 | 1 | 1 | 1 | 1 | 0 | 1 | 0 | 1 | 1 | 213 | 224 |  |
| R.QEYEQLIAK.N | Y | 71.79 | 1120.5764 | 9 | -0.5 | 561.2952 | 2 | 11.61 | 3 | F3:2396 | 05112019\_RID\_1313\_NaNaRj\_F3.raw |  | 1.76E7 | 1.79E5 |  | 4.73E5 |  |  |  | 6.13E4 | 1.08E5 | 6 | 0 | 2 | 1 | 0 | 1 | 0 | 0 | 0 | 1 | 1 | 328 | 336 |  |
| Y.HNLLEGGQEDFESSGAGK.I | Y | 71.38 | 1873.8442 | 18 | -0.1 | 625.6219 | 3 | 35.80 | 2 | F2:10620 | 09012020\_NaNaRj\_F2.raw |  | 1.67E5 |  |  |  |  |  |  |  |  | 1 | 0 | 1 | 0 | 0 | 0 | 0 | 0 | 0 | 0 | 0 | 455 | 472 |  |
| K.NYSPYYNTIDDLKDQIVDLTVGNNK.T | Y | 66.14 | 2901.4031 | 25 | 0.3 | 968.1420 | 3 | 41.70 | 8 | F8:16019 | 05112019\_RID\_1313\_NaNaRj\_F8.raw |  |  |  |  |  | 1.86E4 |  | 3.82E5 |  |  | 2 | 0 | 0 | 0 | 0 | 0 | 1 | 0 | 1 | 0 | 0 | 200 | 224 |  |
| S.ASSLGGGFGGGSR.G | Y | 66.10 | 1108.5261 | 13 | -0.5 | 555.2701 | 2 | 23.93 | 2 | F2:5810 | 09012020\_NaNaRj\_F2.raw |  | 1.49E5 |  |  |  |  |  |  |  |  | 1 | 0 | 1 | 0 | 0 | 0 | 0 | 0 | 0 | 0 | 0 | 83 | 95 |  |
| R.QVLDNLTMEK.S | Y | 65.98 | 1189.6013 | 10 | 0.0 | 595.8079 | 2 | 41.95 | 2 | F2:13188 | 09012020\_NaNaRj\_F2.raw |  | 2.24E6 | 1.3E5 |  |  |  |  |  | 1.04E4 | 1.05E4 | 4 | 0 | 1 | 1 | 0 | 0 | 0 | 0 | 0 | 1 | 1 | 262 | 271 |  |
| R.QVLDNLTM(+15.99)EK.S | Y | 62.66 | 1205.5962 | 10 | -0.1 | 603.8053 | 2 | 11.61 | 3 | F3:2399 | 05112019\_RID\_1313\_NaNaRj\_F3.raw |  | 1.33E6 | 1.26E5 |  |  |  |  |  |  |  | 2 | 0 | 1 | 1 | 0 | 0 | 0 | 0 | 0 | 0 | 0 | 262 | 271 | Oxidation (M) |
| Y.YNTIDDLKDQIVDLTVGNNK.T | Y | 61.91 | 2277.1489 | 20 | 0.1 | 760.0570 | 3 | 30.57 | 7 | F7:8623 | 05112019\_RID\_1313\_NaNaRj\_F7.raw |  |  |  | 3.21E4 |  |  | 7.5E4 | 7.35E4 |  |  | 3 | 0 | 0 | 0 | 1 | 0 | 0 | 1 | 1 | 0 | 0 | 205 | 224 |  |
| K.TLLDIDNTR.M | Y | 61.89 | 1059.5560 | 9 | 0.2 | 530.7854 | 2 | 12.36 | 4 | F4:3093 | 05112019\_RID\_1313\_NaNaRj\_F4.raw | 1.22E5 | 2.77E7 | 6.18E5 | 2.57E5 | 9.09E5 |  | 2.38E5 | 9.1E4 | 1.14E5 | 1.16E5 | 11 | 1 | 3 | 1 | 1 | 1 | 0 | 1 | 1 | 1 | 1 | 225 | 233 |  |
| R.QEIEC(+57.02)QNQEYSLLLSIK.M | Y | 61.32 | 2094.0303 | 17 | 0.5 | 1048.0229 | 2 | 25.30 | 6 | F6:10843 | 05112019\_RID\_1313\_NaNaRj\_F6.raw |  |  |  | 4.75E5 |  | 7.15E5 | 2.35E4 | 5E5 |  |  | 7 | 0 | 0 | 0 | 2 | 0 | 1 | 1 | 3 | 0 | 0 | 428 | 444 | Carbamidomethylation |
| R.FSSSSGYGGGSSR.V | Y | 60.39 | 1234.5214 | 13 | 0.1 | 618.2680 | 2 | 13.07 | 2 | F2:2264 | 09012020\_NaNaRj\_F2.raw | 1.18E4 | 5.95E4 |  |  | 1.47E5 |  |  |  | 1.03E4 | 3.37E4 | 5 | 1 | 1 | 0 | 0 | 1 | 0 | 0 | 0 | 1 | 1 | 47 | 59 |  |
| R.IKFEM(+15.99)EQNLR.Q | Y | 60.18 | 1322.6653 | 10 | 0.1 | 441.8958 | 3 | 24.62 | 2 | F2:6231 | 09012020\_NaNaRj\_F2.raw |  | 8.72E5 |  |  |  |  |  |  |  |  | 2 | 0 | 2 | 0 | 0 | 0 | 0 | 0 | 0 | 0 | 0 | 241 | 250 | Oxidation (M) |
| D.VNVEINVAPGK.D | Y | 59.08 | 1138.6346 | 11 | -1.1 | 570.3240 | 2 | 38.01 | 2 | F2:11562 | 09012020\_NaNaRj\_F2.raw |  | 2.91E5 |  |  |  |  |  |  |  |  | 1 | 0 | 1 | 0 | 0 | 0 | 0 | 0 | 0 | 0 | 0 | 307 | 317 |  |
| K.FEMEQNLR.Q | Y | 59.05 | 1065.4913 | 8 | -0.3 | 533.7528 | 2 | 34.12 | 2 | F2:9927 | 09012020\_NaNaRj\_F2.raw |  | 1.86E6 |  |  |  |  |  |  |  |  | 1 | 0 | 1 | 0 | 0 | 0 | 0 | 0 | 0 | 0 | 0 | 243 | 250 |  |
| K.EIETYHNLLEGGQE.D | Y | 58.61 | 1630.7474 | 14 | 0.9 | 816.3817 | 2 | 50.32 | 2 | F2:16763 | 09012020\_NaNaRj\_F2.raw |  | 1.54E5 |  |  |  |  |  |  |  |  | 1 | 0 | 1 | 0 | 0 | 0 | 0 | 0 | 0 | 0 | 0 | 450 | 463 |  |
| M.IQEQISNLEAQITDVR.Q | Y | 57.69 | 1855.9639 | 16 | 0.5 | 928.9897 | 2 | 19.14 | 8 | F8:6493 | 05112019\_RID\_1313\_NaNaRj\_F8.raw |  |  |  | 4.33E4 |  |  |  | 5.23E4 |  |  | 3 | 0 | 0 | 0 | 1 | 0 | 0 | 0 | 2 | 0 | 0 | 412 | 427 |  |
| E.QISNLEAQITDVR.Q | Y | 57.48 | 1485.7787 | 13 | 0.8 | 743.8972 | 2 | 50.76 | 2 | F2:16898 | 09012020\_NaNaRj\_F2.raw |  | 3.79E5 |  |  |  |  |  |  |  |  | 1 | 0 | 1 | 0 | 0 | 0 | 0 | 0 | 0 | 0 | 0 | 415 | 427 |  |
| S.NLEAQITDVR.Q | Y | 56.60 | 1157.6040 | 10 | 1.1 | 579.8099 | 2 | 39.94 | 2 | F2:12370 | 09012020\_NaNaRj\_F2.raw |  | 4.14E6 |  |  |  |  |  |  |  |  | 1 | 0 | 1 | 0 | 0 | 0 | 0 | 0 | 0 | 0 | 0 | 418 | 427 |  |
| K.NYSPYYNTIDDLK.D | Y | 56.53 | 1604.7358 | 13 | 0.8 | 803.3759 | 2 | 13.30 | 8 | F8:3728 | 05112019\_RID\_1313\_NaNaRj\_F8.raw |  | 3.25E5 |  | 7.33E4 |  | 0 | 4.66E4 | 5.07E4 |  |  | 5 | 0 | 1 | 0 | 1 | 0 | 1 | 1 | 1 | 0 | 0 | 200 | 212 |  |
| K.IQDWYDKK.G | Y | 55.69 | 1094.5397 | 8 | 0.9 | 548.2776 | 2 | 21.29 | 2 | F2:4819 | 09012020\_NaNaRj\_F2.raw |  | 9.62E5 |  |  |  |  |  |  |  |  | 1 | 0 | 1 | 0 | 0 | 0 | 0 | 0 | 0 | 0 | 0 | 185 | 192 |  |
| K.SDLEMQYETL.Q | Y | 55.37 | 1227.5330 | 10 | 0.9 | 614.7743 | 2 | 65.32 | 2 | F2:21431 | 09012020\_NaNaRj\_F2.raw |  | 1.24E6 |  |  |  |  |  |  |  |  | 1 | 0 | 1 | 0 | 0 | 0 | 0 | 0 | 0 | 0 | 0 | 272 | 281 |  |
| L.LEGGQEDFESSGAGK.I | Y | 54.96 | 1509.6583 | 15 | 0.1 | 755.8365 | 2 | 24.09 | 2 | F2:5879 | 09012020\_NaNaRj\_F2.raw |  | 2.56E4 |  |  |  |  |  |  |  |  | 1 | 0 | 1 | 0 | 0 | 0 | 0 | 0 | 0 | 0 | 0 | 458 | 472 |  |
| K.STMQELNSR.L | Y | 54.32 | 1064.4921 | 9 | -0.3 | 533.2532 | 2 | 10.99 | 10 | F10:1730 | 05112019\_RID\_1313\_NaNaRj\_F10.raw |  | 3.15E5 | 6.63E3 |  | 2.15E4 |  |  |  |  | 3.46E4 | 4 | 0 | 1 | 1 | 0 | 1 | 0 | 0 | 0 | 0 | 1 | 155 | 163 |  |
| N.SGDVNVEINVAPGK.D | Y | 52.34 | 1397.7150 | 14 | 0.9 | 699.8654 | 2 | 40.32 | 2 | F2:12570 | 09012020\_NaNaRj\_F2.raw |  | 5.59E4 |  |  |  |  |  |  |  |  | 1 | 0 | 1 | 0 | 0 | 0 | 0 | 0 | 0 | 0 | 0 | 304 | 317 |  |
| K.IQDWYDK.K | Y | 52.17 | 966.4447 | 7 | 0.1 | 484.2297 | 2 | 11.62 | 5 | F5:2478 | 05112019\_RID\_1313\_NaNaRj\_F5.raw | 0 | 0 | 1.47E4 |  | 2.22E5 |  |  |  | 1.49E4 | 1.59E4 | 6 | 1 | 1 | 1 | 0 | 1 | 0 | 0 | 0 | 1 | 1 | 185 | 191 |  |
| G.NYGGGSGSGGGSGGGYGGGSGSR.G | Y | 52.16 | 1847.7418 | 23 | 1.0 | 924.8791 | 2 | 10.70 | 5 | F5:1590 | 05112019\_RID\_1313\_NaNaRj\_F5.raw |  |  |  |  | 1.93E5 |  |  |  |  |  | 1 | 0 | 0 | 0 | 0 | 1 | 0 | 0 | 0 | 0 | 0 | 557 | 579 |  |
| F.GGFGGGAGGGDGGILTANEK.S | Y | 51.89 | 1690.7910 | 20 | 0.2 | 846.4030 | 2 | 11.96 | 5 | F5:2714 | 05112019\_RID\_1313\_NaNaRj\_F5.raw |  |  |  |  | 1.09E5 |  |  |  |  | 9.5E3 | 2 | 0 | 0 | 0 | 0 | 1 | 0 | 0 | 0 | 0 | 1 | 135 | 154 |  |
| I.SNLEAQITDVR.Q | Y | 51.60 | 1244.6361 | 11 | 0.6 | 623.3257 | 2 | 39.91 | 2 | F2:12383 | 09012020\_NaNaRj\_F2.raw |  | 2.4E4 |  |  |  |  |  |  |  |  | 1 | 0 | 1 | 0 | 0 | 0 | 0 | 0 | 0 | 0 | 0 | 417 | 427 |  |
| R.M(+15.99)TLDDFR.I | Y | 50.72 | 912.4011 | 7 | 0.4 | 457.2080 | 2 | 32.20 | 2 | F2:9117 | 09012020\_NaNaRj\_F2.raw |  | 0 | 1.38E5 |  | 9.31E4 |  |  |  |  | 1.93E4 | 4 | 0 | 1 | 1 | 0 | 1 | 0 | 0 | 0 | 0 | 1 | 234 | 240 | Oxidation (M) |
| F.SASSLGGGFGGGSR.G | Y | 50.26 | 1195.5581 | 14 | -0.1 | 598.7863 | 2 | 25.35 | 2 | F2:6357 | 09012020\_NaNaRj\_F2.raw | 4.27E3 | 6.68E5 |  |  |  |  |  |  |  | 1.1E4 | 4 | 1 | 2 | 0 | 0 | 0 | 0 | 0 | 0 | 0 | 1 | 82 | 95 |  |
| K.FEM(+15.99)EQNLR.Q | Y | 49.22 | 1081.4862 | 8 | 0.4 | 541.7506 | 2 | 34.18 | 2 | F2:9938 | 09012020\_NaNaRj\_F2.raw |  | 1.35E5 |  |  | 2.75E4 |  |  |  |  |  | 2 | 0 | 1 | 0 | 0 | 1 | 0 | 0 | 0 | 0 | 0 | 243 | 250 | Oxidation (M) |
| R.MTLDDFR.I | Y | 48.46 | 896.4062 | 7 | -0.7 | 449.2101 | 2 | 45.21 | 2 | F2:14760 | 09012020\_NaNaRj\_F2.raw |  | 1.55E7 | 2.13E4 |  | 3.21E4 |  |  |  |  |  | 4 | 0 | 2 | 1 | 0 | 1 | 0 | 0 | 0 | 0 | 0 | 234 | 240 |  |
| R.LASYLDK.V | N | 47.75 | 808.4330 | 7 | 0.6 | 405.2241 | 2 | 11.34 | 5 | F5:2185 | 05112019\_RID\_1313\_NaNaRj\_F5.raw | 3.03E4 | 9.71E6 | 7.78E4 |  | 7.33E5 |  |  |  |  | 3.94E4 | 5 | 1 | 1 | 1 | 0 | 1 | 0 | 0 | 0 | 0 | 1 | 164 | 170 |  |
| R.YC(+57.02)GQLQMIQEQISNLEAQITDVR.Q | Y | 47.63 | 2736.3210 | 23 | 0.6 | 913.1149 | 3 | 59.63 | 6 | F6:22579 | 05112019\_RID\_1313\_NaNaRj\_F6.raw |  |  |  |  |  | 1.55E4 |  |  |  |  | 1 | 0 | 0 | 0 | 0 | 0 | 1 | 0 | 0 | 0 | 0 | 405 | 427 | Carbamidomethylation |
| K.NYSPYYNTID.D | Y | 47.47 | 1248.5299 | 10 | 0.9 | 625.2728 | 2 | 51.66 | 2 | F2:17193 | 09012020\_NaNaRj\_F2.raw |  | 5.37E6 |  |  |  |  |  |  |  |  | 2 | 0 | 2 | 0 | 0 | 0 | 0 | 0 | 0 | 0 | 0 | 200 | 209 |  |
| R.QFSSSYLSR.S | Y | 47.39 | 1073.5142 | 9 | 0.3 | 537.7645 | 2 | 11.52 | 3 | F3:2314 | 05112019\_RID\_1313\_NaNaRj\_F3.raw |  | 2.64E6 | 5.66E4 |  |  |  |  |  |  |  | 2 | 0 | 1 | 1 | 0 | 0 | 0 | 0 | 0 | 0 | 0 | 5 | 13 |  |
| R.HGVQELEIELQ.S | Y | 46.46 | 1293.6565 | 11 | 0.3 | 647.8357 | 2 | 53.72 | 2 | F2:17923 | 09012020\_NaNaRj\_F2.raw |  | 2.95E6 |  |  |  |  |  |  |  |  | 1 | 0 | 1 | 0 | 0 | 0 | 0 | 0 | 0 | 0 | 0 | 375 | 385 |  |
| Q.ISNLEAQITDVR.Q | Y | 46.06 | 1357.7201 | 12 | 0.3 | 679.8676 | 2 | 12.26 | 5 | F5:3048 | 05112019\_RID\_1313\_NaNaRj\_F5.raw |  |  |  |  | 6.96E4 |  |  |  |  |  | 1 | 0 | 0 | 0 | 0 | 1 | 0 | 0 | 0 | 0 | 0 | 416 | 427 |  |
| S.SLGGGFGGGSR.G | Y | 46.00 | 950.4570 | 11 | 0.2 | 476.2358 | 2 | 22.25 | 2 | F2:5311 | 09012020\_NaNaRj\_F2.raw |  | 7.77E6 |  |  | 7.6E4 |  |  |  |  |  | 2 | 0 | 1 | 0 | 0 | 1 | 0 | 0 | 0 | 0 | 0 | 85 | 95 |  |
| S.YGGGSGGGFSASSLGGGFGGGSR.G | Y | 44.68 | 1934.8506 | 23 | 2.7 | 968.4352 | 2 | 49.89 | 2 | F2:16582 | 09012020\_NaNaRj\_F2.raw |  | 1.15E4 |  |  |  |  |  |  |  |  | 1 | 0 | 1 | 0 | 0 | 0 | 0 | 0 | 0 | 0 | 0 | 73 | 95 |  |
| R.YC(+57.02)GQLQM(+15.99)IQEQISNLEAQITDVR.Q | Y | 43.88 | 2752.3159 | 23 | 0.4 | 1377.1658 | 2 | 54.08 | 6 | F6:21189 | 05112019\_RID\_1313\_NaNaRj\_F6.raw |  |  |  |  |  | 1.35E4 |  |  |  |  | 1 | 0 | 0 | 0 | 0 | 0 | 1 | 0 | 0 | 0 | 0 | 405 | 427 | Carbamidomethylation; Oxidation (M) |
| K.STM(+15.99)QELNSR.L | Y | 41.78 | 1080.4869 | 9 | 0.2 | 541.2509 | 2 | 17.36 | 2 | F2:3314 | 09012020\_NaNaRj\_F2.raw |  | 8.58E4 |  |  |  |  |  |  |  |  | 1 | 0 | 1 | 0 | 0 | 0 | 0 | 0 | 0 | 0 | 0 | 155 | 163 | Oxidation (M) |
| K.SDLEM(+15.99)QYETLQEELM(+15.99)ALK.K | Y | 41.27 | 2202.0071 | 18 | 0.6 | 1102.0115 | 2 | 42.72 | 6 | F6:18144 | 05112019\_RID\_1313\_NaNaRj\_F6.raw |  |  |  |  |  | 6.65E4 |  |  |  |  | 1 | 0 | 0 | 0 | 0 | 0 | 1 | 0 | 0 | 0 | 0 | 272 | 289 | Oxidation (M) |
| Y.YNTIDDLK.D | Y | 40.06 | 980.4814 | 8 | 0.5 | 491.2482 | 2 | 11.54 | 5 | F5:2417 | 05112019\_RID\_1313\_NaNaRj\_F5.raw |  |  |  |  | 3.7E4 |  |  |  |  |  | 1 | 0 | 0 | 0 | 0 | 1 | 0 | 0 | 0 | 0 | 0 | 205 | 212 |  |
| total 52 peptides |  |  |  |  |  |  |  |  |  |  |  |  |  |  |  |  |  |  |  |  |  |  |  |  |  |  |  |  |  |  |  |  |  |  |  |
| --- | --- | --- | --- | --- | --- | --- | --- | --- | --- | --- | --- | --- | --- | --- | --- | --- | --- | --- | --- | --- | --- | --- | --- | --- | --- | --- | --- | --- | --- | --- | --- | --- | --- | --- | --- |

#CONTAM#K1C9\_HUMAN|

back to list

  

| Protein Coverage
| Supporting Peptides
|

Protein Coverage:

Supporting Peptides:

| Peptide | Uniq | -10lgP | Mass | Length | ppm | m/z | z | RT | Fraction | Scan | Source File | Area F1 | Area F2 | Area F3 | Area F4 | Area F5 | Area F6 | Area F7 | Area F8 | Area F9 | Area F10 | #Feature | #Feature F1 | #Feature F2 | #Feature F3 | #Feature F4 | #Feature F5 | #Feature F6 | #Feature F7 | #Feature F8 | #Feature F9 | #Feature F10 | Start | End | PTM |
| --- | --- | --- | --- | --- | --- | --- | --- | --- | --- | --- | --- | --- | --- | --- | --- | --- | --- | --- | --- | --- | --- | --- | --- | --- | --- | --- | --- | --- | --- | --- | --- | --- | --- | --- | --- |
| K.VQALEEANNDLENK.I | Y | 113.29 | 1585.7583 | 14 | 1.5 | 793.8876 | 2 | 11.54 | 4 | F4:2364 | 05112019\_RID\_1313\_NaNaRj\_F4.raw | 1.42E5 | 1.12E7 | 1.8E5 | 0 | 7.37E5 |  | 1.21E5 | 9.23E4 | 1.11E5 | 1.21E5 | 13 | 1 | 4 | 1 | 1 | 2 | 0 | 1 | 1 | 1 | 1 | 171 | 184 |  |
| R.GGSGGSYGGGGSGGGYGGGSGSR.G | Y | 112.60 | 1790.7203 | 23 | 0.5 | 896.3679 | 2 | 12.46 | 2 | F2:2033 | 09012020\_NaNaRj\_F2.raw |  | 1.19E6 | 1.1E6 |  | 2.24E5 |  |  |  |  | 7.09E4 | 4 | 0 | 1 | 1 | 0 | 1 | 0 | 0 | 0 | 0 | 1 | 491 | 513 |  |
| K.EIETYHNLLEGGQEDFESSGAGK.I | Y | 107.87 | 2509.1245 | 23 | 1.5 | 837.3834 | 3 | 12.55 | 6 | F6:3190 | 05112019\_RID\_1313\_NaNaRj\_F6.raw |  | 3.71E5 |  | 5.5E5 |  | 3.71E5 | 2.78E5 | 2.89E5 | 1.22E5 |  | 9 | 0 | 2 | 0 | 2 | 0 | 1 | 1 | 2 | 1 | 0 | 450 | 472 |  |
| R.SGGGGGGGLGSGGSIR.S | Y | 99.07 | 1231.5905 | 16 | 0.7 | 616.8029 | 2 | 10.96 | 5 | F5:1815 | 05112019\_RID\_1313\_NaNaRj\_F5.raw |  | 0 |  |  | 3.12E5 |  |  |  | 6.84E4 | 6.06E4 | 4 | 0 | 1 | 0 | 0 | 1 | 0 | 0 | 0 | 1 | 1 | 14 | 29 |  |
| R.GGSGGSHGGGSGFGGESGGSYGGGEEASGSGGGYGGGSGK.S | Y | 96.68 | 3222.2742 | 40 | -0.3 | 1075.0984 | 3 | 11.03 | 3 | F3:1882 | 05112019\_RID\_1313\_NaNaRj\_F3.raw |  |  | 1.73E5 | 8.07E4 |  |  |  |  |  |  | 2 | 0 | 0 | 1 | 1 | 0 | 0 | 0 | 0 | 0 | 0 | 580 | 619 |  |
| Y.SYGGGSGGGFSASSLGGGFGGGSR.G | Y | 82.29 | 2021.8827 | 24 | 1.6 | 1011.9502 | 2 | 12.48 | 6 | F6:3134 | 05112019\_RID\_1313\_NaNaRj\_F6.raw |  |  |  | 1.94E5 |  | 7.44E4 | 9.41E4 |  | 3.63E4 |  | 4 | 0 | 0 | 0 | 1 | 0 | 1 | 1 | 0 | 1 | 0 | 72 | 95 |  |
| R.QGVDADINGLR.Q | Y | 78.90 | 1156.5836 | 11 | 0.5 | 579.2994 | 2 | 11.72 | 4 | F4:2538 | 05112019\_RID\_1313\_NaNaRj\_F4.raw | 0 | 9.09E6 | 7.17E5 | 5.04E4 | 2.45E5 |  | 6.06E4 |  | 2.98E4 | 8.23E4 | 9 | 1 | 2 | 1 | 1 | 1 | 0 | 1 | 0 | 1 | 1 | 251 | 261 |  |
| R.HGVQELEIELQSQLSK.K | Y | 77.69 | 1836.9581 | 16 | 0.2 | 613.3268 | 3 | 17.38 | 8 | F8:5648 | 05112019\_RID\_1313\_NaNaRj\_F8.raw |  |  |  | 3.61E5 |  | 1.2E5 | 2.13E5 | 3.71E5 | 5.69E4 |  | 6 | 0 | 0 | 0 | 1 | 0 | 1 | 1 | 2 | 1 | 0 | 375 | 390 |  |
| K.DQIVDLTVGNNK.T | Y | 72.89 | 1314.6780 | 12 | 0.7 | 658.3467 | 2 | 46.67 | 2 | F2:15219 | 09012020\_NaNaRj\_F2.raw | 3.47E4 | 5.1E5 | 7.42E3 | 6.58E4 | 1.8E5 |  | 4.19E4 |  | 3.69E4 | 2.11E4 | 8 | 1 | 1 | 1 | 1 | 1 | 0 | 1 | 0 | 1 | 1 | 213 | 224 |  |
| R.QEYEQLIAK.N | Y | 71.79 | 1120.5764 | 9 | -0.5 | 561.2952 | 2 | 11.61 | 3 | F3:2396 | 05112019\_RID\_1313\_NaNaRj\_F3.raw |  | 1.76E7 | 1.79E5 |  | 4.73E5 |  |  |  | 6.13E4 | 1.08E5 | 6 | 0 | 2 | 1 | 0 | 1 | 0 | 0 | 0 | 1 | 1 | 328 | 336 |  |
| Y.HNLLEGGQEDFESSGAGK.I | Y | 71.38 | 1873.8442 | 18 | -0.1 | 625.6219 | 3 | 35.80 | 2 | F2:10620 | 09012020\_NaNaRj\_F2.raw |  | 1.67E5 |  |  |  |  |  |  |  |  | 1 | 0 | 1 | 0 | 0 | 0 | 0 | 0 | 0 | 0 | 0 | 455 | 472 |  |
| K.NYSPYYNTIDDLKDQIVDLTVGNNK.T | Y | 66.14 | 2901.4031 | 25 | 0.3 | 968.1420 | 3 | 41.70 | 8 | F8:16019 | 05112019\_RID\_1313\_NaNaRj\_F8.raw |  |  |  |  |  | 1.86E4 |  | 3.82E5 |  |  | 2 | 0 | 0 | 0 | 0 | 0 | 1 | 0 | 1 | 0 | 0 | 200 | 224 |  |
| S.ASSLGGGFGGGSR.G | Y | 66.10 | 1108.5261 | 13 | -0.5 | 555.2701 | 2 | 23.93 | 2 | F2:5810 | 09012020\_NaNaRj\_F2.raw |  | 1.49E5 |  |  |  |  |  |  |  |  | 1 | 0 | 1 | 0 | 0 | 0 | 0 | 0 | 0 | 0 | 0 | 83 | 95 |  |
| R.QVLDNLTMEK.S | Y | 65.98 | 1189.6013 | 10 | 0.0 | 595.8079 | 2 | 41.95 | 2 | F2:13188 | 09012020\_NaNaRj\_F2.raw |  | 2.24E6 | 1.3E5 |  |  |  |  |  | 1.04E4 | 1.05E4 | 4 | 0 | 1 | 1 | 0 | 0 | 0 | 0 | 0 | 1 | 1 | 262 | 271 |  |
| R.QVLDNLTM(+15.99)EK.S | Y | 62.66 | 1205.5962 | 10 | -0.1 | 603.8053 | 2 | 11.61 | 3 | F3:2399 | 05112019\_RID\_1313\_NaNaRj\_F3.raw |  | 1.33E6 | 1.26E5 |  |  |  |  |  |  |  | 2 | 0 | 1 | 1 | 0 | 0 | 0 | 0 | 0 | 0 | 0 | 262 | 271 | Oxidation (M) |
| Y.YNTIDDLKDQIVDLTVGNNK.T | Y | 61.91 | 2277.1489 | 20 | 0.1 | 760.0570 | 3 | 30.57 | 7 | F7:8623 | 05112019\_RID\_1313\_NaNaRj\_F7.raw |  |  |  | 3.21E4 |  |  | 7.5E4 | 7.35E4 |  |  | 3 | 0 | 0 | 0 | 1 | 0 | 0 | 1 | 1 | 0 | 0 | 205 | 224 |  |
| K.TLLDIDNTR.M | Y | 61.89 | 1059.5560 | 9 | 0.2 | 530.7854 | 2 | 12.36 | 4 | F4:3093 | 05112019\_RID\_1313\_NaNaRj\_F4.raw | 1.22E5 | 2.77E7 | 6.18E5 | 2.57E5 | 9.09E5 |  | 2.38E5 | 9.1E4 | 1.14E5 | 1.16E5 | 11 | 1 | 3 | 1 | 1 | 1 | 0 | 1 | 1 | 1 | 1 | 225 | 233 |  |
| R.QEIEC(+57.02)QNQEYSLLLSIK.M | Y | 61.32 | 2094.0303 | 17 | 0.5 | 1048.0229 | 2 | 25.30 | 6 | F6:10843 | 05112019\_RID\_1313\_NaNaRj\_F6.raw |  |  |  | 4.75E5 |  | 7.15E5 | 2.35E4 | 5E5 |  |  | 7 | 0 | 0 | 0 | 2 | 0 | 1 | 1 | 3 | 0 | 0 | 428 | 444 | Carbamidomethylation |
| R.FSSSSGYGGGSSR.V | Y | 60.39 | 1234.5214 | 13 | 0.1 | 618.2680 | 2 | 13.07 | 2 | F2:2264 | 09012020\_NaNaRj\_F2.raw | 1.18E4 | 5.95E4 |  |  | 1.47E5 |  |  |  | 1.03E4 | 3.37E4 | 5 | 1 | 1 | 0 | 0 | 1 | 0 | 0 | 0 | 1 | 1 | 47 | 59 |  |
| R.IKFEM(+15.99)EQNLR.Q | Y | 60.18 | 1322.6653 | 10 | 0.1 | 441.8958 | 3 | 24.62 | 2 | F2:6231 | 09012020\_NaNaRj\_F2.raw |  | 8.72E5 |  |  |  |  |  |  |  |  | 2 | 0 | 2 | 0 | 0 | 0 | 0 | 0 | 0 | 0 | 0 | 241 | 250 | Oxidation (M) |
| D.VNVEINVAPGK.D | Y | 59.08 | 1138.6346 | 11 | -1.1 | 570.3240 | 2 | 38.01 | 2 | F2:11562 | 09012020\_NaNaRj\_F2.raw |  | 2.91E5 |  |  |  |  |  |  |  |  | 1 | 0 | 1 | 0 | 0 | 0 | 0 | 0 | 0 | 0 | 0 | 307 | 317 |  |
| K.FEMEQNLR.Q | Y | 59.05 | 1065.4913 | 8 | -0.3 | 533.7528 | 2 | 34.12 | 2 | F2:9927 | 09012020\_NaNaRj\_F2.raw |  | 1.86E6 |  |  |  |  |  |  |  |  | 1 | 0 | 1 | 0 | 0 | 0 | 0 | 0 | 0 | 0 | 0 | 243 | 250 |  |
| K.EIETYHNLLEGGQE.D | Y | 58.61 | 1630.7474 | 14 | 0.9 | 816.3817 | 2 | 50.32 | 2 | F2:16763 | 09012020\_NaNaRj\_F2.raw |  | 1.54E5 |  |  |  |  |  |  |  |  | 1 | 0 | 1 | 0 | 0 | 0 | 0 | 0 | 0 | 0 | 0 | 450 | 463 |  |
| M.IQEQISNLEAQITDVR.Q | Y | 57.69 | 1855.9639 | 16 | 0.5 | 928.9897 | 2 | 19.14 | 8 | F8:6493 | 05112019\_RID\_1313\_NaNaRj\_F8.raw |  |  |  | 4.33E4 |  |  |  | 5.23E4 |  |  | 3 | 0 | 0 | 0 | 1 | 0 | 0 | 0 | 2 | 0 | 0 | 412 | 427 |  |
| E.QISNLEAQITDVR.Q | Y | 57.48 | 1485.7787 | 13 | 0.8 | 743.8972 | 2 | 50.76 | 2 | F2:16898 | 09012020\_NaNaRj\_F2.raw |  | 3.79E5 |  |  |  |  |  |  |  |  | 1 | 0 | 1 | 0 | 0 | 0 | 0 | 0 | 0 | 0 | 0 | 415 | 427 |  |
| S.NLEAQITDVR.Q | Y | 56.60 | 1157.6040 | 10 | 1.1 | 579.8099 | 2 | 39.94 | 2 | F2:12370 | 09012020\_NaNaRj\_F2.raw |  | 4.14E6 |  |  |  |  |  |  |  |  | 1 | 0 | 1 | 0 | 0 | 0 | 0 | 0 | 0 | 0 | 0 | 418 | 427 |  |
| K.NYSPYYNTIDDLK.D | Y | 56.53 | 1604.7358 | 13 | 0.8 | 803.3759 | 2 | 13.30 | 8 | F8:3728 | 05112019\_RID\_1313\_NaNaRj\_F8.raw |  | 3.25E5 |  | 7.33E4 |  | 0 | 4.66E4 | 5.07E4 |  |  | 5 | 0 | 1 | 0 | 1 | 0 | 1 | 1 | 1 | 0 | 0 | 200 | 212 |  |
| K.IQDWYDKK.G | Y | 55.69 | 1094.5397 | 8 | 0.9 | 548.2776 | 2 | 21.29 | 2 | F2:4819 | 09012020\_NaNaRj\_F2.raw |  | 9.62E5 |  |  |  |  |  |  |  |  | 1 | 0 | 1 | 0 | 0 | 0 | 0 | 0 | 0 | 0 | 0 | 185 | 192 |  |
| K.SDLEMQYETL.Q | Y | 55.37 | 1227.5330 | 10 | 0.9 | 614.7743 | 2 | 65.32 | 2 | F2:21431 | 09012020\_NaNaRj\_F2.raw |  | 1.24E6 |  |  |  |  |  |  |  |  | 1 | 0 | 1 | 0 | 0 | 0 | 0 | 0 | 0 | 0 | 0 | 272 | 281 |  |
| L.LEGGQEDFESSGAGK.I | Y | 54.96 | 1509.6583 | 15 | 0.1 | 755.8365 | 2 | 24.09 | 2 | F2:5879 | 09012020\_NaNaRj\_F2.raw |  | 2.56E4 |  |  |  |  |  |  |  |  | 1 | 0 | 1 | 0 | 0 | 0 | 0 | 0 | 0 | 0 | 0 | 458 | 472 |  |
| K.STMQELNSR.L | Y | 54.32 | 1064.4921 | 9 | -0.3 | 533.2532 | 2 | 10.99 | 10 | F10:1730 | 05112019\_RID\_1313\_NaNaRj\_F10.raw |  | 3.15E5 | 6.63E3 |  | 2.15E4 |  |  |  |  | 3.46E4 | 4 | 0 | 1 | 1 | 0 | 1 | 0 | 0 | 0 | 0 | 1 | 155 | 163 |  |
| N.SGDVNVEINVAPGK.D | Y | 52.34 | 1397.7150 | 14 | 0.9 | 699.8654 | 2 | 40.32 | 2 | F2:12570 | 09012020\_NaNaRj\_F2.raw |  | 5.59E4 |  |  |  |  |  |  |  |  | 1 | 0 | 1 | 0 | 0 | 0 | 0 | 0 | 0 | 0 | 0 | 304 | 317 |  |
| K.IQDWYDK.K | Y | 52.17 | 966.4447 | 7 | 0.1 | 484.2297 | 2 | 11.62 | 5 | F5:2478 | 05112019\_RID\_1313\_NaNaRj\_F5.raw | 0 | 0 | 1.47E4 |  | 2.22E5 |  |  |  | 1.49E4 | 1.59E4 | 6 | 1 | 1 | 1 | 0 | 1 | 0 | 0 | 0 | 1 | 1 | 185 | 191 |  |
| G.NYGGGSGSGGGSGGGYGGGSGSR.G | Y | 52.16 | 1847.7418 | 23 | 1.0 | 924.8791 | 2 | 10.70 | 5 | F5:1590 | 05112019\_RID\_1313\_NaNaRj\_F5.raw |  |  |  |  | 1.93E5 |  |  |  |  |  | 1 | 0 | 0 | 0 | 0 | 1 | 0 | 0 | 0 | 0 | 0 | 557 | 579 |  |
| L.GGFGGGAGGGDGGILTANEK.S | Y | 51.89 | 1690.7910 | 20 | 0.2 | 846.4030 | 2 | 11.96 | 5 | F5:2714 | 05112019\_RID\_1313\_NaNaRj\_F5.raw |  |  |  |  | 1.09E5 |  |  |  |  | 9.5E3 | 2 | 0 | 0 | 0 | 0 | 1 | 0 | 0 | 0 | 0 | 1 | 135 | 154 |  |
| I.SNLEAQITDVR.Q | Y | 51.60 | 1244.6361 | 11 | 0.6 | 623.3257 | 2 | 39.91 | 2 | F2:12383 | 09012020\_NaNaRj\_F2.raw |  | 2.4E4 |  |  |  |  |  |  |  |  | 1 | 0 | 1 | 0 | 0 | 0 | 0 | 0 | 0 | 0 | 0 | 417 | 427 |  |
| R.M(+15.99)TLDDFR.I | Y | 50.72 | 912.4011 | 7 | 0.4 | 457.2080 | 2 | 32.20 | 2 | F2:9117 | 09012020\_NaNaRj\_F2.raw |  | 0 | 1.38E5 |  | 9.31E4 |  |  |  |  | 1.93E4 | 4 | 0 | 1 | 1 | 0 | 1 | 0 | 0 | 0 | 0 | 1 | 234 | 240 | Oxidation (M) |
| F.SASSLGGGFGGGSR.G | Y | 50.26 | 1195.5581 | 14 | -0.1 | 598.7863 | 2 | 25.35 | 2 | F2:6357 | 09012020\_NaNaRj\_F2.raw | 4.27E3 | 6.68E5 |  |  |  |  |  |  |  | 1.1E4 | 4 | 1 | 2 | 0 | 0 | 0 | 0 | 0 | 0 | 0 | 1 | 82 | 95 |  |
| K.FEM(+15.99)EQNLR.Q | Y | 49.22 | 1081.4862 | 8 | 0.4 | 541.7506 | 2 | 34.18 | 2 | F2:9938 | 09012020\_NaNaRj\_F2.raw |  | 1.35E5 |  |  | 2.75E4 |  |  |  |  |  | 2 | 0 | 1 | 0 | 0 | 1 | 0 | 0 | 0 | 0 | 0 | 243 | 250 | Oxidation (M) |
| R.MTLDDFR.I | Y | 48.46 | 896.4062 | 7 | -0.7 | 449.2101 | 2 | 45.21 | 2 | F2:14760 | 09012020\_NaNaRj\_F2.raw |  | 1.55E7 | 2.13E4 |  | 3.21E4 |  |  |  |  |  | 4 | 0 | 2 | 1 | 0 | 1 | 0 | 0 | 0 | 0 | 0 | 234 | 240 |  |
| R.LASYLDK.V | N | 47.75 | 808.4330 | 7 | 0.6 | 405.2241 | 2 | 11.34 | 5 | F5:2185 | 05112019\_RID\_1313\_NaNaRj\_F5.raw | 3.03E4 | 9.71E6 | 7.78E4 |  | 7.33E5 |  |  |  |  | 3.94E4 | 5 | 1 | 1 | 1 | 0 | 1 | 0 | 0 | 0 | 0 | 1 | 164 | 170 |  |
| R.YC(+57.02)GQLQMIQEQISNLEAQITDVR.Q | Y | 47.63 | 2736.3210 | 23 | 0.6 | 913.1149 | 3 | 59.63 | 6 | F6:22579 | 05112019\_RID\_1313\_NaNaRj\_F6.raw |  |  |  |  |  | 1.55E4 |  |  |  |  | 1 | 0 | 0 | 0 | 0 | 0 | 1 | 0 | 0 | 0 | 0 | 405 | 427 | Carbamidomethylation |
| K.NYSPYYNTID.D | Y | 47.47 | 1248.5299 | 10 | 0.9 | 625.2728 | 2 | 51.66 | 2 | F2:17193 | 09012020\_NaNaRj\_F2.raw |  | 5.37E6 |  |  |  |  |  |  |  |  | 2 | 0 | 2 | 0 | 0 | 0 | 0 | 0 | 0 | 0 | 0 | 200 | 209 |  |
| R.QFSSSYLSR.S | Y | 47.39 | 1073.5142 | 9 | 0.3 | 537.7645 | 2 | 11.52 | 3 | F3:2314 | 05112019\_RID\_1313\_NaNaRj\_F3.raw |  | 2.64E6 | 5.66E4 |  |  |  |  |  |  |  | 2 | 0 | 1 | 1 | 0 | 0 | 0 | 0 | 0 | 0 | 0 | 5 | 13 |  |
| R.HGVQELEIELQ.S | Y | 46.46 | 1293.6565 | 11 | 0.3 | 647.8357 | 2 | 53.72 | 2 | F2:17923 | 09012020\_NaNaRj\_F2.raw |  | 2.95E6 |  |  |  |  |  |  |  |  | 1 | 0 | 1 | 0 | 0 | 0 | 0 | 0 | 0 | 0 | 0 | 375 | 385 |  |
| Q.ISNLEAQITDVR.Q | Y | 46.06 | 1357.7201 | 12 | 0.3 | 679.8676 | 2 | 12.26 | 5 | F5:3048 | 05112019\_RID\_1313\_NaNaRj\_F5.raw |  |  |  |  | 6.96E4 |  |  |  |  |  | 1 | 0 | 0 | 0 | 0 | 1 | 0 | 0 | 0 | 0 | 0 | 416 | 427 |  |
| S.SLGGGFGGGSR.G | Y | 46.00 | 950.4570 | 11 | 0.2 | 476.2358 | 2 | 22.25 | 2 | F2:5311 | 09012020\_NaNaRj\_F2.raw |  | 7.77E6 |  |  | 7.6E4 |  |  |  |  |  | 2 | 0 | 1 | 0 | 0 | 1 | 0 | 0 | 0 | 0 | 0 | 85 | 95 |  |
| S.YGGGSGGGFSASSLGGGFGGGSR.G | Y | 44.68 | 1934.8506 | 23 | 2.7 | 968.4352 | 2 | 49.89 | 2 | F2:16582 | 09012020\_NaNaRj\_F2.raw |  | 1.15E4 |  |  |  |  |  |  |  |  | 1 | 0 | 1 | 0 | 0 | 0 | 0 | 0 | 0 | 0 | 0 | 73 | 95 |  |
| R.YC(+57.02)GQLQM(+15.99)IQEQISNLEAQITDVR.Q | Y | 43.88 | 2752.3159 | 23 | 0.4 | 1377.1658 | 2 | 54.08 | 6 | F6:21189 | 05112019\_RID\_1313\_NaNaRj\_F6.raw |  |  |  |  |  | 1.35E4 |  |  |  |  | 1 | 0 | 0 | 0 | 0 | 0 | 1 | 0 | 0 | 0 | 0 | 405 | 427 | Carbamidomethylation; Oxidation (M) |
| K.STM(+15.99)QELNSR.L | Y | 41.78 | 1080.4869 | 9 | 0.2 | 541.2509 | 2 | 17.36 | 2 | F2:3314 | 09012020\_NaNaRj\_F2.raw |  | 8.58E4 |  |  |  |  |  |  |  |  | 1 | 0 | 1 | 0 | 0 | 0 | 0 | 0 | 0 | 0 | 0 | 155 | 163 | Oxidation (M) |
| K.SDLEM(+15.99)QYETLQEELM(+15.99)ALK.K | Y | 41.27 | 2202.0071 | 18 | 0.6 | 1102.0115 | 2 | 42.72 | 6 | F6:18144 | 05112019\_RID\_1313\_NaNaRj\_F6.raw |  |  |  |  |  | 6.65E4 |  |  |  |  | 1 | 0 | 0 | 0 | 0 | 0 | 1 | 0 | 0 | 0 | 0 | 272 | 289 | Oxidation (M) |
| Y.YNTIDDLK.D | Y | 40.06 | 980.4814 | 8 | 0.5 | 491.2482 | 2 | 11.54 | 5 | F5:2417 | 05112019\_RID\_1313\_NaNaRj\_F5.raw |  |  |  |  | 3.7E4 |  |  |  |  |  | 1 | 0 | 0 | 0 | 0 | 1 | 0 | 0 | 0 | 0 | 0 | 205 | 212 |  |
| total 52 peptides |  |  |  |  |  |  |  |  |  |  |  |  |  |  |  |  |  |  |  |  |  |  |  |  |  |  |  |  |  |  |  |  |  |  |  |
| --- | --- | --- | --- | --- | --- | --- | --- | --- | --- | --- | --- | --- | --- | --- | --- | --- | --- | --- | --- | --- | --- | --- | --- | --- | --- | --- | --- | --- | --- | --- | --- | --- | --- | --- | --- |

P13645|K1C10\_HUMAN

back to list

  

| Protein Coverage
| Supporting Peptides
|

Protein Coverage:

Supporting Peptides:

| Peptide | Uniq | -10lgP | Mass | Length | ppm | m/z | z | RT | Fraction | Scan | Source File | Area F1 | Area F2 | Area F3 | Area F4 | Area F5 | Area F6 | Area F7 | Area F8 | Area F9 | Area F10 | #Feature | #Feature F1 | #Feature F2 | #Feature F3 | #Feature F4 | #Feature F5 | #Feature F6 | #Feature F7 | #Feature F8 | #Feature F9 | #Feature F10 | Start | End | PTM |
| --- | --- | --- | --- | --- | --- | --- | --- | --- | --- | --- | --- | --- | --- | --- | --- | --- | --- | --- | --- | --- | --- | --- | --- | --- | --- | --- | --- | --- | --- | --- | --- | --- | --- | --- | --- |
| K.ELTTEIDNNIEQISSYK.S | Y | 109.90 | 1995.9636 | 17 | -1.8 | 998.9872 | 2 | 20.06 | 6 | F6:8155 | 05112019\_RID\_1313\_NaNaRj\_F6.raw | 6.82E4 |  | 7.54E4 | 7.16E5 | 2.88E5 | 9.41E5 | 5.91E5 | 3.34E5 | 1.5E4 |  | 10 | 1 | 0 | 1 | 1 | 2 | 1 | 2 | 1 | 1 | 0 | 346 | 362 |  |
| R.AETEC(+57.02)QNTEYQQLLDIK.I | Y | 106.55 | 2081.9575 | 17 | -0.4 | 1041.9856 | 2 | 16.44 | 6 | F6:5707 | 05112019\_RID\_1313\_NaNaRj\_F6.raw | 1.06E4 |  | 1.19E6 | 7.6E4 | 2.78E6 | 1.27E6 |  | 4.43E5 | 1.36E4 | 2.58E4 | 13 | 1 | 0 | 2 | 1 | 3 | 2 | 0 | 2 | 1 | 1 | 423 | 439 | Carbamidomethylation |
| R.SGGGGGGGGC(+57.02)GGGGGVSSLR.I | Y | 103.19 | 1548.6699 | 20 | 0.4 | 775.3425 | 2 | 10.93 | 5 | F5:1784 | 05112019\_RID\_1313\_NaNaRj\_F5.raw |  | 1.62E5 |  |  | 6.91E4 |  |  |  |  |  | 3 | 0 | 2 | 0 | 0 | 1 | 0 | 0 | 0 | 0 | 0 | 16 | 35 | Carbamidomethylation |
| K.GSLGGGFSSGGFSGGSFSR.G | Y | 101.13 | 1706.7648 | 19 | 0.4 | 854.3900 | 2 | 13.16 | 4 | F4:3754 | 05112019\_RID\_1313\_NaNaRj\_F4.raw |  | 2.24E5 | 1.31E5 | 1.55E5 | 5.83E4 | 2.48E5 | 1.12E5 | 1.73E5 |  |  | 8 | 0 | 2 | 1 | 1 | 1 | 1 | 1 | 1 | 0 | 0 | 41 | 59 |  |
| R.SQYEQLAEQNR.K | Y | 93.89 | 1364.6321 | 11 | 1.0 | 683.3240 | 2 | 24.28 | 2 | F2:5938 | 09012020\_NaNaRj\_F2.raw | 0 | 8.99E5 | 0 |  | 2.45E6 |  | 1.2E5 |  | 1.15E4 | 7.74E4 | 7 | 1 | 1 | 1 | 0 | 1 | 0 | 1 | 0 | 1 | 1 | 323 | 333 |  |
| R.ALEESNYELEGK.I | Y | 90.43 | 1380.6409 | 12 | 0.1 | 691.3278 | 2 | 11.46 | 3 | F3:2247 | 05112019\_RID\_1313\_NaNaRj\_F3.raw | 9.63E4 | 6.1E6 | 1.08E6 |  | 2.3E6 |  | 8.87E4 |  | 1.05E4 | 1.42E5 | 8 | 1 | 2 | 1 | 0 | 1 | 0 | 1 | 0 | 1 | 1 | 166 | 177 |  |
| R.GSSGGGC(+57.02)FGGSSGGYGGLGGFGGGSFR.G | Y | 86.51 | 2341.9771 | 27 | -0.3 | 1171.9955 | 2 | 16.44 | 6 | F6:5735 | 05112019\_RID\_1313\_NaNaRj\_F6.raw |  |  |  | 3.53E5 |  | 6.41E5 |  | 1.91E5 |  |  | 4 | 0 | 0 | 0 | 1 | 0 | 2 | 0 | 1 | 0 | 0 | 60 | 86 | Carbamidomethylation |
| K.QSLEASLAETEGR.Y | N | 84.81 | 1389.6736 | 13 | 0.5 | 695.8444 | 2 | 11.87 | 5 | F5:2708 | 05112019\_RID\_1313\_NaNaRj\_F5.raw | 2.09E4 | 5.42E4 | 2.8E5 |  | 1.2E6 |  | 1.56E5 |  |  | 3.36E4 | 6 | 1 | 1 | 1 | 0 | 1 | 0 | 1 | 0 | 0 | 1 | 387 | 399 |  |
| R.NVSTGDVNVEM(+15.99)NAAPGVDLTQLLNNM(+15.99)R.S | Y | 74.96 | 2903.3752 | 27 | 1.2 | 968.8002 | 3 | 28.97 | 3 | F3:5515 | 05112019\_RID\_1313\_NaNaRj\_F3.raw |  |  | 3.09E4 |  |  |  |  |  |  |  | 1 | 0 | 0 | 1 | 0 | 0 | 0 | 0 | 0 | 0 | 0 | 296 | 322 | Oxidation (M) |
| R.NVQALEIELQSQLALK.Q | Y | 73.99 | 1796.0043 | 16 | 0.4 | 899.0098 | 2 | 33.15 | 4 | F4:9790 | 05112019\_RID\_1313\_NaNaRj\_F4.raw |  |  |  | 4.15E4 |  | 7.44E5 | 7.92E4 |  |  |  | 7 | 0 | 0 | 0 | 1 | 0 | 4 | 2 | 0 | 0 | 0 | 371 | 386 |  |
| R.SLLEGEGSSGGGGR.G | Y | 73.93 | 1261.5898 | 14 | -0.1 | 631.8021 | 2 | 11.22 | 3 | F3:2025 | 05112019\_RID\_1313\_NaNaRj\_F3.raw | 5.64E3 | 2.16E6 | 1.11E5 | 0 |  |  |  |  |  | 3.94E4 | 6 | 1 | 2 | 1 | 1 | 0 | 0 | 0 | 0 | 0 | 1 | 451 | 464 |  |
| S.GGGGGGGGC(+57.02)GGGGGVSSLR.I | Y | 69.97 | 1461.6378 | 19 | -0.3 | 731.8260 | 2 | 15.09 | 2 | F2:2792 | 09012020\_NaNaRj\_F2.raw |  | 2.09E3 |  |  |  |  |  |  |  |  | 1 | 0 | 1 | 0 | 0 | 0 | 0 | 0 | 0 | 0 | 0 | 17 | 35 | Carbamidomethylation |
| R.VLDELTLTK.A | N | 69.64 | 1030.5911 | 9 | -0.4 | 516.3026 | 2 | 12.62 | 4 | F4:3299 | 05112019\_RID\_1313\_NaNaRj\_F4.raw | 1.03E5 | 1.18E7 | 7.95E5 | 2.87E5 | 1.99E6 | 2.83E5 | 2.47E5 | 6.56E4 |  | 7.86E4 | 10 | 1 | 2 | 1 | 1 | 1 | 1 | 1 | 1 | 0 | 1 | 258 | 266 |  |
| K.VTMQNLNDR.L | N | 68.03 | 1089.5237 | 9 | 0.5 | 545.7694 | 2 | 11.19 | 5 | F5:2040 | 05112019\_RID\_1313\_NaNaRj\_F5.raw |  |  | 1.98E5 |  | 2.56E5 |  |  |  |  | 4.68E4 | 3 | 0 | 0 | 1 | 0 | 1 | 0 | 0 | 0 | 0 | 1 | 148 | 156 |  |
| R.SQYEQLAEQNRK.D | Y | 67.60 | 1492.7269 | 12 | -1.3 | 498.5823 | 3 | 10.84 | 3 | F3:1698 | 05112019\_RID\_1313\_NaNaRj\_F3.raw |  |  | 2.43E5 |  | 4.86E4 |  |  |  |  | 1.4E4 | 3 | 0 | 0 | 1 | 0 | 1 | 0 | 0 | 0 | 0 | 1 | 323 | 334 |  |
| K.NQILNLTTDNANILLQIDNAR.L | Y | 67.48 | 2366.2554 | 21 | 1.0 | 1184.1361 | 2 | 38.81 | 8 | F8:14673 | 05112019\_RID\_1313\_NaNaRj\_F8.raw |  |  |  |  |  |  | 3.65E4 | 4E5 |  |  | 4 | 0 | 0 | 0 | 0 | 0 | 0 | 2 | 2 | 0 | 0 | 208 | 228 |  |
| R.LENEIQTYR.S | N | 65.53 | 1164.5775 | 9 | 0.1 | 583.2961 | 2 | 11.38 | 5 | F5:2209 | 05112019\_RID\_1313\_NaNaRj\_F5.raw | 7.77E4 | 9.7E6 | 2.68E5 | 1.28E5 | 2.96E6 |  |  |  |  | 1.17E5 | 9 | 1 | 4 | 1 | 1 | 1 | 0 | 0 | 0 | 0 | 1 | 442 | 450 |  |
| R.QSVEADINGLR.R | N | 63.94 | 1200.6099 | 11 | -0.1 | 601.3121 | 2 | 39.03 | 2 | F2:11958 | 09012020\_NaNaRj\_F2.raw | 4.07E4 | 4.05E6 |  |  | 0 |  |  |  |  |  | 5 | 1 | 3 | 0 | 0 | 1 | 0 | 0 | 0 | 0 | 0 | 246 | 256 |  |
| R.LKYENEVALR.Q | Y | 63.84 | 1233.6716 | 10 | 0.3 | 412.2313 | 3 | 27.80 | 2 | F2:7341 | 09012020\_NaNaRj\_F2.raw |  | 1.72E6 |  |  | 9.97E4 |  |  |  |  |  | 2 | 0 | 1 | 0 | 0 | 1 | 0 | 0 | 0 | 0 | 0 | 236 | 245 |  |
| S.ALEEQLQQIR.A | Y | 59.44 | 1226.6619 | 10 | 0.0 | 614.3382 | 2 | 40.21 | 2 | F2:12465 | 09012020\_NaNaRj\_F2.raw |  | 5.5E5 |  |  |  |  |  |  |  |  | 1 | 0 | 1 | 0 | 0 | 0 | 0 | 0 | 0 | 0 | 0 | 413 | 422 |  |
| K.ADLEMQIESLTEELAYLK.K | Y | 58.77 | 2095.0396 | 18 | 1.4 | 699.3547 | 3 | 62.60 | 6 | F6:23151 | 05112019\_RID\_1313\_NaNaRj\_F6.raw |  |  |  |  |  | 2.04E4 |  |  |  |  | 1 | 0 | 0 | 0 | 0 | 0 | 1 | 0 | 0 | 0 | 0 | 267 | 284 |  |
| Q.ISALEEQLQQIR.A | Y | 58.33 | 1426.7780 | 12 | 0.8 | 714.3968 | 2 | 14.58 | 6 | F6:4506 | 05112019\_RID\_1313\_NaNaRj\_F6.raw |  | 1.05E6 |  |  | 6.62E5 | 4.55E5 |  | 1.87E5 |  |  | 5 | 0 | 1 | 0 | 0 | 2 | 1 | 0 | 1 | 0 | 0 | 411 | 422 |  |
| L.RQSVEADINGLR.R | N | 57.75 | 1356.7109 | 12 | -0.5 | 453.2440 | 3 | 28.20 | 2 | F2:7574 | 09012020\_NaNaRj\_F2.raw |  | 4.85E4 |  |  |  |  |  |  |  |  | 1 | 0 | 1 | 0 | 0 | 0 | 0 | 0 | 0 | 0 | 0 | 245 | 256 |  |
| K.YENEVALR.Q | Y | 57.12 | 992.4927 | 8 | 0.5 | 497.2538 | 2 | 11.32 | 5 | F5:2160 | 05112019\_RID\_1313\_NaNaRj\_F5.raw | 2.96E4 | 2.79E6 | 6.92E4 |  | 1.23E6 |  |  |  |  | 4.51E4 | 6 | 1 | 2 | 1 | 0 | 1 | 0 | 0 | 0 | 0 | 1 | 238 | 245 |  |
| K.DAEAWFNEK.S | N | 56.45 | 1108.4825 | 9 | 0.2 | 555.2487 | 2 | 13.37 | 4 | F4:3903 | 05112019\_RID\_1313\_NaNaRj\_F4.raw | 3.31E4 | 2.81E6 | 5.8E5 | 1.8E5 | 1.24E6 | 2.74E5 | 1.77E5 |  | 9.52E3 | 3.81E4 | 11 | 1 | 2 | 1 | 1 | 2 | 1 | 1 | 0 | 1 | 1 | 335 | 343 |  |
| K.IRLENEIQTYR.S | N | 50.89 | 1433.7627 | 11 | -0.3 | 478.9280 | 3 | 34.87 | 2 | F2:10211 | 09012020\_NaNaRj\_F2.raw |  | 1.25E6 |  |  |  |  |  |  |  |  | 2 | 0 | 2 | 0 | 0 | 0 | 0 | 0 | 0 | 0 | 0 | 440 | 450 |  |
| E.SLTEELAYLK.K | N | 49.27 | 1165.6230 | 10 | 0.9 | 583.8193 | 2 | 58.68 | 2 | F2:19462 | 09012020\_NaNaRj\_F2.raw |  | 3.58E6 |  |  |  |  |  |  |  |  | 1 | 0 | 1 | 0 | 0 | 0 | 0 | 0 | 0 | 0 | 0 | 275 | 284 |  |
| K.ADLEM(+15.99)QIESLTEELAYLK.K | Y | 48.80 | 2111.0344 | 18 | 1.7 | 1056.5262 | 2 | 64.29 | 6 | F6:23464 | 05112019\_RID\_1313\_NaNaRj\_F6.raw |  |  |  |  |  | 6.31E4 |  |  |  |  | 2 | 0 | 0 | 0 | 0 | 0 | 2 | 0 | 0 | 0 | 0 | 267 | 284 | Oxidation (M) |
| R.YC(+57.02)VQLSQIQAQ.I | Y | 48.51 | 1336.6445 | 11 | 0.8 | 669.3301 | 2 | 14.68 | 7 | F7:4165 | 05112019\_RID\_1313\_NaNaRj\_F7.raw |  |  |  |  | 6.78E5 |  | 6.6E4 |  |  |  | 2 | 0 | 0 | 0 | 0 | 1 | 0 | 1 | 0 | 0 | 0 | 400 | 410 | Carbamidomethylation |
| S.SGGYGGLGGFGGGSFR.G | Y | 48.41 | 1431.6531 | 16 | -0.6 | 716.8334 | 2 | 13.56 | 5 | F5:3559 | 05112019\_RID\_1313\_NaNaRj\_F5.raw |  |  |  |  | 1.35E4 |  |  |  |  |  | 1 | 0 | 0 | 0 | 0 | 1 | 0 | 0 | 0 | 0 | 0 | 71 | 86 |  |
| R.LASYLDK.V | N | 47.75 | 808.4330 | 7 | 0.6 | 405.2241 | 2 | 11.34 | 5 | F5:2185 | 05112019\_RID\_1313\_NaNaRj\_F5.raw | 3.03E4 | 9.71E6 | 7.78E4 |  | 7.33E5 |  |  |  |  | 3.94E4 | 5 | 1 | 1 | 1 | 0 | 1 | 0 | 0 | 0 | 0 | 1 | 157 | 163 |  |
| R.YC(+57.02)VQLSQIQAQISALEEQLQQIR.A | Y | 47.64 | 2745.4119 | 23 | 1.0 | 916.1455 | 3 | 62.78 | 6 | F6:23170 | 05112019\_RID\_1313\_NaNaRj\_F6.raw |  |  |  |  |  | 8.11E4 |  |  |  |  | 1 | 0 | 0 | 0 | 0 | 0 | 1 | 0 | 0 | 0 | 0 | 400 | 422 | Carbamidomethylation |
| R.LAADDFR.L | N | 46.83 | 806.3922 | 7 | 0.6 | 404.2036 | 2 | 11.46 | 5 | F5:2311 | 05112019\_RID\_1313\_NaNaRj\_F5.raw |  | 5.1E6 | 5.72E4 |  | 4.73E5 |  | 1.38E4 |  |  | 3.3E4 | 6 | 0 | 2 | 1 | 0 | 1 | 0 | 1 | 0 | 0 | 1 | 229 | 235 |  |
| L.SQIQAQISALEEQLQQIR.A | Y | 45.42 | 2082.1069 | 18 | -1.7 | 695.0417 | 3 | 41.60 | 6 | F6:17805 | 05112019\_RID\_1313\_NaNaRj\_F6.raw |  |  |  |  |  | 1.35E4 |  |  |  |  | 1 | 0 | 0 | 0 | 0 | 0 | 1 | 0 | 0 | 0 | 0 | 405 | 422 |  |
| R.LASYLDKVR.A | N | 45.41 | 1063.6025 | 9 | 1.0 | 532.8091 | 2 | 27.68 | 2 | F2:7299 | 09012020\_NaNaRj\_F2.raw |  | 2.48E5 |  |  |  |  |  |  |  |  | 1 | 0 | 1 | 0 | 0 | 0 | 0 | 0 | 0 | 0 | 0 | 157 | 165 |  |
| D.NANILLQIDNAR.L | N | 45.41 | 1353.7365 | 12 | 0.5 | 677.8759 | 2 | 55.43 | 2 | F2:18549 | 09012020\_NaNaRj\_F2.raw |  | 4.41E4 |  |  |  |  |  |  |  |  | 1 | 0 | 1 | 0 | 0 | 0 | 0 | 0 | 0 | 0 | 0 | 217 | 228 |  |
| N.ILLQIDNAR.L | N | 45.23 | 1054.6135 | 9 | 0.6 | 528.3143 | 2 | 12.34 | 7 | F7:3056 | 05112019\_RID\_1313\_NaNaRj\_F7.raw |  |  |  |  |  |  | 3.53E4 |  |  |  | 1 | 0 | 0 | 0 | 0 | 0 | 0 | 1 | 0 | 0 | 0 | 220 | 228 |  |
| G.GSSGGYGGLGGFGGGSFR.G | Y | 44.03 | 1575.7065 | 18 | -0.2 | 788.8604 | 2 | 52.01 | 2 | F2:17353 | 09012020\_NaNaRj\_F2.raw |  | 1.87E4 |  |  |  |  |  |  |  |  | 1 | 0 | 1 | 0 | 0 | 0 | 0 | 0 | 0 | 0 | 0 | 69 | 86 |  |
| F.GGGFGGGFGGDGGLLSGNEK.V | Y | 41.85 | 1738.7910 | 20 | 0.8 | 870.4034 | 2 | 16.63 | 5 | F5:4271 | 05112019\_RID\_1313\_NaNaRj\_F5.raw |  |  |  |  | 2.56E4 |  |  |  |  |  | 1 | 0 | 0 | 0 | 0 | 1 | 0 | 0 | 0 | 0 | 0 | 128 | 147 |  |
| F.SSGGFSGGSFSR.G | N | 41.79 | 1131.4945 | 12 | 0.2 | 566.7546 | 2 | 11.34 | 5 | F5:2187 | 05112019\_RID\_1313\_NaNaRj\_F5.raw |  |  |  |  | 8.47E4 |  |  |  |  |  | 1 | 0 | 0 | 0 | 0 | 1 | 0 | 0 | 0 | 0 | 0 | 48 | 59 |  |
| K.SEITELR.R | N | 41.71 | 846.4447 | 7 | 0.9 | 424.2300 | 2 | 11.36 | 5 | F5:2206 | 05112019\_RID\_1313\_NaNaRj\_F5.raw |  |  |  |  | 9.35E5 |  |  |  |  |  | 1 | 0 | 0 | 0 | 0 | 1 | 0 | 0 | 0 | 0 | 0 | 363 | 369 |  |
| R.NVQALEIELQ.S | Y | 40.56 | 1155.6135 | 10 | 0.1 | 578.8141 | 2 | 68.09 | 2 | F2:22189 | 09012020\_NaNaRj\_F2.raw |  | 1.48E6 |  |  |  |  |  |  |  |  | 1 | 0 | 1 | 0 | 0 | 0 | 0 | 0 | 0 | 0 | 0 | 371 | 380 |  |
| K.VTM(+15.99)QNLNDR.L | N | 40.47 | 1105.5186 | 9 | 1.2 | 553.7672 | 2 | 17.56 | 2 | F2:3384 | 09012020\_NaNaRj\_F2.raw |  | 0 |  |  |  |  |  |  |  |  | 1 | 0 | 1 | 0 | 0 | 0 | 0 | 0 | 0 | 0 | 0 | 148 | 156 | Oxidation (M) |
| K.NQILNLTTDNAN.I | Y | 40.29 | 1329.6525 | 12 | 0.0 | 665.8335 | 2 | 15.80 | 5 | F5:4082 | 05112019\_RID\_1313\_NaNaRj\_F5.raw |  |  |  |  | 5.3E5 |  |  |  |  |  | 1 | 0 | 0 | 0 | 0 | 1 | 0 | 0 | 0 | 0 | 0 | 208 | 219 |  |
| total 44 peptides |  |  |  |  |  |  |  |  |  |  |  |  |  |  |  |  |  |  |  |  |  |  |  |  |  |  |  |  |  |  |  |  |  |  |  |
| --- | --- | --- | --- | --- | --- | --- | --- | --- | --- | --- | --- | --- | --- | --- | --- | --- | --- | --- | --- | --- | --- | --- | --- | --- | --- | --- | --- | --- | --- | --- | --- | --- | --- | --- | --- |

P04264|K2C1\_HUMAN

back to list

  

| Protein Coverage
| Supporting Peptides
|

Protein Coverage:

Supporting Peptides:

| Peptide | Uniq | -10lgP | Mass | Length | ppm | m/z | z | RT | Fraction | Scan | Source File | Area F1 | Area F2 | Area F3 | Area F4 | Area F5 | Area F6 | Area F7 | Area F8 | Area F9 | Area F10 | #Feature | #Feature F1 | #Feature F2 | #Feature F3 | #Feature F4 | #Feature F5 | #Feature F6 | #Feature F7 | #Feature F8 | #Feature F9 | #Feature F10 | Start | End | PTM |
| --- | --- | --- | --- | --- | --- | --- | --- | --- | --- | --- | --- | --- | --- | --- | --- | --- | --- | --- | --- | --- | --- | --- | --- | --- | --- | --- | --- | --- | --- | --- | --- | --- | --- | --- | --- |
| R.FSSC(+57.02)GGGGGSFGAGGGFGSR.S | Y | 109.10 | 1764.7274 | 20 | 1.0 | 883.3718 | 2 | 11.71 | 7 | F7:2525 | 05112019\_RID\_1313\_NaNaRj\_F7.raw | 2.12E4 | 5.64E5 | 1.65E5 | 3.29E5 | 9.42E4 | 1.12E5 | 1.47E5 | 1.04E5 | 3.61E4 | 1.6E4 | 12 | 1 | 3 | 1 | 1 | 1 | 1 | 1 | 1 | 1 | 1 | 46 | 65 | Carbamidomethylation |
| R.SGGGFSSGSAGIINYQR.R | Y | 108.42 | 1656.7855 | 17 | 0.7 | 829.4006 | 2 | 43.46 | 2 | F2:13784 | 09012020\_NaNaRj\_F2.raw |  | 1.41E6 | 1.3E5 |  | 2.22E5 |  |  |  |  | 6.62E3 | 7 | 0 | 3 | 2 | 0 | 1 | 0 | 0 | 0 | 0 | 1 | 13 | 29 |  |
| R.THNLEPYFESFINNLR.R | Y | 100.33 | 1992.9694 | 16 | 0.7 | 665.3309 | 3 | 32.95 | 6 | F6:14718 | 05112019\_RID\_1313\_NaNaRj\_F6.raw |  |  |  |  |  | 1.42E6 | 8.49E5 | 1.11E6 |  |  | 5 | 0 | 0 | 0 | 0 | 0 | 2 | 1 | 2 | 0 | 0 | 224 | 239 |  |
| R.GGGGGGYGSGGSSYGSGGGSYGSGGGGGGGR.G | Y | 97.21 | 2382.9446 | 31 | 0.3 | 1192.4800 | 2 | 10.96 | 3 | F3:1821 | 05112019\_RID\_1313\_NaNaRj\_F3.raw |  | 2.3E4 | 3.06E5 | 6.72E4 | 2.24E5 |  |  |  |  | 6.65E4 | 7 | 0 | 1 | 2 | 1 | 2 | 0 | 0 | 0 | 0 | 1 | 519 | 549 |  |
| K.QISNLQQSISDAEQR.G | Y | 94.90 | 1715.8438 | 15 | -0.2 | 858.9290 | 2 | 11.87 | 7 | F7:2664 | 05112019\_RID\_1313\_NaNaRj\_F7.raw |  | 3.73E5 | 1.64E4 | 2.36E5 | 7.2E4 | 6.87E4 | 1.67E5 | 9.23E4 |  |  | 8 | 0 | 1 | 1 | 1 | 2 | 1 | 1 | 1 | 0 | 0 | 418 | 432 |  |
| K.LNDLEDALQQAK.E | Y | 94.02 | 1356.6885 | 12 | 1.1 | 679.3522 | 2 | 49.83 | 2 | F2:16674 | 09012020\_NaNaRj\_F2.raw | 3.31E5 | 2.34E7 | 1.8E5 | 9.99E5 | 3E6 | 1.2E6 | 6.47E5 | 5.55E5 | 2E5 | 1.41E5 | 15 | 1 | 3 | 1 | 1 | 3 | 1 | 2 | 1 | 1 | 1 | 444 | 455 |  |
| R.FLEQQNQVLQTK.W | N | 88.32 | 1474.7780 | 12 | 0.9 | 738.3969 | 2 | 11.66 | 3 | F3:2423 | 05112019\_RID\_1313\_NaNaRj\_F3.raw | 3.29E5 | 6.5E6 | 2.66E6 | 9.27E5 | 3.5E6 |  | 4.3E5 | 1.41E5 | 1.56E5 | 4.01E5 | 11 | 1 | 2 | 1 | 1 | 2 | 0 | 1 | 1 | 1 | 1 | 200 | 211 |  |
| R.TNAENEFVTIK.K | N | 87.64 | 1264.6299 | 11 | 0.5 | 633.3225 | 2 | 11.87 | 1 | F1:2610 | 05112019\_RID\_1313\_NaNaRj\_F1.raw | 1.49E5 | 3.38E7 | 4.51E5 | 4.28E5 | 2.06E6 | 2.15E5 | 2.42E5 |  | 9.25E4 | 2.42E5 | 11 | 1 | 3 | 1 | 1 | 1 | 1 | 1 | 0 | 1 | 1 | 278 | 288 |  |
| R.TNAENEFVTIKK.D | N | 83.94 | 1392.7249 | 12 | 0.3 | 465.2491 | 3 | 11.28 | 3 | F3:2082 | 05112019\_RID\_1313\_NaNaRj\_F3.raw |  | 7.12E5 | 2.84E5 |  |  |  |  |  |  |  | 3 | 0 | 1 | 2 | 0 | 0 | 0 | 0 | 0 | 0 | 0 | 278 | 289 |  |
| K.YEELQITAGR.H | N | 83.22 | 1178.5931 | 10 | 1.2 | 590.3046 | 2 | 11.78 | 1 | F1:2548 | 05112019\_RID\_1313\_NaNaRj\_F1.raw | 1.61E5 | 2.9E7 | 5.44E5 | 0 |  |  | 2.71E5 |  | 8.56E4 | 1.7E5 | 12 | 1 | 6 | 1 | 1 | 0 | 0 | 1 | 0 | 1 | 1 | 377 | 386 |  |
| R.SLDLDSIIAEVK.A | N | 77.46 | 1301.7078 | 12 | 0.1 | 651.8612 | 2 | 36.15 | 9 | F9:7311 | 05112019\_RID\_1313\_NaNaRj\_F9.raw |  |  | 5.36E4 | 1.7E6 | 7.12E4 | 1.73E6 | 1.45E6 | 1.17E6 | 1.5E5 |  | 8 | 0 | 0 | 1 | 2 | 1 | 1 | 1 | 1 | 1 | 0 | 344 | 355 |  |
| K.SLNNQFASFIDK.V | N | 74.11 | 1382.6830 | 12 | 0.2 | 692.3489 | 2 | 17.46 | 7 | F7:5238 | 05112019\_RID\_1313\_NaNaRj\_F7.raw |  | 3.79E6 | 1.31E5 | 1.14E6 | 5.46E5 | 1.42E6 | 8.15E5 | 6.94E5 | 1.55E5 |  | 10 | 0 | 1 | 1 | 1 | 2 | 1 | 2 | 1 | 1 | 0 | 186 | 197 |  |
| K.WELLQQVDTSTR.T | N | 73.71 | 1474.7416 | 12 | 0.8 | 738.3787 | 2 | 17.24 | 5 | F5:4439 | 05112019\_RID\_1313\_NaNaRj\_F5.raw |  | 1.44E6 |  | 5.02E4 | 4.41E5 |  | 2.74E5 | 5.29E4 |  |  | 7 | 0 | 2 | 0 | 1 | 2 | 0 | 1 | 1 | 0 | 0 | 212 | 223 |  |
| K.AEAESLYQSK.Y | Y | 68.94 | 1124.5349 | 10 | 0.8 | 563.2752 | 2 | 11.09 | 10 | F10:1780 | 05112019\_RID\_1313\_NaNaRj\_F10.raw | 6.14E4 | 1.72E6 | 9.29E4 | 3.39E4 | 6.02E5 |  |  |  | 5.22E4 | 6.54E4 | 8 | 1 | 2 | 1 | 1 | 1 | 0 | 0 | 0 | 1 | 1 | 367 | 376 |  |
| K.AQYEDIAQK.S | N | 67.86 | 1064.5138 | 9 | 0.5 | 533.2645 | 2 | 11.04 | 5 | F5:1897 | 05112019\_RID\_1313\_NaNaRj\_F5.raw | 6.84E4 | 8.74E6 | 3.03E5 | 4.6E4 | 6.07E5 |  |  |  | 5.67E4 | 1.46E5 | 7 | 1 | 1 | 1 | 1 | 1 | 0 | 0 | 0 | 1 | 1 | 356 | 364 |  |
| R.NKYEDEINKR.T | N | 67.54 | 1307.6470 | 10 | 0.5 | 436.8898 | 3 | 12.46 | 2 | F2:2012 | 09012020\_NaNaRj\_F2.raw |  | 4.15E6 |  |  | 2.01E5 |  |  |  |  |  | 3 | 0 | 2 | 0 | 0 | 1 | 0 | 0 | 0 | 0 | 0 | 268 | 277 |  |
| K.DVDGAYMTK.V | Y | 67.08 | 998.4379 | 9 | 0.3 | 500.2264 | 2 | 24.99 | 2 | F2:6251 | 09012020\_NaNaRj\_F2.raw |  | 1.49E6 | 7.35E4 |  | 5.71E4 |  |  |  |  | 1.34E4 | 4 | 0 | 1 | 1 | 0 | 1 | 0 | 0 | 0 | 0 | 1 | 290 | 298 |  |
| R.THNLEPYFESF.I | Y | 65.63 | 1382.6143 | 11 | 0.6 | 692.3148 | 2 | 19.81 | 4 | F4:7045 | 05112019\_RID\_1313\_NaNaRj\_F4.raw |  | 3.86E5 |  | 1.73E5 |  | 1.8E5 | 1.37E5 | 1.79E5 | 3.53E4 |  | 6 | 0 | 1 | 0 | 1 | 0 | 1 | 1 | 1 | 1 | 0 | 224 | 234 |  |
| G.GGGGSFGAGGGFGSR.S | Y | 65.53 | 1226.5428 | 15 | 0.4 | 614.2789 | 2 | 29.22 | 2 | F2:7916 | 09012020\_NaNaRj\_F2.raw |  | 1.92E5 |  |  |  |  |  |  |  |  | 1 | 0 | 1 | 0 | 0 | 0 | 0 | 0 | 0 | 0 | 0 | 51 | 65 |  |
| K.SKAEAESLYQSK.Y | Y | 65.49 | 1339.6619 | 12 | 0.0 | 447.5612 | 3 | 10.70 | 3 | F3:1634 | 05112019\_RID\_1313\_NaNaRj\_F3.raw |  | 3.85E5 | 3.7E4 |  | 9.07E4 |  |  |  |  | 8.5E3 | 5 | 0 | 1 | 1 | 0 | 2 | 0 | 0 | 0 | 0 | 1 | 365 | 376 |  |
| K.LALDLEIATYR.T | N | 64.98 | 1276.7026 | 11 | 0.6 | 639.3589 | 2 | 23.94 | 4 | F4:7990 | 05112019\_RID\_1313\_NaNaRj\_F4.raw |  |  |  | 2.97E5 |  | 1.1E6 | 2.57E5 | 5.86E5 |  |  | 7 | 0 | 0 | 0 | 2 | 0 | 2 | 2 | 1 | 0 | 0 | 473 | 483 |  |
| L.NVEIDPEIQK.V | N | 60.97 | 1183.6084 | 10 | 0.0 | 592.8115 | 2 | 11.69 | 5 | F5:2552 | 05112019\_RID\_1313\_NaNaRj\_F5.raw |  | 2.41E7 | 1.33E5 | 8.76E4 | 7.36E5 |  |  |  |  | 7.48E4 | 5 | 0 | 1 | 1 | 1 | 1 | 0 | 0 | 0 | 0 | 1 | 166 | 175 |  |
| R.DYQELMNTK.L | N | 60.25 | 1140.5121 | 9 | -0.2 | 571.2632 | 2 | 11.79 | 10 | F10:2283 | 05112019\_RID\_1313\_NaNaRj\_F10.raw |  | 1.15E7 | 8.45E4 |  | 1.1E5 |  |  |  |  | 3.39E4 | 6 | 0 | 3 | 1 | 0 | 1 | 0 | 0 | 0 | 0 | 1 | 464 | 472 |  |
| R.DYQELM(+15.99)NTK.L | N | 58.76 | 1156.5071 | 9 | 0.9 | 579.2614 | 2 | 22.92 | 2 | F2:5493 | 09012020\_NaNaRj\_F2.raw |  | 1.68E6 |  |  | 5.45E4 |  |  |  |  |  | 4 | 0 | 3 | 0 | 0 | 1 | 0 | 0 | 0 | 0 | 0 | 464 | 472 | Oxidation (M) |
| R.MSGEC(+57.02)APNVSVSVSTSHTTISGGGSR.G | Y | 58.56 | 2564.1594 | 26 | 1.1 | 855.7280 | 3 | 11.53 | 8 | F8:2340 | 05112019\_RID\_1313\_NaNaRj\_F8.raw |  |  |  |  |  |  |  | 0 |  |  | 1 | 0 | 0 | 0 | 0 | 0 | 0 | 0 | 1 | 0 | 0 | 493 | 518 | Carbamidomethylation |
| R.TLLEGEESR.M | N | 57.64 | 1032.5087 | 9 | 0.3 | 517.2618 | 2 | 11.27 | 5 | F5:2143 | 05112019\_RID\_1313\_NaNaRj\_F5.raw |  | 9.96E6 | 2.21E5 |  | 1.1E6 |  |  |  |  | 1.35E5 | 4 | 0 | 1 | 1 | 0 | 1 | 0 | 0 | 0 | 0 | 1 | 484 | 492 |  |
| R.SLVNLGGSK.S | N | 55.77 | 873.4919 | 9 | -0.4 | 437.7531 | 2 | 11.62 | 5 | F5:2477 | 05112019\_RID\_1313\_NaNaRj\_F5.raw |  | 1.13E7 | 1.69E5 |  | 4.78E5 |  |  |  |  | 9.55E4 | 5 | 0 | 2 | 1 | 0 | 1 | 0 | 0 | 0 | 0 | 1 | 66 | 74 |  |
| R.MSGEC(+57.02)APNVSV.S | N | 54.83 | 1149.4795 | 11 | -0.1 | 575.7469 | 2 | 41.70 | 2 | F2:13034 | 09012020\_NaNaRj\_F2.raw |  | 3.82E5 |  |  |  |  |  |  |  |  | 1 | 0 | 1 | 0 | 0 | 0 | 0 | 0 | 0 | 0 | 0 | 493 | 503 | Carbamidomethylation |
| K.NMQDMVEDYR.N | Y | 53.74 | 1299.5223 | 10 | 0.7 | 650.7689 | 2 | 43.40 | 2 | F2:13769 | 09012020\_NaNaRj\_F2.raw |  | 5.5E5 |  |  |  |  |  |  |  |  | 1 | 0 | 1 | 0 | 0 | 0 | 0 | 0 | 0 | 0 | 0 | 258 | 267 |  |
| G.GGSFGAGGGFGSR.S | Y | 52.50 | 1112.4999 | 13 | 1.0 | 557.2578 | 2 | 28.78 | 2 | F2:7743 | 09012020\_NaNaRj\_F2.raw |  | 1.01E5 |  |  |  |  |  |  |  |  | 1 | 0 | 1 | 0 | 0 | 0 | 0 | 0 | 0 | 0 | 0 | 53 | 65 |  |
| Y.GSGGGSYGSGGGGGGHGSYGSGSSSGGYR.G | Y | 52.27 | 2408.9602 | 29 | 0.8 | 803.9946 | 3 | 12.49 | 2 | F2:2054 | 09012020\_NaNaRj\_F2.raw |  | 7.82E4 |  |  |  |  |  |  |  |  | 1 | 0 | 1 | 0 | 0 | 0 | 0 | 0 | 0 | 0 | 0 | 560 | 588 |  |
| K.NMQDM(+15.99)VEDYR.N | Y | 51.31 | 1315.5173 | 10 | 0.5 | 658.7663 | 2 | 11.46 | 5 | F5:2304 | 05112019\_RID\_1313\_NaNaRj\_F5.raw |  |  |  |  | 4.51E4 |  |  |  |  |  | 1 | 0 | 0 | 0 | 0 | 1 | 0 | 0 | 0 | 0 | 0 | 258 | 267 | Oxidation (M) |
| K.IEISELNR.V | N | 51.06 | 972.5240 | 8 | 1.0 | 487.2698 | 2 | 11.75 | 4 | F4:2563 | 05112019\_RID\_1313\_NaNaRj\_F4.raw | 1.58E5 | 3.49E7 | 2.83E5 | 1.86E5 | 1.66E6 |  | 1.21E5 |  | 6.34E4 | 1.22E5 | 8 | 1 | 1 | 1 | 1 | 1 | 0 | 1 | 0 | 1 | 1 | 396 | 403 |  |
| W.ELLQQVDTSTR.T | N | 48.95 | 1288.6622 | 11 | 0.4 | 645.3386 | 2 | 11.63 | 3 | F3:2405 | 05112019\_RID\_1313\_NaNaRj\_F3.raw |  |  | 1.96E4 |  |  |  |  |  |  |  | 1 | 0 | 0 | 1 | 0 | 0 | 0 | 0 | 0 | 0 | 0 | 213 | 223 |  |
| N.QSLLQPLNVEIDPEIQK.V | N | 48.20 | 1963.0625 | 17 | 0.0 | 655.3615 | 3 | 29.00 | 7 | F7:8298 | 05112019\_RID\_1313\_NaNaRj\_F7.raw |  |  |  | 2.17E4 |  |  | 9.77E3 |  |  |  | 2 | 0 | 0 | 0 | 1 | 0 | 0 | 1 | 0 | 0 | 0 | 159 | 175 |  |
| Q.FASFIDK.V | N | 47.57 | 826.4225 | 7 | 0.0 | 414.2185 | 2 | 39.16 | 2 | F2:12055 | 09012020\_NaNaRj\_F2.raw | 6.35E3 | 4.99E6 |  |  | 4.92E5 |  |  |  | 5.38E3 |  | 4 | 1 | 1 | 0 | 0 | 1 | 0 | 0 | 0 | 1 | 0 | 191 | 197 |  |
| L.ALDLEIATYR.T | N | 47.50 | 1163.6187 | 10 | -0.1 | 582.8165 | 2 | 56.73 | 2 | F2:18887 | 09012020\_NaNaRj\_F2.raw |  | 2.31E5 |  |  |  |  |  |  |  |  | 1 | 0 | 1 | 0 | 0 | 0 | 0 | 0 | 0 | 0 | 0 | 474 | 483 |  |
| C.GGGGGSFGAGGGFGSR.S | Y | 45.61 | 1283.5642 | 16 | 0.6 | 642.7898 | 2 | 29.23 | 2 | F2:8097 | 09012020\_NaNaRj\_F2.raw |  | 8.58E5 |  |  |  |  |  |  |  |  | 1 | 0 | 1 | 0 | 0 | 0 | 0 | 0 | 0 | 0 | 0 | 50 | 65 |  |
| S.LLQPLNVEIDPEIQK.V | N | 45.59 | 1747.9719 | 15 | -0.8 | 874.9926 | 2 | 23.64 | 5 | F5:5523 | 05112019\_RID\_1313\_NaNaRj\_F5.raw |  |  |  |  | 1.17E4 |  |  |  |  |  | 1 | 0 | 0 | 0 | 0 | 1 | 0 | 0 | 0 | 0 | 0 | 161 | 175 |  |
| F.SSGSAGIINYQR.R | Y | 44.42 | 1251.6207 | 12 | 0.2 | 626.8177 | 2 | 11.44 | 5 | F5:2299 | 05112019\_RID\_1313\_NaNaRj\_F5.raw |  |  |  |  | 1.9E4 |  |  |  |  |  | 1 | 0 | 0 | 0 | 0 | 1 | 0 | 0 | 0 | 0 | 0 | 18 | 29 |  |
| K.NM(+15.99)QDM(+15.99)VEDYR.N | Y | 43.97 | 1331.5122 | 10 | 0.3 | 666.7635 | 2 | 11.02 | 5 | F5:1896 | 05112019\_RID\_1313\_NaNaRj\_F5.raw |  |  |  |  | 6.17E4 |  |  |  |  |  | 1 | 0 | 0 | 0 | 0 | 1 | 0 | 0 | 0 | 0 | 0 | 258 | 267 | Oxidation (M) |
| K.DVDGAYM(+15.99)TK.V | Y | 41.00 | 1014.4328 | 9 | 0.9 | 508.2241 | 2 | 25.00 | 2 | F2:6225 | 09012020\_NaNaRj\_F2.raw |  | 3.33E5 |  |  |  |  |  |  |  |  | 1 | 0 | 1 | 0 | 0 | 0 | 0 | 0 | 0 | 0 | 0 | 290 | 298 | Oxidation (M) |
| total 42 peptides |  |  |  |  |  |  |  |  |  |  |  |  |  |  |  |  |  |  |  |  |  |  |  |  |  |  |  |  |  |  |  |  |  |  |  |
| --- | --- | --- | --- | --- | --- | --- | --- | --- | --- | --- | --- | --- | --- | --- | --- | --- | --- | --- | --- | --- | --- | --- | --- | --- | --- | --- | --- | --- | --- | --- | --- | --- | --- | --- | --- |

P35908|K22E\_HUMAN

back to list

  

| Protein Coverage
| Supporting Peptides
|

Protein Coverage:

Supporting Peptides:

| Peptide | Uniq | -10lgP | Mass | Length | ppm | m/z | z | RT | Fraction | Scan | Source File | Area F1 | Area F2 | Area F3 | Area F4 | Area F5 | Area F6 | Area F7 | Area F8 | Area F9 | Area F10 | #Feature | #Feature F1 | #Feature F2 | #Feature F3 | #Feature F4 | #Feature F5 | #Feature F6 | #Feature F7 | #Feature F8 | #Feature F9 | #Feature F10 | Start | End | PTM |
| --- | --- | --- | --- | --- | --- | --- | --- | --- | --- | --- | --- | --- | --- | --- | --- | --- | --- | --- | --- | --- | --- | --- | --- | --- | --- | --- | --- | --- | --- | --- | --- | --- | --- | --- | --- |
| R.GSSSGGGYSSGSSSYGSGGR.Q | Y | 98.87 | 1739.6982 | 20 | 0.7 | 870.8570 | 2 | 12.49 | 2 | F2:2053 | 09012020\_NaNaRj\_F2.raw |  | 1.19E5 |  |  | 4.86E4 |  |  |  |  | 8.02E3 | 3 | 0 | 1 | 0 | 0 | 1 | 0 | 0 | 0 | 0 | 1 | 525 | 544 |  |
| R.GGSGGGGSISGGGYGSGGGSGGR.Y | Y | 98.58 | 1740.7411 | 23 | 0.8 | 871.3785 | 2 | 13.08 | 2 | F2:2242 | 09012020\_NaNaRj\_F2.raw |  | 2.68E5 | 3.97E4 |  | 7.56E4 |  |  |  |  | 8.85E3 | 4 | 0 | 1 | 1 | 0 | 1 | 0 | 0 | 0 | 0 | 1 | 550 | 572 |  |
| R.GFSSGSAVVSGGSR.R | N | 97.96 | 1253.6000 | 14 | 0.0 | 627.8073 | 2 | 11.19 | 5 | F5:2046 | 05112019\_RID\_1313\_NaNaRj\_F5.raw | 9.41E3 | 2.28E6 | 1.39E5 |  | 7.3E5 |  |  |  |  |  | 7 | 1 | 4 | 1 | 0 | 1 | 0 | 0 | 0 | 0 | 0 | 21 | 34 |  |
| K.NVQDAIADAEQR.G | Y | 91.75 | 1328.6321 | 12 | 0.3 | 665.3235 | 2 | 11.69 | 5 | F5:2546 | 05112019\_RID\_1313\_NaNaRj\_F5.raw |  | 6.18E5 | 3.59E4 |  | 5.62E5 |  |  |  |  |  | 3 | 0 | 1 | 1 | 0 | 1 | 0 | 0 | 0 | 0 | 0 | 419 | 430 |  |
| K.LNDLEEALQQAK.E | Y | 89.70 | 1370.7041 | 12 | 1.2 | 686.3602 | 2 | 13.68 | 6 | F6:3928 | 05112019\_RID\_1313\_NaNaRj\_F6.raw | 6.61E4 | 1.77E6 | 3.98E4 | 3.28E5 | 2.23E6 | 0 | 1.96E5 |  |  |  | 7 | 1 | 1 | 1 | 1 | 1 | 1 | 1 | 0 | 0 | 0 | 442 | 453 |  |
| R.HGGGGGGFGGGGFGSR.S | Y | 89.34 | 1319.5754 | 16 | 0.8 | 440.8661 | 3 | 11.02 | 5 | F5:1894 | 05112019\_RID\_1313\_NaNaRj\_F5.raw |  | 1.43E6 |  |  | 8.4E4 |  |  |  |  |  | 4 | 0 | 3 | 0 | 0 | 1 | 0 | 0 | 0 | 0 | 0 | 46 | 61 |  |
| R.FLEQQNQVLQTK.W | N | 88.32 | 1474.7780 | 12 | 0.9 | 738.3969 | 2 | 11.66 | 3 | F3:2423 | 05112019\_RID\_1313\_NaNaRj\_F3.raw | 3.29E5 | 6.5E6 | 2.66E6 | 9.27E5 | 3.5E6 |  | 4.3E5 | 1.41E5 | 1.56E5 | 4.01E5 | 11 | 1 | 2 | 1 | 1 | 2 | 0 | 1 | 1 | 1 | 1 | 198 | 209 |  |
| R.SKEEAEALYHSK.Y | Y | 83.99 | 1390.6729 | 12 | -0.4 | 464.5647 | 3 | 10.70 | 5 | F5:1586 | 05112019\_RID\_1313\_NaNaRj\_F5.raw |  | 1.07E5 |  |  | 4.57E5 |  |  |  |  |  | 2 | 0 | 1 | 0 | 0 | 1 | 0 | 0 | 0 | 0 | 0 | 363 | 374 |  |
| R.TAAENDFVTLK.K | N | 82.86 | 1207.6084 | 11 | 1.1 | 604.8121 | 2 | 11.96 | 5 | F5:2787 | 05112019\_RID\_1313\_NaNaRj\_F5.raw | 4.39E4 |  | 1.35E5 | 7.28E4 | 1.59E6 | 7.25E4 | 5.3E4 |  |  | 3.84E4 | 7 | 1 | 0 | 1 | 1 | 1 | 1 | 1 | 0 | 0 | 1 | 276 | 286 |  |
| A.GGGGGFGAAGGFGGR.G | Y | 81.59 | 1180.5374 | 15 | 0.5 | 591.2762 | 2 | 33.30 | 2 | F2:9557 | 09012020\_NaNaRj\_F2.raw |  | 8.19E5 |  |  |  |  |  |  |  |  | 1 | 0 | 1 | 0 | 0 | 0 | 0 | 0 | 0 | 0 | 0 | 78 | 92 |  |
| S.VAGGGGGFGAAGGFGGR.G | Y | 80.08 | 1350.6428 | 17 | 0.8 | 676.3292 | 2 | 11.73 | 5 | F5:2591 | 05112019\_RID\_1313\_NaNaRj\_F5.raw |  |  | 2.01E4 |  | 4.16E4 |  |  |  |  |  | 2 | 0 | 0 | 1 | 0 | 1 | 0 | 0 | 0 | 0 | 0 | 76 | 92 |  |
| R.NLDLDSIIAEVK.A | N | 78.64 | 1328.7188 | 12 | 0.5 | 665.3670 | 2 | 35.93 | 7 | F7:9727 | 05112019\_RID\_1313\_NaNaRj\_F7.raw |  |  |  | 6.48E5 | 5.9E4 | 9.04E5 | 6.22E5 | 3.5E5 |  |  | 5 | 0 | 0 | 0 | 1 | 1 | 1 | 1 | 1 | 0 | 0 | 342 | 353 |  |
| K.YEELQVTVGR.H | Y | 76.81 | 1192.6088 | 10 | 0.4 | 597.3119 | 2 | 40.02 | 2 | F2:12397 | 09012020\_NaNaRj\_F2.raw |  | 1.54E6 |  | 5.87E4 |  |  |  |  |  |  | 2 | 0 | 1 | 0 | 1 | 0 | 0 | 0 | 0 | 0 | 0 | 375 | 384 |  |
| K.AQYEEIAQR.S | N | 74.78 | 1106.5356 | 9 | -0.4 | 554.2749 | 2 | 11.11 | 3 | F3:1935 | 05112019\_RID\_1313\_NaNaRj\_F3.raw | 2.4E4 | 2.26E6 | 2.18E5 |  | 9.57E5 |  |  |  |  | 4.57E4 | 6 | 1 | 2 | 1 | 0 | 1 | 0 | 0 | 0 | 0 | 1 | 354 | 362 |  |
| I.SVAGGGGGFGAAGGFGGR.G | Y | 70.53 | 1437.6748 | 18 | 1.1 | 719.8455 | 2 | 39.15 | 2 | F2:12082 | 09012020\_NaNaRj\_F2.raw |  | 6.83E4 |  |  |  |  |  |  |  |  | 1 | 0 | 1 | 0 | 0 | 0 | 0 | 0 | 0 | 0 | 0 | 75 | 92 |  |
| K.LALDVEIATYR.K | N | 67.43 | 1262.6870 | 11 | 0.5 | 632.3511 | 2 | 18.91 | 6 | F6:7379 | 05112019\_RID\_1313\_NaNaRj\_F6.raw |  |  |  | 8.76E4 |  | 3.23E5 | 1.04E5 |  |  |  | 4 | 0 | 0 | 0 | 1 | 0 | 2 | 1 | 0 | 0 | 0 | 471 | 481 |  |
| V.AGGGGGFGAAGGFGGR.G | Y | 66.81 | 1251.5745 | 16 | -0.1 | 626.7944 | 2 | 33.80 | 2 | F2:9768 | 09012020\_NaNaRj\_F2.raw |  | 4.79E4 |  |  |  |  |  |  |  |  | 1 | 0 | 1 | 0 | 0 | 0 | 0 | 0 | 0 | 0 | 0 | 77 | 92 |  |
| R.TAAENDFVTLKK.D | N | 66.51 | 1335.7034 | 12 | -0.3 | 446.2416 | 3 | 11.32 | 3 | F3:2144 | 05112019\_RID\_1313\_NaNaRj\_F3.raw |  |  | 7.72E4 |  |  |  |  |  |  |  | 2 | 0 | 0 | 2 | 0 | 0 | 0 | 0 | 0 | 0 | 0 | 276 | 287 |  |
| R.STSSFSC(+57.02)LSR.H | Y | 64.30 | 1130.5026 | 10 | 0.5 | 566.2589 | 2 | 11.42 | 5 | F5:2259 | 05112019\_RID\_1313\_NaNaRj\_F5.raw |  |  | 6.77E4 |  | 5.54E5 |  |  |  |  | 1.05E4 | 3 | 0 | 0 | 1 | 0 | 1 | 0 | 0 | 0 | 0 | 1 | 36 | 45 | Carbamidomethylation |
| K.VDLLNQEIEFLK.V | Y | 64.07 | 1459.7922 | 12 | 0.5 | 730.9037 | 2 | 33.43 | 6 | F6:14758 | 05112019\_RID\_1313\_NaNaRj\_F6.raw |  |  | 9.89E3 | 3.69E5 |  | 6.68E5 | 4.01E5 | 2.29E5 |  |  | 5 | 0 | 0 | 1 | 1 | 0 | 1 | 1 | 1 | 0 | 0 | 303 | 314 |  |
| R.FGGFGGPGGVGGL.G | Y | 63.25 | 1077.5243 | 13 | 1.4 | 539.7702 | 2 | 73.40 | 2 | F2:23699 | 09012020\_NaNaRj\_F2.raw |  | 1.31E5 |  |  |  |  |  |  |  |  | 1 | 0 | 1 | 0 | 0 | 0 | 0 | 0 | 0 | 0 | 0 | 123 | 135 |  |
| K.VDPEIQNVK.A | N | 59.85 | 1040.5502 | 9 | -0.3 | 521.2822 | 2 | 24.00 | 2 | F2:5835 | 09012020\_NaNaRj\_F2.raw |  | 1.46E6 | 1.15E5 |  | 8E5 |  |  |  |  |  | 3 | 0 | 1 | 1 | 0 | 1 | 0 | 0 | 0 | 0 | 0 | 167 | 175 |  |
| K.VLYDAEISQIHQ.S | Y | 59.33 | 1414.7092 | 12 | 0.9 | 708.3625 | 2 | 12.55 | 5 | F5:3205 | 05112019\_RID\_1313\_NaNaRj\_F5.raw |  | 5.87E5 |  |  | 3.33E4 |  |  |  |  |  | 2 | 0 | 1 | 0 | 0 | 1 | 0 | 0 | 0 | 0 | 0 | 315 | 326 |  |
| R.YLDGLTAER.T | Y | 58.90 | 1036.5189 | 9 | 0.0 | 519.2667 | 2 | 11.82 | 5 | F5:2666 | 05112019\_RID\_1313\_NaNaRj\_F5.raw |  |  |  |  | 8.33E5 |  |  |  |  |  | 1 | 0 | 0 | 0 | 0 | 1 | 0 | 0 | 0 | 0 | 0 | 239 | 247 |  |
| K.VLYDAEISQIHQS.V | Y | 58.89 | 1501.7412 | 13 | 0.4 | 751.8782 | 2 | 12.20 | 5 | F5:3014 | 05112019\_RID\_1313\_NaNaRj\_F5.raw |  |  |  |  | 3.11E4 |  |  |  |  |  | 1 | 0 | 0 | 0 | 0 | 1 | 0 | 0 | 0 | 0 | 0 | 315 | 327 |  |
| R.SLVGLGGTK.S | N | 57.64 | 830.4861 | 9 | 0.5 | 416.2505 | 2 | 11.61 | 5 | F5:2459 | 05112019\_RID\_1313\_NaNaRj\_F5.raw |  | 1.45E6 | 0 |  | 0 |  |  |  |  | 0 | 4 | 0 | 1 | 1 | 0 | 1 | 0 | 0 | 0 | 0 | 1 | 62 | 70 |  |
| K.DVDNAYMIK.V | Y | 56.43 | 1067.4957 | 9 | -0.7 | 534.7548 | 2 | 11.89 | 3 | F3:2615 | 05112019\_RID\_1313\_NaNaRj\_F3.raw |  |  | 1.46E5 |  | 7.67E4 |  |  |  |  |  | 2 | 0 | 0 | 1 | 0 | 1 | 0 | 0 | 0 | 0 | 0 | 288 | 296 |  |
| R.DYQELMNVK.L | N | 56.23 | 1138.5328 | 9 | 0.4 | 570.2739 | 2 | 12.38 | 3 | F3:2842 | 05112019\_RID\_1313\_NaNaRj\_F3.raw |  |  | 3.71E4 |  | 1.59E5 |  |  |  |  | 2.62E3 | 3 | 0 | 0 | 1 | 0 | 1 | 0 | 0 | 0 | 0 | 1 | 462 | 470 |  |
| K.LLEGEEC(+57.02)R.M | N | 54.31 | 1004.4597 | 8 | -0.6 | 503.2368 | 2 | 11.02 | 5 | F5:1874 | 05112019\_RID\_1313\_NaNaRj\_F5.raw |  |  |  |  | 1.16E5 |  |  |  |  |  | 1 | 0 | 0 | 0 | 0 | 1 | 0 | 0 | 0 | 0 | 0 | 483 | 490 | Carbamidomethylation |
| K.DVDNAYM(+15.99)IK.V | Y | 51.15 | 1083.4906 | 9 | 0.8 | 542.7530 | 2 | 11.52 | 5 | F5:2397 | 05112019\_RID\_1313\_NaNaRj\_F5.raw |  |  |  |  | 4.36E4 |  |  |  |  |  | 1 | 0 | 0 | 0 | 0 | 1 | 0 | 0 | 0 | 0 | 0 | 288 | 296 | Oxidation (M) |
| K.IEISELNR.V | N | 51.06 | 972.5240 | 8 | 1.0 | 487.2698 | 2 | 11.75 | 4 | F4:2563 | 05112019\_RID\_1313\_NaNaRj\_F4.raw | 1.58E5 | 3.49E7 | 2.83E5 | 1.86E5 | 1.66E6 |  | 1.21E5 |  | 6.34E4 | 1.22E5 | 8 | 1 | 1 | 1 | 1 | 1 | 0 | 1 | 0 | 1 | 1 | 394 | 401 |  |
| R.GGGFGGGSSFGGGSGF.S | Y | 50.72 | 1290.5265 | 16 | 0.7 | 646.2710 | 2 | 60.92 | 2 | F2:20178 | 09012020\_NaNaRj\_F2.raw |  | 1.48E5 |  |  |  |  |  |  |  |  | 1 | 0 | 1 | 0 | 0 | 0 | 0 | 0 | 0 | 0 | 0 | 93 | 108 |  |
| R.FGGFGGPGGVGGLGGPGGFGPGGYPGGIHEVSVNQSLLQPLNVK.V | Y | 50.54 | 4091.0652 | 44 | 0.0 | 1023.7736 | 4 | 39.48 | 6 | F6:17168 | 05112019\_RID\_1313\_NaNaRj\_F6.raw |  |  |  |  |  | 1.92E5 |  |  |  |  | 1 | 0 | 0 | 0 | 0 | 0 | 1 | 0 | 0 | 0 | 0 | 123 | 166 |  |
| F.SGGGFGGGGFGGGR.F | Y | 50.54 | 1125.4951 | 14 | 0.8 | 563.7553 | 2 | 11.46 | 5 | F5:2332 | 05112019\_RID\_1313\_NaNaRj\_F5.raw |  |  |  |  | 2.48E5 |  |  |  |  |  | 1 | 0 | 0 | 0 | 0 | 1 | 0 | 0 | 0 | 0 | 0 | 109 | 122 |  |
| R.GGGFGGGSSFGGGSGFSGGGFGGGGFGGGR.F | Y | 49.84 | 2398.0110 | 30 | -1.3 | 800.3433 | 3 | 18.60 | 6 | F6:7242 | 05112019\_RID\_1313\_NaNaRj\_F6.raw |  |  |  |  |  | 3.64E4 |  | 1.76E4 |  |  | 2 | 0 | 0 | 0 | 0 | 0 | 1 | 0 | 1 | 0 | 0 | 93 | 122 |  |
| R.M(+15.99)SGDLSSNVTVSVTSSTISSNVASK.A | Y | 48.19 | 2473.1853 | 25 | 0.3 | 825.4026 | 3 | 12.51 | 3 | F3:2888 | 05112019\_RID\_1313\_NaNaRj\_F3.raw |  |  | 8.35E3 |  |  |  |  |  |  |  | 1 | 0 | 0 | 1 | 0 | 0 | 0 | 0 | 0 | 0 | 0 | 491 | 515 | Oxidation (M) |
| K.FASFIDK.V | N | 47.57 | 826.4225 | 7 | 0.0 | 414.2185 | 2 | 39.16 | 2 | F2:12055 | 09012020\_NaNaRj\_F2.raw | 6.35E3 | 4.99E6 |  |  | 4.92E5 |  |  |  | 5.38E3 |  | 4 | 1 | 1 | 0 | 0 | 1 | 0 | 0 | 0 | 1 | 0 | 189 | 195 |  |
| S.VTSSTISSNVASK.A | Y | 47.36 | 1279.6620 | 13 | 0.7 | 640.8387 | 2 | 18.70 | 2 | F2:3685 | 09012020\_NaNaRj\_F2.raw |  | 3.23E4 |  |  |  |  |  |  |  |  | 1 | 0 | 1 | 0 | 0 | 0 | 0 | 0 | 0 | 0 | 0 | 503 | 515 |  |
| V.SVNQSLLQPLNVK.V | Y | 45.28 | 1438.8143 | 13 | 0.7 | 720.4150 | 2 | 14.09 | 3 | F3:3184 | 05112019\_RID\_1313\_NaNaRj\_F3.raw |  |  | 1.8E4 |  |  |  |  |  |  |  | 1 | 0 | 0 | 1 | 0 | 0 | 0 | 0 | 0 | 0 | 0 | 154 | 166 |  |
| N.VGTRPINLEPIFQGYIDSLK.R | Y | 44.88 | 2259.2263 | 20 | 0.1 | 754.0828 | 3 | 37.12 | 6 | F6:16305 | 05112019\_RID\_1313\_NaNaRj\_F6.raw |  |  |  |  |  | 1.63E5 |  |  |  |  | 1 | 0 | 0 | 0 | 0 | 0 | 1 | 0 | 0 | 0 | 0 | 218 | 237 |  |
| R.DYQELM(+15.99)NVK.L | N | 42.67 | 1154.5277 | 9 | 0.4 | 578.2714 | 2 | 11.50 | 5 | F5:2339 | 05112019\_RID\_1313\_NaNaRj\_F5.raw |  | 0 |  |  | 9.19E4 |  |  |  |  |  | 2 | 0 | 1 | 0 | 0 | 1 | 0 | 0 | 0 | 0 | 0 | 462 | 470 | Oxidation (M) |
| L.NVKVDPEIQNVK.A | N | 41.39 | 1381.7565 | 12 | 0.2 | 691.8856 | 2 | 25.13 | 2 | F2:6303 | 09012020\_NaNaRj\_F2.raw |  | 3.23E4 |  |  |  |  |  |  |  |  | 1 | 0 | 1 | 0 | 0 | 0 | 0 | 0 | 0 | 0 | 0 | 164 | 175 |  |
| N.QSLLQPLNVK.V | Y | 41.38 | 1138.6710 | 10 | 0.0 | 570.3428 | 2 | 13.20 | 5 | F5:3423 | 05112019\_RID\_1313\_NaNaRj\_F5.raw |  |  |  |  | 1.3E5 |  |  |  |  |  | 1 | 0 | 0 | 0 | 0 | 1 | 0 | 0 | 0 | 0 | 0 | 157 | 166 |  |
| G.FSSGSAVVSGGSR.R | N | 40.32 | 1196.5785 | 13 | 1.5 | 599.2974 | 2 | 20.30 | 2 | F2:4363 | 09012020\_NaNaRj\_F2.raw |  | 1.05E4 |  |  |  |  |  |  |  |  | 1 | 0 | 1 | 0 | 0 | 0 | 0 | 0 | 0 | 0 | 0 | 22 | 34 |  |
| total 44 peptides |  |  |  |  |  |  |  |  |  |  |  |  |  |  |  |  |  |  |  |  |  |  |  |  |  |  |  |  |  |  |  |  |  |  |  |
| --- | --- | --- | --- | --- | --- | --- | --- | --- | --- | --- | --- | --- | --- | --- | --- | --- | --- | --- | --- | --- | --- | --- | --- | --- | --- | --- | --- | --- | --- | --- | --- | --- | --- | --- | --- |

P82885|VESP\_NAJKA

back to list

  

| Protein Coverage
| Supporting Peptides
|

Protein Coverage:

Supporting Peptides:

| Peptide | Uniq | -10lgP | Mass | Length | ppm | m/z | z | RT | Fraction | Scan | Source File | Area F1 | Area F2 | Area F3 | Area F4 | Area F5 | Area F6 | Area F7 | Area F8 | Area F9 | Area F10 | #Feature | #Feature F1 | #Feature F2 | #Feature F3 | #Feature F4 | #Feature F5 | #Feature F6 | #Feature F7 | #Feature F8 | #Feature F9 | #Feature F10 | Start | End | PTM |
| --- | --- | --- | --- | --- | --- | --- | --- | --- | --- | --- | --- | --- | --- | --- | --- | --- | --- | --- | --- | --- | --- | --- | --- | --- | --- | --- | --- | --- | --- | --- | --- | --- | --- | --- | --- |
| K.TVENVGVSQVAPDNPER.F | Y | 119.43 | 1809.8856 | 17 | -0.4 | 905.9497 | 2 | 11.54 | 8 | F8:2345 | 05112019\_RID\_1313\_NaNaRj\_F8.raw | 0 |  |  |  |  |  |  | 1.41E6 | 2.32E7 | 3.29E4 | 10 | 1 | 0 | 0 | 0 | 0 | 0 | 0 | 2 | 6 | 1 | 30 | 46 |  |
| K.ADVTFDSNTAFESLVVSPDKK.T | N | 109.82 | 2269.1113 | 21 | 0.9 | 757.3784 | 3 | 20.22 | 9 | F9:4660 | 05112019\_RID\_1313\_NaNaRj\_F9.raw |  |  |  |  |  | 7.55E4 |  | 2.78E6 | 1.05E7 |  | 10 | 0 | 0 | 0 | 0 | 0 | 1 | 0 | 4 | 5 | 0 | 9 | 29 |  |
| R.FDGSPC(+57.02)VLGSPGFR.S | Y | 104.48 | 1494.6925 | 14 | 0.1 | 748.3536 | 2 | 14.12 | 9 | F9:3252 | 05112019\_RID\_1313\_NaNaRj\_F9.raw |  |  |  | 1.58E5 |  | 2.18E5 | 5.67E4 | 1.33E6 | 9.17E6 |  | 8 | 0 | 0 | 0 | 1 | 0 | 2 | 1 | 1 | 3 | 0 | 47 | 60 | Carbamidomethylation |
| K.ADVTFDSNTAFESLVVSPDK.K | N | 90.02 | 2141.0164 | 20 | 0.2 | 1071.5156 | 2 | 30.21 | 9 | F9:6348 | 05112019\_RID\_1313\_NaNaRj\_F9.raw |  |  |  |  |  |  |  | 9.02E5 | 2.32E6 |  | 5 | 0 | 0 | 0 | 0 | 0 | 0 | 0 | 1 | 4 | 0 | 9 | 28 |  |
| D.SNTAFESLVVSPDK.K | N | 67.93 | 1492.7408 | 14 | 0.9 | 747.3784 | 2 | 14.36 | 9 | F9:3328 | 05112019\_RID\_1313\_NaNaRj\_F9.raw |  |  |  |  |  |  |  |  | 4.18E4 |  | 1 | 0 | 0 | 0 | 0 | 0 | 0 | 0 | 0 | 1 | 0 | 15 | 28 |  |
| N.VGVSQVAPDNPER.F | Y | 66.77 | 1366.6841 | 13 | 0.2 | 684.3495 | 2 | 11.26 | 9 | F9:1983 | 05112019\_RID\_1313\_NaNaRj\_F9.raw |  |  |  |  |  |  |  |  | 1.04E5 |  | 1 | 0 | 0 | 0 | 0 | 0 | 0 | 0 | 0 | 1 | 0 | 34 | 46 |  |
| N.TAFESLVVSPDKK.T | N | 65.30 | 1419.7609 | 13 | 0.4 | 710.8880 | 2 | 11.79 | 9 | F9:2460 | 05112019\_RID\_1313\_NaNaRj\_F9.raw |  |  |  |  |  |  |  |  | 1.11E5 |  | 2 | 0 | 0 | 0 | 0 | 0 | 0 | 0 | 0 | 2 | 0 | 17 | 29 |  |
| R.EWAVGLAGK.S | N | 62.41 | 929.4970 | 9 | 0.5 | 465.7560 | 2 | 12.16 | 9 | F9:2726 | 05112019\_RID\_1313\_NaNaRj\_F9.raw |  |  |  |  |  |  |  |  | 9.28E6 |  | 3 | 0 | 0 | 0 | 0 | 0 | 0 | 0 | 0 | 3 | 0 | 75 | 83 |  |
| T.AFESLVVSPDK.K | N | 57.37 | 1190.6183 | 11 | -0.3 | 596.3162 | 2 | 12.36 | 9 | F9:2859 | 05112019\_RID\_1313\_NaNaRj\_F9.raw |  |  |  |  |  |  |  |  | 6.47E4 |  | 1 | 0 | 0 | 0 | 0 | 0 | 0 | 0 | 0 | 1 | 0 | 18 | 28 |  |
| Q.KADVTFDSNTAFESLVVSPDK.K | N | 53.23 | 2269.1113 | 21 | 0.1 | 757.3778 | 3 | 19.33 | 9 | F9:4389 | 05112019\_RID\_1313\_NaNaRj\_F9.raw |  |  |  |  |  |  |  | 1.75E4 | 1.94E5 |  | 3 | 0 | 0 | 0 | 0 | 0 | 0 | 0 | 1 | 2 | 0 | 8 | 28 |  |
| K.TVENVGVSQVAP.D | Y | 52.85 | 1198.6194 | 12 | 0.4 | 600.3172 | 2 | 12.32 | 3 | F3:2817 | 05112019\_RID\_1313\_NaNaRj\_F3.raw |  |  | 3.22E5 |  |  |  |  |  |  |  | 1 | 0 | 0 | 1 | 0 | 0 | 0 | 0 | 0 | 0 | 0 | 30 | 41 |  |
| SPPGNWQK.A | N | 52.39 | 912.4453 | 8 | 0.6 | 457.2302 | 2 | 10.87 | 9 | F9:1676 | 05112019\_RID\_1313\_NaNaRj\_F9.raw |  |  |  |  |  |  |  |  | 1.15E5 |  | 1 | 0 | 0 | 0 | 0 | 0 | 0 | 0 | 0 | 1 | 0 | 1 | 8 |  |
| N.TAFESLVVSPDK.K | N | 47.26 | 1291.6659 | 12 | 0.8 | 646.8408 | 2 | 13.82 | 9 | F9:3188 | 05112019\_RID\_1313\_NaNaRj\_F9.raw |  |  |  |  |  |  |  |  | 9.65E4 |  | 1 | 0 | 0 | 0 | 0 | 0 | 0 | 0 | 0 | 1 | 0 | 17 | 28 |  |
| F.ESLVVSPDK.K | N | 44.68 | 972.5128 | 9 | 0.2 | 487.2638 | 2 | 11.44 | 1 | F1:2215 | 05112019\_RID\_1313\_NaNaRj\_F1.raw | 2.21E4 |  |  |  |  |  |  |  |  |  | 1 | 1 | 0 | 0 | 0 | 0 | 0 | 0 | 0 | 0 | 0 | 20 | 28 |  |
| C.VLGSPGFR.S | N | 44.13 | 831.4603 | 8 | -0.9 | 416.7370 | 2 | 11.52 | 9 | F9:2203 | 05112019\_RID\_1313\_NaNaRj\_F9.raw |  |  |  |  |  |  |  |  | 2.03E6 |  | 1 | 0 | 0 | 0 | 0 | 0 | 0 | 0 | 0 | 1 | 0 | 53 | 60 |  |
| T.AFESLVVSPDKK.T | N | 42.32 | 1318.7133 | 12 | 0.2 | 660.3640 | 2 | 11.61 | 9 | F9:2325 | 05112019\_RID\_1313\_NaNaRj\_F9.raw |  |  |  |  |  |  |  |  | 4.9E3 |  | 1 | 0 | 0 | 0 | 0 | 0 | 0 | 0 | 0 | 1 | 0 | 18 | 29 |  |
| F.DGSPC(+57.02)VLGSPGFR.S | Y | 40.89 | 1347.6241 | 13 | 0.2 | 674.8195 | 2 | 12.46 | 9 | F9:2905 | 05112019\_RID\_1313\_NaNaRj\_F9.raw |  |  |  |  |  |  |  |  | 2.84E4 |  | 1 | 0 | 0 | 0 | 0 | 0 | 0 | 0 | 0 | 1 | 0 | 48 | 60 | Carbamidomethylation |
| total 17 peptides |  |  |  |  |  |  |  |  |  |  |  |  |  |  |  |  |  |  |  |  |  |  |  |  |  |  |  |  |  |  |  |  |  |  |  |
| --- | --- | --- | --- | --- | --- | --- | --- | --- | --- | --- | --- | --- | --- | --- | --- | --- | --- | --- | --- | --- | --- | --- | --- | --- | --- | --- | --- | --- | --- | --- | --- | --- | --- | --- | --- |

P01441|3SA2\_NAJOX

back to list

  

| Protein Coverage
| Supporting Peptides
|

Protein Coverage:

Supporting Peptides:

| Peptide | Uniq | -10lgP | Mass | Length | ppm | m/z | z | RT | Fraction | Scan | Source File | Area F1 | Area F2 | Area F3 | Area F4 | Area F5 | Area F6 | Area F7 | Area F8 | Area F9 | Area F10 | #Feature | #Feature F1 | #Feature F2 | #Feature F3 | #Feature F4 | #Feature F5 | #Feature F6 | #Feature F7 | #Feature F8 | #Feature F9 | #Feature F10 | Start | End | PTM |
| --- | --- | --- | --- | --- | --- | --- | --- | --- | --- | --- | --- | --- | --- | --- | --- | --- | --- | --- | --- | --- | --- | --- | --- | --- | --- | --- | --- | --- | --- | --- | --- | --- | --- | --- | --- |
| K.M(+15.99)FMVAAPHVPVK.R | Y | 104.27 | 1341.6937 | 12 | -0.8 | 671.8536 | 2 | 11.79 | 8 | F8:2583 | 05112019\_RID\_1313\_NaNaRj\_F8.raw |  |  |  |  |  | 5.36E6 |  | 5.11E7 |  |  | 4 | 0 | 0 | 0 | 0 | 0 | 2 | 0 | 2 | 0 | 0 | 24 | 35 | Oxidation (M) |
| K.SSLLVKYVC(+57.02)C(+57.02)NTDKC(+57.02)N | Y | 100.84 | 1959.8853 | 16 | -3.4 | 654.3002 | 3 | 11.71 | 8 | F8:2517 | 05112019\_RID\_1313\_NaNaRj\_F8.raw |  |  |  |  |  |  |  | 8.21E5 |  |  | 2 | 0 | 0 | 0 | 0 | 0 | 0 | 0 | 2 | 0 | 0 | 45 | 60 | Carbamidomethylation |
| K.MFMVAAPHVPVK.R | Y | 100.46 | 1325.6989 | 12 | 1.6 | 663.8578 | 2 | 12.11 | 8 | F8:2827 | 05112019\_RID\_1313\_NaNaRj\_F8.raw |  |  |  |  |  | 1.31E6 | 1.2E6 | 1.63E8 | 2.02E4 |  | 18 | 0 | 0 | 0 | 0 | 0 | 2 | 2 | 13 | 1 | 0 | 24 | 35 |  |
| R.GC(+57.02)IDVC(+57.02)PKSSLLVKYVC(+57.02)C(+57.02)NTDKC(+57.02)N | Y | 98.37 | 2889.2952 | 24 | 1.7 | 723.3323 | 4 | 12.12 | 8 | F8:2875 | 05112019\_RID\_1313\_NaNaRj\_F8.raw |  |  |  |  |  |  |  | 3.8E6 |  |  | 2 | 0 | 0 | 0 | 0 | 0 | 0 | 0 | 2 | 0 | 0 | 37 | 60 | Carbamidomethylation |
| K.MFM(+15.99)VAAPHVPVK.R | Y | 98.10 | 1341.6937 | 12 | -0.5 | 448.2383 | 3 | 11.82 | 7 | F7:2621 | 05112019\_RID\_1313\_NaNaRj\_F7.raw |  |  |  |  |  |  | 1.77E6 | 3.42E6 | 4.14E4 |  | 6 | 0 | 0 | 0 | 0 | 0 | 0 | 2 | 3 | 1 | 0 | 24 | 35 | Oxidation (M) |
| K.M(+15.99)FM(+15.99)VAAPHVPVK.R | Y | 81.59 | 1357.6886 | 12 | 0.0 | 453.5701 | 3 | 11.62 | 6 | F6:2407 | 05112019\_RID\_1313\_NaNaRj\_F6.raw |  |  |  |  |  | 9.4E5 | 3.78E5 | 9.65E6 | 5.11E4 |  | 10 | 0 | 0 | 0 | 0 | 0 | 1 | 1 | 6 | 2 | 0 | 24 | 35 | Oxidation (M) |
| K.SSLLVKYVC(+57.02)C(+57.02)NTDK.C | Y | 77.76 | 1685.8116 | 14 | -3.3 | 843.9103 | 2 | 11.71 | 8 | F8:2528 | 05112019\_RID\_1313\_NaNaRj\_F8.raw |  |  |  |  |  |  |  | 2.71E5 |  |  | 1 | 0 | 0 | 0 | 0 | 0 | 0 | 0 | 1 | 0 | 0 | 45 | 58 | Carbamidomethylation |
| M.FMVAAPHVPVK.R | Y | 74.94 | 1194.6583 | 11 | -2.7 | 598.3348 | 2 | 11.73 | 8 | F8:2536 | 05112019\_RID\_1313\_NaNaRj\_F8.raw |  |  |  |  |  |  |  | 4.91E6 |  |  | 2 | 0 | 0 | 0 | 0 | 0 | 0 | 0 | 2 | 0 | 0 | 25 | 35 |  |
| M.FM(+15.99)VAAPHVPVK.R | Y | 72.32 | 1210.6532 | 11 | 0.0 | 404.5583 | 3 | 11.59 | 8 | F8:2402 | 05112019\_RID\_1313\_NaNaRj\_F8.raw |  |  |  |  |  |  |  | 3.23E5 |  |  | 1 | 0 | 0 | 0 | 0 | 0 | 0 | 0 | 1 | 0 | 0 | 25 | 35 | Oxidation (M) |
| K.MFMVAAPHVPVKR.G | Y | 69.66 | 1481.7999 | 13 | 0.2 | 494.9407 | 3 | 11.62 | 8 | F8:2431 | 05112019\_RID\_1313\_NaNaRj\_F8.raw |  |  |  |  |  |  |  | 7.79E5 |  |  | 2 | 0 | 0 | 0 | 0 | 0 | 0 | 0 | 2 | 0 | 0 | 24 | 36 |  |
| K.NLC(+57.02)YKMFMVAAPHVPVKR.G | Y | 68.72 | 2160.1157 | 18 | 1.0 | 541.0367 | 4 | 12.00 | 8 | F8:2785 | 05112019\_RID\_1313\_NaNaRj\_F8.raw |  |  |  |  |  |  |  | 1.39E5 |  |  | 1 | 0 | 0 | 0 | 0 | 0 | 0 | 0 | 1 | 0 | 0 | 19 | 36 | Carbamidomethylation |
| R.GC(+57.02)IDVC(+57.02)PKSSLLVK.Y | N | 68.52 | 1574.8160 | 14 | -3.9 | 788.4122 | 2 | 11.71 | 8 | F8:2538 | 05112019\_RID\_1313\_NaNaRj\_F8.raw |  |  |  |  |  |  | 0 | 3.04E6 |  |  | 3 | 0 | 0 | 0 | 0 | 0 | 0 | 1 | 2 | 0 | 0 | 37 | 50 | Carbamidomethylation |
| F.MVAAPHVPVK.R | Y | 67.93 | 1047.5898 | 10 | -0.5 | 524.8019 | 2 | 11.22 | 8 | F8:2059 | 05112019\_RID\_1313\_NaNaRj\_F8.raw |  |  |  |  |  |  |  | 7.66E6 |  |  | 2 | 0 | 0 | 0 | 0 | 0 | 0 | 0 | 2 | 0 | 0 | 26 | 35 |  |
| K.MFMVAAPHVPVKRG.C | Y | 64.23 | 1538.8214 | 14 | -0.8 | 513.9473 | 3 | 11.62 | 8 | F8:2429 | 05112019\_RID\_1313\_NaNaRj\_F8.raw |  |  |  |  |  |  |  | 8.37E5 |  |  | 2 | 0 | 0 | 0 | 0 | 0 | 0 | 0 | 2 | 0 | 0 | 24 | 37 |  |
| M.VAAPHVPVK.R | Y | 62.64 | 916.5494 | 9 | 0.6 | 459.2822 | 2 | 11.22 | 8 | F8:2537 | 05112019\_RID\_1313\_NaNaRj\_F8.raw |  |  |  |  |  |  |  | 6.95E6 |  |  | 3 | 0 | 0 | 0 | 0 | 0 | 0 | 0 | 3 | 0 | 0 | 27 | 35 |  |
| K.M(+15.99)FMVAAPHVPVKRG.C | Y | 62.01 | 1554.8163 | 14 | 1.3 | 519.2800 | 3 | 11.46 | 8 | F8:2291 | 05112019\_RID\_1313\_NaNaRj\_F8.raw |  |  |  |  |  |  |  | 7.06E5 |  |  | 1 | 0 | 0 | 0 | 0 | 0 | 0 | 0 | 1 | 0 | 0 | 24 | 37 | Oxidation (M) |
| V.AAPHVPVK.R | Y | 61.37 | 817.4810 | 8 | 0.9 | 409.7481 | 2 | 11.26 | 8 | F8:2171 | 05112019\_RID\_1313\_NaNaRj\_F8.raw |  |  |  |  |  |  | 3.39E5 | 1.41E7 |  |  | 4 | 0 | 0 | 0 | 0 | 0 | 0 | 1 | 3 | 0 | 0 | 28 | 35 |  |
| K.KLVPLFSKTC(+57.02)PAGK.N | Y | 61.21 | 1544.8749 | 14 | -1.1 | 515.9650 | 3 | 11.35 | 8 | F8:2194 | 05112019\_RID\_1313\_NaNaRj\_F8.raw |  |  |  |  |  |  |  | 1.87E5 |  |  | 1 | 0 | 0 | 0 | 0 | 0 | 0 | 0 | 1 | 0 | 0 | 5 | 18 | Carbamidomethylation |
| K.TC(+57.02)PAGKNLC(+57.02)YK.M | N | 59.00 | 1310.6111 | 11 | 0.7 | 437.8779 | 3 | 10.68 | 8 | F8:1633 | 05112019\_RID\_1313\_NaNaRj\_F8.raw |  |  |  |  |  |  |  | 2E5 |  |  | 1 | 0 | 0 | 0 | 0 | 0 | 0 | 0 | 1 | 0 | 0 | 13 | 23 | Carbamidomethylation |
| R.GC(+57.02)IDVC(+57.02)PK.S | N | 56.82 | 947.4205 | 8 | -2.7 | 474.7162 | 2 | 11.15 | 8 | F8:2003 | 05112019\_RID\_1313\_NaNaRj\_F8.raw | 1E5 | 1.9E5 | 1.09E4 | 8.89E4 | 5.11E5 | 3.82E8 |  | 3.84E7 | 5.76E5 | 1.59E4 | 23 | 1 | 1 | 1 | 1 | 1 | 14 | 0 | 2 | 1 | 1 | 37 | 44 | Carbamidomethylation |
| K.M(+15.99)FMVAAPHVPVKR.G | Y | 56.73 | 1497.7948 | 13 | 0.9 | 500.2727 | 3 | 11.46 | 8 | F8:2292 | 05112019\_RID\_1313\_NaNaRj\_F8.raw |  |  |  |  |  |  |  | 4.68E5 |  |  | 1 | 0 | 0 | 0 | 0 | 0 | 0 | 0 | 1 | 0 | 0 | 24 | 36 | Oxidation (M) |
| F.M(+15.99)VAAPHVPVK.R | Y | 53.62 | 1063.5848 | 10 | 0.9 | 532.8002 | 2 | 11.06 | 8 | F8:1928 | 05112019\_RID\_1313\_NaNaRj\_F8.raw |  |  |  |  |  |  |  | 6.82E4 |  |  | 1 | 0 | 0 | 0 | 0 | 0 | 0 | 0 | 1 | 0 | 0 | 26 | 35 | Oxidation (M) |
| K.KLVPLFSK.T | Y | 50.67 | 930.5902 | 8 | -3.9 | 466.3006 | 2 | 11.76 | 8 | F8:2552 | 05112019\_RID\_1313\_NaNaRj\_F8.raw |  |  |  |  |  | 1.49E7 | 7.45E6 | 3.24E8 | 3.57E5 |  | 16 | 0 | 0 | 0 | 0 | 0 | 1 | 1 | 13 | 1 | 0 | 5 | 12 |  |
| K.SSLLVKYVC(+57.02).C | N | 50.14 | 1067.5685 | 9 | -0.1 | 534.7914 | 2 | 12.51 | 8 | F8:3206 | 05112019\_RID\_1313\_NaNaRj\_F8.raw |  |  |  |  |  |  |  | 2.17E5 |  |  | 1 | 0 | 0 | 0 | 0 | 0 | 0 | 0 | 1 | 0 | 0 | 45 | 53 | Carbamidomethylation |
| K.SSLLVKYVC(+57.02)C(+57.02).N | N | 43.03 | 1227.5991 | 10 | 0.0 | 614.8068 | 2 | 12.81 | 8 | F8:3446 | 05112019\_RID\_1313\_NaNaRj\_F8.raw |  |  |  |  |  |  |  | 0 |  |  | 1 | 0 | 0 | 0 | 0 | 0 | 0 | 0 | 1 | 0 | 0 | 45 | 54 | Carbamidomethylation |
| A.PHVPVKR.G | Y | 40.96 | 831.5079 | 7 | 0.3 | 416.7613 | 2 | 10.70 | 8 | F8:1628 | 05112019\_RID\_1313\_NaNaRj\_F8.raw |  |  |  |  |  |  |  | 0 |  |  | 1 | 0 | 0 | 0 | 0 | 0 | 0 | 0 | 1 | 0 | 0 | 30 | 36 |  |
| K.KLVPLFSKTC(+57.02)PAGKN.L | Y | 40.23 | 1658.9177 | 15 | 0.1 | 830.4662 | 2 | 11.43 | 8 | F8:2271 | 05112019\_RID\_1313\_NaNaRj\_F8.raw |  |  |  |  |  |  |  | 4.75E5 |  |  | 1 | 0 | 0 | 0 | 0 | 0 | 0 | 0 | 1 | 0 | 0 | 5 | 19 | Carbamidomethylation |
| K.LVPLFSK.T | Y | 40.09 | 802.4952 | 7 | 0.3 | 402.2550 | 2 | 17.88 | 8 | F8:5925 | 05112019\_RID\_1313\_NaNaRj\_F8.raw |  |  |  |  |  |  |  | 0 |  |  | 2 | 0 | 0 | 0 | 0 | 0 | 0 | 0 | 2 | 0 | 0 | 6 | 12 |  |
| total 28 peptides |  |  |  |  |  |  |  |  |  |  |  |  |  |  |  |  |  |  |  |  |  |  |  |  |  |  |  |  |  |  |  |  |  |  |  |
| --- | --- | --- | --- | --- | --- | --- | --- | --- | --- | --- | --- | --- | --- | --- | --- | --- | --- | --- | --- | --- | --- | --- | --- | --- | --- | --- | --- | --- | --- | --- | --- | --- | --- | --- | --- |

P01391|3L21\_NAJKA

back to list

  

| Protein Coverage
| Supporting Peptides
|

Protein Coverage:

Supporting Peptides:

| Peptide | Uniq | -10lgP | Mass | Length | ppm | m/z | z | RT | Fraction | Scan | Source File | Area F1 | Area F2 | Area F3 | Area F4 | Area F5 | Area F6 | Area F7 | Area F8 | Area F9 | Area F10 | #Feature | #Feature F1 | #Feature F2 | #Feature F3 | #Feature F4 | #Feature F5 | #Feature F6 | #Feature F7 | #Feature F8 | #Feature F9 | #Feature F10 | Start | End | PTM |
| --- | --- | --- | --- | --- | --- | --- | --- | --- | --- | --- | --- | --- | --- | --- | --- | --- | --- | --- | --- | --- | --- | --- | --- | --- | --- | --- | --- | --- | --- | --- | --- | --- | --- | --- | --- |
| K.TGVDIQC(+57.02)C(+57.02)STDNC(+57.02)NPFPTR.K | Y | 124.59 | 2240.9248 | 19 | 0.2 | 1121.4698 | 2 | 12.12 | 4 | F4:2826 | 05112019\_RID\_1313\_NaNaRj\_F4.raw |  |  |  | 3.42E8 | 8.68E5 | 3.23E6 | 8.02E5 | 4.39E5 | 1.42E5 | 6.89E4 | 26 | 0 | 0 | 0 | 18 | 2 | 1 | 2 | 1 | 1 | 1 | 50 | 68 | Carbamidomethylation |
| R.VDLGC(+57.02)AATC(+57.02)PTVK.T | N | 95.15 | 1390.6584 | 13 | 0.6 | 696.3369 | 2 | 11.58 | 5 | F5:2437 | 05112019\_RID\_1313\_NaNaRj\_F5.raw |  |  |  | 2.44E8 | 6.6E5 |  | 2.18E5 | 7.04E4 | 3.17E4 | 2.72E4 | 18 | 0 | 0 | 0 | 13 | 1 | 0 | 1 | 1 | 1 | 1 | 37 | 49 | Carbamidomethylation |
| R.C(+57.02)FITPDITSKDC(+57.02)PNGHVC(+57.02)YTK.T | N | 90.07 | 2512.1184 | 21 | -2.0 | 629.0356 | 4 | 11.75 | 4 | F4:2463 | 05112019\_RID\_1313\_NaNaRj\_F4.raw |  |  |  | 1.89E6 |  |  |  |  |  |  | 2 | 0 | 0 | 0 | 2 | 0 | 0 | 0 | 0 | 0 | 0 | 3 | 23 | Carbamidomethylation |
| Q.C(+57.02)C(+57.02)STDNC(+57.02)NPFPTR.K | Y | 89.67 | 1627.6178 | 13 | -1.4 | 814.8151 | 2 | 11.43 | 4 | F4:2263 | 05112019\_RID\_1313\_NaNaRj\_F4.raw |  |  |  | 1.32E6 |  |  |  |  |  |  | 1 | 0 | 0 | 0 | 1 | 0 | 0 | 0 | 0 | 0 | 0 | 56 | 68 | Carbamidomethylation |
| R.C(+57.02)FITPDITSK.D | N | 84.64 | 1180.5798 | 10 | 0.8 | 591.2977 | 2 | 12.69 | 4 | F4:3370 | 05112019\_RID\_1313\_NaNaRj\_F4.raw |  |  |  | 5.53E6 | 3.95E5 |  |  | 2.06E5 | 7.86E4 | 2.43E4 | 14 | 0 | 0 | 0 | 10 | 1 | 0 | 0 | 1 | 1 | 1 | 3 | 12 | Carbamidomethylation |
| K.RVDLGC(+57.02)AATC(+57.02)PTVK.T | N | 82.64 | 1546.7595 | 14 | 0.0 | 774.3870 | 2 | 11.26 | 4 | F4:2100 | 05112019\_RID\_1313\_NaNaRj\_F4.raw |  |  |  | 8.74E6 |  |  |  |  |  |  | 2 | 0 | 0 | 0 | 2 | 0 | 0 | 0 | 0 | 0 | 0 | 36 | 49 | Carbamidomethylation |
| C.C(+57.02)STDNC(+57.02)NPFPTR.K | Y | 72.66 | 1467.5872 | 12 | -1.4 | 734.7998 | 2 | 11.43 | 4 | F4:2262 | 05112019\_RID\_1313\_NaNaRj\_F4.raw |  |  |  | 5.38E5 |  |  |  |  |  |  | 1 | 0 | 0 | 0 | 1 | 0 | 0 | 0 | 0 | 0 | 0 | 57 | 68 | Carbamidomethylation |
| IRC(+57.02)FITPDITSK.D | N | 61.23 | 1449.7650 | 12 | -0.7 | 484.2619 | 3 | 11.86 | 4 | F4:2674 | 05112019\_RID\_1313\_NaNaRj\_F4.raw |  |  |  | 4.28E4 |  |  |  |  |  |  | 1 | 0 | 0 | 0 | 1 | 0 | 0 | 0 | 0 | 0 | 0 | 1 | 12 | Carbamidomethylation |
| C.STDNC(+57.02)NPFPTR.K | Y | 56.18 | 1307.5564 | 11 | 0.4 | 654.7858 | 2 | 11.42 | 5 | F5:2270 | 05112019\_RID\_1313\_NaNaRj\_F5.raw |  |  |  |  | 9.91E4 |  |  |  | 1.1E4 |  | 2 | 0 | 0 | 0 | 0 | 1 | 0 | 0 | 0 | 1 | 0 | 58 | 68 | Carbamidomethylation |
| D.IQC(+57.02)C(+57.02)STDNC(+57.02)NPFPTR.K | Y | 52.63 | 1868.7604 | 15 | 1.4 | 935.3887 | 2 | 11.56 | 4 | F4:2375 | 05112019\_RID\_1313\_NaNaRj\_F4.raw |  |  | 1.47E4 | 0 |  |  |  |  |  |  | 2 | 0 | 0 | 1 | 1 | 0 | 0 | 0 | 0 | 0 | 0 | 54 | 68 | Carbamidomethylation |
| K.DC(+57.02)PNGHVC(+57.02)Y.T | N | 47.04 | 1120.4066 | 9 | -0.3 | 561.2104 | 2 | 11.16 | 4 | F4:2024 | 05112019\_RID\_1313\_NaNaRj\_F4.raw |  |  |  | 1.84E6 |  |  |  |  |  |  | 1 | 0 | 0 | 0 | 1 | 0 | 0 | 0 | 0 | 0 | 0 | 13 | 21 | Carbamidomethylation |
| IRC(+57.02)FITPDITSKDC(+57.02)PNGHVC(+57.02)YTK.T | N | 46.82 | 2781.3037 | 23 | 1.4 | 557.2688 | 5 | 11.57 | 4 | F4:2385 | 05112019\_RID\_1313\_NaNaRj\_F4.raw |  |  |  | 0 |  |  |  |  |  |  | 1 | 0 | 0 | 0 | 1 | 0 | 0 | 0 | 0 | 0 | 0 | 1 | 23 | Carbamidomethylation |
| D.LGC(+57.02)AATC(+57.02)PTVK.T | N | 45.08 | 1176.5631 | 11 | 0.1 | 589.2889 | 2 | 11.03 | 4 | F4:1924 | 05112019\_RID\_1313\_NaNaRj\_F4.raw |  |  |  | 2.8E4 |  |  |  |  |  |  | 1 | 0 | 0 | 0 | 1 | 0 | 0 | 0 | 0 | 0 | 0 | 39 | 49 | Carbamidomethylation |
| R.GKRVDLGC(+57.02)AATC(+57.02)PTVK.T | Y | 40.41 | 1731.8760 | 16 | 0.9 | 578.2998 | 3 | 10.94 | 4 | F4:1838 | 05112019\_RID\_1313\_NaNaRj\_F4.raw |  |  |  | 1.17E4 |  |  |  |  |  |  | 1 | 0 | 0 | 0 | 1 | 0 | 0 | 0 | 0 | 0 | 0 | 34 | 49 | Carbamidomethylation |
| R.VDLGC(+57.02)AATC(+57.02)PTVKTGVDIQC(+57.02)C(+57.02)STDNC(+57.02)NPFPTR.K | Y | 40.24 | 3613.5728 | 32 | -1.1 | 1205.5302 | 3 | 13.10 | 4 | F4:3689 | 05112019\_RID\_1313\_NaNaRj\_F4.raw |  |  |  | 1.69E5 |  |  |  |  |  |  | 1 | 0 | 0 | 0 | 1 | 0 | 0 | 0 | 0 | 0 | 0 | 37 | 68 | Carbamidomethylation |
| total 15 peptides |  |  |  |  |  |  |  |  |  |  |  |  |  |  |  |  |  |  |  |  |  |  |  |  |  |  |  |  |  |  |  |  |  |  |  |
| --- | --- | --- | --- | --- | --- | --- | --- | --- | --- | --- | --- | --- | --- | --- | --- | --- | --- | --- | --- | --- | --- | --- | --- | --- | --- | --- | --- | --- | --- | --- | --- | --- | --- | --- | --- |

P02538|K2C6A\_HUMAN

back to list

  

| Protein Coverage
| Supporting Peptides
|

Protein Coverage:

Supporting Peptides:

| Peptide | Uniq | -10lgP | Mass | Length | ppm | m/z | z | RT | Fraction | Scan | Source File | Area F1 | Area F2 | Area F3 | Area F4 | Area F5 | Area F6 | Area F7 | Area F8 | Area F9 | Area F10 | #Feature | #Feature F1 | #Feature F2 | #Feature F3 | #Feature F4 | #Feature F5 | #Feature F6 | #Feature F7 | #Feature F8 | #Feature F9 | #Feature F10 | Start | End | PTM |
| --- | --- | --- | --- | --- | --- | --- | --- | --- | --- | --- | --- | --- | --- | --- | --- | --- | --- | --- | --- | --- | --- | --- | --- | --- | --- | --- | --- | --- | --- | --- | --- | --- | --- | --- | --- |
| R.GSGGLGGAC(+57.02)GGAGFGSR.S | N | 99.57 | 1423.6262 | 17 | 0.8 | 712.8209 | 2 | 11.40 | 5 | F5:2249 | 05112019\_RID\_1313\_NaNaRj\_F5.raw |  | 1.58E5 | 2.69E4 |  | 1.41E5 |  |  |  |  |  | 3 | 0 | 1 | 1 | 0 | 1 | 0 | 0 | 0 | 0 | 0 | 43 | 59 | Carbamidomethylation |
| R.ISIGGGSC(+57.02)AISGGYGSR.A | N | 94.11 | 1597.7518 | 17 | 1.4 | 799.8843 | 2 | 11.80 | 5 | F5:2667 | 05112019\_RID\_1313\_NaNaRj\_F5.raw |  | 1.3E5 | 6.2E4 |  | 1.81E5 |  |  |  |  |  | 3 | 0 | 1 | 1 | 0 | 1 | 0 | 0 | 0 | 0 | 0 | 70 | 86 | Carbamidomethylation |
| R.AIGGGLSSVGGGSSTIK.Y | N | 92.75 | 1446.7678 | 17 | -0.3 | 724.3910 | 2 | 11.63 | 3 | F3:2413 | 05112019\_RID\_1313\_NaNaRj\_F3.raw |  | 1.87E5 | 6.28E4 |  | 5.02E4 |  |  |  |  |  | 3 | 0 | 1 | 1 | 0 | 1 | 0 | 0 | 0 | 0 | 0 | 534 | 550 |  |
| R.NLDLDSIIAEVK.A | N | 78.64 | 1328.7188 | 12 | 0.5 | 665.3670 | 2 | 35.93 | 7 | F7:9727 | 05112019\_RID\_1313\_NaNaRj\_F7.raw |  |  |  | 6.48E5 | 5.9E4 | 9.04E5 | 6.22E5 | 3.5E5 |  |  | 5 | 0 | 0 | 0 | 1 | 1 | 1 | 1 | 1 | 0 | 0 | 327 | 338 |  |
| K.AQYEEIAQR.S | N | 74.78 | 1106.5356 | 9 | -0.4 | 554.2749 | 2 | 11.11 | 3 | F3:1935 | 05112019\_RID\_1313\_NaNaRj\_F3.raw | 2.4E4 | 2.26E6 | 2.18E5 |  | 9.57E5 |  |  |  |  | 4.57E4 | 6 | 1 | 2 | 1 | 0 | 1 | 0 | 0 | 0 | 0 | 1 | 339 | 347 |  |
| R.TAAENEFVTLK.K | N | 74.57 | 1221.6240 | 11 | -0.3 | 611.8191 | 2 | 11.96 | 5 | F5:2796 | 05112019\_RID\_1313\_NaNaRj\_F5.raw |  |  | 2.94E4 |  | 1.82E5 |  |  |  |  |  | 2 | 0 | 0 | 1 | 0 | 1 | 0 | 0 | 0 | 0 | 0 | 261 | 271 |  |
| K.NKYEDEINKR.T | N | 67.54 | 1307.6470 | 10 | 0.5 | 436.8898 | 3 | 12.46 | 2 | F2:2012 | 09012020\_NaNaRj\_F2.raw |  | 4.15E6 |  |  | 2.01E5 |  |  |  |  |  | 3 | 0 | 2 | 0 | 0 | 1 | 0 | 0 | 0 | 0 | 0 | 251 | 260 |  |
| K.LALDVEIATYR.K | N | 67.43 | 1262.6870 | 11 | 0.5 | 632.3511 | 2 | 18.91 | 6 | F6:7379 | 05112019\_RID\_1313\_NaNaRj\_F6.raw |  |  |  | 8.76E4 |  | 3.23E5 | 1.04E5 |  |  |  | 4 | 0 | 0 | 0 | 1 | 0 | 2 | 1 | 0 | 0 | 0 | 456 | 466 |  |
| K.ADTLTDEINFLR.A | N | 64.21 | 1406.7041 | 12 | 0.2 | 704.3595 | 2 | 26.03 | 3 | F3:5073 | 05112019\_RID\_1313\_NaNaRj\_F3.raw |  | 6.48E4 | 2.18E4 | 6.2E4 | 1.56E4 |  | 1.02E5 | 4.06E3 |  |  | 6 | 0 | 1 | 1 | 1 | 1 | 0 | 1 | 1 | 0 | 0 | 288 | 299 |  |
| K.LEGLEDALQK.A | N | 63.85 | 1114.5869 | 10 | 0.9 | 558.3012 | 2 | 12.23 | 5 | F5:3018 | 05112019\_RID\_1313\_NaNaRj\_F5.raw |  | 1.46E5 |  |  | 1.42E5 |  |  |  |  |  | 2 | 0 | 1 | 0 | 0 | 1 | 0 | 0 | 0 | 0 | 0 | 427 | 436 |  |
| R.AEAESWYQTK.Y | N | 63.25 | 1211.5459 | 10 | 0.5 | 606.7805 | 2 | 11.43 | 3 | F3:2226 | 05112019\_RID\_1313\_NaNaRj\_F3.raw |  | 6.72E4 | 1.05E4 |  | 7.56E4 |  |  |  |  | 3.71E3 | 4 | 0 | 1 | 1 | 0 | 1 | 0 | 0 | 0 | 0 | 1 | 350 | 359 |  |
| R.SGFSSVSVSR.S | Y | 62.78 | 1011.4985 | 10 | 0.0 | 506.7565 | 2 | 11.34 | 3 | F3:2145 | 05112019\_RID\_1313\_NaNaRj\_F3.raw |  | 7.54E4 | 1.65E4 |  | 9.06E4 |  |  |  |  | 5.86E3 | 4 | 0 | 1 | 1 | 0 | 1 | 0 | 0 | 0 | 0 | 1 | 31 | 40 |  |
| K.YEELQVTAGR.H | N | 62.06 | 1164.5775 | 10 | 0.7 | 583.2964 | 2 | 31.74 | 2 | F2:8917 | 09012020\_NaNaRj\_F2.raw |  | 1.62E5 | 3.12E4 |  |  |  |  |  |  |  | 2 | 0 | 1 | 1 | 0 | 0 | 0 | 0 | 0 | 0 | 0 | 360 | 369 |  |
| K.WTLLQEQGTK.T | N | 56.02 | 1202.6295 | 10 | 0.6 | 602.3224 | 2 | 12.51 | 5 | F5:3187 | 05112019\_RID\_1313\_NaNaRj\_F5.raw |  | 1.05E6 |  |  | 9.31E4 |  |  |  |  |  | 2 | 0 | 1 | 0 | 0 | 1 | 0 | 0 | 0 | 0 | 0 | 195 | 204 |  |
| R.QLDSIVGER.G | N | 54.54 | 1015.5298 | 9 | 0.6 | 508.7725 | 2 | 11.56 | 3 | F3:2354 | 05112019\_RID\_1313\_NaNaRj\_F3.raw |  | 1.03E6 | 6.68E4 |  | 1.52E5 |  |  |  |  | 2.38E4 | 4 | 0 | 1 | 1 | 0 | 1 | 0 | 0 | 0 | 0 | 1 | 224 | 232 |  |
| K.LLEGEEC(+57.02)R.L | N | 54.31 | 1004.4597 | 8 | -0.6 | 503.2368 | 2 | 11.02 | 5 | F5:1874 | 05112019\_RID\_1313\_NaNaRj\_F5.raw |  |  |  |  | 1.16E5 |  |  |  |  |  | 1 | 0 | 0 | 0 | 0 | 1 | 0 | 0 | 0 | 0 | 0 | 468 | 475 | Carbamidomethylation |
| K.EYQELMNVK.L | N | 53.06 | 1152.5485 | 9 | -0.9 | 577.2810 | 2 | 37.95 | 2 | F2:11586 | 09012020\_NaNaRj\_F2.raw |  | 2.37E5 |  |  |  |  |  |  |  |  | 1 | 0 | 1 | 0 | 0 | 0 | 0 | 0 | 0 | 0 | 0 | 447 | 455 |  |
| R.QNLEPLFEQYINNLR.R | N | 52.74 | 1889.9635 | 15 | 0.8 | 630.9956 | 3 | 41.57 | 6 | F6:17761 | 05112019\_RID\_1313\_NaNaRj\_F6.raw |  |  |  |  |  | 5.84E4 |  |  |  |  | 1 | 0 | 0 | 0 | 0 | 0 | 1 | 0 | 0 | 0 | 0 | 208 | 222 |  |
| R.GM(+15.99)QDLVEDFK.N | Y | 51.40 | 1196.5383 | 10 | -0.3 | 599.2762 | 2 | 12.08 | 3 | F3:2733 | 05112019\_RID\_1313\_NaNaRj\_F3.raw |  |  | 4.26E3 |  |  |  |  |  |  |  | 1 | 0 | 0 | 1 | 0 | 0 | 0 | 0 | 0 | 0 | 0 | 241 | 250 | Oxidation (M) |
| K.DVDAAYMNK.V | N | 48.86 | 1025.4487 | 9 | 0.7 | 513.7320 | 2 | 11.29 | 3 | F3:2108 | 05112019\_RID\_1313\_NaNaRj\_F3.raw |  |  | 3.06E3 |  |  |  |  |  |  |  | 1 | 0 | 0 | 1 | 0 | 0 | 0 | 0 | 0 | 0 | 0 | 273 | 281 |  |
| K.FASFIDK.V | N | 47.57 | 826.4225 | 7 | 0.0 | 414.2185 | 2 | 39.16 | 2 | F2:12055 | 09012020\_NaNaRj\_F2.raw | 6.35E3 | 4.99E6 |  |  | 4.92E5 |  |  |  | 5.38E3 |  | 4 | 1 | 1 | 0 | 0 | 1 | 0 | 0 | 0 | 1 | 0 | 174 | 180 |  |
| K.QEIAEINR.M | N | 42.46 | 971.5036 | 8 | 0.3 | 486.7592 | 2 | 11.15 | 5 | F5:2013 | 05112019\_RID\_1313\_NaNaRj\_F5.raw |  |  |  |  | 4.16E4 |  |  |  |  |  | 1 | 0 | 0 | 0 | 0 | 1 | 0 | 0 | 0 | 0 | 0 | 379 | 386 |  |
| total 22 peptides |  |  |  |  |  |  |  |  |  |  |  |  |  |  |  |  |  |  |  |  |  |  |  |  |  |  |  |  |  |  |  |  |  |  |  |
| --- | --- | --- | --- | --- | --- | --- | --- | --- | --- | --- | --- | --- | --- | --- | --- | --- | --- | --- | --- | --- | --- | --- | --- | --- | --- | --- | --- | --- | --- | --- | --- | --- | --- | --- | --- |

P04259|K2C6B\_HUMAN

back to list

  

| Protein Coverage
| Supporting Peptides
|

Protein Coverage:

Supporting Peptides:

| Peptide | Uniq | -10lgP | Mass | Length | ppm | m/z | z | RT | Fraction | Scan | Source File | Area F1 | Area F2 | Area F3 | Area F4 | Area F5 | Area F6 | Area F7 | Area F8 | Area F9 | Area F10 | #Feature | #Feature F1 | #Feature F2 | #Feature F3 | #Feature F4 | #Feature F5 | #Feature F6 | #Feature F7 | #Feature F8 | #Feature F9 | #Feature F10 | Start | End | PTM |
| --- | --- | --- | --- | --- | --- | --- | --- | --- | --- | --- | --- | --- | --- | --- | --- | --- | --- | --- | --- | --- | --- | --- | --- | --- | --- | --- | --- | --- | --- | --- | --- | --- | --- | --- | --- |
| R.GSGGLGGAC(+57.02)GGAGFGSR.S | N | 99.57 | 1423.6262 | 17 | 0.8 | 712.8209 | 2 | 11.40 | 5 | F5:2249 | 05112019\_RID\_1313\_NaNaRj\_F5.raw |  | 1.58E5 | 2.69E4 |  | 1.41E5 |  |  |  |  |  | 3 | 0 | 1 | 1 | 0 | 1 | 0 | 0 | 0 | 0 | 0 | 43 | 59 | Carbamidomethylation |
| R.ISIGGGSC(+57.02)AISGGYGSR.A | N | 94.11 | 1597.7518 | 17 | 1.4 | 799.8843 | 2 | 11.80 | 5 | F5:2667 | 05112019\_RID\_1313\_NaNaRj\_F5.raw |  | 1.3E5 | 6.2E4 |  | 1.81E5 |  |  |  |  |  | 3 | 0 | 1 | 1 | 0 | 1 | 0 | 0 | 0 | 0 | 0 | 70 | 86 | Carbamidomethylation |
| K.YEELQITAGR.H | N | 83.22 | 1178.5931 | 10 | 1.2 | 590.3046 | 2 | 11.78 | 1 | F1:2548 | 05112019\_RID\_1313\_NaNaRj\_F1.raw | 1.61E5 | 2.9E7 | 5.44E5 | 0 |  |  | 2.71E5 |  | 8.56E4 | 1.7E5 | 12 | 1 | 6 | 1 | 1 | 0 | 0 | 1 | 0 | 1 | 1 | 360 | 369 |  |
| R.ATGGGLSSVGGGSSTIK.Y | Y | 78.73 | 1434.7314 | 17 | 0.4 | 718.3733 | 2 | 11.28 | 3 | F3:2095 | 05112019\_RID\_1313\_NaNaRj\_F3.raw |  | 3.69E4 | 1.18E4 |  | 2.06E4 |  |  |  |  |  | 3 | 0 | 1 | 1 | 0 | 1 | 0 | 0 | 0 | 0 | 0 | 534 | 550 |  |
| R.NLDLDSIIAEVK.A | N | 78.64 | 1328.7188 | 12 | 0.5 | 665.3670 | 2 | 35.93 | 7 | F7:9727 | 05112019\_RID\_1313\_NaNaRj\_F7.raw |  |  |  | 6.48E5 | 5.9E4 | 9.04E5 | 6.22E5 | 3.5E5 |  |  | 5 | 0 | 0 | 0 | 1 | 1 | 1 | 1 | 1 | 0 | 0 | 327 | 338 |  |
| K.AQYEEIAQR.S | N | 74.78 | 1106.5356 | 9 | -0.4 | 554.2749 | 2 | 11.11 | 3 | F3:1935 | 05112019\_RID\_1313\_NaNaRj\_F3.raw | 2.4E4 | 2.26E6 | 2.18E5 |  | 9.57E5 |  |  |  |  | 4.57E4 | 6 | 1 | 2 | 1 | 0 | 1 | 0 | 0 | 0 | 0 | 1 | 339 | 347 |  |
| R.TAAENEFVTLK.K | N | 74.57 | 1221.6240 | 11 | -0.3 | 611.8191 | 2 | 11.96 | 5 | F5:2796 | 05112019\_RID\_1313\_NaNaRj\_F5.raw |  |  | 2.94E4 |  | 1.82E5 |  |  |  |  |  | 2 | 0 | 0 | 1 | 0 | 1 | 0 | 0 | 0 | 0 | 0 | 261 | 271 |  |
| K.NKYEDEINKR.T | N | 67.54 | 1307.6470 | 10 | 0.5 | 436.8898 | 3 | 12.46 | 2 | F2:2012 | 09012020\_NaNaRj\_F2.raw |  | 4.15E6 |  |  | 2.01E5 |  |  |  |  |  | 3 | 0 | 2 | 0 | 0 | 1 | 0 | 0 | 0 | 0 | 0 | 251 | 260 |  |
| K.LALDVEIATYR.K | N | 67.43 | 1262.6870 | 11 | 0.5 | 632.3511 | 2 | 18.91 | 6 | F6:7379 | 05112019\_RID\_1313\_NaNaRj\_F6.raw |  |  |  | 8.76E4 |  | 3.23E5 | 1.04E5 |  |  |  | 4 | 0 | 0 | 0 | 1 | 0 | 2 | 1 | 0 | 0 | 0 | 456 | 466 |  |
| K.ADTLTDEINFLR.A | N | 64.21 | 1406.7041 | 12 | 0.2 | 704.3595 | 2 | 26.03 | 3 | F3:5073 | 05112019\_RID\_1313\_NaNaRj\_F3.raw |  | 6.48E4 | 2.18E4 | 6.2E4 | 1.56E4 |  | 1.02E5 | 4.06E3 |  |  | 6 | 0 | 1 | 1 | 1 | 1 | 0 | 1 | 1 | 0 | 0 | 288 | 299 |  |
| K.LEGLEDALQK.A | N | 63.85 | 1114.5869 | 10 | 0.9 | 558.3012 | 2 | 12.23 | 5 | F5:3018 | 05112019\_RID\_1313\_NaNaRj\_F5.raw |  | 1.46E5 |  |  | 1.42E5 |  |  |  |  |  | 2 | 0 | 1 | 0 | 0 | 1 | 0 | 0 | 0 | 0 | 0 | 427 | 436 |  |
| R.AEAESWYQTK.Y | N | 63.25 | 1211.5459 | 10 | 0.5 | 606.7805 | 2 | 11.43 | 3 | F3:2226 | 05112019\_RID\_1313\_NaNaRj\_F3.raw |  | 6.72E4 | 1.05E4 |  | 7.56E4 |  |  |  |  | 3.71E3 | 4 | 0 | 1 | 1 | 0 | 1 | 0 | 0 | 0 | 0 | 1 | 350 | 359 |  |
| R.SGFSSISVSR.S | N | 63.14 | 1025.5142 | 10 | -0.9 | 513.7639 | 2 | 11.65 | 5 | F5:2503 | 05112019\_RID\_1313\_NaNaRj\_F5.raw |  | 7.48E4 | 1.36E4 |  | 7.05E4 |  |  |  |  |  | 3 | 0 | 1 | 1 | 0 | 1 | 0 | 0 | 0 | 0 | 0 | 31 | 40 |  |
| K.WTLLQEQGTK.T | N | 56.02 | 1202.6295 | 10 | 0.6 | 602.3224 | 2 | 12.51 | 5 | F5:3187 | 05112019\_RID\_1313\_NaNaRj\_F5.raw |  | 1.05E6 |  |  | 9.31E4 |  |  |  |  |  | 2 | 0 | 1 | 0 | 0 | 1 | 0 | 0 | 0 | 0 | 0 | 195 | 204 |  |
| K.LLEGEEC(+57.02)R.L | N | 54.31 | 1004.4597 | 8 | -0.6 | 503.2368 | 2 | 11.02 | 5 | F5:1874 | 05112019\_RID\_1313\_NaNaRj\_F5.raw |  |  |  |  | 1.16E5 |  |  |  |  |  | 1 | 0 | 0 | 0 | 0 | 1 | 0 | 0 | 0 | 0 | 0 | 468 | 475 | Carbamidomethylation |
| K.EYQELMNVK.L | N | 53.06 | 1152.5485 | 9 | -0.9 | 577.2810 | 2 | 37.95 | 2 | F2:11586 | 09012020\_NaNaRj\_F2.raw |  | 2.37E5 |  |  |  |  |  |  |  |  | 1 | 0 | 1 | 0 | 0 | 0 | 0 | 0 | 0 | 0 | 0 | 447 | 455 |  |
| R.QNLEPLFEQYINNLR.R | N | 52.74 | 1889.9635 | 15 | 0.8 | 630.9956 | 3 | 41.57 | 6 | F6:17761 | 05112019\_RID\_1313\_NaNaRj\_F6.raw |  |  |  |  |  | 5.84E4 |  |  |  |  | 1 | 0 | 0 | 0 | 0 | 0 | 1 | 0 | 0 | 0 | 0 | 208 | 222 |  |
| K.DVDAAYMNK.V | N | 48.86 | 1025.4487 | 9 | 0.7 | 513.7320 | 2 | 11.29 | 3 | F3:2108 | 05112019\_RID\_1313\_NaNaRj\_F3.raw |  |  | 3.06E3 |  |  |  |  |  |  |  | 1 | 0 | 0 | 1 | 0 | 0 | 0 | 0 | 0 | 0 | 0 | 273 | 281 |  |
| K.FASFIDK.V | N | 47.57 | 826.4225 | 7 | 0.0 | 414.2185 | 2 | 39.16 | 2 | F2:12055 | 09012020\_NaNaRj\_F2.raw | 6.35E3 | 4.99E6 |  |  | 4.92E5 |  |  |  | 5.38E3 |  | 4 | 1 | 1 | 0 | 0 | 1 | 0 | 0 | 0 | 1 | 0 | 174 | 180 |  |
| R.QLDNIVGER.G | Y | 42.56 | 1042.5406 | 9 | -0.1 | 522.2775 | 2 | 11.56 | 5 | F5:2422 | 05112019\_RID\_1313\_NaNaRj\_F5.raw |  |  |  |  | 3.7E4 |  |  |  |  |  | 1 | 0 | 0 | 0 | 0 | 1 | 0 | 0 | 0 | 0 | 0 | 224 | 232 |  |
| K.QEIAEINR.M | N | 42.46 | 971.5036 | 8 | 0.3 | 486.7592 | 2 | 11.15 | 5 | F5:2013 | 05112019\_RID\_1313\_NaNaRj\_F5.raw |  |  |  |  | 4.16E4 |  |  |  |  |  | 1 | 0 | 0 | 0 | 0 | 1 | 0 | 0 | 0 | 0 | 0 | 379 | 386 |  |
| L.NLQIDPAIQR.V | N | 41.80 | 1166.6407 | 10 | 0.0 | 584.3276 | 2 | 11.92 | 5 | F5:2753 | 05112019\_RID\_1313\_NaNaRj\_F5.raw |  | 1.63E5 |  |  | 5.71E4 |  |  |  |  |  | 2 | 0 | 1 | 0 | 0 | 1 | 0 | 0 | 0 | 0 | 0 | 149 | 158 |  |
| total 22 peptides |  |  |  |  |  |  |  |  |  |  |  |  |  |  |  |  |  |  |  |  |  |  |  |  |  |  |  |  |  |  |  |  |  |  |  |
| --- | --- | --- | --- | --- | --- | --- | --- | --- | --- | --- | --- | --- | --- | --- | --- | --- | --- | --- | --- | --- | --- | --- | --- | --- | --- | --- | --- | --- | --- | --- | --- | --- | --- | --- | --- |

Q5YF89|NGFV2\_NAJSP

back to list

  

| Protein Coverage
| Supporting Peptides
|

Protein Coverage:

Supporting Peptides:

| Peptide | Uniq | -10lgP | Mass | Length | ppm | m/z | z | RT | Fraction | Scan | Source File | Area F1 | Area F2 | Area F3 | Area F4 | Area F5 | Area F6 | Area F7 | Area F8 | Area F9 | Area F10 | #Feature | #Feature F1 | #Feature F2 | #Feature F3 | #Feature F4 | #Feature F5 | #Feature F6 | #Feature F7 | #Feature F8 | #Feature F9 | #Feature F10 | Start | End | PTM |
| --- | --- | --- | --- | --- | --- | --- | --- | --- | --- | --- | --- | --- | --- | --- | --- | --- | --- | --- | --- | --- | --- | --- | --- | --- | --- | --- | --- | --- | --- | --- | --- | --- | --- | --- | --- |
| R.EDHPVHNLGEHSVC(+57.02)DSVSAWVTK.T | Y | 126.38 | 2602.1870 | 23 | -2.9 | 868.4004 | 3 | 11.71 | 8 | F8:2516 | 05112019\_RID\_1313\_NaNaRj\_F8.raw |  |  |  |  |  |  | 8.45E5 | 1.67E7 |  |  | 6 | 0 | 0 | 0 | 0 | 0 | 0 | 2 | 4 | 0 | 0 | 126 | 148 | Carbamidomethylation |
| K.GNTVTVMENVNLDNK.V | Y | 97.18 | 1646.7933 | 15 | 0.3 | 824.4042 | 2 | 12.04 | 8 | F8:2796 | 05112019\_RID\_1313\_NaNaRj\_F8.raw |  |  |  |  |  |  | 9.91E4 | 3.32E6 | 1.87E4 |  | 3 | 0 | 0 | 0 | 0 | 0 | 0 | 1 | 1 | 1 | 0 | 156 | 170 |  |
| K.GNTVTVM(+15.99)ENVNLDNK.V | Y | 96.79 | 1662.7883 | 15 | 0.2 | 832.4016 | 2 | 11.51 | 8 | F8:2319 | 05112019\_RID\_1313\_NaNaRj\_F8.raw |  |  |  |  |  |  | 2.7E5 | 8.81E5 |  |  | 2 | 0 | 0 | 0 | 0 | 0 | 0 | 1 | 1 | 0 | 0 | 156 | 170 | Oxidation (M) |
| N.LGEHSVC(+57.02)DSVSAWVTK.T | Y | 94.53 | 1773.8356 | 16 | 0.9 | 887.9259 | 2 | 12.12 | 8 | F8:2881 | 05112019\_RID\_1313\_NaNaRj\_F8.raw |  |  |  |  |  |  |  | 1.17E6 |  |  | 2 | 0 | 0 | 0 | 0 | 0 | 0 | 0 | 2 | 0 | 0 | 133 | 148 | Carbamidomethylation |
| R.GIDSSHWNSYC(+57.02)TETDTFIK.A | Y | 92.49 | 2259.9744 | 19 | 0.6 | 754.3325 | 3 | 12.51 | 8 | F8:3188 | 05112019\_RID\_1313\_NaNaRj\_F8.raw |  |  |  |  |  |  |  | 1.8E6 |  |  | 2 | 0 | 0 | 0 | 0 | 0 | 0 | 0 | 2 | 0 | 0 | 193 | 211 | Carbamidomethylation |
| R.IDTAC(+57.02)VC(+57.02)VITK.K | Y | 89.80 | 1278.6312 | 11 | -1.2 | 640.3221 | 2 | 11.78 | 8 | F8:2574 | 05112019\_RID\_1313\_NaNaRj\_F8.raw |  |  | 4.39E3 |  |  | 2.81E5 | 1.7E6 | 9.15E6 | 4.61E4 |  | 8 | 0 | 0 | 1 | 0 | 0 | 1 | 1 | 4 | 1 | 0 | 227 | 237 | Carbamidomethylation |
| K.ALTMEGNQASWR.F | Y | 86.01 | 1362.6350 | 12 | -3.3 | 682.3225 | 2 | 11.75 | 8 | F8:2560 | 05112019\_RID\_1313\_NaNaRj\_F8.raw |  |  |  |  |  |  | 1.37E5 | 1.07E6 |  |  | 2 | 0 | 0 | 0 | 0 | 0 | 0 | 1 | 1 | 0 | 0 | 212 | 223 |  |
| N.SYC(+57.02)TETDTFIK.A | Y | 64.70 | 1363.5966 | 11 | -0.9 | 682.8049 | 2 | 11.81 | 8 | F8:2594 | 05112019\_RID\_1313\_NaNaRj\_F8.raw |  |  |  |  | 4.78E4 |  | 1.93E6 | 5.74E6 |  |  | 3 | 0 | 0 | 0 | 0 | 1 | 0 | 1 | 1 | 0 | 0 | 201 | 211 | Carbamidomethylation |
| H.SVC(+57.02)DSVSAWVTK.T | Y | 61.94 | 1337.6285 | 12 | 0.7 | 669.8220 | 2 | 13.16 | 8 | F8:3633 | 05112019\_RID\_1313\_NaNaRj\_F8.raw |  |  |  |  |  |  |  | 7.07E5 |  |  | 1 | 0 | 0 | 0 | 0 | 0 | 0 | 0 | 1 | 0 | 0 | 137 | 148 | Carbamidomethylation |
| H.NLGEHSVC(+57.02)DSVSAWVTK.T | Y | 60.58 | 1887.8785 | 17 | 0.9 | 630.3007 | 3 | 12.17 | 8 | F8:2922 | 05112019\_RID\_1313\_NaNaRj\_F8.raw |  |  |  |  |  |  |  | 1.68E5 |  |  | 1 | 0 | 0 | 0 | 0 | 0 | 0 | 0 | 1 | 0 | 0 | 132 | 148 | Carbamidomethylation |
| K.QYFFETK.C | Y | 60.50 | 961.4545 | 7 | -1.4 | 481.7338 | 2 | 12.08 | 8 | F8:2823 | 05112019\_RID\_1313\_NaNaRj\_F8.raw |  |  |  |  | 8.5E4 | 2.11E5 | 1.14E6 | 4.21E6 |  | 3.02E3 | 5 | 0 | 0 | 0 | 0 | 1 | 1 | 1 | 1 | 0 | 1 | 174 | 180 |  |
| K.ALTM(+15.99)EGNQASWR.F | Y | 52.64 | 1378.6299 | 12 | 0.3 | 690.3224 | 2 | 11.36 | 7 | F7:2217 | 05112019\_RID\_1313\_NaNaRj\_F7.raw |  |  |  |  |  |  | 9.8E4 |  |  |  | 1 | 0 | 0 | 0 | 0 | 0 | 0 | 1 | 0 | 0 | 0 | 212 | 223 | Oxidation (M) |
| R.FIRIDTAC(+57.02)VC(+57.02)VITKK.T | Y | 52.15 | 1822.9797 | 15 | -1.9 | 608.6660 | 3 | 11.84 | 8 | F8:2652 | 05112019\_RID\_1313\_NaNaRj\_F8.raw |  |  |  |  |  |  |  | 1.01E5 |  |  | 1 | 0 | 0 | 0 | 0 | 0 | 0 | 0 | 1 | 0 | 0 | 224 | 238 | Carbamidomethylation |
| R.FIRIDTAC(+57.02)VC(+57.02)VITK.K | Y | 44.61 | 1694.8848 | 14 | 1.0 | 565.9694 | 3 | 12.95 | 7 | F7:3382 | 05112019\_RID\_1313\_NaNaRj\_F7.raw |  |  |  |  |  |  | 5.94E4 | 5.26E5 |  |  | 2 | 0 | 0 | 0 | 0 | 0 | 0 | 1 | 1 | 0 | 0 | 224 | 237 | Carbamidomethylation |
| total 14 peptides |  |  |  |  |  |  |  |  |  |  |  |  |  |  |  |  |  |  |  |  |  |  |  |  |  |  |  |  |  |  |  |  |  |  |  |
| --- | --- | --- | --- | --- | --- | --- | --- | --- | --- | --- | --- | --- | --- | --- | --- | --- | --- | --- | --- | --- | --- | --- | --- | --- | --- | --- | --- | --- | --- | --- | --- | --- | --- | --- | --- |

P08779|K1C16\_HUMAN

back to list

  

| Protein Coverage
| Supporting Peptides
|

Protein Coverage:

Supporting Peptides:

| Peptide | Uniq | -10lgP | Mass | Length | ppm | m/z | z | RT | Fraction | Scan | Source File | Area F1 | Area F2 | Area F3 | Area F4 | Area F5 | Area F6 | Area F7 | Area F8 | Area F9 | Area F10 | #Feature | #Feature F1 | #Feature F2 | #Feature F3 | #Feature F4 | #Feature F5 | #Feature F6 | #Feature F7 | #Feature F8 | #Feature F9 | #Feature F10 | Start | End | PTM |
| --- | --- | --- | --- | --- | --- | --- | --- | --- | --- | --- | --- | --- | --- | --- | --- | --- | --- | --- | --- | --- | --- | --- | --- | --- | --- | --- | --- | --- | --- | --- | --- | --- | --- | --- | --- |
| R.APSTYGGGLSVSSR.F | Y | 91.60 | 1337.6575 | 14 | 0.4 | 669.8363 | 2 | 11.44 | 5 | F5:2291 | 05112019\_RID\_1313\_NaNaRj\_F5.raw |  | 6.25E4 | 6.09E4 |  | 1.81E5 |  |  |  |  | 3.28E4 | 4 | 0 | 1 | 1 | 0 | 1 | 0 | 0 | 0 | 0 | 1 | 42 | 55 |  |
| K.EVASNSELVQSSR.S | Y | 84.86 | 1404.6844 | 13 | 0.9 | 703.3501 | 2 | 11.12 | 5 | F5:1977 | 05112019\_RID\_1313\_NaNaRj\_F5.raw |  | 5.15E4 | 9.04E3 |  | 1.1E5 |  |  |  |  |  | 3 | 0 | 1 | 1 | 0 | 1 | 0 | 0 | 0 | 0 | 0 | 318 | 330 |  |
| K.GSC(+57.02)GIGGGIGGGSSR.I | N | 79.96 | 1277.5782 | 15 | 1.4 | 639.7973 | 2 | 11.11 | 5 | F5:1952 | 05112019\_RID\_1313\_NaNaRj\_F5.raw |  | 4.15E5 | 2.34E4 |  | 6.04E4 |  |  |  |  |  | 3 | 0 | 1 | 1 | 0 | 1 | 0 | 0 | 0 | 0 | 0 | 16 | 30 | Carbamidomethylation |
| K.TEELNKEVASNSELVQSSR.S | Y | 78.88 | 2119.0393 | 19 | 0.9 | 707.3544 | 3 | 11.41 | 3 | F3:2214 | 05112019\_RID\_1313\_NaNaRj\_F3.raw |  |  | 2.81E4 |  |  |  |  |  |  |  | 1 | 0 | 0 | 1 | 0 | 0 | 0 | 0 | 0 | 0 | 0 | 312 | 330 |  |
| R.ALEEANADLEVK.I | N | 77.48 | 1300.6510 | 12 | -0.4 | 651.3325 | 2 | 11.60 | 3 | F3:2384 | 05112019\_RID\_1313\_NaNaRj\_F3.raw |  | 8.85E5 | 2.04E5 |  | 2.3E5 |  |  |  |  | 4.99E4 | 4 | 0 | 1 | 1 | 0 | 1 | 0 | 0 | 0 | 0 | 1 | 137 | 148 |  |
| R.GQTGGDVNVEM(+15.99)DAAPGVDLSR.I | Y | 76.39 | 2102.9539 | 21 | 0.6 | 1052.4849 | 2 | 11.86 | 10 | F10:2340 | 05112019\_RID\_1313\_NaNaRj\_F10.raw |  |  | 6.14E4 |  |  |  |  |  |  | 1.76E4 | 2 | 0 | 0 | 1 | 0 | 0 | 0 | 0 | 0 | 0 | 1 | 264 | 284 | Oxidation (M) |
| R.LLEGEDAHLSSQQASGQSYSSR.E | Y | 75.41 | 2349.0833 | 22 | 0.5 | 784.0354 | 3 | 11.32 | 5 | F5:2178 | 05112019\_RID\_1313\_NaNaRj\_F5.raw |  |  |  |  | 4.24E4 |  |  |  |  |  | 1 | 0 | 0 | 0 | 0 | 1 | 0 | 0 | 0 | 0 | 0 | 420 | 441 |  |
| R.GQTGGDVNVEMDAAPGVDLSR.I | Y | 71.24 | 2086.9590 | 21 | 0.5 | 1044.4873 | 2 | 12.85 | 3 | F3:2951 | 05112019\_RID\_1313\_NaNaRj\_F3.raw |  |  | 1.7E4 |  |  |  |  |  |  |  | 1 | 0 | 0 | 1 | 0 | 0 | 0 | 0 | 0 | 0 | 0 | 264 | 284 |  |
| K.VTMQNLNDR.L | N | 68.03 | 1089.5237 | 9 | 0.5 | 545.7694 | 2 | 11.19 | 5 | F5:2040 | 05112019\_RID\_1313\_NaNaRj\_F5.raw |  |  | 1.98E5 |  | 2.56E5 |  |  |  |  | 4.68E4 | 3 | 0 | 0 | 1 | 0 | 1 | 0 | 0 | 0 | 0 | 1 | 119 | 127 |  |
| R.ISSVLAGGSC(+57.02)R.A | N | 66.82 | 1105.5549 | 11 | -0.6 | 553.7844 | 2 | 11.32 | 5 | F5:2186 | 05112019\_RID\_1313\_NaNaRj\_F5.raw |  |  | 3.06E4 |  | 2.29E5 |  |  |  |  |  | 2 | 0 | 0 | 1 | 0 | 1 | 0 | 0 | 0 | 0 | 0 | 31 | 41 | Carbamidomethylation |
| K.ASLENSLEETK.G | N | 65.33 | 1219.5931 | 11 | 1.0 | 610.8044 | 2 | 11.45 | 3 | F3:2249 | 05112019\_RID\_1313\_NaNaRj\_F3.raw |  | 6.65E5 | 5.9E4 |  |  |  |  |  |  |  | 2 | 0 | 1 | 1 | 0 | 0 | 0 | 0 | 0 | 0 | 0 | 355 | 365 |  |
| R.QTVEADVNGLR.R | Y | 63.71 | 1200.6099 | 11 | 0.6 | 601.3126 | 2 | 11.53 | 5 | F5:2382 | 05112019\_RID\_1313\_NaNaRj\_F5.raw |  |  | 8.98E3 |  | 0 |  |  |  |  |  | 2 | 0 | 0 | 1 | 0 | 1 | 0 | 0 | 0 | 0 | 0 | 214 | 224 |  |
| R.LEQEIATYR.R | N | 59.43 | 1121.5717 | 9 | -0.1 | 561.7930 | 2 | 11.44 | 5 | F5:2290 | 05112019\_RID\_1313\_NaNaRj\_F5.raw |  | 1.62E6 | 3.06E4 |  | 4.88E5 |  |  |  |  | 1.13E4 | 4 | 0 | 1 | 1 | 0 | 1 | 0 | 0 | 0 | 0 | 1 | 410 | 418 |  |
| R.VLDELTLAR.T | N | 57.93 | 1028.5865 | 9 | -0.6 | 515.3002 | 2 | 13.17 | 5 | F5:3428 | 05112019\_RID\_1313\_NaNaRj\_F5.raw |  |  | 2.21E5 | 7.14E4 | 4.87E5 | 1.16E5 | 6.95E4 | 2.69E4 |  | 2.37E4 | 7 | 0 | 0 | 1 | 1 | 1 | 1 | 1 | 1 | 0 | 1 | 226 | 234 |  |
| K.IIAATIENAQPILQIDNAR.L | Y | 57.07 | 2063.1375 | 19 | 0.4 | 1032.5764 | 2 | 23.18 | 4 | F4:7853 | 05112019\_RID\_1313\_NaNaRj\_F4.raw |  |  |  | 2.75E4 |  |  | 8.08E4 | 2.3E4 |  |  | 4 | 0 | 0 | 0 | 1 | 0 | 0 | 1 | 2 | 0 | 0 | 178 | 196 |  |
| R.DAETWFLSK.T | Y | 56.88 | 1095.5237 | 9 | 0.0 | 548.7691 | 2 | 62.56 | 2 | F2:20651 | 09012020\_NaNaRj\_F2.raw |  | 1.43E5 | 6.3E3 | 1.13E4 |  | 8.96E3 | 4.3E4 |  |  |  | 5 | 0 | 1 | 1 | 1 | 0 | 1 | 1 | 0 | 0 | 0 | 303 | 311 |  |
| L.IGSVEEQLAQLR.C | N | 52.90 | 1341.7252 | 12 | 0.3 | 671.8701 | 2 | 53.67 | 2 | F2:17947 | 09012020\_NaNaRj\_F2.raw |  | 5.83E4 |  |  | 4.4E4 |  |  |  |  |  | 2 | 0 | 1 | 0 | 0 | 1 | 0 | 0 | 0 | 0 | 0 | 379 | 390 |  |
| K.TRLEQEIATYR.R | N | 50.16 | 1378.7205 | 11 | 0.4 | 690.3678 | 2 | 32.95 | 2 | F2:9394 | 09012020\_NaNaRj\_F2.raw |  | 4.37E5 |  |  |  |  |  |  |  |  | 1 | 0 | 1 | 0 | 0 | 0 | 0 | 0 | 0 | 0 | 0 | 408 | 418 |  |
| R.LASYLDK.V | N | 47.75 | 808.4330 | 7 | 0.6 | 405.2241 | 2 | 11.34 | 5 | F5:2185 | 05112019\_RID\_1313\_NaNaRj\_F5.raw | 3.03E4 | 9.71E6 | 7.78E4 |  | 7.33E5 |  |  |  |  | 3.94E4 | 5 | 1 | 1 | 1 | 0 | 1 | 0 | 0 | 0 | 0 | 1 | 128 | 134 |  |
| R.LAADDFR.T | N | 46.83 | 806.3922 | 7 | 0.6 | 404.2036 | 2 | 11.46 | 5 | F5:2311 | 05112019\_RID\_1313\_NaNaRj\_F5.raw |  | 5.1E6 | 5.72E4 |  | 4.73E5 |  | 1.38E4 |  |  | 3.3E4 | 6 | 0 | 2 | 1 | 0 | 1 | 0 | 1 | 0 | 0 | 1 | 197 | 203 |  |
| R.LASYLDKVR.A | N | 45.41 | 1063.6025 | 9 | 1.0 | 532.8091 | 2 | 27.68 | 2 | F2:7299 | 09012020\_NaNaRj\_F2.raw |  | 2.48E5 |  |  |  |  |  |  |  |  | 1 | 0 | 1 | 0 | 0 | 0 | 0 | 0 | 0 | 0 | 0 | 128 | 136 |  |
| A.GGDGLLVGSEK.V | N | 42.45 | 1030.5294 | 11 | 0.8 | 516.2724 | 2 | 29.32 | 2 | F2:7957 | 09012020\_NaNaRj\_F2.raw |  | 7.7E4 |  |  |  |  |  |  |  |  | 1 | 0 | 1 | 0 | 0 | 0 | 0 | 0 | 0 | 0 | 0 | 108 | 118 |  |
| R.TKYEHELALR.Q | Y | 41.95 | 1258.6670 | 10 | -0.5 | 420.5627 | 3 | 18.41 | 2 | F2:3580 | 09012020\_NaNaRj\_F2.raw |  | 5.75E4 |  |  |  |  |  |  |  |  | 1 | 0 | 1 | 0 | 0 | 0 | 0 | 0 | 0 | 0 | 0 | 204 | 213 |  |
| K.VTM(+15.99)QNLNDR.L | N | 40.47 | 1105.5186 | 9 | 1.2 | 553.7672 | 2 | 17.56 | 2 | F2:3384 | 09012020\_NaNaRj\_F2.raw |  | 0 |  |  |  |  |  |  |  |  | 1 | 0 | 1 | 0 | 0 | 0 | 0 | 0 | 0 | 0 | 0 | 119 | 127 | Oxidation (M) |
| total 24 peptides |  |  |  |  |  |  |  |  |  |  |  |  |  |  |  |  |  |  |  |  |  |  |  |  |  |  |  |  |  |  |  |  |  |  |  |
| --- | --- | --- | --- | --- | --- | --- | --- | --- | --- | --- | --- | --- | --- | --- | --- | --- | --- | --- | --- | --- | --- | --- | --- | --- | --- | --- | --- | --- | --- | --- | --- | --- | --- | --- | --- |

P02533|K1C14\_HUMAN

back to list

  

| Protein Coverage
| Supporting Peptides
|

Protein Coverage:

Supporting Peptides:

| Peptide | Uniq | -10lgP | Mass | Length | ppm | m/z | z | RT | Fraction | Scan | Source File | Area F1 | Area F2 | Area F3 | Area F4 | Area F5 | Area F6 | Area F7 | Area F8 | Area F9 | Area F10 | #Feature | #Feature F1 | #Feature F2 | #Feature F3 | #Feature F4 | #Feature F5 | #Feature F6 | #Feature F7 | #Feature F8 | #Feature F9 | #Feature F10 | Start | End | PTM |
| --- | --- | --- | --- | --- | --- | --- | --- | --- | --- | --- | --- | --- | --- | --- | --- | --- | --- | --- | --- | --- | --- | --- | --- | --- | --- | --- | --- | --- | --- | --- | --- | --- | --- | --- | --- |
| R.APSTYGGGLSVSSSR.F | Y | 99.12 | 1424.6896 | 15 | 0.2 | 713.3522 | 2 | 11.37 | 3 | F3:2171 | 05112019\_RID\_1313\_NaNaRj\_F3.raw |  | 3.54E5 | 9.35E4 |  | 8.47E4 |  |  |  |  | 2.99E4 | 5 | 0 | 2 | 1 | 0 | 1 | 0 | 0 | 0 | 0 | 1 | 42 | 56 |  |
| R.EVATNSELVQSGK.S | N | 80.27 | 1360.6833 | 13 | 0.4 | 681.3492 | 2 | 11.13 | 3 | F3:1953 | 05112019\_RID\_1313\_NaNaRj\_F3.raw |  | 5.22E5 | 6.8E4 |  | 8.04E4 |  |  |  |  |  | 3 | 0 | 1 | 1 | 0 | 1 | 0 | 0 | 0 | 0 | 0 | 316 | 328 |  |
| K.GSC(+57.02)GIGGGIGGGSSR.I | N | 79.96 | 1277.5782 | 15 | 1.4 | 639.7973 | 2 | 11.11 | 5 | F5:1952 | 05112019\_RID\_1313\_NaNaRj\_F5.raw |  | 4.15E5 | 2.34E4 |  | 6.04E4 |  |  |  |  |  | 3 | 0 | 1 | 1 | 0 | 1 | 0 | 0 | 0 | 0 | 0 | 16 | 30 | Carbamidomethylation |
| R.ALEEANADLEVK.I | N | 77.48 | 1300.6510 | 12 | -0.4 | 651.3325 | 2 | 11.60 | 3 | F3:2384 | 05112019\_RID\_1313\_NaNaRj\_F3.raw |  | 8.85E5 | 2.04E5 |  | 2.3E5 |  |  |  |  | 4.99E4 | 4 | 0 | 1 | 1 | 0 | 1 | 0 | 0 | 0 | 0 | 1 | 135 | 146 |  |
| R.GQVGGDVNVEM(+15.99)DAAPGVDLSR.I | N | 75.25 | 2100.9746 | 21 | 0.6 | 1051.4952 | 2 | 12.10 | 3 | F3:2730 | 05112019\_RID\_1313\_NaNaRj\_F3.raw |  |  | 6.41E4 |  | 2.14E4 |  |  |  |  | 7.34E3 | 3 | 0 | 0 | 1 | 0 | 1 | 0 | 0 | 0 | 0 | 1 | 262 | 282 | Oxidation (M) |
| K.VTMQNLNDR.L | N | 68.03 | 1089.5237 | 9 | 0.5 | 545.7694 | 2 | 11.19 | 5 | F5:2040 | 05112019\_RID\_1313\_NaNaRj\_F5.raw |  |  | 1.98E5 |  | 2.56E5 |  |  |  |  | 4.68E4 | 3 | 0 | 0 | 1 | 0 | 1 | 0 | 0 | 0 | 0 | 1 | 117 | 125 |  |
| R.ISSVLAGGSC(+57.02)R.A | N | 66.82 | 1105.5549 | 11 | -0.6 | 553.7844 | 2 | 11.32 | 5 | F5:2186 | 05112019\_RID\_1313\_NaNaRj\_F5.raw |  |  | 3.06E4 |  | 2.29E5 |  |  |  |  |  | 2 | 0 | 0 | 1 | 0 | 1 | 0 | 0 | 0 | 0 | 0 | 31 | 41 | Carbamidomethylation |
| K.DAEEWFFTK.T | Y | 66.81 | 1171.5186 | 9 | -0.1 | 586.7665 | 2 | 24.10 | 4 | F4:8047 | 05112019\_RID\_1313\_NaNaRj\_F4.raw |  | 1.37E6 |  | 4.58E4 | 2.58E4 |  | 1.85E4 |  |  |  | 5 | 0 | 1 | 0 | 2 | 1 | 0 | 1 | 0 | 0 | 0 | 301 | 309 |  |
| K.ASLENSLEETK.G | N | 65.33 | 1219.5931 | 11 | 1.0 | 610.8044 | 2 | 11.45 | 3 | F3:2249 | 05112019\_RID\_1313\_NaNaRj\_F3.raw |  | 6.65E5 | 5.9E4 |  |  |  |  |  |  |  | 2 | 0 | 1 | 1 | 0 | 0 | 0 | 0 | 0 | 0 | 0 | 353 | 363 |  |
| R.LEQEIATYR.R | N | 59.43 | 1121.5717 | 9 | -0.1 | 561.7930 | 2 | 11.44 | 5 | F5:2290 | 05112019\_RID\_1313\_NaNaRj\_F5.raw |  | 1.62E6 | 3.06E4 |  | 4.88E5 |  |  |  |  | 1.13E4 | 4 | 0 | 1 | 1 | 0 | 1 | 0 | 0 | 0 | 0 | 1 | 408 | 416 |  |
| R.VLDELTLAR.A | N | 57.93 | 1028.5865 | 9 | -0.6 | 515.3002 | 2 | 13.17 | 5 | F5:3428 | 05112019\_RID\_1313\_NaNaRj\_F5.raw |  |  | 2.21E5 | 7.14E4 | 4.87E5 | 1.16E5 | 6.95E4 | 2.69E4 |  | 2.37E4 | 7 | 0 | 0 | 1 | 1 | 1 | 1 | 1 | 1 | 0 | 1 | 224 | 232 |  |
| R.LLEGEDAHLSSSQFSSGSQSSR.D | Y | 56.28 | 2308.0566 | 22 | 0.5 | 770.3599 | 3 | 35.97 | 2 | F2:10702 | 09012020\_NaNaRj\_F2.raw |  | 2.4E5 |  |  | 2.24E4 |  |  |  |  |  | 2 | 0 | 1 | 0 | 0 | 1 | 0 | 0 | 0 | 0 | 0 | 418 | 439 |  |
| K.ILTATVDNANVLLQIDNAR.L | Y | 52.97 | 2053.1167 | 19 | 1.0 | 1027.5667 | 2 | 27.29 | 4 | F4:8673 | 05112019\_RID\_1313\_NaNaRj\_F4.raw |  |  |  | 3.17E4 |  | 6.45E4 |  | 2.45E4 |  |  | 4 | 0 | 0 | 0 | 1 | 0 | 2 | 0 | 1 | 0 | 0 | 176 | 194 |  |
| M.IGSVEEQLAQLR.C | N | 52.90 | 1341.7252 | 12 | 0.3 | 671.8701 | 2 | 53.67 | 2 | F2:17947 | 09012020\_NaNaRj\_F2.raw |  | 5.83E4 |  |  | 4.4E4 |  |  |  |  |  | 2 | 0 | 1 | 0 | 0 | 1 | 0 | 0 | 0 | 0 | 0 | 377 | 388 |  |
| K.TRLEQEIATYR.R | N | 50.16 | 1378.7205 | 11 | 0.4 | 690.3678 | 2 | 32.95 | 2 | F2:9394 | 09012020\_NaNaRj\_F2.raw |  | 4.37E5 |  |  |  |  |  |  |  |  | 1 | 0 | 1 | 0 | 0 | 0 | 0 | 0 | 0 | 0 | 0 | 406 | 416 |  |
| R.LASYLDK.V | N | 47.75 | 808.4330 | 7 | 0.6 | 405.2241 | 2 | 11.34 | 5 | F5:2185 | 05112019\_RID\_1313\_NaNaRj\_F5.raw | 3.03E4 | 9.71E6 | 7.78E4 |  | 7.33E5 |  |  |  |  | 3.94E4 | 5 | 1 | 1 | 1 | 0 | 1 | 0 | 0 | 0 | 0 | 1 | 126 | 132 |  |
| R.TKYETELNLR.M | Y | 46.91 | 1265.6615 | 10 | 0.4 | 422.8946 | 3 | 27.69 | 2 | F2:7353 | 09012020\_NaNaRj\_F2.raw |  | 4.14E4 |  |  |  |  |  |  |  |  | 1 | 0 | 1 | 0 | 0 | 0 | 0 | 0 | 0 | 0 | 0 | 202 | 211 |  |
| R.LAADDFR.T | N | 46.83 | 806.3922 | 7 | 0.6 | 404.2036 | 2 | 11.46 | 5 | F5:2311 | 05112019\_RID\_1313\_NaNaRj\_F5.raw |  | 5.1E6 | 5.72E4 |  | 4.73E5 |  | 1.38E4 |  |  | 3.3E4 | 6 | 0 | 2 | 1 | 0 | 1 | 0 | 1 | 0 | 0 | 1 | 195 | 201 |  |
| R.LASYLDKVR.A | N | 45.41 | 1063.6025 | 9 | 1.0 | 532.8091 | 2 | 27.68 | 2 | F2:7299 | 09012020\_NaNaRj\_F2.raw |  | 2.48E5 |  |  |  |  |  |  |  |  | 1 | 0 | 1 | 0 | 0 | 0 | 0 | 0 | 0 | 0 | 0 | 126 | 134 |  |
| K.NHEEEMNALR.G | N | 43.68 | 1241.5459 | 10 | 0.1 | 621.7803 | 2 | 14.43 | 2 | F2:2709 | 09012020\_NaNaRj\_F2.raw |  | 1.14E5 |  |  |  |  |  |  |  |  | 1 | 0 | 1 | 0 | 0 | 0 | 0 | 0 | 0 | 0 | 0 | 252 | 261 |  |
| A.GGDGLLVGSEK.V | N | 42.45 | 1030.5294 | 11 | 0.8 | 516.2724 | 2 | 29.32 | 2 | F2:7957 | 09012020\_NaNaRj\_F2.raw |  | 7.7E4 |  |  |  |  |  |  |  |  | 1 | 0 | 1 | 0 | 0 | 0 | 0 | 0 | 0 | 0 | 0 | 106 | 116 |  |
| R.MSVEADINGLR.R | N | 41.73 | 1203.5918 | 11 | 0.7 | 602.8036 | 2 | 46.59 | 2 | F2:15175 | 09012020\_NaNaRj\_F2.raw |  | 1.13E5 |  |  |  |  |  |  |  |  | 1 | 0 | 1 | 0 | 0 | 0 | 0 | 0 | 0 | 0 | 0 | 212 | 222 |  |
| K.VTM(+15.99)QNLNDR.L | N | 40.47 | 1105.5186 | 9 | 1.2 | 553.7672 | 2 | 17.56 | 2 | F2:3384 | 09012020\_NaNaRj\_F2.raw |  | 0 |  |  |  |  |  |  |  |  | 1 | 0 | 1 | 0 | 0 | 0 | 0 | 0 | 0 | 0 | 0 | 117 | 125 | Oxidation (M) |
| total 23 peptides |  |  |  |  |  |  |  |  |  |  |  |  |  |  |  |  |  |  |  |  |  |  |  |  |  |  |  |  |  |  |  |  |  |  |  |
| --- | --- | --- | --- | --- | --- | --- | --- | --- | --- | --- | --- | --- | --- | --- | --- | --- | --- | --- | --- | --- | --- | --- | --- | --- | --- | --- | --- | --- | --- | --- | --- | --- | --- | --- | --- |

P13647|K2C5\_HUMAN

back to list

  

| Protein Coverage
| Supporting Peptides
|

Protein Coverage:

Supporting Peptides:

| Peptide | Uniq | -10lgP | Mass | Length | ppm | m/z | z | RT | Fraction | Scan | Source File | Area F1 | Area F2 | Area F3 | Area F4 | Area F5 | Area F6 | Area F7 | Area F8 | Area F9 | Area F10 | #Feature | #Feature F1 | #Feature F2 | #Feature F3 | #Feature F4 | #Feature F5 | #Feature F6 | #Feature F7 | #Feature F8 | #Feature F9 | #Feature F10 | Start | End | PTM |
| --- | --- | --- | --- | --- | --- | --- | --- | --- | --- | --- | --- | --- | --- | --- | --- | --- | --- | --- | --- | --- | --- | --- | --- | --- | --- | --- | --- | --- | --- | --- | --- | --- | --- | --- | --- |
| R.VSLAGAC(+57.02)GVGGYGSR.S | Y | 79.63 | 1409.6721 | 15 | 0.6 | 705.8438 | 2 | 11.65 | 3 | F3:2425 | 05112019\_RID\_1313\_NaNaRj\_F3.raw |  | 4.74E5 | 5.69E4 |  | 7.62E4 |  |  |  |  | 5.7E3 | 4 | 0 | 1 | 1 | 0 | 1 | 0 | 0 | 0 | 0 | 1 | 49 | 63 | Carbamidomethylation |
| R.NLDLDSIIAEVK.A | N | 78.64 | 1328.7188 | 12 | 0.5 | 665.3670 | 2 | 35.93 | 7 | F7:9727 | 05112019\_RID\_1313\_NaNaRj\_F7.raw |  |  |  | 6.48E5 | 5.9E4 | 9.04E5 | 6.22E5 | 3.5E5 |  |  | 5 | 0 | 0 | 0 | 1 | 1 | 1 | 1 | 1 | 0 | 0 | 332 | 343 |  |
| R.SFSTASAITPSVSR.T | N | 76.56 | 1409.7151 | 14 | -0.1 | 705.8647 | 2 | 40.34 | 2 | F2:12553 | 09012020\_NaNaRj\_F2.raw |  | 1.19E5 | 3.97E4 |  | 3.46E4 |  |  |  |  |  | 3 | 0 | 1 | 1 | 0 | 1 | 0 | 0 | 0 | 0 | 0 | 16 | 29 |  |
| R.GLGVGFGSGGGSSSSVK.F | Y | 72.33 | 1438.7052 | 17 | 0.7 | 720.3604 | 2 | 11.70 | 3 | F3:2469 | 05112019\_RID\_1313\_NaNaRj\_F3.raw |  |  | 1.01E4 |  |  |  |  |  |  |  | 1 | 0 | 0 | 1 | 0 | 0 | 0 | 0 | 0 | 0 | 0 | 560 | 576 |  |
| K.AQYEEIANR.S | N | 68.34 | 1092.5199 | 9 | 0.9 | 547.2677 | 2 | 11.09 | 10 | F10:1783 | 05112019\_RID\_1313\_NaNaRj\_F10.raw |  |  | 3.99E4 |  | 7.48E4 |  |  |  |  | 1.38E4 | 3 | 0 | 0 | 1 | 0 | 1 | 0 | 0 | 0 | 0 | 1 | 344 | 352 |  |
| R.TEAESWYQTK.Y | N | 67.78 | 1241.5564 | 10 | 0.3 | 621.7856 | 2 | 11.45 | 3 | F3:2241 | 05112019\_RID\_1313\_NaNaRj\_F3.raw |  | 6.73E5 | 1.58E4 |  | 8.48E4 |  |  |  |  | 6.41E3 | 4 | 0 | 1 | 1 | 0 | 1 | 0 | 0 | 0 | 0 | 1 | 355 | 364 |  |
| K.NKYEDEINKR.T | N | 67.54 | 1307.6470 | 10 | 0.5 | 436.8898 | 3 | 12.46 | 2 | F2:2012 | 09012020\_NaNaRj\_F2.raw |  | 4.15E6 |  |  | 2.01E5 |  |  |  |  |  | 3 | 0 | 2 | 0 | 0 | 1 | 0 | 0 | 0 | 0 | 0 | 256 | 265 |  |
| K.LALDVEIATYR.K | N | 67.43 | 1262.6870 | 11 | 0.5 | 632.3511 | 2 | 18.91 | 6 | F6:7379 | 05112019\_RID\_1313\_NaNaRj\_F6.raw |  |  |  | 8.76E4 |  | 3.23E5 | 1.04E5 |  |  |  | 4 | 0 | 0 | 0 | 1 | 0 | 2 | 1 | 0 | 0 | 0 | 461 | 471 |  |
| K.YEELQQTAGR.H | N | 67.42 | 1193.5676 | 10 | 0.1 | 597.7911 | 2 | 11.02 | 5 | F5:1895 | 05112019\_RID\_1313\_NaNaRj\_F5.raw |  | 4.41E5 | 2.42E4 |  | 4.26E4 |  |  |  |  | 6.51E3 | 4 | 0 | 1 | 1 | 0 | 1 | 0 | 0 | 0 | 0 | 1 | 365 | 374 |  |
| R.ISISTSGGSFR.N | Y | 61.36 | 1110.5669 | 11 | -0.7 | 556.2903 | 2 | 35.56 | 2 | F2:10520 | 09012020\_NaNaRj\_F2.raw |  | 1.83E6 | 7.72E4 |  | 6.14E4 |  |  |  |  | 1.41E4 | 6 | 0 | 3 | 1 | 0 | 1 | 0 | 0 | 0 | 0 | 1 | 74 | 84 |  |
| K.WTLLQEQGTK.T | N | 56.02 | 1202.6295 | 10 | 0.6 | 602.3224 | 2 | 12.51 | 5 | F5:3187 | 05112019\_RID\_1313\_NaNaRj\_F5.raw |  | 1.05E6 |  |  | 9.31E4 |  |  |  |  |  | 2 | 0 | 1 | 0 | 0 | 1 | 0 | 0 | 0 | 0 | 0 | 200 | 209 |  |
| K.VDALM(+15.99)DEINFMK.M | N | 54.67 | 1440.6628 | 12 | 0.7 | 721.3392 | 2 | 62.85 | 2 | F2:20750 | 09012020\_NaNaRj\_F2.raw |  | 3.29E4 |  |  |  |  |  |  |  |  | 1 | 0 | 1 | 0 | 0 | 0 | 0 | 0 | 0 | 0 | 0 | 293 | 304 | Oxidation (M) |
| R.QLDSIVGER.G | N | 54.54 | 1015.5298 | 9 | 0.6 | 508.7725 | 2 | 11.56 | 3 | F3:2354 | 05112019\_RID\_1313\_NaNaRj\_F3.raw |  | 1.03E6 | 6.68E4 |  | 1.52E5 |  |  |  |  | 2.38E4 | 4 | 0 | 1 | 1 | 0 | 1 | 0 | 0 | 0 | 0 | 1 | 229 | 237 |  |
| K.LLEGEEC(+57.02)R.L | N | 54.31 | 1004.4597 | 8 | -0.6 | 503.2368 | 2 | 11.02 | 5 | F5:1874 | 05112019\_RID\_1313\_NaNaRj\_F5.raw |  |  |  |  | 1.16E5 |  |  |  |  |  | 1 | 0 | 0 | 0 | 0 | 1 | 0 | 0 | 0 | 0 | 0 | 473 | 480 | Carbamidomethylation |
| K.LAELEEALQK.A | Y | 53.32 | 1142.6183 | 10 | 0.7 | 572.3168 | 2 | 12.15 | 5 | F5:2950 | 05112019\_RID\_1313\_NaNaRj\_F5.raw | 5.18E3 | 2.27E6 | 2.86E4 |  | 1.58E5 |  |  |  |  | 7.41E3 | 8 | 1 | 4 | 1 | 0 | 1 | 0 | 0 | 0 | 0 | 1 | 432 | 441 |  |
| R.QNLEPLFEQYINNLR.R | N | 52.74 | 1889.9635 | 15 | 0.8 | 630.9956 | 3 | 41.57 | 6 | F6:17761 | 05112019\_RID\_1313\_NaNaRj\_F6.raw |  |  |  |  |  | 5.84E4 |  |  |  |  | 1 | 0 | 0 | 0 | 0 | 0 | 1 | 0 | 0 | 0 | 0 | 213 | 227 |  |
| R.LSGEGVGPVNIS.V | N | 52.12 | 1127.5823 | 12 | -0.3 | 564.7982 | 2 | 52.35 | 2 | F2:17477 | 09012020\_NaNaRj\_F2.raw |  | 1.63E5 |  |  |  |  |  |  |  |  | 1 | 0 | 1 | 0 | 0 | 0 | 0 | 0 | 0 | 0 | 0 | 481 | 492 |  |
| R.SLYNLGGSK.R | N | 51.63 | 937.4869 | 9 | 0.7 | 469.7510 | 2 | 31.03 | 2 | F2:8692 | 09012020\_NaNaRj\_F2.raw |  | 6.59E5 |  |  | 2.21E4 |  |  |  |  |  | 2 | 0 | 1 | 0 | 0 | 1 | 0 | 0 | 0 | 0 | 0 | 64 | 72 |  |
| K.DVDAAYMNK.V | N | 48.86 | 1025.4487 | 9 | 0.7 | 513.7320 | 2 | 11.29 | 3 | F3:2108 | 05112019\_RID\_1313\_NaNaRj\_F3.raw |  |  | 3.06E3 |  |  |  |  |  |  |  | 1 | 0 | 0 | 1 | 0 | 0 | 0 | 0 | 0 | 0 | 0 | 278 | 286 |  |
| R.NMQDLVEDFK.N | N | 48.42 | 1237.5648 | 10 | 0.1 | 619.7897 | 2 | 59.86 | 2 | F2:19843 | 09012020\_NaNaRj\_F2.raw |  | 2.42E5 |  |  |  |  |  |  |  |  | 1 | 0 | 1 | 0 | 0 | 0 | 0 | 0 | 0 | 0 | 0 | 246 | 255 |  |
| R.EYQELMNTK.L | N | 48.01 | 1154.5277 | 9 | -0.2 | 578.2710 | 2 | 29.29 | 2 | F2:7914 | 09012020\_NaNaRj\_F2.raw |  | 6.6E5 |  |  |  |  |  |  |  |  | 1 | 0 | 1 | 0 | 0 | 0 | 0 | 0 | 0 | 0 | 0 | 452 | 460 |  |
| R.TTAENEFVMLK.K | N | 47.65 | 1281.6274 | 11 | 1.0 | 641.8217 | 2 | 49.66 | 2 | F2:16423 | 09012020\_NaNaRj\_F2.raw |  | 7.86E5 | 1.14E4 |  | 1.62E4 |  |  |  |  |  | 3 | 0 | 1 | 1 | 0 | 1 | 0 | 0 | 0 | 0 | 0 | 266 | 276 |  |
| K.FASFIDK.V | N | 47.57 | 826.4225 | 7 | 0.0 | 414.2185 | 2 | 39.16 | 2 | F2:12055 | 09012020\_NaNaRj\_F2.raw | 6.35E3 | 4.99E6 |  |  | 4.92E5 |  |  |  | 5.38E3 |  | 4 | 1 | 1 | 0 | 0 | 1 | 0 | 0 | 0 | 1 | 0 | 179 | 185 |  |
| R.TTAENEFVM(+15.99)LK.K | N | 44.36 | 1297.6224 | 11 | 0.7 | 649.8190 | 2 | 11.96 | 5 | F5:2797 | 05112019\_RID\_1313\_NaNaRj\_F5.raw |  |  |  |  | 4.67E4 |  |  |  |  |  | 1 | 0 | 0 | 0 | 0 | 1 | 0 | 0 | 0 | 0 | 0 | 266 | 276 | Oxidation (M) |
| A.SAITPSVSR.T | N | 40.17 | 916.4978 | 9 | -0.1 | 459.2561 | 2 | 19.97 | 2 | F2:4211 | 09012020\_NaNaRj\_F2.raw |  | 7.86E4 |  |  |  |  |  |  |  |  | 1 | 0 | 1 | 0 | 0 | 0 | 0 | 0 | 0 | 0 | 0 | 21 | 29 |  |
| total 25 peptides |  |  |  |  |  |  |  |  |  |  |  |  |  |  |  |  |  |  |  |  |  |  |  |  |  |  |  |  |  |  |  |  |  |  |  |
| --- | --- | --- | --- | --- | --- | --- | --- | --- | --- | --- | --- | --- | --- | --- | --- | --- | --- | --- | --- | --- | --- | --- | --- | --- | --- | --- | --- | --- | --- | --- | --- | --- | --- | --- | --- |

P25498|PA2AE\_NAJOX

back to list

  

| Protein Coverage
| Supporting Peptides
|

Protein Coverage:

Supporting Peptides:

| Peptide | Uniq | -10lgP | Mass | Length | ppm | m/z | z | RT | Fraction | Scan | Source File | Area F1 | Area F2 | Area F3 | Area F4 | Area F5 | Area F6 | Area F7 | Area F8 | Area F9 | Area F10 | #Feature | #Feature F1 | #Feature F2 | #Feature F3 | #Feature F4 | #Feature F5 | #Feature F6 | #Feature F7 | #Feature F8 | #Feature F9 | #Feature F10 | Start | End | PTM |
| --- | --- | --- | --- | --- | --- | --- | --- | --- | --- | --- | --- | --- | --- | --- | --- | --- | --- | --- | --- | --- | --- | --- | --- | --- | --- | --- | --- | --- | --- | --- | --- | --- | --- | --- | --- |
| K.TYSYEC(+57.02)SQGTLTC(+57.02)K.G | N | 113.63 | 1696.7073 | 14 | 0.8 | 849.3616 | 2 | 11.36 | 7 | F7:2195 | 05112019\_RID\_1313\_NaNaRj\_F7.raw |  |  |  |  | 5.87E4 | 1.81E8 | 9.34E6 | 6.34E5 | 5.1E4 | 1.71E4 | 13 | 0 | 0 | 0 | 0 | 1 | 8 | 1 | 1 | 1 | 1 | 66 | 79 | Carbamidomethylation |
| T.YSYEC(+57.02)SQGTLTC(+57.02)K.G | N | 83.50 | 1595.6595 | 13 | -1.9 | 798.8356 | 2 | 11.31 | 8 | F8:2155 | 05112019\_RID\_1313\_NaNaRj\_F8.raw |  |  |  |  |  |  | 4.19E4 | 2.33E5 |  |  | 2 | 0 | 0 | 0 | 0 | 0 | 0 | 1 | 1 | 0 | 0 | 67 | 79 | Carbamidomethylation |
| Y.SYEC(+57.02)SQGTLTC(+57.02)K.G | N | 80.85 | 1432.5963 | 12 | -0.8 | 717.3049 | 2 | 10.99 | 8 | F8:1884 | 05112019\_RID\_1313\_NaNaRj\_F8.raw |  |  |  |  |  | 2.31E5 | 2.11E5 | 8.52E5 |  |  | 3 | 0 | 0 | 0 | 0 | 0 | 1 | 1 | 1 | 0 | 0 | 68 | 79 | Carbamidomethylation |
| R.GGSGTPVDDLDR.C | N | 79.15 | 1187.5417 | 12 | -1.8 | 594.7771 | 2 | 11.41 | 6 | F6:2201 | 05112019\_RID\_1313\_NaNaRj\_F6.raw |  |  |  |  |  | 5.21E7 |  |  | 4.03E4 |  | 4 | 0 | 0 | 0 | 0 | 0 | 3 | 0 | 0 | 1 | 0 | 31 | 42 |  |
| K.ISGC(+57.02)WPYFKTYSYEC(+57.02)SQGTLTC(+57.02)K.G | N | 75.72 | 2835.2341 | 23 | 0.4 | 946.0857 | 3 | 15.40 | 6 | F6:5076 | 05112019\_RID\_1313\_NaNaRj\_F6.raw |  |  |  |  |  | 8.77E5 |  |  |  |  | 1 | 0 | 0 | 0 | 0 | 0 | 1 | 0 | 0 | 0 | 0 | 57 | 79 | Carbamidomethylation |
| K.ISGC(+57.02)WPYFK.T | N | 74.23 | 1156.5375 | 9 | 1.7 | 579.2770 | 2 | 16.27 | 6 | F6:5756 | 05112019\_RID\_1313\_NaNaRj\_F6.raw |  |  |  |  |  | 3.02E8 | 8.06E6 | 1.78E6 |  |  | 12 | 0 | 0 | 0 | 0 | 0 | 8 | 3 | 1 | 0 | 0 | 57 | 65 | Carbamidomethylation |
| K.TYSYEC(+57.02)SQGTLTC(+57.02)KG.D | N | 70.12 | 1753.7288 | 15 | -0.1 | 877.8716 | 2 | 11.39 | 6 | F6:2169 | 05112019\_RID\_1313\_NaNaRj\_F6.raw |  |  |  |  |  | 5.33E7 | 4.5E6 |  |  |  | 2 | 0 | 0 | 0 | 0 | 0 | 1 | 1 | 0 | 0 | 0 | 66 | 80 | Carbamidomethylation |
| S.YEC(+57.02)SQGTLTC(+57.02)K.G | N | 61.39 | 1345.5642 | 11 | 0.4 | 673.7897 | 2 | 10.92 | 8 | F8:1818 | 05112019\_RID\_1313\_NaNaRj\_F8.raw |  |  |  |  |  |  |  | 2.81E4 |  |  | 1 | 0 | 0 | 0 | 0 | 0 | 0 | 0 | 1 | 0 | 0 | 69 | 79 | Carbamidomethylation |
| R.LAAIC(+57.02)FAGAPYN.N | N | 53.65 | 1266.6067 | 12 | 0.3 | 634.3108 | 2 | 25.26 | 7 | F7:7409 | 05112019\_RID\_1313\_NaNaRj\_F7.raw |  |  |  |  |  | 8.75E5 | 2.92E6 |  |  |  | 2 | 0 | 0 | 0 | 0 | 0 | 1 | 1 | 0 | 0 | 0 | 95 | 106 | Carbamidomethylation |
| R.LAAIC(+57.02)FAGAPY.N | N | 51.44 | 1152.5637 | 11 | 0.2 | 577.2892 | 2 | 30.44 | 8 | F8:11368 | 05112019\_RID\_1313\_NaNaRj\_F8.raw |  |  |  |  |  |  |  | 1.27E6 |  |  | 1 | 0 | 0 | 0 | 0 | 0 | 0 | 0 | 1 | 0 | 0 | 95 | 105 | Carbamidomethylation |
| R.SWLDFANYGC(+57.02)YC(+57.02)GRGGSGTPVDDLDR.C | Y | 41.08 | 2937.2446 | 26 | -6.3 | 980.0826 | 3 | 43.14 | 8 | F8:16804 | 05112019\_RID\_1313\_NaNaRj\_F8.raw |  |  |  |  |  |  |  | 4.39E4 |  |  | 1 | 0 | 0 | 0 | 0 | 0 | 0 | 0 | 1 | 0 | 0 | 17 | 42 | Carbamidomethylation |
| total 11 peptides |  |  |  |  |  |  |  |  |  |  |  |  |  |  |  |  |  |  |  |  |  |  |  |  |  |  |  |  |  |  |  |  |  |  |  |
| --- | --- | --- | --- | --- | --- | --- | --- | --- | --- | --- | --- | --- | --- | --- | --- | --- | --- | --- | --- | --- | --- | --- | --- | --- | --- | --- | --- | --- | --- | --- | --- | --- | --- | --- | --- |

P25668|3L21\_NAJNA

back to list

  

| Protein Coverage
| Supporting Peptides
|

Protein Coverage:

Supporting Peptides:

| Peptide | Uniq | -10lgP | Mass | Length | ppm | m/z | z | RT | Fraction | Scan | Source File | Area F1 | Area F2 | Area F3 | Area F4 | Area F5 | Area F6 | Area F7 | Area F8 | Area F9 | Area F10 | #Feature | #Feature F1 | #Feature F2 | #Feature F3 | #Feature F4 | #Feature F5 | #Feature F6 | #Feature F7 | #Feature F8 | #Feature F9 | #Feature F10 | Start | End | PTM |
| --- | --- | --- | --- | --- | --- | --- | --- | --- | --- | --- | --- | --- | --- | --- | --- | --- | --- | --- | --- | --- | --- | --- | --- | --- | --- | --- | --- | --- | --- | --- | --- | --- | --- | --- | --- |
| R.VDLGC(+57.02)AATC(+57.02)PTVR.T | N | 110.91 | 1418.6646 | 13 | 0.3 | 710.3398 | 2 | 11.61 | 4 | F4:2429 | 05112019\_RID\_1313\_NaNaRj\_F4.raw |  |  |  | 9.59E6 | 2.85E4 | 0 | 6.81E4 | 0 |  | 3.03E4 | 6 | 0 | 0 | 0 | 1 | 1 | 1 | 1 | 1 | 0 | 1 | 37 | 49 | Carbamidomethylation |
| R.C(+57.02)FITPDITSKDC(+57.02)PNGHVC(+57.02)YTK.T | N | 90.07 | 2512.1184 | 21 | -2.0 | 629.0356 | 4 | 11.75 | 4 | F4:2463 | 05112019\_RID\_1313\_NaNaRj\_F4.raw |  |  |  | 1.89E6 |  |  |  |  |  |  | 2 | 0 | 0 | 0 | 2 | 0 | 0 | 0 | 0 | 0 | 0 | 3 | 23 | Carbamidomethylation |
| R.C(+57.02)FITPDITSK.D | N | 84.64 | 1180.5798 | 10 | 0.8 | 591.2977 | 2 | 12.69 | 4 | F4:3370 | 05112019\_RID\_1313\_NaNaRj\_F4.raw |  |  |  | 5.53E6 | 3.95E5 |  |  | 2.06E5 | 7.86E4 | 2.43E4 | 14 | 0 | 0 | 0 | 10 | 1 | 0 | 0 | 1 | 1 | 1 | 3 | 12 | Carbamidomethylation |
| K.RVDLGC(+57.02)AATC(+57.02)PTVR.T | N | 61.64 | 1574.7657 | 14 | 0.8 | 525.9296 | 3 | 11.31 | 4 | F4:2180 | 05112019\_RID\_1313\_NaNaRj\_F4.raw |  |  |  | 6.83E4 |  |  |  |  |  |  | 1 | 0 | 0 | 0 | 1 | 0 | 0 | 0 | 0 | 0 | 0 | 36 | 49 | Carbamidomethylation |
| K.TWC(+57.02)DGFC(+57.02)SIR.G | Y | 61.57 | 1300.5328 | 10 | 0.0 | 651.2737 | 2 | 12.76 | 4 | F4:4538 | 05112019\_RID\_1313\_NaNaRj\_F4.raw |  |  |  | 3.1E8 |  |  |  |  |  |  | 7 | 0 | 0 | 0 | 7 | 0 | 0 | 0 | 0 | 0 | 0 | 24 | 33 | Carbamidomethylation |
| IRC(+57.02)FITPDITSK.D | N | 61.23 | 1449.7650 | 12 | -0.7 | 484.2619 | 3 | 11.86 | 4 | F4:2674 | 05112019\_RID\_1313\_NaNaRj\_F4.raw |  |  |  | 4.28E4 |  |  |  |  |  |  | 1 | 0 | 0 | 0 | 1 | 0 | 0 | 0 | 0 | 0 | 0 | 1 | 12 | Carbamidomethylation |
| K.DC(+57.02)PNGHVC(+57.02)Y.T | N | 47.04 | 1120.4066 | 9 | -0.3 | 561.2104 | 2 | 11.16 | 4 | F4:2024 | 05112019\_RID\_1313\_NaNaRj\_F4.raw |  |  |  | 1.84E6 |  |  |  |  |  |  | 1 | 0 | 0 | 0 | 1 | 0 | 0 | 0 | 0 | 0 | 0 | 13 | 21 | Carbamidomethylation |
| IRC(+57.02)FITPDITSKDC(+57.02)PNGHVC(+57.02)YTK.T | N | 46.82 | 2781.3037 | 23 | 1.4 | 557.2688 | 5 | 11.57 | 4 | F4:2385 | 05112019\_RID\_1313\_NaNaRj\_F4.raw |  |  |  | 0 |  |  |  |  |  |  | 1 | 0 | 0 | 0 | 1 | 0 | 0 | 0 | 0 | 0 | 0 | 1 | 23 | Carbamidomethylation |
| W.C(+57.02)DGFC(+57.02)SIR.G | Y | 41.77 | 1013.4059 | 8 | 0.0 | 507.7102 | 2 | 11.47 | 4 | F4:2310 | 05112019\_RID\_1313\_NaNaRj\_F4.raw |  |  |  | 7.71E4 |  |  |  |  |  |  | 1 | 0 | 0 | 0 | 1 | 0 | 0 | 0 | 0 | 0 | 0 | 26 | 33 | Carbamidomethylation |
| total 9 peptides |  |  |  |  |  |  |  |  |  |  |  |  |  |  |  |  |  |  |  |  |  |  |  |  |  |  |  |  |  |  |  |  |  |  |  |
| --- | --- | --- | --- | --- | --- | --- | --- | --- | --- | --- | --- | --- | --- | --- | --- | --- | --- | --- | --- | --- | --- | --- | --- | --- | --- | --- | --- | --- | --- | --- | --- | --- | --- | --- | --- |

P25669|3L22\_NAJNA

back to list

  

| Protein Coverage
| Supporting Peptides
|

Protein Coverage:

Supporting Peptides:

| Peptide | Uniq | -10lgP | Mass | Length | ppm | m/z | z | RT | Fraction | Scan | Source File | Area F1 | Area F2 | Area F3 | Area F4 | Area F5 | Area F6 | Area F7 | Area F8 | Area F9 | Area F10 | #Feature | #Feature F1 | #Feature F2 | #Feature F3 | #Feature F4 | #Feature F5 | #Feature F6 | #Feature F7 | #Feature F8 | #Feature F9 | #Feature F10 | Start | End | PTM |
| --- | --- | --- | --- | --- | --- | --- | --- | --- | --- | --- | --- | --- | --- | --- | --- | --- | --- | --- | --- | --- | --- | --- | --- | --- | --- | --- | --- | --- | --- | --- | --- | --- | --- | --- | --- |
| R.VDLGC(+57.02)AATC(+57.02)PTVR.T | N | 110.91 | 1418.6646 | 13 | 0.3 | 710.3398 | 2 | 11.61 | 4 | F4:2429 | 05112019\_RID\_1313\_NaNaRj\_F4.raw |  |  |  | 9.59E6 | 2.85E4 | 0 | 6.81E4 | 0 |  | 3.03E4 | 6 | 0 | 0 | 0 | 1 | 1 | 1 | 1 | 1 | 0 | 1 | 37 | 49 | Carbamidomethylation |
| R.C(+57.02)FITPDITSKDC(+57.02)PNGHVC(+57.02)YTK.T | N | 90.07 | 2512.1184 | 21 | -2.0 | 629.0356 | 4 | 11.75 | 4 | F4:2463 | 05112019\_RID\_1313\_NaNaRj\_F4.raw |  |  |  | 1.89E6 |  |  |  |  |  |  | 2 | 0 | 0 | 0 | 2 | 0 | 0 | 0 | 0 | 0 | 0 | 3 | 23 | Carbamidomethylation |
| R.C(+57.02)FITPDITSK.D | N | 84.64 | 1180.5798 | 10 | 0.8 | 591.2977 | 2 | 12.69 | 4 | F4:3370 | 05112019\_RID\_1313\_NaNaRj\_F4.raw |  |  |  | 5.53E6 | 3.95E5 |  |  | 2.06E5 | 7.86E4 | 2.43E4 | 14 | 0 | 0 | 0 | 10 | 1 | 0 | 0 | 1 | 1 | 1 | 3 | 12 | Carbamidomethylation |
| K.TWC(+57.02)DGFC(+57.02)SSR.G | Y | 68.70 | 1274.4808 | 10 | -0.5 | 638.2474 | 2 | 11.79 | 4 | F4:2601 | 05112019\_RID\_1313\_NaNaRj\_F4.raw |  |  |  | 2.54E6 |  |  |  |  |  |  | 1 | 0 | 0 | 0 | 1 | 0 | 0 | 0 | 0 | 0 | 0 | 24 | 33 | Carbamidomethylation |
| K.RVDLGC(+57.02)AATC(+57.02)PTVR.T | N | 61.64 | 1574.7657 | 14 | 0.8 | 525.9296 | 3 | 11.31 | 4 | F4:2180 | 05112019\_RID\_1313\_NaNaRj\_F4.raw |  |  |  | 6.83E4 |  |  |  |  |  |  | 1 | 0 | 0 | 0 | 1 | 0 | 0 | 0 | 0 | 0 | 0 | 36 | 49 | Carbamidomethylation |
| IRC(+57.02)FITPDITSK.D | N | 61.23 | 1449.7650 | 12 | -0.7 | 484.2619 | 3 | 11.86 | 4 | F4:2674 | 05112019\_RID\_1313\_NaNaRj\_F4.raw |  |  |  | 4.28E4 |  |  |  |  |  |  | 1 | 0 | 0 | 0 | 1 | 0 | 0 | 0 | 0 | 0 | 0 | 1 | 12 | Carbamidomethylation |
| K.DC(+57.02)PNGHVC(+57.02)Y.T | N | 47.04 | 1120.4066 | 9 | -0.3 | 561.2104 | 2 | 11.16 | 4 | F4:2024 | 05112019\_RID\_1313\_NaNaRj\_F4.raw |  |  |  | 1.84E6 |  |  |  |  |  |  | 1 | 0 | 0 | 0 | 1 | 0 | 0 | 0 | 0 | 0 | 0 | 13 | 21 | Carbamidomethylation |
| IRC(+57.02)FITPDITSKDC(+57.02)PNGHVC(+57.02)YTK.T | N | 46.82 | 2781.3037 | 23 | 1.4 | 557.2688 | 5 | 11.57 | 4 | F4:2385 | 05112019\_RID\_1313\_NaNaRj\_F4.raw |  |  |  | 0 |  |  |  |  |  |  | 1 | 0 | 0 | 0 | 1 | 0 | 0 | 0 | 0 | 0 | 0 | 1 | 23 | Carbamidomethylation |
| total 8 peptides |  |  |  |  |  |  |  |  |  |  |  |  |  |  |  |  |  |  |  |  |  |  |  |  |  |  |  |  |  |  |  |  |  |  |  |
| --- | --- | --- | --- | --- | --- | --- | --- | --- | --- | --- | --- | --- | --- | --- | --- | --- | --- | --- | --- | --- | --- | --- | --- | --- | --- | --- | --- | --- | --- | --- | --- | --- | --- | --- | --- |

P84808|CRVP2\_NAJKA

back to list

  

| Protein Coverage
| Supporting Peptides
|

Protein Coverage:

Supporting Peptides:

| Peptide | Uniq | -10lgP | Mass | Length | ppm | m/z | z | RT | Fraction | Scan | Source File | Area F1 | Area F2 | Area F3 | Area F4 | Area F5 | Area F6 | Area F7 | Area F8 | Area F9 | Area F10 | #Feature | #Feature F1 | #Feature F2 | #Feature F3 | #Feature F4 | #Feature F5 | #Feature F6 | #Feature F7 | #Feature F8 | #Feature F9 | #Feature F10 | Start | End | PTM |
| --- | --- | --- | --- | --- | --- | --- | --- | --- | --- | --- | --- | --- | --- | --- | --- | --- | --- | --- | --- | --- | --- | --- | --- | --- | --- | --- | --- | --- | --- | --- | --- | --- | --- | --- | --- |
| K.SGPPC(+57.02)GDC(+57.02)PSAC(+57.02)VNGLC(+57.02)TNPC(+57.02)K.H | N | 76.64 | 2406.9482 | 22 | 0.4 | 1204.4819 | 2 | 11.57 | 9 | F9:2245 | 05112019\_RID\_1313\_NaNaRj\_F9.raw |  |  |  |  |  |  |  |  | 1.74E6 |  | 2 | 0 | 0 | 0 | 0 | 0 | 0 | 0 | 0 | 2 | 0 | 180 | 201 | Carbamidomethylation |
| K.FVYGVGANPPGSVIGH.Y | Y | 71.48 | 1569.7939 | 16 | 0.4 | 785.9045 | 2 | 14.77 | 9 | F9:3530 | 05112019\_RID\_1313\_NaNaRj\_F9.raw |  |  |  |  |  |  |  |  | 1.38E6 |  | 2 | 0 | 0 | 0 | 0 | 0 | 0 | 0 | 0 | 2 | 0 | 119 | 134 |  |
| R.VIQSWYDENK.K | N | 71.33 | 1280.6036 | 10 | -0.2 | 641.3090 | 2 | 11.71 | 9 | F9:2380 | 05112019\_RID\_1313\_NaNaRj\_F9.raw |  |  |  |  |  |  |  |  | 8.45E4 |  | 1 | 0 | 0 | 0 | 0 | 0 | 0 | 0 | 0 | 1 | 0 | 108 | 117 |  |
| K.QNAC(+57.02)QTEWM(+15.99)K.S | Y | 66.91 | 1310.5383 | 10 | 0.6 | 656.2769 | 2 | 10.98 | 9 | F9:1742 | 05112019\_RID\_1313\_NaNaRj\_F9.raw |  |  |  |  |  |  |  |  | 2.42E4 |  | 1 | 0 | 0 | 0 | 0 | 0 | 0 | 0 | 0 | 1 | 0 | 215 | 224 | Carbamidomethylation; Oxidation (M) |
| K.QNAC(+57.02)QTEWMK.S | Y | 65.52 | 1294.5435 | 10 | 0.5 | 648.2793 | 2 | 11.41 | 9 | F9:2106 | 05112019\_RID\_1313\_NaNaRj\_F9.raw |  |  |  |  |  |  |  |  | 1.24E5 |  | 1 | 0 | 0 | 0 | 0 | 0 | 0 | 0 | 0 | 1 | 0 | 215 | 224 | Carbamidomethylation |
| R.C(+57.02)SFAHSPPHLR.T | Y | 64.66 | 1307.6193 | 11 | -0.5 | 436.8802 | 3 | 10.91 | 9 | F9:1703 | 05112019\_RID\_1313\_NaNaRj\_F9.raw |  |  |  |  |  |  |  |  | 1.2E5 |  | 1 | 0 | 0 | 0 | 0 | 0 | 0 | 0 | 0 | 1 | 0 | 75 | 85 | Carbamidomethylation |
| R.VIQSWYDENKK.F | Y | 63.71 | 1408.6986 | 11 | 0.5 | 705.3569 | 2 | 11.28 | 9 | F9:1995 | 05112019\_RID\_1313\_NaNaRj\_F9.raw |  |  |  |  |  |  |  |  | 1.12E6 |  | 2 | 0 | 0 | 0 | 0 | 0 | 0 | 0 | 0 | 2 | 0 | 108 | 118 |  |
| R.NMLQM(+15.99)EWNSNAAQNAK.R | Y | 62.99 | 1864.8196 | 16 | 0.6 | 933.4176 | 2 | 11.82 | 9 | F9:2487 | 05112019\_RID\_1313\_NaNaRj\_F9.raw |  |  |  |  |  |  |  |  | 9.32E4 |  | 1 | 0 | 0 | 0 | 0 | 0 | 0 | 0 | 0 | 1 | 0 | 54 | 69 | Oxidation (M) |
| K.IGC(+57.02)GENLFMSSQPYAWSR.V | Y | 61.62 | 2101.9351 | 18 | 0.7 | 1051.9756 | 2 | 24.90 | 9 | F9:5380 | 05112019\_RID\_1313\_NaNaRj\_F9.raw |  |  |  |  |  |  |  |  | 9.07E4 |  | 1 | 0 | 0 | 0 | 0 | 0 | 0 | 0 | 0 | 1 | 0 | 90 | 107 | Carbamidomethylation |
| K.C(+57.02)AASC(+57.02)FC(+57.02)R.T | Y | 58.92 | 1030.3783 | 8 | 0.2 | 516.1965 | 2 | 10.94 | 9 | F9:1723 | 05112019\_RID\_1313\_NaNaRj\_F9.raw |  |  |  |  |  |  |  |  | 1.9E5 |  | 1 | 0 | 0 | 0 | 0 | 0 | 0 | 0 | 0 | 1 | 0 | 227 | 234 | Carbamidomethylation |
| G.TVDFASESSNKR.E | N | 54.28 | 1339.6367 | 12 | 0.4 | 447.5530 | 3 | 10.84 | 9 | F9:1673 | 05112019\_RID\_1313\_NaNaRj\_F9.raw |  |  |  |  |  |  |  |  | 3.73E4 |  | 1 | 0 | 0 | 0 | 0 | 0 | 0 | 0 | 0 | 1 | 0 | 20 | 31 |  |
| R.NM(+15.99)LQM(+15.99)EWNSNAAQNAK.R | Y | 53.84 | 1880.8146 | 16 | 0.4 | 941.4150 | 2 | 11.45 | 9 | F9:2143 | 05112019\_RID\_1313\_NaNaRj\_F9.raw |  |  |  |  |  |  |  |  | 9.54E4 |  | 1 | 0 | 0 | 0 | 0 | 0 | 0 | 0 | 0 | 1 | 0 | 54 | 69 | Oxidation (M) |
| N.IIGSIATPYK.S | N | 53.07 | 1061.6121 | 10 | 0.8 | 531.8137 | 2 | 11.81 | 9 | F9:2486 | 05112019\_RID\_1313\_NaNaRj\_F9.raw |  |  |  |  |  |  |  |  | 6.92E5 |  | 1 | 0 | 0 | 0 | 0 | 0 | 0 | 0 | 0 | 1 | 0 | 170 | 179 |  |
| Q.YC(+57.02)PAGNIIGSIATPYK.S | N | 52.91 | 1723.8604 | 16 | 0.0 | 862.9374 | 2 | 19.53 | 9 | F9:4434 | 05112019\_RID\_1313\_NaNaRj\_F9.raw |  |  |  |  |  |  |  |  | 4.07E4 |  | 1 | 0 | 0 | 0 | 0 | 0 | 0 | 0 | 0 | 1 | 0 | 164 | 179 | Carbamidomethylation |
| Y.C(+57.02)PAGNIIGSIATPYK.S | N | 51.57 | 1560.7970 | 15 | 0.1 | 781.4059 | 2 | 15.72 | 9 | F9:3644 | 05112019\_RID\_1313\_NaNaRj\_F9.raw |  |  |  |  |  |  |  |  | 2.02E5 |  | 1 | 0 | 0 | 0 | 0 | 0 | 0 | 0 | 0 | 1 | 0 | 165 | 179 | Carbamidomethylation |
| K.FVYGVGANPPGSVIGHYT.Q | Y | 48.81 | 1833.9049 | 18 | 0.8 | 917.9604 | 2 | 17.87 | 9 | F9:4076 | 05112019\_RID\_1313\_NaNaRj\_F9.raw |  |  |  |  |  |  |  |  | 1.1E5 |  | 1 | 0 | 0 | 0 | 0 | 0 | 0 | 0 | 0 | 1 | 0 | 119 | 136 |  |
| K.FVYGVGANPPGSVIGHY.T | Y | 46.09 | 1732.8573 | 17 | 0.5 | 867.4364 | 2 | 18.13 | 9 | F9:4128 | 05112019\_RID\_1313\_NaNaRj\_F9.raw |  |  |  |  |  |  |  |  | 1.31E5 |  | 1 | 0 | 0 | 0 | 0 | 0 | 0 | 0 | 0 | 1 | 0 | 119 | 135 |  |
| K.SGPPC(+57.02)GDC(+57.02)PSAC(+57.02)VN.G | N | 46.03 | 1476.5432 | 14 | 0.9 | 739.2795 | 2 | 11.31 | 9 | F9:2013 | 05112019\_RID\_1313\_NaNaRj\_F9.raw |  |  |  |  |  |  |  |  | 1.16E5 |  | 1 | 0 | 0 | 0 | 0 | 0 | 0 | 0 | 0 | 1 | 0 | 180 | 193 | Carbamidomethylation |
| A.GNIIGSIATPYK.S | N | 45.44 | 1232.6765 | 12 | -0.1 | 617.3455 | 2 | 12.86 | 9 | F9:3004 | 05112019\_RID\_1313\_NaNaRj\_F9.raw |  |  |  |  |  |  |  |  | 8.57E4 |  | 1 | 0 | 0 | 0 | 0 | 0 | 0 | 0 | 0 | 1 | 0 | 168 | 179 |  |
| R.NMLQMEWNSNAAQNAK.R | Y | 44.21 | 1848.8247 | 16 | 0.7 | 925.4203 | 2 | 13.00 | 9 | F9:3033 | 05112019\_RID\_1313\_NaNaRj\_F9.raw |  |  |  |  |  |  |  |  | 3.71E4 |  | 1 | 0 | 0 | 0 | 0 | 0 | 0 | 0 | 0 | 1 | 0 | 54 | 69 |  |
| total 20 peptides |  |  |  |  |  |  |  |  |  |  |  |  |  |  |  |  |  |  |  |  |  |  |  |  |  |  |  |  |  |  |  |  |  |  |  |
| --- | --- | --- | --- | --- | --- | --- | --- | --- | --- | --- | --- | --- | --- | --- | --- | --- | --- | --- | --- | --- | --- | --- | --- | --- | --- | --- | --- | --- | --- | --- | --- | --- | --- | --- | --- |

P25672|3L24\_NAJNA

back to list

  

| Protein Coverage
| Supporting Peptides
|

Protein Coverage:

Supporting Peptides:

| Peptide | Uniq | -10lgP | Mass | Length | ppm | m/z | z | RT | Fraction | Scan | Source File | Area F1 | Area F2 | Area F3 | Area F4 | Area F5 | Area F6 | Area F7 | Area F8 | Area F9 | Area F10 | #Feature | #Feature F1 | #Feature F2 | #Feature F3 | #Feature F4 | #Feature F5 | #Feature F6 | #Feature F7 | #Feature F8 | #Feature F9 | #Feature F10 | Start | End | PTM |
| --- | --- | --- | --- | --- | --- | --- | --- | --- | --- | --- | --- | --- | --- | --- | --- | --- | --- | --- | --- | --- | --- | --- | --- | --- | --- | --- | --- | --- | --- | --- | --- | --- | --- | --- | --- |
| R.VDLGC(+57.02)AATC(+57.02)PTVK.T | N | 95.15 | 1390.6584 | 13 | 0.6 | 696.3369 | 2 | 11.58 | 5 | F5:2437 | 05112019\_RID\_1313\_NaNaRj\_F5.raw |  |  |  | 2.44E8 | 6.6E5 |  | 2.18E5 | 7.04E4 | 3.17E4 | 2.72E4 | 18 | 0 | 0 | 0 | 13 | 1 | 0 | 1 | 1 | 1 | 1 | 37 | 49 | Carbamidomethylation |
| R.C(+57.02)FITPDITSKDC(+57.02)PNGHVC(+57.02)YTK.T | N | 90.07 | 2512.1184 | 21 | -2.0 | 629.0356 | 4 | 11.75 | 4 | F4:2463 | 05112019\_RID\_1313\_NaNaRj\_F4.raw |  |  |  | 1.89E6 |  |  |  |  |  |  | 2 | 0 | 0 | 0 | 2 | 0 | 0 | 0 | 0 | 0 | 0 | 3 | 23 | Carbamidomethylation |
| R.C(+57.02)FITPDITSK.D | N | 84.64 | 1180.5798 | 10 | 0.8 | 591.2977 | 2 | 12.69 | 4 | F4:3370 | 05112019\_RID\_1313\_NaNaRj\_F4.raw |  |  |  | 5.53E6 | 3.95E5 |  |  | 2.06E5 | 7.86E4 | 2.43E4 | 14 | 0 | 0 | 0 | 10 | 1 | 0 | 0 | 1 | 1 | 1 | 3 | 12 | Carbamidomethylation |
| E.RVDLGC(+57.02)AATC(+57.02)PTVK.T | N | 82.64 | 1546.7595 | 14 | 0.0 | 774.3870 | 2 | 11.26 | 4 | F4:2100 | 05112019\_RID\_1313\_NaNaRj\_F4.raw |  |  |  | 8.74E6 |  |  |  |  |  |  | 2 | 0 | 0 | 0 | 2 | 0 | 0 | 0 | 0 | 0 | 0 | 36 | 49 | Carbamidomethylation |
| IRC(+57.02)FITPDITSK.D | N | 61.23 | 1449.7650 | 12 | -0.7 | 484.2619 | 3 | 11.86 | 4 | F4:2674 | 05112019\_RID\_1313\_NaNaRj\_F4.raw |  |  |  | 4.28E4 |  |  |  |  |  |  | 1 | 0 | 0 | 0 | 1 | 0 | 0 | 0 | 0 | 0 | 0 | 1 | 12 | Carbamidomethylation |
| K.DC(+57.02)PNGHVC(+57.02)Y.T | N | 47.04 | 1120.4066 | 9 | -0.3 | 561.2104 | 2 | 11.16 | 4 | F4:2024 | 05112019\_RID\_1313\_NaNaRj\_F4.raw |  |  |  | 1.84E6 |  |  |  |  |  |  | 1 | 0 | 0 | 0 | 1 | 0 | 0 | 0 | 0 | 0 | 0 | 13 | 21 | Carbamidomethylation |
| IRC(+57.02)FITPDITSKDC(+57.02)PNGHVC(+57.02)YTK.T | N | 46.82 | 2781.3037 | 23 | 1.4 | 557.2688 | 5 | 11.57 | 4 | F4:2385 | 05112019\_RID\_1313\_NaNaRj\_F4.raw |  |  |  | 0 |  |  |  |  |  |  | 1 | 0 | 0 | 0 | 1 | 0 | 0 | 0 | 0 | 0 | 0 | 1 | 23 | Carbamidomethylation |
| K.TWC(+57.02)DGFC(+57.02)R.I | Y | 46.58 | 1100.4167 | 8 | 0.1 | 551.2157 | 2 | 11.82 | 5 | F5:2687 | 05112019\_RID\_1313\_NaNaRj\_F5.raw |  |  |  | 6.81E4 | 5.73E4 |  |  |  |  |  | 4 | 0 | 0 | 0 | 3 | 1 | 0 | 0 | 0 | 0 | 0 | 24 | 31 | Carbamidomethylation |
| D.LGC(+57.02)AATC(+57.02)PTVK.T | N | 45.08 | 1176.5631 | 11 | 0.1 | 589.2889 | 2 | 11.03 | 4 | F4:1924 | 05112019\_RID\_1313\_NaNaRj\_F4.raw |  |  |  | 2.8E4 |  |  |  |  |  |  | 1 | 0 | 0 | 0 | 1 | 0 | 0 | 0 | 0 | 0 | 0 | 39 | 49 | Carbamidomethylation |
| total 9 peptides |  |  |  |  |  |  |  |  |  |  |  |  |  |  |  |  |  |  |  |  |  |  |  |  |  |  |  |  |  |  |  |  |  |  |  |
| --- | --- | --- | --- | --- | --- | --- | --- | --- | --- | --- | --- | --- | --- | --- | --- | --- | --- | --- | --- | --- | --- | --- | --- | --- | --- | --- | --- | --- | --- | --- | --- | --- | --- | --- | --- |

P02768|ALBU\_HUMAN

back to list

  

| Protein Coverage
| Supporting Peptides
|

Protein Coverage:

Supporting Peptides:

| Peptide | Uniq | -10lgP | Mass | Length | ppm | m/z | z | RT | Fraction | Scan | Source File | Area F1 | Area F2 | Area F3 | Area F4 | Area F5 | Area F6 | Area F7 | Area F8 | Area F9 | Area F10 | #Feature | #Feature F1 | #Feature F2 | #Feature F3 | #Feature F4 | #Feature F5 | #Feature F6 | #Feature F7 | #Feature F8 | #Feature F9 | #Feature F10 | Start | End | PTM |
| --- | --- | --- | --- | --- | --- | --- | --- | --- | --- | --- | --- | --- | --- | --- | --- | --- | --- | --- | --- | --- | --- | --- | --- | --- | --- | --- | --- | --- | --- | --- | --- | --- | --- | --- | --- |
| K.DVFLGMFLYEYAR.R | Y | 73.20 | 1622.7803 | 13 | 1.1 | 812.3983 | 2 | 98.33 | 2 | F2:35078 | 09012020\_NaNaRj\_F2.raw |  | 3.78E4 |  |  |  |  |  |  |  |  | 1 | 0 | 1 | 0 | 0 | 0 | 0 | 0 | 0 | 0 | 0 | 348 | 360 |  |
| K.AVM(+15.99)DDFAAFVEK.C | Y | 72.27 | 1357.6223 | 12 | 0.2 | 679.8185 | 2 | 58.79 | 2 | F2:19518 | 09012020\_NaNaRj\_F2.raw |  | 2.02E5 |  |  |  |  |  |  |  |  | 2 | 0 | 2 | 0 | 0 | 0 | 0 | 0 | 0 | 0 | 0 | 570 | 581 | Oxidation (M) |
| K.LVNEVTEFAK.T | Y | 65.11 | 1148.6077 | 10 | 0.8 | 575.3116 | 2 | 11.91 | 3 | F3:2629 | 05112019\_RID\_1313\_NaNaRj\_F3.raw |  | 2.17E6 | 5.87E4 |  |  |  |  |  |  |  | 3 | 0 | 2 | 1 | 0 | 0 | 0 | 0 | 0 | 0 | 0 | 66 | 75 |  |
| K.YIC(+57.02)ENQDSISSK.L | Y | 61.24 | 1442.6348 | 12 | 0.2 | 722.3248 | 2 | 11.07 | 3 | F3:1912 | 05112019\_RID\_1313\_NaNaRj\_F3.raw |  |  | 1.07E4 |  |  |  |  |  |  |  | 1 | 0 | 0 | 1 | 0 | 0 | 0 | 0 | 0 | 0 | 0 | 287 | 298 | Carbamidomethylation |
| K.VPQVSTPTLVEVSR.N | N | 60.76 | 1510.8354 | 14 | 0.2 | 756.4252 | 2 | 12.69 | 3 | F3:2919 | 05112019\_RID\_1313\_NaNaRj\_F3.raw | 3.42E4 |  | 1.53E4 |  |  |  |  |  |  |  | 2 | 1 | 0 | 1 | 0 | 0 | 0 | 0 | 0 | 0 | 0 | 439 | 452 |  |
| K.LVAASQAALGL | Y | 59.06 | 1012.5917 | 11 | 0.3 | 507.3033 | 2 | 63.23 | 2 | F2:20845 | 09012020\_NaNaRj\_F2.raw |  | 1.97E6 | 9.22E3 |  |  |  |  |  |  |  | 2 | 0 | 1 | 1 | 0 | 0 | 0 | 0 | 0 | 0 | 0 | 599 | 609 |  |
| K.QTALVELVK.H | Y | 57.05 | 999.5964 | 9 | -0.3 | 500.8054 | 2 | 45.91 | 2 | F2:14906 | 09012020\_NaNaRj\_F2.raw |  | 3.2E5 | 2.2E4 |  |  |  |  |  |  |  | 2 | 0 | 1 | 1 | 0 | 0 | 0 | 0 | 0 | 0 | 0 | 550 | 558 |  |
| K.SLHTLFGDK.L | N | 56.52 | 1016.5291 | 9 | -0.4 | 509.2716 | 2 | 31.29 | 2 | F2:8765 | 09012020\_NaNaRj\_F2.raw |  | 1.05E5 |  |  |  |  |  |  |  |  | 1 | 0 | 1 | 0 | 0 | 0 | 0 | 0 | 0 | 0 | 0 | 89 | 97 |  |
| K.TC(+57.02)VADESAENC(+57.02)DK.S | N | 55.39 | 1497.5712 | 13 | 0.7 | 749.7934 | 2 | 12.46 | 2 | F2:2050 | 09012020\_NaNaRj\_F2.raw |  | 1.43E5 |  |  |  |  |  |  |  |  | 1 | 0 | 1 | 0 | 0 | 0 | 0 | 0 | 0 | 0 | 0 | 76 | 88 | Carbamidomethylation |
| K.DDNPNLPR.L | Y | 55.02 | 939.4410 | 8 | 0.0 | 470.7278 | 2 | 21.14 | 2 | F2:4882 | 09012020\_NaNaRj\_F2.raw |  | 8.15E5 |  |  |  |  |  |  |  |  | 2 | 0 | 2 | 0 | 0 | 0 | 0 | 0 | 0 | 0 | 0 | 131 | 138 |  |
| R.FKDLGEENFK.A | Y | 49.30 | 1225.5979 | 10 | 0.3 | 613.8064 | 2 | 28.72 | 2 | F2:7734 | 09012020\_NaNaRj\_F2.raw |  | 1.71E5 |  |  |  |  |  |  |  |  | 1 | 0 | 1 | 0 | 0 | 0 | 0 | 0 | 0 | 0 | 0 | 35 | 44 |  |
| K.KVPQVSTPTLVEVSR.N | N | 48.71 | 1638.9304 | 15 | 0.3 | 547.3176 | 3 | 41.58 | 2 | F2:12974 | 09012020\_NaNaRj\_F2.raw |  | 2.51E5 |  |  |  |  |  |  |  |  | 1 | 0 | 1 | 0 | 0 | 0 | 0 | 0 | 0 | 0 | 0 | 438 | 452 |  |
| K.AAFTEC(+57.02)C(+57.02)QAADK.A | Y | 48.43 | 1370.5594 | 12 | 0.2 | 686.2871 | 2 | 19.85 | 2 | F2:4129 | 09012020\_NaNaRj\_F2.raw |  | 1.07E6 |  |  |  |  |  |  |  |  | 1 | 0 | 1 | 0 | 0 | 0 | 0 | 0 | 0 | 0 | 0 | 187 | 198 | Carbamidomethylation |
| K.TYETTLEK.C | Y | 47.59 | 983.4811 | 8 | 0.0 | 492.7479 | 2 | 19.91 | 2 | F2:4221 | 09012020\_NaNaRj\_F2.raw |  | 8.88E5 |  |  |  |  |  |  |  |  | 1 | 0 | 1 | 0 | 0 | 0 | 0 | 0 | 0 | 0 | 0 | 376 | 383 |  |
| K.AEFAEVSK.L | Y | 46.29 | 879.4338 | 8 | 0.7 | 440.7245 | 2 | 20.66 | 2 | F2:4474 | 09012020\_NaNaRj\_F2.raw |  | 4.5E5 |  |  |  |  |  |  |  |  | 1 | 0 | 1 | 0 | 0 | 0 | 0 | 0 | 0 | 0 | 0 | 250 | 257 |  |
| K.VFDEFKPLVEEPQ.N | Y | 45.04 | 1575.7820 | 13 | -0.2 | 788.8981 | 2 | 65.29 | 2 | F2:21442 | 09012020\_NaNaRj\_F2.raw |  | 4.68E4 |  |  |  |  |  |  |  |  | 1 | 0 | 1 | 0 | 0 | 0 | 0 | 0 | 0 | 0 | 0 | 397 | 409 |  |
| K.C(+57.02)C(+57.02)TESLVNR.R | N | 42.52 | 1137.4907 | 9 | -0.1 | 569.7526 | 2 | 19.84 | 2 | F2:4143 | 09012020\_NaNaRj\_F2.raw |  | 2.81E4 |  |  |  |  |  |  |  |  | 1 | 0 | 1 | 0 | 0 | 0 | 0 | 0 | 0 | 0 | 0 | 500 | 508 | Carbamidomethylation |
| total 17 peptides |  |  |  |  |  |  |  |  |  |  |  |  |  |  |  |  |  |  |  |  |  |  |  |  |  |  |  |  |  |  |  |  |  |  |  |
| --- | --- | --- | --- | --- | --- | --- | --- | --- | --- | --- | --- | --- | --- | --- | --- | --- | --- | --- | --- | --- | --- | --- | --- | --- | --- | --- | --- | --- | --- | --- | --- | --- | --- | --- | --- |

#CONTAM#ALBU\_HUMAN|

back to list

  

| Protein Coverage
| Supporting Peptides
|

Protein Coverage:

Supporting Peptides:

| Peptide | Uniq | -10lgP | Mass | Length | ppm | m/z | z | RT | Fraction | Scan | Source File | Area F1 | Area F2 | Area F3 | Area F4 | Area F5 | Area F6 | Area F7 | Area F8 | Area F9 | Area F10 | #Feature | #Feature F1 | #Feature F2 | #Feature F3 | #Feature F4 | #Feature F5 | #Feature F6 | #Feature F7 | #Feature F8 | #Feature F9 | #Feature F10 | Start | End | PTM |
| --- | --- | --- | --- | --- | --- | --- | --- | --- | --- | --- | --- | --- | --- | --- | --- | --- | --- | --- | --- | --- | --- | --- | --- | --- | --- | --- | --- | --- | --- | --- | --- | --- | --- | --- | --- |
| K.DVFLGMFLYEYAR.R | Y | 73.20 | 1622.7803 | 13 | 1.1 | 812.3983 | 2 | 98.33 | 2 | F2:35078 | 09012020\_NaNaRj\_F2.raw |  | 3.78E4 |  |  |  |  |  |  |  |  | 1 | 0 | 1 | 0 | 0 | 0 | 0 | 0 | 0 | 0 | 0 | 348 | 360 |  |
| K.AVM(+15.99)DDFAAFVEK.C | Y | 72.27 | 1357.6223 | 12 | 0.2 | 679.8185 | 2 | 58.79 | 2 | F2:19518 | 09012020\_NaNaRj\_F2.raw |  | 2.02E5 |  |  |  |  |  |  |  |  | 2 | 0 | 2 | 0 | 0 | 0 | 0 | 0 | 0 | 0 | 0 | 570 | 581 | Oxidation (M) |
| K.LVNEVTEFAK.T | Y | 65.11 | 1148.6077 | 10 | 0.8 | 575.3116 | 2 | 11.91 | 3 | F3:2629 | 05112019\_RID\_1313\_NaNaRj\_F3.raw |  | 2.17E6 | 5.87E4 |  |  |  |  |  |  |  | 3 | 0 | 2 | 1 | 0 | 0 | 0 | 0 | 0 | 0 | 0 | 66 | 75 |  |
| K.YIC(+57.02)ENQDSISSK.L | Y | 61.24 | 1442.6348 | 12 | 0.2 | 722.3248 | 2 | 11.07 | 3 | F3:1912 | 05112019\_RID\_1313\_NaNaRj\_F3.raw |  |  | 1.07E4 |  |  |  |  |  |  |  | 1 | 0 | 0 | 1 | 0 | 0 | 0 | 0 | 0 | 0 | 0 | 287 | 298 | Carbamidomethylation |
| K.VPQVSTPTLVEVSR.N | N | 60.76 | 1510.8354 | 14 | 0.2 | 756.4252 | 2 | 12.69 | 3 | F3:2919 | 05112019\_RID\_1313\_NaNaRj\_F3.raw | 3.42E4 |  | 1.53E4 |  |  |  |  |  |  |  | 2 | 1 | 0 | 1 | 0 | 0 | 0 | 0 | 0 | 0 | 0 | 439 | 452 |  |
| K.LVAASQAALGL | Y | 59.06 | 1012.5917 | 11 | 0.3 | 507.3033 | 2 | 63.23 | 2 | F2:20845 | 09012020\_NaNaRj\_F2.raw |  | 1.97E6 | 9.22E3 |  |  |  |  |  |  |  | 2 | 0 | 1 | 1 | 0 | 0 | 0 | 0 | 0 | 0 | 0 | 599 | 609 |  |
| K.QTALVELVK.H | Y | 57.05 | 999.5964 | 9 | -0.3 | 500.8054 | 2 | 45.91 | 2 | F2:14906 | 09012020\_NaNaRj\_F2.raw |  | 3.2E5 | 2.2E4 |  |  |  |  |  |  |  | 2 | 0 | 1 | 1 | 0 | 0 | 0 | 0 | 0 | 0 | 0 | 550 | 558 |  |
| K.SLHTLFGDK.L | N | 56.52 | 1016.5291 | 9 | -0.4 | 509.2716 | 2 | 31.29 | 2 | F2:8765 | 09012020\_NaNaRj\_F2.raw |  | 1.05E5 |  |  |  |  |  |  |  |  | 1 | 0 | 1 | 0 | 0 | 0 | 0 | 0 | 0 | 0 | 0 | 89 | 97 |  |
| K.TC(+57.02)VADESAENC(+57.02)DK.S | N | 55.39 | 1497.5712 | 13 | 0.7 | 749.7934 | 2 | 12.46 | 2 | F2:2050 | 09012020\_NaNaRj\_F2.raw |  | 1.43E5 |  |  |  |  |  |  |  |  | 1 | 0 | 1 | 0 | 0 | 0 | 0 | 0 | 0 | 0 | 0 | 76 | 88 | Carbamidomethylation |
| K.DDNPNLPR.L | Y | 55.02 | 939.4410 | 8 | 0.0 | 470.7278 | 2 | 21.14 | 2 | F2:4882 | 09012020\_NaNaRj\_F2.raw |  | 8.15E5 |  |  |  |  |  |  |  |  | 2 | 0 | 2 | 0 | 0 | 0 | 0 | 0 | 0 | 0 | 0 | 131 | 138 |  |
| R.FKDLGEENFK.A | Y | 49.30 | 1225.5979 | 10 | 0.3 | 613.8064 | 2 | 28.72 | 2 | F2:7734 | 09012020\_NaNaRj\_F2.raw |  | 1.71E5 |  |  |  |  |  |  |  |  | 1 | 0 | 1 | 0 | 0 | 0 | 0 | 0 | 0 | 0 | 0 | 35 | 44 |  |
| K.KVPQVSTPTLVEVSR.N | N | 48.71 | 1638.9304 | 15 | 0.3 | 547.3176 | 3 | 41.58 | 2 | F2:12974 | 09012020\_NaNaRj\_F2.raw |  | 2.51E5 |  |  |  |  |  |  |  |  | 1 | 0 | 1 | 0 | 0 | 0 | 0 | 0 | 0 | 0 | 0 | 438 | 452 |  |
| K.AAFTEC(+57.02)C(+57.02)QAADK.A | Y | 48.43 | 1370.5594 | 12 | 0.2 | 686.2871 | 2 | 19.85 | 2 | F2:4129 | 09012020\_NaNaRj\_F2.raw |  | 1.07E6 |  |  |  |  |  |  |  |  | 1 | 0 | 1 | 0 | 0 | 0 | 0 | 0 | 0 | 0 | 0 | 187 | 198 | Carbamidomethylation |
| K.TYETTLEK.C | Y | 47.59 | 983.4811 | 8 | 0.0 | 492.7479 | 2 | 19.91 | 2 | F2:4221 | 09012020\_NaNaRj\_F2.raw |  | 8.88E5 |  |  |  |  |  |  |  |  | 1 | 0 | 1 | 0 | 0 | 0 | 0 | 0 | 0 | 0 | 0 | 376 | 383 |  |
| K.AEFAEVSK.L | Y | 46.29 | 879.4338 | 8 | 0.7 | 440.7245 | 2 | 20.66 | 2 | F2:4474 | 09012020\_NaNaRj\_F2.raw |  | 4.5E5 |  |  |  |  |  |  |  |  | 1 | 0 | 1 | 0 | 0 | 0 | 0 | 0 | 0 | 0 | 0 | 250 | 257 |  |
| K.VFDEFKPLVEEPQ.N | Y | 45.04 | 1575.7820 | 13 | -0.2 | 788.8981 | 2 | 65.29 | 2 | F2:21442 | 09012020\_NaNaRj\_F2.raw |  | 4.68E4 |  |  |  |  |  |  |  |  | 1 | 0 | 1 | 0 | 0 | 0 | 0 | 0 | 0 | 0 | 0 | 397 | 409 |  |
| K.C(+57.02)C(+57.02)TESLVNR.R | N | 42.52 | 1137.4907 | 9 | -0.1 | 569.7526 | 2 | 19.84 | 2 | F2:4143 | 09012020\_NaNaRj\_F2.raw |  | 2.81E4 |  |  |  |  |  |  |  |  | 1 | 0 | 1 | 0 | 0 | 0 | 0 | 0 | 0 | 0 | 0 | 500 | 508 | Carbamidomethylation |
| total 17 peptides |  |  |  |  |  |  |  |  |  |  |  |  |  |  |  |  |  |  |  |  |  |  |  |  |  |  |  |  |  |  |  |  |  |  |  |
| --- | --- | --- | --- | --- | --- | --- | --- | --- | --- | --- | --- | --- | --- | --- | --- | --- | --- | --- | --- | --- | --- | --- | --- | --- | --- | --- | --- | --- | --- | --- | --- | --- | --- | --- | --- |

P86538|3SA2A\_NAJNA

back to list

  

| Protein Coverage
| Supporting Peptides
|

Protein Coverage:

Supporting Peptides:

| Peptide | Uniq | -10lgP | Mass | Length | ppm | m/z | z | RT | Fraction | Scan | Source File | Area F1 | Area F2 | Area F3 | Area F4 | Area F5 | Area F6 | Area F7 | Area F8 | Area F9 | Area F10 | #Feature | #Feature F1 | #Feature F2 | #Feature F3 | #Feature F4 | #Feature F5 | #Feature F6 | #Feature F7 | #Feature F8 | #Feature F9 | #Feature F10 | Start | End | PTM |
| --- | --- | --- | --- | --- | --- | --- | --- | --- | --- | --- | --- | --- | --- | --- | --- | --- | --- | --- | --- | --- | --- | --- | --- | --- | --- | --- | --- | --- | --- | --- | --- | --- | --- | --- | --- |
| K.NSLLVKYEC(+57.02)C(+57.02)NTDRC(+57.02)N | N | 97.16 | 2044.8765 | 16 | -1.0 | 682.6321 | 3 | 11.41 | 6 | F6:2203 | 05112019\_RID\_1313\_NaNaRj\_F6.raw |  |  |  |  |  | 2.67E8 | 4.74E5 | 0 |  |  | 13 | 0 | 0 | 0 | 0 | 0 | 9 | 2 | 2 | 0 | 0 | 45 | 60 | Carbamidomethylation |
| R.GC(+57.02)IDVC(+57.02)PKNSLLVK.Y | N | 95.44 | 1601.8269 | 14 | 2.0 | 801.9224 | 2 | 11.71 | 6 | F6:2473 | 05112019\_RID\_1313\_NaNaRj\_F6.raw |  |  |  |  |  | 6.21E6 |  |  |  |  | 2 | 0 | 0 | 0 | 0 | 0 | 2 | 0 | 0 | 0 | 0 | 37 | 50 | Carbamidomethylation |
| R.GC(+57.02)IDVC(+57.02)PKNSLLVKYEC(+57.02)C(+57.02)NTDRC(+57.02)N | N | 92.04 | 2974.2864 | 24 | 0.9 | 744.5795 | 4 | 11.76 | 6 | F6:2537 | 05112019\_RID\_1313\_NaNaRj\_F6.raw |  |  |  |  |  | 3.72E6 |  |  |  |  | 1 | 0 | 0 | 0 | 0 | 0 | 1 | 0 | 0 | 0 | 0 | 37 | 60 | Carbamidomethylation |
| K.NSLLVKYEC(+57.02)C(+57.02)NTDR.C | N | 77.99 | 1770.8029 | 14 | -0.4 | 591.2747 | 3 | 11.44 | 6 | F6:2243 | 05112019\_RID\_1313\_NaNaRj\_F6.raw |  |  |  |  |  | 1.95E7 |  |  |  |  | 1 | 0 | 0 | 0 | 0 | 0 | 1 | 0 | 0 | 0 | 0 | 45 | 58 | Carbamidomethylation |
| K.MFM(+15.99)VSDLTIPVK.R | Y | 57.22 | 1395.7142 | 12 | 0.9 | 698.8650 | 2 | 26.87 | 6 | F6:11906 | 05112019\_RID\_1313\_NaNaRj\_F6.raw |  |  |  |  |  | 3.12E4 |  |  |  |  | 2 | 0 | 0 | 0 | 0 | 0 | 2 | 0 | 0 | 0 | 0 | 24 | 35 | Oxidation (M) |
| R.GC(+57.02)IDVC(+57.02)PK.N | N | 56.82 | 947.4205 | 8 | -2.7 | 474.7162 | 2 | 11.15 | 8 | F8:2003 | 05112019\_RID\_1313\_NaNaRj\_F8.raw | 1E5 | 1.9E5 | 1.09E4 | 8.89E4 | 5.11E5 | 3.82E8 |  | 3.84E7 | 5.76E5 | 1.59E4 | 23 | 1 | 1 | 1 | 1 | 1 | 14 | 0 | 2 | 1 | 1 | 37 | 44 | Carbamidomethylation |
| K.NSLLVKYEC(+57.02).C | N | 48.59 | 1124.5536 | 9 | -0.5 | 563.2838 | 2 | 11.86 | 6 | F6:2634 | 05112019\_RID\_1313\_NaNaRj\_F6.raw |  |  |  |  |  | 5.36E5 |  |  |  |  | 1 | 0 | 0 | 0 | 0 | 0 | 1 | 0 | 0 | 0 | 0 | 45 | 53 | Carbamidomethylation |
| K.M(+15.99)FM(+15.99)VSDLTIPVK.R | Y | 46.77 | 1411.7091 | 12 | 0.2 | 706.8619 | 2 | 20.84 | 6 | F6:8772 | 05112019\_RID\_1313\_NaNaRj\_F6.raw |  |  |  |  |  | 2.99E5 |  |  |  |  | 1 | 0 | 0 | 0 | 0 | 0 | 1 | 0 | 0 | 0 | 0 | 24 | 35 | Oxidation (M) |
| K.NSLLVKYE.C | N | 42.14 | 964.5229 | 8 | -0.5 | 483.2685 | 2 | 11.81 | 6 | F6:2616 | 05112019\_RID\_1313\_NaNaRj\_F6.raw |  |  |  |  |  | 1.98E5 |  |  |  |  | 1 | 0 | 0 | 0 | 0 | 0 | 1 | 0 | 0 | 0 | 0 | 45 | 52 |  |
| total 9 peptides |  |  |  |  |  |  |  |  |  |  |  |  |  |  |  |  |  |  |  |  |  |  |  |  |  |  |  |  |  |  |  |  |  |  |  |
| --- | --- | --- | --- | --- | --- | --- | --- | --- | --- | --- | --- | --- | --- | --- | --- | --- | --- | --- | --- | --- | --- | --- | --- | --- | --- | --- | --- | --- | --- | --- | --- | --- | --- | --- | --- |

P82942|VM3K\_NAJKA

back to list

  

| Protein Coverage
| Supporting Peptides
|

Protein Coverage:

Supporting Peptides:

| Peptide | Uniq | -10lgP | Mass | Length | ppm | m/z | z | RT | Fraction | Scan | Source File | Area F1 | Area F2 | Area F3 | Area F4 | Area F5 | Area F6 | Area F7 | Area F8 | Area F9 | Area F10 | #Feature | #Feature F1 | #Feature F2 | #Feature F3 | #Feature F4 | #Feature F5 | #Feature F6 | #Feature F7 | #Feature F8 | #Feature F9 | #Feature F10 | Start | End | PTM |
| --- | --- | --- | --- | --- | --- | --- | --- | --- | --- | --- | --- | --- | --- | --- | --- | --- | --- | --- | --- | --- | --- | --- | --- | --- | --- | --- | --- | --- | --- | --- | --- | --- | --- | --- | --- |
| K.HDC(+57.02)DLPELC(+57.02)TGQSAEC(+57.02)PTDSLQR.N | Y | 123.82 | 2688.1213 | 23 | 0.0 | 897.0477 | 3 | 11.87 | 8 | F8:2673 | 05112019\_RID\_1313\_NaNaRj\_F8.raw |  |  |  |  |  |  |  | 1.02E6 | 7.11E4 |  | 3 | 0 | 0 | 0 | 0 | 0 | 0 | 0 | 2 | 1 | 0 | 262 | 284 | Carbamidomethylation |
| R.NGHPC(+57.02)QNNQGYC(+57.02)YNGK.C | N | 69.82 | 1909.7584 | 16 | -0.1 | 637.5934 | 3 | 10.66 | 9 | F9:1607 | 05112019\_RID\_1313\_NaNaRj\_F9.raw |  |  |  |  |  |  |  |  | 6.59E4 |  | 1 | 0 | 0 | 0 | 0 | 0 | 0 | 0 | 0 | 1 | 0 | 285 | 300 | Carbamidomethylation |
| R.DYQEYLLR.D | Y | 61.75 | 1098.5345 | 8 | -0.2 | 550.2744 | 2 | 14.66 | 9 | F9:3406 | 05112019\_RID\_1313\_NaNaRj\_F9.raw |  |  |  |  |  | 5.84E4 |  | 1.42E5 | 9.46E4 |  | 3 | 0 | 0 | 0 | 0 | 0 | 1 | 0 | 1 | 1 | 0 | 191 | 198 |  |
| R.TAPAFQFSSC(+57.02)SIR.D | N | 60.29 | 1470.6925 | 13 | 1.0 | 736.3542 | 2 | 12.55 | 9 | F9:2932 | 05112019\_RID\_1313\_NaNaRj\_F9.raw |  |  |  |  |  |  | 1.39E5 |  | 1.85E4 |  | 2 | 0 | 0 | 0 | 0 | 0 | 0 | 1 | 0 | 1 | 0 | 178 | 190 | Carbamidomethylation |
| R.VYEM(+15.99)INAVNTK.F | N | 59.01 | 1296.6384 | 11 | 0.3 | 649.3267 | 2 | 11.32 | 9 | F9:2053 | 05112019\_RID\_1313\_NaNaRj\_F9.raw |  |  |  |  |  |  |  |  | 4.99E4 |  | 1 | 0 | 0 | 0 | 0 | 0 | 0 | 0 | 0 | 1 | 0 | 41 | 51 | Oxidation (M) |
| R.DRPQC(+57.02)ILNKPLSTDIVSPAIC(+57.02)GN.Y | Y | 55.86 | 2567.2834 | 23 | -0.3 | 856.7682 | 3 | 17.88 | 8 | F8:5918 | 05112019\_RID\_1313\_NaNaRj\_F8.raw |  |  |  |  |  |  |  | 1.89E4 |  |  | 1 | 0 | 0 | 0 | 0 | 0 | 0 | 0 | 1 | 0 | 0 | 199 | 221 | Carbamidomethylation |
| K.C(+57.02)PTLTNQC(+57.02)IALLGPHFTVSPK.G | Y | 45.45 | 2353.1921 | 21 | 0.6 | 785.4051 | 3 | 19.32 | 9 | F9:4392 | 05112019\_RID\_1313\_NaNaRj\_F9.raw |  |  |  |  |  |  |  |  | 5.68E4 |  | 1 | 0 | 0 | 0 | 0 | 0 | 0 | 0 | 0 | 1 | 0 | 301 | 321 | Carbamidomethylation |
| R.QTVLLPR.K | Y | 40.73 | 825.5072 | 7 | 0.9 | 413.7612 | 2 | 11.65 | 9 | F9:2348 | 05112019\_RID\_1313\_NaNaRj\_F9.raw |  |  |  |  |  |  |  |  | 2.52E4 |  | 1 | 0 | 0 | 0 | 0 | 0 | 0 | 0 | 0 | 1 | 0 | 91 | 97 |  |
| total 8 peptides |  |  |  |  |  |  |  |  |  |  |  |  |  |  |  |  |  |  |  |  |  |  |  |  |  |  |  |  |  |  |  |  |  |  |  |
| --- | --- | --- | --- | --- | --- | --- | --- | --- | --- | --- | --- | --- | --- | --- | --- | --- | --- | --- | --- | --- | --- | --- | --- | --- | --- | --- | --- | --- | --- | --- | --- | --- | --- | --- | --- |

Q04695|K1C17\_HUMAN

back to list

  

| Protein Coverage
| Supporting Peptides
|

Protein Coverage:

Supporting Peptides:

| Peptide | Uniq | -10lgP | Mass | Length | ppm | m/z | z | RT | Fraction | Scan | Source File | Area F1 | Area F2 | Area F3 | Area F4 | Area F5 | Area F6 | Area F7 | Area F8 | Area F9 | Area F10 | #Feature | #Feature F1 | #Feature F2 | #Feature F3 | #Feature F4 | #Feature F5 | #Feature F6 | #Feature F7 | #Feature F8 | #Feature F9 | #Feature F10 | Start | End | PTM |
| --- | --- | --- | --- | --- | --- | --- | --- | --- | --- | --- | --- | --- | --- | --- | --- | --- | --- | --- | --- | --- | --- | --- | --- | --- | --- | --- | --- | --- | --- | --- | --- | --- | --- | --- | --- |
| R.EVATNSELVQSGK.S | N | 80.27 | 1360.6833 | 13 | 0.4 | 681.3492 | 2 | 11.13 | 3 | F3:1953 | 05112019\_RID\_1313\_NaNaRj\_F3.raw |  | 5.22E5 | 6.8E4 |  | 8.04E4 |  |  |  |  |  | 3 | 0 | 1 | 1 | 0 | 1 | 0 | 0 | 0 | 0 | 0 | 285 | 297 |  |
| K.ASLEGNLAETENR.Y | Y | 78.72 | 1402.6688 | 13 | 0.2 | 702.3418 | 2 | 11.48 | 5 | F5:2335 | 05112019\_RID\_1313\_NaNaRj\_F5.raw |  |  |  |  | 6.06E4 |  |  |  |  |  | 1 | 0 | 0 | 0 | 0 | 1 | 0 | 0 | 0 | 0 | 0 | 322 | 334 |  |
| R.ALEEANTELEVK.I | N | 71.18 | 1344.6772 | 12 | 0.2 | 673.3460 | 2 | 11.61 | 3 | F3:2400 | 05112019\_RID\_1313\_NaNaRj\_F3.raw |  |  | 3.95E4 |  |  |  |  |  |  |  | 1 | 0 | 0 | 1 | 0 | 0 | 0 | 0 | 0 | 0 | 0 | 104 | 115 |  |
| R.LEQEIATYR.R | N | 59.43 | 1121.5717 | 9 | -0.1 | 561.7930 | 2 | 11.44 | 5 | F5:2290 | 05112019\_RID\_1313\_NaNaRj\_F5.raw |  | 1.62E6 | 3.06E4 |  | 4.88E5 |  |  |  |  | 1.13E4 | 4 | 0 | 1 | 1 | 0 | 1 | 0 | 0 | 0 | 0 | 1 | 377 | 385 |  |
| R.VLDELTLAR.A | N | 57.93 | 1028.5865 | 9 | -0.6 | 515.3002 | 2 | 13.17 | 5 | F5:3428 | 05112019\_RID\_1313\_NaNaRj\_F5.raw |  |  | 2.21E5 | 7.14E4 | 4.87E5 | 1.16E5 | 6.95E4 | 2.69E4 |  | 2.37E4 | 7 | 0 | 0 | 1 | 1 | 1 | 1 | 1 | 1 | 0 | 1 | 193 | 201 |  |
| R.GQVGGEINVEM(+15.99)DAAPGVDLSR.I | N | 55.86 | 2129.0059 | 21 | 0.7 | 1065.5110 | 2 | 12.62 | 3 | F3:2908 | 05112019\_RID\_1313\_NaNaRj\_F3.raw |  |  | 2.08E4 |  |  |  |  |  |  |  | 1 | 0 | 0 | 1 | 0 | 0 | 0 | 0 | 0 | 0 | 0 | 231 | 251 | Oxidation (M) |
| L.IGSVEEQLAQLR.C | N | 52.90 | 1341.7252 | 12 | 0.3 | 671.8701 | 2 | 53.67 | 2 | F2:17947 | 09012020\_NaNaRj\_F2.raw |  | 5.83E4 |  |  | 4.4E4 |  |  |  |  |  | 2 | 0 | 1 | 0 | 0 | 1 | 0 | 0 | 0 | 0 | 0 | 346 | 357 |  |
| K.TRLEQEIATYR.R | N | 50.16 | 1378.7205 | 11 | 0.4 | 690.3678 | 2 | 32.95 | 2 | F2:9394 | 09012020\_NaNaRj\_F2.raw |  | 4.37E5 |  |  |  |  |  |  |  |  | 1 | 0 | 1 | 0 | 0 | 0 | 0 | 0 | 0 | 0 | 0 | 375 | 385 |  |
| R.LASYLDK.V | N | 47.75 | 808.4330 | 7 | 0.6 | 405.2241 | 2 | 11.34 | 5 | F5:2185 | 05112019\_RID\_1313\_NaNaRj\_F5.raw | 3.03E4 | 9.71E6 | 7.78E4 |  | 7.33E5 |  |  |  |  | 3.94E4 | 5 | 1 | 1 | 1 | 0 | 1 | 0 | 0 | 0 | 0 | 1 | 95 | 101 |  |
| R.LAADDFR.T | N | 46.83 | 806.3922 | 7 | 0.6 | 404.2036 | 2 | 11.46 | 5 | F5:2311 | 05112019\_RID\_1313\_NaNaRj\_F5.raw |  | 5.1E6 | 5.72E4 |  | 4.73E5 |  | 1.38E4 |  |  | 3.3E4 | 6 | 0 | 2 | 1 | 0 | 1 | 0 | 1 | 0 | 0 | 1 | 164 | 170 |  |
| R.LASYLDKVR.A | N | 45.41 | 1063.6025 | 9 | 1.0 | 532.8091 | 2 | 27.68 | 2 | F2:7299 | 09012020\_NaNaRj\_F2.raw |  | 2.48E5 |  |  |  |  |  |  |  |  | 1 | 0 | 1 | 0 | 0 | 0 | 0 | 0 | 0 | 0 | 0 | 95 | 103 |  |
| D.NANILLQIDNAR.L | N | 45.41 | 1353.7365 | 12 | 0.5 | 677.8759 | 2 | 55.43 | 2 | F2:18549 | 09012020\_NaNaRj\_F2.raw |  | 4.41E4 |  |  |  |  |  |  |  |  | 1 | 0 | 1 | 0 | 0 | 0 | 0 | 0 | 0 | 0 | 0 | 152 | 163 |  |
| N.ILLQIDNAR.L | N | 45.23 | 1054.6135 | 9 | 0.6 | 528.3143 | 2 | 12.34 | 7 | F7:3056 | 05112019\_RID\_1313\_NaNaRj\_F7.raw |  |  |  |  |  |  | 3.53E4 |  |  |  | 1 | 0 | 0 | 0 | 0 | 0 | 0 | 1 | 0 | 0 | 0 | 155 | 163 |  |
| K.NHEEEMNALR.G | N | 43.68 | 1241.5459 | 10 | 0.1 | 621.7803 | 2 | 14.43 | 2 | F2:2709 | 09012020\_NaNaRj\_F2.raw |  | 1.14E5 |  |  |  |  |  |  |  |  | 1 | 0 | 1 | 0 | 0 | 0 | 0 | 0 | 0 | 0 | 0 | 221 | 230 |  |
| total 14 peptides |  |  |  |  |  |  |  |  |  |  |  |  |  |  |  |  |  |  |  |  |  |  |  |  |  |  |  |  |  |  |  |  |  |  |  |
| --- | --- | --- | --- | --- | --- | --- | --- | --- | --- | --- | --- | --- | --- | --- | --- | --- | --- | --- | --- | --- | --- | --- | --- | --- | --- | --- | --- | --- | --- | --- | --- | --- | --- | --- | --- |

P01427|3S11\_NAJOX

back to list

  

| Protein Coverage
| Supporting Peptides
|

Protein Coverage:

Supporting Peptides:

| Peptide | Uniq | -10lgP | Mass | Length | ppm | m/z | z | RT | Fraction | Scan | Source File | Area F1 | Area F2 | Area F3 | Area F4 | Area F5 | Area F6 | Area F7 | Area F8 | Area F9 | Area F10 | #Feature | #Feature F1 | #Feature F2 | #Feature F3 | #Feature F4 | #Feature F5 | #Feature F6 | #Feature F7 | #Feature F8 | #Feature F9 | #Feature F10 | Start | End | PTM |
| --- | --- | --- | --- | --- | --- | --- | --- | --- | --- | --- | --- | --- | --- | --- | --- | --- | --- | --- | --- | --- | --- | --- | --- | --- | --- | --- | --- | --- | --- | --- | --- | --- | --- | --- | --- |
| LEC(+57.02)HNQQSSQPPTTK.T | Y | 106.73 | 1753.8053 | 15 | -0.3 | 585.6089 | 3 | 10.77 | 1 | F1:1609 | 05112019\_RID\_1313\_NaNaRj\_F1.raw | 2.84E5 |  |  |  |  |  |  |  |  |  | 1 | 1 | 0 | 0 | 0 | 0 | 0 | 0 | 0 | 0 | 0 | 1 | 15 | Carbamidomethylation |
| K.VKPGVNLNC(+57.02)C(+57.02)R.T | Y | 84.51 | 1315.6489 | 11 | 1.4 | 439.5575 | 3 | 10.94 | 1 | F1:1755 | 05112019\_RID\_1313\_NaNaRj\_F1.raw | 1.95E7 | 4.54E7 | 2.13E7 | 4.48E5 | 8.02E3 | 1.59E4 |  |  | 4.27E3 |  | 24 | 4 | 9 | 6 | 2 | 1 | 1 | 0 | 0 | 1 | 0 | 45 | 55 | Carbamidomethylation |
| K.PGVNLNC(+57.02)C(+57.02)R.T | Y | 74.97 | 1088.4856 | 9 | 0.2 | 545.2502 | 2 | 10.94 | 1 | F1:1757 | 05112019\_RID\_1313\_NaNaRj\_F1.raw | 2.48E6 |  | 2.14E6 |  |  |  |  |  |  |  | 2 | 1 | 0 | 1 | 0 | 0 | 0 | 0 | 0 | 0 | 0 | 47 | 55 | Carbamidomethylation |
| K.VKPGVNLNC(+57.02)C(+57.02).R | Y | 66.02 | 1159.5479 | 10 | -0.5 | 580.7809 | 2 | 11.23 | 3 | F3:2051 | 05112019\_RID\_1313\_NaNaRj\_F3.raw | 1.14E5 | 5.1E6 | 1.31E5 |  |  |  |  |  |  |  | 3 | 1 | 1 | 1 | 0 | 0 | 0 | 0 | 0 | 0 | 0 | 45 | 54 | Carbamidomethylation |
| K.VKPGVNLNC(+57.02).C | Y | 58.21 | 999.5172 | 9 | 0.8 | 500.7662 | 2 | 23.38 | 2 | F2:5731 | 09012020\_NaNaRj\_F2.raw | 6.6E5 | 6.16E7 | 8.7E5 |  |  |  |  |  |  |  | 4 | 1 | 2 | 1 | 0 | 0 | 0 | 0 | 0 | 0 | 0 | 45 | 53 | Carbamidomethylation |
| K.KWWSDHR.G | Y | 50.93 | 1013.4832 | 7 | -0.1 | 507.7488 | 2 | 13.09 | 2 | F2:2244 | 09012020\_NaNaRj\_F2.raw |  | 8.29E5 |  |  |  |  |  |  |  |  | 4 | 0 | 4 | 0 | 0 | 0 | 0 | 0 | 0 | 0 | 0 | 26 | 32 |  |
| V.KPGVNLNC(+57.02)C(+57.02)R.T | Y | 41.93 | 1216.5804 | 10 | 1.0 | 609.2981 | 2 | 10.95 | 1 | F1:1751 | 05112019\_RID\_1313\_NaNaRj\_F1.raw | 2.88E5 |  |  |  |  |  |  |  |  |  | 1 | 1 | 0 | 0 | 0 | 0 | 0 | 0 | 0 | 0 | 0 | 46 | 55 | Carbamidomethylation |
| total 7 peptides |  |  |  |  |  |  |  |  |  |  |  |  |  |  |  |  |  |  |  |  |  |  |  |  |  |  |  |  |  |  |  |  |  |  |  |
| --- | --- | --- | --- | --- | --- | --- | --- | --- | --- | --- | --- | --- | --- | --- | --- | --- | --- | --- | --- | --- | --- | --- | --- | --- | --- | --- | --- | --- | --- | --- | --- | --- | --- | --- | --- |

P06394|K1C10\_BOVIN

back to list

  

| Protein Coverage
| Supporting Peptides
|

Protein Coverage:

Supporting Peptides:

| Peptide | Uniq | -10lgP | Mass | Length | ppm | m/z | z | RT | Fraction | Scan | Source File | Area F1 | Area F2 | Area F3 | Area F4 | Area F5 | Area F6 | Area F7 | Area F8 | Area F9 | Area F10 | #Feature | #Feature F1 | #Feature F2 | #Feature F3 | #Feature F4 | #Feature F5 | #Feature F6 | #Feature F7 | #Feature F8 | #Feature F9 | #Feature F10 | Start | End | PTM |
| --- | --- | --- | --- | --- | --- | --- | --- | --- | --- | --- | --- | --- | --- | --- | --- | --- | --- | --- | --- | --- | --- | --- | --- | --- | --- | --- | --- | --- | --- | --- | --- | --- | --- | --- | --- |
| K.QSLEASLAETEGR.Y | N | 84.81 | 1389.6736 | 13 | 0.5 | 695.8444 | 2 | 11.87 | 5 | F5:2708 | 05112019\_RID\_1313\_NaNaRj\_F5.raw | 2.09E4 | 5.42E4 | 2.8E5 |  | 1.2E6 |  | 1.56E5 |  |  | 3.36E4 | 6 | 1 | 1 | 1 | 0 | 1 | 0 | 1 | 0 | 0 | 1 | 368 | 380 |  |
| R.VLDELTLTK.T | N | 69.64 | 1030.5911 | 9 | -0.4 | 516.3026 | 2 | 12.62 | 4 | F4:3299 | 05112019\_RID\_1313\_NaNaRj\_F4.raw | 1.03E5 | 1.18E7 | 7.95E5 | 2.87E5 | 1.99E6 | 2.83E5 | 2.47E5 | 6.56E4 |  | 7.86E4 | 10 | 1 | 2 | 1 | 1 | 1 | 1 | 1 | 1 | 0 | 1 | 239 | 247 |  |
| R.LENEIQTYR.S | N | 65.53 | 1164.5775 | 9 | 0.1 | 583.2961 | 2 | 11.38 | 5 | F5:2209 | 05112019\_RID\_1313\_NaNaRj\_F5.raw | 7.77E4 | 9.7E6 | 2.68E5 | 1.28E5 | 2.96E6 |  |  |  |  | 1.17E5 | 9 | 1 | 4 | 1 | 1 | 1 | 0 | 0 | 0 | 0 | 1 | 423 | 431 |  |
| R.QSVEADINGLR.R | N | 63.94 | 1200.6099 | 11 | -0.1 | 601.3121 | 2 | 39.03 | 2 | F2:11958 | 09012020\_NaNaRj\_F2.raw | 4.07E4 | 4.05E6 |  |  | 0 |  |  |  |  |  | 5 | 1 | 3 | 0 | 0 | 1 | 0 | 0 | 0 | 0 | 0 | 227 | 237 |  |
| L.RQSVEADINGLR.R | N | 57.75 | 1356.7109 | 12 | -0.5 | 453.2440 | 3 | 28.20 | 2 | F2:7574 | 09012020\_NaNaRj\_F2.raw |  | 4.85E4 |  |  |  |  |  |  |  |  | 1 | 0 | 1 | 0 | 0 | 0 | 0 | 0 | 0 | 0 | 0 | 226 | 237 |  |
| R.DAEAWFNEK.S | N | 56.45 | 1108.4825 | 9 | 0.2 | 555.2487 | 2 | 13.37 | 4 | F4:3903 | 05112019\_RID\_1313\_NaNaRj\_F4.raw | 3.31E4 | 2.81E6 | 5.8E5 | 1.8E5 | 1.24E6 | 2.74E5 | 1.77E5 |  | 9.52E3 | 3.81E4 | 11 | 1 | 2 | 1 | 1 | 2 | 1 | 1 | 0 | 1 | 1 | 316 | 324 |  |
| K.IRLENEIQTYR.S | N | 50.89 | 1433.7627 | 11 | -0.3 | 478.9280 | 3 | 34.87 | 2 | F2:10211 | 09012020\_NaNaRj\_F2.raw |  | 1.25E6 |  |  |  |  |  |  |  |  | 2 | 0 | 2 | 0 | 0 | 0 | 0 | 0 | 0 | 0 | 0 | 421 | 431 |  |
| E.SLTEELAYLK.K | N | 49.27 | 1165.6230 | 10 | 0.9 | 583.8193 | 2 | 58.68 | 2 | F2:19462 | 09012020\_NaNaRj\_F2.raw |  | 3.58E6 |  |  |  |  |  |  |  |  | 1 | 0 | 1 | 0 | 0 | 0 | 0 | 0 | 0 | 0 | 0 | 256 | 265 |  |
| R.LASYLDK.V | N | 47.75 | 808.4330 | 7 | 0.6 | 405.2241 | 2 | 11.34 | 5 | F5:2185 | 05112019\_RID\_1313\_NaNaRj\_F5.raw | 3.03E4 | 9.71E6 | 7.78E4 |  | 7.33E5 |  |  |  |  | 3.94E4 | 5 | 1 | 1 | 1 | 0 | 1 | 0 | 0 | 0 | 0 | 1 | 138 | 144 |  |
| R.LAADDFR.L | N | 46.83 | 806.3922 | 7 | 0.6 | 404.2036 | 2 | 11.46 | 5 | F5:2311 | 05112019\_RID\_1313\_NaNaRj\_F5.raw |  | 5.1E6 | 5.72E4 |  | 4.73E5 |  | 1.38E4 |  |  | 3.3E4 | 6 | 0 | 2 | 1 | 0 | 1 | 0 | 1 | 0 | 0 | 1 | 210 | 216 |  |
| R.LASYLDKVR.A | N | 45.41 | 1063.6025 | 9 | 1.0 | 532.8091 | 2 | 27.68 | 2 | F2:7299 | 09012020\_NaNaRj\_F2.raw |  | 2.48E5 |  |  |  |  |  |  |  |  | 1 | 0 | 1 | 0 | 0 | 0 | 0 | 0 | 0 | 0 | 0 | 138 | 146 |  |
| Y.SSGGFSGGSFSR.G | N | 41.79 | 1131.4945 | 12 | 0.2 | 566.7546 | 2 | 11.34 | 5 | F5:2187 | 05112019\_RID\_1313\_NaNaRj\_F5.raw |  |  |  |  | 8.47E4 |  |  |  |  |  | 1 | 0 | 0 | 0 | 0 | 1 | 0 | 0 | 0 | 0 | 0 | 40 | 51 |  |
| K.SEITELR.R | N | 41.71 | 846.4447 | 7 | 0.9 | 424.2300 | 2 | 11.36 | 5 | F5:2206 | 05112019\_RID\_1313\_NaNaRj\_F5.raw |  |  |  |  | 9.35E5 |  |  |  |  |  | 1 | 0 | 0 | 0 | 0 | 1 | 0 | 0 | 0 | 0 | 0 | 344 | 350 |  |
| R.SQYEQLAEKNR.R | Y | 41.17 | 1364.6685 | 11 | 5.3 | 683.3451 | 2 | 11.34 | 5 | F5:2221 | 05112019\_RID\_1313\_NaNaRj\_F5.raw |  |  |  |  | 1.32E4 |  |  |  |  |  | 1 | 0 | 0 | 0 | 0 | 1 | 0 | 0 | 0 | 0 | 0 | 304 | 314 |  |
| total 14 peptides |  |  |  |  |  |  |  |  |  |  |  |  |  |  |  |  |  |  |  |  |  |  |  |  |  |  |  |  |  |  |  |  |  |  |  |
| --- | --- | --- | --- | --- | --- | --- | --- | --- | --- | --- | --- | --- | --- | --- | --- | --- | --- | --- | --- | --- | --- | --- | --- | --- | --- | --- | --- | --- | --- | --- | --- | --- | --- | --- | --- |

D5LMJ3|VM3A\_NAJAT

back to list

  

| Protein Coverage
| Supporting Peptides
|

Protein Coverage:

Supporting Peptides:

| Peptide | Uniq | -10lgP | Mass | Length | ppm | m/z | z | RT | Fraction | Scan | Source File | Area F1 | Area F2 | Area F3 | Area F4 | Area F5 | Area F6 | Area F7 | Area F8 | Area F9 | Area F10 | #Feature | #Feature F1 | #Feature F2 | #Feature F3 | #Feature F4 | #Feature F5 | #Feature F6 | #Feature F7 | #Feature F8 | #Feature F9 | #Feature F10 | Start | End | PTM |
| --- | --- | --- | --- | --- | --- | --- | --- | --- | --- | --- | --- | --- | --- | --- | --- | --- | --- | --- | --- | --- | --- | --- | --- | --- | --- | --- | --- | --- | --- | --- | --- | --- | --- | --- | --- |
| K.C(+57.02)GTLYC(+57.02)TEIK.K | Y | 83.89 | 1243.5577 | 10 | -1.0 | 622.7855 | 2 | 11.57 | 8 | F8:2386 | 05112019\_RID\_1313\_NaNaRj\_F8.raw |  |  |  |  |  |  |  | 2.96E5 | 1.19E5 | 6.53E3 | 3 | 0 | 0 | 0 | 0 | 0 | 0 | 0 | 1 | 1 | 1 | 560 | 569 | Carbamidomethylation |
| K.TGC(+57.02)IVPVSPR.D | Y | 78.07 | 1084.5699 | 10 | 0.4 | 543.2925 | 2 | 11.59 | 9 | F9:2285 | 05112019\_RID\_1313\_NaNaRj\_F9.raw |  |  |  |  |  |  |  |  | 8.35E4 |  | 1 | 0 | 0 | 0 | 0 | 0 | 0 | 0 | 0 | 1 | 0 | 571 | 580 | Carbamidomethylation |
| R.NGHPC(+57.02)QNNQGYC(+57.02)YNGK.C | N | 69.82 | 1909.7584 | 16 | -0.1 | 637.5934 | 3 | 10.66 | 9 | F9:1607 | 05112019\_RID\_1313\_NaNaRj\_F9.raw |  |  |  |  |  |  |  |  | 6.59E4 |  | 1 | 0 | 0 | 0 | 0 | 0 | 0 | 0 | 0 | 1 | 0 | 492 | 507 | Carbamidomethylation |
| R.ERPQC(+57.02)ILNKPSR.K | Y | 64.92 | 1496.7881 | 12 | -0.5 | 499.9364 | 3 | 10.69 | 9 | F9:1629 | 05112019\_RID\_1313\_NaNaRj\_F9.raw |  |  |  |  |  |  |  | 8.8E4 | 4.84E4 |  | 3 | 0 | 0 | 0 | 0 | 0 | 0 | 0 | 2 | 1 | 0 | 389 | 400 | Carbamidomethylation |
| W.GSVAVVQDYSR.R | Y | 62.94 | 1179.5884 | 11 | -1.4 | 590.8007 | 2 | 11.44 | 8 | F8:2273 | 05112019\_RID\_1313\_NaNaRj\_F8.raw |  |  |  |  |  |  |  | 1.58E5 | 2.46E5 | 4.08E3 | 3 | 0 | 0 | 0 | 0 | 0 | 0 | 0 | 1 | 1 | 1 | 317 | 327 |  |
| R.VYEMVNYLNTK.Y | Y | 62.10 | 1372.6697 | 11 | 0.1 | 687.3422 | 2 | 13.42 | 8 | F8:3751 | 05112019\_RID\_1313\_NaNaRj\_F8.raw |  |  |  |  |  |  |  | 5.97E5 | 5.94E4 |  | 2 | 0 | 0 | 0 | 0 | 0 | 0 | 0 | 1 | 1 | 0 | 231 | 241 |  |
| K.YIEFYLVVDNK.M | Y | 61.47 | 1401.7180 | 11 | 0.1 | 701.8663 | 2 | 27.17 | 8 | F8:9934 | 05112019\_RID\_1313\_NaNaRj\_F8.raw |  |  |  |  |  |  |  | 1.06E5 |  |  | 1 | 0 | 0 | 0 | 0 | 0 | 0 | 0 | 1 | 0 | 0 | 206 | 216 |  |
| K.LQPHAQC(+57.02)DSEEC(+57.02)C(+57.02)EK.C | Y | 60.57 | 1889.7343 | 15 | -0.1 | 630.9186 | 3 | 10.66 | 9 | F9:1608 | 05112019\_RID\_1313\_NaNaRj\_F9.raw |  |  |  |  |  |  |  | 8.19E4 | 4.78E4 |  | 2 | 0 | 0 | 0 | 0 | 0 | 0 | 0 | 1 | 1 | 0 | 440 | 454 | Carbamidomethylation |
| R.VYEM(+15.99)VNYLNTK.Y | Y | 55.15 | 1388.6646 | 11 | 0.1 | 695.3396 | 2 | 11.69 | 9 | F9:2373 | 05112019\_RID\_1313\_NaNaRj\_F9.raw |  |  |  |  |  |  |  | 5.2E3 | 7.57E4 |  | 3 | 0 | 0 | 0 | 0 | 0 | 0 | 0 | 2 | 1 | 0 | 231 | 241 | Oxidation (M) |
| K.C(+57.02)PTM(+15.99)ENQC(+57.02)ITLLGPNYTVGPAGC(+57.02)FK.N | Y | 41.60 | 2843.2751 | 25 | -0.1 | 1422.6447 | 2 | 23.97 | 8 | F8:8194 | 05112019\_RID\_1313\_NaNaRj\_F8.raw |  |  |  |  |  |  |  | 2.28E4 |  |  | 1 | 0 | 0 | 0 | 0 | 0 | 0 | 0 | 1 | 0 | 0 | 508 | 532 | Carbamidomethylation; Oxidation (M) |
| total 10 peptides |  |  |  |  |  |  |  |  |  |  |  |  |  |  |  |  |  |  |  |  |  |  |  |  |  |  |  |  |  |  |  |  |  |  |  |
| --- | --- | --- | --- | --- | --- | --- | --- | --- | --- | --- | --- | --- | --- | --- | --- | --- | --- | --- | --- | --- | --- | --- | --- | --- | --- | --- | --- | --- | --- | --- | --- | --- | --- | --- | --- |

P00761|TRYP\_PIG

back to list

  

| Protein Coverage
| Supporting Peptides
|

Protein Coverage:

Supporting Peptides:

| Peptide | Uniq | -10lgP | Mass | Length | ppm | m/z | z | RT | Fraction | Scan | Source File | Area F1 | Area F2 | Area F3 | Area F4 | Area F5 | Area F6 | Area F7 | Area F8 | Area F9 | Area F10 | #Feature | #Feature F1 | #Feature F2 | #Feature F3 | #Feature F4 | #Feature F5 | #Feature F6 | #Feature F7 | #Feature F8 | #Feature F9 | #Feature F10 | Start | End | PTM |
| --- | --- | --- | --- | --- | --- | --- | --- | --- | --- | --- | --- | --- | --- | --- | --- | --- | --- | --- | --- | --- | --- | --- | --- | --- | --- | --- | --- | --- | --- | --- | --- | --- | --- | --- | --- |
| K.IITHPNFNGN.T | Y | 68.93 | 1125.5566 | 10 | 0.1 | 563.7856 | 2 | 11.43 | 9 | F9:2133 | 05112019\_RID\_1313\_NaNaRj\_F9.raw | 2E5 |  | 9.84E4 |  |  |  |  |  | 3.18E5 | 7.24E4 | 4 | 1 | 0 | 1 | 0 | 0 | 0 | 0 | 0 | 1 | 1 | 78 | 87 |  |
| R.LGEHNIDVLEGNEQFIN.A | Y | 63.34 | 1939.9275 | 17 | 0.3 | 970.9713 | 2 | 62.87 | 2 | F2:20827 | 09012020\_NaNaRj\_F2.raw |  | 1.89E6 | 2.13E5 | 2.73E6 |  | 1.68E6 |  |  | 4.22E6 |  | 6 | 0 | 1 | 1 | 1 | 0 | 1 | 0 | 0 | 2 | 0 | 58 | 74 |  |
| R.LGEHNIDVLEGNEQ.F | Y | 62.68 | 1565.7322 | 14 | 0.4 | 783.8737 | 2 | 12.02 | 1 | F1:2740 | 05112019\_RID\_1313\_NaNaRj\_F1.raw | 6.96E4 |  |  |  |  |  |  |  |  |  | 1 | 1 | 0 | 0 | 0 | 0 | 0 | 0 | 0 | 0 | 0 | 58 | 71 |  |
| K.SSYPGQITGN.M | Y | 61.41 | 1022.4669 | 10 | 0.3 | 512.2408 | 2 | 11.66 | 9 | F9:2347 | 05112019\_RID\_1313\_NaNaRj\_F9.raw | 3.21E5 |  | 1.13E5 |  |  |  |  |  | 1.05E5 |  | 3 | 1 | 0 | 1 | 0 | 0 | 0 | 0 | 0 | 1 | 0 | 158 | 167 |  |
| R.LGEHNIDVLEGNEQF.I | Y | 59.65 | 1712.8005 | 15 | 0.6 | 857.4081 | 2 | 17.14 | 8 | F8:5563 | 05112019\_RID\_1313\_NaNaRj\_F8.raw |  |  |  | 2.36E5 |  |  |  | 1.16E5 | 6.53E5 |  | 3 | 0 | 0 | 0 | 1 | 0 | 0 | 0 | 1 | 1 | 0 | 58 | 72 |  |
| N.YVNWIQQTIAAN | Y | 57.47 | 1419.7146 | 12 | 0.5 | 710.8649 | 2 | 31.53 | 1 | F1:6441 | 05112019\_RID\_1313\_NaNaRj\_F1.raw | 3E6 |  |  |  |  |  |  |  | 2.58E6 |  | 3 | 1 | 0 | 0 | 0 | 0 | 0 | 0 | 0 | 2 | 0 | 220 | 231 |  |
| K.LSSPATLNSR.V | N | 56.40 | 1044.5564 | 10 | -1.0 | 523.2850 | 2 | 11.24 | 1 | F1:2001 | 05112019\_RID\_1313\_NaNaRj\_F1.raw | 3.24E5 | 1.6E6 | 5.55E4 | 0 | 1.26E5 |  |  |  | 2.06E5 |  | 6 | 1 | 1 | 1 | 1 | 1 | 0 | 0 | 0 | 1 | 0 | 98 | 107 |  |
| R.SC(+57.02)AAAGTEC(+57.02)LI.S | Y | 56.26 | 1151.4951 | 11 | 0.7 | 576.7552 | 2 | 50.51 | 2 | F2:16806 | 09012020\_NaNaRj\_F2.raw |  | 7.15E5 |  |  |  |  |  |  |  |  | 1 | 0 | 1 | 0 | 0 | 0 | 0 | 0 | 0 | 0 | 0 | 116 | 126 | Carbamidomethylation |
| K.IVGGYTC(+57.02)AAN.S | Y | 51.16 | 1024.4647 | 10 | 0.8 | 513.2401 | 2 | 11.58 | 1 | F1:2339 | 05112019\_RID\_1313\_NaNaRj\_F1.raw | 1.14E5 |  |  |  |  |  |  |  |  |  | 1 | 1 | 0 | 0 | 0 | 0 | 0 | 0 | 0 | 0 | 0 | 9 | 18 | Carbamidomethylation |
| D.VLEGNEQFINAAK.I | N | 50.49 | 1431.7357 | 13 | 0.8 | 716.8757 | 2 | 40.68 | 2 | F2:12665 | 09012020\_NaNaRj\_F2.raw |  | 1.17E4 |  |  |  |  |  |  |  |  | 1 | 0 | 1 | 0 | 0 | 0 | 0 | 0 | 0 | 0 | 0 | 65 | 77 |  |
| K.IITHPNFN.G | Y | 50.20 | 954.4923 | 8 | 0.0 | 478.2534 | 2 | 30.36 | 2 | F2:8544 | 09012020\_NaNaRj\_F2.raw |  | 4.65E6 |  |  |  |  |  |  |  |  | 1 | 0 | 1 | 0 | 0 | 0 | 0 | 0 | 0 | 0 | 0 | 78 | 85 |  |
| R.SC(+57.02)AAAGTEC(+57.02)LIS.G | Y | 48.81 | 1238.5271 | 12 | 0.2 | 620.2709 | 2 | 43.76 | 2 | F2:13885 | 09012020\_NaNaRj\_F2.raw |  | 8.74E5 |  |  |  |  |  |  |  |  | 1 | 0 | 1 | 0 | 0 | 0 | 0 | 0 | 0 | 0 | 0 | 116 | 127 | Carbamidomethylation |
| K.SSGSSYPSLLQC(+57.02).L | Y | 48.29 | 1284.5656 | 12 | 0.6 | 643.2904 | 2 | 57.89 | 2 | F2:19231 | 09012020\_NaNaRj\_F2.raw |  | 2.97E5 |  |  |  |  |  |  |  |  | 1 | 0 | 1 | 0 | 0 | 0 | 0 | 0 | 0 | 0 | 0 | 134 | 145 | Carbamidomethylation |
| R.LGEHNIDVLEGNEQFINAA.K | Y | 48.23 | 2082.0017 | 19 | 0.4 | 1042.0085 | 2 | 21.68 | 6 | F6:9015 | 05112019\_RID\_1313\_NaNaRj\_F6.raw |  |  |  |  |  | 3.69E5 |  |  |  |  | 1 | 0 | 0 | 0 | 0 | 0 | 1 | 0 | 0 | 0 | 0 | 58 | 76 |  |
| R.LGEHNIDVLEGN.E | Y | 48.02 | 1308.6310 | 12 | 0.2 | 655.3229 | 2 | 12.09 | 9 | F9:2697 | 05112019\_RID\_1313\_NaNaRj\_F9.raw |  |  |  |  |  |  |  |  | 1.61E4 |  | 1 | 0 | 0 | 0 | 0 | 0 | 0 | 0 | 0 | 1 | 0 | 58 | 69 |  |
| N.WIQQTIAAN | N | 41.69 | 1043.5400 | 9 | 0.1 | 522.7773 | 2 | 13.31 | 5 | F5:3459 | 05112019\_RID\_1313\_NaNaRj\_F5.raw |  |  |  |  | 1.55E5 |  |  |  |  |  | 1 | 0 | 0 | 0 | 0 | 1 | 0 | 0 | 0 | 0 | 0 | 223 | 231 |  |
| total 16 peptides |  |  |  |  |  |  |  |  |  |  |  |  |  |  |  |  |  |  |  |  |  |  |  |  |  |  |  |  |  |  |  |  |  |  |  |
| --- | --- | --- | --- | --- | --- | --- | --- | --- | --- | --- | --- | --- | --- | --- | --- | --- | --- | --- | --- | --- | --- | --- | --- | --- | --- | --- | --- | --- | --- | --- | --- | --- | --- | --- | --- |

#CONTAM#TRYP\_PIG|

back to list

  

| Protein Coverage
| Supporting Peptides
|

Protein Coverage:

Supporting Peptides:

| Peptide | Uniq | -10lgP | Mass | Length | ppm | m/z | z | RT | Fraction | Scan | Source File | Area F1 | Area F2 | Area F3 | Area F4 | Area F5 | Area F6 | Area F7 | Area F8 | Area F9 | Area F10 | #Feature | #Feature F1 | #Feature F2 | #Feature F3 | #Feature F4 | #Feature F5 | #Feature F6 | #Feature F7 | #Feature F8 | #Feature F9 | #Feature F10 | Start | End | PTM |
| --- | --- | --- | --- | --- | --- | --- | --- | --- | --- | --- | --- | --- | --- | --- | --- | --- | --- | --- | --- | --- | --- | --- | --- | --- | --- | --- | --- | --- | --- | --- | --- | --- | --- | --- | --- |
| K.IITHPNFNGN.T | Y | 68.93 | 1125.5566 | 10 | 0.1 | 563.7856 | 2 | 11.43 | 9 | F9:2133 | 05112019\_RID\_1313\_NaNaRj\_F9.raw | 2E5 |  | 9.84E4 |  |  |  |  |  | 3.18E5 | 7.24E4 | 4 | 1 | 0 | 1 | 0 | 0 | 0 | 0 | 0 | 1 | 1 | 78 | 87 |  |
| R.LGEHNIDVLEGNEQFIN.A | Y | 63.34 | 1939.9275 | 17 | 0.3 | 970.9713 | 2 | 62.87 | 2 | F2:20827 | 09012020\_NaNaRj\_F2.raw |  | 1.89E6 | 2.13E5 | 2.73E6 |  | 1.68E6 |  |  | 4.22E6 |  | 6 | 0 | 1 | 1 | 1 | 0 | 1 | 0 | 0 | 2 | 0 | 58 | 74 |  |
| R.LGEHNIDVLEGNEQ.F | Y | 62.68 | 1565.7322 | 14 | 0.4 | 783.8737 | 2 | 12.02 | 1 | F1:2740 | 05112019\_RID\_1313\_NaNaRj\_F1.raw | 6.96E4 |  |  |  |  |  |  |  |  |  | 1 | 1 | 0 | 0 | 0 | 0 | 0 | 0 | 0 | 0 | 0 | 58 | 71 |  |
| K.SSYPGQITGN.M | Y | 61.41 | 1022.4669 | 10 | 0.3 | 512.2408 | 2 | 11.66 | 9 | F9:2347 | 05112019\_RID\_1313\_NaNaRj\_F9.raw | 3.21E5 |  | 1.13E5 |  |  |  |  |  | 1.05E5 |  | 3 | 1 | 0 | 1 | 0 | 0 | 0 | 0 | 0 | 1 | 0 | 158 | 167 |  |
| R.LGEHNIDVLEGNEQF.I | Y | 59.65 | 1712.8005 | 15 | 0.6 | 857.4081 | 2 | 17.14 | 8 | F8:5563 | 05112019\_RID\_1313\_NaNaRj\_F8.raw |  |  |  | 2.36E5 |  |  |  | 1.16E5 | 6.53E5 |  | 3 | 0 | 0 | 0 | 1 | 0 | 0 | 0 | 1 | 1 | 0 | 58 | 72 |  |
| N.YVNWIQQTIAAN | Y | 57.47 | 1419.7146 | 12 | 0.5 | 710.8649 | 2 | 31.53 | 1 | F1:6441 | 05112019\_RID\_1313\_NaNaRj\_F1.raw | 3E6 |  |  |  |  |  |  |  | 2.58E6 |  | 3 | 1 | 0 | 0 | 0 | 0 | 0 | 0 | 0 | 2 | 0 | 220 | 231 |  |
| K.LSSPATLNSR.V | N | 56.40 | 1044.5564 | 10 | -1.0 | 523.2850 | 2 | 11.24 | 1 | F1:2001 | 05112019\_RID\_1313\_NaNaRj\_F1.raw | 3.24E5 | 1.6E6 | 5.55E4 | 0 | 1.26E5 |  |  |  | 2.06E5 |  | 6 | 1 | 1 | 1 | 1 | 1 | 0 | 0 | 0 | 1 | 0 | 98 | 107 |  |
| R.SC(+57.02)AAAGTEC(+57.02)LI.S | Y | 56.26 | 1151.4951 | 11 | 0.7 | 576.7552 | 2 | 50.51 | 2 | F2:16806 | 09012020\_NaNaRj\_F2.raw |  | 7.15E5 |  |  |  |  |  |  |  |  | 1 | 0 | 1 | 0 | 0 | 0 | 0 | 0 | 0 | 0 | 0 | 116 | 126 | Carbamidomethylation |
| K.IVGGYTC(+57.02)AAN.S | Y | 51.16 | 1024.4647 | 10 | 0.8 | 513.2401 | 2 | 11.58 | 1 | F1:2339 | 05112019\_RID\_1313\_NaNaRj\_F1.raw | 1.14E5 |  |  |  |  |  |  |  |  |  | 1 | 1 | 0 | 0 | 0 | 0 | 0 | 0 | 0 | 0 | 0 | 9 | 18 | Carbamidomethylation |
| D.VLEGNEQFINAAK.I | N | 50.49 | 1431.7357 | 13 | 0.8 | 716.8757 | 2 | 40.68 | 2 | F2:12665 | 09012020\_NaNaRj\_F2.raw |  | 1.17E4 |  |  |  |  |  |  |  |  | 1 | 0 | 1 | 0 | 0 | 0 | 0 | 0 | 0 | 0 | 0 | 65 | 77 |  |
| K.IITHPNFN.G | Y | 50.20 | 954.4923 | 8 | 0.0 | 478.2534 | 2 | 30.36 | 2 | F2:8544 | 09012020\_NaNaRj\_F2.raw |  | 4.65E6 |  |  |  |  |  |  |  |  | 1 | 0 | 1 | 0 | 0 | 0 | 0 | 0 | 0 | 0 | 0 | 78 | 85 |  |
| R.SC(+57.02)AAAGTEC(+57.02)LIS.G | Y | 48.81 | 1238.5271 | 12 | 0.2 | 620.2709 | 2 | 43.76 | 2 | F2:13885 | 09012020\_NaNaRj\_F2.raw |  | 8.74E5 |  |  |  |  |  |  |  |  | 1 | 0 | 1 | 0 | 0 | 0 | 0 | 0 | 0 | 0 | 0 | 116 | 127 | Carbamidomethylation |
| K.SSGSSYPSLLQC(+57.02).L | Y | 48.29 | 1284.5656 | 12 | 0.6 | 643.2904 | 2 | 57.89 | 2 | F2:19231 | 09012020\_NaNaRj\_F2.raw |  | 2.97E5 |  |  |  |  |  |  |  |  | 1 | 0 | 1 | 0 | 0 | 0 | 0 | 0 | 0 | 0 | 0 | 134 | 145 | Carbamidomethylation |
| R.LGEHNIDVLEGNEQFINAA.K | Y | 48.23 | 2082.0017 | 19 | 0.4 | 1042.0085 | 2 | 21.68 | 6 | F6:9015 | 05112019\_RID\_1313\_NaNaRj\_F6.raw |  |  |  |  |  | 3.69E5 |  |  |  |  | 1 | 0 | 0 | 0 | 0 | 0 | 1 | 0 | 0 | 0 | 0 | 58 | 76 |  |
| R.LGEHNIDVLEGN.E | Y | 48.02 | 1308.6310 | 12 | 0.2 | 655.3229 | 2 | 12.09 | 9 | F9:2697 | 05112019\_RID\_1313\_NaNaRj\_F9.raw |  |  |  |  |  |  |  |  | 1.61E4 |  | 1 | 0 | 0 | 0 | 0 | 0 | 0 | 0 | 0 | 1 | 0 | 58 | 69 |  |
| N.WIQQTIAAN | N | 41.69 | 1043.5400 | 9 | 0.1 | 522.7773 | 2 | 13.31 | 5 | F5:3459 | 05112019\_RID\_1313\_NaNaRj\_F5.raw |  |  |  |  | 1.55E5 |  |  |  |  |  | 1 | 0 | 0 | 0 | 0 | 1 | 0 | 0 | 0 | 0 | 0 | 223 | 231 |  |
| total 16 peptides |  |  |  |  |  |  |  |  |  |  |  |  |  |  |  |  |  |  |  |  |  |  |  |  |  |  |  |  |  |  |  |  |  |  |  |
| --- | --- | --- | --- | --- | --- | --- | --- | --- | --- | --- | --- | --- | --- | --- | --- | --- | --- | --- | --- | --- | --- | --- | --- | --- | --- | --- | --- | --- | --- | --- | --- | --- | --- | --- | --- |

P20229|VKTTI\_NAJNA

back to list

  

| Protein Coverage
| Supporting Peptides
|

Protein Coverage:

Supporting Peptides:

| Peptide | Uniq | -10lgP | Mass | Length | ppm | m/z | z | RT | Fraction | Scan | Source File | Area F1 | Area F2 | Area F3 | Area F4 | Area F5 | Area F6 | Area F7 | Area F8 | Area F9 | Area F10 | #Feature | #Feature F1 | #Feature F2 | #Feature F3 | #Feature F4 | #Feature F5 | #Feature F6 | #Feature F7 | #Feature F8 | #Feature F9 | #Feature F10 | Start | End | PTM |
| --- | --- | --- | --- | --- | --- | --- | --- | --- | --- | --- | --- | --- | --- | --- | --- | --- | --- | --- | --- | --- | --- | --- | --- | --- | --- | --- | --- | --- | --- | --- | --- | --- | --- | --- | --- |
| K.AHKPAFYYNK.D | Y | 87.66 | 1237.6244 | 10 | 1.0 | 413.5491 | 3 | 12.46 | 2 | F2:2028 | 09012020\_NaNaRj\_F2.raw |  | 2.04E6 |  | 1.54E6 |  |  |  |  |  |  | 3 | 0 | 2 | 0 | 1 | 0 | 0 | 0 | 0 | 0 | 0 | 16 | 25 |  |
| K.FIYGGC(+57.02)GGNANR.F | Y | 85.14 | 1284.5669 | 12 | 0.6 | 643.2911 | 2 | 11.33 | 4 | F4:2176 | 05112019\_RID\_1313\_NaNaRj\_F4.raw |  | 1.95E7 |  | 1.86E6 |  | 5.38E4 | 6.12E4 |  |  |  | 9 | 0 | 6 | 0 | 1 | 0 | 1 | 1 | 0 | 0 | 0 | 33 | 44 | Carbamidomethylation |
| RPGFC(+57.02)ELPAAK.G | Y | 80.08 | 1244.6335 | 11 | -0.7 | 415.8848 | 3 | 11.29 | 4 | F4:2142 | 05112019\_RID\_1313\_NaNaRj\_F4.raw |  | 4.02E7 |  | 2.17E6 |  | 1.01E4 |  |  |  |  | 7 | 0 | 4 | 0 | 2 | 0 | 1 | 0 | 0 | 0 | 0 | 1 | 11 | Carbamidomethylation |
| Q.KFIYGGC(+57.02)GGNANR.F | Y | 69.04 | 1412.6619 | 13 | -0.3 | 471.8944 | 3 | 16.42 | 2 | F2:3076 | 09012020\_NaNaRj\_F2.raw |  | 7.74E4 |  |  |  |  |  |  |  |  | 1 | 0 | 1 | 0 | 0 | 0 | 0 | 0 | 0 | 0 | 0 | 32 | 44 | Carbamidomethylation |
| R.TIDEC(+57.02)NRTC(+57.02)VG | N | 46.81 | 1323.5547 | 11 | 0.3 | 662.7848 | 2 | 10.95 | 5 | F5:1816 | 05112019\_RID\_1313\_NaNaRj\_F5.raw |  |  |  |  | 4.15E4 |  |  |  |  |  | 1 | 0 | 0 | 0 | 0 | 1 | 0 | 0 | 0 | 0 | 0 | 47 | 57 | Carbamidomethylation |
| K.AHKPAFYY.N | Y | 44.37 | 995.4865 | 8 | 0.7 | 498.7509 | 2 | 24.13 | 2 | F2:5888 | 09012020\_NaNaRj\_F2.raw |  | 3.37E5 |  |  |  |  |  |  |  |  | 1 | 0 | 1 | 0 | 0 | 0 | 0 | 0 | 0 | 0 | 0 | 16 | 23 |  |
| total 6 peptides |  |  |  |  |  |  |  |  |  |  |  |  |  |  |  |  |  |  |  |  |  |  |  |  |  |  |  |  |  |  |  |  |  |  |  |
| --- | --- | --- | --- | --- | --- | --- | --- | --- | --- | --- | --- | --- | --- | --- | --- | --- | --- | --- | --- | --- | --- | --- | --- | --- | --- | --- | --- | --- | --- | --- | --- | --- | --- | --- | --- |

P82464|3SO8\_NAJKA

back to list

  

| Protein Coverage
| Supporting Peptides
|

Protein Coverage:

Supporting Peptides:

| Peptide | Uniq | -10lgP | Mass | Length | ppm | m/z | z | RT | Fraction | Scan | Source File | Area F1 | Area F2 | Area F3 | Area F4 | Area F5 | Area F6 | Area F7 | Area F8 | Area F9 | Area F10 | #Feature | #Feature F1 | #Feature F2 | #Feature F3 | #Feature F4 | #Feature F5 | #Feature F6 | #Feature F7 | #Feature F8 | #Feature F9 | #Feature F10 | Start | End | PTM |
| --- | --- | --- | --- | --- | --- | --- | --- | --- | --- | --- | --- | --- | --- | --- | --- | --- | --- | --- | --- | --- | --- | --- | --- | --- | --- | --- | --- | --- | --- | --- | --- | --- | --- | --- | --- |
| R.TSETTEIC(+57.02)PDSWYFC(+57.02)YK.I | Y | 94.33 | 2185.8972 | 17 | 0.3 | 1093.9562 | 2 | 23.19 | 6 | F6:9899 | 05112019\_RID\_1313\_NaNaRj\_F6.raw |  |  |  |  |  | 1.24E7 |  |  |  |  | 1 | 0 | 0 | 0 | 0 | 0 | 1 | 0 | 0 | 0 | 0 | 10 | 26 | Carbamidomethylation |
| R.GC(+57.02)TFTC(+57.02)PELRPTGIYVYC(+57.02).C | Y | 84.90 | 2192.9692 | 18 | 0.7 | 1097.4927 | 2 | 22.46 | 6 | F6:9393 | 05112019\_RID\_1313\_NaNaRj\_F6.raw |  |  |  |  |  | 1.65E6 |  |  |  |  | 2 | 0 | 0 | 0 | 0 | 0 | 2 | 0 | 0 | 0 | 0 | 40 | 57 | Carbamidomethylation |
| R.GC(+57.02)TFTC(+57.02)PELRPTGIYVY.C | Y | 80.71 | 2032.9386 | 17 | 0.7 | 1017.4773 | 2 | 23.59 | 6 | F6:9933 | 05112019\_RID\_1313\_NaNaRj\_F6.raw |  |  |  |  |  | 4.3E6 |  |  |  |  | 1 | 0 | 0 | 0 | 0 | 0 | 1 | 0 | 0 | 0 | 0 | 40 | 56 | Carbamidomethylation |
| R.GC(+57.02)TFTC(+57.02)PELRPTGIYVYC(+57.02)C(+57.02)R.R | Y | 72.10 | 2509.1011 | 20 | 0.5 | 837.3748 | 3 | 14.33 | 6 | F6:4284 | 05112019\_RID\_1313\_NaNaRj\_F6.raw |  |  |  |  |  | 2.55E6 |  |  |  |  | 1 | 0 | 0 | 0 | 0 | 0 | 1 | 0 | 0 | 0 | 0 | 40 | 59 | Carbamidomethylation |
| K.ISLADGNDVR.I | Y | 63.10 | 1058.5356 | 10 | 0.6 | 530.2754 | 2 | 11.46 | 6 | F6:2282 | 05112019\_RID\_1313\_NaNaRj\_F6.raw |  |  |  |  |  | 9.94E5 | 3.63E4 |  |  |  | 2 | 0 | 0 | 0 | 0 | 0 | 1 | 1 | 0 | 0 | 0 | 27 | 36 |  |
| total 5 peptides |  |  |  |  |  |  |  |  |  |  |  |  |  |  |  |  |  |  |  |  |  |  |  |  |  |  |  |  |  |  |  |  |  |  |  |
| --- | --- | --- | --- | --- | --- | --- | --- | --- | --- | --- | --- | --- | --- | --- | --- | --- | --- | --- | --- | --- | --- | --- | --- | --- | --- | --- | --- | --- | --- | --- | --- | --- | --- | --- | --- |

Q7T1K6|CRVP1\_NAJAT

back to list

  

| Protein Coverage
| Supporting Peptides
|

Protein Coverage:

Supporting Peptides:

| Peptide | Uniq | -10lgP | Mass | Length | ppm | m/z | z | RT | Fraction | Scan | Source File | Area F1 | Area F2 | Area F3 | Area F4 | Area F5 | Area F6 | Area F7 | Area F8 | Area F9 | Area F10 | #Feature | #Feature F1 | #Feature F2 | #Feature F3 | #Feature F4 | #Feature F5 | #Feature F6 | #Feature F7 | #Feature F8 | #Feature F9 | #Feature F10 | Start | End | PTM |
| --- | --- | --- | --- | --- | --- | --- | --- | --- | --- | --- | --- | --- | --- | --- | --- | --- | --- | --- | --- | --- | --- | --- | --- | --- | --- | --- | --- | --- | --- | --- | --- | --- | --- | --- | --- |
| K.EIVDLHNSLR.R | Y | 66.57 | 1194.6356 | 10 | 0.8 | 598.3256 | 2 | 11.57 | 9 | F9:2262 | 05112019\_RID\_1313\_NaNaRj\_F9.raw |  |  |  |  |  |  |  |  | 1.65E5 |  | 2 | 0 | 0 | 0 | 0 | 0 | 0 | 0 | 0 | 2 | 0 | 35 | 44 |  |
| K.M(+15.99)EWYPEAASNAER.W | Y | 66.03 | 1568.6565 | 13 | 0.4 | 785.3358 | 2 | 11.73 | 10 | F10:2240 | 05112019\_RID\_1313\_NaNaRj\_F10.raw |  |  |  |  |  |  |  |  | 6.96E4 | 2.01E5 | 2 | 0 | 0 | 0 | 0 | 0 | 0 | 0 | 0 | 1 | 1 | 57 | 69 | Oxidation (M) |
| G.NVDFNSESTR.R | Y | 62.72 | 1167.5156 | 10 | 0.0 | 584.7651 | 2 | 11.12 | 10 | F10:1789 | 05112019\_RID\_1313\_NaNaRj\_F10.raw |  |  |  |  |  |  |  |  | 2.48E5 | 1.09E6 | 2 | 0 | 0 | 0 | 0 | 0 | 0 | 0 | 0 | 1 | 1 | 19 | 28 |  |
| K.SNC(+57.02)PASC(+57.02)FC(+57.02)R.N | Y | 60.53 | 1257.4689 | 10 | -0.2 | 629.7416 | 2 | 11.13 | 9 | F9:1860 | 05112019\_RID\_1313\_NaNaRj\_F9.raw |  |  |  |  | 1.34E5 |  |  |  | 1.63E5 | 1.15E6 | 3 | 0 | 0 | 0 | 0 | 1 | 0 | 0 | 0 | 1 | 1 | 226 | 235 | Carbamidomethylation |
| F.YVC(+57.02)QYC(+57.02)PSGNFQGK.T | Y | 59.85 | 1706.7181 | 14 | 0.8 | 854.3670 | 2 | 11.47 | 9 | F9:2165 | 05112019\_RID\_1313\_NaNaRj\_F9.raw |  |  |  |  |  |  |  |  | 5.51E4 | 2.09E5 | 2 | 0 | 0 | 0 | 0 | 0 | 0 | 0 | 0 | 1 | 1 | 161 | 174 | Carbamidomethylation |
| K.MEWYPEAASNAER.W | Y | 57.89 | 1552.6616 | 13 | -0.1 | 777.3380 | 2 | 12.14 | 9 | F9:2742 | 05112019\_RID\_1313\_NaNaRj\_F9.raw |  |  |  |  |  |  |  |  | 3.13E4 | 7.58E4 | 2 | 0 | 0 | 0 | 0 | 0 | 0 | 0 | 0 | 1 | 1 | 57 | 69 |  |
| R.VSPTASNMLK.M | Y | 57.35 | 1046.5430 | 10 | 0.8 | 524.2792 | 2 | 11.37 | 9 | F9:2080 | 05112019\_RID\_1313\_NaNaRj\_F9.raw |  |  |  |  |  |  |  |  | 4.07E4 | 2.11E5 | 2 | 0 | 0 | 0 | 0 | 0 | 0 | 0 | 0 | 1 | 1 | 47 | 56 |  |
| R.VLEGIQC(+57.02)GESIYM(+15.99)SSNAR.T | Y | 54.80 | 2028.9244 | 18 | 0.3 | 1015.4698 | 2 | 12.11 | 10 | F10:2440 | 05112019\_RID\_1313\_NaNaRj\_F10.raw |  |  |  |  |  |  |  |  | 1.49E5 | 4.26E4 | 3 | 0 | 0 | 0 | 0 | 0 | 0 | 0 | 0 | 2 | 1 | 85 | 102 | Carbamidomethylation; Oxidation (M) |
| N.C(+57.02)PASC(+57.02)FC(+57.02)R.N | N | 52.73 | 1056.3939 | 8 | 0.0 | 529.2042 | 2 | 10.99 | 10 | F10:1734 | 05112019\_RID\_1313\_NaNaRj\_F10.raw |  |  |  |  |  |  |  |  |  | 1.9E4 | 1 | 0 | 0 | 0 | 0 | 0 | 0 | 0 | 0 | 0 | 1 | 228 | 235 | Carbamidomethylation |
| R.VSPTASNM(+15.99)LK.M | Y | 51.38 | 1062.5380 | 10 | 0.6 | 532.2766 | 2 | 10.82 | 10 | F10:1670 | 05112019\_RID\_1313\_NaNaRj\_F10.raw |  |  |  |  |  |  |  |  |  | 2.66E3 | 1 | 0 | 0 | 0 | 0 | 0 | 0 | 0 | 0 | 0 | 1 | 47 | 56 | Oxidation (M) |
| C.QYC(+57.02)PSGNFQGK.T | Y | 49.03 | 1284.5557 | 11 | 0.6 | 643.2855 | 2 | 11.08 | 10 | F10:1773 | 05112019\_RID\_1313\_NaNaRj\_F10.raw |  |  |  |  |  |  |  |  | 2.5E4 | 6.2E4 | 2 | 0 | 0 | 0 | 0 | 0 | 0 | 0 | 0 | 1 | 1 | 164 | 174 | Carbamidomethylation |
| F.GNVDFNSESTR.R | Y | 48.57 | 1224.5371 | 11 | 0.1 | 613.2759 | 2 | 11.22 | 9 | F9:1949 | 05112019\_RID\_1313\_NaNaRj\_F9.raw |  |  |  |  |  |  |  |  | 1.84E4 |  | 1 | 0 | 0 | 0 | 0 | 0 | 0 | 0 | 0 | 1 | 0 | 18 | 28 |  |
| R.VLEGIQC(+57.02)GESIYMSSNAR.T | Y | 44.04 | 2012.9296 | 18 | -0.4 | 1007.4716 | 2 | 13.73 | 9 | F9:3186 | 05112019\_RID\_1313\_NaNaRj\_F9.raw |  |  |  |  |  |  |  |  | 8.43E3 |  | 1 | 0 | 0 | 0 | 0 | 0 | 0 | 0 | 0 | 1 | 0 | 85 | 102 | Carbamidomethylation |
| total 13 peptides |  |  |  |  |  |  |  |  |  |  |  |  |  |  |  |  |  |  |  |  |  |  |  |  |  |  |  |  |  |  |  |  |  |  |  |
| --- | --- | --- | --- | --- | --- | --- | --- | --- | --- | --- | --- | --- | --- | --- | --- | --- | --- | --- | --- | --- | --- | --- | --- | --- | --- | --- | --- | --- | --- | --- | --- | --- | --- | --- | --- |

P84805|CRVP1\_NAJKA

back to list

  

| Protein Coverage
| Supporting Peptides
|

Protein Coverage:

Supporting Peptides:

| Peptide | Uniq | -10lgP | Mass | Length | ppm | m/z | z | RT | Fraction | Scan | Source File | Area F1 | Area F2 | Area F3 | Area F4 | Area F5 | Area F6 | Area F7 | Area F8 | Area F9 | Area F10 | #Feature | #Feature F1 | #Feature F2 | #Feature F3 | #Feature F4 | #Feature F5 | #Feature F6 | #Feature F7 | #Feature F8 | #Feature F9 | #Feature F10 | Start | End | PTM |
| --- | --- | --- | --- | --- | --- | --- | --- | --- | --- | --- | --- | --- | --- | --- | --- | --- | --- | --- | --- | --- | --- | --- | --- | --- | --- | --- | --- | --- | --- | --- | --- | --- | --- | --- | --- |
| K.EIVDLHNSLR.R | Y | 66.57 | 1194.6356 | 10 | 0.8 | 598.3256 | 2 | 11.57 | 9 | F9:2262 | 05112019\_RID\_1313\_NaNaRj\_F9.raw |  |  |  |  |  |  |  |  | 1.65E5 |  | 2 | 0 | 0 | 0 | 0 | 0 | 0 | 0 | 0 | 2 | 0 | 35 | 44 |  |
| K.M(+15.99)EWYPEAASNAER.W | Y | 66.03 | 1568.6565 | 13 | 0.4 | 785.3358 | 2 | 11.73 | 10 | F10:2240 | 05112019\_RID\_1313\_NaNaRj\_F10.raw |  |  |  |  |  |  |  |  | 6.96E4 | 2.01E5 | 2 | 0 | 0 | 0 | 0 | 0 | 0 | 0 | 0 | 1 | 1 | 57 | 69 | Oxidation (M) |
| G.NVDFNSESTR.R | Y | 62.72 | 1167.5156 | 10 | 0.0 | 584.7651 | 2 | 11.12 | 10 | F10:1789 | 05112019\_RID\_1313\_NaNaRj\_F10.raw |  |  |  |  |  |  |  |  | 2.48E5 | 1.09E6 | 2 | 0 | 0 | 0 | 0 | 0 | 0 | 0 | 0 | 1 | 1 | 19 | 28 |  |
| K.SNC(+57.02)PASC(+57.02)FC(+57.02)R.N | Y | 60.53 | 1257.4689 | 10 | -0.2 | 629.7416 | 2 | 11.13 | 9 | F9:1860 | 05112019\_RID\_1313\_NaNaRj\_F9.raw |  |  |  |  | 1.34E5 |  |  |  | 1.63E5 | 1.15E6 | 3 | 0 | 0 | 0 | 0 | 1 | 0 | 0 | 0 | 1 | 1 | 226 | 235 | Carbamidomethylation |
| F.YVC(+57.02)QYC(+57.02)PSGNFQGK.T | Y | 59.85 | 1706.7181 | 14 | 0.8 | 854.3670 | 2 | 11.47 | 9 | F9:2165 | 05112019\_RID\_1313\_NaNaRj\_F9.raw |  |  |  |  |  |  |  |  | 5.51E4 | 2.09E5 | 2 | 0 | 0 | 0 | 0 | 0 | 0 | 0 | 0 | 1 | 1 | 161 | 174 | Carbamidomethylation |
| K.MEWYPEAASNAER.W | Y | 57.89 | 1552.6616 | 13 | -0.1 | 777.3380 | 2 | 12.14 | 9 | F9:2742 | 05112019\_RID\_1313\_NaNaRj\_F9.raw |  |  |  |  |  |  |  |  | 3.13E4 | 7.58E4 | 2 | 0 | 0 | 0 | 0 | 0 | 0 | 0 | 0 | 1 | 1 | 57 | 69 |  |
| R.VSPTASNMLK.M | Y | 57.35 | 1046.5430 | 10 | 0.8 | 524.2792 | 2 | 11.37 | 9 | F9:2080 | 05112019\_RID\_1313\_NaNaRj\_F9.raw |  |  |  |  |  |  |  |  | 4.07E4 | 2.11E5 | 2 | 0 | 0 | 0 | 0 | 0 | 0 | 0 | 0 | 1 | 1 | 47 | 56 |  |
| R.VLEGIQC(+57.02)GESIYM(+15.99)SSNAR.T | Y | 54.80 | 2028.9244 | 18 | 0.3 | 1015.4698 | 2 | 12.11 | 10 | F10:2440 | 05112019\_RID\_1313\_NaNaRj\_F10.raw |  |  |  |  |  |  |  |  | 1.49E5 | 4.26E4 | 3 | 0 | 0 | 0 | 0 | 0 | 0 | 0 | 0 | 2 | 1 | 85 | 102 | Carbamidomethylation; Oxidation (M) |
| N.C(+57.02)PASC(+57.02)FC(+57.02)R.N | N | 52.73 | 1056.3939 | 8 | 0.0 | 529.2042 | 2 | 10.99 | 10 | F10:1734 | 05112019\_RID\_1313\_NaNaRj\_F10.raw |  |  |  |  |  |  |  |  |  | 1.9E4 | 1 | 0 | 0 | 0 | 0 | 0 | 0 | 0 | 0 | 0 | 1 | 228 | 235 | Carbamidomethylation |
| R.VSPTASNM(+15.99)LK.M | Y | 51.38 | 1062.5380 | 10 | 0.6 | 532.2766 | 2 | 10.82 | 10 | F10:1670 | 05112019\_RID\_1313\_NaNaRj\_F10.raw |  |  |  |  |  |  |  |  |  | 2.66E3 | 1 | 0 | 0 | 0 | 0 | 0 | 0 | 0 | 0 | 0 | 1 | 47 | 56 | Oxidation (M) |
| C.QYC(+57.02)PSGNFQGK.T | Y | 49.03 | 1284.5557 | 11 | 0.6 | 643.2855 | 2 | 11.08 | 10 | F10:1773 | 05112019\_RID\_1313\_NaNaRj\_F10.raw |  |  |  |  |  |  |  |  | 2.5E4 | 6.2E4 | 2 | 0 | 0 | 0 | 0 | 0 | 0 | 0 | 0 | 1 | 1 | 164 | 174 | Carbamidomethylation |
| F.GNVDFNSESTR.R | Y | 48.57 | 1224.5371 | 11 | 0.1 | 613.2759 | 2 | 11.22 | 9 | F9:1949 | 05112019\_RID\_1313\_NaNaRj\_F9.raw |  |  |  |  |  |  |  |  | 1.84E4 |  | 1 | 0 | 0 | 0 | 0 | 0 | 0 | 0 | 0 | 1 | 0 | 18 | 28 |  |
| R.VLEGIQC(+57.02)GESIYMSSNAR.T | Y | 44.04 | 2012.9296 | 18 | -0.4 | 1007.4716 | 2 | 13.73 | 9 | F9:3186 | 05112019\_RID\_1313\_NaNaRj\_F9.raw |  |  |  |  |  |  |  |  | 8.43E3 |  | 1 | 0 | 0 | 0 | 0 | 0 | 0 | 0 | 0 | 1 | 0 | 85 | 102 | Carbamidomethylation |
| total 13 peptides |  |  |  |  |  |  |  |  |  |  |  |  |  |  |  |  |  |  |  |  |  |  |  |  |  |  |  |  |  |  |  |  |  |  |  |
| --- | --- | --- | --- | --- | --- | --- | --- | --- | --- | --- | --- | --- | --- | --- | --- | --- | --- | --- | --- | --- | --- | --- | --- | --- | --- | --- | --- | --- | --- | --- | --- | --- | --- | --- | --- |

P10117|PA2H1\_LATCO

back to list

  

| Protein Coverage
| Supporting Peptides
|

Protein Coverage:

Supporting Peptides:

| Peptide | Uniq | -10lgP | Mass | Length | ppm | m/z | z | RT | Fraction | Scan | Source File | Area F1 | Area F2 | Area F3 | Area F4 | Area F5 | Area F6 | Area F7 | Area F8 | Area F9 | Area F10 | #Feature | #Feature F1 | #Feature F2 | #Feature F3 | #Feature F4 | #Feature F5 | #Feature F6 | #Feature F7 | #Feature F8 | #Feature F9 | #Feature F10 | Start | End | PTM |
| --- | --- | --- | --- | --- | --- | --- | --- | --- | --- | --- | --- | --- | --- | --- | --- | --- | --- | --- | --- | --- | --- | --- | --- | --- | --- | --- | --- | --- | --- | --- | --- | --- | --- | --- | --- |
| D.YGC(+57.02)YC(+57.02)GPGGSGTPVDDLDR.C | Y | 90.80 | 2044.8254 | 19 | 0.6 | 1023.4207 | 2 | 11.92 | 8 | F8:2719 | 05112019\_RID\_1313\_NaNaRj\_F8.raw |  |  |  |  |  |  |  | 2.06E5 |  |  | 1 | 0 | 0 | 0 | 0 | 0 | 0 | 0 | 1 | 0 | 0 | 25 | 43 | Carbamidomethylation |
| Y.C(+57.02)GPGGSGTPVDDLDR.C | Y | 86.81 | 1501.6467 | 15 | -1.7 | 751.8293 | 2 | 11.31 | 8 | F8:2167 | 05112019\_RID\_1313\_NaNaRj\_F8.raw |  |  |  |  |  |  | 1.62E4 | 4.17E4 | 2.69E3 |  | 3 | 0 | 0 | 0 | 0 | 0 | 0 | 1 | 1 | 1 | 0 | 29 | 43 | Carbamidomethylation |
| P.GGSGTPVDDLDR.C | N | 79.15 | 1187.5417 | 12 | -1.8 | 594.7771 | 2 | 11.41 | 6 | F6:2201 | 05112019\_RID\_1313\_NaNaRj\_F6.raw |  |  |  |  |  | 5.21E7 |  |  | 4.03E4 |  | 4 | 0 | 0 | 0 | 0 | 0 | 3 | 0 | 0 | 1 | 0 | 32 | 43 |  |
| M.DYGC(+57.02)YC(+57.02)GPGGSGTPVDDLDR.C | Y | 60.29 | 2159.8523 | 20 | 0.9 | 1080.9344 | 2 | 12.66 | 6 | F6:3224 | 05112019\_RID\_1313\_NaNaRj\_F6.raw |  |  |  |  |  | 2.92E5 |  | 2.26E4 |  |  | 2 | 0 | 0 | 0 | 0 | 0 | 1 | 0 | 1 | 0 | 0 | 24 | 43 | Carbamidomethylation |
| Y.GC(+57.02)YC(+57.02)GPGGSGTPVDDLDR.C | Y | 60.29 | 1881.7621 | 18 | 0.4 | 941.8887 | 2 | 11.75 | 7 | F7:2573 | 05112019\_RID\_1313\_NaNaRj\_F7.raw |  |  |  |  |  |  | 1.24E4 |  |  |  | 1 | 0 | 0 | 0 | 0 | 0 | 0 | 1 | 0 | 0 | 0 | 26 | 43 | Carbamidomethylation |
| C.YC(+57.02)GPGGSGTPVDDLDR.C | Y | 48.57 | 1664.7100 | 16 | -3.6 | 833.3593 | 2 | 11.71 | 8 | F8:2541 | 05112019\_RID\_1313\_NaNaRj\_F8.raw |  |  |  |  |  |  |  | 3.21E5 |  |  | 1 | 0 | 0 | 0 | 0 | 0 | 0 | 0 | 1 | 0 | 0 | 28 | 43 | Carbamidomethylation |
| total 6 peptides |  |  |  |  |  |  |  |  |  |  |  |  |  |  |  |  |  |  |  |  |  |  |  |  |  |  |  |  |  |  |  |  |  |  |  |
| --- | --- | --- | --- | --- | --- | --- | --- | --- | --- | --- | --- | --- | --- | --- | --- | --- | --- | --- | --- | --- | --- | --- | --- | --- | --- | --- | --- | --- | --- | --- | --- | --- | --- | --- | --- |

P01946|HBA\_RAT

back to list

  

| Protein Coverage
| Supporting Peptides
|

Protein Coverage:

Supporting Peptides:

| Peptide | Uniq | -10lgP | Mass | Length | ppm | m/z | z | RT | Fraction | Scan | Source File | Area F1 | Area F2 | Area F3 | Area F4 | Area F5 | Area F6 | Area F7 | Area F8 | Area F9 | Area F10 | #Feature | #Feature F1 | #Feature F2 | #Feature F3 | #Feature F4 | #Feature F5 | #Feature F6 | #Feature F7 | #Feature F8 | #Feature F9 | #Feature F10 | Start | End | PTM |
| --- | --- | --- | --- | --- | --- | --- | --- | --- | --- | --- | --- | --- | --- | --- | --- | --- | --- | --- | --- | --- | --- | --- | --- | --- | --- | --- | --- | --- | --- | --- | --- | --- | --- | --- | --- |
| K.AADHVEDLPGALSTLSDLHAHK.L | Y | 96.68 | 2296.1448 | 22 | -0.2 | 460.2361 | 5 | 12.26 | 1 | F1:2884 | 05112019\_RID\_1313\_NaNaRj\_F1.raw | 2.82E5 |  | 4.19E4 |  |  |  |  |  | 2.02E4 |  | 6 | 3 | 0 | 2 | 0 | 0 | 0 | 0 | 0 | 1 | 0 | 70 | 91 |  |
| K.TYFSHIDVSPGSAQVK.A | Y | 91.86 | 1734.8577 | 16 | 0.3 | 868.4364 | 2 | 11.77 | 1 | F1:2521 | 05112019\_RID\_1313\_NaNaRj\_F1.raw | 6.7E5 |  | 1.11E4 |  |  |  |  |  | 7.67E4 | 8.58E4 | 6 | 2 | 0 | 1 | 0 | 0 | 0 | 0 | 0 | 1 | 2 | 42 | 57 |  |
| K.IGGHGGEYGEEALQR.M | Y | 59.91 | 1571.7328 | 15 | -0.2 | 524.9181 | 3 | 11.14 | 1 | F1:1946 | 05112019\_RID\_1313\_NaNaRj\_F1.raw | 4.45E4 |  |  |  |  |  |  |  | 6.22E3 |  | 2 | 1 | 0 | 0 | 0 | 0 | 0 | 0 | 0 | 1 | 0 | 18 | 32 |  |
| K.FLASVSTVLTSK.Y | Y | 52.67 | 1251.7074 | 12 | 0.6 | 626.8613 | 2 | 13.01 | 1 | F1:3571 | 05112019\_RID\_1313\_NaNaRj\_F1.raw | 6.84E5 |  |  |  |  |  |  |  | 4.08E4 |  | 5 | 4 | 0 | 0 | 0 | 0 | 0 | 0 | 0 | 1 | 0 | 129 | 140 |  |
| R.VDPVNFK.F | Y | 49.13 | 817.4333 | 7 | 0.5 | 409.7242 | 2 | 11.56 | 1 | F1:2331 | 05112019\_RID\_1313\_NaNaRj\_F1.raw | 3.79E3 |  |  |  |  |  |  |  |  |  | 1 | 1 | 0 | 0 | 0 | 0 | 0 | 0 | 0 | 0 | 0 | 94 | 100 |  |
| M.FAAFPTTK.T | Y | 43.69 | 881.4647 | 8 | 0.6 | 441.7399 | 2 | 11.78 | 1 | F1:2550 | 05112019\_RID\_1313\_NaNaRj\_F1.raw | 5.09E4 |  | 8.16E3 |  |  |  |  |  |  |  | 2 | 1 | 0 | 1 | 0 | 0 | 0 | 0 | 0 | 0 | 0 | 34 | 41 |  |
| total 6 peptides |  |  |  |  |  |  |  |  |  |  |  |  |  |  |  |  |  |  |  |  |  |  |  |  |  |  |  |  |  |  |  |  |  |  |  |
| --- | --- | --- | --- | --- | --- | --- | --- | --- | --- | --- | --- | --- | --- | --- | --- | --- | --- | --- | --- | --- | --- | --- | --- | --- | --- | --- | --- | --- | --- | --- | --- | --- | --- | --- | --- |

P62377|3SOFL\_NAJNA

back to list

  

| Protein Coverage
| Supporting Peptides
|

Protein Coverage:

Supporting Peptides:

| Peptide | Uniq | -10lgP | Mass | Length | ppm | m/z | z | RT | Fraction | Scan | Source File | Area F1 | Area F2 | Area F3 | Area F4 | Area F5 | Area F6 | Area F7 | Area F8 | Area F9 | Area F10 | #Feature | #Feature F1 | #Feature F2 | #Feature F3 | #Feature F4 | #Feature F5 | #Feature F6 | #Feature F7 | #Feature F8 | #Feature F9 | #Feature F10 | Start | End | PTM |
| --- | --- | --- | --- | --- | --- | --- | --- | --- | --- | --- | --- | --- | --- | --- | --- | --- | --- | --- | --- | --- | --- | --- | --- | --- | --- | --- | --- | --- | --- | --- | --- | --- | --- | --- | --- |
| K.C(+57.02)HNTQLPFIYK.T | Y | 101.99 | 1419.6969 | 11 | 0.2 | 710.8558 | 2 | 11.87 | 7 | F7:2661 | 05112019\_RID\_1313\_NaNaRj\_F7.raw |  |  |  |  |  | 2.9E6 | 3.61E7 | 4.4E5 |  |  | 6 | 0 | 0 | 0 | 0 | 0 | 2 | 3 | 1 | 0 | 0 | 3 | 13 | Carbamidomethylation |
| K.FPLKFPVK.R | Y | 56.32 | 974.5953 | 8 | -0.8 | 488.3045 | 2 | 12.12 | 7 | F7:2867 | 05112019\_RID\_1313\_NaNaRj\_F7.raw |  |  |  |  |  |  | 2.53E5 |  |  |  | 1 | 0 | 0 | 0 | 0 | 0 | 0 | 1 | 0 | 0 | 0 | 30 | 37 |  |
| K.FPLKFPVKR.G | Y | 55.22 | 1130.6964 | 9 | -0.6 | 377.9059 | 3 | 11.56 | 7 | F7:2388 | 05112019\_RID\_1313\_NaNaRj\_F7.raw |  |  |  |  |  |  | 3.16E5 |  |  |  | 1 | 0 | 0 | 0 | 0 | 0 | 0 | 1 | 0 | 0 | 0 | 30 | 38 |  |
| LKC(+57.02)HNTQLPFIYK.T | Y | 51.51 | 1660.8759 | 13 | -0.3 | 416.2261 | 4 | 11.56 | 7 | F7:2390 | 05112019\_RID\_1313\_NaNaRj\_F7.raw |  |  |  |  |  |  | 2.66E5 |  |  |  | 1 | 0 | 0 | 0 | 0 | 0 | 0 | 1 | 0 | 0 | 0 | 1 | 13 | Carbamidomethylation |
| K.C(+57.02)HNTQLPF.I | Y | 43.53 | 1015.4545 | 8 | 1.7 | 508.7354 | 2 | 12.11 | 8 | F8:2864 | 05112019\_RID\_1313\_NaNaRj\_F8.raw |  |  |  |  |  |  |  | 3.03E6 |  |  | 1 | 0 | 0 | 0 | 0 | 0 | 0 | 0 | 1 | 0 | 0 | 3 | 10 | Carbamidomethylation |
| total 5 peptides |  |  |  |  |  |  |  |  |  |  |  |  |  |  |  |  |  |  |  |  |  |  |  |  |  |  |  |  |  |  |  |  |  |  |  |
| --- | --- | --- | --- | --- | --- | --- | --- | --- | --- | --- | --- | --- | --- | --- | --- | --- | --- | --- | --- | --- | --- | --- | --- | --- | --- | --- | --- | --- | --- | --- | --- | --- | --- | --- | --- |

Q9W716|3SOFV\_NAJAT

back to list

  

| Protein Coverage
| Supporting Peptides
|

Protein Coverage:

Supporting Peptides:

| Peptide | Uniq | -10lgP | Mass | Length | ppm | m/z | z | RT | Fraction | Scan | Source File | Area F1 | Area F2 | Area F3 | Area F4 | Area F5 | Area F6 | Area F7 | Area F8 | Area F9 | Area F10 | #Feature | #Feature F1 | #Feature F2 | #Feature F3 | #Feature F4 | #Feature F5 | #Feature F6 | #Feature F7 | #Feature F8 | #Feature F9 | #Feature F10 | Start | End | PTM |
| --- | --- | --- | --- | --- | --- | --- | --- | --- | --- | --- | --- | --- | --- | --- | --- | --- | --- | --- | --- | --- | --- | --- | --- | --- | --- | --- | --- | --- | --- | --- | --- | --- | --- | --- | --- |
| K.C(+57.02)HNTQLPFIYK.T | Y | 101.99 | 1419.6969 | 11 | 0.2 | 710.8558 | 2 | 11.87 | 7 | F7:2661 | 05112019\_RID\_1313\_NaNaRj\_F7.raw |  |  |  |  |  | 2.9E6 | 3.61E7 | 4.4E5 |  |  | 6 | 0 | 0 | 0 | 0 | 0 | 2 | 3 | 1 | 0 | 0 | 24 | 34 | Carbamidomethylation |
| K.FPLKFPVK.R | Y | 56.32 | 974.5953 | 8 | -0.8 | 488.3045 | 2 | 12.12 | 7 | F7:2867 | 05112019\_RID\_1313\_NaNaRj\_F7.raw |  |  |  |  |  |  | 2.53E5 |  |  |  | 1 | 0 | 0 | 0 | 0 | 0 | 0 | 1 | 0 | 0 | 0 | 51 | 58 |  |
| K.FPLKFPVKR.G | Y | 55.22 | 1130.6964 | 9 | -0.6 | 377.9059 | 3 | 11.56 | 7 | F7:2388 | 05112019\_RID\_1313\_NaNaRj\_F7.raw |  |  |  |  |  |  | 3.16E5 |  |  |  | 1 | 0 | 0 | 0 | 0 | 0 | 0 | 1 | 0 | 0 | 0 | 51 | 59 |  |
| T.LKC(+57.02)HNTQLPFIYK.T | Y | 51.51 | 1660.8759 | 13 | -0.3 | 416.2261 | 4 | 11.56 | 7 | F7:2390 | 05112019\_RID\_1313\_NaNaRj\_F7.raw |  |  |  |  |  |  | 2.66E5 |  |  |  | 1 | 0 | 0 | 0 | 0 | 0 | 0 | 1 | 0 | 0 | 0 | 22 | 34 | Carbamidomethylation |
| K.C(+57.02)HNTQLPF.I | Y | 43.53 | 1015.4545 | 8 | 1.7 | 508.7354 | 2 | 12.11 | 8 | F8:2864 | 05112019\_RID\_1313\_NaNaRj\_F8.raw |  |  |  |  |  |  |  | 3.03E6 |  |  | 1 | 0 | 0 | 0 | 0 | 0 | 0 | 0 | 1 | 0 | 0 | 24 | 31 | Carbamidomethylation |
| total 5 peptides |  |  |  |  |  |  |  |  |  |  |  |  |  |  |  |  |  |  |  |  |  |  |  |  |  |  |  |  |  |  |  |  |  |  |  |
| --- | --- | --- | --- | --- | --- | --- | --- | --- | --- | --- | --- | --- | --- | --- | --- | --- | --- | --- | --- | --- | --- | --- | --- | --- | --- | --- | --- | --- | --- | --- | --- | --- | --- | --- | --- |

P62375|3SOF5\_NAJAT

back to list

  

| Protein Coverage
| Supporting Peptides
|

Protein Coverage:

Supporting Peptides:

| Peptide | Uniq | -10lgP | Mass | Length | ppm | m/z | z | RT | Fraction | Scan | Source File | Area F1 | Area F2 | Area F3 | Area F4 | Area F5 | Area F6 | Area F7 | Area F8 | Area F9 | Area F10 | #Feature | #Feature F1 | #Feature F2 | #Feature F3 | #Feature F4 | #Feature F5 | #Feature F6 | #Feature F7 | #Feature F8 | #Feature F9 | #Feature F10 | Start | End | PTM |
| --- | --- | --- | --- | --- | --- | --- | --- | --- | --- | --- | --- | --- | --- | --- | --- | --- | --- | --- | --- | --- | --- | --- | --- | --- | --- | --- | --- | --- | --- | --- | --- | --- | --- | --- | --- |
| K.C(+57.02)HNTQLPFIYK.T | Y | 101.99 | 1419.6969 | 11 | 0.2 | 710.8558 | 2 | 11.87 | 7 | F7:2661 | 05112019\_RID\_1313\_NaNaRj\_F7.raw |  |  |  |  |  | 2.9E6 | 3.61E7 | 4.4E5 |  |  | 6 | 0 | 0 | 0 | 0 | 0 | 2 | 3 | 1 | 0 | 0 | 24 | 34 | Carbamidomethylation |
| K.FPLKFPVK.R | Y | 56.32 | 974.5953 | 8 | -0.8 | 488.3045 | 2 | 12.12 | 7 | F7:2867 | 05112019\_RID\_1313\_NaNaRj\_F7.raw |  |  |  |  |  |  | 2.53E5 |  |  |  | 1 | 0 | 0 | 0 | 0 | 0 | 0 | 1 | 0 | 0 | 0 | 51 | 58 |  |
| K.FPLKFPVKR.G | Y | 55.22 | 1130.6964 | 9 | -0.6 | 377.9059 | 3 | 11.56 | 7 | F7:2388 | 05112019\_RID\_1313\_NaNaRj\_F7.raw |  |  |  |  |  |  | 3.16E5 |  |  |  | 1 | 0 | 0 | 0 | 0 | 0 | 0 | 1 | 0 | 0 | 0 | 51 | 59 |  |
| T.LKC(+57.02)HNTQLPFIYK.T | Y | 51.51 | 1660.8759 | 13 | -0.3 | 416.2261 | 4 | 11.56 | 7 | F7:2390 | 05112019\_RID\_1313\_NaNaRj\_F7.raw |  |  |  |  |  |  | 2.66E5 |  |  |  | 1 | 0 | 0 | 0 | 0 | 0 | 0 | 1 | 0 | 0 | 0 | 22 | 34 | Carbamidomethylation |
| K.C(+57.02)HNTQLPF.I | Y | 43.53 | 1015.4545 | 8 | 1.7 | 508.7354 | 2 | 12.11 | 8 | F8:2864 | 05112019\_RID\_1313\_NaNaRj\_F8.raw |  |  |  |  |  |  |  | 3.03E6 |  |  | 1 | 0 | 0 | 0 | 0 | 0 | 0 | 0 | 1 | 0 | 0 | 24 | 31 | Carbamidomethylation |
| total 5 peptides |  |  |  |  |  |  |  |  |  |  |  |  |  |  |  |  |  |  |  |  |  |  |  |  |  |  |  |  |  |  |  |  |  |  |  |
| --- | --- | --- | --- | --- | --- | --- | --- | --- | --- | --- | --- | --- | --- | --- | --- | --- | --- | --- | --- | --- | --- | --- | --- | --- | --- | --- | --- | --- | --- | --- | --- | --- | --- | --- | --- |

Q91126|3SOFA\_NAJAT

back to list

  

| Protein Coverage
| Supporting Peptides
|

Protein Coverage:

Supporting Peptides:

| Peptide | Uniq | -10lgP | Mass | Length | ppm | m/z | z | RT | Fraction | Scan | Source File | Area F1 | Area F2 | Area F3 | Area F4 | Area F5 | Area F6 | Area F7 | Area F8 | Area F9 | Area F10 | #Feature | #Feature F1 | #Feature F2 | #Feature F3 | #Feature F4 | #Feature F5 | #Feature F6 | #Feature F7 | #Feature F8 | #Feature F9 | #Feature F10 | Start | End | PTM |
| --- | --- | --- | --- | --- | --- | --- | --- | --- | --- | --- | --- | --- | --- | --- | --- | --- | --- | --- | --- | --- | --- | --- | --- | --- | --- | --- | --- | --- | --- | --- | --- | --- | --- | --- | --- |
| K.C(+57.02)HNTQLPFIYK.T | Y | 101.99 | 1419.6969 | 11 | 0.2 | 710.8558 | 2 | 11.87 | 7 | F7:2661 | 05112019\_RID\_1313\_NaNaRj\_F7.raw |  |  |  |  |  | 2.9E6 | 3.61E7 | 4.4E5 |  |  | 6 | 0 | 0 | 0 | 0 | 0 | 2 | 3 | 1 | 0 | 0 | 24 | 34 | Carbamidomethylation |
| K.FPLKFPVK.R | Y | 56.32 | 974.5953 | 8 | -0.8 | 488.3045 | 2 | 12.12 | 7 | F7:2867 | 05112019\_RID\_1313\_NaNaRj\_F7.raw |  |  |  |  |  |  | 2.53E5 |  |  |  | 1 | 0 | 0 | 0 | 0 | 0 | 0 | 1 | 0 | 0 | 0 | 51 | 58 |  |
| K.FPLKFPVKR.G | Y | 55.22 | 1130.6964 | 9 | -0.6 | 377.9059 | 3 | 11.56 | 7 | F7:2388 | 05112019\_RID\_1313\_NaNaRj\_F7.raw |  |  |  |  |  |  | 3.16E5 |  |  |  | 1 | 0 | 0 | 0 | 0 | 0 | 0 | 1 | 0 | 0 | 0 | 51 | 59 |  |
| T.LKC(+57.02)HNTQLPFIYK.T | Y | 51.51 | 1660.8759 | 13 | -0.3 | 416.2261 | 4 | 11.56 | 7 | F7:2390 | 05112019\_RID\_1313\_NaNaRj\_F7.raw |  |  |  |  |  |  | 2.66E5 |  |  |  | 1 | 0 | 0 | 0 | 0 | 0 | 0 | 1 | 0 | 0 | 0 | 22 | 34 | Carbamidomethylation |
| K.C(+57.02)HNTQLPF.I | Y | 43.53 | 1015.4545 | 8 | 1.7 | 508.7354 | 2 | 12.11 | 8 | F8:2864 | 05112019\_RID\_1313\_NaNaRj\_F8.raw |  |  |  |  |  |  |  | 3.03E6 |  |  | 1 | 0 | 0 | 0 | 0 | 0 | 0 | 0 | 1 | 0 | 0 | 24 | 31 | Carbamidomethylation |
| total 5 peptides |  |  |  |  |  |  |  |  |  |  |  |  |  |  |  |  |  |  |  |  |  |  |  |  |  |  |  |  |  |  |  |  |  |  |  |
| --- | --- | --- | --- | --- | --- | --- | --- | --- | --- | --- | --- | --- | --- | --- | --- | --- | --- | --- | --- | --- | --- | --- | --- | --- | --- | --- | --- | --- | --- | --- | --- | --- | --- | --- | --- |

Q91137|3SOFL\_NAJAT

back to list

  

| Protein Coverage
| Supporting Peptides
|

Protein Coverage:

Supporting Peptides:

| Peptide | Uniq | -10lgP | Mass | Length | ppm | m/z | z | RT | Fraction | Scan | Source File | Area F1 | Area F2 | Area F3 | Area F4 | Area F5 | Area F6 | Area F7 | Area F8 | Area F9 | Area F10 | #Feature | #Feature F1 | #Feature F2 | #Feature F3 | #Feature F4 | #Feature F5 | #Feature F6 | #Feature F7 | #Feature F8 | #Feature F9 | #Feature F10 | Start | End | PTM |
| --- | --- | --- | --- | --- | --- | --- | --- | --- | --- | --- | --- | --- | --- | --- | --- | --- | --- | --- | --- | --- | --- | --- | --- | --- | --- | --- | --- | --- | --- | --- | --- | --- | --- | --- | --- |
| K.C(+57.02)HNTQLPFIYK.T | Y | 101.99 | 1419.6969 | 11 | 0.2 | 710.8558 | 2 | 11.87 | 7 | F7:2661 | 05112019\_RID\_1313\_NaNaRj\_F7.raw |  |  |  |  |  | 2.9E6 | 3.61E7 | 4.4E5 |  |  | 6 | 0 | 0 | 0 | 0 | 0 | 2 | 3 | 1 | 0 | 0 | 24 | 34 | Carbamidomethylation |
| K.FPLKFPVK.R | Y | 56.32 | 974.5953 | 8 | -0.8 | 488.3045 | 2 | 12.12 | 7 | F7:2867 | 05112019\_RID\_1313\_NaNaRj\_F7.raw |  |  |  |  |  |  | 2.53E5 |  |  |  | 1 | 0 | 0 | 0 | 0 | 0 | 0 | 1 | 0 | 0 | 0 | 51 | 58 |  |
| K.FPLKFPVKR.G | Y | 55.22 | 1130.6964 | 9 | -0.6 | 377.9059 | 3 | 11.56 | 7 | F7:2388 | 05112019\_RID\_1313\_NaNaRj\_F7.raw |  |  |  |  |  |  | 3.16E5 |  |  |  | 1 | 0 | 0 | 0 | 0 | 0 | 0 | 1 | 0 | 0 | 0 | 51 | 59 |  |
| T.LKC(+57.02)HNTQLPFIYK.T | Y | 51.51 | 1660.8759 | 13 | -0.3 | 416.2261 | 4 | 11.56 | 7 | F7:2390 | 05112019\_RID\_1313\_NaNaRj\_F7.raw |  |  |  |  |  |  | 2.66E5 |  |  |  | 1 | 0 | 0 | 0 | 0 | 0 | 0 | 1 | 0 | 0 | 0 | 22 | 34 | Carbamidomethylation |
| K.C(+57.02)HNTQLPF.I | Y | 43.53 | 1015.4545 | 8 | 1.7 | 508.7354 | 2 | 12.11 | 8 | F8:2864 | 05112019\_RID\_1313\_NaNaRj\_F8.raw |  |  |  |  |  |  |  | 3.03E6 |  |  | 1 | 0 | 0 | 0 | 0 | 0 | 0 | 0 | 1 | 0 | 0 | 24 | 31 | Carbamidomethylation |
| total 5 peptides |  |  |  |  |  |  |  |  |  |  |  |  |  |  |  |  |  |  |  |  |  |  |  |  |  |  |  |  |  |  |  |  |  |  |  |
| --- | --- | --- | --- | --- | --- | --- | --- | --- | --- | --- | --- | --- | --- | --- | --- | --- | --- | --- | --- | --- | --- | --- | --- | --- | --- | --- | --- | --- | --- | --- | --- | --- | --- | --- | --- |

Q91996|3SOFP\_NAJAT

back to list

  

| Protein Coverage
| Supporting Peptides
|

Protein Coverage:

Supporting Peptides:

| Peptide | Uniq | -10lgP | Mass | Length | ppm | m/z | z | RT | Fraction | Scan | Source File | Area F1 | Area F2 | Area F3 | Area F4 | Area F5 | Area F6 | Area F7 | Area F8 | Area F9 | Area F10 | #Feature | #Feature F1 | #Feature F2 | #Feature F3 | #Feature F4 | #Feature F5 | #Feature F6 | #Feature F7 | #Feature F8 | #Feature F9 | #Feature F10 | Start | End | PTM |
| --- | --- | --- | --- | --- | --- | --- | --- | --- | --- | --- | --- | --- | --- | --- | --- | --- | --- | --- | --- | --- | --- | --- | --- | --- | --- | --- | --- | --- | --- | --- | --- | --- | --- | --- | --- |
| K.C(+57.02)HNTQLPFIYK.T | Y | 101.99 | 1419.6969 | 11 | 0.2 | 710.8558 | 2 | 11.87 | 7 | F7:2661 | 05112019\_RID\_1313\_NaNaRj\_F7.raw |  |  |  |  |  | 2.9E6 | 3.61E7 | 4.4E5 |  |  | 6 | 0 | 0 | 0 | 0 | 0 | 2 | 3 | 1 | 0 | 0 | 24 | 34 | Carbamidomethylation |
| K.FPLKFPVK.R | Y | 56.32 | 974.5953 | 8 | -0.8 | 488.3045 | 2 | 12.12 | 7 | F7:2867 | 05112019\_RID\_1313\_NaNaRj\_F7.raw |  |  |  |  |  |  | 2.53E5 |  |  |  | 1 | 0 | 0 | 0 | 0 | 0 | 0 | 1 | 0 | 0 | 0 | 51 | 58 |  |
| K.FPLKFPVKR.G | Y | 55.22 | 1130.6964 | 9 | -0.6 | 377.9059 | 3 | 11.56 | 7 | F7:2388 | 05112019\_RID\_1313\_NaNaRj\_F7.raw |  |  |  |  |  |  | 3.16E5 |  |  |  | 1 | 0 | 0 | 0 | 0 | 0 | 0 | 1 | 0 | 0 | 0 | 51 | 59 |  |
| T.LKC(+57.02)HNTQLPFIYK.T | Y | 51.51 | 1660.8759 | 13 | -0.3 | 416.2261 | 4 | 11.56 | 7 | F7:2390 | 05112019\_RID\_1313\_NaNaRj\_F7.raw |  |  |  |  |  |  | 2.66E5 |  |  |  | 1 | 0 | 0 | 0 | 0 | 0 | 0 | 1 | 0 | 0 | 0 | 22 | 34 | Carbamidomethylation |
| K.C(+57.02)HNTQLPF.I | Y | 43.53 | 1015.4545 | 8 | 1.7 | 508.7354 | 2 | 12.11 | 8 | F8:2864 | 05112019\_RID\_1313\_NaNaRj\_F8.raw |  |  |  |  |  |  |  | 3.03E6 |  |  | 1 | 0 | 0 | 0 | 0 | 0 | 0 | 0 | 1 | 0 | 0 | 24 | 31 | Carbamidomethylation |
| total 5 peptides |  |  |  |  |  |  |  |  |  |  |  |  |  |  |  |  |  |  |  |  |  |  |  |  |  |  |  |  |  |  |  |  |  |  |  |
| --- | --- | --- | --- | --- | --- | --- | --- | --- | --- | --- | --- | --- | --- | --- | --- | --- | --- | --- | --- | --- | --- | --- | --- | --- | --- | --- | --- | --- | --- | --- | --- | --- | --- | --- | --- |

O43790|KRT86\_HUMAN

back to list

  

| Protein Coverage
| Supporting Peptides
|

Protein Coverage:

Supporting Peptides:

| Peptide | Uniq | -10lgP | Mass | Length | ppm | m/z | z | RT | Fraction | Scan | Source File | Area F1 | Area F2 | Area F3 | Area F4 | Area F5 | Area F6 | Area F7 | Area F8 | Area F9 | Area F10 | #Feature | #Feature F1 | #Feature F2 | #Feature F3 | #Feature F4 | #Feature F5 | #Feature F6 | #Feature F7 | #Feature F8 | #Feature F9 | #Feature F10 | Start | End | PTM |
| --- | --- | --- | --- | --- | --- | --- | --- | --- | --- | --- | --- | --- | --- | --- | --- | --- | --- | --- | --- | --- | --- | --- | --- | --- | --- | --- | --- | --- | --- | --- | --- | --- | --- | --- | --- |
| K.LEAAVAQSEQQGEAALSDAR.C | Y | 105.26 | 2042.9868 | 20 | 0.5 | 1022.5012 | 2 | 11.85 | 3 | F3:2599 | 05112019\_RID\_1313\_NaNaRj\_F3.raw |  |  | 4.54E4 |  |  |  |  |  |  |  | 2 | 0 | 0 | 2 | 0 | 0 | 0 | 0 | 0 | 0 | 0 | 346 | 365 |  |
| R.GGVVC(+57.02)GDLC(+57.02)ASTTAPVVSTR.V | Y | 78.99 | 2005.9561 | 20 | 0.8 | 1003.9861 | 2 | 12.06 | 3 | F3:2716 | 05112019\_RID\_1313\_NaNaRj\_F3.raw |  |  | 2.17E4 |  |  |  |  |  |  |  | 1 | 0 | 0 | 1 | 0 | 0 | 0 | 0 | 0 | 0 | 0 | 433 | 452 | Carbamidomethylation |
| K.LAELEGALQK.A | N | 55.73 | 1070.5972 | 10 | 0.8 | 536.3063 | 2 | 11.77 | 3 | F3:2545 | 05112019\_RID\_1313\_NaNaRj\_F3.raw |  |  | 2.85E4 |  |  |  |  |  |  |  | 1 | 0 | 0 | 1 | 0 | 0 | 0 | 0 | 0 | 0 | 0 | 368 | 377 |  |
| K.AQYDDIVTR.S | Y | 50.35 | 1079.5247 | 9 | 1.0 | 540.7701 | 2 | 11.44 | 3 | F3:2239 | 05112019\_RID\_1313\_NaNaRj\_F3.raw |  |  | 1.58E4 |  |  |  |  |  |  |  | 1 | 0 | 0 | 1 | 0 | 0 | 0 | 0 | 0 | 0 | 0 | 280 | 288 |  |
| R.VSSVPSNSNVVVGTTNAC(+57.02)APSAR.V | Y | 41.82 | 2273.1069 | 23 | 1.3 | 758.7106 | 3 | 11.51 | 3 | F3:2322 | 05112019\_RID\_1313\_NaNaRj\_F3.raw |  |  | 5.12E3 |  |  |  |  |  |  |  | 1 | 0 | 0 | 1 | 0 | 0 | 0 | 0 | 0 | 0 | 0 | 453 | 475 | Carbamidomethylation |
| total 5 peptides |  |  |  |  |  |  |  |  |  |  |  |  |  |  |  |  |  |  |  |  |  |  |  |  |  |  |  |  |  |  |  |  |  |  |  |
| --- | --- | --- | --- | --- | --- | --- | --- | --- | --- | --- | --- | --- | --- | --- | --- | --- | --- | --- | --- | --- | --- | --- | --- | --- | --- | --- | --- | --- | --- | --- | --- | --- | --- | --- | --- |

#CONTAM#KRHB6\_HUMAN|

back to list

  

| Protein Coverage
| Supporting Peptides
|

Protein Coverage:

Supporting Peptides:

| Peptide | Uniq | -10lgP | Mass | Length | ppm | m/z | z | RT | Fraction | Scan | Source File | Area F1 | Area F2 | Area F3 | Area F4 | Area F5 | Area F6 | Area F7 | Area F8 | Area F9 | Area F10 | #Feature | #Feature F1 | #Feature F2 | #Feature F3 | #Feature F4 | #Feature F5 | #Feature F6 | #Feature F7 | #Feature F8 | #Feature F9 | #Feature F10 | Start | End | PTM |
| --- | --- | --- | --- | --- | --- | --- | --- | --- | --- | --- | --- | --- | --- | --- | --- | --- | --- | --- | --- | --- | --- | --- | --- | --- | --- | --- | --- | --- | --- | --- | --- | --- | --- | --- | --- |
| K.LEAAVAQSEQQGEAALSDAR.C | Y | 105.26 | 2042.9868 | 20 | 0.5 | 1022.5012 | 2 | 11.85 | 3 | F3:2599 | 05112019\_RID\_1313\_NaNaRj\_F3.raw |  |  | 4.54E4 |  |  |  |  |  |  |  | 2 | 0 | 0 | 2 | 0 | 0 | 0 | 0 | 0 | 0 | 0 | 346 | 365 |  |
| R.GGVVC(+57.02)GDLC(+57.02)ASTTAPVVSTR.V | Y | 78.99 | 2005.9561 | 20 | 0.8 | 1003.9861 | 2 | 12.06 | 3 | F3:2716 | 05112019\_RID\_1313\_NaNaRj\_F3.raw |  |  | 2.17E4 |  |  |  |  |  |  |  | 1 | 0 | 0 | 1 | 0 | 0 | 0 | 0 | 0 | 0 | 0 | 433 | 452 | Carbamidomethylation |
| K.LAELEGALQK.A | N | 55.73 | 1070.5972 | 10 | 0.8 | 536.3063 | 2 | 11.77 | 3 | F3:2545 | 05112019\_RID\_1313\_NaNaRj\_F3.raw |  |  | 2.85E4 |  |  |  |  |  |  |  | 1 | 0 | 0 | 1 | 0 | 0 | 0 | 0 | 0 | 0 | 0 | 368 | 377 |  |
| K.AQYDDIVTR.S | Y | 50.35 | 1079.5247 | 9 | 1.0 | 540.7701 | 2 | 11.44 | 3 | F3:2239 | 05112019\_RID\_1313\_NaNaRj\_F3.raw |  |  | 1.58E4 |  |  |  |  |  |  |  | 1 | 0 | 0 | 1 | 0 | 0 | 0 | 0 | 0 | 0 | 0 | 280 | 288 |  |
| R.VSSVPSNSNVVVGTTNAC(+57.02)APSAR.V | Y | 41.82 | 2273.1069 | 23 | 1.3 | 758.7106 | 3 | 11.51 | 3 | F3:2322 | 05112019\_RID\_1313\_NaNaRj\_F3.raw |  |  | 5.12E3 |  |  |  |  |  |  |  | 1 | 0 | 0 | 1 | 0 | 0 | 0 | 0 | 0 | 0 | 0 | 453 | 475 | Carbamidomethylation |
| total 5 peptides |  |  |  |  |  |  |  |  |  |  |  |  |  |  |  |  |  |  |  |  |  |  |  |  |  |  |  |  |  |  |  |  |  |  |  |
| --- | --- | --- | --- | --- | --- | --- | --- | --- | --- | --- | --- | --- | --- | --- | --- | --- | --- | --- | --- | --- | --- | --- | --- | --- | --- | --- | --- | --- | --- | --- | --- | --- | --- | --- | --- |

P19012|K1C15\_HUMAN

back to list

  

| Protein Coverage
| Supporting Peptides
|

Protein Coverage:

Supporting Peptides:

| Peptide | Uniq | -10lgP | Mass | Length | ppm | m/z | z | RT | Fraction | Scan | Source File | Area F1 | Area F2 | Area F3 | Area F4 | Area F5 | Area F6 | Area F7 | Area F8 | Area F9 | Area F10 | #Feature | #Feature F1 | #Feature F2 | #Feature F3 | #Feature F4 | #Feature F5 | #Feature F6 | #Feature F7 | #Feature F8 | #Feature F9 | #Feature F10 | Start | End | PTM |
| --- | --- | --- | --- | --- | --- | --- | --- | --- | --- | --- | --- | --- | --- | --- | --- | --- | --- | --- | --- | --- | --- | --- | --- | --- | --- | --- | --- | --- | --- | --- | --- | --- | --- | --- | --- |
| R.ALEEANADLEVK.I | N | 77.48 | 1300.6510 | 12 | -0.4 | 651.3325 | 2 | 11.60 | 3 | F3:2384 | 05112019\_RID\_1313\_NaNaRj\_F3.raw |  | 8.85E5 | 2.04E5 |  | 2.3E5 |  |  |  |  | 4.99E4 | 4 | 0 | 1 | 1 | 0 | 1 | 0 | 0 | 0 | 0 | 1 | 125 | 136 |  |
| R.LEQEIATYR.S | N | 59.43 | 1121.5717 | 9 | -0.1 | 561.7930 | 2 | 11.44 | 5 | F5:2290 | 05112019\_RID\_1313\_NaNaRj\_F5.raw |  | 1.62E6 | 3.06E4 |  | 4.88E5 |  |  |  |  | 1.13E4 | 4 | 0 | 1 | 1 | 0 | 1 | 0 | 0 | 0 | 0 | 1 | 399 | 407 |  |
| R.VLDELTLAR.T | N | 57.93 | 1028.5865 | 9 | -0.6 | 515.3002 | 2 | 13.17 | 5 | F5:3428 | 05112019\_RID\_1313\_NaNaRj\_F5.raw |  |  | 2.21E5 | 7.14E4 | 4.87E5 | 1.16E5 | 6.95E4 | 2.69E4 |  | 2.37E4 | 7 | 0 | 0 | 1 | 1 | 1 | 1 | 1 | 1 | 0 | 1 | 215 | 223 |  |
| K.TRLEQEIATYR.S | N | 50.16 | 1378.7205 | 11 | 0.4 | 690.3678 | 2 | 32.95 | 2 | F2:9394 | 09012020\_NaNaRj\_F2.raw |  | 4.37E5 |  |  |  |  |  |  |  |  | 1 | 0 | 1 | 0 | 0 | 0 | 0 | 0 | 0 | 0 | 0 | 397 | 407 |  |
| R.LASYLDK.V | N | 47.75 | 808.4330 | 7 | 0.6 | 405.2241 | 2 | 11.34 | 5 | F5:2185 | 05112019\_RID\_1313\_NaNaRj\_F5.raw | 3.03E4 | 9.71E6 | 7.78E4 |  | 7.33E5 |  |  |  |  | 3.94E4 | 5 | 1 | 1 | 1 | 0 | 1 | 0 | 0 | 0 | 0 | 1 | 116 | 122 |  |
| R.LAADDFR.L | N | 46.83 | 806.3922 | 7 | 0.6 | 404.2036 | 2 | 11.46 | 5 | F5:2311 | 05112019\_RID\_1313\_NaNaRj\_F5.raw |  | 5.1E6 | 5.72E4 |  | 4.73E5 |  | 1.38E4 |  |  | 3.3E4 | 6 | 0 | 2 | 1 | 0 | 1 | 0 | 1 | 0 | 0 | 1 | 186 | 192 |  |
| V.GGGFGGGFGGGDGGLLSGNEK.I | Y | 45.85 | 1795.8125 | 21 | 0.5 | 898.9139 | 2 | 16.60 | 5 | F5:4267 | 05112019\_RID\_1313\_NaNaRj\_F5.raw |  |  |  |  | 1.78E4 |  |  |  |  |  | 1 | 0 | 0 | 0 | 0 | 1 | 0 | 0 | 0 | 0 | 0 | 86 | 106 |  |
| R.LASYLDKVR.A | N | 45.41 | 1063.6025 | 9 | 1.0 | 532.8091 | 2 | 27.68 | 2 | F2:7299 | 09012020\_NaNaRj\_F2.raw |  | 2.48E5 |  |  |  |  |  |  |  |  | 1 | 0 | 1 | 0 | 0 | 0 | 0 | 0 | 0 | 0 | 0 | 116 | 124 |  |
| G.GGFGGGDGGLLSGNEK.I | Y | 44.60 | 1420.6582 | 16 | 0.2 | 711.3365 | 2 | 11.96 | 5 | F5:2806 | 05112019\_RID\_1313\_NaNaRj\_F5.raw |  |  |  |  | 1.91E4 |  |  |  |  |  | 1 | 0 | 0 | 0 | 0 | 1 | 0 | 0 | 0 | 0 | 0 | 91 | 106 |  |
| total 9 peptides |  |  |  |  |  |  |  |  |  |  |  |  |  |  |  |  |  |  |  |  |  |  |  |  |  |  |  |  |  |  |  |  |  |  |  |
| --- | --- | --- | --- | --- | --- | --- | --- | --- | --- | --- | --- | --- | --- | --- | --- | --- | --- | --- | --- | --- | --- | --- | --- | --- | --- | --- | --- | --- | --- | --- | --- | --- | --- | --- | --- |

P15924|DESP\_HUMAN

back to list

  

| Protein Coverage
| Supporting Peptides
|

Protein Coverage:

Supporting Peptides:

| Peptide | Uniq | -10lgP | Mass | Length | ppm | m/z | z | RT | Fraction | Scan | Source File | Area F1 | Area F2 | Area F3 | Area F4 | Area F5 | Area F6 | Area F7 | Area F8 | Area F9 | Area F10 | #Feature | #Feature F1 | #Feature F2 | #Feature F3 | #Feature F4 | #Feature F5 | #Feature F6 | #Feature F7 | #Feature F8 | #Feature F9 | #Feature F10 | Start | End | PTM |
| --- | --- | --- | --- | --- | --- | --- | --- | --- | --- | --- | --- | --- | --- | --- | --- | --- | --- | --- | --- | --- | --- | --- | --- | --- | --- | --- | --- | --- | --- | --- | --- | --- | --- | --- | --- |
| K.YGDGIQLTR.S | Y | 61.46 | 1021.5192 | 9 | 0.0 | 511.7669 | 2 | 33.76 | 2 | F2:9758 | 09012020\_NaNaRj\_F2.raw |  | 1.24E5 |  |  |  |  |  |  |  |  | 1 | 0 | 1 | 0 | 0 | 0 | 0 | 0 | 0 | 0 | 0 | 95 | 103 |  |
| R.VVIVDPETNK.E | Y | 57.46 | 1112.6077 | 10 | -0.8 | 557.3107 | 2 | 31.20 | 2 | F2:8736 | 09012020\_NaNaRj\_F2.raw |  | 5.24E4 |  |  |  |  |  |  |  |  | 1 | 0 | 1 | 0 | 0 | 0 | 0 | 0 | 0 | 0 | 0 | 2466 | 2475 |  |
| K.FGDSNTVMR.F | Y | 48.89 | 1025.4600 | 9 | 0.7 | 513.7376 | 2 | 24.49 | 2 | F2:6009 | 09012020\_NaNaRj\_F2.raw |  | 3.81E4 |  |  |  |  |  |  |  |  | 1 | 0 | 1 | 0 | 0 | 0 | 0 | 0 | 0 | 0 | 0 | 917 | 925 |  |
| R.LPVEEAYK.R | Y | 47.78 | 947.4963 | 8 | -0.5 | 474.7552 | 2 | 27.49 | 2 | F2:7246 | 09012020\_NaNaRj\_F2.raw |  | 9.71E4 |  |  |  |  |  |  |  |  | 1 | 0 | 1 | 0 | 0 | 0 | 0 | 0 | 0 | 0 | 0 | 2310 | 2317 |  |
| K.NLPLADQGSSHHITVK.I | Y | 47.00 | 1715.8955 | 16 | -0.3 | 572.9723 | 3 | 25.25 | 2 | F2:6351 | 09012020\_NaNaRj\_F2.raw |  | 1.04E5 |  |  |  |  |  |  |  |  | 1 | 0 | 1 | 0 | 0 | 0 | 0 | 0 | 0 | 0 | 0 | 690 | 705 |  |
| R.AELIVQPELK.Y | Y | 45.06 | 1138.6597 | 10 | 0.9 | 570.3376 | 2 | 12.47 | 3 | F3:2875 | 05112019\_RID\_1313\_NaNaRj\_F3.raw |  |  | 1.33E4 |  |  |  |  |  |  |  | 1 | 0 | 0 | 1 | 0 | 0 | 0 | 0 | 0 | 0 | 0 | 85 | 94 |  |
| R.VLLQEEGTR.K | Y | 44.62 | 1043.5610 | 9 | -0.1 | 522.7877 | 2 | 24.74 | 2 | F2:6131 | 09012020\_NaNaRj\_F2.raw |  | 1.59E5 |  |  |  |  |  |  |  |  | 1 | 0 | 1 | 0 | 0 | 0 | 0 | 0 | 0 | 0 | 0 | 1176 | 1184 |  |
| R.LLEAQIATGGIIDPK.E | Y | 43.56 | 1537.8715 | 15 | 0.6 | 769.9435 | 2 | 17.48 | 3 | F3:3777 | 05112019\_RID\_1313\_NaNaRj\_F3.raw |  |  | 2.05E4 |  |  |  |  |  |  |  | 1 | 0 | 0 | 1 | 0 | 0 | 0 | 0 | 0 | 0 | 0 | 2367 | 2381 |  |
| R.QLQNIIQATSR.E | Y | 43.19 | 1270.6993 | 11 | 0.6 | 636.3573 | 2 | 11.83 | 3 | F3:2585 | 05112019\_RID\_1313\_NaNaRj\_F3.raw |  |  | 8.12E3 |  |  |  |  |  |  |  | 1 | 0 | 0 | 1 | 0 | 0 | 0 | 0 | 0 | 0 | 0 | 271 | 281 |  |
| K.LASLEELKR.Q | Y | 40.51 | 1057.6132 | 9 | 0.2 | 529.8140 | 2 | 28.45 | 2 | F2:7618 | 09012020\_NaNaRj\_F2.raw |  | 5.19E4 |  |  |  |  |  |  |  |  | 1 | 0 | 1 | 0 | 0 | 0 | 0 | 0 | 0 | 0 | 0 | 1076 | 1084 |  |
| total 10 peptides |  |  |  |  |  |  |  |  |  |  |  |  |  |  |  |  |  |  |  |  |  |  |  |  |  |  |  |  |  |  |  |  |  |  |  |
| --- | --- | --- | --- | --- | --- | --- | --- | --- | --- | --- | --- | --- | --- | --- | --- | --- | --- | --- | --- | --- | --- | --- | --- | --- | --- | --- | --- | --- | --- | --- | --- | --- | --- | --- | --- |

D6PXE8|VM3B\_NAJAT

back to list

  

| Protein Coverage
| Supporting Peptides
|

Protein Coverage:

Supporting Peptides:

| Peptide | Uniq | -10lgP | Mass | Length | ppm | m/z | z | RT | Fraction | Scan | Source File | Area F1 | Area F2 | Area F3 | Area F4 | Area F5 | Area F6 | Area F7 | Area F8 | Area F9 | Area F10 | #Feature | #Feature F1 | #Feature F2 | #Feature F3 | #Feature F4 | #Feature F5 | #Feature F6 | #Feature F7 | #Feature F8 | #Feature F9 | #Feature F10 | Start | End | PTM |
| --- | --- | --- | --- | --- | --- | --- | --- | --- | --- | --- | --- | --- | --- | --- | --- | --- | --- | --- | --- | --- | --- | --- | --- | --- | --- | --- | --- | --- | --- | --- | --- | --- | --- | --- | --- |
| K.DDC(+57.02)DLPELC(+57.02)TGQSAEC(+57.02)PTDSLQR.N | Y | 69.60 | 2666.0894 | 23 | -0.1 | 1334.0519 | 2 | 14.92 | 8 | F8:4502 | 05112019\_RID\_1313\_NaNaRj\_F8.raw |  |  |  |  |  | 5.16E5 |  | 3.75E6 | 1.17E5 |  | 7 | 0 | 0 | 0 | 0 | 0 | 2 | 0 | 4 | 1 | 0 | 454 | 476 | Carbamidomethylation |
| R.TAPAFQFSSC(+57.02)SIR.E | N | 60.29 | 1470.6925 | 13 | 1.0 | 736.3542 | 2 | 12.55 | 9 | F9:2932 | 05112019\_RID\_1313\_NaNaRj\_F9.raw |  |  |  |  |  |  | 1.39E5 |  | 1.85E4 |  | 2 | 0 | 0 | 0 | 0 | 0 | 0 | 1 | 0 | 1 | 0 | 370 | 382 | Carbamidomethylation |
| A.LIGLEIWSNK.D | Y | 60.20 | 1171.6600 | 10 | 0.7 | 586.8377 | 2 | 24.46 | 8 | F8:8402 | 05112019\_RID\_1313\_NaNaRj\_F8.raw |  |  |  |  |  |  |  | 3.83E5 |  |  | 1 | 0 | 0 | 0 | 0 | 0 | 0 | 0 | 1 | 0 | 0 | 252 | 261 |  |
| R.VYEM(+15.99)INAVNTK.F | N | 59.01 | 1296.6384 | 11 | 0.3 | 649.3267 | 2 | 11.32 | 9 | F9:2053 | 05112019\_RID\_1313\_NaNaRj\_F9.raw |  |  |  |  |  |  |  |  | 4.99E4 |  | 1 | 0 | 0 | 0 | 0 | 0 | 0 | 0 | 0 | 1 | 0 | 232 | 242 | Oxidation (M) |
| H.IALIGLEIWSNK.D | Y | 56.71 | 1355.7812 | 12 | 0.9 | 678.8985 | 2 | 37.36 | 8 | F8:13964 | 05112019\_RID\_1313\_NaNaRj\_F8.raw |  |  |  |  |  |  | 1.88E5 | 1.83E6 | 5.48E4 |  | 4 | 0 | 0 | 0 | 0 | 0 | 0 | 1 | 2 | 1 | 0 | 250 | 261 |  |
| I.HIALIGLEIWSNK.D | Y | 54.45 | 1492.8402 | 13 | 0.2 | 498.6208 | 3 | 24.23 | 8 | F8:8275 | 05112019\_RID\_1313\_NaNaRj\_F8.raw |  |  |  |  |  |  |  | 1.01E5 |  |  | 1 | 0 | 0 | 0 | 0 | 0 | 0 | 0 | 1 | 0 | 0 | 249 | 261 |  |
| R.NDNAQLLTGIDFNGNTVGR.A | Y | 50.63 | 2017.9817 | 19 | 1.6 | 1009.9998 | 2 | 20.33 | 8 | F8:6955 | 05112019\_RID\_1313\_NaNaRj\_F8.raw |  |  |  |  |  |  |  | 2.18E4 |  |  | 1 | 0 | 0 | 0 | 0 | 0 | 0 | 0 | 1 | 0 | 0 | 291 | 309 |  |
| total 7 peptides |  |  |  |  |  |  |  |  |  |  |  |  |  |  |  |  |  |  |  |  |  |  |  |  |  |  |  |  |  |  |  |  |  |  |  |
| --- | --- | --- | --- | --- | --- | --- | --- | --- | --- | --- | --- | --- | --- | --- | --- | --- | --- | --- | --- | --- | --- | --- | --- | --- | --- | --- | --- | --- | --- | --- | --- | --- | --- | --- | --- |

D3TTC1|VM3KL\_NAJAT

back to list

  

| Protein Coverage
| Supporting Peptides
|

Protein Coverage:

Supporting Peptides:

| Peptide | Uniq | -10lgP | Mass | Length | ppm | m/z | z | RT | Fraction | Scan | Source File | Area F1 | Area F2 | Area F3 | Area F4 | Area F5 | Area F6 | Area F7 | Area F8 | Area F9 | Area F10 | #Feature | #Feature F1 | #Feature F2 | #Feature F3 | #Feature F4 | #Feature F5 | #Feature F6 | #Feature F7 | #Feature F8 | #Feature F9 | #Feature F10 | Start | End | PTM |
| --- | --- | --- | --- | --- | --- | --- | --- | --- | --- | --- | --- | --- | --- | --- | --- | --- | --- | --- | --- | --- | --- | --- | --- | --- | --- | --- | --- | --- | --- | --- | --- | --- | --- | --- | --- |
| K.DDC(+57.02)DLPELC(+57.02)TGQSAEC(+57.02)PTDSLQR.N | Y | 69.60 | 2666.0894 | 23 | -0.1 | 1334.0519 | 2 | 14.92 | 8 | F8:4502 | 05112019\_RID\_1313\_NaNaRj\_F8.raw |  |  |  |  |  | 5.16E5 |  | 3.75E6 | 1.17E5 |  | 7 | 0 | 0 | 0 | 0 | 0 | 2 | 0 | 4 | 1 | 0 | 454 | 476 | Carbamidomethylation |
| R.TAPAFQFSSC(+57.02)SIR.E | N | 60.29 | 1470.6925 | 13 | 1.0 | 736.3542 | 2 | 12.55 | 9 | F9:2932 | 05112019\_RID\_1313\_NaNaRj\_F9.raw |  |  |  |  |  |  | 1.39E5 |  | 1.85E4 |  | 2 | 0 | 0 | 0 | 0 | 0 | 0 | 1 | 0 | 1 | 0 | 370 | 382 | Carbamidomethylation |
| A.LIGLEIWSNK.D | Y | 60.20 | 1171.6600 | 10 | 0.7 | 586.8377 | 2 | 24.46 | 8 | F8:8402 | 05112019\_RID\_1313\_NaNaRj\_F8.raw |  |  |  |  |  |  |  | 3.83E5 |  |  | 1 | 0 | 0 | 0 | 0 | 0 | 0 | 0 | 1 | 0 | 0 | 252 | 261 |  |
| R.VYEM(+15.99)INAVNTK.F | N | 59.01 | 1296.6384 | 11 | 0.3 | 649.3267 | 2 | 11.32 | 9 | F9:2053 | 05112019\_RID\_1313\_NaNaRj\_F9.raw |  |  |  |  |  |  |  |  | 4.99E4 |  | 1 | 0 | 0 | 0 | 0 | 0 | 0 | 0 | 0 | 1 | 0 | 232 | 242 | Oxidation (M) |
| H.IALIGLEIWSNK.D | Y | 56.71 | 1355.7812 | 12 | 0.9 | 678.8985 | 2 | 37.36 | 8 | F8:13964 | 05112019\_RID\_1313\_NaNaRj\_F8.raw |  |  |  |  |  |  | 1.88E5 | 1.83E6 | 5.48E4 |  | 4 | 0 | 0 | 0 | 0 | 0 | 0 | 1 | 2 | 1 | 0 | 250 | 261 |  |
| I.HIALIGLEIWSNK.D | Y | 54.45 | 1492.8402 | 13 | 0.2 | 498.6208 | 3 | 24.23 | 8 | F8:8275 | 05112019\_RID\_1313\_NaNaRj\_F8.raw |  |  |  |  |  |  |  | 1.01E5 |  |  | 1 | 0 | 0 | 0 | 0 | 0 | 0 | 0 | 1 | 0 | 0 | 249 | 261 |  |
| R.NDNAQLLTGIDFNGNTVGR.A | Y | 50.63 | 2017.9817 | 19 | 1.6 | 1009.9998 | 2 | 20.33 | 8 | F8:6955 | 05112019\_RID\_1313\_NaNaRj\_F8.raw |  |  |  |  |  |  |  | 2.18E4 |  |  | 1 | 0 | 0 | 0 | 0 | 0 | 0 | 0 | 1 | 0 | 0 | 291 | 309 |  |
| total 7 peptides |  |  |  |  |  |  |  |  |  |  |  |  |  |  |  |  |  |  |  |  |  |  |  |  |  |  |  |  |  |  |  |  |  |  |  |
| --- | --- | --- | --- | --- | --- | --- | --- | --- | --- | --- | --- | --- | --- | --- | --- | --- | --- | --- | --- | --- | --- | --- | --- | --- | --- | --- | --- | --- | --- | --- | --- | --- | --- | --- | --- |

P81605|DCD\_HUMAN

back to list

  

| Protein Coverage
| Supporting Peptides
|

Protein Coverage:

Supporting Peptides:

| Peptide | Uniq | -10lgP | Mass | Length | ppm | m/z | z | RT | Fraction | Scan | Source File | Area F1 | Area F2 | Area F3 | Area F4 | Area F5 | Area F6 | Area F7 | Area F8 | Area F9 | Area F10 | #Feature | #Feature F1 | #Feature F2 | #Feature F3 | #Feature F4 | #Feature F5 | #Feature F6 | #Feature F7 | #Feature F8 | #Feature F9 | #Feature F10 | Start | End | PTM |
| --- | --- | --- | --- | --- | --- | --- | --- | --- | --- | --- | --- | --- | --- | --- | --- | --- | --- | --- | --- | --- | --- | --- | --- | --- | --- | --- | --- | --- | --- | --- | --- | --- | --- | --- | --- |
| A.YDPEAASAPGSGNPC(+57.02)HEASAAQK.E | Y | 83.74 | 2313.9919 | 23 | 0.6 | 772.3384 | 3 | 11.00 | 1 | F1:1830 | 05112019\_RID\_1313\_NaNaRj\_F1.raw | 1.36E5 | 1.2E6 | 7.85E4 | 5.56E4 |  |  |  |  |  |  | 4 | 1 | 1 | 1 | 1 | 0 | 0 | 0 | 0 | 0 | 0 | 20 | 42 | Carbamidomethylation |
| K.DAVEDLESVGK.G | Y | 73.02 | 1160.5560 | 11 | 0.9 | 581.2858 | 2 | 12.45 | 5 | F5:3149 | 05112019\_RID\_1313\_NaNaRj\_F5.raw | 1.36E4 | 8.3E5 | 2.21E4 |  | 1.07E5 |  |  |  | 1.64E4 | 5.73E3 | 6 | 1 | 1 | 1 | 0 | 1 | 0 | 0 | 0 | 1 | 1 | 86 | 96 |  |
| K.ENAGEDPGLAR.Q | Y | 66.68 | 1127.5206 | 11 | 0.3 | 564.7678 | 2 | 10.96 | 5 | F5:1814 | 05112019\_RID\_1313\_NaNaRj\_F5.raw | 8.34E4 |  |  |  | 2.32E5 |  |  |  | 7.78E4 | 3.75E4 | 4 | 1 | 0 | 0 | 0 | 1 | 0 | 0 | 0 | 1 | 1 | 43 | 53 |  |
| K.LGKDAVEDLESVGK.G | Y | 54.25 | 1458.7566 | 14 | 1.1 | 487.2600 | 3 | 41.53 | 2 | F2:12995 | 09012020\_NaNaRj\_F2.raw |  | 8.28E4 |  |  |  |  |  |  |  |  | 1 | 0 | 1 | 0 | 0 | 0 | 0 | 0 | 0 | 0 | 0 | 83 | 96 |  |
| total 4 peptides |  |  |  |  |  |  |  |  |  |  |  |  |  |  |  |  |  |  |  |  |  |  |  |  |  |  |  |  |  |  |  |  |  |  |  |
| --- | --- | --- | --- | --- | --- | --- | --- | --- | --- | --- | --- | --- | --- | --- | --- | --- | --- | --- | --- | --- | --- | --- | --- | --- | --- | --- | --- | --- | --- | --- | --- | --- | --- | --- | --- |

P01400|3NO2B\_NAJME

back to list

  

| Protein Coverage
| Supporting Peptides
|

Protein Coverage:

Supporting Peptides:

| Peptide | Uniq | -10lgP | Mass | Length | ppm | m/z | z | RT | Fraction | Scan | Source File | Area F1 | Area F2 | Area F3 | Area F4 | Area F5 | Area F6 | Area F7 | Area F8 | Area F9 | Area F10 | #Feature | #Feature F1 | #Feature F2 | #Feature F3 | #Feature F4 | #Feature F5 | #Feature F6 | #Feature F7 | #Feature F8 | #Feature F9 | #Feature F10 | Start | End | PTM |
| --- | --- | --- | --- | --- | --- | --- | --- | --- | --- | --- | --- | --- | --- | --- | --- | --- | --- | --- | --- | --- | --- | --- | --- | --- | --- | --- | --- | --- | --- | --- | --- | --- | --- | --- | --- |
| R.FYEGNLLGK.R | N | 69.74 | 1039.5338 | 9 | 0.1 | 520.7742 | 2 | 12.75 | 4 | F4:3423 | 05112019\_RID\_1313\_NaNaRj\_F4.raw |  |  |  | 3.92E8 |  | 8.38E5 | 4.1E5 | 9.44E4 |  |  | 10 | 0 | 0 | 0 | 7 | 0 | 1 | 1 | 1 | 0 | 0 | 28 | 36 |  |
| LTC(+57.02)LIC(+57.02)PEKYC(+57.02)NK.V | N | 67.27 | 1697.7939 | 13 | 0.6 | 566.9389 | 3 | 11.61 | 4 | F4:2457 | 05112019\_RID\_1313\_NaNaRj\_F4.raw |  |  |  | 1.02E6 |  |  |  |  |  |  | 2 | 0 | 0 | 0 | 2 | 0 | 0 | 0 | 0 | 0 | 0 | 1 | 13 | Carbamidomethylation |
| LTC(+57.02)LIC(+57.02)PEKYC(+57.02)NKVHTC(+57.02)R.N | N | 63.67 | 2351.1006 | 18 | 0.7 | 471.2277 | 5 | 11.36 | 4 | F4:2200 | 05112019\_RID\_1313\_NaNaRj\_F4.raw |  |  |  | 4.71E5 |  |  |  |  |  |  | 2 | 0 | 0 | 0 | 2 | 0 | 0 | 0 | 0 | 0 | 0 | 1 | 18 | Carbamidomethylation |
| LTC(+57.02)LIC(+57.02)PEK.Y | N | 62.81 | 1132.5620 | 9 | -1.0 | 567.2877 | 2 | 11.81 | 4 | F4:2581 | 05112019\_RID\_1313\_NaNaRj\_F4.raw |  |  |  | 4.45E8 | 3.88E4 | 7.2E5 | 3.64E5 |  |  |  | 29 | 0 | 0 | 0 | 26 | 1 | 1 | 1 | 0 | 0 | 0 | 1 | 9 | Carbamidomethylation |
| R.FYEGNLLGKR.Y | Y | 60.78 | 1195.6349 | 10 | -1.7 | 399.5516 | 3 | 11.56 | 4 | F4:2404 | 05112019\_RID\_1313\_NaNaRj\_F4.raw |  |  |  | 4.02E5 |  |  |  |  |  |  | 1 | 0 | 0 | 0 | 1 | 0 | 0 | 0 | 0 | 0 | 0 | 28 | 37 |  |
| K.RFYEGNLLGK.R | Y | 59.54 | 1195.6349 | 10 | 0.3 | 598.8249 | 2 | 11.61 | 4 | F4:2433 | 05112019\_RID\_1313\_NaNaRj\_F4.raw |  |  |  | 4.51E6 |  |  |  |  |  |  | 1 | 0 | 0 | 0 | 1 | 0 | 0 | 0 | 0 | 0 | 0 | 27 | 36 |  |
| R.EIVEC(+57.02)C(+57.02)STDK.C | N | 48.63 | 1239.5111 | 10 | 0.6 | 620.7632 | 2 | 10.79 | 4 | F4:1698 | 05112019\_RID\_1313\_NaNaRj\_F4.raw |  |  |  | 5E4 |  |  |  |  |  |  | 1 | 0 | 0 | 0 | 1 | 0 | 0 | 0 | 0 | 0 | 0 | 53 | 62 | Carbamidomethylation |
| total 7 peptides |  |  |  |  |  |  |  |  |  |  |  |  |  |  |  |  |  |  |  |  |  |  |  |  |  |  |  |  |  |  |  |  |  |  |  |
| --- | --- | --- | --- | --- | --- | --- | --- | --- | --- | --- | --- | --- | --- | --- | --- | --- | --- | --- | --- | --- | --- | --- | --- | --- | --- | --- | --- | --- | --- | --- | --- | --- | --- | --- | --- |

P29182|3NO28\_NAJNA

back to list

  

| Protein Coverage
| Supporting Peptides
|

Protein Coverage:

Supporting Peptides:

| Peptide | Uniq | -10lgP | Mass | Length | ppm | m/z | z | RT | Fraction | Scan | Source File | Area F1 | Area F2 | Area F3 | Area F4 | Area F5 | Area F6 | Area F7 | Area F8 | Area F9 | Area F10 | #Feature | #Feature F1 | #Feature F2 | #Feature F3 | #Feature F4 | #Feature F5 | #Feature F6 | #Feature F7 | #Feature F8 | #Feature F9 | #Feature F10 | Start | End | PTM |
| --- | --- | --- | --- | --- | --- | --- | --- | --- | --- | --- | --- | --- | --- | --- | --- | --- | --- | --- | --- | --- | --- | --- | --- | --- | --- | --- | --- | --- | --- | --- | --- | --- | --- | --- | --- |
| LTC(+57.02)LNC(+57.02)PEVYC(+57.02)R.R | Y | 91.44 | 1583.6895 | 12 | -0.1 | 792.8519 | 2 | 11.76 | 6 | F6:2540 | 05112019\_RID\_1313\_NaNaRj\_F6.raw |  |  |  | 3.48E8 | 1.69E4 | 4.12E5 | 4.26E5 |  |  |  | 24 | 0 | 0 | 0 | 21 | 1 | 1 | 1 | 0 | 0 | 0 | 1 | 12 | Carbamidomethylation |
| L.TC(+57.02)LNC(+57.02)PEVYC(+57.02)R.R | Y | 63.37 | 1470.6053 | 11 | -1.4 | 736.3089 | 2 | 11.51 | 4 | F4:2351 | 05112019\_RID\_1313\_NaNaRj\_F4.raw |  |  |  | 6.47E5 |  |  |  |  |  |  | 1 | 0 | 0 | 0 | 1 | 0 | 0 | 0 | 0 | 0 | 0 | 2 | 12 | Carbamidomethylation |
| LTC(+57.02)LNC(+57.02)PEVY.C | Y | 62.87 | 1267.5577 | 10 | 0.8 | 634.7866 | 2 | 15.47 | 4 | F4:5570 | 05112019\_RID\_1313\_NaNaRj\_F4.raw |  |  |  | 4.4E7 |  |  |  |  |  |  | 4 | 0 | 0 | 0 | 4 | 0 | 0 | 0 | 0 | 0 | 0 | 1 | 10 | Carbamidomethylation |
| C.LNC(+57.02)PEVYC(+57.02)R.R | Y | 52.46 | 1209.5271 | 9 | -0.1 | 605.7708 | 2 | 11.36 | 4 | F4:2197 | 05112019\_RID\_1313\_NaNaRj\_F4.raw |  |  |  | 2.49E5 |  |  |  |  |  |  | 1 | 0 | 0 | 0 | 1 | 0 | 0 | 0 | 0 | 0 | 0 | 4 | 12 | Carbamidomethylation |
| L.NC(+57.02)PEVYC(+57.02)R.R | Y | 49.31 | 1096.4430 | 8 | -0.4 | 549.2286 | 2 | 10.93 | 4 | F4:1795 | 05112019\_RID\_1313\_NaNaRj\_F4.raw |  |  |  | 1.82E5 |  |  |  |  |  |  | 1 | 0 | 0 | 0 | 1 | 0 | 0 | 0 | 0 | 0 | 0 | 5 | 12 | Carbamidomethylation |
| total 5 peptides |  |  |  |  |  |  |  |  |  |  |  |  |  |  |  |  |  |  |  |  |  |  |  |  |  |  |  |  |  |  |  |  |  |  |  |
| --- | --- | --- | --- | --- | --- | --- | --- | --- | --- | --- | --- | --- | --- | --- | --- | --- | --- | --- | --- | --- | --- | --- | --- | --- | --- | --- | --- | --- | --- | --- | --- | --- | --- | --- | --- |

P29181|3NO27\_NAJNA

back to list

  

| Protein Coverage
| Supporting Peptides
|

Protein Coverage:

Supporting Peptides:

| Peptide | Uniq | -10lgP | Mass | Length | ppm | m/z | z | RT | Fraction | Scan | Source File | Area F1 | Area F2 | Area F3 | Area F4 | Area F5 | Area F6 | Area F7 | Area F8 | Area F9 | Area F10 | #Feature | #Feature F1 | #Feature F2 | #Feature F3 | #Feature F4 | #Feature F5 | #Feature F6 | #Feature F7 | #Feature F8 | #Feature F9 | #Feature F10 | Start | End | PTM |
| --- | --- | --- | --- | --- | --- | --- | --- | --- | --- | --- | --- | --- | --- | --- | --- | --- | --- | --- | --- | --- | --- | --- | --- | --- | --- | --- | --- | --- | --- | --- | --- | --- | --- | --- | --- |
| LTC(+57.02)LNC(+57.02)PEVYC(+57.02)R.R | Y | 91.44 | 1583.6895 | 12 | -0.1 | 792.8519 | 2 | 11.76 | 6 | F6:2540 | 05112019\_RID\_1313\_NaNaRj\_F6.raw |  |  |  | 3.48E8 | 1.69E4 | 4.12E5 | 4.26E5 |  |  |  | 24 | 0 | 0 | 0 | 21 | 1 | 1 | 1 | 0 | 0 | 0 | 1 | 12 | Carbamidomethylation |
| L.TC(+57.02)LNC(+57.02)PEVYC(+57.02)R.R | Y | 63.37 | 1470.6053 | 11 | -1.4 | 736.3089 | 2 | 11.51 | 4 | F4:2351 | 05112019\_RID\_1313\_NaNaRj\_F4.raw |  |  |  | 6.47E5 |  |  |  |  |  |  | 1 | 0 | 0 | 0 | 1 | 0 | 0 | 0 | 0 | 0 | 0 | 2 | 12 | Carbamidomethylation |
| LTC(+57.02)LNC(+57.02)PEVY.C | Y | 62.87 | 1267.5577 | 10 | 0.8 | 634.7866 | 2 | 15.47 | 4 | F4:5570 | 05112019\_RID\_1313\_NaNaRj\_F4.raw |  |  |  | 4.4E7 |  |  |  |  |  |  | 4 | 0 | 0 | 0 | 4 | 0 | 0 | 0 | 0 | 0 | 0 | 1 | 10 | Carbamidomethylation |
| C.LNC(+57.02)PEVYC(+57.02)R.R | Y | 52.46 | 1209.5271 | 9 | -0.1 | 605.7708 | 2 | 11.36 | 4 | F4:2197 | 05112019\_RID\_1313\_NaNaRj\_F4.raw |  |  |  | 2.49E5 |  |  |  |  |  |  | 1 | 0 | 0 | 0 | 1 | 0 | 0 | 0 | 0 | 0 | 0 | 4 | 12 | Carbamidomethylation |
| L.NC(+57.02)PEVYC(+57.02)R.R | Y | 49.31 | 1096.4430 | 8 | -0.4 | 549.2286 | 2 | 10.93 | 4 | F4:1795 | 05112019\_RID\_1313\_NaNaRj\_F4.raw |  |  |  | 1.82E5 |  |  |  |  |  |  | 1 | 0 | 0 | 0 | 1 | 0 | 0 | 0 | 0 | 0 | 0 | 5 | 12 | Carbamidomethylation |
| total 5 peptides |  |  |  |  |  |  |  |  |  |  |  |  |  |  |  |  |  |  |  |  |  |  |  |  |  |  |  |  |  |  |  |  |  |  |  |
| --- | --- | --- | --- | --- | --- | --- | --- | --- | --- | --- | --- | --- | --- | --- | --- | --- | --- | --- | --- | --- | --- | --- | --- | --- | --- | --- | --- | --- | --- | --- | --- | --- | --- | --- | --- |

P07525|3SAT\_NAJAT

back to list

  

| Protein Coverage
| Supporting Peptides
|

Protein Coverage:

Supporting Peptides:

| Peptide | Uniq | -10lgP | Mass | Length | ppm | m/z | z | RT | Fraction | Scan | Source File | Area F1 | Area F2 | Area F3 | Area F4 | Area F5 | Area F6 | Area F7 | Area F8 | Area F9 | Area F10 | #Feature | #Feature F1 | #Feature F2 | #Feature F3 | #Feature F4 | #Feature F5 | #Feature F6 | #Feature F7 | #Feature F8 | #Feature F9 | #Feature F10 | Start | End | PTM |
| --- | --- | --- | --- | --- | --- | --- | --- | --- | --- | --- | --- | --- | --- | --- | --- | --- | --- | --- | --- | --- | --- | --- | --- | --- | --- | --- | --- | --- | --- | --- | --- | --- | --- | --- | --- |
| R.GC(+57.02)IDVC(+57.02)PKSSLLVK.Y | N | 68.52 | 1574.8160 | 14 | -3.9 | 788.4122 | 2 | 11.71 | 8 | F8:2538 | 05112019\_RID\_1313\_NaNaRj\_F8.raw |  |  |  |  |  |  | 0 | 3.04E6 |  |  | 3 | 0 | 0 | 0 | 0 | 0 | 0 | 1 | 2 | 0 | 0 | 37 | 50 | Carbamidomethylation |
| K.TC(+57.02)PAGKNLC(+57.02)YK.M | N | 59.00 | 1310.6111 | 11 | 0.7 | 437.8779 | 3 | 10.68 | 8 | F8:1633 | 05112019\_RID\_1313\_NaNaRj\_F8.raw |  |  |  |  |  |  |  | 2E5 |  |  | 1 | 0 | 0 | 0 | 0 | 0 | 0 | 0 | 1 | 0 | 0 | 13 | 23 | Carbamidomethylation |
| R.GC(+57.02)IDVC(+57.02)PK.S | N | 56.82 | 947.4205 | 8 | -2.7 | 474.7162 | 2 | 11.15 | 8 | F8:2003 | 05112019\_RID\_1313\_NaNaRj\_F8.raw | 1E5 | 1.9E5 | 1.09E4 | 8.89E4 | 5.11E5 | 3.82E8 |  | 3.84E7 | 5.76E5 | 1.59E4 | 23 | 1 | 1 | 1 | 1 | 1 | 14 | 0 | 2 | 1 | 1 | 37 | 44 | Carbamidomethylation |
| K.MFMVSNK.M | N | 53.69 | 855.3983 | 7 | -1.2 | 428.7059 | 2 | 11.64 | 6 | F6:2428 | 05112019\_RID\_1313\_NaNaRj\_F6.raw |  |  |  |  |  | 2.47E5 | 3.99E5 |  |  |  | 2 | 0 | 0 | 0 | 0 | 0 | 1 | 1 | 0 | 0 | 0 | 24 | 30 |  |
| K.SSLLVKYVC(+57.02).C | N | 50.14 | 1067.5685 | 9 | -0.1 | 534.7914 | 2 | 12.51 | 8 | F8:3206 | 05112019\_RID\_1313\_NaNaRj\_F8.raw |  |  |  |  |  |  |  | 2.17E5 |  |  | 1 | 0 | 0 | 0 | 0 | 0 | 0 | 0 | 1 | 0 | 0 | 45 | 53 | Carbamidomethylation |
| K.SSLLVKYVC(+57.02)C(+57.02).N | N | 43.03 | 1227.5991 | 10 | 0.0 | 614.8068 | 2 | 12.81 | 8 | F8:3446 | 05112019\_RID\_1313\_NaNaRj\_F8.raw |  |  |  |  |  |  |  | 0 |  |  | 1 | 0 | 0 | 0 | 0 | 0 | 0 | 0 | 1 | 0 | 0 | 45 | 54 | Carbamidomethylation |
| K.NLC(+57.02)YKMFM(+15.99)VSNKMVPVKR.G | Y | 41.84 | 2260.1353 | 18 | 1.4 | 566.0419 | 4 | 11.85 | 8 | F8:2644 | 05112019\_RID\_1313\_NaNaRj\_F8.raw |  |  |  |  |  |  |  | 9.99E5 |  |  | 1 | 0 | 0 | 0 | 0 | 0 | 0 | 0 | 1 | 0 | 0 | 19 | 36 | Carbamidomethylation; Oxidation (M) |
| K.LVPLFYK.T | N | 40.17 | 878.5266 | 7 | -0.1 | 440.2705 | 2 | 16.00 | 9 | F9:3716 | 05112019\_RID\_1313\_NaNaRj\_F9.raw |  |  |  |  |  |  |  |  | 4.25E5 |  | 1 | 0 | 0 | 0 | 0 | 0 | 0 | 0 | 0 | 1 | 0 | 6 | 12 |  |
| total 8 peptides |  |  |  |  |  |  |  |  |  |  |  |  |  |  |  |  |  |  |  |  |  |  |  |  |  |  |  |  |  |  |  |  |  |  |  |
| --- | --- | --- | --- | --- | --- | --- | --- | --- | --- | --- | --- | --- | --- | --- | --- | --- | --- | --- | --- | --- | --- | --- | --- | --- | --- | --- | --- | --- | --- | --- | --- | --- | --- | --- | --- |

Q9PST3|3SA2B\_NAJSP

back to list

  

| Protein Coverage
| Supporting Peptides
|

Protein Coverage:

Supporting Peptides:

| Peptide | Uniq | -10lgP | Mass | Length | ppm | m/z | z | RT | Fraction | Scan | Source File | Area F1 | Area F2 | Area F3 | Area F4 | Area F5 | Area F6 | Area F7 | Area F8 | Area F9 | Area F10 | #Feature | #Feature F1 | #Feature F2 | #Feature F3 | #Feature F4 | #Feature F5 | #Feature F6 | #Feature F7 | #Feature F8 | #Feature F9 | #Feature F10 | Start | End | PTM |
| --- | --- | --- | --- | --- | --- | --- | --- | --- | --- | --- | --- | --- | --- | --- | --- | --- | --- | --- | --- | --- | --- | --- | --- | --- | --- | --- | --- | --- | --- | --- | --- | --- | --- | --- | --- |
| R.GC(+57.02)IDVC(+57.02)PKSSLLVK.Y | N | 68.52 | 1574.8160 | 14 | -3.9 | 788.4122 | 2 | 11.71 | 8 | F8:2538 | 05112019\_RID\_1313\_NaNaRj\_F8.raw |  |  |  |  |  |  | 0 | 3.04E6 |  |  | 3 | 0 | 0 | 0 | 0 | 0 | 0 | 1 | 2 | 0 | 0 | 58 | 71 | Carbamidomethylation |
| K.TC(+57.02)PAGKNLC(+57.02)YK.M | N | 59.00 | 1310.6111 | 11 | 0.7 | 437.8779 | 3 | 10.68 | 8 | F8:1633 | 05112019\_RID\_1313\_NaNaRj\_F8.raw |  |  |  |  |  |  |  | 2E5 |  |  | 1 | 0 | 0 | 0 | 0 | 0 | 0 | 0 | 1 | 0 | 0 | 34 | 44 | Carbamidomethylation |
| R.GC(+57.02)IDVC(+57.02)PK.S | N | 56.82 | 947.4205 | 8 | -2.7 | 474.7162 | 2 | 11.15 | 8 | F8:2003 | 05112019\_RID\_1313\_NaNaRj\_F8.raw | 1E5 | 1.9E5 | 1.09E4 | 8.89E4 | 5.11E5 | 3.82E8 |  | 3.84E7 | 5.76E5 | 1.59E4 | 23 | 1 | 1 | 1 | 1 | 1 | 14 | 0 | 2 | 1 | 1 | 58 | 65 | Carbamidomethylation |
| K.M(+15.99)YM(+15.99)VATPK.V | Y | 50.93 | 971.4456 | 8 | -0.2 | 486.7300 | 2 | 11.04 | 9 | F9:1808 | 05112019\_RID\_1313\_NaNaRj\_F9.raw |  |  |  |  |  |  |  |  | 1.71E4 |  | 1 | 0 | 0 | 0 | 0 | 0 | 0 | 0 | 0 | 1 | 0 | 45 | 52 | Oxidation (M) |
| K.SSLLVKYVC(+57.02).C | N | 50.14 | 1067.5685 | 9 | -0.1 | 534.7914 | 2 | 12.51 | 8 | F8:3206 | 05112019\_RID\_1313\_NaNaRj\_F8.raw |  |  |  |  |  |  |  | 2.17E5 |  |  | 1 | 0 | 0 | 0 | 0 | 0 | 0 | 0 | 1 | 0 | 0 | 66 | 74 | Carbamidomethylation |
| K.SSLLVKYVC(+57.02)C(+57.02).N | N | 43.03 | 1227.5991 | 10 | 0.0 | 614.8068 | 2 | 12.81 | 8 | F8:3446 | 05112019\_RID\_1313\_NaNaRj\_F8.raw |  |  |  |  |  |  |  | 0 |  |  | 1 | 0 | 0 | 0 | 0 | 0 | 0 | 0 | 1 | 0 | 0 | 66 | 75 | Carbamidomethylation |
| K.MYM(+15.99)VATPK.V | Y | 42.71 | 955.4507 | 8 | -1.0 | 478.7321 | 2 | 11.30 | 9 | F9:2027 | 05112019\_RID\_1313\_NaNaRj\_F9.raw |  |  |  |  |  |  |  |  | 2.2E4 |  | 1 | 0 | 0 | 0 | 0 | 0 | 0 | 0 | 0 | 1 | 0 | 45 | 52 | Oxidation (M) |
| K.LVPLFYK.T | N | 40.17 | 878.5266 | 7 | -0.1 | 440.2705 | 2 | 16.00 | 9 | F9:3716 | 05112019\_RID\_1313\_NaNaRj\_F9.raw |  |  |  |  |  |  |  |  | 4.25E5 |  | 1 | 0 | 0 | 0 | 0 | 0 | 0 | 0 | 0 | 1 | 0 | 27 | 33 |  |
| total 8 peptides |  |  |  |  |  |  |  |  |  |  |  |  |  |  |  |  |  |  |  |  |  |  |  |  |  |  |  |  |  |  |  |  |  |  |  |
| --- | --- | --- | --- | --- | --- | --- | --- | --- | --- | --- | --- | --- | --- | --- | --- | --- | --- | --- | --- | --- | --- | --- | --- | --- | --- | --- | --- | --- | --- | --- | --- | --- | --- | --- | --- |

A0A0U5AUY6|3SA9\_NAJNA

back to list

  

| Protein Coverage
| Supporting Peptides
|

Protein Coverage:

Supporting Peptides:

| Peptide | Uniq | -10lgP | Mass | Length | ppm | m/z | z | RT | Fraction | Scan | Source File | Area F1 | Area F2 | Area F3 | Area F4 | Area F5 | Area F6 | Area F7 | Area F8 | Area F9 | Area F10 | #Feature | #Feature F1 | #Feature F2 | #Feature F3 | #Feature F4 | #Feature F5 | #Feature F6 | #Feature F7 | #Feature F8 | #Feature F9 | #Feature F10 | Start | End | PTM |
| --- | --- | --- | --- | --- | --- | --- | --- | --- | --- | --- | --- | --- | --- | --- | --- | --- | --- | --- | --- | --- | --- | --- | --- | --- | --- | --- | --- | --- | --- | --- | --- | --- | --- | --- | --- |
| R.GC(+57.02)IDVC(+57.02)PKSSLLVK.Y | N | 68.52 | 1574.8160 | 14 | -3.9 | 788.4122 | 2 | 11.71 | 8 | F8:2538 | 05112019\_RID\_1313\_NaNaRj\_F8.raw |  |  |  |  |  |  | 0 | 3.04E6 |  |  | 3 | 0 | 0 | 0 | 0 | 0 | 0 | 1 | 2 | 0 | 0 | 49 | 62 | Carbamidomethylation |
| K.TC(+57.02)PAGKNLC(+57.02)YK.M | N | 59.00 | 1310.6111 | 11 | 0.7 | 437.8779 | 3 | 10.68 | 8 | F8:1633 | 05112019\_RID\_1313\_NaNaRj\_F8.raw |  |  |  |  |  |  |  | 2E5 |  |  | 1 | 0 | 0 | 0 | 0 | 0 | 0 | 0 | 1 | 0 | 0 | 25 | 35 | Carbamidomethylation |
| R.GC(+57.02)IDVC(+57.02)PK.S | N | 56.82 | 947.4205 | 8 | -2.7 | 474.7162 | 2 | 11.15 | 8 | F8:2003 | 05112019\_RID\_1313\_NaNaRj\_F8.raw | 1E5 | 1.9E5 | 1.09E4 | 8.89E4 | 5.11E5 | 3.82E8 |  | 3.84E7 | 5.76E5 | 1.59E4 | 23 | 1 | 1 | 1 | 1 | 1 | 14 | 0 | 2 | 1 | 1 | 49 | 56 | Carbamidomethylation |
| K.M(+15.99)YM(+15.99)VATPK.V | Y | 50.93 | 971.4456 | 8 | -0.2 | 486.7300 | 2 | 11.04 | 9 | F9:1808 | 05112019\_RID\_1313\_NaNaRj\_F9.raw |  |  |  |  |  |  |  |  | 1.71E4 |  | 1 | 0 | 0 | 0 | 0 | 0 | 0 | 0 | 0 | 1 | 0 | 36 | 43 | Oxidation (M) |
| K.SSLLVKYVC(+57.02).C | N | 50.14 | 1067.5685 | 9 | -0.1 | 534.7914 | 2 | 12.51 | 8 | F8:3206 | 05112019\_RID\_1313\_NaNaRj\_F8.raw |  |  |  |  |  |  |  | 2.17E5 |  |  | 1 | 0 | 0 | 0 | 0 | 0 | 0 | 0 | 1 | 0 | 0 | 57 | 65 | Carbamidomethylation |
| K.SSLLVKYVC(+57.02)C(+57.02).N | N | 43.03 | 1227.5991 | 10 | 0.0 | 614.8068 | 2 | 12.81 | 8 | F8:3446 | 05112019\_RID\_1313\_NaNaRj\_F8.raw |  |  |  |  |  |  |  | 0 |  |  | 1 | 0 | 0 | 0 | 0 | 0 | 0 | 0 | 1 | 0 | 0 | 57 | 66 | Carbamidomethylation |
| K.MYM(+15.99)VATPK.V | Y | 42.71 | 955.4507 | 8 | -1.0 | 478.7321 | 2 | 11.30 | 9 | F9:2027 | 05112019\_RID\_1313\_NaNaRj\_F9.raw |  |  |  |  |  |  |  |  | 2.2E4 |  | 1 | 0 | 0 | 0 | 0 | 0 | 0 | 0 | 0 | 1 | 0 | 36 | 43 | Oxidation (M) |
| K.LVPLFYK.T | N | 40.17 | 878.5266 | 7 | -0.1 | 440.2705 | 2 | 16.00 | 9 | F9:3716 | 05112019\_RID\_1313\_NaNaRj\_F9.raw |  |  |  |  |  |  |  |  | 4.25E5 |  | 1 | 0 | 0 | 0 | 0 | 0 | 0 | 0 | 0 | 1 | 0 | 18 | 24 |  |
| total 8 peptides |  |  |  |  |  |  |  |  |  |  |  |  |  |  |  |  |  |  |  |  |  |  |  |  |  |  |  |  |  |  |  |  |  |  |  |
| --- | --- | --- | --- | --- | --- | --- | --- | --- | --- | --- | --- | --- | --- | --- | --- | --- | --- | --- | --- | --- | --- | --- | --- | --- | --- | --- | --- | --- | --- | --- | --- | --- | --- | --- | --- |

Q9PST4|3SA2A\_NAJSP

back to list

  

| Protein Coverage
| Supporting Peptides
|

Protein Coverage:

Supporting Peptides:

| Peptide | Uniq | -10lgP | Mass | Length | ppm | m/z | z | RT | Fraction | Scan | Source File | Area F1 | Area F2 | Area F3 | Area F4 | Area F5 | Area F6 | Area F7 | Area F8 | Area F9 | Area F10 | #Feature | #Feature F1 | #Feature F2 | #Feature F3 | #Feature F4 | #Feature F5 | #Feature F6 | #Feature F7 | #Feature F8 | #Feature F9 | #Feature F10 | Start | End | PTM |
| --- | --- | --- | --- | --- | --- | --- | --- | --- | --- | --- | --- | --- | --- | --- | --- | --- | --- | --- | --- | --- | --- | --- | --- | --- | --- | --- | --- | --- | --- | --- | --- | --- | --- | --- | --- |
| R.GC(+57.02)IDVC(+57.02)PKSSLLVK.Y | N | 68.52 | 1574.8160 | 14 | -3.9 | 788.4122 | 2 | 11.71 | 8 | F8:2538 | 05112019\_RID\_1313\_NaNaRj\_F8.raw |  |  |  |  |  |  | 0 | 3.04E6 |  |  | 3 | 0 | 0 | 0 | 0 | 0 | 0 | 1 | 2 | 0 | 0 | 58 | 71 | Carbamidomethylation |
| K.TC(+57.02)PAGKNLC(+57.02)YK.M | N | 59.00 | 1310.6111 | 11 | 0.7 | 437.8779 | 3 | 10.68 | 8 | F8:1633 | 05112019\_RID\_1313\_NaNaRj\_F8.raw |  |  |  |  |  |  |  | 2E5 |  |  | 1 | 0 | 0 | 0 | 0 | 0 | 0 | 0 | 1 | 0 | 0 | 34 | 44 | Carbamidomethylation |
| R.GC(+57.02)IDVC(+57.02)PK.S | N | 56.82 | 947.4205 | 8 | -2.7 | 474.7162 | 2 | 11.15 | 8 | F8:2003 | 05112019\_RID\_1313\_NaNaRj\_F8.raw | 1E5 | 1.9E5 | 1.09E4 | 8.89E4 | 5.11E5 | 3.82E8 |  | 3.84E7 | 5.76E5 | 1.59E4 | 23 | 1 | 1 | 1 | 1 | 1 | 14 | 0 | 2 | 1 | 1 | 58 | 65 | Carbamidomethylation |
| K.M(+15.99)YM(+15.99)VATPK.V | Y | 50.93 | 971.4456 | 8 | -0.2 | 486.7300 | 2 | 11.04 | 9 | F9:1808 | 05112019\_RID\_1313\_NaNaRj\_F9.raw |  |  |  |  |  |  |  |  | 1.71E4 |  | 1 | 0 | 0 | 0 | 0 | 0 | 0 | 0 | 0 | 1 | 0 | 45 | 52 | Oxidation (M) |
| K.SSLLVKYVC(+57.02).C | N | 50.14 | 1067.5685 | 9 | -0.1 | 534.7914 | 2 | 12.51 | 8 | F8:3206 | 05112019\_RID\_1313\_NaNaRj\_F8.raw |  |  |  |  |  |  |  | 2.17E5 |  |  | 1 | 0 | 0 | 0 | 0 | 0 | 0 | 0 | 1 | 0 | 0 | 66 | 74 | Carbamidomethylation |
| K.SSLLVKYVC(+57.02)C(+57.02).N | N | 43.03 | 1227.5991 | 10 | 0.0 | 614.8068 | 2 | 12.81 | 8 | F8:3446 | 05112019\_RID\_1313\_NaNaRj\_F8.raw |  |  |  |  |  |  |  | 0 |  |  | 1 | 0 | 0 | 0 | 0 | 0 | 0 | 0 | 1 | 0 | 0 | 66 | 75 | Carbamidomethylation |
| K.MYM(+15.99)VATPK.V | Y | 42.71 | 955.4507 | 8 | -1.0 | 478.7321 | 2 | 11.30 | 9 | F9:2027 | 05112019\_RID\_1313\_NaNaRj\_F9.raw |  |  |  |  |  |  |  |  | 2.2E4 |  | 1 | 0 | 0 | 0 | 0 | 0 | 0 | 0 | 0 | 1 | 0 | 45 | 52 | Oxidation (M) |
| K.LVPLFYK.T | N | 40.17 | 878.5266 | 7 | -0.1 | 440.2705 | 2 | 16.00 | 9 | F9:3716 | 05112019\_RID\_1313\_NaNaRj\_F9.raw |  |  |  |  |  |  |  |  | 4.25E5 |  | 1 | 0 | 0 | 0 | 0 | 0 | 0 | 0 | 0 | 1 | 0 | 27 | 33 |  |
| total 8 peptides |  |  |  |  |  |  |  |  |  |  |  |  |  |  |  |  |  |  |  |  |  |  |  |  |  |  |  |  |  |  |  |  |  |  |  |
| --- | --- | --- | --- | --- | --- | --- | --- | --- | --- | --- | --- | --- | --- | --- | --- | --- | --- | --- | --- | --- | --- | --- | --- | --- | --- | --- | --- | --- | --- | --- | --- | --- | --- | --- | --- |

P02091|HBB1\_RAT

back to list

  

| Protein Coverage
| Supporting Peptides
|

Protein Coverage:

Supporting Peptides:

| Peptide | Uniq | -10lgP | Mass | Length | ppm | m/z | z | RT | Fraction | Scan | Source File | Area F1 | Area F2 | Area F3 | Area F4 | Area F5 | Area F6 | Area F7 | Area F8 | Area F9 | Area F10 | #Feature | #Feature F1 | #Feature F2 | #Feature F3 | #Feature F4 | #Feature F5 | #Feature F6 | #Feature F7 | #Feature F8 | #Feature F9 | #Feature F10 | Start | End | PTM |
| --- | --- | --- | --- | --- | --- | --- | --- | --- | --- | --- | --- | --- | --- | --- | --- | --- | --- | --- | --- | --- | --- | --- | --- | --- | --- | --- | --- | --- | --- | --- | --- | --- | --- | --- | --- |
| K.VVAGVASALAHK.Y | Y | 68.98 | 1121.6556 | 12 | 0.2 | 561.8352 | 2 | 11.36 | 1 | F1:2155 | 05112019\_RID\_1313\_NaNaRj\_F1.raw | 1.29E4 |  |  |  |  |  |  |  |  | 1.42E3 | 2 | 1 | 0 | 0 | 0 | 0 | 0 | 0 | 0 | 0 | 1 | 134 | 145 |  |
| K.VINAFNDGLK.H | Y | 62.21 | 1089.5818 | 10 | 0.3 | 545.7983 | 2 | 11.90 | 10 | F10:2360 | 05112019\_RID\_1313\_NaNaRj\_F10.raw | 8.86E4 |  | 2.1E4 |  | 1.21E4 |  |  |  | 1.5E4 | 1.64E4 | 5 | 1 | 0 | 1 | 0 | 1 | 0 | 0 | 0 | 1 | 1 | 68 | 77 |  |
| K.AAVNGLWGK.V | Y | 53.08 | 914.4974 | 9 | -0.4 | 458.2558 | 2 | 11.84 | 10 | F10:2330 | 05112019\_RID\_1313\_NaNaRj\_F10.raw |  |  |  |  |  |  |  |  |  | 5.43E3 | 1 | 0 | 0 | 0 | 0 | 0 | 0 | 0 | 0 | 0 | 1 | 10 | 18 |  |
| K.EFTPC(+57.02)AQAAFQK.V | Y | 53.02 | 1396.6445 | 12 | 1.1 | 699.3303 | 2 | 11.80 | 1 | F1:2558 | 05112019\_RID\_1313\_NaNaRj\_F1.raw | 7.89E3 |  |  |  |  |  |  |  |  |  | 1 | 1 | 0 | 0 | 0 | 0 | 0 | 0 | 0 | 0 | 0 | 122 | 133 | Carbamidomethylation |
| K.VNPDDVGGEALGR.L | Y | 43.62 | 1297.6262 | 13 | 1.4 | 649.8213 | 2 | 11.56 | 9 | F9:2256 | 05112019\_RID\_1313\_NaNaRj\_F9.raw |  |  |  |  |  |  |  |  | 9.39E3 |  | 1 | 0 | 0 | 0 | 0 | 0 | 0 | 0 | 0 | 1 | 0 | 19 | 31 |  |
| K.LHVDPENFR.L | Y | 43.20 | 1125.5566 | 9 | -0.3 | 376.1927 | 3 | 11.50 | 1 | F1:2281 | 05112019\_RID\_1313\_NaNaRj\_F1.raw | 3.9E3 |  |  |  |  |  |  |  |  |  | 1 | 1 | 0 | 0 | 0 | 0 | 0 | 0 | 0 | 0 | 0 | 97 | 105 |  |
| R.LLVVYPWTQR.Y | Y | 42.23 | 1273.7183 | 10 | 0.1 | 637.8665 | 2 | 23.41 | 9 | F9:5133 | 05112019\_RID\_1313\_NaNaRj\_F9.raw |  |  |  |  |  |  |  |  | 2.54E4 |  | 1 | 0 | 0 | 0 | 0 | 0 | 0 | 0 | 0 | 1 | 0 | 32 | 41 |  |
| M.VHLTDAEK.A | Y | 41.74 | 911.4712 | 8 | -0.6 | 456.7426 | 2 | 10.77 | 1 | F1:1618 | 05112019\_RID\_1313\_NaNaRj\_F1.raw | 3.43E4 |  |  |  |  |  |  |  |  |  | 1 | 1 | 0 | 0 | 0 | 0 | 0 | 0 | 0 | 0 | 0 | 2 | 9 |  |
| total 8 peptides |  |  |  |  |  |  |  |  |  |  |  |  |  |  |  |  |  |  |  |  |  |  |  |  |  |  |  |  |  |  |  |  |  |  |  |
| --- | --- | --- | --- | --- | --- | --- | --- | --- | --- | --- | --- | --- | --- | --- | --- | --- | --- | --- | --- | --- | --- | --- | --- | --- | --- | --- | --- | --- | --- | --- | --- | --- | --- | --- | --- |

A8QL58|OXLA\_NAJAT

back to list

  

| Protein Coverage
| Supporting Peptides
|

Protein Coverage:

Supporting Peptides:

| Peptide | Uniq | -10lgP | Mass | Length | ppm | m/z | z | RT | Fraction | Scan | Source File | Area F1 | Area F2 | Area F3 | Area F4 | Area F5 | Area F6 | Area F7 | Area F8 | Area F9 | Area F10 | #Feature | #Feature F1 | #Feature F2 | #Feature F3 | #Feature F4 | #Feature F5 | #Feature F6 | #Feature F7 | #Feature F8 | #Feature F9 | #Feature F10 | Start | End | PTM |
| --- | --- | --- | --- | --- | --- | --- | --- | --- | --- | --- | --- | --- | --- | --- | --- | --- | --- | --- | --- | --- | --- | --- | --- | --- | --- | --- | --- | --- | --- | --- | --- | --- | --- | --- | --- |
| K.TC(+57.02)ADIVINDLSLIHDLPK.R | Y | 79.47 | 2036.0612 | 18 | 0.4 | 679.6946 | 3 | 34.26 | 7 | F7:9393 | 05112019\_RID\_1313\_NaNaRj\_F7.raw |  |  |  |  |  |  | 5.08E4 | 5.97E4 |  |  | 2 | 0 | 0 | 0 | 0 | 0 | 0 | 1 | 1 | 0 | 0 | 405 | 422 | Carbamidomethylation |
| A.DDSDFFQALDTK.T | Y | 59.76 | 1400.6095 | 12 | 0.2 | 701.3121 | 2 | 24.92 | 7 | F7:7256 | 05112019\_RID\_1313\_NaNaRj\_F7.raw |  |  |  |  |  |  | 3.5E4 |  |  |  | 1 | 0 | 0 | 0 | 0 | 0 | 0 | 1 | 0 | 0 | 0 | 393 | 404 |  |
| K.TFVTADYVIVC(+57.02)STSR.A | Y | 48.49 | 1717.8345 | 15 | 0.5 | 859.9249 | 2 | 17.79 | 6 | F6:6665 | 05112019\_RID\_1313\_NaNaRj\_F6.raw |  |  |  |  |  | 8.95E4 |  |  |  |  | 1 | 0 | 0 | 0 | 0 | 0 | 1 | 0 | 0 | 0 | 0 | 300 | 314 | Carbamidomethylation |
| R.IYFEPPLPPK.K | N | 47.13 | 1199.6589 | 10 | 0.7 | 600.8372 | 2 | 17.27 | 4 | F4:6303 | 05112019\_RID\_1313\_NaNaRj\_F4.raw |  |  |  | 2.87E4 |  |  |  |  |  |  | 1 | 0 | 0 | 0 | 1 | 0 | 0 | 0 | 0 | 0 | 0 | 319 | 328 |  |
| Y.VLADDSDFFQALDTK.T | Y | 44.71 | 1683.7991 | 15 | 1.7 | 842.9083 | 2 | 28.15 | 9 | F9:5933 | 05112019\_RID\_1313\_NaNaRj\_F9.raw |  |  |  |  |  |  |  |  | 2.03E4 |  | 1 | 0 | 0 | 0 | 0 | 0 | 0 | 0 | 0 | 1 | 0 | 390 | 404 |  |
| total 5 peptides |  |  |  |  |  |  |  |  |  |  |  |  |  |  |  |  |  |  |  |  |  |  |  |  |  |  |  |  |  |  |  |  |  |  |  |
| --- | --- | --- | --- | --- | --- | --- | --- | --- | --- | --- | --- | --- | --- | --- | --- | --- | --- | --- | --- | --- | --- | --- | --- | --- | --- | --- | --- | --- | --- | --- | --- | --- | --- | --- | --- |

Q9PVK7|VM3\_NAJKA

back to list

  

| Protein Coverage
| Supporting Peptides
|

Protein Coverage:

Supporting Peptides:

| Peptide | Uniq | -10lgP | Mass | Length | ppm | m/z | z | RT | Fraction | Scan | Source File | Area F1 | Area F2 | Area F3 | Area F4 | Area F5 | Area F6 | Area F7 | Area F8 | Area F9 | Area F10 | #Feature | #Feature F1 | #Feature F2 | #Feature F3 | #Feature F4 | #Feature F5 | #Feature F6 | #Feature F7 | #Feature F8 | #Feature F9 | #Feature F10 | Start | End | PTM |
| --- | --- | --- | --- | --- | --- | --- | --- | --- | --- | --- | --- | --- | --- | --- | --- | --- | --- | --- | --- | --- | --- | --- | --- | --- | --- | --- | --- | --- | --- | --- | --- | --- | --- | --- | --- |
| R.NDNAQLLTGIDFNGTPVGLAYIGSIC(+57.02)NPK.T | Y | 70.21 | 3061.5178 | 29 | 0.6 | 1021.5139 | 3 | 49.39 | 6 | F6:19978 | 05112019\_RID\_1313\_NaNaRj\_F6.raw |  |  |  |  |  | 1.35E5 |  |  |  |  | 1 | 0 | 0 | 0 | 0 | 0 | 1 | 0 | 0 | 0 | 0 | 279 | 307 | Carbamidomethylation |
| K.ATLDLFGEWR.E | Y | 65.12 | 1206.6033 | 10 | 0.0 | 604.3089 | 2 | 30.54 | 6 | F6:13690 | 05112019\_RID\_1313\_NaNaRj\_F6.raw |  |  |  |  |  | 1.12E5 | 1.31E5 | 4.18E4 |  |  | 3 | 0 | 0 | 0 | 0 | 0 | 1 | 1 | 1 | 0 | 0 | 260 | 269 |  |
| L.LTGIDFNGTPVGLAYIGSIC(+57.02)NPK.T | Y | 57.50 | 2406.2253 | 23 | 0.5 | 1204.1206 | 2 | 40.76 | 6 | F6:17530 | 05112019\_RID\_1313\_NaNaRj\_F6.raw |  |  |  |  |  | 4.42E5 |  |  |  |  | 2 | 0 | 0 | 0 | 0 | 0 | 2 | 0 | 0 | 0 | 0 | 285 | 307 | Carbamidomethylation |
| K.DDC(+57.02)DLPELC(+57.02)TGQSAEC(+57.02)PTDVFQR.N | Y | 53.38 | 2712.1101 | 23 | 0.2 | 1357.0626 | 2 | 22.83 | 6 | F6:9551 | 05112019\_RID\_1313\_NaNaRj\_F6.raw |  |  |  |  |  | 2.77E5 | 1.03E5 |  |  |  | 2 | 0 | 0 | 0 | 0 | 0 | 1 | 1 | 0 | 0 | 0 | 459 | 481 | Carbamidomethylation |
| G.TPVGLAYIGSIC(+57.02)NPK.T | Y | 40.49 | 1588.8282 | 15 | 2.6 | 795.4235 | 2 | 18.05 | 6 | F6:6899 | 05112019\_RID\_1313\_NaNaRj\_F6.raw |  |  |  |  |  | 1.56E4 |  |  |  |  | 1 | 0 | 0 | 0 | 0 | 0 | 1 | 0 | 0 | 0 | 0 | 293 | 307 | Carbamidomethylation |
| total 5 peptides |  |  |  |  |  |  |  |  |  |  |  |  |  |  |  |  |  |  |  |  |  |  |  |  |  |  |  |  |  |  |  |  |  |  |  |
| --- | --- | --- | --- | --- | --- | --- | --- | --- | --- | --- | --- | --- | --- | --- | --- | --- | --- | --- | --- | --- | --- | --- | --- | --- | --- | --- | --- | --- | --- | --- | --- | --- | --- | --- | --- |

Q91132|VCO3\_NAJKA

back to list

  

| Protein Coverage
| Supporting Peptides
|

Protein Coverage:

Supporting Peptides:

| Peptide | Uniq | -10lgP | Mass | Length | ppm | m/z | z | RT | Fraction | Scan | Source File | Area F1 | Area F2 | Area F3 | Area F4 | Area F5 | Area F6 | Area F7 | Area F8 | Area F9 | Area F10 | #Feature | #Feature F1 | #Feature F2 | #Feature F3 | #Feature F4 | #Feature F5 | #Feature F6 | #Feature F7 | #Feature F8 | #Feature F9 | #Feature F10 | Start | End | PTM |
| --- | --- | --- | --- | --- | --- | --- | --- | --- | --- | --- | --- | --- | --- | --- | --- | --- | --- | --- | --- | --- | --- | --- | --- | --- | --- | --- | --- | --- | --- | --- | --- | --- | --- | --- | --- |
| K.SDFGC(+57.02)TAGSGQNNLGVFEDAGLALTTSTNLNTK.Q | Y | 63.32 | 3359.5576 | 33 | 0.3 | 1120.8601 | 3 | 39.09 | 6 | F6:17045 | 05112019\_RID\_1313\_NaNaRj\_F6.raw |  |  |  |  |  | 2.69E5 |  |  |  |  | 1 | 0 | 0 | 0 | 0 | 0 | 1 | 0 | 0 | 0 | 0 | 605 | 637 | Carbamidomethylation |
| R.QNQYVVVQVTGPQVR.L | Y | 58.08 | 1713.9163 | 15 | 1.2 | 857.9664 | 2 | 12.17 | 8 | F8:2927 | 05112019\_RID\_1313\_NaNaRj\_F8.raw |  |  |  |  |  |  |  | 0 | 1.34E4 |  | 2 | 0 | 0 | 0 | 0 | 0 | 0 | 0 | 1 | 1 | 0 | 100 | 114 |  |
| G.ALYTLITPAVLR.T | Y | 55.59 | 1329.8020 | 12 | 0.8 | 665.9088 | 2 | 29.71 | 8 | F8:11125 | 05112019\_RID\_1313\_NaNaRj\_F8.raw |  |  |  |  |  |  |  | 1.17E5 |  |  | 1 | 0 | 0 | 0 | 0 | 0 | 0 | 0 | 1 | 0 | 0 | 23 | 34 |  |
| K.IIIQGDPVAQIIEN.S | Y | 42.75 | 1521.8402 | 14 | 1.3 | 761.9283 | 2 | 35.20 | 5 | F5:7510 | 05112019\_RID\_1313\_NaNaRj\_F5.raw |  |  |  |  | 1.25E4 |  |  |  |  |  | 1 | 0 | 0 | 0 | 0 | 1 | 0 | 0 | 0 | 0 | 0 | 963 | 976 |  |
| K.GIYTPGSPVLYR.V | Y | 41.74 | 1321.7030 | 12 | 0.4 | 661.8591 | 2 | 12.77 | 9 | F9:2986 | 05112019\_RID\_1313\_NaNaRj\_F9.raw |  |  |  |  |  |  |  |  | 1.75E4 |  | 1 | 0 | 0 | 0 | 0 | 0 | 0 | 0 | 0 | 1 | 0 | 135 | 146 |  |
| total 5 peptides |  |  |  |  |  |  |  |  |  |  |  |  |  |  |  |  |  |  |  |  |  |  |  |  |  |  |  |  |  |  |  |  |  |  |  |
| --- | --- | --- | --- | --- | --- | --- | --- | --- | --- | --- | --- | --- | --- | --- | --- | --- | --- | --- | --- | --- | --- | --- | --- | --- | --- | --- | --- | --- | --- | --- | --- | --- | --- | --- | --- |

P82463|3SUC2\_NAJKA

back to list

  

| Protein Coverage
| Supporting Peptides
|

Protein Coverage:

Supporting Peptides:

| Peptide | Uniq | -10lgP | Mass | Length | ppm | m/z | z | RT | Fraction | Scan | Source File | Area F1 | Area F2 | Area F3 | Area F4 | Area F5 | Area F6 | Area F7 | Area F8 | Area F9 | Area F10 | #Feature | #Feature F1 | #Feature F2 | #Feature F3 | #Feature F4 | #Feature F5 | #Feature F6 | #Feature F7 | #Feature F8 | #Feature F9 | #Feature F10 | Start | End | PTM |
| --- | --- | --- | --- | --- | --- | --- | --- | --- | --- | --- | --- | --- | --- | --- | --- | --- | --- | --- | --- | --- | --- | --- | --- | --- | --- | --- | --- | --- | --- | --- | --- | --- | --- | --- | --- |
| R.GC(+57.02)AATC(+57.02)PIAENR.D | Y | 78.31 | 1318.5758 | 12 | 0.8 | 660.2957 | 2 | 11.02 | 5 | F5:1873 | 05112019\_RID\_1313\_NaNaRj\_F5.raw |  |  |  |  | 6.65E5 |  |  |  |  |  | 1 | 0 | 0 | 0 | 0 | 1 | 0 | 0 | 0 | 0 | 0 | 41 | 52 | Carbamidomethylation |
| R.DVIEC(+57.02)C(+57.02)STDK.C | Y | 72.21 | 1225.4955 | 10 | 0.8 | 613.7555 | 2 | 11.02 | 5 | F5:1883 | 05112019\_RID\_1313\_NaNaRj\_F5.raw |  |  |  |  | 3.64E5 |  |  |  |  |  | 1 | 0 | 0 | 0 | 0 | 1 | 0 | 0 | 0 | 0 | 0 | 53 | 62 | Carbamidomethylation |
| K.SIFGVTTEDC(+57.02)PDGQNLC(+57.02)FK.R | Y | 64.11 | 2186.9612 | 19 | 0.7 | 1094.4886 | 2 | 21.40 | 6 | F6:8874 | 05112019\_RID\_1313\_NaNaRj\_F6.raw |  |  |  |  | 3E5 | 1.51E6 | 6.99E4 | 2.56E4 |  |  | 7 | 0 | 0 | 0 | 0 | 2 | 3 | 1 | 1 | 0 | 0 | 8 | 26 | Carbamidomethylation |
| total 3 peptides |  |  |  |  |  |  |  |  |  |  |  |  |  |  |  |  |  |  |  |  |  |  |  |  |  |  |  |  |  |  |  |  |  |  |  |
| --- | --- | --- | --- | --- | --- | --- | --- | --- | --- | --- | --- | --- | --- | --- | --- | --- | --- | --- | --- | --- | --- | --- | --- | --- | --- | --- | --- | --- | --- | --- | --- | --- | --- | --- | --- |

Q7Z794|K2C1B\_HUMAN

back to list

  

| Protein Coverage
| Supporting Peptides
|

Protein Coverage:

Supporting Peptides:

| Peptide | Uniq | -10lgP | Mass | Length | ppm | m/z | z | RT | Fraction | Scan | Source File | Area F1 | Area F2 | Area F3 | Area F4 | Area F5 | Area F6 | Area F7 | Area F8 | Area F9 | Area F10 | #Feature | #Feature F1 | #Feature F2 | #Feature F3 | #Feature F4 | #Feature F5 | #Feature F6 | #Feature F7 | #Feature F8 | #Feature F9 | #Feature F10 | Start | End | PTM |
| --- | --- | --- | --- | --- | --- | --- | --- | --- | --- | --- | --- | --- | --- | --- | --- | --- | --- | --- | --- | --- | --- | --- | --- | --- | --- | --- | --- | --- | --- | --- | --- | --- | --- | --- | --- |
| R.FLEQQNQVLQTK.W | N | 88.32 | 1474.7780 | 12 | 0.9 | 738.3969 | 2 | 11.66 | 3 | F3:2423 | 05112019\_RID\_1313\_NaNaRj\_F3.raw | 3.29E5 | 6.5E6 | 2.66E6 | 9.27E5 | 3.5E6 |  | 4.3E5 | 1.41E5 | 1.56E5 | 4.01E5 | 11 | 1 | 2 | 1 | 1 | 2 | 0 | 1 | 1 | 1 | 1 | 184 | 195 |  |
| K.FASFIDK.V | N | 47.57 | 826.4225 | 7 | 0.0 | 414.2185 | 2 | 39.16 | 2 | F2:12055 | 09012020\_NaNaRj\_F2.raw | 6.35E3 | 4.99E6 |  |  | 4.92E5 |  |  |  | 5.38E3 |  | 4 | 1 | 1 | 0 | 0 | 1 | 0 | 0 | 0 | 1 | 0 | 175 | 181 |  |
| K.LQDLEEALQQSK.E | Y | 46.40 | 1400.7147 | 12 | 0.3 | 701.3649 | 2 | 47.50 | 2 | F2:15609 | 09012020\_NaNaRj\_F2.raw |  | 1.49E4 |  |  |  |  |  |  |  |  | 1 | 0 | 1 | 0 | 0 | 0 | 0 | 0 | 0 | 0 | 0 | 428 | 439 |  |
| R.SMQDVVEDYK.S | Y | 40.28 | 1212.5332 | 10 | -1.2 | 607.2731 | 2 | 39.70 | 2 | F2:12299 | 09012020\_NaNaRj\_F2.raw |  | 2.57E4 |  |  |  |  |  |  |  |  | 1 | 0 | 1 | 0 | 0 | 0 | 0 | 0 | 0 | 0 | 0 | 242 | 251 |  |
| total 4 peptides |  |  |  |  |  |  |  |  |  |  |  |  |  |  |  |  |  |  |  |  |  |  |  |  |  |  |  |  |  |  |  |  |  |  |  |
| --- | --- | --- | --- | --- | --- | --- | --- | --- | --- | --- | --- | --- | --- | --- | --- | --- | --- | --- | --- | --- | --- | --- | --- | --- | --- | --- | --- | --- | --- | --- | --- | --- | --- | --- | --- |

P85520|3NO2\_NAJOX

back to list

  

| Protein Coverage
| Supporting Peptides
|

Protein Coverage:

Supporting Peptides:

| Peptide | Uniq | -10lgP | Mass | Length | ppm | m/z | z | RT | Fraction | Scan | Source File | Area F1 | Area F2 | Area F3 | Area F4 | Area F5 | Area F6 | Area F7 | Area F8 | Area F9 | Area F10 | #Feature | #Feature F1 | #Feature F2 | #Feature F3 | #Feature F4 | #Feature F5 | #Feature F6 | #Feature F7 | #Feature F8 | #Feature F9 | #Feature F10 | Start | End | PTM |
| --- | --- | --- | --- | --- | --- | --- | --- | --- | --- | --- | --- | --- | --- | --- | --- | --- | --- | --- | --- | --- | --- | --- | --- | --- | --- | --- | --- | --- | --- | --- | --- | --- | --- | --- | --- |
| LTC(+57.02)LIC(+57.02)PEKYC(+57.02)NK.V | N | 67.27 | 1697.7939 | 13 | 0.6 | 566.9389 | 3 | 11.61 | 4 | F4:2457 | 05112019\_RID\_1313\_NaNaRj\_F4.raw |  |  |  | 1.02E6 |  |  |  |  |  |  | 2 | 0 | 0 | 0 | 2 | 0 | 0 | 0 | 0 | 0 | 0 | 1 | 13 | Carbamidomethylation |
| LTC(+57.02)LIC(+57.02)PEKYC(+57.02)NKVHTC(+57.02)R.N | N | 63.67 | 2351.1006 | 18 | 0.7 | 471.2277 | 5 | 11.36 | 4 | F4:2200 | 05112019\_RID\_1313\_NaNaRj\_F4.raw |  |  |  | 4.71E5 |  |  |  |  |  |  | 2 | 0 | 0 | 0 | 2 | 0 | 0 | 0 | 0 | 0 | 0 | 1 | 18 | Carbamidomethylation |
| LTC(+57.02)LIC(+57.02)PEK.Y | N | 62.81 | 1132.5620 | 9 | -1.0 | 567.2877 | 2 | 11.81 | 4 | F4:2581 | 05112019\_RID\_1313\_NaNaRj\_F4.raw |  |  |  | 4.45E8 | 3.88E4 | 7.2E5 | 3.64E5 |  |  |  | 29 | 0 | 0 | 0 | 26 | 1 | 1 | 1 | 0 | 0 | 0 | 1 | 9 | Carbamidomethylation |
| R.YIRGC(+57.02)AATC(+57.02)PEAKPR.E | Y | 54.73 | 1748.8450 | 15 | 0.2 | 438.2186 | 4 | 10.68 | 4 | F4:1606 | 05112019\_RID\_1313\_NaNaRj\_F4.raw |  |  |  | 3.49E5 |  |  |  |  |  |  | 1 | 0 | 0 | 0 | 1 | 0 | 0 | 0 | 0 | 0 | 0 | 38 | 52 | Carbamidomethylation |
| R.EIVEC(+57.02)C(+57.02)STDK.C | N | 48.63 | 1239.5111 | 10 | 0.6 | 620.7632 | 2 | 10.79 | 4 | F4:1698 | 05112019\_RID\_1313\_NaNaRj\_F4.raw |  |  |  | 5E4 |  |  |  |  |  |  | 1 | 0 | 0 | 0 | 1 | 0 | 0 | 0 | 0 | 0 | 0 | 53 | 62 | Carbamidomethylation |
| total 5 peptides |  |  |  |  |  |  |  |  |  |  |  |  |  |  |  |  |  |  |  |  |  |  |  |  |  |  |  |  |  |  |  |  |  |  |  |
| --- | --- | --- | --- | --- | --- | --- | --- | --- | --- | --- | --- | --- | --- | --- | --- | --- | --- | --- | --- | --- | --- | --- | --- | --- | --- | --- | --- | --- | --- | --- | --- | --- | --- | --- | --- |

P01452|3SA4\_NAJMO

back to list

  

| Protein Coverage
| Supporting Peptides
|

Protein Coverage:

Supporting Peptides:

| Peptide | Uniq | -10lgP | Mass | Length | ppm | m/z | z | RT | Fraction | Scan | Source File | Area F1 | Area F2 | Area F3 | Area F4 | Area F5 | Area F6 | Area F7 | Area F8 | Area F9 | Area F10 | #Feature | #Feature F1 | #Feature F2 | #Feature F3 | #Feature F4 | #Feature F5 | #Feature F6 | #Feature F7 | #Feature F8 | #Feature F9 | #Feature F10 | Start | End | PTM |
| --- | --- | --- | --- | --- | --- | --- | --- | --- | --- | --- | --- | --- | --- | --- | --- | --- | --- | --- | --- | --- | --- | --- | --- | --- | --- | --- | --- | --- | --- | --- | --- | --- | --- | --- | --- |
| LKC(+57.02)NKLIPIAYK.T | N | 89.61 | 1459.8584 | 12 | 0.4 | 487.6269 | 3 | 11.46 | 6 | F6:2267 | 05112019\_RID\_1313\_NaNaRj\_F6.raw |  |  |  |  |  | 3.07E6 | 7.98E4 |  |  |  | 3 | 0 | 0 | 0 | 0 | 0 | 2 | 1 | 0 | 0 | 0 | 1 | 12 | Carbamidomethylation |
| K.LIPIAYKTC(+57.02)PEGK.N | Y | 52.25 | 1488.8010 | 13 | -0.2 | 497.2742 | 3 | 11.96 | 8 | F8:2756 | 05112019\_RID\_1313\_NaNaRj\_F8.raw |  |  |  |  |  |  | 2.3E4 | 2.15E5 |  |  | 2 | 0 | 0 | 0 | 0 | 0 | 0 | 1 | 1 | 0 | 0 | 6 | 18 | Carbamidomethylation |
| C.NKLIPIAYK.T | N | 40.64 | 1058.6488 | 9 | 0.8 | 530.3321 | 2 | 11.69 | 6 | F6:2403 | 05112019\_RID\_1313\_NaNaRj\_F6.raw |  |  |  |  |  | 3.23E6 |  |  |  |  | 1 | 0 | 0 | 0 | 0 | 0 | 1 | 0 | 0 | 0 | 0 | 4 | 12 |  |
| total 3 peptides |  |  |  |  |  |  |  |  |  |  |  |  |  |  |  |  |  |  |  |  |  |  |  |  |  |  |  |  |  |  |  |  |  |  |  |
| --- | --- | --- | --- | --- | --- | --- | --- | --- | --- | --- | --- | --- | --- | --- | --- | --- | --- | --- | --- | --- | --- | --- | --- | --- | --- | --- | --- | --- | --- | --- | --- | --- | --- | --- | --- |

P01876|IGHA1\_HUMAN

back to list

  

| Protein Coverage
| Supporting Peptides
|

Protein Coverage:

Supporting Peptides:

| Peptide | Uniq | -10lgP | Mass | Length | ppm | m/z | z | RT | Fraction | Scan | Source File | Area F1 | Area F2 | Area F3 | Area F4 | Area F5 | Area F6 | Area F7 | Area F8 | Area F9 | Area F10 | #Feature | #Feature F1 | #Feature F2 | #Feature F3 | #Feature F4 | #Feature F5 | #Feature F6 | #Feature F7 | #Feature F8 | #Feature F9 | #Feature F10 | Start | End | PTM |
| --- | --- | --- | --- | --- | --- | --- | --- | --- | --- | --- | --- | --- | --- | --- | --- | --- | --- | --- | --- | --- | --- | --- | --- | --- | --- | --- | --- | --- | --- | --- | --- | --- | --- | --- | --- |
| K.TPLTATLSK.S | Y | 64.72 | 930.5386 | 9 | 0.4 | 466.2767 | 2 | 29.71 | 2 | F2:8116 | 09012020\_NaNaRj\_F2.raw |  | 2.66E5 |  |  |  |  |  |  |  |  | 1 | 0 | 1 | 0 | 0 | 0 | 0 | 0 | 0 | 0 | 0 | 213 | 221 |  |
| R.WLQGSQELPR.E | Y | 61.62 | 1212.6251 | 10 | 1.3 | 607.3206 | 2 | 40.27 | 2 | F2:12569 | 09012020\_NaNaRj\_F2.raw |  | 5.16E5 |  |  |  |  |  |  |  |  | 1 | 0 | 1 | 0 | 0 | 0 | 0 | 0 | 0 | 0 | 0 | 264 | 273 |  |
| R.QEPSQGTTTFAVTSILR.V | Y | 48.75 | 1834.9425 | 17 | 2.2 | 918.4806 | 2 | 63.74 | 2 | F2:21025 | 09012020\_NaNaRj\_F2.raw |  | 8.07E3 |  |  |  |  |  |  |  |  | 1 | 0 | 1 | 0 | 0 | 0 | 0 | 0 | 0 | 0 | 0 | 283 | 299 |  |
| R.DASGVTFTWTPSSGK.S | Y | 43.43 | 1539.7205 | 15 | 1.1 | 770.8683 | 2 | 57.46 | 2 | F2:19092 | 09012020\_NaNaRj\_F2.raw |  | 7.8E4 |  |  |  |  |  |  |  |  | 1 | 0 | 1 | 0 | 0 | 0 | 0 | 0 | 0 | 0 | 0 | 154 | 168 |  |
| T.WSESGQGVTAR.N | Y | 41.43 | 1176.5524 | 11 | 1.0 | 589.2841 | 2 | 20.73 | 2 | F2:4533 | 09012020\_NaNaRj\_F2.raw |  | 2.13E4 |  |  |  |  |  |  |  |  | 1 | 0 | 1 | 0 | 0 | 0 | 0 | 0 | 0 | 0 | 0 | 41 | 51 |  |
| total 5 peptides |  |  |  |  |  |  |  |  |  |  |  |  |  |  |  |  |  |  |  |  |  |  |  |  |  |  |  |  |  |  |  |  |  |  |  |
| --- | --- | --- | --- | --- | --- | --- | --- | --- | --- | --- | --- | --- | --- | --- | --- | --- | --- | --- | --- | --- | --- | --- | --- | --- | --- | --- | --- | --- | --- | --- | --- | --- | --- | --- | --- |

P20758|IGHA1\_GORGO

back to list

  

| Protein Coverage
| Supporting Peptides
|

Protein Coverage:

Supporting Peptides:

| Peptide | Uniq | -10lgP | Mass | Length | ppm | m/z | z | RT | Fraction | Scan | Source File | Area F1 | Area F2 | Area F3 | Area F4 | Area F5 | Area F6 | Area F7 | Area F8 | Area F9 | Area F10 | #Feature | #Feature F1 | #Feature F2 | #Feature F3 | #Feature F4 | #Feature F5 | #Feature F6 | #Feature F7 | #Feature F8 | #Feature F9 | #Feature F10 | Start | End | PTM |
| --- | --- | --- | --- | --- | --- | --- | --- | --- | --- | --- | --- | --- | --- | --- | --- | --- | --- | --- | --- | --- | --- | --- | --- | --- | --- | --- | --- | --- | --- | --- | --- | --- | --- | --- | --- |
| K.TPLTATLSK.S | Y | 64.72 | 930.5386 | 9 | 0.4 | 466.2767 | 2 | 29.71 | 2 | F2:8116 | 09012020\_NaNaRj\_F2.raw |  | 2.66E5 |  |  |  |  |  |  |  |  | 1 | 0 | 1 | 0 | 0 | 0 | 0 | 0 | 0 | 0 | 0 | 213 | 221 |  |
| R.WLQGSQELPR.E | Y | 61.62 | 1212.6251 | 10 | 1.3 | 607.3206 | 2 | 40.27 | 2 | F2:12569 | 09012020\_NaNaRj\_F2.raw |  | 5.16E5 |  |  |  |  |  |  |  |  | 1 | 0 | 1 | 0 | 0 | 0 | 0 | 0 | 0 | 0 | 0 | 264 | 273 |  |
| R.QEPSQGTTTFAVTSILR.V | Y | 48.75 | 1834.9425 | 17 | 2.2 | 918.4806 | 2 | 63.74 | 2 | F2:21025 | 09012020\_NaNaRj\_F2.raw |  | 8.07E3 |  |  |  |  |  |  |  |  | 1 | 0 | 1 | 0 | 0 | 0 | 0 | 0 | 0 | 0 | 0 | 283 | 299 |  |
| R.DASGVTFTWTPSSGK.S | Y | 43.43 | 1539.7205 | 15 | 1.1 | 770.8683 | 2 | 57.46 | 2 | F2:19092 | 09012020\_NaNaRj\_F2.raw |  | 7.8E4 |  |  |  |  |  |  |  |  | 1 | 0 | 1 | 0 | 0 | 0 | 0 | 0 | 0 | 0 | 0 | 154 | 168 |  |
| T.WSESGQGVTAR.N | Y | 41.43 | 1176.5524 | 11 | 1.0 | 589.2841 | 2 | 20.73 | 2 | F2:4533 | 09012020\_NaNaRj\_F2.raw |  | 2.13E4 |  |  |  |  |  |  |  |  | 1 | 0 | 1 | 0 | 0 | 0 | 0 | 0 | 0 | 0 | 0 | 41 | 51 |  |
| total 5 peptides |  |  |  |  |  |  |  |  |  |  |  |  |  |  |  |  |  |  |  |  |  |  |  |  |  |  |  |  |  |  |  |  |  |  |  |
| --- | --- | --- | --- | --- | --- | --- | --- | --- | --- | --- | --- | --- | --- | --- | --- | --- | --- | --- | --- | --- | --- | --- | --- | --- | --- | --- | --- | --- | --- | --- | --- | --- | --- | --- | --- |

P25676|3SO61\_HEMHA

back to list

  

| Protein Coverage
| Supporting Peptides
|

Protein Coverage:

Supporting Peptides:

| Peptide | Uniq | -10lgP | Mass | Length | ppm | m/z | z | RT | Fraction | Scan | Source File | Area F1 | Area F2 | Area F3 | Area F4 | Area F5 | Area F6 | Area F7 | Area F8 | Area F9 | Area F10 | #Feature | #Feature F1 | #Feature F2 | #Feature F3 | #Feature F4 | #Feature F5 | #Feature F6 | #Feature F7 | #Feature F8 | #Feature F9 | #Feature F10 | Start | End | PTM |
| --- | --- | --- | --- | --- | --- | --- | --- | --- | --- | --- | --- | --- | --- | --- | --- | --- | --- | --- | --- | --- | --- | --- | --- | --- | --- | --- | --- | --- | --- | --- | --- | --- | --- | --- | --- |
| K.GC(+57.02)VASC(+57.02)PSFESHYK.F | Y | 83.59 | 1627.6759 | 14 | 0.9 | 814.8459 | 2 | 11.21 | 5 | F5:2045 | 05112019\_RID\_1313\_NaNaRj\_F5.raw |  |  |  | 4.57E5 | 7.91E6 | 0 |  |  |  |  | 4 | 0 | 0 | 0 | 1 | 2 | 1 | 0 | 0 | 0 | 0 | 36 | 49 | Carbamidomethylation |
| K.GC(+57.02)VASC(+57.02)PSFESH.Y | Y | 52.40 | 1336.5176 | 12 | 0.8 | 669.2666 | 2 | 11.38 | 5 | F5:2227 | 05112019\_RID\_1313\_NaNaRj\_F5.raw |  |  |  |  | 1E6 |  |  |  |  |  | 1 | 0 | 0 | 0 | 0 | 1 | 0 | 0 | 0 | 0 | 0 | 36 | 47 | Carbamidomethylation |
| C.VASC(+57.02)PSFESHYK.F | Y | 48.51 | 1410.6238 | 12 | 0.7 | 706.3196 | 2 | 11.06 | 5 | F5:1920 | 05112019\_RID\_1313\_NaNaRj\_F5.raw |  |  |  |  | 5.27E5 |  |  |  |  |  | 2 | 0 | 0 | 0 | 0 | 2 | 0 | 0 | 0 | 0 | 0 | 38 | 49 | Carbamidomethylation |
| E.KGC(+57.02)VASC(+57.02)PSFESHYK.F | Y | 44.51 | 1755.7709 | 15 | -0.3 | 586.2640 | 3 | 10.96 | 5 | F5:1817 | 05112019\_RID\_1313\_NaNaRj\_F5.raw |  |  |  |  | 1.43E5 |  |  |  |  |  | 1 | 0 | 0 | 0 | 0 | 1 | 0 | 0 | 0 | 0 | 0 | 35 | 49 | Carbamidomethylation |
| total 4 peptides |  |  |  |  |  |  |  |  |  |  |  |  |  |  |  |  |  |  |  |  |  |  |  |  |  |  |  |  |  |  |  |  |  |  |  |
| --- | --- | --- | --- | --- | --- | --- | --- | --- | --- | --- | --- | --- | --- | --- | --- | --- | --- | --- | --- | --- | --- | --- | --- | --- | --- | --- | --- | --- | --- | --- | --- | --- | --- | --- | --- |

Q8WNW3|PLAK\_PIG

back to list

  

| Protein Coverage
| Supporting Peptides
|

Protein Coverage:

Supporting Peptides:

| Peptide | Uniq | -10lgP | Mass | Length | ppm | m/z | z | RT | Fraction | Scan | Source File | Area F1 | Area F2 | Area F3 | Area F4 | Area F5 | Area F6 | Area F7 | Area F8 | Area F9 | Area F10 | #Feature | #Feature F1 | #Feature F2 | #Feature F3 | #Feature F4 | #Feature F5 | #Feature F6 | #Feature F7 | #Feature F8 | #Feature F9 | #Feature F10 | Start | End | PTM |
| --- | --- | --- | --- | --- | --- | --- | --- | --- | --- | --- | --- | --- | --- | --- | --- | --- | --- | --- | --- | --- | --- | --- | --- | --- | --- | --- | --- | --- | --- | --- | --- | --- | --- | --- | --- |
| K.LLNDEDPVVVTK.A | Y | 69.95 | 1340.7188 | 12 | 0.6 | 671.3671 | 2 | 42.95 | 2 | F2:13616 | 09012020\_NaNaRj\_F2.raw |  | 1.2E5 | 1.73E4 |  |  |  |  |  |  |  | 2 | 0 | 1 | 1 | 0 | 0 | 0 | 0 | 0 | 0 | 0 | 150 | 161 |  |
| K.SGGIPALVR.M | Y | 50.49 | 868.5130 | 9 | 0.0 | 435.2638 | 2 | 39.03 | 2 | F2:12062 | 09012020\_NaNaRj\_F2.raw |  | 1.85E5 |  |  |  |  |  |  |  |  | 1 | 0 | 1 | 0 | 0 | 0 | 0 | 0 | 0 | 0 | 0 | 225 | 233 |  |
| R.LNYGIPAIVK.L | Y | 48.83 | 1086.6437 | 10 | 0.3 | 544.3293 | 2 | 56.24 | 2 | F2:18732 | 09012020\_NaNaRj\_F2.raw |  | 2.08E5 |  |  |  |  |  |  |  |  | 1 | 0 | 1 | 0 | 0 | 0 | 0 | 0 | 0 | 0 | 0 | 478 | 487 |  |
| K.LLNQPNQWPLVK.A | Y | 47.65 | 1448.8140 | 12 | 0.3 | 725.4145 | 2 | 58.05 | 2 | F2:19262 | 09012020\_NaNaRj\_F2.raw |  | 1.13E5 |  |  |  |  |  |  |  |  | 1 | 0 | 1 | 0 | 0 | 0 | 0 | 0 | 0 | 0 | 0 | 488 | 499 |  |
| R.ALMGSPQLVAAVVR.T | Y | 40.69 | 1410.8016 | 14 | 0.1 | 706.4081 | 2 | 63.65 | 2 | F2:20998 | 09012020\_NaNaRj\_F2.raw |  | 8.06E3 |  |  |  |  |  |  |  |  | 1 | 0 | 1 | 0 | 0 | 0 | 0 | 0 | 0 | 0 | 0 | 178 | 191 |  |
| total 5 peptides |  |  |  |  |  |  |  |  |  |  |  |  |  |  |  |  |  |  |  |  |  |  |  |  |  |  |  |  |  |  |  |  |  |  |  |
| --- | --- | --- | --- | --- | --- | --- | --- | --- | --- | --- | --- | --- | --- | --- | --- | --- | --- | --- | --- | --- | --- | --- | --- | --- | --- | --- | --- | --- | --- | --- | --- | --- | --- | --- | --- |

P14923|PLAK\_HUMAN

back to list

  

| Protein Coverage
| Supporting Peptides
|

Protein Coverage:

Supporting Peptides:

| Peptide | Uniq | -10lgP | Mass | Length | ppm | m/z | z | RT | Fraction | Scan | Source File | Area F1 | Area F2 | Area F3 | Area F4 | Area F5 | Area F6 | Area F7 | Area F8 | Area F9 | Area F10 | #Feature | #Feature F1 | #Feature F2 | #Feature F3 | #Feature F4 | #Feature F5 | #Feature F6 | #Feature F7 | #Feature F8 | #Feature F9 | #Feature F10 | Start | End | PTM |
| --- | --- | --- | --- | --- | --- | --- | --- | --- | --- | --- | --- | --- | --- | --- | --- | --- | --- | --- | --- | --- | --- | --- | --- | --- | --- | --- | --- | --- | --- | --- | --- | --- | --- | --- | --- |
| K.LLNDEDPVVVTK.A | Y | 69.95 | 1340.7188 | 12 | 0.6 | 671.3671 | 2 | 42.95 | 2 | F2:13616 | 09012020\_NaNaRj\_F2.raw |  | 1.2E5 | 1.73E4 |  |  |  |  |  |  |  | 2 | 0 | 1 | 1 | 0 | 0 | 0 | 0 | 0 | 0 | 0 | 150 | 161 |  |
| K.SGGIPALVR.M | Y | 50.49 | 868.5130 | 9 | 0.0 | 435.2638 | 2 | 39.03 | 2 | F2:12062 | 09012020\_NaNaRj\_F2.raw |  | 1.85E5 |  |  |  |  |  |  |  |  | 1 | 0 | 1 | 0 | 0 | 0 | 0 | 0 | 0 | 0 | 0 | 225 | 233 |  |
| R.LNYGIPAIVK.L | Y | 48.83 | 1086.6437 | 10 | 0.3 | 544.3293 | 2 | 56.24 | 2 | F2:18732 | 09012020\_NaNaRj\_F2.raw |  | 2.08E5 |  |  |  |  |  |  |  |  | 1 | 0 | 1 | 0 | 0 | 0 | 0 | 0 | 0 | 0 | 0 | 478 | 487 |  |
| K.LLNQPNQWPLVK.A | Y | 47.65 | 1448.8140 | 12 | 0.3 | 725.4145 | 2 | 58.05 | 2 | F2:19262 | 09012020\_NaNaRj\_F2.raw |  | 1.13E5 |  |  |  |  |  |  |  |  | 1 | 0 | 1 | 0 | 0 | 0 | 0 | 0 | 0 | 0 | 0 | 488 | 499 |  |
| R.ALMGSPQLVAAVVR.T | Y | 40.69 | 1410.8016 | 14 | 0.1 | 706.4081 | 2 | 63.65 | 2 | F2:20998 | 09012020\_NaNaRj\_F2.raw |  | 8.06E3 |  |  |  |  |  |  |  |  | 1 | 0 | 1 | 0 | 0 | 0 | 0 | 0 | 0 | 0 | 0 | 178 | 191 |  |
| total 5 peptides |  |  |  |  |  |  |  |  |  |  |  |  |  |  |  |  |  |  |  |  |  |  |  |  |  |  |  |  |  |  |  |  |  |  |  |
| --- | --- | --- | --- | --- | --- | --- | --- | --- | --- | --- | --- | --- | --- | --- | --- | --- | --- | --- | --- | --- | --- | --- | --- | --- | --- | --- | --- | --- | --- | --- | --- | --- | --- | --- | --- |

Q8SPJ1|PLAK\_BOVIN

back to list

  

| Protein Coverage
| Supporting Peptides
|

Protein Coverage:

Supporting Peptides:

| Peptide | Uniq | -10lgP | Mass | Length | ppm | m/z | z | RT | Fraction | Scan | Source File | Area F1 | Area F2 | Area F3 | Area F4 | Area F5 | Area F6 | Area F7 | Area F8 | Area F9 | Area F10 | #Feature | #Feature F1 | #Feature F2 | #Feature F3 | #Feature F4 | #Feature F5 | #Feature F6 | #Feature F7 | #Feature F8 | #Feature F9 | #Feature F10 | Start | End | PTM |
| --- | --- | --- | --- | --- | --- | --- | --- | --- | --- | --- | --- | --- | --- | --- | --- | --- | --- | --- | --- | --- | --- | --- | --- | --- | --- | --- | --- | --- | --- | --- | --- | --- | --- | --- | --- |
| K.LLNDEDPVVVTK.A | Y | 69.95 | 1340.7188 | 12 | 0.6 | 671.3671 | 2 | 42.95 | 2 | F2:13616 | 09012020\_NaNaRj\_F2.raw |  | 1.2E5 | 1.73E4 |  |  |  |  |  |  |  | 2 | 0 | 1 | 1 | 0 | 0 | 0 | 0 | 0 | 0 | 0 | 150 | 161 |  |
| K.SGGIPALVR.M | Y | 50.49 | 868.5130 | 9 | 0.0 | 435.2638 | 2 | 39.03 | 2 | F2:12062 | 09012020\_NaNaRj\_F2.raw |  | 1.85E5 |  |  |  |  |  |  |  |  | 1 | 0 | 1 | 0 | 0 | 0 | 0 | 0 | 0 | 0 | 0 | 225 | 233 |  |
| R.LNYGIPAIVK.L | Y | 48.83 | 1086.6437 | 10 | 0.3 | 544.3293 | 2 | 56.24 | 2 | F2:18732 | 09012020\_NaNaRj\_F2.raw |  | 2.08E5 |  |  |  |  |  |  |  |  | 1 | 0 | 1 | 0 | 0 | 0 | 0 | 0 | 0 | 0 | 0 | 478 | 487 |  |
| K.LLNQPNQWPLVK.A | Y | 47.65 | 1448.8140 | 12 | 0.3 | 725.4145 | 2 | 58.05 | 2 | F2:19262 | 09012020\_NaNaRj\_F2.raw |  | 1.13E5 |  |  |  |  |  |  |  |  | 1 | 0 | 1 | 0 | 0 | 0 | 0 | 0 | 0 | 0 | 0 | 488 | 499 |  |
| R.ALMGSPQLVAAVVR.T | Y | 40.69 | 1410.8016 | 14 | 0.1 | 706.4081 | 2 | 63.65 | 2 | F2:20998 | 09012020\_NaNaRj\_F2.raw |  | 8.06E3 |  |  |  |  |  |  |  |  | 1 | 0 | 1 | 0 | 0 | 0 | 0 | 0 | 0 | 0 | 0 | 178 | 191 |  |
| total 5 peptides |  |  |  |  |  |  |  |  |  |  |  |  |  |  |  |  |  |  |  |  |  |  |  |  |  |  |  |  |  |  |  |  |  |  |  |
| --- | --- | --- | --- | --- | --- | --- | --- | --- | --- | --- | --- | --- | --- | --- | --- | --- | --- | --- | --- | --- | --- | --- | --- | --- | --- | --- | --- | --- | --- | --- | --- | --- | --- | --- | --- |

Q02257|PLAK\_MOUSE

back to list

  

| Protein Coverage
| Supporting Peptides
|

Protein Coverage:

Supporting Peptides:

| Peptide | Uniq | -10lgP | Mass | Length | ppm | m/z | z | RT | Fraction | Scan | Source File | Area F1 | Area F2 | Area F3 | Area F4 | Area F5 | Area F6 | Area F7 | Area F8 | Area F9 | Area F10 | #Feature | #Feature F1 | #Feature F2 | #Feature F3 | #Feature F4 | #Feature F5 | #Feature F6 | #Feature F7 | #Feature F8 | #Feature F9 | #Feature F10 | Start | End | PTM |
| --- | --- | --- | --- | --- | --- | --- | --- | --- | --- | --- | --- | --- | --- | --- | --- | --- | --- | --- | --- | --- | --- | --- | --- | --- | --- | --- | --- | --- | --- | --- | --- | --- | --- | --- | --- |
| K.LLNDEDPVVVTK.A | Y | 69.95 | 1340.7188 | 12 | 0.6 | 671.3671 | 2 | 42.95 | 2 | F2:13616 | 09012020\_NaNaRj\_F2.raw |  | 1.2E5 | 1.73E4 |  |  |  |  |  |  |  | 2 | 0 | 1 | 1 | 0 | 0 | 0 | 0 | 0 | 0 | 0 | 150 | 161 |  |
| K.SGGIPALVR.M | Y | 50.49 | 868.5130 | 9 | 0.0 | 435.2638 | 2 | 39.03 | 2 | F2:12062 | 09012020\_NaNaRj\_F2.raw |  | 1.85E5 |  |  |  |  |  |  |  |  | 1 | 0 | 1 | 0 | 0 | 0 | 0 | 0 | 0 | 0 | 0 | 225 | 233 |  |
| R.LNYGIPAIVK.L | Y | 48.83 | 1086.6437 | 10 | 0.3 | 544.3293 | 2 | 56.24 | 2 | F2:18732 | 09012020\_NaNaRj\_F2.raw |  | 2.08E5 |  |  |  |  |  |  |  |  | 1 | 0 | 1 | 0 | 0 | 0 | 0 | 0 | 0 | 0 | 0 | 478 | 487 |  |
| K.LLNQPNQWPLVK.A | Y | 47.65 | 1448.8140 | 12 | 0.3 | 725.4145 | 2 | 58.05 | 2 | F2:19262 | 09012020\_NaNaRj\_F2.raw |  | 1.13E5 |  |  |  |  |  |  |  |  | 1 | 0 | 1 | 0 | 0 | 0 | 0 | 0 | 0 | 0 | 0 | 488 | 499 |  |
| R.ALMGSPQLVAAVVR.T | Y | 40.69 | 1410.8016 | 14 | 0.1 | 706.4081 | 2 | 63.65 | 2 | F2:20998 | 09012020\_NaNaRj\_F2.raw |  | 8.06E3 |  |  |  |  |  |  |  |  | 1 | 0 | 1 | 0 | 0 | 0 | 0 | 0 | 0 | 0 | 0 | 178 | 191 |  |
| total 5 peptides |  |  |  |  |  |  |  |  |  |  |  |  |  |  |  |  |  |  |  |  |  |  |  |  |  |  |  |  |  |  |  |  |  |  |  |
| --- | --- | --- | --- | --- | --- | --- | --- | --- | --- | --- | --- | --- | --- | --- | --- | --- | --- | --- | --- | --- | --- | --- | --- | --- | --- | --- | --- | --- | --- | --- | --- | --- | --- | --- | --- |

Q6P0K8|PLAK\_RAT

back to list

  

| Protein Coverage
| Supporting Peptides
|

Protein Coverage:

Supporting Peptides:

| Peptide | Uniq | -10lgP | Mass | Length | ppm | m/z | z | RT | Fraction | Scan | Source File | Area F1 | Area F2 | Area F3 | Area F4 | Area F5 | Area F6 | Area F7 | Area F8 | Area F9 | Area F10 | #Feature | #Feature F1 | #Feature F2 | #Feature F3 | #Feature F4 | #Feature F5 | #Feature F6 | #Feature F7 | #Feature F8 | #Feature F9 | #Feature F10 | Start | End | PTM |
| --- | --- | --- | --- | --- | --- | --- | --- | --- | --- | --- | --- | --- | --- | --- | --- | --- | --- | --- | --- | --- | --- | --- | --- | --- | --- | --- | --- | --- | --- | --- | --- | --- | --- | --- | --- |
| K.LLNDEDPVVVTK.A | Y | 69.95 | 1340.7188 | 12 | 0.6 | 671.3671 | 2 | 42.95 | 2 | F2:13616 | 09012020\_NaNaRj\_F2.raw |  | 1.2E5 | 1.73E4 |  |  |  |  |  |  |  | 2 | 0 | 1 | 1 | 0 | 0 | 0 | 0 | 0 | 0 | 0 | 150 | 161 |  |
| K.SGGIPALVR.M | Y | 50.49 | 868.5130 | 9 | 0.0 | 435.2638 | 2 | 39.03 | 2 | F2:12062 | 09012020\_NaNaRj\_F2.raw |  | 1.85E5 |  |  |  |  |  |  |  |  | 1 | 0 | 1 | 0 | 0 | 0 | 0 | 0 | 0 | 0 | 0 | 225 | 233 |  |
| R.LNYGIPAIVK.L | Y | 48.83 | 1086.6437 | 10 | 0.3 | 544.3293 | 2 | 56.24 | 2 | F2:18732 | 09012020\_NaNaRj\_F2.raw |  | 2.08E5 |  |  |  |  |  |  |  |  | 1 | 0 | 1 | 0 | 0 | 0 | 0 | 0 | 0 | 0 | 0 | 478 | 487 |  |
| K.LLNQPNQWPLVK.A | Y | 47.65 | 1448.8140 | 12 | 0.3 | 725.4145 | 2 | 58.05 | 2 | F2:19262 | 09012020\_NaNaRj\_F2.raw |  | 1.13E5 |  |  |  |  |  |  |  |  | 1 | 0 | 1 | 0 | 0 | 0 | 0 | 0 | 0 | 0 | 0 | 488 | 499 |  |
| R.ALMGSPQLVAAVVR.T | Y | 40.69 | 1410.8016 | 14 | 0.1 | 706.4081 | 2 | 63.65 | 2 | F2:20998 | 09012020\_NaNaRj\_F2.raw |  | 8.06E3 |  |  |  |  |  |  |  |  | 1 | 0 | 1 | 0 | 0 | 0 | 0 | 0 | 0 | 0 | 0 | 178 | 191 |  |
| total 5 peptides |  |  |  |  |  |  |  |  |  |  |  |  |  |  |  |  |  |  |  |  |  |  |  |  |  |  |  |  |  |  |  |  |  |  |  |
| --- | --- | --- | --- | --- | --- | --- | --- | --- | --- | --- | --- | --- | --- | --- | --- | --- | --- | --- | --- | --- | --- | --- | --- | --- | --- | --- | --- | --- | --- | --- | --- | --- | --- | --- | --- |

Q5ZPJ7|VKT\_NAJAT

back to list

  

| Protein Coverage
| Supporting Peptides
|

Protein Coverage:

Supporting Peptides:

| Peptide | Uniq | -10lgP | Mass | Length | ppm | m/z | z | RT | Fraction | Scan | Source File | Area F1 | Area F2 | Area F3 | Area F4 | Area F5 | Area F6 | Area F7 | Area F8 | Area F9 | Area F10 | #Feature | #Feature F1 | #Feature F2 | #Feature F3 | #Feature F4 | #Feature F5 | #Feature F6 | #Feature F7 | #Feature F8 | #Feature F9 | #Feature F10 | Start | End | PTM |
| --- | --- | --- | --- | --- | --- | --- | --- | --- | --- | --- | --- | --- | --- | --- | --- | --- | --- | --- | --- | --- | --- | --- | --- | --- | --- | --- | --- | --- | --- | --- | --- | --- | --- | --- | --- |
| N.TC(+57.02)HSFTYSGC(+57.02)GGNANR.F | Y | 54.77 | 1787.7104 | 16 | 0.9 | 596.9113 | 3 | 10.89 | 5 | F5:1734 | 05112019\_RID\_1313\_NaNaRj\_F5.raw |  |  |  |  | 8.98E4 |  |  |  |  |  | 1 | 0 | 0 | 0 | 0 | 1 | 0 | 0 | 0 | 0 | 0 | 53 | 68 | Carbamidomethylation |
| Y.SNTC(+57.02)HSFTYSGC(+57.02)GGNANR.F | Y | 49.82 | 1988.7854 | 18 | 0.6 | 663.9362 | 3 | 10.92 | 5 | F5:1767 | 05112019\_RID\_1313\_NaNaRj\_F5.raw |  |  |  |  | 4.16E4 |  |  |  |  |  | 1 | 0 | 0 | 0 | 0 | 1 | 0 | 0 | 0 | 0 | 0 | 51 | 68 | Carbamidomethylation |
| H.SFTYSGC(+57.02)GGNANR.F | Y | 49.67 | 1389.5731 | 13 | 0.3 | 695.7941 | 2 | 11.11 | 5 | F5:1960 | 05112019\_RID\_1313\_NaNaRj\_F5.raw |  |  |  |  | 2.97E4 |  |  |  |  |  | 1 | 0 | 0 | 0 | 0 | 1 | 0 | 0 | 0 | 0 | 0 | 56 | 68 | Carbamidomethylation |
| R.TIDEC(+57.02)NRTC(+57.02)VG | N | 46.81 | 1323.5547 | 11 | 0.3 | 662.7848 | 2 | 10.95 | 5 | F5:1816 | 05112019\_RID\_1313\_NaNaRj\_F5.raw |  |  |  |  | 4.15E4 |  |  |  |  |  | 1 | 0 | 0 | 0 | 0 | 1 | 0 | 0 | 0 | 0 | 0 | 71 | 81 | Carbamidomethylation |
| Q.YSNTC(+57.02)HSFTYSGC(+57.02)GGNANR.F | Y | 46.27 | 2151.8486 | 19 | 0.7 | 718.2906 | 3 | 11.03 | 4 | F4:1906 | 05112019\_RID\_1313\_NaNaRj\_F4.raw |  |  |  | 6.99E4 |  |  |  |  |  |  | 1 | 0 | 0 | 0 | 1 | 0 | 0 | 0 | 0 | 0 | 0 | 50 | 68 | Carbamidomethylation |
| total 5 peptides |  |  |  |  |  |  |  |  |  |  |  |  |  |  |  |  |  |  |  |  |  |  |  |  |  |  |  |  |  |  |  |  |  |  |  |
| --- | --- | --- | --- | --- | --- | --- | --- | --- | --- | --- | --- | --- | --- | --- | --- | --- | --- | --- | --- | --- | --- | --- | --- | --- | --- | --- | --- | --- | --- | --- | --- | --- | --- | --- | --- |

A1TKQ5|CH60\_ACIAC

back to list

  

| Protein Coverage
| Supporting Peptides
|

Protein Coverage:

Supporting Peptides:

| Peptide | Uniq | -10lgP | Mass | Length | ppm | m/z | z | RT | Fraction | Scan | Source File | Area F1 | Area F2 | Area F3 | Area F4 | Area F5 | Area F6 | Area F7 | Area F8 | Area F9 | Area F10 | #Feature | #Feature F1 | #Feature F2 | #Feature F3 | #Feature F4 | #Feature F5 | #Feature F6 | #Feature F7 | #Feature F8 | #Feature F9 | #Feature F10 | Start | End | PTM |
| --- | --- | --- | --- | --- | --- | --- | --- | --- | --- | --- | --- | --- | --- | --- | --- | --- | --- | --- | --- | --- | --- | --- | --- | --- | --- | --- | --- | --- | --- | --- | --- | --- | --- | --- | --- |
| K.EGVITVEDGK.S | N | 53.32 | 1045.5292 | 10 | 0.2 | 523.7720 | 2 | 26.82 | 2 | F2:6999 | 09012020\_NaNaRj\_F2.raw |  | 3.72E4 |  |  |  |  |  |  |  |  | 1 | 0 | 1 | 0 | 0 | 0 | 0 | 0 | 0 | 0 | 0 | 172 | 181 |  |
| K.LAGGVAVIK.V | N | 48.11 | 826.5276 | 9 | -0.2 | 414.2710 | 2 | 30.76 | 2 | F2:8594 | 09012020\_NaNaRj\_F2.raw |  | 0 |  |  |  |  |  |  |  |  | 1 | 0 | 1 | 0 | 0 | 0 | 0 | 0 | 0 | 0 | 0 | 372 | 380 |  |
| K.AVTALVAELK.K | Y | 47.50 | 1013.6121 | 10 | 0.8 | 507.8137 | 2 | 50.76 | 2 | F2:16903 | 09012020\_NaNaRj\_F2.raw |  | 1.53E5 |  |  |  |  |  |  |  |  | 1 | 0 | 1 | 0 | 0 | 0 | 0 | 0 | 0 | 0 | 0 | 123 | 132 |  |
| K.VTLADLGQAK.R | N | 44.53 | 1014.5709 | 10 | -0.3 | 508.2926 | 2 | 40.69 | 2 | F2:12633 | 09012020\_NaNaRj\_F2.raw |  | 2.89E5 |  |  |  |  |  |  |  |  | 1 | 0 | 1 | 0 | 0 | 0 | 0 | 0 | 0 | 0 | 0 | 312 | 321 |  |
| total 4 peptides |  |  |  |  |  |  |  |  |  |  |  |  |  |  |  |  |  |  |  |  |  |  |  |  |  |  |  |  |  |  |  |  |  |  |  |
| --- | --- | --- | --- | --- | --- | --- | --- | --- | --- | --- | --- | --- | --- | --- | --- | --- | --- | --- | --- | --- | --- | --- | --- | --- | --- | --- | --- | --- | --- | --- | --- | --- | --- | --- | --- |

#CONTAM#K1H1\_HUMAN|

back to list

  

| Protein Coverage
| Supporting Peptides
|

Protein Coverage:

Supporting Peptides:

| Peptide | Uniq | -10lgP | Mass | Length | ppm | m/z | z | RT | Fraction | Scan | Source File | Area F1 | Area F2 | Area F3 | Area F4 | Area F5 | Area F6 | Area F7 | Area F8 | Area F9 | Area F10 | #Feature | #Feature F1 | #Feature F2 | #Feature F3 | #Feature F4 | #Feature F5 | #Feature F6 | #Feature F7 | #Feature F8 | #Feature F9 | #Feature F10 | Start | End | PTM |
| --- | --- | --- | --- | --- | --- | --- | --- | --- | --- | --- | --- | --- | --- | --- | --- | --- | --- | --- | --- | --- | --- | --- | --- | --- | --- | --- | --- | --- | --- | --- | --- | --- | --- | --- | --- |
| R.LEC(+57.02)EINTYR.S | Y | 59.33 | 1196.5496 | 9 | -0.3 | 599.2819 | 2 | 11.36 | 3 | F3:2165 | 05112019\_RID\_1313\_NaNaRj\_F3.raw |  |  | 7.19E3 |  |  |  |  |  |  |  | 1 | 0 | 0 | 1 | 0 | 0 | 0 | 0 | 0 | 0 | 0 | 349 | 357 | Carbamidomethylation |
| K.LAADDFR.T | N | 46.83 | 806.3922 | 7 | 0.6 | 404.2036 | 2 | 11.46 | 5 | F5:2311 | 05112019\_RID\_1313\_NaNaRj\_F5.raw |  | 5.1E6 | 5.72E4 |  | 4.73E5 |  | 1.38E4 |  |  | 3.3E4 | 6 | 0 | 2 | 1 | 0 | 1 | 0 | 1 | 0 | 0 | 1 | 136 | 142 |  |
| R.QNQEYQVLLDVR.A | Y | 44.36 | 1503.7681 | 12 | 0.7 | 752.8918 | 2 | 15.59 | 3 | F3:3465 | 05112019\_RID\_1313\_NaNaRj\_F3.raw |  |  | 2.01E4 |  |  |  |  |  |  |  | 1 | 0 | 0 | 1 | 0 | 0 | 0 | 0 | 0 | 0 | 0 | 335 | 346 |  |
| R.DSLENTLTESEAR.Y | Y | 40.98 | 1463.6740 | 13 | 0.1 | 732.8444 | 2 | 12.06 | 3 | F3:2720 | 05112019\_RID\_1313\_NaNaRj\_F3.raw |  |  | 5.78E3 |  |  |  |  |  |  |  | 1 | 0 | 0 | 1 | 0 | 0 | 0 | 0 | 0 | 0 | 0 | 294 | 306 |  |
| total 4 peptides |  |  |  |  |  |  |  |  |  |  |  |  |  |  |  |  |  |  |  |  |  |  |  |  |  |  |  |  |  |  |  |  |  |  |  |
| --- | --- | --- | --- | --- | --- | --- | --- | --- | --- | --- | --- | --- | --- | --- | --- | --- | --- | --- | --- | --- | --- | --- | --- | --- | --- | --- | --- | --- | --- | --- | --- | --- | --- | --- | --- |

A5A6M5|K1H1\_PANTR

back to list

  

| Protein Coverage
| Supporting Peptides
|

Protein Coverage:

Supporting Peptides:

| Peptide | Uniq | -10lgP | Mass | Length | ppm | m/z | z | RT | Fraction | Scan | Source File | Area F1 | Area F2 | Area F3 | Area F4 | Area F5 | Area F6 | Area F7 | Area F8 | Area F9 | Area F10 | #Feature | #Feature F1 | #Feature F2 | #Feature F3 | #Feature F4 | #Feature F5 | #Feature F6 | #Feature F7 | #Feature F8 | #Feature F9 | #Feature F10 | Start | End | PTM |
| --- | --- | --- | --- | --- | --- | --- | --- | --- | --- | --- | --- | --- | --- | --- | --- | --- | --- | --- | --- | --- | --- | --- | --- | --- | --- | --- | --- | --- | --- | --- | --- | --- | --- | --- | --- |
| R.LEC(+57.02)EINTYR.S | Y | 59.33 | 1196.5496 | 9 | -0.3 | 599.2819 | 2 | 11.36 | 3 | F3:2165 | 05112019\_RID\_1313\_NaNaRj\_F3.raw |  |  | 7.19E3 |  |  |  |  |  |  |  | 1 | 0 | 0 | 1 | 0 | 0 | 0 | 0 | 0 | 0 | 0 | 349 | 357 | Carbamidomethylation |
| K.LAADDFR.T | N | 46.83 | 806.3922 | 7 | 0.6 | 404.2036 | 2 | 11.46 | 5 | F5:2311 | 05112019\_RID\_1313\_NaNaRj\_F5.raw |  | 5.1E6 | 5.72E4 |  | 4.73E5 |  | 1.38E4 |  |  | 3.3E4 | 6 | 0 | 2 | 1 | 0 | 1 | 0 | 1 | 0 | 0 | 1 | 136 | 142 |  |
| R.QNQEYQVLLDVR.A | Y | 44.36 | 1503.7681 | 12 | 0.7 | 752.8918 | 2 | 15.59 | 3 | F3:3465 | 05112019\_RID\_1313\_NaNaRj\_F3.raw |  |  | 2.01E4 |  |  |  |  |  |  |  | 1 | 0 | 0 | 1 | 0 | 0 | 0 | 0 | 0 | 0 | 0 | 335 | 346 |  |
| R.DSLENTLTESEAR.Y | Y | 40.98 | 1463.6740 | 13 | 0.1 | 732.8444 | 2 | 12.06 | 3 | F3:2720 | 05112019\_RID\_1313\_NaNaRj\_F3.raw |  |  | 5.78E3 |  |  |  |  |  |  |  | 1 | 0 | 0 | 1 | 0 | 0 | 0 | 0 | 0 | 0 | 0 | 294 | 306 |  |
| total 4 peptides |  |  |  |  |  |  |  |  |  |  |  |  |  |  |  |  |  |  |  |  |  |  |  |  |  |  |  |  |  |  |  |  |  |  |  |
| --- | --- | --- | --- | --- | --- | --- | --- | --- | --- | --- | --- | --- | --- | --- | --- | --- | --- | --- | --- | --- | --- | --- | --- | --- | --- | --- | --- | --- | --- | --- | --- | --- | --- | --- | --- |

Q15323|K1H1\_HUMAN

back to list

  

| Protein Coverage
| Supporting Peptides
|

Protein Coverage:

Supporting Peptides:

| Peptide | Uniq | -10lgP | Mass | Length | ppm | m/z | z | RT | Fraction | Scan | Source File | Area F1 | Area F2 | Area F3 | Area F4 | Area F5 | Area F6 | Area F7 | Area F8 | Area F9 | Area F10 | #Feature | #Feature F1 | #Feature F2 | #Feature F3 | #Feature F4 | #Feature F5 | #Feature F6 | #Feature F7 | #Feature F8 | #Feature F9 | #Feature F10 | Start | End | PTM |
| --- | --- | --- | --- | --- | --- | --- | --- | --- | --- | --- | --- | --- | --- | --- | --- | --- | --- | --- | --- | --- | --- | --- | --- | --- | --- | --- | --- | --- | --- | --- | --- | --- | --- | --- | --- |
| R.LEC(+57.02)EINTYR.S | Y | 59.33 | 1196.5496 | 9 | -0.3 | 599.2819 | 2 | 11.36 | 3 | F3:2165 | 05112019\_RID\_1313\_NaNaRj\_F3.raw |  |  | 7.19E3 |  |  |  |  |  |  |  | 1 | 0 | 0 | 1 | 0 | 0 | 0 | 0 | 0 | 0 | 0 | 349 | 357 | Carbamidomethylation |
| K.LAADDFR.T | N | 46.83 | 806.3922 | 7 | 0.6 | 404.2036 | 2 | 11.46 | 5 | F5:2311 | 05112019\_RID\_1313\_NaNaRj\_F5.raw |  | 5.1E6 | 5.72E4 |  | 4.73E5 |  | 1.38E4 |  |  | 3.3E4 | 6 | 0 | 2 | 1 | 0 | 1 | 0 | 1 | 0 | 0 | 1 | 136 | 142 |  |
| R.QNQEYQVLLDVR.A | Y | 44.36 | 1503.7681 | 12 | 0.7 | 752.8918 | 2 | 15.59 | 3 | F3:3465 | 05112019\_RID\_1313\_NaNaRj\_F3.raw |  |  | 2.01E4 |  |  |  |  |  |  |  | 1 | 0 | 0 | 1 | 0 | 0 | 0 | 0 | 0 | 0 | 0 | 335 | 346 |  |
| R.DSLENTLTESEAR.Y | Y | 40.98 | 1463.6740 | 13 | 0.1 | 732.8444 | 2 | 12.06 | 3 | F3:2720 | 05112019\_RID\_1313\_NaNaRj\_F3.raw |  |  | 5.78E3 |  |  |  |  |  |  |  | 1 | 0 | 0 | 1 | 0 | 0 | 0 | 0 | 0 | 0 | 0 | 294 | 306 |  |
| total 4 peptides |  |  |  |  |  |  |  |  |  |  |  |  |  |  |  |  |  |  |  |  |  |  |  |  |  |  |  |  |  |  |  |  |  |  |  |
| --- | --- | --- | --- | --- | --- | --- | --- | --- | --- | --- | --- | --- | --- | --- | --- | --- | --- | --- | --- | --- | --- | --- | --- | --- | --- | --- | --- | --- | --- | --- | --- | --- | --- | --- | --- |

Q8N1N4|K2C78\_HUMAN

back to list

  

| Protein Coverage
| Supporting Peptides
|

Protein Coverage:

Supporting Peptides:

| Peptide | Uniq | -10lgP | Mass | Length | ppm | m/z | z | RT | Fraction | Scan | Source File | Area F1 | Area F2 | Area F3 | Area F4 | Area F5 | Area F6 | Area F7 | Area F8 | Area F9 | Area F10 | #Feature | #Feature F1 | #Feature F2 | #Feature F3 | #Feature F4 | #Feature F5 | #Feature F6 | #Feature F7 | #Feature F8 | #Feature F9 | #Feature F10 | Start | End | PTM |
| --- | --- | --- | --- | --- | --- | --- | --- | --- | --- | --- | --- | --- | --- | --- | --- | --- | --- | --- | --- | --- | --- | --- | --- | --- | --- | --- | --- | --- | --- | --- | --- | --- | --- | --- | --- |
| R.LLEGEEC(+57.02)R.M | N | 54.31 | 1004.4597 | 8 | -0.6 | 503.2368 | 2 | 11.02 | 5 | F5:1874 | 05112019\_RID\_1313\_NaNaRj\_F5.raw |  |  |  |  | 1.16E5 |  |  |  |  |  | 1 | 0 | 0 | 0 | 0 | 1 | 0 | 0 | 0 | 0 | 0 | 416 | 423 | Carbamidomethylation |
| K.VDELEAALR.M | Y | 50.31 | 1014.5345 | 9 | 0.9 | 508.2750 | 2 | 42.92 | 2 | F2:13583 | 09012020\_NaNaRj\_F2.raw |  | 1.01E5 |  |  | 9.88E3 |  |  |  |  |  | 3 | 0 | 2 | 0 | 0 | 1 | 0 | 0 | 0 | 0 | 0 | 375 | 383 |  |
| Q.FASFIDK.V | N | 47.57 | 826.4225 | 7 | 0.0 | 414.2185 | 2 | 39.16 | 2 | F2:12055 | 09012020\_NaNaRj\_F2.raw | 6.35E3 | 4.99E6 |  |  | 4.92E5 |  |  |  | 5.38E3 |  | 4 | 1 | 1 | 0 | 0 | 1 | 0 | 0 | 0 | 1 | 0 | 122 | 128 |  |
| K.QLEQLQGER.G | Y | 42.75 | 1099.5621 | 9 | 1.0 | 550.7889 | 2 | 20.93 | 2 | F2:4668 | 09012020\_NaNaRj\_F2.raw |  | 2.7E4 |  |  |  |  |  |  |  |  | 1 | 0 | 1 | 0 | 0 | 0 | 0 | 0 | 0 | 0 | 0 | 172 | 180 |  |
| total 4 peptides |  |  |  |  |  |  |  |  |  |  |  |  |  |  |  |  |  |  |  |  |  |  |  |  |  |  |  |  |  |  |  |  |  |  |  |
| --- | --- | --- | --- | --- | --- | --- | --- | --- | --- | --- | --- | --- | --- | --- | --- | --- | --- | --- | --- | --- | --- | --- | --- | --- | --- | --- | --- | --- | --- | --- | --- | --- | --- | --- | --- |

Q02413|DSG1\_HUMAN

back to list

  

| Protein Coverage
| Supporting Peptides
|

Protein Coverage:

Supporting Peptides:

| Peptide | Uniq | -10lgP | Mass | Length | ppm | m/z | z | RT | Fraction | Scan | Source File | Area F1 | Area F2 | Area F3 | Area F4 | Area F5 | Area F6 | Area F7 | Area F8 | Area F9 | Area F10 | #Feature | #Feature F1 | #Feature F2 | #Feature F3 | #Feature F4 | #Feature F5 | #Feature F6 | #Feature F7 | #Feature F8 | #Feature F9 | #Feature F10 | Start | End | PTM |
| --- | --- | --- | --- | --- | --- | --- | --- | --- | --- | --- | --- | --- | --- | --- | --- | --- | --- | --- | --- | --- | --- | --- | --- | --- | --- | --- | --- | --- | --- | --- | --- | --- | --- | --- | --- |
| R.DGSNVIVTER.V | Y | 63.32 | 1088.5461 | 10 | -0.2 | 545.2802 | 2 | 26.26 | 2 | F2:6771 | 09012020\_NaNaRj\_F2.raw |  | 1.38E4 |  |  |  |  |  |  |  |  | 1 | 0 | 1 | 0 | 0 | 0 | 0 | 0 | 0 | 0 | 0 | 888 | 897 |  |
| R.YVM(+15.99)GNNPADLLAVDSR.T | Y | 50.43 | 1749.8356 | 16 | 1.0 | 875.9260 | 2 | 15.22 | 3 | F3:3412 | 05112019\_RID\_1313\_NaNaRj\_F3.raw |  |  | 1.37E4 |  |  |  |  |  |  |  | 1 | 0 | 0 | 1 | 0 | 0 | 0 | 0 | 0 | 0 | 0 | 423 | 438 | Oxidation (M) |
| R.M(+15.99)TGFELTEGVK.T | Y | 48.35 | 1226.5853 | 11 | 0.8 | 614.3004 | 2 | 12.04 | 3 | F3:2708 | 05112019\_RID\_1313\_NaNaRj\_F3.raw |  |  | 5.14E3 |  |  |  |  |  |  |  | 1 | 0 | 0 | 1 | 0 | 0 | 0 | 0 | 0 | 0 | 0 | 651 | 661 | Oxidation (M) |
| R.MTGFELTEGVK.T | Y | 40.98 | 1210.5903 | 11 | 0.2 | 606.3026 | 2 | 49.68 | 2 | F2:16481 | 09012020\_NaNaRj\_F2.raw |  | 4.97E4 |  |  |  |  |  |  |  |  | 1 | 0 | 1 | 0 | 0 | 0 | 0 | 0 | 0 | 0 | 0 | 651 | 661 |  |
| total 4 peptides |  |  |  |  |  |  |  |  |  |  |  |  |  |  |  |  |  |  |  |  |  |  |  |  |  |  |  |  |  |  |  |  |  |  |  |
| --- | --- | --- | --- | --- | --- | --- | --- | --- | --- | --- | --- | --- | --- | --- | --- | --- | --- | --- | --- | --- | --- | --- | --- | --- | --- | --- | --- | --- | --- | --- | --- | --- | --- | --- | --- |

P00601|PA2A3\_NAJME

back to list

  

| Protein Coverage
| Supporting Peptides
|

Protein Coverage:

Supporting Peptides:

| Peptide | Uniq | -10lgP | Mass | Length | ppm | m/z | z | RT | Fraction | Scan | Source File | Area F1 | Area F2 | Area F3 | Area F4 | Area F5 | Area F6 | Area F7 | Area F8 | Area F9 | Area F10 | #Feature | #Feature F1 | #Feature F2 | #Feature F3 | #Feature F4 | #Feature F5 | #Feature F6 | #Feature F7 | #Feature F8 | #Feature F9 | #Feature F10 | Start | End | PTM |
| --- | --- | --- | --- | --- | --- | --- | --- | --- | --- | --- | --- | --- | --- | --- | --- | --- | --- | --- | --- | --- | --- | --- | --- | --- | --- | --- | --- | --- | --- | --- | --- | --- | --- | --- | --- |
| R.GGSGTPVDDLDR.C | N | 79.15 | 1187.5417 | 12 | -1.8 | 594.7771 | 2 | 11.41 | 6 | F6:2201 | 05112019\_RID\_1313\_NaNaRj\_F6.raw |  |  |  |  |  | 5.21E7 |  |  | 4.03E4 |  | 4 | 0 | 0 | 0 | 0 | 0 | 3 | 0 | 0 | 1 | 0 | 31 | 42 |  |
| K.ISGC(+57.02)WPYIK.T | Y | 45.72 | 1122.5532 | 9 | -0.1 | 562.2838 | 2 | 14.33 | 6 | F6:4327 | 05112019\_RID\_1313\_NaNaRj\_F6.raw |  |  |  |  |  | 6.81E4 |  |  |  |  | 1 | 0 | 0 | 0 | 0 | 0 | 1 | 0 | 0 | 0 | 0 | 57 | 65 | Carbamidomethylation |
| total 2 peptides |  |  |  |  |  |  |  |  |  |  |  |  |  |  |  |  |  |  |  |  |  |  |  |  |  |  |  |  |  |  |  |  |  |  |  |
| --- | --- | --- | --- | --- | --- | --- | --- | --- | --- | --- | --- | --- | --- | --- | --- | --- | --- | --- | --- | --- | --- | --- | --- | --- | --- | --- | --- | --- | --- | --- | --- | --- | --- | --- | --- |

P00600|PA2A2\_NAJME

back to list

  

| Protein Coverage
| Supporting Peptides
|

Protein Coverage:

Supporting Peptides:

| Peptide | Uniq | -10lgP | Mass | Length | ppm | m/z | z | RT | Fraction | Scan | Source File | Area F1 | Area F2 | Area F3 | Area F4 | Area F5 | Area F6 | Area F7 | Area F8 | Area F9 | Area F10 | #Feature | #Feature F1 | #Feature F2 | #Feature F3 | #Feature F4 | #Feature F5 | #Feature F6 | #Feature F7 | #Feature F8 | #Feature F9 | #Feature F10 | Start | End | PTM |
| --- | --- | --- | --- | --- | --- | --- | --- | --- | --- | --- | --- | --- | --- | --- | --- | --- | --- | --- | --- | --- | --- | --- | --- | --- | --- | --- | --- | --- | --- | --- | --- | --- | --- | --- | --- |
| R.GGSGTPVDDLDR.C | N | 79.15 | 1187.5417 | 12 | -1.8 | 594.7771 | 2 | 11.41 | 6 | F6:2201 | 05112019\_RID\_1313\_NaNaRj\_F6.raw |  |  |  |  |  | 5.21E7 |  |  | 4.03E4 |  | 4 | 0 | 0 | 0 | 0 | 0 | 3 | 0 | 0 | 1 | 0 | 31 | 42 |  |
| K.ISGC(+57.02)WPYIK.T | Y | 45.72 | 1122.5532 | 9 | -0.1 | 562.2838 | 2 | 14.33 | 6 | F6:4327 | 05112019\_RID\_1313\_NaNaRj\_F6.raw |  |  |  |  |  | 6.81E4 |  |  |  |  | 1 | 0 | 0 | 0 | 0 | 0 | 1 | 0 | 0 | 0 | 0 | 57 | 65 | Carbamidomethylation |
| total 2 peptides |  |  |  |  |  |  |  |  |  |  |  |  |  |  |  |  |  |  |  |  |  |  |  |  |  |  |  |  |  |  |  |  |  |  |  |
| --- | --- | --- | --- | --- | --- | --- | --- | --- | --- | --- | --- | --- | --- | --- | --- | --- | --- | --- | --- | --- | --- | --- | --- | --- | --- | --- | --- | --- | --- | --- | --- | --- | --- | --- | --- |

Q86YZ3|HORN\_HUMAN

back to list

  

| Protein Coverage
| Supporting Peptides
|

Protein Coverage:

Supporting Peptides:

| Peptide | Uniq | -10lgP | Mass | Length | ppm | m/z | z | RT | Fraction | Scan | Source File | Area F1 | Area F2 | Area F3 | Area F4 | Area F5 | Area F6 | Area F7 | Area F8 | Area F9 | Area F10 | #Feature | #Feature F1 | #Feature F2 | #Feature F3 | #Feature F4 | #Feature F5 | #Feature F6 | #Feature F7 | #Feature F8 | #Feature F9 | #Feature F10 | Start | End | PTM |
| --- | --- | --- | --- | --- | --- | --- | --- | --- | --- | --- | --- | --- | --- | --- | --- | --- | --- | --- | --- | --- | --- | --- | --- | --- | --- | --- | --- | --- | --- | --- | --- | --- | --- | --- | --- |
| R.HGSGSGQSSSYSPYGSGSGWSSSR.G | Y | 67.49 | 2347.9690 | 24 | 0.4 | 783.6639 | 3 | 26.61 | 2 | F2:6888 | 09012020\_NaNaRj\_F2.raw |  | 6.32E4 |  |  |  |  |  |  |  |  | 1 | 0 | 1 | 0 | 0 | 0 | 0 | 0 | 0 | 0 | 0 | 1484 | 1507 |  |
| R.HGSGSGQSSSYGPYGSGSGWSSSR.G | Y | 47.12 | 2317.9585 | 24 | 1.6 | 773.6613 | 3 | 26.86 | 2 | F2:6982 | 09012020\_NaNaRj\_F2.raw |  | 2.48E4 |  |  |  |  |  |  |  |  | 1 | 0 | 1 | 0 | 0 | 0 | 0 | 0 | 0 | 0 | 0 | 1954 | 1977 |  |
| total 2 peptides |  |  |  |  |  |  |  |  |  |  |  |  |  |  |  |  |  |  |  |  |  |  |  |  |  |  |  |  |  |  |  |  |  |  |  |
| --- | --- | --- | --- | --- | --- | --- | --- | --- | --- | --- | --- | --- | --- | --- | --- | --- | --- | --- | --- | --- | --- | --- | --- | --- | --- | --- | --- | --- | --- | --- | --- | --- | --- | --- | --- |

C1IC49|3NO25\_WALAE

back to list

  

| Protein Coverage
| Supporting Peptides
|

Protein Coverage:

Supporting Peptides:

| Peptide | Uniq | -10lgP | Mass | Length | ppm | m/z | z | RT | Fraction | Scan | Source File | Area F1 | Area F2 | Area F3 | Area F4 | Area F5 | Area F6 | Area F7 | Area F8 | Area F9 | Area F10 | #Feature | #Feature F1 | #Feature F2 | #Feature F3 | #Feature F4 | #Feature F5 | #Feature F6 | #Feature F7 | #Feature F8 | #Feature F9 | #Feature F10 | Start | End | PTM |
| --- | --- | --- | --- | --- | --- | --- | --- | --- | --- | --- | --- | --- | --- | --- | --- | --- | --- | --- | --- | --- | --- | --- | --- | --- | --- | --- | --- | --- | --- | --- | --- | --- | --- | --- | --- |
| T.FYEGNLLGK.Q | N | 69.74 | 1039.5338 | 9 | 0.1 | 520.7742 | 2 | 12.75 | 4 | F4:3423 | 05112019\_RID\_1313\_NaNaRj\_F4.raw |  |  |  | 3.92E8 |  | 8.38E5 | 4.1E5 | 9.44E4 |  |  | 10 | 0 | 0 | 0 | 7 | 0 | 1 | 1 | 1 | 0 | 0 | 49 | 57 |  |
| R.GC(+57.02)AATC(+57.02)PEARPR.E | Y | 56.06 | 1344.6027 | 12 | -8.0 | 673.3032 | 2 | 10.80 | 4 | F4:1725 | 05112019\_RID\_1313\_NaNaRj\_F4.raw |  |  |  | 1.3E4 |  |  |  |  |  |  | 1 | 0 | 0 | 0 | 1 | 0 | 0 | 0 | 0 | 0 | 0 | 62 | 73 | Carbamidomethylation |
| total 2 peptides |  |  |  |  |  |  |  |  |  |  |  |  |  |  |  |  |  |  |  |  |  |  |  |  |  |  |  |  |  |  |  |  |  |  |  |
| --- | --- | --- | --- | --- | --- | --- | --- | --- | --- | --- | --- | --- | --- | --- | --- | --- | --- | --- | --- | --- | --- | --- | --- | --- | --- | --- | --- | --- | --- | --- | --- | --- | --- | --- | --- |

P78386|KRT85\_HUMAN

back to list

  

| Protein Coverage
| Supporting Peptides
|

Protein Coverage:

Supporting Peptides:

| Peptide | Uniq | -10lgP | Mass | Length | ppm | m/z | z | RT | Fraction | Scan | Source File | Area F1 | Area F2 | Area F3 | Area F4 | Area F5 | Area F6 | Area F7 | Area F8 | Area F9 | Area F10 | #Feature | #Feature F1 | #Feature F2 | #Feature F3 | #Feature F4 | #Feature F5 | #Feature F6 | #Feature F7 | #Feature F8 | #Feature F9 | #Feature F10 | Start | End | PTM |
| --- | --- | --- | --- | --- | --- | --- | --- | --- | --- | --- | --- | --- | --- | --- | --- | --- | --- | --- | --- | --- | --- | --- | --- | --- | --- | --- | --- | --- | --- | --- | --- | --- | --- | --- | --- |
| R.GGVSC(+57.02)GGLSYSTTPGR.Q | Y | 69.28 | 1554.7096 | 16 | 0.5 | 778.3625 | 2 | 11.43 | 3 | F3:2232 | 05112019\_RID\_1313\_NaNaRj\_F3.raw |  |  | 8.51E3 |  |  |  |  |  |  |  | 1 | 0 | 0 | 1 | 0 | 0 | 0 | 0 | 0 | 0 | 0 | 450 | 465 | Carbamidomethylation |
| K.LAELEGALQK.A | N | 55.73 | 1070.5972 | 10 | 0.8 | 536.3063 | 2 | 11.77 | 3 | F3:2545 | 05112019\_RID\_1313\_NaNaRj\_F3.raw |  |  | 2.85E4 |  |  |  |  |  |  |  | 1 | 0 | 0 | 1 | 0 | 0 | 0 | 0 | 0 | 0 | 0 | 385 | 394 |  |
| total 2 peptides |  |  |  |  |  |  |  |  |  |  |  |  |  |  |  |  |  |  |  |  |  |  |  |  |  |  |  |  |  |  |  |  |  |  |  |
| --- | --- | --- | --- | --- | --- | --- | --- | --- | --- | --- | --- | --- | --- | --- | --- | --- | --- | --- | --- | --- | --- | --- | --- | --- | --- | --- | --- | --- | --- | --- | --- | --- | --- | --- | --- |

#CONTAM#KRHB5\_HUMAN|

back to list

  

| Protein Coverage
| Supporting Peptides
|

Protein Coverage:

Supporting Peptides:

| Peptide | Uniq | -10lgP | Mass | Length | ppm | m/z | z | RT | Fraction | Scan | Source File | Area F1 | Area F2 | Area F3 | Area F4 | Area F5 | Area F6 | Area F7 | Area F8 | Area F9 | Area F10 | #Feature | #Feature F1 | #Feature F2 | #Feature F3 | #Feature F4 | #Feature F5 | #Feature F6 | #Feature F7 | #Feature F8 | #Feature F9 | #Feature F10 | Start | End | PTM |
| --- | --- | --- | --- | --- | --- | --- | --- | --- | --- | --- | --- | --- | --- | --- | --- | --- | --- | --- | --- | --- | --- | --- | --- | --- | --- | --- | --- | --- | --- | --- | --- | --- | --- | --- | --- |
| R.GGVSC(+57.02)GGLSYSTTPGR.Q | Y | 69.28 | 1554.7096 | 16 | 0.5 | 778.3625 | 2 | 11.43 | 3 | F3:2232 | 05112019\_RID\_1313\_NaNaRj\_F3.raw |  |  | 8.51E3 |  |  |  |  |  |  |  | 1 | 0 | 0 | 1 | 0 | 0 | 0 | 0 | 0 | 0 | 0 | 450 | 465 | Carbamidomethylation |
| K.LAELEGALQK.A | N | 55.73 | 1070.5972 | 10 | 0.8 | 536.3063 | 2 | 11.77 | 3 | F3:2545 | 05112019\_RID\_1313\_NaNaRj\_F3.raw |  |  | 2.85E4 |  |  |  |  |  |  |  | 1 | 0 | 0 | 1 | 0 | 0 | 0 | 0 | 0 | 0 | 0 | 385 | 394 |  |
| total 2 peptides |  |  |  |  |  |  |  |  |  |  |  |  |  |  |  |  |  |  |  |  |  |  |  |  |  |  |  |  |  |  |  |  |  |  |  |
| --- | --- | --- | --- | --- | --- | --- | --- | --- | --- | --- | --- | --- | --- | --- | --- | --- | --- | --- | --- | --- | --- | --- | --- | --- | --- | --- | --- | --- | --- | --- | --- | --- | --- | --- | --- |

P06871|TRY1\_CANLF

back to list

  

| Protein Coverage
| Supporting Peptides
|

Protein Coverage:

Supporting Peptides:

| Peptide | Uniq | -10lgP | Mass | Length | ppm | m/z | z | RT | Fraction | Scan | Source File | Area F1 | Area F2 | Area F3 | Area F4 | Area F5 | Area F6 | Area F7 | Area F8 | Area F9 | Area F10 | #Feature | #Feature F1 | #Feature F2 | #Feature F3 | #Feature F4 | #Feature F5 | #Feature F6 | #Feature F7 | #Feature F8 | #Feature F9 | #Feature F10 | Start | End | PTM |
| --- | --- | --- | --- | --- | --- | --- | --- | --- | --- | --- | --- | --- | --- | --- | --- | --- | --- | --- | --- | --- | --- | --- | --- | --- | --- | --- | --- | --- | --- | --- | --- | --- | --- | --- | --- |
| K.LSSPATLNSR.V | N | 56.40 | 1044.5564 | 10 | -1.0 | 523.2850 | 2 | 11.24 | 1 | F1:2001 | 05112019\_RID\_1313\_NaNaRj\_F1.raw | 3.24E5 | 1.6E6 | 5.55E4 | 0 | 1.26E5 |  |  |  | 2.06E5 |  | 6 | 1 | 1 | 1 | 1 | 1 | 0 | 0 | 0 | 1 | 0 | 113 | 122 |  |
| R.NSVPYQVSLN.S | Y | 52.47 | 1119.5560 | 10 | 0.0 | 560.7853 | 2 | 52.94 | 2 | F2:17667 | 09012020\_NaNaRj\_F2.raw |  | 2.05E5 |  |  |  |  |  |  |  |  | 1 | 0 | 1 | 0 | 0 | 0 | 0 | 0 | 0 | 0 | 0 | 33 | 42 |  |
| S.WIQQTIAAN | N | 41.69 | 1043.5400 | 9 | 0.1 | 522.7773 | 2 | 13.31 | 5 | F5:3459 | 05112019\_RID\_1313\_NaNaRj\_F5.raw |  |  |  |  | 1.55E5 |  |  |  |  |  | 1 | 0 | 0 | 0 | 0 | 1 | 0 | 0 | 0 | 0 | 0 | 238 | 246 |  |
| total 3 peptides |  |  |  |  |  |  |  |  |  |  |  |  |  |  |  |  |  |  |  |  |  |  |  |  |  |  |  |  |  |  |  |  |  |  |  |
| --- | --- | --- | --- | --- | --- | --- | --- | --- | --- | --- | --- | --- | --- | --- | --- | --- | --- | --- | --- | --- | --- | --- | --- | --- | --- | --- | --- | --- | --- | --- | --- | --- | --- | --- | --- |

P02845|VIT2\_CHICK

back to list

  

| Protein Coverage
| Supporting Peptides
|

Protein Coverage:

Supporting Peptides:

| Peptide | Uniq | -10lgP | Mass | Length | ppm | m/z | z | RT | Fraction | Scan | Source File | Area F1 | Area F2 | Area F3 | Area F4 | Area F5 | Area F6 | Area F7 | Area F8 | Area F9 | Area F10 | #Feature | #Feature F1 | #Feature F2 | #Feature F3 | #Feature F4 | #Feature F5 | #Feature F6 | #Feature F7 | #Feature F8 | #Feature F9 | #Feature F10 | Start | End | PTM |
| --- | --- | --- | --- | --- | --- | --- | --- | --- | --- | --- | --- | --- | --- | --- | --- | --- | --- | --- | --- | --- | --- | --- | --- | --- | --- | --- | --- | --- | --- | --- | --- | --- | --- | --- | --- |
| R.LPLSLPVGPR.I | Y | 49.26 | 1047.6440 | 10 | 0.7 | 524.8297 | 2 | 17.80 | 9 | F9:4085 | 05112019\_RID\_1313\_NaNaRj\_F9.raw |  |  |  |  |  | 3.72E4 | 3.25E4 |  | 2.07E4 |  | 3 | 0 | 0 | 0 | 0 | 0 | 1 | 1 | 0 | 1 | 0 | 1543 | 1552 |  |
| total 1 peptides |  |  |  |  |  |  |  |  |  |  |  |  |  |  |  |  |  |  |  |  |  |  |  |  |  |  |  |  |  |  |  |  |  |  |  |
| --- | --- | --- | --- | --- | --- | --- | --- | --- | --- | --- | --- | --- | --- | --- | --- | --- | --- | --- | --- | --- | --- | --- | --- | --- | --- | --- | --- | --- | --- | --- | --- | --- | --- | --- | --- |

P01453|3SAA\_NAJHA

back to list

  

| Protein Coverage
| Supporting Peptides
|

Protein Coverage:

Supporting Peptides:

| Peptide | Uniq | -10lgP | Mass | Length | ppm | m/z | z | RT | Fraction | Scan | Source File | Area F1 | Area F2 | Area F3 | Area F4 | Area F5 | Area F6 | Area F7 | Area F8 | Area F9 | Area F10 | #Feature | #Feature F1 | #Feature F2 | #Feature F3 | #Feature F4 | #Feature F5 | #Feature F6 | #Feature F7 | #Feature F8 | #Feature F9 | #Feature F10 | Start | End | PTM |
| --- | --- | --- | --- | --- | --- | --- | --- | --- | --- | --- | --- | --- | --- | --- | --- | --- | --- | --- | --- | --- | --- | --- | --- | --- | --- | --- | --- | --- | --- | --- | --- | --- | --- | --- | --- |
| R.GC(+57.02)IDVC(+57.02)PK.N | N | 56.82 | 947.4205 | 8 | -2.7 | 474.7162 | 2 | 11.15 | 8 | F8:2003 | 05112019\_RID\_1313\_NaNaRj\_F8.raw | 1E5 | 1.9E5 | 1.09E4 | 8.89E4 | 5.11E5 | 3.82E8 |  | 3.84E7 | 5.76E5 | 1.59E4 | 23 | 1 | 1 | 1 | 1 | 1 | 14 | 0 | 2 | 1 | 1 | 37 | 44 | Carbamidomethylation |
| K.MFMVSTSTVPVK.R | Y | 45.15 | 1325.6724 | 12 | -7.3 | 663.8386 | 2 | 16.85 | 8 | F8:5433 | 05112019\_RID\_1313\_NaNaRj\_F8.raw |  |  |  |  |  |  |  | 1.84E5 |  |  | 1 | 0 | 0 | 0 | 0 | 0 | 0 | 0 | 1 | 0 | 0 | 24 | 35 |  |
| total 2 peptides |  |  |  |  |  |  |  |  |  |  |  |  |  |  |  |  |  |  |  |  |  |  |  |  |  |  |  |  |  |  |  |  |  |  |  |
| --- | --- | --- | --- | --- | --- | --- | --- | --- | --- | --- | --- | --- | --- | --- | --- | --- | --- | --- | --- | --- | --- | --- | --- | --- | --- | --- | --- | --- | --- | --- | --- | --- | --- | --- | --- |

P01454|3SA9\_NAJHA

back to list

  

| Protein Coverage
| Supporting Peptides
|

Protein Coverage:

Supporting Peptides:

| Peptide | Uniq | -10lgP | Mass | Length | ppm | m/z | z | RT | Fraction | Scan | Source File | Area F1 | Area F2 | Area F3 | Area F4 | Area F5 | Area F6 | Area F7 | Area F8 | Area F9 | Area F10 | #Feature | #Feature F1 | #Feature F2 | #Feature F3 | #Feature F4 | #Feature F5 | #Feature F6 | #Feature F7 | #Feature F8 | #Feature F9 | #Feature F10 | Start | End | PTM |
| --- | --- | --- | --- | --- | --- | --- | --- | --- | --- | --- | --- | --- | --- | --- | --- | --- | --- | --- | --- | --- | --- | --- | --- | --- | --- | --- | --- | --- | --- | --- | --- | --- | --- | --- | --- |
| R.GC(+57.02)IDVC(+57.02)PK.D | N | 56.82 | 947.4205 | 8 | -2.7 | 474.7162 | 2 | 11.15 | 8 | F8:2003 | 05112019\_RID\_1313\_NaNaRj\_F8.raw | 1E5 | 1.9E5 | 1.09E4 | 8.89E4 | 5.11E5 | 3.82E8 |  | 3.84E7 | 5.76E5 | 1.59E4 | 23 | 1 | 1 | 1 | 1 | 1 | 14 | 0 | 2 | 1 | 1 | 37 | 44 | Carbamidomethylation |
| K.MFMVSTSTVPVK.R | Y | 45.15 | 1325.6724 | 12 | -7.3 | 663.8386 | 2 | 16.85 | 8 | F8:5433 | 05112019\_RID\_1313\_NaNaRj\_F8.raw |  |  |  |  |  |  |  | 1.84E5 |  |  | 1 | 0 | 0 | 0 | 0 | 0 | 0 | 0 | 1 | 0 | 0 | 24 | 35 |  |
| total 2 peptides |  |  |  |  |  |  |  |  |  |  |  |  |  |  |  |  |  |  |  |  |  |  |  |  |  |  |  |  |  |  |  |  |  |  |  |
| --- | --- | --- | --- | --- | --- | --- | --- | --- | --- | --- | --- | --- | --- | --- | --- | --- | --- | --- | --- | --- | --- | --- | --- | --- | --- | --- | --- | --- | --- | --- | --- | --- | --- | --- | --- |

P01455|3SA1\_NAJHA

back to list

  

| Protein Coverage
| Supporting Peptides
|

Protein Coverage:

Supporting Peptides:

| Peptide | Uniq | -10lgP | Mass | Length | ppm | m/z | z | RT | Fraction | Scan | Source File | Area F1 | Area F2 | Area F3 | Area F4 | Area F5 | Area F6 | Area F7 | Area F8 | Area F9 | Area F10 | #Feature | #Feature F1 | #Feature F2 | #Feature F3 | #Feature F4 | #Feature F5 | #Feature F6 | #Feature F7 | #Feature F8 | #Feature F9 | #Feature F10 | Start | End | PTM |
| --- | --- | --- | --- | --- | --- | --- | --- | --- | --- | --- | --- | --- | --- | --- | --- | --- | --- | --- | --- | --- | --- | --- | --- | --- | --- | --- | --- | --- | --- | --- | --- | --- | --- | --- | --- |
| R.GC(+57.02)IDVC(+57.02)PK.N | N | 56.82 | 947.4205 | 8 | -2.7 | 474.7162 | 2 | 11.15 | 8 | F8:2003 | 05112019\_RID\_1313\_NaNaRj\_F8.raw | 1E5 | 1.9E5 | 1.09E4 | 8.89E4 | 5.11E5 | 3.82E8 |  | 3.84E7 | 5.76E5 | 1.59E4 | 23 | 1 | 1 | 1 | 1 | 1 | 14 | 0 | 2 | 1 | 1 | 37 | 44 | Carbamidomethylation |
| K.MFMVSTSTVPVK.R | Y | 45.15 | 1325.6724 | 12 | -7.3 | 663.8386 | 2 | 16.85 | 8 | F8:5433 | 05112019\_RID\_1313\_NaNaRj\_F8.raw |  |  |  |  |  |  |  | 1.84E5 |  |  | 1 | 0 | 0 | 0 | 0 | 0 | 0 | 0 | 1 | 0 | 0 | 24 | 35 |  |
| total 2 peptides |  |  |  |  |  |  |  |  |  |  |  |  |  |  |  |  |  |  |  |  |  |  |  |  |  |  |  |  |  |  |  |  |  |  |  |
| --- | --- | --- | --- | --- | --- | --- | --- | --- | --- | --- | --- | --- | --- | --- | --- | --- | --- | --- | --- | --- | --- | --- | --- | --- | --- | --- | --- | --- | --- | --- | --- | --- | --- | --- | --- |

P01456|3SA1\_NAJNI

back to list

  

| Protein Coverage
| Supporting Peptides
|

Protein Coverage:

Supporting Peptides:

| Peptide | Uniq | -10lgP | Mass | Length | ppm | m/z | z | RT | Fraction | Scan | Source File | Area F1 | Area F2 | Area F3 | Area F4 | Area F5 | Area F6 | Area F7 | Area F8 | Area F9 | Area F10 | #Feature | #Feature F1 | #Feature F2 | #Feature F3 | #Feature F4 | #Feature F5 | #Feature F6 | #Feature F7 | #Feature F8 | #Feature F9 | #Feature F10 | Start | End | PTM |
| --- | --- | --- | --- | --- | --- | --- | --- | --- | --- | --- | --- | --- | --- | --- | --- | --- | --- | --- | --- | --- | --- | --- | --- | --- | --- | --- | --- | --- | --- | --- | --- | --- | --- | --- | --- |
| R.GC(+57.02)IDVC(+57.02)PK.D | N | 56.82 | 947.4205 | 8 | -2.7 | 474.7162 | 2 | 11.15 | 8 | F8:2003 | 05112019\_RID\_1313\_NaNaRj\_F8.raw | 1E5 | 1.9E5 | 1.09E4 | 8.89E4 | 5.11E5 | 3.82E8 |  | 3.84E7 | 5.76E5 | 1.59E4 | 23 | 1 | 1 | 1 | 1 | 1 | 14 | 0 | 2 | 1 | 1 | 37 | 44 | Carbamidomethylation |
| K.MFMVSTSTVPVK.R | Y | 45.15 | 1325.6724 | 12 | -7.3 | 663.8386 | 2 | 16.85 | 8 | F8:5433 | 05112019\_RID\_1313\_NaNaRj\_F8.raw |  |  |  |  |  |  |  | 1.84E5 |  |  | 1 | 0 | 0 | 0 | 0 | 0 | 0 | 0 | 1 | 0 | 0 | 24 | 35 |  |
| total 2 peptides |  |  |  |  |  |  |  |  |  |  |  |  |  |  |  |  |  |  |  |  |  |  |  |  |  |  |  |  |  |  |  |  |  |  |  |
| --- | --- | --- | --- | --- | --- | --- | --- | --- | --- | --- | --- | --- | --- | --- | --- | --- | --- | --- | --- | --- | --- | --- | --- | --- | --- | --- | --- | --- | --- | --- | --- | --- | --- | --- | --- |

B6EWW8|V5NTD\_GLOBR

back to list

  

| Protein Coverage
| Supporting Peptides
|

Protein Coverage:

Supporting Peptides:

| Peptide | Uniq | -10lgP | Mass | Length | ppm | m/z | z | RT | Fraction | Scan | Source File | Area F1 | Area F2 | Area F3 | Area F4 | Area F5 | Area F6 | Area F7 | Area F8 | Area F9 | Area F10 | #Feature | #Feature F1 | #Feature F2 | #Feature F3 | #Feature F4 | #Feature F5 | #Feature F6 | #Feature F7 | #Feature F8 | #Feature F9 | #Feature F10 | Start | End | PTM |
| --- | --- | --- | --- | --- | --- | --- | --- | --- | --- | --- | --- | --- | --- | --- | --- | --- | --- | --- | --- | --- | --- | --- | --- | --- | --- | --- | --- | --- | --- | --- | --- | --- | --- | --- | --- |
| R.FHEC(+57.02)NLGNLIC(+57.02)DAVIYNNVR.H | Y | 68.63 | 2420.1365 | 20 | 0.7 | 807.7200 | 3 | 28.68 | 6 | F6:12849 | 05112019\_RID\_1313\_NaNaRj\_F6.raw |  |  |  |  |  | 1.15E5 | 3.95E5 |  |  |  | 2 | 0 | 0 | 0 | 0 | 0 | 1 | 1 | 0 | 0 | 0 | 369 | 388 | Carbamidomethylation |
| total 1 peptides |  |  |  |  |  |  |  |  |  |  |  |  |  |  |  |  |  |  |  |  |  |  |  |  |  |  |  |  |  |  |  |  |  |  |  |
| --- | --- | --- | --- | --- | --- | --- | --- | --- | --- | --- | --- | --- | --- | --- | --- | --- | --- | --- | --- | --- | --- | --- | --- | --- | --- | --- | --- | --- | --- | --- | --- | --- | --- | --- | --- |

P35030|TRY3\_HUMAN

back to list

  

| Protein Coverage
| Supporting Peptides
|

Protein Coverage:

Supporting Peptides:

| Peptide | Uniq | -10lgP | Mass | Length | ppm | m/z | z | RT | Fraction | Scan | Source File | Area F1 | Area F2 | Area F3 | Area F4 | Area F5 | Area F6 | Area F7 | Area F8 | Area F9 | Area F10 | #Feature | #Feature F1 | #Feature F2 | #Feature F3 | #Feature F4 | #Feature F5 | #Feature F6 | #Feature F7 | #Feature F8 | #Feature F9 | #Feature F10 | Start | End | PTM |
| --- | --- | --- | --- | --- | --- | --- | --- | --- | --- | --- | --- | --- | --- | --- | --- | --- | --- | --- | --- | --- | --- | --- | --- | --- | --- | --- | --- | --- | --- | --- | --- | --- | --- | --- | --- |
| K.VLEGNEQFINAAK.I | N | 50.49 | 1431.7357 | 13 | 0.8 | 716.8757 | 2 | 40.68 | 2 | F2:12665 | 09012020\_NaNaRj\_F2.raw |  | 1.17E4 |  |  |  |  |  |  |  |  | 1 | 0 | 1 | 0 | 0 | 0 | 0 | 0 | 0 | 0 | 0 | 137 | 149 |  |
| K.VLEGNEQFIN.A | Y | 43.71 | 1161.5665 | 10 | 0.3 | 581.7907 | 2 | 50.08 | 2 | F2:16777 | 09012020\_NaNaRj\_F2.raw |  | 4.46E7 |  |  |  |  |  |  |  |  | 1 | 0 | 1 | 0 | 0 | 0 | 0 | 0 | 0 | 0 | 0 | 137 | 146 |  |
| K.VLEGNEQFINAA.K | Y | 42.76 | 1303.6407 | 12 | 0.4 | 652.8279 | 2 | 55.72 | 2 | F2:18574 | 09012020\_NaNaRj\_F2.raw |  | 8.66E6 |  |  |  |  |  |  |  |  | 1 | 0 | 1 | 0 | 0 | 0 | 0 | 0 | 0 | 0 | 0 | 137 | 148 |  |
| total 3 peptides |  |  |  |  |  |  |  |  |  |  |  |  |  |  |  |  |  |  |  |  |  |  |  |  |  |  |  |  |  |  |  |  |  |  |  |
| --- | --- | --- | --- | --- | --- | --- | --- | --- | --- | --- | --- | --- | --- | --- | --- | --- | --- | --- | --- | --- | --- | --- | --- | --- | --- | --- | --- | --- | --- | --- | --- | --- | --- | --- | --- |

O62823|CASA1\_BUBBU

back to list

  

| Protein Coverage
| Supporting Peptides
|

Protein Coverage:

Supporting Peptides:

| Peptide | Uniq | -10lgP | Mass | Length | ppm | m/z | z | RT | Fraction | Scan | Source File | Area F1 | Area F2 | Area F3 | Area F4 | Area F5 | Area F6 | Area F7 | Area F8 | Area F9 | Area F10 | #Feature | #Feature F1 | #Feature F2 | #Feature F3 | #Feature F4 | #Feature F5 | #Feature F6 | #Feature F7 | #Feature F8 | #Feature F9 | #Feature F10 | Start | End | PTM |
| --- | --- | --- | --- | --- | --- | --- | --- | --- | --- | --- | --- | --- | --- | --- | --- | --- | --- | --- | --- | --- | --- | --- | --- | --- | --- | --- | --- | --- | --- | --- | --- | --- | --- | --- | --- |
| R.FFVAPFPEVFGK.E | Y | 59.53 | 1383.7227 | 12 | 0.7 | 692.8691 | 2 | 41.38 | 8 | F8:15912 | 05112019\_RID\_1313\_NaNaRj\_F8.raw |  |  |  |  |  |  |  | 3.78E4 |  |  | 1 | 0 | 0 | 0 | 0 | 0 | 0 | 0 | 1 | 0 | 0 | 38 | 49 |  |
| R.YLGYLEQLLR.L | Y | 51.42 | 1266.6971 | 10 | 0.1 | 634.3559 | 2 | 33.84 | 8 | F8:12607 | 05112019\_RID\_1313\_NaNaRj\_F8.raw |  |  |  |  |  |  |  | 4.03E4 |  |  | 1 | 0 | 0 | 0 | 0 | 0 | 0 | 0 | 1 | 0 | 0 | 106 | 115 |  |
| total 2 peptides |  |  |  |  |  |  |  |  |  |  |  |  |  |  |  |  |  |  |  |  |  |  |  |  |  |  |  |  |  |  |  |  |  |  |  |
| --- | --- | --- | --- | --- | --- | --- | --- | --- | --- | --- | --- | --- | --- | --- | --- | --- | --- | --- | --- | --- | --- | --- | --- | --- | --- | --- | --- | --- | --- | --- | --- | --- | --- | --- | --- |

#CONTAM#CAS1\_BOVIN|

back to list

  

| Protein Coverage
| Supporting Peptides
|

Protein Coverage:

Supporting Peptides:

| Peptide | Uniq | -10lgP | Mass | Length | ppm | m/z | z | RT | Fraction | Scan | Source File | Area F1 | Area F2 | Area F3 | Area F4 | Area F5 | Area F6 | Area F7 | Area F8 | Area F9 | Area F10 | #Feature | #Feature F1 | #Feature F2 | #Feature F3 | #Feature F4 | #Feature F5 | #Feature F6 | #Feature F7 | #Feature F8 | #Feature F9 | #Feature F10 | Start | End | PTM |
| --- | --- | --- | --- | --- | --- | --- | --- | --- | --- | --- | --- | --- | --- | --- | --- | --- | --- | --- | --- | --- | --- | --- | --- | --- | --- | --- | --- | --- | --- | --- | --- | --- | --- | --- | --- |
| R.FFVAPFPEVFGK.E | Y | 59.53 | 1383.7227 | 12 | 0.7 | 692.8691 | 2 | 41.38 | 8 | F8:15912 | 05112019\_RID\_1313\_NaNaRj\_F8.raw |  |  |  |  |  |  |  | 3.78E4 |  |  | 1 | 0 | 0 | 0 | 0 | 0 | 0 | 0 | 1 | 0 | 0 | 38 | 49 |  |
| R.YLGYLEQLLR.L | Y | 51.42 | 1266.6971 | 10 | 0.1 | 634.3559 | 2 | 33.84 | 8 | F8:12607 | 05112019\_RID\_1313\_NaNaRj\_F8.raw |  |  |  |  |  |  |  | 4.03E4 |  |  | 1 | 0 | 0 | 0 | 0 | 0 | 0 | 0 | 1 | 0 | 0 | 106 | 115 |  |
| total 2 peptides |  |  |  |  |  |  |  |  |  |  |  |  |  |  |  |  |  |  |  |  |  |  |  |  |  |  |  |  |  |  |  |  |  |  |  |
| --- | --- | --- | --- | --- | --- | --- | --- | --- | --- | --- | --- | --- | --- | --- | --- | --- | --- | --- | --- | --- | --- | --- | --- | --- | --- | --- | --- | --- | --- | --- | --- | --- | --- | --- | --- |

P02662|CASA1\_BOVIN

back to list

  

| Protein Coverage
| Supporting Peptides
|

Protein Coverage:

Supporting Peptides:

| Peptide | Uniq | -10lgP | Mass | Length | ppm | m/z | z | RT | Fraction | Scan | Source File | Area F1 | Area F2 | Area F3 | Area F4 | Area F5 | Area F6 | Area F7 | Area F8 | Area F9 | Area F10 | #Feature | #Feature F1 | #Feature F2 | #Feature F3 | #Feature F4 | #Feature F5 | #Feature F6 | #Feature F7 | #Feature F8 | #Feature F9 | #Feature F10 | Start | End | PTM |
| --- | --- | --- | --- | --- | --- | --- | --- | --- | --- | --- | --- | --- | --- | --- | --- | --- | --- | --- | --- | --- | --- | --- | --- | --- | --- | --- | --- | --- | --- | --- | --- | --- | --- | --- | --- |
| R.FFVAPFPEVFGK.E | Y | 59.53 | 1383.7227 | 12 | 0.7 | 692.8691 | 2 | 41.38 | 8 | F8:15912 | 05112019\_RID\_1313\_NaNaRj\_F8.raw |  |  |  |  |  |  |  | 3.78E4 |  |  | 1 | 0 | 0 | 0 | 0 | 0 | 0 | 0 | 1 | 0 | 0 | 38 | 49 |  |
| R.YLGYLEQLLR.L | Y | 51.42 | 1266.6971 | 10 | 0.1 | 634.3559 | 2 | 33.84 | 8 | F8:12607 | 05112019\_RID\_1313\_NaNaRj\_F8.raw |  |  |  |  |  |  |  | 4.03E4 |  |  | 1 | 0 | 0 | 0 | 0 | 0 | 0 | 0 | 1 | 0 | 0 | 106 | 115 |  |
| total 2 peptides |  |  |  |  |  |  |  |  |  |  |  |  |  |  |  |  |  |  |  |  |  |  |  |  |  |  |  |  |  |  |  |  |  |  |  |
| --- | --- | --- | --- | --- | --- | --- | --- | --- | --- | --- | --- | --- | --- | --- | --- | --- | --- | --- | --- | --- | --- | --- | --- | --- | --- | --- | --- | --- | --- | --- | --- | --- | --- | --- | --- |

#CONTAM#KRHB4\_HUMAN|

back to list

  

| Protein Coverage
| Supporting Peptides
|

Protein Coverage:

Supporting Peptides:

| Peptide | Uniq | -10lgP | Mass | Length | ppm | m/z | z | RT | Fraction | Scan | Source File | Area F1 | Area F2 | Area F3 | Area F4 | Area F5 | Area F6 | Area F7 | Area F8 | Area F9 | Area F10 | #Feature | #Feature F1 | #Feature F2 | #Feature F3 | #Feature F4 | #Feature F5 | #Feature F6 | #Feature F7 | #Feature F8 | #Feature F9 | #Feature F10 | Start | End | PTM |
| --- | --- | --- | --- | --- | --- | --- | --- | --- | --- | --- | --- | --- | --- | --- | --- | --- | --- | --- | --- | --- | --- | --- | --- | --- | --- | --- | --- | --- | --- | --- | --- | --- | --- | --- | --- |
| K.FASFIDK.V | N | 47.57 | 826.4225 | 7 | 0.0 | 414.2185 | 2 | 39.16 | 2 | F2:12055 | 09012020\_NaNaRj\_F2.raw | 6.35E3 | 4.99E6 |  |  | 4.92E5 |  |  |  | 5.38E3 |  | 4 | 1 | 1 | 0 | 0 | 1 | 0 | 0 | 0 | 1 | 0 | 176 | 182 |  |
| K.DVDAAFM(+15.99)NK.S | N | 46.19 | 1025.4487 | 9 | 1.5 | 513.7324 | 2 | 25.82 | 2 | F2:6545 | 09012020\_NaNaRj\_F2.raw |  | 6.11E4 |  |  |  |  |  |  |  |  | 1 | 0 | 1 | 0 | 0 | 0 | 0 | 0 | 0 | 0 | 0 | 273 | 281 | Oxidation (M) |
| R.VGGVGVPAAPSITAVTVNK.S | Y | 41.09 | 1735.9832 | 19 | 0.5 | 868.9993 | 2 | 14.71 | 3 | F3:3321 | 05112019\_RID\_1313\_NaNaRj\_F3.raw |  |  | 5.28E3 |  |  |  |  |  |  |  | 1 | 0 | 0 | 1 | 0 | 0 | 0 | 0 | 0 | 0 | 0 | 126 | 144 |  |
| total 3 peptides |  |  |  |  |  |  |  |  |  |  |  |  |  |  |  |  |  |  |  |  |  |  |  |  |  |  |  |  |  |  |  |  |  |  |  |
| --- | --- | --- | --- | --- | --- | --- | --- | --- | --- | --- | --- | --- | --- | --- | --- | --- | --- | --- | --- | --- | --- | --- | --- | --- | --- | --- | --- | --- | --- | --- | --- | --- | --- | --- | --- |

Q9NSB2|KRT84\_HUMAN

back to list

  

| Protein Coverage
| Supporting Peptides
|

Protein Coverage:

Supporting Peptides:

| Peptide | Uniq | -10lgP | Mass | Length | ppm | m/z | z | RT | Fraction | Scan | Source File | Area F1 | Area F2 | Area F3 | Area F4 | Area F5 | Area F6 | Area F7 | Area F8 | Area F9 | Area F10 | #Feature | #Feature F1 | #Feature F2 | #Feature F3 | #Feature F4 | #Feature F5 | #Feature F6 | #Feature F7 | #Feature F8 | #Feature F9 | #Feature F10 | Start | End | PTM |
| --- | --- | --- | --- | --- | --- | --- | --- | --- | --- | --- | --- | --- | --- | --- | --- | --- | --- | --- | --- | --- | --- | --- | --- | --- | --- | --- | --- | --- | --- | --- | --- | --- | --- | --- | --- |
| K.FASFIDK.V | N | 47.57 | 826.4225 | 7 | 0.0 | 414.2185 | 2 | 39.16 | 2 | F2:12055 | 09012020\_NaNaRj\_F2.raw | 6.35E3 | 4.99E6 |  |  | 4.92E5 |  |  |  | 5.38E3 |  | 4 | 1 | 1 | 0 | 0 | 1 | 0 | 0 | 0 | 1 | 0 | 176 | 182 |  |
| K.DVDAAFM(+15.99)NK.S | N | 46.19 | 1025.4487 | 9 | 1.5 | 513.7324 | 2 | 25.82 | 2 | F2:6545 | 09012020\_NaNaRj\_F2.raw |  | 6.11E4 |  |  |  |  |  |  |  |  | 1 | 0 | 1 | 0 | 0 | 0 | 0 | 0 | 0 | 0 | 0 | 273 | 281 | Oxidation (M) |
| R.VGGVGVPAAPSITAVTVNK.S | Y | 41.09 | 1735.9832 | 19 | 0.5 | 868.9993 | 2 | 14.71 | 3 | F3:3321 | 05112019\_RID\_1313\_NaNaRj\_F3.raw |  |  | 5.28E3 |  |  |  |  |  |  |  | 1 | 0 | 0 | 1 | 0 | 0 | 0 | 0 | 0 | 0 | 0 | 126 | 144 |  |
| total 3 peptides |  |  |  |  |  |  |  |  |  |  |  |  |  |  |  |  |  |  |  |  |  |  |  |  |  |  |  |  |  |  |  |  |  |  |  |
| --- | --- | --- | --- | --- | --- | --- | --- | --- | --- | --- | --- | --- | --- | --- | --- | --- | --- | --- | --- | --- | --- | --- | --- | --- | --- | --- | --- | --- | --- | --- | --- | --- | --- | --- | --- |

Q9TSI0|CASB\_BUBBU

back to list

  

| Protein Coverage
| Supporting Peptides
|

Protein Coverage:

Supporting Peptides:

| Peptide | Uniq | -10lgP | Mass | Length | ppm | m/z | z | RT | Fraction | Scan | Source File | Area F1 | Area F2 | Area F3 | Area F4 | Area F5 | Area F6 | Area F7 | Area F8 | Area F9 | Area F10 | #Feature | #Feature F1 | #Feature F2 | #Feature F3 | #Feature F4 | #Feature F5 | #Feature F6 | #Feature F7 | #Feature F8 | #Feature F9 | #Feature F10 | Start | End | PTM |
| --- | --- | --- | --- | --- | --- | --- | --- | --- | --- | --- | --- | --- | --- | --- | --- | --- | --- | --- | --- | --- | --- | --- | --- | --- | --- | --- | --- | --- | --- | --- | --- | --- | --- | --- | --- |
| L.YQEPVLGPVR.G | Y | 43.48 | 1156.6240 | 10 | 0.8 | 579.3198 | 2 | 11.83 | 9 | F9:2509 | 05112019\_RID\_1313\_NaNaRj\_F9.raw |  |  |  |  |  |  |  |  | 1.17E4 |  | 1 | 0 | 0 | 0 | 0 | 0 | 0 | 0 | 0 | 1 | 0 | 208 | 217 |  |
| total 1 peptides |  |  |  |  |  |  |  |  |  |  |  |  |  |  |  |  |  |  |  |  |  |  |  |  |  |  |  |  |  |  |  |  |  |  |  |
| --- | --- | --- | --- | --- | --- | --- | --- | --- | --- | --- | --- | --- | --- | --- | --- | --- | --- | --- | --- | --- | --- | --- | --- | --- | --- | --- | --- | --- | --- | --- | --- | --- | --- | --- | --- |

Q9NZT1|CALL5\_HUMAN

back to list

  

| Protein Coverage
| Supporting Peptides
|

Protein Coverage:

Supporting Peptides:

| Peptide | Uniq | -10lgP | Mass | Length | ppm | m/z | z | RT | Fraction | Scan | Source File | Area F1 | Area F2 | Area F3 | Area F4 | Area F5 | Area F6 | Area F7 | Area F8 | Area F9 | Area F10 | #Feature | #Feature F1 | #Feature F2 | #Feature F3 | #Feature F4 | #Feature F5 | #Feature F6 | #Feature F7 | #Feature F8 | #Feature F9 | #Feature F10 | Start | End | PTM |
| --- | --- | --- | --- | --- | --- | --- | --- | --- | --- | --- | --- | --- | --- | --- | --- | --- | --- | --- | --- | --- | --- | --- | --- | --- | --- | --- | --- | --- | --- | --- | --- | --- | --- | --- | --- |
| K.AFSAVDTDGNGTINAQELGAALK.A | Y | 60.68 | 2262.1128 | 23 | 0.7 | 1132.0645 | 2 | 20.48 | 8 | F8:7010 | 05112019\_RID\_1313\_NaNaRj\_F8.raw |  |  |  |  |  |  |  | 1.39E4 |  |  | 1 | 0 | 0 | 0 | 0 | 0 | 0 | 0 | 1 | 0 | 0 | 16 | 38 |  |
| total 1 peptides |  |  |  |  |  |  |  |  |  |  |  |  |  |  |  |  |  |  |  |  |  |  |  |  |  |  |  |  |  |  |  |  |  |  |  |
| --- | --- | --- | --- | --- | --- | --- | --- | --- | --- | --- | --- | --- | --- | --- | --- | --- | --- | --- | --- | --- | --- | --- | --- | --- | --- | --- | --- | --- | --- | --- | --- | --- | --- | --- | --- |

P82849|3S1B2\_NAJKA

back to list

  

| Protein Coverage
| Supporting Peptides
|

Protein Coverage:

Supporting Peptides:

| Peptide | Uniq | -10lgP | Mass | Length | ppm | m/z | z | RT | Fraction | Scan | Source File | Area F1 | Area F2 | Area F3 | Area F4 | Area F5 | Area F6 | Area F7 | Area F8 | Area F9 | Area F10 | #Feature | #Feature F1 | #Feature F2 | #Feature F3 | #Feature F4 | #Feature F5 | #Feature F6 | #Feature F7 | #Feature F8 | #Feature F9 | #Feature F10 | Start | End | PTM |
| --- | --- | --- | --- | --- | --- | --- | --- | --- | --- | --- | --- | --- | --- | --- | --- | --- | --- | --- | --- | --- | --- | --- | --- | --- | --- | --- | --- | --- | --- | --- | --- | --- | --- | --- | --- |
| LEC(+57.02)HNQQSSQTPTTTGC(+57.02).S | Y | 78.89 | 1947.8052 | 17 | -0.3 | 974.9095 | 2 | 10.64 | 3 | F3:1595 | 05112019\_RID\_1313\_NaNaRj\_F3.raw |  |  | 2.9E6 |  |  |  |  |  |  |  | 1 | 0 | 0 | 1 | 0 | 0 | 0 | 0 | 0 | 0 | 0 | 1 | 17 | Carbamidomethylation |
| total 1 peptides |  |  |  |  |  |  |  |  |  |  |  |  |  |  |  |  |  |  |  |  |  |  |  |  |  |  |  |  |  |  |  |  |  |  |  |
| --- | --- | --- | --- | --- | --- | --- | --- | --- | --- | --- | --- | --- | --- | --- | --- | --- | --- | --- | --- | --- | --- | --- | --- | --- | --- | --- | --- | --- | --- | --- | --- | --- | --- | --- | --- |

P10981|ACT5\_DROME

back to list

  

| Protein Coverage
| Supporting Peptides
|

Protein Coverage:

Supporting Peptides:

| Peptide | Uniq | -10lgP | Mass | Length | ppm | m/z | z | RT | Fraction | Scan | Source File | Area F1 | Area F2 | Area F3 | Area F4 | Area F5 | Area F6 | Area F7 | Area F8 | Area F9 | Area F10 | #Feature | #Feature F1 | #Feature F2 | #Feature F3 | #Feature F4 | #Feature F5 | #Feature F6 | #Feature F7 | #Feature F8 | #Feature F9 | #Feature F10 | Start | End | PTM |
| --- | --- | --- | --- | --- | --- | --- | --- | --- | --- | --- | --- | --- | --- | --- | --- | --- | --- | --- | --- | --- | --- | --- | --- | --- | --- | --- | --- | --- | --- | --- | --- | --- | --- | --- | --- |
| K.AGFAGDDAPR.A | Y | 50.72 | 975.4410 | 10 | -0.8 | 488.7274 | 2 | 19.85 | 2 | F2:4188 | 09012020\_NaNaRj\_F2.raw |  | 2.81E4 |  |  |  |  |  |  |  |  | 1 | 0 | 1 | 0 | 0 | 0 | 0 | 0 | 0 | 0 | 0 | 20 | 29 |  |
| total 1 peptides |  |  |  |  |  |  |  |  |  |  |  |  |  |  |  |  |  |  |  |  |  |  |  |  |  |  |  |  |  |  |  |  |  |  |  |
| --- | --- | --- | --- | --- | --- | --- | --- | --- | --- | --- | --- | --- | --- | --- | --- | --- | --- | --- | --- | --- | --- | --- | --- | --- | --- | --- | --- | --- | --- | --- | --- | --- | --- | --- | --- |

P04752|ACT3\_XENLA

back to list

  

| Protein Coverage
| Supporting Peptides
|

Protein Coverage:

Supporting Peptides:

| Peptide | Uniq | -10lgP | Mass | Length | ppm | m/z | z | RT | Fraction | Scan | Source File | Area F1 | Area F2 | Area F3 | Area F4 | Area F5 | Area F6 | Area F7 | Area F8 | Area F9 | Area F10 | #Feature | #Feature F1 | #Feature F2 | #Feature F3 | #Feature F4 | #Feature F5 | #Feature F6 | #Feature F7 | #Feature F8 | #Feature F9 | #Feature F10 | Start | End | PTM |
| --- | --- | --- | --- | --- | --- | --- | --- | --- | --- | --- | --- | --- | --- | --- | --- | --- | --- | --- | --- | --- | --- | --- | --- | --- | --- | --- | --- | --- | --- | --- | --- | --- | --- | --- | --- |
| K.AGFAGDDAPR.A | Y | 50.72 | 975.4410 | 10 | -0.8 | 488.7274 | 2 | 19.85 | 2 | F2:4188 | 09012020\_NaNaRj\_F2.raw |  | 2.81E4 |  |  |  |  |  |  |  |  | 1 | 0 | 1 | 0 | 0 | 0 | 0 | 0 | 0 | 0 | 0 | 21 | 30 |  |
| total 1 peptides |  |  |  |  |  |  |  |  |  |  |  |  |  |  |  |  |  |  |  |  |  |  |  |  |  |  |  |  |  |  |  |  |  |  |  |
| --- | --- | --- | --- | --- | --- | --- | --- | --- | --- | --- | --- | --- | --- | --- | --- | --- | --- | --- | --- | --- | --- | --- | --- | --- | --- | --- | --- | --- | --- | --- | --- | --- | --- | --- | --- |

P62737|ACTA\_MOUSE

back to list

  

| Protein Coverage
| Supporting Peptides
|

Protein Coverage:

Supporting Peptides:

| Peptide | Uniq | -10lgP | Mass | Length | ppm | m/z | z | RT | Fraction | Scan | Source File | Area F1 | Area F2 | Area F3 | Area F4 | Area F5 | Area F6 | Area F7 | Area F8 | Area F9 | Area F10 | #Feature | #Feature F1 | #Feature F2 | #Feature F3 | #Feature F4 | #Feature F5 | #Feature F6 | #Feature F7 | #Feature F8 | #Feature F9 | #Feature F10 | Start | End | PTM |
| --- | --- | --- | --- | --- | --- | --- | --- | --- | --- | --- | --- | --- | --- | --- | --- | --- | --- | --- | --- | --- | --- | --- | --- | --- | --- | --- | --- | --- | --- | --- | --- | --- | --- | --- | --- |
| K.AGFAGDDAPR.A | Y | 50.72 | 975.4410 | 10 | -0.8 | 488.7274 | 2 | 19.85 | 2 | F2:4188 | 09012020\_NaNaRj\_F2.raw |  | 2.81E4 |  |  |  |  |  |  |  |  | 1 | 0 | 1 | 0 | 0 | 0 | 0 | 0 | 0 | 0 | 0 | 21 | 30 |  |
| total 1 peptides |  |  |  |  |  |  |  |  |  |  |  |  |  |  |  |  |  |  |  |  |  |  |  |  |  |  |  |  |  |  |  |  |  |  |  |
| --- | --- | --- | --- | --- | --- | --- | --- | --- | --- | --- | --- | --- | --- | --- | --- | --- | --- | --- | --- | --- | --- | --- | --- | --- | --- | --- | --- | --- | --- | --- | --- | --- | --- | --- | --- |

P60710|ACTB\_MOUSE

back to list

  

| Protein Coverage
| Supporting Peptides
|

Protein Coverage:

Supporting Peptides:

| Peptide | Uniq | -10lgP | Mass | Length | ppm | m/z | z | RT | Fraction | Scan | Source File | Area F1 | Area F2 | Area F3 | Area F4 | Area F5 | Area F6 | Area F7 | Area F8 | Area F9 | Area F10 | #Feature | #Feature F1 | #Feature F2 | #Feature F3 | #Feature F4 | #Feature F5 | #Feature F6 | #Feature F7 | #Feature F8 | #Feature F9 | #Feature F10 | Start | End | PTM |
| --- | --- | --- | --- | --- | --- | --- | --- | --- | --- | --- | --- | --- | --- | --- | --- | --- | --- | --- | --- | --- | --- | --- | --- | --- | --- | --- | --- | --- | --- | --- | --- | --- | --- | --- | --- |
| K.AGFAGDDAPR.A | Y | 50.72 | 975.4410 | 10 | -0.8 | 488.7274 | 2 | 19.85 | 2 | F2:4188 | 09012020\_NaNaRj\_F2.raw |  | 2.81E4 |  |  |  |  |  |  |  |  | 1 | 0 | 1 | 0 | 0 | 0 | 0 | 0 | 0 | 0 | 0 | 19 | 28 |  |
| total 1 peptides |  |  |  |  |  |  |  |  |  |  |  |  |  |  |  |  |  |  |  |  |  |  |  |  |  |  |  |  |  |  |  |  |  |  |  |
| --- | --- | --- | --- | --- | --- | --- | --- | --- | --- | --- | --- | --- | --- | --- | --- | --- | --- | --- | --- | --- | --- | --- | --- | --- | --- | --- | --- | --- | --- | --- | --- | --- | --- | --- | --- |

O18840|ACTB\_CANLF

back to list

  

| Protein Coverage
| Supporting Peptides
|

Protein Coverage:

Supporting Peptides:

| Peptide | Uniq | -10lgP | Mass | Length | ppm | m/z | z | RT | Fraction | Scan | Source File | Area F1 | Area F2 | Area F3 | Area F4 | Area F5 | Area F6 | Area F7 | Area F8 | Area F9 | Area F10 | #Feature | #Feature F1 | #Feature F2 | #Feature F3 | #Feature F4 | #Feature F5 | #Feature F6 | #Feature F7 | #Feature F8 | #Feature F9 | #Feature F10 | Start | End | PTM |
| --- | --- | --- | --- | --- | --- | --- | --- | --- | --- | --- | --- | --- | --- | --- | --- | --- | --- | --- | --- | --- | --- | --- | --- | --- | --- | --- | --- | --- | --- | --- | --- | --- | --- | --- | --- |
| K.AGFAGDDAPR.A | Y | 50.72 | 975.4410 | 10 | -0.8 | 488.7274 | 2 | 19.85 | 2 | F2:4188 | 09012020\_NaNaRj\_F2.raw |  | 2.81E4 |  |  |  |  |  |  |  |  | 1 | 0 | 1 | 0 | 0 | 0 | 0 | 0 | 0 | 0 | 0 | 19 | 28 |  |
| total 1 peptides |  |  |  |  |  |  |  |  |  |  |  |  |  |  |  |  |  |  |  |  |  |  |  |  |  |  |  |  |  |  |  |  |  |  |  |
| --- | --- | --- | --- | --- | --- | --- | --- | --- | --- | --- | --- | --- | --- | --- | --- | --- | --- | --- | --- | --- | --- | --- | --- | --- | --- | --- | --- | --- | --- | --- | --- | --- | --- | --- | --- |

Q5R6G0|ACTB\_PONAB

back to list

  

| Protein Coverage
| Supporting Peptides
|

Protein Coverage:

Supporting Peptides:

| Peptide | Uniq | -10lgP | Mass | Length | ppm | m/z | z | RT | Fraction | Scan | Source File | Area F1 | Area F2 | Area F3 | Area F4 | Area F5 | Area F6 | Area F7 | Area F8 | Area F9 | Area F10 | #Feature | #Feature F1 | #Feature F2 | #Feature F3 | #Feature F4 | #Feature F5 | #Feature F6 | #Feature F7 | #Feature F8 | #Feature F9 | #Feature F10 | Start | End | PTM |
| --- | --- | --- | --- | --- | --- | --- | --- | --- | --- | --- | --- | --- | --- | --- | --- | --- | --- | --- | --- | --- | --- | --- | --- | --- | --- | --- | --- | --- | --- | --- | --- | --- | --- | --- | --- |
| K.AGFAGDDAPR.A | Y | 50.72 | 975.4410 | 10 | -0.8 | 488.7274 | 2 | 19.85 | 2 | F2:4188 | 09012020\_NaNaRj\_F2.raw |  | 2.81E4 |  |  |  |  |  |  |  |  | 1 | 0 | 1 | 0 | 0 | 0 | 0 | 0 | 0 | 0 | 0 | 19 | 28 |  |
| total 1 peptides |  |  |  |  |  |  |  |  |  |  |  |  |  |  |  |  |  |  |  |  |  |  |  |  |  |  |  |  |  |  |  |  |  |  |  |
| --- | --- | --- | --- | --- | --- | --- | --- | --- | --- | --- | --- | --- | --- | --- | --- | --- | --- | --- | --- | --- | --- | --- | --- | --- | --- | --- | --- | --- | --- | --- | --- | --- | --- | --- | --- |

Q711N9|ACTB\_MESAU

back to list

  

| Protein Coverage
| Supporting Peptides
|

Protein Coverage:

Supporting Peptides:

| Peptide | Uniq | -10lgP | Mass | Length | ppm | m/z | z | RT | Fraction | Scan | Source File | Area F1 | Area F2 | Area F3 | Area F4 | Area F5 | Area F6 | Area F7 | Area F8 | Area F9 | Area F10 | #Feature | #Feature F1 | #Feature F2 | #Feature F3 | #Feature F4 | #Feature F5 | #Feature F6 | #Feature F7 | #Feature F8 | #Feature F9 | #Feature F10 | Start | End | PTM |
| --- | --- | --- | --- | --- | --- | --- | --- | --- | --- | --- | --- | --- | --- | --- | --- | --- | --- | --- | --- | --- | --- | --- | --- | --- | --- | --- | --- | --- | --- | --- | --- | --- | --- | --- | --- |
| K.AGFAGDDAPR.A | Y | 50.72 | 975.4410 | 10 | -0.8 | 488.7274 | 2 | 19.85 | 2 | F2:4188 | 09012020\_NaNaRj\_F2.raw |  | 2.81E4 |  |  |  |  |  |  |  |  | 1 | 0 | 1 | 0 | 0 | 0 | 0 | 0 | 0 | 0 | 0 | 19 | 28 |  |
| total 1 peptides |  |  |  |  |  |  |  |  |  |  |  |  |  |  |  |  |  |  |  |  |  |  |  |  |  |  |  |  |  |  |  |  |  |  |  |
| --- | --- | --- | --- | --- | --- | --- | --- | --- | --- | --- | --- | --- | --- | --- | --- | --- | --- | --- | --- | --- | --- | --- | --- | --- | --- | --- | --- | --- | --- | --- | --- | --- | --- | --- | --- |

P63260|ACTG\_MOUSE

back to list

  

| Protein Coverage
| Supporting Peptides
|

Protein Coverage:

Supporting Peptides:

| Peptide | Uniq | -10lgP | Mass | Length | ppm | m/z | z | RT | Fraction | Scan | Source File | Area F1 | Area F2 | Area F3 | Area F4 | Area F5 | Area F6 | Area F7 | Area F8 | Area F9 | Area F10 | #Feature | #Feature F1 | #Feature F2 | #Feature F3 | #Feature F4 | #Feature F5 | #Feature F6 | #Feature F7 | #Feature F8 | #Feature F9 | #Feature F10 | Start | End | PTM |
| --- | --- | --- | --- | --- | --- | --- | --- | --- | --- | --- | --- | --- | --- | --- | --- | --- | --- | --- | --- | --- | --- | --- | --- | --- | --- | --- | --- | --- | --- | --- | --- | --- | --- | --- | --- |
| K.AGFAGDDAPR.A | Y | 50.72 | 975.4410 | 10 | -0.8 | 488.7274 | 2 | 19.85 | 2 | F2:4188 | 09012020\_NaNaRj\_F2.raw |  | 2.81E4 |  |  |  |  |  |  |  |  | 1 | 0 | 1 | 0 | 0 | 0 | 0 | 0 | 0 | 0 | 0 | 19 | 28 |  |
| total 1 peptides |  |  |  |  |  |  |  |  |  |  |  |  |  |  |  |  |  |  |  |  |  |  |  |  |  |  |  |  |  |  |  |  |  |  |  |
| --- | --- | --- | --- | --- | --- | --- | --- | --- | --- | --- | --- | --- | --- | --- | --- | --- | --- | --- | --- | --- | --- | --- | --- | --- | --- | --- | --- | --- | --- | --- | --- | --- | --- | --- | --- |

Q5JAK2|ACTG\_PELLE

back to list

  

| Protein Coverage
| Supporting Peptides
|

Protein Coverage:

Supporting Peptides:

| Peptide | Uniq | -10lgP | Mass | Length | ppm | m/z | z | RT | Fraction | Scan | Source File | Area F1 | Area F2 | Area F3 | Area F4 | Area F5 | Area F6 | Area F7 | Area F8 | Area F9 | Area F10 | #Feature | #Feature F1 | #Feature F2 | #Feature F3 | #Feature F4 | #Feature F5 | #Feature F6 | #Feature F7 | #Feature F8 | #Feature F9 | #Feature F10 | Start | End | PTM |
| --- | --- | --- | --- | --- | --- | --- | --- | --- | --- | --- | --- | --- | --- | --- | --- | --- | --- | --- | --- | --- | --- | --- | --- | --- | --- | --- | --- | --- | --- | --- | --- | --- | --- | --- | --- |
| K.AGFAGDDAPR.A | Y | 50.72 | 975.4410 | 10 | -0.8 | 488.7274 | 2 | 19.85 | 2 | F2:4188 | 09012020\_NaNaRj\_F2.raw |  | 2.81E4 |  |  |  |  |  |  |  |  | 1 | 0 | 1 | 0 | 0 | 0 | 0 | 0 | 0 | 0 | 0 | 19 | 28 |  |
| total 1 peptides |  |  |  |  |  |  |  |  |  |  |  |  |  |  |  |  |  |  |  |  |  |  |  |  |  |  |  |  |  |  |  |  |  |  |  |
| --- | --- | --- | --- | --- | --- | --- | --- | --- | --- | --- | --- | --- | --- | --- | --- | --- | --- | --- | --- | --- | --- | --- | --- | --- | --- | --- | --- | --- | --- | --- | --- | --- | --- | --- | --- |

P63261|ACTG\_HUMAN

back to list

  

| Protein Coverage
| Supporting Peptides
|

Protein Coverage:

Supporting Peptides:

| Peptide | Uniq | -10lgP | Mass | Length | ppm | m/z | z | RT | Fraction | Scan | Source File | Area F1 | Area F2 | Area F3 | Area F4 | Area F5 | Area F6 | Area F7 | Area F8 | Area F9 | Area F10 | #Feature | #Feature F1 | #Feature F2 | #Feature F3 | #Feature F4 | #Feature F5 | #Feature F6 | #Feature F7 | #Feature F8 | #Feature F9 | #Feature F10 | Start | End | PTM |
| --- | --- | --- | --- | --- | --- | --- | --- | --- | --- | --- | --- | --- | --- | --- | --- | --- | --- | --- | --- | --- | --- | --- | --- | --- | --- | --- | --- | --- | --- | --- | --- | --- | --- | --- | --- |
| K.AGFAGDDAPR.A | Y | 50.72 | 975.4410 | 10 | -0.8 | 488.7274 | 2 | 19.85 | 2 | F2:4188 | 09012020\_NaNaRj\_F2.raw |  | 2.81E4 |  |  |  |  |  |  |  |  | 1 | 0 | 1 | 0 | 0 | 0 | 0 | 0 | 0 | 0 | 0 | 19 | 28 |  |
| total 1 peptides |  |  |  |  |  |  |  |  |  |  |  |  |  |  |  |  |  |  |  |  |  |  |  |  |  |  |  |  |  |  |  |  |  |  |  |
| --- | --- | --- | --- | --- | --- | --- | --- | --- | --- | --- | --- | --- | --- | --- | --- | --- | --- | --- | --- | --- | --- | --- | --- | --- | --- | --- | --- | --- | --- | --- | --- | --- | --- | --- | --- |

P53505|ACT5\_XENLA

back to list

  

| Protein Coverage
| Supporting Peptides
|

Protein Coverage:

Supporting Peptides:

| Peptide | Uniq | -10lgP | Mass | Length | ppm | m/z | z | RT | Fraction | Scan | Source File | Area F1 | Area F2 | Area F3 | Area F4 | Area F5 | Area F6 | Area F7 | Area F8 | Area F9 | Area F10 | #Feature | #Feature F1 | #Feature F2 | #Feature F3 | #Feature F4 | #Feature F5 | #Feature F6 | #Feature F7 | #Feature F8 | #Feature F9 | #Feature F10 | Start | End | PTM |
| --- | --- | --- | --- | --- | --- | --- | --- | --- | --- | --- | --- | --- | --- | --- | --- | --- | --- | --- | --- | --- | --- | --- | --- | --- | --- | --- | --- | --- | --- | --- | --- | --- | --- | --- | --- |
| K.AGFAGDDAPR.A | Y | 50.72 | 975.4410 | 10 | -0.8 | 488.7274 | 2 | 19.85 | 2 | F2:4188 | 09012020\_NaNaRj\_F2.raw |  | 2.81E4 |  |  |  |  |  |  |  |  | 1 | 0 | 1 | 0 | 0 | 0 | 0 | 0 | 0 | 0 | 0 | 20 | 29 |  |
| total 1 peptides |  |  |  |  |  |  |  |  |  |  |  |  |  |  |  |  |  |  |  |  |  |  |  |  |  |  |  |  |  |  |  |  |  |  |  |
| --- | --- | --- | --- | --- | --- | --- | --- | --- | --- | --- | --- | --- | --- | --- | --- | --- | --- | --- | --- | --- | --- | --- | --- | --- | --- | --- | --- | --- | --- | --- | --- | --- | --- | --- | --- |

P84185|ACT5C\_ANOGA

back to list

  

| Protein Coverage
| Supporting Peptides
|

Protein Coverage:

Supporting Peptides:

| Peptide | Uniq | -10lgP | Mass | Length | ppm | m/z | z | RT | Fraction | Scan | Source File | Area F1 | Area F2 | Area F3 | Area F4 | Area F5 | Area F6 | Area F7 | Area F8 | Area F9 | Area F10 | #Feature | #Feature F1 | #Feature F2 | #Feature F3 | #Feature F4 | #Feature F5 | #Feature F6 | #Feature F7 | #Feature F8 | #Feature F9 | #Feature F10 | Start | End | PTM |
| --- | --- | --- | --- | --- | --- | --- | --- | --- | --- | --- | --- | --- | --- | --- | --- | --- | --- | --- | --- | --- | --- | --- | --- | --- | --- | --- | --- | --- | --- | --- | --- | --- | --- | --- | --- |
| K.AGFAGDDAPR.A | Y | 50.72 | 975.4410 | 10 | -0.8 | 488.7274 | 2 | 19.85 | 2 | F2:4188 | 09012020\_NaNaRj\_F2.raw |  | 2.81E4 |  |  |  |  |  |  |  |  | 1 | 0 | 1 | 0 | 0 | 0 | 0 | 0 | 0 | 0 | 0 | 20 | 29 |  |
| total 1 peptides |  |  |  |  |  |  |  |  |  |  |  |  |  |  |  |  |  |  |  |  |  |  |  |  |  |  |  |  |  |  |  |  |  |  |  |
| --- | --- | --- | --- | --- | --- | --- | --- | --- | --- | --- | --- | --- | --- | --- | --- | --- | --- | --- | --- | --- | --- | --- | --- | --- | --- | --- | --- | --- | --- | --- | --- | --- | --- | --- | --- |

P68135|ACTS\_RABIT

back to list

  

| Protein Coverage
| Supporting Peptides
|

Protein Coverage:

Supporting Peptides:

| Peptide | Uniq | -10lgP | Mass | Length | ppm | m/z | z | RT | Fraction | Scan | Source File | Area F1 | Area F2 | Area F3 | Area F4 | Area F5 | Area F6 | Area F7 | Area F8 | Area F9 | Area F10 | #Feature | #Feature F1 | #Feature F2 | #Feature F3 | #Feature F4 | #Feature F5 | #Feature F6 | #Feature F7 | #Feature F8 | #Feature F9 | #Feature F10 | Start | End | PTM |
| --- | --- | --- | --- | --- | --- | --- | --- | --- | --- | --- | --- | --- | --- | --- | --- | --- | --- | --- | --- | --- | --- | --- | --- | --- | --- | --- | --- | --- | --- | --- | --- | --- | --- | --- | --- |
| K.AGFAGDDAPR.A | Y | 50.72 | 975.4410 | 10 | -0.8 | 488.7274 | 2 | 19.85 | 2 | F2:4188 | 09012020\_NaNaRj\_F2.raw |  | 2.81E4 |  |  |  |  |  |  |  |  | 1 | 0 | 1 | 0 | 0 | 0 | 0 | 0 | 0 | 0 | 0 | 21 | 30 |  |
| total 1 peptides |  |  |  |  |  |  |  |  |  |  |  |  |  |  |  |  |  |  |  |  |  |  |  |  |  |  |  |  |  |  |  |  |  |  |  |
| --- | --- | --- | --- | --- | --- | --- | --- | --- | --- | --- | --- | --- | --- | --- | --- | --- | --- | --- | --- | --- | --- | --- | --- | --- | --- | --- | --- | --- | --- | --- | --- | --- | --- | --- | --- |

P68137|ACTS\_PIG

back to list

  

| Protein Coverage
| Supporting Peptides
|

Protein Coverage:

Supporting Peptides:

| Peptide | Uniq | -10lgP | Mass | Length | ppm | m/z | z | RT | Fraction | Scan | Source File | Area F1 | Area F2 | Area F3 | Area F4 | Area F5 | Area F6 | Area F7 | Area F8 | Area F9 | Area F10 | #Feature | #Feature F1 | #Feature F2 | #Feature F3 | #Feature F4 | #Feature F5 | #Feature F6 | #Feature F7 | #Feature F8 | #Feature F9 | #Feature F10 | Start | End | PTM |
| --- | --- | --- | --- | --- | --- | --- | --- | --- | --- | --- | --- | --- | --- | --- | --- | --- | --- | --- | --- | --- | --- | --- | --- | --- | --- | --- | --- | --- | --- | --- | --- | --- | --- | --- | --- |
| K.AGFAGDDAPR.A | Y | 50.72 | 975.4410 | 10 | -0.8 | 488.7274 | 2 | 19.85 | 2 | F2:4188 | 09012020\_NaNaRj\_F2.raw |  | 2.81E4 |  |  |  |  |  |  |  |  | 1 | 0 | 1 | 0 | 0 | 0 | 0 | 0 | 0 | 0 | 0 | 21 | 30 |  |
| total 1 peptides |  |  |  |  |  |  |  |  |  |  |  |  |  |  |  |  |  |  |  |  |  |  |  |  |  |  |  |  |  |  |  |  |  |  |  |
| --- | --- | --- | --- | --- | --- | --- | --- | --- | --- | --- | --- | --- | --- | --- | --- | --- | --- | --- | --- | --- | --- | --- | --- | --- | --- | --- | --- | --- | --- | --- | --- | --- | --- | --- | --- |

P53455|ACT\_AJECG

back to list

  

| Protein Coverage
| Supporting Peptides
|

Protein Coverage:

Supporting Peptides:

| Peptide | Uniq | -10lgP | Mass | Length | ppm | m/z | z | RT | Fraction | Scan | Source File | Area F1 | Area F2 | Area F3 | Area F4 | Area F5 | Area F6 | Area F7 | Area F8 | Area F9 | Area F10 | #Feature | #Feature F1 | #Feature F2 | #Feature F3 | #Feature F4 | #Feature F5 | #Feature F6 | #Feature F7 | #Feature F8 | #Feature F9 | #Feature F10 | Start | End | PTM |
| --- | --- | --- | --- | --- | --- | --- | --- | --- | --- | --- | --- | --- | --- | --- | --- | --- | --- | --- | --- | --- | --- | --- | --- | --- | --- | --- | --- | --- | --- | --- | --- | --- | --- | --- | --- |
| K.AGFAGDDAPR.A | Y | 50.72 | 975.4410 | 10 | -0.8 | 488.7274 | 2 | 19.85 | 2 | F2:4188 | 09012020\_NaNaRj\_F2.raw |  | 2.81E4 |  |  |  |  |  |  |  |  | 1 | 0 | 1 | 0 | 0 | 0 | 0 | 0 | 0 | 0 | 0 | 19 | 28 |  |
| total 1 peptides |  |  |  |  |  |  |  |  |  |  |  |  |  |  |  |  |  |  |  |  |  |  |  |  |  |  |  |  |  |  |  |  |  |  |  |
| --- | --- | --- | --- | --- | --- | --- | --- | --- | --- | --- | --- | --- | --- | --- | --- | --- | --- | --- | --- | --- | --- | --- | --- | --- | --- | --- | --- | --- | --- | --- | --- | --- | --- | --- | --- |

P83967|ACT6\_DROME

back to list

  

| Protein Coverage
| Supporting Peptides
|

Protein Coverage:

Supporting Peptides:

| Peptide | Uniq | -10lgP | Mass | Length | ppm | m/z | z | RT | Fraction | Scan | Source File | Area F1 | Area F2 | Area F3 | Area F4 | Area F5 | Area F6 | Area F7 | Area F8 | Area F9 | Area F10 | #Feature | #Feature F1 | #Feature F2 | #Feature F3 | #Feature F4 | #Feature F5 | #Feature F6 | #Feature F7 | #Feature F8 | #Feature F9 | #Feature F10 | Start | End | PTM |
| --- | --- | --- | --- | --- | --- | --- | --- | --- | --- | --- | --- | --- | --- | --- | --- | --- | --- | --- | --- | --- | --- | --- | --- | --- | --- | --- | --- | --- | --- | --- | --- | --- | --- | --- | --- |
| K.AGFAGDDAPR.A | Y | 50.72 | 975.4410 | 10 | -0.8 | 488.7274 | 2 | 19.85 | 2 | F2:4188 | 09012020\_NaNaRj\_F2.raw |  | 2.81E4 |  |  |  |  |  |  |  |  | 1 | 0 | 1 | 0 | 0 | 0 | 0 | 0 | 0 | 0 | 0 | 20 | 29 |  |
| total 1 peptides |  |  |  |  |  |  |  |  |  |  |  |  |  |  |  |  |  |  |  |  |  |  |  |  |  |  |  |  |  |  |  |  |  |  |  |
| --- | --- | --- | --- | --- | --- | --- | --- | --- | --- | --- | --- | --- | --- | --- | --- | --- | --- | --- | --- | --- | --- | --- | --- | --- | --- | --- | --- | --- | --- | --- | --- | --- | --- | --- | --- |

P91754|ACT\_LUMRU

back to list

  

| Protein Coverage
| Supporting Peptides
|

Protein Coverage:

Supporting Peptides:

| Peptide | Uniq | -10lgP | Mass | Length | ppm | m/z | z | RT | Fraction | Scan | Source File | Area F1 | Area F2 | Area F3 | Area F4 | Area F5 | Area F6 | Area F7 | Area F8 | Area F9 | Area F10 | #Feature | #Feature F1 | #Feature F2 | #Feature F3 | #Feature F4 | #Feature F5 | #Feature F6 | #Feature F7 | #Feature F8 | #Feature F9 | #Feature F10 | Start | End | PTM |
| --- | --- | --- | --- | --- | --- | --- | --- | --- | --- | --- | --- | --- | --- | --- | --- | --- | --- | --- | --- | --- | --- | --- | --- | --- | --- | --- | --- | --- | --- | --- | --- | --- | --- | --- | --- |
| K.AGFAGDDAPR.A | Y | 50.72 | 975.4410 | 10 | -0.8 | 488.7274 | 2 | 19.85 | 2 | F2:4188 | 09012020\_NaNaRj\_F2.raw |  | 2.81E4 |  |  |  |  |  |  |  |  | 1 | 0 | 1 | 0 | 0 | 0 | 0 | 0 | 0 | 0 | 0 | 16 | 25 |  |
| total 1 peptides |  |  |  |  |  |  |  |  |  |  |  |  |  |  |  |  |  |  |  |  |  |  |  |  |  |  |  |  |  |  |  |  |  |  |  |
| --- | --- | --- | --- | --- | --- | --- | --- | --- | --- | --- | --- | --- | --- | --- | --- | --- | --- | --- | --- | --- | --- | --- | --- | --- | --- | --- | --- | --- | --- | --- | --- | --- | --- | --- | --- |

P18499|ACTF\_STRPU

back to list

  

| Protein Coverage
| Supporting Peptides
|

Protein Coverage:

Supporting Peptides:

| Peptide | Uniq | -10lgP | Mass | Length | ppm | m/z | z | RT | Fraction | Scan | Source File | Area F1 | Area F2 | Area F3 | Area F4 | Area F5 | Area F6 | Area F7 | Area F8 | Area F9 | Area F10 | #Feature | #Feature F1 | #Feature F2 | #Feature F3 | #Feature F4 | #Feature F5 | #Feature F6 | #Feature F7 | #Feature F8 | #Feature F9 | #Feature F10 | Start | End | PTM |
| --- | --- | --- | --- | --- | --- | --- | --- | --- | --- | --- | --- | --- | --- | --- | --- | --- | --- | --- | --- | --- | --- | --- | --- | --- | --- | --- | --- | --- | --- | --- | --- | --- | --- | --- | --- |
| K.AGFAGDDAPR.A | Y | 50.72 | 975.4410 | 10 | -0.8 | 488.7274 | 2 | 19.85 | 2 | F2:4188 | 09012020\_NaNaRj\_F2.raw |  | 2.81E4 |  |  |  |  |  |  |  |  | 1 | 0 | 1 | 0 | 0 | 0 | 0 | 0 | 0 | 0 | 0 | 20 | 29 |  |
| total 1 peptides |  |  |  |  |  |  |  |  |  |  |  |  |  |  |  |  |  |  |  |  |  |  |  |  |  |  |  |  |  |  |  |  |  |  |  |
| --- | --- | --- | --- | --- | --- | --- | --- | --- | --- | --- | --- | --- | --- | --- | --- | --- | --- | --- | --- | --- | --- | --- | --- | --- | --- | --- | --- | --- | --- | --- | --- | --- | --- | --- | --- |

P53474|ACTE\_STRPU

back to list

  

| Protein Coverage
| Supporting Peptides
|

Protein Coverage:

Supporting Peptides:

| Peptide | Uniq | -10lgP | Mass | Length | ppm | m/z | z | RT | Fraction | Scan | Source File | Area F1 | Area F2 | Area F3 | Area F4 | Area F5 | Area F6 | Area F7 | Area F8 | Area F9 | Area F10 | #Feature | #Feature F1 | #Feature F2 | #Feature F3 | #Feature F4 | #Feature F5 | #Feature F6 | #Feature F7 | #Feature F8 | #Feature F9 | #Feature F10 | Start | End | PTM |
| --- | --- | --- | --- | --- | --- | --- | --- | --- | --- | --- | --- | --- | --- | --- | --- | --- | --- | --- | --- | --- | --- | --- | --- | --- | --- | --- | --- | --- | --- | --- | --- | --- | --- | --- | --- |
| K.AGFAGDDAPR.A | Y | 50.72 | 975.4410 | 10 | -0.8 | 488.7274 | 2 | 19.85 | 2 | F2:4188 | 09012020\_NaNaRj\_F2.raw |  | 2.81E4 |  |  |  |  |  |  |  |  | 1 | 0 | 1 | 0 | 0 | 0 | 0 | 0 | 0 | 0 | 0 | 20 | 29 |  |
| total 1 peptides |  |  |  |  |  |  |  |  |  |  |  |  |  |  |  |  |  |  |  |  |  |  |  |  |  |  |  |  |  |  |  |  |  |  |  |
| --- | --- | --- | --- | --- | --- | --- | --- | --- | --- | --- | --- | --- | --- | --- | --- | --- | --- | --- | --- | --- | --- | --- | --- | --- | --- | --- | --- | --- | --- | --- | --- | --- | --- | --- | --- |

P01040|CYTA\_HUMAN

back to list

  

| Protein Coverage
| Supporting Peptides
|

Protein Coverage:

Supporting Peptides:

| Peptide | Uniq | -10lgP | Mass | Length | ppm | m/z | z | RT | Fraction | Scan | Source File | Area F1 | Area F2 | Area F3 | Area F4 | Area F5 | Area F6 | Area F7 | Area F8 | Area F9 | Area F10 | #Feature | #Feature F1 | #Feature F2 | #Feature F3 | #Feature F4 | #Feature F5 | #Feature F6 | #Feature F7 | #Feature F8 | #Feature F9 | #Feature F10 | Start | End | PTM |
| --- | --- | --- | --- | --- | --- | --- | --- | --- | --- | --- | --- | --- | --- | --- | --- | --- | --- | --- | --- | --- | --- | --- | --- | --- | --- | --- | --- | --- | --- | --- | --- | --- | --- | --- | --- |
| K.TQVVAGTNYYIK.V | Y | 43.63 | 1355.7085 | 12 | 0.8 | 678.8621 | 2 | 11.72 | 3 | F3:2504 | 05112019\_RID\_1313\_NaNaRj\_F3.raw |  |  | 9.8E3 |  |  |  |  |  |  |  | 1 | 0 | 0 | 1 | 0 | 0 | 0 | 0 | 0 | 0 | 0 | 45 | 56 |  |
| K.PATPEIQEIVDK.V | Y | 42.50 | 1338.7030 | 12 | 0.6 | 670.3592 | 2 | 47.06 | 2 | F2:15429 | 09012020\_NaNaRj\_F2.raw |  | 8.21E4 |  |  |  |  |  |  |  |  | 1 | 0 | 1 | 0 | 0 | 0 | 0 | 0 | 0 | 0 | 0 | 11 | 22 |  |
| total 2 peptides |  |  |  |  |  |  |  |  |  |  |  |  |  |  |  |  |  |  |  |  |  |  |  |  |  |  |  |  |  |  |  |  |  |  |  |
| --- | --- | --- | --- | --- | --- | --- | --- | --- | --- | --- | --- | --- | --- | --- | --- | --- | --- | --- | --- | --- | --- | --- | --- | --- | --- | --- | --- | --- | --- | --- | --- | --- | --- | --- | --- |

Q5T749|KPRP\_HUMAN

back to list

  

| Protein Coverage
| Supporting Peptides
|

Protein Coverage:

Supporting Peptides:

| Peptide | Uniq | -10lgP | Mass | Length | ppm | m/z | z | RT | Fraction | Scan | Source File | Area F1 | Area F2 | Area F3 | Area F4 | Area F5 | Area F6 | Area F7 | Area F8 | Area F9 | Area F10 | #Feature | #Feature F1 | #Feature F2 | #Feature F3 | #Feature F4 | #Feature F5 | #Feature F6 | #Feature F7 | #Feature F8 | #Feature F9 | #Feature F10 | Start | End | PTM |
| --- | --- | --- | --- | --- | --- | --- | --- | --- | --- | --- | --- | --- | --- | --- | --- | --- | --- | --- | --- | --- | --- | --- | --- | --- | --- | --- | --- | --- | --- | --- | --- | --- | --- | --- | --- |
| R.C(+57.02)PVEIPPIR.R | Y | 51.67 | 1079.5797 | 9 | 0.4 | 540.7974 | 2 | 12.24 | 5 | F5:3035 | 05112019\_RID\_1313\_NaNaRj\_F5.raw |  |  |  |  | 5.15E4 |  |  |  |  |  | 1 | 0 | 0 | 0 | 0 | 1 | 0 | 0 | 0 | 0 | 0 | 343 | 351 | Carbamidomethylation |
| total 1 peptides |  |  |  |  |  |  |  |  |  |  |  |  |  |  |  |  |  |  |  |  |  |  |  |  |  |  |  |  |  |  |  |  |  |  |  |
| --- | --- | --- | --- | --- | --- | --- | --- | --- | --- | --- | --- | --- | --- | --- | --- | --- | --- | --- | --- | --- | --- | --- | --- | --- | --- | --- | --- | --- | --- | --- | --- | --- | --- | --- | --- |

P14613|3S1B1\_NAJKA

back to list

  

| Protein Coverage
| Supporting Peptides
|

Protein Coverage:

Supporting Peptides:

| Peptide | Uniq | -10lgP | Mass | Length | ppm | m/z | z | RT | Fraction | Scan | Source File | Area F1 | Area F2 | Area F3 | Area F4 | Area F5 | Area F6 | Area F7 | Area F8 | Area F9 | Area F10 | #Feature | #Feature F1 | #Feature F2 | #Feature F3 | #Feature F4 | #Feature F5 | #Feature F6 | #Feature F7 | #Feature F8 | #Feature F9 | #Feature F10 | Start | End | PTM |
| --- | --- | --- | --- | --- | --- | --- | --- | --- | --- | --- | --- | --- | --- | --- | --- | --- | --- | --- | --- | --- | --- | --- | --- | --- | --- | --- | --- | --- | --- | --- | --- | --- | --- | --- | --- |
| K.NGIEINC(+57.02)C(+57.02)TTDR.C | Y | 68.86 | 1451.6133 | 12 | 0.6 | 726.8143 | 2 | 11.39 | 4 | F4:2247 | 05112019\_RID\_1313\_NaNaRj\_F4.raw |  | 2.97E5 |  | 2.32E4 |  |  |  |  |  |  | 2 | 0 | 1 | 0 | 1 | 0 | 0 | 0 | 0 | 0 | 0 | 48 | 59 | Carbamidomethylation |
| total 1 peptides |  |  |  |  |  |  |  |  |  |  |  |  |  |  |  |  |  |  |  |  |  |  |  |  |  |  |  |  |  |  |  |  |  |  |  |
| --- | --- | --- | --- | --- | --- | --- | --- | --- | --- | --- | --- | --- | --- | --- | --- | --- | --- | --- | --- | --- | --- | --- | --- | --- | --- | --- | --- | --- | --- | --- | --- | --- | --- | --- | --- |

Q9PSN6|3S13\_NAJSP

back to list

  

| Protein Coverage
| Supporting Peptides
|

Protein Coverage:

Supporting Peptides:

| Peptide | Uniq | -10lgP | Mass | Length | ppm | m/z | z | RT | Fraction | Scan | Source File | Area F1 | Area F2 | Area F3 | Area F4 | Area F5 | Area F6 | Area F7 | Area F8 | Area F9 | Area F10 | #Feature | #Feature F1 | #Feature F2 | #Feature F3 | #Feature F4 | #Feature F5 | #Feature F6 | #Feature F7 | #Feature F8 | #Feature F9 | #Feature F10 | Start | End | PTM |
| --- | --- | --- | --- | --- | --- | --- | --- | --- | --- | --- | --- | --- | --- | --- | --- | --- | --- | --- | --- | --- | --- | --- | --- | --- | --- | --- | --- | --- | --- | --- | --- | --- | --- | --- | --- |
| K.NGIEINC(+57.02)C(+57.02)TTDR.C | Y | 68.86 | 1451.6133 | 12 | 0.6 | 726.8143 | 2 | 11.39 | 4 | F4:2247 | 05112019\_RID\_1313\_NaNaRj\_F4.raw |  | 2.97E5 |  | 2.32E4 |  |  |  |  |  |  | 2 | 0 | 1 | 0 | 1 | 0 | 0 | 0 | 0 | 0 | 0 | 48 | 59 | Carbamidomethylation |
| total 1 peptides |  |  |  |  |  |  |  |  |  |  |  |  |  |  |  |  |  |  |  |  |  |  |  |  |  |  |  |  |  |  |  |  |  |  |  |
| --- | --- | --- | --- | --- | --- | --- | --- | --- | --- | --- | --- | --- | --- | --- | --- | --- | --- | --- | --- | --- | --- | --- | --- | --- | --- | --- | --- | --- | --- | --- | --- | --- | --- | --- | --- |

P60771|3S1CB\_NAJKA

back to list

  

| Protein Coverage
| Supporting Peptides
|

Protein Coverage:

Supporting Peptides:

| Peptide | Uniq | -10lgP | Mass | Length | ppm | m/z | z | RT | Fraction | Scan | Source File | Area F1 | Area F2 | Area F3 | Area F4 | Area F5 | Area F6 | Area F7 | Area F8 | Area F9 | Area F10 | #Feature | #Feature F1 | #Feature F2 | #Feature F3 | #Feature F4 | #Feature F5 | #Feature F6 | #Feature F7 | #Feature F8 | #Feature F9 | #Feature F10 | Start | End | PTM |
| --- | --- | --- | --- | --- | --- | --- | --- | --- | --- | --- | --- | --- | --- | --- | --- | --- | --- | --- | --- | --- | --- | --- | --- | --- | --- | --- | --- | --- | --- | --- | --- | --- | --- | --- | --- |
| K.NGIEINC(+57.02)C(+57.02)TTDR.C | Y | 68.86 | 1451.6133 | 12 | 0.6 | 726.8143 | 2 | 11.39 | 4 | F4:2247 | 05112019\_RID\_1313\_NaNaRj\_F4.raw |  | 2.97E5 |  | 2.32E4 |  |  |  |  |  |  | 2 | 0 | 1 | 0 | 1 | 0 | 0 | 0 | 0 | 0 | 0 | 69 | 80 | Carbamidomethylation |
| total 1 peptides |  |  |  |  |  |  |  |  |  |  |  |  |  |  |  |  |  |  |  |  |  |  |  |  |  |  |  |  |  |  |  |  |  |  |  |
| --- | --- | --- | --- | --- | --- | --- | --- | --- | --- | --- | --- | --- | --- | --- | --- | --- | --- | --- | --- | --- | --- | --- | --- | --- | --- | --- | --- | --- | --- | --- | --- | --- | --- | --- | --- |

P60770|3S1CB\_NAJAT

back to list

  

| Protein Coverage
| Supporting Peptides
|

Protein Coverage:

Supporting Peptides:

| Peptide | Uniq | -10lgP | Mass | Length | ppm | m/z | z | RT | Fraction | Scan | Source File | Area F1 | Area F2 | Area F3 | Area F4 | Area F5 | Area F6 | Area F7 | Area F8 | Area F9 | Area F10 | #Feature | #Feature F1 | #Feature F2 | #Feature F3 | #Feature F4 | #Feature F5 | #Feature F6 | #Feature F7 | #Feature F8 | #Feature F9 | #Feature F10 | Start | End | PTM |
| --- | --- | --- | --- | --- | --- | --- | --- | --- | --- | --- | --- | --- | --- | --- | --- | --- | --- | --- | --- | --- | --- | --- | --- | --- | --- | --- | --- | --- | --- | --- | --- | --- | --- | --- | --- |
| K.NGIEINC(+57.02)C(+57.02)TTDR.C | Y | 68.86 | 1451.6133 | 12 | 0.6 | 726.8143 | 2 | 11.39 | 4 | F4:2247 | 05112019\_RID\_1313\_NaNaRj\_F4.raw |  | 2.97E5 |  | 2.32E4 |  |  |  |  |  |  | 2 | 0 | 1 | 0 | 1 | 0 | 0 | 0 | 0 | 0 | 0 | 69 | 80 | Carbamidomethylation |
| total 1 peptides |  |  |  |  |  |  |  |  |  |  |  |  |  |  |  |  |  |  |  |  |  |  |  |  |  |  |  |  |  |  |  |  |  |  |  |
| --- | --- | --- | --- | --- | --- | --- | --- | --- | --- | --- | --- | --- | --- | --- | --- | --- | --- | --- | --- | --- | --- | --- | --- | --- | --- | --- | --- | --- | --- | --- | --- | --- | --- | --- | --- |

Q9PTT0|3S1CB\_NAJNA

back to list

  

| Protein Coverage
| Supporting Peptides
|

Protein Coverage:

Supporting Peptides:

| Peptide | Uniq | -10lgP | Mass | Length | ppm | m/z | z | RT | Fraction | Scan | Source File | Area F1 | Area F2 | Area F3 | Area F4 | Area F5 | Area F6 | Area F7 | Area F8 | Area F9 | Area F10 | #Feature | #Feature F1 | #Feature F2 | #Feature F3 | #Feature F4 | #Feature F5 | #Feature F6 | #Feature F7 | #Feature F8 | #Feature F9 | #Feature F10 | Start | End | PTM |
| --- | --- | --- | --- | --- | --- | --- | --- | --- | --- | --- | --- | --- | --- | --- | --- | --- | --- | --- | --- | --- | --- | --- | --- | --- | --- | --- | --- | --- | --- | --- | --- | --- | --- | --- | --- |
| K.NGIEINC(+57.02)C(+57.02)TTDR.C | Y | 68.86 | 1451.6133 | 12 | 0.6 | 726.8143 | 2 | 11.39 | 4 | F4:2247 | 05112019\_RID\_1313\_NaNaRj\_F4.raw |  | 2.97E5 |  | 2.32E4 |  |  |  |  |  |  | 2 | 0 | 1 | 0 | 1 | 0 | 0 | 0 | 0 | 0 | 0 | 69 | 80 | Carbamidomethylation |
| total 1 peptides |  |  |  |  |  |  |  |  |  |  |  |  |  |  |  |  |  |  |  |  |  |  |  |  |  |  |  |  |  |  |  |  |  |  |  |
| --- | --- | --- | --- | --- | --- | --- | --- | --- | --- | --- | --- | --- | --- | --- | --- | --- | --- | --- | --- | --- | --- | --- | --- | --- | --- | --- | --- | --- | --- | --- | --- | --- | --- | --- | --- |

Q5D862|FILA2\_HUMAN

back to list

  

| Protein Coverage
| Supporting Peptides
|

Protein Coverage:

Supporting Peptides:

| Peptide | Uniq | -10lgP | Mass | Length | ppm | m/z | z | RT | Fraction | Scan | Source File | Area F1 | Area F2 | Area F3 | Area F4 | Area F5 | Area F6 | Area F7 | Area F8 | Area F9 | Area F10 | #Feature | #Feature F1 | #Feature F2 | #Feature F3 | #Feature F4 | #Feature F5 | #Feature F6 | #Feature F7 | #Feature F8 | #Feature F9 | #Feature F10 | Start | End | PTM |
| --- | --- | --- | --- | --- | --- | --- | --- | --- | --- | --- | --- | --- | --- | --- | --- | --- | --- | --- | --- | --- | --- | --- | --- | --- | --- | --- | --- | --- | --- | --- | --- | --- | --- | --- | --- |
| R.SVVTVIDVFYK.Y | Y | 50.66 | 1268.7017 | 11 | 0.8 | 635.3586 | 2 | 30.08 | 6 | F6:13470 | 05112019\_RID\_1313\_NaNaRj\_F6.raw |  |  |  | 1.63E4 |  | 2.51E4 |  |  |  |  | 2 | 0 | 0 | 0 | 1 | 0 | 1 | 0 | 0 | 0 | 0 | 7 | 17 |  |
| total 1 peptides |  |  |  |  |  |  |  |  |  |  |  |  |  |  |  |  |  |  |  |  |  |  |  |  |  |  |  |  |  |  |  |  |  |  |  |
| --- | --- | --- | --- | --- | --- | --- | --- | --- | --- | --- | --- | --- | --- | --- | --- | --- | --- | --- | --- | --- | --- | --- | --- | --- | --- | --- | --- | --- | --- | --- | --- | --- | --- | --- | --- |

P23764|GPX3\_RAT

back to list

  

| Protein Coverage
| Supporting Peptides
|

Protein Coverage:

Supporting Peptides:

| Peptide | Uniq | -10lgP | Mass | Length | ppm | m/z | z | RT | Fraction | Scan | Source File | Area F1 | Area F2 | Area F3 | Area F4 | Area F5 | Area F6 | Area F7 | Area F8 | Area F9 | Area F10 | #Feature | #Feature F1 | #Feature F2 | #Feature F3 | #Feature F4 | #Feature F5 | #Feature F6 | #Feature F7 | #Feature F8 | #Feature F9 | #Feature F10 | Start | End | PTM |
| --- | --- | --- | --- | --- | --- | --- | --- | --- | --- | --- | --- | --- | --- | --- | --- | --- | --- | --- | --- | --- | --- | --- | --- | --- | --- | --- | --- | --- | --- | --- | --- | --- | --- | --- | --- |
| G.LVILGFPC(+57.02)NQFGK.Q | Y | 44.93 | 1491.7908 | 13 | 0.9 | 746.9033 | 2 | 28.51 | 9 | F9:6008 | 05112019\_RID\_1313\_NaNaRj\_F9.raw |  |  |  |  |  | 5.49E4 |  | 2.1E5 | 2.48E4 |  | 3 | 0 | 0 | 0 | 0 | 0 | 1 | 0 | 1 | 1 | 0 | 94 | 106 | Carbamidomethylation |
| F.GLVILGFPC(+57.02)NQFGK.Q | Y | 41.74 | 1548.8123 | 14 | -0.1 | 775.4133 | 2 | 30.71 | 6 | F6:13771 | 05112019\_RID\_1313\_NaNaRj\_F6.raw |  |  |  |  |  | 2.08E4 |  |  |  |  | 1 | 0 | 0 | 0 | 0 | 0 | 1 | 0 | 0 | 0 | 0 | 93 | 106 | Carbamidomethylation |
| total 2 peptides |  |  |  |  |  |  |  |  |  |  |  |  |  |  |  |  |  |  |  |  |  |  |  |  |  |  |  |  |  |  |  |  |  |  |  |
| --- | --- | --- | --- | --- | --- | --- | --- | --- | --- | --- | --- | --- | --- | --- | --- | --- | --- | --- | --- | --- | --- | --- | --- | --- | --- | --- | --- | --- | --- | --- | --- | --- | --- | --- | --- |

Q4AEH4|GPX3\_MACFU

back to list

  

| Protein Coverage
| Supporting Peptides
|

Protein Coverage:

Supporting Peptides:

| Peptide | Uniq | -10lgP | Mass | Length | ppm | m/z | z | RT | Fraction | Scan | Source File | Area F1 | Area F2 | Area F3 | Area F4 | Area F5 | Area F6 | Area F7 | Area F8 | Area F9 | Area F10 | #Feature | #Feature F1 | #Feature F2 | #Feature F3 | #Feature F4 | #Feature F5 | #Feature F6 | #Feature F7 | #Feature F8 | #Feature F9 | #Feature F10 | Start | End | PTM |
| --- | --- | --- | --- | --- | --- | --- | --- | --- | --- | --- | --- | --- | --- | --- | --- | --- | --- | --- | --- | --- | --- | --- | --- | --- | --- | --- | --- | --- | --- | --- | --- | --- | --- | --- | --- |
| G.LVLLGFPC(+57.02)NQFGK.Q | Y | 44.93 | 1491.7908 | 13 | 0.9 | 746.9033 | 2 | 28.51 | 9 | F9:6008 | 05112019\_RID\_1313\_NaNaRj\_F9.raw |  |  |  |  |  | 5.49E4 |  | 2.1E5 | 2.48E4 |  | 3 | 0 | 0 | 0 | 0 | 0 | 1 | 0 | 1 | 1 | 0 | 94 | 106 | Carbamidomethylation |
| F.GLVLLGFPC(+57.02)NQFGK.Q | Y | 41.74 | 1548.8123 | 14 | -0.1 | 775.4133 | 2 | 30.71 | 6 | F6:13771 | 05112019\_RID\_1313\_NaNaRj\_F6.raw |  |  |  |  |  | 2.08E4 |  |  |  |  | 1 | 0 | 0 | 0 | 0 | 0 | 1 | 0 | 0 | 0 | 0 | 93 | 106 | Carbamidomethylation |
| total 2 peptides |  |  |  |  |  |  |  |  |  |  |  |  |  |  |  |  |  |  |  |  |  |  |  |  |  |  |  |  |  |  |  |  |  |  |  |
| --- | --- | --- | --- | --- | --- | --- | --- | --- | --- | --- | --- | --- | --- | --- | --- | --- | --- | --- | --- | --- | --- | --- | --- | --- | --- | --- | --- | --- | --- | --- | --- | --- | --- | --- | --- |

P22352|GPX3\_HUMAN

back to list

  

| Protein Coverage
| Supporting Peptides
|

Protein Coverage:

Supporting Peptides:

| Peptide | Uniq | -10lgP | Mass | Length | ppm | m/z | z | RT | Fraction | Scan | Source File | Area F1 | Area F2 | Area F3 | Area F4 | Area F5 | Area F6 | Area F7 | Area F8 | Area F9 | Area F10 | #Feature | #Feature F1 | #Feature F2 | #Feature F3 | #Feature F4 | #Feature F5 | #Feature F6 | #Feature F7 | #Feature F8 | #Feature F9 | #Feature F10 | Start | End | PTM |
| --- | --- | --- | --- | --- | --- | --- | --- | --- | --- | --- | --- | --- | --- | --- | --- | --- | --- | --- | --- | --- | --- | --- | --- | --- | --- | --- | --- | --- | --- | --- | --- | --- | --- | --- | --- |
| G.LVILGFPC(+57.02)NQFGK.Q | Y | 44.93 | 1491.7908 | 13 | 0.9 | 746.9033 | 2 | 28.51 | 9 | F9:6008 | 05112019\_RID\_1313\_NaNaRj\_F9.raw |  |  |  |  |  | 5.49E4 |  | 2.1E5 | 2.48E4 |  | 3 | 0 | 0 | 0 | 0 | 0 | 1 | 0 | 1 | 1 | 0 | 94 | 106 | Carbamidomethylation |
| F.GLVILGFPC(+57.02)NQFGK.Q | Y | 41.74 | 1548.8123 | 14 | -0.1 | 775.4133 | 2 | 30.71 | 6 | F6:13771 | 05112019\_RID\_1313\_NaNaRj\_F6.raw |  |  |  |  |  | 2.08E4 |  |  |  |  | 1 | 0 | 0 | 0 | 0 | 0 | 1 | 0 | 0 | 0 | 0 | 93 | 106 | Carbamidomethylation |
| total 2 peptides |  |  |  |  |  |  |  |  |  |  |  |  |  |  |  |  |  |  |  |  |  |  |  |  |  |  |  |  |  |  |  |  |  |  |  |
| --- | --- | --- | --- | --- | --- | --- | --- | --- | --- | --- | --- | --- | --- | --- | --- | --- | --- | --- | --- | --- | --- | --- | --- | --- | --- | --- | --- | --- | --- | --- | --- | --- | --- | --- | --- |

Q5RFG3|GPX3\_PONPY

back to list

  

| Protein Coverage
| Supporting Peptides
|

Protein Coverage:

Supporting Peptides:

| Peptide | Uniq | -10lgP | Mass | Length | ppm | m/z | z | RT | Fraction | Scan | Source File | Area F1 | Area F2 | Area F3 | Area F4 | Area F5 | Area F6 | Area F7 | Area F8 | Area F9 | Area F10 | #Feature | #Feature F1 | #Feature F2 | #Feature F3 | #Feature F4 | #Feature F5 | #Feature F6 | #Feature F7 | #Feature F8 | #Feature F9 | #Feature F10 | Start | End | PTM |
| --- | --- | --- | --- | --- | --- | --- | --- | --- | --- | --- | --- | --- | --- | --- | --- | --- | --- | --- | --- | --- | --- | --- | --- | --- | --- | --- | --- | --- | --- | --- | --- | --- | --- | --- | --- |
| G.LVILGFPC(+57.02)NQFGK.Q | Y | 44.93 | 1491.7908 | 13 | 0.9 | 746.9033 | 2 | 28.51 | 9 | F9:6008 | 05112019\_RID\_1313\_NaNaRj\_F9.raw |  |  |  |  |  | 5.49E4 |  | 2.1E5 | 2.48E4 |  | 3 | 0 | 0 | 0 | 0 | 0 | 1 | 0 | 1 | 1 | 0 | 94 | 106 | Carbamidomethylation |
| F.GLVILGFPC(+57.02)NQFGK.Q | Y | 41.74 | 1548.8123 | 14 | -0.1 | 775.4133 | 2 | 30.71 | 6 | F6:13771 | 05112019\_RID\_1313\_NaNaRj\_F6.raw |  |  |  |  |  | 2.08E4 |  |  |  |  | 1 | 0 | 0 | 0 | 0 | 0 | 1 | 0 | 0 | 0 | 0 | 93 | 106 | Carbamidomethylation |
| total 2 peptides |  |  |  |  |  |  |  |  |  |  |  |  |  |  |  |  |  |  |  |  |  |  |  |  |  |  |  |  |  |  |  |  |  |  |  |
| --- | --- | --- | --- | --- | --- | --- | --- | --- | --- | --- | --- | --- | --- | --- | --- | --- | --- | --- | --- | --- | --- | --- | --- | --- | --- | --- | --- | --- | --- | --- | --- | --- | --- | --- | --- |

P37141|GPX3\_BOVIN

back to list

  

| Protein Coverage
| Supporting Peptides
|

Protein Coverage:

Supporting Peptides:

| Peptide | Uniq | -10lgP | Mass | Length | ppm | m/z | z | RT | Fraction | Scan | Source File | Area F1 | Area F2 | Area F3 | Area F4 | Area F5 | Area F6 | Area F7 | Area F8 | Area F9 | Area F10 | #Feature | #Feature F1 | #Feature F2 | #Feature F3 | #Feature F4 | #Feature F5 | #Feature F6 | #Feature F7 | #Feature F8 | #Feature F9 | #Feature F10 | Start | End | PTM |
| --- | --- | --- | --- | --- | --- | --- | --- | --- | --- | --- | --- | --- | --- | --- | --- | --- | --- | --- | --- | --- | --- | --- | --- | --- | --- | --- | --- | --- | --- | --- | --- | --- | --- | --- | --- |
| G.LVILGFPC(+57.02)NQFGK.Q | Y | 44.93 | 1491.7908 | 13 | 0.9 | 746.9033 | 2 | 28.51 | 9 | F9:6008 | 05112019\_RID\_1313\_NaNaRj\_F9.raw |  |  |  |  |  | 5.49E4 |  | 2.1E5 | 2.48E4 |  | 3 | 0 | 0 | 0 | 0 | 0 | 1 | 0 | 1 | 1 | 0 | 94 | 106 | Carbamidomethylation |
| F.GLVILGFPC(+57.02)NQFGK.Q | Y | 41.74 | 1548.8123 | 14 | -0.1 | 775.4133 | 2 | 30.71 | 6 | F6:13771 | 05112019\_RID\_1313\_NaNaRj\_F6.raw |  |  |  |  |  | 2.08E4 |  |  |  |  | 1 | 0 | 0 | 0 | 0 | 0 | 1 | 0 | 0 | 0 | 0 | 93 | 106 | Carbamidomethylation |
| total 2 peptides |  |  |  |  |  |  |  |  |  |  |  |  |  |  |  |  |  |  |  |  |  |  |  |  |  |  |  |  |  |  |  |  |  |  |  |
| --- | --- | --- | --- | --- | --- | --- | --- | --- | --- | --- | --- | --- | --- | --- | --- | --- | --- | --- | --- | --- | --- | --- | --- | --- | --- | --- | --- | --- | --- | --- | --- | --- | --- | --- | --- |

Q4AEH5|GPX3\_HYLLA

back to list

  

| Protein Coverage
| Supporting Peptides
|

Protein Coverage:

Supporting Peptides:

| Peptide | Uniq | -10lgP | Mass | Length | ppm | m/z | z | RT | Fraction | Scan | Source File | Area F1 | Area F2 | Area F3 | Area F4 | Area F5 | Area F6 | Area F7 | Area F8 | Area F9 | Area F10 | #Feature | #Feature F1 | #Feature F2 | #Feature F3 | #Feature F4 | #Feature F5 | #Feature F6 | #Feature F7 | #Feature F8 | #Feature F9 | #Feature F10 | Start | End | PTM |
| --- | --- | --- | --- | --- | --- | --- | --- | --- | --- | --- | --- | --- | --- | --- | --- | --- | --- | --- | --- | --- | --- | --- | --- | --- | --- | --- | --- | --- | --- | --- | --- | --- | --- | --- | --- |
| G.LVILGFPC(+57.02)NQFGK.Q | Y | 44.93 | 1491.7908 | 13 | 0.9 | 746.9033 | 2 | 28.51 | 9 | F9:6008 | 05112019\_RID\_1313\_NaNaRj\_F9.raw |  |  |  |  |  | 5.49E4 |  | 2.1E5 | 2.48E4 |  | 3 | 0 | 0 | 0 | 0 | 0 | 1 | 0 | 1 | 1 | 0 | 94 | 106 | Carbamidomethylation |
| F.GLVILGFPC(+57.02)NQFGK.Q | Y | 41.74 | 1548.8123 | 14 | -0.1 | 775.4133 | 2 | 30.71 | 6 | F6:13771 | 05112019\_RID\_1313\_NaNaRj\_F6.raw |  |  |  |  |  | 2.08E4 |  |  |  |  | 1 | 0 | 0 | 0 | 0 | 0 | 1 | 0 | 0 | 0 | 0 | 93 | 106 | Carbamidomethylation |
| total 2 peptides |  |  |  |  |  |  |  |  |  |  |  |  |  |  |  |  |  |  |  |  |  |  |  |  |  |  |  |  |  |  |  |  |  |  |  |
| --- | --- | --- | --- | --- | --- | --- | --- | --- | --- | --- | --- | --- | --- | --- | --- | --- | --- | --- | --- | --- | --- | --- | --- | --- | --- | --- | --- | --- | --- | --- | --- | --- | --- | --- | --- |

P21765|GPX5\_MOUSE

back to list

  

| Protein Coverage
| Supporting Peptides
|

Protein Coverage:

Supporting Peptides:

| Peptide | Uniq | -10lgP | Mass | Length | ppm | m/z | z | RT | Fraction | Scan | Source File | Area F1 | Area F2 | Area F3 | Area F4 | Area F5 | Area F6 | Area F7 | Area F8 | Area F9 | Area F10 | #Feature | #Feature F1 | #Feature F2 | #Feature F3 | #Feature F4 | #Feature F5 | #Feature F6 | #Feature F7 | #Feature F8 | #Feature F9 | #Feature F10 | Start | End | PTM |
| --- | --- | --- | --- | --- | --- | --- | --- | --- | --- | --- | --- | --- | --- | --- | --- | --- | --- | --- | --- | --- | --- | --- | --- | --- | --- | --- | --- | --- | --- | --- | --- | --- | --- | --- | --- |
| G.LVILGFPC(+57.02)NQFGK.Q | Y | 44.93 | 1491.7908 | 13 | 0.9 | 746.9033 | 2 | 28.51 | 9 | F9:6008 | 05112019\_RID\_1313\_NaNaRj\_F9.raw |  |  |  |  |  | 5.49E4 |  | 2.1E5 | 2.48E4 |  | 3 | 0 | 0 | 0 | 0 | 0 | 1 | 0 | 1 | 1 | 0 | 94 | 106 | Carbamidomethylation |
| F.GLVILGFPC(+57.02)NQFGK.Q | Y | 41.74 | 1548.8123 | 14 | -0.1 | 775.4133 | 2 | 30.71 | 6 | F6:13771 | 05112019\_RID\_1313\_NaNaRj\_F6.raw |  |  |  |  |  | 2.08E4 |  |  |  |  | 1 | 0 | 0 | 0 | 0 | 0 | 1 | 0 | 0 | 0 | 0 | 93 | 106 | Carbamidomethylation |
| total 2 peptides |  |  |  |  |  |  |  |  |  |  |  |  |  |  |  |  |  |  |  |  |  |  |  |  |  |  |  |  |  |  |  |  |  |  |  |
| --- | --- | --- | --- | --- | --- | --- | --- | --- | --- | --- | --- | --- | --- | --- | --- | --- | --- | --- | --- | --- | --- | --- | --- | --- | --- | --- | --- | --- | --- | --- | --- | --- | --- | --- | --- |

P30710|GPX5\_RAT

back to list

  

| Protein Coverage
| Supporting Peptides
|

Protein Coverage:

Supporting Peptides:

| Peptide | Uniq | -10lgP | Mass | Length | ppm | m/z | z | RT | Fraction | Scan | Source File | Area F1 | Area F2 | Area F3 | Area F4 | Area F5 | Area F6 | Area F7 | Area F8 | Area F9 | Area F10 | #Feature | #Feature F1 | #Feature F2 | #Feature F3 | #Feature F4 | #Feature F5 | #Feature F6 | #Feature F7 | #Feature F8 | #Feature F9 | #Feature F10 | Start | End | PTM |
| --- | --- | --- | --- | --- | --- | --- | --- | --- | --- | --- | --- | --- | --- | --- | --- | --- | --- | --- | --- | --- | --- | --- | --- | --- | --- | --- | --- | --- | --- | --- | --- | --- | --- | --- | --- |
| G.LVILGFPC(+57.02)NQFGK.Q | Y | 44.93 | 1491.7908 | 13 | 0.9 | 746.9033 | 2 | 28.51 | 9 | F9:6008 | 05112019\_RID\_1313\_NaNaRj\_F9.raw |  |  |  |  |  | 5.49E4 |  | 2.1E5 | 2.48E4 |  | 3 | 0 | 0 | 0 | 0 | 0 | 1 | 0 | 1 | 1 | 0 | 94 | 106 | Carbamidomethylation |
| F.GLVILGFPC(+57.02)NQFGK.Q | Y | 41.74 | 1548.8123 | 14 | -0.1 | 775.4133 | 2 | 30.71 | 6 | F6:13771 | 05112019\_RID\_1313\_NaNaRj\_F6.raw |  |  |  |  |  | 2.08E4 |  |  |  |  | 1 | 0 | 0 | 0 | 0 | 0 | 1 | 0 | 0 | 0 | 0 | 93 | 106 | Carbamidomethylation |
| total 2 peptides |  |  |  |  |  |  |  |  |  |  |  |  |  |  |  |  |  |  |  |  |  |  |  |  |  |  |  |  |  |  |  |  |  |  |  |
| --- | --- | --- | --- | --- | --- | --- | --- | --- | --- | --- | --- | --- | --- | --- | --- | --- | --- | --- | --- | --- | --- | --- | --- | --- | --- | --- | --- | --- | --- | --- | --- | --- | --- | --- | --- |

P12273|PIP\_HUMAN

back to list

  

| Protein Coverage
| Supporting Peptides
|

Protein Coverage:

Supporting Peptides:

| Peptide | Uniq | -10lgP | Mass | Length | ppm | m/z | z | RT | Fraction | Scan | Source File | Area F1 | Area F2 | Area F3 | Area F4 | Area F5 | Area F6 | Area F7 | Area F8 | Area F9 | Area F10 | #Feature | #Feature F1 | #Feature F2 | #Feature F3 | #Feature F4 | #Feature F5 | #Feature F6 | #Feature F7 | #Feature F8 | #Feature F9 | #Feature F10 | Start | End | PTM |
| --- | --- | --- | --- | --- | --- | --- | --- | --- | --- | --- | --- | --- | --- | --- | --- | --- | --- | --- | --- | --- | --- | --- | --- | --- | --- | --- | --- | --- | --- | --- | --- | --- | --- | --- | --- |
| R.ELGIC(+57.02)PDDAAVIPIK.N | Y | 65.45 | 1609.8385 | 15 | 1.0 | 805.9273 | 2 | 66.63 | 2 | F2:21806 | 09012020\_NaNaRj\_F2.raw |  | 3.63E4 |  |  | 1.28E4 |  |  |  |  |  | 2 | 0 | 1 | 0 | 0 | 1 | 0 | 0 | 0 | 0 | 0 | 119 | 133 | Carbamidomethylation |
| total 1 peptides |  |  |  |  |  |  |  |  |  |  |  |  |  |  |  |  |  |  |  |  |  |  |  |  |  |  |  |  |  |  |  |  |  |  |  |
| --- | --- | --- | --- | --- | --- | --- | --- | --- | --- | --- | --- | --- | --- | --- | --- | --- | --- | --- | --- | --- | --- | --- | --- | --- | --- | --- | --- | --- | --- | --- | --- | --- | --- | --- | --- |

A0A885|PIP\_GORGO

back to list

  

| Protein Coverage
| Supporting Peptides
|

Protein Coverage:

Supporting Peptides:

| Peptide | Uniq | -10lgP | Mass | Length | ppm | m/z | z | RT | Fraction | Scan | Source File | Area F1 | Area F2 | Area F3 | Area F4 | Area F5 | Area F6 | Area F7 | Area F8 | Area F9 | Area F10 | #Feature | #Feature F1 | #Feature F2 | #Feature F3 | #Feature F4 | #Feature F5 | #Feature F6 | #Feature F7 | #Feature F8 | #Feature F9 | #Feature F10 | Start | End | PTM |
| --- | --- | --- | --- | --- | --- | --- | --- | --- | --- | --- | --- | --- | --- | --- | --- | --- | --- | --- | --- | --- | --- | --- | --- | --- | --- | --- | --- | --- | --- | --- | --- | --- | --- | --- | --- |
| Q.ELGIC(+57.02)PDDAAVIPIK.N | Y | 65.45 | 1609.8385 | 15 | 1.0 | 805.9273 | 2 | 66.63 | 2 | F2:21806 | 09012020\_NaNaRj\_F2.raw |  | 3.63E4 |  |  | 1.28E4 |  |  |  |  |  | 2 | 0 | 1 | 0 | 0 | 1 | 0 | 0 | 0 | 0 | 0 | 119 | 133 | Carbamidomethylation |
| total 1 peptides |  |  |  |  |  |  |  |  |  |  |  |  |  |  |  |  |  |  |  |  |  |  |  |  |  |  |  |  |  |  |  |  |  |  |  |
| --- | --- | --- | --- | --- | --- | --- | --- | --- | --- | --- | --- | --- | --- | --- | --- | --- | --- | --- | --- | --- | --- | --- | --- | --- | --- | --- | --- | --- | --- | --- | --- | --- | --- | --- | --- |

P60989|PIP\_PANTR

back to list

  

| Protein Coverage
| Supporting Peptides
|

Protein Coverage:

Supporting Peptides:

| Peptide | Uniq | -10lgP | Mass | Length | ppm | m/z | z | RT | Fraction | Scan | Source File | Area F1 | Area F2 | Area F3 | Area F4 | Area F5 | Area F6 | Area F7 | Area F8 | Area F9 | Area F10 | #Feature | #Feature F1 | #Feature F2 | #Feature F3 | #Feature F4 | #Feature F5 | #Feature F6 | #Feature F7 | #Feature F8 | #Feature F9 | #Feature F10 | Start | End | PTM |
| --- | --- | --- | --- | --- | --- | --- | --- | --- | --- | --- | --- | --- | --- | --- | --- | --- | --- | --- | --- | --- | --- | --- | --- | --- | --- | --- | --- | --- | --- | --- | --- | --- | --- | --- | --- |
| Q.ELGIC(+57.02)PDDAAVIPIK.N | Y | 65.45 | 1609.8385 | 15 | 1.0 | 805.9273 | 2 | 66.63 | 2 | F2:21806 | 09012020\_NaNaRj\_F2.raw |  | 3.63E4 |  |  | 1.28E4 |  |  |  |  |  | 2 | 0 | 1 | 0 | 0 | 1 | 0 | 0 | 0 | 0 | 0 | 119 | 133 | Carbamidomethylation |
| total 1 peptides |  |  |  |  |  |  |  |  |  |  |  |  |  |  |  |  |  |  |  |  |  |  |  |  |  |  |  |  |  |  |  |  |  |  |  |
| --- | --- | --- | --- | --- | --- | --- | --- | --- | --- | --- | --- | --- | --- | --- | --- | --- | --- | --- | --- | --- | --- | --- | --- | --- | --- | --- | --- | --- | --- | --- | --- | --- | --- | --- | --- |

A0A888|PIP\_PONPY

back to list

  

| Protein Coverage
| Supporting Peptides
|

Protein Coverage:

Supporting Peptides:

| Peptide | Uniq | -10lgP | Mass | Length | ppm | m/z | z | RT | Fraction | Scan | Source File | Area F1 | Area F2 | Area F3 | Area F4 | Area F5 | Area F6 | Area F7 | Area F8 | Area F9 | Area F10 | #Feature | #Feature F1 | #Feature F2 | #Feature F3 | #Feature F4 | #Feature F5 | #Feature F6 | #Feature F7 | #Feature F8 | #Feature F9 | #Feature F10 | Start | End | PTM |
| --- | --- | --- | --- | --- | --- | --- | --- | --- | --- | --- | --- | --- | --- | --- | --- | --- | --- | --- | --- | --- | --- | --- | --- | --- | --- | --- | --- | --- | --- | --- | --- | --- | --- | --- | --- |
| R.ELGIC(+57.02)PDDAAVIPIK.N | Y | 65.45 | 1609.8385 | 15 | 1.0 | 805.9273 | 2 | 66.63 | 2 | F2:21806 | 09012020\_NaNaRj\_F2.raw |  | 3.63E4 |  |  | 1.28E4 |  |  |  |  |  | 2 | 0 | 1 | 0 | 0 | 1 | 0 | 0 | 0 | 0 | 0 | 119 | 133 | Carbamidomethylation |
| total 1 peptides |  |  |  |  |  |  |  |  |  |  |  |  |  |  |  |  |  |  |  |  |  |  |  |  |  |  |  |  |  |  |  |  |  |  |  |
| --- | --- | --- | --- | --- | --- | --- | --- | --- | --- | --- | --- | --- | --- | --- | --- | --- | --- | --- | --- | --- | --- | --- | --- | --- | --- | --- | --- | --- | --- | --- | --- | --- | --- | --- | --- |

A0A889|PIP\_SYMSY

back to list

  

| Protein Coverage
| Supporting Peptides
|

Protein Coverage:

Supporting Peptides:

| Peptide | Uniq | -10lgP | Mass | Length | ppm | m/z | z | RT | Fraction | Scan | Source File | Area F1 | Area F2 | Area F3 | Area F4 | Area F5 | Area F6 | Area F7 | Area F8 | Area F9 | Area F10 | #Feature | #Feature F1 | #Feature F2 | #Feature F3 | #Feature F4 | #Feature F5 | #Feature F6 | #Feature F7 | #Feature F8 | #Feature F9 | #Feature F10 | Start | End | PTM |
| --- | --- | --- | --- | --- | --- | --- | --- | --- | --- | --- | --- | --- | --- | --- | --- | --- | --- | --- | --- | --- | --- | --- | --- | --- | --- | --- | --- | --- | --- | --- | --- | --- | --- | --- | --- |
| R.ELGIC(+57.02)PDDAAVIPIK.N | Y | 65.45 | 1609.8385 | 15 | 1.0 | 805.9273 | 2 | 66.63 | 2 | F2:21806 | 09012020\_NaNaRj\_F2.raw |  | 3.63E4 |  |  | 1.28E4 |  |  |  |  |  | 2 | 0 | 1 | 0 | 0 | 1 | 0 | 0 | 0 | 0 | 0 | 119 | 133 | Carbamidomethylation |
| total 1 peptides |  |  |  |  |  |  |  |  |  |  |  |  |  |  |  |  |  |  |  |  |  |  |  |  |  |  |  |  |  |  |  |  |  |  |  |
| --- | --- | --- | --- | --- | --- | --- | --- | --- | --- | --- | --- | --- | --- | --- | --- | --- | --- | --- | --- | --- | --- | --- | --- | --- | --- | --- | --- | --- | --- | --- | --- | --- | --- | --- | --- |

A0A890|PIP\_HYLAG

back to list

  

| Protein Coverage
| Supporting Peptides
|

Protein Coverage:

Supporting Peptides:

| Peptide | Uniq | -10lgP | Mass | Length | ppm | m/z | z | RT | Fraction | Scan | Source File | Area F1 | Area F2 | Area F3 | Area F4 | Area F5 | Area F6 | Area F7 | Area F8 | Area F9 | Area F10 | #Feature | #Feature F1 | #Feature F2 | #Feature F3 | #Feature F4 | #Feature F5 | #Feature F6 | #Feature F7 | #Feature F8 | #Feature F9 | #Feature F10 | Start | End | PTM |
| --- | --- | --- | --- | --- | --- | --- | --- | --- | --- | --- | --- | --- | --- | --- | --- | --- | --- | --- | --- | --- | --- | --- | --- | --- | --- | --- | --- | --- | --- | --- | --- | --- | --- | --- | --- |
| R.ELGIC(+57.02)PDDAAVIPIK.S | Y | 65.45 | 1609.8385 | 15 | 1.0 | 805.9273 | 2 | 66.63 | 2 | F2:21806 | 09012020\_NaNaRj\_F2.raw |  | 3.63E4 |  |  | 1.28E4 |  |  |  |  |  | 2 | 0 | 1 | 0 | 0 | 1 | 0 | 0 | 0 | 0 | 0 | 119 | 133 | Carbamidomethylation |
| total 1 peptides |  |  |  |  |  |  |  |  |  |  |  |  |  |  |  |  |  |  |  |  |  |  |  |  |  |  |  |  |  |  |  |  |  |  |  |
| --- | --- | --- | --- | --- | --- | --- | --- | --- | --- | --- | --- | --- | --- | --- | --- | --- | --- | --- | --- | --- | --- | --- | --- | --- | --- | --- | --- | --- | --- | --- | --- | --- | --- | --- | --- |

Q08554|DSC1\_HUMAN

back to list

  

| Protein Coverage
| Supporting Peptides
|

Protein Coverage:

Supporting Peptides:

| Peptide | Uniq | -10lgP | Mass | Length | ppm | m/z | z | RT | Fraction | Scan | Source File | Area F1 | Area F2 | Area F3 | Area F4 | Area F5 | Area F6 | Area F7 | Area F8 | Area F9 | Area F10 | #Feature | #Feature F1 | #Feature F2 | #Feature F3 | #Feature F4 | #Feature F5 | #Feature F6 | #Feature F7 | #Feature F8 | #Feature F9 | #Feature F10 | Start | End | PTM |
| --- | --- | --- | --- | --- | --- | --- | --- | --- | --- | --- | --- | --- | --- | --- | --- | --- | --- | --- | --- | --- | --- | --- | --- | --- | --- | --- | --- | --- | --- | --- | --- | --- | --- | --- | --- |
| K.DTGDIFC(+57.02)TR.S | Y | 44.59 | 1083.4655 | 9 | 1.0 | 542.7405 | 2 | 12.28 | 5 | F5:3076 | 05112019\_RID\_1313\_NaNaRj\_F5.raw |  |  |  |  | 1.23E4 |  |  |  |  |  | 1 | 0 | 0 | 0 | 0 | 1 | 0 | 0 | 0 | 0 | 0 | 190 | 198 | Carbamidomethylation |
| K.ILQQIPDHPK.H | Y | 41.31 | 1187.6663 | 10 | -0.9 | 396.8957 | 3 | 23.44 | 2 | F2:5628 | 09012020\_NaNaRj\_F2.raw |  | 3.77E4 |  |  |  |  |  |  |  |  | 1 | 0 | 1 | 0 | 0 | 0 | 0 | 0 | 0 | 0 | 0 | 283 | 292 |  |
| total 2 peptides |  |  |  |  |  |  |  |  |  |  |  |  |  |  |  |  |  |  |  |  |  |  |  |  |  |  |  |  |  |  |  |  |  |  |  |
| --- | --- | --- | --- | --- | --- | --- | --- | --- | --- | --- | --- | --- | --- | --- | --- | --- | --- | --- | --- | --- | --- | --- | --- | --- | --- | --- | --- | --- | --- | --- | --- | --- | --- | --- | --- |

E3P6P4|CYT\_NAJKA

back to list

  

| Protein Coverage
| Supporting Peptides
|

Protein Coverage:

Supporting Peptides:

| Peptide | Uniq | -10lgP | Mass | Length | ppm | m/z | z | RT | Fraction | Scan | Source File | Area F1 | Area F2 | Area F3 | Area F4 | Area F5 | Area F6 | Area F7 | Area F8 | Area F9 | Area F10 | #Feature | #Feature F1 | #Feature F2 | #Feature F3 | #Feature F4 | #Feature F5 | #Feature F6 | #Feature F7 | #Feature F8 | #Feature F9 | #Feature F10 | Start | End | PTM |
| --- | --- | --- | --- | --- | --- | --- | --- | --- | --- | --- | --- | --- | --- | --- | --- | --- | --- | --- | --- | --- | --- | --- | --- | --- | --- | --- | --- | --- | --- | --- | --- | --- | --- | --- | --- |
| R.VVEAQSQVVAGEK.Y | Y | 63.66 | 1342.7092 | 13 | 0.1 | 672.3619 | 2 | 11.06 | 8 | F8:1943 | 05112019\_RID\_1313\_NaNaRj\_F8.raw |  |  |  |  |  |  |  | 1.37E5 |  |  | 1 | 0 | 0 | 0 | 0 | 0 | 0 | 0 | 1 | 0 | 0 | 67 | 79 |  |
| total 1 peptides |  |  |  |  |  |  |  |  |  |  |  |  |  |  |  |  |  |  |  |  |  |  |  |  |  |  |  |  |  |  |  |  |  |  |  |
| --- | --- | --- | --- | --- | --- | --- | --- | --- | --- | --- | --- | --- | --- | --- | --- | --- | --- | --- | --- | --- | --- | --- | --- | --- | --- | --- | --- | --- | --- | --- | --- | --- | --- | --- | --- |

Q5T750|XP32\_HUMAN

back to list

  

| Protein Coverage
| Supporting Peptides
|

Protein Coverage:

Supporting Peptides:

| Peptide | Uniq | -10lgP | Mass | Length | ppm | m/z | z | RT | Fraction | Scan | Source File | Area F1 | Area F2 | Area F3 | Area F4 | Area F5 | Area F6 | Area F7 | Area F8 | Area F9 | Area F10 | #Feature | #Feature F1 | #Feature F2 | #Feature F3 | #Feature F4 | #Feature F5 | #Feature F6 | #Feature F7 | #Feature F8 | #Feature F9 | #Feature F10 | Start | End | PTM |
| --- | --- | --- | --- | --- | --- | --- | --- | --- | --- | --- | --- | --- | --- | --- | --- | --- | --- | --- | --- | --- | --- | --- | --- | --- | --- | --- | --- | --- | --- | --- | --- | --- | --- | --- | --- |
| R.TFGVSPLR.R | Y | 45.20 | 875.4865 | 8 | 0.0 | 438.7505 | 2 | 39.42 | 2 | F2:12177 | 09012020\_NaNaRj\_F2.raw |  | 5.88E5 |  |  |  |  |  |  |  |  | 1 | 0 | 1 | 0 | 0 | 0 | 0 | 0 | 0 | 0 | 0 | 177 | 184 |  |
| total 1 peptides |  |  |  |  |  |  |  |  |  |  |  |  |  |  |  |  |  |  |  |  |  |  |  |  |  |  |  |  |  |  |  |  |  |  |  |
| --- | --- | --- | --- | --- | --- | --- | --- | --- | --- | --- | --- | --- | --- | --- | --- | --- | --- | --- | --- | --- | --- | --- | --- | --- | --- | --- | --- | --- | --- | --- | --- | --- | --- | --- | --- |

Q96P63|SPB12\_HUMAN

back to list

  

| Protein Coverage
| Supporting Peptides
|

Protein Coverage:

Supporting Peptides:

| Peptide | Uniq | -10lgP | Mass | Length | ppm | m/z | z | RT | Fraction | Scan | Source File | Area F1 | Area F2 | Area F3 | Area F4 | Area F5 | Area F6 | Area F7 | Area F8 | Area F9 | Area F10 | #Feature | #Feature F1 | #Feature F2 | #Feature F3 | #Feature F4 | #Feature F5 | #Feature F6 | #Feature F7 | #Feature F8 | #Feature F9 | #Feature F10 | Start | End | PTM |
| --- | --- | --- | --- | --- | --- | --- | --- | --- | --- | --- | --- | --- | --- | --- | --- | --- | --- | --- | --- | --- | --- | --- | --- | --- | --- | --- | --- | --- | --- | --- | --- | --- | --- | --- | --- |
| R.IGFIEEVK.A | Y | 41.50 | 933.5171 | 8 | -0.1 | 467.7658 | 2 | 46.30 | 2 | F2:15099 | 09012020\_NaNaRj\_F2.raw |  | 0 |  |  |  |  |  |  |  |  | 1 | 0 | 1 | 0 | 0 | 0 | 0 | 0 | 0 | 0 | 0 | 232 | 239 |  |
| total 1 peptides |  |  |  |  |  |  |  |  |  |  |  |  |  |  |  |  |  |  |  |  |  |  |  |  |  |  |  |  |  |  |  |  |  |  |  |
| --- | --- | --- | --- | --- | --- | --- | --- | --- | --- | --- | --- | --- | --- | --- | --- | --- | --- | --- | --- | --- | --- | --- | --- | --- | --- | --- | --- | --- | --- | --- | --- | --- | --- | --- | --- |

A2SLF9|EFTU\_METPP

back to list

  

| Protein Coverage
| Supporting Peptides
|

Protein Coverage:

Supporting Peptides:

| Peptide | Uniq | -10lgP | Mass | Length | ppm | m/z | z | RT | Fraction | Scan | Source File | Area F1 | Area F2 | Area F3 | Area F4 | Area F5 | Area F6 | Area F7 | Area F8 | Area F9 | Area F10 | #Feature | #Feature F1 | #Feature F2 | #Feature F3 | #Feature F4 | #Feature F5 | #Feature F6 | #Feature F7 | #Feature F8 | #Feature F9 | #Feature F10 | Start | End | PTM |
| --- | --- | --- | --- | --- | --- | --- | --- | --- | --- | --- | --- | --- | --- | --- | --- | --- | --- | --- | --- | --- | --- | --- | --- | --- | --- | --- | --- | --- | --- | --- | --- | --- | --- | --- | --- |
| R.TTDVTGAVELPK.D | Y | 57.02 | 1229.6503 | 12 | 0.9 | 615.8329 | 2 | 41.12 | 2 | F2:12784 | 09012020\_NaNaRj\_F2.raw |  | 8.54E4 |  |  |  |  |  |  |  |  | 1 | 0 | 1 | 0 | 0 | 0 | 0 | 0 | 0 | 0 | 0 | 337 | 348 |  |
| total 1 peptides |  |  |  |  |  |  |  |  |  |  |  |  |  |  |  |  |  |  |  |  |  |  |  |  |  |  |  |  |  |  |  |  |  |  |  |
| --- | --- | --- | --- | --- | --- | --- | --- | --- | --- | --- | --- | --- | --- | --- | --- | --- | --- | --- | --- | --- | --- | --- | --- | --- | --- | --- | --- | --- | --- | --- | --- | --- | --- | --- | --- |

B8HVR7|EFTU\_CYAP4

back to list

  

| Protein Coverage
| Supporting Peptides
|

Protein Coverage:

Supporting Peptides:

| Peptide | Uniq | -10lgP | Mass | Length | ppm | m/z | z | RT | Fraction | Scan | Source File | Area F1 | Area F2 | Area F3 | Area F4 | Area F5 | Area F6 | Area F7 | Area F8 | Area F9 | Area F10 | #Feature | #Feature F1 | #Feature F2 | #Feature F3 | #Feature F4 | #Feature F5 | #Feature F6 | #Feature F7 | #Feature F8 | #Feature F9 | #Feature F10 | Start | End | PTM |
| --- | --- | --- | --- | --- | --- | --- | --- | --- | --- | --- | --- | --- | --- | --- | --- | --- | --- | --- | --- | --- | --- | --- | --- | --- | --- | --- | --- | --- | --- | --- | --- | --- | --- | --- | --- |
| Y.DFDGDNIPIIR.G | Y | 56.51 | 1273.6302 | 11 | 0.8 | 637.8229 | 2 | 62.89 | 2 | F2:20775 | 09012020\_NaNaRj\_F2.raw |  | 5.04E4 |  |  |  |  |  |  |  |  | 1 | 0 | 1 | 0 | 0 | 0 | 0 | 0 | 0 | 0 | 0 | 162 | 172 |  |
| total 1 peptides |  |  |  |  |  |  |  |  |  |  |  |  |  |  |  |  |  |  |  |  |  |  |  |  |  |  |  |  |  |  |  |  |  |  |  |
| --- | --- | --- | --- | --- | --- | --- | --- | --- | --- | --- | --- | --- | --- | --- | --- | --- | --- | --- | --- | --- | --- | --- | --- | --- | --- | --- | --- | --- | --- | --- | --- | --- | --- | --- | --- |

A4VVK7|DNLJ\_STRSY

back to list

  

| Protein Coverage
| Supporting Peptides
|

Protein Coverage:

Supporting Peptides:

| Peptide | Uniq | -10lgP | Mass | Length | ppm | m/z | z | RT | Fraction | Scan | Source File | Area F1 | Area F2 | Area F3 | Area F4 | Area F5 | Area F6 | Area F7 | Area F8 | Area F9 | Area F10 | #Feature | #Feature F1 | #Feature F2 | #Feature F3 | #Feature F4 | #Feature F5 | #Feature F6 | #Feature F7 | #Feature F8 | #Feature F9 | #Feature F10 | Start | End | PTM |
| --- | --- | --- | --- | --- | --- | --- | --- | --- | --- | --- | --- | --- | --- | --- | --- | --- | --- | --- | --- | --- | --- | --- | --- | --- | --- | --- | --- | --- | --- | --- | --- | --- | --- | --- | --- |
| H.YEDEVALR.C | Y | 56.47 | 993.4767 | 8 | 0.4 | 497.7458 | 2 | 11.46 | 5 | F5:2314 | 05112019\_RID\_1313\_NaNaRj\_F5.raw |  |  |  |  | 2.43E4 |  |  |  |  |  | 1 | 0 | 0 | 0 | 0 | 1 | 0 | 0 | 0 | 0 | 0 | 405 | 412 |  |
| total 1 peptides |  |  |  |  |  |  |  |  |  |  |  |  |  |  |  |  |  |  |  |  |  |  |  |  |  |  |  |  |  |  |  |  |  |  |  |
| --- | --- | --- | --- | --- | --- | --- | --- | --- | --- | --- | --- | --- | --- | --- | --- | --- | --- | --- | --- | --- | --- | --- | --- | --- | --- | --- | --- | --- | --- | --- | --- | --- | --- | --- | --- |

A4W1W6|DNLJ\_STRS2

back to list

  

| Protein Coverage
| Supporting Peptides
|

Protein Coverage:

Supporting Peptides:

| Peptide | Uniq | -10lgP | Mass | Length | ppm | m/z | z | RT | Fraction | Scan | Source File | Area F1 | Area F2 | Area F3 | Area F4 | Area F5 | Area F6 | Area F7 | Area F8 | Area F9 | Area F10 | #Feature | #Feature F1 | #Feature F2 | #Feature F3 | #Feature F4 | #Feature F5 | #Feature F6 | #Feature F7 | #Feature F8 | #Feature F9 | #Feature F10 | Start | End | PTM |
| --- | --- | --- | --- | --- | --- | --- | --- | --- | --- | --- | --- | --- | --- | --- | --- | --- | --- | --- | --- | --- | --- | --- | --- | --- | --- | --- | --- | --- | --- | --- | --- | --- | --- | --- | --- |
| H.YEDEVALR.C | Y | 56.47 | 993.4767 | 8 | 0.4 | 497.7458 | 2 | 11.46 | 5 | F5:2314 | 05112019\_RID\_1313\_NaNaRj\_F5.raw |  |  |  |  | 2.43E4 |  |  |  |  |  | 1 | 0 | 0 | 0 | 0 | 1 | 0 | 0 | 0 | 0 | 0 | 405 | 412 |  |
| total 1 peptides |  |  |  |  |  |  |  |  |  |  |  |  |  |  |  |  |  |  |  |  |  |  |  |  |  |  |  |  |  |  |  |  |  |  |  |
| --- | --- | --- | --- | --- | --- | --- | --- | --- | --- | --- | --- | --- | --- | --- | --- | --- | --- | --- | --- | --- | --- | --- | --- | --- | --- | --- | --- | --- | --- | --- | --- | --- | --- | --- | --- |

B2KE31|DNLJ\_ELUMP

back to list

  

| Protein Coverage
| Supporting Peptides
|

Protein Coverage:

Supporting Peptides:

| Peptide | Uniq | -10lgP | Mass | Length | ppm | m/z | z | RT | Fraction | Scan | Source File | Area F1 | Area F2 | Area F3 | Area F4 | Area F5 | Area F6 | Area F7 | Area F8 | Area F9 | Area F10 | #Feature | #Feature F1 | #Feature F2 | #Feature F3 | #Feature F4 | #Feature F5 | #Feature F6 | #Feature F7 | #Feature F8 | #Feature F9 | #Feature F10 | Start | End | PTM |
| --- | --- | --- | --- | --- | --- | --- | --- | --- | --- | --- | --- | --- | --- | --- | --- | --- | --- | --- | --- | --- | --- | --- | --- | --- | --- | --- | --- | --- | --- | --- | --- | --- | --- | --- | --- |
| R.YEDEVAIR.C | Y | 56.47 | 993.4767 | 8 | 0.4 | 497.7458 | 2 | 11.46 | 5 | F5:2314 | 05112019\_RID\_1313\_NaNaRj\_F5.raw |  |  |  |  | 2.43E4 |  |  |  |  |  | 1 | 0 | 0 | 0 | 0 | 1 | 0 | 0 | 0 | 0 | 0 | 409 | 416 |  |
| total 1 peptides |  |  |  |  |  |  |  |  |  |  |  |  |  |  |  |  |  |  |  |  |  |  |  |  |  |  |  |  |  |  |  |  |  |  |  |
| --- | --- | --- | --- | --- | --- | --- | --- | --- | --- | --- | --- | --- | --- | --- | --- | --- | --- | --- | --- | --- | --- | --- | --- | --- | --- | --- | --- | --- | --- | --- | --- | --- | --- | --- | --- |

A2YVE5|H2A3\_ORYSI

back to list

  

| Protein Coverage
| Supporting Peptides
|

Protein Coverage:

Supporting Peptides:

| Peptide | Uniq | -10lgP | Mass | Length | ppm | m/z | z | RT | Fraction | Scan | Source File | Area F1 | Area F2 | Area F3 | Area F4 | Area F5 | Area F6 | Area F7 | Area F8 | Area F9 | Area F10 | #Feature | #Feature F1 | #Feature F2 | #Feature F3 | #Feature F4 | #Feature F5 | #Feature F6 | #Feature F7 | #Feature F8 | #Feature F9 | #Feature F10 | Start | End | PTM |
| --- | --- | --- | --- | --- | --- | --- | --- | --- | --- | --- | --- | --- | --- | --- | --- | --- | --- | --- | --- | --- | --- | --- | --- | --- | --- | --- | --- | --- | --- | --- | --- | --- | --- | --- | --- |
| K.AGLQFPVGR.I | Y | 52.83 | 943.5239 | 9 | 0.5 | 472.7695 | 2 | 45.81 | 2 | F2:14894 | 09012020\_NaNaRj\_F2.raw |  | 6.76E4 |  |  |  |  |  |  |  |  | 1 | 0 | 1 | 0 | 0 | 0 | 0 | 0 | 0 | 0 | 0 | 23 | 31 |  |
| total 1 peptides |  |  |  |  |  |  |  |  |  |  |  |  |  |  |  |  |  |  |  |  |  |  |  |  |  |  |  |  |  |  |  |  |  |  |  |
| --- | --- | --- | --- | --- | --- | --- | --- | --- | --- | --- | --- | --- | --- | --- | --- | --- | --- | --- | --- | --- | --- | --- | --- | --- | --- | --- | --- | --- | --- | --- | --- | --- | --- | --- | --- |

Q8LLP5|H2AXA\_ORYSJ

back to list

  

| Protein Coverage
| Supporting Peptides
|

Protein Coverage:

Supporting Peptides:

| Peptide | Uniq | -10lgP | Mass | Length | ppm | m/z | z | RT | Fraction | Scan | Source File | Area F1 | Area F2 | Area F3 | Area F4 | Area F5 | Area F6 | Area F7 | Area F8 | Area F9 | Area F10 | #Feature | #Feature F1 | #Feature F2 | #Feature F3 | #Feature F4 | #Feature F5 | #Feature F6 | #Feature F7 | #Feature F8 | #Feature F9 | #Feature F10 | Start | End | PTM |
| --- | --- | --- | --- | --- | --- | --- | --- | --- | --- | --- | --- | --- | --- | --- | --- | --- | --- | --- | --- | --- | --- | --- | --- | --- | --- | --- | --- | --- | --- | --- | --- | --- | --- | --- | --- |
| K.AGLQFPVGR.I | Y | 52.83 | 943.5239 | 9 | 0.5 | 472.7695 | 2 | 45.81 | 2 | F2:14894 | 09012020\_NaNaRj\_F2.raw |  | 6.76E4 |  |  |  |  |  |  |  |  | 1 | 0 | 1 | 0 | 0 | 0 | 0 | 0 | 0 | 0 | 0 | 25 | 33 |  |
| total 1 peptides |  |  |  |  |  |  |  |  |  |  |  |  |  |  |  |  |  |  |  |  |  |  |  |  |  |  |  |  |  |  |  |  |  |  |  |
| --- | --- | --- | --- | --- | --- | --- | --- | --- | --- | --- | --- | --- | --- | --- | --- | --- | --- | --- | --- | --- | --- | --- | --- | --- | --- | --- | --- | --- | --- | --- | --- | --- | --- | --- | --- |

P69142|H2AE\_PSAMI

back to list

  

| Protein Coverage
| Supporting Peptides
|

Protein Coverage:

Supporting Peptides:

| Peptide | Uniq | -10lgP | Mass | Length | ppm | m/z | z | RT | Fraction | Scan | Source File | Area F1 | Area F2 | Area F3 | Area F4 | Area F5 | Area F6 | Area F7 | Area F8 | Area F9 | Area F10 | #Feature | #Feature F1 | #Feature F2 | #Feature F3 | #Feature F4 | #Feature F5 | #Feature F6 | #Feature F7 | #Feature F8 | #Feature F9 | #Feature F10 | Start | End | PTM |
| --- | --- | --- | --- | --- | --- | --- | --- | --- | --- | --- | --- | --- | --- | --- | --- | --- | --- | --- | --- | --- | --- | --- | --- | --- | --- | --- | --- | --- | --- | --- | --- | --- | --- | --- | --- |
| R.AGLQFPVGR.V | Y | 52.83 | 943.5239 | 9 | 0.5 | 472.7695 | 2 | 45.81 | 2 | F2:14894 | 09012020\_NaNaRj\_F2.raw |  | 6.76E4 |  |  |  |  |  |  |  |  | 1 | 0 | 1 | 0 | 0 | 0 | 0 | 0 | 0 | 0 | 0 | 21 | 29 |  |
| total 1 peptides |  |  |  |  |  |  |  |  |  |  |  |  |  |  |  |  |  |  |  |  |  |  |  |  |  |  |  |  |  |  |  |  |  |  |  |
| --- | --- | --- | --- | --- | --- | --- | --- | --- | --- | --- | --- | --- | --- | --- | --- | --- | --- | --- | --- | --- | --- | --- | --- | --- | --- | --- | --- | --- | --- | --- | --- | --- | --- | --- | --- |

Q3ZBX9|H2AJ\_BOVIN

back to list

  

| Protein Coverage
| Supporting Peptides
|

Protein Coverage:

Supporting Peptides:

| Peptide | Uniq | -10lgP | Mass | Length | ppm | m/z | z | RT | Fraction | Scan | Source File | Area F1 | Area F2 | Area F3 | Area F4 | Area F5 | Area F6 | Area F7 | Area F8 | Area F9 | Area F10 | #Feature | #Feature F1 | #Feature F2 | #Feature F3 | #Feature F4 | #Feature F5 | #Feature F6 | #Feature F7 | #Feature F8 | #Feature F9 | #Feature F10 | Start | End | PTM |
| --- | --- | --- | --- | --- | --- | --- | --- | --- | --- | --- | --- | --- | --- | --- | --- | --- | --- | --- | --- | --- | --- | --- | --- | --- | --- | --- | --- | --- | --- | --- | --- | --- | --- | --- | --- |
| R.AGLQFPVGR.V | Y | 52.83 | 943.5239 | 9 | 0.5 | 472.7695 | 2 | 45.81 | 2 | F2:14894 | 09012020\_NaNaRj\_F2.raw |  | 6.76E4 |  |  |  |  |  |  |  |  | 1 | 0 | 1 | 0 | 0 | 0 | 0 | 0 | 0 | 0 | 0 | 22 | 30 |  |
| total 1 peptides |  |  |  |  |  |  |  |  |  |  |  |  |  |  |  |  |  |  |  |  |  |  |  |  |  |  |  |  |  |  |  |  |  |  |  |
| --- | --- | --- | --- | --- | --- | --- | --- | --- | --- | --- | --- | --- | --- | --- | --- | --- | --- | --- | --- | --- | --- | --- | --- | --- | --- | --- | --- | --- | --- | --- | --- | --- | --- | --- | --- |

Q6WV67|H2A\_MYTTR

back to list

  

| Protein Coverage
| Supporting Peptides
|

Protein Coverage:

Supporting Peptides:

| Peptide | Uniq | -10lgP | Mass | Length | ppm | m/z | z | RT | Fraction | Scan | Source File | Area F1 | Area F2 | Area F3 | Area F4 | Area F5 | Area F6 | Area F7 | Area F8 | Area F9 | Area F10 | #Feature | #Feature F1 | #Feature F2 | #Feature F3 | #Feature F4 | #Feature F5 | #Feature F6 | #Feature F7 | #Feature F8 | #Feature F9 | #Feature F10 | Start | End | PTM |
| --- | --- | --- | --- | --- | --- | --- | --- | --- | --- | --- | --- | --- | --- | --- | --- | --- | --- | --- | --- | --- | --- | --- | --- | --- | --- | --- | --- | --- | --- | --- | --- | --- | --- | --- | --- |
| R.AGLQFPVGR.I | Y | 52.83 | 943.5239 | 9 | 0.5 | 472.7695 | 2 | 45.81 | 2 | F2:14894 | 09012020\_NaNaRj\_F2.raw |  | 6.76E4 |  |  |  |  |  |  |  |  | 1 | 0 | 1 | 0 | 0 | 0 | 0 | 0 | 0 | 0 | 0 | 21 | 29 |  |
| total 1 peptides |  |  |  |  |  |  |  |  |  |  |  |  |  |  |  |  |  |  |  |  |  |  |  |  |  |  |  |  |  |  |  |  |  |  |  |
| --- | --- | --- | --- | --- | --- | --- | --- | --- | --- | --- | --- | --- | --- | --- | --- | --- | --- | --- | --- | --- | --- | --- | --- | --- | --- | --- | --- | --- | --- | --- | --- | --- | --- | --- | --- |

Q8I0T3|H2A\_MYTED

back to list

  

| Protein Coverage
| Supporting Peptides
|

Protein Coverage:

Supporting Peptides:

| Peptide | Uniq | -10lgP | Mass | Length | ppm | m/z | z | RT | Fraction | Scan | Source File | Area F1 | Area F2 | Area F3 | Area F4 | Area F5 | Area F6 | Area F7 | Area F8 | Area F9 | Area F10 | #Feature | #Feature F1 | #Feature F2 | #Feature F3 | #Feature F4 | #Feature F5 | #Feature F6 | #Feature F7 | #Feature F8 | #Feature F9 | #Feature F10 | Start | End | PTM |
| --- | --- | --- | --- | --- | --- | --- | --- | --- | --- | --- | --- | --- | --- | --- | --- | --- | --- | --- | --- | --- | --- | --- | --- | --- | --- | --- | --- | --- | --- | --- | --- | --- | --- | --- | --- |
| R.AGLQFPVGR.I | Y | 52.83 | 943.5239 | 9 | 0.5 | 472.7695 | 2 | 45.81 | 2 | F2:14894 | 09012020\_NaNaRj\_F2.raw |  | 6.76E4 |  |  |  |  |  |  |  |  | 1 | 0 | 1 | 0 | 0 | 0 | 0 | 0 | 0 | 0 | 0 | 21 | 29 |  |
| total 1 peptides |  |  |  |  |  |  |  |  |  |  |  |  |  |  |  |  |  |  |  |  |  |  |  |  |  |  |  |  |  |  |  |  |  |  |  |
| --- | --- | --- | --- | --- | --- | --- | --- | --- | --- | --- | --- | --- | --- | --- | --- | --- | --- | --- | --- | --- | --- | --- | --- | --- | --- | --- | --- | --- | --- | --- | --- | --- | --- | --- | --- |

Q6WV88|H2A\_MYTGA

back to list

  

| Protein Coverage
| Supporting Peptides
|

Protein Coverage:

Supporting Peptides:

| Peptide | Uniq | -10lgP | Mass | Length | ppm | m/z | z | RT | Fraction | Scan | Source File | Area F1 | Area F2 | Area F3 | Area F4 | Area F5 | Area F6 | Area F7 | Area F8 | Area F9 | Area F10 | #Feature | #Feature F1 | #Feature F2 | #Feature F3 | #Feature F4 | #Feature F5 | #Feature F6 | #Feature F7 | #Feature F8 | #Feature F9 | #Feature F10 | Start | End | PTM |
| --- | --- | --- | --- | --- | --- | --- | --- | --- | --- | --- | --- | --- | --- | --- | --- | --- | --- | --- | --- | --- | --- | --- | --- | --- | --- | --- | --- | --- | --- | --- | --- | --- | --- | --- | --- |
| R.AGLQFPVGR.I | Y | 52.83 | 943.5239 | 9 | 0.5 | 472.7695 | 2 | 45.81 | 2 | F2:14894 | 09012020\_NaNaRj\_F2.raw |  | 6.76E4 |  |  |  |  |  |  |  |  | 1 | 0 | 1 | 0 | 0 | 0 | 0 | 0 | 0 | 0 | 0 | 21 | 29 |  |
| total 1 peptides |  |  |  |  |  |  |  |  |  |  |  |  |  |  |  |  |  |  |  |  |  |  |  |  |  |  |  |  |  |  |  |  |  |  |  |
| --- | --- | --- | --- | --- | --- | --- | --- | --- | --- | --- | --- | --- | --- | --- | --- | --- | --- | --- | --- | --- | --- | --- | --- | --- | --- | --- | --- | --- | --- | --- | --- | --- | --- | --- | --- |

Q07135|H2AO\_CHITH

back to list

  

| Protein Coverage
| Supporting Peptides
|

Protein Coverage:

Supporting Peptides:

| Peptide | Uniq | -10lgP | Mass | Length | ppm | m/z | z | RT | Fraction | Scan | Source File | Area F1 | Area F2 | Area F3 | Area F4 | Area F5 | Area F6 | Area F7 | Area F8 | Area F9 | Area F10 | #Feature | #Feature F1 | #Feature F2 | #Feature F3 | #Feature F4 | #Feature F5 | #Feature F6 | #Feature F7 | #Feature F8 | #Feature F9 | #Feature F10 | Start | End | PTM |
| --- | --- | --- | --- | --- | --- | --- | --- | --- | --- | --- | --- | --- | --- | --- | --- | --- | --- | --- | --- | --- | --- | --- | --- | --- | --- | --- | --- | --- | --- | --- | --- | --- | --- | --- | --- |
| R.AGLQFPVGR.I | Y | 52.83 | 943.5239 | 9 | 0.5 | 472.7695 | 2 | 45.81 | 2 | F2:14894 | 09012020\_NaNaRj\_F2.raw |  | 6.76E4 |  |  |  |  |  |  |  |  | 1 | 0 | 1 | 0 | 0 | 0 | 0 | 0 | 0 | 0 | 0 | 21 | 29 |  |
| total 1 peptides |  |  |  |  |  |  |  |  |  |  |  |  |  |  |  |  |  |  |  |  |  |  |  |  |  |  |  |  |  |  |  |  |  |  |  |
| --- | --- | --- | --- | --- | --- | --- | --- | --- | --- | --- | --- | --- | --- | --- | --- | --- | --- | --- | --- | --- | --- | --- | --- | --- | --- | --- | --- | --- | --- | --- | --- | --- | --- | --- | --- |

Q5ZMD6|H2AZ\_CHICK

back to list

  

| Protein Coverage
| Supporting Peptides
|

Protein Coverage:

Supporting Peptides:

| Peptide | Uniq | -10lgP | Mass | Length | ppm | m/z | z | RT | Fraction | Scan | Source File | Area F1 | Area F2 | Area F3 | Area F4 | Area F5 | Area F6 | Area F7 | Area F8 | Area F9 | Area F10 | #Feature | #Feature F1 | #Feature F2 | #Feature F3 | #Feature F4 | #Feature F5 | #Feature F6 | #Feature F7 | #Feature F8 | #Feature F9 | #Feature F10 | Start | End | PTM |
| --- | --- | --- | --- | --- | --- | --- | --- | --- | --- | --- | --- | --- | --- | --- | --- | --- | --- | --- | --- | --- | --- | --- | --- | --- | --- | --- | --- | --- | --- | --- | --- | --- | --- | --- | --- |
| R.AGLQFPVGR.I | Y | 52.83 | 943.5239 | 9 | 0.5 | 472.7695 | 2 | 45.81 | 2 | F2:14894 | 09012020\_NaNaRj\_F2.raw |  | 6.76E4 |  |  |  |  |  |  |  |  | 1 | 0 | 1 | 0 | 0 | 0 | 0 | 0 | 0 | 0 | 0 | 24 | 32 |  |
| total 1 peptides |  |  |  |  |  |  |  |  |  |  |  |  |  |  |  |  |  |  |  |  |  |  |  |  |  |  |  |  |  |  |  |  |  |  |  |
| --- | --- | --- | --- | --- | --- | --- | --- | --- | --- | --- | --- | --- | --- | --- | --- | --- | --- | --- | --- | --- | --- | --- | --- | --- | --- | --- | --- | --- | --- | --- | --- | --- | --- | --- | --- |

Q32LA7|H2AV\_BOVIN

back to list

  

| Protein Coverage
| Supporting Peptides
|

Protein Coverage:

Supporting Peptides:

| Peptide | Uniq | -10lgP | Mass | Length | ppm | m/z | z | RT | Fraction | Scan | Source File | Area F1 | Area F2 | Area F3 | Area F4 | Area F5 | Area F6 | Area F7 | Area F8 | Area F9 | Area F10 | #Feature | #Feature F1 | #Feature F2 | #Feature F3 | #Feature F4 | #Feature F5 | #Feature F6 | #Feature F7 | #Feature F8 | #Feature F9 | #Feature F10 | Start | End | PTM |
| --- | --- | --- | --- | --- | --- | --- | --- | --- | --- | --- | --- | --- | --- | --- | --- | --- | --- | --- | --- | --- | --- | --- | --- | --- | --- | --- | --- | --- | --- | --- | --- | --- | --- | --- | --- |
| R.AGLQFPVGR.I | Y | 52.83 | 943.5239 | 9 | 0.5 | 472.7695 | 2 | 45.81 | 2 | F2:14894 | 09012020\_NaNaRj\_F2.raw |  | 6.76E4 |  |  |  |  |  |  |  |  | 1 | 0 | 1 | 0 | 0 | 0 | 0 | 0 | 0 | 0 | 0 | 24 | 32 |  |
| total 1 peptides |  |  |  |  |  |  |  |  |  |  |  |  |  |  |  |  |  |  |  |  |  |  |  |  |  |  |  |  |  |  |  |  |  |  |  |
| --- | --- | --- | --- | --- | --- | --- | --- | --- | --- | --- | --- | --- | --- | --- | --- | --- | --- | --- | --- | --- | --- | --- | --- | --- | --- | --- | --- | --- | --- | --- | --- | --- | --- | --- | --- |

P22647|H2AZ\_ONCMY

back to list

  

| Protein Coverage
| Supporting Peptides
|

Protein Coverage:

Supporting Peptides:

| Peptide | Uniq | -10lgP | Mass | Length | ppm | m/z | z | RT | Fraction | Scan | Source File | Area F1 | Area F2 | Area F3 | Area F4 | Area F5 | Area F6 | Area F7 | Area F8 | Area F9 | Area F10 | #Feature | #Feature F1 | #Feature F2 | #Feature F3 | #Feature F4 | #Feature F5 | #Feature F6 | #Feature F7 | #Feature F8 | #Feature F9 | #Feature F10 | Start | End | PTM |
| --- | --- | --- | --- | --- | --- | --- | --- | --- | --- | --- | --- | --- | --- | --- | --- | --- | --- | --- | --- | --- | --- | --- | --- | --- | --- | --- | --- | --- | --- | --- | --- | --- | --- | --- | --- |
| R.AGLQFPVGR.I | Y | 52.83 | 943.5239 | 9 | 0.5 | 472.7695 | 2 | 45.81 | 2 | F2:14894 | 09012020\_NaNaRj\_F2.raw |  | 6.76E4 |  |  |  |  |  |  |  |  | 1 | 0 | 1 | 0 | 0 | 0 | 0 | 0 | 0 | 0 | 0 | 24 | 32 |  |
| total 1 peptides |  |  |  |  |  |  |  |  |  |  |  |  |  |  |  |  |  |  |  |  |  |  |  |  |  |  |  |  |  |  |  |  |  |  |  |
| --- | --- | --- | --- | --- | --- | --- | --- | --- | --- | --- | --- | --- | --- | --- | --- | --- | --- | --- | --- | --- | --- | --- | --- | --- | --- | --- | --- | --- | --- | --- | --- | --- | --- | --- | --- |

Q5RC42|H2AZ\_PONAB

back to list

  

| Protein Coverage
| Supporting Peptides
|

Protein Coverage:

Supporting Peptides:

| Peptide | Uniq | -10lgP | Mass | Length | ppm | m/z | z | RT | Fraction | Scan | Source File | Area F1 | Area F2 | Area F3 | Area F4 | Area F5 | Area F6 | Area F7 | Area F8 | Area F9 | Area F10 | #Feature | #Feature F1 | #Feature F2 | #Feature F3 | #Feature F4 | #Feature F5 | #Feature F6 | #Feature F7 | #Feature F8 | #Feature F9 | #Feature F10 | Start | End | PTM |
| --- | --- | --- | --- | --- | --- | --- | --- | --- | --- | --- | --- | --- | --- | --- | --- | --- | --- | --- | --- | --- | --- | --- | --- | --- | --- | --- | --- | --- | --- | --- | --- | --- | --- | --- | --- |
| R.AGLQFPVGR.I | Y | 52.83 | 943.5239 | 9 | 0.5 | 472.7695 | 2 | 45.81 | 2 | F2:14894 | 09012020\_NaNaRj\_F2.raw |  | 6.76E4 |  |  |  |  |  |  |  |  | 1 | 0 | 1 | 0 | 0 | 0 | 0 | 0 | 0 | 0 | 0 | 24 | 32 |  |
| total 1 peptides |  |  |  |  |  |  |  |  |  |  |  |  |  |  |  |  |  |  |  |  |  |  |  |  |  |  |  |  |  |  |  |  |  |  |  |
| --- | --- | --- | --- | --- | --- | --- | --- | --- | --- | --- | --- | --- | --- | --- | --- | --- | --- | --- | --- | --- | --- | --- | --- | --- | --- | --- | --- | --- | --- | --- | --- | --- | --- | --- | --- |

P70094|H2AZL\_XENLA

back to list

  

| Protein Coverage
| Supporting Peptides
|

Protein Coverage:

Supporting Peptides:

| Peptide | Uniq | -10lgP | Mass | Length | ppm | m/z | z | RT | Fraction | Scan | Source File | Area F1 | Area F2 | Area F3 | Area F4 | Area F5 | Area F6 | Area F7 | Area F8 | Area F9 | Area F10 | #Feature | #Feature F1 | #Feature F2 | #Feature F3 | #Feature F4 | #Feature F5 | #Feature F6 | #Feature F7 | #Feature F8 | #Feature F9 | #Feature F10 | Start | End | PTM |
| --- | --- | --- | --- | --- | --- | --- | --- | --- | --- | --- | --- | --- | --- | --- | --- | --- | --- | --- | --- | --- | --- | --- | --- | --- | --- | --- | --- | --- | --- | --- | --- | --- | --- | --- | --- |
| R.AGLQFPVGR.I | Y | 52.83 | 943.5239 | 9 | 0.5 | 472.7695 | 2 | 45.81 | 2 | F2:14894 | 09012020\_NaNaRj\_F2.raw |  | 6.76E4 |  |  |  |  |  |  |  |  | 1 | 0 | 1 | 0 | 0 | 0 | 0 | 0 | 0 | 0 | 0 | 24 | 32 |  |
| total 1 peptides |  |  |  |  |  |  |  |  |  |  |  |  |  |  |  |  |  |  |  |  |  |  |  |  |  |  |  |  |  |  |  |  |  |  |  |
| --- | --- | --- | --- | --- | --- | --- | --- | --- | --- | --- | --- | --- | --- | --- | --- | --- | --- | --- | --- | --- | --- | --- | --- | --- | --- | --- | --- | --- | --- | --- | --- | --- | --- | --- | --- |

P70082|H2AJ\_CHICK

back to list

  

| Protein Coverage
| Supporting Peptides
|

Protein Coverage:

Supporting Peptides:

| Peptide | Uniq | -10lgP | Mass | Length | ppm | m/z | z | RT | Fraction | Scan | Source File | Area F1 | Area F2 | Area F3 | Area F4 | Area F5 | Area F6 | Area F7 | Area F8 | Area F9 | Area F10 | #Feature | #Feature F1 | #Feature F2 | #Feature F3 | #Feature F4 | #Feature F5 | #Feature F6 | #Feature F7 | #Feature F8 | #Feature F9 | #Feature F10 | Start | End | PTM |
| --- | --- | --- | --- | --- | --- | --- | --- | --- | --- | --- | --- | --- | --- | --- | --- | --- | --- | --- | --- | --- | --- | --- | --- | --- | --- | --- | --- | --- | --- | --- | --- | --- | --- | --- | --- |
| R.AGLQFPVGR.V | Y | 52.83 | 943.5239 | 9 | 0.5 | 472.7695 | 2 | 45.81 | 2 | F2:14894 | 09012020\_NaNaRj\_F2.raw |  | 6.76E4 |  |  |  |  |  |  |  |  | 1 | 0 | 1 | 0 | 0 | 0 | 0 | 0 | 0 | 0 | 0 | 22 | 30 |  |
| total 1 peptides |  |  |  |  |  |  |  |  |  |  |  |  |  |  |  |  |  |  |  |  |  |  |  |  |  |  |  |  |  |  |  |  |  |  |  |
| --- | --- | --- | --- | --- | --- | --- | --- | --- | --- | --- | --- | --- | --- | --- | --- | --- | --- | --- | --- | --- | --- | --- | --- | --- | --- | --- | --- | --- | --- | --- | --- | --- | --- | --- | --- |

P02263|H2A4\_CHICK

back to list

  

| Protein Coverage
| Supporting Peptides
|

Protein Coverage:

Supporting Peptides:

| Peptide | Uniq | -10lgP | Mass | Length | ppm | m/z | z | RT | Fraction | Scan | Source File | Area F1 | Area F2 | Area F3 | Area F4 | Area F5 | Area F6 | Area F7 | Area F8 | Area F9 | Area F10 | #Feature | #Feature F1 | #Feature F2 | #Feature F3 | #Feature F4 | #Feature F5 | #Feature F6 | #Feature F7 | #Feature F8 | #Feature F9 | #Feature F10 | Start | End | PTM |
| --- | --- | --- | --- | --- | --- | --- | --- | --- | --- | --- | --- | --- | --- | --- | --- | --- | --- | --- | --- | --- | --- | --- | --- | --- | --- | --- | --- | --- | --- | --- | --- | --- | --- | --- | --- |
| R.AGLQFPVGR.V | Y | 52.83 | 943.5239 | 9 | 0.5 | 472.7695 | 2 | 45.81 | 2 | F2:14894 | 09012020\_NaNaRj\_F2.raw |  | 6.76E4 |  |  |  |  |  |  |  |  | 1 | 0 | 1 | 0 | 0 | 0 | 0 | 0 | 0 | 0 | 0 | 22 | 30 |  |
| total 1 peptides |  |  |  |  |  |  |  |  |  |  |  |  |  |  |  |  |  |  |  |  |  |  |  |  |  |  |  |  |  |  |  |  |  |  |  |
| --- | --- | --- | --- | --- | --- | --- | --- | --- | --- | --- | --- | --- | --- | --- | --- | --- | --- | --- | --- | --- | --- | --- | --- | --- | --- | --- | --- | --- | --- | --- | --- | --- | --- | --- | --- |

A1A4R1|H2A2C\_BOVIN

back to list

  

| Protein Coverage
| Supporting Peptides
|

Protein Coverage:

Supporting Peptides:

| Peptide | Uniq | -10lgP | Mass | Length | ppm | m/z | z | RT | Fraction | Scan | Source File | Area F1 | Area F2 | Area F3 | Area F4 | Area F5 | Area F6 | Area F7 | Area F8 | Area F9 | Area F10 | #Feature | #Feature F1 | #Feature F2 | #Feature F3 | #Feature F4 | #Feature F5 | #Feature F6 | #Feature F7 | #Feature F8 | #Feature F9 | #Feature F10 | Start | End | PTM |
| --- | --- | --- | --- | --- | --- | --- | --- | --- | --- | --- | --- | --- | --- | --- | --- | --- | --- | --- | --- | --- | --- | --- | --- | --- | --- | --- | --- | --- | --- | --- | --- | --- | --- | --- | --- |
| R.AGLQFPVGR.V | Y | 52.83 | 943.5239 | 9 | 0.5 | 472.7695 | 2 | 45.81 | 2 | F2:14894 | 09012020\_NaNaRj\_F2.raw |  | 6.76E4 |  |  |  |  |  |  |  |  | 1 | 0 | 1 | 0 | 0 | 0 | 0 | 0 | 0 | 0 | 0 | 22 | 30 |  |
| total 1 peptides |  |  |  |  |  |  |  |  |  |  |  |  |  |  |  |  |  |  |  |  |  |  |  |  |  |  |  |  |  |  |  |  |  |  |  |
| --- | --- | --- | --- | --- | --- | --- | --- | --- | --- | --- | --- | --- | --- | --- | --- | --- | --- | --- | --- | --- | --- | --- | --- | --- | --- | --- | --- | --- | --- | --- | --- | --- | --- | --- | --- |

Q64523|H2A2C\_MOUSE

back to list

  

| Protein Coverage
| Supporting Peptides
|

Protein Coverage:

Supporting Peptides:

| Peptide | Uniq | -10lgP | Mass | Length | ppm | m/z | z | RT | Fraction | Scan | Source File | Area F1 | Area F2 | Area F3 | Area F4 | Area F5 | Area F6 | Area F7 | Area F8 | Area F9 | Area F10 | #Feature | #Feature F1 | #Feature F2 | #Feature F3 | #Feature F4 | #Feature F5 | #Feature F6 | #Feature F7 | #Feature F8 | #Feature F9 | #Feature F10 | Start | End | PTM |
| --- | --- | --- | --- | --- | --- | --- | --- | --- | --- | --- | --- | --- | --- | --- | --- | --- | --- | --- | --- | --- | --- | --- | --- | --- | --- | --- | --- | --- | --- | --- | --- | --- | --- | --- | --- |
| R.AGLQFPVGR.V | Y | 52.83 | 943.5239 | 9 | 0.5 | 472.7695 | 2 | 45.81 | 2 | F2:14894 | 09012020\_NaNaRj\_F2.raw |  | 6.76E4 |  |  |  |  |  |  |  |  | 1 | 0 | 1 | 0 | 0 | 0 | 0 | 0 | 0 | 0 | 0 | 22 | 30 |  |
| total 1 peptides |  |  |  |  |  |  |  |  |  |  |  |  |  |  |  |  |  |  |  |  |  |  |  |  |  |  |  |  |  |  |  |  |  |  |  |
| --- | --- | --- | --- | --- | --- | --- | --- | --- | --- | --- | --- | --- | --- | --- | --- | --- | --- | --- | --- | --- | --- | --- | --- | --- | --- | --- | --- | --- | --- | --- | --- | --- | --- | --- | --- |

Q16777|H2A2C\_HUMAN

back to list

  

| Protein Coverage
| Supporting Peptides
|

Protein Coverage:

Supporting Peptides:

| Peptide | Uniq | -10lgP | Mass | Length | ppm | m/z | z | RT | Fraction | Scan | Source File | Area F1 | Area F2 | Area F3 | Area F4 | Area F5 | Area F6 | Area F7 | Area F8 | Area F9 | Area F10 | #Feature | #Feature F1 | #Feature F2 | #Feature F3 | #Feature F4 | #Feature F5 | #Feature F6 | #Feature F7 | #Feature F8 | #Feature F9 | #Feature F10 | Start | End | PTM |
| --- | --- | --- | --- | --- | --- | --- | --- | --- | --- | --- | --- | --- | --- | --- | --- | --- | --- | --- | --- | --- | --- | --- | --- | --- | --- | --- | --- | --- | --- | --- | --- | --- | --- | --- | --- |
| R.AGLQFPVGR.V | Y | 52.83 | 943.5239 | 9 | 0.5 | 472.7695 | 2 | 45.81 | 2 | F2:14894 | 09012020\_NaNaRj\_F2.raw |  | 6.76E4 |  |  |  |  |  |  |  |  | 1 | 0 | 1 | 0 | 0 | 0 | 0 | 0 | 0 | 0 | 0 | 22 | 30 |  |
| total 1 peptides |  |  |  |  |  |  |  |  |  |  |  |  |  |  |  |  |  |  |  |  |  |  |  |  |  |  |  |  |  |  |  |  |  |  |  |
| --- | --- | --- | --- | --- | --- | --- | --- | --- | --- | --- | --- | --- | --- | --- | --- | --- | --- | --- | --- | --- | --- | --- | --- | --- | --- | --- | --- | --- | --- | --- | --- | --- | --- | --- | --- |

Q8BFU2|H2A3\_MOUSE

back to list

  

| Protein Coverage
| Supporting Peptides
|

Protein Coverage:

Supporting Peptides:

| Peptide | Uniq | -10lgP | Mass | Length | ppm | m/z | z | RT | Fraction | Scan | Source File | Area F1 | Area F2 | Area F3 | Area F4 | Area F5 | Area F6 | Area F7 | Area F8 | Area F9 | Area F10 | #Feature | #Feature F1 | #Feature F2 | #Feature F3 | #Feature F4 | #Feature F5 | #Feature F6 | #Feature F7 | #Feature F8 | #Feature F9 | #Feature F10 | Start | End | PTM |
| --- | --- | --- | --- | --- | --- | --- | --- | --- | --- | --- | --- | --- | --- | --- | --- | --- | --- | --- | --- | --- | --- | --- | --- | --- | --- | --- | --- | --- | --- | --- | --- | --- | --- | --- | --- |
| R.AGLQFPVGR.V | Y | 52.83 | 943.5239 | 9 | 0.5 | 472.7695 | 2 | 45.81 | 2 | F2:14894 | 09012020\_NaNaRj\_F2.raw |  | 6.76E4 |  |  |  |  |  |  |  |  | 1 | 0 | 1 | 0 | 0 | 0 | 0 | 0 | 0 | 0 | 0 | 22 | 30 |  |
| total 1 peptides |  |  |  |  |  |  |  |  |  |  |  |  |  |  |  |  |  |  |  |  |  |  |  |  |  |  |  |  |  |  |  |  |  |  |  |
| --- | --- | --- | --- | --- | --- | --- | --- | --- | --- | --- | --- | --- | --- | --- | --- | --- | --- | --- | --- | --- | --- | --- | --- | --- | --- | --- | --- | --- | --- | --- | --- | --- | --- | --- | --- |

P0CC09|H2A2A\_RAT

back to list

  

| Protein Coverage
| Supporting Peptides
|

Protein Coverage:

Supporting Peptides:

| Peptide | Uniq | -10lgP | Mass | Length | ppm | m/z | z | RT | Fraction | Scan | Source File | Area F1 | Area F2 | Area F3 | Area F4 | Area F5 | Area F6 | Area F7 | Area F8 | Area F9 | Area F10 | #Feature | #Feature F1 | #Feature F2 | #Feature F3 | #Feature F4 | #Feature F5 | #Feature F6 | #Feature F7 | #Feature F8 | #Feature F9 | #Feature F10 | Start | End | PTM |
| --- | --- | --- | --- | --- | --- | --- | --- | --- | --- | --- | --- | --- | --- | --- | --- | --- | --- | --- | --- | --- | --- | --- | --- | --- | --- | --- | --- | --- | --- | --- | --- | --- | --- | --- | --- |
| R.AGLQFPVGR.V | Y | 52.83 | 943.5239 | 9 | 0.5 | 472.7695 | 2 | 45.81 | 2 | F2:14894 | 09012020\_NaNaRj\_F2.raw |  | 6.76E4 |  |  |  |  |  |  |  |  | 1 | 0 | 1 | 0 | 0 | 0 | 0 | 0 | 0 | 0 | 0 | 22 | 30 |  |
| total 1 peptides |  |  |  |  |  |  |  |  |  |  |  |  |  |  |  |  |  |  |  |  |  |  |  |  |  |  |  |  |  |  |  |  |  |  |  |
| --- | --- | --- | --- | --- | --- | --- | --- | --- | --- | --- | --- | --- | --- | --- | --- | --- | --- | --- | --- | --- | --- | --- | --- | --- | --- | --- | --- | --- | --- | --- | --- | --- | --- | --- | --- |

P20671|H2A1D\_HUMAN

back to list

  

| Protein Coverage
| Supporting Peptides
|

Protein Coverage:

Supporting Peptides:

| Peptide | Uniq | -10lgP | Mass | Length | ppm | m/z | z | RT | Fraction | Scan | Source File | Area F1 | Area F2 | Area F3 | Area F4 | Area F5 | Area F6 | Area F7 | Area F8 | Area F9 | Area F10 | #Feature | #Feature F1 | #Feature F2 | #Feature F3 | #Feature F4 | #Feature F5 | #Feature F6 | #Feature F7 | #Feature F8 | #Feature F9 | #Feature F10 | Start | End | PTM |
| --- | --- | --- | --- | --- | --- | --- | --- | --- | --- | --- | --- | --- | --- | --- | --- | --- | --- | --- | --- | --- | --- | --- | --- | --- | --- | --- | --- | --- | --- | --- | --- | --- | --- | --- | --- |
| R.AGLQFPVGR.V | Y | 52.83 | 943.5239 | 9 | 0.5 | 472.7695 | 2 | 45.81 | 2 | F2:14894 | 09012020\_NaNaRj\_F2.raw |  | 6.76E4 |  |  |  |  |  |  |  |  | 1 | 0 | 1 | 0 | 0 | 0 | 0 | 0 | 0 | 0 | 0 | 22 | 30 |  |
| total 1 peptides |  |  |  |  |  |  |  |  |  |  |  |  |  |  |  |  |  |  |  |  |  |  |  |  |  |  |  |  |  |  |  |  |  |  |  |
| --- | --- | --- | --- | --- | --- | --- | --- | --- | --- | --- | --- | --- | --- | --- | --- | --- | --- | --- | --- | --- | --- | --- | --- | --- | --- | --- | --- | --- | --- | --- | --- | --- | --- | --- | --- |

Q64522|H2A2B\_MOUSE

back to list

  

| Protein Coverage
| Supporting Peptides
|

Protein Coverage:

Supporting Peptides:

| Peptide | Uniq | -10lgP | Mass | Length | ppm | m/z | z | RT | Fraction | Scan | Source File | Area F1 | Area F2 | Area F3 | Area F4 | Area F5 | Area F6 | Area F7 | Area F8 | Area F9 | Area F10 | #Feature | #Feature F1 | #Feature F2 | #Feature F3 | #Feature F4 | #Feature F5 | #Feature F6 | #Feature F7 | #Feature F8 | #Feature F9 | #Feature F10 | Start | End | PTM |
| --- | --- | --- | --- | --- | --- | --- | --- | --- | --- | --- | --- | --- | --- | --- | --- | --- | --- | --- | --- | --- | --- | --- | --- | --- | --- | --- | --- | --- | --- | --- | --- | --- | --- | --- | --- |
| R.AGLQFPVGR.V | Y | 52.83 | 943.5239 | 9 | 0.5 | 472.7695 | 2 | 45.81 | 2 | F2:14894 | 09012020\_NaNaRj\_F2.raw |  | 6.76E4 |  |  |  |  |  |  |  |  | 1 | 0 | 1 | 0 | 0 | 0 | 0 | 0 | 0 | 0 | 0 | 22 | 30 |  |
| total 1 peptides |  |  |  |  |  |  |  |  |  |  |  |  |  |  |  |  |  |  |  |  |  |  |  |  |  |  |  |  |  |  |  |  |  |  |  |
| --- | --- | --- | --- | --- | --- | --- | --- | --- | --- | --- | --- | --- | --- | --- | --- | --- | --- | --- | --- | --- | --- | --- | --- | --- | --- | --- | --- | --- | --- | --- | --- | --- | --- | --- | --- |

Q9LHQ5|H2A2\_ARATH

back to list

  

| Protein Coverage
| Supporting Peptides
|

Protein Coverage:

Supporting Peptides:

| Peptide | Uniq | -10lgP | Mass | Length | ppm | m/z | z | RT | Fraction | Scan | Source File | Area F1 | Area F2 | Area F3 | Area F4 | Area F5 | Area F6 | Area F7 | Area F8 | Area F9 | Area F10 | #Feature | #Feature F1 | #Feature F2 | #Feature F3 | #Feature F4 | #Feature F5 | #Feature F6 | #Feature F7 | #Feature F8 | #Feature F9 | #Feature F10 | Start | End | PTM |
| --- | --- | --- | --- | --- | --- | --- | --- | --- | --- | --- | --- | --- | --- | --- | --- | --- | --- | --- | --- | --- | --- | --- | --- | --- | --- | --- | --- | --- | --- | --- | --- | --- | --- | --- | --- |
| K.AGLQFPVGR.I | Y | 52.83 | 943.5239 | 9 | 0.5 | 472.7695 | 2 | 45.81 | 2 | F2:14894 | 09012020\_NaNaRj\_F2.raw |  | 6.76E4 |  |  |  |  |  |  |  |  | 1 | 0 | 1 | 0 | 0 | 0 | 0 | 0 | 0 | 0 | 0 | 23 | 31 |  |
| total 1 peptides |  |  |  |  |  |  |  |  |  |  |  |  |  |  |  |  |  |  |  |  |  |  |  |  |  |  |  |  |  |  |  |  |  |  |  |
| --- | --- | --- | --- | --- | --- | --- | --- | --- | --- | --- | --- | --- | --- | --- | --- | --- | --- | --- | --- | --- | --- | --- | --- | --- | --- | --- | --- | --- | --- | --- | --- | --- | --- | --- | --- |

Q6CUC8|H2AZ\_KLULA

back to list

  

| Protein Coverage
| Supporting Peptides
|

Protein Coverage:

Supporting Peptides:

| Peptide | Uniq | -10lgP | Mass | Length | ppm | m/z | z | RT | Fraction | Scan | Source File | Area F1 | Area F2 | Area F3 | Area F4 | Area F5 | Area F6 | Area F7 | Area F8 | Area F9 | Area F10 | #Feature | #Feature F1 | #Feature F2 | #Feature F3 | #Feature F4 | #Feature F5 | #Feature F6 | #Feature F7 | #Feature F8 | #Feature F9 | #Feature F10 | Start | End | PTM |
| --- | --- | --- | --- | --- | --- | --- | --- | --- | --- | --- | --- | --- | --- | --- | --- | --- | --- | --- | --- | --- | --- | --- | --- | --- | --- | --- | --- | --- | --- | --- | --- | --- | --- | --- | --- |
| R.AGLQFPVGR.V | Y | 52.83 | 943.5239 | 9 | 0.5 | 472.7695 | 2 | 45.81 | 2 | F2:14894 | 09012020\_NaNaRj\_F2.raw |  | 6.76E4 |  |  |  |  |  |  |  |  | 1 | 0 | 1 | 0 | 0 | 0 | 0 | 0 | 0 | 0 | 0 | 29 | 37 |  |
| total 1 peptides |  |  |  |  |  |  |  |  |  |  |  |  |  |  |  |  |  |  |  |  |  |  |  |  |  |  |  |  |  |  |  |  |  |  |  |
| --- | --- | --- | --- | --- | --- | --- | --- | --- | --- | --- | --- | --- | --- | --- | --- | --- | --- | --- | --- | --- | --- | --- | --- | --- | --- | --- | --- | --- | --- | --- | --- | --- | --- | --- | --- |

A3GHC1|H2AZ\_PICST

back to list

  

| Protein Coverage
| Supporting Peptides
|

Protein Coverage:

Supporting Peptides:

| Peptide | Uniq | -10lgP | Mass | Length | ppm | m/z | z | RT | Fraction | Scan | Source File | Area F1 | Area F2 | Area F3 | Area F4 | Area F5 | Area F6 | Area F7 | Area F8 | Area F9 | Area F10 | #Feature | #Feature F1 | #Feature F2 | #Feature F3 | #Feature F4 | #Feature F5 | #Feature F6 | #Feature F7 | #Feature F8 | #Feature F9 | #Feature F10 | Start | End | PTM |
| --- | --- | --- | --- | --- | --- | --- | --- | --- | --- | --- | --- | --- | --- | --- | --- | --- | --- | --- | --- | --- | --- | --- | --- | --- | --- | --- | --- | --- | --- | --- | --- | --- | --- | --- | --- |
| R.AGLQFPVGR.I | Y | 52.83 | 943.5239 | 9 | 0.5 | 472.7695 | 2 | 45.81 | 2 | F2:14894 | 09012020\_NaNaRj\_F2.raw |  | 6.76E4 |  |  |  |  |  |  |  |  | 1 | 0 | 1 | 0 | 0 | 0 | 0 | 0 | 0 | 0 | 0 | 28 | 36 |  |
| total 1 peptides |  |  |  |  |  |  |  |  |  |  |  |  |  |  |  |  |  |  |  |  |  |  |  |  |  |  |  |  |  |  |  |  |  |  |  |
| --- | --- | --- | --- | --- | --- | --- | --- | --- | --- | --- | --- | --- | --- | --- | --- | --- | --- | --- | --- | --- | --- | --- | --- | --- | --- | --- | --- | --- | --- | --- | --- | --- | --- | --- | --- |

Q9C944|H2AV3\_ARATH

back to list

  

| Protein Coverage
| Supporting Peptides
|

Protein Coverage:

Supporting Peptides:

| Peptide | Uniq | -10lgP | Mass | Length | ppm | m/z | z | RT | Fraction | Scan | Source File | Area F1 | Area F2 | Area F3 | Area F4 | Area F5 | Area F6 | Area F7 | Area F8 | Area F9 | Area F10 | #Feature | #Feature F1 | #Feature F2 | #Feature F3 | #Feature F4 | #Feature F5 | #Feature F6 | #Feature F7 | #Feature F8 | #Feature F9 | #Feature F10 | Start | End | PTM |
| --- | --- | --- | --- | --- | --- | --- | --- | --- | --- | --- | --- | --- | --- | --- | --- | --- | --- | --- | --- | --- | --- | --- | --- | --- | --- | --- | --- | --- | --- | --- | --- | --- | --- | --- | --- |
| R.AGLQFPVGR.V | Y | 52.83 | 943.5239 | 9 | 0.5 | 472.7695 | 2 | 45.81 | 2 | F2:14894 | 09012020\_NaNaRj\_F2.raw |  | 6.76E4 |  |  |  |  |  |  |  |  | 1 | 0 | 1 | 0 | 0 | 0 | 0 | 0 | 0 | 0 | 0 | 33 | 41 |  |
| total 1 peptides |  |  |  |  |  |  |  |  |  |  |  |  |  |  |  |  |  |  |  |  |  |  |  |  |  |  |  |  |  |  |  |  |  |  |  |
| --- | --- | --- | --- | --- | --- | --- | --- | --- | --- | --- | --- | --- | --- | --- | --- | --- | --- | --- | --- | --- | --- | --- | --- | --- | --- | --- | --- | --- | --- | --- | --- | --- | --- | --- | --- |

Q43208|H2A4\_WHEAT

back to list

  

| Protein Coverage
| Supporting Peptides
|

Protein Coverage:

Supporting Peptides:

| Peptide | Uniq | -10lgP | Mass | Length | ppm | m/z | z | RT | Fraction | Scan | Source File | Area F1 | Area F2 | Area F3 | Area F4 | Area F5 | Area F6 | Area F7 | Area F8 | Area F9 | Area F10 | #Feature | #Feature F1 | #Feature F2 | #Feature F3 | #Feature F4 | #Feature F5 | #Feature F6 | #Feature F7 | #Feature F8 | #Feature F9 | #Feature F10 | Start | End | PTM |
| --- | --- | --- | --- | --- | --- | --- | --- | --- | --- | --- | --- | --- | --- | --- | --- | --- | --- | --- | --- | --- | --- | --- | --- | --- | --- | --- | --- | --- | --- | --- | --- | --- | --- | --- | --- |
| K.AGLQFPVGR.I | Y | 52.83 | 943.5239 | 9 | 0.5 | 472.7695 | 2 | 45.81 | 2 | F2:14894 | 09012020\_NaNaRj\_F2.raw |  | 6.76E4 |  |  |  |  |  |  |  |  | 1 | 0 | 1 | 0 | 0 | 0 | 0 | 0 | 0 | 0 | 0 | 23 | 31 |  |
| total 1 peptides |  |  |  |  |  |  |  |  |  |  |  |  |  |  |  |  |  |  |  |  |  |  |  |  |  |  |  |  |  |  |  |  |  |  |  |
| --- | --- | --- | --- | --- | --- | --- | --- | --- | --- | --- | --- | --- | --- | --- | --- | --- | --- | --- | --- | --- | --- | --- | --- | --- | --- | --- | --- | --- | --- | --- | --- | --- | --- | --- | --- |

A2YMC6|H2A2\_ORYSI

back to list

  

| Protein Coverage
| Supporting Peptides
|

Protein Coverage:

Supporting Peptides:

| Peptide | Uniq | -10lgP | Mass | Length | ppm | m/z | z | RT | Fraction | Scan | Source File | Area F1 | Area F2 | Area F3 | Area F4 | Area F5 | Area F6 | Area F7 | Area F8 | Area F9 | Area F10 | #Feature | #Feature F1 | #Feature F2 | #Feature F3 | #Feature F4 | #Feature F5 | #Feature F6 | #Feature F7 | #Feature F8 | #Feature F9 | #Feature F10 | Start | End | PTM |
| --- | --- | --- | --- | --- | --- | --- | --- | --- | --- | --- | --- | --- | --- | --- | --- | --- | --- | --- | --- | --- | --- | --- | --- | --- | --- | --- | --- | --- | --- | --- | --- | --- | --- | --- | --- |
| K.AGLQFPVGR.I | Y | 52.83 | 943.5239 | 9 | 0.5 | 472.7695 | 2 | 45.81 | 2 | F2:14894 | 09012020\_NaNaRj\_F2.raw |  | 6.76E4 |  |  |  |  |  |  |  |  | 1 | 0 | 1 | 0 | 0 | 0 | 0 | 0 | 0 | 0 | 0 | 23 | 31 |  |
| total 1 peptides |  |  |  |  |  |  |  |  |  |  |  |  |  |  |  |  |  |  |  |  |  |  |  |  |  |  |  |  |  |  |  |  |  |  |  |
| --- | --- | --- | --- | --- | --- | --- | --- | --- | --- | --- | --- | --- | --- | --- | --- | --- | --- | --- | --- | --- | --- | --- | --- | --- | --- | --- | --- | --- | --- | --- | --- | --- | --- | --- | --- |

Q4PEF9|H2A\_USTMA

back to list

  

| Protein Coverage
| Supporting Peptides
|

Protein Coverage:

Supporting Peptides:

| Peptide | Uniq | -10lgP | Mass | Length | ppm | m/z | z | RT | Fraction | Scan | Source File | Area F1 | Area F2 | Area F3 | Area F4 | Area F5 | Area F6 | Area F7 | Area F8 | Area F9 | Area F10 | #Feature | #Feature F1 | #Feature F2 | #Feature F3 | #Feature F4 | #Feature F5 | #Feature F6 | #Feature F7 | #Feature F8 | #Feature F9 | #Feature F10 | Start | End | PTM |
| --- | --- | --- | --- | --- | --- | --- | --- | --- | --- | --- | --- | --- | --- | --- | --- | --- | --- | --- | --- | --- | --- | --- | --- | --- | --- | --- | --- | --- | --- | --- | --- | --- | --- | --- | --- |
| K.AGLQFPVGR.I | Y | 52.83 | 943.5239 | 9 | 0.5 | 472.7695 | 2 | 45.81 | 2 | F2:14894 | 09012020\_NaNaRj\_F2.raw |  | 6.76E4 |  |  |  |  |  |  |  |  | 1 | 0 | 1 | 0 | 0 | 0 | 0 | 0 | 0 | 0 | 0 | 25 | 33 |  |
| total 1 peptides |  |  |  |  |  |  |  |  |  |  |  |  |  |  |  |  |  |  |  |  |  |  |  |  |  |  |  |  |  |  |  |  |  |  |  |
| --- | --- | --- | --- | --- | --- | --- | --- | --- | --- | --- | --- | --- | --- | --- | --- | --- | --- | --- | --- | --- | --- | --- | --- | --- | --- | --- | --- | --- | --- | --- | --- | --- | --- | --- | --- |

Q84MP7|H2AV3\_ORYSJ

back to list

  

| Protein Coverage
| Supporting Peptides
|

Protein Coverage:

Supporting Peptides:

| Peptide | Uniq | -10lgP | Mass | Length | ppm | m/z | z | RT | Fraction | Scan | Source File | Area F1 | Area F2 | Area F3 | Area F4 | Area F5 | Area F6 | Area F7 | Area F8 | Area F9 | Area F10 | #Feature | #Feature F1 | #Feature F2 | #Feature F3 | #Feature F4 | #Feature F5 | #Feature F6 | #Feature F7 | #Feature F8 | #Feature F9 | #Feature F10 | Start | End | PTM |
| --- | --- | --- | --- | --- | --- | --- | --- | --- | --- | --- | --- | --- | --- | --- | --- | --- | --- | --- | --- | --- | --- | --- | --- | --- | --- | --- | --- | --- | --- | --- | --- | --- | --- | --- | --- |
| R.AGLQFPVGR.I | Y | 52.83 | 943.5239 | 9 | 0.5 | 472.7695 | 2 | 45.81 | 2 | F2:14894 | 09012020\_NaNaRj\_F2.raw |  | 6.76E4 |  |  |  |  |  |  |  |  | 1 | 0 | 1 | 0 | 0 | 0 | 0 | 0 | 0 | 0 | 0 | 36 | 44 |  |
| total 1 peptides |  |  |  |  |  |  |  |  |  |  |  |  |  |  |  |  |  |  |  |  |  |  |  |  |  |  |  |  |  |  |  |  |  |  |  |
| --- | --- | --- | --- | --- | --- | --- | --- | --- | --- | --- | --- | --- | --- | --- | --- | --- | --- | --- | --- | --- | --- | --- | --- | --- | --- | --- | --- | --- | --- | --- | --- | --- | --- | --- | --- |

Q8H7Y8|H2AV1\_ORYSJ

back to list

  

| Protein Coverage
| Supporting Peptides
|

Protein Coverage:

Supporting Peptides:

| Peptide | Uniq | -10lgP | Mass | Length | ppm | m/z | z | RT | Fraction | Scan | Source File | Area F1 | Area F2 | Area F3 | Area F4 | Area F5 | Area F6 | Area F7 | Area F8 | Area F9 | Area F10 | #Feature | #Feature F1 | #Feature F2 | #Feature F3 | #Feature F4 | #Feature F5 | #Feature F6 | #Feature F7 | #Feature F8 | #Feature F9 | #Feature F10 | Start | End | PTM |
| --- | --- | --- | --- | --- | --- | --- | --- | --- | --- | --- | --- | --- | --- | --- | --- | --- | --- | --- | --- | --- | --- | --- | --- | --- | --- | --- | --- | --- | --- | --- | --- | --- | --- | --- | --- |
| R.AGLQFPVGR.I | Y | 52.83 | 943.5239 | 9 | 0.5 | 472.7695 | 2 | 45.81 | 2 | F2:14894 | 09012020\_NaNaRj\_F2.raw |  | 6.76E4 |  |  |  |  |  |  |  |  | 1 | 0 | 1 | 0 | 0 | 0 | 0 | 0 | 0 | 0 | 0 | 37 | 45 |  |
| total 1 peptides |  |  |  |  |  |  |  |  |  |  |  |  |  |  |  |  |  |  |  |  |  |  |  |  |  |  |  |  |  |  |  |  |  |  |  |
| --- | --- | --- | --- | --- | --- | --- | --- | --- | --- | --- | --- | --- | --- | --- | --- | --- | --- | --- | --- | --- | --- | --- | --- | --- | --- | --- | --- | --- | --- | --- | --- | --- | --- | --- | --- |

O65759|H2AX\_CICAR

back to list

  

| Protein Coverage
| Supporting Peptides
|

Protein Coverage:

Supporting Peptides:

| Peptide | Uniq | -10lgP | Mass | Length | ppm | m/z | z | RT | Fraction | Scan | Source File | Area F1 | Area F2 | Area F3 | Area F4 | Area F5 | Area F6 | Area F7 | Area F8 | Area F9 | Area F10 | #Feature | #Feature F1 | #Feature F2 | #Feature F3 | #Feature F4 | #Feature F5 | #Feature F6 | #Feature F7 | #Feature F8 | #Feature F9 | #Feature F10 | Start | End | PTM |
| --- | --- | --- | --- | --- | --- | --- | --- | --- | --- | --- | --- | --- | --- | --- | --- | --- | --- | --- | --- | --- | --- | --- | --- | --- | --- | --- | --- | --- | --- | --- | --- | --- | --- | --- | --- |
| K.AGLQFPVGR.I | Y | 52.83 | 943.5239 | 9 | 0.5 | 472.7695 | 2 | 45.81 | 2 | F2:14894 | 09012020\_NaNaRj\_F2.raw |  | 6.76E4 |  |  |  |  |  |  |  |  | 1 | 0 | 1 | 0 | 0 | 0 | 0 | 0 | 0 | 0 | 0 | 26 | 34 |  |
| total 1 peptides |  |  |  |  |  |  |  |  |  |  |  |  |  |  |  |  |  |  |  |  |  |  |  |  |  |  |  |  |  |  |  |  |  |  |  |
| --- | --- | --- | --- | --- | --- | --- | --- | --- | --- | --- | --- | --- | --- | --- | --- | --- | --- | --- | --- | --- | --- | --- | --- | --- | --- | --- | --- | --- | --- | --- | --- | --- | --- | --- | --- |

Q9S9K7|H2AXB\_ARATH

back to list

  

| Protein Coverage
| Supporting Peptides
|

Protein Coverage:

Supporting Peptides:

| Peptide | Uniq | -10lgP | Mass | Length | ppm | m/z | z | RT | Fraction | Scan | Source File | Area F1 | Area F2 | Area F3 | Area F4 | Area F5 | Area F6 | Area F7 | Area F8 | Area F9 | Area F10 | #Feature | #Feature F1 | #Feature F2 | #Feature F3 | #Feature F4 | #Feature F5 | #Feature F6 | #Feature F7 | #Feature F8 | #Feature F9 | #Feature F10 | Start | End | PTM |
| --- | --- | --- | --- | --- | --- | --- | --- | --- | --- | --- | --- | --- | --- | --- | --- | --- | --- | --- | --- | --- | --- | --- | --- | --- | --- | --- | --- | --- | --- | --- | --- | --- | --- | --- | --- |
| K.AGLQFPVGR.I | Y | 52.83 | 943.5239 | 9 | 0.5 | 472.7695 | 2 | 45.81 | 2 | F2:14894 | 09012020\_NaNaRj\_F2.raw |  | 6.76E4 |  |  |  |  |  |  |  |  | 1 | 0 | 1 | 0 | 0 | 0 | 0 | 0 | 0 | 0 | 0 | 29 | 37 |  |
| total 1 peptides |  |  |  |  |  |  |  |  |  |  |  |  |  |  |  |  |  |  |  |  |  |  |  |  |  |  |  |  |  |  |  |  |  |  |  |
| --- | --- | --- | --- | --- | --- | --- | --- | --- | --- | --- | --- | --- | --- | --- | --- | --- | --- | --- | --- | --- | --- | --- | --- | --- | --- | --- | --- | --- | --- | --- | --- | --- | --- | --- | --- |

P16104|H2AX\_HUMAN

back to list

  

| Protein Coverage
| Supporting Peptides
|

Protein Coverage:

Supporting Peptides:

| Peptide | Uniq | -10lgP | Mass | Length | ppm | m/z | z | RT | Fraction | Scan | Source File | Area F1 | Area F2 | Area F3 | Area F4 | Area F5 | Area F6 | Area F7 | Area F8 | Area F9 | Area F10 | #Feature | #Feature F1 | #Feature F2 | #Feature F3 | #Feature F4 | #Feature F5 | #Feature F6 | #Feature F7 | #Feature F8 | #Feature F9 | #Feature F10 | Start | End | PTM |
| --- | --- | --- | --- | --- | --- | --- | --- | --- | --- | --- | --- | --- | --- | --- | --- | --- | --- | --- | --- | --- | --- | --- | --- | --- | --- | --- | --- | --- | --- | --- | --- | --- | --- | --- | --- |
| R.AGLQFPVGR.V | Y | 52.83 | 943.5239 | 9 | 0.5 | 472.7695 | 2 | 45.81 | 2 | F2:14894 | 09012020\_NaNaRj\_F2.raw |  | 6.76E4 |  |  |  |  |  |  |  |  | 1 | 0 | 1 | 0 | 0 | 0 | 0 | 0 | 0 | 0 | 0 | 22 | 30 |  |
| total 1 peptides |  |  |  |  |  |  |  |  |  |  |  |  |  |  |  |  |  |  |  |  |  |  |  |  |  |  |  |  |  |  |  |  |  |  |  |
| --- | --- | --- | --- | --- | --- | --- | --- | --- | --- | --- | --- | --- | --- | --- | --- | --- | --- | --- | --- | --- | --- | --- | --- | --- | --- | --- | --- | --- | --- | --- | --- | --- | --- | --- | --- |

Q9FJE8|H2A7\_ARATH

back to list

  

| Protein Coverage
| Supporting Peptides
|

Protein Coverage:

Supporting Peptides:

| Peptide | Uniq | -10lgP | Mass | Length | ppm | m/z | z | RT | Fraction | Scan | Source File | Area F1 | Area F2 | Area F3 | Area F4 | Area F5 | Area F6 | Area F7 | Area F8 | Area F9 | Area F10 | #Feature | #Feature F1 | #Feature F2 | #Feature F3 | #Feature F4 | #Feature F5 | #Feature F6 | #Feature F7 | #Feature F8 | #Feature F9 | #Feature F10 | Start | End | PTM |
| --- | --- | --- | --- | --- | --- | --- | --- | --- | --- | --- | --- | --- | --- | --- | --- | --- | --- | --- | --- | --- | --- | --- | --- | --- | --- | --- | --- | --- | --- | --- | --- | --- | --- | --- | --- |
| K.AGLQFPVGR.I | Y | 52.83 | 943.5239 | 9 | 0.5 | 472.7695 | 2 | 45.81 | 2 | F2:14894 | 09012020\_NaNaRj\_F2.raw |  | 6.76E4 |  |  |  |  |  |  |  |  | 1 | 0 | 1 | 0 | 0 | 0 | 0 | 0 | 0 | 0 | 0 | 31 | 39 |  |
| total 1 peptides |  |  |  |  |  |  |  |  |  |  |  |  |  |  |  |  |  |  |  |  |  |  |  |  |  |  |  |  |  |  |  |  |  |  |  |
| --- | --- | --- | --- | --- | --- | --- | --- | --- | --- | --- | --- | --- | --- | --- | --- | --- | --- | --- | --- | --- | --- | --- | --- | --- | --- | --- | --- | --- | --- | --- | --- | --- | --- | --- | --- |

P02277|H2A3\_WHEAT

back to list

  

| Protein Coverage
| Supporting Peptides
|

Protein Coverage:

Supporting Peptides:

| Peptide | Uniq | -10lgP | Mass | Length | ppm | m/z | z | RT | Fraction | Scan | Source File | Area F1 | Area F2 | Area F3 | Area F4 | Area F5 | Area F6 | Area F7 | Area F8 | Area F9 | Area F10 | #Feature | #Feature F1 | #Feature F2 | #Feature F3 | #Feature F4 | #Feature F5 | #Feature F6 | #Feature F7 | #Feature F8 | #Feature F9 | #Feature F10 | Start | End | PTM |
| --- | --- | --- | --- | --- | --- | --- | --- | --- | --- | --- | --- | --- | --- | --- | --- | --- | --- | --- | --- | --- | --- | --- | --- | --- | --- | --- | --- | --- | --- | --- | --- | --- | --- | --- | --- |
| K.AGLQFPVGR.I | Y | 52.83 | 943.5239 | 9 | 0.5 | 472.7695 | 2 | 45.81 | 2 | F2:14894 | 09012020\_NaNaRj\_F2.raw |  | 6.76E4 |  |  |  |  |  |  |  |  | 1 | 0 | 1 | 0 | 0 | 0 | 0 | 0 | 0 | 0 | 0 | 28 | 36 |  |
| total 1 peptides |  |  |  |  |  |  |  |  |  |  |  |  |  |  |  |  |  |  |  |  |  |  |  |  |  |  |  |  |  |  |  |  |  |  |  |
| --- | --- | --- | --- | --- | --- | --- | --- | --- | --- | --- | --- | --- | --- | --- | --- | --- | --- | --- | --- | --- | --- | --- | --- | --- | --- | --- | --- | --- | --- | --- | --- | --- | --- | --- | --- |

P58370|3NO48\_MICCO

back to list

  

| Protein Coverage
| Supporting Peptides
|

Protein Coverage:

Supporting Peptides:

| Peptide | Uniq | -10lgP | Mass | Length | ppm | m/z | z | RT | Fraction | Scan | Source File | Area F1 | Area F2 | Area F3 | Area F4 | Area F5 | Area F6 | Area F7 | Area F8 | Area F9 | Area F10 | #Feature | #Feature F1 | #Feature F2 | #Feature F3 | #Feature F4 | #Feature F5 | #Feature F6 | #Feature F7 | #Feature F8 | #Feature F9 | #Feature F10 | Start | End | PTM |
| --- | --- | --- | --- | --- | --- | --- | --- | --- | --- | --- | --- | --- | --- | --- | --- | --- | --- | --- | --- | --- | --- | --- | --- | --- | --- | --- | --- | --- | --- | --- | --- | --- | --- | --- | --- |
| R.IDRGC(+57.02)AATC(+57.02)PTVK.P | Y | 51.22 | 1447.6912 | 13 | -7.3 | 724.8476 | 2 | 11.61 | 7 | F7:2432 | 05112019\_RID\_1313\_NaNaRj\_F7.raw |  |  |  | 0 |  |  | 1.6E5 |  |  |  | 2 | 0 | 0 | 0 | 1 | 0 | 0 | 1 | 0 | 0 | 0 | 60 | 72 | Carbamidomethylation |
| total 1 peptides |  |  |  |  |  |  |  |  |  |  |  |  |  |  |  |  |  |  |  |  |  |  |  |  |  |  |  |  |  |  |  |  |  |  |  |
| --- | --- | --- | --- | --- | --- | --- | --- | --- | --- | --- | --- | --- | --- | --- | --- | --- | --- | --- | --- | --- | --- | --- | --- | --- | --- | --- | --- | --- | --- | --- | --- | --- | --- | --- | --- |

P05109|S10A8\_HUMAN

back to list

  

| Protein Coverage
| Supporting Peptides
|

Protein Coverage:

Supporting Peptides:

| Peptide | Uniq | -10lgP | Mass | Length | ppm | m/z | z | RT | Fraction | Scan | Source File | Area F1 | Area F2 | Area F3 | Area F4 | Area F5 | Area F6 | Area F7 | Area F8 | Area F9 | Area F10 | #Feature | #Feature F1 | #Feature F2 | #Feature F3 | #Feature F4 | #Feature F5 | #Feature F6 | #Feature F7 | #Feature F8 | #Feature F9 | #Feature F10 | Start | End | PTM |
| --- | --- | --- | --- | --- | --- | --- | --- | --- | --- | --- | --- | --- | --- | --- | --- | --- | --- | --- | --- | --- | --- | --- | --- | --- | --- | --- | --- | --- | --- | --- | --- | --- | --- | --- | --- |
| K.LLETEC(+57.02)PQYIR.K | Y | 50.11 | 1420.7020 | 11 | -0.4 | 711.3580 | 2 | 11.85 | 3 | F3:2594 | 05112019\_RID\_1313\_NaNaRj\_F3.raw |  |  | 1.47E4 |  |  |  |  |  |  |  | 1 | 0 | 0 | 1 | 0 | 0 | 0 | 0 | 0 | 0 | 0 | 37 | 47 | Carbamidomethylation |
| total 1 peptides |  |  |  |  |  |  |  |  |  |  |  |  |  |  |  |  |  |  |  |  |  |  |  |  |  |  |  |  |  |  |  |  |  |  |  |
| --- | --- | --- | --- | --- | --- | --- | --- | --- | --- | --- | --- | --- | --- | --- | --- | --- | --- | --- | --- | --- | --- | --- | --- | --- | --- | --- | --- | --- | --- | --- | --- | --- | --- | --- | --- |

Q802B2|3NO2A\_NAJSP

back to list

  

| Protein Coverage
| Supporting Peptides
|

Protein Coverage:

Supporting Peptides:

| Peptide | Uniq | -10lgP | Mass | Length | ppm | m/z | z | RT | Fraction | Scan | Source File | Area F1 | Area F2 | Area F3 | Area F4 | Area F5 | Area F6 | Area F7 | Area F8 | Area F9 | Area F10 | #Feature | #Feature F1 | #Feature F2 | #Feature F3 | #Feature F4 | #Feature F5 | #Feature F6 | #Feature F7 | #Feature F8 | #Feature F9 | #Feature F10 | Start | End | PTM |
| --- | --- | --- | --- | --- | --- | --- | --- | --- | --- | --- | --- | --- | --- | --- | --- | --- | --- | --- | --- | --- | --- | --- | --- | --- | --- | --- | --- | --- | --- | --- | --- | --- | --- | --- | --- |
| R.EIVQC(+57.02)C(+57.02)STDRC(+57.02)NR | Y | 49.58 | 1696.7080 | 13 | -6.1 | 849.3561 | 2 | 10.97 | 4 | F4:1863 | 05112019\_RID\_1313\_NaNaRj\_F4.raw |  |  |  | 3.65E4 |  |  |  |  |  |  | 1 | 0 | 0 | 0 | 1 | 0 | 0 | 0 | 0 | 0 | 0 | 74 | 86 | Carbamidomethylation |
| total 1 peptides |  |  |  |  |  |  |  |  |  |  |  |  |  |  |  |  |  |  |  |  |  |  |  |  |  |  |  |  |  |  |  |  |  |  |  |
| --- | --- | --- | --- | --- | --- | --- | --- | --- | --- | --- | --- | --- | --- | --- | --- | --- | --- | --- | --- | --- | --- | --- | --- | --- | --- | --- | --- | --- | --- | --- | --- | --- | --- | --- | --- |

O97370|EURM3\_EURMA

back to list

  

| Protein Coverage
| Supporting Peptides
|

Protein Coverage:

Supporting Peptides:

| Peptide | Uniq | -10lgP | Mass | Length | ppm | m/z | z | RT | Fraction | Scan | Source File | Area F1 | Area F2 | Area F3 | Area F4 | Area F5 | Area F6 | Area F7 | Area F8 | Area F9 | Area F10 | #Feature | #Feature F1 | #Feature F2 | #Feature F3 | #Feature F4 | #Feature F5 | #Feature F6 | #Feature F7 | #Feature F8 | #Feature F9 | #Feature F10 | Start | End | PTM |
| --- | --- | --- | --- | --- | --- | --- | --- | --- | --- | --- | --- | --- | --- | --- | --- | --- | --- | --- | --- | --- | --- | --- | --- | --- | --- | --- | --- | --- | --- | --- | --- | --- | --- | --- | --- |
| V.GIVSWGYGC(+57.02)ARK.G | Y | 48.02 | 1352.6659 | 12 | -8.1 | 677.3348 | 2 | 12.17 | 9 | F9:2768 | 05112019\_RID\_1313\_NaNaRj\_F9.raw |  |  |  |  |  |  |  |  | 3.97E4 |  | 1 | 0 | 0 | 0 | 0 | 0 | 0 | 0 | 0 | 1 | 0 | 228 | 239 | Carbamidomethylation |
| total 1 peptides |  |  |  |  |  |  |  |  |  |  |  |  |  |  |  |  |  |  |  |  |  |  |  |  |  |  |  |  |  |  |  |  |  |  |  |
| --- | --- | --- | --- | --- | --- | --- | --- | --- | --- | --- | --- | --- | --- | --- | --- | --- | --- | --- | --- | --- | --- | --- | --- | --- | --- | --- | --- | --- | --- | --- | --- | --- | --- | --- | --- |

P39675|DERP3\_DERPT

back to list

  

| Protein Coverage
| Supporting Peptides
|

Protein Coverage:

Supporting Peptides:

| Peptide | Uniq | -10lgP | Mass | Length | ppm | m/z | z | RT | Fraction | Scan | Source File | Area F1 | Area F2 | Area F3 | Area F4 | Area F5 | Area F6 | Area F7 | Area F8 | Area F9 | Area F10 | #Feature | #Feature F1 | #Feature F2 | #Feature F3 | #Feature F4 | #Feature F5 | #Feature F6 | #Feature F7 | #Feature F8 | #Feature F9 | #Feature F10 | Start | End | PTM |
| --- | --- | --- | --- | --- | --- | --- | --- | --- | --- | --- | --- | --- | --- | --- | --- | --- | --- | --- | --- | --- | --- | --- | --- | --- | --- | --- | --- | --- | --- | --- | --- | --- | --- | --- | --- |
| V.GIVSWGYGC(+57.02)ARK.G | Y | 48.02 | 1352.6659 | 12 | -8.1 | 677.3348 | 2 | 12.17 | 9 | F9:2768 | 05112019\_RID\_1313\_NaNaRj\_F9.raw |  |  |  |  |  |  |  |  | 3.97E4 |  | 1 | 0 | 0 | 0 | 0 | 0 | 0 | 0 | 0 | 1 | 0 | 228 | 239 | Carbamidomethylation |
| total 1 peptides |  |  |  |  |  |  |  |  |  |  |  |  |  |  |  |  |  |  |  |  |  |  |  |  |  |  |  |  |  |  |  |  |  |  |  |
| --- | --- | --- | --- | --- | --- | --- | --- | --- | --- | --- | --- | --- | --- | --- | --- | --- | --- | --- | --- | --- | --- | --- | --- | --- | --- | --- | --- | --- | --- | --- | --- | --- | --- | --- | --- |

P49275|DERF3\_DERFA

back to list

  

| Protein Coverage
| Supporting Peptides
|

Protein Coverage:

Supporting Peptides:

| Peptide | Uniq | -10lgP | Mass | Length | ppm | m/z | z | RT | Fraction | Scan | Source File | Area F1 | Area F2 | Area F3 | Area F4 | Area F5 | Area F6 | Area F7 | Area F8 | Area F9 | Area F10 | #Feature | #Feature F1 | #Feature F2 | #Feature F3 | #Feature F4 | #Feature F5 | #Feature F6 | #Feature F7 | #Feature F8 | #Feature F9 | #Feature F10 | Start | End | PTM |
| --- | --- | --- | --- | --- | --- | --- | --- | --- | --- | --- | --- | --- | --- | --- | --- | --- | --- | --- | --- | --- | --- | --- | --- | --- | --- | --- | --- | --- | --- | --- | --- | --- | --- | --- | --- |
| V.GIVSWGYGC(+57.02)ARK.G | Y | 48.02 | 1352.6659 | 12 | -8.1 | 677.3348 | 2 | 12.17 | 9 | F9:2768 | 05112019\_RID\_1313\_NaNaRj\_F9.raw |  |  |  |  |  |  |  |  | 3.97E4 |  | 1 | 0 | 0 | 0 | 0 | 0 | 0 | 0 | 0 | 1 | 0 | 226 | 237 | Carbamidomethylation |
| total 1 peptides |  |  |  |  |  |  |  |  |  |  |  |  |  |  |  |  |  |  |  |  |  |  |  |  |  |  |  |  |  |  |  |  |  |  |  |
| --- | --- | --- | --- | --- | --- | --- | --- | --- | --- | --- | --- | --- | --- | --- | --- | --- | --- | --- | --- | --- | --- | --- | --- | --- | --- | --- | --- | --- | --- | --- | --- | --- | --- | --- | --- |

Q1LPI5|ENO1\_CUPMC

back to list

  

| Protein Coverage
| Supporting Peptides
|

Protein Coverage:

Supporting Peptides:

| Peptide | Uniq | -10lgP | Mass | Length | ppm | m/z | z | RT | Fraction | Scan | Source File | Area F1 | Area F2 | Area F3 | Area F4 | Area F5 | Area F6 | Area F7 | Area F8 | Area F9 | Area F10 | #Feature | #Feature F1 | #Feature F2 | #Feature F3 | #Feature F4 | #Feature F5 | #Feature F6 | #Feature F7 | #Feature F8 | #Feature F9 | #Feature F10 | Start | End | PTM |
| --- | --- | --- | --- | --- | --- | --- | --- | --- | --- | --- | --- | --- | --- | --- | --- | --- | --- | --- | --- | --- | --- | --- | --- | --- | --- | --- | --- | --- | --- | --- | --- | --- | --- | --- | --- |
| R.VQLVGDDLFVTNTK.I | Y | 47.18 | 1547.8195 | 14 | 0.5 | 774.9174 | 2 | 62.56 | 2 | F2:20681 | 09012020\_NaNaRj\_F2.raw |  | 2E4 |  |  |  |  |  |  |  |  | 1 | 0 | 1 | 0 | 0 | 0 | 0 | 0 | 0 | 0 | 0 | 309 | 322 |  |
| total 1 peptides |  |  |  |  |  |  |  |  |  |  |  |  |  |  |  |  |  |  |  |  |  |  |  |  |  |  |  |  |  |  |  |  |  |  |  |
| --- | --- | --- | --- | --- | --- | --- | --- | --- | --- | --- | --- | --- | --- | --- | --- | --- | --- | --- | --- | --- | --- | --- | --- | --- | --- | --- | --- | --- | --- | --- | --- | --- | --- | --- | --- |

Q47WR1|ENO\_COLP3

back to list

  

| Protein Coverage
| Supporting Peptides
|

Protein Coverage:

Supporting Peptides:

| Peptide | Uniq | -10lgP | Mass | Length | ppm | m/z | z | RT | Fraction | Scan | Source File | Area F1 | Area F2 | Area F3 | Area F4 | Area F5 | Area F6 | Area F7 | Area F8 | Area F9 | Area F10 | #Feature | #Feature F1 | #Feature F2 | #Feature F3 | #Feature F4 | #Feature F5 | #Feature F6 | #Feature F7 | #Feature F8 | #Feature F9 | #Feature F10 | Start | End | PTM |
| --- | --- | --- | --- | --- | --- | --- | --- | --- | --- | --- | --- | --- | --- | --- | --- | --- | --- | --- | --- | --- | --- | --- | --- | --- | --- | --- | --- | --- | --- | --- | --- | --- | --- | --- | --- |
| K.VQIVGDDLFVTNTK.I | Y | 47.18 | 1547.8195 | 14 | 0.5 | 774.9174 | 2 | 62.56 | 2 | F2:20681 | 09012020\_NaNaRj\_F2.raw |  | 2E4 |  |  |  |  |  |  |  |  | 1 | 0 | 1 | 0 | 0 | 0 | 0 | 0 | 0 | 0 | 0 | 313 | 326 |  |
| total 1 peptides |  |  |  |  |  |  |  |  |  |  |  |  |  |  |  |  |  |  |  |  |  |  |  |  |  |  |  |  |  |  |  |  |  |  |  |
| --- | --- | --- | --- | --- | --- | --- | --- | --- | --- | --- | --- | --- | --- | --- | --- | --- | --- | --- | --- | --- | --- | --- | --- | --- | --- | --- | --- | --- | --- | --- | --- | --- | --- | --- | --- |

P52552|PRDX2\_PIG

back to list

  

| Protein Coverage
| Supporting Peptides
|

Protein Coverage:

Supporting Peptides:

| Peptide | Uniq | -10lgP | Mass | Length | ppm | m/z | z | RT | Fraction | Scan | Source File | Area F1 | Area F2 | Area F3 | Area F4 | Area F5 | Area F6 | Area F7 | Area F8 | Area F9 | Area F10 | #Feature | #Feature F1 | #Feature F2 | #Feature F3 | #Feature F4 | #Feature F5 | #Feature F6 | #Feature F7 | #Feature F8 | #Feature F9 | #Feature F10 | Start | End | PTM |
| --- | --- | --- | --- | --- | --- | --- | --- | --- | --- | --- | --- | --- | --- | --- | --- | --- | --- | --- | --- | --- | --- | --- | --- | --- | --- | --- | --- | --- | --- | --- | --- | --- | --- | --- | --- |
| R.QITVNDLPVGR.X | Y | 43.71 | 1210.6670 | 11 | 0.9 | 606.3413 | 2 | 12.25 | 5 | F5:3060 | 05112019\_RID\_1313\_NaNaRj\_F5.raw |  |  | 6.21E3 |  | 7.88E3 |  |  |  |  |  | 2 | 0 | 0 | 1 | 0 | 1 | 0 | 0 | 0 | 0 | 0 | 101 | 111 |  |
| total 1 peptides |  |  |  |  |  |  |  |  |  |  |  |  |  |  |  |  |  |  |  |  |  |  |  |  |  |  |  |  |  |  |  |  |  |  |  |
| --- | --- | --- | --- | --- | --- | --- | --- | --- | --- | --- | --- | --- | --- | --- | --- | --- | --- | --- | --- | --- | --- | --- | --- | --- | --- | --- | --- | --- | --- | --- | --- | --- | --- | --- | --- |

Q8K3U7|PRDX2\_CRIGR

back to list

  

| Protein Coverage
| Supporting Peptides
|

Protein Coverage:

Supporting Peptides:

| Peptide | Uniq | -10lgP | Mass | Length | ppm | m/z | z | RT | Fraction | Scan | Source File | Area F1 | Area F2 | Area F3 | Area F4 | Area F5 | Area F6 | Area F7 | Area F8 | Area F9 | Area F10 | #Feature | #Feature F1 | #Feature F2 | #Feature F3 | #Feature F4 | #Feature F5 | #Feature F6 | #Feature F7 | #Feature F8 | #Feature F9 | #Feature F10 | Start | End | PTM |
| --- | --- | --- | --- | --- | --- | --- | --- | --- | --- | --- | --- | --- | --- | --- | --- | --- | --- | --- | --- | --- | --- | --- | --- | --- | --- | --- | --- | --- | --- | --- | --- | --- | --- | --- | --- |
| R.QITVNDLPVGR.S | Y | 43.71 | 1210.6670 | 11 | 0.9 | 606.3413 | 2 | 12.25 | 5 | F5:3060 | 05112019\_RID\_1313\_NaNaRj\_F5.raw |  |  | 6.21E3 |  | 7.88E3 |  |  |  |  |  | 2 | 0 | 0 | 1 | 0 | 1 | 0 | 0 | 0 | 0 | 0 | 140 | 150 |  |
| total 1 peptides |  |  |  |  |  |  |  |  |  |  |  |  |  |  |  |  |  |  |  |  |  |  |  |  |  |  |  |  |  |  |  |  |  |  |  |
| --- | --- | --- | --- | --- | --- | --- | --- | --- | --- | --- | --- | --- | --- | --- | --- | --- | --- | --- | --- | --- | --- | --- | --- | --- | --- | --- | --- | --- | --- | --- | --- | --- | --- | --- | --- |

Q06830|PRDX1\_HUMAN

back to list

  

| Protein Coverage
| Supporting Peptides
|

Protein Coverage:

Supporting Peptides:

| Peptide | Uniq | -10lgP | Mass | Length | ppm | m/z | z | RT | Fraction | Scan | Source File | Area F1 | Area F2 | Area F3 | Area F4 | Area F5 | Area F6 | Area F7 | Area F8 | Area F9 | Area F10 | #Feature | #Feature F1 | #Feature F2 | #Feature F3 | #Feature F4 | #Feature F5 | #Feature F6 | #Feature F7 | #Feature F8 | #Feature F9 | #Feature F10 | Start | End | PTM |
| --- | --- | --- | --- | --- | --- | --- | --- | --- | --- | --- | --- | --- | --- | --- | --- | --- | --- | --- | --- | --- | --- | --- | --- | --- | --- | --- | --- | --- | --- | --- | --- | --- | --- | --- | --- |
| R.QITVNDLPVGR.S | Y | 43.71 | 1210.6670 | 11 | 0.9 | 606.3413 | 2 | 12.25 | 5 | F5:3060 | 05112019\_RID\_1313\_NaNaRj\_F5.raw |  |  | 6.21E3 |  | 7.88E3 |  |  |  |  |  | 2 | 0 | 0 | 1 | 0 | 1 | 0 | 0 | 0 | 0 | 0 | 141 | 151 |  |
| total 1 peptides |  |  |  |  |  |  |  |  |  |  |  |  |  |  |  |  |  |  |  |  |  |  |  |  |  |  |  |  |  |  |  |  |  |  |  |
| --- | --- | --- | --- | --- | --- | --- | --- | --- | --- | --- | --- | --- | --- | --- | --- | --- | --- | --- | --- | --- | --- | --- | --- | --- | --- | --- | --- | --- | --- | --- | --- | --- | --- | --- | --- |

#CONTAM#PRDX1\_HUMAN|

back to list

  

| Protein Coverage
| Supporting Peptides
|

Protein Coverage:

Supporting Peptides:

| Peptide | Uniq | -10lgP | Mass | Length | ppm | m/z | z | RT | Fraction | Scan | Source File | Area F1 | Area F2 | Area F3 | Area F4 | Area F5 | Area F6 | Area F7 | Area F8 | Area F9 | Area F10 | #Feature | #Feature F1 | #Feature F2 | #Feature F3 | #Feature F4 | #Feature F5 | #Feature F6 | #Feature F7 | #Feature F8 | #Feature F9 | #Feature F10 | Start | End | PTM |
| --- | --- | --- | --- | --- | --- | --- | --- | --- | --- | --- | --- | --- | --- | --- | --- | --- | --- | --- | --- | --- | --- | --- | --- | --- | --- | --- | --- | --- | --- | --- | --- | --- | --- | --- | --- |
| R.QITVNDLPVGR.S | Y | 43.71 | 1210.6670 | 11 | 0.9 | 606.3413 | 2 | 12.25 | 5 | F5:3060 | 05112019\_RID\_1313\_NaNaRj\_F5.raw |  |  | 6.21E3 |  | 7.88E3 |  |  |  |  |  | 2 | 0 | 0 | 1 | 0 | 1 | 0 | 0 | 0 | 0 | 0 | 141 | 151 |  |
| total 1 peptides |  |  |  |  |  |  |  |  |  |  |  |  |  |  |  |  |  |  |  |  |  |  |  |  |  |  |  |  |  |  |  |  |  |  |  |
| --- | --- | --- | --- | --- | --- | --- | --- | --- | --- | --- | --- | --- | --- | --- | --- | --- | --- | --- | --- | --- | --- | --- | --- | --- | --- | --- | --- | --- | --- | --- | --- | --- | --- | --- | --- |

Q6B4U9|PRDX1\_MYOLU

back to list

  

| Protein Coverage
| Supporting Peptides
|

Protein Coverage:

Supporting Peptides:

| Peptide | Uniq | -10lgP | Mass | Length | ppm | m/z | z | RT | Fraction | Scan | Source File | Area F1 | Area F2 | Area F3 | Area F4 | Area F5 | Area F6 | Area F7 | Area F8 | Area F9 | Area F10 | #Feature | #Feature F1 | #Feature F2 | #Feature F3 | #Feature F4 | #Feature F5 | #Feature F6 | #Feature F7 | #Feature F8 | #Feature F9 | #Feature F10 | Start | End | PTM |
| --- | --- | --- | --- | --- | --- | --- | --- | --- | --- | --- | --- | --- | --- | --- | --- | --- | --- | --- | --- | --- | --- | --- | --- | --- | --- | --- | --- | --- | --- | --- | --- | --- | --- | --- | --- |
| R.QITVNDLPVGR.S | Y | 43.71 | 1210.6670 | 11 | 0.9 | 606.3413 | 2 | 12.25 | 5 | F5:3060 | 05112019\_RID\_1313\_NaNaRj\_F5.raw |  |  | 6.21E3 |  | 7.88E3 |  |  |  |  |  | 2 | 0 | 0 | 1 | 0 | 1 | 0 | 0 | 0 | 0 | 0 | 141 | 151 |  |
| total 1 peptides |  |  |  |  |  |  |  |  |  |  |  |  |  |  |  |  |  |  |  |  |  |  |  |  |  |  |  |  |  |  |  |  |  |  |  |
| --- | --- | --- | --- | --- | --- | --- | --- | --- | --- | --- | --- | --- | --- | --- | --- | --- | --- | --- | --- | --- | --- | --- | --- | --- | --- | --- | --- | --- | --- | --- | --- | --- | --- | --- | --- |

Q5RC63|PRDX2\_PONAB

back to list

  

| Protein Coverage
| Supporting Peptides
|

Protein Coverage:

Supporting Peptides:

| Peptide | Uniq | -10lgP | Mass | Length | ppm | m/z | z | RT | Fraction | Scan | Source File | Area F1 | Area F2 | Area F3 | Area F4 | Area F5 | Area F6 | Area F7 | Area F8 | Area F9 | Area F10 | #Feature | #Feature F1 | #Feature F2 | #Feature F3 | #Feature F4 | #Feature F5 | #Feature F6 | #Feature F7 | #Feature F8 | #Feature F9 | #Feature F10 | Start | End | PTM |
| --- | --- | --- | --- | --- | --- | --- | --- | --- | --- | --- | --- | --- | --- | --- | --- | --- | --- | --- | --- | --- | --- | --- | --- | --- | --- | --- | --- | --- | --- | --- | --- | --- | --- | --- | --- |
| R.QITVNDLPVGR.S | Y | 43.71 | 1210.6670 | 11 | 0.9 | 606.3413 | 2 | 12.25 | 5 | F5:3060 | 05112019\_RID\_1313\_NaNaRj\_F5.raw |  |  | 6.21E3 |  | 7.88E3 |  |  |  |  |  | 2 | 0 | 0 | 1 | 0 | 1 | 0 | 0 | 0 | 0 | 0 | 140 | 150 |  |
| total 1 peptides |  |  |  |  |  |  |  |  |  |  |  |  |  |  |  |  |  |  |  |  |  |  |  |  |  |  |  |  |  |  |  |  |  |  |  |
| --- | --- | --- | --- | --- | --- | --- | --- | --- | --- | --- | --- | --- | --- | --- | --- | --- | --- | --- | --- | --- | --- | --- | --- | --- | --- | --- | --- | --- | --- | --- | --- | --- | --- | --- | --- |

Q9V3P0|PRDX1\_DROME

back to list

  

| Protein Coverage
| Supporting Peptides
|

Protein Coverage:

Supporting Peptides:

| Peptide | Uniq | -10lgP | Mass | Length | ppm | m/z | z | RT | Fraction | Scan | Source File | Area F1 | Area F2 | Area F3 | Area F4 | Area F5 | Area F6 | Area F7 | Area F8 | Area F9 | Area F10 | #Feature | #Feature F1 | #Feature F2 | #Feature F3 | #Feature F4 | #Feature F5 | #Feature F6 | #Feature F7 | #Feature F8 | #Feature F9 | #Feature F10 | Start | End | PTM |
| --- | --- | --- | --- | --- | --- | --- | --- | --- | --- | --- | --- | --- | --- | --- | --- | --- | --- | --- | --- | --- | --- | --- | --- | --- | --- | --- | --- | --- | --- | --- | --- | --- | --- | --- | --- |
| R.QITVNDLPVGR.S | Y | 43.71 | 1210.6670 | 11 | 0.9 | 606.3413 | 2 | 12.25 | 5 | F5:3060 | 05112019\_RID\_1313\_NaNaRj\_F5.raw |  |  | 6.21E3 |  | 7.88E3 |  |  |  |  |  | 2 | 0 | 0 | 1 | 0 | 1 | 0 | 0 | 0 | 0 | 0 | 136 | 146 |  |
| total 1 peptides |  |  |  |  |  |  |  |  |  |  |  |  |  |  |  |  |  |  |  |  |  |  |  |  |  |  |  |  |  |  |  |  |  |  |  |
| --- | --- | --- | --- | --- | --- | --- | --- | --- | --- | --- | --- | --- | --- | --- | --- | --- | --- | --- | --- | --- | --- | --- | --- | --- | --- | --- | --- | --- | --- | --- | --- | --- | --- | --- | --- |

Q9NL98|PRDX\_ASCSU

back to list

  

| Protein Coverage
| Supporting Peptides
|

Protein Coverage:

Supporting Peptides:

| Peptide | Uniq | -10lgP | Mass | Length | ppm | m/z | z | RT | Fraction | Scan | Source File | Area F1 | Area F2 | Area F3 | Area F4 | Area F5 | Area F6 | Area F7 | Area F8 | Area F9 | Area F10 | #Feature | #Feature F1 | #Feature F2 | #Feature F3 | #Feature F4 | #Feature F5 | #Feature F6 | #Feature F7 | #Feature F8 | #Feature F9 | #Feature F10 | Start | End | PTM |
| --- | --- | --- | --- | --- | --- | --- | --- | --- | --- | --- | --- | --- | --- | --- | --- | --- | --- | --- | --- | --- | --- | --- | --- | --- | --- | --- | --- | --- | --- | --- | --- | --- | --- | --- | --- |
| R.QITVNDLPVGR.S | Y | 43.71 | 1210.6670 | 11 | 0.9 | 606.3413 | 2 | 12.25 | 5 | F5:3060 | 05112019\_RID\_1313\_NaNaRj\_F5.raw |  |  | 6.21E3 |  | 7.88E3 |  |  |  |  |  | 2 | 0 | 0 | 1 | 0 | 1 | 0 | 0 | 0 | 0 | 0 | 138 | 148 |  |
| total 1 peptides |  |  |  |  |  |  |  |  |  |  |  |  |  |  |  |  |  |  |  |  |  |  |  |  |  |  |  |  |  |  |  |  |  |  |  |
| --- | --- | --- | --- | --- | --- | --- | --- | --- | --- | --- | --- | --- | --- | --- | --- | --- | --- | --- | --- | --- | --- | --- | --- | --- | --- | --- | --- | --- | --- | --- | --- | --- | --- | --- | --- |

P35704|PRDX2\_RAT

back to list

  

| Protein Coverage
| Supporting Peptides
|

Protein Coverage:

Supporting Peptides:

| Peptide | Uniq | -10lgP | Mass | Length | ppm | m/z | z | RT | Fraction | Scan | Source File | Area F1 | Area F2 | Area F3 | Area F4 | Area F5 | Area F6 | Area F7 | Area F8 | Area F9 | Area F10 | #Feature | #Feature F1 | #Feature F2 | #Feature F3 | #Feature F4 | #Feature F5 | #Feature F6 | #Feature F7 | #Feature F8 | #Feature F9 | #Feature F10 | Start | End | PTM |
| --- | --- | --- | --- | --- | --- | --- | --- | --- | --- | --- | --- | --- | --- | --- | --- | --- | --- | --- | --- | --- | --- | --- | --- | --- | --- | --- | --- | --- | --- | --- | --- | --- | --- | --- | --- |
| R.QITVNDLPVGR.S | Y | 43.71 | 1210.6670 | 11 | 0.9 | 606.3413 | 2 | 12.25 | 5 | F5:3060 | 05112019\_RID\_1313\_NaNaRj\_F5.raw |  |  | 6.21E3 |  | 7.88E3 |  |  |  |  |  | 2 | 0 | 0 | 1 | 0 | 1 | 0 | 0 | 0 | 0 | 0 | 140 | 150 |  |
| total 1 peptides |  |  |  |  |  |  |  |  |  |  |  |  |  |  |  |  |  |  |  |  |  |  |  |  |  |  |  |  |  |  |  |  |  |  |  |
| --- | --- | --- | --- | --- | --- | --- | --- | --- | --- | --- | --- | --- | --- | --- | --- | --- | --- | --- | --- | --- | --- | --- | --- | --- | --- | --- | --- | --- | --- | --- | --- | --- | --- | --- | --- |

Q61171|PRDX2\_MOUSE

back to list

  

| Protein Coverage
| Supporting Peptides
|

Protein Coverage:

Supporting Peptides:

| Peptide | Uniq | -10lgP | Mass | Length | ppm | m/z | z | RT | Fraction | Scan | Source File | Area F1 | Area F2 | Area F3 | Area F4 | Area F5 | Area F6 | Area F7 | Area F8 | Area F9 | Area F10 | #Feature | #Feature F1 | #Feature F2 | #Feature F3 | #Feature F4 | #Feature F5 | #Feature F6 | #Feature F7 | #Feature F8 | #Feature F9 | #Feature F10 | Start | End | PTM |
| --- | --- | --- | --- | --- | --- | --- | --- | --- | --- | --- | --- | --- | --- | --- | --- | --- | --- | --- | --- | --- | --- | --- | --- | --- | --- | --- | --- | --- | --- | --- | --- | --- | --- | --- | --- |
| R.QITVNDLPVGR.S | Y | 43.71 | 1210.6670 | 11 | 0.9 | 606.3413 | 2 | 12.25 | 5 | F5:3060 | 05112019\_RID\_1313\_NaNaRj\_F5.raw |  |  | 6.21E3 |  | 7.88E3 |  |  |  |  |  | 2 | 0 | 0 | 1 | 0 | 1 | 0 | 0 | 0 | 0 | 0 | 140 | 150 |  |
| total 1 peptides |  |  |  |  |  |  |  |  |  |  |  |  |  |  |  |  |  |  |  |  |  |  |  |  |  |  |  |  |  |  |  |  |  |  |  |
| --- | --- | --- | --- | --- | --- | --- | --- | --- | --- | --- | --- | --- | --- | --- | --- | --- | --- | --- | --- | --- | --- | --- | --- | --- | --- | --- | --- | --- | --- | --- | --- | --- | --- | --- | --- |

P32119|PRDX2\_HUMAN

back to list

  

| Protein Coverage
| Supporting Peptides
|

Protein Coverage:

Supporting Peptides:

| Peptide | Uniq | -10lgP | Mass | Length | ppm | m/z | z | RT | Fraction | Scan | Source File | Area F1 | Area F2 | Area F3 | Area F4 | Area F5 | Area F6 | Area F7 | Area F8 | Area F9 | Area F10 | #Feature | #Feature F1 | #Feature F2 | #Feature F3 | #Feature F4 | #Feature F5 | #Feature F6 | #Feature F7 | #Feature F8 | #Feature F9 | #Feature F10 | Start | End | PTM |
| --- | --- | --- | --- | --- | --- | --- | --- | --- | --- | --- | --- | --- | --- | --- | --- | --- | --- | --- | --- | --- | --- | --- | --- | --- | --- | --- | --- | --- | --- | --- | --- | --- | --- | --- | --- |
| R.QITVNDLPVGR.S | Y | 43.71 | 1210.6670 | 11 | 0.9 | 606.3413 | 2 | 12.25 | 5 | F5:3060 | 05112019\_RID\_1313\_NaNaRj\_F5.raw |  |  | 6.21E3 |  | 7.88E3 |  |  |  |  |  | 2 | 0 | 0 | 1 | 0 | 1 | 0 | 0 | 0 | 0 | 0 | 140 | 150 |  |
| total 1 peptides |  |  |  |  |  |  |  |  |  |  |  |  |  |  |  |  |  |  |  |  |  |  |  |  |  |  |  |  |  |  |  |  |  |  |  |
| --- | --- | --- | --- | --- | --- | --- | --- | --- | --- | --- | --- | --- | --- | --- | --- | --- | --- | --- | --- | --- | --- | --- | --- | --- | --- | --- | --- | --- | --- | --- | --- | --- | --- | --- | --- |

Q2PFZ3|PRDX2\_MACFA

back to list

  

| Protein Coverage
| Supporting Peptides
|

Protein Coverage:

Supporting Peptides:

| Peptide | Uniq | -10lgP | Mass | Length | ppm | m/z | z | RT | Fraction | Scan | Source File | Area F1 | Area F2 | Area F3 | Area F4 | Area F5 | Area F6 | Area F7 | Area F8 | Area F9 | Area F10 | #Feature | #Feature F1 | #Feature F2 | #Feature F3 | #Feature F4 | #Feature F5 | #Feature F6 | #Feature F7 | #Feature F8 | #Feature F9 | #Feature F10 | Start | End | PTM |
| --- | --- | --- | --- | --- | --- | --- | --- | --- | --- | --- | --- | --- | --- | --- | --- | --- | --- | --- | --- | --- | --- | --- | --- | --- | --- | --- | --- | --- | --- | --- | --- | --- | --- | --- | --- |
| R.QITVNDLPVGR.S | Y | 43.71 | 1210.6670 | 11 | 0.9 | 606.3413 | 2 | 12.25 | 5 | F5:3060 | 05112019\_RID\_1313\_NaNaRj\_F5.raw |  |  | 6.21E3 |  | 7.88E3 |  |  |  |  |  | 2 | 0 | 0 | 1 | 0 | 1 | 0 | 0 | 0 | 0 | 0 | 140 | 150 |  |
| total 1 peptides |  |  |  |  |  |  |  |  |  |  |  |  |  |  |  |  |  |  |  |  |  |  |  |  |  |  |  |  |  |  |  |  |  |  |  |
| --- | --- | --- | --- | --- | --- | --- | --- | --- | --- | --- | --- | --- | --- | --- | --- | --- | --- | --- | --- | --- | --- | --- | --- | --- | --- | --- | --- | --- | --- | --- | --- | --- | --- | --- | --- |

P31025|LCN1\_HUMAN

back to list

  

| Protein Coverage
| Supporting Peptides
|

Protein Coverage:

Supporting Peptides:

| Peptide | Uniq | -10lgP | Mass | Length | ppm | m/z | z | RT | Fraction | Scan | Source File | Area F1 | Area F2 | Area F3 | Area F4 | Area F5 | Area F6 | Area F7 | Area F8 | Area F9 | Area F10 | #Feature | #Feature F1 | #Feature F2 | #Feature F3 | #Feature F4 | #Feature F5 | #Feature F6 | #Feature F7 | #Feature F8 | #Feature F9 | #Feature F10 | Start | End | PTM |
| --- | --- | --- | --- | --- | --- | --- | --- | --- | --- | --- | --- | --- | --- | --- | --- | --- | --- | --- | --- | --- | --- | --- | --- | --- | --- | --- | --- | --- | --- | --- | --- | --- | --- | --- | --- |
| K.NNLEALEDFEK.A | Y | 42.15 | 1320.6198 | 11 | 0.2 | 661.3173 | 2 | 14.42 | 9 | F9:3345 | 05112019\_RID\_1313\_NaNaRj\_F9.raw |  |  |  |  |  |  |  |  | 1.97E4 |  | 1 | 0 | 0 | 0 | 0 | 0 | 0 | 0 | 0 | 1 | 0 | 140 | 150 |  |
| total 1 peptides |  |  |  |  |  |  |  |  |  |  |  |  |  |  |  |  |  |  |  |  |  |  |  |  |  |  |  |  |  |  |  |  |  |  |  |
| --- | --- | --- | --- | --- | --- | --- | --- | --- | --- | --- | --- | --- | --- | --- | --- | --- | --- | --- | --- | --- | --- | --- | --- | --- | --- | --- | --- | --- | --- | --- | --- | --- | --- | --- | --- |

B4RYQ8|EFTU\_ALTMD

back to list

  

| Protein Coverage
| Supporting Peptides
|

Protein Coverage:

Supporting Peptides:

| Peptide | Uniq | -10lgP | Mass | Length | ppm | m/z | z | RT | Fraction | Scan | Source File | Area F1 | Area F2 | Area F3 | Area F4 | Area F5 | Area F6 | Area F7 | Area F8 | Area F9 | Area F10 | #Feature | #Feature F1 | #Feature F2 | #Feature F3 | #Feature F4 | #Feature F5 | #Feature F6 | #Feature F7 | #Feature F8 | #Feature F9 | #Feature F10 | Start | End | PTM |
| --- | --- | --- | --- | --- | --- | --- | --- | --- | --- | --- | --- | --- | --- | --- | --- | --- | --- | --- | --- | --- | --- | --- | --- | --- | --- | --- | --- | --- | --- | --- | --- | --- | --- | --- | --- |
| E.LIAPIAMEEGLR.F | Y | 41.87 | 1311.7220 | 12 | 0.6 | 656.8687 | 2 | 62.09 | 2 | F2:20495 | 09012020\_NaNaRj\_F2.raw |  | 3.1E4 |  |  |  |  |  |  |  |  | 1 | 0 | 1 | 0 | 0 | 0 | 0 | 0 | 0 | 0 | 0 | 363 | 374 |  |
| total 1 peptides |  |  |  |  |  |  |  |  |  |  |  |  |  |  |  |  |  |  |  |  |  |  |  |  |  |  |  |  |  |  |  |  |  |  |  |
| --- | --- | --- | --- | --- | --- | --- | --- | --- | --- | --- | --- | --- | --- | --- | --- | --- | --- | --- | --- | --- | --- | --- | --- | --- | --- | --- | --- | --- | --- | --- | --- | --- | --- | --- | --- |

Q3J8Q0|EFTU\_NITOC

back to list

  

| Protein Coverage
| Supporting Peptides
|

Protein Coverage:

Supporting Peptides:

| Peptide | Uniq | -10lgP | Mass | Length | ppm | m/z | z | RT | Fraction | Scan | Source File | Area F1 | Area F2 | Area F3 | Area F4 | Area F5 | Area F6 | Area F7 | Area F8 | Area F9 | Area F10 | #Feature | #Feature F1 | #Feature F2 | #Feature F3 | #Feature F4 | #Feature F5 | #Feature F6 | #Feature F7 | #Feature F8 | #Feature F9 | #Feature F10 | Start | End | PTM |
| --- | --- | --- | --- | --- | --- | --- | --- | --- | --- | --- | --- | --- | --- | --- | --- | --- | --- | --- | --- | --- | --- | --- | --- | --- | --- | --- | --- | --- | --- | --- | --- | --- | --- | --- | --- |
| S.LIAPIAMEEGLR.F | Y | 41.87 | 1311.7220 | 12 | 0.6 | 656.8687 | 2 | 62.09 | 2 | F2:20495 | 09012020\_NaNaRj\_F2.raw |  | 3.1E4 |  |  |  |  |  |  |  |  | 1 | 0 | 1 | 0 | 0 | 0 | 0 | 0 | 0 | 0 | 0 | 365 | 376 |  |
| total 1 peptides |  |  |  |  |  |  |  |  |  |  |  |  |  |  |  |  |  |  |  |  |  |  |  |  |  |  |  |  |  |  |  |  |  |  |  |
| --- | --- | --- | --- | --- | --- | --- | --- | --- | --- | --- | --- | --- | --- | --- | --- | --- | --- | --- | --- | --- | --- | --- | --- | --- | --- | --- | --- | --- | --- | --- | --- | --- | --- | --- | --- |

P33167|EFTU\_BURCE

back to list

  

| Protein Coverage
| Supporting Peptides
|

Protein Coverage:

Supporting Peptides:

| Peptide | Uniq | -10lgP | Mass | Length | ppm | m/z | z | RT | Fraction | Scan | Source File | Area F1 | Area F2 | Area F3 | Area F4 | Area F5 | Area F6 | Area F7 | Area F8 | Area F9 | Area F10 | #Feature | #Feature F1 | #Feature F2 | #Feature F3 | #Feature F4 | #Feature F5 | #Feature F6 | #Feature F7 | #Feature F8 | #Feature F9 | #Feature F10 | Start | End | PTM |
| --- | --- | --- | --- | --- | --- | --- | --- | --- | --- | --- | --- | --- | --- | --- | --- | --- | --- | --- | --- | --- | --- | --- | --- | --- | --- | --- | --- | --- | --- | --- | --- | --- | --- | --- | --- |
| K.LIAPIAMEEGLR.F | Y | 41.87 | 1311.7220 | 12 | 0.6 | 656.8687 | 2 | 62.09 | 2 | F2:20495 | 09012020\_NaNaRj\_F2.raw |  | 3.1E4 |  |  |  |  |  |  |  |  | 1 | 0 | 1 | 0 | 0 | 0 | 0 | 0 | 0 | 0 | 0 | 365 | 376 |  |
| total 1 peptides |  |  |  |  |  |  |  |  |  |  |  |  |  |  |  |  |  |  |  |  |  |  |  |  |  |  |  |  |  |  |  |  |  |  |  |
| --- | --- | --- | --- | --- | --- | --- | --- | --- | --- | --- | --- | --- | --- | --- | --- | --- | --- | --- | --- | --- | --- | --- | --- | --- | --- | --- | --- | --- | --- | --- | --- | --- | --- | --- | --- |

Q1H4N9|EFTU2\_METFK

back to list

  

| Protein Coverage
| Supporting Peptides
|

Protein Coverage:

Supporting Peptides:

| Peptide | Uniq | -10lgP | Mass | Length | ppm | m/z | z | RT | Fraction | Scan | Source File | Area F1 | Area F2 | Area F3 | Area F4 | Area F5 | Area F6 | Area F7 | Area F8 | Area F9 | Area F10 | #Feature | #Feature F1 | #Feature F2 | #Feature F3 | #Feature F4 | #Feature F5 | #Feature F6 | #Feature F7 | #Feature F8 | #Feature F9 | #Feature F10 | Start | End | PTM |
| --- | --- | --- | --- | --- | --- | --- | --- | --- | --- | --- | --- | --- | --- | --- | --- | --- | --- | --- | --- | --- | --- | --- | --- | --- | --- | --- | --- | --- | --- | --- | --- | --- | --- | --- | --- |
| S.LIAPIAMEEGLR.F | Y | 41.87 | 1311.7220 | 12 | 0.6 | 656.8687 | 2 | 62.09 | 2 | F2:20495 | 09012020\_NaNaRj\_F2.raw |  | 3.1E4 |  |  |  |  |  |  |  |  | 1 | 0 | 1 | 0 | 0 | 0 | 0 | 0 | 0 | 0 | 0 | 365 | 376 |  |
| total 1 peptides |  |  |  |  |  |  |  |  |  |  |  |  |  |  |  |  |  |  |  |  |  |  |  |  |  |  |  |  |  |  |  |  |  |  |  |
| --- | --- | --- | --- | --- | --- | --- | --- | --- | --- | --- | --- | --- | --- | --- | --- | --- | --- | --- | --- | --- | --- | --- | --- | --- | --- | --- | --- | --- | --- | --- | --- | --- | --- | --- | --- |

A1KRF9|EFTU\_NEIMF

back to list

  

| Protein Coverage
| Supporting Peptides
|

Protein Coverage:

Supporting Peptides:

| Peptide | Uniq | -10lgP | Mass | Length | ppm | m/z | z | RT | Fraction | Scan | Source File | Area F1 | Area F2 | Area F3 | Area F4 | Area F5 | Area F6 | Area F7 | Area F8 | Area F9 | Area F10 | #Feature | #Feature F1 | #Feature F2 | #Feature F3 | #Feature F4 | #Feature F5 | #Feature F6 | #Feature F7 | #Feature F8 | #Feature F9 | #Feature F10 | Start | End | PTM |
| --- | --- | --- | --- | --- | --- | --- | --- | --- | --- | --- | --- | --- | --- | --- | --- | --- | --- | --- | --- | --- | --- | --- | --- | --- | --- | --- | --- | --- | --- | --- | --- | --- | --- | --- | --- |
| E.LIAPIAMEEGLR.F | Y | 41.87 | 1311.7220 | 12 | 0.6 | 656.8687 | 2 | 62.09 | 2 | F2:20495 | 09012020\_NaNaRj\_F2.raw |  | 3.1E4 |  |  |  |  |  |  |  |  | 1 | 0 | 1 | 0 | 0 | 0 | 0 | 0 | 0 | 0 | 0 | 363 | 374 |  |
| total 1 peptides |  |  |  |  |  |  |  |  |  |  |  |  |  |  |  |  |  |  |  |  |  |  |  |  |  |  |  |  |  |  |  |  |  |  |  |
| --- | --- | --- | --- | --- | --- | --- | --- | --- | --- | --- | --- | --- | --- | --- | --- | --- | --- | --- | --- | --- | --- | --- | --- | --- | --- | --- | --- | --- | --- | --- | --- | --- | --- | --- | --- |

Q1H4Q1|EFTU1\_METFK

back to list

  

| Protein Coverage
| Supporting Peptides
|

Protein Coverage:

Supporting Peptides:

| Peptide | Uniq | -10lgP | Mass | Length | ppm | m/z | z | RT | Fraction | Scan | Source File | Area F1 | Area F2 | Area F3 | Area F4 | Area F5 | Area F6 | Area F7 | Area F8 | Area F9 | Area F10 | #Feature | #Feature F1 | #Feature F2 | #Feature F3 | #Feature F4 | #Feature F5 | #Feature F6 | #Feature F7 | #Feature F8 | #Feature F9 | #Feature F10 | Start | End | PTM |
| --- | --- | --- | --- | --- | --- | --- | --- | --- | --- | --- | --- | --- | --- | --- | --- | --- | --- | --- | --- | --- | --- | --- | --- | --- | --- | --- | --- | --- | --- | --- | --- | --- | --- | --- | --- |
| S.LIAPIAMEEGLR.F | Y | 41.87 | 1311.7220 | 12 | 0.6 | 656.8687 | 2 | 62.09 | 2 | F2:20495 | 09012020\_NaNaRj\_F2.raw |  | 3.1E4 |  |  |  |  |  |  |  |  | 1 | 0 | 1 | 0 | 0 | 0 | 0 | 0 | 0 | 0 | 0 | 365 | 376 |  |
| total 1 peptides |  |  |  |  |  |  |  |  |  |  |  |  |  |  |  |  |  |  |  |  |  |  |  |  |  |  |  |  |  |  |  |  |  |  |  |
| --- | --- | --- | --- | --- | --- | --- | --- | --- | --- | --- | --- | --- | --- | --- | --- | --- | --- | --- | --- | --- | --- | --- | --- | --- | --- | --- | --- | --- | --- | --- | --- | --- | --- | --- | --- |

Q11HA6|EFTU\_CHESB

back to list

  

| Protein Coverage
| Supporting Peptides
|

Protein Coverage:

Supporting Peptides:

| Peptide | Uniq | -10lgP | Mass | Length | ppm | m/z | z | RT | Fraction | Scan | Source File | Area F1 | Area F2 | Area F3 | Area F4 | Area F5 | Area F6 | Area F7 | Area F8 | Area F9 | Area F10 | #Feature | #Feature F1 | #Feature F2 | #Feature F3 | #Feature F4 | #Feature F5 | #Feature F6 | #Feature F7 | #Feature F8 | #Feature F9 | #Feature F10 | Start | End | PTM |
| --- | --- | --- | --- | --- | --- | --- | --- | --- | --- | --- | --- | --- | --- | --- | --- | --- | --- | --- | --- | --- | --- | --- | --- | --- | --- | --- | --- | --- | --- | --- | --- | --- | --- | --- | --- |
| K.TTVTGVEMFR.K | Y | 41.45 | 1139.5645 | 10 | 1.0 | 570.7901 | 2 | 45.85 | 2 | F2:14864 | 09012020\_NaNaRj\_F2.raw |  | 1.1E5 |  |  |  |  |  |  |  |  | 1 | 0 | 1 | 0 | 0 | 0 | 0 | 0 | 0 | 0 | 0 | 251 | 260 |  |
| total 1 peptides |  |  |  |  |  |  |  |  |  |  |  |  |  |  |  |  |  |  |  |  |  |  |  |  |  |  |  |  |  |  |  |  |  |  |  |
| --- | --- | --- | --- | --- | --- | --- | --- | --- | --- | --- | --- | --- | --- | --- | --- | --- | --- | --- | --- | --- | --- | --- | --- | --- | --- | --- | --- | --- | --- | --- | --- | --- | --- | --- | --- |

Q5QWA3|EFTU\_IDILO

back to list

  

| Protein Coverage
| Supporting Peptides
|

Protein Coverage:

Supporting Peptides:

| Peptide | Uniq | -10lgP | Mass | Length | ppm | m/z | z | RT | Fraction | Scan | Source File | Area F1 | Area F2 | Area F3 | Area F4 | Area F5 | Area F6 | Area F7 | Area F8 | Area F9 | Area F10 | #Feature | #Feature F1 | #Feature F2 | #Feature F3 | #Feature F4 | #Feature F5 | #Feature F6 | #Feature F7 | #Feature F8 | #Feature F9 | #Feature F10 | Start | End | PTM |
| --- | --- | --- | --- | --- | --- | --- | --- | --- | --- | --- | --- | --- | --- | --- | --- | --- | --- | --- | --- | --- | --- | --- | --- | --- | --- | --- | --- | --- | --- | --- | --- | --- | --- | --- | --- |
| K.TTVTGVEMFR.K | Y | 41.45 | 1139.5645 | 10 | 1.0 | 570.7901 | 2 | 45.85 | 2 | F2:14864 | 09012020\_NaNaRj\_F2.raw |  | 1.1E5 |  |  |  |  |  |  |  |  | 1 | 0 | 1 | 0 | 0 | 0 | 0 | 0 | 0 | 0 | 0 | 254 | 263 |  |
| total 1 peptides |  |  |  |  |  |  |  |  |  |  |  |  |  |  |  |  |  |  |  |  |  |  |  |  |  |  |  |  |  |  |  |  |  |  |  |
| --- | --- | --- | --- | --- | --- | --- | --- | --- | --- | --- | --- | --- | --- | --- | --- | --- | --- | --- | --- | --- | --- | --- | --- | --- | --- | --- | --- | --- | --- | --- | --- | --- | --- | --- | --- |

Q2YM08|EFTU\_BRUA2

back to list

  

| Protein Coverage
| Supporting Peptides
|

Protein Coverage:

Supporting Peptides:

| Peptide | Uniq | -10lgP | Mass | Length | ppm | m/z | z | RT | Fraction | Scan | Source File | Area F1 | Area F2 | Area F3 | Area F4 | Area F5 | Area F6 | Area F7 | Area F8 | Area F9 | Area F10 | #Feature | #Feature F1 | #Feature F2 | #Feature F3 | #Feature F4 | #Feature F5 | #Feature F6 | #Feature F7 | #Feature F8 | #Feature F9 | #Feature F10 | Start | End | PTM |
| --- | --- | --- | --- | --- | --- | --- | --- | --- | --- | --- | --- | --- | --- | --- | --- | --- | --- | --- | --- | --- | --- | --- | --- | --- | --- | --- | --- | --- | --- | --- | --- | --- | --- | --- | --- |
| K.TTVTGVEMFR.K | Y | 41.45 | 1139.5645 | 10 | 1.0 | 570.7901 | 2 | 45.85 | 2 | F2:14864 | 09012020\_NaNaRj\_F2.raw |  | 1.1E5 |  |  |  |  |  |  |  |  | 1 | 0 | 1 | 0 | 0 | 0 | 0 | 0 | 0 | 0 | 0 | 251 | 260 |  |
| total 1 peptides |  |  |  |  |  |  |  |  |  |  |  |  |  |  |  |  |  |  |  |  |  |  |  |  |  |  |  |  |  |  |  |  |  |  |  |
| --- | --- | --- | --- | --- | --- | --- | --- | --- | --- | --- | --- | --- | --- | --- | --- | --- | --- | --- | --- | --- | --- | --- | --- | --- | --- | --- | --- | --- | --- | --- | --- | --- | --- | --- | --- |

P64025|EFTU\_BRUSU

back to list

  

| Protein Coverage
| Supporting Peptides
|

Protein Coverage:

Supporting Peptides:

| Peptide | Uniq | -10lgP | Mass | Length | ppm | m/z | z | RT | Fraction | Scan | Source File | Area F1 | Area F2 | Area F3 | Area F4 | Area F5 | Area F6 | Area F7 | Area F8 | Area F9 | Area F10 | #Feature | #Feature F1 | #Feature F2 | #Feature F3 | #Feature F4 | #Feature F5 | #Feature F6 | #Feature F7 | #Feature F8 | #Feature F9 | #Feature F10 | Start | End | PTM |
| --- | --- | --- | --- | --- | --- | --- | --- | --- | --- | --- | --- | --- | --- | --- | --- | --- | --- | --- | --- | --- | --- | --- | --- | --- | --- | --- | --- | --- | --- | --- | --- | --- | --- | --- | --- |
| K.TTVTGVEMFR.K | Y | 41.45 | 1139.5645 | 10 | 1.0 | 570.7901 | 2 | 45.85 | 2 | F2:14864 | 09012020\_NaNaRj\_F2.raw |  | 1.1E5 |  |  |  |  |  |  |  |  | 1 | 0 | 1 | 0 | 0 | 0 | 0 | 0 | 0 | 0 | 0 | 251 | 260 |  |
| total 1 peptides |  |  |  |  |  |  |  |  |  |  |  |  |  |  |  |  |  |  |  |  |  |  |  |  |  |  |  |  |  |  |  |  |  |  |  |
| --- | --- | --- | --- | --- | --- | --- | --- | --- | --- | --- | --- | --- | --- | --- | --- | --- | --- | --- | --- | --- | --- | --- | --- | --- | --- | --- | --- | --- | --- | --- | --- | --- | --- | --- | --- |

A9M5Q2|EFTU\_BRUC2

back to list

  

| Protein Coverage
| Supporting Peptides
|

Protein Coverage:

Supporting Peptides:

| Peptide | Uniq | -10lgP | Mass | Length | ppm | m/z | z | RT | Fraction | Scan | Source File | Area F1 | Area F2 | Area F3 | Area F4 | Area F5 | Area F6 | Area F7 | Area F8 | Area F9 | Area F10 | #Feature | #Feature F1 | #Feature F2 | #Feature F3 | #Feature F4 | #Feature F5 | #Feature F6 | #Feature F7 | #Feature F8 | #Feature F9 | #Feature F10 | Start | End | PTM |
| --- | --- | --- | --- | --- | --- | --- | --- | --- | --- | --- | --- | --- | --- | --- | --- | --- | --- | --- | --- | --- | --- | --- | --- | --- | --- | --- | --- | --- | --- | --- | --- | --- | --- | --- | --- |
| K.TTVTGVEMFR.K | Y | 41.45 | 1139.5645 | 10 | 1.0 | 570.7901 | 2 | 45.85 | 2 | F2:14864 | 09012020\_NaNaRj\_F2.raw |  | 1.1E5 |  |  |  |  |  |  |  |  | 1 | 0 | 1 | 0 | 0 | 0 | 0 | 0 | 0 | 0 | 0 | 251 | 260 |  |
| total 1 peptides |  |  |  |  |  |  |  |  |  |  |  |  |  |  |  |  |  |  |  |  |  |  |  |  |  |  |  |  |  |  |  |  |  |  |  |
| --- | --- | --- | --- | --- | --- | --- | --- | --- | --- | --- | --- | --- | --- | --- | --- | --- | --- | --- | --- | --- | --- | --- | --- | --- | --- | --- | --- | --- | --- | --- | --- | --- | --- | --- | --- |

P64024|EFTU\_BRUME

back to list

  

| Protein Coverage
| Supporting Peptides
|

Protein Coverage:

Supporting Peptides:

| Peptide | Uniq | -10lgP | Mass | Length | ppm | m/z | z | RT | Fraction | Scan | Source File | Area F1 | Area F2 | Area F3 | Area F4 | Area F5 | Area F6 | Area F7 | Area F8 | Area F9 | Area F10 | #Feature | #Feature F1 | #Feature F2 | #Feature F3 | #Feature F4 | #Feature F5 | #Feature F6 | #Feature F7 | #Feature F8 | #Feature F9 | #Feature F10 | Start | End | PTM |
| --- | --- | --- | --- | --- | --- | --- | --- | --- | --- | --- | --- | --- | --- | --- | --- | --- | --- | --- | --- | --- | --- | --- | --- | --- | --- | --- | --- | --- | --- | --- | --- | --- | --- | --- | --- |
| K.TTVTGVEMFR.K | Y | 41.45 | 1139.5645 | 10 | 1.0 | 570.7901 | 2 | 45.85 | 2 | F2:14864 | 09012020\_NaNaRj\_F2.raw |  | 1.1E5 |  |  |  |  |  |  |  |  | 1 | 0 | 1 | 0 | 0 | 0 | 0 | 0 | 0 | 0 | 0 | 251 | 260 |  |
| total 1 peptides |  |  |  |  |  |  |  |  |  |  |  |  |  |  |  |  |  |  |  |  |  |  |  |  |  |  |  |  |  |  |  |  |  |  |  |
| --- | --- | --- | --- | --- | --- | --- | --- | --- | --- | --- | --- | --- | --- | --- | --- | --- | --- | --- | --- | --- | --- | --- | --- | --- | --- | --- | --- | --- | --- | --- | --- | --- | --- | --- | --- |

B0CH34|EFTU\_BRUSI

back to list

  

| Protein Coverage
| Supporting Peptides
|

Protein Coverage:

Supporting Peptides:

| Peptide | Uniq | -10lgP | Mass | Length | ppm | m/z | z | RT | Fraction | Scan | Source File | Area F1 | Area F2 | Area F3 | Area F4 | Area F5 | Area F6 | Area F7 | Area F8 | Area F9 | Area F10 | #Feature | #Feature F1 | #Feature F2 | #Feature F3 | #Feature F4 | #Feature F5 | #Feature F6 | #Feature F7 | #Feature F8 | #Feature F9 | #Feature F10 | Start | End | PTM |
| --- | --- | --- | --- | --- | --- | --- | --- | --- | --- | --- | --- | --- | --- | --- | --- | --- | --- | --- | --- | --- | --- | --- | --- | --- | --- | --- | --- | --- | --- | --- | --- | --- | --- | --- | --- |
| K.TTVTGVEMFR.K | Y | 41.45 | 1139.5645 | 10 | 1.0 | 570.7901 | 2 | 45.85 | 2 | F2:14864 | 09012020\_NaNaRj\_F2.raw |  | 1.1E5 |  |  |  |  |  |  |  |  | 1 | 0 | 1 | 0 | 0 | 0 | 0 | 0 | 0 | 0 | 0 | 251 | 260 |  |
| total 1 peptides |  |  |  |  |  |  |  |  |  |  |  |  |  |  |  |  |  |  |  |  |  |  |  |  |  |  |  |  |  |  |  |  |  |  |  |
| --- | --- | --- | --- | --- | --- | --- | --- | --- | --- | --- | --- | --- | --- | --- | --- | --- | --- | --- | --- | --- | --- | --- | --- | --- | --- | --- | --- | --- | --- | --- | --- | --- | --- | --- | --- |

A5VR08|EFTU\_BRUO2

back to list

  

| Protein Coverage
| Supporting Peptides
|

Protein Coverage:

Supporting Peptides:

| Peptide | Uniq | -10lgP | Mass | Length | ppm | m/z | z | RT | Fraction | Scan | Source File | Area F1 | Area F2 | Area F3 | Area F4 | Area F5 | Area F6 | Area F7 | Area F8 | Area F9 | Area F10 | #Feature | #Feature F1 | #Feature F2 | #Feature F3 | #Feature F4 | #Feature F5 | #Feature F6 | #Feature F7 | #Feature F8 | #Feature F9 | #Feature F10 | Start | End | PTM |
| --- | --- | --- | --- | --- | --- | --- | --- | --- | --- | --- | --- | --- | --- | --- | --- | --- | --- | --- | --- | --- | --- | --- | --- | --- | --- | --- | --- | --- | --- | --- | --- | --- | --- | --- | --- |
| K.TTVTGVEMFR.K | Y | 41.45 | 1139.5645 | 10 | 1.0 | 570.7901 | 2 | 45.85 | 2 | F2:14864 | 09012020\_NaNaRj\_F2.raw |  | 1.1E5 |  |  |  |  |  |  |  |  | 1 | 0 | 1 | 0 | 0 | 0 | 0 | 0 | 0 | 0 | 0 | 251 | 260 |  |
| total 1 peptides |  |  |  |  |  |  |  |  |  |  |  |  |  |  |  |  |  |  |  |  |  |  |  |  |  |  |  |  |  |  |  |  |  |  |  |
| --- | --- | --- | --- | --- | --- | --- | --- | --- | --- | --- | --- | --- | --- | --- | --- | --- | --- | --- | --- | --- | --- | --- | --- | --- | --- | --- | --- | --- | --- | --- | --- | --- | --- | --- | --- |

A5DPU0|MAK5\_PICGU

back to list

  

| Protein Coverage
| Supporting Peptides
|

Protein Coverage:

Supporting Peptides:

| Peptide | Uniq | -10lgP | Mass | Length | ppm | m/z | z | RT | Fraction | Scan | Source File | Area F1 | Area F2 | Area F3 | Area F4 | Area F5 | Area F6 | Area F7 | Area F8 | Area F9 | Area F10 | #Feature | #Feature F1 | #Feature F2 | #Feature F3 | #Feature F4 | #Feature F5 | #Feature F6 | #Feature F7 | #Feature F8 | #Feature F9 | #Feature F10 | Start | End | PTM |
| --- | --- | --- | --- | --- | --- | --- | --- | --- | --- | --- | --- | --- | --- | --- | --- | --- | --- | --- | --- | --- | --- | --- | --- | --- | --- | --- | --- | --- | --- | --- | --- | --- | --- | --- | --- |
| L.C(+57.02)VVSVC(+57.02)NMPPNKKK.G | Y | 41.05 | 1659.8259 | 14 | 2.7 | 830.9225 | 2 | 12.34 | 6 | F6:3018 | 05112019\_RID\_1313\_NaNaRj\_F6.raw |  |  |  |  |  | 4.89E5 |  |  |  |  | 1 | 0 | 0 | 0 | 0 | 0 | 1 | 0 | 0 | 0 | 0 | 11 | 24 | Carbamidomethylation |
| total 1 peptides |  |  |  |  |  |  |  |  |  |  |  |  |  |  |  |  |  |  |  |  |  |  |  |  |  |  |  |  |  |  |  |  |  |  |  |
| --- | --- | --- | --- | --- | --- | --- | --- | --- | --- | --- | --- | --- | --- | --- | --- | --- | --- | --- | --- | --- | --- | --- | --- | --- | --- | --- | --- | --- | --- | --- | --- | --- | --- | --- | --- |

B8V7Q1|PCDL\_ACRMI

back to list

  

| Protein Coverage
| Supporting Peptides
|

Protein Coverage:

Supporting Peptides:

| Peptide | Uniq | -10lgP | Mass | Length | ppm | m/z | z | RT | Fraction | Scan | Source File | Area F1 | Area F2 | Area F3 | Area F4 | Area F5 | Area F6 | Area F7 | Area F8 | Area F9 | Area F10 | #Feature | #Feature F1 | #Feature F2 | #Feature F3 | #Feature F4 | #Feature F5 | #Feature F6 | #Feature F7 | #Feature F8 | #Feature F9 | #Feature F10 | Start | End | PTM |
| --- | --- | --- | --- | --- | --- | --- | --- | --- | --- | --- | --- | --- | --- | --- | --- | --- | --- | --- | --- | --- | --- | --- | --- | --- | --- | --- | --- | --- | --- | --- | --- | --- | --- | --- | --- |
| R.EFGAAVSLDIK.T | Y | 40.25 | 1148.6077 | 11 | 0.8 | 575.3116 | 2 | 50.87 | 2 | F2:16945 | 09012020\_NaNaRj\_F2.raw |  | 3.36E5 |  |  |  |  |  |  |  |  | 1 | 0 | 1 | 0 | 0 | 0 | 0 | 0 | 0 | 0 | 0 | 1873 | 1883 |  |
| total 1 peptides |  |  |  |  |  |  |  |  |  |  |  |  |  |  |  |  |  |  |  |  |  |  |  |  |  |  |  |  |  |  |  |  |  |  |  |
| --- | --- | --- | --- | --- | --- | --- | --- | --- | --- | --- | --- | --- | --- | --- | --- | --- | --- | --- | --- | --- | --- | --- | --- | --- | --- | --- | --- | --- | --- | --- | --- | --- | --- | --- | --- |

Peptide List

  
  

---

Prepared with PEAKS ™ (bioinfor.com)
