# Supplementary material for: Biogeographical venom variation in the Indian spectacled cobra (Naja naja) underscores the pressing need for pan-India efficacious snakebite therapy
Source: PLoS Negl Trop Dis. 2021 Feb 18;15(2):e0009150. doi: 10.1371/journal.pntd.0009150 (PMC7924803; doi:10.1371/journal.pntd.0009150)
Supplement: S1 Data — (ZIP) [file pntd.0009150.s009.zip › S1 Data/N. naja_West Bengal/N. naja_West Bengal.html]

proteins


Summary

  

# 1. Notes

# 2. Result Statistics

**Figure 1.**
False discovery rate (FDR) curve. X axis is the number of peptide-spectrum matches (PSM) being kept. Y axis is the corresponding FDR.

  


  

**Figure 2.**
PSM score distribution.  **(a)**
Distribution of PEAKS peptide score;  **(b)**
Scatterplot of PEAKS peptide score versus precursor mass error.

**(a)**

**(b)**

**Figure 3.**
Distribution of peptide feature detection.  **(a)**
Feature m/z distribution;  **(b)**
Feature RT distribution

**(a)**

**(b)**

**Figure 4.**
Distribution of identified peptide features.  **(a)**
Feature abundance distribution  **(b)**
 *De novo*
sequencing validation.

**(a)**

**(b)**

**Table 1.**
Statistics of data.

|  |  |
| --- | --- |
| # of MS scans | 193864 |
| # of MS/MS scans | 128425 |
| # of Features | 128429 |
| # of Chimera scans | 18488 |


**Table 2.**
Result filtration parameters.

|  |  |
| --- | --- |
| Peptide -10lgP | ≥41 |
| Peptide Ascore | ≥0 |
| Protein -10lgP | ≥20 |
| Proteins unique peptides | ≥1 |
| De novo ALC Score | ≥50% |


**Table 3.**
Statistics of filtered result.

|  |  |
| --- | --- |
| Peptide-Spectrum Matches | 1369 |
| Peptide sequences | 291 |
| Protein groups | 47 |
| Proteins | 78 |
| Proteins (#Unique Peptides) | 32 (>2); 9 (=2); 37 (=1); |
| FDR (Peptide-Spectrum Matches) | 0.1% |
| FDR (Peptide Sequences) | 0.7% |
| FDR (Protein Group) | 0.0% |
| De Novo Only Spectra | 26493 |
| # of identified Features | 15647 |

**Table 4.**
PTM profile.

| Name | ∆Mass | Position | #PSM | -10lgP | Abundance | AScore || Carbamidomethyl | 57.02 | C | 1028 | 117.03 | 7.11E9 | 1000.00 |
| Oxidation | 15.99 | M | 23 | 67.92 | 9.77E7 | 1000.00 |

# 3. Experiment Control

**Figure 5.**
Precursor mass error of peptide-spectrum matches (PSM) in filtered result.  **(a)**
Distribution of precursor mass error in ppm;  **(b)**
Scatterplot of precursor m/z versus precursor mass error in ppm.

**(a)**

**(b)**

**Table 5.**
Number of identified peptides in each sample by the number of missed cleavages

|  |  |  |  |  |  |
| --- | --- | --- | --- | --- | --- |
| Missed Cleavages | 0 | 1 | 2 | 3 | 4+ |
| F4 | 73 | 12 | 1 | 0 | 0 |
| F1 | 6 | 1 | 0 | 0 | 0 |
| F10 | 1 | 0 | 0 | 0 | 0 |
| F2 | 3 | 0 | 0 | 0 | 0 |
| F3 | 1 | 0 | 0 | 0 | 0 |
| F5 | 47 | 10 | 4 | 0 | 0 |
| F6 | 54 | 7 | 1 | 0 | 0 |
| F7 | 29 | 2 | 0 | 0 | 0 |
| F8 | 21 | 4 | 4 | 0 | 0 |
| F9 | 9 | 1 | 0 | 0 | 0 |

# 4. Other Information

**Table 6.**
Search parameters.

Search Engine Name: PEAKS  
Parent Mass Error Tolerance: 10.0 ppm  
Fragment Mass Error Tolerance: 0.6 Da  
Precursor Mass Search Type: monoisotopic  
Enzyme: Trypsin  
Max Missed Cleavages: 2  
Digest Mode: Semispecific  
Fixed Modifications:   
  Carbamidomethylation: 57.02  
Variable Modifications:   
  Oxidation (M): 15.99  
Max Variable PTM Per Peptide: 3  
Database: SwissProt  
Taxon: All  
Contaminant Database: cRAP\_contaminants  
Searched Entry: 560234  
FDR Estimation: Enabled  
Merge Options: no merge  
Precursor Options: corrected  
Charge Options: no correction  
Filter Charge: 2 - 8  
Process: true  
Associate chimera: yes

**Table 7.**
Instrument parameters.

Fractions: 09012020\_NaNaWb\_F4.raw, 27122019\_RID\_1315\_NaNaWb\_F1.  
raw, 27122019\_RID\_1315\_NaNaWb\_F10.raw, 27122019\_RID\_1315\_NaNa  
Wb\_F2.raw, 27122019\_RID\_1315\_NaNaWb\_F3.raw, 27122019\_RID\_1315  
\_NaNaWb\_F5.raw, 27122019\_RID\_1315\_NaNaWb\_F6.raw, 27122019\_RID  
\_1315\_NaNaWb\_F7.raw, 27122019\_RID\_1315\_NaNaWb\_F8.raw, 2712201  
9\_RID\_1315\_NaNaWb\_F9.raw  
Ion Source: ESI(nano-spray)  
Fragmentation Mode: CID, CAD(y and b ions)  
MS Scan Mode: FT-ICR/Orbitrap  
MS/MS Scan Mode: FT-ICR/Orbitrap

  

Protein List

  

Protein Accession Contains:

Protein Description Contains:

Peptide Sample Area >=

Protein Ptm Contains:

| Protein Group | Protein ID | Accession | -10lgP | Coverage (%) | Coverage (%) F4 | Coverage (%) F1 | Coverage (%) F10 | Coverage (%) F2 | Coverage (%) F3 | Coverage (%) F5 | Coverage (%) F6 | Coverage (%) F7 | Coverage (%) F8 | Coverage (%) F9 | Area F4 | Area F1 | Area F10 | Area F2 | Area F3 | Area F5 | Area F6 | Area F7 | Area F8 | Area F9 | #Peptides | #Unique | #Spec F4 | #Spec F1 | #Spec F10 | #Spec F2 | #Spec F3 | #Spec F5 | #Spec F6 | #Spec F7 | #Spec F8 | #Spec F9 | PTM | Avg. Mass | Description |
| --- | --- | --- | --- | --- | --- | --- | --- | --- | --- | --- | --- | --- | --- | --- | --- | --- | --- | --- | --- | --- | --- | --- | --- | --- | --- | --- | --- | --- | --- | --- | --- | --- | --- | --- | --- | --- | --- | --- | --- |
| 2 | 36 | P01391\|3L21\_NAJKA | 389.67 | 83 | 79 | 18 | 56 | 21 | 41 | 83 | 61 | 75 | 30 | 75 | 8.24E9 |  | 2.51E8 | 3.43E8 | 2.31E8 | 1.17E10 | 3.43E8 | 1.17E8 |  | 8.66E9 | 34 | 7 | 178 | 2 | 8 | 4 | 4 | 137 | 5 | 4 | 3 | 50 | Y | 7831 | Alpha-cobratoxin OS=Naja kaouthia OX=8649 PE=1 SV=1 |
| 3 | 34 | P25668\|3L21\_NAJNA | 335.05 | 96 | 85 | 0 | 62 | 0 | 14 | 80 | 34 | 75 | 62 | 49 | 7.86E7 |  | 9.9E7 |  |  | 1.01E10 |  |  | 1.35E7 |  | 24 | 2 | 219 | 0 | 9 | 0 | 1 | 106 | 7 | 6 | 5 | 37 | Y | 7847 | Long neurotoxin 1 OS=Naja naja OX=35670 PE=1 SV=1 |
| 4 | 49 | P25672\|3L24\_NAJNA | 316.50 | 96 | 66 | 0 | 30 | 0 | 14 | 80 | 34 | 86 | 30 | 59 |  |  |  |  |  | 5.48E8 |  | 1.6E6 |  | 4.04E6 | 24 | 3 | 81 | 0 | 5 | 0 | 1 | 103 | 3 | 5 | 3 | 23 | Y | 7889 | Long neurotoxin 4 OS=Naja naja OX=35670 PE=1 SV=1 |
| 10 | 2 | P04264\|K2C1\_HUMAN | 311.83 | 35 | 28 | 8 | 4 | 0 | 6 | 18 | 22 | 18 | 16 | 4 | 2.13E7 | 5.62E7 | 4.36E6 |  | 2.47E6 | 7.48E6 | 2.42E8 | 3.32E7 | 3.52E7 |  | 25 | 17 | 23 | 5 | 2 | 0 | 3 | 11 | 14 | 10 | 9 | 2 | Y | 66039 | Keratin, type II cytoskeletal 1 OS=Homo sapiens OX=9606 GN=KRT1 PE=1 SV=6 |
| 13 | 4 | #CONTAM#K1C10\_HUMAN\| | 282.49 | 40 | 23 | 7 | 2 | 6 | 3 | 19 | 18 | 6 | 13 | 0 | 1.17E7 | 3.81E6 | 6.55E5 | 1.76E6 | 0E0 | 1.12E7 | 1.74E8 | 3.67E6 | 1.65E7 |  | 26 | 19 | 22 | 3 | 1 | 3 | 1 | 12 | 14 | 3 | 8 | 0 | Y | 59519 | sp\|#CONTAM#K1C10\_HUMAN\| |
| 13 | 5 | P13645\|K1C10\_HUMAN | 282.49 | 41 | 23 | 7 | 2 | 6 | 3 | 19 | 19 | 6 | 13 | 0 | 1.17E7 | 3.81E6 | 6.55E5 | 1.76E6 | 0E0 | 1.12E7 | 1.74E8 | 3.67E6 | 1.65E7 |  | 26 | 19 | 22 | 3 | 1 | 3 | 1 | 12 | 14 | 3 | 8 | 0 | Y | 58827 | Keratin, type I cytoskeletal 10 OS=Homo sapiens OX=9606 GN=KRT10 PE=1 SV=6 |
| 9 | 79 | P25674\|3L21\_NAJHH | 276.01 | 58 | 49 | 18 | 0 | 18 | 18 | 58 | 20 | 18 | 0 | 37 | 3.43E7 |  |  |  |  | 1.05E7 |  |  |  |  | 14 | 2 | 48 | 2 | 0 | 1 | 1 | 48 | 1 | 1 | 0 | 9 | Y | 7821 | Long neurotoxin 1 OS=Naja haje haje OX=8642 PE=1 SV=1 |
| 15 | 6 | P35527\|K1C9\_HUMAN | 272.23 | 38 | 18 | 5 | 3 | 1 | 0 | 5 | 21 | 21 | 8 | 3 | 7.14E6 | 9.4E6 | 2.71E6 | 7.31E5 |  | 2.5E5 | 7.85E7 | 2.41E7 | 1.18E7 | 7.08E5 | 18 | 17 | 15 | 2 | 1 | 1 | 0 | 3 | 11 | 8 | 3 | 1 | Y | 62064 | Keratin, type I cytoskeletal 9 OS=Homo sapiens OX=9606 GN=KRT9 PE=1 SV=3 |
| 15 | 7 | #CONTAM#K1C9\_HUMAN\| | 272.23 | 38 | 18 | 5 | 3 | 1 | 0 | 5 | 21 | 21 | 8 | 3 | 7.14E6 | 9.4E6 | 2.71E6 | 7.31E5 |  | 2.5E5 | 7.85E7 | 2.41E7 | 1.18E7 | 7.08E5 | 18 | 17 | 15 | 2 | 1 | 1 | 0 | 3 | 11 | 8 | 3 | 1 | Y | 62129 | sp\|#CONTAM#K1C9\_HUMAN\| |
| 16 | 9 | P35908\|K22E\_HUMAN | 268.26 | 36 | 26 | 6 | 0 | 0 | 0 | 8 | 15 | 3 | 8 | 2 | 4.97E6 | 2.57E6 |  |  |  | 3.67E5 | 7.71E7 | 0E0 | 5.35E6 |  | 18 | 12 | 14 | 3 | 0 | 0 | 0 | 5 | 9 | 2 | 4 | 1 | Y | 65433 | Keratin, type II cytoskeletal 2 epidermal OS=Homo sapiens OX=9606 GN=KRT2 PE=1 SV=2 |
| 14 | 87 | P25498\|PA2AE\_NAJOX | 243.00 | 52 | 0 | 0 | 12 | 0 | 0 | 12 | 52 | 29 | 0 | 12 |  |  |  |  |  |  | 2.36E7 |  |  |  | 10 | 1 | 0 | 0 | 1 | 0 | 0 | 1 | 48 | 8 | 0 | 1 | Y | 13229 | Acidic phospholipase A2 E OS=Naja oxiana OX=8657 PE=1 SV=1 |
| 17 | 11 | P02768\|ALBU\_HUMAN | 226.70 | 28 | 24 | 0 | 0 | 4 | 0 | 20 | 2 | 5 | 0 | 2 | 6.67E6 |  |  | 4.69E5 |  | 3.26E6 | 4.47E5 | 7.05E5 |  | 0E0 | 18 | 11 | 18 | 0 | 0 | 2 | 0 | 13 | 1 | 3 | 0 | 1 | Y | 69367 | Serum albumin OS=Homo sapiens OX=9606 GN=ALB PE=1 SV=2 |
| 17 | 12 | #CONTAM#ALBU\_HUMAN\| | 226.70 | 28 | 24 | 0 | 0 | 4 | 0 | 20 | 2 | 5 | 0 | 2 | 6.67E6 |  |  | 4.69E5 |  | 3.26E6 | 4.47E5 | 7.05E5 |  | 0E0 | 18 | 11 | 18 | 0 | 0 | 2 | 0 | 13 | 1 | 3 | 0 | 1 | Y | 69367 | sp\|#CONTAM#ALBU\_HUMAN\| |
| 25 | 18 | P13647\|K2C5\_HUMAN | 197.02 | 19 | 8 | 0 | 0 | 0 | 0 | 7 | 4 | 4 | 4 | 0 | 3.36E5 |  |  |  |  | 0E0 | 3.78E6 | 1.07E6 |  |  | 10 | 3 | 5 | 0 | 0 | 0 | 0 | 4 | 2 | 2 | 2 | 0 | Y | 62378 | Keratin, type II cytoskeletal 5 OS=Homo sapiens OX=9606 GN=KRT5 PE=1 SV=3 |
| 23 | 15 | P02533\|K1C14\_HUMAN | 193.61 | 27 | 12 | 0 | 0 | 0 | 0 | 9 | 9 | 0 | 8 | 0 | 9.12E4 |  |  |  |  |  | 0E0 |  | 1.94E5 |  | 12 | 4 | 8 | 0 | 0 | 0 | 0 | 4 | 3 | 0 | 3 | 0 | N | 51562 | Keratin, type I cytoskeletal 14 OS=Homo sapiens OX=9606 GN=KRT14 PE=1 SV=4 |
| 31 | 32 | D5LMJ3\|VM3A\_NAJAT | 189.17 | 9 | 6 | 0 | 0 | 0 | 0 | 3 | 0 | 5 | 0 | 0 | 3.34E7 |  |  |  |  | 7.03E6 |  | 1.22E7 |  |  | 6 | 6 | 9 | 0 | 0 | 0 | 0 | 3 | 0 | 3 | 0 | 0 | Y | 68254 | Zinc metalloproteinase-disintegrin-like atrase-A OS=Naja atra OX=8656 PE=2 SV=1 |
| 22 | 40 | Q5YF89\|NGFV2\_NAJSP | 176.32 | 32 | 0 | 0 | 0 | 0 | 0 | 0 | 0 | 32 | 10 | 0 |  |  |  |  |  |  |  | 1.13E9 | 1.66E6 |  | 8 | 8 | 0 | 0 | 0 | 0 | 0 | 0 | 0 | 16 | 1 | 0 | Y | 27030 | Venom nerve growth factor 2 OS=Naja sputatrix OX=33626 PE=2 SV=1 |
| 19 | 143 | P01427\|3S11\_NAJOX | 176.19 | 54 | 18 | 30 | 0 | 18 | 43 | 18 | 0 | 0 | 0 | 43 | 1.15E6 | 1.78E9 |  | 2.27E8 | 2.37E9 | 3.56E6 |  |  |  | 7.88E7 | 5 | 5 | 1 | 10 | 0 | 5 | 9 | 2 | 0 | 0 | 0 | 4 | Y | 6885 | Short neurotoxin 1 OS=Naja oxiana OX=8657 PE=1 SV=1 |
| 32 | 37 | P84808\|CRVP2\_NAJKA | 166.23 | 54 | 0 | 0 | 0 | 0 | 0 | 0 | 0 | 0 | 54 | 0 |  |  |  |  |  |  |  |  | 2.98E8 |  | 10 | 10 | 0 | 0 | 0 | 0 | 0 | 0 | 0 | 0 | 11 | 0 | Y | 26216 | Cysteine-rich venom protein kaouthin-2 OS=Naja kaouthia OX=8649 PE=1 SV=2 |
| 18 | 65 | Q9PST3\|3SA2B\_NAJSP | 159.16 | 68 | 10 | 0 | 0 | 0 | 0 | 10 | 20 | 20 | 68 | 0 |  |  |  |  |  |  | 4.02E6 | 3.44E7 | 2.55E8 |  | 7 | 2 | 1 | 0 | 0 | 0 | 0 | 1 | 4 | 2 | 22 | 0 | Y | 9042 | Cytotoxin 2b OS=Naja sputatrix OX=33626 PE=3 SV=1 |
| 18 | 66 | Q9PST4\|3SA2A\_NAJSP | 159.16 | 68 | 10 | 0 | 0 | 0 | 0 | 10 | 20 | 20 | 68 | 0 |  |  |  |  |  |  | 4.02E6 | 3.44E7 | 2.55E8 |  | 7 | 2 | 1 | 0 | 0 | 0 | 0 | 1 | 4 | 2 | 22 | 0 | Y | 9054 | Cytotoxin 2a OS=Naja sputatrix OX=33626 PE=2 SV=1 |
| 18 | 67 | A0A0U5AUY6\|3SA9\_NAJNA | 159.16 | 76 | 11 | 0 | 0 | 0 | 0 | 11 | 22 | 22 | 76 | 0 |  |  |  |  |  |  | 4.02E6 | 3.44E7 | 2.55E8 |  | 7 | 2 | 1 | 0 | 0 | 0 | 0 | 1 | 4 | 2 | 22 | 0 | Y | 8041 | Cytotoxin 9 (Fragment) OS=Naja naja OX=35670 PE=1 SV=1 |
| 18 | 75 | P86541\|3SAA\_NAJNA | 159.16 | 92 | 13 | 0 | 0 | 0 | 0 | 13 | 27 | 27 | 92 | 0 |  |  |  |  |  |  | 4.02E6 | 3.44E7 | 2.55E8 |  | 7 | 2 | 1 | 0 | 0 | 0 | 0 | 1 | 4 | 2 | 22 | 0 | Y | 6764 | Cytotoxin 10 OS=Naja naja OX=35670 PE=1 SV=2 |
| 45 | 494 | P10117\|PA2H1\_LATCO | 149.80 | 13 | 0 | 0 | 0 | 0 | 0 | 0 | 10 | 13 | 0 | 0 |  |  |  |  |  |  |  | 3.94E6 |  |  | 3 | 1 | 0 | 0 | 0 | 0 | 0 | 0 | 7 | 4 | 0 | 0 | Y | 13024 | Basic phospholipase A2 homolog 1 OS=Laticauda colubrina OX=8628 PE=1 SV=1 |
| 21 | 131 | P86382\|3SA7\_NAJNA | 149.73 | 62 | 13 | 0 | 0 | 0 | 0 | 13 | 62 | 13 | 15 | 0 |  |  |  |  |  |  | 6.64E9 |  |  |  | 6 | 4 | 1 | 0 | 0 | 0 | 0 | 1 | 14 | 1 | 6 | 0 | Y | 6792 | Cytotoxin 7 OS=Naja naja OX=35670 PE=1 SV=1 |
| 21 | 149 | P86540\|3SA8\_NAJNA | 149.73 | 62 | 13 | 0 | 0 | 0 | 0 | 13 | 62 | 13 | 15 | 0 |  |  |  |  |  |  | 6.64E9 |  |  |  | 6 | 4 | 1 | 0 | 0 | 0 | 0 | 1 | 14 | 1 | 6 | 0 | Y | 6793 | Cytotoxin 8 OS=Naja naja OX=35670 PE=1 SV=2 |
| 48 | 90 | P82464\|3SO8\_NAJKA | 147.47 | 86 | 0 | 0 | 0 | 0 | 0 | 0 | 42 | 15 | 0 | 60 |  |  |  |  |  |  | 2.17E7 | 1.03E6 |  | 2.54E8 | 6 | 6 | 0 | 0 | 0 | 0 | 0 | 0 | 2 | 1 | 0 | 6 | Y | 7624 | Muscarinic toxin-like protein 3 OS=Naja kaouthia OX=8649 PE=1 SV=1 |
| 58 | 144 | P82885\|VESP\_NAJKA | 147.02 | 48 | 0 | 0 | 0 | 0 | 0 | 0 | 0 | 16 | 32 | 0 |  |  |  |  |  |  |  | 0E0 | 1.74E8 |  | 4 | 4 | 0 | 0 | 0 | 0 | 0 | 0 | 0 | 1 | 4 | 0 | Y | 12038 | Thaicobrin OS=Naja kaouthia OX=8649 PE=1 SV=1 |
| 34 | 339 | Q91137\|3SOFL\_NAJAT | 137.93 | 16 | 13 | 0 | 0 | 0 | 0 | 0 | 13 | 16 | 13 | 0 | 3.5E5 |  |  |  |  |  | 6.33E7 | 8.91E8 | 0E0 |  | 3 | 3 | 1 | 0 | 0 | 0 | 0 | 0 | 5 | 7 | 1 | 0 | Y | 9309 | Cytotoxin homolog 5 OS=Naja atra OX=8656 PE=3 SV=1 |
| 34 | 370 | Q9W716\|3SOFV\_NAJAT | 137.93 | 16 | 13 | 0 | 0 | 0 | 0 | 0 | 13 | 16 | 13 | 0 | 3.5E5 |  |  |  |  |  | 6.33E7 | 8.91E8 | 0E0 |  | 3 | 3 | 1 | 0 | 0 | 0 | 0 | 0 | 5 | 7 | 1 | 0 | Y | 9333 | Cytotoxin homolog 5V OS=Naja atra OX=8656 PE=3 SV=1 |
| 34 | 371 | Q91126\|3SOFA\_NAJAT | 137.93 | 16 | 13 | 0 | 0 | 0 | 0 | 0 | 13 | 16 | 13 | 0 | 3.5E5 |  |  |  |  |  | 6.33E7 | 8.91E8 | 0E0 |  | 3 | 3 | 1 | 0 | 0 | 0 | 0 | 0 | 5 | 7 | 1 | 0 | Y | 9319 | Cardiotoxin 7a OS=Naja atra OX=8656 PE=3 SV=1 |
| 34 | 372 | Q91996\|3SOFP\_NAJAT | 137.93 | 16 | 13 | 0 | 0 | 0 | 0 | 0 | 13 | 16 | 13 | 0 | 3.5E5 |  |  |  |  |  | 6.33E7 | 8.91E8 | 0E0 |  | 3 | 3 | 1 | 0 | 0 | 0 | 0 | 0 | 5 | 7 | 1 | 0 | Y | 9305 | Cardiotoxin 7'' OS=Naja atra OX=8656 PE=3 SV=1 |
| 34 | 378 | P62377\|3SOFL\_NAJNA | 137.93 | 21 | 18 | 0 | 0 | 0 | 0 | 0 | 18 | 21 | 18 | 0 | 3.5E5 |  |  |  |  |  | 6.33E7 | 8.91E8 | 0E0 |  | 3 | 3 | 1 | 0 | 0 | 0 | 0 | 0 | 5 | 7 | 1 | 0 | Y | 7014 | Cytotoxin-like basic protein OS=Naja naja OX=35670 PE=1 SV=1 |
| 34 | 379 | P62375\|3SOF5\_NAJAT | 137.93 | 16 | 13 | 0 | 0 | 0 | 0 | 0 | 13 | 16 | 13 | 0 | 3.5E5 |  |  |  |  |  | 6.33E7 | 8.91E8 | 0E0 |  | 3 | 3 | 1 | 0 | 0 | 0 | 0 | 0 | 5 | 7 | 1 | 0 | Y | 9323 | Cytotoxin A5 OS=Naja atra OX=8656 PE=1 SV=1 |
| 34 | 496 | P62390\|3SOFB\_NAJHA | 137.93 | 21 | 18 | 0 | 0 | 0 | 0 | 0 | 18 | 21 | 18 | 0 | 3.5E5 |  |  |  |  |  | 6.33E7 | 8.91E8 | 0E0 |  | 3 | 3 | 1 | 0 | 0 | 0 | 0 | 0 | 5 | 7 | 1 | 0 | Y | 6842 | Cytotoxin 11 OS=Naja annulifera OX=96794 PE=1 SV=1 |
| 34 | 497 | P62394\|3SOFB\_NAJHH | 137.93 | 21 | 18 | 0 | 0 | 0 | 0 | 0 | 18 | 21 | 18 | 0 | 3.5E5 |  |  |  |  |  | 6.33E7 | 8.91E8 | 0E0 |  | 3 | 3 | 1 | 0 | 0 | 0 | 0 | 0 | 5 | 7 | 1 | 0 | Y | 6842 | Cytotoxin 11 OS=Naja haje haje OX=8642 PE=1 SV=1 |
| 34 | 498 | P14541\|3SOFH\_NAJKA | 137.93 | 21 | 18 | 0 | 0 | 0 | 0 | 0 | 18 | 21 | 18 | 0 | 3.5E5 |  |  |  |  |  | 6.33E7 | 8.91E8 | 0E0 |  | 3 | 3 | 1 | 0 | 0 | 0 | 0 | 0 | 5 | 7 | 1 | 0 | Y | 6994 | Cytotoxin homolog OS=Naja kaouthia OX=8649 PE=1 SV=1 |
| 49 | 156 | P20229\|VKTTI\_NAJNA | 137.86 | 58 | 40 | 0 | 0 | 0 | 37 | 19 | 0 | 0 | 0 | 0 | 2.19E6 |  |  |  | 3.29E8 | 3.61E5 |  |  |  |  | 5 | 5 | 4 | 0 | 0 | 0 | 3 | 2 | 0 | 0 | 0 | 0 | Y | 6371 | Kunitz-type serine protease inhibitor OS=Naja naja OX=35670 PE=1 SV=1 |
| 28 | 172 | P80245\|3SAN\_NAJAT | 125.44 | 42 | 10 | 0 | 0 | 0 | 0 | 10 | 22 | 10 | 30 | 0 |  |  |  |  |  |  | 2.61E5 |  |  |  | 4 | 1 | 1 | 0 | 0 | 0 | 0 | 1 | 4 | 1 | 10 | 0 | Y | 8980 | Cytotoxin 6 OS=Naja atra OX=8656 PE=1 SV=2 |
| 28 | 173 | Q98965\|3SA6\_NAJAT | 125.44 | 41 | 10 | 0 | 0 | 0 | 0 | 10 | 22 | 10 | 29 | 0 |  |  |  |  |  |  | 2.61E5 |  |  |  | 4 | 1 | 1 | 0 | 0 | 0 | 0 | 1 | 4 | 1 | 10 | 0 | Y | 9201 | Cytotoxin 6 OS=Naja atra OX=8656 PE=3 SV=1 |
| 46 | 502 | F8J2D0\|PA214\_DRYCN | 123.44 | 9 | 0 | 0 | 0 | 0 | 0 | 0 | 9 | 8 | 0 | 0 |  |  |  |  |  |  | 3.4E6 |  |  |  | 3 | 1 | 0 | 0 | 0 | 0 | 0 | 0 | 8 | 3 | 0 | 0 | N | 16055 | Phospholipase A2 147 OS=Drysdalia coronoides OX=66186 PE=1 SV=1 |
| 46 | 503 | F8J2D2\|PA222\_DRYCN | 123.44 | 9 | 0 | 0 | 0 | 0 | 0 | 0 | 9 | 8 | 0 | 0 |  |  |  |  |  |  | 3.4E6 |  |  |  | 3 | 1 | 0 | 0 | 0 | 0 | 0 | 0 | 8 | 3 | 0 | 0 | N | 16011 | Phospholipase A2 PS22 OS=Drysdalia coronoides OX=66186 PE=1 SV=1 |
| 52 | 145 | Q7Z794\|K2C1B\_HUMAN | 121.65 | 5 | 5 | 0 | 0 | 0 | 0 | 3 | 2 | 0 | 2 | 2 | 1.66E6 |  |  |  |  |  |  |  |  |  | 3 | 1 | 4 | 0 | 0 | 0 | 0 | 2 | 2 | 0 | 1 | 1 | N | 61901 | Keratin, type II cytoskeletal 1b OS=Homo sapiens OX=9606 GN=KRT77 PE=2 SV=3 |
| 26 | 485 | P58370\|3NO48\_MICCO | 117.76 | 15 | 15 | 0 | 0 | 0 | 0 | 15 | 0 | 15 | 15 | 15 | 1.16E8 |  |  |  |  | 3.71E9 |  | 7.18E7 | 2.07E7 | 7.01E7 | 2 | 2 | 5 | 0 | 0 | 0 | 0 | 10 | 0 | 1 | 1 | 1 | Y | 9591 | Alpha-neurotoxin homolog 8 OS=Micrurus corallinus OX=54390 GN=NXH8 PE=1 SV=1 |
| 42 | 72 | P00761\|TRYP\_PIG | 110.59 | 15 | 15 | 0 | 0 | 0 | 0 | 0 | 0 | 0 | 0 | 0 | 1.07E7 |  |  |  |  |  |  |  |  |  | 3 | 3 | 3 | 0 | 0 | 0 | 0 | 0 | 0 | 0 | 0 | 0 | N | 24409 | Trypsin OS=Sus scrofa OX=9823 PE=1 SV=1 |
| 42 | 73 | #CONTAM#TRYP\_PIG\| | 110.59 | 15 | 15 | 0 | 0 | 0 | 0 | 0 | 0 | 0 | 0 | 0 | 1.07E7 |  |  |  |  |  |  |  |  |  | 3 | 3 | 3 | 0 | 0 | 0 | 0 | 0 | 0 | 0 | 0 | 0 | N | 24409 | sp\|#CONTAM#TRYP\_PIG\| |
| 55 | 205 | D6PXE8\|VM3B\_NAJAT | 108.75 | 4 | 4 | 0 | 0 | 0 | 0 | 0 | 4 | 0 | 0 | 0 | 1.55E7 |  |  |  |  |  | 0E0 |  |  |  | 1 | 1 | 2 | 0 | 0 | 0 | 0 | 0 | 1 | 0 | 0 | 0 | Y | 66246 | Zinc metalloproteinase-disintegrin-like atrase-B OS=Naja atra OX=8656 PE=1 SV=1 |
| 55 | 207 | D3TTC1\|VM3KL\_NAJAT | 108.75 | 4 | 4 | 0 | 0 | 0 | 0 | 0 | 4 | 0 | 0 | 0 | 1.55E7 |  |  |  |  |  | 0E0 |  |  |  | 1 | 1 | 2 | 0 | 0 | 0 | 0 | 0 | 1 | 0 | 0 | 0 | Y | 66292 | Zinc metalloproteinase-disintegrin-like kaouthiagin-like OS=Naja atra OX=8656 PE=1 SV=1 |
| 59 | 216 | Q6IZ95\|3NO24\_BUNCA | 103.03 | 22 | 10 | 0 | 0 | 0 | 0 | 10 | 0 | 0 | 0 | 22 |  |  |  |  |  |  |  |  |  | 2.11E7 | 2 | 1 | 3 | 0 | 0 | 0 | 0 | 1 | 0 | 0 | 0 | 2 | Y | 9723 | Neurotoxin Ntx4 OS=Bungarus candidus OX=92438 PE=3 SV=1 |
| 59 | 217 | A2CKF7\|3NO2\_BUNFA | 103.03 | 22 | 10 | 0 | 0 | 0 | 0 | 10 | 0 | 0 | 0 | 22 |  |  |  |  |  |  |  |  |  | 2.11E7 | 2 | 1 | 3 | 0 | 0 | 0 | 0 | 1 | 0 | 0 | 0 | 2 | Y | 9723 | Neurotoxin 3FTx-LT OS=Bungarus fasciatus OX=8613 PE=1 SV=1 |
| 59 | 233 | O93422\|3NO2H\_NAJAT | 103.03 | 22 | 10 | 0 | 0 | 0 | 0 | 10 | 0 | 0 | 0 | 22 |  |  |  |  |  |  |  |  |  | 2.11E7 | 2 | 1 | 3 | 0 | 0 | 0 | 0 | 1 | 0 | 0 | 0 | 2 | Y | 9815 | Long neurotoxin homolog OS=Naja atra OX=8656 PE=3 SV=1 |
| 59 | 234 | Q9YGI2\|3NO21\_NAJAT | 103.03 | 22 | 10 | 0 | 0 | 0 | 0 | 10 | 0 | 0 | 0 | 22 |  |  |  |  |  |  |  |  |  | 2.11E7 | 2 | 1 | 3 | 0 | 0 | 0 | 0 | 1 | 0 | 0 | 0 | 2 | Y | 9845 | Probable weak neurotoxin NNAM1 OS=Naja atra OX=8656 PE=3 SV=1 |
| 59 | 252 | P01400\|3NO2B\_NAJME | 103.03 | 29 | 14 | 0 | 0 | 0 | 0 | 14 | 0 | 0 | 0 | 29 |  |  |  |  |  |  |  |  |  | 2.11E7 | 2 | 1 | 3 | 0 | 0 | 0 | 0 | 1 | 0 | 0 | 0 | 2 | Y | 7430 | Weak toxin S4C11 OS=Naja melanoleuca OX=8643 PE=1 SV=1 |
| 59 | 302 | P85520\|3NO2\_NAJOX | 103.03 | 29 | 14 | 0 | 0 | 0 | 0 | 14 | 0 | 0 | 0 | 29 |  |  |  |  |  |  |  |  |  | 2.11E7 | 2 | 1 | 3 | 0 | 0 | 0 | 0 | 1 | 0 | 0 | 0 | 2 | Y | 7482 | Oxiana weak toxin OS=Naja oxiana OX=8657 PE=1 SV=1 |
| 62 | 215 | Q9W717\|3SO1\_NAJAT | 100.28 | 31 | 0 | 0 | 0 | 0 | 0 | 0 | 31 | 9 | 0 | 0 |  |  |  |  |  |  | 4.96E7 | 2.4E7 |  |  | 2 | 2 | 0 | 0 | 0 | 0 | 0 | 0 | 3 | 1 | 0 | 0 | Y | 9695 | Neurotoxin-like protein NTL2 OS=Naja atra OX=8656 PE=3 SV=1 |
| 65 | 189 | Q91132\|VCO3\_NAJKA | 94.99 | 2 | 0 | 0 | 0 | 0 | 0 | 0 | 0 | 2 | 0 | 0 |  |  |  |  |  |  |  | 1.76E5 |  |  | 2 | 1 | 0 | 0 | 0 | 0 | 0 | 1 | 0 | 2 | 0 | 0 | Y | 184517 | Cobra venom factor OS=Naja kaouthia OX=8649 PE=1 SV=1 |
| 61 | 187 | P82942\|VM3K\_NAJKA | 79.51 | 11 | 0 | 0 | 0 | 0 | 0 | 0 | 6 | 0 | 5 | 0 |  |  |  |  |  |  | 2.12E6 |  | 5.52E5 |  | 2 | 2 | 0 | 0 | 0 | 0 | 0 | 0 | 1 | 0 | 1 | 0 | Y | 44493 | Hemorrhagic metalloproteinase-disintegrin-like kaouthiagin OS=Naja kaouthia OX=8649 PE=1 SV=1 |
| 63 | 195 | P81605\|DCD\_HUMAN | 77.60 | 10 | 0 | 0 | 0 | 0 | 0 | 0 | 10 | 0 | 0 | 0 |  |  |  |  |  |  | 5.6E5 |  |  |  | 1 | 1 | 0 | 0 | 0 | 0 | 0 | 0 | 1 | 0 | 0 | 0 | N | 11284 | Dermcidin OS=Homo sapiens OX=9606 GN=DCD PE=1 SV=2 |
| 105 | 337 | Q7LZI1\|PLIB\_NAJKA | 73.89 | 8 | 0 | 0 | 0 | 0 | 0 | 0 | 0 | 8 | 0 | 0 |  |  |  |  |  |  |  | 4.19E5 |  |  | 1 | 1 | 0 | 0 | 0 | 0 | 0 | 0 | 0 | 1 | 0 | 0 | N | 20452 | Phospholipase A2 inhibitor 31 kDa subunit OS=Naja kaouthia OX=8649 PE=1 SV=1 |
| 67 | 294 | Q9PSN6\|3S13\_NAJSP | 71.28 | 19 | 19 | 0 | 0 | 19 | 0 | 19 | 0 | 0 | 0 | 0 | 9.81E5 |  |  | 1.15E7 |  | 3.21E5 |  |  |  |  | 1 | 1 | 1 | 0 | 0 | 1 | 0 | 1 | 0 | 0 | 0 | 0 | Y | 6958 | Neurotoxin 3 OS=Naja sputatrix OX=33626 PE=1 SV=1 |
| 67 | 295 | P14613\|3S1B1\_NAJKA | 71.28 | 19 | 19 | 0 | 0 | 19 | 0 | 19 | 0 | 0 | 0 | 0 | 9.81E5 |  |  | 1.15E7 |  | 3.21E5 |  |  |  |  | 1 | 1 | 1 | 0 | 0 | 1 | 0 | 1 | 0 | 0 | 0 | 0 | Y | 6983 | Short neurotoxin 1 OS=Naja kaouthia OX=8649 PE=1 SV=1 |
| 67 | 296 | P60770\|3S1CB\_NAJAT | 71.28 | 14 | 14 | 0 | 0 | 14 | 0 | 14 | 0 | 0 | 0 | 0 | 9.81E5 |  |  | 1.15E7 |  | 3.21E5 |  |  |  |  | 1 | 1 | 1 | 0 | 0 | 1 | 0 | 1 | 0 | 0 | 0 | 0 | Y | 9262 | Cobrotoxin OS=Naja atra OX=8656 PE=1 SV=1 |
| 67 | 297 | P60771\|3S1CB\_NAJKA | 71.28 | 14 | 14 | 0 | 0 | 14 | 0 | 14 | 0 | 0 | 0 | 0 | 9.81E5 |  |  | 1.15E7 |  | 3.21E5 |  |  |  |  | 1 | 1 | 1 | 0 | 0 | 1 | 0 | 1 | 0 | 0 | 0 | 0 | Y | 9262 | Cobrotoxin OS=Naja kaouthia OX=8649 PE=1 SV=2 |
| 67 | 298 | Q9PTT0\|3S1CB\_NAJNA | 71.28 | 14 | 14 | 0 | 0 | 14 | 0 | 14 | 0 | 0 | 0 | 0 | 9.81E5 |  |  | 1.15E7 |  | 3.21E5 |  |  |  |  | 1 | 1 | 1 | 0 | 0 | 1 | 0 | 1 | 0 | 0 | 0 | 0 | Y | 9262 | Cobrotoxin homolog OS=Naja naja OX=35670 PE=1 SV=1 |
| 69 | 193 | Q9PVK7\|VM3\_NAJKA | 65.09 | 2 | 0 | 0 | 0 | 0 | 0 | 0 | 2 | 2 | 0 | 0 |  |  |  |  |  |  | 1.45E6 | 5.08E5 |  |  | 1 | 1 | 0 | 0 | 0 | 0 | 0 | 0 | 1 | 1 | 0 | 0 | N | 67662 | Zinc metalloproteinase-disintegrin-like cobrin OS=Naja kaouthia OX=8649 PE=2 SV=1 |
| 78 | 323 | F8RKW1\|VM34\_DRYCN | 62.07 | 3 | 3 | 0 | 0 | 0 | 0 | 0 | 0 | 0 | 0 | 0 | 2.38E5 |  |  |  |  |  |  |  |  |  | 1 | 1 | 1 | 0 | 0 | 0 | 0 | 0 | 0 | 0 | 0 | 0 | Y | 68240 | Zinc metalloproteinase-disintegrin-like MTP4 OS=Drysdalia coronoides OX=66186 PE=1 SV=1 |
| 101 | 373 | P82849\|3S1B2\_NAJKA | 59.75 | 27 | 0 | 0 | 0 | 27 | 0 | 0 | 0 | 0 | 0 | 0 |  |  |  | 3.76E7 |  |  |  |  |  |  | 1 | 1 | 0 | 0 | 0 | 1 | 0 | 0 | 0 | 0 | 0 | 0 | Y | 6862 | Cobrotoxin II OS=Naja kaouthia OX=8649 PE=1 SV=1 |
| 71 | 430 | P0CAR1\|3S1D1\_MICPY | 54.64 | 25 | 0 | 0 | 0 | 25 | 0 | 0 | 0 | 0 | 0 | 0 |  |  |  | 5.03E5 |  |  |  |  |  |  | 1 | 1 | 0 | 0 | 0 | 1 | 0 | 0 | 0 | 0 | 0 | 0 | Y | 6533 | Short neurotoxin D1 OS=Micrurus pyrrhocryptus OX=129468 PE=1 SV=1 |
| 35 | 274 | Q2VQM5\|VIT3\_SOLIN | 54.54 | 0 | 0 | 0 | 0 | 0 | 0 | 0 | 0 | 0 | 0 | 0 |  |  |  |  |  |  | 2.8E8 |  | 2.65E10 |  | 1 | 1 | 0 | 0 | 0 | 0 | 0 | 0 | 2 | 0 | 10 | 0 | N | 201052 | Vitellogenin-3 OS=Solenopsis invicta OX=13686 PE=2 SV=1 |
| 35 | 327 | Q2VQM6\|VIT2\_SOLIN | 54.54 | 0 | 0 | 0 | 0 | 0 | 0 | 0 | 0 | 0 | 0 | 0 |  |  |  |  |  |  | 2.8E8 |  | 2.65E10 |  | 1 | 1 | 0 | 0 | 0 | 0 | 0 | 0 | 2 | 0 | 10 | 0 | N | 204974 | Vitellogenin-2 OS=Solenopsis invicta OX=13686 PE=2 SV=1 |
| 112 | 434 | Q5VSP4\|LC1L1\_HUMAN | 50.25 | 7 | 0 | 7 | 0 | 0 | 0 | 0 | 0 | 0 | 0 | 0 |  | 0E0 |  |  |  |  |  |  |  |  | 1 | 1 | 0 | 1 | 0 | 0 | 0 | 0 | 0 | 0 | 0 | 0 | N | 17918 | Putative lipocalin 1-like protein 1 OS=Homo sapiens OX=9606 GN=LCN1P1 PE=5 SV=1 |
| 112 | 510 | P31025\|LCN1\_HUMAN | 50.25 | 6 | 0 | 6 | 0 | 0 | 0 | 0 | 0 | 0 | 0 | 0 |  | 0E0 |  |  |  |  |  |  |  |  | 1 | 1 | 0 | 1 | 0 | 0 | 0 | 0 | 0 | 0 | 0 | 0 | N | 19250 | Lipocalin-1 OS=Homo sapiens OX=9606 GN=LCN1 PE=1 SV=1 |
| 118 | 515 | A1TYI9\|RL7\_MARHV | 46.34 | 8 | 8 | 0 | 0 | 0 | 0 | 0 | 0 | 0 | 0 | 0 | 2.13E6 |  |  |  |  |  |  |  |  |  | 1 | 1 | 1 | 0 | 0 | 0 | 0 | 0 | 0 | 0 | 0 | 0 | N | 12791 | 50S ribosomal protein L7/L12 OS=Marinobacter hydrocarbonoclasticus (strain ATCC 700491 / DSM 11845 / VT8) OX=351348 GN=rplL PE=3 SV=1 |
| 68 | 190 | P84805\|CRVP1\_NAJKA | 43.45 | 4 | 0 | 0 | 0 | 0 | 0 | 4 | 0 | 0 | 0 | 0 |  |  |  |  |  | 5.81E4 |  |  |  |  | 1 | 1 | 0 | 0 | 0 | 0 | 0 | 1 | 0 | 0 | 0 | 0 | Y | 26846 | Cysteine-rich venom protein kaouthin-1 OS=Naja kaouthia OX=8649 PE=1 SV=2 |
| 68 | 191 | Q7T1K6\|CRVP1\_NAJAT | 43.45 | 4 | 0 | 0 | 0 | 0 | 0 | 4 | 0 | 0 | 0 | 0 |  |  |  |  |  | 5.81E4 |  |  |  |  | 1 | 1 | 0 | 0 | 0 | 0 | 0 | 1 | 0 | 0 | 0 | 0 | Y | 26882 | Cysteine-rich venom protein natrin-1 OS=Naja atra OX=8656 PE=1 SV=1 |
| 110 | 538 | P50079\|HSV2\_YEAST | 43.04 | 2 | 2 | 0 | 0 | 0 | 0 | 0 | 0 | 0 | 0 | 0 | 1.63E5 |  |  |  |  |  |  |  |  |  | 1 | 1 | 1 | 0 | 0 | 0 | 0 | 0 | 0 | 0 | 0 | 0 | N | 51235 | SVP1-like protein 2 OS=Saccharomyces cerevisiae (strain ATCC 204508 / S288c) OX=559292 GN=HSV2 PE=1 SV=1 |
| 84 | 275 | P37398\|VIV\_ORYSJ | 42.69 | 2 | 0 | 0 | 0 | 0 | 0 | 0 | 0 | 0 | 0 | 2 |  |  |  |  |  |  |  |  |  | 6.52E5 | 1 | 1 | 0 | 0 | 0 | 0 | 0 | 0 | 0 | 0 | 0 | 1 | N | 75802 | B3 domain-containing protein VP1 OS=Oryza sativa subsp. japonica OX=39947 GN=VP1 PE=1 SV=3 |
| 98 | 525 | Q9LQU4\|PCR2\_ARATH | 42.21 | 6 | 0 | 0 | 0 | 0 | 0 | 6 | 0 | 0 | 0 | 0 |  |  |  |  |  | 1.41E6 |  |  |  |  | 1 | 1 | 0 | 0 | 0 | 0 | 0 | 1 | 0 | 0 | 0 | 0 | Y | 16742 | Protein PLANT CADMIUM RESISTANCE 2 OS=Arabidopsis thaliana OX=3702 GN=PCR2 PE=1 SV=1 |
| total 78 proteins |  |  |  |  |  |  |  |  |  |  |  |  |  |  |  |  |  |  |  |  |  |  |  |  |  |  |  |  |  |  |  |  |  |  |  |  |  |  |  |
| --- | --- | --- | --- | --- | --- | --- | --- | --- | --- | --- | --- | --- | --- | --- | --- | --- | --- | --- | --- | --- | --- | --- | --- | --- | --- | --- | --- | --- | --- | --- | --- | --- | --- | --- | --- | --- | --- | --- | --- |

  

P01391|3L21\_NAJKA

back to list

  

| Protein Coverage
| Supporting Peptides
|

Protein Coverage:

Supporting Peptides:

| Peptide | Uniq | -10lgP | Mass | Length | ppm | m/z | z | RT | Fraction | Scan | Source File | Area F4 | Area F1 | Area F10 | Area F2 | Area F3 | Area F5 | Area F6 | Area F7 | Area F8 | Area F9 | #Feature | #Feature F4 | #Feature F1 | #Feature F10 | #Feature F2 | #Feature F3 | #Feature F5 | #Feature F6 | #Feature F7 | #Feature F8 | #Feature F9 | Start | End | PTM |
| --- | --- | --- | --- | --- | --- | --- | --- | --- | --- | --- | --- | --- | --- | --- | --- | --- | --- | --- | --- | --- | --- | --- | --- | --- | --- | --- | --- | --- | --- | --- | --- | --- | --- | --- | --- |
| K.TGVDIQC(+57.02)C(+57.02)STDNC(+57.02)NPFPTRK.R | Y | 116.62 | 2369.0198 | 20 | 0.0 | 790.6805 | 3 | 26.78 | 6 | F6:9413 | 27122019\_RID\_1315\_NaNaWb\_F5.raw | 1.75E7 |  |  |  |  | 2.41E7 |  |  |  |  | 6 | 5 | 0 | 0 | 0 | 0 | 1 | 0 | 0 | 0 | 0 | 50 | 69 | Carbamidomethylation |
| R.C(+57.02)FITPDITSKDC(+57.02)PNGHVC(+57.02)YTK.T | N | 115.92 | 2512.1184 | 21 | 0.4 | 838.3804 | 3 | 28.23 | 6 | F6:10384 | 27122019\_RID\_1315\_NaNaWb\_F5.raw |  |  | 3.52E6 |  |  | 5.93E7 |  |  | 5.37E5 | 9.5E5 | 9 | 0 | 0 | 2 | 0 | 0 | 4 | 0 | 0 | 1 | 2 | 3 | 23 | Carbamidomethylation |
| K.TGVDIQC(+57.02)C(+57.02)STDNC(+57.02)NPFPTR.K | Y | 113.29 | 2240.9248 | 19 | -0.9 | 1121.4686 | 2 | 35.11 | 6 | F6:15031 | 27122019\_RID\_1315\_NaNaWb\_F5.raw | 8.13E9 |  | 2.51E8 |  | 1.62E8 | 1.16E10 | 3.43E8 | 1.17E8 |  | 8.65E9 | 100 | 50 | 0 | 2 | 0 | 1 | 24 | 2 | 1 | 0 | 20 | 50 | 68 | Carbamidomethylation |
| R.VDLGC(+57.02)AATC(+57.02)PTVK.T | N | 107.87 | 1390.6584 | 13 | 0.4 | 696.3368 | 2 | 25.60 | 6 | F6:8613 | 27122019\_RID\_1315\_NaNaWb\_F5.raw | 1.71E8 |  |  |  |  | 6.5E9 |  | 0 |  | 1.36E8 | 34 | 4 | 0 | 0 | 0 | 0 | 27 | 0 | 1 | 0 | 2 | 37 | 49 | Carbamidomethylation |
| V.KTGVDIQC(+57.02)C(+57.02)STDNC(+57.02)NPFPTR.K | Y | 97.23 | 2369.0198 | 20 | -0.6 | 790.6801 | 3 | 26.49 | 6 | F6:9169 | 27122019\_RID\_1315\_NaNaWb\_F5.raw |  |  |  |  |  | 4.88E6 |  |  |  | 2.34E6 | 2 | 0 | 0 | 0 | 0 | 0 | 1 | 0 | 0 | 0 | 1 | 49 | 68 | Carbamidomethylation |
| Q.C(+57.02)C(+57.02)STDNC(+57.02)NPFPTR.K | N | 93.32 | 1627.6178 | 13 | 0.4 | 814.8165 | 2 | 21.63 | 6 | F6:6170 | 27122019\_RID\_1315\_NaNaWb\_F5.raw | 2.89E8 | 4.91E6 |  | 1.39E7 | 3.67E6 | 5.21E7 |  |  |  | 5.46E7 | 24 | 15 | 1 | 0 | 1 | 1 | 4 | 0 | 0 | 0 | 2 | 56 | 68 | Carbamidomethylation |
| R.C(+57.02)FITPDITSK.D | N | 88.74 | 1180.5798 | 10 | 0.6 | 591.2975 | 2 | 32.45 | 6 | F6:13205 | 27122019\_RID\_1315\_NaNaWb\_F5.raw | 8.21E9 |  | 2.41E8 |  | 8.76E5 | 1.01E10 | 3.52E8 | 2.64E8 | 7.65E7 | 4.99E9 | 127 | 62 | 0 | 2 | 0 | 1 | 40 | 2 | 1 | 2 | 17 | 3 | 12 | Carbamidomethylation |
| C.C(+57.02)STDNC(+57.02)NPFPTR.K | N | 86.21 | 1467.5872 | 12 | 0.1 | 734.8009 | 2 | 21.30 | 6 | F6:6148 | 27122019\_RID\_1315\_NaNaWb\_F5.raw | 1.04E8 | 2.26E6 |  |  |  | 4.53E7 |  |  |  |  | 10 | 5 | 1 | 0 | 0 | 0 | 4 | 0 | 0 | 0 | 0 | 57 | 68 | Carbamidomethylation |
| D.IQC(+57.02)C(+57.02)STDNC(+57.02)NPFPTR.K | Y | 84.42 | 1868.7604 | 15 | 0.0 | 935.3875 | 2 | 34.34 | 1 | F1:14391 | 09012020\_NaNaWb\_F4.raw | 8.48E7 |  |  | 3.43E8 |  | 1.4E5 |  |  |  |  | 12 | 7 | 0 | 0 | 3 | 0 | 2 | 0 | 0 | 0 | 0 | 54 | 68 | Carbamidomethylation |
| IRC(+57.02)FITPDITSKDC(+57.02)PNGHVC(+57.02)YTK.T | N | 83.77 | 2781.3037 | 23 | 0.7 | 557.2684 | 5 | 25.81 | 6 | F6:8733 | 27122019\_RID\_1315\_NaNaWb\_F5.raw |  |  |  |  |  | 3.8E6 |  |  |  |  | 2 | 0 | 0 | 0 | 0 | 0 | 2 | 0 | 0 | 0 | 0 | 1 | 23 | Carbamidomethylation |
| C.STDNC(+57.02)NPFPTR.K | N | 81.69 | 1307.5564 | 11 | 0.6 | 654.7859 | 2 | 20.44 | 6 | F6:5588 | 27122019\_RID\_1315\_NaNaWb\_F5.raw | 8.16E8 |  |  |  |  | 7.73E8 |  |  |  | 7.53E8 | 17 | 7 | 0 | 0 | 0 | 0 | 7 | 0 | 0 | 0 | 3 | 58 | 68 | Carbamidomethylation |
| I.QC(+57.02)C(+57.02)STDNC(+57.02)NPFPTR.K | Y | 68.60 | 1755.6764 | 14 | 0.5 | 878.8459 | 2 | 21.56 | 6 | F6:6172 | 27122019\_RID\_1315\_NaNaWb\_F5.raw |  |  |  |  |  | 2.21E4 |  |  |  |  | 1 | 0 | 0 | 0 | 0 | 0 | 1 | 0 | 0 | 0 | 0 | 55 | 68 | Carbamidomethylation |
| S.TDNC(+57.02)NPFPTR.K | N | 68.43 | 1220.5244 | 10 | -0.5 | 611.2692 | 2 | 20.77 | 6 | F6:5754 | 27122019\_RID\_1315\_NaNaWb\_F5.raw | 1.36E7 |  |  |  |  | 3.22E6 |  |  |  | 1.64E7 | 4 | 2 | 0 | 0 | 0 | 0 | 1 | 0 | 0 | 0 | 1 | 59 | 68 | Carbamidomethylation |
| R.C(+57.02)FITPDITSKD.C | N | 64.39 | 1295.6067 | 11 | -3.3 | 648.8085 | 2 | 48.18 | 1 | F1:23773 | 09012020\_NaNaWb\_F4.raw | 2.51E6 |  |  |  |  |  |  |  |  |  | 1 | 1 | 0 | 0 | 0 | 0 | 0 | 0 | 0 | 0 | 0 | 3 | 13 | Carbamidomethylation |
| R.GKRVDLGC(+57.02)AATC(+57.02)PTVK.T | N | 61.52 | 1731.8760 | 16 | -0.3 | 578.2991 | 3 | 12.32 | 6 | F6:2146 | 27122019\_RID\_1315\_NaNaWb\_F5.raw |  |  |  |  |  | 0 |  |  |  |  | 1 | 0 | 0 | 0 | 0 | 0 | 1 | 0 | 0 | 0 | 0 | 34 | 49 | Carbamidomethylation |
| R.VDLGC(+57.02)AATC(+57.02)PTV.K | N | 58.41 | 1262.5635 | 12 | 0.1 | 632.2891 | 2 | 52.53 | 1 | F1:26732 | 09012020\_NaNaWb\_F4.raw | 3.81E7 |  |  |  |  |  |  |  |  |  | 2 | 2 | 0 | 0 | 0 | 0 | 0 | 0 | 0 | 0 | 0 | 37 | 48 | Carbamidomethylation |
| V.DIQC(+57.02)C(+57.02)STDNC(+57.02)NPFPTR.K | Y | 58.32 | 1983.7874 | 16 | 1.2 | 992.9021 | 2 | 41.60 | 1 | F1:18935 | 09012020\_NaNaWb\_F4.raw | 5.16E5 |  | 0 |  | 6.87E7 |  |  |  |  |  | 3 | 1 | 0 | 1 | 0 | 1 | 0 | 0 | 0 | 0 | 0 | 53 | 68 | Carbamidomethylation |
| IRC(+57.02)FITPDITSK.D | N | 57.96 | 1449.7650 | 12 | 0.6 | 484.2626 | 3 | 41.31 | 1 | F1:18707 | 09012020\_NaNaWb\_F4.raw | 3.82E6 |  |  |  |  | 1.09E7 |  |  |  |  | 4 | 1 | 0 | 0 | 0 | 0 | 3 | 0 | 0 | 0 | 0 | 1 | 12 | Carbamidomethylation |
| K.TGVDIQC(+57.02)C(+57.02)STDN.C | Y | 57.34 | 1368.5286 | 12 | -0.1 | 685.2715 | 2 | 23.95 | 6 | F6:7516 | 27122019\_RID\_1315\_NaNaWb\_F5.raw | 3.81E6 |  |  |  |  | 5.15E6 |  |  |  |  | 3 | 1 | 0 | 0 | 0 | 0 | 2 | 0 | 0 | 0 | 0 | 50 | 61 | Carbamidomethylation |
| T.DNC(+57.02)NPFPTR.K | N | 56.81 | 1119.4767 | 9 | -0.5 | 560.7454 | 2 | 23.23 | 10 | F10:4636 | 27122019\_RID\_1315\_NaNaWb\_F9.raw | 6.62E6 |  |  |  |  |  |  |  |  | 2.34E6 | 3 | 2 | 0 | 0 | 0 | 0 | 0 | 0 | 0 | 0 | 1 | 60 | 68 | Carbamidomethylation |
| K.TGVDIQC(+57.02)C(+57.02).S | N | 55.08 | 951.3790 | 8 | 0.0 | 476.6968 | 2 | 24.90 | 6 | F6:8460 | 27122019\_RID\_1315\_NaNaWb\_F5.raw | 3.45E7 |  |  |  |  | 6.39E7 |  |  |  |  | 5 | 2 | 0 | 0 | 0 | 0 | 3 | 0 | 0 | 0 | 0 | 50 | 57 | Carbamidomethylation |
| K.TGVDIQC(+57.02)C(+57.02)STD.N | N | 53.50 | 1254.4857 | 11 | -0.2 | 628.2500 | 2 | 33.47 | 1 | F1:13928 | 09012020\_NaNaWb\_F4.raw | 1.33E8 |  |  |  |  |  |  |  |  |  | 2 | 2 | 0 | 0 | 0 | 0 | 0 | 0 | 0 | 0 | 0 | 50 | 60 | Carbamidomethylation |
| K.DC(+57.02)PNGHVC(+57.02)YTK.T | N | 52.39 | 1349.5493 | 11 | -0.1 | 450.8570 | 3 | 12.27 | 3 | F3:2057 | 27122019\_RID\_1315\_NaNaWb\_F10.raw | 4.99E5 |  | 0 |  |  |  |  | 0 |  | 0 | 4 | 1 | 0 | 1 | 0 | 0 | 0 | 0 | 1 | 0 | 1 | 13 | 23 | Carbamidomethylation |
| N.GHVC(+57.02)YTK.T | N | 51.73 | 863.3960 | 7 | 0.3 | 432.7054 | 2 | 12.44 | 1 | F1:2007 | 09012020\_NaNaWb\_F4.raw | 1.01E5 |  |  |  |  |  |  |  |  |  | 1 | 1 | 0 | 0 | 0 | 0 | 0 | 0 | 0 | 0 | 0 | 17 | 23 | Carbamidomethylation |
| D.LGC(+57.02)AATC(+57.02)PTVK.T | N | 50.42 | 1176.5631 | 11 | -0.2 | 589.2887 | 2 | 12.76 | 6 | F6:2510 | 27122019\_RID\_1315\_NaNaWb\_F5.raw |  |  |  |  |  | 1.98E6 |  |  |  |  | 2 | 0 | 0 | 0 | 0 | 0 | 2 | 0 | 0 | 0 | 0 | 39 | 49 | Carbamidomethylation |
| K.DC(+57.02)PNGHVC(+57.02)Y.T | N | 47.20 | 1120.4066 | 9 | 0.5 | 561.2109 | 2 | 16.66 | 6 | F6:3831 | 27122019\_RID\_1315\_NaNaWb\_F5.raw |  |  |  |  |  | 1.39E8 |  |  |  |  | 3 | 0 | 0 | 0 | 0 | 0 | 3 | 0 | 0 | 0 | 0 | 13 | 21 | Carbamidomethylation |
| K.TGVDIQC(+57.02)C(+57.02)S.T | N | 46.72 | 1038.4110 | 9 | 1.0 | 520.2133 | 2 | 31.11 | 1 | F1:12168 | 09012020\_NaNaWb\_F4.raw | 2.3E6 |  |  |  |  |  |  |  |  |  | 1 | 1 | 0 | 0 | 0 | 0 | 0 | 0 | 0 | 0 | 0 | 50 | 58 | Carbamidomethylation |
| D.NC(+57.02)NPFPTR.K | N | 46.38 | 1004.4498 | 8 | 0.2 | 503.2322 | 2 | 25.51 | 1 | F1:8810 | 09012020\_NaNaWb\_F4.raw | 0 |  |  |  |  |  |  |  |  |  | 2 | 2 | 0 | 0 | 0 | 0 | 0 | 0 | 0 | 0 | 0 | 61 | 68 | Carbamidomethylation |
| K.RVDLGC(+57.02)AATC(+57.02)PTVK.T | N | 45.12 | 1546.7595 | 14 | 0.6 | 516.5941 | 3 | 19.45 | 6 | F6:4933 | 27122019\_RID\_1315\_NaNaWb\_F5.raw |  |  |  |  |  | 3.17E7 | 7.72E5 |  |  |  | 3 | 0 | 0 | 0 | 0 | 0 | 2 | 1 | 0 | 0 | 0 | 36 | 49 | Carbamidomethylation |
| C.FITPDITSK.D | N | 43.60 | 1020.5491 | 9 | -0.5 | 511.2816 | 2 | 31.63 | 6 | F6:12528 | 27122019\_RID\_1315\_NaNaWb\_F5.raw | 8.4E7 |  |  |  |  | 8.32E6 |  |  |  |  | 2 | 1 | 0 | 0 | 0 | 0 | 1 | 0 | 0 | 0 | 0 | 4 | 12 |  |
| R.VDLGC(+57.02)AATC(+57.02)PT.V | N | 42.06 | 1163.4951 | 11 | 0.7 | 582.7552 | 2 | 40.40 | 1 | F1:18369 | 09012020\_NaNaWb\_F4.raw | 5.03E7 |  |  |  |  |  |  |  |  |  | 2 | 2 | 0 | 0 | 0 | 0 | 0 | 0 | 0 | 0 | 0 | 37 | 47 | Carbamidomethylation |
| R.C(+57.02)FITPDITS.K | N | 41.76 | 1052.4849 | 9 | -0.6 | 527.2494 | 2 | 63.41 | 1 | F1:34301 | 09012020\_NaNaWb\_F4.raw | 1.5E7 |  |  |  |  |  |  |  |  |  | 1 | 1 | 0 | 0 | 0 | 0 | 0 | 0 | 0 | 0 | 0 | 3 | 11 | Carbamidomethylation |
| F.ITPDITSK.D | N | 41.58 | 873.4807 | 8 | 0.6 | 437.7479 | 2 | 16.37 | 6 | F6:3716 | 27122019\_RID\_1315\_NaNaWb\_F5.raw |  |  |  |  |  | 1.43E7 |  |  |  |  | 1 | 0 | 0 | 0 | 0 | 0 | 1 | 0 | 0 | 0 | 0 | 5 | 12 |  |
| I.RC(+57.02)FITPDITSK.D | N | 41.24 | 1336.6809 | 11 | -0.1 | 669.3477 | 2 | 25.02 | 6 | F6:8274 | 27122019\_RID\_1315\_NaNaWb\_F5.raw |  |  |  |  |  | 4.51E5 |  |  |  |  | 1 | 0 | 0 | 0 | 0 | 0 | 1 | 0 | 0 | 0 | 0 | 2 | 12 | Carbamidomethylation |
| total 34 peptides |  |  |  |  |  |  |  |  |  |  |  |  |  |  |  |  |  |  |  |  |  |  |  |  |  |  |  |  |  |  |  |  |  |  |  |
| --- | --- | --- | --- | --- | --- | --- | --- | --- | --- | --- | --- | --- | --- | --- | --- | --- | --- | --- | --- | --- | --- | --- | --- | --- | --- | --- | --- | --- | --- | --- | --- | --- | --- | --- | --- |

P25668|3L21\_NAJNA

back to list

  

| Protein Coverage
| Supporting Peptides
|

Protein Coverage:

Supporting Peptides:

| Peptide | Uniq | -10lgP | Mass | Length | ppm | m/z | z | RT | Fraction | Scan | Source File | Area F4 | Area F1 | Area F10 | Area F2 | Area F3 | Area F5 | Area F6 | Area F7 | Area F8 | Area F9 | #Feature | #Feature F4 | #Feature F1 | #Feature F10 | #Feature F2 | #Feature F3 | #Feature F5 | #Feature F6 | #Feature F7 | #Feature F8 | #Feature F9 | Start | End | PTM |
| --- | --- | --- | --- | --- | --- | --- | --- | --- | --- | --- | --- | --- | --- | --- | --- | --- | --- | --- | --- | --- | --- | --- | --- | --- | --- | --- | --- | --- | --- | --- | --- | --- | --- | --- | --- |
| R.VDLGC(+57.02)AATC(+57.02)PTVR.T | N | 117.03 | 1418.6646 | 13 | 0.4 | 710.3398 | 2 | 26.59 | 6 | F6:9379 | 27122019\_RID\_1315\_NaNaWb\_F5.raw | 1.34E10 |  | 1.7E8 |  |  | 7.17E9 | 2.25E8 | 2.34E8 | 5.91E7 | 8.97E9 | 166 | 111 | 0 | 3 | 0 | 0 | 29 | 4 | 3 | 1 | 15 | 37 | 49 | Carbamidomethylation |
| R.C(+57.02)FITPDITSKDC(+57.02)PNGHVC(+57.02)YTK.T | N | 115.92 | 2512.1184 | 21 | 0.4 | 838.3804 | 3 | 28.23 | 6 | F6:10384 | 27122019\_RID\_1315\_NaNaWb\_F5.raw |  |  | 3.52E6 |  |  | 5.93E7 |  |  | 5.37E5 | 9.5E5 | 9 | 0 | 0 | 2 | 0 | 0 | 4 | 0 | 0 | 1 | 2 | 3 | 23 | Carbamidomethylation |
| R.C(+57.02)FITPDITSK.D | N | 88.74 | 1180.5798 | 10 | 0.6 | 591.2975 | 2 | 32.45 | 6 | F6:13205 | 27122019\_RID\_1315\_NaNaWb\_F5.raw | 8.21E9 |  | 2.41E8 |  | 8.76E5 | 1.01E10 | 3.52E8 | 2.64E8 | 7.65E7 | 4.99E9 | 127 | 62 | 0 | 2 | 0 | 1 | 40 | 2 | 1 | 2 | 17 | 3 | 12 | Carbamidomethylation |
| K.RVDLGC(+57.02)AATC(+57.02)PTVR.T | N | 88.05 | 1574.7657 | 14 | -0.2 | 788.3900 | 2 | 28.64 | 1 | F1:10481 | 09012020\_NaNaWb\_F4.raw | 8.3E8 |  |  |  |  | 3.35E7 | 4.95E6 |  |  | 2.27E8 | 26 | 21 | 0 | 0 | 0 | 0 | 2 | 1 | 0 | 0 | 2 | 36 | 49 | Carbamidomethylation |
| IRC(+57.02)FITPDITSKDC(+57.02)PNGHVC(+57.02)YTK.T | N | 83.77 | 2781.3037 | 23 | 0.7 | 557.2684 | 5 | 25.81 | 6 | F6:8733 | 27122019\_RID\_1315\_NaNaWb\_F5.raw |  |  |  |  |  | 3.8E6 |  |  |  |  | 2 | 0 | 0 | 0 | 0 | 0 | 2 | 0 | 0 | 0 | 0 | 1 | 23 | Carbamidomethylation |
| R.GKRVDLGC(+57.02)AATC(+57.02)PTVR.T | N | 79.55 | 1759.8821 | 16 | -0.2 | 587.6345 | 3 | 12.39 | 6 | F6:2196 | 27122019\_RID\_1315\_NaNaWb\_F5.raw | 2.69E7 |  |  |  |  | 5.44E6 |  |  |  |  | 6 | 3 | 0 | 0 | 0 | 0 | 3 | 0 | 0 | 0 | 0 | 34 | 49 | Carbamidomethylation |
| R.C(+57.02)FITPDITSKD.C | N | 64.39 | 1295.6067 | 11 | -3.3 | 648.8085 | 2 | 48.18 | 1 | F1:23773 | 09012020\_NaNaWb\_F4.raw | 2.51E6 |  |  |  |  |  |  |  |  |  | 1 | 1 | 0 | 0 | 0 | 0 | 0 | 0 | 0 | 0 | 0 | 3 | 13 | Carbamidomethylation |
| D.LGC(+57.02)AATC(+57.02)PTVR.T | N | 62.31 | 1204.5692 | 11 | 0.2 | 603.2920 | 2 | 15.68 | 6 | F6:3348 | 27122019\_RID\_1315\_NaNaWb\_F5.raw | 7.09E6 |  |  |  |  | 2.2E6 |  |  |  |  | 3 | 2 | 0 | 0 | 0 | 0 | 1 | 0 | 0 | 0 | 0 | 39 | 49 | Carbamidomethylation |
| R.VDLGC(+57.02)AATC(+57.02)PTV.R | N | 58.41 | 1262.5635 | 12 | 0.1 | 632.2891 | 2 | 52.53 | 1 | F1:26732 | 09012020\_NaNaWb\_F4.raw | 3.81E7 |  |  |  |  |  |  |  |  |  | 2 | 2 | 0 | 0 | 0 | 0 | 0 | 0 | 0 | 0 | 0 | 37 | 48 | Carbamidomethylation |
| IRC(+57.02)FITPDITSK.D | N | 57.96 | 1449.7650 | 12 | 0.6 | 484.2626 | 3 | 41.31 | 1 | F1:18707 | 09012020\_NaNaWb\_F4.raw | 3.82E6 |  |  |  |  | 1.09E7 |  |  |  |  | 4 | 1 | 0 | 0 | 0 | 0 | 3 | 0 | 0 | 0 | 0 | 1 | 12 | Carbamidomethylation |
| K.TWC(+57.02)DGFC(+57.02)SIR.G | Y | 55.58 | 1300.5328 | 10 | 0.5 | 651.2740 | 2 | 37.69 | 6 | F6:16628 | 27122019\_RID\_1315\_NaNaWb\_F5.raw | 7.86E7 |  | 9.9E7 |  |  | 1.01E10 |  |  | 1.35E7 |  | 19 | 5 | 0 | 1 | 0 | 0 | 12 | 0 | 0 | 1 | 0 | 24 | 33 | Carbamidomethylation |
| R.TGVDIQC(+57.02)C(+57.02).S | N | 55.08 | 951.3790 | 8 | 0.0 | 476.6968 | 2 | 24.90 | 6 | F6:8460 | 27122019\_RID\_1315\_NaNaWb\_F5.raw | 3.45E7 |  |  |  |  | 6.39E7 |  |  |  |  | 5 | 2 | 0 | 0 | 0 | 0 | 3 | 0 | 0 | 0 | 0 | 50 | 57 | Carbamidomethylation |
| R.TGVDIQC(+57.02)C(+57.02)STD.D | N | 53.50 | 1254.4857 | 11 | -0.2 | 628.2500 | 2 | 33.47 | 1 | F1:13928 | 09012020\_NaNaWb\_F4.raw | 1.33E8 |  |  |  |  |  |  |  |  |  | 2 | 2 | 0 | 0 | 0 | 0 | 0 | 0 | 0 | 0 | 0 | 50 | 60 | Carbamidomethylation |
| K.DC(+57.02)PNGHVC(+57.02)YTK.T | N | 52.39 | 1349.5493 | 11 | -0.1 | 450.8570 | 3 | 12.27 | 3 | F3:2057 | 27122019\_RID\_1315\_NaNaWb\_F10.raw | 4.99E5 |  | 0 |  |  |  |  | 0 |  | 0 | 4 | 1 | 0 | 1 | 0 | 0 | 0 | 0 | 1 | 0 | 1 | 13 | 23 | Carbamidomethylation |
| N.GHVC(+57.02)YTK.T | N | 51.73 | 863.3960 | 7 | 0.3 | 432.7054 | 2 | 12.44 | 1 | F1:2007 | 09012020\_NaNaWb\_F4.raw | 1.01E5 |  |  |  |  |  |  |  |  |  | 1 | 1 | 0 | 0 | 0 | 0 | 0 | 0 | 0 | 0 | 0 | 17 | 23 | Carbamidomethylation |
| K.DC(+57.02)PNGHVC(+57.02)Y.T | N | 47.20 | 1120.4066 | 9 | 0.5 | 561.2109 | 2 | 16.66 | 6 | F6:3831 | 27122019\_RID\_1315\_NaNaWb\_F5.raw |  |  |  |  |  | 1.39E8 |  |  |  |  | 3 | 0 | 0 | 0 | 0 | 0 | 3 | 0 | 0 | 0 | 0 | 13 | 21 | Carbamidomethylation |
| R.TGVDIQC(+57.02)C(+57.02)S.T | N | 46.72 | 1038.4110 | 9 | 1.0 | 520.2133 | 2 | 31.11 | 1 | F1:12168 | 09012020\_NaNaWb\_F4.raw | 2.3E6 |  |  |  |  |  |  |  |  |  | 1 | 1 | 0 | 0 | 0 | 0 | 0 | 0 | 0 | 0 | 0 | 50 | 58 | Carbamidomethylation |
| C.DGFC(+57.02)SIR.G | Y | 45.95 | 853.3752 | 7 | 0.6 | 427.6952 | 2 | 22.30 | 6 | F6:6563 | 27122019\_RID\_1315\_NaNaWb\_F5.raw |  |  |  |  |  | 7.69E6 |  |  |  |  | 1 | 0 | 0 | 0 | 0 | 0 | 1 | 0 | 0 | 0 | 0 | 27 | 33 | Carbamidomethylation |
| C.FITPDITSK.D | N | 43.60 | 1020.5491 | 9 | -0.5 | 511.2816 | 2 | 31.63 | 6 | F6:12528 | 27122019\_RID\_1315\_NaNaWb\_F5.raw | 8.4E7 |  |  |  |  | 8.32E6 |  |  |  |  | 2 | 1 | 0 | 0 | 0 | 0 | 1 | 0 | 0 | 0 | 0 | 4 | 12 |  |
| R.TGVDIQC(+57.02)C(+57.02)STDDC(+57.02)DPFPTR.K | N | 42.96 | 2242.8928 | 19 | 8.9 | 1122.4636 | 2 | 37.32 | 8 | F8:9056 | 27122019\_RID\_1315\_NaNaWb\_F7.raw |  |  |  |  |  |  |  | 0 |  |  | 1 | 0 | 0 | 0 | 0 | 0 | 0 | 0 | 1 | 0 | 0 | 50 | 68 | Carbamidomethylation |
| R.VDLGC(+57.02)AATC(+57.02)PT.V | N | 42.06 | 1163.4951 | 11 | 0.7 | 582.7552 | 2 | 40.40 | 1 | F1:18369 | 09012020\_NaNaWb\_F4.raw | 5.03E7 |  |  |  |  |  |  |  |  |  | 2 | 2 | 0 | 0 | 0 | 0 | 0 | 0 | 0 | 0 | 0 | 37 | 47 | Carbamidomethylation |
| R.C(+57.02)FITPDITS.K | N | 41.76 | 1052.4849 | 9 | -0.6 | 527.2494 | 2 | 63.41 | 1 | F1:34301 | 09012020\_NaNaWb\_F4.raw | 1.5E7 |  |  |  |  |  |  |  |  |  | 1 | 1 | 0 | 0 | 0 | 0 | 0 | 0 | 0 | 0 | 0 | 3 | 11 | Carbamidomethylation |
| F.ITPDITSK.D | N | 41.58 | 873.4807 | 8 | 0.6 | 437.7479 | 2 | 16.37 | 6 | F6:3716 | 27122019\_RID\_1315\_NaNaWb\_F5.raw |  |  |  |  |  | 1.43E7 |  |  |  |  | 1 | 0 | 0 | 0 | 0 | 0 | 1 | 0 | 0 | 0 | 0 | 5 | 12 |  |
| I.RC(+57.02)FITPDITSK.D | N | 41.24 | 1336.6809 | 11 | -0.1 | 669.3477 | 2 | 25.02 | 6 | F6:8274 | 27122019\_RID\_1315\_NaNaWb\_F5.raw |  |  |  |  |  | 4.51E5 |  |  |  |  | 1 | 0 | 0 | 0 | 0 | 0 | 1 | 0 | 0 | 0 | 0 | 2 | 12 | Carbamidomethylation |
| total 24 peptides |  |  |  |  |  |  |  |  |  |  |  |  |  |  |  |  |  |  |  |  |  |  |  |  |  |  |  |  |  |  |  |  |  |  |  |
| --- | --- | --- | --- | --- | --- | --- | --- | --- | --- | --- | --- | --- | --- | --- | --- | --- | --- | --- | --- | --- | --- | --- | --- | --- | --- | --- | --- | --- | --- | --- | --- | --- | --- | --- | --- |

P25672|3L24\_NAJNA

back to list

  

| Protein Coverage
| Supporting Peptides
|

Protein Coverage:

Supporting Peptides:

| Peptide | Uniq | -10lgP | Mass | Length | ppm | m/z | z | RT | Fraction | Scan | Source File | Area F4 | Area F1 | Area F10 | Area F2 | Area F3 | Area F5 | Area F6 | Area F7 | Area F8 | Area F9 | #Feature | #Feature F4 | #Feature F1 | #Feature F10 | #Feature F2 | #Feature F3 | #Feature F5 | #Feature F6 | #Feature F7 | #Feature F8 | #Feature F9 | Start | End | PTM |
| --- | --- | --- | --- | --- | --- | --- | --- | --- | --- | --- | --- | --- | --- | --- | --- | --- | --- | --- | --- | --- | --- | --- | --- | --- | --- | --- | --- | --- | --- | --- | --- | --- | --- | --- | --- |
| R.C(+57.02)FITPDITSKDC(+57.02)PNGHVC(+57.02)YTK.T | N | 115.92 | 2512.1184 | 21 | 0.4 | 838.3804 | 3 | 28.23 | 6 | F6:10384 | 27122019\_RID\_1315\_NaNaWb\_F5.raw |  |  | 3.52E6 |  |  | 5.93E7 |  |  | 5.37E5 | 9.5E5 | 9 | 0 | 0 | 2 | 0 | 0 | 4 | 0 | 0 | 1 | 2 | 3 | 23 | Carbamidomethylation |
| R.VDLGC(+57.02)AATC(+57.02)PTVK.T | N | 107.87 | 1390.6584 | 13 | 0.4 | 696.3368 | 2 | 25.60 | 6 | F6:8613 | 27122019\_RID\_1315\_NaNaWb\_F5.raw | 1.71E8 |  |  |  |  | 6.5E9 |  | 0 |  | 1.36E8 | 34 | 4 | 0 | 0 | 0 | 0 | 27 | 0 | 1 | 0 | 2 | 37 | 49 | Carbamidomethylation |
| R.C(+57.02)FITPDITSK.D | N | 88.74 | 1180.5798 | 10 | 0.6 | 591.2975 | 2 | 32.45 | 6 | F6:13205 | 27122019\_RID\_1315\_NaNaWb\_F5.raw | 8.21E9 |  | 2.41E8 |  | 8.76E5 | 1.01E10 | 3.52E8 | 2.64E8 | 7.65E7 | 4.99E9 | 127 | 62 | 0 | 2 | 0 | 1 | 40 | 2 | 1 | 2 | 17 | 3 | 12 | Carbamidomethylation |
| IRC(+57.02)FITPDITSKDC(+57.02)PNGHVC(+57.02)YTK.T | N | 83.77 | 2781.3037 | 23 | 0.7 | 557.2684 | 5 | 25.81 | 6 | F6:8733 | 27122019\_RID\_1315\_NaNaWb\_F5.raw |  |  |  |  |  | 3.8E6 |  |  |  |  | 2 | 0 | 0 | 0 | 0 | 0 | 2 | 0 | 0 | 0 | 0 | 1 | 23 | Carbamidomethylation |
| K.TWC(+57.02)DGFC(+57.02)R.I | Y | 72.24 | 1100.4167 | 8 | 1.0 | 551.2162 | 2 | 26.69 | 6 | F6:9272 | 27122019\_RID\_1315\_NaNaWb\_F5.raw |  |  |  |  |  | 5.44E8 |  | 1.6E6 |  | 4.04E6 | 13 | 0 | 0 | 0 | 0 | 0 | 11 | 0 | 1 | 0 | 1 | 24 | 31 | Carbamidomethylation |
| R.GERVDLGC(+57.02)AATC(+57.02)PTVK.T | Y | 69.36 | 1732.8236 | 16 | 0.0 | 578.6152 | 3 | 20.76 | 6 | F6:5720 | 27122019\_RID\_1315\_NaNaWb\_F5.raw |  |  |  |  |  | 3.41E6 |  |  |  |  | 1 | 0 | 0 | 0 | 0 | 0 | 1 | 0 | 0 | 0 | 0 | 34 | 49 | Carbamidomethylation |
| R.C(+57.02)FITPDITSKD.C | N | 64.39 | 1295.6067 | 11 | -3.3 | 648.8085 | 2 | 48.18 | 1 | F1:23773 | 09012020\_NaNaWb\_F4.raw | 2.51E6 |  |  |  |  |  |  |  |  |  | 1 | 1 | 0 | 0 | 0 | 0 | 0 | 0 | 0 | 0 | 0 | 3 | 13 | Carbamidomethylation |
| R.VDLGC(+57.02)AATC(+57.02)PTV.K | N | 58.41 | 1262.5635 | 12 | 0.1 | 632.2891 | 2 | 52.53 | 1 | F1:26732 | 09012020\_NaNaWb\_F4.raw | 3.81E7 |  |  |  |  |  |  |  |  |  | 2 | 2 | 0 | 0 | 0 | 0 | 0 | 0 | 0 | 0 | 0 | 37 | 48 | Carbamidomethylation |
| IRC(+57.02)FITPDITSK.D | N | 57.96 | 1449.7650 | 12 | 0.6 | 484.2626 | 3 | 41.31 | 1 | F1:18707 | 09012020\_NaNaWb\_F4.raw | 3.82E6 |  |  |  |  | 1.09E7 |  |  |  |  | 4 | 1 | 0 | 0 | 0 | 0 | 3 | 0 | 0 | 0 | 0 | 1 | 12 | Carbamidomethylation |
| K.TGVDIQC(+57.02)C(+57.02).S | N | 55.08 | 951.3790 | 8 | 0.0 | 476.6968 | 2 | 24.90 | 6 | F6:8460 | 27122019\_RID\_1315\_NaNaWb\_F5.raw | 3.45E7 |  |  |  |  | 6.39E7 |  |  |  |  | 5 | 2 | 0 | 0 | 0 | 0 | 3 | 0 | 0 | 0 | 0 | 50 | 57 | Carbamidomethylation |
| K.TGVDIQC(+57.02)C(+57.02)STD.D | N | 53.50 | 1254.4857 | 11 | -0.2 | 628.2500 | 2 | 33.47 | 1 | F1:13928 | 09012020\_NaNaWb\_F4.raw | 1.33E8 |  |  |  |  |  |  |  |  |  | 2 | 2 | 0 | 0 | 0 | 0 | 0 | 0 | 0 | 0 | 0 | 50 | 60 | Carbamidomethylation |
| K.DC(+57.02)PNGHVC(+57.02)YTK.T | N | 52.39 | 1349.5493 | 11 | -0.1 | 450.8570 | 3 | 12.27 | 3 | F3:2057 | 27122019\_RID\_1315\_NaNaWb\_F10.raw | 4.99E5 |  | 0 |  |  |  |  | 0 |  | 0 | 4 | 1 | 0 | 1 | 0 | 0 | 0 | 0 | 1 | 0 | 1 | 13 | 23 | Carbamidomethylation |
| N.GHVC(+57.02)YTK.T | N | 51.73 | 863.3960 | 7 | 0.3 | 432.7054 | 2 | 12.44 | 1 | F1:2007 | 09012020\_NaNaWb\_F4.raw | 1.01E5 |  |  |  |  |  |  |  |  |  | 1 | 1 | 0 | 0 | 0 | 0 | 0 | 0 | 0 | 0 | 0 | 17 | 23 | Carbamidomethylation |
| D.LGC(+57.02)AATC(+57.02)PTVK.T | N | 50.42 | 1176.5631 | 11 | -0.2 | 589.2887 | 2 | 12.76 | 6 | F6:2510 | 27122019\_RID\_1315\_NaNaWb\_F5.raw |  |  |  |  |  | 1.98E6 |  |  |  |  | 2 | 0 | 0 | 0 | 0 | 0 | 2 | 0 | 0 | 0 | 0 | 39 | 49 | Carbamidomethylation |
| R.IRGERVDLGC(+57.02)AATC(+57.02)PTVK.T | Y | 48.49 | 2002.0088 | 18 | 0.8 | 501.5099 | 4 | 19.08 | 6 | F6:4760 | 27122019\_RID\_1315\_NaNaWb\_F5.raw |  |  |  |  |  | 1E6 |  |  |  |  | 2 | 0 | 0 | 0 | 0 | 0 | 2 | 0 | 0 | 0 | 0 | 32 | 49 | Carbamidomethylation |
| K.DC(+57.02)PNGHVC(+57.02)Y.T | N | 47.20 | 1120.4066 | 9 | 0.5 | 561.2109 | 2 | 16.66 | 6 | F6:3831 | 27122019\_RID\_1315\_NaNaWb\_F5.raw |  |  |  |  |  | 1.39E8 |  |  |  |  | 3 | 0 | 0 | 0 | 0 | 0 | 3 | 0 | 0 | 0 | 0 | 13 | 21 | Carbamidomethylation |
| K.TGVDIQC(+57.02)C(+57.02)S.T | N | 46.72 | 1038.4110 | 9 | 1.0 | 520.2133 | 2 | 31.11 | 1 | F1:12168 | 09012020\_NaNaWb\_F4.raw | 2.3E6 |  |  |  |  |  |  |  |  |  | 1 | 1 | 0 | 0 | 0 | 0 | 0 | 0 | 0 | 0 | 0 | 50 | 58 | Carbamidomethylation |
| E.RVDLGC(+57.02)AATC(+57.02)PTVK.T | N | 45.12 | 1546.7595 | 14 | 0.6 | 516.5941 | 3 | 19.45 | 6 | F6:4933 | 27122019\_RID\_1315\_NaNaWb\_F5.raw |  |  |  |  |  | 3.17E7 | 7.72E5 |  |  |  | 3 | 0 | 0 | 0 | 0 | 0 | 2 | 1 | 0 | 0 | 0 | 36 | 49 | Carbamidomethylation |
| C.FITPDITSK.D | N | 43.60 | 1020.5491 | 9 | -0.5 | 511.2816 | 2 | 31.63 | 6 | F6:12528 | 27122019\_RID\_1315\_NaNaWb\_F5.raw | 8.4E7 |  |  |  |  | 8.32E6 |  |  |  |  | 2 | 1 | 0 | 0 | 0 | 0 | 1 | 0 | 0 | 0 | 0 | 4 | 12 |  |
| K.TGVDIQC(+57.02)C(+57.02)STDDC(+57.02)DPFPTR.K | N | 42.96 | 2242.8928 | 19 | 8.9 | 1122.4636 | 2 | 37.32 | 8 | F8:9056 | 27122019\_RID\_1315\_NaNaWb\_F7.raw |  |  |  |  |  |  |  | 0 |  |  | 1 | 0 | 0 | 0 | 0 | 0 | 0 | 0 | 1 | 0 | 0 | 50 | 68 | Carbamidomethylation |
| R.VDLGC(+57.02)AATC(+57.02)PT.V | N | 42.06 | 1163.4951 | 11 | 0.7 | 582.7552 | 2 | 40.40 | 1 | F1:18369 | 09012020\_NaNaWb\_F4.raw | 5.03E7 |  |  |  |  |  |  |  |  |  | 2 | 2 | 0 | 0 | 0 | 0 | 0 | 0 | 0 | 0 | 0 | 37 | 47 | Carbamidomethylation |
| R.C(+57.02)FITPDITS.K | N | 41.76 | 1052.4849 | 9 | -0.6 | 527.2494 | 2 | 63.41 | 1 | F1:34301 | 09012020\_NaNaWb\_F4.raw | 1.5E7 |  |  |  |  |  |  |  |  |  | 1 | 1 | 0 | 0 | 0 | 0 | 0 | 0 | 0 | 0 | 0 | 3 | 11 | Carbamidomethylation |
| F.ITPDITSK.D | N | 41.58 | 873.4807 | 8 | 0.6 | 437.7479 | 2 | 16.37 | 6 | F6:3716 | 27122019\_RID\_1315\_NaNaWb\_F5.raw |  |  |  |  |  | 1.43E7 |  |  |  |  | 1 | 0 | 0 | 0 | 0 | 0 | 1 | 0 | 0 | 0 | 0 | 5 | 12 |  |
| I.RC(+57.02)FITPDITSK.D | N | 41.24 | 1336.6809 | 11 | -0.1 | 669.3477 | 2 | 25.02 | 6 | F6:8274 | 27122019\_RID\_1315\_NaNaWb\_F5.raw |  |  |  |  |  | 4.51E5 |  |  |  |  | 1 | 0 | 0 | 0 | 0 | 0 | 1 | 0 | 0 | 0 | 0 | 2 | 12 | Carbamidomethylation |
| total 24 peptides |  |  |  |  |  |  |  |  |  |  |  |  |  |  |  |  |  |  |  |  |  |  |  |  |  |  |  |  |  |  |  |  |  |  |  |
| --- | --- | --- | --- | --- | --- | --- | --- | --- | --- | --- | --- | --- | --- | --- | --- | --- | --- | --- | --- | --- | --- | --- | --- | --- | --- | --- | --- | --- | --- | --- | --- | --- | --- | --- | --- |

P04264|K2C1\_HUMAN

back to list

  

| Protein Coverage
| Supporting Peptides
|

Protein Coverage:

Supporting Peptides:

| Peptide | Uniq | -10lgP | Mass | Length | ppm | m/z | z | RT | Fraction | Scan | Source File | Area F4 | Area F1 | Area F10 | Area F2 | Area F3 | Area F5 | Area F6 | Area F7 | Area F8 | Area F9 | #Feature | #Feature F4 | #Feature F1 | #Feature F10 | #Feature F2 | #Feature F3 | #Feature F5 | #Feature F6 | #Feature F7 | #Feature F8 | #Feature F9 | Start | End | PTM |
| --- | --- | --- | --- | --- | --- | --- | --- | --- | --- | --- | --- | --- | --- | --- | --- | --- | --- | --- | --- | --- | --- | --- | --- | --- | --- | --- | --- | --- | --- | --- | --- | --- | --- | --- | --- |
| K.WELLQQVDTSTR.T | Y | 93.00 | 1474.7416 | 12 | -0.7 | 738.3776 | 2 | 44.69 | 7 | F7:15527 | 27122019\_RID\_1315\_NaNaWb\_F6.raw | 6.47E5 | 1.12E7 | 6.88E5 |  |  | 8.9E5 | 3.86E7 | 1.03E7 | 9.82E6 |  | 7 | 1 | 1 | 1 | 0 | 0 | 1 | 1 | 1 | 1 | 0 | 212 | 223 |  |
| R.THNLEPYFESFINNLR.R | Y | 86.27 | 1992.9694 | 16 | 0.4 | 665.3307 | 3 | 57.75 | 7 | F7:21468 | 27122019\_RID\_1315\_NaNaWb\_F6.raw |  |  |  |  |  |  | 6.01E7 | 9.3E5 |  |  | 3 | 0 | 0 | 0 | 0 | 0 | 0 | 2 | 1 | 0 | 0 | 224 | 239 |  |
| K.YEELQITAGR.H | N | 83.09 | 1178.5931 | 10 | 1.6 | 590.3048 | 2 | 28.00 | 6 | F6:10173 | 27122019\_RID\_1315\_NaNaWb\_F5.raw | 1.63E6 |  |  |  |  | 8.42E5 |  | 1.28E7 | 9.14E6 |  | 5 | 1 | 0 | 0 | 0 | 0 | 1 | 0 | 1 | 2 | 0 | 377 | 386 |  |
| K.QISNLQQSISDAEQR.G | Y | 83.00 | 1715.8438 | 15 | 1.2 | 858.9302 | 2 | 42.50 | 1 | F1:19777 | 09012020\_NaNaWb\_F4.raw | 1.64E6 | 1.1E7 |  |  |  | 1.45E6 | 6.56E6 | 3.51E6 |  |  | 9 | 2 | 2 | 0 | 0 | 0 | 2 | 1 | 2 | 0 | 0 | 418 | 432 |  |
| K.SKAEAESLYQSK.Y | Y | 80.43 | 1339.6619 | 12 | 1.2 | 670.8390 | 2 | 12.86 | 1 | F1:2273 | 09012020\_NaNaWb\_F4.raw | 1.58E5 |  |  |  |  |  |  |  |  |  | 1 | 1 | 0 | 0 | 0 | 0 | 0 | 0 | 0 | 0 | 0 | 365 | 376 |  |
| R.FSSC(+57.02)GGGGGSFGAGGGFGSR.S | Y | 79.57 | 1764.7274 | 20 | -0.8 | 883.3702 | 2 | 28.36 | 6 | F6:10362 | 27122019\_RID\_1315\_NaNaWb\_F5.raw | 4.08E5 |  |  |  |  | 6.65E5 | 2.11E7 |  | 3.65E6 |  | 4 | 1 | 0 | 0 | 0 | 0 | 1 | 1 | 0 | 1 | 0 | 46 | 65 | Carbamidomethylation |
| K.LNDLEDALQQAK.E | Y | 78.87 | 1356.6885 | 12 | 0.1 | 679.3516 | 2 | 49.64 | 1 | F1:24721 | 09012020\_NaNaWb\_F4.raw | 4.26E6 | 1.77E7 |  |  |  |  | 5.32E7 | 5.57E6 | 1.29E7 |  | 6 | 2 | 1 | 0 | 0 | 0 | 0 | 1 | 1 | 1 | 0 | 444 | 455 |  |
| R.FLEQQNQVLQTK.W | N | 77.68 | 1474.7780 | 12 | 0.6 | 738.3967 | 2 | 37.47 | 1 | F1:16185 | 09012020\_NaNaWb\_F4.raw | 5.37E6 |  |  |  |  | 2.57E6 | 6.32E7 |  | 9.44E6 | 3E6 | 6 | 1 | 0 | 0 | 0 | 0 | 1 | 2 | 0 | 1 | 1 | 200 | 211 |  |
| R.SGGGFSSGSAGIINYQR.R | Y | 72.01 | 1656.7855 | 17 | -0.5 | 829.3997 | 2 | 32.22 | 8 | F8:7496 | 27122019\_RID\_1315\_NaNaWb\_F7.raw |  |  |  |  | 1.86E5 | 1.3E5 |  | 2.92E5 |  |  | 3 | 0 | 0 | 0 | 0 | 1 | 1 | 0 | 1 | 0 | 0 | 13 | 29 |  |
| R.SLDLDSIIAEVK.A | N | 71.42 | 1301.7078 | 12 | 0.1 | 651.8612 | 2 | 61.91 | 7 | F7:22999 | 27122019\_RID\_1315\_NaNaWb\_F6.raw | 3.36E5 |  |  |  | 5.38E5 |  | 6.91E7 |  | 2.1E7 | 3.54E6 | 5 | 1 | 0 | 0 | 0 | 1 | 0 | 1 | 0 | 1 | 1 | 344 | 355 |  |
| K.SLNNQFASFIDK.V | Y | 66.63 | 1382.6830 | 12 | 0.6 | 692.3492 | 2 | 59.35 | 1 | F1:31381 | 09012020\_NaNaWb\_F4.raw | 1.4E6 | 1.63E7 |  |  |  | 2.32E6 |  | 1.15E7 |  |  | 4 | 1 | 1 | 0 | 0 | 0 | 1 | 0 | 1 | 0 | 0 | 186 | 197 |  |
| R.NKYEDEINKR.T | N | 64.65 | 1307.6470 | 10 | 0.7 | 436.8899 | 3 | 12.47 | 1 | F1:2039 | 09012020\_NaNaWb\_F4.raw | 5.64E5 |  |  |  |  |  |  |  |  |  | 1 | 1 | 0 | 0 | 0 | 0 | 0 | 0 | 0 | 0 | 0 | 268 | 277 |  |
| K.AEAESLYQSK.Y | Y | 64.13 | 1124.5349 | 10 | 0.1 | 563.2748 | 2 | 20.58 | 1 | F1:5952 | 09012020\_NaNaWb\_F4.raw | 5.01E5 |  |  |  |  | 1.83E5 | 2.96E6 |  |  |  | 3 | 1 | 0 | 0 | 0 | 0 | 1 | 1 | 0 | 0 | 0 | 367 | 376 |  |
| R.TNAENEFVTIK.K | Y | 62.37 | 1264.6299 | 11 | 0.7 | 633.3226 | 2 | 28.73 | 6 | F6:10539 | 27122019\_RID\_1315\_NaNaWb\_F5.raw | 4.88E6 |  | 3.68E6 |  | 2.28E6 | 1.84E6 | 4.64E7 |  | 8.78E6 |  | 6 | 1 | 0 | 1 | 0 | 1 | 1 | 1 | 0 | 1 | 0 | 278 | 288 |  |
| L.NVEIDPEIQK.V | Y | 60.39 | 1183.6084 | 10 | 0.8 | 592.8120 | 2 | 36.02 | 1 | F1:15409 | 09012020\_NaNaWb\_F4.raw | 2.93E6 |  |  |  |  |  |  |  |  |  | 1 | 1 | 0 | 0 | 0 | 0 | 0 | 0 | 0 | 0 | 0 | 166 | 175 |  |
| K.LALDLEIATYR.T | N | 58.31 | 1276.7026 | 11 | 0.6 | 639.3590 | 2 | 52.67 | 7 | F7:18724 | 27122019\_RID\_1315\_NaNaWb\_F6.raw |  |  |  |  |  |  | 3.49E7 |  | 4E6 |  | 3 | 0 | 0 | 0 | 0 | 0 | 0 | 2 | 0 | 1 | 0 | 473 | 483 |  |
| R.DYQELMNTK.L | N | 57.20 | 1140.5121 | 9 | 0.6 | 571.2637 | 2 | 38.16 | 1 | F1:16532 | 09012020\_NaNaWb\_F4.raw | 4.4E5 |  |  |  |  |  |  |  |  |  | 1 | 1 | 0 | 0 | 0 | 0 | 0 | 0 | 0 | 0 | 0 | 464 | 472 |  |
| R.TLLEGEESR.M | Y | 53.68 | 1032.5087 | 9 | 0.0 | 517.2616 | 2 | 17.74 | 7 | F7:4143 | 27122019\_RID\_1315\_NaNaWb\_F6.raw |  |  |  |  |  |  | 1.29E7 |  |  |  | 1 | 0 | 0 | 0 | 0 | 0 | 0 | 1 | 0 | 0 | 0 | 484 | 492 |  |
| R.TNAENEFVTIKK.D | Y | 50.16 | 1392.7249 | 12 | -0.1 | 465.2488 | 3 | 26.75 | 1 | F1:9522 | 09012020\_NaNaWb\_F4.raw | 1.9E5 |  |  |  |  |  |  | 1.18E6 |  |  | 2 | 1 | 0 | 0 | 0 | 0 | 0 | 0 | 1 | 0 | 0 | 278 | 289 |  |
| R.THNLEPYFE.S | Y | 46.86 | 1148.5138 | 9 | -0.1 | 575.2641 | 2 | 44.22 | 1 | F1:20947 | 09012020\_NaNaWb\_F4.raw | 2.6E6 |  |  |  |  |  |  |  |  |  | 1 | 1 | 0 | 0 | 0 | 0 | 0 | 0 | 0 | 0 | 0 | 224 | 232 |  |
| R.SLVNLGGSK.S | Y | 45.53 | 873.4919 | 9 | 0.1 | 437.7533 | 2 | 30.21 | 1 | F1:11766 | 09012020\_NaNaWb\_F4.raw | 1.55E6 |  |  |  |  |  |  |  |  |  | 2 | 2 | 0 | 0 | 0 | 0 | 0 | 0 | 0 | 0 | 0 | 66 | 74 |  |
| Q.FASFIDK.V | N | 45.32 | 826.4225 | 7 | -0.7 | 414.2182 | 2 | 39.06 | 1 | F1:17107 | 09012020\_NaNaWb\_F4.raw | 1.07E6 |  |  |  |  | 1.59E5 |  |  |  |  | 2 | 1 | 0 | 0 | 0 | 0 | 1 | 0 | 0 | 0 | 0 | 191 | 197 |  |
| K.IEISELNR.V | N | 44.99 | 972.5240 | 8 | -0.1 | 487.2692 | 2 | 26.67 | 8 | F8:5869 | 27122019\_RID\_1315\_NaNaWb\_F7.raw |  |  |  |  |  |  |  | 4.09E6 |  |  | 1 | 0 | 0 | 0 | 0 | 0 | 0 | 0 | 1 | 0 | 0 | 396 | 403 |  |
| G.GGGGSFGAGGGFGSR.S | Y | 42.44 | 1226.5428 | 15 | 0.6 | 614.2791 | 2 | 29.32 | 1 | F1:10933 | 09012020\_NaNaWb\_F4.raw | 5.81E4 |  |  |  |  |  |  |  |  |  | 1 | 1 | 0 | 0 | 0 | 0 | 0 | 0 | 0 | 0 | 0 | 51 | 65 |  |
| R.THNLEPYFESF.I | Y | 41.84 | 1382.6143 | 11 | 0.8 | 692.3149 | 2 | 62.79 | 1 | F1:33743 | 09012020\_NaNaWb\_F4.raw | 1.07E5 |  |  |  |  |  |  |  |  |  | 1 | 1 | 0 | 0 | 0 | 0 | 0 | 0 | 0 | 0 | 0 | 224 | 234 |  |
| total 25 peptides |  |  |  |  |  |  |  |  |  |  |  |  |  |  |  |  |  |  |  |  |  |  |  |  |  |  |  |  |  |  |  |  |  |  |  |
| --- | --- | --- | --- | --- | --- | --- | --- | --- | --- | --- | --- | --- | --- | --- | --- | --- | --- | --- | --- | --- | --- | --- | --- | --- | --- | --- | --- | --- | --- | --- | --- | --- | --- | --- | --- |

#CONTAM#K1C10\_HUMAN|

back to list

  

| Protein Coverage
| Supporting Peptides
|

Protein Coverage:

Supporting Peptides:

| Peptide | Uniq | -10lgP | Mass | Length | ppm | m/z | z | RT | Fraction | Scan | Source File | Area F4 | Area F1 | Area F10 | Area F2 | Area F3 | Area F5 | Area F6 | Area F7 | Area F8 | Area F9 | #Feature | #Feature F4 | #Feature F1 | #Feature F10 | #Feature F2 | #Feature F3 | #Feature F5 | #Feature F6 | #Feature F7 | #Feature F8 | #Feature F9 | Start | End | PTM |
| --- | --- | --- | --- | --- | --- | --- | --- | --- | --- | --- | --- | --- | --- | --- | --- | --- | --- | --- | --- | --- | --- | --- | --- | --- | --- | --- | --- | --- | --- | --- | --- | --- | --- | --- | --- |
| R.ALEESNYELEGK.I | Y | 81.22 | 1380.6409 | 12 | -0.1 | 691.3276 | 2 | 23.77 | 7 | F7:6974 | 27122019\_RID\_1315\_NaNaWb\_F6.raw | 2.17E6 |  | 6.55E5 | 1.17E6 |  | 2.52E6 | 2.07E7 | 2.26E6 |  |  | 7 | 2 | 0 | 1 | 1 | 0 | 1 | 1 | 1 | 0 | 0 | 166 | 177 |  |
| K.NQILNLTTDNANILLQIDNAR.L | Y | 78.77 | 2366.2554 | 21 | -0.9 | 1184.1339 | 2 | 63.33 | 7 | F7:23654 | 27122019\_RID\_1315\_NaNaWb\_F6.raw |  |  |  |  |  |  | 1.91E7 |  | 1.08E6 |  | 5 | 0 | 0 | 0 | 0 | 0 | 0 | 2 | 0 | 3 | 0 | 208 | 228 |  |
| R.SQYEQLAEQNR.K | Y | 78.49 | 1364.6321 | 11 | 0.6 | 683.3237 | 2 | 18.04 | 6 | F6:4236 | 27122019\_RID\_1315\_NaNaWb\_F5.raw | 1.62E6 |  |  |  |  | 1.05E6 | 1.96E7 |  | 3.33E6 |  | 4 | 1 | 0 | 0 | 0 | 0 | 1 | 1 | 0 | 1 | 0 | 323 | 333 |  |
| R.VLDELTLTK.A | N | 77.31 | 1030.5911 | 9 | 0.6 | 516.3031 | 2 | 35.28 | 6 | F6:15130 | 27122019\_RID\_1315\_NaNaWb\_F5.raw | 2.85E6 |  |  |  |  | 1.8E6 |  |  |  |  | 3 | 2 | 0 | 0 | 0 | 0 | 1 | 0 | 0 | 0 | 0 | 258 | 266 |  |
| R.LENEIQTYR.S | Y | 75.36 | 1164.5775 | 9 | 0.4 | 583.2963 | 2 | 20.28 | 6 | F6:5453 | 27122019\_RID\_1315\_NaNaWb\_F5.raw | 3.28E6 |  |  |  |  | 1.33E6 |  |  | 6.26E6 |  | 3 | 1 | 0 | 0 | 0 | 0 | 1 | 0 | 0 | 1 | 0 | 442 | 450 |  |
| K.QSLEASLAETEGR.Y | Y | 75.30 | 1389.6736 | 13 | 0.7 | 695.8445 | 2 | 29.52 | 6 | F6:11144 | 27122019\_RID\_1315\_NaNaWb\_F5.raw |  |  |  | 5.85E5 |  | 1.56E6 | 4.02E7 |  |  |  | 4 | 0 | 0 | 0 | 1 | 0 | 1 | 2 | 0 | 0 | 0 | 387 | 399 |  |
| R.NVQALEIELQSQLALK.Q | Y | 73.18 | 1796.0043 | 16 | 0.3 | 899.0096 | 2 | 58.69 | 7 | F7:21750 | 27122019\_RID\_1315\_NaNaWb\_F6.raw |  |  |  |  |  |  | 1.37E7 |  |  |  | 3 | 0 | 0 | 0 | 0 | 0 | 0 | 3 | 0 | 0 | 0 | 371 | 386 |  |
| R.LKYENEVALR.Q | Y | 66.27 | 1233.6716 | 10 | 0.0 | 412.2311 | 3 | 19.86 | 6 | F6:5212 | 27122019\_RID\_1315\_NaNaWb\_F5.raw | 1.49E6 |  |  |  |  | 7.16E5 |  |  |  |  | 4 | 2 | 0 | 0 | 0 | 0 | 2 | 0 | 0 | 0 | 0 | 236 | 245 |  |
| R.QSVEADINGLR.R | N | 65.36 | 1200.6099 | 11 | 0.6 | 601.3126 | 2 | 28.51 | 6 | F6:10424 | 27122019\_RID\_1315\_NaNaWb\_F5.raw | 1.88E6 |  |  | 0 |  | 1.8E6 |  |  |  |  | 3 | 1 | 0 | 0 | 1 | 0 | 1 | 0 | 0 | 0 | 0 | 246 | 256 |  |
| Q.ISALEEQLQQIR.A | Y | 62.38 | 1426.7780 | 12 | 1.0 | 714.3970 | 2 | 54.82 | 1 | F1:28321 | 09012020\_NaNaWb\_F4.raw | 3.32E5 |  |  |  |  | 1.83E6 | 1.82E7 | 2.91E5 |  |  | 4 | 1 | 0 | 0 | 0 | 0 | 1 | 1 | 1 | 0 | 0 | 411 | 422 |  |
| K.DAEAWFNEK.S | Y | 61.70 | 1108.4825 | 9 | 0.9 | 555.2490 | 2 | 36.81 | 6 | F6:16020 | 27122019\_RID\_1315\_NaNaWb\_F5.raw | 1.08E6 |  |  |  |  | 1.83E6 |  | 1.12E6 |  |  | 3 | 1 | 0 | 0 | 0 | 0 | 1 | 0 | 1 | 0 | 0 | 335 | 343 |  |
| K.VTMQNLNDR.L | N | 58.10 | 1089.5237 | 9 | -0.1 | 545.7690 | 2 | 22.50 | 1 | F1:7071 | 09012020\_NaNaWb\_F4.raw | 3.69E5 |  |  |  |  |  |  |  |  |  | 1 | 1 | 0 | 0 | 0 | 0 | 0 | 0 | 0 | 0 | 0 | 148 | 156 |  |
| K.YENEVALR.Q | Y | 55.47 | 992.4927 | 8 | 0.8 | 497.2540 | 2 | 25.18 | 1 | F1:8570 | 09012020\_NaNaWb\_F4.raw | 8.94E5 | 1.14E6 |  |  |  |  |  |  |  |  | 2 | 1 | 1 | 0 | 0 | 0 | 0 | 0 | 0 | 0 | 0 | 238 | 245 |  |
| R.SQYEQLAEQNRK.D | Y | 53.68 | 1492.7269 | 12 | -0.5 | 498.5827 | 3 | 16.41 | 1 | F1:4032 | 09012020\_NaNaWb\_F4.raw | 6.64E5 |  |  |  |  |  |  |  |  |  | 2 | 2 | 0 | 0 | 0 | 0 | 0 | 0 | 0 | 0 | 0 | 323 | 334 |  |
| R.AETEC(+57.02)QNTEYQQLLDIK.I | Y | 52.34 | 2081.9575 | 17 | 0.0 | 1041.9861 | 2 | 44.15 | 7 | F7:15228 | 27122019\_RID\_1315\_NaNaWb\_F6.raw |  |  |  |  | 0 |  | 3.91E7 |  |  |  | 3 | 0 | 0 | 0 | 0 | 1 | 0 | 2 | 0 | 0 | 0 | 423 | 439 | Carbamidomethylation |
| R.LAADDFR.L | N | 50.17 | 806.3922 | 7 | 0.0 | 404.2034 | 2 | 27.69 | 1 | F1:10242 | 09012020\_NaNaWb\_F4.raw | 1.68E6 |  |  |  |  |  | 7.08E6 |  |  |  | 3 | 2 | 0 | 0 | 0 | 0 | 0 | 1 | 0 | 0 | 0 | 229 | 235 |  |
| Q.AQISALEEQLQQIR.A | Y | 49.07 | 1625.8737 | 14 | 1.0 | 813.9449 | 2 | 62.25 | 1 | F1:33394 | 09012020\_NaNaWb\_F4.raw | 2.24E4 |  |  |  |  |  |  |  |  |  | 1 | 1 | 0 | 0 | 0 | 0 | 0 | 0 | 0 | 0 | 0 | 409 | 422 |  |
| K.ELTTEIDNNIEQISSYK.S | Y | 48.53 | 1995.9636 | 17 | 0.2 | 998.9893 | 2 | 49.16 | 9 | F9:16788 | 27122019\_RID\_1315\_NaNaWb\_F8.raw |  |  |  |  |  |  |  |  | 4.77E6 |  | 2 | 0 | 0 | 0 | 0 | 0 | 0 | 0 | 0 | 2 | 0 | 346 | 362 |  |
| D.NANILLQIDNAR.L | N | 47.72 | 1353.7365 | 12 | 0.6 | 677.8759 | 2 | 55.42 | 1 | F1:28744 | 09012020\_NaNaWb\_F4.raw | 8.43E4 |  |  |  |  |  |  |  |  |  | 1 | 1 | 0 | 0 | 0 | 0 | 0 | 0 | 0 | 0 | 0 | 217 | 228 |  |
| R.LASYLDK.V | N | 46.86 | 808.4330 | 7 | -0.5 | 405.2236 | 2 | 24.16 | 1 | F1:8330 | 09012020\_NaNaWb\_F4.raw | 1.42E6 |  |  |  |  | 2.35E5 |  |  |  |  | 2 | 1 | 0 | 0 | 0 | 0 | 1 | 0 | 0 | 0 | 0 | 157 | 163 |  |
| T.EELAYLK.K | N | 44.65 | 864.4592 | 7 | -0.2 | 433.2368 | 2 | 34.81 | 1 | F1:14659 | 09012020\_NaNaWb\_F4.raw | 0 |  |  |  |  |  |  |  |  |  | 1 | 1 | 0 | 0 | 0 | 0 | 0 | 0 | 0 | 0 | 0 | 278 | 284 |  |
| K.GSLGGGFSSGGFSGGSFSR.G | Y | 44.24 | 1706.7648 | 19 | -0.5 | 854.3892 | 2 | 38.78 | 2 | F2:9433 | 27122019\_RID\_1315\_NaNaWb\_F1.raw |  | 1.4E6 |  |  |  |  |  |  | 1.05E6 |  | 2 | 0 | 1 | 0 | 0 | 0 | 0 | 0 | 0 | 1 | 0 | 41 | 59 |  |
| K.NQILNLTTDNAN.I | Y | 44.00 | 1329.6525 | 12 | 0.3 | 665.8337 | 2 | 41.95 | 7 | F7:14394 | 27122019\_RID\_1315\_NaNaWb\_F6.raw |  |  |  |  |  |  | 3.13E6 |  |  |  | 1 | 0 | 0 | 0 | 0 | 0 | 0 | 1 | 0 | 0 | 0 | 208 | 219 |  |
| K.SEITELR.R | Y | 43.76 | 846.4447 | 7 | -0.3 | 424.2295 | 2 | 18.74 | 6 | F6:4577 | 27122019\_RID\_1315\_NaNaWb\_F5.raw |  |  |  |  |  | 3.64E5 |  |  |  |  | 1 | 0 | 0 | 0 | 0 | 0 | 1 | 0 | 0 | 0 | 0 | 363 | 369 |  |
| R.SLLEGEGSSGGGGR.G | Y | 42.11 | 1261.5898 | 14 | 0.7 | 631.8026 | 2 | 16.86 | 2 | F2:3719 | 27122019\_RID\_1315\_NaNaWb\_F1.raw |  | 1.26E6 |  |  |  |  |  |  |  |  | 1 | 0 | 1 | 0 | 0 | 0 | 0 | 0 | 0 | 0 | 0 | 451 | 464 |  |
| E.YQQLLDIK.I | Y | 41.20 | 1019.5651 | 8 | 0.6 | 510.7901 | 2 | 44.72 | 1 | F1:21322 | 09012020\_NaNaWb\_F4.raw | 1.53E5 |  |  |  |  |  |  |  |  |  | 1 | 1 | 0 | 0 | 0 | 0 | 0 | 0 | 0 | 0 | 0 | 432 | 439 |  |
| total 26 peptides |  |  |  |  |  |  |  |  |  |  |  |  |  |  |  |  |  |  |  |  |  |  |  |  |  |  |  |  |  |  |  |  |  |  |  |
| --- | --- | --- | --- | --- | --- | --- | --- | --- | --- | --- | --- | --- | --- | --- | --- | --- | --- | --- | --- | --- | --- | --- | --- | --- | --- | --- | --- | --- | --- | --- | --- | --- | --- | --- | --- |

P13645|K1C10\_HUMAN

back to list

  

| Protein Coverage
| Supporting Peptides
|

Protein Coverage:

Supporting Peptides:

| Peptide | Uniq | -10lgP | Mass | Length | ppm | m/z | z | RT | Fraction | Scan | Source File | Area F4 | Area F1 | Area F10 | Area F2 | Area F3 | Area F5 | Area F6 | Area F7 | Area F8 | Area F9 | #Feature | #Feature F4 | #Feature F1 | #Feature F10 | #Feature F2 | #Feature F3 | #Feature F5 | #Feature F6 | #Feature F7 | #Feature F8 | #Feature F9 | Start | End | PTM |
| --- | --- | --- | --- | --- | --- | --- | --- | --- | --- | --- | --- | --- | --- | --- | --- | --- | --- | --- | --- | --- | --- | --- | --- | --- | --- | --- | --- | --- | --- | --- | --- | --- | --- | --- | --- |
| R.ALEESNYELEGK.I | Y | 81.22 | 1380.6409 | 12 | -0.1 | 691.3276 | 2 | 23.77 | 7 | F7:6974 | 27122019\_RID\_1315\_NaNaWb\_F6.raw | 2.17E6 |  | 6.55E5 | 1.17E6 |  | 2.52E6 | 2.07E7 | 2.26E6 |  |  | 7 | 2 | 0 | 1 | 1 | 0 | 1 | 1 | 1 | 0 | 0 | 166 | 177 |  |
| K.NQILNLTTDNANILLQIDNAR.L | Y | 78.77 | 2366.2554 | 21 | -0.9 | 1184.1339 | 2 | 63.33 | 7 | F7:23654 | 27122019\_RID\_1315\_NaNaWb\_F6.raw |  |  |  |  |  |  | 1.91E7 |  | 1.08E6 |  | 5 | 0 | 0 | 0 | 0 | 0 | 0 | 2 | 0 | 3 | 0 | 208 | 228 |  |
| R.SQYEQLAEQNR.K | Y | 78.49 | 1364.6321 | 11 | 0.6 | 683.3237 | 2 | 18.04 | 6 | F6:4236 | 27122019\_RID\_1315\_NaNaWb\_F5.raw | 1.62E6 |  |  |  |  | 1.05E6 | 1.96E7 |  | 3.33E6 |  | 4 | 1 | 0 | 0 | 0 | 0 | 1 | 1 | 0 | 1 | 0 | 323 | 333 |  |
| R.VLDELTLTK.A | N | 77.31 | 1030.5911 | 9 | 0.6 | 516.3031 | 2 | 35.28 | 6 | F6:15130 | 27122019\_RID\_1315\_NaNaWb\_F5.raw | 2.85E6 |  |  |  |  | 1.8E6 |  |  |  |  | 3 | 2 | 0 | 0 | 0 | 0 | 1 | 0 | 0 | 0 | 0 | 258 | 266 |  |
| R.LENEIQTYR.S | Y | 75.36 | 1164.5775 | 9 | 0.4 | 583.2963 | 2 | 20.28 | 6 | F6:5453 | 27122019\_RID\_1315\_NaNaWb\_F5.raw | 3.28E6 |  |  |  |  | 1.33E6 |  |  | 6.26E6 |  | 3 | 1 | 0 | 0 | 0 | 0 | 1 | 0 | 0 | 1 | 0 | 442 | 450 |  |
| K.QSLEASLAETEGR.Y | Y | 75.30 | 1389.6736 | 13 | 0.7 | 695.8445 | 2 | 29.52 | 6 | F6:11144 | 27122019\_RID\_1315\_NaNaWb\_F5.raw |  |  |  | 5.85E5 |  | 1.56E6 | 4.02E7 |  |  |  | 4 | 0 | 0 | 0 | 1 | 0 | 1 | 2 | 0 | 0 | 0 | 387 | 399 |  |
| R.NVQALEIELQSQLALK.Q | Y | 73.18 | 1796.0043 | 16 | 0.3 | 899.0096 | 2 | 58.69 | 7 | F7:21750 | 27122019\_RID\_1315\_NaNaWb\_F6.raw |  |  |  |  |  |  | 1.37E7 |  |  |  | 3 | 0 | 0 | 0 | 0 | 0 | 0 | 3 | 0 | 0 | 0 | 371 | 386 |  |
| R.LKYENEVALR.Q | Y | 66.27 | 1233.6716 | 10 | 0.0 | 412.2311 | 3 | 19.86 | 6 | F6:5212 | 27122019\_RID\_1315\_NaNaWb\_F5.raw | 1.49E6 |  |  |  |  | 7.16E5 |  |  |  |  | 4 | 2 | 0 | 0 | 0 | 0 | 2 | 0 | 0 | 0 | 0 | 236 | 245 |  |
| R.QSVEADINGLR.R | N | 65.36 | 1200.6099 | 11 | 0.6 | 601.3126 | 2 | 28.51 | 6 | F6:10424 | 27122019\_RID\_1315\_NaNaWb\_F5.raw | 1.88E6 |  |  | 0 |  | 1.8E6 |  |  |  |  | 3 | 1 | 0 | 0 | 1 | 0 | 1 | 0 | 0 | 0 | 0 | 246 | 256 |  |
| Q.ISALEEQLQQIR.A | Y | 62.38 | 1426.7780 | 12 | 1.0 | 714.3970 | 2 | 54.82 | 1 | F1:28321 | 09012020\_NaNaWb\_F4.raw | 3.32E5 |  |  |  |  | 1.83E6 | 1.82E7 | 2.91E5 |  |  | 4 | 1 | 0 | 0 | 0 | 0 | 1 | 1 | 1 | 0 | 0 | 411 | 422 |  |
| K.DAEAWFNEK.S | Y | 61.70 | 1108.4825 | 9 | 0.9 | 555.2490 | 2 | 36.81 | 6 | F6:16020 | 27122019\_RID\_1315\_NaNaWb\_F5.raw | 1.08E6 |  |  |  |  | 1.83E6 |  | 1.12E6 |  |  | 3 | 1 | 0 | 0 | 0 | 0 | 1 | 0 | 1 | 0 | 0 | 335 | 343 |  |
| K.VTMQNLNDR.L | N | 58.10 | 1089.5237 | 9 | -0.1 | 545.7690 | 2 | 22.50 | 1 | F1:7071 | 09012020\_NaNaWb\_F4.raw | 3.69E5 |  |  |  |  |  |  |  |  |  | 1 | 1 | 0 | 0 | 0 | 0 | 0 | 0 | 0 | 0 | 0 | 148 | 156 |  |
| K.YENEVALR.Q | Y | 55.47 | 992.4927 | 8 | 0.8 | 497.2540 | 2 | 25.18 | 1 | F1:8570 | 09012020\_NaNaWb\_F4.raw | 8.94E5 | 1.14E6 |  |  |  |  |  |  |  |  | 2 | 1 | 1 | 0 | 0 | 0 | 0 | 0 | 0 | 0 | 0 | 238 | 245 |  |
| R.SQYEQLAEQNRK.D | Y | 53.68 | 1492.7269 | 12 | -0.5 | 498.5827 | 3 | 16.41 | 1 | F1:4032 | 09012020\_NaNaWb\_F4.raw | 6.64E5 |  |  |  |  |  |  |  |  |  | 2 | 2 | 0 | 0 | 0 | 0 | 0 | 0 | 0 | 0 | 0 | 323 | 334 |  |
| R.AETEC(+57.02)QNTEYQQLLDIK.I | Y | 52.34 | 2081.9575 | 17 | 0.0 | 1041.9861 | 2 | 44.15 | 7 | F7:15228 | 27122019\_RID\_1315\_NaNaWb\_F6.raw |  |  |  |  | 0 |  | 3.91E7 |  |  |  | 3 | 0 | 0 | 0 | 0 | 1 | 0 | 2 | 0 | 0 | 0 | 423 | 439 | Carbamidomethylation |
| R.LAADDFR.L | N | 50.17 | 806.3922 | 7 | 0.0 | 404.2034 | 2 | 27.69 | 1 | F1:10242 | 09012020\_NaNaWb\_F4.raw | 1.68E6 |  |  |  |  |  | 7.08E6 |  |  |  | 3 | 2 | 0 | 0 | 0 | 0 | 0 | 1 | 0 | 0 | 0 | 229 | 235 |  |
| Q.AQISALEEQLQQIR.A | Y | 49.07 | 1625.8737 | 14 | 1.0 | 813.9449 | 2 | 62.25 | 1 | F1:33394 | 09012020\_NaNaWb\_F4.raw | 2.24E4 |  |  |  |  |  |  |  |  |  | 1 | 1 | 0 | 0 | 0 | 0 | 0 | 0 | 0 | 0 | 0 | 409 | 422 |  |
| K.ELTTEIDNNIEQISSYK.S | Y | 48.53 | 1995.9636 | 17 | 0.2 | 998.9893 | 2 | 49.16 | 9 | F9:16788 | 27122019\_RID\_1315\_NaNaWb\_F8.raw |  |  |  |  |  |  |  |  | 4.77E6 |  | 2 | 0 | 0 | 0 | 0 | 0 | 0 | 0 | 0 | 2 | 0 | 346 | 362 |  |
| D.NANILLQIDNAR.L | N | 47.72 | 1353.7365 | 12 | 0.6 | 677.8759 | 2 | 55.42 | 1 | F1:28744 | 09012020\_NaNaWb\_F4.raw | 8.43E4 |  |  |  |  |  |  |  |  |  | 1 | 1 | 0 | 0 | 0 | 0 | 0 | 0 | 0 | 0 | 0 | 217 | 228 |  |
| R.LASYLDK.V | N | 46.86 | 808.4330 | 7 | -0.5 | 405.2236 | 2 | 24.16 | 1 | F1:8330 | 09012020\_NaNaWb\_F4.raw | 1.42E6 |  |  |  |  | 2.35E5 |  |  |  |  | 2 | 1 | 0 | 0 | 0 | 0 | 1 | 0 | 0 | 0 | 0 | 157 | 163 |  |
| T.EELAYLK.K | N | 44.65 | 864.4592 | 7 | -0.2 | 433.2368 | 2 | 34.81 | 1 | F1:14659 | 09012020\_NaNaWb\_F4.raw | 0 |  |  |  |  |  |  |  |  |  | 1 | 1 | 0 | 0 | 0 | 0 | 0 | 0 | 0 | 0 | 0 | 278 | 284 |  |
| K.GSLGGGFSSGGFSGGSFSR.G | Y | 44.24 | 1706.7648 | 19 | -0.5 | 854.3892 | 2 | 38.78 | 2 | F2:9433 | 27122019\_RID\_1315\_NaNaWb\_F1.raw |  | 1.4E6 |  |  |  |  |  |  | 1.05E6 |  | 2 | 0 | 1 | 0 | 0 | 0 | 0 | 0 | 0 | 1 | 0 | 41 | 59 |  |
| K.NQILNLTTDNAN.I | Y | 44.00 | 1329.6525 | 12 | 0.3 | 665.8337 | 2 | 41.95 | 7 | F7:14394 | 27122019\_RID\_1315\_NaNaWb\_F6.raw |  |  |  |  |  |  | 3.13E6 |  |  |  | 1 | 0 | 0 | 0 | 0 | 0 | 0 | 1 | 0 | 0 | 0 | 208 | 219 |  |
| K.SEITELR.R | Y | 43.76 | 846.4447 | 7 | -0.3 | 424.2295 | 2 | 18.74 | 6 | F6:4577 | 27122019\_RID\_1315\_NaNaWb\_F5.raw |  |  |  |  |  | 3.64E5 |  |  |  |  | 1 | 0 | 0 | 0 | 0 | 0 | 1 | 0 | 0 | 0 | 0 | 363 | 369 |  |
| R.SLLEGEGSSGGGGR.G | Y | 42.11 | 1261.5898 | 14 | 0.7 | 631.8026 | 2 | 16.86 | 2 | F2:3719 | 27122019\_RID\_1315\_NaNaWb\_F1.raw |  | 1.26E6 |  |  |  |  |  |  |  |  | 1 | 0 | 1 | 0 | 0 | 0 | 0 | 0 | 0 | 0 | 0 | 451 | 464 |  |
| E.YQQLLDIK.I | Y | 41.20 | 1019.5651 | 8 | 0.6 | 510.7901 | 2 | 44.72 | 1 | F1:21322 | 09012020\_NaNaWb\_F4.raw | 1.53E5 |  |  |  |  |  |  |  |  |  | 1 | 1 | 0 | 0 | 0 | 0 | 0 | 0 | 0 | 0 | 0 | 432 | 439 |  |
| total 26 peptides |  |  |  |  |  |  |  |  |  |  |  |  |  |  |  |  |  |  |  |  |  |  |  |  |  |  |  |  |  |  |  |  |  |  |  |
| --- | --- | --- | --- | --- | --- | --- | --- | --- | --- | --- | --- | --- | --- | --- | --- | --- | --- | --- | --- | --- | --- | --- | --- | --- | --- | --- | --- | --- | --- | --- | --- | --- | --- | --- | --- |

P25674|3L21\_NAJHH

back to list

  

| Protein Coverage
| Supporting Peptides
|

Protein Coverage:

Supporting Peptides:

| Peptide | Uniq | -10lgP | Mass | Length | ppm | m/z | z | RT | Fraction | Scan | Source File | Area F4 | Area F1 | Area F10 | Area F2 | Area F3 | Area F5 | Area F6 | Area F7 | Area F8 | Area F9 | #Feature | #Feature F4 | #Feature F1 | #Feature F10 | #Feature F2 | #Feature F3 | #Feature F5 | #Feature F6 | #Feature F7 | #Feature F8 | #Feature F9 | Start | End | PTM |
| --- | --- | --- | --- | --- | --- | --- | --- | --- | --- | --- | --- | --- | --- | --- | --- | --- | --- | --- | --- | --- | --- | --- | --- | --- | --- | --- | --- | --- | --- | --- | --- | --- | --- | --- | --- |
| R.VDLGC(+57.02)AATC(+57.02)PTVK.P | N | 107.87 | 1390.6584 | 13 | 0.4 | 696.3368 | 2 | 25.60 | 6 | F6:8613 | 27122019\_RID\_1315\_NaNaWb\_F5.raw | 1.71E8 |  |  |  |  | 6.5E9 |  | 0 |  | 1.36E8 | 34 | 4 | 0 | 0 | 0 | 0 | 27 | 0 | 1 | 0 | 2 | 37 | 49 | Carbamidomethylation |
| K.C(+57.02)C(+57.02)STDNC(+57.02)NPFPTR.K | N | 93.32 | 1627.6178 | 13 | 0.4 | 814.8165 | 2 | 21.63 | 6 | F6:6170 | 27122019\_RID\_1315\_NaNaWb\_F5.raw | 2.89E8 | 4.91E6 |  | 1.39E7 | 3.67E6 | 5.21E7 |  |  |  | 5.46E7 | 24 | 15 | 1 | 0 | 1 | 1 | 4 | 0 | 0 | 0 | 2 | 56 | 68 | Carbamidomethylation |
| C.C(+57.02)STDNC(+57.02)NPFPTR.K | N | 86.21 | 1467.5872 | 12 | 0.1 | 734.8009 | 2 | 21.30 | 6 | F6:6148 | 27122019\_RID\_1315\_NaNaWb\_F5.raw | 1.04E8 | 2.26E6 |  |  |  | 4.53E7 |  |  |  |  | 10 | 5 | 1 | 0 | 0 | 0 | 4 | 0 | 0 | 0 | 0 | 57 | 68 | Carbamidomethylation |
| C.STDNC(+57.02)NPFPTR.K | N | 81.69 | 1307.5564 | 11 | 0.6 | 654.7859 | 2 | 20.44 | 6 | F6:5588 | 27122019\_RID\_1315\_NaNaWb\_F5.raw | 8.16E8 |  |  |  |  | 7.73E8 |  |  |  | 7.53E8 | 17 | 7 | 0 | 0 | 0 | 0 | 7 | 0 | 0 | 0 | 3 | 58 | 68 | Carbamidomethylation |
| S.TDNC(+57.02)NPFPTR.K | N | 68.43 | 1220.5244 | 10 | -0.5 | 611.2692 | 2 | 20.77 | 6 | F6:5754 | 27122019\_RID\_1315\_NaNaWb\_F5.raw | 1.36E7 |  |  |  |  | 3.22E6 |  |  |  | 1.64E7 | 4 | 2 | 0 | 0 | 0 | 0 | 1 | 0 | 0 | 0 | 1 | 59 | 68 | Carbamidomethylation |
| R.VDLGC(+57.02)AATC(+57.02)PTVKPGV.D | Y | 64.59 | 1643.8011 | 16 | 7.1 | 548.9449 | 3 | 22.10 | 6 | F6:6497 | 27122019\_RID\_1315\_NaNaWb\_F5.raw |  |  |  |  |  | 1.83E5 |  |  |  |  | 1 | 0 | 0 | 0 | 0 | 0 | 1 | 0 | 0 | 0 | 0 | 37 | 52 | Carbamidomethylation |
| R.GKRVDLGC(+57.02)AATC(+57.02)PTVK.P | N | 61.52 | 1731.8760 | 16 | -0.3 | 578.2991 | 3 | 12.32 | 6 | F6:2146 | 27122019\_RID\_1315\_NaNaWb\_F5.raw |  |  |  |  |  | 0 |  |  |  |  | 1 | 0 | 0 | 0 | 0 | 0 | 1 | 0 | 0 | 0 | 0 | 34 | 49 | Carbamidomethylation |
| R.VDLGC(+57.02)AATC(+57.02)PTV.K | N | 58.41 | 1262.5635 | 12 | 0.1 | 632.2891 | 2 | 52.53 | 1 | F1:26732 | 09012020\_NaNaWb\_F4.raw | 3.81E7 |  |  |  |  |  |  |  |  |  | 2 | 2 | 0 | 0 | 0 | 0 | 0 | 0 | 0 | 0 | 0 | 37 | 48 | Carbamidomethylation |
| T.DNC(+57.02)NPFPTR.K | N | 56.81 | 1119.4767 | 9 | -0.5 | 560.7454 | 2 | 23.23 | 10 | F10:4636 | 27122019\_RID\_1315\_NaNaWb\_F9.raw | 6.62E6 |  |  |  |  |  |  |  |  | 2.34E6 | 3 | 2 | 0 | 0 | 0 | 0 | 0 | 0 | 0 | 0 | 1 | 60 | 68 | Carbamidomethylation |
| C.PDGHVC(+57.02)YTK.M | Y | 54.71 | 1075.4757 | 9 | -0.1 | 538.7451 | 2 | 17.17 | 1 | F1:4269 | 09012020\_NaNaWb\_F4.raw | 3.43E7 |  |  |  |  | 1.03E7 |  |  |  |  | 7 | 6 | 0 | 0 | 0 | 0 | 1 | 0 | 0 | 0 | 0 | 15 | 23 | Carbamidomethylation |
| D.GHVC(+57.02)YTK.M | N | 51.73 | 863.3960 | 7 | 0.3 | 432.7054 | 2 | 12.44 | 1 | F1:2007 | 09012020\_NaNaWb\_F4.raw | 1.01E5 |  |  |  |  |  |  |  |  |  | 1 | 1 | 0 | 0 | 0 | 0 | 0 | 0 | 0 | 0 | 0 | 17 | 23 | Carbamidomethylation |
| D.NC(+57.02)NPFPTR.K | N | 46.38 | 1004.4498 | 8 | 0.2 | 503.2322 | 2 | 25.51 | 1 | F1:8810 | 09012020\_NaNaWb\_F4.raw | 0 |  |  |  |  |  |  |  |  |  | 2 | 2 | 0 | 0 | 0 | 0 | 0 | 0 | 0 | 0 | 0 | 61 | 68 | Carbamidomethylation |
| K.RVDLGC(+57.02)AATC(+57.02)PTVK.P | N | 45.12 | 1546.7595 | 14 | 0.6 | 516.5941 | 3 | 19.45 | 6 | F6:4933 | 27122019\_RID\_1315\_NaNaWb\_F5.raw |  |  |  |  |  | 3.17E7 | 7.72E5 |  |  |  | 3 | 0 | 0 | 0 | 0 | 0 | 2 | 1 | 0 | 0 | 0 | 36 | 49 | Carbamidomethylation |
| R.VDLGC(+57.02)AATC(+57.02)PT.V | N | 42.06 | 1163.4951 | 11 | 0.7 | 582.7552 | 2 | 40.40 | 1 | F1:18369 | 09012020\_NaNaWb\_F4.raw | 5.03E7 |  |  |  |  |  |  |  |  |  | 2 | 2 | 0 | 0 | 0 | 0 | 0 | 0 | 0 | 0 | 0 | 37 | 47 | Carbamidomethylation |
| total 14 peptides |  |  |  |  |  |  |  |  |  |  |  |  |  |  |  |  |  |  |  |  |  |  |  |  |  |  |  |  |  |  |  |  |  |  |  |
| --- | --- | --- | --- | --- | --- | --- | --- | --- | --- | --- | --- | --- | --- | --- | --- | --- | --- | --- | --- | --- | --- | --- | --- | --- | --- | --- | --- | --- | --- | --- | --- | --- | --- | --- | --- |

P35527|K1C9\_HUMAN

back to list

  

| Protein Coverage
| Supporting Peptides
|

Protein Coverage:

Supporting Peptides:

| Peptide | Uniq | -10lgP | Mass | Length | ppm | m/z | z | RT | Fraction | Scan | Source File | Area F4 | Area F1 | Area F10 | Area F2 | Area F3 | Area F5 | Area F6 | Area F7 | Area F8 | Area F9 | #Feature | #Feature F4 | #Feature F1 | #Feature F10 | #Feature F2 | #Feature F3 | #Feature F5 | #Feature F6 | #Feature F7 | #Feature F8 | #Feature F9 | Start | End | PTM |
| --- | --- | --- | --- | --- | --- | --- | --- | --- | --- | --- | --- | --- | --- | --- | --- | --- | --- | --- | --- | --- | --- | --- | --- | --- | --- | --- | --- | --- | --- | --- | --- | --- | --- | --- | --- |
| R.SGGGGGGGLGSGGSIR.S | Y | 88.62 | 1231.5905 | 16 | -0.6 | 616.8021 | 2 | 12.28 | 8 | F8:2093 | 27122019\_RID\_1315\_NaNaWb\_F7.raw |  |  | 2.71E6 |  |  |  |  | 6.32E6 |  | 7.08E5 | 3 | 0 | 0 | 1 | 0 | 0 | 0 | 0 | 1 | 0 | 1 | 14 | 29 |  |
| R.GGSGGSYGGGGSGGGYGGGSGSR.G | Y | 86.84 | 1790.7203 | 23 | -0.1 | 896.3674 | 2 | 12.26 | 8 | F8:2063 | 27122019\_RID\_1315\_NaNaWb\_F7.raw | 2.02E5 |  |  |  |  |  |  | 2.11E6 |  |  | 2 | 1 | 0 | 0 | 0 | 0 | 0 | 0 | 1 | 0 | 0 | 491 | 513 |  |
| K.VQALEEANNDLENK.I | Y | 78.05 | 1585.7583 | 14 | 0.1 | 793.8865 | 2 | 24.67 | 7 | F7:7329 | 27122019\_RID\_1315\_NaNaWb\_F6.raw | 9.66E5 |  |  |  |  | 1.27E5 | 2.01E7 |  | 4.29E6 |  | 5 | 2 | 0 | 0 | 0 | 0 | 1 | 1 | 0 | 1 | 0 | 171 | 184 |  |
| K.EIETYHNLLEGGQEDFESSGAGK.I | Y | 78.03 | 2509.1245 | 23 | -2.1 | 1255.5669 | 2 | 39.53 | 7 | F7:13381 | 27122019\_RID\_1315\_NaNaWb\_F6.raw | 1.09E5 |  |  |  |  |  | 2.54E7 | 4.83E6 |  |  | 6 | 2 | 0 | 0 | 0 | 0 | 0 | 3 | 1 | 0 | 0 | 450 | 472 |  |
| R.QGVDADINGLR.Q | Y | 74.97 | 1156.5836 | 11 | 0.6 | 579.2994 | 2 | 36.73 | 1 | F1:15794 | 09012020\_NaNaWb\_F4.raw | 1.08E6 |  |  |  |  | 1.23E5 |  | 6.43E6 |  |  | 3 | 1 | 0 | 0 | 0 | 0 | 1 | 0 | 1 | 0 | 0 | 251 | 261 |  |
| R.HGVQELEIELQSQLSK.K | Y | 68.59 | 1836.9581 | 16 | -0.2 | 613.3265 | 3 | 45.42 | 7 | F7:15804 | 27122019\_RID\_1315\_NaNaWb\_F6.raw |  |  |  |  |  |  | 2.32E7 |  | 3.08E6 |  | 3 | 0 | 0 | 0 | 0 | 0 | 0 | 2 | 0 | 1 | 0 | 375 | 390 |  |
| Q.ISNLEAQITDVR.Q | Y | 64.00 | 1357.7201 | 12 | 1.4 | 679.8683 | 2 | 34.75 | 7 | F7:11675 | 27122019\_RID\_1315\_NaNaWb\_F6.raw |  |  |  |  |  |  | 1.2E6 |  |  |  | 1 | 0 | 0 | 0 | 0 | 0 | 0 | 1 | 0 | 0 | 0 | 416 | 427 |  |
| K.NYSPYYNTIDDLK.D | Y | 57.29 | 1604.7358 | 13 | 0.2 | 803.3754 | 2 | 39.54 | 7 | F7:13344 | 27122019\_RID\_1315\_NaNaWb\_F6.raw |  |  |  |  |  |  | 3.99E6 | 0 |  |  | 2 | 0 | 0 | 0 | 0 | 0 | 0 | 1 | 1 | 0 | 0 | 200 | 212 |  |
| R.LASYLDKVQALEEANNDLENK.I | Y | 56.61 | 2376.1809 | 21 | 1.2 | 793.0685 | 3 | 45.12 | 8 | F8:11063 | 27122019\_RID\_1315\_NaNaWb\_F7.raw |  |  |  |  |  |  |  | 1.14E6 |  |  | 1 | 0 | 0 | 0 | 0 | 0 | 0 | 0 | 1 | 0 | 0 | 164 | 184 |  |
| R.QEIEC(+57.02)QNQEYSLLLSIK.M | Y | 55.73 | 2094.0303 | 17 | -0.9 | 1048.0215 | 2 | 53.15 | 8 | F8:12910 | 27122019\_RID\_1315\_NaNaWb\_F7.raw |  |  |  |  |  |  |  | 5.02E5 | 4.41E6 |  | 2 | 0 | 0 | 0 | 0 | 0 | 0 | 0 | 1 | 1 | 0 | 428 | 444 | Carbamidomethylation |
| K.STMQELNSR.L | Y | 52.24 | 1064.4921 | 9 | -0.4 | 533.2531 | 2 | 12.37 | 8 | F8:2154 | 27122019\_RID\_1315\_NaNaWb\_F7.raw |  |  |  |  |  |  |  | 2.8E6 |  |  | 1 | 0 | 0 | 0 | 0 | 0 | 0 | 0 | 1 | 0 | 0 | 155 | 163 |  |
| K.IQDWYDKK.G | Y | 51.30 | 1094.5397 | 8 | 1.5 | 548.2780 | 2 | 21.39 | 1 | F1:6491 | 09012020\_NaNaWb\_F4.raw | 8.28E4 |  |  |  |  |  |  |  |  |  | 1 | 1 | 0 | 0 | 0 | 0 | 0 | 0 | 0 | 0 | 0 | 185 | 192 |  |
| R.QEYEQLIAK.N | Y | 50.46 | 1120.5764 | 9 | 0.3 | 561.2957 | 2 | 34.91 | 1 | F1:14602 | 09012020\_NaNaWb\_F4.raw | 1.65E6 |  |  | 7.31E5 |  |  |  |  |  |  | 3 | 2 | 0 | 0 | 1 | 0 | 0 | 0 | 0 | 0 | 0 | 328 | 336 |  |
| R.M(+15.99)TLDDFR.I | Y | 49.53 | 912.4011 | 7 | -0.2 | 457.2078 | 2 | 31.59 | 1 | F1:12593 | 09012020\_NaNaWb\_F4.raw | 4.83E5 |  |  |  |  |  |  |  |  |  | 2 | 2 | 0 | 0 | 0 | 0 | 0 | 0 | 0 | 0 | 0 | 234 | 240 | Oxidation (M) |
| R.MTLDDFR.I | Y | 48.79 | 896.4062 | 7 | -0.7 | 449.2101 | 2 | 45.19 | 1 | F1:21718 | 09012020\_NaNaWb\_F4.raw | 1.71E5 |  |  |  |  |  | 0 |  |  |  | 2 | 1 | 0 | 0 | 0 | 0 | 0 | 1 | 0 | 0 | 0 | 234 | 240 |  |
| F.GGFGGGAGGGDGGILTANEK.S | Y | 48.69 | 1690.7910 | 20 | 0.0 | 846.4028 | 2 | 32.53 | 7 | F7:10695 | 27122019\_RID\_1315\_NaNaWb\_F6.raw |  |  |  |  |  |  | 0 |  |  |  | 1 | 0 | 0 | 0 | 0 | 0 | 0 | 1 | 0 | 0 | 0 | 135 | 154 |  |
| Y.SYGGGSGGGFSASSLGGGFGGGSR.G | Y | 46.93 | 2021.8827 | 24 | -1.2 | 1011.9474 | 2 | 37.53 | 2 | F2:9193 | 27122019\_RID\_1315\_NaNaWb\_F1.raw |  | 1.41E5 |  |  |  |  | 4.64E6 |  |  |  | 2 | 0 | 1 | 0 | 0 | 0 | 0 | 1 | 0 | 0 | 0 | 72 | 95 |  |
| R.LASYLDK.V | N | 46.86 | 808.4330 | 7 | -0.5 | 405.2236 | 2 | 24.16 | 1 | F1:8330 | 09012020\_NaNaWb\_F4.raw | 1.42E6 |  |  |  |  | 2.35E5 |  |  |  |  | 2 | 1 | 0 | 0 | 0 | 0 | 1 | 0 | 0 | 0 | 0 | 164 | 170 |  |
| K.TLLDIDNTR.M | Y | 44.94 | 1059.5560 | 9 | 0.9 | 530.7858 | 2 | 43.33 | 1 | F1:20564 | 09012020\_NaNaWb\_F4.raw | 2.4E6 | 9.26E6 |  |  |  |  |  |  |  |  | 3 | 2 | 1 | 0 | 0 | 0 | 0 | 0 | 0 | 0 | 0 | 225 | 233 |  |
| total 19 peptides |  |  |  |  |  |  |  |  |  |  |  |  |  |  |  |  |  |  |  |  |  |  |  |  |  |  |  |  |  |  |  |  |  |  |  |
| --- | --- | --- | --- | --- | --- | --- | --- | --- | --- | --- | --- | --- | --- | --- | --- | --- | --- | --- | --- | --- | --- | --- | --- | --- | --- | --- | --- | --- | --- | --- | --- | --- | --- | --- | --- |

#CONTAM#K1C9\_HUMAN|

back to list

  

| Protein Coverage
| Supporting Peptides
|

Protein Coverage:

Supporting Peptides:

| Peptide | Uniq | -10lgP | Mass | Length | ppm | m/z | z | RT | Fraction | Scan | Source File | Area F4 | Area F1 | Area F10 | Area F2 | Area F3 | Area F5 | Area F6 | Area F7 | Area F8 | Area F9 | #Feature | #Feature F4 | #Feature F1 | #Feature F10 | #Feature F2 | #Feature F3 | #Feature F5 | #Feature F6 | #Feature F7 | #Feature F8 | #Feature F9 | Start | End | PTM |
| --- | --- | --- | --- | --- | --- | --- | --- | --- | --- | --- | --- | --- | --- | --- | --- | --- | --- | --- | --- | --- | --- | --- | --- | --- | --- | --- | --- | --- | --- | --- | --- | --- | --- | --- | --- |
| R.SGGGGGGGLGSGGSIR.S | Y | 88.62 | 1231.5905 | 16 | -0.6 | 616.8021 | 2 | 12.28 | 8 | F8:2093 | 27122019\_RID\_1315\_NaNaWb\_F7.raw |  |  | 2.71E6 |  |  |  |  | 6.32E6 |  | 7.08E5 | 3 | 0 | 0 | 1 | 0 | 0 | 0 | 0 | 1 | 0 | 1 | 14 | 29 |  |
| R.GGSGGSYGGGGSGGGYGGGSGSR.G | Y | 86.84 | 1790.7203 | 23 | -0.1 | 896.3674 | 2 | 12.26 | 8 | F8:2063 | 27122019\_RID\_1315\_NaNaWb\_F7.raw | 2.02E5 |  |  |  |  |  |  | 2.11E6 |  |  | 2 | 1 | 0 | 0 | 0 | 0 | 0 | 0 | 1 | 0 | 0 | 491 | 513 |  |
| K.VQALEEANNDLENK.I | Y | 78.05 | 1585.7583 | 14 | 0.1 | 793.8865 | 2 | 24.67 | 7 | F7:7329 | 27122019\_RID\_1315\_NaNaWb\_F6.raw | 9.66E5 |  |  |  |  | 1.27E5 | 2.01E7 |  | 4.29E6 |  | 5 | 2 | 0 | 0 | 0 | 0 | 1 | 1 | 0 | 1 | 0 | 171 | 184 |  |
| K.EIETYHNLLEGGQEDFESSGAGK.I | Y | 78.03 | 2509.1245 | 23 | -2.1 | 1255.5669 | 2 | 39.53 | 7 | F7:13381 | 27122019\_RID\_1315\_NaNaWb\_F6.raw | 1.09E5 |  |  |  |  |  | 2.54E7 | 4.83E6 |  |  | 6 | 2 | 0 | 0 | 0 | 0 | 0 | 3 | 1 | 0 | 0 | 450 | 472 |  |
| R.QGVDADINGLR.Q | Y | 74.97 | 1156.5836 | 11 | 0.6 | 579.2994 | 2 | 36.73 | 1 | F1:15794 | 09012020\_NaNaWb\_F4.raw | 1.08E6 |  |  |  |  | 1.23E5 |  | 6.43E6 |  |  | 3 | 1 | 0 | 0 | 0 | 0 | 1 | 0 | 1 | 0 | 0 | 251 | 261 |  |
| R.HGVQELEIELQSQLSK.K | Y | 68.59 | 1836.9581 | 16 | -0.2 | 613.3265 | 3 | 45.42 | 7 | F7:15804 | 27122019\_RID\_1315\_NaNaWb\_F6.raw |  |  |  |  |  |  | 2.32E7 |  | 3.08E6 |  | 3 | 0 | 0 | 0 | 0 | 0 | 0 | 2 | 0 | 1 | 0 | 375 | 390 |  |
| Q.ISNLEAQITDVR.Q | Y | 64.00 | 1357.7201 | 12 | 1.4 | 679.8683 | 2 | 34.75 | 7 | F7:11675 | 27122019\_RID\_1315\_NaNaWb\_F6.raw |  |  |  |  |  |  | 1.2E6 |  |  |  | 1 | 0 | 0 | 0 | 0 | 0 | 0 | 1 | 0 | 0 | 0 | 416 | 427 |  |
| K.NYSPYYNTIDDLK.D | Y | 57.29 | 1604.7358 | 13 | 0.2 | 803.3754 | 2 | 39.54 | 7 | F7:13344 | 27122019\_RID\_1315\_NaNaWb\_F6.raw |  |  |  |  |  |  | 3.99E6 | 0 |  |  | 2 | 0 | 0 | 0 | 0 | 0 | 0 | 1 | 1 | 0 | 0 | 200 | 212 |  |
| R.LASYLDKVQALEEANNDLENK.I | Y | 56.61 | 2376.1809 | 21 | 1.2 | 793.0685 | 3 | 45.12 | 8 | F8:11063 | 27122019\_RID\_1315\_NaNaWb\_F7.raw |  |  |  |  |  |  |  | 1.14E6 |  |  | 1 | 0 | 0 | 0 | 0 | 0 | 0 | 0 | 1 | 0 | 0 | 164 | 184 |  |
| R.QEIEC(+57.02)QNQEYSLLLSIK.M | Y | 55.73 | 2094.0303 | 17 | -0.9 | 1048.0215 | 2 | 53.15 | 8 | F8:12910 | 27122019\_RID\_1315\_NaNaWb\_F7.raw |  |  |  |  |  |  |  | 5.02E5 | 4.41E6 |  | 2 | 0 | 0 | 0 | 0 | 0 | 0 | 0 | 1 | 1 | 0 | 428 | 444 | Carbamidomethylation |
| K.STMQELNSR.L | Y | 52.24 | 1064.4921 | 9 | -0.4 | 533.2531 | 2 | 12.37 | 8 | F8:2154 | 27122019\_RID\_1315\_NaNaWb\_F7.raw |  |  |  |  |  |  |  | 2.8E6 |  |  | 1 | 0 | 0 | 0 | 0 | 0 | 0 | 0 | 1 | 0 | 0 | 155 | 163 |  |
| K.IQDWYDKK.G | Y | 51.30 | 1094.5397 | 8 | 1.5 | 548.2780 | 2 | 21.39 | 1 | F1:6491 | 09012020\_NaNaWb\_F4.raw | 8.28E4 |  |  |  |  |  |  |  |  |  | 1 | 1 | 0 | 0 | 0 | 0 | 0 | 0 | 0 | 0 | 0 | 185 | 192 |  |
| R.QEYEQLIAK.N | Y | 50.46 | 1120.5764 | 9 | 0.3 | 561.2957 | 2 | 34.91 | 1 | F1:14602 | 09012020\_NaNaWb\_F4.raw | 1.65E6 |  |  | 7.31E5 |  |  |  |  |  |  | 3 | 2 | 0 | 0 | 1 | 0 | 0 | 0 | 0 | 0 | 0 | 328 | 336 |  |
| R.M(+15.99)TLDDFR.I | Y | 49.53 | 912.4011 | 7 | -0.2 | 457.2078 | 2 | 31.59 | 1 | F1:12593 | 09012020\_NaNaWb\_F4.raw | 4.83E5 |  |  |  |  |  |  |  |  |  | 2 | 2 | 0 | 0 | 0 | 0 | 0 | 0 | 0 | 0 | 0 | 234 | 240 | Oxidation (M) |
| R.MTLDDFR.I | Y | 48.79 | 896.4062 | 7 | -0.7 | 449.2101 | 2 | 45.19 | 1 | F1:21718 | 09012020\_NaNaWb\_F4.raw | 1.71E5 |  |  |  |  |  | 0 |  |  |  | 2 | 1 | 0 | 0 | 0 | 0 | 0 | 1 | 0 | 0 | 0 | 234 | 240 |  |
| L.GGFGGGAGGGDGGILTANEK.S | Y | 48.69 | 1690.7910 | 20 | 0.0 | 846.4028 | 2 | 32.53 | 7 | F7:10695 | 27122019\_RID\_1315\_NaNaWb\_F6.raw |  |  |  |  |  |  | 0 |  |  |  | 1 | 0 | 0 | 0 | 0 | 0 | 0 | 1 | 0 | 0 | 0 | 135 | 154 |  |
| Y.SYGGGSGGGFSASSLGGGFGGGSR.G | Y | 46.93 | 2021.8827 | 24 | -1.2 | 1011.9474 | 2 | 37.53 | 2 | F2:9193 | 27122019\_RID\_1315\_NaNaWb\_F1.raw |  | 1.41E5 |  |  |  |  | 4.64E6 |  |  |  | 2 | 0 | 1 | 0 | 0 | 0 | 0 | 1 | 0 | 0 | 0 | 72 | 95 |  |
| R.LASYLDK.V | N | 46.86 | 808.4330 | 7 | -0.5 | 405.2236 | 2 | 24.16 | 1 | F1:8330 | 09012020\_NaNaWb\_F4.raw | 1.42E6 |  |  |  |  | 2.35E5 |  |  |  |  | 2 | 1 | 0 | 0 | 0 | 0 | 1 | 0 | 0 | 0 | 0 | 164 | 170 |  |
| K.TLLDIDNTR.M | Y | 44.94 | 1059.5560 | 9 | 0.9 | 530.7858 | 2 | 43.33 | 1 | F1:20564 | 09012020\_NaNaWb\_F4.raw | 2.4E6 | 9.26E6 |  |  |  |  |  |  |  |  | 3 | 2 | 1 | 0 | 0 | 0 | 0 | 0 | 0 | 0 | 0 | 225 | 233 |  |
| total 19 peptides |  |  |  |  |  |  |  |  |  |  |  |  |  |  |  |  |  |  |  |  |  |  |  |  |  |  |  |  |  |  |  |  |  |  |  |
| --- | --- | --- | --- | --- | --- | --- | --- | --- | --- | --- | --- | --- | --- | --- | --- | --- | --- | --- | --- | --- | --- | --- | --- | --- | --- | --- | --- | --- | --- | --- | --- | --- | --- | --- | --- |

P35908|K22E\_HUMAN

back to list

  

| Protein Coverage
| Supporting Peptides
|

Protein Coverage:

Supporting Peptides:

| Peptide | Uniq | -10lgP | Mass | Length | ppm | m/z | z | RT | Fraction | Scan | Source File | Area F4 | Area F1 | Area F10 | Area F2 | Area F3 | Area F5 | Area F6 | Area F7 | Area F8 | Area F9 | #Feature | #Feature F4 | #Feature F1 | #Feature F10 | #Feature F2 | #Feature F3 | #Feature F5 | #Feature F6 | #Feature F7 | #Feature F8 | #Feature F9 | Start | End | PTM |
| --- | --- | --- | --- | --- | --- | --- | --- | --- | --- | --- | --- | --- | --- | --- | --- | --- | --- | --- | --- | --- | --- | --- | --- | --- | --- | --- | --- | --- | --- | --- | --- | --- | --- | --- | --- |
| R.GFSSGSAVVSGGSR.R | N | 87.03 | 1253.6000 | 14 | 0.2 | 627.8074 | 2 | 17.40 | 7 | F7:4012 | 27122019\_RID\_1315\_NaNaWb\_F6.raw |  | 0 |  |  |  | 7.19E4 | 3.16E6 |  | 2.26E5 |  | 4 | 0 | 1 | 0 | 0 | 0 | 1 | 1 | 0 | 1 | 0 | 21 | 34 |  |
| K.NVQDAIADAEQR.G | Y | 84.59 | 1328.6321 | 12 | 0.3 | 665.3235 | 2 | 37.00 | 1 | F1:15970 | 09012020\_NaNaWb\_F4.raw | 1.4E6 | 1.02E6 |  |  |  |  | 1.78E7 |  |  |  | 3 | 1 | 1 | 0 | 0 | 0 | 0 | 1 | 0 | 0 | 0 | 419 | 430 |  |
| R.FLEQQNQVLQTK.W | N | 77.68 | 1474.7780 | 12 | 0.6 | 738.3967 | 2 | 37.47 | 1 | F1:16185 | 09012020\_NaNaWb\_F4.raw | 5.37E6 |  |  |  |  | 2.57E6 | 6.32E7 |  | 9.44E6 | 3E6 | 6 | 1 | 0 | 0 | 0 | 0 | 1 | 2 | 0 | 1 | 1 | 198 | 209 |  |
| R.HGGGGGGFGGGGFGSR.S | Y | 70.94 | 1319.5754 | 16 | -0.2 | 440.8657 | 3 | 12.51 | 7 | F7:2245 | 27122019\_RID\_1315\_NaNaWb\_F6.raw | 4.01E5 |  |  |  |  |  | 9.24E5 |  |  |  | 3 | 2 | 0 | 0 | 0 | 0 | 0 | 1 | 0 | 0 | 0 | 46 | 61 |  |
| R.TAAENDFVTLK.K | Y | 68.83 | 1207.6084 | 11 | 0.7 | 604.8119 | 2 | 40.77 | 1 | F1:18380 | 09012020\_NaNaWb\_F4.raw | 1.8E6 | 1.55E6 |  |  |  | 3.08E5 | 1.34E7 |  |  |  | 4 | 1 | 1 | 0 | 0 | 0 | 1 | 1 | 0 | 0 | 0 | 276 | 286 |  |
| K.YEELQVTVGR.H | Y | 68.82 | 1192.6088 | 10 | 0.2 | 597.3118 | 2 | 29.46 | 7 | F7:9548 | 27122019\_RID\_1315\_NaNaWb\_F6.raw | 1.12E5 |  |  |  |  |  | 1.08E7 |  |  |  | 2 | 1 | 0 | 0 | 0 | 0 | 0 | 1 | 0 | 0 | 0 | 375 | 384 |  |
| S.VAGGGGGFGAAGGFGGR.G | Y | 68.16 | 1350.6428 | 17 | 1.2 | 676.3295 | 2 | 36.72 | 1 | F1:15872 | 09012020\_NaNaWb\_F4.raw | 2.64E5 |  |  |  |  |  |  |  |  |  | 1 | 1 | 0 | 0 | 0 | 0 | 0 | 0 | 0 | 0 | 0 | 76 | 92 |  |
| K.LNDLEEALQQAK.E | Y | 64.39 | 1370.7041 | 12 | -0.6 | 686.3589 | 2 | 52.25 | 1 | F1:26623 | 09012020\_NaNaWb\_F4.raw | 7E5 |  |  |  |  |  | 2.23E7 |  |  |  | 3 | 2 | 0 | 0 | 0 | 0 | 0 | 1 | 0 | 0 | 0 | 442 | 453 |  |
| R.YLDGLTAER.T | Y | 63.80 | 1036.5189 | 9 | 0.7 | 519.2671 | 2 | 27.79 | 7 | F7:8841 | 27122019\_RID\_1315\_NaNaWb\_F6.raw |  |  |  |  |  |  | 1.18E7 |  |  |  | 1 | 0 | 0 | 0 | 0 | 0 | 0 | 1 | 0 | 0 | 0 | 239 | 247 |  |
| F.SGGGFGGGGFGGGR.F | Y | 55.47 | 1125.4951 | 14 | 0.3 | 563.7550 | 2 | 28.14 | 1 | F1:10269 | 09012020\_NaNaWb\_F4.raw | 2.15E5 |  |  |  |  |  |  |  |  |  | 1 | 1 | 0 | 0 | 0 | 0 | 0 | 0 | 0 | 0 | 0 | 109 | 122 |  |
| K.VDLLNQEIEFLK.V | Y | 53.00 | 1459.7922 | 12 | 0.7 | 730.9039 | 2 | 59.79 | 9 | F9:23218 | 27122019\_RID\_1315\_NaNaWb\_F8.raw |  |  |  |  |  |  |  | 0 | 5.35E6 |  | 2 | 0 | 0 | 0 | 0 | 0 | 0 | 0 | 1 | 1 | 0 | 303 | 314 |  |
| K.VDPEIQNVK.A | N | 50.90 | 1040.5502 | 9 | 0.4 | 521.2826 | 2 | 23.90 | 1 | F1:7945 | 09012020\_NaNaWb\_F4.raw | 5.17E5 |  |  |  |  |  |  |  |  |  | 1 | 1 | 0 | 0 | 0 | 0 | 0 | 0 | 0 | 0 | 0 | 167 | 175 |  |
| R.GGSGGGGSISGGGYGSGGGSGGR.Y | Y | 49.52 | 1740.7411 | 23 | 0.3 | 871.3781 | 2 | 13.03 | 1 | F1:2345 | 09012020\_NaNaWb\_F4.raw | 4.82E4 |  |  |  |  |  |  |  |  |  | 1 | 1 | 0 | 0 | 0 | 0 | 0 | 0 | 0 | 0 | 0 | 550 | 572 |  |
| K.FASFIDK.V | N | 45.32 | 826.4225 | 7 | -0.7 | 414.2182 | 2 | 39.06 | 1 | F1:17107 | 09012020\_NaNaWb\_F4.raw | 1.07E6 |  |  |  |  | 1.59E5 |  |  |  |  | 2 | 1 | 0 | 0 | 0 | 0 | 1 | 0 | 0 | 0 | 0 | 189 | 195 |  |
| K.IEISELNR.V | N | 44.99 | 972.5240 | 8 | -0.1 | 487.2692 | 2 | 26.67 | 8 | F8:5869 | 27122019\_RID\_1315\_NaNaWb\_F7.raw |  |  |  |  |  |  |  | 4.09E6 |  |  | 1 | 0 | 0 | 0 | 0 | 0 | 0 | 0 | 1 | 0 | 0 | 394 | 401 |  |
| K.LALDVEIATYR.K | N | 44.57 | 1262.6870 | 11 | 0.9 | 632.3514 | 2 | 47.00 | 9 | F9:15558 | 27122019\_RID\_1315\_NaNaWb\_F8.raw |  |  |  |  |  |  |  |  | 7.18E5 |  | 1 | 0 | 0 | 0 | 0 | 0 | 0 | 0 | 0 | 1 | 0 | 471 | 481 |  |
| R.STSSFSC(+57.02)LSR.H | Y | 42.22 | 1130.5026 | 10 | 0.1 | 566.2586 | 2 | 19.99 | 6 | F6:5315 | 27122019\_RID\_1315\_NaNaWb\_F5.raw |  |  |  |  |  | 5.9E4 |  |  |  |  | 1 | 0 | 0 | 0 | 0 | 0 | 1 | 0 | 0 | 0 | 0 | 36 | 45 | Carbamidomethylation |
| R.GSSSGGGYSSGSSSYGSGGR.Q | Y | 41.25 | 1739.6982 | 20 | 1.0 | 870.8573 | 2 | 12.51 | 1 | F1:2073 | 09012020\_NaNaWb\_F4.raw | 3.36E4 |  |  |  |  |  |  |  |  |  | 1 | 1 | 0 | 0 | 0 | 0 | 0 | 0 | 0 | 0 | 0 | 525 | 544 |  |
| total 18 peptides |  |  |  |  |  |  |  |  |  |  |  |  |  |  |  |  |  |  |  |  |  |  |  |  |  |  |  |  |  |  |  |  |  |  |  |
| --- | --- | --- | --- | --- | --- | --- | --- | --- | --- | --- | --- | --- | --- | --- | --- | --- | --- | --- | --- | --- | --- | --- | --- | --- | --- | --- | --- | --- | --- | --- | --- | --- | --- | --- | --- |

P25498|PA2AE\_NAJOX

back to list

  

| Protein Coverage
| Supporting Peptides
|

Protein Coverage:

Supporting Peptides:

| Peptide | Uniq | -10lgP | Mass | Length | ppm | m/z | z | RT | Fraction | Scan | Source File | Area F4 | Area F1 | Area F10 | Area F2 | Area F3 | Area F5 | Area F6 | Area F7 | Area F8 | Area F9 | #Feature | #Feature F4 | #Feature F1 | #Feature F10 | #Feature F2 | #Feature F3 | #Feature F5 | #Feature F6 | #Feature F7 | #Feature F8 | #Feature F9 | Start | End | PTM |
| --- | --- | --- | --- | --- | --- | --- | --- | --- | --- | --- | --- | --- | --- | --- | --- | --- | --- | --- | --- | --- | --- | --- | --- | --- | --- | --- | --- | --- | --- | --- | --- | --- | --- | --- | --- |
| K.TYSYEC(+57.02)SQGTLTC(+57.02)K.G | N | 103.55 | 1696.7073 | 14 | 0.3 | 849.3611 | 2 | 22.71 | 7 | F7:6516 | 27122019\_RID\_1315\_NaNaWb\_F6.raw |  |  | 7.08E6 |  |  | 0 | 1.39E10 | 5.85E8 |  | 5.84E6 | 32 | 0 | 0 | 1 | 0 | 0 | 1 | 25 | 4 | 0 | 1 | 66 | 79 | Carbamidomethylation |
| Y.SYEC(+57.02)SQGTLTC(+57.02)K.G | N | 77.43 | 1432.5963 | 12 | 0.2 | 717.3055 | 2 | 12.79 | 7 | F7:2393 | 27122019\_RID\_1315\_NaNaWb\_F6.raw |  |  |  |  |  |  | 6.81E7 |  |  |  | 1 | 0 | 0 | 0 | 0 | 0 | 0 | 1 | 0 | 0 | 0 | 68 | 79 | Carbamidomethylation |
| K.TYSYEC(+57.02)SQGTLTC(+57.02)KG.D | N | 77.40 | 1753.7288 | 15 | 0.2 | 877.8718 | 2 | 21.30 | 7 | F7:5936 | 27122019\_RID\_1315\_NaNaWb\_F6.raw |  |  |  |  |  |  | 8.76E8 |  |  |  | 2 | 0 | 0 | 0 | 0 | 0 | 0 | 2 | 0 | 0 | 0 | 66 | 80 | Carbamidomethylation |
| K.ISGC(+57.02)WPYFK.T | N | 76.65 | 1156.5375 | 9 | -0.2 | 579.2759 | 2 | 44.67 | 7 | F7:15552 | 27122019\_RID\_1315\_NaNaWb\_F6.raw |  |  |  |  |  |  | 1.23E10 | 3.34E8 |  |  | 10 | 0 | 0 | 0 | 0 | 0 | 0 | 9 | 1 | 0 | 0 | 57 | 65 | Carbamidomethylation |
| R.GGSGTPVDDLDR.C | N | 76.07 | 1187.5417 | 12 | 0.1 | 594.7782 | 2 | 22.36 | 7 | F7:6369 | 27122019\_RID\_1315\_NaNaWb\_F6.raw |  |  |  |  |  |  | 5.85E9 | 4.29E8 |  |  | 9 | 0 | 0 | 0 | 0 | 0 | 0 | 6 | 3 | 0 | 0 | 31 | 42 |  |
| K.TYSYEC(+57.02)SQGTLTC(+57.02)KGDNNSC(+57.02)AASVC(+57.02)DC(+57.02)DR.L | Y | 68.55 | 3378.2952 | 29 | -0.5 | 1127.1051 | 3 | 20.78 | 7 | F7:5569 | 27122019\_RID\_1315\_NaNaWb\_F6.raw |  |  |  |  |  |  | 2.36E7 |  |  |  | 1 | 0 | 0 | 0 | 0 | 0 | 0 | 1 | 0 | 0 | 0 | 66 | 94 | Carbamidomethylation |
| R.LAAIC(+57.02)FAGAPYN.N | N | 63.10 | 1266.6067 | 12 | -0.7 | 634.3102 | 2 | 53.56 | 7 | F7:19226 | 27122019\_RID\_1315\_NaNaWb\_F6.raw |  |  |  |  |  |  | 1.15E8 |  |  |  | 1 | 0 | 0 | 0 | 0 | 0 | 0 | 1 | 0 | 0 | 0 | 95 | 106 | Carbamidomethylation |
| I.SGC(+57.02)WPYFK.T | N | 50.79 | 1043.4535 | 8 | -0.3 | 522.7339 | 2 | 36.89 | 7 | F7:12451 | 27122019\_RID\_1315\_NaNaWb\_F6.raw |  |  |  |  |  |  | 1.32E7 |  |  |  | 1 | 0 | 0 | 0 | 0 | 0 | 0 | 1 | 0 | 0 | 0 | 58 | 65 | Carbamidomethylation |
| T.YSYEC(+57.02)SQGTLTC(+57.02)K.G | N | 48.60 | 1595.6595 | 13 | 0.2 | 798.8372 | 2 | 20.44 | 7 | F7:5339 | 27122019\_RID\_1315\_NaNaWb\_F6.raw |  |  |  |  |  |  | 5.1E6 |  |  |  | 1 | 0 | 0 | 0 | 0 | 0 | 0 | 1 | 0 | 0 | 0 | 67 | 79 | Carbamidomethylation |
| G.GSGTPVDDLDR.C | N | 47.11 | 1130.5204 | 11 | 0.2 | 566.2676 | 2 | 21.06 | 7 | F7:5676 | 27122019\_RID\_1315\_NaNaWb\_F6.raw |  |  |  |  |  |  | 3.97E7 |  |  |  | 1 | 0 | 0 | 0 | 0 | 0 | 0 | 1 | 0 | 0 | 0 | 32 | 42 |  |
| total 10 peptides |  |  |  |  |  |  |  |  |  |  |  |  |  |  |  |  |  |  |  |  |  |  |  |  |  |  |  |  |  |  |  |  |  |  |  |
| --- | --- | --- | --- | --- | --- | --- | --- | --- | --- | --- | --- | --- | --- | --- | --- | --- | --- | --- | --- | --- | --- | --- | --- | --- | --- | --- | --- | --- | --- | --- | --- | --- | --- | --- | --- |

P02768|ALBU\_HUMAN

back to list

  

| Protein Coverage
| Supporting Peptides
|

Protein Coverage:

Supporting Peptides:

| Peptide | Uniq | -10lgP | Mass | Length | ppm | m/z | z | RT | Fraction | Scan | Source File | Area F4 | Area F1 | Area F10 | Area F2 | Area F3 | Area F5 | Area F6 | Area F7 | Area F8 | Area F9 | #Feature | #Feature F4 | #Feature F1 | #Feature F10 | #Feature F2 | #Feature F3 | #Feature F5 | #Feature F6 | #Feature F7 | #Feature F8 | #Feature F9 | Start | End | PTM |
| --- | --- | --- | --- | --- | --- | --- | --- | --- | --- | --- | --- | --- | --- | --- | --- | --- | --- | --- | --- | --- | --- | --- | --- | --- | --- | --- | --- | --- | --- | --- | --- | --- | --- | --- | --- |
| K.KVPQVSTPTLVEVSR.N | N | 77.70 | 1638.9304 | 15 | -0.1 | 547.3174 | 3 | 30.00 | 6 | F6:11514 | 27122019\_RID\_1315\_NaNaWb\_F5.raw | 1.18E5 |  |  |  |  | 4.42E5 |  |  |  |  | 2 | 1 | 0 | 0 | 0 | 0 | 1 | 0 | 0 | 0 | 0 | 438 | 452 |  |
| R.ETYGEMADC(+57.02)C(+57.02)AK.Q | Y | 62.70 | 1433.5261 | 12 | 2.1 | 717.7719 | 2 | 24.60 | 1 | F1:8362 | 09012020\_NaNaWb\_F4.raw | 1.22E5 |  |  |  |  | 8.05E4 |  |  |  |  | 2 | 1 | 0 | 0 | 0 | 0 | 1 | 0 | 0 | 0 | 0 | 106 | 117 | Carbamidomethylation |
| K.TC(+57.02)VADESAENC(+57.02)DK.S | N | 62.46 | 1497.5712 | 13 | 0.5 | 749.7932 | 2 | 12.49 | 1 | F1:2052 | 09012020\_NaNaWb\_F4.raw | 1.52E5 |  |  |  |  |  |  |  |  |  | 1 | 1 | 0 | 0 | 0 | 0 | 0 | 0 | 0 | 0 | 0 | 76 | 88 | Carbamidomethylation |
| K.AAFTEC(+57.02)C(+57.02)QAADK.A | Y | 61.95 | 1370.5594 | 12 | 0.5 | 686.2874 | 2 | 20.05 | 1 | F1:5668 | 09012020\_NaNaWb\_F4.raw | 6.84E5 |  |  | 4.69E5 |  | 5.38E5 | 4.47E5 | 0 |  |  | 5 | 1 | 0 | 0 | 1 | 0 | 1 | 1 | 1 | 0 | 0 | 187 | 198 | Carbamidomethylation |
| K.YIC(+57.02)ENQDSISSK.L | Y | 61.01 | 1442.6348 | 12 | -0.1 | 722.3246 | 2 | 15.81 | 8 | F8:2865 | 27122019\_RID\_1315\_NaNaWb\_F7.raw |  |  |  |  |  | 7.28E5 |  | 7.05E5 |  |  | 2 | 0 | 0 | 0 | 0 | 0 | 1 | 0 | 1 | 0 | 0 | 287 | 298 | Carbamidomethylation |
| K.AEFAEVSK.L | Y | 60.24 | 879.4338 | 8 | 1.0 | 440.7246 | 2 | 14.58 | 6 | F6:3053 | 27122019\_RID\_1315\_NaNaWb\_F5.raw |  |  |  |  |  | 4.61E5 |  |  |  |  | 2 | 0 | 0 | 0 | 0 | 0 | 2 | 0 | 0 | 0 | 0 | 250 | 257 |  |
| K.DDNPNLPR.L | Y | 57.95 | 939.4410 | 8 | 0.6 | 470.7280 | 2 | 21.25 | 1 | F1:6349 | 09012020\_NaNaWb\_F4.raw | 7.71E5 |  |  |  |  | 4.51E5 |  |  |  |  | 2 | 1 | 0 | 0 | 0 | 0 | 1 | 0 | 0 | 0 | 0 | 131 | 138 |  |
| R.FKDLGEENFK.A | Y | 57.60 | 1225.5979 | 10 | 0.8 | 409.5403 | 3 | 20.58 | 6 | F6:5651 | 27122019\_RID\_1315\_NaNaWb\_F5.raw | 2.88E5 |  |  |  |  | 8.06E4 |  |  |  |  | 3 | 2 | 0 | 0 | 0 | 0 | 1 | 0 | 0 | 0 | 0 | 35 | 44 |  |
| K.LVNEVTEFAK.T | N | 56.41 | 1148.6077 | 10 | 0.7 | 575.3115 | 2 | 31.09 | 4 | F4:6431 | 27122019\_RID\_1315\_NaNaWb\_F2.raw | 1.9E6 |  |  | 0 |  | 8.54E5 |  |  |  |  | 3 | 1 | 0 | 0 | 1 | 0 | 1 | 0 | 0 | 0 | 0 | 66 | 75 |  |
| K.QTALVELVK.H | Y | 54.95 | 999.5964 | 9 | 0.5 | 500.8058 | 2 | 45.88 | 1 | F1:22241 | 09012020\_NaNaWb\_F4.raw | 1.33E5 |  |  |  |  |  |  |  |  |  | 1 | 1 | 0 | 0 | 0 | 0 | 0 | 0 | 0 | 0 | 0 | 550 | 558 |  |
| K.KQTALVELVK.H | Y | 54.68 | 1127.6914 | 10 | 0.6 | 564.8533 | 2 | 31.28 | 1 | F1:12362 | 09012020\_NaNaWb\_F4.raw | 9.72E4 |  |  |  |  |  |  |  |  |  | 1 | 1 | 0 | 0 | 0 | 0 | 0 | 0 | 0 | 0 | 0 | 549 | 558 |  |
| K.YLYEIAR.R | N | 52.67 | 926.4861 | 7 | -0.5 | 464.2501 | 2 | 39.36 | 1 | F1:17351 | 09012020\_NaNaWb\_F4.raw | 8.61E5 |  |  |  |  |  |  |  |  |  | 1 | 1 | 0 | 0 | 0 | 0 | 0 | 0 | 0 | 0 | 0 | 162 | 168 |  |
| K.TYETTLEK.C | Y | 52.49 | 983.4811 | 8 | 0.4 | 492.7480 | 2 | 20.47 | 1 | F1:5935 | 09012020\_NaNaWb\_F4.raw | 0 |  |  |  |  |  |  |  |  |  | 1 | 1 | 0 | 0 | 0 | 0 | 0 | 0 | 0 | 0 | 0 | 376 | 383 |  |
| K.FQNALLVR.Y | N | 48.58 | 959.5552 | 8 | 0.4 | 480.7851 | 2 | 30.76 | 8 | F8:7006 | 27122019\_RID\_1315\_NaNaWb\_F7.raw | 1.34E6 |  |  |  |  | 6.05E5 |  | 8.37E5 |  |  | 4 | 2 | 0 | 0 | 0 | 0 | 1 | 0 | 1 | 0 | 0 | 427 | 434 |  |
| K.LC(+57.02)TVATLR.E | Y | 46.08 | 932.5114 | 8 | 0.1 | 467.2630 | 2 | 21.80 | 6 | F6:6304 | 27122019\_RID\_1315\_NaNaWb\_F5.raw | 2.69E6 |  |  |  |  | 9.21E5 |  |  |  |  | 2 | 1 | 0 | 0 | 0 | 0 | 1 | 0 | 0 | 0 | 0 | 98 | 105 | Carbamidomethylation |
| K.SLHTLFGDK.L | N | 45.10 | 1016.5291 | 9 | 0.6 | 509.2721 | 2 | 22.98 | 6 | F6:6938 | 27122019\_RID\_1315\_NaNaWb\_F5.raw | 6.19E4 |  |  |  |  | 6.91E4 |  |  |  |  | 2 | 1 | 0 | 0 | 0 | 0 | 1 | 0 | 0 | 0 | 0 | 89 | 97 |  |
| K.LVAASQAALGL | Y | 44.99 | 1012.5917 | 11 | 0.6 | 507.3034 | 2 | 48.45 | 10 | F10:12407 | 27122019\_RID\_1315\_NaNaWb\_F9.raw | 1.89E6 |  |  |  |  |  |  |  |  | 0 | 2 | 1 | 0 | 0 | 0 | 0 | 0 | 0 | 0 | 0 | 1 | 599 | 609 |  |
| K.LVTDLTK.V | N | 42.68 | 788.4644 | 7 | -0.3 | 395.2393 | 2 | 19.85 | 6 | F6:5211 | 27122019\_RID\_1315\_NaNaWb\_F5.raw | 1.56E6 |  |  |  |  | 5.19E5 |  |  |  |  | 2 | 1 | 0 | 0 | 0 | 0 | 1 | 0 | 0 | 0 | 0 | 258 | 264 |  |
| total 18 peptides |  |  |  |  |  |  |  |  |  |  |  |  |  |  |  |  |  |  |  |  |  |  |  |  |  |  |  |  |  |  |  |  |  |  |  |
| --- | --- | --- | --- | --- | --- | --- | --- | --- | --- | --- | --- | --- | --- | --- | --- | --- | --- | --- | --- | --- | --- | --- | --- | --- | --- | --- | --- | --- | --- | --- | --- | --- | --- | --- | --- |

#CONTAM#ALBU\_HUMAN|

back to list

  

| Protein Coverage
| Supporting Peptides
|

Protein Coverage:

Supporting Peptides:

| Peptide | Uniq | -10lgP | Mass | Length | ppm | m/z | z | RT | Fraction | Scan | Source File | Area F4 | Area F1 | Area F10 | Area F2 | Area F3 | Area F5 | Area F6 | Area F7 | Area F8 | Area F9 | #Feature | #Feature F4 | #Feature F1 | #Feature F10 | #Feature F2 | #Feature F3 | #Feature F5 | #Feature F6 | #Feature F7 | #Feature F8 | #Feature F9 | Start | End | PTM |
| --- | --- | --- | --- | --- | --- | --- | --- | --- | --- | --- | --- | --- | --- | --- | --- | --- | --- | --- | --- | --- | --- | --- | --- | --- | --- | --- | --- | --- | --- | --- | --- | --- | --- | --- | --- |
| K.KVPQVSTPTLVEVSR.N | N | 77.70 | 1638.9304 | 15 | -0.1 | 547.3174 | 3 | 30.00 | 6 | F6:11514 | 27122019\_RID\_1315\_NaNaWb\_F5.raw | 1.18E5 |  |  |  |  | 4.42E5 |  |  |  |  | 2 | 1 | 0 | 0 | 0 | 0 | 1 | 0 | 0 | 0 | 0 | 438 | 452 |  |
| R.ETYGEMADC(+57.02)C(+57.02)AK.Q | Y | 62.70 | 1433.5261 | 12 | 2.1 | 717.7719 | 2 | 24.60 | 1 | F1:8362 | 09012020\_NaNaWb\_F4.raw | 1.22E5 |  |  |  |  | 8.05E4 |  |  |  |  | 2 | 1 | 0 | 0 | 0 | 0 | 1 | 0 | 0 | 0 | 0 | 106 | 117 | Carbamidomethylation |
| K.TC(+57.02)VADESAENC(+57.02)DK.S | N | 62.46 | 1497.5712 | 13 | 0.5 | 749.7932 | 2 | 12.49 | 1 | F1:2052 | 09012020\_NaNaWb\_F4.raw | 1.52E5 |  |  |  |  |  |  |  |  |  | 1 | 1 | 0 | 0 | 0 | 0 | 0 | 0 | 0 | 0 | 0 | 76 | 88 | Carbamidomethylation |
| K.AAFTEC(+57.02)C(+57.02)QAADK.A | Y | 61.95 | 1370.5594 | 12 | 0.5 | 686.2874 | 2 | 20.05 | 1 | F1:5668 | 09012020\_NaNaWb\_F4.raw | 6.84E5 |  |  | 4.69E5 |  | 5.38E5 | 4.47E5 | 0 |  |  | 5 | 1 | 0 | 0 | 1 | 0 | 1 | 1 | 1 | 0 | 0 | 187 | 198 | Carbamidomethylation |
| K.YIC(+57.02)ENQDSISSK.L | Y | 61.01 | 1442.6348 | 12 | -0.1 | 722.3246 | 2 | 15.81 | 8 | F8:2865 | 27122019\_RID\_1315\_NaNaWb\_F7.raw |  |  |  |  |  | 7.28E5 |  | 7.05E5 |  |  | 2 | 0 | 0 | 0 | 0 | 0 | 1 | 0 | 1 | 0 | 0 | 287 | 298 | Carbamidomethylation |
| K.AEFAEVSK.L | Y | 60.24 | 879.4338 | 8 | 1.0 | 440.7246 | 2 | 14.58 | 6 | F6:3053 | 27122019\_RID\_1315\_NaNaWb\_F5.raw |  |  |  |  |  | 4.61E5 |  |  |  |  | 2 | 0 | 0 | 0 | 0 | 0 | 2 | 0 | 0 | 0 | 0 | 250 | 257 |  |
| K.DDNPNLPR.L | Y | 57.95 | 939.4410 | 8 | 0.6 | 470.7280 | 2 | 21.25 | 1 | F1:6349 | 09012020\_NaNaWb\_F4.raw | 7.71E5 |  |  |  |  | 4.51E5 |  |  |  |  | 2 | 1 | 0 | 0 | 0 | 0 | 1 | 0 | 0 | 0 | 0 | 131 | 138 |  |
| R.FKDLGEENFK.A | Y | 57.60 | 1225.5979 | 10 | 0.8 | 409.5403 | 3 | 20.58 | 6 | F6:5651 | 27122019\_RID\_1315\_NaNaWb\_F5.raw | 2.88E5 |  |  |  |  | 8.06E4 |  |  |  |  | 3 | 2 | 0 | 0 | 0 | 0 | 1 | 0 | 0 | 0 | 0 | 35 | 44 |  |
| K.LVNEVTEFAK.T | N | 56.41 | 1148.6077 | 10 | 0.7 | 575.3115 | 2 | 31.09 | 4 | F4:6431 | 27122019\_RID\_1315\_NaNaWb\_F2.raw | 1.9E6 |  |  | 0 |  | 8.54E5 |  |  |  |  | 3 | 1 | 0 | 0 | 1 | 0 | 1 | 0 | 0 | 0 | 0 | 66 | 75 |  |
| K.QTALVELVK.H | Y | 54.95 | 999.5964 | 9 | 0.5 | 500.8058 | 2 | 45.88 | 1 | F1:22241 | 09012020\_NaNaWb\_F4.raw | 1.33E5 |  |  |  |  |  |  |  |  |  | 1 | 1 | 0 | 0 | 0 | 0 | 0 | 0 | 0 | 0 | 0 | 550 | 558 |  |
| K.KQTALVELVK.H | Y | 54.68 | 1127.6914 | 10 | 0.6 | 564.8533 | 2 | 31.28 | 1 | F1:12362 | 09012020\_NaNaWb\_F4.raw | 9.72E4 |  |  |  |  |  |  |  |  |  | 1 | 1 | 0 | 0 | 0 | 0 | 0 | 0 | 0 | 0 | 0 | 549 | 558 |  |
| K.YLYEIAR.R | N | 52.67 | 926.4861 | 7 | -0.5 | 464.2501 | 2 | 39.36 | 1 | F1:17351 | 09012020\_NaNaWb\_F4.raw | 8.61E5 |  |  |  |  |  |  |  |  |  | 1 | 1 | 0 | 0 | 0 | 0 | 0 | 0 | 0 | 0 | 0 | 162 | 168 |  |
| K.TYETTLEK.C | Y | 52.49 | 983.4811 | 8 | 0.4 | 492.7480 | 2 | 20.47 | 1 | F1:5935 | 09012020\_NaNaWb\_F4.raw | 0 |  |  |  |  |  |  |  |  |  | 1 | 1 | 0 | 0 | 0 | 0 | 0 | 0 | 0 | 0 | 0 | 376 | 383 |  |
| K.FQNALLVR.Y | N | 48.58 | 959.5552 | 8 | 0.4 | 480.7851 | 2 | 30.76 | 8 | F8:7006 | 27122019\_RID\_1315\_NaNaWb\_F7.raw | 1.34E6 |  |  |  |  | 6.05E5 |  | 8.37E5 |  |  | 4 | 2 | 0 | 0 | 0 | 0 | 1 | 0 | 1 | 0 | 0 | 427 | 434 |  |
| K.LC(+57.02)TVATLR.E | Y | 46.08 | 932.5114 | 8 | 0.1 | 467.2630 | 2 | 21.80 | 6 | F6:6304 | 27122019\_RID\_1315\_NaNaWb\_F5.raw | 2.69E6 |  |  |  |  | 9.21E5 |  |  |  |  | 2 | 1 | 0 | 0 | 0 | 0 | 1 | 0 | 0 | 0 | 0 | 98 | 105 | Carbamidomethylation |
| K.SLHTLFGDK.L | N | 45.10 | 1016.5291 | 9 | 0.6 | 509.2721 | 2 | 22.98 | 6 | F6:6938 | 27122019\_RID\_1315\_NaNaWb\_F5.raw | 6.19E4 |  |  |  |  | 6.91E4 |  |  |  |  | 2 | 1 | 0 | 0 | 0 | 0 | 1 | 0 | 0 | 0 | 0 | 89 | 97 |  |
| K.LVAASQAALGL | Y | 44.99 | 1012.5917 | 11 | 0.6 | 507.3034 | 2 | 48.45 | 10 | F10:12407 | 27122019\_RID\_1315\_NaNaWb\_F9.raw | 1.89E6 |  |  |  |  |  |  |  |  | 0 | 2 | 1 | 0 | 0 | 0 | 0 | 0 | 0 | 0 | 0 | 1 | 599 | 609 |  |
| K.LVTDLTK.V | N | 42.68 | 788.4644 | 7 | -0.3 | 395.2393 | 2 | 19.85 | 6 | F6:5211 | 27122019\_RID\_1315\_NaNaWb\_F5.raw | 1.56E6 |  |  |  |  | 5.19E5 |  |  |  |  | 2 | 1 | 0 | 0 | 0 | 0 | 1 | 0 | 0 | 0 | 0 | 258 | 264 |  |
| total 18 peptides |  |  |  |  |  |  |  |  |  |  |  |  |  |  |  |  |  |  |  |  |  |  |  |  |  |  |  |  |  |  |  |  |  |  |  |
| --- | --- | --- | --- | --- | --- | --- | --- | --- | --- | --- | --- | --- | --- | --- | --- | --- | --- | --- | --- | --- | --- | --- | --- | --- | --- | --- | --- | --- | --- | --- | --- | --- | --- | --- | --- |

P13647|K2C5\_HUMAN

back to list

  

| Protein Coverage
| Supporting Peptides
|

Protein Coverage:

Supporting Peptides:

| Peptide | Uniq | -10lgP | Mass | Length | ppm | m/z | z | RT | Fraction | Scan | Source File | Area F4 | Area F1 | Area F10 | Area F2 | Area F3 | Area F5 | Area F6 | Area F7 | Area F8 | Area F9 | #Feature | #Feature F4 | #Feature F1 | #Feature F10 | #Feature F2 | #Feature F3 | #Feature F5 | #Feature F6 | #Feature F7 | #Feature F8 | #Feature F9 | Start | End | PTM |
| --- | --- | --- | --- | --- | --- | --- | --- | --- | --- | --- | --- | --- | --- | --- | --- | --- | --- | --- | --- | --- | --- | --- | --- | --- | --- | --- | --- | --- | --- | --- | --- | --- | --- | --- | --- |
| R.VSLAGAC(+57.02)GVGGYGSR.S | Y | 73.47 | 1409.6721 | 15 | 0.1 | 705.8434 | 2 | 27.08 | 7 | F7:8599 | 27122019\_RID\_1315\_NaNaWb\_F6.raw |  |  |  |  |  |  | 1.42E6 |  |  |  | 1 | 0 | 0 | 0 | 0 | 0 | 0 | 1 | 0 | 0 | 0 | 49 | 63 | Carbamidomethylation |
| K.NKYEDEINKR.T | N | 64.65 | 1307.6470 | 10 | 0.7 | 436.8899 | 3 | 12.47 | 1 | F1:2039 | 09012020\_NaNaWb\_F4.raw | 5.64E5 |  |  |  |  |  |  |  |  |  | 1 | 1 | 0 | 0 | 0 | 0 | 0 | 0 | 0 | 0 | 0 | 256 | 265 |  |
| K.LAELEEALQK.A | Y | 59.05 | 1142.6183 | 10 | 0.2 | 572.3165 | 2 | 43.85 | 1 | F1:20659 | 09012020\_NaNaWb\_F4.raw | 3.36E5 |  |  |  |  |  |  |  |  |  | 1 | 1 | 0 | 0 | 0 | 0 | 0 | 0 | 0 | 0 | 0 | 432 | 441 |  |
| K.YEELQQTAGR.H | N | 56.93 | 1193.5676 | 10 | 1.1 | 597.7917 | 2 | 20.05 | 1 | F1:5661 | 09012020\_NaNaWb\_F4.raw | 9.36E4 |  |  |  |  |  |  | 0 |  |  | 2 | 1 | 0 | 0 | 0 | 0 | 0 | 0 | 1 | 0 | 0 | 365 | 374 |  |
| R.ISISTSGGSFR.N | Y | 55.64 | 1110.5669 | 11 | -0.7 | 556.2903 | 2 | 26.47 | 8 | F8:5815 | 27122019\_RID\_1315\_NaNaWb\_F7.raw |  |  |  |  |  | 0 | 2.36E6 | 1.07E6 |  |  | 3 | 0 | 0 | 0 | 0 | 0 | 1 | 1 | 1 | 0 | 0 | 74 | 84 |  |
| R.TEAESWYQTK.Y | N | 54.05 | 1241.5564 | 10 | 0.0 | 621.7855 | 2 | 30.21 | 1 | F1:11451 | 09012020\_NaNaWb\_F4.raw | 1.12E5 |  |  |  |  | 2.25E4 |  |  |  |  | 2 | 1 | 0 | 0 | 0 | 0 | 1 | 0 | 0 | 0 | 0 | 355 | 364 |  |
| R.QNLEPLFEQYINNLR.R | N | 51.10 | 1889.9635 | 15 | -0.1 | 945.9890 | 2 | 65.20 | 9 | F9:25290 | 27122019\_RID\_1315\_NaNaWb\_F8.raw |  |  |  |  |  |  |  |  | 1.37E6 |  | 1 | 0 | 0 | 0 | 0 | 0 | 0 | 0 | 0 | 1 | 0 | 213 | 227 |  |
| R.SFSTASAITPSVSR.T | N | 46.23 | 1409.7151 | 14 | 0.6 | 705.8652 | 2 | 29.49 | 6 | F6:11175 | 27122019\_RID\_1315\_NaNaWb\_F5.raw |  |  |  |  |  | 5.39E4 |  |  |  |  | 1 | 0 | 0 | 0 | 0 | 0 | 1 | 0 | 0 | 0 | 0 | 16 | 29 |  |
| K.FASFIDK.V | N | 45.32 | 826.4225 | 7 | -0.7 | 414.2182 | 2 | 39.06 | 1 | F1:17107 | 09012020\_NaNaWb\_F4.raw | 1.07E6 |  |  |  |  | 1.59E5 |  |  |  |  | 2 | 1 | 0 | 0 | 0 | 0 | 1 | 0 | 0 | 0 | 0 | 179 | 185 |  |
| K.LALDVEIATYR.K | N | 44.57 | 1262.6870 | 11 | 0.9 | 632.3514 | 2 | 47.00 | 9 | F9:15558 | 27122019\_RID\_1315\_NaNaWb\_F8.raw |  |  |  |  |  |  |  |  | 7.18E5 |  | 1 | 0 | 0 | 0 | 0 | 0 | 0 | 0 | 0 | 1 | 0 | 461 | 471 |  |
| total 10 peptides |  |  |  |  |  |  |  |  |  |  |  |  |  |  |  |  |  |  |  |  |  |  |  |  |  |  |  |  |  |  |  |  |  |  |  |
| --- | --- | --- | --- | --- | --- | --- | --- | --- | --- | --- | --- | --- | --- | --- | --- | --- | --- | --- | --- | --- | --- | --- | --- | --- | --- | --- | --- | --- | --- | --- | --- | --- | --- | --- | --- |

P02533|K1C14\_HUMAN

back to list

  

| Protein Coverage
| Supporting Peptides
|

Protein Coverage:

Supporting Peptides:

| Peptide | Uniq | -10lgP | Mass | Length | ppm | m/z | z | RT | Fraction | Scan | Source File | Area F4 | Area F1 | Area F10 | Area F2 | Area F3 | Area F5 | Area F6 | Area F7 | Area F8 | Area F9 | #Feature | #Feature F4 | #Feature F1 | #Feature F10 | #Feature F2 | #Feature F3 | #Feature F5 | #Feature F6 | #Feature F7 | #Feature F8 | #Feature F9 | Start | End | PTM |
| --- | --- | --- | --- | --- | --- | --- | --- | --- | --- | --- | --- | --- | --- | --- | --- | --- | --- | --- | --- | --- | --- | --- | --- | --- | --- | --- | --- | --- | --- | --- | --- | --- | --- | --- | --- |
| R.LLEGEDAHLSSSQFSSGSQSSR.D | Y | 72.27 | 2308.0566 | 22 | -0.1 | 770.3594 | 3 | 25.93 | 7 | F7:8068 | 27122019\_RID\_1315\_NaNaWb\_F6.raw |  |  |  |  |  |  | 0 |  |  |  | 1 | 0 | 0 | 0 | 0 | 0 | 0 | 1 | 0 | 0 | 0 | 418 | 439 |  |
| R.APSTYGGGLSVSSSR.F | Y | 66.67 | 1424.6896 | 15 | -0.4 | 713.3518 | 2 | 21.62 | 7 | F7:5972 | 27122019\_RID\_1315\_NaNaWb\_F6.raw |  |  |  |  |  |  | 0 |  | 1.94E5 |  | 2 | 0 | 0 | 0 | 0 | 0 | 0 | 1 | 0 | 1 | 0 | 42 | 56 |  |
| R.ALEEANADLEVK.I | N | 63.59 | 1300.6510 | 12 | 0.5 | 651.3331 | 2 | 25.96 | 6 | F6:8824 | 27122019\_RID\_1315\_NaNaWb\_F5.raw |  |  |  |  |  | 1.64E5 |  |  | 1.47E6 |  | 2 | 0 | 0 | 0 | 0 | 0 | 1 | 0 | 0 | 1 | 0 | 135 | 146 |  |
| K.VTMQNLNDR.L | N | 58.10 | 1089.5237 | 9 | -0.1 | 545.7690 | 2 | 22.50 | 1 | F1:7071 | 09012020\_NaNaWb\_F4.raw | 3.69E5 |  |  |  |  |  |  |  |  |  | 1 | 1 | 0 | 0 | 0 | 0 | 0 | 0 | 0 | 0 | 0 | 117 | 125 |  |
| K.DAEEWFFTK.T | Y | 56.59 | 1171.5186 | 9 | 0.4 | 586.7668 | 2 | 67.41 | 1 | F1:36283 | 09012020\_NaNaWb\_F4.raw | 9.12E4 |  |  |  |  |  |  |  |  |  | 1 | 1 | 0 | 0 | 0 | 0 | 0 | 0 | 0 | 0 | 0 | 301 | 309 |  |
| R.LAADDFR.T | N | 50.17 | 806.3922 | 7 | 0.0 | 404.2034 | 2 | 27.69 | 1 | F1:10242 | 09012020\_NaNaWb\_F4.raw | 1.68E6 |  |  |  |  |  | 7.08E6 |  |  |  | 3 | 2 | 0 | 0 | 0 | 0 | 0 | 1 | 0 | 0 | 0 | 195 | 201 |  |
| R.LASYLDK.V | N | 46.86 | 808.4330 | 7 | -0.5 | 405.2236 | 2 | 24.16 | 1 | F1:8330 | 09012020\_NaNaWb\_F4.raw | 1.42E6 |  |  |  |  | 2.35E5 |  |  |  |  | 2 | 1 | 0 | 0 | 0 | 0 | 1 | 0 | 0 | 0 | 0 | 126 | 132 |  |
| R.LEQEIATYR.R | N | 46.77 | 1121.5717 | 9 | 0.4 | 561.7933 | 2 | 21.34 | 6 | F6:6049 | 27122019\_RID\_1315\_NaNaWb\_F5.raw | 1.86E5 |  |  |  |  | 1.35E5 |  |  |  |  | 2 | 1 | 0 | 0 | 0 | 0 | 1 | 0 | 0 | 0 | 0 | 408 | 416 |  |
| K.EELAYLK.K | N | 44.65 | 864.4592 | 7 | -0.2 | 433.2368 | 2 | 34.81 | 1 | F1:14659 | 09012020\_NaNaWb\_F4.raw | 0 |  |  |  |  |  |  |  |  |  | 1 | 1 | 0 | 0 | 0 | 0 | 0 | 0 | 0 | 0 | 0 | 244 | 250 |  |
| R.TKYETELNLR.M | Y | 44.57 | 1265.6615 | 10 | 0.2 | 422.8945 | 3 | 27.89 | 1 | F1:10196 | 09012020\_NaNaWb\_F4.raw | 0 |  |  |  |  |  |  |  |  |  | 1 | 1 | 0 | 0 | 0 | 0 | 0 | 0 | 0 | 0 | 0 | 202 | 211 |  |
| R.EVATNSELVQSGK.S | N | 44.36 | 1360.6833 | 13 | 1.3 | 681.3499 | 2 | 16.90 | 6 | F6:3843 | 27122019\_RID\_1315\_NaNaWb\_F5.raw |  |  |  |  |  | 2.54E4 |  |  |  |  | 1 | 0 | 0 | 0 | 0 | 0 | 1 | 0 | 0 | 0 | 0 | 316 | 328 |  |
| R.VLDELTLAR.A | N | 42.16 | 1028.5865 | 9 | 0.2 | 515.3007 | 2 | 37.25 | 9 | F9:11276 | 27122019\_RID\_1315\_NaNaWb\_F8.raw |  |  |  |  |  |  |  |  | 1.4E6 |  | 1 | 0 | 0 | 0 | 0 | 0 | 0 | 0 | 0 | 1 | 0 | 224 | 232 |  |
| total 12 peptides |  |  |  |  |  |  |  |  |  |  |  |  |  |  |  |  |  |  |  |  |  |  |  |  |  |  |  |  |  |  |  |  |  |  |  |
| --- | --- | --- | --- | --- | --- | --- | --- | --- | --- | --- | --- | --- | --- | --- | --- | --- | --- | --- | --- | --- | --- | --- | --- | --- | --- | --- | --- | --- | --- | --- | --- | --- | --- | --- | --- |

D5LMJ3|VM3A\_NAJAT

back to list

  

| Protein Coverage
| Supporting Peptides
|

Protein Coverage:

Supporting Peptides:

| Peptide | Uniq | -10lgP | Mass | Length | ppm | m/z | z | RT | Fraction | Scan | Source File | Area F4 | Area F1 | Area F10 | Area F2 | Area F3 | Area F5 | Area F6 | Area F7 | Area F8 | Area F9 | #Feature | #Feature F4 | #Feature F1 | #Feature F10 | #Feature F2 | #Feature F3 | #Feature F5 | #Feature F6 | #Feature F7 | #Feature F8 | #Feature F9 | Start | End | PTM |
| --- | --- | --- | --- | --- | --- | --- | --- | --- | --- | --- | --- | --- | --- | --- | --- | --- | --- | --- | --- | --- | --- | --- | --- | --- | --- | --- | --- | --- | --- | --- | --- | --- | --- | --- | --- |
| K.C(+57.02)GTLYC(+57.02)TEIK.K | Y | 80.44 | 1243.5577 | 10 | -0.4 | 622.7859 | 2 | 24.33 | 6 | F6:7686 | 27122019\_RID\_1315\_NaNaWb\_F5.raw | 3.63E5 |  |  |  |  | 6.62E6 |  | 6.41E6 |  |  | 4 | 1 | 0 | 0 | 0 | 0 | 2 | 0 | 1 | 0 | 0 | 560 | 569 | Carbamidomethylation |
| K.LQPHAQC(+57.02)DSEEC(+57.02)C(+57.02)EK.C | Y | 77.58 | 1889.7343 | 15 | 0.4 | 630.9189 | 3 | 12.44 | 1 | F1:2004 | 09012020\_NaNaWb\_F4.raw | 1.03E6 |  |  |  |  |  |  |  |  |  | 2 | 2 | 0 | 0 | 0 | 0 | 0 | 0 | 0 | 0 | 0 | 440 | 454 | Carbamidomethylation |
| K.TGC(+57.02)IVPVSPR.D | Y | 71.68 | 1084.5699 | 10 | -0.5 | 543.2920 | 2 | 34.09 | 1 | F1:14310 | 09012020\_NaNaWb\_F4.raw | 2.84E7 |  |  |  |  |  |  | 8.92E5 |  |  | 5 | 4 | 0 | 0 | 0 | 0 | 0 | 0 | 1 | 0 | 0 | 571 | 580 | Carbamidomethylation |
| N.YTVGPAGC(+57.02)FK.N | Y | 63.34 | 1098.5168 | 10 | 0.5 | 550.2660 | 2 | 25.69 | 6 | F6:8695 | 27122019\_RID\_1315\_NaNaWb\_F5.raw |  |  |  |  |  | 4.13E5 |  |  |  |  | 1 | 0 | 0 | 0 | 0 | 0 | 1 | 0 | 0 | 0 | 0 | 523 | 532 | Carbamidomethylation |
| R.VYEM(+15.99)VNYLNTK.Y | Y | 44.72 | 1388.6646 | 11 | 1.0 | 695.3403 | 2 | 27.99 | 8 | F8:6180 | 27122019\_RID\_1315\_NaNaWb\_F7.raw |  |  |  |  |  |  |  | 4.94E6 |  |  | 1 | 0 | 0 | 0 | 0 | 0 | 0 | 0 | 1 | 0 | 0 | 231 | 241 | Oxidation (M) |
| K.KTGC(+57.02)IVPVSPR.D | Y | 44.55 | 1212.6648 | 11 | 0.2 | 405.2290 | 3 | 22.87 | 1 | F1:7518 | 09012020\_NaNaWb\_F4.raw | 3.64E6 |  |  |  |  |  |  |  |  |  | 2 | 2 | 0 | 0 | 0 | 0 | 0 | 0 | 0 | 0 | 0 | 570 | 580 | Carbamidomethylation |
| total 6 peptides |  |  |  |  |  |  |  |  |  |  |  |  |  |  |  |  |  |  |  |  |  |  |  |  |  |  |  |  |  |  |  |  |  |  |  |
| --- | --- | --- | --- | --- | --- | --- | --- | --- | --- | --- | --- | --- | --- | --- | --- | --- | --- | --- | --- | --- | --- | --- | --- | --- | --- | --- | --- | --- | --- | --- | --- | --- | --- | --- | --- |

Q5YF89|NGFV2\_NAJSP

back to list

  

| Protein Coverage
| Supporting Peptides
|

Protein Coverage:

Supporting Peptides:

| Peptide | Uniq | -10lgP | Mass | Length | ppm | m/z | z | RT | Fraction | Scan | Source File | Area F4 | Area F1 | Area F10 | Area F2 | Area F3 | Area F5 | Area F6 | Area F7 | Area F8 | Area F9 | #Feature | #Feature F4 | #Feature F1 | #Feature F10 | #Feature F2 | #Feature F3 | #Feature F5 | #Feature F6 | #Feature F7 | #Feature F8 | #Feature F9 | Start | End | PTM |
| --- | --- | --- | --- | --- | --- | --- | --- | --- | --- | --- | --- | --- | --- | --- | --- | --- | --- | --- | --- | --- | --- | --- | --- | --- | --- | --- | --- | --- | --- | --- | --- | --- | --- | --- | --- |
| K.ALTMEGNQASWR.F | Y | 70.91 | 1362.6350 | 12 | -0.1 | 682.3247 | 2 | 28.76 | 8 | F8:6390 | 27122019\_RID\_1315\_NaNaWb\_F7.raw |  |  |  |  |  |  |  | 1.9E7 |  |  | 1 | 0 | 0 | 0 | 0 | 0 | 0 | 0 | 1 | 0 | 0 | 212 | 223 |  |
| K.GNTVTVM(+15.99)ENVNLDNK.V | Y | 67.92 | 1662.7883 | 15 | -0.2 | 832.4012 | 2 | 24.62 | 8 | F8:5190 | 27122019\_RID\_1315\_NaNaWb\_F7.raw |  |  |  |  |  |  |  | 9.77E7 |  |  | 2 | 0 | 0 | 0 | 0 | 0 | 0 | 0 | 2 | 0 | 0 | 156 | 170 | Oxidation (M) |
| K.GNTVTVMENVNLDNK.V | Y | 64.23 | 1646.7933 | 15 | -0.2 | 824.4037 | 2 | 33.85 | 8 | F8:7971 | 27122019\_RID\_1315\_NaNaWb\_F7.raw |  |  |  |  |  |  |  | 1.78E7 |  |  | 1 | 0 | 0 | 0 | 0 | 0 | 0 | 0 | 1 | 0 | 0 | 156 | 170 |  |
| K.ALTM(+15.99)EGNQASWR.F | Y | 53.28 | 1378.6299 | 12 | 0.3 | 690.3224 | 2 | 20.46 | 8 | F8:4040 | 27122019\_RID\_1315\_NaNaWb\_F7.raw |  |  |  |  |  |  |  | 9.06E7 |  |  | 1 | 0 | 0 | 0 | 0 | 0 | 0 | 0 | 1 | 0 | 0 | 212 | 223 | Oxidation (M) |
| R.EDHPVHNLGEHSVC(+57.02)DSVSAWVTK.T | Y | 53.14 | 2602.1870 | 23 | -0.3 | 651.5538 | 4 | 30.89 | 8 | F8:6972 | 27122019\_RID\_1315\_NaNaWb\_F7.raw |  |  |  |  |  |  |  | 3E8 | 1.66E6 |  | 5 | 0 | 0 | 0 | 0 | 0 | 0 | 0 | 4 | 1 | 0 | 126 | 148 | Carbamidomethylation |
| P.VHNLGEHSVC(+57.02)DSVSAWVTK.T | Y | 51.35 | 2124.0059 | 19 | 0.3 | 709.0095 | 3 | 30.99 | 8 | F8:7059 | 27122019\_RID\_1315\_NaNaWb\_F7.raw |  |  |  |  |  |  |  | 1.2E7 |  |  | 1 | 0 | 0 | 0 | 0 | 0 | 0 | 0 | 1 | 0 | 0 | 130 | 148 | Carbamidomethylation |
| R.IDTAC(+57.02)VC(+57.02)VITK.K | Y | 49.41 | 1278.6312 | 11 | -0.4 | 640.3226 | 2 | 29.59 | 8 | F8:6734 | 27122019\_RID\_1315\_NaNaWb\_F7.raw |  |  |  |  |  |  |  | 3.11E8 |  |  | 3 | 0 | 0 | 0 | 0 | 0 | 0 | 0 | 3 | 0 | 0 | 227 | 237 | Carbamidomethylation |
| N.TVTVM(+15.99)ENVNLDNK.V | Y | 46.38 | 1491.7239 | 13 | 0.6 | 746.8696 | 2 | 23.90 | 8 | F8:5003 | 27122019\_RID\_1315\_NaNaWb\_F7.raw |  |  |  |  |  |  |  | 3.94E6 |  |  | 1 | 0 | 0 | 0 | 0 | 0 | 0 | 0 | 1 | 0 | 0 | 158 | 170 | Oxidation (M) |
| K.QYFFETK.C | Y | 44.34 | 961.4545 | 7 | -0.4 | 481.7343 | 2 | 31.52 | 8 | F8:7377 | 27122019\_RID\_1315\_NaNaWb\_F7.raw |  |  |  |  |  |  |  | 2.76E8 |  |  | 1 | 0 | 0 | 0 | 0 | 0 | 0 | 0 | 1 | 0 | 0 | 174 | 180 |  |
| Y.C(+57.02)TETDTFIK.A | Y | 41.38 | 1113.5012 | 9 | -0.1 | 557.7578 | 2 | 22.31 | 8 | F8:4598 | 27122019\_RID\_1315\_NaNaWb\_F7.raw |  |  |  |  |  |  |  | 5.2E6 |  |  | 1 | 0 | 0 | 0 | 0 | 0 | 0 | 0 | 1 | 0 | 0 | 203 | 211 | Carbamidomethylation |
| total 10 peptides |  |  |  |  |  |  |  |  |  |  |  |  |  |  |  |  |  |  |  |  |  |  |  |  |  |  |  |  |  |  |  |  |  |  |  |
| --- | --- | --- | --- | --- | --- | --- | --- | --- | --- | --- | --- | --- | --- | --- | --- | --- | --- | --- | --- | --- | --- | --- | --- | --- | --- | --- | --- | --- | --- | --- | --- | --- | --- | --- | --- |

P01427|3S11\_NAJOX

back to list

  

| Protein Coverage
| Supporting Peptides
|

Protein Coverage:

Supporting Peptides:

| Peptide | Uniq | -10lgP | Mass | Length | ppm | m/z | z | RT | Fraction | Scan | Source File | Area F4 | Area F1 | Area F10 | Area F2 | Area F3 | Area F5 | Area F6 | Area F7 | Area F8 | Area F9 | #Feature | #Feature F4 | #Feature F1 | #Feature F10 | #Feature F2 | #Feature F3 | #Feature F5 | #Feature F6 | #Feature F7 | #Feature F8 | #Feature F9 | Start | End | PTM |
| --- | --- | --- | --- | --- | --- | --- | --- | --- | --- | --- | --- | --- | --- | --- | --- | --- | --- | --- | --- | --- | --- | --- | --- | --- | --- | --- | --- | --- | --- | --- | --- | --- | --- | --- | --- |
| LEC(+57.02)HNQQSSQPPTTK.T | Y | 91.90 | 1753.8053 | 15 | 1.4 | 877.9111 | 2 | 12.25 | 10 | F10:2063 | 27122019\_RID\_1315\_NaNaWb\_F9.raw |  |  |  |  | 8.82E8 |  |  |  |  | 1.4E7 | 8 | 0 | 0 | 0 | 0 | 6 | 0 | 0 | 0 | 0 | 2 | 1 | 15 | Carbamidomethylation |
| K.VKPGVNLNC(+57.02)C(+57.02)R.T | Y | 69.73 | 1315.6489 | 11 | 0.4 | 439.5571 | 3 | 12.28 | 10 | F10:2089 | 27122019\_RID\_1315\_NaNaWb\_F9.raw | 1.15E6 | 9.9E8 |  | 2.27E8 | 1.49E9 | 3.56E6 |  |  |  | 6.48E7 | 20 | 1 | 7 | 0 | 5 | 3 | 2 | 0 | 0 | 0 | 2 | 45 | 55 | Carbamidomethylation |
| K.PGVNLNC(+57.02)C(+57.02)R.T | Y | 51.93 | 1088.4856 | 9 | 0.1 | 545.2501 | 2 | 12.27 | 2 | F2:2070 | 27122019\_RID\_1315\_NaNaWb\_F1.raw |  | 7.09E7 |  |  |  |  |  |  |  |  | 1 | 0 | 1 | 0 | 0 | 0 | 0 | 0 | 0 | 0 | 0 | 47 | 55 | Carbamidomethylation |
| K.KWWSDHR.G | Y | 49.90 | 1013.4832 | 7 | 0.1 | 507.7489 | 2 | 12.27 | 2 | F2:2078 | 27122019\_RID\_1315\_NaNaWb\_F1.raw |  | 1.13E7 |  |  |  |  |  |  |  |  | 1 | 0 | 1 | 0 | 0 | 0 | 0 | 0 | 0 | 0 | 0 | 26 | 32 |  |
| K.VKPGVNLN.C | Y | 41.60 | 839.4865 | 8 | -0.6 | 420.7502 | 2 | 13.41 | 2 | F2:2743 | 27122019\_RID\_1315\_NaNaWb\_F1.raw |  | 7.06E8 |  |  |  |  |  |  |  |  | 1 | 0 | 1 | 0 | 0 | 0 | 0 | 0 | 0 | 0 | 0 | 45 | 52 |  |
| total 5 peptides |  |  |  |  |  |  |  |  |  |  |  |  |  |  |  |  |  |  |  |  |  |  |  |  |  |  |  |  |  |  |  |  |  |  |  |
| --- | --- | --- | --- | --- | --- | --- | --- | --- | --- | --- | --- | --- | --- | --- | --- | --- | --- | --- | --- | --- | --- | --- | --- | --- | --- | --- | --- | --- | --- | --- | --- | --- | --- | --- | --- |

P84808|CRVP2\_NAJKA

back to list

  

| Protein Coverage
| Supporting Peptides
|

Protein Coverage:

Supporting Peptides:

| Peptide | Uniq | -10lgP | Mass | Length | ppm | m/z | z | RT | Fraction | Scan | Source File | Area F4 | Area F1 | Area F10 | Area F2 | Area F3 | Area F5 | Area F6 | Area F7 | Area F8 | Area F9 | #Feature | #Feature F4 | #Feature F1 | #Feature F10 | #Feature F2 | #Feature F3 | #Feature F5 | #Feature F6 | #Feature F7 | #Feature F8 | #Feature F9 | Start | End | PTM |
| --- | --- | --- | --- | --- | --- | --- | --- | --- | --- | --- | --- | --- | --- | --- | --- | --- | --- | --- | --- | --- | --- | --- | --- | --- | --- | --- | --- | --- | --- | --- | --- | --- | --- | --- | --- |
| K.IGC(+57.02)GENLFM(+15.99)SSQPYAWSR.V | Y | 53.89 | 2117.9299 | 18 | -0.3 | 1059.9719 | 2 | 47.94 | 9 | F9:16086 | 27122019\_RID\_1315\_NaNaWb\_F8.raw |  |  |  |  |  |  |  |  | 2.44E7 |  | 1 | 0 | 0 | 0 | 0 | 0 | 0 | 0 | 0 | 1 | 0 | 90 | 107 | Carbamidomethylation; Oxidation (M) |
| C.QYC(+57.02)PAGNIIGSIATPYK.S | Y | 53.68 | 1851.9188 | 17 | 0.0 | 926.9667 | 2 | 48.13 | 9 | F9:16152 | 27122019\_RID\_1315\_NaNaWb\_F8.raw |  |  |  |  |  |  |  |  | 2.08E7 |  | 1 | 0 | 0 | 0 | 0 | 0 | 0 | 0 | 0 | 1 | 0 | 163 | 179 | Carbamidomethylation |
| R.C(+57.02)SFAHSPPHLR.T | Y | 53.10 | 1307.6193 | 11 | 1.7 | 436.8811 | 3 | 12.25 | 9 | F9:2077 | 27122019\_RID\_1315\_NaNaWb\_F8.raw |  |  |  |  |  |  |  |  | 3.11E7 |  | 1 | 0 | 0 | 0 | 0 | 0 | 0 | 0 | 0 | 1 | 0 | 75 | 85 | Carbamidomethylation |
| R.NM(+15.99)LQMEWNSNAAQNAK.R | Y | 50.83 | 1864.8196 | 16 | -0.5 | 933.4166 | 2 | 30.48 | 9 | F9:8566 | 27122019\_RID\_1315\_NaNaWb\_F8.raw |  |  |  |  |  |  |  |  | 2.08E6 |  | 1 | 0 | 0 | 0 | 0 | 0 | 0 | 0 | 0 | 1 | 0 | 54 | 69 | Oxidation (M) |
| K.C(+57.02)AASC(+57.02)FC(+57.02)R.T | Y | 48.69 | 1030.3783 | 8 | 0.9 | 516.1969 | 2 | 12.27 | 9 | F9:2099 | 27122019\_RID\_1315\_NaNaWb\_F8.raw |  |  |  |  |  |  |  |  | 1.3E7 |  | 1 | 0 | 0 | 0 | 0 | 0 | 0 | 0 | 0 | 1 | 0 | 227 | 234 | Carbamidomethylation |
| K.SGPPC(+57.02)GDC(+57.02)PSAC(+57.02)VNGLC(+57.02)TNPC(+57.02)K.H | Y | 47.13 | 2406.9482 | 22 | -0.6 | 1204.4807 | 2 | 28.06 | 9 | F9:7526 | 27122019\_RID\_1315\_NaNaWb\_F8.raw |  |  |  |  |  |  |  |  | 1.12E8 |  | 1 | 0 | 0 | 0 | 0 | 0 | 0 | 0 | 0 | 1 | 0 | 180 | 201 | Carbamidomethylation |
| Q.IVWYNSHLLGC(+57.02)GAAK.C | Y | 47.09 | 1687.8505 | 15 | 2.2 | 844.9343 | 2 | 36.13 | 9 | F9:10857 | 27122019\_RID\_1315\_NaNaWb\_F8.raw |  |  |  |  |  |  |  |  | 9.27E5 |  | 1 | 0 | 0 | 0 | 0 | 0 | 0 | 0 | 0 | 1 | 0 | 138 | 152 | Carbamidomethylation |
| Y.VC(+57.02)QYC(+57.02)PAGNIIGSIATPYK.S | Y | 44.85 | 2111.0181 | 19 | -0.3 | 1056.5160 | 2 | 50.08 | 9 | F9:17340 | 27122019\_RID\_1315\_NaNaWb\_F8.raw |  |  |  |  |  |  |  |  | 5.66E6 |  | 1 | 0 | 0 | 0 | 0 | 0 | 0 | 0 | 0 | 1 | 0 | 161 | 179 | Carbamidomethylation |
| K.FVYGVGANPPGSVIGHYTQIVWYNSHLLGC(+57.02)GAAK.C | Y | 42.39 | 3631.8035 | 34 | -0.1 | 908.9580 | 4 | 58.75 | 9 | F9:22652 | 27122019\_RID\_1315\_NaNaWb\_F8.raw |  |  |  |  |  |  |  |  | 7.39E7 |  | 1 | 0 | 0 | 0 | 0 | 0 | 0 | 0 | 0 | 1 | 0 | 119 | 152 | Carbamidomethylation |
| Q.YC(+57.02)PAGNIIGSIATPYK.S | Y | 42.23 | 1723.8604 | 16 | 0.7 | 575.6278 | 3 | 47.55 | 9 | F9:15905 | 27122019\_RID\_1315\_NaNaWb\_F8.raw |  |  |  |  |  |  |  |  | 5.13E5 |  | 1 | 0 | 0 | 0 | 0 | 0 | 0 | 0 | 0 | 1 | 0 | 164 | 179 | Carbamidomethylation |
| R.NMLQMEWNSNAAQNAK.R | Y | 41.10 | 1848.8247 | 16 | 0.2 | 925.4198 | 2 | 38.37 | 9 | F9:11682 | 27122019\_RID\_1315\_NaNaWb\_F8.raw |  |  |  |  |  |  |  |  | 1.33E7 |  | 1 | 0 | 0 | 0 | 0 | 0 | 0 | 0 | 0 | 1 | 0 | 54 | 69 |  |
| total 11 peptides |  |  |  |  |  |  |  |  |  |  |  |  |  |  |  |  |  |  |  |  |  |  |  |  |  |  |  |  |  |  |  |  |  |  |  |
| --- | --- | --- | --- | --- | --- | --- | --- | --- | --- | --- | --- | --- | --- | --- | --- | --- | --- | --- | --- | --- | --- | --- | --- | --- | --- | --- | --- | --- | --- | --- | --- | --- | --- | --- | --- |

Q9PST3|3SA2B\_NAJSP

back to list

  

| Protein Coverage
| Supporting Peptides
|

Protein Coverage:

Supporting Peptides:

| Peptide | Uniq | -10lgP | Mass | Length | ppm | m/z | z | RT | Fraction | Scan | Source File | Area F4 | Area F1 | Area F10 | Area F2 | Area F3 | Area F5 | Area F6 | Area F7 | Area F8 | Area F9 | #Feature | #Feature F4 | #Feature F1 | #Feature F10 | #Feature F2 | #Feature F3 | #Feature F5 | #Feature F6 | #Feature F7 | #Feature F8 | #Feature F9 | Start | End | PTM |
| --- | --- | --- | --- | --- | --- | --- | --- | --- | --- | --- | --- | --- | --- | --- | --- | --- | --- | --- | --- | --- | --- | --- | --- | --- | --- | --- | --- | --- | --- | --- | --- | --- | --- | --- | --- |
| R.GC(+57.02)IDVC(+57.02)PK.S | N | 61.16 | 947.4205 | 8 | 0.3 | 474.7177 | 2 | 15.80 | 7 | F7:3597 | 27122019\_RID\_1315\_NaNaWb\_F6.raw | 1.83E6 |  |  |  |  | 4.15E5 | 1.88E9 | 3E8 | 4.74E9 |  | 11 | 1 | 0 | 0 | 0 | 0 | 1 | 3 | 1 | 5 | 0 | 58 | 65 | Carbamidomethylation |
| T.LKC(+57.02)NKLVPLFYK.T | N | 55.21 | 1521.8741 | 12 | 0.3 | 508.2988 | 3 | 27.93 | 9 | F9:7425 | 27122019\_RID\_1315\_NaNaWb\_F8.raw |  |  |  |  |  |  |  |  | 2.47E8 |  | 4 | 0 | 0 | 0 | 0 | 0 | 0 | 0 | 0 | 4 | 0 | 22 | 33 | Carbamidomethylation |
| K.LVPLFYKTC(+57.02)PAGK.N | N | 54.89 | 1492.8112 | 13 | 0.7 | 747.4134 | 2 | 31.69 | 9 | F9:9094 | 27122019\_RID\_1315\_NaNaWb\_F8.raw |  |  |  |  |  |  |  |  | 1.62E7 |  | 1 | 0 | 0 | 0 | 0 | 0 | 0 | 0 | 0 | 1 | 0 | 27 | 39 | Carbamidomethylation |
| K.SSLLVKYVC(+57.02)C(+57.02)NTDRC(+57.02)N | N | 50.38 | 1987.8914 | 16 | 0.3 | 663.6379 | 3 | 28.94 | 9 | F9:8080 | 27122019\_RID\_1315\_NaNaWb\_F8.raw |  |  |  |  |  |  |  |  | 4.69E8 |  | 2 | 0 | 0 | 0 | 0 | 0 | 0 | 0 | 0 | 2 | 0 | 66 | 81 | Carbamidomethylation |
| K.MYM(+15.99)VATPKVPVKR.G | Y | 49.98 | 1534.8363 | 13 | 0.9 | 512.6198 | 3 | 22.53 | 9 | F9:5665 | 27122019\_RID\_1315\_NaNaWb\_F8.raw |  |  |  |  |  |  |  |  | 2.88E6 |  | 1 | 0 | 0 | 0 | 0 | 0 | 0 | 0 | 0 | 1 | 0 | 45 | 57 | Oxidation (M) |
| K.SSLLVKYVC(+57.02)C(+57.02)NTDR.C | N | 47.78 | 1713.8179 | 14 | 0.3 | 857.9165 | 2 | 27.06 | 9 | F9:7132 | 27122019\_RID\_1315\_NaNaWb\_F8.raw |  |  |  |  |  |  |  |  | 6.49E7 |  | 3 | 0 | 0 | 0 | 0 | 0 | 0 | 0 | 0 | 3 | 0 | 66 | 79 | Carbamidomethylation |
| K.M(+15.99)YM(+15.99)VATPK.V | Y | 46.06 | 971.4456 | 8 | -0.7 | 486.7297 | 2 | 12.66 | 9 | F9:2332 | 27122019\_RID\_1315\_NaNaWb\_F8.raw |  |  |  |  |  |  |  |  | 5.49E7 |  | 2 | 0 | 0 | 0 | 0 | 0 | 0 | 0 | 0 | 2 | 0 | 45 | 52 | Oxidation (M) |
| K.MYM(+15.99)VATPK.V | Y | 45.57 | 955.4507 | 8 | 0.0 | 478.7326 | 2 | 18.86 | 7 | F7:4518 | 27122019\_RID\_1315\_NaNaWb\_F6.raw |  |  |  |  |  |  | 4.02E6 | 3.44E7 | 8.2E7 |  | 3 | 0 | 0 | 0 | 0 | 0 | 0 | 1 | 1 | 1 | 0 | 45 | 52 | Oxidation (M) |
| K.MYMVATPKVPVKR.G | Y | 43.32 | 1518.8414 | 13 | 0.0 | 507.2878 | 3 | 22.53 | 9 | F9:5664 | 27122019\_RID\_1315\_NaNaWb\_F8.raw |  |  |  |  |  |  |  |  | 1.99E7 |  | 2 | 0 | 0 | 0 | 0 | 0 | 0 | 0 | 0 | 2 | 0 | 45 | 57 |  |
| K.M(+15.99)YMVATPK.V | Y | 41.36 | 955.4507 | 8 | 0.0 | 478.7326 | 2 | 22.91 | 9 | F9:6089 | 27122019\_RID\_1315\_NaNaWb\_F8.raw |  |  |  |  |  |  |  |  | 9.54E7 |  | 1 | 0 | 0 | 0 | 0 | 0 | 0 | 0 | 0 | 1 | 0 | 45 | 52 | Oxidation (M) |
| total 10 peptides |  |  |  |  |  |  |  |  |  |  |  |  |  |  |  |  |  |  |  |  |  |  |  |  |  |  |  |  |  |  |  |  |  |  |  |
| --- | --- | --- | --- | --- | --- | --- | --- | --- | --- | --- | --- | --- | --- | --- | --- | --- | --- | --- | --- | --- | --- | --- | --- | --- | --- | --- | --- | --- | --- | --- | --- | --- | --- | --- | --- |

Q9PST4|3SA2A\_NAJSP

back to list

  

| Protein Coverage
| Supporting Peptides
|

Protein Coverage:

Supporting Peptides:

| Peptide | Uniq | -10lgP | Mass | Length | ppm | m/z | z | RT | Fraction | Scan | Source File | Area F4 | Area F1 | Area F10 | Area F2 | Area F3 | Area F5 | Area F6 | Area F7 | Area F8 | Area F9 | #Feature | #Feature F4 | #Feature F1 | #Feature F10 | #Feature F2 | #Feature F3 | #Feature F5 | #Feature F6 | #Feature F7 | #Feature F8 | #Feature F9 | Start | End | PTM |
| --- | --- | --- | --- | --- | --- | --- | --- | --- | --- | --- | --- | --- | --- | --- | --- | --- | --- | --- | --- | --- | --- | --- | --- | --- | --- | --- | --- | --- | --- | --- | --- | --- | --- | --- | --- |
| R.GC(+57.02)IDVC(+57.02)PK.S | N | 61.16 | 947.4205 | 8 | 0.3 | 474.7177 | 2 | 15.80 | 7 | F7:3597 | 27122019\_RID\_1315\_NaNaWb\_F6.raw | 1.83E6 |  |  |  |  | 4.15E5 | 1.88E9 | 3E8 | 4.74E9 |  | 11 | 1 | 0 | 0 | 0 | 0 | 1 | 3 | 1 | 5 | 0 | 58 | 65 | Carbamidomethylation |
| T.LKC(+57.02)NKLVPLFYK.T | N | 55.21 | 1521.8741 | 12 | 0.3 | 508.2988 | 3 | 27.93 | 9 | F9:7425 | 27122019\_RID\_1315\_NaNaWb\_F8.raw |  |  |  |  |  |  |  |  | 2.47E8 |  | 4 | 0 | 0 | 0 | 0 | 0 | 0 | 0 | 0 | 4 | 0 | 22 | 33 | Carbamidomethylation |
| K.LVPLFYKTC(+57.02)PAGK.N | N | 54.89 | 1492.8112 | 13 | 0.7 | 747.4134 | 2 | 31.69 | 9 | F9:9094 | 27122019\_RID\_1315\_NaNaWb\_F8.raw |  |  |  |  |  |  |  |  | 1.62E7 |  | 1 | 0 | 0 | 0 | 0 | 0 | 0 | 0 | 0 | 1 | 0 | 27 | 39 | Carbamidomethylation |
| K.SSLLVKYVC(+57.02)C(+57.02)NTDRC(+57.02)N | N | 50.38 | 1987.8914 | 16 | 0.3 | 663.6379 | 3 | 28.94 | 9 | F9:8080 | 27122019\_RID\_1315\_NaNaWb\_F8.raw |  |  |  |  |  |  |  |  | 4.69E8 |  | 2 | 0 | 0 | 0 | 0 | 0 | 0 | 0 | 0 | 2 | 0 | 66 | 81 | Carbamidomethylation |
| K.MYM(+15.99)VATPKVPVKR.G | Y | 49.98 | 1534.8363 | 13 | 0.9 | 512.6198 | 3 | 22.53 | 9 | F9:5665 | 27122019\_RID\_1315\_NaNaWb\_F8.raw |  |  |  |  |  |  |  |  | 2.88E6 |  | 1 | 0 | 0 | 0 | 0 | 0 | 0 | 0 | 0 | 1 | 0 | 45 | 57 | Oxidation (M) |
| K.SSLLVKYVC(+57.02)C(+57.02)NTDR.C | N | 47.78 | 1713.8179 | 14 | 0.3 | 857.9165 | 2 | 27.06 | 9 | F9:7132 | 27122019\_RID\_1315\_NaNaWb\_F8.raw |  |  |  |  |  |  |  |  | 6.49E7 |  | 3 | 0 | 0 | 0 | 0 | 0 | 0 | 0 | 0 | 3 | 0 | 66 | 79 | Carbamidomethylation |
| K.M(+15.99)YM(+15.99)VATPK.V | Y | 46.06 | 971.4456 | 8 | -0.7 | 486.7297 | 2 | 12.66 | 9 | F9:2332 | 27122019\_RID\_1315\_NaNaWb\_F8.raw |  |  |  |  |  |  |  |  | 5.49E7 |  | 2 | 0 | 0 | 0 | 0 | 0 | 0 | 0 | 0 | 2 | 0 | 45 | 52 | Oxidation (M) |
| K.MYM(+15.99)VATPK.V | Y | 45.57 | 955.4507 | 8 | 0.0 | 478.7326 | 2 | 18.86 | 7 | F7:4518 | 27122019\_RID\_1315\_NaNaWb\_F6.raw |  |  |  |  |  |  | 4.02E6 | 3.44E7 | 8.2E7 |  | 3 | 0 | 0 | 0 | 0 | 0 | 0 | 1 | 1 | 1 | 0 | 45 | 52 | Oxidation (M) |
| K.MYMVATPKVPVKR.G | Y | 43.32 | 1518.8414 | 13 | 0.0 | 507.2878 | 3 | 22.53 | 9 | F9:5664 | 27122019\_RID\_1315\_NaNaWb\_F8.raw |  |  |  |  |  |  |  |  | 1.99E7 |  | 2 | 0 | 0 | 0 | 0 | 0 | 0 | 0 | 0 | 2 | 0 | 45 | 57 |  |
| K.M(+15.99)YMVATPK.V | Y | 41.36 | 955.4507 | 8 | 0.0 | 478.7326 | 2 | 22.91 | 9 | F9:6089 | 27122019\_RID\_1315\_NaNaWb\_F8.raw |  |  |  |  |  |  |  |  | 9.54E7 |  | 1 | 0 | 0 | 0 | 0 | 0 | 0 | 0 | 0 | 1 | 0 | 45 | 52 | Oxidation (M) |
| total 10 peptides |  |  |  |  |  |  |  |  |  |  |  |  |  |  |  |  |  |  |  |  |  |  |  |  |  |  |  |  |  |  |  |  |  |  |  |
| --- | --- | --- | --- | --- | --- | --- | --- | --- | --- | --- | --- | --- | --- | --- | --- | --- | --- | --- | --- | --- | --- | --- | --- | --- | --- | --- | --- | --- | --- | --- | --- | --- | --- | --- | --- |

A0A0U5AUY6|3SA9\_NAJNA

back to list

  

| Protein Coverage
| Supporting Peptides
|

Protein Coverage:

Supporting Peptides:

| Peptide | Uniq | -10lgP | Mass | Length | ppm | m/z | z | RT | Fraction | Scan | Source File | Area F4 | Area F1 | Area F10 | Area F2 | Area F3 | Area F5 | Area F6 | Area F7 | Area F8 | Area F9 | #Feature | #Feature F4 | #Feature F1 | #Feature F10 | #Feature F2 | #Feature F3 | #Feature F5 | #Feature F6 | #Feature F7 | #Feature F8 | #Feature F9 | Start | End | PTM |
| --- | --- | --- | --- | --- | --- | --- | --- | --- | --- | --- | --- | --- | --- | --- | --- | --- | --- | --- | --- | --- | --- | --- | --- | --- | --- | --- | --- | --- | --- | --- | --- | --- | --- | --- | --- |
| R.GC(+57.02)IDVC(+57.02)PK.S | N | 61.16 | 947.4205 | 8 | 0.3 | 474.7177 | 2 | 15.80 | 7 | F7:3597 | 27122019\_RID\_1315\_NaNaWb\_F6.raw | 1.83E6 |  |  |  |  | 4.15E5 | 1.88E9 | 3E8 | 4.74E9 |  | 11 | 1 | 0 | 0 | 0 | 0 | 1 | 3 | 1 | 5 | 0 | 49 | 56 | Carbamidomethylation |
| T.LKC(+57.02)NKLVPLFYK.T | N | 55.21 | 1521.8741 | 12 | 0.3 | 508.2988 | 3 | 27.93 | 9 | F9:7425 | 27122019\_RID\_1315\_NaNaWb\_F8.raw |  |  |  |  |  |  |  |  | 2.47E8 |  | 4 | 0 | 0 | 0 | 0 | 0 | 0 | 0 | 0 | 4 | 0 | 13 | 24 | Carbamidomethylation |
| K.LVPLFYKTC(+57.02)PAGK.N | N | 54.89 | 1492.8112 | 13 | 0.7 | 747.4134 | 2 | 31.69 | 9 | F9:9094 | 27122019\_RID\_1315\_NaNaWb\_F8.raw |  |  |  |  |  |  |  |  | 1.62E7 |  | 1 | 0 | 0 | 0 | 0 | 0 | 0 | 0 | 0 | 1 | 0 | 18 | 30 | Carbamidomethylation |
| K.SSLLVKYVC(+57.02)C(+57.02)NTDRC(+57.02)N | N | 50.38 | 1987.8914 | 16 | 0.3 | 663.6379 | 3 | 28.94 | 9 | F9:8080 | 27122019\_RID\_1315\_NaNaWb\_F8.raw |  |  |  |  |  |  |  |  | 4.69E8 |  | 2 | 0 | 0 | 0 | 0 | 0 | 0 | 0 | 0 | 2 | 0 | 57 | 72 | Carbamidomethylation |
| K.MYM(+15.99)VATPKVPVKR.G | Y | 49.98 | 1534.8363 | 13 | 0.9 | 512.6198 | 3 | 22.53 | 9 | F9:5665 | 27122019\_RID\_1315\_NaNaWb\_F8.raw |  |  |  |  |  |  |  |  | 2.88E6 |  | 1 | 0 | 0 | 0 | 0 | 0 | 0 | 0 | 0 | 1 | 0 | 36 | 48 | Oxidation (M) |
| K.SSLLVKYVC(+57.02)C(+57.02)NTDR.C | N | 47.78 | 1713.8179 | 14 | 0.3 | 857.9165 | 2 | 27.06 | 9 | F9:7132 | 27122019\_RID\_1315\_NaNaWb\_F8.raw |  |  |  |  |  |  |  |  | 6.49E7 |  | 3 | 0 | 0 | 0 | 0 | 0 | 0 | 0 | 0 | 3 | 0 | 57 | 70 | Carbamidomethylation |
| K.M(+15.99)YM(+15.99)VATPK.V | Y | 46.06 | 971.4456 | 8 | -0.7 | 486.7297 | 2 | 12.66 | 9 | F9:2332 | 27122019\_RID\_1315\_NaNaWb\_F8.raw |  |  |  |  |  |  |  |  | 5.49E7 |  | 2 | 0 | 0 | 0 | 0 | 0 | 0 | 0 | 0 | 2 | 0 | 36 | 43 | Oxidation (M) |
| K.MYM(+15.99)VATPK.V | Y | 45.57 | 955.4507 | 8 | 0.0 | 478.7326 | 2 | 18.86 | 7 | F7:4518 | 27122019\_RID\_1315\_NaNaWb\_F6.raw |  |  |  |  |  |  | 4.02E6 | 3.44E7 | 8.2E7 |  | 3 | 0 | 0 | 0 | 0 | 0 | 0 | 1 | 1 | 1 | 0 | 36 | 43 | Oxidation (M) |
| K.MYMVATPKVPVKR.G | Y | 43.32 | 1518.8414 | 13 | 0.0 | 507.2878 | 3 | 22.53 | 9 | F9:5664 | 27122019\_RID\_1315\_NaNaWb\_F8.raw |  |  |  |  |  |  |  |  | 1.99E7 |  | 2 | 0 | 0 | 0 | 0 | 0 | 0 | 0 | 0 | 2 | 0 | 36 | 48 |  |
| K.M(+15.99)YMVATPK.V | Y | 41.36 | 955.4507 | 8 | 0.0 | 478.7326 | 2 | 22.91 | 9 | F9:6089 | 27122019\_RID\_1315\_NaNaWb\_F8.raw |  |  |  |  |  |  |  |  | 9.54E7 |  | 1 | 0 | 0 | 0 | 0 | 0 | 0 | 0 | 0 | 1 | 0 | 36 | 43 | Oxidation (M) |
| total 10 peptides |  |  |  |  |  |  |  |  |  |  |  |  |  |  |  |  |  |  |  |  |  |  |  |  |  |  |  |  |  |  |  |  |  |  |  |
| --- | --- | --- | --- | --- | --- | --- | --- | --- | --- | --- | --- | --- | --- | --- | --- | --- | --- | --- | --- | --- | --- | --- | --- | --- | --- | --- | --- | --- | --- | --- | --- | --- | --- | --- | --- |

P86541|3SAA\_NAJNA

back to list

  

| Protein Coverage
| Supporting Peptides
|

Protein Coverage:

Supporting Peptides:

| Peptide | Uniq | -10lgP | Mass | Length | ppm | m/z | z | RT | Fraction | Scan | Source File | Area F4 | Area F1 | Area F10 | Area F2 | Area F3 | Area F5 | Area F6 | Area F7 | Area F8 | Area F9 | #Feature | #Feature F4 | #Feature F1 | #Feature F10 | #Feature F2 | #Feature F3 | #Feature F5 | #Feature F6 | #Feature F7 | #Feature F8 | #Feature F9 | Start | End | PTM |
| --- | --- | --- | --- | --- | --- | --- | --- | --- | --- | --- | --- | --- | --- | --- | --- | --- | --- | --- | --- | --- | --- | --- | --- | --- | --- | --- | --- | --- | --- | --- | --- | --- | --- | --- | --- |
| R.GC(+57.02)IDVC(+57.02)PK.S | N | 61.16 | 947.4205 | 8 | 0.3 | 474.7177 | 2 | 15.80 | 7 | F7:3597 | 27122019\_RID\_1315\_NaNaWb\_F6.raw | 1.83E6 |  |  |  |  | 4.15E5 | 1.88E9 | 3E8 | 4.74E9 |  | 11 | 1 | 0 | 0 | 0 | 0 | 1 | 3 | 1 | 5 | 0 | 37 | 44 | Carbamidomethylation |
| LKC(+57.02)NKLVPLFYK.T | N | 55.21 | 1521.8741 | 12 | 0.3 | 508.2988 | 3 | 27.93 | 9 | F9:7425 | 27122019\_RID\_1315\_NaNaWb\_F8.raw |  |  |  |  |  |  |  |  | 2.47E8 |  | 4 | 0 | 0 | 0 | 0 | 0 | 0 | 0 | 0 | 4 | 0 | 1 | 12 | Carbamidomethylation |
| K.LVPLFYKTC(+57.02)PAGK.D | N | 54.89 | 1492.8112 | 13 | 0.7 | 747.4134 | 2 | 31.69 | 9 | F9:9094 | 27122019\_RID\_1315\_NaNaWb\_F8.raw |  |  |  |  |  |  |  |  | 1.62E7 |  | 1 | 0 | 0 | 0 | 0 | 0 | 0 | 0 | 0 | 1 | 0 | 6 | 18 | Carbamidomethylation |
| K.SSLLVKYVC(+57.02)C(+57.02)NTDRC(+57.02)N | N | 50.38 | 1987.8914 | 16 | 0.3 | 663.6379 | 3 | 28.94 | 9 | F9:8080 | 27122019\_RID\_1315\_NaNaWb\_F8.raw |  |  |  |  |  |  |  |  | 4.69E8 |  | 2 | 0 | 0 | 0 | 0 | 0 | 0 | 0 | 0 | 2 | 0 | 45 | 60 | Carbamidomethylation |
| K.MYM(+15.99)VATPKVPVKR.G | Y | 49.98 | 1534.8363 | 13 | 0.9 | 512.6198 | 3 | 22.53 | 9 | F9:5665 | 27122019\_RID\_1315\_NaNaWb\_F8.raw |  |  |  |  |  |  |  |  | 2.88E6 |  | 1 | 0 | 0 | 0 | 0 | 0 | 0 | 0 | 0 | 1 | 0 | 24 | 36 | Oxidation (M) |
| K.SSLLVKYVC(+57.02)C(+57.02)NTDR.C | N | 47.78 | 1713.8179 | 14 | 0.3 | 857.9165 | 2 | 27.06 | 9 | F9:7132 | 27122019\_RID\_1315\_NaNaWb\_F8.raw |  |  |  |  |  |  |  |  | 6.49E7 |  | 3 | 0 | 0 | 0 | 0 | 0 | 0 | 0 | 0 | 3 | 0 | 45 | 58 | Carbamidomethylation |
| K.M(+15.99)YM(+15.99)VATPK.V | Y | 46.06 | 971.4456 | 8 | -0.7 | 486.7297 | 2 | 12.66 | 9 | F9:2332 | 27122019\_RID\_1315\_NaNaWb\_F8.raw |  |  |  |  |  |  |  |  | 5.49E7 |  | 2 | 0 | 0 | 0 | 0 | 0 | 0 | 0 | 0 | 2 | 0 | 24 | 31 | Oxidation (M) |
| K.MYM(+15.99)VATPK.V | Y | 45.57 | 955.4507 | 8 | 0.0 | 478.7326 | 2 | 18.86 | 7 | F7:4518 | 27122019\_RID\_1315\_NaNaWb\_F6.raw |  |  |  |  |  |  | 4.02E6 | 3.44E7 | 8.2E7 |  | 3 | 0 | 0 | 0 | 0 | 0 | 0 | 1 | 1 | 1 | 0 | 24 | 31 | Oxidation (M) |
| K.MYMVATPKVPVKR.G | Y | 43.32 | 1518.8414 | 13 | 0.0 | 507.2878 | 3 | 22.53 | 9 | F9:5664 | 27122019\_RID\_1315\_NaNaWb\_F8.raw |  |  |  |  |  |  |  |  | 1.99E7 |  | 2 | 0 | 0 | 0 | 0 | 0 | 0 | 0 | 0 | 2 | 0 | 24 | 36 |  |
| K.M(+15.99)YMVATPK.V | Y | 41.36 | 955.4507 | 8 | 0.0 | 478.7326 | 2 | 22.91 | 9 | F9:6089 | 27122019\_RID\_1315\_NaNaWb\_F8.raw |  |  |  |  |  |  |  |  | 9.54E7 |  | 1 | 0 | 0 | 0 | 0 | 0 | 0 | 0 | 0 | 1 | 0 | 24 | 31 | Oxidation (M) |
| total 10 peptides |  |  |  |  |  |  |  |  |  |  |  |  |  |  |  |  |  |  |  |  |  |  |  |  |  |  |  |  |  |  |  |  |  |  |  |
| --- | --- | --- | --- | --- | --- | --- | --- | --- | --- | --- | --- | --- | --- | --- | --- | --- | --- | --- | --- | --- | --- | --- | --- | --- | --- | --- | --- | --- | --- | --- | --- | --- | --- | --- | --- |

P10117|PA2H1\_LATCO

back to list

  

| Protein Coverage
| Supporting Peptides
|

Protein Coverage:

Supporting Peptides:

| Peptide | Uniq | -10lgP | Mass | Length | ppm | m/z | z | RT | Fraction | Scan | Source File | Area F4 | Area F1 | Area F10 | Area F2 | Area F3 | Area F5 | Area F6 | Area F7 | Area F8 | Area F9 | #Feature | #Feature F4 | #Feature F1 | #Feature F10 | #Feature F2 | #Feature F3 | #Feature F5 | #Feature F6 | #Feature F7 | #Feature F8 | #Feature F9 | Start | End | PTM |
| --- | --- | --- | --- | --- | --- | --- | --- | --- | --- | --- | --- | --- | --- | --- | --- | --- | --- | --- | --- | --- | --- | --- | --- | --- | --- | --- | --- | --- | --- | --- | --- | --- | --- | --- | --- |
| Y.C(+57.02)GPGGSGTPVDDLDR.C | Y | 86.01 | 1501.6467 | 15 | -0.4 | 751.8303 | 2 | 24.62 | 8 | F8:5250 | 27122019\_RID\_1315\_NaNaWb\_F7.raw |  |  |  |  |  |  |  | 3.94E6 |  |  | 1 | 0 | 0 | 0 | 0 | 0 | 0 | 0 | 1 | 0 | 0 | 29 | 43 | Carbamidomethylation |
| P.GGSGTPVDDLDR.C | N | 76.07 | 1187.5417 | 12 | 0.1 | 594.7782 | 2 | 22.36 | 7 | F7:6369 | 27122019\_RID\_1315\_NaNaWb\_F6.raw |  |  |  |  |  |  | 5.85E9 | 4.29E8 |  |  | 9 | 0 | 0 | 0 | 0 | 0 | 0 | 6 | 3 | 0 | 0 | 32 | 43 |  |
| G.GSGTPVDDLDR.C | N | 47.11 | 1130.5204 | 11 | 0.2 | 566.2676 | 2 | 21.06 | 7 | F7:5676 | 27122019\_RID\_1315\_NaNaWb\_F6.raw |  |  |  |  |  |  | 3.97E7 |  |  |  | 1 | 0 | 0 | 0 | 0 | 0 | 0 | 1 | 0 | 0 | 0 | 33 | 43 |  |
| total 3 peptides |  |  |  |  |  |  |  |  |  |  |  |  |  |  |  |  |  |  |  |  |  |  |  |  |  |  |  |  |  |  |  |  |  |  |  |
| --- | --- | --- | --- | --- | --- | --- | --- | --- | --- | --- | --- | --- | --- | --- | --- | --- | --- | --- | --- | --- | --- | --- | --- | --- | --- | --- | --- | --- | --- | --- | --- | --- | --- | --- | --- |

P86382|3SA7\_NAJNA

back to list

  

| Protein Coverage
| Supporting Peptides
|

Protein Coverage:

Supporting Peptides:

| Peptide | Uniq | -10lgP | Mass | Length | ppm | m/z | z | RT | Fraction | Scan | Source File | Area F4 | Area F1 | Area F10 | Area F2 | Area F3 | Area F5 | Area F6 | Area F7 | Area F8 | Area F9 | #Feature | #Feature F4 | #Feature F1 | #Feature F10 | #Feature F2 | #Feature F3 | #Feature F5 | #Feature F6 | #Feature F7 | #Feature F8 | #Feature F9 | Start | End | PTM |
| --- | --- | --- | --- | --- | --- | --- | --- | --- | --- | --- | --- | --- | --- | --- | --- | --- | --- | --- | --- | --- | --- | --- | --- | --- | --- | --- | --- | --- | --- | --- | --- | --- | --- | --- | --- |
| K.NSLLVKYEC(+57.02)C(+57.02)NTDRC(+57.02)N | Y | 66.08 | 2044.8765 | 16 | -0.5 | 682.6324 | 3 | 22.17 | 7 | F7:6197 | 27122019\_RID\_1315\_NaNaWb\_F6.raw |  |  |  |  |  |  | 3.87E7 |  |  |  | 2 | 0 | 0 | 0 | 0 | 0 | 0 | 2 | 0 | 0 | 0 | 45 | 60 | Carbamidomethylation |
| R.GC(+57.02)IDVC(+57.02)PK.N | N | 61.16 | 947.4205 | 8 | 0.3 | 474.7177 | 2 | 15.80 | 7 | F7:3597 | 27122019\_RID\_1315\_NaNaWb\_F6.raw | 1.83E6 |  |  |  |  | 4.15E5 | 1.88E9 | 3E8 | 4.74E9 |  | 11 | 1 | 0 | 0 | 0 | 0 | 1 | 3 | 1 | 5 | 0 | 37 | 44 | Carbamidomethylation |
| K.LIPLAYKTC(+57.02)PAGK.D | Y | 52.43 | 1430.7955 | 13 | 0.0 | 477.9391 | 3 | 24.90 | 7 | F7:7498 | 27122019\_RID\_1315\_NaNaWb\_F6.raw |  |  |  |  |  |  | 6.77E6 |  |  |  | 1 | 0 | 0 | 0 | 0 | 0 | 0 | 1 | 0 | 0 | 0 | 6 | 18 | Carbamidomethylation |
| R.GC(+57.02)IDVC(+57.02)PKN.S | N | 50.02 | 1061.4634 | 9 | -0.8 | 531.7385 | 2 | 16.77 | 9 | F9:3508 | 27122019\_RID\_1315\_NaNaWb\_F8.raw |  |  |  |  |  |  |  |  | 2.99E8 |  | 1 | 0 | 0 | 0 | 0 | 0 | 0 | 0 | 0 | 1 | 0 | 37 | 45 | Carbamidomethylation |
| K.LIPLAYK.T | Y | 49.84 | 816.5109 | 7 | -0.1 | 409.2627 | 2 | 33.52 | 7 | F7:11169 | 27122019\_RID\_1315\_NaNaWb\_F6.raw |  |  |  |  |  |  | 6.59E9 |  |  |  | 7 | 0 | 0 | 0 | 0 | 0 | 0 | 7 | 0 | 0 | 0 | 6 | 12 |  |
| K.NSLLVKYEC(+57.02)C(+57.02)NTDR.C | Y | 49.53 | 1770.8029 | 14 | -1.6 | 591.2739 | 3 | 24.26 | 7 | F7:7200 | 27122019\_RID\_1315\_NaNaWb\_F6.raw |  |  |  |  |  |  | 0 |  |  |  | 1 | 0 | 0 | 0 | 0 | 0 | 0 | 1 | 0 | 0 | 0 | 45 | 58 | Carbamidomethylation |
| total 6 peptides |  |  |  |  |  |  |  |  |  |  |  |  |  |  |  |  |  |  |  |  |  |  |  |  |  |  |  |  |  |  |  |  |  |  |  |
| --- | --- | --- | --- | --- | --- | --- | --- | --- | --- | --- | --- | --- | --- | --- | --- | --- | --- | --- | --- | --- | --- | --- | --- | --- | --- | --- | --- | --- | --- | --- | --- | --- | --- | --- | --- |

P86540|3SA8\_NAJNA

back to list

  

| Protein Coverage
| Supporting Peptides
|

Protein Coverage:

Supporting Peptides:

| Peptide | Uniq | -10lgP | Mass | Length | ppm | m/z | z | RT | Fraction | Scan | Source File | Area F4 | Area F1 | Area F10 | Area F2 | Area F3 | Area F5 | Area F6 | Area F7 | Area F8 | Area F9 | #Feature | #Feature F4 | #Feature F1 | #Feature F10 | #Feature F2 | #Feature F3 | #Feature F5 | #Feature F6 | #Feature F7 | #Feature F8 | #Feature F9 | Start | End | PTM |
| --- | --- | --- | --- | --- | --- | --- | --- | --- | --- | --- | --- | --- | --- | --- | --- | --- | --- | --- | --- | --- | --- | --- | --- | --- | --- | --- | --- | --- | --- | --- | --- | --- | --- | --- | --- |
| K.NSLLVKYEC(+57.02)C(+57.02)NTDRC(+57.02)N | Y | 66.08 | 2044.8765 | 16 | -0.5 | 682.6324 | 3 | 22.17 | 7 | F7:6197 | 27122019\_RID\_1315\_NaNaWb\_F6.raw |  |  |  |  |  |  | 3.87E7 |  |  |  | 2 | 0 | 0 | 0 | 0 | 0 | 0 | 2 | 0 | 0 | 0 | 45 | 60 | Carbamidomethylation |
| R.GC(+57.02)IDVC(+57.02)PK.N | N | 61.16 | 947.4205 | 8 | 0.3 | 474.7177 | 2 | 15.80 | 7 | F7:3597 | 27122019\_RID\_1315\_NaNaWb\_F6.raw | 1.83E6 |  |  |  |  | 4.15E5 | 1.88E9 | 3E8 | 4.74E9 |  | 11 | 1 | 0 | 0 | 0 | 0 | 1 | 3 | 1 | 5 | 0 | 37 | 44 | Carbamidomethylation |
| K.LIPLAYKTC(+57.02)PAGK.D | Y | 52.43 | 1430.7955 | 13 | 0.0 | 477.9391 | 3 | 24.90 | 7 | F7:7498 | 27122019\_RID\_1315\_NaNaWb\_F6.raw |  |  |  |  |  |  | 6.77E6 |  |  |  | 1 | 0 | 0 | 0 | 0 | 0 | 0 | 1 | 0 | 0 | 0 | 6 | 18 | Carbamidomethylation |
| R.GC(+57.02)IDVC(+57.02)PKN.S | N | 50.02 | 1061.4634 | 9 | -0.8 | 531.7385 | 2 | 16.77 | 9 | F9:3508 | 27122019\_RID\_1315\_NaNaWb\_F8.raw |  |  |  |  |  |  |  |  | 2.99E8 |  | 1 | 0 | 0 | 0 | 0 | 0 | 0 | 0 | 0 | 1 | 0 | 37 | 45 | Carbamidomethylation |
| K.LIPLAYK.T | Y | 49.84 | 816.5109 | 7 | -0.1 | 409.2627 | 2 | 33.52 | 7 | F7:11169 | 27122019\_RID\_1315\_NaNaWb\_F6.raw |  |  |  |  |  |  | 6.59E9 |  |  |  | 7 | 0 | 0 | 0 | 0 | 0 | 0 | 7 | 0 | 0 | 0 | 6 | 12 |  |
| K.NSLLVKYEC(+57.02)C(+57.02)NTDR.C | Y | 49.53 | 1770.8029 | 14 | -1.6 | 591.2739 | 3 | 24.26 | 7 | F7:7200 | 27122019\_RID\_1315\_NaNaWb\_F6.raw |  |  |  |  |  |  | 0 |  |  |  | 1 | 0 | 0 | 0 | 0 | 0 | 0 | 1 | 0 | 0 | 0 | 45 | 58 | Carbamidomethylation |
| total 6 peptides |  |  |  |  |  |  |  |  |  |  |  |  |  |  |  |  |  |  |  |  |  |  |  |  |  |  |  |  |  |  |  |  |  |  |  |
| --- | --- | --- | --- | --- | --- | --- | --- | --- | --- | --- | --- | --- | --- | --- | --- | --- | --- | --- | --- | --- | --- | --- | --- | --- | --- | --- | --- | --- | --- | --- | --- | --- | --- | --- | --- |

P82464|3SO8\_NAJKA

back to list

  

| Protein Coverage
| Supporting Peptides
|

Protein Coverage:

Supporting Peptides:

| Peptide | Uniq | -10lgP | Mass | Length | ppm | m/z | z | RT | Fraction | Scan | Source File | Area F4 | Area F1 | Area F10 | Area F2 | Area F3 | Area F5 | Area F6 | Area F7 | Area F8 | Area F9 | #Feature | #Feature F4 | #Feature F1 | #Feature F10 | #Feature F2 | #Feature F3 | #Feature F5 | #Feature F6 | #Feature F7 | #Feature F8 | #Feature F9 | Start | End | PTM |
| --- | --- | --- | --- | --- | --- | --- | --- | --- | --- | --- | --- | --- | --- | --- | --- | --- | --- | --- | --- | --- | --- | --- | --- | --- | --- | --- | --- | --- | --- | --- | --- | --- | --- | --- | --- |
| K.ISLADGNDVR.I | Y | 66.65 | 1058.5356 | 10 | 0.2 | 530.2752 | 2 | 22.22 | 8 | F8:4577 | 27122019\_RID\_1315\_NaNaWb\_F7.raw |  |  |  |  |  |  | 7.46E6 | 1.03E6 |  | 1.72E8 | 3 | 0 | 0 | 0 | 0 | 0 | 0 | 1 | 1 | 0 | 1 | 27 | 36 |  |
| TIC(+57.02)YNHLTR.T | Y | 60.87 | 1176.5709 | 9 | -0.6 | 589.2924 | 2 | 12.76 | 10 | F10:2300 | 27122019\_RID\_1315\_NaNaWb\_F9.raw |  |  |  |  |  |  |  |  |  | 5.18E7 | 2 | 0 | 0 | 0 | 0 | 0 | 0 | 0 | 0 | 0 | 2 | 1 | 9 | Carbamidomethylation |
| R.GC(+57.02)TFTC(+57.02)PELRPTGIYVYC(+57.02).C | Y | 51.08 | 2192.9692 | 18 | -0.5 | 1097.4913 | 2 | 51.13 | 10 | F10:12964 | 27122019\_RID\_1315\_NaNaWb\_F9.raw |  |  |  |  |  |  |  |  |  | 8.95E6 | 1 | 0 | 0 | 0 | 0 | 0 | 0 | 0 | 0 | 0 | 1 | 40 | 57 | Carbamidomethylation |
| R.TSETTEIC(+57.02)PDSWYFC(+57.02)YK.I | Y | 50.94 | 2185.8972 | 17 | -0.8 | 1093.9550 | 2 | 51.43 | 7 | F7:18171 | 27122019\_RID\_1315\_NaNaWb\_F6.raw |  |  |  |  |  |  | 1.43E7 |  |  |  | 1 | 0 | 0 | 0 | 0 | 0 | 0 | 1 | 0 | 0 | 0 | 10 | 26 | Carbamidomethylation |
| R.GC(+57.02)TFTC(+57.02)PELRPT.G | Y | 44.99 | 1437.6381 | 12 | -0.3 | 719.8261 | 2 | 30.95 | 10 | F10:6906 | 27122019\_RID\_1315\_NaNaWb\_F9.raw |  |  |  |  |  |  |  |  |  | 2.98E6 | 1 | 0 | 0 | 0 | 0 | 0 | 0 | 0 | 0 | 0 | 1 | 40 | 51 | Carbamidomethylation |
| R.GC(+57.02)TFTC(+57.02)PELRPTGIYVYC(+57.02)C(+57.02)R.R | Y | 41.12 | 2509.1011 | 20 | 0.0 | 837.3743 | 3 | 42.31 | 10 | F10:10748 | 27122019\_RID\_1315\_NaNaWb\_F9.raw |  |  |  |  |  |  |  |  |  | 1.77E7 | 1 | 0 | 0 | 0 | 0 | 0 | 0 | 0 | 0 | 0 | 1 | 40 | 59 | Carbamidomethylation |
| total 6 peptides |  |  |  |  |  |  |  |  |  |  |  |  |  |  |  |  |  |  |  |  |  |  |  |  |  |  |  |  |  |  |  |  |  |  |  |
| --- | --- | --- | --- | --- | --- | --- | --- | --- | --- | --- | --- | --- | --- | --- | --- | --- | --- | --- | --- | --- | --- | --- | --- | --- | --- | --- | --- | --- | --- | --- | --- | --- | --- | --- | --- |

P82885|VESP\_NAJKA

back to list

  

| Protein Coverage
| Supporting Peptides
|

Protein Coverage:

Supporting Peptides:

| Peptide | Uniq | -10lgP | Mass | Length | ppm | m/z | z | RT | Fraction | Scan | Source File | Area F4 | Area F1 | Area F10 | Area F2 | Area F3 | Area F5 | Area F6 | Area F7 | Area F8 | Area F9 | #Feature | #Feature F4 | #Feature F1 | #Feature F10 | #Feature F2 | #Feature F3 | #Feature F5 | #Feature F6 | #Feature F7 | #Feature F8 | #Feature F9 | Start | End | PTM |
| --- | --- | --- | --- | --- | --- | --- | --- | --- | --- | --- | --- | --- | --- | --- | --- | --- | --- | --- | --- | --- | --- | --- | --- | --- | --- | --- | --- | --- | --- | --- | --- | --- | --- | --- | --- |
| K.TVENVGVSQVAPDNPER.F | Y | 81.36 | 1809.8856 | 17 | 1.1 | 905.9511 | 2 | 25.85 | 8 | F8:5661 | 27122019\_RID\_1315\_NaNaWb\_F7.raw |  |  |  |  |  |  |  | 0 |  |  | 1 | 0 | 0 | 0 | 0 | 0 | 0 | 0 | 1 | 0 | 0 | 30 | 46 |  |
| K.ADVTFDSNTAFESLVVSPDK.K | Y | 63.65 | 2141.0164 | 20 | -0.3 | 1071.5151 | 2 | 58.07 | 9 | F9:22296 | 27122019\_RID\_1315\_NaNaWb\_F8.raw |  |  |  |  |  |  |  |  | 1.85E7 |  | 1 | 0 | 0 | 0 | 0 | 0 | 0 | 0 | 0 | 1 | 0 | 9 | 28 |  |
| K.ADVTFDSNTAFESLVVSPDKK.T | Y | 62.93 | 2269.1113 | 21 | -0.4 | 757.3774 | 3 | 49.27 | 9 | F9:16804 | 27122019\_RID\_1315\_NaNaWb\_F8.raw |  |  |  |  |  |  |  |  | 9.95E7 |  | 1 | 0 | 0 | 0 | 0 | 0 | 0 | 0 | 0 | 1 | 0 | 9 | 29 |  |
| R.FDGSPC(+57.02)VLGSPGFR.S | Y | 51.41 | 1494.6925 | 14 | 0.6 | 748.3540 | 2 | 40.76 | 9 | F9:12595 | 27122019\_RID\_1315\_NaNaWb\_F8.raw |  |  |  |  |  |  |  |  | 5.61E7 |  | 2 | 0 | 0 | 0 | 0 | 0 | 0 | 0 | 0 | 2 | 0 | 47 | 60 | Carbamidomethylation |
| total 4 peptides |  |  |  |  |  |  |  |  |  |  |  |  |  |  |  |  |  |  |  |  |  |  |  |  |  |  |  |  |  |  |  |  |  |  |  |
| --- | --- | --- | --- | --- | --- | --- | --- | --- | --- | --- | --- | --- | --- | --- | --- | --- | --- | --- | --- | --- | --- | --- | --- | --- | --- | --- | --- | --- | --- | --- | --- | --- | --- | --- | --- |

Q91137|3SOFL\_NAJAT

back to list

  

| Protein Coverage
| Supporting Peptides
|

Protein Coverage:

Supporting Peptides:

| Peptide | Uniq | -10lgP | Mass | Length | ppm | m/z | z | RT | Fraction | Scan | Source File | Area F4 | Area F1 | Area F10 | Area F2 | Area F3 | Area F5 | Area F6 | Area F7 | Area F8 | Area F9 | #Feature | #Feature F4 | #Feature F1 | #Feature F10 | #Feature F2 | #Feature F3 | #Feature F5 | #Feature F6 | #Feature F7 | #Feature F8 | #Feature F9 | Start | End | PTM |
| --- | --- | --- | --- | --- | --- | --- | --- | --- | --- | --- | --- | --- | --- | --- | --- | --- | --- | --- | --- | --- | --- | --- | --- | --- | --- | --- | --- | --- | --- | --- | --- | --- | --- | --- | --- |
| K.C(+57.02)HNTQLPFIYK.T | Y | 85.60 | 1419.6969 | 11 | 0.3 | 710.8560 | 2 | 29.97 | 7 | F7:9696 | 27122019\_RID\_1315\_NaNaWb\_F6.raw | 3.5E5 |  |  |  |  |  | 6.33E7 | 8.73E8 | 0 |  | 12 | 1 | 0 | 0 | 0 | 0 | 0 | 5 | 5 | 1 | 0 | 24 | 34 | Carbamidomethylation |
| T.LKC(+57.02)HNTQLPFIYK.T | Y | 42.37 | 1660.8759 | 13 | -0.5 | 554.6323 | 3 | 24.63 | 8 | F8:5254 | 27122019\_RID\_1315\_NaNaWb\_F7.raw |  |  |  |  |  |  |  | 6.88E6 |  |  | 1 | 0 | 0 | 0 | 0 | 0 | 0 | 0 | 1 | 0 | 0 | 22 | 34 | Carbamidomethylation |
| H.NTQLPFIYK.T | Y | 41.86 | 1122.6073 | 9 | 1.1 | 562.3115 | 2 | 40.87 | 8 | F8:9948 | 27122019\_RID\_1315\_NaNaWb\_F7.raw |  |  |  |  |  |  |  | 1.12E7 |  |  | 1 | 0 | 0 | 0 | 0 | 0 | 0 | 0 | 1 | 0 | 0 | 26 | 34 |  |
| total 3 peptides |  |  |  |  |  |  |  |  |  |  |  |  |  |  |  |  |  |  |  |  |  |  |  |  |  |  |  |  |  |  |  |  |  |  |  |
| --- | --- | --- | --- | --- | --- | --- | --- | --- | --- | --- | --- | --- | --- | --- | --- | --- | --- | --- | --- | --- | --- | --- | --- | --- | --- | --- | --- | --- | --- | --- | --- | --- | --- | --- | --- |

Q9W716|3SOFV\_NAJAT

back to list

  

| Protein Coverage
| Supporting Peptides
|

Protein Coverage:

Supporting Peptides:

| Peptide | Uniq | -10lgP | Mass | Length | ppm | m/z | z | RT | Fraction | Scan | Source File | Area F4 | Area F1 | Area F10 | Area F2 | Area F3 | Area F5 | Area F6 | Area F7 | Area F8 | Area F9 | #Feature | #Feature F4 | #Feature F1 | #Feature F10 | #Feature F2 | #Feature F3 | #Feature F5 | #Feature F6 | #Feature F7 | #Feature F8 | #Feature F9 | Start | End | PTM |
| --- | --- | --- | --- | --- | --- | --- | --- | --- | --- | --- | --- | --- | --- | --- | --- | --- | --- | --- | --- | --- | --- | --- | --- | --- | --- | --- | --- | --- | --- | --- | --- | --- | --- | --- | --- |
| K.C(+57.02)HNTQLPFIYK.T | Y | 85.60 | 1419.6969 | 11 | 0.3 | 710.8560 | 2 | 29.97 | 7 | F7:9696 | 27122019\_RID\_1315\_NaNaWb\_F6.raw | 3.5E5 |  |  |  |  |  | 6.33E7 | 8.73E8 | 0 |  | 12 | 1 | 0 | 0 | 0 | 0 | 0 | 5 | 5 | 1 | 0 | 24 | 34 | Carbamidomethylation |
| T.LKC(+57.02)HNTQLPFIYK.T | Y | 42.37 | 1660.8759 | 13 | -0.5 | 554.6323 | 3 | 24.63 | 8 | F8:5254 | 27122019\_RID\_1315\_NaNaWb\_F7.raw |  |  |  |  |  |  |  | 6.88E6 |  |  | 1 | 0 | 0 | 0 | 0 | 0 | 0 | 0 | 1 | 0 | 0 | 22 | 34 | Carbamidomethylation |
| H.NTQLPFIYK.T | Y | 41.86 | 1122.6073 | 9 | 1.1 | 562.3115 | 2 | 40.87 | 8 | F8:9948 | 27122019\_RID\_1315\_NaNaWb\_F7.raw |  |  |  |  |  |  |  | 1.12E7 |  |  | 1 | 0 | 0 | 0 | 0 | 0 | 0 | 0 | 1 | 0 | 0 | 26 | 34 |  |
| total 3 peptides |  |  |  |  |  |  |  |  |  |  |  |  |  |  |  |  |  |  |  |  |  |  |  |  |  |  |  |  |  |  |  |  |  |  |  |
| --- | --- | --- | --- | --- | --- | --- | --- | --- | --- | --- | --- | --- | --- | --- | --- | --- | --- | --- | --- | --- | --- | --- | --- | --- | --- | --- | --- | --- | --- | --- | --- | --- | --- | --- | --- |

Q91126|3SOFA\_NAJAT

back to list

  

| Protein Coverage
| Supporting Peptides
|

Protein Coverage:

Supporting Peptides:

| Peptide | Uniq | -10lgP | Mass | Length | ppm | m/z | z | RT | Fraction | Scan | Source File | Area F4 | Area F1 | Area F10 | Area F2 | Area F3 | Area F5 | Area F6 | Area F7 | Area F8 | Area F9 | #Feature | #Feature F4 | #Feature F1 | #Feature F10 | #Feature F2 | #Feature F3 | #Feature F5 | #Feature F6 | #Feature F7 | #Feature F8 | #Feature F9 | Start | End | PTM |
| --- | --- | --- | --- | --- | --- | --- | --- | --- | --- | --- | --- | --- | --- | --- | --- | --- | --- | --- | --- | --- | --- | --- | --- | --- | --- | --- | --- | --- | --- | --- | --- | --- | --- | --- | --- |
| K.C(+57.02)HNTQLPFIYK.T | Y | 85.60 | 1419.6969 | 11 | 0.3 | 710.8560 | 2 | 29.97 | 7 | F7:9696 | 27122019\_RID\_1315\_NaNaWb\_F6.raw | 3.5E5 |  |  |  |  |  | 6.33E7 | 8.73E8 | 0 |  | 12 | 1 | 0 | 0 | 0 | 0 | 0 | 5 | 5 | 1 | 0 | 24 | 34 | Carbamidomethylation |
| T.LKC(+57.02)HNTQLPFIYK.T | Y | 42.37 | 1660.8759 | 13 | -0.5 | 554.6323 | 3 | 24.63 | 8 | F8:5254 | 27122019\_RID\_1315\_NaNaWb\_F7.raw |  |  |  |  |  |  |  | 6.88E6 |  |  | 1 | 0 | 0 | 0 | 0 | 0 | 0 | 0 | 1 | 0 | 0 | 22 | 34 | Carbamidomethylation |
| H.NTQLPFIYK.T | Y | 41.86 | 1122.6073 | 9 | 1.1 | 562.3115 | 2 | 40.87 | 8 | F8:9948 | 27122019\_RID\_1315\_NaNaWb\_F7.raw |  |  |  |  |  |  |  | 1.12E7 |  |  | 1 | 0 | 0 | 0 | 0 | 0 | 0 | 0 | 1 | 0 | 0 | 26 | 34 |  |
| total 3 peptides |  |  |  |  |  |  |  |  |  |  |  |  |  |  |  |  |  |  |  |  |  |  |  |  |  |  |  |  |  |  |  |  |  |  |  |
| --- | --- | --- | --- | --- | --- | --- | --- | --- | --- | --- | --- | --- | --- | --- | --- | --- | --- | --- | --- | --- | --- | --- | --- | --- | --- | --- | --- | --- | --- | --- | --- | --- | --- | --- | --- |

Q91996|3SOFP\_NAJAT

back to list

  

| Protein Coverage
| Supporting Peptides
|

Protein Coverage:

Supporting Peptides:

| Peptide | Uniq | -10lgP | Mass | Length | ppm | m/z | z | RT | Fraction | Scan | Source File | Area F4 | Area F1 | Area F10 | Area F2 | Area F3 | Area F5 | Area F6 | Area F7 | Area F8 | Area F9 | #Feature | #Feature F4 | #Feature F1 | #Feature F10 | #Feature F2 | #Feature F3 | #Feature F5 | #Feature F6 | #Feature F7 | #Feature F8 | #Feature F9 | Start | End | PTM |
| --- | --- | --- | --- | --- | --- | --- | --- | --- | --- | --- | --- | --- | --- | --- | --- | --- | --- | --- | --- | --- | --- | --- | --- | --- | --- | --- | --- | --- | --- | --- | --- | --- | --- | --- | --- |
| K.C(+57.02)HNTQLPFIYK.T | Y | 85.60 | 1419.6969 | 11 | 0.3 | 710.8560 | 2 | 29.97 | 7 | F7:9696 | 27122019\_RID\_1315\_NaNaWb\_F6.raw | 3.5E5 |  |  |  |  |  | 6.33E7 | 8.73E8 | 0 |  | 12 | 1 | 0 | 0 | 0 | 0 | 0 | 5 | 5 | 1 | 0 | 24 | 34 | Carbamidomethylation |
| T.LKC(+57.02)HNTQLPFIYK.T | Y | 42.37 | 1660.8759 | 13 | -0.5 | 554.6323 | 3 | 24.63 | 8 | F8:5254 | 27122019\_RID\_1315\_NaNaWb\_F7.raw |  |  |  |  |  |  |  | 6.88E6 |  |  | 1 | 0 | 0 | 0 | 0 | 0 | 0 | 0 | 1 | 0 | 0 | 22 | 34 | Carbamidomethylation |
| H.NTQLPFIYK.T | Y | 41.86 | 1122.6073 | 9 | 1.1 | 562.3115 | 2 | 40.87 | 8 | F8:9948 | 27122019\_RID\_1315\_NaNaWb\_F7.raw |  |  |  |  |  |  |  | 1.12E7 |  |  | 1 | 0 | 0 | 0 | 0 | 0 | 0 | 0 | 1 | 0 | 0 | 26 | 34 |  |
| total 3 peptides |  |  |  |  |  |  |  |  |  |  |  |  |  |  |  |  |  |  |  |  |  |  |  |  |  |  |  |  |  |  |  |  |  |  |  |
| --- | --- | --- | --- | --- | --- | --- | --- | --- | --- | --- | --- | --- | --- | --- | --- | --- | --- | --- | --- | --- | --- | --- | --- | --- | --- | --- | --- | --- | --- | --- | --- | --- | --- | --- | --- |

P62377|3SOFL\_NAJNA

back to list

  

| Protein Coverage
| Supporting Peptides
|

Protein Coverage:

Supporting Peptides:

| Peptide | Uniq | -10lgP | Mass | Length | ppm | m/z | z | RT | Fraction | Scan | Source File | Area F4 | Area F1 | Area F10 | Area F2 | Area F3 | Area F5 | Area F6 | Area F7 | Area F8 | Area F9 | #Feature | #Feature F4 | #Feature F1 | #Feature F10 | #Feature F2 | #Feature F3 | #Feature F5 | #Feature F6 | #Feature F7 | #Feature F8 | #Feature F9 | Start | End | PTM |
| --- | --- | --- | --- | --- | --- | --- | --- | --- | --- | --- | --- | --- | --- | --- | --- | --- | --- | --- | --- | --- | --- | --- | --- | --- | --- | --- | --- | --- | --- | --- | --- | --- | --- | --- | --- |
| K.C(+57.02)HNTQLPFIYK.T | Y | 85.60 | 1419.6969 | 11 | 0.3 | 710.8560 | 2 | 29.97 | 7 | F7:9696 | 27122019\_RID\_1315\_NaNaWb\_F6.raw | 3.5E5 |  |  |  |  |  | 6.33E7 | 8.73E8 | 0 |  | 12 | 1 | 0 | 0 | 0 | 0 | 0 | 5 | 5 | 1 | 0 | 3 | 13 | Carbamidomethylation |
| LKC(+57.02)HNTQLPFIYK.T | Y | 42.37 | 1660.8759 | 13 | -0.5 | 554.6323 | 3 | 24.63 | 8 | F8:5254 | 27122019\_RID\_1315\_NaNaWb\_F7.raw |  |  |  |  |  |  |  | 6.88E6 |  |  | 1 | 0 | 0 | 0 | 0 | 0 | 0 | 0 | 1 | 0 | 0 | 1 | 13 | Carbamidomethylation |
| H.NTQLPFIYK.T | Y | 41.86 | 1122.6073 | 9 | 1.1 | 562.3115 | 2 | 40.87 | 8 | F8:9948 | 27122019\_RID\_1315\_NaNaWb\_F7.raw |  |  |  |  |  |  |  | 1.12E7 |  |  | 1 | 0 | 0 | 0 | 0 | 0 | 0 | 0 | 1 | 0 | 0 | 5 | 13 |  |
| total 3 peptides |  |  |  |  |  |  |  |  |  |  |  |  |  |  |  |  |  |  |  |  |  |  |  |  |  |  |  |  |  |  |  |  |  |  |  |
| --- | --- | --- | --- | --- | --- | --- | --- | --- | --- | --- | --- | --- | --- | --- | --- | --- | --- | --- | --- | --- | --- | --- | --- | --- | --- | --- | --- | --- | --- | --- | --- | --- | --- | --- | --- |

P62375|3SOF5\_NAJAT

back to list

  

| Protein Coverage
| Supporting Peptides
|

Protein Coverage:

Supporting Peptides:

| Peptide | Uniq | -10lgP | Mass | Length | ppm | m/z | z | RT | Fraction | Scan | Source File | Area F4 | Area F1 | Area F10 | Area F2 | Area F3 | Area F5 | Area F6 | Area F7 | Area F8 | Area F9 | #Feature | #Feature F4 | #Feature F1 | #Feature F10 | #Feature F2 | #Feature F3 | #Feature F5 | #Feature F6 | #Feature F7 | #Feature F8 | #Feature F9 | Start | End | PTM |
| --- | --- | --- | --- | --- | --- | --- | --- | --- | --- | --- | --- | --- | --- | --- | --- | --- | --- | --- | --- | --- | --- | --- | --- | --- | --- | --- | --- | --- | --- | --- | --- | --- | --- | --- | --- |
| K.C(+57.02)HNTQLPFIYK.T | Y | 85.60 | 1419.6969 | 11 | 0.3 | 710.8560 | 2 | 29.97 | 7 | F7:9696 | 27122019\_RID\_1315\_NaNaWb\_F6.raw | 3.5E5 |  |  |  |  |  | 6.33E7 | 8.73E8 | 0 |  | 12 | 1 | 0 | 0 | 0 | 0 | 0 | 5 | 5 | 1 | 0 | 24 | 34 | Carbamidomethylation |
| T.LKC(+57.02)HNTQLPFIYK.T | Y | 42.37 | 1660.8759 | 13 | -0.5 | 554.6323 | 3 | 24.63 | 8 | F8:5254 | 27122019\_RID\_1315\_NaNaWb\_F7.raw |  |  |  |  |  |  |  | 6.88E6 |  |  | 1 | 0 | 0 | 0 | 0 | 0 | 0 | 0 | 1 | 0 | 0 | 22 | 34 | Carbamidomethylation |
| H.NTQLPFIYK.T | Y | 41.86 | 1122.6073 | 9 | 1.1 | 562.3115 | 2 | 40.87 | 8 | F8:9948 | 27122019\_RID\_1315\_NaNaWb\_F7.raw |  |  |  |  |  |  |  | 1.12E7 |  |  | 1 | 0 | 0 | 0 | 0 | 0 | 0 | 0 | 1 | 0 | 0 | 26 | 34 |  |
| total 3 peptides |  |  |  |  |  |  |  |  |  |  |  |  |  |  |  |  |  |  |  |  |  |  |  |  |  |  |  |  |  |  |  |  |  |  |  |
| --- | --- | --- | --- | --- | --- | --- | --- | --- | --- | --- | --- | --- | --- | --- | --- | --- | --- | --- | --- | --- | --- | --- | --- | --- | --- | --- | --- | --- | --- | --- | --- | --- | --- | --- | --- |

P62390|3SOFB\_NAJHA

back to list

  

| Protein Coverage
| Supporting Peptides
|

Protein Coverage:

Supporting Peptides:

| Peptide | Uniq | -10lgP | Mass | Length | ppm | m/z | z | RT | Fraction | Scan | Source File | Area F4 | Area F1 | Area F10 | Area F2 | Area F3 | Area F5 | Area F6 | Area F7 | Area F8 | Area F9 | #Feature | #Feature F4 | #Feature F1 | #Feature F10 | #Feature F2 | #Feature F3 | #Feature F5 | #Feature F6 | #Feature F7 | #Feature F8 | #Feature F9 | Start | End | PTM |
| --- | --- | --- | --- | --- | --- | --- | --- | --- | --- | --- | --- | --- | --- | --- | --- | --- | --- | --- | --- | --- | --- | --- | --- | --- | --- | --- | --- | --- | --- | --- | --- | --- | --- | --- | --- |
| K.C(+57.02)HNTQLPFIYK.T | Y | 85.60 | 1419.6969 | 11 | 0.3 | 710.8560 | 2 | 29.97 | 7 | F7:9696 | 27122019\_RID\_1315\_NaNaWb\_F6.raw | 3.5E5 |  |  |  |  |  | 6.33E7 | 8.73E8 | 0 |  | 12 | 1 | 0 | 0 | 0 | 0 | 0 | 5 | 5 | 1 | 0 | 3 | 13 | Carbamidomethylation |
| LKC(+57.02)HNTQLPFIYK.T | Y | 42.37 | 1660.8759 | 13 | -0.5 | 554.6323 | 3 | 24.63 | 8 | F8:5254 | 27122019\_RID\_1315\_NaNaWb\_F7.raw |  |  |  |  |  |  |  | 6.88E6 |  |  | 1 | 0 | 0 | 0 | 0 | 0 | 0 | 0 | 1 | 0 | 0 | 1 | 13 | Carbamidomethylation |
| H.NTQLPFIYK.T | Y | 41.86 | 1122.6073 | 9 | 1.1 | 562.3115 | 2 | 40.87 | 8 | F8:9948 | 27122019\_RID\_1315\_NaNaWb\_F7.raw |  |  |  |  |  |  |  | 1.12E7 |  |  | 1 | 0 | 0 | 0 | 0 | 0 | 0 | 0 | 1 | 0 | 0 | 5 | 13 |  |
| total 3 peptides |  |  |  |  |  |  |  |  |  |  |  |  |  |  |  |  |  |  |  |  |  |  |  |  |  |  |  |  |  |  |  |  |  |  |  |
| --- | --- | --- | --- | --- | --- | --- | --- | --- | --- | --- | --- | --- | --- | --- | --- | --- | --- | --- | --- | --- | --- | --- | --- | --- | --- | --- | --- | --- | --- | --- | --- | --- | --- | --- | --- |

P62394|3SOFB\_NAJHH

back to list

  

| Protein Coverage
| Supporting Peptides
|

Protein Coverage:

Supporting Peptides:

| Peptide | Uniq | -10lgP | Mass | Length | ppm | m/z | z | RT | Fraction | Scan | Source File | Area F4 | Area F1 | Area F10 | Area F2 | Area F3 | Area F5 | Area F6 | Area F7 | Area F8 | Area F9 | #Feature | #Feature F4 | #Feature F1 | #Feature F10 | #Feature F2 | #Feature F3 | #Feature F5 | #Feature F6 | #Feature F7 | #Feature F8 | #Feature F9 | Start | End | PTM |
| --- | --- | --- | --- | --- | --- | --- | --- | --- | --- | --- | --- | --- | --- | --- | --- | --- | --- | --- | --- | --- | --- | --- | --- | --- | --- | --- | --- | --- | --- | --- | --- | --- | --- | --- | --- |
| K.C(+57.02)HNTQLPFIYK.T | Y | 85.60 | 1419.6969 | 11 | 0.3 | 710.8560 | 2 | 29.97 | 7 | F7:9696 | 27122019\_RID\_1315\_NaNaWb\_F6.raw | 3.5E5 |  |  |  |  |  | 6.33E7 | 8.73E8 | 0 |  | 12 | 1 | 0 | 0 | 0 | 0 | 0 | 5 | 5 | 1 | 0 | 3 | 13 | Carbamidomethylation |
| LKC(+57.02)HNTQLPFIYK.T | Y | 42.37 | 1660.8759 | 13 | -0.5 | 554.6323 | 3 | 24.63 | 8 | F8:5254 | 27122019\_RID\_1315\_NaNaWb\_F7.raw |  |  |  |  |  |  |  | 6.88E6 |  |  | 1 | 0 | 0 | 0 | 0 | 0 | 0 | 0 | 1 | 0 | 0 | 1 | 13 | Carbamidomethylation |
| H.NTQLPFIYK.T | Y | 41.86 | 1122.6073 | 9 | 1.1 | 562.3115 | 2 | 40.87 | 8 | F8:9948 | 27122019\_RID\_1315\_NaNaWb\_F7.raw |  |  |  |  |  |  |  | 1.12E7 |  |  | 1 | 0 | 0 | 0 | 0 | 0 | 0 | 0 | 1 | 0 | 0 | 5 | 13 |  |
| total 3 peptides |  |  |  |  |  |  |  |  |  |  |  |  |  |  |  |  |  |  |  |  |  |  |  |  |  |  |  |  |  |  |  |  |  |  |  |
| --- | --- | --- | --- | --- | --- | --- | --- | --- | --- | --- | --- | --- | --- | --- | --- | --- | --- | --- | --- | --- | --- | --- | --- | --- | --- | --- | --- | --- | --- | --- | --- | --- | --- | --- | --- |

P14541|3SOFH\_NAJKA

back to list

  

| Protein Coverage
| Supporting Peptides
|

Protein Coverage:

Supporting Peptides:

| Peptide | Uniq | -10lgP | Mass | Length | ppm | m/z | z | RT | Fraction | Scan | Source File | Area F4 | Area F1 | Area F10 | Area F2 | Area F3 | Area F5 | Area F6 | Area F7 | Area F8 | Area F9 | #Feature | #Feature F4 | #Feature F1 | #Feature F10 | #Feature F2 | #Feature F3 | #Feature F5 | #Feature F6 | #Feature F7 | #Feature F8 | #Feature F9 | Start | End | PTM |
| --- | --- | --- | --- | --- | --- | --- | --- | --- | --- | --- | --- | --- | --- | --- | --- | --- | --- | --- | --- | --- | --- | --- | --- | --- | --- | --- | --- | --- | --- | --- | --- | --- | --- | --- | --- |
| K.C(+57.02)HNTQLPFIYK.T | Y | 85.60 | 1419.6969 | 11 | 0.3 | 710.8560 | 2 | 29.97 | 7 | F7:9696 | 27122019\_RID\_1315\_NaNaWb\_F6.raw | 3.5E5 |  |  |  |  |  | 6.33E7 | 8.73E8 | 0 |  | 12 | 1 | 0 | 0 | 0 | 0 | 0 | 5 | 5 | 1 | 0 | 3 | 13 | Carbamidomethylation |
| LKC(+57.02)HNTQLPFIYK.T | Y | 42.37 | 1660.8759 | 13 | -0.5 | 554.6323 | 3 | 24.63 | 8 | F8:5254 | 27122019\_RID\_1315\_NaNaWb\_F7.raw |  |  |  |  |  |  |  | 6.88E6 |  |  | 1 | 0 | 0 | 0 | 0 | 0 | 0 | 0 | 1 | 0 | 0 | 1 | 13 | Carbamidomethylation |
| H.NTQLPFIYK.T | Y | 41.86 | 1122.6073 | 9 | 1.1 | 562.3115 | 2 | 40.87 | 8 | F8:9948 | 27122019\_RID\_1315\_NaNaWb\_F7.raw |  |  |  |  |  |  |  | 1.12E7 |  |  | 1 | 0 | 0 | 0 | 0 | 0 | 0 | 0 | 1 | 0 | 0 | 5 | 13 |  |
| total 3 peptides |  |  |  |  |  |  |  |  |  |  |  |  |  |  |  |  |  |  |  |  |  |  |  |  |  |  |  |  |  |  |  |  |  |  |  |
| --- | --- | --- | --- | --- | --- | --- | --- | --- | --- | --- | --- | --- | --- | --- | --- | --- | --- | --- | --- | --- | --- | --- | --- | --- | --- | --- | --- | --- | --- | --- | --- | --- | --- | --- | --- |

P20229|VKTTI\_NAJNA

back to list

  

| Protein Coverage
| Supporting Peptides
|

Protein Coverage:

Supporting Peptides:

| Peptide | Uniq | -10lgP | Mass | Length | ppm | m/z | z | RT | Fraction | Scan | Source File | Area F4 | Area F1 | Area F10 | Area F2 | Area F3 | Area F5 | Area F6 | Area F7 | Area F8 | Area F9 | #Feature | #Feature F4 | #Feature F1 | #Feature F10 | #Feature F2 | #Feature F3 | #Feature F5 | #Feature F6 | #Feature F7 | #Feature F8 | #Feature F9 | Start | End | PTM |
| --- | --- | --- | --- | --- | --- | --- | --- | --- | --- | --- | --- | --- | --- | --- | --- | --- | --- | --- | --- | --- | --- | --- | --- | --- | --- | --- | --- | --- | --- | --- | --- | --- | --- | --- | --- |
| K.FIYGGC(+57.02)GGNANR.F | Y | 63.07 | 1284.5669 | 12 | 1.0 | 643.2914 | 2 | 25.86 | 1 | F1:9026 | 09012020\_NaNaWb\_F4.raw | 3.05E5 |  |  |  |  |  |  |  |  |  | 1 | 1 | 0 | 0 | 0 | 0 | 0 | 0 | 0 | 0 | 0 | 33 | 44 | Carbamidomethylation |
| RPGFC(+57.02)ELPAAK.G | Y | 62.22 | 1244.6335 | 11 | -0.1 | 415.8851 | 3 | 26.40 | 1 | F1:9267 | 09012020\_NaNaWb\_F4.raw | 3.34E5 |  |  |  | 2.66E8 | 3.61E5 |  |  |  |  | 4 | 1 | 0 | 0 | 0 | 1 | 2 | 0 | 0 | 0 | 0 | 1 | 11 | Carbamidomethylation |
| P.GFC(+57.02)ELPAAK.G | Y | 57.75 | 991.4797 | 9 | -0.3 | 496.7469 | 2 | 33.04 | 1 | F1:13464 | 09012020\_NaNaWb\_F4.raw | 4.66E5 |  |  |  |  |  |  |  |  |  | 1 | 1 | 0 | 0 | 0 | 0 | 0 | 0 | 0 | 0 | 0 | 3 | 11 | Carbamidomethylation |
| K.AHKPAFYYNK.D | Y | 57.35 | 1237.6244 | 10 | 0.2 | 619.8196 | 2 | 12.29 | 5 | F5:2073 | 27122019\_RID\_1315\_NaNaWb\_F3.raw |  |  |  |  | 6.29E7 |  |  |  |  |  | 2 | 0 | 0 | 0 | 0 | 2 | 0 | 0 | 0 | 0 | 0 | 16 | 25 |  |
| G.FC(+57.02)ELPAAK.G | Y | 50.42 | 934.4582 | 8 | 0.0 | 468.2364 | 2 | 28.62 | 1 | F1:10485 | 09012020\_NaNaWb\_F4.raw | 1.09E6 |  |  |  |  |  |  |  |  |  | 1 | 1 | 0 | 0 | 0 | 0 | 0 | 0 | 0 | 0 | 0 | 4 | 11 | Carbamidomethylation |
| total 5 peptides |  |  |  |  |  |  |  |  |  |  |  |  |  |  |  |  |  |  |  |  |  |  |  |  |  |  |  |  |  |  |  |  |  |  |  |
| --- | --- | --- | --- | --- | --- | --- | --- | --- | --- | --- | --- | --- | --- | --- | --- | --- | --- | --- | --- | --- | --- | --- | --- | --- | --- | --- | --- | --- | --- | --- | --- | --- | --- | --- | --- |

P80245|3SAN\_NAJAT

back to list

  

| Protein Coverage
| Supporting Peptides
|

Protein Coverage:

Supporting Peptides:

| Peptide | Uniq | -10lgP | Mass | Length | ppm | m/z | z | RT | Fraction | Scan | Source File | Area F4 | Area F1 | Area F10 | Area F2 | Area F3 | Area F5 | Area F6 | Area F7 | Area F8 | Area F9 | #Feature | #Feature F4 | #Feature F1 | #Feature F10 | #Feature F2 | #Feature F3 | #Feature F5 | #Feature F6 | #Feature F7 | #Feature F8 | #Feature F9 | Start | End | PTM |
| --- | --- | --- | --- | --- | --- | --- | --- | --- | --- | --- | --- | --- | --- | --- | --- | --- | --- | --- | --- | --- | --- | --- | --- | --- | --- | --- | --- | --- | --- | --- | --- | --- | --- | --- | --- |
| R.GC(+57.02)IDVC(+57.02)PK.S | N | 61.16 | 947.4205 | 8 | 0.3 | 474.7177 | 2 | 15.80 | 7 | F7:3597 | 27122019\_RID\_1315\_NaNaWb\_F6.raw | 1.83E6 |  |  |  |  | 4.15E5 | 1.88E9 | 3E8 | 4.74E9 |  | 11 | 1 | 0 | 0 | 0 | 0 | 1 | 3 | 1 | 5 | 0 | 58 | 65 | Carbamidomethylation |
| K.SSLLVKYVC(+57.02)C(+57.02)NTDRC(+57.02)N | N | 50.38 | 1987.8914 | 16 | 0.3 | 663.6379 | 3 | 28.94 | 9 | F9:8080 | 27122019\_RID\_1315\_NaNaWb\_F8.raw |  |  |  |  |  |  |  |  | 4.69E8 |  | 2 | 0 | 0 | 0 | 0 | 0 | 0 | 0 | 0 | 2 | 0 | 66 | 81 | Carbamidomethylation |
| K.SSLLVKYVC(+57.02)C(+57.02)NTDR.C | N | 47.78 | 1713.8179 | 14 | 0.3 | 857.9165 | 2 | 27.06 | 9 | F9:7132 | 27122019\_RID\_1315\_NaNaWb\_F8.raw |  |  |  |  |  |  |  |  | 6.49E7 |  | 3 | 0 | 0 | 0 | 0 | 0 | 0 | 0 | 0 | 3 | 0 | 66 | 79 | Carbamidomethylation |
| K.C(+57.02)NQLIPPFYK.T | Y | 42.79 | 1278.6431 | 10 | -0.5 | 640.3285 | 2 | 42.45 | 7 | F7:14640 | 27122019\_RID\_1315\_NaNaWb\_F6.raw |  |  |  |  |  |  | 2.61E5 |  |  |  | 1 | 0 | 0 | 0 | 0 | 0 | 0 | 1 | 0 | 0 | 0 | 24 | 33 | Carbamidomethylation |
| total 4 peptides |  |  |  |  |  |  |  |  |  |  |  |  |  |  |  |  |  |  |  |  |  |  |  |  |  |  |  |  |  |  |  |  |  |  |  |
| --- | --- | --- | --- | --- | --- | --- | --- | --- | --- | --- | --- | --- | --- | --- | --- | --- | --- | --- | --- | --- | --- | --- | --- | --- | --- | --- | --- | --- | --- | --- | --- | --- | --- | --- | --- |

Q98965|3SA6\_NAJAT

back to list

  

| Protein Coverage
| Supporting Peptides
|

Protein Coverage:

Supporting Peptides:

| Peptide | Uniq | -10lgP | Mass | Length | ppm | m/z | z | RT | Fraction | Scan | Source File | Area F4 | Area F1 | Area F10 | Area F2 | Area F3 | Area F5 | Area F6 | Area F7 | Area F8 | Area F9 | #Feature | #Feature F4 | #Feature F1 | #Feature F10 | #Feature F2 | #Feature F3 | #Feature F5 | #Feature F6 | #Feature F7 | #Feature F8 | #Feature F9 | Start | End | PTM |
| --- | --- | --- | --- | --- | --- | --- | --- | --- | --- | --- | --- | --- | --- | --- | --- | --- | --- | --- | --- | --- | --- | --- | --- | --- | --- | --- | --- | --- | --- | --- | --- | --- | --- | --- | --- |
| R.GC(+57.02)IDVC(+57.02)PK.S | N | 61.16 | 947.4205 | 8 | 0.3 | 474.7177 | 2 | 15.80 | 7 | F7:3597 | 27122019\_RID\_1315\_NaNaWb\_F6.raw | 1.83E6 |  |  |  |  | 4.15E5 | 1.88E9 | 3E8 | 4.74E9 |  | 11 | 1 | 0 | 0 | 0 | 0 | 1 | 3 | 1 | 5 | 0 | 58 | 65 | Carbamidomethylation |
| K.SSLLVKYVC(+57.02)C(+57.02)NTDRC(+57.02)N.N | N | 50.38 | 1987.8914 | 16 | 0.3 | 663.6379 | 3 | 28.94 | 9 | F9:8080 | 27122019\_RID\_1315\_NaNaWb\_F8.raw |  |  |  |  |  |  |  |  | 4.69E8 |  | 2 | 0 | 0 | 0 | 0 | 0 | 0 | 0 | 0 | 2 | 0 | 66 | 81 | Carbamidomethylation |
| K.SSLLVKYVC(+57.02)C(+57.02)NTDR.C | N | 47.78 | 1713.8179 | 14 | 0.3 | 857.9165 | 2 | 27.06 | 9 | F9:7132 | 27122019\_RID\_1315\_NaNaWb\_F8.raw |  |  |  |  |  |  |  |  | 6.49E7 |  | 3 | 0 | 0 | 0 | 0 | 0 | 0 | 0 | 0 | 3 | 0 | 66 | 79 | Carbamidomethylation |
| K.C(+57.02)NQLIPPFYK.T | Y | 42.79 | 1278.6431 | 10 | -0.5 | 640.3285 | 2 | 42.45 | 7 | F7:14640 | 27122019\_RID\_1315\_NaNaWb\_F6.raw |  |  |  |  |  |  | 2.61E5 |  |  |  | 1 | 0 | 0 | 0 | 0 | 0 | 0 | 1 | 0 | 0 | 0 | 24 | 33 | Carbamidomethylation |
| total 4 peptides |  |  |  |  |  |  |  |  |  |  |  |  |  |  |  |  |  |  |  |  |  |  |  |  |  |  |  |  |  |  |  |  |  |  |  |
| --- | --- | --- | --- | --- | --- | --- | --- | --- | --- | --- | --- | --- | --- | --- | --- | --- | --- | --- | --- | --- | --- | --- | --- | --- | --- | --- | --- | --- | --- | --- | --- | --- | --- | --- | --- |

F8J2D0|PA214\_DRYCN

back to list

  

| Protein Coverage
| Supporting Peptides
|

Protein Coverage:

Supporting Peptides:

| Peptide | Uniq | -10lgP | Mass | Length | ppm | m/z | z | RT | Fraction | Scan | Source File | Area F4 | Area F1 | Area F10 | Area F2 | Area F3 | Area F5 | Area F6 | Area F7 | Area F8 | Area F9 | #Feature | #Feature F4 | #Feature F1 | #Feature F10 | #Feature F2 | #Feature F3 | #Feature F5 | #Feature F6 | #Feature F7 | #Feature F8 | #Feature F9 | Start | End | PTM |
| --- | --- | --- | --- | --- | --- | --- | --- | --- | --- | --- | --- | --- | --- | --- | --- | --- | --- | --- | --- | --- | --- | --- | --- | --- | --- | --- | --- | --- | --- | --- | --- | --- | --- | --- | --- |
| A.GGSGTPVDDLDR.C | N | 76.07 | 1187.5417 | 12 | 0.1 | 594.7782 | 2 | 22.36 | 7 | F7:6369 | 27122019\_RID\_1315\_NaNaWb\_F6.raw |  |  |  |  |  |  | 5.85E9 | 4.29E8 |  |  | 9 | 0 | 0 | 0 | 0 | 0 | 0 | 6 | 3 | 0 | 0 | 59 | 70 |  |
| G.GSGTPVDDLDR.C | N | 47.11 | 1130.5204 | 11 | 0.2 | 566.2676 | 2 | 21.06 | 7 | F7:5676 | 27122019\_RID\_1315\_NaNaWb\_F6.raw |  |  |  |  |  |  | 3.97E7 |  |  |  | 1 | 0 | 0 | 0 | 0 | 0 | 0 | 1 | 0 | 0 | 0 | 60 | 70 |  |
| G.AGGSGTPVDDLDR.C | Y | 41.29 | 1258.5789 | 13 | 0.0 | 630.2967 | 2 | 22.27 | 7 | F7:6285 | 27122019\_RID\_1315\_NaNaWb\_F6.raw |  |  |  |  |  |  | 3.4E6 |  |  |  | 1 | 0 | 0 | 0 | 0 | 0 | 0 | 1 | 0 | 0 | 0 | 58 | 70 |  |
| total 3 peptides |  |  |  |  |  |  |  |  |  |  |  |  |  |  |  |  |  |  |  |  |  |  |  |  |  |  |  |  |  |  |  |  |  |  |  |
| --- | --- | --- | --- | --- | --- | --- | --- | --- | --- | --- | --- | --- | --- | --- | --- | --- | --- | --- | --- | --- | --- | --- | --- | --- | --- | --- | --- | --- | --- | --- | --- | --- | --- | --- | --- |

F8J2D2|PA222\_DRYCN

back to list

  

| Protein Coverage
| Supporting Peptides
|

Protein Coverage:

Supporting Peptides:

| Peptide | Uniq | -10lgP | Mass | Length | ppm | m/z | z | RT | Fraction | Scan | Source File | Area F4 | Area F1 | Area F10 | Area F2 | Area F3 | Area F5 | Area F6 | Area F7 | Area F8 | Area F9 | #Feature | #Feature F4 | #Feature F1 | #Feature F10 | #Feature F2 | #Feature F3 | #Feature F5 | #Feature F6 | #Feature F7 | #Feature F8 | #Feature F9 | Start | End | PTM |
| --- | --- | --- | --- | --- | --- | --- | --- | --- | --- | --- | --- | --- | --- | --- | --- | --- | --- | --- | --- | --- | --- | --- | --- | --- | --- | --- | --- | --- | --- | --- | --- | --- | --- | --- | --- |
| A.GGSGTPVDDLDR.C | N | 76.07 | 1187.5417 | 12 | 0.1 | 594.7782 | 2 | 22.36 | 7 | F7:6369 | 27122019\_RID\_1315\_NaNaWb\_F6.raw |  |  |  |  |  |  | 5.85E9 | 4.29E8 |  |  | 9 | 0 | 0 | 0 | 0 | 0 | 0 | 6 | 3 | 0 | 0 | 59 | 70 |  |
| G.GSGTPVDDLDR.C | N | 47.11 | 1130.5204 | 11 | 0.2 | 566.2676 | 2 | 21.06 | 7 | F7:5676 | 27122019\_RID\_1315\_NaNaWb\_F6.raw |  |  |  |  |  |  | 3.97E7 |  |  |  | 1 | 0 | 0 | 0 | 0 | 0 | 0 | 1 | 0 | 0 | 0 | 60 | 70 |  |
| G.AGGSGTPVDDLDR.C | Y | 41.29 | 1258.5789 | 13 | 0.0 | 630.2967 | 2 | 22.27 | 7 | F7:6285 | 27122019\_RID\_1315\_NaNaWb\_F6.raw |  |  |  |  |  |  | 3.4E6 |  |  |  | 1 | 0 | 0 | 0 | 0 | 0 | 0 | 1 | 0 | 0 | 0 | 58 | 70 |  |
| total 3 peptides |  |  |  |  |  |  |  |  |  |  |  |  |  |  |  |  |  |  |  |  |  |  |  |  |  |  |  |  |  |  |  |  |  |  |  |
| --- | --- | --- | --- | --- | --- | --- | --- | --- | --- | --- | --- | --- | --- | --- | --- | --- | --- | --- | --- | --- | --- | --- | --- | --- | --- | --- | --- | --- | --- | --- | --- | --- | --- | --- | --- |

Q7Z794|K2C1B\_HUMAN

back to list

  

| Protein Coverage
| Supporting Peptides
|

Protein Coverage:

Supporting Peptides:

| Peptide | Uniq | -10lgP | Mass | Length | ppm | m/z | z | RT | Fraction | Scan | Source File | Area F4 | Area F1 | Area F10 | Area F2 | Area F3 | Area F5 | Area F6 | Area F7 | Area F8 | Area F9 | #Feature | #Feature F4 | #Feature F1 | #Feature F10 | #Feature F2 | #Feature F3 | #Feature F5 | #Feature F6 | #Feature F7 | #Feature F8 | #Feature F9 | Start | End | PTM |
| --- | --- | --- | --- | --- | --- | --- | --- | --- | --- | --- | --- | --- | --- | --- | --- | --- | --- | --- | --- | --- | --- | --- | --- | --- | --- | --- | --- | --- | --- | --- | --- | --- | --- | --- | --- |
| R.FLEQQNQVLQTK.W | N | 77.68 | 1474.7780 | 12 | 0.6 | 738.3967 | 2 | 37.47 | 1 | F1:16185 | 09012020\_NaNaWb\_F4.raw | 5.37E6 |  |  |  |  | 2.57E6 | 6.32E7 |  | 9.44E6 | 3E6 | 6 | 1 | 0 | 0 | 0 | 0 | 1 | 2 | 0 | 1 | 1 | 184 | 195 |  |
| R.TQYELIAQR.S | Y | 57.71 | 1120.5876 | 9 | -9.7 | 561.2957 | 2 | 34.91 | 1 | F1:14922 | 09012020\_NaNaWb\_F4.raw | 1.66E6 |  |  |  |  |  |  |  |  |  | 2 | 2 | 0 | 0 | 0 | 0 | 0 | 0 | 0 | 0 | 0 | 340 | 348 |  |
| K.FASFIDK.V | N | 45.32 | 826.4225 | 7 | -0.7 | 414.2182 | 2 | 39.06 | 1 | F1:17107 | 09012020\_NaNaWb\_F4.raw | 1.07E6 |  |  |  |  | 1.59E5 |  |  |  |  | 2 | 1 | 0 | 0 | 0 | 0 | 1 | 0 | 0 | 0 | 0 | 175 | 181 |  |
| total 3 peptides |  |  |  |  |  |  |  |  |  |  |  |  |  |  |  |  |  |  |  |  |  |  |  |  |  |  |  |  |  |  |  |  |  |  |  |
| --- | --- | --- | --- | --- | --- | --- | --- | --- | --- | --- | --- | --- | --- | --- | --- | --- | --- | --- | --- | --- | --- | --- | --- | --- | --- | --- | --- | --- | --- | --- | --- | --- | --- | --- | --- |

P58370|3NO48\_MICCO

back to list

  

| Protein Coverage
| Supporting Peptides
|

Protein Coverage:

Supporting Peptides:

| Peptide | Uniq | -10lgP | Mass | Length | ppm | m/z | z | RT | Fraction | Scan | Source File | Area F4 | Area F1 | Area F10 | Area F2 | Area F3 | Area F5 | Area F6 | Area F7 | Area F8 | Area F9 | #Feature | #Feature F4 | #Feature F1 | #Feature F10 | #Feature F2 | #Feature F3 | #Feature F5 | #Feature F6 | #Feature F7 | #Feature F8 | #Feature F9 | Start | End | PTM |
| --- | --- | --- | --- | --- | --- | --- | --- | --- | --- | --- | --- | --- | --- | --- | --- | --- | --- | --- | --- | --- | --- | --- | --- | --- | --- | --- | --- | --- | --- | --- | --- | --- | --- | --- | --- |
| R.IDRGC(+57.02)AATC(+57.02)PTVK.P | Y | 71.62 | 1447.6912 | 13 | -7.9 | 724.8472 | 2 | 27.23 | 6 | F6:9646 | 27122019\_RID\_1315\_NaNaWb\_F5.raw | 9.94E7 |  |  |  |  | 3.71E9 |  | 7.18E7 | 2.07E7 | 7.01E7 | 17 | 4 | 0 | 0 | 0 | 0 | 10 | 0 | 1 | 1 | 1 | 60 | 72 | Carbamidomethylation |
| R.IDRGC(+57.02)AATC(+57.02)PTV.K | Y | 47.08 | 1319.5962 | 12 | -7.6 | 660.8004 | 2 | 54.39 | 1 | F1:28308 | 09012020\_NaNaWb\_F4.raw | 1.7E7 |  |  |  |  |  |  |  |  |  | 1 | 1 | 0 | 0 | 0 | 0 | 0 | 0 | 0 | 0 | 0 | 60 | 71 | Carbamidomethylation |
| total 2 peptides |  |  |  |  |  |  |  |  |  |  |  |  |  |  |  |  |  |  |  |  |  |  |  |  |  |  |  |  |  |  |  |  |  |  |  |
| --- | --- | --- | --- | --- | --- | --- | --- | --- | --- | --- | --- | --- | --- | --- | --- | --- | --- | --- | --- | --- | --- | --- | --- | --- | --- | --- | --- | --- | --- | --- | --- | --- | --- | --- | --- |

P00761|TRYP\_PIG

back to list

  

| Protein Coverage
| Supporting Peptides
|

Protein Coverage:

Supporting Peptides:

| Peptide | Uniq | -10lgP | Mass | Length | ppm | m/z | z | RT | Fraction | Scan | Source File | Area F4 | Area F1 | Area F10 | Area F2 | Area F3 | Area F5 | Area F6 | Area F7 | Area F8 | Area F9 | #Feature | #Feature F4 | #Feature F1 | #Feature F10 | #Feature F2 | #Feature F3 | #Feature F5 | #Feature F6 | #Feature F7 | #Feature F8 | #Feature F9 | Start | End | PTM |
| --- | --- | --- | --- | --- | --- | --- | --- | --- | --- | --- | --- | --- | --- | --- | --- | --- | --- | --- | --- | --- | --- | --- | --- | --- | --- | --- | --- | --- | --- | --- | --- | --- | --- | --- | --- |
| R.LGEHNIDVLEGNEQFIN.A | Y | 49.20 | 1939.9275 | 17 | 0.7 | 970.9717 | 2 | 62.83 | 1 | F1:33830 | 09012020\_NaNaWb\_F4.raw | 9.25E6 |  |  |  |  |  |  |  |  |  | 1 | 1 | 0 | 0 | 0 | 0 | 0 | 0 | 0 | 0 | 0 | 58 | 74 |  |
| K.LSSPATLNSR.V | Y | 45.79 | 1044.5564 | 10 | 0.6 | 523.2858 | 2 | 22.88 | 1 | F1:7279 | 09012020\_NaNaWb\_F4.raw | 8.19E5 |  |  |  |  |  |  |  |  |  | 1 | 1 | 0 | 0 | 0 | 0 | 0 | 0 | 0 | 0 | 0 | 98 | 107 |  |
| K.IITHPNFN.G | Y | 43.66 | 954.4923 | 8 | 0.7 | 478.2538 | 2 | 30.30 | 1 | F1:11512 | 09012020\_NaNaWb\_F4.raw | 6.18E5 |  |  |  |  |  |  |  |  |  | 1 | 1 | 0 | 0 | 0 | 0 | 0 | 0 | 0 | 0 | 0 | 78 | 85 |  |
| total 3 peptides |  |  |  |  |  |  |  |  |  |  |  |  |  |  |  |  |  |  |  |  |  |  |  |  |  |  |  |  |  |  |  |  |  |  |  |
| --- | --- | --- | --- | --- | --- | --- | --- | --- | --- | --- | --- | --- | --- | --- | --- | --- | --- | --- | --- | --- | --- | --- | --- | --- | --- | --- | --- | --- | --- | --- | --- | --- | --- | --- | --- |

#CONTAM#TRYP\_PIG|

back to list

  

| Protein Coverage
| Supporting Peptides
|

Protein Coverage:

Supporting Peptides:

| Peptide | Uniq | -10lgP | Mass | Length | ppm | m/z | z | RT | Fraction | Scan | Source File | Area F4 | Area F1 | Area F10 | Area F2 | Area F3 | Area F5 | Area F6 | Area F7 | Area F8 | Area F9 | #Feature | #Feature F4 | #Feature F1 | #Feature F10 | #Feature F2 | #Feature F3 | #Feature F5 | #Feature F6 | #Feature F7 | #Feature F8 | #Feature F9 | Start | End | PTM |
| --- | --- | --- | --- | --- | --- | --- | --- | --- | --- | --- | --- | --- | --- | --- | --- | --- | --- | --- | --- | --- | --- | --- | --- | --- | --- | --- | --- | --- | --- | --- | --- | --- | --- | --- | --- |
| R.LGEHNIDVLEGNEQFIN.A | Y | 49.20 | 1939.9275 | 17 | 0.7 | 970.9717 | 2 | 62.83 | 1 | F1:33830 | 09012020\_NaNaWb\_F4.raw | 9.25E6 |  |  |  |  |  |  |  |  |  | 1 | 1 | 0 | 0 | 0 | 0 | 0 | 0 | 0 | 0 | 0 | 58 | 74 |  |
| K.LSSPATLNSR.V | Y | 45.79 | 1044.5564 | 10 | 0.6 | 523.2858 | 2 | 22.88 | 1 | F1:7279 | 09012020\_NaNaWb\_F4.raw | 8.19E5 |  |  |  |  |  |  |  |  |  | 1 | 1 | 0 | 0 | 0 | 0 | 0 | 0 | 0 | 0 | 0 | 98 | 107 |  |
| K.IITHPNFN.G | Y | 43.66 | 954.4923 | 8 | 0.7 | 478.2538 | 2 | 30.30 | 1 | F1:11512 | 09012020\_NaNaWb\_F4.raw | 6.18E5 |  |  |  |  |  |  |  |  |  | 1 | 1 | 0 | 0 | 0 | 0 | 0 | 0 | 0 | 0 | 0 | 78 | 85 |  |
| total 3 peptides |  |  |  |  |  |  |  |  |  |  |  |  |  |  |  |  |  |  |  |  |  |  |  |  |  |  |  |  |  |  |  |  |  |  |  |
| --- | --- | --- | --- | --- | --- | --- | --- | --- | --- | --- | --- | --- | --- | --- | --- | --- | --- | --- | --- | --- | --- | --- | --- | --- | --- | --- | --- | --- | --- | --- | --- | --- | --- | --- | --- |

D6PXE8|VM3B\_NAJAT

back to list

  

| Protein Coverage
| Supporting Peptides
|

Protein Coverage:

Supporting Peptides:

| Peptide | Uniq | -10lgP | Mass | Length | ppm | m/z | z | RT | Fraction | Scan | Source File | Area F4 | Area F1 | Area F10 | Area F2 | Area F3 | Area F5 | Area F6 | Area F7 | Area F8 | Area F9 | #Feature | #Feature F4 | #Feature F1 | #Feature F10 | #Feature F2 | #Feature F3 | #Feature F5 | #Feature F6 | #Feature F7 | #Feature F8 | #Feature F9 | Start | End | PTM |
| --- | --- | --- | --- | --- | --- | --- | --- | --- | --- | --- | --- | --- | --- | --- | --- | --- | --- | --- | --- | --- | --- | --- | --- | --- | --- | --- | --- | --- | --- | --- | --- | --- | --- | --- | --- |
| K.DDC(+57.02)DLPELC(+57.02)TGQSAEC(+57.02)PTDSLQR.N | Y | 77.84 | 2666.0894 | 23 | 0.8 | 889.7045 | 3 | 57.20 | 1 | F1:29990 | 09012020\_NaNaWb\_F4.raw | 1.55E7 |  |  |  |  |  | 0 |  |  |  | 3 | 2 | 0 | 0 | 0 | 0 | 0 | 1 | 0 | 0 | 0 | 454 | 476 | Carbamidomethylation |
| total 1 peptides |  |  |  |  |  |  |  |  |  |  |  |  |  |  |  |  |  |  |  |  |  |  |  |  |  |  |  |  |  |  |  |  |  |  |  |
| --- | --- | --- | --- | --- | --- | --- | --- | --- | --- | --- | --- | --- | --- | --- | --- | --- | --- | --- | --- | --- | --- | --- | --- | --- | --- | --- | --- | --- | --- | --- | --- | --- | --- | --- | --- |

D3TTC1|VM3KL\_NAJAT

back to list

  

| Protein Coverage
| Supporting Peptides
|

Protein Coverage:

Supporting Peptides:

| Peptide | Uniq | -10lgP | Mass | Length | ppm | m/z | z | RT | Fraction | Scan | Source File | Area F4 | Area F1 | Area F10 | Area F2 | Area F3 | Area F5 | Area F6 | Area F7 | Area F8 | Area F9 | #Feature | #Feature F4 | #Feature F1 | #Feature F10 | #Feature F2 | #Feature F3 | #Feature F5 | #Feature F6 | #Feature F7 | #Feature F8 | #Feature F9 | Start | End | PTM |
| --- | --- | --- | --- | --- | --- | --- | --- | --- | --- | --- | --- | --- | --- | --- | --- | --- | --- | --- | --- | --- | --- | --- | --- | --- | --- | --- | --- | --- | --- | --- | --- | --- | --- | --- | --- |
| K.DDC(+57.02)DLPELC(+57.02)TGQSAEC(+57.02)PTDSLQR.N | Y | 77.84 | 2666.0894 | 23 | 0.8 | 889.7045 | 3 | 57.20 | 1 | F1:29990 | 09012020\_NaNaWb\_F4.raw | 1.55E7 |  |  |  |  |  | 0 |  |  |  | 3 | 2 | 0 | 0 | 0 | 0 | 0 | 1 | 0 | 0 | 0 | 454 | 476 | Carbamidomethylation |
| total 1 peptides |  |  |  |  |  |  |  |  |  |  |  |  |  |  |  |  |  |  |  |  |  |  |  |  |  |  |  |  |  |  |  |  |  |  |  |
| --- | --- | --- | --- | --- | --- | --- | --- | --- | --- | --- | --- | --- | --- | --- | --- | --- | --- | --- | --- | --- | --- | --- | --- | --- | --- | --- | --- | --- | --- | --- | --- | --- | --- | --- | --- |

Q6IZ95|3NO24\_BUNCA

back to list

  

| Protein Coverage
| Supporting Peptides
|

Protein Coverage:

Supporting Peptides:

| Peptide | Uniq | -10lgP | Mass | Length | ppm | m/z | z | RT | Fraction | Scan | Source File | Area F4 | Area F1 | Area F10 | Area F2 | Area F3 | Area F5 | Area F6 | Area F7 | Area F8 | Area F9 | #Feature | #Feature F4 | #Feature F1 | #Feature F10 | #Feature F2 | #Feature F3 | #Feature F5 | #Feature F6 | #Feature F7 | #Feature F8 | #Feature F9 | Start | End | PTM |
| --- | --- | --- | --- | --- | --- | --- | --- | --- | --- | --- | --- | --- | --- | --- | --- | --- | --- | --- | --- | --- | --- | --- | --- | --- | --- | --- | --- | --- | --- | --- | --- | --- | --- | --- | --- |
| H.EIVEC(+57.02)C(+57.02)STDK.C | Y | 72.66 | 1239.5111 | 10 | 0.6 | 620.7632 | 2 | 12.27 | 10 | F10:2076 | 27122019\_RID\_1315\_NaNaWb\_F9.raw |  |  |  |  |  |  |  |  |  | 2.11E7 | 1 | 0 | 0 | 0 | 0 | 0 | 0 | 0 | 0 | 0 | 1 | 74 | 83 | Carbamidomethylation |
| T.LTC(+57.02)LIC(+57.02)PEK.Y | N | 60.73 | 1132.5620 | 9 | -0.1 | 567.2882 | 2 | 37.35 | 1 | F1:16059 | 09012020\_NaNaWb\_F4.raw | 1.64E7 |  |  |  |  | 3.6E6 |  |  |  | 2.57E8 | 5 | 3 | 0 | 0 | 0 | 0 | 1 | 0 | 0 | 0 | 1 | 22 | 30 | Carbamidomethylation |
| total 2 peptides |  |  |  |  |  |  |  |  |  |  |  |  |  |  |  |  |  |  |  |  |  |  |  |  |  |  |  |  |  |  |  |  |  |  |  |
| --- | --- | --- | --- | --- | --- | --- | --- | --- | --- | --- | --- | --- | --- | --- | --- | --- | --- | --- | --- | --- | --- | --- | --- | --- | --- | --- | --- | --- | --- | --- | --- | --- | --- | --- | --- |

A2CKF7|3NO2\_BUNFA

back to list

  

| Protein Coverage
| Supporting Peptides
|

Protein Coverage:

Supporting Peptides:

| Peptide | Uniq | -10lgP | Mass | Length | ppm | m/z | z | RT | Fraction | Scan | Source File | Area F4 | Area F1 | Area F10 | Area F2 | Area F3 | Area F5 | Area F6 | Area F7 | Area F8 | Area F9 | #Feature | #Feature F4 | #Feature F1 | #Feature F10 | #Feature F2 | #Feature F3 | #Feature F5 | #Feature F6 | #Feature F7 | #Feature F8 | #Feature F9 | Start | End | PTM |
| --- | --- | --- | --- | --- | --- | --- | --- | --- | --- | --- | --- | --- | --- | --- | --- | --- | --- | --- | --- | --- | --- | --- | --- | --- | --- | --- | --- | --- | --- | --- | --- | --- | --- | --- | --- |
| H.EIVEC(+57.02)C(+57.02)STDK.C | Y | 72.66 | 1239.5111 | 10 | 0.6 | 620.7632 | 2 | 12.27 | 10 | F10:2076 | 27122019\_RID\_1315\_NaNaWb\_F9.raw |  |  |  |  |  |  |  |  |  | 2.11E7 | 1 | 0 | 0 | 0 | 0 | 0 | 0 | 0 | 0 | 0 | 1 | 74 | 83 | Carbamidomethylation |
| T.LTC(+57.02)LIC(+57.02)PEK.Y | N | 60.73 | 1132.5620 | 9 | -0.1 | 567.2882 | 2 | 37.35 | 1 | F1:16059 | 09012020\_NaNaWb\_F4.raw | 1.64E7 |  |  |  |  | 3.6E6 |  |  |  | 2.57E8 | 5 | 3 | 0 | 0 | 0 | 0 | 1 | 0 | 0 | 0 | 1 | 22 | 30 | Carbamidomethylation |
| total 2 peptides |  |  |  |  |  |  |  |  |  |  |  |  |  |  |  |  |  |  |  |  |  |  |  |  |  |  |  |  |  |  |  |  |  |  |  |
| --- | --- | --- | --- | --- | --- | --- | --- | --- | --- | --- | --- | --- | --- | --- | --- | --- | --- | --- | --- | --- | --- | --- | --- | --- | --- | --- | --- | --- | --- | --- | --- | --- | --- | --- | --- |

O93422|3NO2H\_NAJAT

back to list

  

| Protein Coverage
| Supporting Peptides
|

Protein Coverage:

Supporting Peptides:

| Peptide | Uniq | -10lgP | Mass | Length | ppm | m/z | z | RT | Fraction | Scan | Source File | Area F4 | Area F1 | Area F10 | Area F2 | Area F3 | Area F5 | Area F6 | Area F7 | Area F8 | Area F9 | #Feature | #Feature F4 | #Feature F1 | #Feature F10 | #Feature F2 | #Feature F3 | #Feature F5 | #Feature F6 | #Feature F7 | #Feature F8 | #Feature F9 | Start | End | PTM |
| --- | --- | --- | --- | --- | --- | --- | --- | --- | --- | --- | --- | --- | --- | --- | --- | --- | --- | --- | --- | --- | --- | --- | --- | --- | --- | --- | --- | --- | --- | --- | --- | --- | --- | --- | --- |
| R.EIVEC(+57.02)C(+57.02)STDK.C | Y | 72.66 | 1239.5111 | 10 | 0.6 | 620.7632 | 2 | 12.27 | 10 | F10:2076 | 27122019\_RID\_1315\_NaNaWb\_F9.raw |  |  |  |  |  |  |  |  |  | 2.11E7 | 1 | 0 | 0 | 0 | 0 | 0 | 0 | 0 | 0 | 0 | 1 | 74 | 83 | Carbamidomethylation |
| T.LTC(+57.02)LIC(+57.02)PEK.Y | N | 60.73 | 1132.5620 | 9 | -0.1 | 567.2882 | 2 | 37.35 | 1 | F1:16059 | 09012020\_NaNaWb\_F4.raw | 1.64E7 |  |  |  |  | 3.6E6 |  |  |  | 2.57E8 | 5 | 3 | 0 | 0 | 0 | 0 | 1 | 0 | 0 | 0 | 1 | 22 | 30 | Carbamidomethylation |
| total 2 peptides |  |  |  |  |  |  |  |  |  |  |  |  |  |  |  |  |  |  |  |  |  |  |  |  |  |  |  |  |  |  |  |  |  |  |  |
| --- | --- | --- | --- | --- | --- | --- | --- | --- | --- | --- | --- | --- | --- | --- | --- | --- | --- | --- | --- | --- | --- | --- | --- | --- | --- | --- | --- | --- | --- | --- | --- | --- | --- | --- | --- |

Q9YGI2|3NO21\_NAJAT

back to list

  

| Protein Coverage
| Supporting Peptides
|

Protein Coverage:

Supporting Peptides:

| Peptide | Uniq | -10lgP | Mass | Length | ppm | m/z | z | RT | Fraction | Scan | Source File | Area F4 | Area F1 | Area F10 | Area F2 | Area F3 | Area F5 | Area F6 | Area F7 | Area F8 | Area F9 | #Feature | #Feature F4 | #Feature F1 | #Feature F10 | #Feature F2 | #Feature F3 | #Feature F5 | #Feature F6 | #Feature F7 | #Feature F8 | #Feature F9 | Start | End | PTM |
| --- | --- | --- | --- | --- | --- | --- | --- | --- | --- | --- | --- | --- | --- | --- | --- | --- | --- | --- | --- | --- | --- | --- | --- | --- | --- | --- | --- | --- | --- | --- | --- | --- | --- | --- | --- |
| R.EIVEC(+57.02)C(+57.02)STDK.C | Y | 72.66 | 1239.5111 | 10 | 0.6 | 620.7632 | 2 | 12.27 | 10 | F10:2076 | 27122019\_RID\_1315\_NaNaWb\_F9.raw |  |  |  |  |  |  |  |  |  | 2.11E7 | 1 | 0 | 0 | 0 | 0 | 0 | 0 | 0 | 0 | 0 | 1 | 74 | 83 | Carbamidomethylation |
| T.LTC(+57.02)LIC(+57.02)PEK.Y | N | 60.73 | 1132.5620 | 9 | -0.1 | 567.2882 | 2 | 37.35 | 1 | F1:16059 | 09012020\_NaNaWb\_F4.raw | 1.64E7 |  |  |  |  | 3.6E6 |  |  |  | 2.57E8 | 5 | 3 | 0 | 0 | 0 | 0 | 1 | 0 | 0 | 0 | 1 | 22 | 30 | Carbamidomethylation |
| total 2 peptides |  |  |  |  |  |  |  |  |  |  |  |  |  |  |  |  |  |  |  |  |  |  |  |  |  |  |  |  |  |  |  |  |  |  |  |
| --- | --- | --- | --- | --- | --- | --- | --- | --- | --- | --- | --- | --- | --- | --- | --- | --- | --- | --- | --- | --- | --- | --- | --- | --- | --- | --- | --- | --- | --- | --- | --- | --- | --- | --- | --- |

P01400|3NO2B\_NAJME

back to list

  

| Protein Coverage
| Supporting Peptides
|

Protein Coverage:

Supporting Peptides:

| Peptide | Uniq | -10lgP | Mass | Length | ppm | m/z | z | RT | Fraction | Scan | Source File | Area F4 | Area F1 | Area F10 | Area F2 | Area F3 | Area F5 | Area F6 | Area F7 | Area F8 | Area F9 | #Feature | #Feature F4 | #Feature F1 | #Feature F10 | #Feature F2 | #Feature F3 | #Feature F5 | #Feature F6 | #Feature F7 | #Feature F8 | #Feature F9 | Start | End | PTM |
| --- | --- | --- | --- | --- | --- | --- | --- | --- | --- | --- | --- | --- | --- | --- | --- | --- | --- | --- | --- | --- | --- | --- | --- | --- | --- | --- | --- | --- | --- | --- | --- | --- | --- | --- | --- |
| R.EIVEC(+57.02)C(+57.02)STDK.C | Y | 72.66 | 1239.5111 | 10 | 0.6 | 620.7632 | 2 | 12.27 | 10 | F10:2076 | 27122019\_RID\_1315\_NaNaWb\_F9.raw |  |  |  |  |  |  |  |  |  | 2.11E7 | 1 | 0 | 0 | 0 | 0 | 0 | 0 | 0 | 0 | 0 | 1 | 53 | 62 | Carbamidomethylation |
| LTC(+57.02)LIC(+57.02)PEK.Y | N | 60.73 | 1132.5620 | 9 | -0.1 | 567.2882 | 2 | 37.35 | 1 | F1:16059 | 09012020\_NaNaWb\_F4.raw | 1.64E7 |  |  |  |  | 3.6E6 |  |  |  | 2.57E8 | 5 | 3 | 0 | 0 | 0 | 0 | 1 | 0 | 0 | 0 | 1 | 1 | 9 | Carbamidomethylation |
| total 2 peptides |  |  |  |  |  |  |  |  |  |  |  |  |  |  |  |  |  |  |  |  |  |  |  |  |  |  |  |  |  |  |  |  |  |  |  |
| --- | --- | --- | --- | --- | --- | --- | --- | --- | --- | --- | --- | --- | --- | --- | --- | --- | --- | --- | --- | --- | --- | --- | --- | --- | --- | --- | --- | --- | --- | --- | --- | --- | --- | --- | --- |

P85520|3NO2\_NAJOX

back to list

  

| Protein Coverage
| Supporting Peptides
|

Protein Coverage:

Supporting Peptides:

| Peptide | Uniq | -10lgP | Mass | Length | ppm | m/z | z | RT | Fraction | Scan | Source File | Area F4 | Area F1 | Area F10 | Area F2 | Area F3 | Area F5 | Area F6 | Area F7 | Area F8 | Area F9 | #Feature | #Feature F4 | #Feature F1 | #Feature F10 | #Feature F2 | #Feature F3 | #Feature F5 | #Feature F6 | #Feature F7 | #Feature F8 | #Feature F9 | Start | End | PTM |
| --- | --- | --- | --- | --- | --- | --- | --- | --- | --- | --- | --- | --- | --- | --- | --- | --- | --- | --- | --- | --- | --- | --- | --- | --- | --- | --- | --- | --- | --- | --- | --- | --- | --- | --- | --- |
| R.EIVEC(+57.02)C(+57.02)STDK.C | Y | 72.66 | 1239.5111 | 10 | 0.6 | 620.7632 | 2 | 12.27 | 10 | F10:2076 | 27122019\_RID\_1315\_NaNaWb\_F9.raw |  |  |  |  |  |  |  |  |  | 2.11E7 | 1 | 0 | 0 | 0 | 0 | 0 | 0 | 0 | 0 | 0 | 1 | 53 | 62 | Carbamidomethylation |
| LTC(+57.02)LIC(+57.02)PEK.Y | N | 60.73 | 1132.5620 | 9 | -0.1 | 567.2882 | 2 | 37.35 | 1 | F1:16059 | 09012020\_NaNaWb\_F4.raw | 1.64E7 |  |  |  |  | 3.6E6 |  |  |  | 2.57E8 | 5 | 3 | 0 | 0 | 0 | 0 | 1 | 0 | 0 | 0 | 1 | 1 | 9 | Carbamidomethylation |
| total 2 peptides |  |  |  |  |  |  |  |  |  |  |  |  |  |  |  |  |  |  |  |  |  |  |  |  |  |  |  |  |  |  |  |  |  |  |  |
| --- | --- | --- | --- | --- | --- | --- | --- | --- | --- | --- | --- | --- | --- | --- | --- | --- | --- | --- | --- | --- | --- | --- | --- | --- | --- | --- | --- | --- | --- | --- | --- | --- | --- | --- | --- |

Q9W717|3SO1\_NAJAT

back to list

  

| Protein Coverage
| Supporting Peptides
|

Protein Coverage:

Supporting Peptides:

| Peptide | Uniq | -10lgP | Mass | Length | ppm | m/z | z | RT | Fraction | Scan | Source File | Area F4 | Area F1 | Area F10 | Area F2 | Area F3 | Area F5 | Area F6 | Area F7 | Area F8 | Area F9 | #Feature | #Feature F4 | #Feature F1 | #Feature F10 | #Feature F2 | #Feature F3 | #Feature F5 | #Feature F6 | #Feature F7 | #Feature F8 | #Feature F9 | Start | End | PTM |
| --- | --- | --- | --- | --- | --- | --- | --- | --- | --- | --- | --- | --- | --- | --- | --- | --- | --- | --- | --- | --- | --- | --- | --- | --- | --- | --- | --- | --- | --- | --- | --- | --- | --- | --- | --- |
| R.VYVDC(+57.02)C(+57.02)AR.D | Y | 71.13 | 1041.4371 | 8 | -0.3 | 521.7257 | 2 | 12.34 | 8 | F8:2115 | 27122019\_RID\_1315\_NaNaWb\_F7.raw |  |  |  |  |  |  | 4.43E7 | 2.4E7 |  |  | 2 | 0 | 0 | 0 | 0 | 0 | 0 | 1 | 1 | 0 | 0 | 74 | 81 | Carbamidomethylation |
| Y.SIFSETIEIC(+57.02)PDGHNFC(+57.02)FK.K | Y | 58.29 | 2300.0242 | 19 | 0.0 | 767.6820 | 3 | 53.02 | 7 | F7:18885 | 27122019\_RID\_1315\_NaNaWb\_F6.raw |  |  |  |  |  |  | 5.26E6 |  |  |  | 2 | 0 | 0 | 0 | 0 | 0 | 0 | 2 | 0 | 0 | 0 | 29 | 47 | Carbamidomethylation |
| total 2 peptides |  |  |  |  |  |  |  |  |  |  |  |  |  |  |  |  |  |  |  |  |  |  |  |  |  |  |  |  |  |  |  |  |  |  |  |
| --- | --- | --- | --- | --- | --- | --- | --- | --- | --- | --- | --- | --- | --- | --- | --- | --- | --- | --- | --- | --- | --- | --- | --- | --- | --- | --- | --- | --- | --- | --- | --- | --- | --- | --- | --- |

Q91132|VCO3\_NAJKA

back to list

  

| Protein Coverage
| Supporting Peptides
|

Protein Coverage:

Supporting Peptides:

| Peptide | Uniq | -10lgP | Mass | Length | ppm | m/z | z | RT | Fraction | Scan | Source File | Area F4 | Area F1 | Area F10 | Area F2 | Area F3 | Area F5 | Area F6 | Area F7 | Area F8 | Area F9 | #Feature | #Feature F4 | #Feature F1 | #Feature F10 | #Feature F2 | #Feature F3 | #Feature F5 | #Feature F6 | #Feature F7 | #Feature F8 | #Feature F9 | Start | End | PTM |
| --- | --- | --- | --- | --- | --- | --- | --- | --- | --- | --- | --- | --- | --- | --- | --- | --- | --- | --- | --- | --- | --- | --- | --- | --- | --- | --- | --- | --- | --- | --- | --- | --- | --- | --- | --- |
| K.IIIQGDPVAQIIENSIDGSK.L | Y | 66.28 | 2109.1316 | 20 | 0.6 | 1055.5737 | 2 | 69.34 | 8 | F8:16709 | 27122019\_RID\_1315\_NaNaWb\_F7.raw |  |  |  |  |  |  |  | 1.76E5 |  |  | 1 | 0 | 0 | 0 | 0 | 0 | 0 | 0 | 1 | 0 | 0 | 963 | 982 |  |
| K.AAFLEC(+57.02)C(+57.02)R.Y | N | 57.41 | 1025.4423 | 8 | 0.6 | 513.7287 | 2 | 21.87 | 6 | F6:6299 | 27122019\_RID\_1315\_NaNaWb\_F5.raw |  |  |  |  |  | 2.78E6 |  | 0 |  |  | 2 | 0 | 0 | 0 | 0 | 0 | 1 | 0 | 1 | 0 | 0 | 706 | 713 | Carbamidomethylation |
| total 2 peptides |  |  |  |  |  |  |  |  |  |  |  |  |  |  |  |  |  |  |  |  |  |  |  |  |  |  |  |  |  |  |  |  |  |  |  |
| --- | --- | --- | --- | --- | --- | --- | --- | --- | --- | --- | --- | --- | --- | --- | --- | --- | --- | --- | --- | --- | --- | --- | --- | --- | --- | --- | --- | --- | --- | --- | --- | --- | --- | --- | --- |

P82942|VM3K\_NAJKA

back to list

  

| Protein Coverage
| Supporting Peptides
|

Protein Coverage:

Supporting Peptides:

| Peptide | Uniq | -10lgP | Mass | Length | ppm | m/z | z | RT | Fraction | Scan | Source File | Area F4 | Area F1 | Area F10 | Area F2 | Area F3 | Area F5 | Area F6 | Area F7 | Area F8 | Area F9 | #Feature | #Feature F4 | #Feature F1 | #Feature F10 | #Feature F2 | #Feature F3 | #Feature F5 | #Feature F6 | #Feature F7 | #Feature F8 | #Feature F9 | Start | End | PTM |
| --- | --- | --- | --- | --- | --- | --- | --- | --- | --- | --- | --- | --- | --- | --- | --- | --- | --- | --- | --- | --- | --- | --- | --- | --- | --- | --- | --- | --- | --- | --- | --- | --- | --- | --- | --- |
| K.HDC(+57.02)DLPELC(+57.02)TGQSAEC(+57.02)PTDSLQR.N | Y | 46.95 | 2688.1213 | 23 | 0.0 | 897.0477 | 3 | 34.06 | 7 | F7:11382 | 27122019\_RID\_1315\_NaNaWb\_F6.raw |  |  |  |  |  |  | 2.12E6 |  |  |  | 1 | 0 | 0 | 0 | 0 | 0 | 0 | 1 | 0 | 0 | 0 | 262 | 284 | Carbamidomethylation |
| K.C(+57.02)PTLTNQC(+57.02)IALLGPHFTVSPK.G | Y | 42.10 | 2353.1921 | 21 | -1.4 | 785.4036 | 3 | 47.33 | 9 | F9:15784 | 27122019\_RID\_1315\_NaNaWb\_F8.raw |  |  |  |  |  |  |  |  | 5.52E5 |  | 1 | 0 | 0 | 0 | 0 | 0 | 0 | 0 | 0 | 1 | 0 | 301 | 321 | Carbamidomethylation |
| total 2 peptides |  |  |  |  |  |  |  |  |  |  |  |  |  |  |  |  |  |  |  |  |  |  |  |  |  |  |  |  |  |  |  |  |  |  |  |
| --- | --- | --- | --- | --- | --- | --- | --- | --- | --- | --- | --- | --- | --- | --- | --- | --- | --- | --- | --- | --- | --- | --- | --- | --- | --- | --- | --- | --- | --- | --- | --- | --- | --- | --- | --- |

P81605|DCD\_HUMAN

back to list

  

| Protein Coverage
| Supporting Peptides
|

Protein Coverage:

Supporting Peptides:

| Peptide | Uniq | -10lgP | Mass | Length | ppm | m/z | z | RT | Fraction | Scan | Source File | Area F4 | Area F1 | Area F10 | Area F2 | Area F3 | Area F5 | Area F6 | Area F7 | Area F8 | Area F9 | #Feature | #Feature F4 | #Feature F1 | #Feature F10 | #Feature F2 | #Feature F3 | #Feature F5 | #Feature F6 | #Feature F7 | #Feature F8 | #Feature F9 | Start | End | PTM |
| --- | --- | --- | --- | --- | --- | --- | --- | --- | --- | --- | --- | --- | --- | --- | --- | --- | --- | --- | --- | --- | --- | --- | --- | --- | --- | --- | --- | --- | --- | --- | --- | --- | --- | --- | --- |
| K.ENAGEDPGLAR.Q | Y | 46.25 | 1127.5206 | 11 | 0.6 | 564.7679 | 2 | 12.54 | 7 | F7:2262 | 27122019\_RID\_1315\_NaNaWb\_F6.raw |  |  |  |  |  |  | 5.6E5 |  |  |  | 1 | 0 | 0 | 0 | 0 | 0 | 0 | 1 | 0 | 0 | 0 | 43 | 53 |  |
| total 1 peptides |  |  |  |  |  |  |  |  |  |  |  |  |  |  |  |  |  |  |  |  |  |  |  |  |  |  |  |  |  |  |  |  |  |  |  |
| --- | --- | --- | --- | --- | --- | --- | --- | --- | --- | --- | --- | --- | --- | --- | --- | --- | --- | --- | --- | --- | --- | --- | --- | --- | --- | --- | --- | --- | --- | --- | --- | --- | --- | --- | --- |

Q7LZI1|PLIB\_NAJKA

back to list

  

| Protein Coverage
| Supporting Peptides
|

Protein Coverage:

Supporting Peptides:

| Peptide | Uniq | -10lgP | Mass | Length | ppm | m/z | z | RT | Fraction | Scan | Source File | Area F4 | Area F1 | Area F10 | Area F2 | Area F3 | Area F5 | Area F6 | Area F7 | Area F8 | Area F9 | #Feature | #Feature F4 | #Feature F1 | #Feature F10 | #Feature F2 | #Feature F3 | #Feature F5 | #Feature F6 | #Feature F7 | #Feature F8 | #Feature F9 | Start | End | PTM |
| --- | --- | --- | --- | --- | --- | --- | --- | --- | --- | --- | --- | --- | --- | --- | --- | --- | --- | --- | --- | --- | --- | --- | --- | --- | --- | --- | --- | --- | --- | --- | --- | --- | --- | --- | --- |
| K.LGSFDVNVGQNTYVR.G | Y | 73.89 | 1667.8267 | 15 | -0.1 | 834.9205 | 2 | 40.97 | 8 | F8:9999 | 27122019\_RID\_1315\_NaNaWb\_F7.raw |  |  |  |  |  |  |  | 4.19E5 |  |  | 1 | 0 | 0 | 0 | 0 | 0 | 0 | 0 | 1 | 0 | 0 | 56 | 70 |  |
| total 1 peptides |  |  |  |  |  |  |  |  |  |  |  |  |  |  |  |  |  |  |  |  |  |  |  |  |  |  |  |  |  |  |  |  |  |  |  |
| --- | --- | --- | --- | --- | --- | --- | --- | --- | --- | --- | --- | --- | --- | --- | --- | --- | --- | --- | --- | --- | --- | --- | --- | --- | --- | --- | --- | --- | --- | --- | --- | --- | --- | --- | --- |

Q9PSN6|3S13\_NAJSP

back to list

  

| Protein Coverage
| Supporting Peptides
|

Protein Coverage:

Supporting Peptides:

| Peptide | Uniq | -10lgP | Mass | Length | ppm | m/z | z | RT | Fraction | Scan | Source File | Area F4 | Area F1 | Area F10 | Area F2 | Area F3 | Area F5 | Area F6 | Area F7 | Area F8 | Area F9 | #Feature | #Feature F4 | #Feature F1 | #Feature F10 | #Feature F2 | #Feature F3 | #Feature F5 | #Feature F6 | #Feature F7 | #Feature F8 | #Feature F9 | Start | End | PTM |
| --- | --- | --- | --- | --- | --- | --- | --- | --- | --- | --- | --- | --- | --- | --- | --- | --- | --- | --- | --- | --- | --- | --- | --- | --- | --- | --- | --- | --- | --- | --- | --- | --- | --- | --- | --- |
| K.NGIEINC(+57.02)C(+57.02)TTDR.C | Y | 71.28 | 1451.6133 | 12 | 0.2 | 726.8141 | 2 | 28.67 | 1 | F1:10507 | 09012020\_NaNaWb\_F4.raw | 9.81E5 |  |  | 1.15E7 |  | 3.21E5 |  |  |  |  | 3 | 1 | 0 | 0 | 1 | 0 | 1 | 0 | 0 | 0 | 0 | 48 | 59 | Carbamidomethylation |
| total 1 peptides |  |  |  |  |  |  |  |  |  |  |  |  |  |  |  |  |  |  |  |  |  |  |  |  |  |  |  |  |  |  |  |  |  |  |  |
| --- | --- | --- | --- | --- | --- | --- | --- | --- | --- | --- | --- | --- | --- | --- | --- | --- | --- | --- | --- | --- | --- | --- | --- | --- | --- | --- | --- | --- | --- | --- | --- | --- | --- | --- | --- |

P14613|3S1B1\_NAJKA

back to list

  

| Protein Coverage
| Supporting Peptides
|

Protein Coverage:

Supporting Peptides:

| Peptide | Uniq | -10lgP | Mass | Length | ppm | m/z | z | RT | Fraction | Scan | Source File | Area F4 | Area F1 | Area F10 | Area F2 | Area F3 | Area F5 | Area F6 | Area F7 | Area F8 | Area F9 | #Feature | #Feature F4 | #Feature F1 | #Feature F10 | #Feature F2 | #Feature F3 | #Feature F5 | #Feature F6 | #Feature F7 | #Feature F8 | #Feature F9 | Start | End | PTM |
| --- | --- | --- | --- | --- | --- | --- | --- | --- | --- | --- | --- | --- | --- | --- | --- | --- | --- | --- | --- | --- | --- | --- | --- | --- | --- | --- | --- | --- | --- | --- | --- | --- | --- | --- | --- |
| K.NGIEINC(+57.02)C(+57.02)TTDR.C | Y | 71.28 | 1451.6133 | 12 | 0.2 | 726.8141 | 2 | 28.67 | 1 | F1:10507 | 09012020\_NaNaWb\_F4.raw | 9.81E5 |  |  | 1.15E7 |  | 3.21E5 |  |  |  |  | 3 | 1 | 0 | 0 | 1 | 0 | 1 | 0 | 0 | 0 | 0 | 48 | 59 | Carbamidomethylation |
| total 1 peptides |  |  |  |  |  |  |  |  |  |  |  |  |  |  |  |  |  |  |  |  |  |  |  |  |  |  |  |  |  |  |  |  |  |  |  |
| --- | --- | --- | --- | --- | --- | --- | --- | --- | --- | --- | --- | --- | --- | --- | --- | --- | --- | --- | --- | --- | --- | --- | --- | --- | --- | --- | --- | --- | --- | --- | --- | --- | --- | --- | --- |

P60770|3S1CB\_NAJAT

back to list

  

| Protein Coverage
| Supporting Peptides
|

Protein Coverage:

Supporting Peptides:

| Peptide | Uniq | -10lgP | Mass | Length | ppm | m/z | z | RT | Fraction | Scan | Source File | Area F4 | Area F1 | Area F10 | Area F2 | Area F3 | Area F5 | Area F6 | Area F7 | Area F8 | Area F9 | #Feature | #Feature F4 | #Feature F1 | #Feature F10 | #Feature F2 | #Feature F3 | #Feature F5 | #Feature F6 | #Feature F7 | #Feature F8 | #Feature F9 | Start | End | PTM |
| --- | --- | --- | --- | --- | --- | --- | --- | --- | --- | --- | --- | --- | --- | --- | --- | --- | --- | --- | --- | --- | --- | --- | --- | --- | --- | --- | --- | --- | --- | --- | --- | --- | --- | --- | --- |
| K.NGIEINC(+57.02)C(+57.02)TTDR.C | Y | 71.28 | 1451.6133 | 12 | 0.2 | 726.8141 | 2 | 28.67 | 1 | F1:10507 | 09012020\_NaNaWb\_F4.raw | 9.81E5 |  |  | 1.15E7 |  | 3.21E5 |  |  |  |  | 3 | 1 | 0 | 0 | 1 | 0 | 1 | 0 | 0 | 0 | 0 | 69 | 80 | Carbamidomethylation |
| total 1 peptides |  |  |  |  |  |  |  |  |  |  |  |  |  |  |  |  |  |  |  |  |  |  |  |  |  |  |  |  |  |  |  |  |  |  |  |
| --- | --- | --- | --- | --- | --- | --- | --- | --- | --- | --- | --- | --- | --- | --- | --- | --- | --- | --- | --- | --- | --- | --- | --- | --- | --- | --- | --- | --- | --- | --- | --- | --- | --- | --- | --- |

P60771|3S1CB\_NAJKA

back to list

  

| Protein Coverage
| Supporting Peptides
|

Protein Coverage:

Supporting Peptides:

| Peptide | Uniq | -10lgP | Mass | Length | ppm | m/z | z | RT | Fraction | Scan | Source File | Area F4 | Area F1 | Area F10 | Area F2 | Area F3 | Area F5 | Area F6 | Area F7 | Area F8 | Area F9 | #Feature | #Feature F4 | #Feature F1 | #Feature F10 | #Feature F2 | #Feature F3 | #Feature F5 | #Feature F6 | #Feature F7 | #Feature F8 | #Feature F9 | Start | End | PTM |
| --- | --- | --- | --- | --- | --- | --- | --- | --- | --- | --- | --- | --- | --- | --- | --- | --- | --- | --- | --- | --- | --- | --- | --- | --- | --- | --- | --- | --- | --- | --- | --- | --- | --- | --- | --- |
| K.NGIEINC(+57.02)C(+57.02)TTDR.C | Y | 71.28 | 1451.6133 | 12 | 0.2 | 726.8141 | 2 | 28.67 | 1 | F1:10507 | 09012020\_NaNaWb\_F4.raw | 9.81E5 |  |  | 1.15E7 |  | 3.21E5 |  |  |  |  | 3 | 1 | 0 | 0 | 1 | 0 | 1 | 0 | 0 | 0 | 0 | 69 | 80 | Carbamidomethylation |
| total 1 peptides |  |  |  |  |  |  |  |  |  |  |  |  |  |  |  |  |  |  |  |  |  |  |  |  |  |  |  |  |  |  |  |  |  |  |  |
| --- | --- | --- | --- | --- | --- | --- | --- | --- | --- | --- | --- | --- | --- | --- | --- | --- | --- | --- | --- | --- | --- | --- | --- | --- | --- | --- | --- | --- | --- | --- | --- | --- | --- | --- | --- |

Q9PTT0|3S1CB\_NAJNA

back to list

  

| Protein Coverage
| Supporting Peptides
|

Protein Coverage:

Supporting Peptides:

| Peptide | Uniq | -10lgP | Mass | Length | ppm | m/z | z | RT | Fraction | Scan | Source File | Area F4 | Area F1 | Area F10 | Area F2 | Area F3 | Area F5 | Area F6 | Area F7 | Area F8 | Area F9 | #Feature | #Feature F4 | #Feature F1 | #Feature F10 | #Feature F2 | #Feature F3 | #Feature F5 | #Feature F6 | #Feature F7 | #Feature F8 | #Feature F9 | Start | End | PTM |
| --- | --- | --- | --- | --- | --- | --- | --- | --- | --- | --- | --- | --- | --- | --- | --- | --- | --- | --- | --- | --- | --- | --- | --- | --- | --- | --- | --- | --- | --- | --- | --- | --- | --- | --- | --- |
| K.NGIEINC(+57.02)C(+57.02)TTDR.C | Y | 71.28 | 1451.6133 | 12 | 0.2 | 726.8141 | 2 | 28.67 | 1 | F1:10507 | 09012020\_NaNaWb\_F4.raw | 9.81E5 |  |  | 1.15E7 |  | 3.21E5 |  |  |  |  | 3 | 1 | 0 | 0 | 1 | 0 | 1 | 0 | 0 | 0 | 0 | 69 | 80 | Carbamidomethylation |
| total 1 peptides |  |  |  |  |  |  |  |  |  |  |  |  |  |  |  |  |  |  |  |  |  |  |  |  |  |  |  |  |  |  |  |  |  |  |  |
| --- | --- | --- | --- | --- | --- | --- | --- | --- | --- | --- | --- | --- | --- | --- | --- | --- | --- | --- | --- | --- | --- | --- | --- | --- | --- | --- | --- | --- | --- | --- | --- | --- | --- | --- | --- |

Q9PVK7|VM3\_NAJKA

back to list

  

| Protein Coverage
| Supporting Peptides
|

Protein Coverage:

Supporting Peptides:

| Peptide | Uniq | -10lgP | Mass | Length | ppm | m/z | z | RT | Fraction | Scan | Source File | Area F4 | Area F1 | Area F10 | Area F2 | Area F3 | Area F5 | Area F6 | Area F7 | Area F8 | Area F9 | #Feature | #Feature F4 | #Feature F1 | #Feature F10 | #Feature F2 | #Feature F3 | #Feature F5 | #Feature F6 | #Feature F7 | #Feature F8 | #Feature F9 | Start | End | PTM |
| --- | --- | --- | --- | --- | --- | --- | --- | --- | --- | --- | --- | --- | --- | --- | --- | --- | --- | --- | --- | --- | --- | --- | --- | --- | --- | --- | --- | --- | --- | --- | --- | --- | --- | --- | --- |
| K.TSAAVVQDYSK.S | Y | 65.09 | 1167.5771 | 11 | 0.3 | 584.7960 | 2 | 16.50 | 8 | F8:3042 | 27122019\_RID\_1315\_NaNaWb\_F7.raw |  |  |  |  |  |  | 1.45E6 | 5.08E5 |  |  | 2 | 0 | 0 | 0 | 0 | 0 | 0 | 1 | 1 | 0 | 0 | 308 | 318 |  |
| total 1 peptides |  |  |  |  |  |  |  |  |  |  |  |  |  |  |  |  |  |  |  |  |  |  |  |  |  |  |  |  |  |  |  |  |  |  |  |
| --- | --- | --- | --- | --- | --- | --- | --- | --- | --- | --- | --- | --- | --- | --- | --- | --- | --- | --- | --- | --- | --- | --- | --- | --- | --- | --- | --- | --- | --- | --- | --- | --- | --- | --- | --- |

F8RKW1|VM34\_DRYCN

back to list

  

| Protein Coverage
| Supporting Peptides
|

Protein Coverage:

Supporting Peptides:

| Peptide | Uniq | -10lgP | Mass | Length | ppm | m/z | z | RT | Fraction | Scan | Source File | Area F4 | Area F1 | Area F10 | Area F2 | Area F3 | Area F5 | Area F6 | Area F7 | Area F8 | Area F9 | #Feature | #Feature F4 | #Feature F1 | #Feature F10 | #Feature F2 | #Feature F3 | #Feature F5 | #Feature F6 | #Feature F7 | #Feature F8 | #Feature F9 | Start | End | PTM |
| --- | --- | --- | --- | --- | --- | --- | --- | --- | --- | --- | --- | --- | --- | --- | --- | --- | --- | --- | --- | --- | --- | --- | --- | --- | --- | --- | --- | --- | --- | --- | --- | --- | --- | --- | --- |
| R.NGHPC(+57.02)QNNEGYC(+57.02)YNGK.C | Y | 62.07 | 1910.7424 | 16 | 1.1 | 956.3795 | 2 | 12.47 | 1 | F1:2030 | 09012020\_NaNaWb\_F4.raw | 2.38E5 |  |  |  |  |  |  |  |  |  | 1 | 1 | 0 | 0 | 0 | 0 | 0 | 0 | 0 | 0 | 0 | 495 | 510 | Carbamidomethylation |
| total 1 peptides |  |  |  |  |  |  |  |  |  |  |  |  |  |  |  |  |  |  |  |  |  |  |  |  |  |  |  |  |  |  |  |  |  |  |  |
| --- | --- | --- | --- | --- | --- | --- | --- | --- | --- | --- | --- | --- | --- | --- | --- | --- | --- | --- | --- | --- | --- | --- | --- | --- | --- | --- | --- | --- | --- | --- | --- | --- | --- | --- | --- |

P82849|3S1B2\_NAJKA

back to list

  

| Protein Coverage
| Supporting Peptides
|

Protein Coverage:

Supporting Peptides:

| Peptide | Uniq | -10lgP | Mass | Length | ppm | m/z | z | RT | Fraction | Scan | Source File | Area F4 | Area F1 | Area F10 | Area F2 | Area F3 | Area F5 | Area F6 | Area F7 | Area F8 | Area F9 | #Feature | #Feature F4 | #Feature F1 | #Feature F10 | #Feature F2 | #Feature F3 | #Feature F5 | #Feature F6 | #Feature F7 | #Feature F8 | #Feature F9 | Start | End | PTM |
| --- | --- | --- | --- | --- | --- | --- | --- | --- | --- | --- | --- | --- | --- | --- | --- | --- | --- | --- | --- | --- | --- | --- | --- | --- | --- | --- | --- | --- | --- | --- | --- | --- | --- | --- | --- |
| LEC(+57.02)HNQQSSQTPTTTGC(+57.02).S | Y | 59.75 | 1947.8052 | 17 | 0.6 | 974.9104 | 2 | 12.23 | 4 | F4:2059 | 27122019\_RID\_1315\_NaNaWb\_F2.raw |  |  |  | 3.76E7 |  |  |  |  |  |  | 1 | 0 | 0 | 0 | 1 | 0 | 0 | 0 | 0 | 0 | 0 | 1 | 17 | Carbamidomethylation |
| total 1 peptides |  |  |  |  |  |  |  |  |  |  |  |  |  |  |  |  |  |  |  |  |  |  |  |  |  |  |  |  |  |  |  |  |  |  |  |
| --- | --- | --- | --- | --- | --- | --- | --- | --- | --- | --- | --- | --- | --- | --- | --- | --- | --- | --- | --- | --- | --- | --- | --- | --- | --- | --- | --- | --- | --- | --- | --- | --- | --- | --- | --- |

P0CAR1|3S1D1\_MICPY

back to list

  

| Protein Coverage
| Supporting Peptides
|

Protein Coverage:

Supporting Peptides:

| Peptide | Uniq | -10lgP | Mass | Length | ppm | m/z | z | RT | Fraction | Scan | Source File | Area F4 | Area F1 | Area F10 | Area F2 | Area F3 | Area F5 | Area F6 | Area F7 | Area F8 | Area F9 | #Feature | #Feature F4 | #Feature F1 | #Feature F10 | #Feature F2 | #Feature F3 | #Feature F5 | #Feature F6 | #Feature F7 | #Feature F8 | #Feature F9 | Start | End | PTM |
| --- | --- | --- | --- | --- | --- | --- | --- | --- | --- | --- | --- | --- | --- | --- | --- | --- | --- | --- | --- | --- | --- | --- | --- | --- | --- | --- | --- | --- | --- | --- | --- | --- | --- | --- | --- |
| MIC(+57.02)YNQQSSQPPTTK.T | Y | 54.64 | 1781.8076 | 15 | -3.6 | 891.9079 | 2 | 12.46 | 4 | F4:2206 | 27122019\_RID\_1315\_NaNaWb\_F2.raw |  |  |  | 5.03E5 |  |  |  |  |  |  | 1 | 0 | 0 | 0 | 1 | 0 | 0 | 0 | 0 | 0 | 0 | 1 | 15 | Carbamidomethylation |
| total 1 peptides |  |  |  |  |  |  |  |  |  |  |  |  |  |  |  |  |  |  |  |  |  |  |  |  |  |  |  |  |  |  |  |  |  |  |  |
| --- | --- | --- | --- | --- | --- | --- | --- | --- | --- | --- | --- | --- | --- | --- | --- | --- | --- | --- | --- | --- | --- | --- | --- | --- | --- | --- | --- | --- | --- | --- | --- | --- | --- | --- | --- |

Q2VQM5|VIT3\_SOLIN

back to list

  

| Protein Coverage
| Supporting Peptides
|

Protein Coverage:

Supporting Peptides:

| Peptide | Uniq | -10lgP | Mass | Length | ppm | m/z | z | RT | Fraction | Scan | Source File | Area F4 | Area F1 | Area F10 | Area F2 | Area F3 | Area F5 | Area F6 | Area F7 | Area F8 | Area F9 | #Feature | #Feature F4 | #Feature F1 | #Feature F10 | #Feature F2 | #Feature F3 | #Feature F5 | #Feature F6 | #Feature F7 | #Feature F8 | #Feature F9 | Start | End | PTM |
| --- | --- | --- | --- | --- | --- | --- | --- | --- | --- | --- | --- | --- | --- | --- | --- | --- | --- | --- | --- | --- | --- | --- | --- | --- | --- | --- | --- | --- | --- | --- | --- | --- | --- | --- | --- |
| K.LVGPIFYK.L | Y | 54.54 | 935.5480 | 8 | 0.4 | 468.7815 | 2 | 46.67 | 7 | F7:16298 | 27122019\_RID\_1315\_NaNaWb\_F6.raw |  |  |  |  |  |  | 2.8E8 |  | 2.65E10 |  | 12 | 0 | 0 | 0 | 0 | 0 | 0 | 2 | 0 | 10 | 0 | 671 | 678 |  |
| total 1 peptides |  |  |  |  |  |  |  |  |  |  |  |  |  |  |  |  |  |  |  |  |  |  |  |  |  |  |  |  |  |  |  |  |  |  |  |
| --- | --- | --- | --- | --- | --- | --- | --- | --- | --- | --- | --- | --- | --- | --- | --- | --- | --- | --- | --- | --- | --- | --- | --- | --- | --- | --- | --- | --- | --- | --- | --- | --- | --- | --- | --- |

Q2VQM6|VIT2\_SOLIN

back to list

  

| Protein Coverage
| Supporting Peptides
|

Protein Coverage:

Supporting Peptides:

| Peptide | Uniq | -10lgP | Mass | Length | ppm | m/z | z | RT | Fraction | Scan | Source File | Area F4 | Area F1 | Area F10 | Area F2 | Area F3 | Area F5 | Area F6 | Area F7 | Area F8 | Area F9 | #Feature | #Feature F4 | #Feature F1 | #Feature F10 | #Feature F2 | #Feature F3 | #Feature F5 | #Feature F6 | #Feature F7 | #Feature F8 | #Feature F9 | Start | End | PTM |
| --- | --- | --- | --- | --- | --- | --- | --- | --- | --- | --- | --- | --- | --- | --- | --- | --- | --- | --- | --- | --- | --- | --- | --- | --- | --- | --- | --- | --- | --- | --- | --- | --- | --- | --- | --- |
| K.LVGPIFYK.L | Y | 54.54 | 935.5480 | 8 | 0.4 | 468.7815 | 2 | 46.67 | 7 | F7:16298 | 27122019\_RID\_1315\_NaNaWb\_F6.raw |  |  |  |  |  |  | 2.8E8 |  | 2.65E10 |  | 12 | 0 | 0 | 0 | 0 | 0 | 0 | 2 | 0 | 10 | 0 | 691 | 698 |  |
| total 1 peptides |  |  |  |  |  |  |  |  |  |  |  |  |  |  |  |  |  |  |  |  |  |  |  |  |  |  |  |  |  |  |  |  |  |  |  |
| --- | --- | --- | --- | --- | --- | --- | --- | --- | --- | --- | --- | --- | --- | --- | --- | --- | --- | --- | --- | --- | --- | --- | --- | --- | --- | --- | --- | --- | --- | --- | --- | --- | --- | --- | --- |

Q5VSP4|LC1L1\_HUMAN

back to list

  

| Protein Coverage
| Supporting Peptides
|

Protein Coverage:

Supporting Peptides:

| Peptide | Uniq | -10lgP | Mass | Length | ppm | m/z | z | RT | Fraction | Scan | Source File | Area F4 | Area F1 | Area F10 | Area F2 | Area F3 | Area F5 | Area F6 | Area F7 | Area F8 | Area F9 | #Feature | #Feature F4 | #Feature F1 | #Feature F10 | #Feature F2 | #Feature F3 | #Feature F5 | #Feature F6 | #Feature F7 | #Feature F8 | #Feature F9 | Start | End | PTM |
| --- | --- | --- | --- | --- | --- | --- | --- | --- | --- | --- | --- | --- | --- | --- | --- | --- | --- | --- | --- | --- | --- | --- | --- | --- | --- | --- | --- | --- | --- | --- | --- | --- | --- | --- | --- |
| R.GLSTESILIPR.Q | Y | 50.25 | 1184.6765 | 11 | 0.3 | 593.3457 | 2 | 41.48 | 2 | F2:9973 | 27122019\_RID\_1315\_NaNaWb\_F1.raw |  | 0 |  |  |  |  |  |  |  |  | 1 | 0 | 1 | 0 | 0 | 0 | 0 | 0 | 0 | 0 | 0 | 142 | 152 |  |
| total 1 peptides |  |  |  |  |  |  |  |  |  |  |  |  |  |  |  |  |  |  |  |  |  |  |  |  |  |  |  |  |  |  |  |  |  |  |  |
| --- | --- | --- | --- | --- | --- | --- | --- | --- | --- | --- | --- | --- | --- | --- | --- | --- | --- | --- | --- | --- | --- | --- | --- | --- | --- | --- | --- | --- | --- | --- | --- | --- | --- | --- | --- |

P31025|LCN1\_HUMAN

back to list

  

| Protein Coverage
| Supporting Peptides
|

Protein Coverage:

Supporting Peptides:

| Peptide | Uniq | -10lgP | Mass | Length | ppm | m/z | z | RT | Fraction | Scan | Source File | Area F4 | Area F1 | Area F10 | Area F2 | Area F3 | Area F5 | Area F6 | Area F7 | Area F8 | Area F9 | #Feature | #Feature F4 | #Feature F1 | #Feature F10 | #Feature F2 | #Feature F3 | #Feature F5 | #Feature F6 | #Feature F7 | #Feature F8 | #Feature F9 | Start | End | PTM |
| --- | --- | --- | --- | --- | --- | --- | --- | --- | --- | --- | --- | --- | --- | --- | --- | --- | --- | --- | --- | --- | --- | --- | --- | --- | --- | --- | --- | --- | --- | --- | --- | --- | --- | --- | --- |
| R.GLSTESILIPR.Q | Y | 50.25 | 1184.6765 | 11 | 0.3 | 593.3457 | 2 | 41.48 | 2 | F2:9973 | 27122019\_RID\_1315\_NaNaWb\_F1.raw |  | 0 |  |  |  |  |  |  |  |  | 1 | 0 | 1 | 0 | 0 | 0 | 0 | 0 | 0 | 0 | 0 | 156 | 166 |  |
| total 1 peptides |  |  |  |  |  |  |  |  |  |  |  |  |  |  |  |  |  |  |  |  |  |  |  |  |  |  |  |  |  |  |  |  |  |  |  |
| --- | --- | --- | --- | --- | --- | --- | --- | --- | --- | --- | --- | --- | --- | --- | --- | --- | --- | --- | --- | --- | --- | --- | --- | --- | --- | --- | --- | --- | --- | --- | --- | --- | --- | --- | --- |

A1TYI9|RL7\_MARHV

back to list

  

| Protein Coverage
| Supporting Peptides
|

Protein Coverage:

Supporting Peptides:

| Peptide | Uniq | -10lgP | Mass | Length | ppm | m/z | z | RT | Fraction | Scan | Source File | Area F4 | Area F1 | Area F10 | Area F2 | Area F3 | Area F5 | Area F6 | Area F7 | Area F8 | Area F9 | #Feature | #Feature F4 | #Feature F1 | #Feature F10 | #Feature F2 | #Feature F3 | #Feature F5 | #Feature F6 | #Feature F7 | #Feature F8 | #Feature F9 | Start | End | PTM |
| --- | --- | --- | --- | --- | --- | --- | --- | --- | --- | --- | --- | --- | --- | --- | --- | --- | --- | --- | --- | --- | --- | --- | --- | --- | --- | --- | --- | --- | --- | --- | --- | --- | --- | --- | --- |
| F.DVVLTGAGEK.K | Y | 46.34 | 987.5236 | 10 | 0.8 | 494.7695 | 2 | 23.02 | 1 | F1:7656 | 09012020\_NaNaWb\_F4.raw | 2.13E6 |  |  |  |  |  |  |  |  |  | 1 | 1 | 0 | 0 | 0 | 0 | 0 | 0 | 0 | 0 | 0 | 59 | 68 |  |
| total 1 peptides |  |  |  |  |  |  |  |  |  |  |  |  |  |  |  |  |  |  |  |  |  |  |  |  |  |  |  |  |  |  |  |  |  |  |  |
| --- | --- | --- | --- | --- | --- | --- | --- | --- | --- | --- | --- | --- | --- | --- | --- | --- | --- | --- | --- | --- | --- | --- | --- | --- | --- | --- | --- | --- | --- | --- | --- | --- | --- | --- | --- |

P84805|CRVP1\_NAJKA

back to list

  

| Protein Coverage
| Supporting Peptides
|

Protein Coverage:

Supporting Peptides:

| Peptide | Uniq | -10lgP | Mass | Length | ppm | m/z | z | RT | Fraction | Scan | Source File | Area F4 | Area F1 | Area F10 | Area F2 | Area F3 | Area F5 | Area F6 | Area F7 | Area F8 | Area F9 | #Feature | #Feature F4 | #Feature F1 | #Feature F10 | #Feature F2 | #Feature F3 | #Feature F5 | #Feature F6 | #Feature F7 | #Feature F8 | #Feature F9 | Start | End | PTM |
| --- | --- | --- | --- | --- | --- | --- | --- | --- | --- | --- | --- | --- | --- | --- | --- | --- | --- | --- | --- | --- | --- | --- | --- | --- | --- | --- | --- | --- | --- | --- | --- | --- | --- | --- | --- |
| K.SNC(+57.02)PASC(+57.02)FC(+57.02)R.N | Y | 43.45 | 1257.4689 | 10 | -0.2 | 629.7416 | 2 | 15.99 | 6 | F6:3472 | 27122019\_RID\_1315\_NaNaWb\_F5.raw |  |  |  |  |  | 5.81E4 |  |  |  |  | 1 | 0 | 0 | 0 | 0 | 0 | 1 | 0 | 0 | 0 | 0 | 226 | 235 | Carbamidomethylation |
| total 1 peptides |  |  |  |  |  |  |  |  |  |  |  |  |  |  |  |  |  |  |  |  |  |  |  |  |  |  |  |  |  |  |  |  |  |  |  |
| --- | --- | --- | --- | --- | --- | --- | --- | --- | --- | --- | --- | --- | --- | --- | --- | --- | --- | --- | --- | --- | --- | --- | --- | --- | --- | --- | --- | --- | --- | --- | --- | --- | --- | --- | --- |

Q7T1K6|CRVP1\_NAJAT

back to list

  

| Protein Coverage
| Supporting Peptides
|

Protein Coverage:

Supporting Peptides:

| Peptide | Uniq | -10lgP | Mass | Length | ppm | m/z | z | RT | Fraction | Scan | Source File | Area F4 | Area F1 | Area F10 | Area F2 | Area F3 | Area F5 | Area F6 | Area F7 | Area F8 | Area F9 | #Feature | #Feature F4 | #Feature F1 | #Feature F10 | #Feature F2 | #Feature F3 | #Feature F5 | #Feature F6 | #Feature F7 | #Feature F8 | #Feature F9 | Start | End | PTM |
| --- | --- | --- | --- | --- | --- | --- | --- | --- | --- | --- | --- | --- | --- | --- | --- | --- | --- | --- | --- | --- | --- | --- | --- | --- | --- | --- | --- | --- | --- | --- | --- | --- | --- | --- | --- |
| K.SNC(+57.02)PASC(+57.02)FC(+57.02)R.N | Y | 43.45 | 1257.4689 | 10 | -0.2 | 629.7416 | 2 | 15.99 | 6 | F6:3472 | 27122019\_RID\_1315\_NaNaWb\_F5.raw |  |  |  |  |  | 5.81E4 |  |  |  |  | 1 | 0 | 0 | 0 | 0 | 0 | 1 | 0 | 0 | 0 | 0 | 226 | 235 | Carbamidomethylation |
| total 1 peptides |  |  |  |  |  |  |  |  |  |  |  |  |  |  |  |  |  |  |  |  |  |  |  |  |  |  |  |  |  |  |  |  |  |  |  |
| --- | --- | --- | --- | --- | --- | --- | --- | --- | --- | --- | --- | --- | --- | --- | --- | --- | --- | --- | --- | --- | --- | --- | --- | --- | --- | --- | --- | --- | --- | --- | --- | --- | --- | --- | --- |

P50079|HSV2\_YEAST

back to list

  

| Protein Coverage
| Supporting Peptides
|

Protein Coverage:

Supporting Peptides:

| Peptide | Uniq | -10lgP | Mass | Length | ppm | m/z | z | RT | Fraction | Scan | Source File | Area F4 | Area F1 | Area F10 | Area F2 | Area F3 | Area F5 | Area F6 | Area F7 | Area F8 | Area F9 | #Feature | #Feature F4 | #Feature F1 | #Feature F10 | #Feature F2 | #Feature F3 | #Feature F5 | #Feature F6 | #Feature F7 | #Feature F8 | #Feature F9 | Start | End | PTM |
| --- | --- | --- | --- | --- | --- | --- | --- | --- | --- | --- | --- | --- | --- | --- | --- | --- | --- | --- | --- | --- | --- | --- | --- | --- | --- | --- | --- | --- | --- | --- | --- | --- | --- | --- | --- |
| R.IFNTDPLTSK.L | Y | 43.04 | 1134.5920 | 10 | 1.6 | 568.3042 | 2 | 64.87 | 1 | F1:34930 | 09012020\_NaNaWb\_F4.raw | 1.63E5 |  |  |  |  |  |  |  |  |  | 1 | 1 | 0 | 0 | 0 | 0 | 0 | 0 | 0 | 0 | 0 | 41 | 50 |  |
| total 1 peptides |  |  |  |  |  |  |  |  |  |  |  |  |  |  |  |  |  |  |  |  |  |  |  |  |  |  |  |  |  |  |  |  |  |  |  |
| --- | --- | --- | --- | --- | --- | --- | --- | --- | --- | --- | --- | --- | --- | --- | --- | --- | --- | --- | --- | --- | --- | --- | --- | --- | --- | --- | --- | --- | --- | --- | --- | --- | --- | --- | --- |

P37398|VIV\_ORYSJ

back to list

  

| Protein Coverage
| Supporting Peptides
|

Protein Coverage:

Supporting Peptides:

| Peptide | Uniq | -10lgP | Mass | Length | ppm | m/z | z | RT | Fraction | Scan | Source File | Area F4 | Area F1 | Area F10 | Area F2 | Area F3 | Area F5 | Area F6 | Area F7 | Area F8 | Area F9 | #Feature | #Feature F4 | #Feature F1 | #Feature F10 | #Feature F2 | #Feature F3 | #Feature F5 | #Feature F6 | #Feature F7 | #Feature F8 | #Feature F9 | Start | End | PTM |
| --- | --- | --- | --- | --- | --- | --- | --- | --- | --- | --- | --- | --- | --- | --- | --- | --- | --- | --- | --- | --- | --- | --- | --- | --- | --- | --- | --- | --- | --- | --- | --- | --- | --- | --- | --- |
| K.QGGGGGGGGGRGK.A | Y | 42.69 | 1000.4798 | 13 | -4.7 | 501.2448 | 2 | 21.65 | 10 | F10:4219 | 27122019\_RID\_1315\_NaNaWb\_F9.raw |  |  |  |  |  |  |  |  |  | 6.52E5 | 1 | 0 | 0 | 0 | 0 | 0 | 0 | 0 | 0 | 0 | 1 | 19 | 31 |  |
| total 1 peptides |  |  |  |  |  |  |  |  |  |  |  |  |  |  |  |  |  |  |  |  |  |  |  |  |  |  |  |  |  |  |  |  |  |  |  |
| --- | --- | --- | --- | --- | --- | --- | --- | --- | --- | --- | --- | --- | --- | --- | --- | --- | --- | --- | --- | --- | --- | --- | --- | --- | --- | --- | --- | --- | --- | --- | --- | --- | --- | --- | --- |

Q9LQU4|PCR2\_ARATH

back to list

  

| Protein Coverage
| Supporting Peptides
|

Protein Coverage:

Supporting Peptides:

| Peptide | Uniq | -10lgP | Mass | Length | ppm | m/z | z | RT | Fraction | Scan | Source File | Area F4 | Area F1 | Area F10 | Area F2 | Area F3 | Area F5 | Area F6 | Area F7 | Area F8 | Area F9 | #Feature | #Feature F4 | #Feature F1 | #Feature F10 | #Feature F2 | #Feature F3 | #Feature F5 | #Feature F6 | #Feature F7 | #Feature F8 | #Feature F9 | Start | End | PTM |
| --- | --- | --- | --- | --- | --- | --- | --- | --- | --- | --- | --- | --- | --- | --- | --- | --- | --- | --- | --- | --- | --- | --- | --- | --- | --- | --- | --- | --- | --- | --- | --- | --- | --- | --- | --- |
| K.GDDC(+57.02)TDC(+57.02)LK.H | Y | 42.21 | 1082.4009 | 9 | 4.8 | 542.2103 | 2 | 28.71 | 6 | F6:10548 | 27122019\_RID\_1315\_NaNaWb\_F5.raw |  |  |  |  |  | 1.41E6 |  |  |  |  | 1 | 0 | 0 | 0 | 0 | 0 | 1 | 0 | 0 | 0 | 0 | 91 | 99 | Carbamidomethylation |
| total 1 peptides |  |  |  |  |  |  |  |  |  |  |  |  |  |  |  |  |  |  |  |  |  |  |  |  |  |  |  |  |  |  |  |  |  |  |  |
| --- | --- | --- | --- | --- | --- | --- | --- | --- | --- | --- | --- | --- | --- | --- | --- | --- | --- | --- | --- | --- | --- | --- | --- | --- | --- | --- | --- | --- | --- | --- | --- | --- | --- | --- | --- |

Peptide List

  
  

---

Prepared with PEAKS ™ (bioinfor.com)
